# A robust Th-azole framework for highly efficient purification of C_2_H_4_ from a C_2_H_4_/C_2_H_2_/C_2_H_6_ mixture

**DOI:** 10.1038/s41467-020-16960-9

**Published:** 2020-06-22

**Authors:** Zhenzhen Xu, Xiaohong Xiong, Jianbo Xiong, Rajamani Krishna, Libo Li, Yaling Fan, Feng Luo, Banglin Chen

**Affiliations:** 1 State key Laboratory of Nuclear Resources and Environment, School of Chemistry, Biology and Material Science, East China University of Technology, 330013 Nanchang, P. R. China; 2 Van’t Hoff Institute for Molecular Sciences, University of Amsterdam, Science Park 904, 1098 XH Amsterdam, The Netherlands; 3 College of Chemistry and Chemical Engineering, Shanxi Key Laboratory of Gas Energy Efficient and Clean Utilization, Taiyuan University of Technology, 030024 Taiyuan, Shanxi China; 4 Department of Chemistry, University of Texas at San Antonio, San Antonio, TX USA

**Keywords:** Chemical engineering, Metal-organic frameworks, Porous materials

## Abstract

Separation of C_2_H_4_ from C_2_H_4_/C_2_H_2_/C_2_H_6_ mixture with high working capacity is still a challenging task. Herein, we deliberately design a Th-metal-organic framework (MOF) for highly efficient separation of C_2_H_4_ from a binary C_2_H_6_/C_2_H_4_ and ternary C_2_H_4_/C_2_H_2_/C_2_H_6_ mixture. The synthesized MOF Azole-Th-1 shows a UiO-66-type structure with fcu topology built on a Th_6_ secondary building unit and a tetrazole-based linker. Such noticeable structure, is connected by a N,O-donor ligand with high chemical stability. At 100 kPa and 298 K Azole-Th-1 performs excellent separation of C_2_H_4_ (purity > 99.9%) from not only a binary C_2_H_6_/C_2_H_4_ (1:9, v/v) mixture but also a ternary mixture of C_2_H_6_/C_2_H_2_/C_2_H_4_ (9:1:90, v/v/v), and the corresponding working capacity can reach up to 1.13 and 1.34 mmol g^−1^, respectively. The separation mechanism, as unveiled by the density functional theory calculation, is due to a stronger van der Waals interaction between ethane and the MOF skeleton.

## Abstract (web summary)

Separation of ethylene from C2 ternary mixture with high working capacity is a challenging task. Here, the authors report a Th-azole framework (Azole-Th-1) for highly efficient purification of ethylene from C_2_H_4_/C_2_H_6_ and C_2_H_4_/C_2_H_2_/C_2_H_6_ mixtures, respectively.

## Introduction

Ethylene is one of the most widely used feedstock molecules for the production of polymers and high-value organic chemicals^1,2^. It is usually produced by the thermal cracking of hydrocarbons. The removal of ethane and acetylene by-products that inevitably arise during these processes is one of the most challenging chemical separations due to the similarity of the physicochemical properties of ethane (kinetic diameter 4.4 Å, boiling point 184.55 K), ethylene (kinetic diameter 4.2 Å, boiling point 169.42 K), and acetylene kinetic diameter 3.3 Å, boiling point 188.40 K)^3–7^.

At present, cryogenic distillation is the main technology used to separate ethane and ethylene with the requirement of high pressure (5–28 bar) and low temperature (180–258 K)^8,9^, which indicates that this process is expensive and comes with a high energy penalty. And partial hydrogenation of acetylene into ethylene over catalyst^10^ or solvent extraction of cracked olefins^11^ are also involved with the purification of ethylene from acetylene. Adsorptive separation by porous materials is an alternative technology, especially, some metal-organic frameworks (MOFs)^12–21^ with high volume, designable pore characteristics, and countless structural possibilities, can be employed into the gas separation processes, the adsorption selectivity and capacity are higher than the results of conventional adsorbents^22–24^ such as zeolites and carbon-based, especially the adsorption and separation for C_2_H_6_/C_2_H_4_^8,22,25–39^.

For the MOFs with open metal site, the ethylene can easily bind to it, leading to highly selective uptake of ethylene over ethane, due to the electrostatic interaction between the π-electron in ethylene and the positive charge in open metal sites^36,40–44^, such as, HKUST-1^44^, particularly in the low pressure region, is preferential adsorption of ethylene, which is supported by some theoretical calculations^45,46^. In contract, for some special MOFs, when the coordination positions of metal reaches to saturate, they can enable favorable adsorption towards ethane over ethylene through their unique pore wall that affords stronger van der Waals (vdW) interaction between the H of C_2_H_6_ and the MOF skeleton^8,22,25–29,32,34,35,47^. For example, ZIF-7 presents the first example of a microporous solid displaying the selective adsorption of paraffins over olefins^48^. However, MOFs showing such uncommon adsorption phenomenon (C_2_H_6_ over C_2_H_4_) is still rare until now.

Recently, Lu and co-workers^49^ report that they use TJT-100 to obtain the selective adsorption of ethane and acetylene over ethylene from a ternary mixture of C_2_H_2_/C_2_H_6_/C_2_H_4_ (0.5:0.5:99, v/v/v) and achieve a C_2_H_4_ purity greater than 99.9% (working capacity of 0.69 mmol g^−1^) by a single-breakthrough operation. Zaworoko et al.^38^ use a synergistic sorbent separation method for the one-step production of polymer-grade C_2_H_4_ from ternary (C_2_H_2_/C_2_H_6_/C_2_H_4_, working capacity of 0.32 mmol g^−1^) or quaternary (CO_2_/C_2_H_2_/C_2_H_6_/C_2_H_4_) gas mixtures with a series of physisorbents. In this regard, constructing MOFs with high working capacity is still highly desirable from the viewpoint of practical application.

The high-valence metal ions are often used to construct stable MOFs, such as Cr(III) for MIL-101^50^ and Zr(VI) for UiO-66^51^. However, due to the easy-to-hydrolysis nature of both Cr(III) and Zr(IV), it is still difficult to synthesize Cr(III) and Zr(IV) MOFs with high crystallization. Alternatively, another high-valence metal ion of Th(IV) shows less hydrolytic nature, suggesting an optimal metal ion to generate stable MOFs^52–56^. Generally speaking, according to the hard and soft acid and bases (HSAB) principle, the Zr-based or Th-based MOFs are usually constructed by O-donor carboxylic ligands. For example, the typical UiO-66-type structure is connected by various linear O-donor carboxylic ligands, as shown in the left of Supplementary Fig. 1^51,56,57^. As we know, the N,O-donor ligands such as azole series have been attested to be an excellent organic linkers to construct a great number of MOFs (more than 900 tetrazole-based MOFs and more than 5000 triazole-based MOF from CCDC data)^58–60^. However, there is no Zr-based or Th-based MOFs built on N,O-donor ligands. As shown in the right of Supplementary Fig. 1, the construction of azole-based Zr- or Th-based MOFs is possible, because of the comparable coordination direction for carboxylate and azole molecules, while the introduction of azole unit to bind with Zr or Th ions is also possibly a powerful tool to modulate the electronic structure of metal center and the environment of pore wall, consequently leading to unique physical properties.

In this work, we obtain a successful case via solvothermal reaction of Th(NO_3_)_4_ and 4-(1H-tetrazol-5-yl) benzoic acid (TBA). This compound shows a UiO-66-type structure, except for the replacement of secondary building unit such as Zr_6_ by Th_6_ and linkers such as O-donor ligand by N,O-donor ligand. This unique tetrazole-based structure allows it to perform high C_2_H_6_ uptake at room temperature and selective adsorption of C_2_H_6_ over C_2_H_4_, finally resulting in the promising application for C_2_H_4_ separation from the binary C_2_H_6_/C_2_H_4_ and ternary C_2_H_4_/C_2_H_2_/C_2_H_6_ mixture. Furthermore, both the grand canonical Monte Carlo (GCMC) simulations and density functional theory (DFT) calculations are carried out to disclose the separation mechanism.

## Results

### Synthesis, structure, and characterization of Azole-Th-1

The reaction between ligand TBA and Th(NO_3_)_4_ in N,N′-dimethylformamide (DMF) yields colorless octahedral crystals of Azole-Th-1 (Supplementary Fig. 2). The synthesis in detail is listed in Supplementary Information. The purity of the bulk samples was confirmed by powder X-ray diffraction (PXRD, Supplementary Fig. 3).

**Fig. 1:**
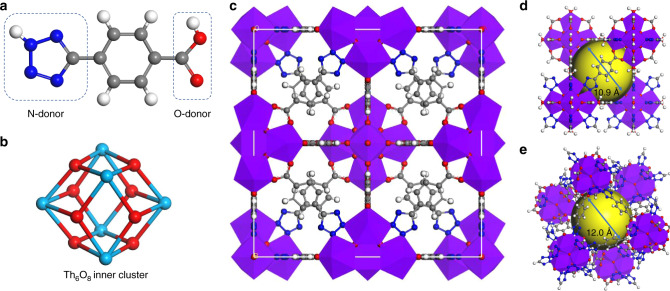
Main structures. **a** Ligand TBA, including carboxylic acid donor (O-donor) and tetrazole donor (N-donor), **b** inner core Th_6_-cluster drawn alone for clarity (Th_6_O_8_), **c** unit cell structure in crystal Azole-Th-1, **d**, **e** two types of cages, including super tetrahedron cage **d** and super octahedron cage **e**. Where, Th-light blue ball **b** and polyhedron style **c**–**e** with purple color, O-red ball, C-gray ball, N-blue ball, and H-white ball.

The PXRD discloses that the octahedral crystals of Azole-Th-1 crystallize in the cubic space group *Fm3m*, similar to UiO-66 (Fig. 1c). The length of *a*, *b*, *c* is 23.984(4) Å, longer than UiO-66-Zr (20.743 (5) Å)^51^ and UiO-66-Th (21.961(13) Å)^56^, mainly due to the longer linker of TBA (ca. 8.4 Å) in Azole-Th-1 than terephthalic acid (ca. 6.8 Å) in UiO-66. Six Th(IV) metal ions are combined together to give the Th_6_O_4_(OH)_4_(H_2_O)_6_ core (Fig. 1b), similar to the Zr_6_O_4_(OH)_4_(H_2_O)_6_ in UiO-66. The Th(IV) ion holds the nine-coordination surrounding with a monocapped square antiprismatic geometry. The Th-O bond length from O^2−^, OH^−^, and H_2_O is varied from 2.33 to 2.55 Å, slight shorter than the Th–N bond length of 2.74 Å. As observed in the literature^61,62^ for the TBA ligand usually showing highly disordered structure, similar trend is observed in Azole-Th-1 (Supplementary Fig. 4). The TBA ligand contacts with Th(IV) ions via both carboxylate and azole bridge, while two additional nitrogen atoms for each TBA ligand are free-standing without coordination. The 3D framework is formed by both the inorganic Th_6_O_4_(OH)_4_(H_2_O)_6_ core and TBA linkers, where each inorganic core connects to twelve identical Th_6_O_4_(OH)_4_(H_2_O)_6_ cores via twelve TBA linkers, finally constructing the UiO-66-like structure. Similarly, two different types of cages, *viz*. a super tetrahedron cage (Fig. 1d) and a super octahedron (Fig. 1e) with the largest cavity diameter of 1.1 nm and 1.2 nm, respectively, is observed in Azole-Th-1. This is larger the corresponding values of 0.88 nm in UiO-66^51^, due to the longer linkers of TBA in Azole-Th-1 than terephthalic acid in UiO-66. The solvent-accessible volume estimated by Platon program^63^ is 50.1% of the unit cell, suggesting high porosity of this MOF.

**Fig. 2:**
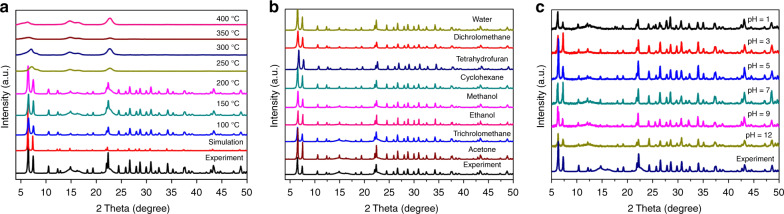
The PXRD patterns of Azole-Th-1 samples. **a** Thermal stability from 100 to 400 °C, including simulated and experimental results, **b** soaking in water and seven different organic solvents 30 days, and **c** soaking in different pH solvents 30 days. Source data are provided as a Source Data file.

**Fig. 3:**
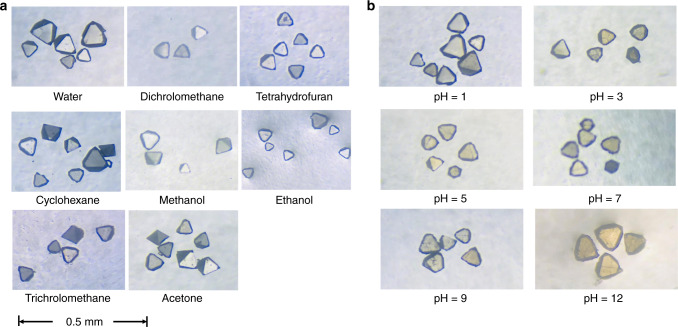
The optical microscope images of Azole-Th-1 samples. **a** after soaking in water and seven different organic solvents 30 days, **b** after soaking in different pH solvents 30 days.

The loss of trapped solvent molecules from Azole-Th-1, according to the thermogravimetric (TG) analysis, is before 75 °C (Supplementary Fig. 5). While the temperature increased to about 250 °C, the crystal structure begins to collapse, which is in agreement with the results of temperature dependent PXRD tests (Fig. 2a). As shown in Fig. 2a, the PXRD of Azole-Th-1 samples from room temperature to 200 °C are matching with the experimental and simulated results. While the temperature reaches to 250 °C, the peaks of PXRD disappear, which indicates that the crystal structure is destroyed by such high temperature. On the other hand, the chemical stability under water and different solvents environment, including seven organic solvents and a broad pH range from 1 to 12, were also traced by PXRD tests (Fig. 2b, c), where respective optical images of crystal were represented in Fig. 3. Note that the crystals of Azole-Th-1 render excellent stability in water and above solvents even after 30 days.

### Adsorption isotherm, selectivity, and breakthrough

**Fig. 4:**
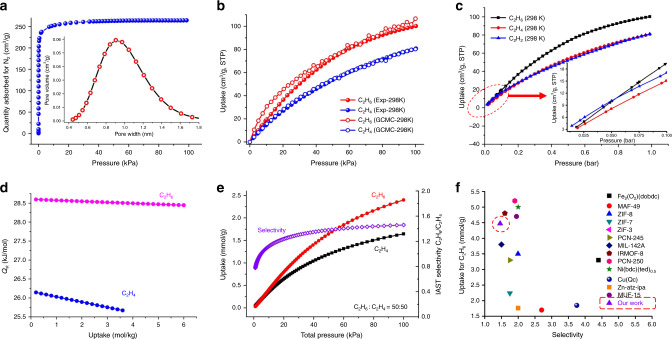
The adsorption and separation data of Azole-Th-1. **a** The N_2_ adsorption at 77 K with the insert of the distribution of pore size. **b** The adsorption isotherms of C_2_H_6_ and C_2_H_4_ at 298 K, including experiments and simulations. **c** Experimental adsorption isotherms of Azole-Th-1 for C_2_H_6_, C_2_H_4_, and C_2_H_2_ at 298 K from 0.01 to 1 bar with the insert of enlargement from 0.01 to 0.1 bar. **d** The adsorption heat enthalpy of C_2_H_6_ and C_2_H_4_, calculated from the single-component C_2_H_6_ and C_2_H_4_ adsorption data at 298 and 273 K. **e** Predicted mixture adsorption isotherms and selectivity of Azole-Th-1 by IAST method for a 50/50 C_2_H_6_/C_2_H_4_ mixture at 298 K. **f** A comparison in selectivity and C_2_H_6_ adsorption capacity at 298 K and 1 bar between the reported top-performing porous adsorbents for C_2_H_6_/C_2_H_4_ separation and our MOF. The purple triangle is our MOF. Source data are provided as a Source Data file.

The moderate thermal stability and high chemical stability of Azole-Th-1 prompts us to study the gas adsorption performances. To characterize the permanent porosity of the obtained material, the N_2_ adsorption isotherm at 77 K was measured. As shown in Fig. 4a, a fully reversible type I isotherm with a Brunauer Emmett Teller (BET) surface area of 983 m^2^ g^−1^ and a uniform pore size around 9.2 Å was exhibited. This pore size is comparable with that calculated LCD (the largest cavity diameter, 10.0 Å) by Zeo++ program^64^ (Supplementary Table 3).

This high porosity and desirable aperture encouraged us to further investigate C_2_H_6_/C_2_H_4_ separations in detail. Adsorption isotherms of single component C_2_H_6_ and C_2_H_4_ were collected at 298 K and 273 K, respectively, as presented in Fig. 4b and Supplementary Fig. 6. The adsorption isotherm of C_2_H_6_ is typically type I with a steep slope, which is a typical feature of strong adsorbates in microporous materials (Fig. 4b)^8,22,25,27–30,32–35^. And the adsorption amounts of C_2_H_6_ at both 273 and 298 K (121.7 and 100.2 cm^3^ g^−1^) were also higher than the corresponding C_2_H_4_ (111.3 and 80.7 cm^3^ g^−1^). Therefore, the Azole-Th-1 has a distinct preference for adsorbing ethane over ethylene. It is well-known that the magnitude of the adsorption enthalpies of porous materials reveals that the affinity of the pore surface toward adsorbents, determining the adsorptive selectivity^39,65^. This can be directly reflected on the adsorption heat enthalpy (*Q*_st_) of C_2_H_6_ and C_2_H_4_, giving 28.6 kJ mol^−1^ for C_2_H_6_ at the zero coverage, significantly higher than the values of 26.1 kJ mol^−1^ for C_2_H_4_ (Fig. 4d), strongly suggesting a higher affinity between host and guest for C_2_H_6_ than C_2_H_4_, where the detail virial-type analysis were provided in Supplementary Fig. 7.

At room temperature and 100 kPa, Azole-Th-1 affords ultrahigh adsorption capacity of C_2_H_6_ up to 100.2 cm^3^ g^−1^. This value exceeds most reported top-performing porous adsorbents for such use as shown in Table S2, including in Fe_2_(O_2_)(dobdc)^35^, MAF-49^22^, ZIF-8^34^, ZIF-7^32^, PCN-245^26^, MIL-142A^28^, Cu(Qc)_2_^37^, Zn-atz-ipa^38^, etc., which are summarized in Fig. 4f. Correspondingly, adsorption capacity of C_2_H_4_ at the same conditions is 81.1 cm^3^ g^−1^, obviously, less than C_2_H_6_ about 20 cm^3^ g^−1^. Hence, selective adsorption of C_2_H_6_ over C_2_H_4_ is suggested. To estimate the adsorption selectivity, we employed the ideal adsorption solution theory (IAST)^66^ to analyze the experimental isotherm data, using composition of 50:50/10:90/1:15 C_2_H_6_/C_2_H_4_, as shown in Fig. 4e and Supplementary Fig. 8. The selectivity of C_2_H_6_ over C_2_H_4_ was up to 1.46 at room temperature and 100 kPa, which is slightly higher than that of other ratio mixture, both 1.44 for 10:90 and 1:15 C_2_H_6_/C_2_H_4_. To the best of our knowledge, Azole-Th-1 should be the first Th-MOF showing such abnormal adsorption behavior (C_2_H_6_ over C_2_H_4_).

**Fig. 5:**
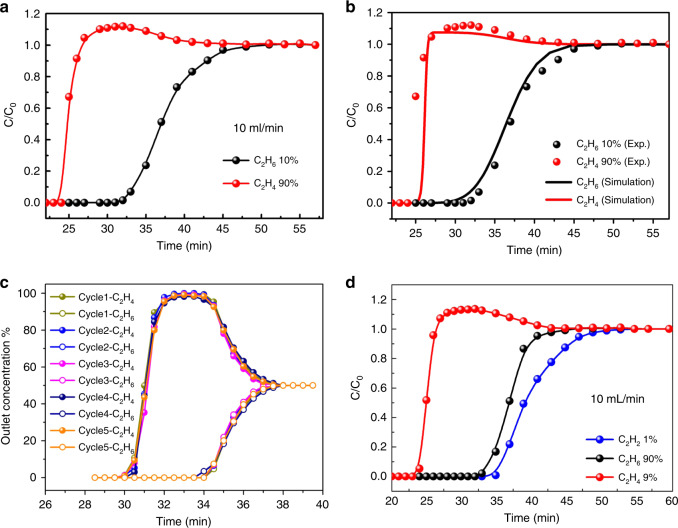
Experimental breakthrough curves at 298 K and 1 bar on Azole-Th-1. **a**, **b** C_2_H_6_/C_2_H_4_ (10/90, v/v) binary mixture. **c** C_2_H_6_/C_2_H_4_ (50/50, v/v) binary mixture for five cycles. **d** C_2_H_6_/C_2_H_4_/C_2_H_2_(90/9/1, v/v/v) ternary mixture separation. Source data are provided as a Source Data file.

Then, in order to check the actual separation ability of the gases mixture, the transient breakthrough simulations for C_2_H_6_/C_2_H_4_ (50/50, v/v) mixtures on Azole-Th-1 material was carried out at 298 K, as shown in Supplementary Fig. 9. This result demonstrates that the potential of producing pure product gas C_2_H_4_ during the time interval Δ*τ*, also suggests excellent C_2_H_6_/C_2_H_4_ separation performance. And then, to further confirm the real practical separation ability, the actual dynamic adsorption breakthrough experiments for C_2_H_6_/C_2_H_4_ (10/90, 50/50, 1/15, v/v) binary mixtures on Azole-Th-1 material were also carried out (Fig. 5a, b, and Supplementary Fig. 10). The C_2_H_4_ broke through the adsorption bed and yield a high purity gas (>99.9%) at first, whereas after a certain time C_2_H_6_ slowly eluted and reached to the equilibrium (Fig. 5a, b, and Supplementary Fig. 10). During this period of time, polymer-grade (>99.9%) C_2_H_4_ can be generated at the outlet. The breakthrough time of ethane was later than that of ethylene for these three ratio mixtures, meaning that the Azole-Th-1 preferred to adsorb ethane over ethylene. The long breakthrough time interval between C_2_H_4_ and C_2_H_6_ suggests that the Azole-Th-1 is quite effective for C_2_H_6_/C_2_H_4_ separation. The experimental breakthrough results was well in consistent with the simulated breakthrough (Fig. 5b, Supplementary Fig. 10b, d), strongly suggesting its superior application for C_2_H_4_ purification. Furthermore, cycling breakthrough experiments on Azole-Th-1 were carried out under the same conditions. The breakthrough time interval for C_2_H_6_/C_2_H_4_ mixtures in five cycles (Fig. 5c) is comparable, showing that this material has a good regenerability. According to the polymer grade C_2_H_4_ produced during time interval at different C_2_H_4_/C_2_H_6_ ratio, 3 min (50/50), 5 min (90/10), and 3.5 min (15/1), the productivities of C_2_H_4_ (>99.9%) were 0.68, 1.13, and 0.79 mmol g^−1^, respectively. Hence, the polymer grade C_2_H_4_ with the max working capacity of 1.13 mmol g^−1^ with >99.9% purity was harvested from 90/10 gas mixture, which working capacity is nearly 1.3 times for Fe_2_(O_2_)(dobdc) (0.79 mmol g^−1^)^35^ and 3.6 times for MAF-49 (0.28 mmol g^−1^)^22^, the two best materials for C_2_H_6_/C_2_H_4_ separation. Some more detailed comparison with other MOF materials is shown in Supplementary Table 2. After the breakthrough experiments, the PXRD pattern of our sample was also consistent with the PXRD before the breakthrough (Supplementary Fig. 11), which further indicated that this material has a good regenerability and high stability.

### Mechanism of gas adsorption by theoretical calculations

Theoretically, the determination of gas adsorption sites in the MOFs is of great significance for the design of some gas storage and separation materials based on MOFs^31,67^. Herein, the ultrahigh C_2_H_6_ storage capacity prompts us to explore the adsorption sites within this Azole-Th-1. Theoretical simulation is a powerful tool enabling us to unveil the adsorption mechanisms and provide the adsorption sites. Therefore, the C_2_H_6_/C_2_H_4_ binding affinity in Azole-Th-1 was firstly investigated by single-component sorption isotherms at 298 K and pressures up to 100 kPa. The GCMC simulations were performed for understanding the interactions and adsorption behaviors of C_2_H_6_ and C_2_H_4_ in Azole-Th-1 at the molecular level^25,26,33,34,39^. As shown in isotherm of C_2_H_6_/C_2_H_4_ adsorption (Fig. 4b), the maximum C_2_H_6_ and C_2_H_4_ uptake for Azole-Th-1 are 106.6 and 80.2 cm^3^ g^−1^ at 100 kPa and 298 K, respectively, both adsorbed tendency and the adsorption quantity are consistent with the experimental results (100.2 and 80.7 cm^3^ g^−1^), where the simulated details are listed in Supplementary Information.

**Fig. 6:**
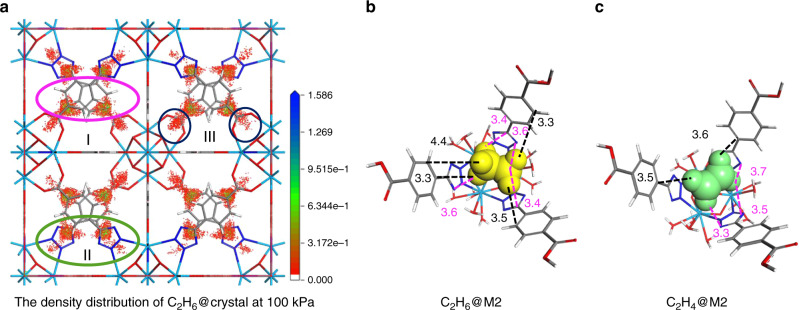
GCMC adsorption simulation and adsorbed structures. **a** The density distribution of C_2_H_6_ on Azole-Th-1 at 100 kPa and 298 K. **b**, **c** The structures of adsorptions for C_2_H_6_ and C_2_H_4_ at M2 model. Where, Th-light blue, O-red, C-gray, N-blue, H-white, C_2_H_6_-yellow molecule, and C_2_H_4_-green molecule, and the unit of distance is Å.

The further investigations on the interaction between C_2_H_6_/C_2_H_4_ and MOF material can help us to understand the mechanism of gas adsorption, which could analysis the discrepancies the interactions between C_2_H_6_ and C_2_H_4_ with our material, respectively. According to the density distribution of C_2_H_6_ on Azole-Th-1 at 298 K and 100 kPa (Fig. 6), there are three main adsorbed areas in our material, including benzene region (I-region), the tetrazol heterocycle region (II-region), and carboxylate region (III-region), respectively. Then, the adsorptions under different pressure were analyzed (Supplementary Fig. 12). Due to the higher polarizability of C_2_H_6_ (44.7 × 10^−25^ cm^3^) compared with C_2_H_4_ (42.5 × 10^−25^ cm^3^)^7^, at the beginning of the adsorption, the C_2_H_6_ molecules are preferentially filled in region I at 2.6 kPa (Supplementary Fig. 12a), but the C_2_H_4_ molecules are almost not adsorbed until 10.3 kPa (Supplementary Fig. 12e). While the pressure is 5.1 kPa, the region II begins to be filled by C_2_H_6_ molecules (Supplementary Fig. 12b), the total capacity of adsorption reaches to 18.5 cm^3^ g^−1^, which corresponds to the adsorption capacity of C_2_H_4_ at 12.8 kPa (18.9 cm^3^ g^−1^), at that moment the region II is empty until the pressure increases to 17.9 kPa (Supplementary Fig. 12f). With the increase of pressure (to 48.7 kPa), region I and region II are almost filled saturated by C_2_H_6_ molecules (Supplementary Fig. 12c), and region III begins to be filled by C_2_H_6_ already, the total capacity of adsorption reaches to 73.2 cm^3^ g^−1^. The vdW interactions between C_2_H_6_ with aromatic rings (regions I and II) and carboxylate (region III) are more remarkable than the interactions between C_2_H_4_ with them. While the pressure reaches to 100 kPa, these three regions are saturated generally by C_2_H_6_ (Supplementary Fig. 12d), whereas, the C_2_H_4_ molecules only continue to be filled into regions I and II (Supplementary Fig. 12g), the C_2_H_6_ uptake (106.2 cm^3^ g^−1^) is significantly greater than the maximum C_2_H_4_ uptake (80.2 cm^3^ g^−1^). Hence, according to the GCMC simulations, the Azole-Th-1 prefers to adsorbing the C_2_H_6_ from 0.001 to 100 kPa at 298 K, and the C_2_H_6_ adsorption capacity is far greater than C_2_H_4_, which could achieve the separation of C_2_H_6_ and C_2_H_4._

And then, according to the obtained adsorption regions, some DFT theoretical calculations about the mechanism of selective C_2_H_6_/C_2_H_4_ in Azole-Th-1 were investigated by the Dmol^3^ program package^68^ in the MS, the detail calculations were also presented in Supplementary Information. The determination of the adsorption point can quantify the interaction between the gas C_2_H_6_/C_2_H_4_ and Azole-Th-1 and analyze the mechanism of the gas adsorption. Because the calculations using the whole unit cell is too large, we used the fragmented cluster models cleaved from the unit cell for modeling the structures and energies to investigate the interaction points of C_2_H_6_/C_2_H_4_ adsorption. Due to the highly disordered structure of TBA, four fragment models were constructed. Accordingly, the fragment M1 to M4 were intercepted (Supplementary Fig. 13). Based on the distribution of density of C_2_H_6_/C_2_H_4_ through GCMC simulations, the geometries of fragmented models bound to C_2_H_6_/C_2_H_4_ were also obtained, as shown in Supplementary Fig. 14. By the calculations of binding energy for adsorbed geometries (Supplementary Table 5), obviously, the interactions between M1 to M4 with C_2_H_6_ (−43.09 kJ mol^−1^) were stronger than the interactions between that of fragmented models with C_2_H_4_ (−33.52 kJ mol^−1^), which results were in agreement with the heat of adsorption *Q*_st_. These can be attributed to the vdW interaction between the C–H in C_2_H_6_/C_2_H_4_ and conjugated π-systems in Azole-Th-1, especially, the conjugated region II (tetrazol).

In our opinion, there are three factors for the stabilities of adsorbed structures, the number of H atoms, the distances of vdW interaction, and the polarizability, where the summary about previous two factors were listed in Supplementary Tab. 6. As shown in Supplementary Fig. 14, C_2_H_6_ and C_2_H_4_ molecules are bound to several aromatic rings of ligands at three directions within a pore through vdW interactions. A single C_2_H_6_ or C_2_H_4_ molecule can form six or four pairs of C–H—*π* interactions with conjugated regions at least, where the vdW interaction between C–H and benzene ring of ligand (region I) are in the majority. The greater the number of H atoms, the stronger the C–H—*π* interaction between gas with different models, and finally the greater the adsorption capacity of C_2_H_6_ compared with C_2_H_4_.

Because of the use of previously unreported ligand, the conjugated region tetrazole can enhance the binding with gas. As shown in Supplementary Fig. 14, C_2_H_6_@M1 (all O-donors of ligands coordinated on the Th(IV) inner cluster), there are two pairs of vdW interactions between C–H with carboxylic acid (region III). However, as to C_2_H_6_@M2 (Fig. 6, all N-donor of ligands coordinated on the Th(IV) inner cluster), because the gas molecule was near to the region II, which contributed to the increasing of the interaction probability between gas and tetrazol. The four pairs of C–H—*π* interactions with region II could make the adsorption structure more stable with −46.90 kJ mol^−1^ binding energy than the structure without the interaction between C_2_H_6_ with tetrazol (C_2_H_6_@M1, −25.87 kJ mol^−1^).

And then, the average distances between C_2_H_6_ with benzene region (I), tetrazol region (II), and carboxylic acid region (III) are 3.67, 3.65, and 3.08 Å, respectively. And the average distances between C_2_H_4_ with these three regions are 3.41 and 3.25 Å (no interaction with region III), which results agreed with the GCMC results (Supplementary Fig. 12g, C_2_H_4_ only filled into regions I and II). According to these distances of interaction, it is hard to analysis the stabilization of the adsorption structures. Besides the distance and number of C–H—*π* interactions, C_2_H_6_ as a more polarizable molecule can interact more strongly by induced dipole interactions with the framework compared to the less polarizable C_2_H_4_ molecule.

### Separation of ternary mixture of C_2_H_6_/C_2_H_2_/C_2_H_4_

In traditional C_2_H_4_ production process, trace amounts of C_2_H_2_ (about 1%) also exist in the ethylene feed. So, this material was further investigated for the simultaneous capture of C_2_H_2_ and C_2_H_6_ from a ternary mixture of C_2_H_6_/C_2_H_2_/C_2_H_4_. As shown in Fig. 5d, highly efficient separation of C_2_H_4_ from a 90:1:9 (v/v/v) gas mixture of C_2_H_6_/C_2_H_2_/C_2_H_4_ was achieved by passing the mixture over a packed column of activated Azole-Th-1 material. It can be observed that C_2_H_4_ achieves a breakthrough first, with no evidence of C_2_H_2_ or C_2_H_6_ flow before its breakthrough, which indicates that this material can produce an high purity C_2_H_4_ (>99.9%) after only a single-breakthrough operation. The working capacity is up to 1.34 mmol g^−1^, far exceeding the top-performing materials reported by Lu et al. (0.69 mmol g^−1^)^49^ and Zaworoko et al. (0.32 mmol g^−1^)^38^, strongly suggesting its promising applications in this target. Note that, according to the adsorption isotherm of C_2_H_2_ (Fig. 4c), the maximum of capacities of C_2_H_2_ (81.1 cm^3^ g^−1^) and C_2_H_4_ (80.7 cm^3^ g^−1^) are almost equal to each other. In addition, the adsorption heat enthalpy of C_2_H_2_ is 25.4 kJ mol^−1^ at the zero coverage, which is lower slightly than *Q*_st_ of C_2_H_4_ (26.1 kJ mol^−1^). And why the high purity of C_2_H_4_ (>99.9%) can be acquired. Therefore, in order to explore this problem, the C_2_H_6_, C_2_H_4_, and C_2_H_2_ at low pressure region (<0.1 bar) was checked (inserted into Fig. 4c). Before pressure of 0.05 bar, the adsorption of C_2_H_2_ is obviously bigger than both C_2_H_6_ and C_2_H_4_, giving a hierarchy of C_2_H_2_>C_2_H_6_>C_2_H_4_. This is well consistent with the separation conditions of 90:1:9 (v/v/v) gas mixture of C_2_H_6_/C_2_H_2_/C_2_H_4_.

## Discussion

In a word, we reported a Th-azole framework (Azole-Th-1) by introducing an previously unreported ligand TBA showing a preferential adsorption of ethane over ethylene. Notably, Azole-Th-1 samples exhibited good stability for soaking in water, various organic solvents, and different pH (1–12) solvents about 30 days, respectively. Moreover, preferential adsorption of ethane over ethylene was confirmed by measuring the adsorption isotherms and breakthrough curves. Azole-Th-1 had relative high ethane and ethylene adsorption capacities, 4.5 and 3.6 mmol g^−1^ at 298 K and 100 kPa, respectively. The adsorption selectivity of binary mixture C_2_H_6_/C_2_H_4_(1:1, v/v) was ~1.46 at pressure below 100 kPa and 298 K. Five cycles of ethane adsorption–desorption cycle experiments revealed that Azole-Th-1 had a good regenerability. The polymer grade C_2_H_4_ with the max working capacities of 1.13 mmol g^−1^ with 99.9% purity was harvested from 10/90 gas mixture. Furthermore, Azole-Th-1 also can purify the C_2_H_4_ (purity >99.9%) from ternary mixture C_2_H_6_/C_2_H_2_/C_2_H_4_ (90:1:9, v/v/v) with working capacities of 1.34 mmol g^−1^. Some DFT calculations suggested that the greater vdW interaction between ethane and Azole-Th-1 than ethylene and material, −43.09 and −33.52 kJ mol^−1^, respectively, which were in agreement with the isosteric heat of ethane (28.6 kJ mol^−1^) and ethylene (26.1 kJ mol^−1^). In brief, these excellent properties, the perfect pH stability and high C_2_H_4_ purity (>99.9%) from a ternary 90:1:9 mixture of C_2_H_6_/C_2_H_2_/C_2_H_4_, etc., make Azole-Th-1 a promising candidate for efficient separation of ethane/ethylene. It will be much more challenging and difficult to separate more complex gas mixtures. Those small gas molecules such as N_2_ and CH_4_ will not affect the ternary C_2_H_6_/C_2_H_2_/C_2_H_4_ separation very much because of their very weak interactions with the framework, leading to very low uptakes of N_2_ and CH_4_ at the room temperature. However, some other gas molecules, particularly CO_2_, is expected to significantly affect the C_2_H_6_/C_2_H_2_/C_2_H_4_ separation, because the uptakes of CO_2_ are comparable to these C2 hydrocarbons. Until now, no suitable porous materials have been reported yet for the efficient C_2_H_6_/C_2_H_2_/C_2_H_4_/CO_2_ separation. Before any porous materials can be realized for this very challenging separation, step-by-step separations by different adsorbents will be necessary to get high purity C2 hydrocarbons.

## Methods

### The synthesis

A mixture of 4-(1H-Tetrazol-5-yl) benzoic acid (TBA, 0.019 g, 0.10 mmol), Th(NO_3_)_4_ (0.048 g, 0.10 mmol), *N,N*′-dimethylformamide (DMF, 3.0 mL), and ionic liquid of tetramethylguanidine chloride (0.015 mg, 0.1 mmol) were placed in a 20 mL screw-capped glass capped jar, then five drops of concentrated hydrochloric acid were added to the mixture. The mixture was sealed and heated at 110 °C for 3 days. The reaction system was cooled to 30 °C with about 6 °C per min cooling rate. After filtration and washed with excess of *N,N*′-dimethylacetamide (DMA), colorless block crystals were collected as a pure phase (see PXRD in Fig. 2).

### Gas-adsorption and breakthrough experiments

The original sample about 100 mg was activated at 60 °C under high vacuum for 12 h in gas adsorption apparatus before the gas adsorption measurement. The BET of the MOFs were investigated by nitrogen adsorption and desorption at 77 K using a Belsorp-max. The single-component isotherms of C_2_H_6_, C_2_H_4_, and C_2_H_2_ were collected at 298 and 273 K on a Belsorp-max. The breakthrough separation apparatus consisted of two fixed-bed stainless steel column. One column was loaded with MOF powder (1.9810 g), while the other reactor was used as a blank control group to stabilize the gas flow. The horizontal reactors were placed in a temperature-controlled environment, maintained at 298 K. The flow rates of all gases mixtures were regulated by mass flow controllers, and the effluent gas stream from the column is monitored by a gas chromatography (TCD-Thermal Conductivity Detector, detection limit 0.1%). Prior to each breakthrough experiment, we regenerated the sample by flushing the adsorption bed with helium gas (100 mL per min) for 30 min at 298 K.

## Supplementary information

Supplementary Information

Peer Review File

## Data Availability

The X-ray crystallographic coordinated for structure reported in this study has been deposited at the Cambridge Crystallographic Data Centre (CCDC), under deposition number 1969398. This data can be obtained free of charge from the CCDC via https://www.ccdc.cam.ac.uk/structures/. The source data underlying Figs. 2, 4, and 5 and Supplementary Figs. 5, 6, 8, 9, 10a, 10c, and 11 are provided as a Source Data file. And other data, if not included in the article or Supplementary Information or Source Data, are available from the authors on request.

## Source data

Source Data
