# Supplementary material for: A robust Th-azole framework for highly efficient purification of C2H4 from a C2H4/C2H2/C2H6 mixture
Source: Nat Commun. 2020 Jun 22;11:3163. doi: 10.1038/s41467-020-16960-9 (PMC7308359; doi:10.1038/s41467-020-16960-9)
Supplement: Supplementary file 1 — Supplementary Information [file 41467_2020_16960_MOESM1_ESM.pdf]

Supplementary Information

A robust Th-Azole framework for highly efficient purification of C_2H_4 from a $C_2H_4/C_2H_2/C_2H_6$ mixture

Xu et al.

Supplementary Information include:

Supplementary Method 1 to 8

Supplementary Tables 1 to 6

Supplementary Figures 1 to 14

Supplementary References 1 to 31

Supplementary Methods 1. Materials and Physical Measurements

Caution! Th-232 used in this study is an α emitter with the daughter of radioactive Ra-228. All thorium compounds used and investigated were operated in an authorized laboratory designed for actinide element studies. Standard precautions for handling radioactive materials should be followed. The reagents and solvents were commercially available and were used as received without further purification, where the ligand 4-(1H-Tetrazol-5-yl) benzoic acid ($C_8H_6N_4O_2$, 95%) from EXTENSION Technology Co., Ltd was purchased and directly used, Thorium nitrate hydrate ($N_4O_{12}Th$, Aladdin), Tetramethylguanidine chloride ($C_5H_{14}N_3Cl$, 98%, Aladdin), hydrochloric acid (HCl, Aladdin), and solvents (N, N'-dimethylformamide and N, N'-dimethylacetamide, HPLC grade of 99.9%) from Aladdin Chemistry Co. Ltd were also purchased and directly used.

The optical images were accepted from Optec SZ810 microscope. Thermogravimetric analysis (TG) was performed by a TGA Q600 thermal analysis system. All TG experiments were performed under a N_2 atmosphere from room temperature to 800 °C at a rate of 2°C/min. Data were analyzed using the TA Universal Analysis software package. X-ray powder diffraction were collected by a Bruker AXS D8 Discover powder diffractometer at 40 kV, 40 mA for Cu Kα (λ = 1.5406 Å) at room temperature in the range of 5-50 degree (2 theta) with a scan speed of 0.1 degree per step. The gas sorption isotherms were collected on a Belsorp-max. Ultrahigh-purity-grade (> 99.999%) N_2, C_2H_6, C_2H_4, and C_2H_2 gases were used in this adsorption measurement. To maintain the experimental temperatures, liquid nitrogen (77 K) and a temperature-programmed water bath (273 and 298 K) were used, respectively.

Supplementary Methods 2. Powder X-ray diffraction (PXRD)

For PXRD measurements, the samples were always kept damp with solvent prior to and during measurements. The two-dimensional image of the Debye rings were integrated with 2 Dp to give 2 theta ~ intensity diffractograms. Predicted powder patterns were generated from single crystal structures using Mercury. Powder diffraction patterns, as shown in the following figure revealed that the structure of Azole-Th-1 remains unchanged after soaked in ethanol, acetone, tetrahydrofuran, pure water, methanol cyclohexane, trichrolomethane, and dichrolomethane, respectively (Fig. 2b). The thermal stabilities from 100 to 400°C were also tested, which results indicated that the structure of Azole-Th-1 began to be destroyed after 20,0 °C (Fig. 2a). The PXRD of soaking in the solvents with the range of pH from 1 to 12 were tested (Fig. 2c). Furthermore, the PXRD before and after breakthrough experiments is also compared in Supplementary Fig. 11.

Supplementary Methods 3. Structure, physical properties and pore shape of Azole-Th-1

The Zeo++ code[1, 2]were used to characterize the geometric features of the crystal structure of Azole-Th-1 by calculating the pore volume with the use of N_2 probe molecule, the pore limiting diameter (i.e., the diameter of smallest pore window), the largest cavity diameter (i.e., the diameter of the largest sphere that can fit

within the pores), and the surface area accessible to a N_2 probe using the coordinated found by X-ray crystallography. The Accelrys Materials Studio (MS) 8.0 software package[3, 4, 5] was used to visualize the MOF structure and pore topology.

Supplementary Methods 4. Calculation of isosteric heat of adsorption

The binding energy is reflected in the isosteric heat of adsorption, Q_{st}, is calculated from the Clausius-Clapeyron equation, as shown in Supplementary Equation 1,

$$Q_{\mathrm{st}} = -RT^2\left(\frac{\partial \ln p}{\partial T}\right)_q \qquad (1)$$

where p is the pressure, T is the temperature, R is the gas constant (8.314 J mol^{-1} K^{-1}). By drawing the ln p vs $1/T$ plot of gas at various loadings, Q_{st} = -slope$\times R$. To extract the coverage-dependent isosteric heat of adsorption, the data were modeled with a virial-type expression[6, 7] composed of parameters a_i and b_i that are independent of temperature:

$$\ln(p) = \ln(N) + \frac{1}{T}\sum_{i=0}^{m} a_i N_i + \sum_{i=0}^{n} b_i N_i \qquad (2)$$

$$Q_{\mathrm{st}} = -R\sum_{i=0}^{m} a_i N_i \qquad (3)$$

where N is the amount adsorbed (or uptake), m and n determine the number of terms required to adequately describe the isotherm. The isosteric heat of adsorption is calculated according to Supplementary Equation 3. The coverage dependencies of Q_{st} calculated from fitting the 273 and 298 K data are presented graphically in Fig. 4d, and the virial equation fit for C_2H_6, C_2H_4, and C_2H_2 adsorption isotherms of Azole-Th-1 are shown in Supplementary Fig. 7.

Supplementary Methods 5. Calculation of selectivity via ideal adsorption solution theory (IAST)

The adsorption selectivity of C_2H_6/C_2H_4 (50/50, 10/90, 1/15, v/v) in Azole-Th-1 was established by using the Ideal Adsorption Solution Theory (IAST). The adsorption selectivity for C_2H_6/C_2H_4 separation is defined by,

$$S_{\mathrm{ads}} = \frac{x_{\mathrm{C_2H_6}}/x_{\mathrm{C_2H_4}}}{y_{\mathrm{C_2H_6}}/y_{\mathrm{C_2H_4}}} \qquad (4)$$

where, $x_{\mathrm{C_2H_6}}$ and $x_{\mathrm{C_2H_4}}$ are the equilibrium adsorption capacity of component C_2H_6 and component C_2H_4, respectively, and $y_{\mathrm{C_2H_6}}$ and $y_{\mathrm{C_2H_4}}$ are the molar fractions of component C_2H_6 and component C_2H_4 in the gas phase.

Fitting details: The adsorption data for C_2H_6, C_2H_4, and C_2H_2 in Azole-Th-1 at 273 K and 298 K were fitted with the single-site Langmuir model,

$$q = q_{\text{sat}} \frac{bp^{\nu}}{1+bp^{\nu}} \qquad (5)$$

with *T*-dependent parameters *b*,

$$b = b_0 \exp\left(\frac{E}{RT}\right) \qquad (6)$$

where the single-site Langmuir parameters for C_2H_6, C_2H_4, and C_2H_2 are provided in Supplementary Tab. 4.

Supplementary Methods 6. Computational calculations

Density Functional Theory calculations: DFT calculations were performed to provide the optimized structures and energies of C_2H_6/C_2H_4 interaction with the frameworks of Azole-Th-1. The Perdew-Burke-Ernzerhof (PBE) function[8, 9] under the generalized gradient approximation (GGA) functional with the double-ξ numerical polarization (DPN) basis set was used by Dmol^3 program package[10] in the MS of Accelrys[3, 4, 5]. Since the calculations using the whole unit cell too large, we used fragmented cluster models cleaved from the unit cells for modeling the partial charges, structures, and energies, the smaller fragmented models were used as presented in Supplementary Fig. 13. The boundaries of models were saturated by H_2O molecules. The tolerances of energy, gradient and displacement convergence were, 2×10^{-4} hartree, 4×10^{-3} hartree Å^{-1}, and 5×10^{-3} Å, respectively. The binding energy (ΔE_{bind}) for C_2H_6/C_2H_4 with models was calculated by Supplementary Equation 7,

$$\Delta E_{\text{bind}} = E_{\text{complex}} - E_{\text{gas}} - E_{\text{MOF(model)}} \qquad (7)$$

where, E_{complex}, E_{gas}, and $E_{\text{MOF (model)}}$ are the total energies of complex of gas with model, single C_2H_6/C_2H_4 gas, and MOF model at the optimized geometries, respectively.

Grand Canonical Monte Carlo (GCMC) Simulations: The GCMC simulations, which were performed by Sorption code[4, 11] in MS software[3, 4, 5], were carried out to investigate on the adsorbed capacity of Azole-Th-1 for C_2H_6/C_2H_4 at 298 K from 0.001 to 100 kPa. A simulation box of 1×1×1 crystallographic unit cell was used. During the simulations, 4×10^{6} steps were performed to guarantee the equilibration and to sample the desired properties, respectively. Rigid framework assumption was used in all simulations. The Dreiding forcefield parameter[12] was used to describe the interactions, the van der Waals interaction with a cutoff of 15.5 Å were depicted by Lenard-Jones 12-6 potential.

Supplementary Methods 7. Thermogravimetric analysis

Thermogravimetric analysis (TG) was performed by a TGA Q600 thermal analysis system. All TG experiments were performed under a N_2 atmosphere from room temperature to 800 °C at a rate of 2 °C /min.

Supplementary Methods 8. Breakthrough curve simulations

The performance of industrial fixed bed adsorbers is dictated by a combination of adsorption selectivity and uptake capacity. Transient breakthrough simulations were carried out for 50/50, 90/10, and 15/1 binary C_2H_4(1)/C_2H_6(2) mixtures and 9/1/90 ternary C_2H_6/C_2H_2/C_2H_4 mixture in Azole-Th-1 operating at a total pressure of 100 kPa and 298 K, using the methodology described in earlier publications.[13, 14, 15, 16] The numerical details of the code implementation are provided online by Krishna and Baur.[17] For the breakthrough simulations, the following parameter values were used: length of packed bed, L = 0.3 m; voidage of packed bed, ε = 0.4; superficial gas velocity at inlet, u = 0.04 m s^{-1}.

The Supplementary Fig. 9 shows result of the breakthrough calculations. The y-axis is the dimensionless concentrations of each component at the exit of the fixed bed, normalized with respect to the inlet feed concentrations. The *x*-axis is the dimensionless time, $\tau = {tu}/{L\varepsilon}$, defined by dividing the actual time, *t*, by the characteristic time, ${L\varepsilon}/{u}$.

Notation

b	Langmuir-Freundlich constant, $\mathrm{Pa}^{-\nu}$
q	component molar loading of species i, mol kg^{-1}
q_{sat}	saturation loading, mol kg^{-1}
L	length of packed bed adsorber, m
t	time, s
T	absolute temperature, K
u	superficial gas velocity in packed bed, m s^{-1}
p	pressure, Pa
R	gas constant, 8.314 J mol^{-1} K^{-1}

Greek letters

ε	voidage of packed bed, dimensionless
ν	Freundlich exponent, dimensionless
τ	time, dimensionless

Supplementary Table 1. Crystal data of disorder Azole-Th-1.

	Azole-Th-1	
Formula	$Th_6O_4(OH)_4(H_2O)_6(TBA)_6$	
Empirical formula	$C_{24}H_{12}N_{12}O_{13}Th_3$	
Formula weight	1372.58	
Temperature (K)	293	
Wavelength (Å)	0.71073	
Crystal system	Cubic	
Space group	$Fm\bar{3}m$	
	a=23.9839	
Unit cell dimensions (Å)	b=23.9839	
	c=23.9839	
Cell Volume (Å^3)	13796.2	
Density	1.322	
Theta range for data collection (degree)	3.4-24.97	
	α=90.000	
Crystal parameters(degree)	β=90.000	
	γ=90.000	
Z	8	
F(000)	4912	
Crystal size (mm)	0.10×0.10×0.08	
GOF	1.084	
Final R indices [I> 2 sigma (I)]	R1=0.0199	wR1=0.0512
Final R indices [all]	R1=0.0225	wR1=0.0532

Supplementary Table 2. A summary of reported porous adsorbents for C_2H_6/C_2H_4 separation at 1bar and 298 K.

	C_2H_6/C_2H_4 Uptakes (mmol g^{-1})	C_2H_6/C_2H_4 Selectivity (50/50)	Q_{st} (kJ mol^{-1})
$Fe_2(O_2)$(dobdc)[18]	3.3/2.5	4.4	66.8/38
MAF-49[19]	1.70/1.65	2.7	60/48
IRMOF-8[18, 20]	4.8/3.4	1.6	52.5/50.5
ZIF-8[21]	3.5/1.8	1.99	22.2/16.3
ZIF-7[22, 23, 24]	2.24/2.2	1.75	27.3/24.7
ZIF-3[21]	6.0/5.5	2.22	28.5/23.8
PCN-250[25]	5.2/4.1	1.9	23.6/21.1
Ni(bdc)$(ted)_{0.5}$[26]	5/3.2	2.0	21.5/18.3
MUF-15[27]	4.7/4.2	1.95	29.2/28.2
PCN-245[28]	3.3/2.4	1.75	23/20.5
MIL-142A[29]	3.8/2.9	1.5	27.3/26.2
$Cu(Qc)_2$[30]	1.85/0.78	3.75	29/25.4
Zn-atz-ipa[31]	1.76/1.75	2.0	45.8/40
**Our MOF**	**4.5/3.6**	**1.46**	**28.6/26.1**

Supplementary Table 3. Some calculated and experimentally determined structural characteristics of Azole-Th-1.

	Azole-Th-1
Geometric surface area calculated (Zeo++)	283.27 m^2 g^{-1}
BET surface area from experimental N_2 isotherm /77 K	982.97 m^2 g^{-1}
Pore volume calculated (Zeo++)	0.11 cm^3 g^{-1}
Pore volume from experimental N_2 isotherm /77 K	0.41 cm^3 g^{-1}
the largest cavity diameter (LCD) calculated(Zeo++)	10.28 Å

Supplementary Table 4. Langmuir-Freundlich parameter fits for C_2H_2, C_2H_4, and C_2H_6 in Azole-Th-1.

	q_{sat}<br>mol kg^{-1}	b_0<br>Pa$^{-\nu}$	E<br>kJ mol^{-1}	ν<br>dimensionless
C_2H_2	11.0	2.260E-8	19.2	0.79
C_2H_4	6.8	4.932E-10	24.8	1.00
C_2H_6	6.2	7.649E-12	32.0	1.18

Supplementary Table 5. Energies of single models and respective complexes and the binding energies for the models bonded C_2H_6/C_2H_4

	E_{MOF} (Hartree)	$E_{MOF-C2H6}$ (Hartree)	$E_{MOF-C2H4}$ (Hartree)	$\Delta E_{bind}(C_2H_6)$ (kJ mol^{-1})	$\Delta E_{bind}(C_2H_4)$ (kJ mol^{-1})
M1	-3443.372	-3523.109	-3521.870	-25.87	-13.33
M2	-3443.374	-3523.119	-3521.885	-46.90	-46.06
M3	-3443.377	-3523.121	-3521.886	-42.91	-41.69
M4	-3443.375	-3523.124	-3521.881	-56.69	-33.01
Average				-43.09	-33.52

Supplementary Table 6. The number of vdW interactions between C-H of C_2H_6/C_2H_4 with different regions in models

	M1		M2		M3		M4		**All (C_2H_6)**	**All (C_2H_4)**
	C_2H_6	C_2H_4	C_2H_6	C_2H_4	C_2H_6	C_2H_4	C_2H_6	C_2H_4		
I region	4(3.70)	4(3.30)	4(3.63)	2(3.55)	4(3.73)	4(3.45)	4(3.60)	3(3.33)	**16(3.67)**	**13(3.41)**
II region	0	0	4(3.50)	3(3.50)	2(3.35)	0	2(4.10)	1(3.00)	**8(3.65)**	**4(3.25)**
III region	2(2.95)	0	0	0	1(3.20)	0	0	0	**3(3.08)**	**0**
**All**	**6(3.45)**	**4(3.30)**	**8(3.57)**	**5(3.52)**	**7(3.43)**	**4(3.45)**	**6(3.77)**	**4(3.33)**		

Noted: the distances of interaction also listed in the brackets, the unit is Å.

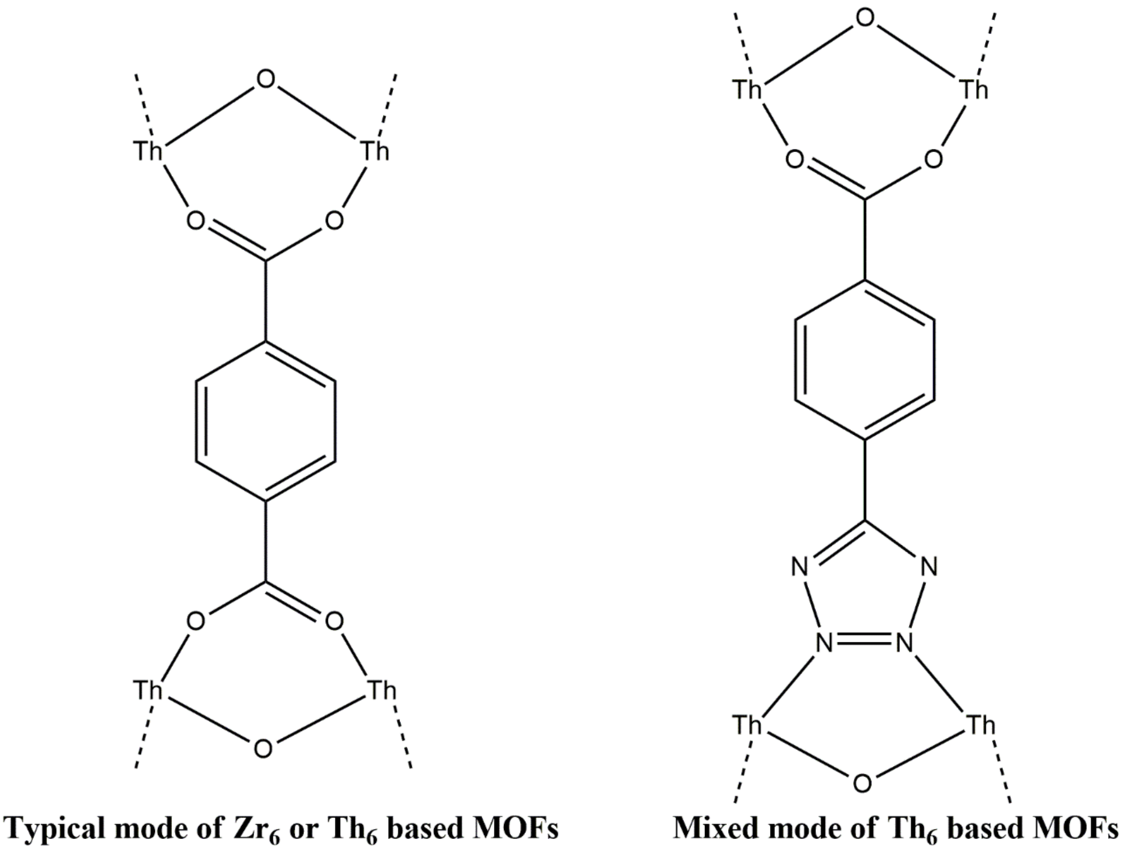

Supplementary Figure 1. Two coordination modes. The modes of Zr(IV) or Th(IV) based MOFs using the carboxylic acid ligand (typical coordination mode) and azole ligand (mixed coordination mode), respectively.

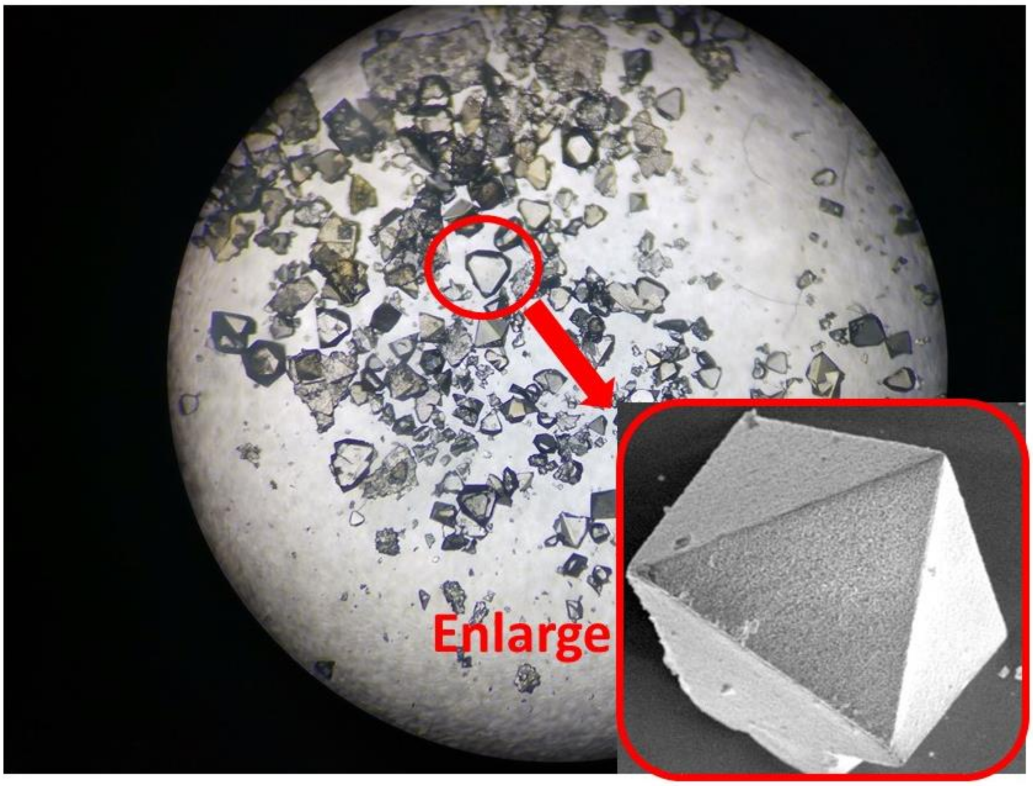

Supplementary Figure 2. Enlarged drawing of optical microscope images of Azole-Th-1 samples. The obvious octahedral crystal was obtained.

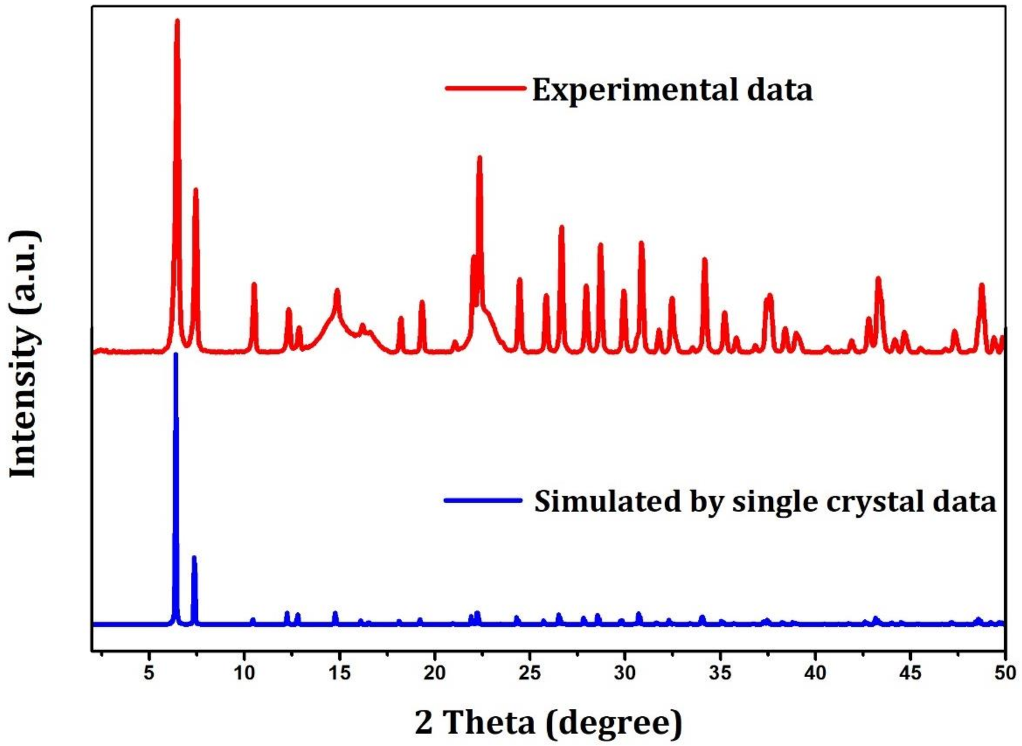

Supplementary Figure 3. **The PXRD patterns of Azole-Th-1 sample.** The experimental results of as-synthesized samples and the simulation from single crystal. Source data are provided as a Source Data file.

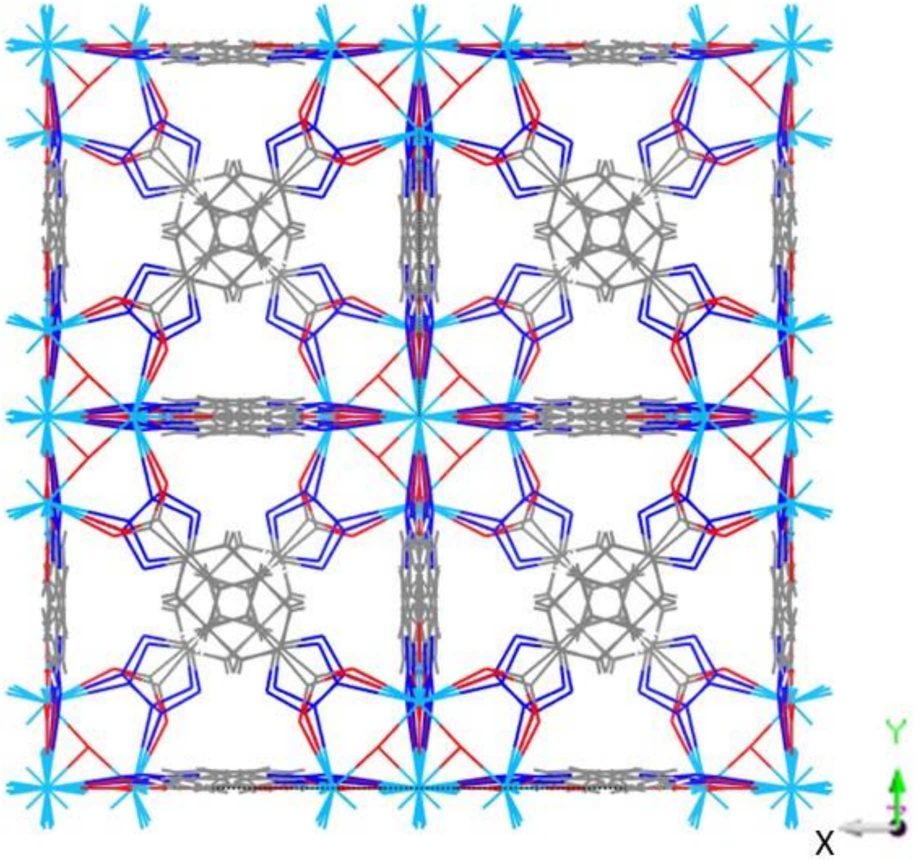

Supplementary Figure 4. **The crystal structure of Azole-Th-1 by the disordered ligand TBA**.

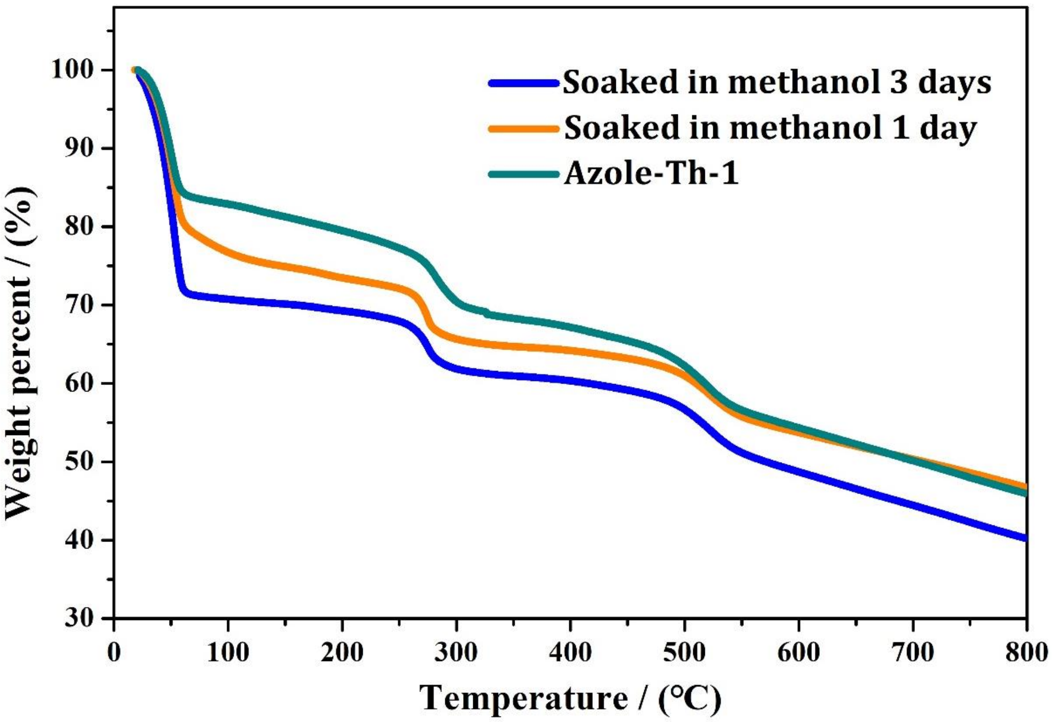

Supplementary Figure 5. The TG analysis. The as-synthesized Azole-Th-1 samples, and the samples soaked in methanol for three days and one day under N_2 atmosphere by 2°C per min of ramp rate. The loss of major trapped solvent DMF from Azole-Th-1 sample is before 75℃, the plateau region is descending slightly due to the incomplete loss of DMF molecules. And then, the plateau region is still not obvious for sample soaked in methanol one day, which indicates that the solvent exchange is still incomplete. After the sample soaked in methanol three days, the clear plateau region appears, the solvent exchange is complete. Source data are provided as a Source Data file.

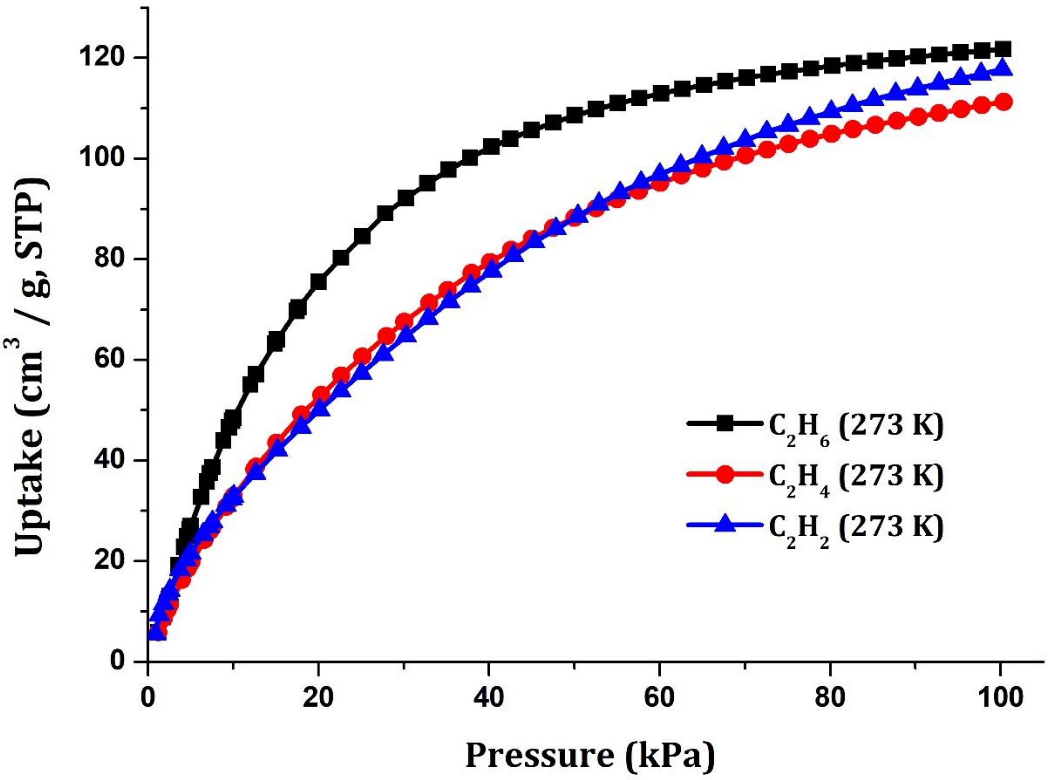

Supplementary Figure 6. The adsorption isotherms of Azole-Th-1. The experimental adsorption isotherms of Azole-Th-1 for C_2H_6, C_2H_4 and C_2H_2 at 273 K from 0.001 to 100 kPa. Source data are provided as a Source Data file.

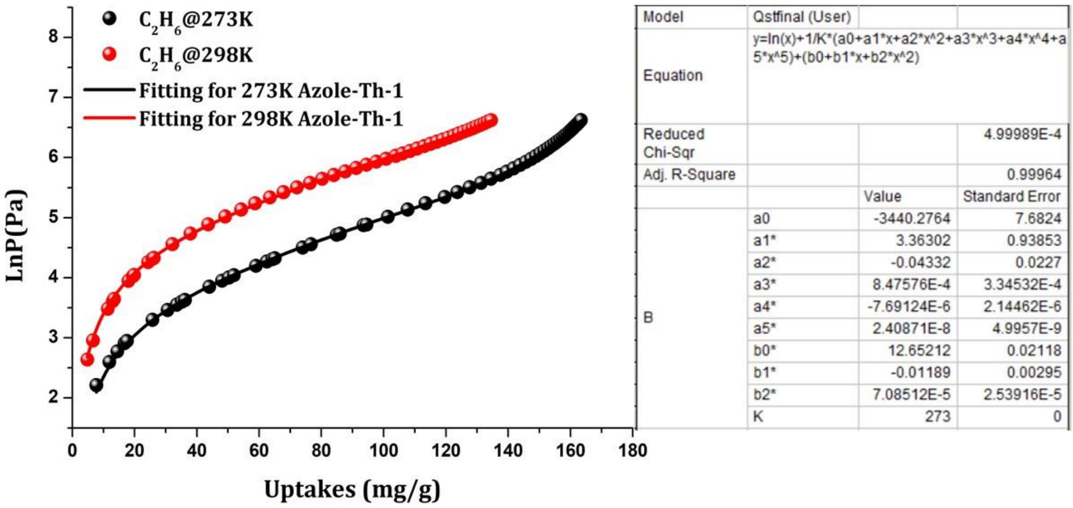

Model	Qstfinal (User)		
Equation	y=ln(x)+1/K*(a0+a1*x+a2*x^2+a3*x^3+a4*x^4+a5*x^5)+(b0+b1*x+b2*x^2)		
Reduced Chi-Sqr			4.99989E-4
Adj. R-Square			0.99964
		Value	Standard Error
B	a0	-3440.2764	7.6824
	a1*	3.36302	0.93853
	a2*	-0.04332	0.0227
	a3*	8.47576E-4	3.34532E-4
	a4*	-7.69124E-6	2.14462E-6
	a5*	2.40871E-8	4.9957E-9
	b0*	12.65212	0.02118
	b1*	-0.01189	0.00295
	b2*	7.08512E-5	2.53916E-5
	K	273	0

(a)

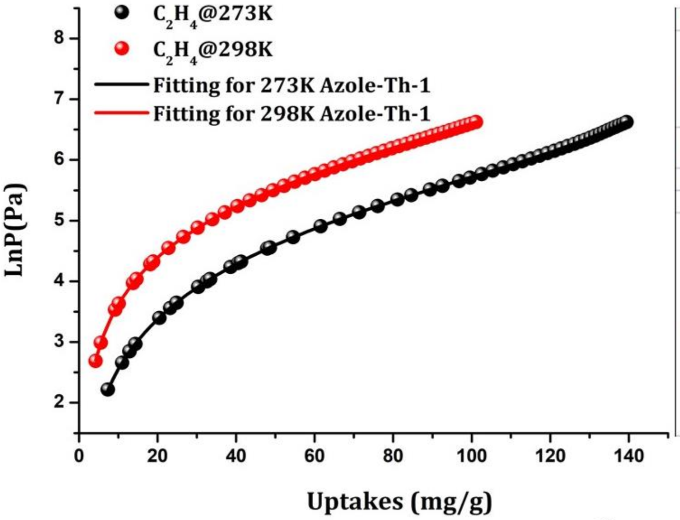

Model	Qstfinal (User)		
Equation	y=ln(x)+1/K*(a0+a1*x+a2*x^2+a3*x^3+a4*x^4+a5*x^5)+(b0+b1*x+b2*x^2)		
Reduced Chi-Sqr			8.28979E-6
Adj. R-Square			0.99999
		Value	Standard Error
B	a0	-3148.3776	10.8867
	a1*	17.29921	0.26691
	a2*	-0.13963	0.00465
	a3*	6.5908E-4	7.61471E-5
	a4*	-4.7508E-6	5.78482E-7
	a5*	1.57899E-8	1.59597E-9
	b0*	11.62162	0.00355
	b1*	-0.04569	8.57449E-4
	b2*	3.11847E-4	7.85013E-6
	K	273	0

(b)

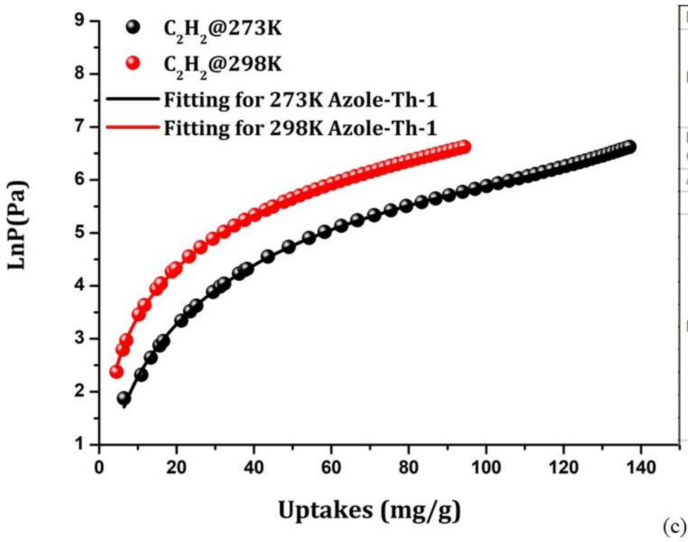

Model	Qstfinal (User)		
Equation	y=ln(x)+1/K*(a0+a1*x+a2*x^2+a3*x^3+a4*x^4+a5*x^5)+(b0+b1*x+b2*x^2)		
Reduced Chi-Sqr			5.97222E-4
Adj. R-Square			0.9996
		Value	Standard Error
B	a0*	-3958.54907	67.77544
	a1*	31.10764	3.07554
	a2*	-0.24712	0.04306
	a3*	8.23674E-4	6.66452E-4
	a4*	-3.0511E-6	5.21375E-6
	a5*	7.83309E-9	1.4774E-8
	b0*	14.0957	0.23212
	b1*	-0.07148	0.01039
	b2*	4.30216E-4	9.74578E-5
	K	273	0

(c)

Supplementary Figure 7. The virial equation fitting curves for adsorption isotherms of Azole-Th-1. (a) C_2H_6, (b) C_2H_4, and (c) C_2H_2.

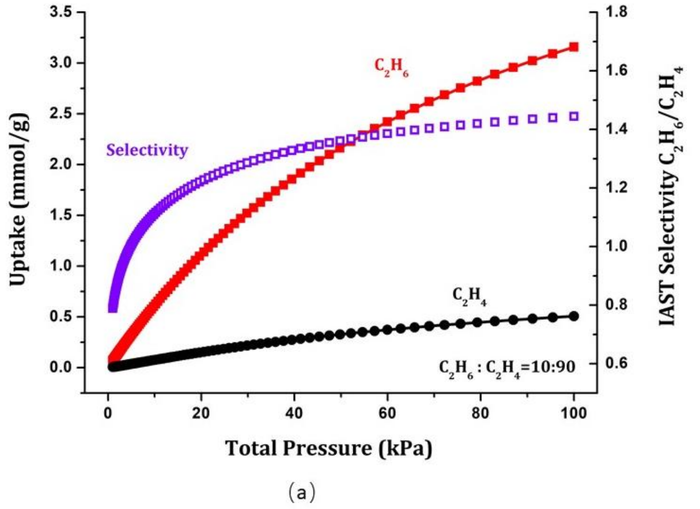

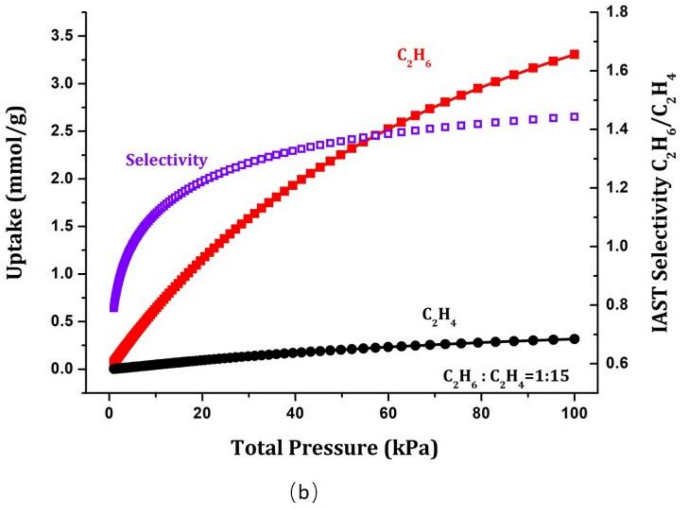

Supplementary Figure 8. Predicted mixture adsorption isotherms and selectivity by IAST method. (a) 10/90 and (b) 1/15 C_2H_6/C_2H_4 mixture at 298 K . Source data are provided as a Source Data file.

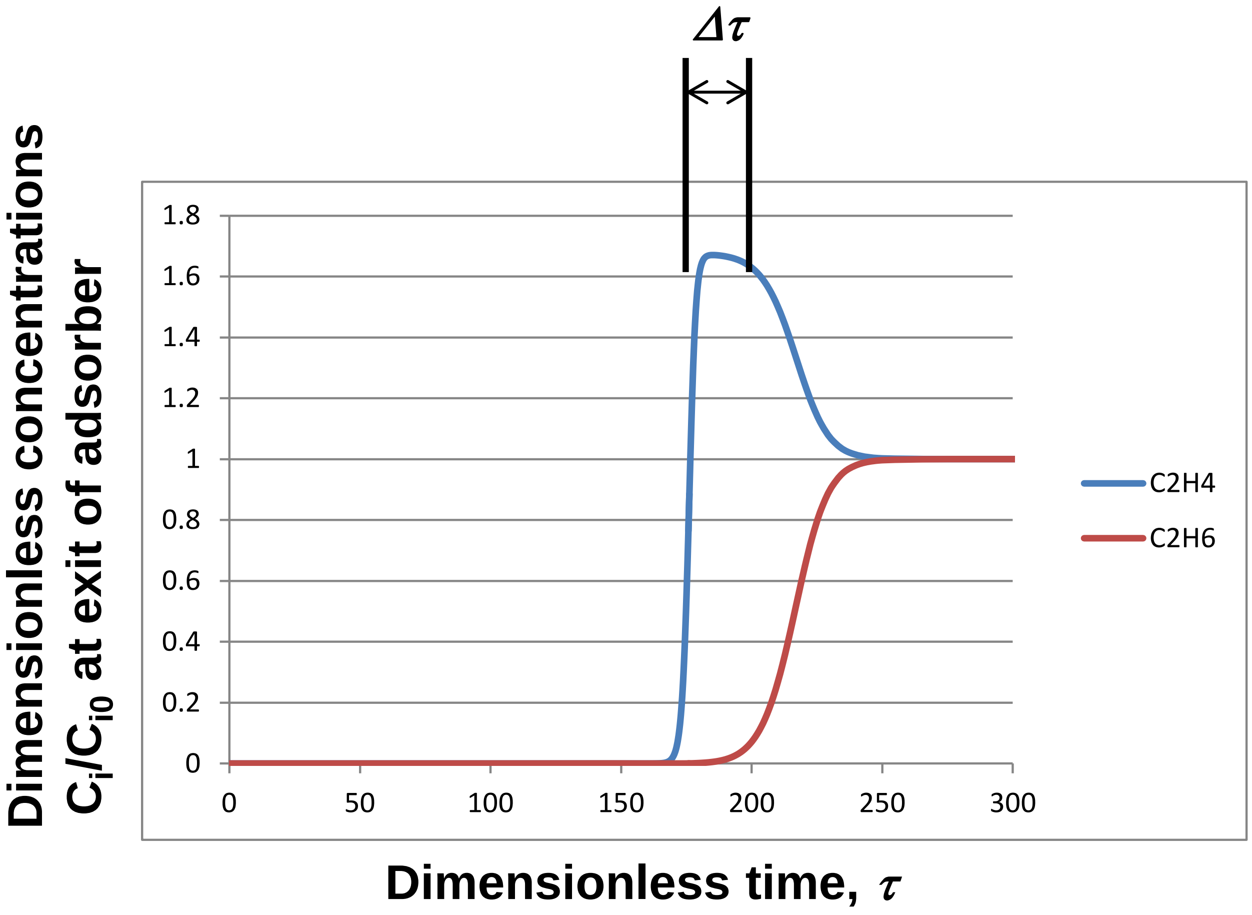

Supplementary Figure 9. The simulated transient breakthrough curve for Azole-Th-1. C_2H_6/C_2H_4 (50:50, v/v) binary mixture at 298 K and 1 bar. Source data are provided as a Source Data file.

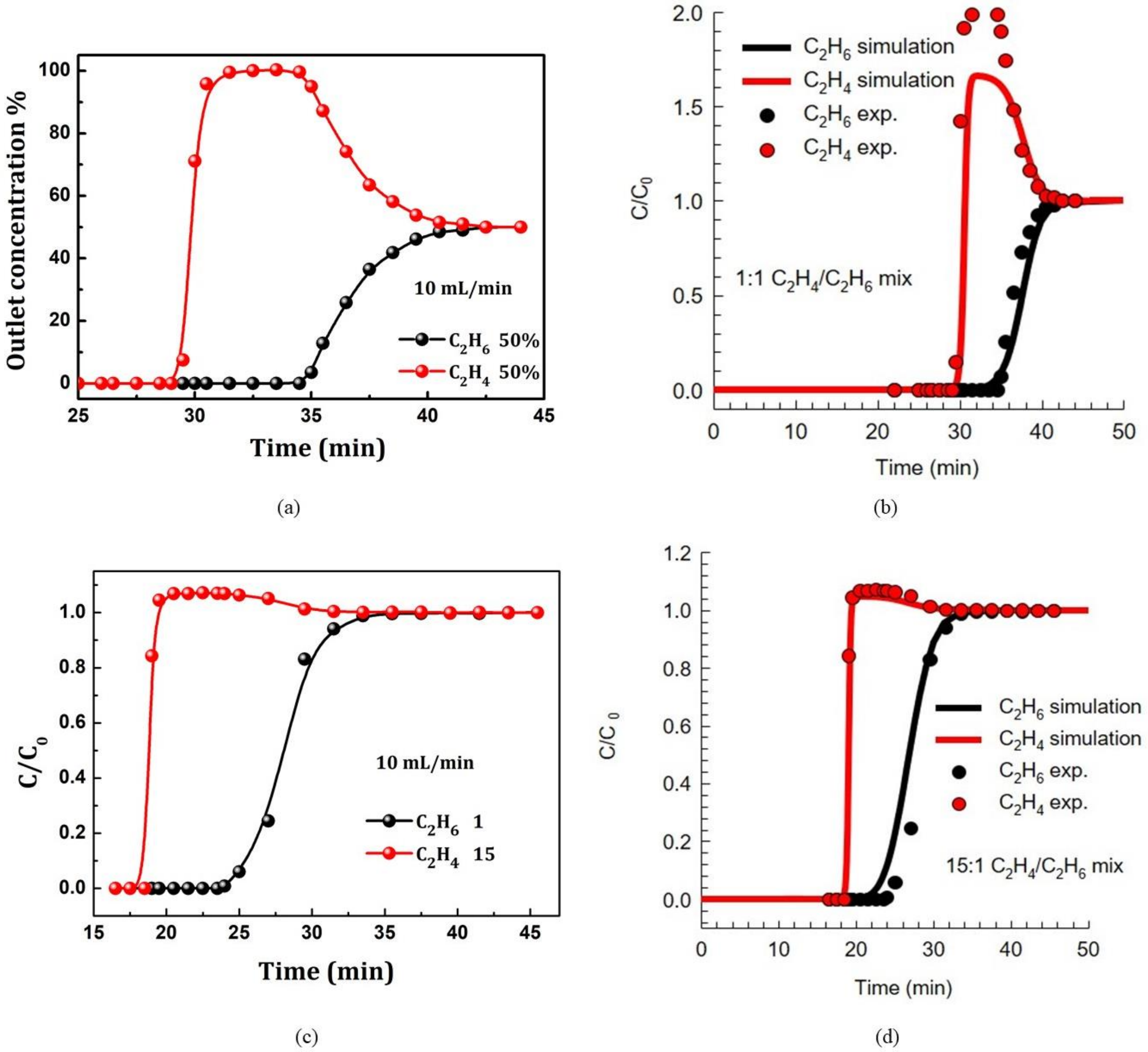

Supplementary Figure 10. The experimental breakthrough curves for Azole-Th-1 at 298 K and 1 bar. (a) and (b) C_2H_6/C_2H_4 (50/50, v/v) and (c) and (d) C_2H_6/C_2H_4 (1/15, v/v) binary mixture. Source data are provided as a Source Data file.

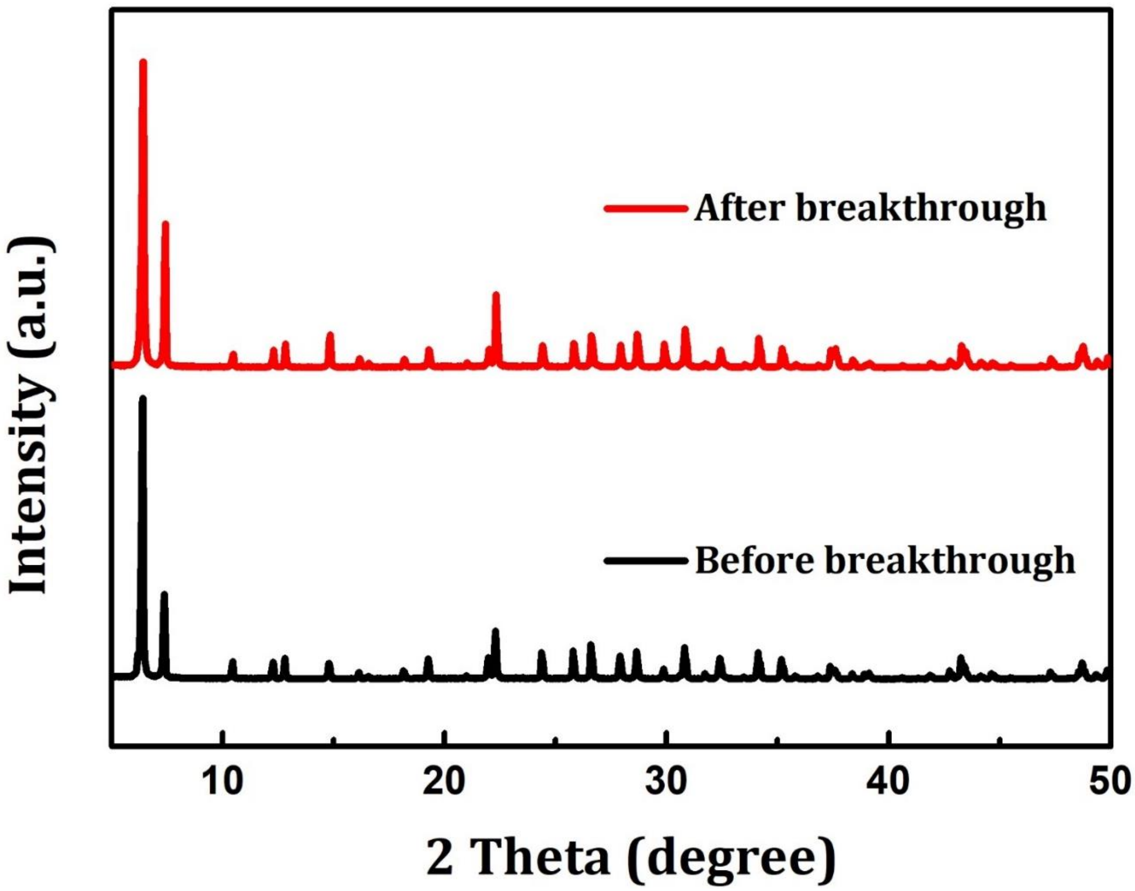

Supplementary Figure 11. The PXRD patterns of Azole-Th-1 samples. Before and after breakthrough experiments for binary mixture C_2H_6/C_2H_4. Source data are provided as a Source Data file.

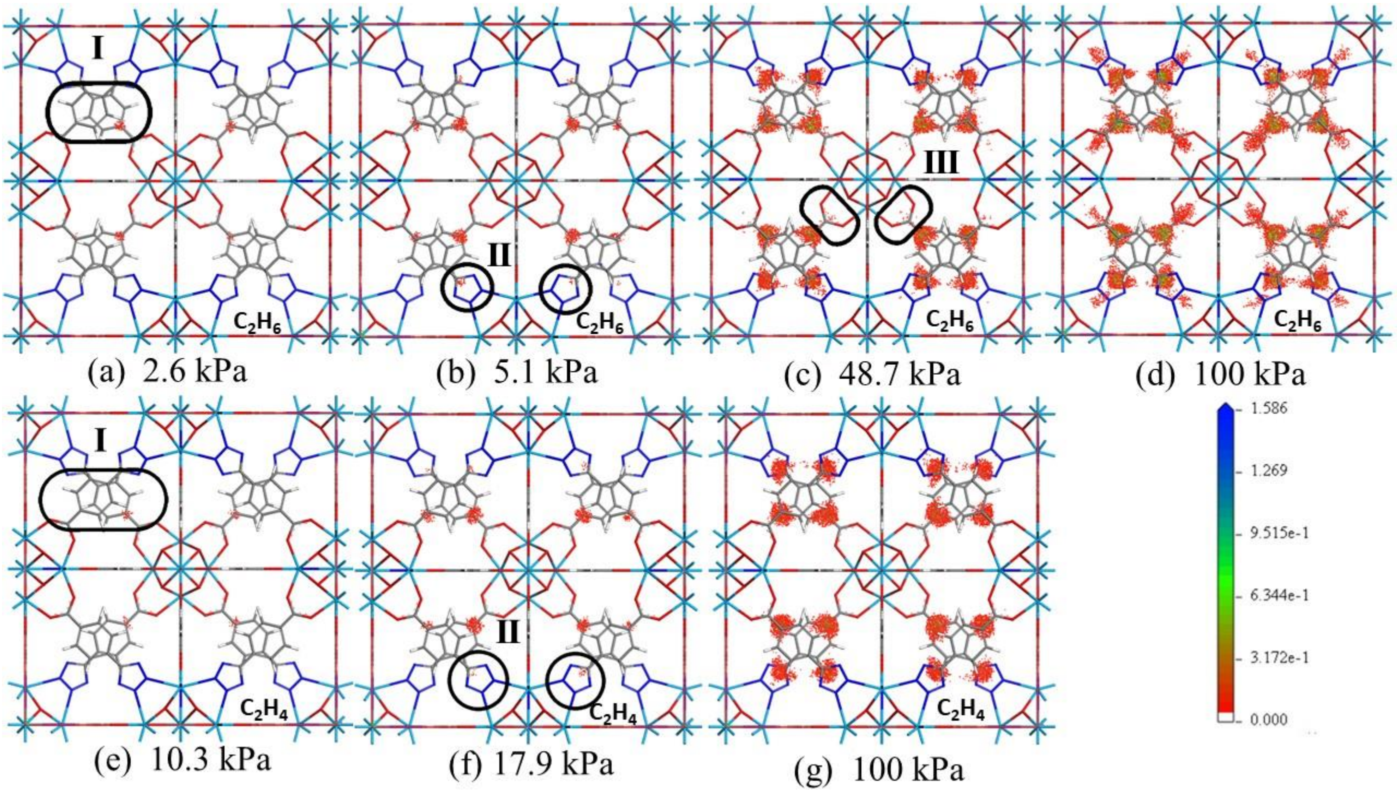

Supplementary Figure 12. The density distribution of C_2H_6/C_2H_4 through GCMC simulations at 298 K. (a) 2.6 kPa, (b) 5.1 kPa, (c) 48.7 kPa, and (d) 100 kPa for C_2H_6, and (e) 10.3 kPa, (f) 17.9 kPa, and (g) 100 kPa for C_2H_4. Where, Th-light blue, O-red, C-grey, N-blue, and H-white, the 'color dots' denoted the size of density distribution according to the color bar.

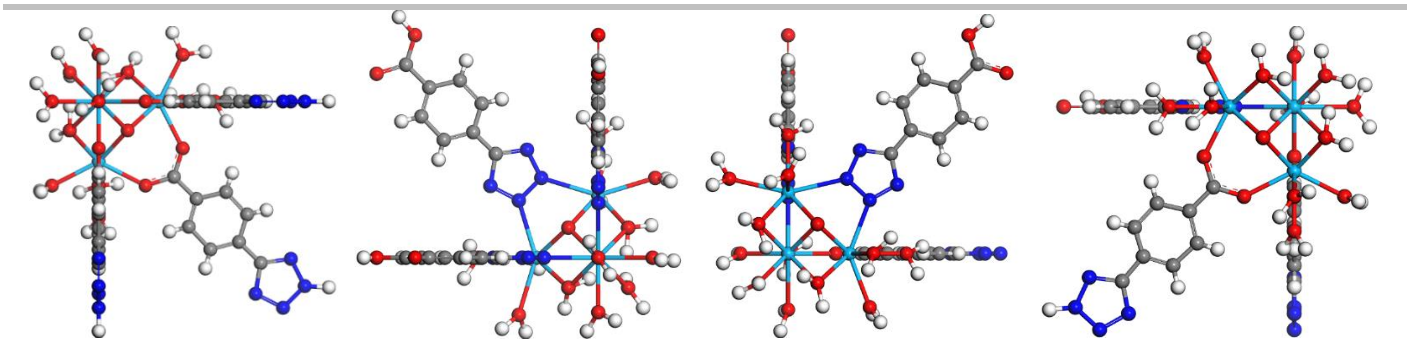

M1 M2 M3 M4

Supplementary Figure 13. The optimized four fragmented models M1 to M4. Where, Th-light blue, O-red, C-grey, N-blue, and H-white.

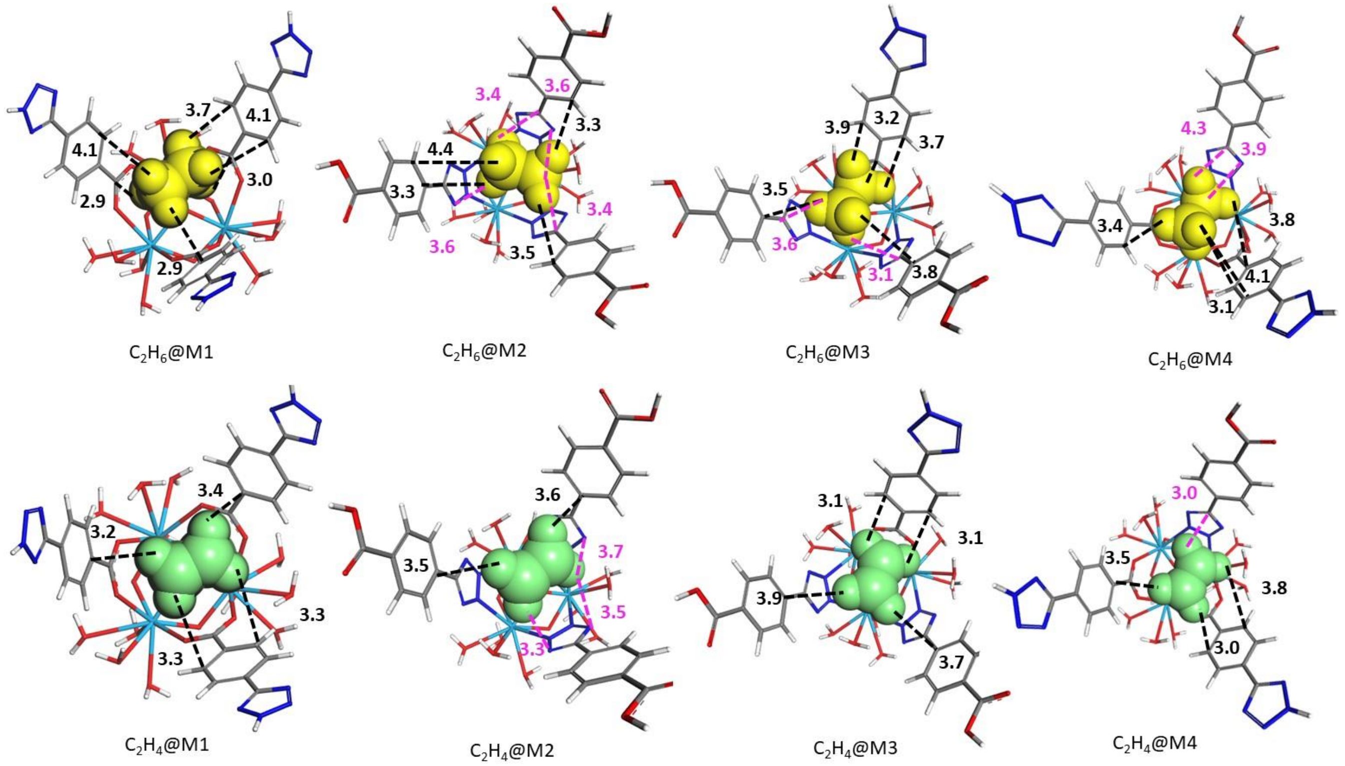

Supplementary Figure 14. The adsorbed structures for C_2H_6/C_2H_4@M1 to M4. Where, C_2H_6-yellow molecule, C_2H_4-green molecule, Th-light blue, O-red, C-grey, N-blue, and H-white, and the unit of distance is Å.

Supplementary References

1. Willems TF, Rycroft CH, Kazi M, Meza JC, Haranczyk M. Algorithms and tools for high-throughput geometry- based analysis of crystalline porous materials. *Microporous Mesoporous Mater* **149**, 134-141 (2012).

2. Martin RL, Smit B, Haranczyk M. Addressing challenges of identifying geometrically diverse sets of crystalline porous materials. *J Chem Inf Model* **522**, 308-318 (2012).

3. Material Studio.). Versiono 8.0 edn. Accelrys Inc. (2014).

4. Allen MP, Tildesley DJ. *Computer Simulation of Liquids*. Clarendon Press (1987).

5. Frenkel D, B S. *Understanding Molecular Simulation: From Algorithms to Applications*. Academic Press (2002).

6. Czepirski L, Jagiello J. Virial-type thermal equation of gas—solid adsorption. *Chem Eng Sci* **44**, 797-801 (1989).

7. Rowsell JLC, Yaghi OM. Effects of functionalization, catenation, and variation of the metal oxide and organic linking units on the low-pressure hydrogen adsorption properties of metal-organic frameworks. *J Am Chem Soc* **128**, 1304-1315 (2006).

8. Perdew JP, Burke K, Ernzerhof M. Generalized gradient approximation made simple. *Phys Rev Lett* **77**, 3865-3868 (1996).

9. Grimme S, Ehrkich S, Goerigk L. Effect of the damping function in dispersion corrected density functional theory. *J Comput Chem* **32**, 1456-1465 (2011).

10. Delley B. From molecules to solids with the $Dmol^3$ approach. *J Chem Phys* **113**, 7756 (2000).

11. Bezus AGK, A. V.; Lopatkin, A. A.; Pham, Q. D. . *J Chem Soc, Faraday Trans 2,* **74,** , 367 (1978).

12. Mayo SL, Olafson BD, III WAG. *J Phy Chem* **94**, 8897-8909 (1990).

13. Krishna R. The Maxwell-Stefan Description of Mixture Diffusion in Nanoporous Crystalline Materials. *Microporous Mesoporous Mater* **185**, 30-50 (2014).

14. Krishna R. Methodologies for Evaluation of Metal-Organic Frameworks in Separation Applications. *RSC Advances* **5**, 52269-52295 (2015).

15. Krishna R. Screening Metal-Organic Frameworks for Mixture Separations in Fixed-Bed Adsorbers using a Combined Selectivity/Capacity Metric. *RSC Advances* **7**, 35724-35737 (2017).

16. Krishna R. Methodologies for Screening and Selection of Crystalline Microporous Materials in Mixture Separations. *Sep Purif Technol* **194**, 281-300 (2018).

17. Krishna R, Baur RD. Adsorption and reaction in zeolites: Modelling and numerical issues. *http://krishnaamsterchemcom/zeolite/*, *University of Amsterdam, Amsterdam*, (1 January 2015).

18. Li L, *et al.* Ethane/ethylene separation in a metal-organic framework with iron peroxo sites. *Science* **362**, 443-446 (2018).

19. Liao PQ, Zhang WX, Zhang JP, Chen XM. Efficient purification of ethene by an ethane-trapping metal-organic framework. *Nat Commun* **6**, 8697 (2015).

20. Pires Jo, Pinto MsL, Saini VK. Ethane selective IRMOF-8 and its significance in ethane−ethylene separation by adsorption. *ACS Appl Mater Interfaces* **6**, 12093−12099 (2014).

21. Wu Y, Chen H, Liu D, Qian Y, Xia H. Adsorption and separation of ethane/ethylene on ZIFs with various topologies: Combining GCMC simulation with the ideal adsorbed solution theory (IAST). *Chem Eng Sci* **124**, 144-153 (2015).

22. Chen D, Wang N, Xu C, Tu G, Zhu W, Krishna R. A combined theoretical and experimental analysis on transient breakthroughs of C_2H_6/C_2H_4 in fixed beds packed with ZIF-7. *Microporous Mesoporous Mater* **208**, 55-65 (2015).

23. Gücüyener C, van den Bergh J, Gascon J, Kapteijn F. Ethane/ethene separation turned on its head: selective ethane adsorption on the metal-organic framework ZIF-7 through a gate-opening mechanism. *J Am Chem Soc* **132**, 17704-17706 (2010).

24. Van den Bergh J, Gucuyener C, Pidko EA, Hensen EJ, Gascon J, Kapteijn F. Understanding the anomalous alkane selectivity of ZIF-7 in the separation of light alkane/alkene mixtures. *Chem Eur J* **17**, 8832–8840 (2011).

25. Chen Y, *et al.* An ethane-trapping MOF PCN-250 for highly selective adsorption of ethane over ethylene. *Chem Eng Sci* **175**, 110-117 (2018).

26. Liang W, *et al.* Ethane selective adsorbent $Ni(bdc)(ted)_{0.5}$ with high uptake and its significance in adsorption separation of ethane and ethylene. *Chem Eng Sci* **148**, 275-281 (2016).

27. Qazvini OT, Babarao R, Shi ZL, Zhang YB, Telfer SG. A robust ethane-trapping metal-organic framework with a high capacity for ethylene purification. *J Am Chem Soc* **141**, 5014-5020 (2019).

28. Lv D, *et al.* Selective adsorption of ethane over ethylene in PCN-245: Impacts of interpenetrated adsorbent. *ACS App Mater Interfaces* **10**, 8366-8373 (2018).

29. Chen Y, *et al.* Highly adsorptive separation of ethane/ethylene by an ethane-selective MOF MIL-142A. *Ind Eng Chem Res* **57**, 4063-4069 (2018).

30. Lin RB, *et al.* Boosting ethane/ethylene separation within isoreticular ultramicroporous metal-organic frameworks. *J Am Chem Soc* **140**, 12940-12946 (2018).

31. Chen K-J, *et al.* Synergistic sorbent separation for one-step ethylene purification from a four-component mixture. *Science* **366**, 241-246 (2019).
