# Supplementary material for: A robust Th-azole framework for highly efficient purification of C2H4 from a C2H4/C2H2/C2H6 mixture
Source: Nat Commun. 2020 Jun 22;11:3163. doi: 10.1038/s41467-020-16960-9 (PMC7308359; doi:10.1038/s41467-020-16960-9)
Supplement: Supplementary file 3 — Source Data [file 41467_2020_16960_MOESM3_ESM.pdf]

A robust Th-Azole framework for highly efficient purification of C_2H_4 from a C_2H_4/C_2H_2/C_2H_6 mixture

Xu et al.

Source Data (as Supplementary Information 2):

Data list

The data for each figures

Data list:

1) Figure 2a: thermal stability from 100 to 400℃
2) Figure 2b: soaking in water and seven different organic solvents 30 days
3) Figure 2c: soaking in different pH solvents 30 days
4) Figure 4a: The N_2 adsorption at 77 K and the distribution of pore size.
5) Figure 4b: The adsorption isotherms of C_2H_6 and C_2H_4 at 298 K, including experiments and simulations
6) Figure 4c: Experimental C_2H_6, C_2H_4, and C_2H_2 adsorption isotherms from 0.01 to 1 bar with the insert of enlargement from 0.01 to 0.1 bar.
7) Figure 4d: The adsorption heat enthalpy of C_2H_6 and C_2H_4, calculated from the single-component C_2H_6 and C_2H_4 adsorption data at 298 K and 273 K.
8) Figure 4e: Predicted mixture adsorption isotherms and selectivity of Azole-Th-1 by IAST method for a 50/50 C_2H_6/C_2H_4 mixture at 298K.
9) Figure 4f: A comparison in selectivity and C_2H_6 adsorption capacity at 298 K and 1 bar between the reported top-performing porous adsorbents for C_2H_6/C_2H_4 separation and our MOF.
10) Figure 5a: Experimental breakthrough curves at 298 K and 1 bar : C_2H_6/C_2H_4 (10/90, v/v) .
11) Figure 5b: Exp. + Simulated breakthrough curves at 298 K and 1 bar : C_2H_6/C_2H_4 (10/90, v/v) .
12) Figure 5c: Experimental breakthrough: C_2H_6/C_2H_4 (50/50, v/v) binary mixture for 5 cycles
13) Figure 5d: Experimental breakthrough: $C_2H_6/C_2H_4/C_2H_2$(90/9/1, v/v/v) ternary mixture separation
14) Supplementary Figure 3: The PXRD patterns: the as-synthesized samples and the simulation from single crystal
15) Supplementary Figure 5: The TG analysis of as-synthesized Azole-Th-1 samples and the samples soaked in methanol for three days and one day under N_2 atmosphere.
16) Supplementary Figure 6: Experimental C_2H_6, C_2H_4 and C_2H_2 adsorption isotherms of Azole-Th-1 at 273 K from 0.001 to 100 kPa.
17) Supplementary Figure 8a: Predicted mixture adsorption isotherms and selectivity of Azole-Th-1 by IAST method for a 10/90 (a) mixture at 298K.

18) Supplementary Figure 8b: Predicted mixture adsorption isotherms and selectivity of Azole-Th-1 by IAST method for a 1/15 (b) C_2H_6/C_2H_4 mixture at 298K.

19) Supplementary Figure 9: The simulated breakthrough curve for C_2H_6/C_2H_4 (50:50, v/v).

20) Supplementary Figure 10a: The experimental breakthrough curves for C_2H_6/C_2H_4 (50/50, v/v).

21) Supplementary Figure 10c: The experimental breakthrough curves for C_2H_6/C_2H_4 (1/15, v/v).

22) Supplementary Figure 11: Before and after breakthrough experiments for binary mixture C_2H_6/C_2H_4.

Figure 2a

2 Theta	Intensity						
degree	a.u.						
a.u.							
MOF-Th(Exp.)		Simulated		100		150	
34	4	0	3	27	3	0	3
34	4.02	0	3.020462	27	3.020462	0	3.020462
34	4.04	0	3.040925	27	3.040925	0	3.040925
34	4.06	0	3.061388	27	3.061388	0	3.061388
0	4.08	0	3.081851	27	3.081851	44	3.081851
4	4.1	0	3.102313	43	3.102313	55	3.102313
0	4.12	0	3.122776	38	3.122776	64	3.122776
0	4.14	0	3.143239	53	3.143239	64	3.143239
0	4.16	0	3.163702	75	3.163702	57	3.163702
0	4.18	0	3.184165	108	3.184165	34	3.184165
0	4.2	0	3.204627	114	3.204627	21	3.204627
22	4.22	0	3.22509	101	3.22509	0	3.22509
45	4.24	0	3.245553	80	3.245553	17	3.245553
56	4.26	0	3.266016	68	3.266016	33	3.266016
61	4.28	0	3.286478	32	3.286478	45	3.286478
74	4.3	0	3.306941	22	3.306941	47	3.306941
73	4.32	0	3.327404	0	3.327404	43	3.327404
46	4.34	0	3.347867	0	3.347867	41	3.347867
46	4.36	0	3.36833	2	3.36833	32	3.36833
54	4.38	0	3.388792	5	3.388792	16	3.388792
70	4.4	0	3.409255	6	3.409255	13	3.409255
92	4.42	0	3.429718	5	3.429718	0	3.429718
116	4.44	0	3.450181	9	3.450181	10	3.450181
120	4.46	0	3.470643	10	3.470643	8	3.470643
104	4.48	0	3.491106	9	3.491106	5	3.491106
94	4.5	0	3.511569	8	3.511569	1	3.511569
88	4.52	0	3.532032	5	3.532032	0	3.532032
68	4.54	0	3.552495	1	3.552495	0	3.552495
77	4.56	0	3.572957	0	3.572957	0	3.572957
74	4.58	0	3.59342	1	3.59342	0	3.59342
55	4.6	0	3.613883	3	3.613883	0	3.613883
22	4.62	0	3.634346	5	3.634346	0	3.634346
48	4.64	0	3.654808	0	3.654808	0	3.654808
59	4.66	0	3.675271	7	3.675271	2	3.675271
46	4.68	0	3.695734	13	3.695734	8	3.695734
62	4.7	0	3.716197	16	3.716197	13	3.716197
88	4.72	0	3.73666	17	3.73666	16	3.73666
88	4.74	0	3.757122	15	3.757122	16	3.757122
77	4.76	0	3.777585	12	3.777585	15	3.777585
55	4.78	0	3.798048	11	3.798048	12	3.798048
32	4.8	0	3.818511	8	3.818511	5	3.818511
0	4.82	0	3.838973	3	3.838973	2	3.838973
4	4.84	0	3.859436	0	3.859436	0	3.859436
2	4.86	0	3.879899	0	3.879899	6	3.879899
0	4.88	0	3.900362	6	3.900362	9	3.900362
0	4.9	0	3.920825	3	3.920825	10	3.920825
0	4.92	0	3.941287	13	3.941287	8	3.941287
0	4.94	19.97874	3.96175	20	3.96175	10	3.96175
0	4.96	20.5456	3.982213	23	3.982213	8	3.982213
0	4.98	21.1372	4.002676	22	4.002676	8	4.002676

0	5	21.7544	4.023139	21	4.023139	6	4.023139
0	5.02	22.3992	4.043602	22	4.043602	9	4.043602
11	5.04	23.073	4.064064	17	4.064064	7	4.064064
34	5.06	23.7776	4.084527	16	4.084527	8	4.084527
50	5.08	24.5152	4.10499	12	4.10499	4	4.10499
52	5.1	25.2874	4.125453	4	4.125453	1	4.125453
62	5.12	26.0968	4.145916	9	4.145916	5	4.145916
62	5.14	26.9456	4.166378	11	4.166378	8	4.166378
53	5.16	27.8364	4.186841	15	4.186841	11	4.186841
36	5.18	28.7722	4.207304	14	4.207304	18	4.207304
23	5.2	29.756	4.227767	15	4.227767	22	4.227767
9	5.22	30.791	4.248229	21	4.248229	31	4.248229
4	5.24	31.881	4.268692	18	4.268692	37	4.268692
8	5.26	33.0298	4.289155	18	4.289155	34	4.289155
0	5.28	34.2418	4.309618	18	4.309618	32	4.309618
0	5.3	35.5216	4.330081	19	4.330081	24	4.330081
12	5.32	36.8746	4.350543	18	4.350543	28	4.350543
27	5.34	38.3064	4.371006	17	4.371006	29	4.371006
36	5.36	39.823	4.391469	17	4.391469	17	4.391469
41	5.38	41.4316	4.411932	19	4.411932	28	4.411932
41	5.4	43.1394	4.432394	16	4.432394	39	4.432394
34	5.42	44.955	4.452857	13	4.452857	51	4.452857
27	5.44	46.8876	4.47332	9	4.47332	59	4.47332
17	5.46	48.9474	4.493783	15	4.493783	57	4.493783
0	5.48	51.1458	4.514246	17	4.514246	53	4.514246
0	5.5	53.4956	4.534708	21	4.534708	39	4.534708
4	5.52	56.0108	4.555171	15	4.555171	30	4.555171
9	5.54	58.7076	4.575634	13	4.575634	20	4.575634
13	5.56	61.6036	4.596097	24	4.596097	0	4.596097
14	5.58	64.719	4.616559	34	4.616559	3	4.616559
12	5.6	68.0766	4.637022	35	4.637022	10	4.637022
11	5.62	71.7018	4.657485	31	4.657485	10	4.657485
12	5.64	75.6242	4.677948	24	4.677948	15	4.677948
9	5.66	79.877	4.698411	20	4.698411	22	4.698411
4	5.68	84.498	4.718873	20	4.718873	29	4.718873
0	5.7	89.5314	4.739336	25	4.739336	38	4.739336
0	5.72	95.0272	4.759799	21	4.759799	35	4.759799
6	5.74	101.044	4.780262	39	4.780262	37	4.780262
10	5.76	107.6496	4.800724	57	4.800724	39	4.800724
12	5.78	114.923	4.821187	58	4.821187	41	4.821187
11	5.8	122.9574	4.84165	60	4.84165	40	4.84165
14	5.82	131.8622	4.862113	52	4.862113	33	4.862113
15	5.84	141.7678	4.882576	38	4.882576	26	4.882576
10	5.86	152.8292	4.903038	30	4.903038	20	4.903038
9	5.88	165.2328	4.923501	25	4.923501	19	4.923501
6	5.9	179.2042	4.943964	19	4.943964	18	4.943964
0	5.92	201.226	4.964427	12	4.964427	4	4.964427
11	5.94	219.394	4.984889	27	4.984889	11	4.984889
20	5.96	240.168	5.005352	28	5.005352	14	5.005352
23	5.98	264.068	5.025815	24	5.025815	20	5.025815
23	6	291.754	5.046278	34	5.046278	18	5.046278
29	6.02	324.07	5.066741	37	5.066741	20	5.066741
29	6.04	362.102	5.087203	46	5.087203	27	5.087203
23	6.06	407.28	5.107666	46	5.107666	33	5.107666
23	6.08	461.494	5.128129	43	5.128129	41	5.128129
15	6.1	527.306	5.148592	50	5.148592	41	5.148592
0	6.12	608.24	5.169055	42	5.169055	33	5.169055
11	6.14	709.24	5.189517	55	5.189517	32	5.189517

14	6.16	837.41	5.20998	55	5.20998	20	5.20998
14	6.18	1003.266	5.230443	48	5.230443	15	5.230443
9	6.2	1223.022	5.250906	55	5.250906	2	5.250906
19	6.22	1523.558	5.271368	64	5.271368	0	5.271368
22	6.24	1954.154	5.291831	74	5.291831	0	5.291831
21	6.26	2612.52	5.312294	74	5.312294	0	5.312294
12	6.28	3688.6	5.332757	80	5.332757	8	5.332757
12	6.3	5504.58	5.35322	78	5.35322	11	5.35322
19	6.32	8484.08	5.373682	63	5.373682	21	5.373682
34	6.34	12899.5	5.394145	69	5.394145	34	5.394145
39	6.36	17899.46	5.414608	56	5.414608	44	5.414608
38	6.38	20000	5.435071	61	5.435071	40	5.435071
28	6.4	16854.3	5.455533	61	5.455533	43	5.455533
31	6.42	11785	5.475996	75	5.475996	40	5.475996
30	6.44	7688.7	5.496459	81	5.496459	43	5.496459
23	6.46	5011.04	5.516922	79	5.516922	55	5.516922
3	6.48	3399.14	5.537385	98	5.537385	55	5.537385
13	6.5	2442.1	5.557847	110	5.557847	55	5.557847
18	6.52	1849.116	5.57831	117	5.57831	63	5.57831
19	6.54	1455.49	5.598773	116	5.598773	63	5.598773
31	6.56	1177.786	5.619236	122	5.619236	68	5.619236
40	6.58	973.444	5.639698	119	5.639698	62	5.639698
38	6.6	818.704	5.660161	95	5.660161	63	5.660161
38	6.62	698.946	5.680624	98	5.680624	55	5.680624
46	6.64	604.592	5.701087	97	5.701087	72	5.701087
45	6.66	529.124	5.72155	98	5.72155	96	5.72155
35	6.68	467.984	5.742012	142	5.742012	97	5.742012
36	6.7	417.918	5.762475	164	5.762475	120	5.762475
27	6.72	376.556	5.782938	179	5.782938	128	5.782938
29	6.74	342.146	5.803401	198	5.803401	135	5.803401
37	6.76	313.37	5.823863	228	5.823863	140	5.823863
34	6.78	289.232	5.844326	230	5.844326	157	5.844326
22	6.8	268.966	5.864789	255	5.864789	155	5.864789
21	6.82	251.99	5.885252	289	5.885252	172	5.885252
22	6.84	237.848	5.905715	314	5.905715	189	5.905715
20	6.86	226.202	5.926177	343	5.926177	209	5.926177
18	6.88	216.794	5.94664	385	5.94664	234	5.94664
15	6.9	209.442	5.967103	407	5.967103	290	5.967103
20	6.92	204.026	5.987566	436	5.987566	329	5.987566
24	6.94	200.488	6.008028	480	6.008028	364	6.008028
24	6.96	198.8302	6.028491	531	6.028491	404	6.028491
14	6.98	199.1158	6.048954	591	6.048954	491	6.048954
7	7	201.48	6.069417	682	6.069417	571	6.069417
20	7.02	206.138	6.08988	763	6.08988	666	6.08988
33	7.04	213.42	6.110342	845	6.110342	758	6.110342
35	7.06	223.788	6.130805	950	6.130805	873	6.130805
47	7.08	237.898	6.151268	1081	6.151268	1003	6.151268
56	7.1	256.676	6.171731	1231	6.171731	1170	6.171731
64	7.12	281.432	6.192193	1409	6.192193	1345	6.192193
64	7.14	314.06	6.212656	1578	6.212656	1511	6.212656
57	7.16	357.344	6.233119	1776	6.233119	1731	6.233119
37	7.18	415.52	6.253582	1974	6.253582	1999	6.253582
19	7.2	495.402	6.274045	2259	6.274045	2305	6.274045
23	7.22	609	6.294507	2552	6.294507	2660	6.294507
24	7.24	779.612	6.31497	2913	6.31497	3090	6.31497
20	7.26	1053.572	6.335433	3336	6.335433	3616	6.335433
28	7.28	1514.926	6.355896	3848	6.355896	4174	6.355896
33	7.3	2288.1	6.376358	4377	6.376358	4822	6.376358

29	7.32	3495.7	6.396821	4925	6.396821	5529	6.396821
30	7.34	5071.64	6.417284	5463	6.417284	6257	6.417284
21	7.36	6259.42	6.437747	5968	6.437747	7051	6.437747
14	7.38	5875.88	6.458209	6414	6.458209	7829	6.458209
2	7.4	4358.58	6.478672	6800	6.478672	8491	6.478672
11	7.42	2902.2	6.499135	7037	6.499135	8922	6.499135
14	7.44	1890.92	6.519598	7153	6.519598	9046	6.519598
25	7.46	1267.158	6.540061	7097	6.540061	8869	6.540061
27	7.48	897.022	6.560523	6873	6.560523	8304	6.560523
27	7.5	672.172	6.580986	6423	6.580986	7450	6.580986
23	7.52	526.542	6.601449	5838	6.601449	6429	6.601449
11	7.54	425.624	6.621912	5185	6.621912	5396	6.621912
16	7.56	352.1	6.642374	4453	6.642374	4452	6.642374
20	7.58	296.682	6.662837	3786	6.662837	3660	6.662837
16	7.6	253.85	6.6833	3151	6.6833	3040	6.6833
32	7.62	220.064	6.703763	2620	6.703763	2539	6.703763
29	7.64	192.9406	6.724226	2213	6.724226	2160	6.724226
52	7.66	170.8284	6.744688	1882	6.744688	1897	6.744688
63	7.68	152.5552	6.765151	1647	6.765151	1661	6.765151
85	7.7	137.2716	6.785614	1433	6.785614	1445	6.785614
106	7.72	124.3498	6.806077	1267	6.806077	1283	6.806077
120	7.74	113.3192	6.826539	1135	6.826539	1152	6.826539
146	7.76	103.8204	6.847002	1014	6.847002	1018	6.847002
167	7.78	95.5758	6.867465	935	6.867465	926	6.867465
174	7.8	88.3682	6.887928	851	6.887928	861	6.887928
181	7.82	82.026	6.908391	797	6.908391	781	6.908391
183	7.84	57.0968	6.928853	753	6.928853	726	6.928853
205	7.86	52.6162	6.949316	712	6.949316	698	6.949316
221	7.88	48.6416	6.969779	695	6.969779	649	6.969779
261	7.9	45.0996	6.990242	663	6.990242	604	6.990242
305	7.92	41.93	7.010705	649	7.010705	593	7.010705
374	7.94	39.0822	7.031167	645	7.031167	568	7.031167
449	7.96	36.5144	7.05163	635	7.05163	545	7.05163
556	7.98	34.1912	7.072093	621	7.072093	553	7.072093
660	8	32.0822	7.092556	613	7.092556	551	7.092556
771	8.02	30.1624	7.113018	624	7.113018	553	7.113018
919	8.04	28.4094	7.133481	644	7.133481	566	7.133481
1081	8.06	26.8046	7.153944	679	7.153944	608	7.153944
1252	8.08	25.332	7.174407	716	7.174407	636	7.174407
1499	8.1	23.9772	7.19487	760	7.19487	691	7.19487
1808	8.12	22.7282	7.215332	837	7.215332	783	7.215332
2199	8.14	21.5742	7.235795	896	7.235795	870	7.235795
2670	8.16	20.5056	7.256258	958	7.256258	1003	7.256258
3228	8.18	19.51458	7.276721	1022	7.276721	1180	7.276721
3791	8.2	18.59354	7.297183	1099	7.297183	1385	7.297183
4449	8.22	17.73614	7.317646	1205	7.317646	1648	7.317646
5269	8.24	16.93666	7.338109	1352	7.338109	1955	7.338109
6300	8.26	16.18996	7.358572	1560	7.358572	2361	7.358572
7694	8.28	15.49154	7.379035	1833	7.379035	2871	7.379035
9421	8.3	14.83732	7.399497	2175	7.399497	3503	7.399497
11396	8.32	14.22366	7.41996	2630	7.41996	4279	7.41996
13245	8.34	13.64724	7.440423	3081	7.440423	5095	7.440423
14555	8.36	13.10514	7.460886	3489	7.460886	5805	7.460886
14866	8.38	12.5947	7.481348	3752	7.481348	6228	7.481348
14041	8.4	12.11348	7.501811	3842	7.501811	6234	7.501811
12315	8.42	11.6593	7.522274	3731	7.522274	5860	7.522274
9948	8.44	11.23018	7.542737	3429	7.542737	5112	7.542737
7386	8.46	10.8243	7.5632	2979	7.5632	4155	7.5632

5088	8.48	10.44002	7.583662	2451	7.583662	3162	7.583662
3299	8.5	10.07584	7.604125	1937	7.604125	2288	7.604125
2211	8.52	9.73036	7.624588	1524	7.624588	1642	7.624588
1619	8.54	9.40236	7.645051	1184	7.645051	1229	7.645051
1337	8.56	9.09064	7.665513	921	7.665513	978	7.665513
1113	8.58	8.79418	7.685976	735	7.685976	823	7.685976
952	8.6	8.51198	7.706439	619	7.706439	679	7.706439
827	8.62	8.24312	7.726902	529	7.726902	586	7.726902
723	8.64	7.98682	7.747365	470	7.747365	505	7.747365
651	8.66	7.74226	7.767827	416	7.767827	433	7.767827
582	8.68	7.50878	7.78829	365	7.78829	395	7.78829
525	8.7	7.28568	7.808753	339	7.808753	360	7.808753
461	8.72	7.07238	7.829216	319	7.829216	305	7.829216
412	8.74	6.86832	7.849678	305	7.849678	278	7.849678
382	8.76	6.67294	7.870141	280	7.870141	251	7.870141
353	8.78	6.4858	7.890604	259	7.890604	236	7.890604
330	8.8	6.3064	7.911067	237	7.911067	218	7.911067
302	8.82	6.13436	7.93153	205	7.93153	193	7.93153
292	8.84	0	7.951992	194	7.951992	191	7.951992
294	8.86	0	7.972455	176	7.972455	181	7.972455
287	8.88	0	7.992918	175	7.992918	185	7.992918
288	8.9	0	8.013381	188	8.013381	182	8.013381
284	8.92	0	8.033843	186	8.033843	159	8.033843
324	8.94	0	8.054306	183	8.054306	130	8.054306
350	8.96	0	8.074769	163	8.074769	102	8.074769
389	8.98	0.37564	8.095232	137	8.095232	89	8.095232
440	9	0.386254	8.115695	122	8.115695	79	8.115695
532	9.02	0.397324	8.136157	95	8.136157	66	8.136157
613	9.04	0.408876	8.15662	77	8.15662	70	8.15662
700	9.06	0.420942	8.177083	75	8.177083	74	8.177083
768	9.08	0.433546	8.197546	86	8.197546	77	8.197546
847	9.1	0.446728	8.218008	89	8.218008	78	8.218008
952	9.12	0.460518	8.238471	93	8.238471	63	8.238471
1098	9.14	0.474956	8.258934	94	8.258934	36	8.258934
1266	9.16	0.490084	8.279397	93	8.279397	28	8.279397
1528	9.18	0.505946	8.29986	96	8.29986	20	8.29986
1944	9.2	0.52259	8.320322	92	8.320322	12	8.320322
2502	9.22	0.54007	8.340785	81	8.340785	25	8.340785
3293	9.24	0.55844	8.361248	72	8.361248	32	8.361248
4365	9.26	0.577764	8.381711	70	8.381711	28	8.381711
5579	9.28	0.598106	8.402173	64	8.402173	30	8.402173
6672	9.3	0.619542	8.422636	65	8.422636	37	8.422636
7289	9.32	0.64215	8.443099	62	8.443099	36	8.443099
7230	9.34	0.666018	8.463562	56	8.463562	29	8.463562
6530	9.36	0.691242	8.484025	53	8.484025	24	8.484025
5356	9.38	0.717924	8.504487	55	8.504487	22	8.504487
3916	9.4	0.74618	8.52495	50	8.52495	16	8.52495
2450	9.42	0.776136	8.545413	47	8.545413	25	8.545413
1300	9.44	0.807932	8.565876	42	8.565876	20	8.565876
621	9.46	0.841722	8.586338	47	8.586338	16	8.586338
370	9.48	0.877672	8.606801	43	8.606801	22	8.606801
346	9.5	0.915974	8.627264	45	8.627264	19	8.627264
341	9.52	0.956838	8.647727	33	8.647727	24	8.647727
318	9.54	1.000494	8.66819	29	8.66819	24	8.66819
260	9.56	1.047204	8.688652	19	8.688652	18	8.688652
223	9.58	1.09726	8.709115	20	8.709115	16	8.709115
183	9.6	1.150988	8.729578	20	8.729578	16	8.729578
142	9.62	1.208756	8.750041	16	8.750041	13	8.750041

115	9.64	1.270978	8.770503	26	8.770503	8	8.770503
103	9.66	1.338122	8.790966	44	8.790966	17	8.790966
90	9.68	1.410724	8.811429	52	8.811429	14	8.811429
79	9.7	1.48939	8.831892	56	8.831892	15	8.831892
71	9.72	1.574812	8.852355	51	8.852355	29	8.852355
90	9.74	1.667784	8.872817	51	8.872817	32	8.872817
77	9.76	1.769224	8.89328	46	8.89328	27	8.89328
90	9.78	1.880188	8.913743	38	8.913743	23	8.913743
89	9.8	2.0019	8.934206	25	8.934206	24	8.934206
87	9.82	2.1358	8.954668	30	8.954668	17	8.954668
91	9.84	2.28358	8.975131	39	8.975131	14	8.975131
83	9.86	2.44718	8.995594	45	8.995594	17	8.995594
72	9.88	2.62896	9.016057	41	9.016057	11	9.016057
50	9.9	2.8317	9.03652	34	9.03652	10	9.03652
49	9.92	3.05876	9.056982	22	9.056982	12	9.056982
46	9.94	3.31416	9.077445	21	9.077445	1	9.077445
50	9.96	3.60282	9.097908	16	9.097908	5	9.097908
51	9.98	3.9307	9.118371	5	9.118371	16	9.118371
53	10	4.30526	9.138833	2	9.138833	26	9.138833
55	10.02	4.7357	9.159296	7	9.159296	29	9.159296
46	10.04	5.23368	9.179759	6	9.179759	29	9.179759
34	10.06	5.814	9.200222	6	9.200222	31	9.200222
30	10.08	6.49572	9.220685	5	9.220685	23	9.220685
24	10.1	7.30372	9.241147	12	9.241147	20	9.241147
29	10.12	8.27086	9.26161	15	9.26161	19	9.26161
15	10.14	9.44138	9.282073	15	9.282073	10	9.282073
25	10.16	10.8758	9.302536	23	9.302536	15	9.302536
13	10.18	12.65848	9.322998	26	9.322998	18	9.322998
24	10.2	14.90984	9.343461	21	9.343461	17	9.343461
29	10.22	17.80626	9.363924	15	9.363924	12	9.363924
27	10.24	21.6158	9.384387	19	9.384387	10	9.384387
27	10.26	26.7716	9.40485	23	9.40485	8	9.40485
32	10.28	34.0426	9.425312	31	9.425312	3	9.425312
30	10.3	44.93	9.445775	45	9.445775	4	9.445775
24	10.32	62.406	9.466238	48	9.466238	10	9.466238
12	10.34	91.7858	9.486701	44	9.486701	11	9.486701
7	10.36	140.742	9.507163	46	9.507163	13	9.507163
0	10.38	216.324	9.527626	34	9.527626	15	9.527626
6	10.4	312.22	9.548089	21	9.548089	18	9.548089
16	10.42	377.642	9.568552	9	9.568552	20	9.568552
21	10.44	345.214	9.589015	7	9.589015	22	9.589015
23	10.46	251.648	9.609477	4	9.609477	20	9.609477
19	10.48	166.0402	9.62994	7	9.62994	25	9.62994
23	10.5	107.5668	9.650403	9	9.650403	27	9.650403
18	10.52	71.7584	9.670866	8	9.670866	35	9.670866
17	10.54	50.5446	9.691328	7	9.691328	28	9.691328
11	10.56	37.6218	9.711791	4	9.711791	28	9.711791
7	10.58	29.2174	9.732254	0	9.732254	26	9.732254
17	10.6	23.3786	9.752717	1	9.752717	30	9.752717
20	10.62	19.12294	9.77318	4	9.77318	31	9.77318
21	10.64	15.91892	9.793642	13	9.793642	26	9.793642
23	10.66	13.44794	9.814105	17	9.814105	22	9.814105
21	10.68	11.5044	9.834568	20	9.834568	16	9.834568
23	10.7	9.94964	9.855031	19	9.855031	12	9.855031
16	10.72	8.68738	9.875493	18	9.875493	12	9.875493
16	10.74	7.64916	9.895956	16	9.895956	13	9.895956
10	10.76	7.44352	9.916419	3	9.916419	28	9.916419
8	10.78	6.73554	9.936882	3	9.936882	47	9.936882

3	10.8	6.13838	9.957345	10	9.957345	68	9.957345
2	10.82	5.63102	9.977807	16	9.977807	79	9.977807
2	10.84	5.19722	9.99827	21	9.99827	73	9.99827
1	10.86	4.82434	10.01873	17	10.01873	63	10.01873
5	10.88	4.50234	10.0392	18	10.0392	51	10.0392
7	10.9	4.22322	10.05966	20	10.05966	50	10.05966
6	10.92	3.98058	10.08012	30	10.08012	54	10.08012
9	10.94	3.76918	10.10058	24	10.10058	61	10.10058
4	10.96	3.58474	10.12105	27	10.12105	73	10.12105
10	10.98	3.42378	10.14151	41	10.14151	93	10.14151
14	11	3.2834	10.16197	60	10.16197	106	10.16197
17	11.02	3.16116	10.18244	69	10.18244	114	10.18244
20	11.04	3.0551	10.2029	80	10.2029	126	10.2029
20	11.06	2.96354	10.22336	110	10.22336	139	10.22336
23	11.08	2.88504	10.24382	138	10.24382	157	10.24382
22	11.1	2.81846	10.26429	171	10.26429	183	10.26429
18	11.12	2.76282	10.28475	204	10.28475	215	10.28475
13	11.14	2.7173	10.30521	235	10.30521	252	10.30521
4	11.16	2.68124	10.32568	280	10.32568	290	10.32568
6	11.18	2.65408	10.34614	339	10.34614	350	10.34614
0	11.2	2.6354	10.3666	417	10.3666	416	10.3666
0	11.22	2.62486	10.38706	508	10.38706	514	10.38706
0	11.24	2.6222	10.40753	612	10.40753	663	10.40753
6	11.26	2.62728	10.42799	756	10.42799	822	10.42799
10	11.28	2.64	10.44845	907	10.44845	1046	10.44845
21	11.3	2.66034	10.46891	1056	10.46891	1323	10.46891
30	11.32	3.42564	10.48938	1204	10.48938	1641	10.48938
40	11.34	3.4822	10.50984	1328	10.50984	1953	10.50984
40	11.36	3.54766	10.5303	1426	10.5303	2195	10.5303
40	11.38	3.62242	10.55077	1480	10.55077	2326	10.55077
41	11.4	3.7069	10.57123	1457	10.57123	2287	10.57123
36	11.42	3.8016	10.59169	1369	10.59169	2100	10.59169
30	11.44	3.90718	10.61215	1227	10.61215	1798	10.61215
28	11.46	4.02436	10.63262	1052	10.63262	1402	10.63262
24	11.48	4.15404	10.65308	869	10.65308	1035	10.65308
28	11.5	4.29722	10.67354	669	10.67354	735	10.67354
32	11.52	4.45512	10.69401	497	10.69401	508	10.69401
31	11.54	4.6291	10.71447	363	10.71447	371	10.71447
31	11.56	4.82076	10.73493	269	10.73493	291	10.73493
34	11.58	5.03196	10.75539	194	10.75539	245	10.75539
35	11.6	5.26492	10.77586	144	10.77586	219	10.77586
40	11.62	5.52216	10.79632	116	10.79632	192	10.79632
40	11.64	5.80668	10.81678	101	10.81678	167	10.81678
39	11.66	6.12198	10.83724	83	10.83724	146	10.83724
36	11.68	6.47222	10.85771	71	10.85771	139	10.85771
31	11.7	6.86238	10.87817	64	10.87817	128	10.87817
22	11.72	7.29834	10.89863	59	10.89863	109	10.89863
17	11.74	7.78724	10.9191	58	10.9191	88	10.9191
22	11.76	8.33768	10.93956	47	10.93956	75	10.93956
19	11.78	8.9602	10.96002	42	10.96002	73	10.96002
13	11.8	9.6677	10.98048	46	10.98048	79	10.98048
28	11.82	10.47628	11.00095	40	11.00095	79	11.00095
34	11.84	11.406	11.02141	30	11.02141	80	11.02141
47	11.86	12.48236	11.04187	19	11.04187	93	11.04187
55	11.88	13.73796	11.06233	8	11.06233	86	11.06233
56	11.9	14.85582	11.0828	2	11.0828	66	11.0828
66	11.92	16.6198	11.10326	6	11.10326	55	11.10326
83	11.94	18.73508	11.12372	11	11.12372	40	11.12372

90	11.96	21.3028	11.14419	18	11.14419	32	11.14419
92	11.98	24.4632	11.16465	23	11.16465	33	11.16465
86	12	28.4144	11.18511	29	11.18511	41	11.18511
110	12.02	33.445	11.20557	27	11.20557	55	11.20557
128	12.04	39.9866	11.22604	29	11.22604	66	11.22604
152	12.06	48.7236	11.2465	23	11.2465	74	11.2465
177	12.08	60.8352	11.26696	19	11.26696	65	11.26696
213	12.1	78.5724	11.28743	9	11.28743	49	11.28743
279	12.12	106.4248	11.30789	10	11.30789	34	11.30789
337	12.14	152.7448	11.32835	17	11.32835	17	11.32835
431	12.16	230.478	11.34881	18	11.34881	2	11.34881
562	12.18	353.89	11.36928	13	11.36928	0	11.36928
708	12.2	522.624	11.38974	16	11.38974	10	11.38974
961	12.22	672.078	11.4102	11	11.4102	15	11.4102
1333	12.24	665.904	11.43066	16	11.43066	32	11.43066
1831	12.26	511.704	11.45113	14	11.45113	42	11.45113
2382	12.28	345.892	11.47159	17	11.47159	58	11.47159
2840	12.3	226.696	11.49205	19	11.49205	76	11.49205
3059	12.32	152.4144	11.51252	21	11.51252	76	11.51252
2983	12.34	108.6206	11.53298	31	11.53298	67	11.53298
2655	12.36	82.6624	11.55344	29	11.55344	67	11.55344
2119	12.38	66.5158	11.5739	30	11.5739	68	11.5739
1466	12.4	55.92	11.59437	33	11.59437	57	11.59437
866	12.42	48.7598	11.61483	29	11.61483	52	11.61483
424	12.44	43.9458	11.63529	28	11.63529	56	11.63529
191	12.46	40.8736	11.65576	25	11.65576	56	11.65576
109	12.48	39.1988	11.67622	19	11.67622	72	11.67622
101	12.5	38.7418	11.69668	14	11.69668	83	11.69668
87	12.52	39.4486	11.71714	15	11.71714	84	11.71714
68	12.54	41.3776	11.73761	19	11.73761	85	11.73761
49	12.56	44.712	11.75807	22	11.75807	103	11.75807
38	12.58	49.7968	11.77853	36	11.77853	114	11.77853
31	12.6	57.2266	11.79899	37	11.79899	103	11.79899
21	12.62	68.0468	11.81946	40	11.81946	105	11.81946
26	12.64	84.2438	11.83992	42	11.83992	108	11.83992
36	12.66	109.8074	11.86038	41	11.86038	103	11.86038
30	12.68	152.4436	11.88085	37	11.88085	99	11.88085
40	12.7	224.932	11.90131	35	11.90131	98	11.90131
43	12.72	343.37	11.92177	43	11.92177	96	11.92177
38	12.74	515.76	11.94223	47	11.94223	91	11.94223
31	12.76	700.122	11.9627	45	11.9627	107	11.9627
24	12.78	755.602	11.98316	55	11.98316	112	11.98316
24	12.8	618.582	12.00362	65	12.00362	119	12.00362
21	12.82	427.608	12.02409	75	12.02409	131	12.02409
23	12.84	279.08	12.04455	94	12.04455	145	12.04455
27	12.86	183.296	12.06501	111	12.06501	166	12.06501
27	12.88	125.8412	12.08547	130	12.08547	183	12.08547
31	12.9	91.5622	12.10594	145	12.10594	228	12.10594
27	12.92	70.0886	12.1264	166	12.1264	267	12.1264
19	12.94	55.66	12.14686	188	12.14686	317	12.14686
5	12.96	45.3724	12.16732	208	12.16732	367	12.16732
5	12.98	37.7324	12.18779	254	12.18779	420	12.18779
7	13	31.8948	12.20825	323	12.20825	485	12.20825
2	13.02	27.3344	12.22871	409	12.22871	555	12.22871
0	13.04	23.7046	12.24918	531	12.24918	665	12.24918
0	13.06	20.7684	12.26964	672	12.26964	794	12.26964
2	13.08	18.35922	12.2901	816	12.2901	947	12.2901
11	13.1	16.35764	12.31056	959	12.31056	1160	12.31056

15	13.12	14.676	12.33103	1090	12.33103	1342	12.33103
30	13.14	13.24902	12.35149	1191	12.35149	1494	12.35149
39	13.16	12.02726	12.37195	1229	12.37195	1552	12.37195
38	13.18	10.97274	12.39242	1214	12.39242	1517	12.39242
41	13.2	10.0559	12.41288	1151	12.41288	1393	12.41288
39	13.22	9.25344	12.43334	1022	12.43334	1212	12.43334
33	13.24	8.54682	12.4538	888	12.4538	1007	12.4538
27	13.26	7.92112	12.47427	742	12.47427	779	12.47427
25	13.28	7.36424	12.49473	584	12.49473	597	12.49473
22	13.3	6.86628	12.51519	454	12.51519	465	12.51519
25	13.32	7.4293	12.53565	349	12.53565	355	12.53565
30	13.34	7.05458	12.55612	270	12.55612	304	12.55612
32	13.36	6.71936	12.57658	209	12.57658	260	12.57658
30	13.38	6.41892	12.59704	159	12.59704	228	12.59704
39	13.4	6.14928	12.61751	133	12.61751	207	12.61751
37	13.42	5.90706	12.63797	114	12.63797	216	12.63797
37	13.44	5.6894	12.65843	115	12.65843	214	12.65843
32	13.46	5.49388	12.67889	117	12.67889	215	12.67889
23	13.48	5.31838	12.69936	120	12.69936	217	12.69936
13	13.5	5.1611	12.71982	143	12.71982	220	12.71982
15	13.52	5.02052	12.74028	163	12.74028	224	12.74028
16	13.54	4.89532	12.76075	184	12.76075	252	12.76075
30	13.56	4.78434	12.78121	212	12.78121	271	12.78121
36	13.58	4.68662	12.80167	235	12.80167	318	12.80167
37	13.6	4.60136	12.82213	264	12.82213	374	12.82213
35	13.62	4.52782	12.8426	293	12.8426	447	12.8426
47	13.64	4.46544	12.86306	313	12.86306	497	12.86306
49	13.66	4.41372	12.88352	345	12.88352	548	12.88352
54	13.68	4.37228	12.90398	363	12.90398	584	12.90398
49	13.7	4.34084	12.92445	381	12.92445	585	12.92445
56	13.72	3.67962	12.94491	377	12.94491	547	12.94491
68	13.74	3.68432	12.96537	361	12.96537	496	12.96537
79	13.76	3.69792	12.98584	324	12.98584	411	12.98584
84	13.78	3.72046	13.0063	270	13.0063	338	13.0063
83	13.8	3.75204	13.02676	206	13.02676	272	13.02676
101	13.82	3.79284	13.04722	155	13.04722	227	13.04722
122	13.84	3.84312	13.06769	109	13.06769	175	13.06769
147	13.86	3.90324	13.08815	72	13.08815	161	13.08815
183	13.88	3.97358	13.10861	41	13.10861	149	13.10861
220	13.9	4.05472	13.12908	29	13.12908	144	13.12908
275	13.92	4.14722	13.14954	21	13.14954	142	13.14954
340	13.94	4.25182	13.17	13	13.17	147	13.17
428	13.96	4.3694	13.19046	9	13.19046	143	13.19046
557	13.98	4.50094	13.21093	8	13.21093	150	13.21093
733	14	4.64756	13.23139	8	13.23139	159	13.23139
976	14.02	4.81064	13.25185	16	13.25185	168	13.25185
1261	14.04	4.9917	13.27231	12	13.27231	145	13.27231
1565	14.06	5.19252	13.29278	12	13.29278	145	13.29278
1814	14.08	5.41518	13.31324	16	13.31324	134	13.31324
1919	14.1	5.6621	13.3337	18	13.3337	137	13.3337
1877	14.12	5.93606	13.35417	17	13.35417	139	13.35417
1671	14.14	6.24032	13.37463	12	13.37463	148	13.37463
1365	14.16	6.57876	13.39509	12	13.39509	161	13.39509
1019	14.18	6.95584	13.41555	14	13.41555	166	13.41555
687	14.2	7.37692	13.43602	15	13.43602	170	13.43602
432	14.22	7.8483	13.45648	14	13.45648	181	13.45648
269	14.24	7.64968	13.47694	13	13.47694	183	13.47694
201	14.26	8.26532	13.49741	16	13.49741	197	13.49741

191	14.28	8.9581	13.51787	19	13.51787	211	13.51787
174	14.3	9.74142	13.53833	17	13.53833	229	13.53833
165	14.32	10.63168	13.55879	13	13.55879	237	13.55879
160	14.34	11.64918	13.57926	6	13.57926	249	13.57926
164	14.36	12.81924	13.59972	0	13.59972	261	13.59972
172	14.38	14.17372	13.62018	4	13.62018	262	13.62018
191	14.4	15.7533	13.64064	6	13.64064	271	13.64064
211	14.42	17.61032	13.66111	4	13.66111	275	13.66111
209	14.44	19.8132	13.68157	9	13.68157	279	13.68157
224	14.46	22.4526	13.70203	12	13.70203	287	13.70203
248	14.48	25.6504	13.7225	13	13.7225	290	13.7225
298	14.5	29.5742	13.74296	13	13.74296	289	13.74296
370	14.52	34.4572	13.76342	11	13.76342	284	13.76342
484	14.54	40.6342	13.78388	12	13.78388	296	13.78388
635	14.56	48.5956	13.80435	11	13.80435	302	13.80435
813	14.58	59.0912	13.82481	13	13.82481	318	13.82481
990	14.6	73.3406	13.84527	14	13.84527	337	13.84527
1104	14.62	93.5318	13.86574	13	13.86574	345	13.86574
1115	14.64	124.2276	13.8862	16	13.8862	365	13.8862
1038	14.66	173.3632	13.90666	14	13.90666	385	13.90666
895	14.68	256.11	13.92712	13	13.92712	404	13.92712
723	14.7	393.43	13.94759	22	13.94759	426	13.94759
505	14.72	603.036	13.96805	35	13.96805	459	13.96805
324	14.74	860.934	13.98851	43	13.98851	487	13.98851
202	14.76	1017.42	14.00897	46	14.00897	499	14.00897
149	14.78	906.406	14.02944	57	14.02944	510	14.02944
145	14.8	651.562	14.0499	68	14.0499	539	14.0499
156	14.82	428.16	14.07036	74	14.07036	542	14.07036
156	14.84	277.8	14.09083	70	14.09083	550	14.09083
170	14.86	186.264	14.11129	66	14.11129	549	14.11129
185	14.88	132.031	14.13175	56	14.13175	554	14.13175
194	14.9	98.832	14.15221	55	14.15221	566	14.15221
191	14.92	77.1034	14.17268	60	14.17268	603	14.17268
216	14.94	61.9334	14.19314	63	14.19314	622	14.19314
223	14.96	50.842	14.2136	69	14.2136	667	14.2136
250	14.98	42.4752	14.23407	81	14.23407	713	14.23407
260	15	36.0142	14.25453	93	14.25453	765	14.25453
248	15.02	30.9286	14.27499	108	14.27499	793	14.27499
240	15.04	26.8588	14.29545	106	14.29545	823	14.29545
228	15.06	23.7094	14.31592	106	14.31592	836	14.31592
209	15.08	20.998	14.33638	90	14.33638	846	14.33638
196	15.1	18.74548	14.35684	88	14.35684	851	14.35684
212	15.12	16.85594	14.3773	83	14.3773	860	14.3773
229	15.14	15.25736	14.39777	81	14.39777	860	14.39777
253	15.16	13.89484	14.41823	79	14.41823	879	14.41823
273	15.18	12.72588	14.43869	78	14.43869	904	14.43869
280	15.2	11.71726	14.45916	94	14.45916	922	14.45916
273	15.22	10.8427	14.47962	118	14.47962	951	14.47962
291	15.24	10.08128	14.50008	137	14.50008	980	14.50008
293	15.26	9.41614	14.52054	157	14.52054	1008	14.52054
299	15.28	8.83362	14.54101	163	14.54101	1024	14.54101
306	15.3	8.32262	14.56147	180	14.56147	1052	14.56147
328	15.32	7.874	14.58193	178	14.58193	1070	14.58193
347	15.34	7.4803	14.6024	176	14.6024	1095	14.6024
372	15.36	7.13534	14.62286	169	14.62286	1109	14.62286
378	15.38	6.83406	14.64332	175	14.64332	1151	14.64332
387	15.4	6.57232	14.66378	195	14.66378	1184	14.66378
391	15.42	6.34676	14.68425	226	14.68425	1226	14.68425

414	15.44	6.15466	14.70471	257	14.70471	1271	14.70471
440	15.46	5.99396	14.72517	282	14.72517	1318	14.72517
459	15.48	5.86308	14.74563	320	14.74563	1352	14.74563
482	15.5	5.76096	14.7661	360	14.7661	1403	14.7661
511	15.52	5.68706	14.78656	394	14.78656	1456	14.78656
545	15.54	5.64128	14.80702	425	14.80702	1510	14.80702
567	15.56	5.62408	14.82749	473	14.82749	1576	14.82749
586	15.58	5.63638	14.84795	547	14.84795	1680	14.84795
599	15.6	5.67978	14.86841	607	14.86841	1768	14.86841
605	15.62	5.75654	14.88887	656	14.88887	1814	14.88887
619	15.64	5.86976	14.90934	679	14.90934	1836	14.90934
642	15.66	6.02356	14.9298	678	14.9298	1809	14.9298
658	15.68	6.22328	14.95026	652	14.95026	1729	14.95026
684	15.7	6.47586	14.97073	596	14.97073	1615	14.97073
703	15.72	6.7903	14.99119	520	14.99119	1506	14.99119
730	15.74	7.17834	15.01165	419	15.01165	1365	15.01165
762	15.76	7.65526	15.03211	350	15.03211	1245	15.03211
791	15.78	8.24138	15.05258	280	15.05258	1173	15.05258
811	15.8	8.96386	15.07304	215	15.07304	1110	15.07304
834	15.82	9.85958	15.0935	184	15.0935	1054	15.0935
876	15.84	10.97956	15.11396	156	15.11396	1026	15.11396
911	15.86	12.39572	15.13443	139	15.13443	990	15.13443
957	15.88	14.21182	15.15489	128	15.15489	972	15.15489
1002	15.9	16.5821	15.17535	125	15.17535	966	15.17535
1024	15.92	19.7464	15.19582	108	15.19582	959	15.19582
1071	15.94	24.1074	15.21628	80	15.21628	914	15.21628
1105	15.96	30.414	15.23674	75	15.23674	861	15.23674
1123	15.98	40.1572	15.2572	61	15.2572	835	15.2572
1152	16	56.1982	15.27767	57	15.27767	808	15.27767
1188	16.02	83.2524	15.29813	60	15.29813	793	15.29813
1235	16.04	127.205	15.31859	53	15.31859	773	15.31859
1249	16.06	190.7946	15.33906	57	15.33906	764	15.33906
1281	16.08	257.95	15.35952	56	15.35952	769	15.35952
1301	16.1	276.712	15.37998	57	15.37998	765	15.37998
1320	16.12	225.794	15.40044	51	15.40044	749	15.40044
1362	16.14	156.5012	15.42091	41	15.42091	718	15.42091
1381	16.16	103.0784	15.44137	38	15.44137	686	15.44137
1405	16.18	68.8712	15.46183	39	15.46183	659	15.46183
1443	16.2	48.5652	15.48229	31	15.48229	643	15.48229
1461	16.22	35.6878	15.50276	27	15.50276	639	15.50276
1470	16.24	28.522	15.52322	22	15.52322	619	15.52322
1484	16.26	24.011	15.54368	22	15.54368	607	15.54368
1521	16.28	21.1528	15.56415	13	15.56415	593	15.56415
1535	16.3	19.46912	15.58461	21	15.58461	583	15.58461
1580	16.32	18.74188	15.60507	22	15.60507	576	15.60507
1623	16.34	18.91586	15.62553	17	15.62553	576	15.62553
1658	16.36	20.0904	15.646	12	15.646	568	15.646
1703	16.38	22.5948	15.66646	17	15.66646	568	15.66646
1741	16.4	27.1948	15.68692	19	15.68692	567	15.68692
1759	16.42	35.4394	15.70739	23	15.70739	580	15.70739
1823	16.44	49.948	15.72785	18	15.72785	563	15.72785
1917	16.46	74.069	15.74831	20	15.74831	557	15.74831
2031	16.48	109.5764	15.76877	11	15.76877	555	15.76877
2185	16.5	148.1374	15.78924	14	15.78924	543	15.78924
2370	16.52	160.6244	15.8097	19	15.8097	545	15.8097
2546	16.54	132.5014	15.83016	18	15.83016	554	15.83016
2707	16.56	92.2664	15.85062	19	15.85062	555	15.85062
2806	16.58	60.707	15.87109	25	15.87109	563	15.87109

2775	16.6	40.2582	15.89155	26	15.89155	552	15.89155
2655	16.62	27.9418	15.91201	32	15.91201	561	15.91201
2476	16.64	20.5638	15.93248	32	15.93248	545	15.93248
2246	16.66	16.17046	15.95294	34	15.95294	547	15.95294
2012	16.68	13.04326	15.9734	23	15.9734	549	15.9734
1838	16.7	10.80332	15.99386	18	15.99386	548	15.99386
1675	16.72	9.13162	16.01433	24	16.01433	569	16.01433
1584	16.74	7.84784	16.03479	19	16.03479	585	16.03479
1545	16.76	6.83992	16.05525	27	16.05525	609	16.05525
1501	16.78	6.03388	16.07572	41	16.07572	633	16.07572
1440	16.8	5.37904	16.09618	64	16.09618	659	16.09618
1409	16.82	4.83972	16.11664	105	16.11664	686	16.11664
1375	16.84	4.39028	16.1371	135	16.1371	719	16.1371
1343	16.86	4.01186	16.15757	171	16.15757	768	16.15757
1304	16.88	3.69044	16.17803	214	16.17803	821	16.17803
1282	16.9	3.41528	16.19849	248	16.19849	873	16.19849
1232	16.92	3.1782	16.21895	275	16.21895	912	16.21895
1220	16.94	2.97278	16.23942	283	16.23942	934	16.23942
1221	16.96	2.79394	16.25988	295	16.25988	935	16.25988
1187	16.98	2.63766	16.28034	290	16.28034	908	16.28034
1161	17	2.5007	16.30081	279	16.30081	872	16.30081
1145	17.02	2.38042	16.32127	253	16.32127	819	16.32127
1125	17.04	2.2747	16.34173	207	16.34173	764	16.34173
1082	17.06	2.18178	16.36219	177	16.36219	729	16.36219
1033	17.08	2.10018	16.38266	153	16.38266	704	16.38266
999	17.1	2.02872	16.40312	124	16.40312	688	16.40312
977	17.12	1.966402	16.42358	102	16.42358	689	16.42358
981	17.14	1.912402	16.44405	93	16.44405	683	16.44405
981	17.16	1.866046	16.46451	83	16.46451	667	16.46451
951	17.18	1.826776	16.48497	84	16.48497	649	16.48497
940	17.2	1.794142	16.50543	77	16.50543	645	16.50543
920	17.22	1.767792	16.5259	65	16.5259	639	16.5259
904	17.24	1.74745	16.54636	62	16.54636	627	16.54636
868	17.26	1.73292	16.56682	62	16.56682	636	16.56682
846	17.28	1.724074	16.58728	71	16.58728	636	16.58728
840	17.3	1.720856	16.60775	86	16.60775	639	16.60775
848	17.32	1.723268	16.62821	93	16.62821	655	16.62821
836	17.34	1.731386	16.64867	103	16.64867	658	16.64867
832	17.36	1.745348	16.66914	104	16.66914	645	16.66914
832	17.38	1.765366	16.6896	110	16.6896	633	16.6896
837	17.4	1.791726	16.71006	89	16.71006	619	16.71006
822	17.42	1.824798	16.73052	80	16.73052	596	16.73052
806	17.44	1.86505	16.75099	75	16.75099	577	16.75099
778	17.46	1.913054	16.77145	61	16.77145	552	16.77145
754	17.48	1.969504	16.79191	66	16.79191	524	16.79191
742	17.5	2.03524	16.81238	60	16.81238	488	16.81238
733	17.52	2.11128	16.83284	45	16.83284	467	16.83284
741	17.54	2.19882	16.8533	45	16.8533	452	16.8533
758	17.56	2.03378	16.87376	45	16.87376	434	16.87376
777	17.58	2.15612	16.89423	44	16.89423	422	16.89423
793	17.6	2.29504	16.91469	34	16.91469	402	16.91469
804	17.62	2.45312	16.93515	36	16.93515	386	16.93515
798	17.64	2.63358	16.95561	33	16.95561	380	16.95561
793	17.66	2.84026	16.97608	26	16.97608	357	16.97608
786	17.68	3.078	16.99654	30	16.99654	326	16.99654
789	17.7	3.35284	17.017	21	17.017	286	17.017
780	17.72	3.67232	17.03747	12	17.03747	275	17.03747
810	17.74	4.0461	17.05793	16	17.05793	259	17.05793

832	17.76	4.91378	17.07839	14	17.07839	249	17.07839
874	17.78	5.44922	17.09885	19	17.09885	238	17.09885
928	17.8	6.08954	17.11932	20	17.11932	223	17.11932
1013	17.82	6.86356	17.13978	20	17.13978	216	17.13978
1085	17.84	7.81066	17.16024	16	17.16024	207	17.16024
1180	17.86	8.98574	17.18071	9	17.18071	187	17.18071
1225	17.88	10.46698	17.20117	10	17.20117	158	17.20117
1241	17.9	12.36872	17.22163	9	17.22163	156	17.22163
1202	17.92	14.86434	17.24209	5	17.24209	165	17.24209
1143	17.94	18.23272	17.26256	15	17.26256	140	17.26256
1071	17.96	22.9664	17.28302	24	17.28302	125	17.28302
988	17.98	29.8702	17.30348	29	17.30348	115	17.30348
931	18	41.1532	17.32394	32	17.32394	102	17.32394
897	18.02	60.0852	17.34441	35	17.34441	95	17.34441
866	18.04	91.686	17.36487	36	17.36487	83	17.36487
874	18.06	140.7676	17.38533	36	17.38533	75	17.38533
880	18.08	204.072	17.4058	36	17.4058	66	17.4058
894	18.1	249.948	17.42626	30	17.42626	87	17.42626
867	18.12	232.1	17.44672	19	17.44672	94	17.44672
863	18.14	170.9174	17.46718	17	17.46718	80	17.46718
857	18.16	113.3914	17.48765	16	17.48765	77	17.48765
864	18.18	73.7458	17.50811	9	17.50811	69	17.50811
879	18.2	49.3918	17.52857	5	17.52857	61	17.52857
889	18.22	34.9774	17.54904	5	17.54904	60	17.54904
897	18.24	26.2374	17.5695	11	17.5695	50	17.5695
904	18.26	20.5908	17.58996	19	17.58996	48	17.58996
927	18.28	16.69524	17.61042	21	17.61042	42	17.61042
914	18.3	13.87656	17.63089	16	17.63089	43	17.63089
878	18.32	11.77196	17.65135	18	17.65135	34	17.65135
860	18.34	10.16534	17.67181	18	17.67181	23	17.67181
823	18.36	8.91774	17.69227	21	17.69227	10	17.69227
802	18.38	7.93582	17.71274	24	17.71274	1	17.71274
768	18.4	7.15498	17.7332	25	17.7332	0	17.7332
737	18.42	6.5295	17.75366	28	17.75366	5	17.75366
708	18.44	6.02642	17.77413	28	17.77413	10	17.77413
669	18.46	5.62164	17.79459	31	17.79459	13	17.79459
659	18.48	5.29722	17.81505	25	17.81505	15	17.81505
640	18.5	5.0398	17.83551	12	17.83551	13	17.83551
610	18.52	4.8393	17.85598	17	17.85598	13	17.85598
588	18.54	4.68818	17.87644	19	17.87644	15	17.87644
564	18.56	4.5808	17.8969	28	17.8969	13	17.8969
536	18.58	4.51312	17.91737	41	17.91737	13	17.91737
512	18.6	4.4823	17.93783	54	17.93783	3	17.93783
495	18.62	4.48658	17.95829	56	17.95829	9	17.95829
477	18.64	4.5252	17.97875	60	17.97875	16	17.97875
462	18.66	4.59822	17.99922	69	17.99922	19	17.99922
444	18.68	4.7066	18.01968	75	18.01968	37	18.01968
427	18.7	4.85222	18.04014	84	18.04014	49	18.04014
390	18.72	5.0379	18.0606	105	18.0606	76	18.0606
365	18.74	5.2676	18.08107	126	18.08107	105	18.08107
333	18.76	5.5466	18.10153	164	18.10153	132	18.10153
309	18.78	5.88172	18.12199	207	18.12199	184	18.12199
294	18.8	6.28184	18.14246	257	18.14246	237	18.14246
285	18.82	6.75828	18.16292	312	18.16292	319	18.16292
265	18.84	7.32572	18.18338	388	18.18338	409	18.18338
264	18.86	8.0031	18.20384	469	18.20384	512	18.20384
246	18.88	8.81524	18.22431	539	18.22431	608	18.22431
226	18.9	9.79492	18.24477	587	18.24477	671	18.24477

196	18.92	10.98602	18.26523	607	18.26523	705	18.26523
183	18.94	12.44824	18.2857	590	18.2857	673	18.2857
167	18.96	14.2642	18.30616	545	18.30616	618	18.30616
160	18.98	16.55064	18.32662	470	18.32662	547	18.32662
138	19	19.47646	18.34708	388	18.34708	445	18.34708
127	19.02	23.2934	18.36755	320	18.36755	351	18.36755
116	19.04	28.3956	18.38801	250	18.38801	271	18.38801
116	19.06	35.4496	18.40847	202	18.40847	215	18.40847
127	19.08	45.7064	18.42893	167	18.42893	165	18.42893
128	19.1	61.6606	18.4494	143	18.4494	129	18.4494
124	19.12	88.0614	18.46986	120	18.46986	95	18.46986
111	19.14	132.6026	18.49032	99	18.49032	53	18.49032
101	19.16	204.574	18.51079	77	18.51079	37	18.51079
84	19.18	307.168	18.53125	61	18.53125	22	18.53125
60	19.2	410.644	18.55171	50	18.55171	11	18.55171
45	19.22	430.324	18.57217	49	18.57217	12	18.57217
29	19.24	343.52	18.59264	34	18.59264	15	18.59264
17	19.26	234.512	18.6131	34	18.6131	18	18.6131
24	19.28	152.2448	18.63356	38	18.63356	18	18.63356
22	19.3	99.8282	18.65403	38	18.65403	17	18.65403
15	19.32	68.5364	18.67449	30	18.67449	10	18.67449
1	19.34	49.8456	18.69495	25	18.69495	5	18.69495
5	19.36	38.0816	18.71541	17	18.71541	0	18.71541
5	19.38	30.1422	18.73588	13	18.73588	3	18.73588
8	19.4	24.469	18.75634	13	18.75634	3	18.75634
8	19.42	20.2544	18.7768	23	18.7768	8	18.7768
7	19.44	17.03674	18.79726	29	18.79726	8	18.79726
2	19.46	14.5266	18.81773	28	18.81773	9	18.81773
3	19.48	12.6242	18.83819	35	18.83819	8	18.83819
0	19.5	11.0175	18.85865	39	18.85865	4	18.85865
4	19.52	9.70328	18.87912	36	18.87912	4	18.87912
8	19.54	8.61514	18.89958	28	18.89958	2	18.89958
14	19.56	7.70434	18.92004	23	18.92004	0	18.92004
20	19.58	6.69628	18.9405	29	18.9405	12	18.9405
24	19.6	6.04642	18.96097	32	18.96097	19	18.96097
29	19.62	5.48872	18.98143	36	18.98143	18	18.98143
39	19.64	5.00676	19.00189	37	19.00189	23	19.00189
57	19.66	4.5876	19.02235	37	19.02235	25	19.02235
91	19.68	4.22098	19.04282	40	19.04282	23	19.04282
111	19.7	3.89862	19.06328	40	19.06328	33	19.06328
177	19.72	3.61384	19.08374	44	19.08374	51	19.08374
284	19.74	3.36118	19.10421	63	19.10421	69	19.10421
456	19.76	3.1361	19.12467	78	19.12467	97	19.12467
707	19.78	2.93492	19.14513	99	19.14513	134	19.14513
984	19.8	2.7545	19.16559	122	19.16559	161	19.16559
1261	19.82	2.59224	19.18606	157	19.18606	197	19.18606
1457	19.84	2.44594	19.20652	200	19.20652	260	19.20652
1546	19.86	2.31374	19.22698	257	19.22698	319	19.22698
1477	19.88	2.19404	19.24745	329	19.24745	417	19.24745
1272	19.9	2.0855	19.26791	419	19.26791	545	19.26791
992	19.92	1.986936	19.28837	549	19.28837	715	19.28837
683	19.94	1.897368	19.30883	676	19.30883	913	19.30883
412	19.96	1.81593	19.3293	792	19.3293	1102	19.3293
226	19.98	1.741876	19.34976	903	19.34976	1261	19.34976
102	20	1.674566	19.37022	985	19.37022	1325	19.37022
62	20.02	1.613448	19.39068	1007	19.39068	1320	19.39068
41	20.04	1.558046	19.41115	964	19.41115	1234	19.41115
42	20.06	1.507948	19.43161	869	19.43161	1057	19.43161

25	20.08	1.46281	19.45207	750	19.45207	854	19.45207
37	20.1	1.422334	19.47254	608	19.47254	635	19.47254
36	20.12	1.386278	19.493	479	19.493	457	19.493
32	20.14	1.354442	19.51346	345	19.51346	315	19.51346
26	20.16	1.32667	19.53392	255	19.53392	219	19.53392
20	20.18	1.302848	19.55439	193	19.55439	166	19.55439
15	20.2	1.282902	19.57485	156	19.57485	123	19.57485
22	20.22	1.266796	19.59531	121	19.59531	102	19.59531
19	20.24	1.254542	19.61578	101	19.61578	99	19.61578
13	20.26	1.24619	19.63624	78	19.63624	82	19.63624
6	20.28	1.241836	19.6567	70	19.6567	74	19.6567
16	20.3	1.241632	19.67716	61	19.67716	64	19.67716
15	20.32	1.245782	19.69763	51	19.69763	54	19.69763
12	20.34	1.254558	19.71809	39	19.71809	44	19.71809
10	20.36	1.26831	19.73855	35	19.73855	37	19.73855
5	20.38	1.287472	19.75901	31	19.75901	30	19.75901
6	20.4	1.312586	19.77948	33	19.77948	33	19.77948
2	20.42	1.344322	19.79994	28	19.79994	25	19.79994
0	20.44	1.383508	19.8204	32	19.8204	33	19.8204
5	20.46	1.995518	19.84087	28	19.84087	28	19.84087
11	20.48	2.06884	19.86133	29	19.86133	25	19.86133
15	20.5	2.15414	19.88179	30	19.88179	21	19.88179
17	20.52	2.25346	19.90225	24	19.90225	14	19.90225
19	20.54	2.3693	19.92272	27	19.92272	18	19.92272
24	20.56	2.50486	19.94318	34	19.94318	10	19.94318
26	20.58	2.66424	19.96364	49	19.96364	11	19.96364
27	20.6	2.85276	19.98411	54	19.98411	14	19.98411
23	20.62	3.07736	20.00457	53	20.00457	16	20.00457
21	20.64	3.34734	20.02503	55	20.02503	19	20.02503
27	20.66	3.67524	20.04549	56	20.04549	19	20.04549
27	20.68	4.0784	20.06596	55	20.06596	18	20.06596
31	20.7	4.17312	20.08642	45	20.08642	14	20.08642
42	20.72	4.82146	20.10688	34	20.10688	18	20.10688
61	20.74	5.65636	20.12734	31	20.12734	25	20.12734
92	20.76	6.75894	20.14781	28	20.14781	21	20.14781
143	20.78	9.7531	20.16827	28	20.16827	30	20.16827
229	20.8	11.95796	20.18873	18	20.18873	30	20.18873
364	20.82	15.32406	20.2092	8	20.2092	26	20.2092
571	20.84	20.818	20.22966	4	20.22966	19	20.22966
886	20.86	30.0372	20.25012	6	20.25012	15	20.25012
1288	20.88	44.9838	20.27058	8	20.27058	7	20.27058
1699	20.9	66.6228	20.29105	6	20.29105	2	20.29105
2052	20.92	89.6162	20.31151	5	20.31151	8	20.31151
2260	20.94	96.3726	20.33197	4	20.33197	5	20.33197
2273	20.96	79.3068	20.35244	14	20.35244	6	20.35244
2090	20.98	55.7556	20.3729	20	20.3729	17	20.3729
1766	21	37.5398	20.39336	22	20.39336	24	20.39336
1338	21.02	25.8692	20.41382	20	20.41382	28	20.41382
917	21.04	18.94274	20.43429	21	20.43429	30	20.43429
579	21.06	14.88382	20.45475	23	20.45475	25	20.45475
346	21.08	12.41234	20.47521	20	20.47521	20	20.47521
192	21.1	10.81898	20.49567	14	20.49567	14	20.49567
120	21.12	9.74698	20.51614	7	20.51614	15	20.51614
81	21.14	9.01326	20.5366	0	20.5366	14	20.5366
71	21.16	8.51478	20.55706	5	20.55706	19	20.55706
60	21.18	8.18806	20.57753	5	20.57753	28	20.57753
70	21.2	7.99184	20.59799	8	20.59799	34	20.59799
59	21.22	7.89856	20.61845	6	20.61845	48	20.61845

58	21.24	7.88964	20.63891	10	20.63891	58	20.63891
42	21.26	7.95252	20.65938	16	20.65938	54	20.65938
36	21.28	8.07894	20.67984	21	20.67984	62	20.67984
24	21.3	8.26374	20.7003	21	20.7003	66	20.7003
14	21.32	8.5042	20.72077	21	20.72077	72	20.72077
10	21.34	8.79952	20.74123	20	20.74123	75	20.74123
19	21.36	9.15056	20.76169	19	20.76169	82	20.76169
11	21.38	9.55978	20.78215	21	20.78215	79	20.78215
15	21.4	10.03118	20.80262	21	20.80262	77	20.80262
17	21.42	10.57034	20.82308	11	20.82308	102	20.82308
15	21.44	11.1847	20.84354	15	20.84354	121	20.84354
9	21.46	11.88376	20.864	17	20.864	132	20.864
16	21.48	12.6796	20.88447	24	20.88447	147	20.88447
13	21.5	13.58744	20.90493	23	20.90493	160	20.90493
10	21.52	14.62642	20.92539	32	20.92539	169	20.92539
7	21.54	15.8209	20.94586	47	20.94586	192	20.94586
5	21.56	17.20198	20.96632	63	20.96632	214	20.96632
1	21.58	18.80986	20.98678	82	20.98678	228	20.98678
0	21.6	20.6972	21.00724	109	21.00724	265	21.00724
7	21.62	22.9338	21.02771	130	21.02771	326	21.02771
14	21.64	25.614	21.04817	158	21.04817	377	21.04817
14	21.66	28.8682	21.06863	179	21.06863	420	21.06863
20	21.68	32.8798	21.0891	197	21.0891	437	21.0891
26	21.7	37.914	21.10956	204	21.10956	446	21.10956
29	21.72	44.3686	21.13002	212	21.13002	433	21.13002
28	21.74	52.8758	21.15048	213	21.15048	422	21.15048
26	21.76	64.5362	21.17095	195	21.17095	398	21.17095
14	21.78	81.4658	21.19141	172	21.19141	372	21.19141
7	21.8	107.855	21.21187	151	21.21187	354	21.21187
14	21.82	151.309	21.23233	125	21.23233	337	21.23233
17	21.84	223.136	21.2528	99	21.2528	319	21.2528
18	21.86	334.646	21.27326	84	21.27326	308	21.27326
23	21.88	480.604	21.29372	79	21.29372	309	21.29372
31	21.9	593.978	21.31419	80	21.31419	306	21.31419
29	21.92	566.28	21.33465	76	21.33465	306	21.33465
32	21.94	433.992	21.35511	80	21.35511	326	21.35511
23	21.96	306.824	21.37557	74	21.37557	347	21.37557
17	21.98	221.44	21.39604	60	21.39604	369	21.39604
25	22	173.4848	21.4165	54	21.4165	388	21.4165
31	22.02	151.477	21.43696	49	21.43696	404	21.43696
35	22.04	146.6472	21.45743	39	21.45743	416	21.45743
36	22.06	154.7686	21.47789	43	21.47789	431	21.47789
32	22.08	176.0066	21.49835	51	21.49835	459	21.49835
31	22.1	215.354	21.51881	59	21.51881	459	21.51881
20	22.12	284.9	21.53928	57	21.53928	475	21.53928
13	22.14	406.7	21.55974	73	21.55974	488	21.55974
0	22.16	611.56	21.5802	83	21.5802	505	21.5802
6	22.18	923.906	21.60066	88	21.60066	530	21.60066
16	22.2	1300.706	21.62113	104	21.62113	566	21.62113
22	22.22	1509.084	21.64159	110	21.64159	613	21.64159
24	22.24	1322.156	21.66205	117	21.66205	649	21.66205
23	22.26	943.89	21.68252	119	21.68252	685	21.68252
31	22.28	620.78	21.70298	128	21.70298	723	21.70298
34	22.3	405.104	21.72344	146	21.72344	742	21.72344
32	22.32	274.054	21.7439	168	21.7439	791	21.7439
23	22.34	196.1788	21.76437	198	21.76437	833	21.76437
20	22.36	148.1748	21.78483	223	21.78483	874	21.78483
25	22.38	116.4958	21.80529	256	21.80529	929	21.80529

34	22.4	94.1216	21.82576	289	21.82576	1016	21.82576
35	22.42	77.7248	21.84622	319	21.84622	1096	21.84622
36	22.44	65.2806	21.86668	373	21.86668	1179	21.86668
48	22.46	55.6136	21.88714	428	21.88714	1283	21.88714
72	22.48	47.9578	21.90761	520	21.90761	1405	21.90761
91	22.5	41.7936	21.92807	630	21.92807	1540	21.92807
140	22.52	36.7578	21.94853	767	21.94853	1743	21.94853
185	22.54	32.591	21.96899	935	21.96899	1991	21.96899
257	22.56	29.104	21.98946	1117	21.98946	2271	21.98946
335	22.58	26.1566	22.00992	1314	22.00992	2593	22.00992
409	22.6	23.6424	22.03038	1491	22.03038	2897	22.03038
446	22.62	21.4806	22.05085	1634	22.05085	3130	22.05085
471	22.64	19.60814	22.07131	1728	22.07131	3304	22.07131
451	22.66	17.97544	22.09177	1742	22.09177	3374	22.09177
402	22.68	16.54326	22.11223	1705	22.11223	3334	22.11223
333	22.7	15.2801	22.1327	1605	22.1327	3202	22.1327
270	22.72	14.16042	22.15316	1489	22.15316	3067	22.15316
192	22.74	13.1634	22.17362	1378	22.17362	2984	22.17362
159	22.76	12.27188	22.19409	1299	22.19409	2938	22.19409
142	22.78	11.47172	22.21455	1291	22.21455	2979	22.21455
128	22.8	10.75102	22.23501	1349	22.23501	3135	22.23501
125	22.82	10.09992	22.25547	1508	22.25547	3412	22.25547
144	22.84	9.51	22.27594	1726	22.27594	3810	22.27594
149	22.86	9.65256	22.2964	2007	22.2964	4305	22.2964
163	22.88	9.18392	22.31686	2352	22.31686	4841	22.31686
172	22.9	8.7591	22.33732	2724	22.33732	5399	22.33732
173	22.92	8.37378	22.35779	3035	22.35779	5906	22.35779
175	22.94	8.02434	22.37825	3238	22.37825	6255	22.37825
193	22.96	7.70766	22.39871	3302	22.39871	6379	22.39871
196	22.98	7.42114	22.41918	3210	22.41918	6301	22.41918
192	23	7.16268	22.43964	2985	22.43964	6024	22.43964
198	23.02	6.93054	22.4601	2661	22.4601	5568	22.4601
205	23.04	6.72346	22.48056	2258	22.48056	5031	22.48056
214	23.06	6.54052	22.50103	1852	22.50103	4512	22.50103
221	23.08	6.3813	22.52149	1507	22.52149	4041	22.52149
234	23.1	6.24586	22.54195	1230	22.54195	3685	22.54195
247	23.12	6.13486	22.56242	1002	22.56242	3422	22.56242
283	23.14	6.04966	22.58288	843	22.58288	3213	22.58288
318	23.16	5.99268	22.60334	718	22.60334	3106	22.60334
344	23.18	5.96754	22.6238	625	22.6238	3042	22.6238
373	23.2	5.97988	22.64427	563	22.64427	2978	22.64427
404	23.22	6.03814	22.66473	502	22.66473	2895	22.66473
435	23.24	6.15512	22.68519	459	22.68519	2847	22.68519
466	23.26	6.3509	22.70565	421	22.70565	2779	22.70565
501	23.28	6.65882	22.72612	392	22.72612	2728	22.72612
541	23.3	7.1399	22.74658	354	22.74658	2684	22.74658
561	23.32	7.91512	22.76704	332	22.76704	2623	22.76704
596	23.34	9.22132	22.78751	321	22.78751	2571	22.78751
631	23.36	11.4661	22.80797	305	22.80797	2551	22.80797
675	23.38	14.65636	22.82843	293	22.82843	2509	22.82843
724	23.4	20.291	22.84889	277	22.84889	2460	22.84889
804	23.42	26.8936	22.86936	259	22.86936	2429	22.86936
918	23.44	30.1464	22.88982	252	22.88982	2401	22.88982
1120	23.46	26.4868	22.91028	236	22.91028	2349	22.91028
1452	23.48	19.89048	22.93075	224	22.93075	2327	22.93075
1911	23.5	14.4265	22.95121	206	22.95121	2267	22.95121
2515	23.52	10.84304	22.97167	197	22.97167	2193	22.97167
3167	23.54	8.709	22.99213	189	22.99213	2131	22.99213

3747	23.56	7.48048	23.0126	183	23.0126	2073	23.0126
4151	23.58	6.76384	23.03306	182	23.03306	1988	23.03306
4292	23.6	6.33376	23.05352	175	23.05352	1903	23.05352
4147	23.62	6.07632	23.07398	163	23.07398	1833	23.07398
3758	23.64	5.9344	23.09445	150	23.09445	1745	23.09445
3246	23.66	5.8773	23.11491	128	23.11491	1678	23.11491
2731	23.68	4.43692	23.13537	118	23.13537	1609	23.13537
2319	23.7	4.54202	23.15584	99	23.15584	1527	23.15584
2106	23.72	4.69584	23.1763	96	23.1763	1470	23.1763
2121	23.74	4.89554	23.19676	95	23.19676	1421	23.19676
2362	23.76	5.14062	23.21722	91	23.21722	1386	23.21722
2893	23.78	5.4325	23.23769	91	23.23769	1336	23.23769
3771	23.8	5.77436	23.25815	83	23.25815	1278	23.25815
4985	23.82	6.171	23.27861	79	23.27861	1219	23.27861
6335	23.84	6.6291	23.29908	74	23.29908	1164	23.29908
7550	23.86	7.1574	23.31954	66	23.31954	1103	23.31954
8419	23.88	7.7671	23.34	63	23.34	1048	23.34
8740	23.9	8.47242	23.36046	61	23.36046	997	23.36046
8451	23.92	9.29156	23.38093	70	23.38093	968	23.38093
7621	23.94	10.24766	23.40139	68	23.40139	943	23.40139
6370	23.96	11.37058	23.42185	61	23.42185	910	23.42185
5043	23.98	12.6991	23.44231	63	23.44231	884	23.44231
3910	24	14.2843	23.46278	57	23.46278	856	23.46278
3092	24.02	16.19424	23.48324	58	23.48324	832	23.48324
2534	24.04	18.52122	23.5037	59	23.5037	807	23.5037
2214	24.06	21.3926	23.52417	59	23.52417	766	23.52417
2078	24.08	24.9878	23.54463	56	23.54463	740	23.54463
2019	24.1	29.5658	23.56509	59	23.56509	717	23.56509
1985	24.12	35.5104	23.58555	56	23.58555	700	23.58555
1931	24.14	43.419	23.60602	56	23.60602	670	23.60602
1901	24.16	54.2924	23.62648	62	23.62648	637	23.62648
1893	24.18	69.9884	23.64694	76	23.64694	609	23.64694
1875	24.2	94.2052	23.66741	76	23.66741	589	23.66741
1858	24.22	134.0566	23.68787	79	23.68787	558	23.68787
1818	24.24	201.322	23.70833	76	23.70833	527	23.70833
1817	24.26	311.094	23.72879	61	23.72879	480	23.72879
1811	24.28	470.18	23.74926	46	23.74926	434	23.74926
1791	24.3	640.106	23.76972	31	23.76972	404	23.76972
1766	24.32	691.196	23.79018	17	23.79018	364	23.79018
1750	24.34	565.234	23.81064	9	23.81064	338	23.81064
1732	24.36	389.822	23.83111	6	23.83111	319	23.83111
1707	24.38	253.492	23.85157	15	23.85157	302	23.85157
1663	24.4	165.6572	23.87203	18	23.87203	306	23.87203
1619	24.42	113.044	23.8925	29	23.8925	298	23.8925
1570	24.44	81.7188	23.91296	32	23.91296	292	23.91296
1531	24.46	62.15	23.93342	27	23.93342	278	23.93342
1477	24.48	49.0446	23.95388	29	23.95388	270	23.95388
1422	24.5	39.7358	23.97435	27	23.97435	260	23.97435
1392	24.52	32.853	23.99481	23	23.99481	252	23.99481
1359	24.54	27.6214	24.01527	20	24.01527	251	24.01527
1304	24.56	23.56	24.03574	19	24.03574	254	24.03574
1289	24.58	20.3494	24.0562	26	24.0562	255	24.0562
1247	24.6	17.7646	24.07666	32	24.07666	258	24.07666
1200	24.62	15.6456	24.09712	42	24.09712	266	24.09712
1165	24.64	13.88988	24.11759	50	24.11759	264	24.11759
1108	24.66	12.42598	24.13805	47	24.13805	264	24.13805
1043	24.68	11.19616	24.15851	61	24.15851	274	24.15851
982	24.7	10.15472	24.17897	83	24.17897	295	24.17897

932	24.72	9.2661	24.19944	106	24.19944	320	24.19944
875	24.74	8.50276	24.2199	135	24.2199	340	24.2199
825	24.76	7.84322	24.24036	162	24.24036	375	24.24036
794	24.78	7.27062	24.26083	207	24.26083	414	24.26083
757	24.8	6.77148	24.28129	262	24.28129	460	24.28129
732	24.82	6.335	24.30175	324	24.30175	529	24.30175
693	24.84	5.95242	24.32221	405	24.32221	614	24.32221
655	24.86	5.61656	24.34268	498	24.34268	739	24.34268
616	24.88	5.32156	24.36314	663	24.36314	927	24.36314
573	24.9	5.03726	24.3836	850	24.3836	1153	24.3836
536	24.92	4.81098	24.40407	1054	24.40407	1403	24.40407
500	24.94	4.61344	24.42453	1280	24.42453	1670	24.42453
470	24.96	4.44192	24.44499	1476	24.44499	1917	24.44499
450	24.98	4.29424	24.46545	1640	24.46545	2110	24.46545
446	25	4.1686	24.48592	1720	24.48592	2209	24.48592
455	25.02	4.06366	24.50638	1709	24.50638	2200	24.50638
454	25.04	3.97834	24.52684	1607	24.52684	2086	24.52684
462	25.06	3.91194	24.5473	1430	24.5473	1869	24.5473
465	25.08	4.8324	24.56777	1243	24.56777	1609	24.56777
443	25.1	4.83024	24.58823	1024	24.58823	1322	24.58823
421	25.12	4.84778	24.60869	807	24.60869	1046	24.60869
377	25.14	4.88552	24.62916	625	24.62916	820	24.62916
341	25.16	4.9444	24.64962	472	24.64962	631	24.64962
298	25.18	5.02564	24.67008	356	24.67008	489	24.67008
259	25.2	5.13102	24.69054	268	24.69054	386	24.69054
225	25.22	5.26274	24.71101	199	24.71101	319	24.71101
203	25.24	5.42372	24.73147	144	24.73147	273	24.73147
174	25.26	5.6176	24.75193	106	24.75193	238	24.75193
161	25.28	5.84898	24.7724	90	24.7724	220	24.7724
136	25.3	6.12376	24.79286	75	24.79286	200	24.79286
127	25.32	6.4493	24.81332	50	24.81332	164	24.81332
105	25.34	6.86688	24.83378	36	24.83378	145	24.83378
107	25.36	7.32614	24.85425	29	24.85425	112	24.85425
110	25.38	7.87388	24.87471	21	24.87471	85	24.87471
106	25.4	8.53108	24.89517	16	24.89517	71	24.89517
101	25.42	9.32576	24.91563	8	24.91563	62	24.91563
99	25.44	10.29598	24.9361	11	24.9361	67	24.9361
92	25.46	11.4944	24.95656	18	24.95656	65	24.95656
95	25.48	12.99526	24.97702	22	24.97702	65	24.97702
87	25.5	14.90594	24.99749	22	24.99749	72	24.99749
82	25.52	17.38656	25.01795	21	25.01795	66	25.01795
72	25.54	20.6874	25.03841	20	25.03841	57	25.03841
81	25.56	25.2328	25.05887	18	25.05887	50	25.05887
97	25.58	31.8188	25.07934	12	25.07934	53	25.07934
100	25.6	42.033	25.0998	2	25.0998	53	25.0998
105	25.62	58.8942	25.12026	0	25.12026	55	25.12026
106	25.64	87.2804	25.14073	0	25.14073	64	25.14073
112	25.66	133.0536	25.16119	1	25.16119	58	25.16119
121	25.68	198.1278	25.18165	0	25.18165	62	25.18165
124	25.7	263.34	25.20211	0	25.20211	71	25.20211
131	25.72	275.062	25.22258	1	25.22258	58	25.22258
157	25.74	219.932	25.24304	3	25.24304	47	25.24304
192	25.76	151.366	25.2635	4	25.2635	38	25.2635
257	25.78	99.1588	25.28396	5	25.28396	29	25.28396
361	25.8	66.4592	25.30443	6	25.30443	11	25.30443
540	25.82	47.0496	25.32489	3	25.32489	7	25.32489
835	25.84	35.566	25.34535	3	25.34535	3	25.34535
1265	25.86	28.4488	25.36582	5	25.36582	8	25.36582

1824	25.88	23.7566	25.38628	8	25.38628	18	25.38628
2397	25.9	20.516	25.40674	11	25.40674	26	25.40674
2897	25.92	18.22462	25.4272	14	25.4272	28	25.4272
3214	25.94	16.59682	25.44767	16	25.44767	27	25.44767
3281	25.96	15.45626	25.46813	16	25.46813	28	25.46813
3090	25.98	14.68888	25.48859	13	25.48859	19	25.48859
2666	26	14.21958	25.50906	9	25.50906	20	25.50906
2105	26.02	13.9993	25.52952	2	25.52952	23	25.52952
1518	26.04	13.99716	25.54998	0	25.54998	25	25.54998
1018	26.06	14.1957	25.57044	0	25.57044	37	25.57044
659	26.08	14.58848	25.59091	0	25.59091	62	25.59091
411	26.1	15.17828	25.61137	10	25.61137	80	25.61137
275	26.12	15.97696	25.63183	32	25.63183	90	25.63183
198	26.14	17.00598	25.65229	52	25.65229	116	25.65229
163	26.16	18.29768	25.67276	73	25.67276	131	25.67276
137	26.18	19.89796	25.69322	99	25.69322	156	25.69322
114	26.2	21.8702	25.71368	133	25.71368	200	25.71368
88	26.22	24.301	25.73415	170	25.73415	247	25.73415
59	26.24	27.3098	25.75461	209	25.75461	300	25.75461
48	26.26	31.0626	25.77507	284	25.77507	393	25.77507
35	26.28	35.7938	25.79553	371	25.79553	528	25.79553
28	26.3	41.8416	25.816	475	25.816	687	25.816
25	26.32	49.706	25.83646	601	25.83646	850	25.83646
22	26.34	60.1582	25.85692	720	25.85692	1004	25.85692
17	26.36	75.1178	25.87739	818	25.87739	1117	25.87739
6	26.38	95.6406	25.89785	876	25.89785	1185	25.89785
3	26.4	126.9622	25.91831	902	25.91831	1199	25.91831
0	26.42	178.0696	25.93877	867	25.93877	1127	25.93877
2	26.44	264.324	25.95924	777	25.95924	1001	25.95924
11	26.46	406.208	25.9797	667	25.9797	860	25.9797
25	26.48	617.798	26.00016	523	26.00016	710	26.00016
34	26.5	861.742	26.02062	394	26.02062	579	26.02062
36	26.52	973.64	26.04109	294	26.04109	456	26.04109
38	26.54	829.596	26.06155	206	26.06155	352	26.06155
33	26.56	583.764	26.08201	147	26.08201	279	26.08201
25	26.58	382.316	26.10248	109	26.10248	212	26.10248
15	26.6	250.09	26.12294	93	26.12294	179	26.12294
12	26.62	170.5708	26.1434	86	26.1434	136	26.1434
18	26.64	123.7448	26.16386	83	26.16386	111	26.16386
22	26.66	95.3544	26.18433	88	26.18433	97	26.18433
29	26.68	77.4592	26.20479	86	26.20479	91	26.20479
26	26.7	66.3584	26.22525	102	26.22525	94	26.22525
24	26.72	60.5894	26.24572	109	26.24572	103	26.24572
22	26.74	59.5326	26.26618	96	26.26618	106	26.26618
14	26.76	61.7286	26.28664	91	26.28664	115	26.28664
6	26.78	62.0326	26.3071	83	26.3071	120	26.3071
1	26.8	54.8686	26.32757	85	26.32757	126	26.32757
6	26.82	43.8418	26.34803	99	26.34803	137	26.34803
15	26.84	34.321	26.36849	119	26.36849	162	26.36849
17	26.86	27.5178	26.38895	149	26.38895	181	26.38895
17	26.88	22.9032	26.40942	173	26.40942	221	26.40942
14	26.9	19.73558	26.42988	226	26.42988	273	26.42988
22	26.92	17.45244	26.45034	279	26.45034	329	26.45034
28	26.94	15.7136	26.47081	326	26.47081	404	26.47081
38	26.96	14.33382	26.49127	408	26.49127	521	26.49127
40	26.98	13.2111	26.51173	528	26.51173	636	26.51173
56	27	12.28386	26.53219	681	26.53219	785	26.53219
65	27.02	11.51122	26.55266	874	26.55266	988	26.55266

70	27.04	10.86436	26.57312	1114	26.57312	1250	26.57312
76	27.06	10.32226	26.59358	1399	26.59358	1587	26.59358
83	27.08	9.86926	26.61405	1724	26.61405	1976	26.61405
93	27.1	9.49358	26.63451	2082	26.63451	2336	26.63451
118	27.12	9.97604	26.65497	2383	26.65497	2679	26.65497
145	27.14	9.75282	26.67543	2620	26.67543	2933	26.67543
206	27.16	9.5873	26.6959	2758	26.6959	3049	26.6959
306	27.18	9.47608	26.71636	2759	26.71636	2989	26.71636
474	27.2	9.15936	26.73682	2618	26.73682	2776	26.73682
737	27.22	9.1581	26.75728	2372	26.75728	2448	26.75728
1136	27.24	9.20764	26.77775	2048	26.77775	2066	26.77775
1608	27.26	9.30912	26.79821	1697	26.79821	1698	26.79821
2056	27.28	9.46472	26.81867	1352	26.81867	1339	26.81867
2390	27.3	9.67788	26.83914	1060	26.83914	1018	26.83914
2566	27.32	9.95336	26.8596	810	26.8596	799	26.8596
2522	27.34	10.29752	26.88006	628	26.88006	620	26.88006
2271	27.36	10.71866	26.90052	487	26.90052	495	26.90052
1875	27.38	11.2274	26.92099	390	26.92099	412	26.92099
1395	27.4	11.83736	26.94145	325	26.94145	350	26.94145
960	27.42	12.56596	26.96191	280	26.96191	310	26.96191
645	27.44	13.43554	26.98238	250	26.98238	275	26.98238
415	27.46	14.475	27.00284	218	27.00284	240	27.00284
263	27.48	15.7221	27.0233	176	27.0233	199	27.0233
169	27.5	17.22662	27.04376	145	27.04376	164	27.04376
140	27.52	19.05528	27.06423	113	27.06423	128	27.06423
115	27.54	21.2986	27.08469	87	27.08469	91	27.08469
97	27.56	24.0822	27.10515	66	27.10515	73	27.10515
94	27.58	27.583	27.12561	59	27.12561	67	27.12561
87	27.6	32.0572	27.14608	55	27.14608	61	27.14608
87	27.62	37.8872	27.16654	51	27.16654	53	27.16654
87	27.64	45.6708	27.187	51	27.187	48	27.187
73	27.66	56.421	27.20747	50	27.20747	38	27.20747
65	27.68	72.0398	27.22793	42	27.22793	32	27.22793
60	27.7	96.3206	27.24839	31	27.24839	30	27.24839
62	27.72	136.4768	27.26885	20	27.26885	27	27.26885
55	27.74	204.166	27.28932	16	27.28932	22	27.28932
57	27.76	313.404	27.30978	13	27.30978	28	27.30978
73	27.78	468.776	27.33024	12	27.33024	24	27.33024
100	27.8	624.516	27.35071	8	27.35071	10	27.35071
123	27.82	652.438	27.37117	6	27.37117	4	27.37117
151	27.84	520.484	27.39163	6	27.39163	12	27.39163
176	27.86	356.324	27.41209	10	27.41209	17	27.41209
221	27.88	232.836	27.43256	10	27.43256	16	27.43256
289	27.9	154.3254	27.45302	7	27.45302	21	27.45302
371	27.92	107.5718	27.47348	3	27.47348	33	27.47348
474	27.94	79.7478	27.49394	7	27.49394	38	27.49394
647	27.96	62.339	27.51441	4	27.51441	34	27.51441
945	27.98	49.7602	27.53487	8	27.53487	25	27.53487
1485	28	41.5824	27.55533	10	27.55533	20	27.55533
2278	28.02	35.6344	27.5758	16	27.5758	20	27.5758
3235	28.04	31.229	27.59626	23	27.59626	30	27.59626
4203	28.06	27.9388	27.61672	37	27.61672	37	27.61672
4992	28.08	25.484	27.63718	46	27.63718	41	27.63718
5523	28.1	23.678	27.65765	70	27.65765	62	27.65765
5620	28.12	22.394	27.67811	88	27.67811	96	27.67811
5248	28.14	21.548	27.69857	105	27.69857	112	27.69857
4508	28.16	21.0862	27.71904	119	27.71904	128	27.71904
3548	28.18	20.9782	27.7395	148	27.7395	150	27.7395

2608	28.2	21.214	27.75996	174	27.75996	177	27.75996
1814	28.22	21.8018	27.78042	207	27.78042	221	27.78042
1204	28.24	22.7382	27.80089	265	27.80089	268	27.80089
774	28.26	24.136	27.82135	351	27.82135	338	27.82135
502	28.28	26.0406	27.84181	451	27.84181	435	27.84181
386	28.3	28.5672	27.86227	590	27.86227	571	27.86227
323	28.32	32.361	27.88274	742	27.88274	739	27.88274
290	28.34	36.7254	27.9032	935	27.9032	940	27.9032
274	28.36	42.4898	27.92366	1123	27.92366	1170	27.92366
246	28.38	50.2138	27.94413	1318	27.94413	1402	27.94413
206	28.4	60.8052	27.96459	1461	27.96459	1601	27.96459
176	28.42	75.8828	27.98505	1544	27.98505	1738	27.98505
146	28.44	98.6098	28.00551	1565	28.00551	1771	28.00551
116	28.46	135.2534	28.02598	1508	28.02598	1726	28.02598
91	28.48	197.0136	28.04644	1369	28.04644	1593	28.04644
72	28.5	300.034	28.0669	1193	28.0669	1396	28.0669
61	28.52	459.086	28.08737	983	28.08737	1160	28.08737
53	28.54	660.588	28.10783	804	28.10783	939	28.10783
45	28.56	797.166	28.12829	620	28.12829	734	28.12829
35	28.58	727.772	28.14875	489	28.14875	572	28.14875
24	28.6	530.792	28.16922	382	28.16922	439	28.16922
15	28.62	351.042	28.18968	310	28.18968	338	28.18968
11	28.64	228.356	28.21014	260	28.21014	258	28.21014
9	28.66	153.2336	28.2306	221	28.2306	216	28.2306
2	28.68	108.7108	28.25107	187	28.25107	183	28.25107
11	28.7	81.5662	28.27153	170	28.27153	161	28.27153
7	28.72	63.8958	28.29199	142	28.29199	166	28.29199
14	28.74	51.61	28.31246	130	28.31246	165	28.31246
18	28.76	42.6506	28.33292	107	28.33292	170	28.33292
19	28.78	35.9032	28.35338	116	28.35338	183	28.35338
19	28.8	30.7002	28.37384	117	28.37384	182	28.37384
16	28.82	26.61	28.39431	130	28.39431	197	28.39431
12	28.84	23.3416	28.41477	143	28.41477	192	28.41477
10	28.86	20.6934	28.43523	160	28.43523	203	28.43523
5	28.88	18.52144	28.4557	187	28.4557	213	28.4557
6	28.9	16.7218	28.47616	217	28.47616	231	28.47616
2	28.92	15.21752	28.49662	261	28.49662	291	28.49662
11	28.94	13.95088	28.51708	309	28.51708	337	28.51708
21	28.96	12.87804	28.53755	384	28.53755	414	28.53755
23	28.98	11.96518	28.55801	484	28.55801	519	28.55801
33	29	11.18602	28.57847	591	28.57847	652	28.57847
38	29.02	10.51994	28.59893	750	28.59893	818	28.59893
44	29.04	10.04218	28.6194	938	28.6194	1025	28.6194
58	29.06	9.5594	28.63986	1186	28.63986	1283	28.63986
61	29.08	9.15036	28.66032	1454	28.66032	1590	28.66032
81	29.1	8.80696	28.68079	1736	28.68079	1913	28.68079
102	29.12	8.52288	28.70125	2029	28.70125	2246	28.70125
122	29.14	8.29322	28.72171	2243	28.72171	2495	28.72171
151	29.16	8.11436	28.74217	2384	28.74217	2662	28.74217
192	29.18	7.9838	28.76264	2420	28.76264	2705	28.76264
248	29.2	7.90004	28.7831	2328	28.7831	2606	28.7831
343	29.22	7.86258	28.80356	2145	28.80356	2373	28.80356
497	29.24	7.87186	28.82403	1888	28.82403	2064	28.82403
749	29.26	8.80648	28.84449	1602	28.84449	1714	28.84449
1128	29.28	8.93942	28.86495	1287	28.86495	1376	28.86495
1636	29.3	8.5122	28.88541	1009	28.88541	1075	28.88541
2138	29.32	8.77806	28.90588	779	28.90588	838	28.90588
2571	29.34	9.11142	28.92634	574	28.92634	652	28.92634

2869	29.36	9.5216	28.9468	424	28.9468	521	28.9468
2983	29.38	10.02072	28.96726	327	28.96726	420	28.96726
2872	29.4	10.62438	28.98773	243	28.98773	345	28.98773
2549	29.42	11.35298	29.00819	195	29.00819	287	29.00819
2082	29.44	12.2331	29.02865	157	29.02865	237	29.02865
1584	29.46	13.29994	29.04912	119	29.04912	201	29.04912
1155	29.48	14.60056	29.06958	92	29.06958	160	29.06958
822	29.5	16.19886	29.09004	71	29.09004	123	29.09004
544	29.52	18.1831	29.1105	53	29.1105	94	29.1105
363	29.54	20.6776	29.13097	48	29.13097	66	29.13097
245	29.56	23.8614	29.15143	45	29.15143	54	29.15143
192	29.58	28.0004	29.17189	50	29.17189	45	29.17189
168	29.6	33.5062	29.19236	46	29.19236	35	29.19236
133	29.62	41.0664	29.21282	46	29.21282	39	29.21282
108	29.64	51.9512	29.23328	37	29.23328	35	29.23328
90	29.66	68.6852	29.25374	24	29.25374	40	29.25374
78	29.68	96.146	29.27421	22	29.27421	36	29.27421
73	29.7	142.4858	29.29467	16	29.29467	25	29.29467
62	29.72	218.146	29.31513	9	29.31513	22	29.31513
63	29.74	328.92	29.33559	13	29.33559	17	29.33559
57	29.76	450.056	29.35606	9	29.35606	27	29.35606
65	29.78	492.214	29.37652	5	29.37652	23	29.37652
78	29.8	407.752	29.39698	2	29.39698	16	29.39698
97	29.82	283.774	29.41745	6	29.41745	14	29.41745
116	29.84	186.0544	29.43791	6	29.43791	7	29.43791
147	29.86	122.7994	29.45837	3	29.45837	11	29.45837
166	29.88	84.8948	29.47883	3	29.47883	7	29.47883
217	29.9	62.4132	29.4993	6	29.4993	0	29.4993
270	29.92	48.4762	29.51976	11	29.51976	5	29.51976
354	29.94	39.237	29.54022	14	29.54022	16	29.54022
482	29.96	32.7544	29.56068	15	29.56068	20	29.56068
725	29.98	28.0348	29.58115	9	29.58115	25	29.58115
1134	30	24.5198	29.60161	10	29.60161	32	29.60161
1759	30.02	21.8656	29.62207	14	29.62207	37	29.62207
2553	30.04	19.09216	29.64254	23	29.64254	52	29.64254
3424	30.06	17.57806	29.663	26	29.663	63	29.663
4169	30.08	16.44302	29.68346	30	29.68346	74	29.68346
4673	30.1	15.6147	29.70392	49	29.70392	83	29.70392
4827	30.12	15.0432	29.72439	77	29.72439	112	29.72439
4617	30.14	14.6949	29.74485	104	29.74485	146	29.74485
4065	30.16	14.67252	29.76531	131	29.76531	173	29.76531
3299	30.18	14.72156	29.78578	165	29.78578	226	29.78578
2488	30.2	14.9614	29.80624	214	29.80624	305	29.80624
1756	30.22	15.40056	29.8267	287	29.8267	410	29.8267
1197	30.24	16.05844	29.84716	394	29.84716	544	29.84716
801	30.26	16.96806	29.86763	527	29.86763	711	29.86763
516	30.28	18.18116	29.88809	678	29.88809	913	29.88809
333	30.3	19.77764	29.90855	844	29.90855	1115	29.90855
243	30.32	21.885	29.92901	1002	29.92901	1330	29.92901
196	30.34	24.7236	29.94948	1129	29.94948	1482	29.94948
166	30.36	28.7084	29.96994	1183	29.96994	1566	29.96994
138	30.38	34.6372	29.9904	1194	29.9904	1595	29.9904
125	30.4	43.9028	30.01087	1146	30.01087	1559	30.01087
101	30.42	58.4796	30.03133	1048	30.03133	1430	30.03133
90	30.44	80.1382	30.05179	920	30.05179	1258	30.05179
71	30.46	107.1276	30.07225	787	30.07225	1055	30.07225
56	30.48	126.5816	30.09272	642	30.09272	855	30.09272
46	30.5	123.212	30.11318	506	30.11318	671	30.11318

38	30.52	107.8648	30.13364	399	30.13364	531	30.13364
21	30.54	97.5898	30.15411	308	30.15411	401	30.15411
12	30.56	97.8086	30.17457	226	30.17457	311	30.17457
11	30.58	109.742	30.19503	176	30.19503	254	30.19503
15	30.6	136.2826	30.21549	142	30.21549	218	30.21549
16	30.62	185.2428	30.23596	111	30.23596	175	30.23596
21	30.64	271.084	30.25642	84	30.25642	157	30.25642
22	30.66	412.512	30.27688	73	30.27688	136	30.27688
22	30.68	618.222	30.29734	57	30.29734	119	30.29734
17	30.7	835.812	30.31781	44	30.31781	118	30.31781
10	30.72	896.026	30.33827	39	30.33827	107	30.33827
4	30.74	729.3	30.35873	34	30.35873	112	30.35873
5	30.76	502.602	30.3792	34	30.3792	113	30.3792
10	30.78	327.51	30.39966	34	30.39966	109	30.39966
9	30.8	214.906	30.42012	42	30.42012	107	30.42012
10	30.82	147.4412	30.44058	47	30.44058	101	30.44058
13	30.84	107.1922	30.46105	55	30.46105	111	30.46105
16	30.86	82.2396	30.48151	78	30.48151	115	30.48151
17	30.88	65.296	30.50197	97	30.50197	121	30.50197
21	30.9	53.227	30.52244	124	30.52244	151	30.52244
20	30.92	44.2808	30.5429	155	30.5429	181	30.5429
21	30.94	37.4636	30.56336	194	30.56336	233	30.56336
26	30.96	32.1568	30.58382	235	30.58382	277	30.58382
26	30.98	27.9518	30.60429	274	30.60429	341	30.60429
36	31	24.5692	30.62475	317	30.62475	405	30.62475
43	31.02	21.8128	30.64521	361	30.64521	469	30.64521
63	31.04	19.54204	30.66567	416	30.66567	540	30.66567
90	31.06	17.65398	30.68614	471	30.68614	594	30.68614
115	31.08	16.12678	30.7066	528	30.7066	656	30.7066
151	31.1	14.7955	30.72706	608	30.72706	740	30.72706
197	31.12	13.6694	30.74753	701	30.74753	846	30.74753
270	31.14	12.7149	30.76799	837	30.76799	979	30.76799
384	31.16	11.90616	30.78845	1000	30.78845	1168	30.78845
576	31.18	11.22328	30.80891	1197	30.80891	1427	30.80891
880	31.2	10.65114	30.82938	1431	30.82938	1737	30.82938
1292	31.22	10.17854	30.84984	1675	30.84984	2049	30.84984
1775	31.24	9.3248	30.8703	1917	30.8703	2320	30.8703
2216	31.26	9.04344	30.89077	2101	30.89077	2512	30.89077
2561	31.28	8.84674	30.91123	2194	30.91123	2607	30.91123
2742	31.3	8.7361	30.93169	2185	30.93169	2568	30.93169
2755	31.32	8.71654	30.95215	2065	30.95215	2397	30.95215
2549	31.34	8.79752	30.97262	1869	30.97262	2110	30.97262
2176	31.36	8.99432	30.99308	1605	30.99308	1789	30.99308
1726	31.38	9.33028	31.01354	1321	31.01354	1459	31.01354
1292	31.4	9.84036	31.034	1038	31.034	1163	31.034
937	31.42	10.57714	31.05447	801	31.05447	893	31.05447
646	31.44	11.62136	31.07493	622	31.07493	672	31.07493
426	31.46	13.1027	31.09539	474	31.09539	515	31.09539
285	31.48	15.24766	31.11586	360	31.11586	423	31.11586
192	31.5	18.4922	31.13632	280	31.13632	341	31.13632
154	31.52	23.707	31.15678	218	31.15678	276	31.15678
125	31.54	32.502	31.17724	170	31.17724	217	31.17724
99	31.56	47.343	31.19771	137	31.19771	180	31.19771
74	31.58	70.8726	31.21817	107	31.21817	142	31.21817
73	31.6	102.7012	31.23863	80	31.23863	113	31.23863
72	31.62	129.7754	31.2591	69	31.2591	84	31.2591
58	31.64	126.6732	31.27956	57	31.27956	52	31.27956
59	31.66	96.9612	31.30002	41	31.30002	36	31.30002

55	31.68	66.0976	31.32048	27	31.32048	30	31.32048
46	31.7	44.1314	31.34095	23	31.34095	16	31.34095
51	31.72	30.4762	31.36141	19	31.36141	6	31.36141
57	31.74	22.4046	31.38187	13	31.38187	5	31.38187
62	31.76	17.58134	31.40233	11	31.40233	3	31.40233
60	31.78	14.5417	31.4228	5	31.4228	9	31.4228
73	31.8	12.51104	31.44326	11	31.44326	15	31.44326
79	31.82	11.10294	31.46372	12	31.46372	17	31.46372
98	31.84	10.11496	31.48419	16	31.48419	15	31.48419
126	31.86	9.4308	31.50465	15	31.50465	16	31.50465
188	31.88	8.97912	31.52511	6	31.52511	19	31.52511
268	31.9	8.71562	31.54557	10	31.54557	20	31.54557
366	31.92	8.6952	31.56604	18	31.56604	27	31.56604
464	31.94	8.74364	31.5865	25	31.5865	31	31.5865
565	31.96	8.84974	31.60696	28	31.60696	40	31.60696
648	31.98	9.19654	31.62743	39	31.62743	62	31.62743
717	32	9.71044	31.64789	68	31.64789	74	31.64789
738	32.02	10.41896	31.66835	100	31.66835	92	31.66835
766	32.04	11.36456	31.68881	130	31.68881	127	31.68881
808	32.06	12.61036	31.70928	169	31.70928	169	31.70928
978	32.08	14.24998	31.72974	210	31.72974	220	31.72974
1265	32.1	16.42394	31.7502	263	31.7502	286	31.7502
1761	32.12	19.3508	31.77066	317	31.77066	346	31.77066
2501	32.14	23.3934	31.79113	358	31.79113	383	31.79113
3332	32.16	29.2168	31.81159	375	31.81159	407	31.81159
4095	32.18	37.2736	31.83205	390	31.83205	419	31.83205
4662	32.2	51.8824	31.85252	380	31.85252	397	31.85252
4927	32.22	76.5214	31.87298	357	31.87298	354	31.87298
4881	32.24	116.9752	31.89344	308	31.89344	308	31.89344
4492	32.26	177.0972	31.9139	262	31.9139	247	31.9139
3846	32.28	245.832	31.93437	210	31.93437	189	31.93437
3044	32.3	276.244	31.95483	159	31.95483	153	31.95483
2287	32.32	234.862	31.97529	127	31.97529	112	31.97529
1674	32.34	166.1486	31.99576	98	31.99576	80	31.99576
1166	32.36	110.5482	32.01622	73	32.01622	63	32.01622
775	32.38	74.7242	32.03668	61	32.03668	59	32.03668
501	32.4	54.1912	32.05714	48	32.05714	52	32.05714
336	32.42	43.8182	32.07761	36	32.07761	40	32.07761
256	32.44	40.454	32.09807	31	32.09807	34	32.09807
211	32.46	42.9698	32.11853	28	32.11853	22	32.11853
169	32.48	51.008	32.13899	25	32.13899	20	32.13899
133	32.5	61.9114	32.15946	24	32.15946	32	32.15946
108	32.52	66.1418	32.17992	37	32.17992	31	32.17992
95	32.54	56.4402	32.20038	52	32.20038	48	32.20038
81	32.56	42.1	32.22085	69	32.22085	68	32.22085
72	32.58	29.6926	32.24131	89	32.24131	85	32.24131
47	32.6	21.3762	32.26177	115	32.26177	113	32.26177
34	32.62	16.17986	32.28223	132	32.28223	149	32.28223
17	32.64	12.93924	32.3027	161	32.3027	189	32.3027
14	32.66	10.81792	32.32316	199	32.32316	239	32.32316
15	32.68	9.33402	32.34362	255	32.34362	322	32.34362
16	32.7	8.2381	32.36409	334	32.36409	424	32.36409
22	32.72	7.39968	32.38455	422	32.38455	527	32.38455
26	32.74	6.74388	32.40501	529	32.40501	655	32.40501
29	32.76	6.2234	32.42547	639	32.42547	791	32.42547
30	32.78	5.80904	32.44594	740	32.44594	929	32.44594
24	32.8	5.47364	32.4664	832	32.4664	1048	32.4664
23	32.82	5.20392	32.48686	890	32.48686	1131	32.48686

10	32.84	4.98846	32.50732	903	32.50732	1155	32.50732
2	32.86	4.8189	32.52779	903	32.52779	1148	32.52779
2	32.88	4.68916	32.54825	873	32.54825	1091	32.54825
12	32.9	4.59484	32.56871	818	32.56871	990	32.56871
24	32.92	4.53284	32.58918	738	32.58918	883	32.58918
48	32.94	4.50124	32.60964	667	32.60964	773	32.60964
87	32.96	4.49904	32.6301	604	32.6301	683	32.6301
126	32.98	4.52616	32.65056	540	32.65056	615	32.65056
226	33	4.58346	32.67103	494	32.67103	557	32.67103
355	33.02	4.67284	32.69149	439	32.69149	518	32.69149
523	33.04	4.7973	32.71195	382	32.71195	470	32.71195
695	33.06	4.96138	32.73242	340	32.73242	436	32.73242
849	33.08	5.17146	32.75288	300	32.75288	377	32.75288
959	33.1	5.43646	32.77334	252	32.77334	314	32.77334
1006	33.12	5.64902	32.7938	209	32.7938	265	32.7938
975	33.14	6.06952	32.81427	178	32.81427	211	32.81427
865	33.16	6.59996	32.83473	146	32.83473	162	32.83473
699	33.18	7.277	32.85519	115	32.85519	128	32.85519
546	33.2	8.15596	32.87565	96	32.87565	102	32.87565
390	33.22	9.3247	32.89612	66	32.89612	80	32.89612
272	33.24	10.9359	32.91658	44	32.91658	59	32.91658
175	33.26	13.2824	32.93704	28	32.93704	46	32.93704
102	33.28	16.94732	32.95751	28	32.95751	34	32.95751
60	33.3	23.0046	32.97797	23	32.97797	24	32.97797
42	33.32	33.0896	32.99843	24	32.99843	18	32.99843
27	33.34	48.9322	33.01889	23	33.01889	19	33.01889
19	33.36	70.1822	33.03936	15	33.03936	18	33.03936
12	33.38	87.983	33.05982	13	33.05982	18	33.05982
6	33.4	85.6784	33.08028	16	33.08028	19	33.08028
0	33.42	66.2196	33.10075	13	33.10075	14	33.10075
2	33.44	46.3034	33.12121	9	33.12121	14	33.12121
13	33.46	32.306	33.14167	7	33.14167	8	33.14167
22	33.48	23.7748	33.16213	6	33.16213	8	33.16213
38	33.5	18.91606	33.1826	4	33.1826	7	33.1826
54	33.52	16.21032	33.20306	3	33.20306	4	33.20306
73	33.54	14.71294	33.22352	3	33.22352	5	33.22352
108	33.56	13.93072	33.24398	0	33.24398	3	33.24398
148	33.58	13.6242	33.26445	3	33.26445	2	33.26445
187	33.6	14.00088	33.28491	9	33.28491	8	33.28491
245	33.62	14.36038	33.30537	12	33.30537	11	33.30537
346	33.64	14.99442	33.32584	22	33.32584	9	33.32584
529	33.66	15.90146	33.3463	29	33.3463	7	33.3463
821	33.68	17.09942	33.36676	32	33.36676	17	33.36676
1210	33.7	18.62546	33.38722	34	33.38722	23	33.38722
1645	33.72	20.5384	33.40769	23	33.40769	34	33.40769
2027	33.74	22.9246	33.42815	27	33.42815	41	33.42815
2307	33.76	25.6426	33.44861	29	33.44861	47	33.44861
2446	33.78	29.4026	33.46908	40	33.46908	62	33.46908
2410	33.8	34.1854	33.48954	47	33.48954	88	33.48954
2216	33.82	40.3608	33.51	63	33.51	99	33.51
1907	33.84	48.4896	33.53046	85	33.53046	112	33.53046
1567	33.86	59.4694	33.55093	96	33.55093	120	33.55093
1257	33.88	74.8706	33.57139	103	33.57139	131	33.57139
1029	33.9	97.725	33.59185	114	33.59185	131	33.59185
867	33.92	134.0694	33.61231	107	33.61231	139	33.61231
742	33.94	194.95	33.63278	112	33.63278	140	33.63278
649	33.96	296.972	33.65324	106	33.65324	139	33.65324
600	33.98	457.112	33.6737	100	33.6737	141	33.6737

556	34	669.54	33.69417	93	33.69417	137	33.69417
504	34.02	839.096	33.71463	89	33.71463	124	33.71463
443	34.04	801.328	33.73509	79	33.73509	112	33.73509
367	34.06	599.778	33.75555	67	33.75555	95	33.75555
275	34.08	399.648	33.77602	64	33.77602	88	33.77602
210	34.1	259.21	33.79648	78	33.79648	93	33.79648
144	34.12	172.3234	33.81694	80	33.81694	118	33.81694
101	34.14	120.9042	33.83741	96	33.83741	143	33.83741
71	34.16	89.9824	33.85787	112	33.85787	169	33.85787
57	34.18	70.3412	33.87833	130	33.87833	198	33.87833
44	34.2	57.1438	33.89879	158	33.89879	217	33.89879
34	34.22	47.831	33.91926	179	33.91926	242	33.91926
31	34.24	40.7396	33.93972	211	33.93972	260	33.93972
28	34.26	34.5932	33.96018	253	33.96018	286	33.96018
20	34.28	29.4468	33.98064	310	33.98064	330	33.98064
11	34.3	25.35	34.00111	376	34.00111	398	34.00111
2	34.32	22.1316	34.02157	448	34.02157	483	34.02157
4	34.34	19.58326	34.04203	553	34.04203	600	34.04203
10	34.36	17.53404	34.0625	701	34.0625	720	34.0625
11	34.38	15.86006	34.08296	860	34.08296	902	34.08296
10	34.4	14.47576	34.10342	1078	34.10342	1098	34.10342
6	34.42	13.32188	34.12388	1325	34.12388	1368	34.12388
6	34.44	12.3559	34.14435	1587	34.14435	1646	34.14435
7	34.46	11.54622	34.16481	1835	34.16481	1909	34.16481
5	34.48	10.86894	34.18527	2033	34.18527	2126	34.18527
3	34.5	10.3058	34.20574	2151	34.20574	2280	34.20574
6	34.52	9.84288	34.2262	2182	34.2262	2340	34.2262
6	34.54	9.4697	34.24666	2130	34.24666	2319	34.24666
5	34.56	9.17866	34.26712	1991	34.26712	2179	34.26712
1	34.58	8.9646	34.28759	1784	34.28759	2003	34.28759
0	34.6	8.82462	34.30805	1558	34.30805	1769	34.30805
2	34.62	8.75784	34.32851	1322	34.32851	1544	34.32851
6	34.64	8.7656	34.34897	1094	34.34897	1295	34.34897
11	34.66	8.85154	34.36944	886	34.36944	1053	34.36944
11	34.68	9.02192	34.3899	708	34.3899	829	34.3899
15	34.7	9.28616	34.41036	566	34.41036	649	34.41036
33	34.72	9.65764	34.43083	450	34.43083	492	34.43083
65	34.74	10.15494	34.45129	353	34.45129	383	34.45129
98	34.76	10.80354	34.47175	273	34.47175	299	34.47175
139	34.78	11.63842	34.49221	223	34.49221	258	34.49221
174	34.8	12.70812	34.51268	173	34.51268	231	34.51268
207	34.82	14.08062	34.53314	144	34.53314	202	34.53314
220	34.84	15.8531	34.5536	117	34.5536	184	34.5536
222	34.86	18.16742	34.57407	92	34.57407	158	34.57407
203	34.88	21.1586	34.59453	74	34.59453	132	34.59453
173	34.9	25.3256	34.61499	60	34.61499	117	34.61499
146	34.92	31.1752	34.63545	51	34.63545	102	34.63545
114	34.94	39.8314	34.65592	43	34.65592	90	34.65592
81	34.96	53.5366	34.67638	38	34.67638	84	34.67638
56	34.98	76.4552	34.69684	37	34.69684	81	34.69684
31	35	115.016	34.7173	32	34.7173	75	34.7173
16	35.02	176.2384	34.73777	30	34.73777	67	34.73777
24	35.04	259.67	34.75823	21	34.75823	56	34.75823
39	35.06	332.578	34.77869	16	34.77869	46	34.77869
55	35.08	327.69	34.79916	9	34.79916	39	34.79916
63	35.1	250.64	34.81962	14	34.81962	30	34.81962
79	35.12	168.7452	34.84008	21	34.84008	42	34.84008
92	35.14	110.0002	34.86054	25	34.86054	52	34.86054

104	35.16	73.3192	34.88101	29	34.88101	69	34.88101
121	35.18	51.5488	34.90147	37	34.90147	73	34.90147
143	35.2	38.4656	34.92193	48	34.92193	87	34.92193
164	35.22	30.1336	34.9424	61	34.9424	88	34.9424
214	35.24	24.508	34.96286	70	34.96286	91	34.96286
263	35.26	20.4478	34.98332	91	34.98332	105	34.98332
323	35.28	17.44838	35.00378	106	35.00378	114	35.00378
426	35.3	15.1892	35.02425	127	35.02425	121	35.02425
596	35.32	13.46892	35.04471	148	35.04471	152	35.04471
893	35.34	12.15556	35.06517	177	35.06517	187	35.06517
1363	35.36	11.16148	35.08564	219	35.08564	233	35.08564
2026	35.38	10.42902	35.1061	283	35.1061	286	35.1061
2758	35.4	9.92246	35.12656	377	35.12656	367	35.12656
3417	35.42	9.62356	35.14702	480	35.14702	457	35.14702
3916	35.44	9.52988	35.16748	616	35.16748	578	35.16748
4163	35.46	9.65592	35.18795	763	35.18795	719	35.18795
4156	35.48	10.03714	35.20841	886	35.20841	863	35.20841
3906	35.5	9.92902	35.22887	995	35.22887	1001	35.22887
3406	35.52	11.09146	35.24934	1056	35.24934	1110	35.24934
2801	35.54	12.9129	35.2698	1063	35.2698	1171	35.2698
2206	35.56	15.83684	35.29026	1013	35.29026	1179	35.29026
1693	35.58	20.7438	35.31073	940	35.31073	1127	35.31073
1257	35.6	29.1834	35.33119	831	35.33119	1036	35.33119
874	35.62	43.322	35.35165	696	35.35165	906	35.35165
575	35.64	64.9492	35.37211	581	35.37211	762	35.37211
378	35.66	91.3998	35.39258	464	35.39258	623	35.39258
276	35.68	106.9088	35.41304	356	35.41304	501	35.41304
239	35.7	94.7694	35.4335	280	35.4335	394	35.4335
197	35.72	68.3188	35.45396	210	35.45396	302	35.45396
168	35.74	45.3494	35.47443	154	35.47443	230	35.47443
141	35.76	29.92	35.49489	112	35.49489	179	35.49489
116	35.78	20.5134	35.51535	101	35.51535	136	35.51535
100	35.8	15.23626	35.53582	92	35.53582	104	35.53582
75	35.82	11.79282	35.55628	83	35.55628	82	35.55628
63	35.84	9.52258	35.57674	75	35.57674	71	35.57674
53	35.86	7.92632	35.59721	62	35.59721	65	35.59721
40	35.88	6.7517	35.61767	58	35.61767	64	35.61767
33	35.9	5.86044	35.63813	58	35.63813	56	35.63813
23	35.92	5.16874	35.65859	61	35.65859	60	35.65859
14	35.94	4.62212	35.67905	62	35.67905	70	35.67905
7	35.96	4.18368	35.69952	68	35.69952	75	35.69952
12	35.98	3.8277	35.71998	90	35.71998	87	35.71998
12	36	3.93346	35.74044	114	35.74044	92	35.74044
11	36.02	3.70352	35.76091	137	35.76091	122	35.76091
18	36.04	3.51506	35.78137	155	35.78137	153	35.78137
17	36.06	3.36076	35.80183	178	35.80183	187	35.80183
21	36.08	3.23506	35.8223	209	35.8223	227	35.8223
22	36.1	3.13378	35.84276	231	35.84276	267	35.84276
18	36.12	3.0537	35.86322	253	35.86322	304	35.86322
11	36.14	2.9924	35.88368	264	35.88368	322	35.88368
8	36.16	2.94806	35.90414	266	35.90414	316	35.90414
20	36.18	2.9194	35.92461	259	35.92461	301	35.92461
26	36.2	2.90554	35.94507	229	35.94507	264	35.94507
42	36.22	2.90596	35.96553	199	35.96553	222	35.96553
72	36.24	2.9206	35.986	165	35.986	181	35.986
94	36.26	2.94964	36.00646	134	36.00646	142	36.00646
126	36.28	2.99376	36.02692	98	36.02692	111	36.02692
152	36.3	3.05398	36.04739	74	36.04739	79	36.04739

192	36.32	3.1319	36.06785	57	36.06785	55	36.06785
266	36.34	3.22972	36.08831	43	36.08831	34	36.08831
381	36.36	3.35052	36.10877	30	36.10877	20	36.10877
576	36.38	3.49848	36.12924	18	36.12924	11	36.12924
848	36.4	3.67928	36.1497	11	36.1497	7	36.1497
1164	36.42	3.90082	36.17016	14	36.17016	0	36.17016
1454	36.44	4.17414	36.19062	14	36.19062	6	36.19062
1667	36.46	4.515	36.21109	17	36.21109	4	36.21109
1780	36.48	4.94654	36.23155	14	36.23155	6	36.23155
1783	36.5	5.5039	36.25201	19	36.25201	3	36.25201
1674	36.52	6.24394	36.27248	15	36.27248	5	36.27248
1478	36.54	7.26808	36.29294	13	36.29294	5	36.29294
1225	36.56	8.4586	36.3134	8	36.3134	6	36.3134
969	36.58	10.84634	36.33387	5	36.33387	5	36.33387
748	36.6	14.79828	36.35433	8	36.35433	9	36.35433
553	36.62	21.2956	36.37479	5	36.37479	11	36.37479
380	36.64	31.1938	36.39525	0	36.39525	11	36.39525
247	36.66	43.4862	36.41571	4	36.41571	9	36.41571
157	36.68	51.3526	36.43618	6	36.43618	9	36.43618
107	36.7	46.7218	36.45664	5	36.45664	6	36.45664
76	36.72	35.0146	36.4771	2	36.4771	4	36.4771
67	36.74	24.6524	36.49757	9	36.49757	3	36.49757
56	36.76	17.75186	36.51803	12	36.51803	0	36.51803
41	36.78	13.8389	36.53849	9	36.53849	2	36.53849
39	36.8	11.59568	36.55896	11	36.55896	7	36.55896
38	36.82	10.40446	36.57942	16	36.57942	10	36.57942
33	36.84	9.81378	36.59988	21	36.59988	12	36.59988
36	36.86	9.59632	36.62034	27	36.62034	9	36.62034
42	36.88	9.64416	36.6408	28	36.6408	7	36.6408
48	36.9	9.90642	36.66127	23	36.66127	7	36.66127
68	36.92	10.36174	36.68173	26	36.68173	16	36.68173
95	36.94	11.00752	36.70219	34	36.70219	19	36.70219
140	36.96	11.8562	36.72266	36	36.72266	23	36.72266
215	36.98	12.93486	36.74312	36	36.74312	43	36.74312
325	37	14.28732	36.76358	44	36.76358	59	36.76358
444	37.02	15.97894	36.78405	59	36.78405	76	36.78405
543	37.04	18.10506	36.80451	83	36.80451	98	36.80451
614	37.06	20.8052	36.82497	93	36.82497	105	36.82497
654	37.08	24.2884	36.84543	99	36.84543	116	36.84543
651	37.1	28.88	36.8659	105	36.8659	129	36.8659
609	37.12	35.1304	36.88636	117	36.88636	138	36.88636
526	37.14	43.9642	36.90682	113	36.90682	131	36.90682
433	37.16	57.6214	36.92728	109	36.92728	130	36.92728
338	37.18	79.826	36.94775	101	36.94775	128	36.94775
262	37.2	116.6954	36.96821	91	36.96821	124	36.96821
191	37.22	175.4528	36.98867	87	36.98867	116	36.98867
126	37.24	258.018	37.00914	88	37.00914	114	37.00914
78	37.26	339.238	37.0296	74	37.0296	100	37.0296
56	37.28	353.186	37.05006	71	37.05006	94	37.05006
38	37.3	288.772	37.07053	79	37.07053	96	37.07053
29	37.32	212.838	37.09099	80	37.09099	90	37.09099
24	37.34	162.0354	37.11145	77	37.11145	89	37.11145
16	37.36	140.41	37.13191	85	37.13191	104	37.13191
13	37.38	146.607	37.15237	88	37.15237	115	37.15237
12	37.4	182.2288	37.17284	95	37.17284	129	37.17284
12	37.42	251.31	37.1933	103	37.1933	151	37.1933
14	37.44	348.662	37.21376	115	37.21376	172	37.21376
16	37.46	426.884	37.23423	139	37.23423	197	37.23423

17	37.48	405.37	37.25469	179	37.25469	227	37.25469
15	37.5	304.912	37.27515	231	37.27515	261	37.27515
11	37.52	205.658	37.29562	294	37.29562	303	37.29562
10	37.54	135.8934	37.31608	371	37.31608	375	37.31608
6	37.56	92.3926	37.33654	462	37.33654	477	37.33654
9	37.58	66.3454	37.357	573	37.357	590	37.357
10	37.6	50.3978	37.37746	703	37.37746	716	37.37746
13	37.62	40.0018	37.39793	843	37.39793	880	37.39793
13	37.64	32.7596	37.41839	985	37.41839	1034	37.41839
12	37.66	27.4626	37.43885	1104	37.43885	1194	37.43885
6	37.68	23.4622	37.45932	1192	37.45932	1302	37.45932
7	37.7	20.3722	37.47978	1240	37.47978	1389	37.47978
3	37.72	17.94532	37.50024	1256	37.50024	1451	37.50024
9	37.74	16.01436	37.52071	1255	37.52071	1489	37.52071
8	37.76	14.46394	37.54117	1252	37.54117	1514	37.54117
12	37.78	13.21248	37.56163	1235	37.56163	1504	37.56163
24	37.8	12.20166	37.58209	1215	37.58209	1506	37.58209
31	37.82	11.38948	37.60256	1202	37.60256	1532	37.60256
38	37.84	10.74586	37.62302	1192	37.62302	1547	37.62302
43	37.86	10.24988	37.64348	1172	37.64348	1551	37.64348
44	37.88	9.88794	37.66394	1123	37.66394	1519	37.66394
46	37.9	9.65276	37.68441	1047	37.68441	1480	37.68441
53	37.92	9.54312	37.70487	962	37.70487	1405	37.70487
64	37.94	9.56412	37.72533	881	37.72533	1304	37.72533
86	37.96	9.72816	37.7458	799	37.7458	1180	37.7458
135	37.98	10.05702	37.76626	683	37.76626	1034	37.76626
193	38	10.58514	37.78672	574	37.78672	896	37.78672
248	38.02	11.36554	37.80719	477	37.80719	749	37.80719
290	38.04	12.47956	37.82765	396	37.82765	622	37.82765
311	38.06	14.05564	37.84811	331	37.84811	505	37.84811
314	38.08	16.3095	37.86857	260	37.86857	405	37.86857
289	38.1	19.64022	37.88903	206	37.88903	333	37.88903
258	38.12	24.842	37.9095	173	37.9095	270	37.9095
206	38.14	33.4584	37.92996	149	37.92996	231	37.92996
165	38.16	48.0532	37.95042	137	37.95042	197	37.95042
131	38.18	72.1268	37.97089	115	37.97089	180	37.97089
109	38.2	107.748	37.99135	106	37.99135	159	37.99135
84	38.22	147.7514	38.01181	94	38.01181	132	38.01181
64	38.24	163.789	38.03228	86	38.03228	121	38.03228
53	38.26	137.8322	38.05274	77	38.05274	100	38.05274
55	38.28	97.1018	38.0732	66	38.0732	82	38.0732
58	38.3	64.4224	38.09366	58	38.09366	81	38.09366
67	38.32	43.1322	38.11412	62	38.11412	73	38.11412
73	38.34	30.3548	38.13459	63	38.13459	72	38.13459
86	38.36	22.8028	38.15505	67	38.15505	77	38.15505
105	38.38	18.1637	38.17551	84	38.17551	89	38.17551
128	38.4	15.1341	38.19598	99	38.19598	102	38.19598
140	38.42	13.05682	38.21644	111	38.21644	113	38.21644
175	38.44	11.59942	38.2369	131	38.2369	132	38.2369
212	38.46	10.57978	38.25737	158	38.25737	146	38.25737
277	38.48	9.8905	38.27783	179	38.27783	172	38.27783
392	38.5	9.4672	38.29829	207	38.29829	203	38.29829
574	38.52	9.27384	38.31875	252	38.31875	232	38.31875
877	38.54	9.29598	38.33922	305	38.33922	272	38.33922
1264	38.56	9.53794	38.35968	362	38.35968	318	38.35968
1670	38.58	10.0239	38.38014	428	38.38014	377	38.38014
2014	38.6	10.80242	38.4006	467	38.4006	434	38.4006
2247	38.62	11.95724	38.42107	503	38.42107	481	38.42107

2365	38.64	13.63036	38.44153	526	38.44153	513	38.44153
2356	38.66	16.07688	38.46199	530	38.46199	529	38.46199
2255	38.68	19.79428	38.48246	510	38.48246	526	38.48246
2115	38.7	25.7754	38.50292	476	38.50292	500	38.50292
2015	38.72	35.8358	38.52338	437	38.52338	449	38.52338
2061	38.74	52.7002	38.54385	393	38.54385	387	38.54385
2207	38.76	78.8402	38.56431	331	38.56431	329	38.56431
2397	38.78	113.8872	38.58477	274	38.58477	287	38.58477
2537	38.8	141.8844	38.60523	218	38.60523	243	38.60523
2595	38.82	136.1494	38.62569	178	38.62569	196	38.62569
2562	38.84	104.2004	38.64616	141	38.64616	157	38.64616
2458	38.86	73.125	38.66662	110	38.66662	133	38.66662
2246	38.88	52.6704	38.68708	96	38.68708	115	38.68708
1962	38.9	42.5176	38.70755	76	38.70755	100	38.70755
1634	38.92	40.902	38.72801	73	38.72801	79	38.72801
1329	38.94	46.4746	38.74847	74	38.74847	71	38.74847
1059	38.96	59.2242	38.76894	79	38.76894	77	38.76894
824	38.98	73.909	38.7894	94	38.7894	84	38.7894
586	39	77.0054	38.80986	113	38.80986	96	38.80986
397	39.02	62.9112	38.83032	132	38.83032	116	38.83032
261	39.04	44.3742	38.85078	160	38.85078	137	38.85078
187	39.06	30.0364	38.87125	186	38.87125	172	38.87125
135	39.08	20.6984	38.89171	220	38.89171	219	38.89171
115	39.1	14.97732	38.91217	257	38.91217	262	38.91217
88	39.12	11.45536	38.93264	314	38.93264	316	38.93264
72	39.14	9.16632	38.9531	371	38.9531	366	38.9531
66	39.16	7.57182	38.97356	429	38.97356	412	38.97356
62	39.18	6.39728	38.99403	467	38.99403	453	38.99403
55	39.2	5.49864	39.01449	486	39.01449	493	39.01449
61	39.22	4.7926	39.03495	488	39.03495	509	39.03495
58	39.24	4.22632	39.05541	484	39.05541	515	39.05541
52	39.26	3.7643	39.07588	477	39.07588	518	39.07588
44	39.28	3.3818	39.09634	456	39.09634	521	39.09634
44	39.3	3.06114	39.1168	453	39.1168	514	39.1168
41	39.32	2.78938	39.13726	446	39.13726	500	39.13726
39	39.34	2.55688	39.15773	432	39.15773	489	39.15773
46	39.36	2.35632	39.17819	411	39.17819	481	39.17819
61	39.38	2.18204	39.19865	382	39.19865	463	39.19865
88	39.4	2.02966	39.21912	336	39.21912	438	39.21912
115	39.42	1.895678	39.23958	302	39.23958	388	39.23958
147	39.44	1.777318	39.26004	257	39.26004	339	39.26004
206	39.46	1.67233	39.28051	229	39.28051	284	39.28051
307	39.48	1.578882	39.30097	186	39.30097	242	39.30097
451	39.5	1.495472	39.32143	163	39.32143	195	39.32143
627	39.52	1.420858	39.34189	135	39.34189	154	39.34189
800	39.54	1.354012	39.36235	112	39.36235	142	39.36235
945	39.56	1.294078	39.38282	89	39.38282	130	39.38282
1041	39.58	1.240338	39.40328	71	39.40328	117	39.40328
1085	39.6	1.192194	39.42374	54	39.42374	101	39.42374
1047	39.62	1.149148	39.44421	49	39.44421	80	39.44421
955	39.64	1.110786	39.46467	35	39.46467	55	39.46467
825	39.66	1.076766	39.48513	32	39.48513	32	39.48513
685	39.68	1.046812	39.5056	22	39.5056	25	39.5056
558	39.7	0.865592	39.52606	22	39.52606	6	39.52606
444	39.72	0.847328	39.54652	14	39.54652	5	39.54652
334	39.74	0.852472	39.56698	16	39.56698	13	39.56698
242	39.76	0.84154	39.58744	15	39.58744	19	39.58744
165	39.78	0.83399	39.60791	14	39.60791	20	39.60791

109	39.8	0.829874	39.62837	13	39.62837	24	39.62837
75	39.82	0.829306	39.64883	9	39.64883	25	39.64883
58	39.84	0.83246	39.6693	8	39.6693	24	39.6693
47	39.86	0.83959	39.68976	11	39.68976	19	39.68976
43	39.88	0.851036	39.71022	12	39.71022	18	39.71022
45	39.9	0.867248	39.73069	17	39.73069	11	39.73069
54	39.92	0.888812	39.75115	21	39.75115	6	39.75115
71	39.94	0.91649	39.77161	29	39.77161	6	39.77161
87	39.96	0.951266	39.79207	27	39.79207	8	39.79207
103	39.98	0.99443	39.81254	29	39.81254	7	39.81254
130	40	1.04767	39.833	26	39.833	17	39.833
188	40.02	1.113242	39.85346	19	39.85346	21	39.85346
281	40.04	1.194192	39.87392	13	39.87392	26	39.87392
407	40.06	1.294722	39.89439	9	39.89439	25	39.89439
554	40.08	1.420752	39.91485	7	39.91485	22	39.91485
677	40.1	1.580892	39.93531	9	39.93531	15	39.93531
790	40.12	1.788246	39.95578	11	39.95578	6	39.95578
853	40.14	2.06442	39.97624	10	39.97624	3	39.97624
876	40.16	2.4488	39.9967	6	39.9967	0	39.9967
848	40.18	3.01792	40.01717	7	40.01717	0	40.01717
804	40.2	3.91408	40.03763	8	40.03763	0	40.03763
763	40.22	5.36286	40.05809	8	40.05809	0	40.05809
735	40.24	7.63002	40.07855	8	40.07855	4	40.07855
726	40.26	10.78814	40.09901	8	40.09901	8	40.09901
718	40.28	13.89246	40.11948	11	40.11948	11	40.11948
679	40.3	14.68956	40.13994	15	40.13994	12	40.13994
630	40.32	12.85412	40.1604	17	40.1604	9	40.1604
577	40.34	10.87378	40.18087	17	40.18087	6	40.18087
526	40.36	10.20812	40.20133	12	40.20133	11	40.20133
459	40.38	11.3488	40.22179	10	40.22179	13	40.22179
399	40.4	14.79172	40.24226	10	40.24226	15	40.24226
344	40.42	21.2208	40.26272	9	40.26272	13	40.26272
282	40.44	30.868	40.28318	8	40.28318	18	40.28318
238	40.46	40.9192	40.30364	4	40.30364	15	40.30364
185	40.48	43.2864	40.3241	0	40.3241	12	40.3241
124	40.5	34.999	40.34457	6	40.34457	7	40.34457
75	40.52	24.1564	40.36503	11	40.36503	0	40.36503
46	40.54	15.865	40.38549	11	40.38549	0	40.38549
32	40.56	10.54918	40.40596	11	40.40596	13	40.40596
24	40.58	7.3651	40.42642	11	40.42642	23	40.42642
14	40.6	5.46246	40.44688	19	40.44688	31	40.44688
10	40.62	4.26794	40.46735	22	40.46735	35	40.46735
8	40.64	3.46562	40.48781	34	40.48781	43	40.48781
14	40.66	2.89634	40.50827	39	40.50827	49	40.50827
18	40.68	2.47778	40.52873	49	40.52873	54	40.52873
20	40.7	2.1631	40.5492	71	40.5492	63	40.5492
21	40.72	1.92311	40.56966	85	40.56966	67	40.56966
18	40.74	1.738592	40.59012	98	40.59012	70	40.59012
27	40.76	1.596528	40.61058	103	40.61058	90	40.61058
25	40.78	1.48794	40.63105	102	40.63105	113	40.63105
22	40.8	1.406604	40.65151	109	40.65151	120	40.65151
17	40.82	1.348254	40.67197	98	40.67197	121	40.67197
15	40.84	1.310078	40.69244	98	40.69244	122	40.69244
14	40.86	1.290424	40.7129	83	40.7129	117	40.7129
14	40.88	1.288654	40.73336	73	40.73336	101	40.73336
12	40.9	1.305104	40.75383	66	40.75383	102	40.75383
6	40.92	1.341168	40.77429	59	40.77429	91	40.77429
0	40.94	1.399522	40.79475	59	40.79475	74	40.79475

4	40.96	1.484542	40.81521	56	40.81521	70	40.81521
3	40.98	1.60303	40.83567	52	40.83567	61	40.83567
5	41	1.76547	40.85614	48	40.85614	49	40.85614
6	41.02	1.98842	40.8766	36	40.8766	33	40.8766
6	41.04	2.29968	40.89706	28	40.89706	28	40.89706
9	41.06	2.75066	40.91753	17	40.91753	21	40.91753
11	41.08	3.44348	40.93799	10	40.93799	11	40.93799
12	41.1	4.5765	40.95845	6	40.95845	22	40.95845
10	41.12	6.48454	40.97892	4	40.97892	21	40.97892
7	41.14	9.60424	40.99938	8	40.99938	22	40.99938
10	41.16	14.44158	41.01984	10	41.01984	24	41.01984
8	41.18	19.6022	41.0403	12	41.0403	19	41.0403
4	41.2	21.6794	41.06076	12	41.06076	20	41.06076
0	41.22	18.39282	41.08123	10	41.08123	14	41.08123
1	41.24	13.24218	41.10169	3	41.10169	13	41.10169
4	41.26	9.13518	41.12215	5	41.12215	11	41.12215
8	41.28	6.49276	41.14262	6	41.14262	6	41.14262
11	41.3	4.94704	41.16308	8	41.16308	4	41.16308
12	41.32	4.07966	41.18354	6	41.18354	3	41.18354
11	41.34	3.59772	41.20401	7	41.20401	6	41.20401
10	41.36	3.3379	41.22447	5	41.22447	8	41.22447
7	41.38	3.2202	41.24493	4	41.24493	6	41.24493
4	41.4	3.20716	41.26539	7	41.26539	4	41.26539
0	41.42	3.28204	41.28586	9	41.28586	8	41.28586
1	41.44	3.43984	41.30632	16	41.30632	13	41.30632
4	41.46	3.68426	41.32678	27	41.32678	16	41.32678
12	41.48	4.02716	41.34724	27	41.34724	22	41.34724
20	41.5	4.49004	41.36771	27	41.36771	28	41.36771
39	41.52	5.10748	41.38817	28	41.38817	29	41.38817
45	41.54	5.93348	41.40863	33	41.40863	26	41.40863
56	41.56	7.05464	41.4291	27	41.4291	26	41.4291
63	41.58	8.61952	41.44956	23	41.44956	19	41.44956
63	41.6	10.90894	41.47002	23	41.47002	23	41.47002
62	41.62	14.48416	41.49049	21	41.49049	28	41.49049
75	41.64	20.412	41.51095	19	41.51095	25	41.51095
97	41.66	30.4126	41.53141	16	41.53141	22	41.53141
126	41.68	47.1036	41.55187	10	41.55187	26	41.55187
160	41.7	70.0088	41.57233	3	41.57233	29	41.57233
202	41.72	92.7972	41.5928	9	41.5928	25	41.5928
228	41.74	96.5762	41.61326	16	41.61326	29	41.61326
244	41.76	77.023	41.63372	17	41.63372	34	41.63372
247	41.78	52.9562	41.65419	19	41.65419	32	41.65419
233	41.8	34.9278	41.67465	26	41.67465	38	41.67465
206	41.82	23.49	41.69511	31	41.69511	36	41.69511
187	41.84	16.91762	41.71558	38	41.71558	35	41.71558
158	41.86	12.88866	41.73604	41	41.73604	41	41.73604
126	41.88	10.3797	41.7565	43	41.7565	54	41.7565
99	41.9	8.71374	41.77696	45	41.77696	67	41.77696
74	41.92	7.55124	41.79742	64	41.79742	76	41.79742
55	41.94	6.675	41.81789	96	41.81789	97	41.81789
34	41.96	6.06808	41.83835	120	41.83835	118	41.83835
25	41.98	5.62544	41.85881	145	41.85881	145	41.85881
16	42	5.30596	41.87928	174	41.87928	170	41.87928
9	42.02	5.08186	41.89974	193	41.89974	197	41.89974
14	42.04	4.93412	41.9202	211	41.9202	228	41.9202
8	42.06	4.84966	41.94067	215	41.94067	247	41.94067
3	42.08	4.81956	41.96113	207	41.96113	262	41.96113
5	42.1	4.83796	41.98159	198	41.98159	254	41.98159

0	42.12	4.90138	42.00205	187	42.00205	233	42.00205
1	42.14	5.00824	42.02252	173	42.02252	203	42.02252
6	42.16	5.15864	42.04298	143	42.04298	178	42.04298
13	42.18	5.35434	42.06344	121	42.06344	160	42.06344
16	42.2	5.59868	42.0839	93	42.0839	145	42.0839
17	42.22	5.89688	42.10437	65	42.10437	140	42.10437
13	42.24	6.25628	42.12483	49	42.12483	127	42.12483
11	42.26	6.68694	42.14529	36	42.14529	111	42.14529
11	42.28	7.20238	42.16576	22	42.16576	101	42.16576
8	42.3	7.82076	42.18622	18	42.18622	81	42.18622
7	42.32	8.5666	42.20668	15	42.20668	59	42.20668
9	42.34	9.4734	42.22715	14	42.22715	38	42.22715
16	42.36	10.58758	42.24761	15	42.24761	32	42.24761
21	42.38	11.97472	42.26807	21	42.26807	30	42.26807
25	42.4	13.72962	42.28853	23	42.28853	28	42.28853
35	42.42	15.99356	42.30899	22	42.30899	23	42.30899
46	42.44	18.98722	42.32946	21	42.32946	19	42.32946
56	42.46	23.084	42.34992	16	42.34992	23	42.34992
63	42.48	28.984	42.37038	19	42.37038	25	42.37038
62	42.5	38.0824	42.39085	18	42.39085	30	42.39085
61	42.52	53.1376	42.41131	16	42.41131	31	42.41131
51	42.54	78.228	42.43177	19	42.43177	48	42.43177
42	42.56	118.6132	42.45224	17	42.45224	58	42.45224
33	42.58	175.9968	42.4727	20	42.4727	60	42.4727
24	42.6	233.6	42.49316	26	42.49316	57	42.49316
29	42.62	244.304	42.51362	43	42.51362	51	42.51362
33	42.64	196.206	42.53408	50	42.53408	61	42.53408
30	42.66	136.2208	42.55455	60	42.55455	65	42.55455
31	42.68	91.2536	42.57501	77	42.57501	72	42.57501
25	42.7	62.898	42.59547	90	42.59547	101	42.59547
21	42.72	46.308	42.61594	103	42.61594	128	42.61594
14	42.74	36.78	42.6364	123	42.6364	165	42.6364
14	42.76	31.2002	42.65686	146	42.65686	201	42.65686
19	42.78	27.8836	42.67733	176	42.67733	248	42.67733
19	42.8	26.0052	42.69779	221	42.69779	301	42.69779
37	42.82	25.1648	42.71825	286	42.71825	358	42.71825
54	42.84	25.1674	42.73871	348	42.73871	439	42.73871
75	42.86	25.9344	42.75918	418	42.75918	528	42.75918
118	42.88	27.4716	42.77964	479	42.77964	618	42.77964
182	42.9	29.8632	42.8001	533	42.8001	699	42.8001
270	42.92	33.2834	42.82056	583	42.82056	760	42.82056
356	42.94	38.0294	42.84103	601	42.84103	798	42.84103
446	42.96	44.5922	42.86149	601	42.86149	810	42.86149
506	42.98	53.8194	42.88195	585	42.88195	794	42.88195
534	43	67.3084	42.90242	552	42.90242	749	42.90242
534	43.02	88.2474	42.92288	509	42.92288	708	42.92288
504	43.04	122.7364	42.94334	458	42.94334	652	42.94334
448	43.06	180.8994	42.96381	412	42.96381	598	42.96381
388	43.08	274.778	42.98427	362	42.98427	526	42.98427
333	43.1	409.74	43.00473	328	43.00473	472	43.00473
283	43.12	550.256	43.02519	310	43.02519	434	43.02519
221	43.14	586.92	43.04565	290	43.04565	398	43.04565
177	43.16	481.46	43.06612	296	43.06612	379	43.06612
129	43.18	343.388	43.08658	317	43.08658	378	43.08658
96	43.2	242.974	43.10704	346	43.10704	386	43.10704
62	43.22	188.9106	43.12751	397	43.12751	442	43.12751
42	43.24	174.7156	43.14797	454	43.14797	487	43.14797
31	43.26	193.6166	43.16843	523	43.16843	549	43.16843

25	43.28	236.296	43.1889	600	43.1889	623	43.1889
24	43.3	272.286	43.20936	716	43.20936	749	43.20936
23	43.32	254.19	43.22982	849	43.22982	898	43.22982
14	43.34	193.0094	43.25028	1001	43.25028	1064	43.25028
15	43.36	133.6046	43.27074	1171	43.27074	1273	43.27074
13	43.38	91.5948	43.29121	1339	43.29121	1486	43.29121
9	43.4	65.0166	43.31167	1500	43.31167	1656	43.31167
0	43.42	48.7062	43.33213	1640	43.33213	1822	43.33213
5	43.44	38.4068	43.3526	1723	43.3526	1905	43.3526
17	43.46	31.4782	43.37306	1762	43.37306	1948	43.37306
23	43.48	26.515	43.39352	1758	43.39352	1959	43.39352
38	43.5	22.7974	43.41399	1742	43.41399	1930	43.41399
41	43.52	19.93156	43.43445	1679	43.43445	1869	43.43445
53	43.54	17.67924	43.45491	1597	43.45491	1799	43.45491
56	43.56	15.88494	43.47537	1510	43.47537	1721	43.47537
56	43.58	14.44304	43.49584	1419	43.49584	1631	43.49584
55	43.6	13.28008	43.5163	1320	43.5163	1520	43.5163
56	43.62	12.34444	43.53676	1214	43.53676	1408	43.53676
73	43.64	11.60006	43.55722	1111	43.55722	1263	43.55722
78	43.66	11.02244	43.57769	1009	43.57769	1150	43.57769
96	43.68	10.59648	43.59815	906	43.59815	1040	43.59815
134	43.7	10.31522	43.61861	811	43.61861	927	43.61861
185	43.72	10.17978	43.63908	707	43.63908	817	43.63908
266	43.74	10.20026	43.65954	608	43.65954	719	43.65954
391	43.76	10.398	43.68	518	43.68	608	43.68
596	43.78	10.8098	43.70047	442	43.70047	517	43.70047
843	43.8	11.49578	43.72093	361	43.72093	442	43.72093
1083	43.82	12.55494	43.74139	292	43.74139	360	43.74139
1312	43.84	14.1604	43.76185	247	43.76185	293	43.76185
1459	43.86	16.64346	43.78231	207	43.78231	251	43.78231
1522	43.88	20.724	43.80278	182	43.80278	216	43.80278
1519	43.9	27.5526	43.82324	162	43.82324	203	43.82324
1433	43.92	39.1916	43.8437	151	43.8437	189	43.8437
1291	43.94	57.9926	43.86417	132	43.86417	178	43.86417
1130	43.96	84.4782	43.88463	126	43.88463	158	43.88463
989	43.98	110.0494	43.90509	126	43.90509	148	43.90509
830	44	112.7836	43.92556	110	43.92556	140	43.92556
692	44.02	89.4592	43.94602	103	43.94602	128	43.94602
578	44.04	61.9674	43.96648	109	43.96648	124	43.96648
454	44.06	41.6232	43.98694	107	43.98694	119	43.98694
354	44.08	28.7876	44.0074	115	44.0074	118	44.0074
319	44.1	20.9626	44.02787	126	44.02787	127	44.02787
294	44.12	16.48306	44.04833	152	44.04833	136	44.04833
291	44.14	13.73038	44.06879	172	44.06879	145	44.06879
307	44.16	11.95854	44.08926	206	44.08926	167	44.08926
343	44.18	10.79924	44.10972	234	44.10972	199	44.10972
399	44.2	10.06822	44.13018	251	44.13018	236	44.13018
521	44.22	9.66928	44.15065	288	44.15065	268	44.15065
697	44.24	9.55478	44.17111	318	44.17111	288	44.17111
987	44.26	9.7106	44.19157	326	44.19157	305	44.19157
1435	44.28	10.15204	44.21203	327	44.21203	308	44.21203
1984	44.3	10.92666	44.2325	324	44.2325	310	44.2325
2532	44.32	12.12604	44.25296	314	44.25296	303	44.25296
2963	44.34	13.91528	44.27342	297	44.27342	288	44.27342
3237	44.36	16.60604	44.29388	277	44.29388	272	44.29388
3326	44.38	20.8276	44.31435	246	44.31435	250	44.31435
3260	44.4	27.8032	44.33481	215	44.33481	233	44.33481
3117	44.42	39.6744	44.35527	195	44.35527	193	44.35527

2912	44.44	59.3606	44.37574	171	44.37574	163	44.37574
2744	44.46	89.0786	44.3962	144	44.3962	144	44.3962
2644	44.48	124.4028	44.41666	117	44.41666	120	44.41666
2566	44.5	142.9932	44.43713	97	44.43713	103	44.43713
2464	44.52	124.8022	44.45759	87	44.45759	96	44.45759
2284	44.54	90.0344	44.47805	81	44.47805	89	44.47805
2024	44.56	61.159	44.49851	88	44.49851	92	44.49851
1758	44.58	42.852	44.51897	96	44.51897	95	44.51897
1522	44.6	32.7464	44.53944	121	44.53944	114	44.53944
1320	44.62	29.4762	44.5599	148	44.5599	133	44.5599
1108	44.64	30.2776	44.58036	191	44.58036	182	44.58036
929	44.66	31.8026	44.60083	235	44.60083	227	44.60083
759	44.68	29.2836	44.62129	282	44.62129	276	44.62129
612	44.7	22.9614	44.64175	333	44.64175	323	44.64175
478	44.72	16.80344	44.66222	378	44.66222	379	44.66222
359	44.74	12.34614	44.68268	398	44.68268	417	44.68268
236	44.76	9.42644	44.70314	424	44.70314	448	44.70314
175	44.78	7.33006	44.7236	426	44.7236	461	44.7236
156	44.8	6.09584	44.74406	419	44.74406	454	44.74406
131	44.82	5.232	44.76453	395	44.76453	429	44.76453
105	44.84	4.59508	44.78499	369	44.78499	402	44.78499
90	44.86	4.10938	44.80545	343	44.80545	367	44.80545
69	44.88	3.73234	44.82592	312	44.82592	341	44.82592
67	44.9	3.4378	44.84638	282	44.84638	318	44.84638
60	44.92	3.20866	44.86684	248	44.86684	292	44.86684
48	44.94	3.03346	44.88731	212	44.88731	265	44.88731
34	44.96	2.9047	44.90777	186	44.90777	236	44.90777
36	44.98	2.8178	44.92823	151	44.92823	195	44.92823
39	45	2.77054	44.94869	123	44.94869	150	44.94869
45	45.02	2.7629	44.96916	99	44.96916	114	44.96916
45	45.04	2.79706	44.98962	83	44.98962	86	44.98962
60	45.06	2.8778	45.01008	70	45.01008	61	45.01008
88	45.08	3.0131	45.03054	53	45.03054	46	45.03054
141	45.1	3.21544	45.05101	36	45.05101	42	45.05101
208	45.12	3.50376	45.07147	26	45.07147	35	45.07147
298	45.14	3.907	45.09193	21	45.09193	32	45.09193
401	45.16	4.47074	45.1124	15	45.1124	22	45.1124
499	45.18	5.30176	45.13286	9	45.13286	9	45.13286
561	45.2	6.48216	45.15332	8	45.15332	7	45.15332
588	45.22	8.32342	45.17379	8	45.17379	10	45.17379
575	45.24	11.37286	45.19425	8	45.19425	4	45.19425
543	45.26	16.55224	45.21471	8	45.21471	0	45.21471
487	45.28	25.058	45.23517	4	45.23517	0	45.23517
429	45.3	37.607	45.25563	0	45.25563	1	45.25563
364	45.32	51.5784	45.2761	7	45.2761	5	45.2761
305	45.34	56.9222	45.29656	13	45.29656	5	45.29656
253	45.36	47.596	45.31702	15	45.31702	5	45.31702
205	45.38	33.305	45.33749	15	45.33749	10	45.33749
153	45.4	21.9014	45.35795	18	45.35795	12	45.35795
105	45.42	14.47744	45.37841	17	45.37841	16	45.37841
69	45.44	10.01064	45.39888	18	45.39888	15	45.39888
47	45.46	7.35224	45.41934	24	45.41934	21	45.41934
32	45.48	5.59416	45.4398	25	45.4398	26	45.4398
37	45.5	4.49422	45.46026	34	45.46026	34	45.46026
37	45.52	3.71716	45.48072	57	45.48072	33	45.48072
55	45.54	3.14634	45.50119	70	45.50119	34	45.50119
88	45.56	2.71668	45.52165	75	45.52165	42	45.52165
142	45.58	2.3885	45.54211	81	45.54211	52	45.54211

230	45.6	2.13628	45.56258	84	45.56258	69	45.56258
353	45.62	1.943624	45.58304	77	45.58304	80	45.58304
517	45.64	1.80031	45.6035	73	45.6035	88	45.6035
678	45.66	1.701206	45.62397	62	45.62397	97	45.62397
804	45.68	1.815002	45.64443	43	45.64443	95	45.64443
886	45.7	1.81681	45.66489	36	45.66489	81	45.66489
914	45.72	1.892104	45.68535	32	45.68535	69	45.68535
898	45.74	2.08586	45.70582	21	45.70582	55	45.70582
844	45.76	2.47348	45.72628	11	45.72628	38	45.72628
767	45.78	3.14734	45.74674	4	45.74674	31	45.74674
681	45.8	4.13992	45.7672	7	45.7672	32	45.7672
608	45.82	5.15458	45.78767	6	45.78767	25	45.78767
552	45.84	5.33708	45.80813	14	45.80813	23	45.80813
489	45.86	4.4456	45.82859	15	45.82859	19	45.82859
407	45.88	3.3306	45.84906	14	45.84906	13	45.84906
325	45.9	2.49576	45.86952	17	45.86952	4	45.86952
246	45.92	1.980142	45.88998	16	45.88998	14	45.88998
182	45.94	1.703004	45.91045	9	45.91045	14	45.91045
143	45.96	1.447622	45.93091	7	45.93091	10	45.93091
111	45.98	1.41516	45.95137	3	45.95137	17	45.95137
81	46	1.373946	45.97183	5	45.97183	17	45.97183
62	46.02	1.265572	45.99229	8	45.99229	17	45.99229
44	46.04	1.139968	46.01276	17	46.01276	21	46.01276
30	46.06	1.044268	46.03322	19	46.03322	24	46.03322
17	46.08	0.983804	46.05368	23	46.05368	24	46.05368
11	46.1	0.950562	46.07415	24	46.07415	24	46.07415
5	46.12	0.936106	46.09461	22	46.09461	26	46.09461
6	46.14	0.934564	46.11507	16	46.11507	25	46.11507
8	46.16	0.923136	46.13554	15	46.13554	21	46.13554
8	46.18	0.939742	46.156	8	46.156	17	46.156
9	46.2	0.96371	46.17646	5	46.17646	11	46.17646
10	46.22	0.994946	46.19692	8	46.19692	6	46.19692
9	46.24	1.033742	46.21738	8	46.21738	7	46.21738
5	46.26	1.08072	46.23785	5	46.23785	7	46.23785
8	46.28	1.136842	46.25831	8	46.25831	6	46.25831
8	46.3	1.203474	46.27877	8	46.27877	7	46.27877
5	46.32	1.282468	46.29924	7	46.29924	5	46.29924
3	46.34	1.376322	46.3197	6	46.3197	8	46.3197
0	46.36	1.488408	46.34016	7	46.34016	7	46.34016
1	46.38	1.62332	46.36063	6	46.36063	8	46.36063
11	46.4	1.787408	46.38109	8	46.38109	7	46.38109
33	46.42	1.98963	46.40155	6	46.40155	8	46.40155
67	46.44	2.2429	46.42201	4	46.42201	6	46.42201
107	46.46	2.56646	46.44248	3	46.44248	2	46.44248
149	46.48	2.99042	46.46294	3	46.46294	3	46.46294
181	46.5	3.58488	46.4834	2	46.4834	5	46.4834
196	46.52	4.40866	46.50386	2	46.50386	3	46.50386
199	46.54	5.67196	46.52433	6	46.52433	4	46.52433
187	46.56	7.73606	46.54479	7	46.54479	4	46.54479
171	46.58	11.17588	46.56525	13	46.56525	1	46.56525
156	46.6	16.66196	46.58572	13	46.58572	4	46.58572
138	46.62	24.3364	46.60618	17	46.60618	4	46.60618
113	46.64	31.7238	46.62664	17	46.62664	16	46.62664
97	46.66	32.5756	46.64711	16	46.64711	29	46.64711
84	46.68	26.0466	46.66757	14	46.66757	39	46.66757
68	46.7	18.38176	46.68803	14	46.68803	47	46.68803
47	46.72	12.7761	46.70849	10	46.70849	54	46.70849
30	46.74	9.32528	46.72895	13	46.72895	59	46.72895

18	46.76	7.386	46.74942	12	46.74942	52	46.74942
15	46.78	6.36076	46.76988	17	46.76988	47	46.76988
10	46.8	5.80994	46.79034	25	46.79034	54	46.79034
5	46.82	5.64262	46.81081	37	46.81081	54	46.81081
0	46.84	5.71236	46.83127	51	46.83127	71	46.83127
0	46.86	5.9857	46.85173	58	46.85173	77	46.85173
7	46.88	6.46114	46.8722	72	46.8722	82	46.8722
10	46.9	7.16162	46.89266	72	46.89266	85	46.89266
14	46.92	8.13576	46.91312	67	46.91312	88	46.91312
19	46.94	9.4662	46.93358	64	46.93358	92	46.93358
20	46.96	11.52704	46.95404	62	46.95404	88	46.95404
19	46.98	14.07874	46.97451	58	46.97451	96	46.97451
17	47	17.78324	46.99497	49	46.99497	95	46.99497
17	47.02	23.4974	47.01543	42	47.01543	89	47.01543
15	47.04	32.8872	47.0359	50	47.0359	90	47.0359
11	47.06	48.7692	47.05636	51	47.05636	80	47.05636
11	47.08	74.8216	47.07682	64	47.07682	81	47.07682
7	47.1	113.7308	47.09729	73	47.09729	82	47.09729
9	47.12	156.9482	47.11775	88	47.11775	85	47.11775
9	47.14	174.2628	47.13821	113	47.13821	102	47.13821
7	47.16	146.2516	47.15867	142	47.15867	104	47.15867
4	47.18	102.3104	47.17914	162	47.17914	127	47.17914
6	47.2	67.0524	47.1996	195	47.1996	138	47.1996
5	47.22	44.0686	47.22006	228	47.22006	160	47.22006
6	47.24	30.2516	47.24052	279	47.24052	201	47.24052
7	47.26	22.0524	47.26099	315	47.26099	249	47.26099
6	47.28	16.97318	47.28145	369	47.28145	303	47.28145
3	47.3	13.60476	47.30191	406	47.30191	365	47.30191
7	47.32	11.2297	47.32238	440	47.32238	425	47.32238
8	47.34	9.4921	47.34284	481	47.34284	467	47.34284
7	47.36	8.1839	47.3633	496	47.3633	485	47.3633
5	47.38	7.17962	47.38377	493	47.38377	499	47.38377
6	47.4	6.39698	47.40423	489	47.40423	477	47.40423
2	47.42	5.78002	47.42469	455	47.42469	447	47.42469
4	47.44	5.28972	47.44515	424	47.44515	415	47.44515
4	47.46	4.8979	47.46561	374	47.46561	369	47.46561
6	47.48	4.58512	47.48608	327	47.48608	327	47.48608
7	47.5	4.33628	47.50654	269	47.50654	288	47.50654
10	47.52	4.14044	47.527	227	47.527	249	47.527
10	47.54	3.9895	47.54747	204	47.54747	197	47.54747
7	47.56	3.8775	47.56793	174	47.56793	160	47.56793
3	47.58	3.8002	47.58839	144	47.58839	123	47.58839
7	47.6	3.75468	47.60886	117	47.60886	81	47.60886
3	47.62	3.7393	47.62932	85	47.62932	54	47.62932
9	47.64	3.75354	47.64978	71	47.64978	32	47.64978
16	47.66	3.79804	47.67024	56	47.67024	20	47.67024
25	47.68	3.8749	47.6907	37	47.6907	19	47.6907
40	47.7	3.98788	47.71117	28	47.71117	14	47.71117
56	47.72	4.14318	47.73163	24	47.73163	10	47.73163
74	47.74	4.35044	47.75209	23	47.75209	8	47.75209
101	47.76	4.76146	47.77256	23	47.77256	11	47.77256
120	47.78	5.13266	47.79302	20	47.79302	7	47.79302
140	47.8	5.64336	47.81348	15	47.81348	2	47.81348
152	47.82	6.3854	47.83395	6	47.83395	1	47.83395
155	47.84	7.53714	47.85441	8	47.85441	0	47.85441
146	47.86	9.40256	47.87487	4	47.87487	1	47.87487
130	47.88	12.38596	47.89533	6	47.89533	0	47.89533
110	47.9	16.76364	47.9158	10	47.9158	1	47.9158

91	47.92	21.794	47.93626	13	47.93626	2	47.93626
78	47.94	24.2402	47.95672	15	47.95672	0	47.95672
73	47.96	21.705	47.97718	27	47.97718	6	47.97718
67	47.98	17.24144	47.99765	34	47.99765	10	47.99765
67	48	13.70838	48.01811	35	48.01811	11	48.01811
66	48.02	11.59192	48.03857	28	48.03857	20	48.03857
66	48.04	10.5766	48.05904	29	48.05904	25	48.05904
70	48.06	10.28284	48.0795	27	48.0795	23	48.0795
76	48.08	10.44708	48.09996	27	48.09996	20	48.09996
90	48.1	10.93224	48.12043	22	48.12043	21	48.12043
105	48.12	11.6874	48.14089	25	48.14089	20	48.14089
131	48.14	12.68218	48.16135	23	48.16135	23	48.16135
170	48.16	14.00836	48.18181	26	48.18181	30	48.18181
252	48.18	15.69754	48.20227	32	48.20227	33	48.20227
365	48.2	18.04798	48.22274	26	48.22274	24	48.22274
508	48.22	20.8166	48.2432	26	48.2432	38	48.2432
669	48.24	24.4334	48.26366	35	48.26366	28	48.26366
817	48.26	29.2902	48.28413	35	48.28413	36	48.28413
915	48.28	36.104	48.30459	36	48.30459	38	48.30459
959	48.3	46.2792	48.32505	40	48.32505	40	48.32505
931	48.32	62.473	48.34552	57	48.34552	47	48.34552
869	48.34	89.022	48.36598	71	48.36598	60	48.36598
783	48.36	131.3006	48.38644	95	48.38644	75	48.38644
690	48.38	191.7658	48.4069	125	48.4069	96	48.4069
615	48.4	255.736	48.42736	146	48.42736	115	48.42736
541	48.42	277.338	48.44783	181	48.44783	167	48.44783
460	48.44	241.168	48.46829	223	48.46829	214	48.46829
393	48.46	195.8712	48.48875	271	48.48875	285	48.48875
301	48.48	175.4978	48.50922	330	48.50922	358	48.50922
214	48.5	190.2136	48.52968	392	48.52968	441	48.52968
137	48.52	244.272	48.55014	468	48.55014	525	48.55014
90	48.54	335.114	48.57061	553	48.57061	608	48.57061
60	48.56	427.406	48.59107	631	48.59107	691	48.59107
34	48.58	435.548	48.61153	714	48.61153	783	48.61153
31	48.6	343.88	48.63199	793	48.63199	882	48.63199
21	48.62	235.92	48.65246	865	48.65246	981	48.65246
13	48.64	155.5956	48.67292	945	48.67292	1073	48.67292
18	48.66	104.477	48.69338	1020	48.69338	1173	48.69338
8	48.68	73.7758	48.71384	1102	48.71384	1304	48.71384
6	48.7	55.2216	48.73431	1166	48.73431	1410	48.73431
7	48.72	43.3834	48.75477	1210	48.75477	1484	48.75477
11	48.74	35.3062	48.77523	1222	48.77523	1523	48.77523
10	48.76	29.5002	48.7957	1188	48.7957	1508	48.7957
6	48.78	25.1852	48.81616	1146	48.81616	1470	48.81616
5	48.8	21.9108	48.83662	1074	48.83662	1391	48.83662
9	48.82	19.3942	48.85709	969	48.85709	1293	48.85709
9	48.84	17.4486	48.87755	869	48.87755	1190	48.87755
8	48.86	15.94856	48.89801	762	48.89801	1093	48.89801
5	48.88	14.80944	48.91847	667	48.91847	1004	48.91847
4	48.9	13.9757	48.93893	572	48.93893	889	48.93893
1	48.92	13.41408	48.9594	479	48.9594	774	48.9594
3	48.94	13.11032	48.97986	389	48.97986	651	48.97986
5	48.96	13.06848	49.00032	297	49.00032	515	49.00032
14	48.98	13.46498	49.02079	226	49.02079	395	49.02079
31	49	14.05322	49.04125	167	49.04125	290	49.04125
48	49.02	15.07348	49.06171	110	49.06171	218	49.06171
69	49.04	16.68994	49.08218	70	49.08218	159	49.08218
82	49.06	19.21136	49.10264	39	49.10264	115	49.10264

87	49.08	23.2574	49.1231	18	49.1231	77	49.1231
83	49.1	30.0486	49.14356	11	49.14356	52	49.14356
77	49.12	41.6978	49.16402	5	49.16402	28	49.16402
74	49.14	61.0742	49.18449	7	49.18449	19	49.18449
70	49.16	90.308	49.20495	4	49.20495	18	49.20495
73	49.18	124.8302	49.22541	14	49.22541	24	49.22541
77	49.2	142.3696	49.24588	33	49.24588	35	49.24588
71	49.22	123.6948	49.26634	49	49.26634	45	49.26634
69	49.24	89.0824	49.2868	75	49.2868	72	49.2868
64	49.26	60.1774	49.30727	109	49.30727	108	49.30727
68	49.28	41.1212	49.32773	146	49.32773	133	49.32773
75	49.3	29.7036	49.34819	184	49.34819	173	49.34819
88	49.32	23.0754	49.36865	210	49.36865	204	49.36865
102	49.34	19.16586	49.38912	232	49.38912	247	49.38912
141	49.36	16.79268	49.40958	242	49.40958	273	49.40958
195	49.38	15.36762	49.43004	240	49.43004	295	49.43004
284	49.4	14.59354	49.4505	219	49.4505	295	49.4505
390	49.42	14.38012	49.47097	189	49.47097	286	49.47097
560	49.44	14.77182	49.49143	159	49.49143	270	49.49143
755	49.46	15.58384	49.51189	135	49.51189	246	49.51189
983	49.48	17.0109	49.53236	111	49.53236	203	49.53236
1185	49.5	19.24224	49.55282	88	49.55282	162	49.55282
1351	49.52	22.6432	49.57328	71	49.57328	119	49.57328
1469	49.54	27.948	49.59375	56	49.59375	81	49.59375
1575	49.56	36.6386	49.61421	42	49.61421	46	49.61421
1685	49.58	51.4442	49.63467	28	49.63467	26	49.63467
1876	49.6	76.4122	49.65513	9	49.65513	5	49.65513
2145	49.62	115.677	49.67559	0	49.67559	0	49.67559
2489	49.64	167.5454	49.69606	0	49.69606	0	49.69606
2791	49.66	208.178	49.71652	13	49.71652	0	49.71652
2989	49.68	197.6932	49.73698	19	49.73698	4	49.73698
3029	49.7	148.0244	49.75745	31	49.75745	7	49.75745
2935	49.72	99.2842	49.77791	56	49.77791	25	49.77791
2697	49.74	65.1932	49.79837	91	49.79837	36	49.79837
2390	49.76	44.1076	49.81884	133	49.81884	68	49.81884
2078	49.78	31.5994	49.8393	172	49.8393	111	49.8393
1815	49.8	24.0266	49.85976	191	49.85976	140	49.85976
1600	49.82	19.14946	49.88022	191	49.88022	159	49.88022
1402	49.84	15.79312	49.90068	181	49.90068	154	49.90068
1177	49.86	13.36936	49.92115	136	49.92115	138	49.92115
914	49.88	11.32978	49.94161	90	49.94161	112	49.94161
668	49.9	9.96508	49.96207	47	49.96207	63	49.96207
455	49.92	8.91548	49.98254	47	49.98254	63	49.98254
269	49.94	8.10206	50.003	47	50.003	63	50.003
146	49.96	7.47096					
78	49.98	6.98482					
42	50	6.6177					
22	50.02	6.35178					
10	50.04	6.1753					
0	50.06	5.68348					
0	50.08	5.67998					
0	50.1	5.75728					
0	50.12	5.92086					
0	50.14	6.1811					
11	50.16	6.55446					
38	50.18	7.06572					
95	50.2	7.79644					
176	50.22	8.71166					

303	50.24	9.93634
438	50.26	11.59684
560	50.28	13.904
641	50.3	17.24606
667	50.32	22.3942
649	50.34	30.846
596	50.36	45.1316
517	50.38	68.557
439	50.4	103.1742
373	50.42	142.04
327	50.44	157.6772
280	50.46	132.6032
228	50.48	93.2048
165	50.5	61.6146
102	50.52	41.073
54	50.54	28.795
14	50.56	21.5976
0	50.58	17.24442
0	50.6	14.47934
0	50.62	12.6755
40	50.64	11.5253
107	50.66	10.84746
219	50.68	10.6279
368	50.7	10.81972
521	50.72	11.46496
624	50.74	12.6829
673	50.76	14.73406
667	50.78	18.16758
596	50.8	24.0632
502	50.82	34.2294
346	50.84	50.9692
261	50.86	75.5
184	50.88	102.1012
184	50.9	110.8492
184	50.92	91.958
	50.94	65.048
	50.96	44.4414
	50.98	32.0246
	51	26.0184
	51.02	24.1912
	51.04	23.7158
	51.06	21.249
	51.08	16.79606
	51.1	12.65092
	51.12	9.65486
	51.14	7.46718
	51.16	6.16852
	51.18	5.29202
	51.2	4.6706
	51.22	4.21178
	51.24	3.86588
	51.26	3.60452
	51.28	3.41032
	51.3	3.27232
	51.32	3.18392
	51.34	3.14182
	51.36	3.14564
	51.38	3.24094

51.4	3.3486
51.42	3.52064
51.44	3.77316
51.46	4.13146
51.48	4.63604
51.5	5.35648
51.52	6.42396
51.54	8.10074
51.56	10.88674
51.58	15.59302
51.6	23.2012
51.62	34.0578
51.64	45.0542
51.66	47.2654
51.68	38.219
51.7	26.7988
51.72	18.21242
51.74	12.80674
51.76	9.66744
51.78	7.90004
51.8	6.91298
51.82	6.38988
51.84	6.24912
51.86	6.29776
51.88	6.5856
51.9	6.9814
51.92	7.84532
51.94	9.13094
51.96	11.04504
51.98	14.0027
52	19.03722
52.02	27.2458
52.04	40.9938
52.06	62.2972
52.08	89.4634
52.1	108.2846
52.12	99.435
52.14	72.9252
52.16	48.5112
52.18	31.7988
52.2	21.5566
52.22	15.49188
52.24	11.80498
52.26	9.4159
52.28	7.76498
52.3	6.57084
52.32	5.68162
52.34	5.00656
52.36	4.38312
52.38	3.98354
52.4	3.6739
52.42	3.4355
52.44	3.25524
52.46	3.12404
52.48	3.03574
52.5	2.9866
52.52	2.97486
52.54	2.9888

52.56	3.1478
52.58	3.26044
52.6	3.42496
52.62	3.65312
52.64	3.96292
52.66	4.38198
52.68	4.9548
52.7	5.75852
52.72	6.94098
52.74	8.79722
52.76	11.87584
52.78	17.02618
52.8	25.1816
52.82	36.3092
52.84	46.1492
52.86	45.781
52.88	35.7788
52.9	25.072
52.92	17.44524
52.94	12.7815
52.96	10.14076
52.98	8.70298
53	7.9533
53.02	7.93276
53.04	7.91346
53.06	8.14534
53.08	8.62006
53.1	9.36056
53.12	10.42446
53.14	11.90712
53.16	14.05522
53.18	17.27284
53.2	22.3512
53.22	30.5618
53.24	43.447
53.26	61.6104
53.28	80.5048
53.3	87.3434
53.32	79.9424
53.34	73.2918
53.36	77.9082
53.38	98.4368
53.4	136.7484
53.42	185.1782
53.44	210.486
53.46	182.7144
53.48	130.65
53.5	86.8124
53.52	57.665
53.54	39.9638
53.56	29.4354
53.58	22.8436
53.6	18.5828
53.62	15.62142
53.64	13.4894
53.66	11.92828
53.68	10.78234
53.7	9.95286

53.72	9.4068
53.74	9.0457
53.76	8.87824
53.78	8.89932
53.8	9.11938
53.82	9.56718
53.84	10.29694
53.86	11.40432
53.88	13.06462
53.9	15.62352
53.92	19.77414
53.94	26.7848
53.96	38.5558
53.98	57.016
54	81.3972
54.02	100.6294
54.04	96.0976
54.06	73.28
54.08	50.9124
54.1	35.4426
54.12	26.1296
54.14	20.9286
54.16	18.2505
54.18	16.9202
54.2	16.52048
54.22	16.84598
54.24	17.8496
54.26	19.59068
54.28	22.236
54.3	26.1072
54.32	31.8274
54.34	40.5624
54.36	54.8566
54.38	79.0472
54.4	119.4354
54.42	181.7376
54.44	260.442
54.46	313.314
54.48	286.08
54.5	210.412
54.52	142.6392
54.54	98.418
54.56	74.5482
54.58	64.3676
54.6	59.9134
54.62	52.4778
54.64	41.1506
54.66	30.7978
54.68	23.2688
54.7	18.1897
54.72	14.7891
54.74	12.43366
54.76	10.71752
54.78	9.34818
54.8	8.32744
54.82	7.51528
54.84	6.8637
54.86	6.45724

54.88	6.04336
54.9	5.52462
54.92	5.29032
54.94	5.1347
54.96	5.05918
54.98	5.07132
55	5.18714
55.02	5.43624
55.04	5.87428
55.06	6.61232
55.08	7.87436
55.1	10.07108
55.12	13.81806
55.14	19.73962
55.16	27.5834
55.18	33.7528
55.2	32.215
55.22	24.7604
55.24	17.46002
55.26	12.3984
55.28	9.33314
55.3	7.7207
55.32	6.78146
55.34	6.30434
55.36	6.1269
55.38	6.18158
55.4	6.4519
55.42	6.95582
55.44	7.74658
55.46	8.93026
55.48	10.71548
55.5	13.43394
55.52	18.10168
55.54	26.0268
55.56	39.0412
55.58	58.2764
55.6	79.8592
55.62	88.5632
55.64	74.8672
55.66	53.521
55.68	36.8598
55.7	26.8506
55.72	22.1044
55.74	20.5598
55.76	19.41182
55.78	16.4966
55.8	12.85324
55.82	9.91262
55.84	7.90476
55.86	6.6145
55.88	5.7949
55.9	5.26722
55.92	4.92608
55.94	4.42202
55.96	4.32152
55.98	4.3083
56	4.37642
56.02	4.5448

56.04	4.78322
56.06	5.12246
56.08	5.5833
56.1	6.17096
56.12	6.9875
56.14	8.07918
56.16	9.56338
56.18	11.64272
56.2	14.70328
56.22	19.51612
56.24	27.5254
56.26	40.9962
56.28	62.4902
56.3	92.2234
56.32	119.5632
56.34	120.243
56.36	93.5986
56.38	63.8988
56.4	42.3436
56.42	28.9068
56.44	21.128
56.46	16.58184
56.48	13.88716
56.5	12.27772
56.52	11.38994
56.54	11.06222
56.56	11.24214
56.58	12.10056
56.6	13.4847
56.62	15.82546
56.64	19.75146
56.66	26.472
56.68	38.1168
56.7	57.2694
56.72	85.231
56.74	115.1798
56.76	124.19
56.78	101.8526
56.8	70.7076
56.82	46.4742
56.84	30.8396
56.86	21.4566
56.88	15.85644
56.9	12.34872
56.92	9.9942
56.94	8.31938
56.96	7.08068
56.98	6.14036
57	5.41288
57.02	4.84198
57.04	4.38932
57.06	4.0282
57.08	3.65656
57.1	3.42954
57.12	3.25218
57.14	3.11756
57.16	3.02148
57.18	2.96166

57.2	2.9377
57.22	2.94172
57.24	2.99754
57.26	3.10314
57.28	3.27192
57.3	3.5271
57.32	3.9118
57.34	4.51206
57.36	5.49652
57.38	7.15526
57.4	9.87924
57.42	13.95012
57.44	18.71048
57.46	21.0902
57.48	18.58558
57.5	14.03478
57.52	10.31552
57.54	7.95596
57.56	6.65574
57.58	6.0382
57.6	5.83648
57.62	5.90714
57.64	6.19154
57.66	6.71812
57.68	7.43584
57.7	8.43692
57.72	9.8237
57.74	11.78704
57.76	14.70162
57.78	19.29088
57.8	26.788
57.82	38.739
57.84	56.601
57.86	77.6432
57.88	89.5672
57.9	83.1784
57.92	71.5722
57.94	68.4976
57.96	78.9626
57.98	103.713
58	135.5446
58.02	149.0984
58.04	126.1054
58.06	89.0976
58.08	58.987
58.1	39.1508
58.12	27.0872
58.14	19.83318
58.16	15.27508
58.18	12.20744
58.2	10.01482
58.22	8.26252
58.24	7.01302
58.26	6.03442
58.28	5.25416
58.3	4.62244
58.32	4.10424
58.34	3.67436

58.36	3.3143
58.38	3.01032
58.4	2.75204
58.42	2.5316
58.44	2.34314
58.46	2.1826
58.48	2.0477
58.5	1.938024
58.52	1.854346
58.54	1.796012
58.56	1.75166
58.58	1.690456
58.6	1.6015
58.62	1.513424
58.64	1.558134
58.66	1.51169
58.68	1.4821
58.7	1.46652
58.72	1.463362
58.74	1.472264
58.76	1.49381
58.78	1.541942
58.8	1.593892
58.82	1.664944
58.84	1.759356
58.86	1.883162
58.88	2.04504
58.9	2.25766
58.92	2.52172
58.94	2.90126
58.96	3.41946
58.98	4.14196
59	5.19518
59.02	6.8628
59.04	9.67462
59.06	14.40656
59.08	21.838
59.1	31.6506
59.12	39.3476
59.14	37.3966
59.16	28.0392
59.18	18.85136
59.2	12.42786
59.22	8.45968
59.24	6.1109
59.26	4.69392
59.28	3.78584
59.3	3.1649
59.32	2.8238
59.34	2.4257
59.36	2.18434
59.38	2.00252
59.4	1.86535
59.42	1.76266
59.44	1.687368
59.46	1.634488
59.48	1.46354
59.5	1.449698

59.52	1.450536
59.54	1.465052
59.56	1.492764
59.58	1.533648
59.6	1.588112
59.62	1.656996
59.64	1.741606
59.66	1.843786
59.68	1.966014
59.7	2.16462
59.72	2.33924
59.74	2.54714
59.76	2.79574
59.78	3.09486
59.8	3.45776
59.82	3.90256
59.84	4.45456
59.86	5.14962
59.88	6.04022
59.9	7.20554
59.92	8.77244
59.94	10.96346
59.96	14.20908
59.98	19.36362
60	27.976
60.02	42.3396
60.04	64.7216
60.06	93.9362
60.08	116.1304
60.1	109.5768
60.12	82.3594
60.14	56.6538
60.16	39.8154
60.18	31.0136
60.2	27.4882
60.22	25.3788
60.24	21.395
60.26	16.443
60.28	12.39392
60.3	9.5984
60.32	7.77938
60.34	6.60512
60.36	5.8313
60.38	5.31136
60.4	4.98774
60.42	4.7773
60.44	4.67908
60.46	4.68326
60.48	4.78864
60.5	5.00232
60.52	5.34006
60.54	5.82918
60.56	6.51322
60.58	7.46114
60.6	8.74886
60.62	10.65414
60.64	13.5329
60.66	18.16988

60.68	25.9848
60.7	39.0678
60.72	59.4472
60.74	85.8758
60.76	105.3802
60.78	98.417
60.8	73.0386
60.82	48.9346
60.84	32.3092
60.86	22.1356
60.88	16.18182
60.9	12.66046
60.92	10.49256
60.94	9.19384
60.96	8.36716
60.98	7.95928
61	7.93212
61.02	8.3182
61.04	9.25094
61.06	11.0482
61.08	14.33858
61.1	20.1296
61.12	29.5778
61.14	42.8236
61.16	55.1912
61.18	55.6632
61.2	43.546
61.22	29.8446
61.24	19.80712
61.26	13.4717
61.28	9.6865
61.3	7.4038
61.32	5.94692
61.34	4.95178
61.36	4.23576
61.38	3.70324
61.4	3.29888
61.42	2.98782
61.44	2.78046
61.46	2.59544
61.48	2.45462
61.5	2.35086
61.52	2.27948
61.54	2.2379
61.56	2.11546
61.58	2.13552
61.6	2.18818
61.62	2.27882
61.64	2.41648
61.66	2.6157
61.68	2.88846
61.7	3.3037
61.72	3.93514
61.74	4.95392
61.76	6.6647
61.78	9.5038
61.8	13.86262
61.82	19.34094

61.84	22.996
61.86	21.09
61.88	15.88382
61.9	11.21752
61.92	8.1104
61.94	6.2999
61.96	5.33846
61.98	4.88436
62	4.76896
62.02	4.8501
62.04	5.12704
62.06	5.6111
62.08	6.34898
62.1	7.44046
62.12	9.08932
62.14	11.70292
62.16	16.01428
62.18	23.0974
62.2	33.9942
62.22	48.0816
62.24	58.7914
62.26	57.145
62.28	48.071
62.3	42.3288
62.32	44.2362
62.34	54.2002
62.36	68.3422
62.38	74.0462
62.4	62.4288
62.42	44.3242
62.44	29.6704
62.46	20.0206
62.48	14.14484
62.5	10.61822
62.52	8.39956
62.54	6.9176
62.56	5.8757
62.58	5.1219
62.6	4.57186
62.62	4.1751
62.64	3.90072
62.66	3.67928
62.68	3.60802
62.7	3.63546
62.72	3.77376
62.74	4.04972
62.76	4.51596
62.78	5.27832
62.8	6.55304
62.82	8.74852
62.84	12.50828
62.86	18.56252
62.88	26.9804
62.9	34.7534
62.92	35.0054
62.94	27.3798
62.96	18.85062
62.98	12.64252

63	8.75634
63.02	6.46826
63.04	5.13668
63.06	4.32286
63.08	3.8159
63.1	3.50952
63.12	3.35462
63.14	3.33282
63.16	3.44936
63.18	3.74002
63.2	4.29232
63.22	5.27668
63.24	6.95836
63.26	9.62742
63.28	13.26532
63.3	16.55146
63.32	16.95502
63.34	15.18234
63.36	14.2597
63.38	15.77126
63.4	20.085
63.42	26.0866
63.44	29.2336
63.46	25.3898
63.48	18.31098
63.5	12.34806
63.52	8.37186
63.54	5.94576
63.56	4.49964
63.58	3.59896
63.6	3.00424
63.62	2.58988
63.64	2.29216
63.66	2.0761
63.68	1.920962
63.7	1.813998
63.72	1.698436
63.74	1.670586
63.76	1.67972
63.78	1.729534
63.8	1.828924
63.82	1.995936
63.84	2.20148
63.86	2.65434
63.88	3.42596
63.9	4.72818
63.92	6.78388
63.94	9.5409
63.96	11.8566
63.98	11.54348
64	9.04836
64.02	6.52646
64.04	4.78756
64.06	3.77214
64.08	3.25344
64.1	3.04358
64.12	3.03226
64.14	3.17212

64.16	3.45614
64.18	3.9058
64.2	4.57374
64.22	5.56594
64.24	7.09802
64.26	9.5992
64.28	13.8255
64.3	20.8362
64.32	31.516
64.34	44.6076
64.36	52.5116
64.38	47.1168
64.4	34.9302
64.42	24.8998
64.44	19.22374
64.46	17.2384
64.48	17.08562
64.5	15.8312
64.52	12.5368
64.54	9.0764
64.56	6.49912
64.58	4.79732
64.6	3.72094
64.62	3.01342
64.64	2.52774
64.66	2.17594
64.68	1.911498
64.7	1.708636
64.72	1.551856
64.74	1.431278
64.76	1.340576
64.78	1.275986
64.8	1.223434
64.82	1.208534
64.84	1.22151
64.86	1.269356
64.88	1.366858
64.9	1.518694
64.92	1.838768
64.94	2.4012
64.96	3.33374
64.98	4.70008
65	6.22034
65.02	6.67292
65.04	5.60232
65.06	4.10912
65.08	2.96112
65.1	2.26216
65.12	1.860782
65.14	1.659188
65.16	1.579448
65.18	1.582218
65.2	1.653866
65.22	1.796856
65.24	2.02748
65.26	2.38204
65.28	2.93614
65.3	3.84376

65.32	5.38694
65.34	7.98572
65.36	12.05864
65.38	17.40004
65.4	21.4864
65.42	20.2706
65.44	15.13198
65.46	10.17124
65.48	6.72014
65.5	4.59282
65.52	3.33472
65.54	2.5759
65.56	2.09004
65.58	1.758906
65.6	1.523228
65.62	1.351406
65.64	1.23388
65.66	1.141004
65.68	1.073866
65.7	1.027354
65.72	0.998144
65.74	0.984206
65.76	0.98455
65.78	0.999104
65.8	1.028726
65.82	1.02584
65.84	1.094244
65.86	1.188514
65.88	1.317564
65.9	1.496656
65.92	1.75442
65.94	2.1479
65.96	2.78614
65.98	3.83746
66	5.60618
66.02	8.04944
66.04	10.59326
66.06	11.27814
66.08	9.4108
66.1	6.93794
66.12	5.09206
66.14	3.98626
66.16	3.41804
66.18	3.19722
66.2	3.19952
66.22	3.36786
66.24	3.69118
66.26	4.19076
66.28	4.92164
66.3	5.99488
66.32	7.63584
66.34	10.29226
66.36	14.75214
66.38	22.1206
66.4	33.3276
66.42	47.0934
66.44	55.5788
66.46	50.4098

66.48	38.462
66.5	29.1624
66.52	25.0294
66.54	25.3504
66.56	27.2202
66.58	25.6614
66.6	20.0044
66.62	14.13064
66.64	9.88142
66.66	7.12326
66.68	5.4142
66.7	4.33576
66.72	3.6178
66.74	3.1126
66.76	2.74382
66.78	2.4701
66.8	2.26708
66.82	2.1196
66.84	2.0183
66.86	1.958132
66.88	1.917298
66.9	1.939162
66.92	2.01014
66.94	2.14616
66.96	2.3808
66.98	2.78268
67	3.47656
67.02	4.67324
67.04	6.49418
67.06	8.84608
67.08	10.57102
67.1	9.9856
67.12	7.84586
67.14	5.8672
67.16	4.57996
67.18	3.87626
67.2	3.58014
67.22	3.54972
67.24	3.71026
67.26	4.04084
67.28	4.55898
67.3	5.318
67.32	6.42488
67.34	8.09376
67.36	10.75806
67.38	15.22748
67.4	22.7576
67.42	34.73
67.44	51.1004
67.46	65.5804
67.48	64.9922
67.5	50.2096
67.52	34.4944
67.54	23.55
67.56	17.24122
67.58	13.9807
67.6	11.81258
67.62	9.5088

67.64	7.36036
67.66	5.73984
67.68	4.63202
67.7	3.87808
67.72	3.365
67.74	3.00796
67.76	2.75674
67.78	2.58356
67.8	2.47324
67.82	2.41792
67.84	2.41478
67.86	2.46534
67.88	2.57578
67.9	2.7068
67.92	2.98318
67.94	3.38698
67.96	3.98064
67.98	4.88598
68	6.347
68.02	8.8133
68.04	12.9678
68.06	19.56786
68.08	28.363
68.1	35.6336
68.12	34.465
68.14	26.2398
68.16	17.8931
68.18	12.01936
68.2	8.41352
68.22	6.32924
68.24	5.13988
68.26	4.45996
68.28	4.0941
68.3	3.95676
68.32	4.02208
68.34	4.30812
68.36	4.89004
68.38	5.94442
68.4	7.81472
68.42	11.0408
68.44	16.22112
68.46	23.3458
68.48	29.7244
68.5	29.7162
68.52	23.777
68.54	17.76884
68.56	14.3136
68.58	13.47226
68.6	14.1176
68.62	13.86456
68.64	11.38766
68.66	8.34388
68.68	5.9601
68.7	4.38464
68.72	3.40072
68.74	2.78478
68.76	2.38258
68.78	2.1066

68.8	1.911532
68.82	1.773572
68.84	1.679414
68.86	1.62146
68.88	1.595868
68.9	1.601788
68.92	1.641334
68.94	1.720238
68.96	1.787882
68.98	1.991542
69	2.31162
69.02	2.8361
69.04	3.72542
69.06	5.19334
69.08	7.45228
69.1	10.2891
69.12	12.17746
69.14	11.21726
69.16	8.5803
69.18	6.29694
69.2	4.77256
69.22	3.93254
69.24	3.55278
69.26	3.46656
69.28	3.58628
69.3	3.88546
69.32	4.3803
69.34	5.12748
69.36	6.24646
69.38	7.98366
69.4	10.8328
69.42	15.66922
69.44	23.7292
69.46	36.0682
69.48	51.3146
69.5	60.696
69.52	54.274
69.54	39.209
69.56	25.945
69.58	16.97296
69.6	11.53284
69.62	8.3155
69.64	6.35396
69.66	5.0788
69.68	4.22202
69.7	3.58802
69.72	3.12056
69.74	2.77172
69.76	2.51084
69.78	2.31782
69.8	2.1794
69.82	2.08712
69.84	2.0361
69.86	2.02448
69.88	2.05324
69.9	2.12662
69.92	2.25284
69.94	2.44602

69.96	2.7298
69.98	3.11914
70	3.74804
70.02	4.7495
70.04	6.43416
70.06	9.27354
70.08	13.83228
70.1	20.258
70.12	26.4694
70.14	27.1754
70.16	21.5892
70.18	15.0035
70.2	10.13764
70.22	7.08994
70.24	5.31354
70.26	4.29732
70.28	3.70892
70.3	3.37132
70.32	3.20012
70.34	3.15746
70.36	3.23062
70.38	3.426
70.4	3.7728
70.42	4.33186
70.44	5.20196
70.46	6.50788
70.48	8.32724
70.5	10.45978
70.52	12.52096
70.54	15.25174
70.56	20.4262
70.58	29.615
70.6	43.2836
70.62	57.4576
70.64	60.4594
70.66	48.634
70.68	33.4806
70.7	21.9738
70.72	14.62662
70.74	10.24322
70.76	7.63698
70.78	6.01346
70.8	4.9372
70.82	4.19032
70.84	3.66144
70.86	3.28894
70.88	3.03682
70.9	2.88474
70.92	2.82374
70.94	2.85506
70.96	2.933
70.98	3.20654
71	3.68016
71.02	4.48876
71.04	5.89484
71.06	8.37366
71.08	12.37154
71.1	18.14962

71.12	24.1382
71.14	25.5512
71.16	20.7446
71.18	14.44998
71.2	9.64582
71.22	6.57722
71.24	4.7535
71.26	3.6815
71.28	3.03032
71.3	2.62
71.32	2.36336
71.34	2.22078
71.36	2.17876
71.38	2.24598
71.4	2.46142
71.42	2.90934
71.44	3.72518
71.46	5.05904
71.48	6.90262
71.5	8.56646
71.52	8.69868
71.54	7.57978
71.56	6.77244
71.58	7.02702
71.6	8.37512
71.62	10.01432
71.64	10.11028
71.66	8.116
71.68	5.75992
71.7	3.97354
71.72	2.82678
71.74	2.13184
71.76	1.708668
71.78	1.43776
71.8	1.253512
71.82	1.12266
71.84	1.027792
71.86	0.958934
71.88	0.909946
71.9	0.87698
71.92	0.857728
71.94	0.851084
71.96	0.857054
71.98	0.87706
72	0.911518
72.02	1.004438
72.04	1.11184
72.06	1.287296
72.08	1.560074
72.1	1.933852
72.12	2.23568
72.14	2.30988
72.16	2.09016
72.18	1.85096
72.2	1.72282
72.22	1.711098
72.24	1.794636
72.26	1.957942

72.28	2.19902
72.3	2.53032
72.32	2.97976
72.34	3.5974
72.36	4.4752
72.38	5.79428
72.4	7.91278
72.42	11.46506
72.44	17.3528
72.46	26.3684
72.48	37.6136
72.5	44.8888
72.52	40.8296
72.54	30.457
72.56	21.6452
72.58	16.49468
72.6	14.34548
72.62	13.46068
72.64	11.6607
72.66	8.9417
72.68	6.54108
72.7	4.85094
72.72	3.75424
72.74	3.05584
72.76	2.6016
72.78	2.29588
72.8	2.08678
72.82	1.947342
72.84	1.863376
72.86	1.827906
72.88	1.838964
72.9	1.899078
72.92	2.01586
72.94	2.20394
72.96	2.4896
72.98	2.9223
73	3.59446
73.02	4.73758
73.04	6.64616
73.06	9.82134
73.08	14.65528
73.1	20.5396
73.12	23.9508
73.14	21.2664
73.16	15.5711
73.18	10.5956
73.2	7.29918
73.22	5.34604
73.24	4.25356
73.26	3.66478
73.28	3.37834
73.3	3.30288
73.32	3.41144
73.34	3.72208
73.36	4.30788
73.38	5.33952
73.4	7.15318
73.42	10.2903

73.44	15.38242
73.46	22.5502
73.48	29.4032
73.5	29.9924
73.52	23.5952
73.54	16.15106
73.56	10.6555
73.58	7.18152
73.6	5.11042
73.62	3.89004
73.64	3.10432
73.66	2.57222
73.68	2.19242
73.7	1.912058
73.72	1.700622
73.74	1.538932
73.76	1.414278
73.78	1.318
73.8	1.24407
73.82	1.188266
73.84	1.147628
73.86	1.120134
73.88	1.104504
73.9	1.100106
73.92	1.10695
73.94	1.125848
73.96	1.158856
73.98	1.168976
74	1.301706
74.02	1.428106
74.04	1.624498
74.06	1.917206
74.08	2.29862
74.1	2.62114
74.12	2.65584
74.14	2.45882
74.16	2.28218
74.18	2.21762
74.2	2.26124
74.22	2.3928
74.24	2.59922
74.26	2.8796
74.28	3.24516
74.3	3.71968
74.32	4.34458
74.34	5.19394
74.36	6.40784
74.38	8.2462
74.4	11.13424
74.42	15.61982
74.44	22.054
74.46	29.394
74.48	33.8752
74.5	34.454
74.52	36.6418
74.54	45.12
74.56	61.3842
74.58	81.3644

74.6	89.643
74.62	75.5486
74.64	53.188
74.66	35.12
74.68	23.285
74.7	16.13952
74.72	11.88642
74.74	9.25212
74.76	7.51416
74.78	6.30624
74.8	5.44226
74.82	4.81994
74.84	4.37892
74.86	4.08332
74.88	3.91414
74.9	3.8664
74.92	3.95014
74.94	4.19756
74.96	4.7438
74.98	5.59942
75	7.1776
75.02	9.90484
75.04	14.262
75.06	20.1406
75.08	25.038
75.1	24.3014
75.12	18.81268
75.14	13.23858
75.16	9.34442
75.18	7.00244
75.2	5.71806
75.22	5.07896
75.24	4.83942
75.26	4.8891
75.28	5.20458
75.3	5.82764
75.32	6.88406
75.34	8.65428
75.36	11.69274
75.38	16.9176
75.4	25.4676
75.42	37.833
75.44	50.7404
75.46	54.005
75.48	43.901
75.5	30.5422
75.52	20.415
75.54	14.10168
75.56	10.48388
75.58	8.26734
75.6	6.527
75.62	5.15388
75.64	4.1462
75.66	3.4266
75.68	2.90976
75.7	2.52966
75.72	2.24178
75.74	2.01812

75.76	1.841396
75.78	1.700402
75.8	1.58751
75.82	1.49732
75.84	1.425936
75.86	1.37053
75.88	1.32906
75.9	1.300094
75.92	1.282678
75.94	1.254682
75.96	1.27427
75.98	1.290684
76	1.318644
76.02	1.359088
76.04	1.41341
76.06	1.399884
76.08	1.490798
76.1	1.60375
76.12	1.74363
76.14	1.91708
76.16	2.13324
76.18	2.40486
76.2	2.75008
76.22	3.19536
76.24	3.78048
76.26	4.5685
76.28	5.66834
76.3	7.28824
76.32	9.84354
76.34	14.1062
76.36	21.2706
76.38	32.6466
76.4	48.1772
76.42	61.8446
76.44	61.113
76.46	46.8574
76.48	31.6282
76.5	20.7182
76.52	13.9488
76.54	9.96318
76.56	7.5321
76.58	5.9252
76.6	4.80832
76.62	4.01554
76.64	3.44436
76.66	3.02884
76.68	2.72602
76.7	2.5083
76.72	2.35836
76.74	2.26592
76.76	2.22584
76.78	2.23724
76.8	2.30344
76.82	2.43284
76.84	2.6406
76.86	2.9525
76.88	3.41352
76.9	4.137

76.92	5.1859
76.94	7.02298
76.96	10.1494
76.98	15.27674
77	22.8056
77.02	31.098
77.04	34.024
77.06	28.3064
77.08	19.85716
77.1	13.19422
77.12	8.89352
77.14	6.338
77.16	4.85288
77.18	3.97236
77.2	3.44012
77.22	3.1343
77.24	3.00514
77.26	3.04778
77.28	3.30464
77.3	3.8898
77.32	5.01718
77.34	6.98564
77.36	10.0218
77.38	13.65444
77.4	15.53044
77.42	13.54166
77.44	9.80438
77.46	6.6605
77.48	4.57156
77.5	3.33198
77.52	2.58
77.54	2.11838
77.56	1.817598
77.58	1.611702
77.6	1.46742
77.62	1.366654
77.64	1.298716
77.66	1.257084
77.68	1.237914
77.7	1.23929
77.72	1.260864
77.74	1.303786
77.76	1.370856
77.78	1.46696
77.8	1.599954
77.82	1.782278
77.84	2.03438
77.86	2.41578
77.88	2.95208
77.9	3.80688
77.92	5.1758
77.94	7.55058
77.96	11.22772
77.98	15.9707
78	19.41944
78.02	18.15546
78.04	13.701
78.06	9.53788

78.08	6.71968
78.1	5.05898
78.12	4.16658
78.14	3.73694
78.16	3.59378
78.18	3.65958
78.2	3.91874
78.22	4.40294
78.24	5.20668
78.26	6.53626
78.28	8.77848
78.3	12.5224
78.32	18.38926
78.34	26.2454
78.36	32.944
78.38	32.9472
78.4	28.0134
78.42	24.4668
78.44	24.9766
78.46	29.2168
78.48	33.4656
78.5	31.426
78.52	23.9108
78.54	16.475
78.56	11.21254
78.58	7.91438
78.6	5.93252
78.62	4.72502
78.64	3.95384
78.66	3.44002
78.68	3.09416
78.7	2.87034
78.72	2.74438
78.74	2.7051
78.76	2.75142
78.78	2.89222
78.8	3.14964
78.82	3.58982
78.84	4.26068
78.86	5.35398
78.88	7.24654
78.9	10.46824
78.92	15.6277
78.94	22.7444
78.96	29.1782
78.98	29.1272
79	22.6664
79.02	15.60022
79.04	10.50884
79.06	7.35562
79.08	5.53418
79.1	4.50828
79.12	3.94104
79.14	3.66192
79.16	3.60156
79.18	3.75132
79.2	4.15874
79.22	4.95674

79.24	6.41624
79.26	8.97558
79.28	13.14228
79.3	18.99338
79.32	24.538
79.34	25.0302
79.36	20.1876
79.38	14.903
79.4	11.61118
79.42	10.35814
79.44	10.13346
79.46	9.24014
79.48	7.24784
79.5	5.27338
79.52	3.82862
79.54	2.8822
79.56	2.2808
79.58	1.890442
79.6	1.624128
79.62	1.433636
79.64	1.293684
79.66	1.190516
79.68	1.116204
79.7	1.066234
79.72	1.038524
79.74	1.033178
79.76	1.05281
79.78	1.103916
79.8	1.200512
79.82	1.371946
79.84	1.646366
79.86	2.17104
79.88	3.02266
79.9	4.22418
79.92	5.38736
79.94	5.52776
79.96	4.50818
79.98	3.28674
80	2.4261

200		250		300		350		400
0	3	49	3	33	3	19	3	12
0	3.020462	49	3.020462	33	3.020462	19	3.020462	12
0	3.040925	49	3.040925	33	3.040925	19	3.040925	12
0	3.061388	49	3.061388	33	3.061388	19	3.061388	12
14	3.081851	40	3.081851	45	3.081851	19	3.081851	12
15	3.102313	46	3.102313	56	3.102313	19	3.102313	39
22	3.122776	38	3.122776	65	3.122776	18	3.122776	45
25	3.143239	60	3.143239	64	3.143239	17	3.143239	40
25	3.163702	69	3.163702	53	3.163702	15	3.163702	48
21	3.184165	62	3.184165	31	3.184165	12	3.184165	45
15	3.204627	71	3.204627	35	3.204627	10	3.204627	36
5	3.22509	60	3.22509	20	3.22509	9	3.22509	20
7	3.245553	56	3.245553	11	3.245553	9	3.245553	0
1	3.266016	44	3.266016	0	3.266016	11	3.266016	0
0	3.286478	20	3.286478	0	3.286478	16	3.286478	3
0	3.306941	9	3.306941	0	3.306941	22	3.306941	5
0	3.327404	0	3.327404	6	3.327404	29	3.327404	5
0	3.347867	0	3.347867	9	3.347867	35	3.347867	2
0	3.36833	5	3.36833	11	3.36833	41	3.36833	8
0	3.388792	4	3.388792	12	3.388792	44	3.388792	8
0	3.409255	7	3.409255	11	3.409255	46	3.409255	11
0	3.429718	9	3.429718	10	3.429718	45	3.429718	12
0	3.450181	7	3.450181	7	3.450181	42	3.450181	8
0	3.470643	7	3.470643	3	3.470643	39	3.470643	9
0	3.491106	6	3.491106	0	3.491106	35	3.491106	7
0	3.511569	3	3.511569	0	3.511569	31	3.511569	11
0	3.532032	0	3.532032	0	3.532032	28	3.532032	10
0	3.552495	3	3.552495	1	3.552495	25	3.552495	7
0	3.572957	1	3.572957	5	3.572957	22	3.572957	5
1	3.59342	3	3.59342	8	3.59342	18	3.59342	4
1	3.613883	4	3.613883	8	3.613883	15	3.613883	5
1	3.634346	0	3.634346	8	3.634346	11	3.634346	3
1	3.654808	5	3.654808	11	3.654808	8	3.654808	1
1	3.675271	11	3.675271	13	3.675271	6	3.675271	3
1	3.695734	14	3.695734	16	3.695734	6	3.695734	3
0	3.716197	15	3.716197	16	3.716197	7	3.716197	0
1	3.73666	13	3.73666	13	3.73666	9	3.73666	2
2	3.757122	11	3.757122	17	3.757122	12	3.757122	3
2	3.777585	6	3.777585	10	3.777585	15	3.777585	2
2	3.798048	8	3.798048	12	3.798048	17	3.798048	5
0	3.818511	3	3.818511	6	3.818511	19	3.818511	6
3	3.838973	3	3.838973	11	3.838973	19	3.838973	6
9	3.859436	11	3.859436	7	3.859436	17	3.859436	0
12	3.879899	13	3.879899	19	3.879899	15	3.879899	1
13	3.900362	12	3.900362	38	3.900362	13	3.900362	10
11	3.920825	12	3.920825	47	3.920825	10	3.920825	16
17	3.941287	10	3.941287	36	3.941287	8	3.941287	22
19	3.96175	6	3.96175	50	3.96175	7	3.96175	27
19	3.982213	6	3.982213	53	3.982213	7	3.982213	20
12	4.002676	4	4.002676	59	4.002676	10	4.002676	26

1	4.023139	3	4.023139	48	4.023139	14	4.023139	23
17	4.043602	10	4.043602	37	4.043602	20	4.043602	37
23	4.064064	19	4.064064	13	4.064064	28	4.064064	41
24	4.084527	36	4.084527	28	4.084527	37	4.084527	49
12	4.10499	44	4.10499	44	4.10499	46	4.10499	56
24	4.125453	50	4.125453	47	4.125453	54	4.125453	51
37	4.145916	49	4.145916	52	4.145916	61	4.145916	33
40	4.166378	38	4.166378	99	4.166378	66	4.166378	33
34	4.186841	28	4.186841	126	4.186841	69	4.186841	11
27	4.207304	17	4.207304	131	4.207304	72	4.207304	16
23	4.227767	12	4.227767	128	4.227767	73	4.227767	14
31	4.248229	12	4.248229	116	4.248229	75	4.248229	41
34	4.268692	30	4.268692	93	4.268692	75	4.268692	69
31	4.289155	56	4.289155	79	4.289155	75	4.289155	91
22	4.309618	72	4.309618	54	4.309618	75	4.309618	106
32	4.330081	84	4.330081	38	4.330081	73	4.330081	106
28	4.350543	77	4.350543	28	4.350543	71	4.350543	116
20	4.371006	71	4.371006	48	4.371006	69	4.371006	126
9	4.391469	62	4.391469	68	4.391469	67	4.391469	128
3	4.411932	52	4.411932	83	4.411932	65	4.411932	146
0	4.432394	30	4.432394	116	4.432394	64	4.432394	160
0	4.452857	21	4.452857	150	4.452857	63	4.452857	167
1	4.47332	25	4.47332	148	4.47332	63	4.47332	194
1	4.493783	27	4.493783	130	4.493783	65	4.493783	202
0	4.514246	26	4.514246	123	4.514246	68	4.514246	209
4	4.534708	19	4.534708	119	4.534708	74	4.534708	223
13	4.555171	12	4.555171	92	4.555171	82	4.555171	224
35	4.575634	11	4.575634	80	4.575634	93	4.575634	232
49	4.596097	13	4.596097	72	4.596097	106	4.596097	233
65	4.616559	13	4.616559	92	4.616559	120	4.616559	256
79	4.637022	19	4.637022	132	4.637022	134	4.637022	250
78	4.657485	32	4.657485	153	4.657485	147	4.657485	253
76	4.677948	46	4.677948	177	4.677948	159	4.677948	273
61	4.698411	51	4.698411	192	4.698411	168	4.698411	294
33	4.718873	49	4.718873	238	4.718873	174	4.718873	316
31	4.739336	40	4.739336	257	4.739336	179	4.739336	361
16	4.759799	28	4.759799	260	4.759799	182	4.759799	354
17	4.780262	10	4.780262	244	4.780262	186	4.780262	365
8	4.800724	8	4.800724	244	4.800724	192	4.800724	368
10	4.821187	10	4.821187	243	4.821187	199	4.821187	388
8	4.84165	17	4.84165	242	4.84165	208	4.84165	403
15	4.862113	18	4.862113	236	4.862113	218	4.862113	414
19	4.882576	21	4.882576	225	4.882576	227	4.882576	428
19	4.903038	19	4.903038	235	4.903038	235	4.903038	437
25	4.923501	23	4.923501	257	4.923501	239	4.923501	441
40	4.943964	22	4.943964	266	4.943964	239	4.943964	464
52	4.964427	16	4.964427	279	4.964427	235	4.964427	452
54	4.984889	11	4.984889	290	4.984889	227	4.984889	445
49	5.005352	14	5.005352	325	5.005352	219	5.005352	453
50	5.025815	12	5.025815	330	5.025815	212	5.025815	482
53	5.046278	21	5.046278	345	5.046278	209	5.046278	510
57	5.066741	26	5.066741	367	5.066741	210	5.066741	499
45	5.087203	18	5.087203	363	5.087203	218	5.087203	522
38	5.107666	28	5.107666	377	5.107666	232	5.107666	521
37	5.128129	38	5.128129	374	5.128129	248	5.128129	549
39	5.148592	40	5.148592	369	5.148592	266	5.148592	556
37	5.169055	40	5.169055	368	5.169055	283	5.169055	552
26	5.189517	30	5.189517	383	5.189517	296	5.189517	542

20	5.20998	29	5.20998	403	5.20998	305	5.20998	548
21	5.230443	24	5.230443	405	5.230443	309	5.230443	586
25	5.250906	20	5.250906	428	5.250906	311	5.250906	615
22	5.271368	23	5.271368	470	5.271368	312	5.271368	645
20	5.291831	7	5.291831	495	5.291831	314	5.291831	661
37	5.312294	21	5.312294	514	5.312294	320	5.312294	660
57	5.332757	39	5.332757	527	5.332757	328	5.332757	670
59	5.35322	62	5.35322	536	5.35322	339	5.35322	640
68	5.373682	62	5.373682	559	5.373682	351	5.373682	619
86	5.394145	78	5.394145	579	5.394145	363	5.394145	611
105	5.414608	91	5.414608	599	5.414608	373	5.414608	612
106	5.435071	91	5.435071	603	5.435071	382	5.435071	649
110	5.455533	91	5.455533	612	5.455533	390	5.455533	670
95	5.475996	102	5.475996	631	5.475996	398	5.475996	693
78	5.496459	89	5.496459	628	5.496459	408	5.496459	682
86	5.516922	87	5.516922	639	5.516922	419	5.516922	701
89	5.537385	77	5.537385	643	5.537385	432	5.537385	714
79	5.557847	59	5.557847	642	5.557847	446	5.557847	709
85	5.57831	39	5.57831	658	5.57831	458	5.57831	703
94	5.598773	34	5.598773	676	5.598773	468	5.598773	701
95	5.619236	43	5.619236	707	5.619236	473	5.619236	701
105	5.639698	62	5.639698	726	5.639698	475	5.639698	705
114	5.660161	88	5.660161	723	5.660161	473	5.660161	707
110	5.680624	112	5.680624	716	5.680624	469	5.680624	711
118	5.701087	136	5.701087	717	5.701087	466	5.701087	703
146	5.72155	163	5.72155	731	5.72155	464	5.72155	738
183	5.742012	165	5.742012	750	5.742012	465	5.742012	752
216	5.762475	171	5.762475	763	5.762475	470	5.762475	749
237	5.782938	172	5.782938	795	5.782938	477	5.782938	765
263	5.803401	155	5.803401	840	5.803401	488	5.803401	770
286	5.823863	158	5.823863	878	5.823863	499	5.823863	789
325	5.844326	166	5.844326	906	5.844326	511	5.844326	800
339	5.864789	173	5.864789	910	5.864789	523	5.864789	822
350	5.885252	186	5.885252	917	5.885252	535	5.885252	840
374	5.905715	205	5.905715	935	5.905715	545	5.905715	848
408	5.926177	232	5.926177	940	5.926177	555	5.926177	869
461	5.94664	239	5.94664	968	5.94664	564	5.94664	885
520	5.967103	242	5.967103	990	5.967103	572	5.967103	889
592	5.987566	241	5.987566	1002	5.987566	579	5.987566	890
707	6.008028	230	6.008028	1014	6.008028	586	6.008028	875
831	6.028491	217	6.028491	1011	6.028491	592	6.028491	872
956	6.048954	224	6.048954	1018	6.048954	599	6.048954	877
1076	6.069417	223	6.069417	1027	6.069417	606	6.069417	905
1199	6.08988	255	6.08988	1039	6.08988	614	6.08988	943
1350	6.110342	279	6.110342	1053	6.110342	624	6.110342	970
1517	6.130805	289	6.130805	1074	6.130805	634	6.130805	999
1720	6.151268	310	6.151268	1105	6.151268	645	6.151268	1029
1936	6.171731	319	6.171731	1126	6.171731	656	6.171731	1067
2229	6.192193	329	6.192193	1150	6.192193	666	6.192193	1066
2586	6.212656	347	6.212656	1181	6.212656	675	6.212656	1078
3066	6.233119	375	6.233119	1225	6.233119	682	6.233119	1095
3700	6.253582	396	6.253582	1252	6.253582	687	6.253582	1116
4562	6.274045	423	6.274045	1289	6.274045	690	6.274045	1139
5649	6.294507	477	6.294507	1322	6.294507	691	6.294507	1134
6902	6.31497	511	6.31497	1366	6.31497	691	6.31497	1124
8198	6.335433	540	6.335433	1396	6.335433	690	6.335433	1117
9424	6.355896	572	6.355896	1429	6.355896	691	6.355896	1076
10461	6.376358	573	6.376358	1459	6.376358	692	6.376358	1072

11264	6.396821	580	6.396821	1479	6.396821	696	6.396821	1038
11739	6.417284	635	6.417284	1495	6.417284	702	6.417284	1032
11896	6.437747	673	6.437747	1515	6.437747	710	6.437747	1031
11775	6.458209	681	6.458209	1495	6.458209	718	6.458209	1027
11415	6.478672	722	6.478672	1514	6.478672	726	6.478672	1038
10864	6.499135	748	6.499135	1525	6.499135	732	6.499135	1024
10087	6.519598	778	6.519598	1570	6.519598	736	6.519598	1047
9132	6.540061	813	6.540061	1604	6.540061	738	6.540061	1044
8031	6.560523	844	6.560523	1663	6.560523	737	6.560523	1012
6899	6.580986	879	6.580986	1708	6.580986	735	6.580986	987
5796	6.601449	904	6.601449	1750	6.601449	732	6.601449	943
4769	6.621912	953	6.621912	1801	6.621912	728	6.621912	902
3862	6.642374	990	6.642374	1843	6.642374	725	6.642374	850
3127	6.662837	1029	6.662837	1886	6.662837	722	6.662837	815
2548	6.6833	1099	6.6833	1925	6.6833	718	6.6833	782
2125	6.703763	1141	6.703763	1946	6.703763	715	6.703763	760
1798	6.724226	1210	6.724226	1993	6.724226	712	6.724226	755
1575	6.744688	1263	6.744688	2046	6.744688	708	6.744688	726
1377	6.765151	1310	6.765151	2106	6.765151	704	6.765151	712
1251	6.785614	1341	6.785614	2138	6.785614	700	6.785614	677
1143	6.806077	1349	6.806077	2157	6.806077	693	6.806077	630
1046	6.826539	1381	6.826539	2205	6.826539	685	6.826539	594
980	6.847002	1422	6.847002	2245	6.847002	674	6.847002	535
910	6.867465	1467	6.867465	2291	6.867465	659	6.867465	501
856	6.887928	1519	6.887928	2316	6.887928	642	6.887928	478
812	6.908391	1577	6.908391	2326	6.908391	622	6.908391	482
798	6.928853	1641	6.928853	2347	6.928853	602	6.928853	479
783	6.949316	1681	6.949316	2421	6.949316	583	6.949316	477
751	6.969779	1700	6.969779	2470	6.969779	566	6.969779	470
745	6.990242	1719	6.990242	2496	6.990242	553	6.990242	450
742	7.010705	1758	7.010705	2504	7.010705	544	7.010705	426
740	7.031167	1767	7.031167	2542	7.031167	539	7.031167	391
759	7.05163	1780	7.05163	2531	7.05163	537	7.05163	347
785	7.072093	1808	7.072093	2524	7.072093	536	7.072093	328
813	7.092556	1824	7.092556	2503	7.092556	533	7.092556	325
871	7.113018	1837	7.113018	2468	7.113018	527	7.113018	334
966	7.133481	1860	7.133481	2465	7.133481	516	7.133481	347
1101	7.153944	1874	7.153944	2489	7.153944	501	7.153944	338
1290	7.174407	1873	7.174407	2468	7.174407	481	7.174407	307
1544	7.19487	1872	7.19487	2438	7.19487	460	7.19487	283
1824	7.215332	1868	7.215332	2357	7.215332	438	7.215332	271
2132	7.235795	1836	7.235795	2291	7.235795	417	7.235795	229
2448	7.256258	1823	7.256258	2175	7.256258	399	7.256258	213
2755	7.276721	1824	7.276721	2068	7.276721	383	7.276721	205
3051	7.297183	1794	7.297183	1951	7.297183	369	7.297183	214
3369	7.317646	1774	7.317646	1862	7.317646	356	7.317646	229
3742	7.338109	1766	7.338109	1791	7.338109	344	7.338109	255
4149	7.358572	1728	7.358572	1706	7.358572	333	7.358572	265
4626	7.379035	1699	7.379035	1654	7.379035	321	7.379035	256
5133	7.399497	1639	7.399497	1589	7.399497	310	7.399497	272
5603	7.41996	1585	7.41996	1492	7.41996	298	7.41996	277
5947	7.440423	1543	7.440423	1445	7.440423	287	7.440423	249
6112	7.460886	1498	7.460886	1373	7.460886	275	7.460886	223
6062	7.481348	1434	7.481348	1311	7.481348	263	7.481348	200
5777	7.501811	1365	7.501811	1252	7.501811	251	7.501811	191
5261	7.522274	1324	7.522274	1206	7.522274	239	7.522274	169
4564	7.542737	1278	7.542737	1155	7.542737	228	7.542737	137
3770	7.5632	1234	7.5632	1114	7.5632	219	7.5632	107

2993	7.583662	1203	7.583662	1116	7.583662	213	7.583662	84
2302	7.604125	1160	7.604125	1064	7.604125	208	7.604125	76
1717	7.624588	1124	7.624588	1039	7.624588	205	7.624588	72
1255	7.645051	1099	7.645051	1026	7.645051	202	7.645051	77
962	7.665513	1061	7.665513	995	7.665513	198	7.665513	73
775	7.685976	1000	7.685976	976	7.685976	192	7.685976	78
653	7.706439	942	7.706439	947	7.706439	184	7.706439	98
565	7.726902	914	7.726902	927	7.726902	173	7.726902	102
497	7.747365	878	7.747365	892	7.747365	161	7.747365	86
447	7.767827	871	7.767827	873	7.767827	147	7.767827	71
398	7.78829	863	7.78829	850	7.78829	134	7.78829	57
373	7.808753	849	7.808753	814	7.808753	122	7.808753	40
329	7.829216	828	7.829216	818	7.829216	111	7.829216	34
311	7.849678	821	7.849678	809	7.849678	101	7.849678	33
300	7.870141	815	7.870141	791	7.870141	92	7.870141	23
282	7.890604	803	7.890604	784	7.890604	83	7.890604	24
267	7.911067	782	7.911067	772	7.911067	75	7.911067	31
246	7.93153	768	7.93153	758	7.93153	68	7.93153	45
238	7.951992	739	7.951992	740	7.951992	63	7.951992	43
227	7.972455	743	7.972455	731	7.972455	60	7.972455	41
220	7.992918	729	7.992918	720	7.992918	61	7.992918	40
208	8.013381	701	8.013381	717	8.013381	64	8.013381	34
190	8.033843	704	8.033843	721	8.033843	69	8.033843	43
179	8.054306	708	8.054306	716	8.054306	74	8.054306	54
153	8.074769	713	8.074769	709	8.074769	78	8.074769	53
146	8.095232	727	8.095232	705	8.095232	79	8.095232	58
136	8.115695	711	8.115695	710	8.115695	76	8.115695	47
124	8.136157	697	8.136157	712	8.136157	70	8.136157	46
121	8.15662	685	8.15662	709	8.15662	62	8.15662	31
123	8.177083	686	8.177083	697	8.177083	53	8.177083	16
117	8.197546	677	8.197546	698	8.197546	44	8.197546	5
111	8.218008	659	8.218008	692	8.218008	37	8.218008	0
107	8.238471	665	8.238471	675	8.238471	32	8.238471	13
91	8.258934	659	8.258934	656	8.258934	29	8.258934	14
80	8.279397	648	8.279397	623	8.279397	28	8.279397	17
77	8.29986	655	8.29986	593	8.29986	28	8.29986	25
65	8.320322	641	8.320322	580	8.320322	28	8.320322	26
65	8.340785	653	8.340785	551	8.340785	27	8.340785	32
55	8.361248	660	8.361248	496	8.361248	25	8.361248	30
52	8.381711	657	8.381711	468	8.381711	23	8.381711	27
49	8.402173	652	8.402173	436	8.402173	20	8.402173	24
53	8.422636	625	8.422636	411	8.422636	16	8.422636	13
61	8.443099	584	8.443099	375	8.443099	13	8.443099	10
65	8.463562	553	8.463562	351	8.463562	10	8.463562	4
64	8.484025	508	8.484025	321	8.484025	8	8.484025	8
68	8.504487	490	8.504487	311	8.504487	6	8.504487	21
58	8.52495	479	8.52495	305	8.52495	6	8.52495	31
63	8.545413	473	8.545413	283	8.545413	7	8.545413	32
59	8.565876	459	8.565876	262	8.565876	8	8.565876	37
51	8.586338	457	8.586338	249	8.586338	10	8.586338	46
39	8.606801	458	8.606801	210	8.606801	12	8.606801	59
25	8.627264	449	8.627264	193	8.627264	15	8.627264	58
32	8.647727	418	8.647727	159	8.647727	17	8.647727	47
28	8.66819	390	8.66819	143	8.66819	18	8.66819	55
29	8.688652	358	8.688652	142	8.688652	19	8.688652	53
32	8.709115	334	8.709115	141	8.709115	20	8.709115	60
29	8.729578	316	8.729578	137	8.729578	20	8.729578	48
45	8.750041	279	8.750041	138	8.750041	20	8.750041	40

51	8.770503	255	8.770503	141	8.770503	20	8.770503	44
47	8.790966	249	8.790966	139	8.790966	19	8.790966	45
32	8.811429	242	8.811429	132	8.811429	18	8.811429	47
21	8.831892	235	8.831892	118	8.831892	17	8.831892	26
25	8.852355	215	8.852355	88	8.852355	15	8.852355	18
19	8.872817	198	8.872817	97	8.872817	14	8.872817	34
21	8.89328	173	8.89328	95	8.89328	13	8.89328	34
13	8.913743	161	8.913743	85	8.913743	12	8.913743	39
15	8.934206	150	8.934206	68	8.934206	11	8.934206	30
15	8.954668	139	8.954668	57	8.954668	12	8.954668	37
13	8.975131	137	8.975131	52	8.975131	12	8.975131	40
10	8.995594	135	8.995594	52	8.995594	13	8.995594	35
11	9.016057	119	9.016057	39	9.016057	13	9.016057	22
17	9.03652	114	9.03652	20	9.03652	13	9.03652	7
21	9.056982	103	9.056982	0	9.056982	13	9.056982	4
24	9.077445	93	9.077445	17	9.077445	12	9.077445	10
25	9.097908	70	9.097908	27	9.097908	11	9.097908	20
24	9.118371	72	9.118371	39	9.118371	10	9.118371	22
20	9.138833	57	9.138833	42	9.138833	10	9.138833	24
11	9.159296	60	9.159296	41	9.159296	10	9.159296	28
5	9.179759	66	9.179759	39	9.179759	10	9.179759	24
8	9.200222	63	9.200222	38	9.200222	11	9.200222	27
13	9.220685	48	9.220685	30	9.220685	11	9.220685	25
7	9.241147	51	9.241147	22	9.241147	10	9.241147	21
12	9.26161	42	9.26161	10	9.26161	10	9.26161	12
21	9.282073	50	9.282073	18	9.282073	9	9.282073	8
24	9.302536	45	9.302536	26	9.302536	7	9.302536	23
26	9.322998	44	9.322998	32	9.322998	6	9.322998	22
21	9.343461	31	9.343461	35	9.343461	5	9.343461	28
19	9.363924	37	9.363924	32	9.363924	4	9.363924	32
11	9.384387	40	9.384387	29	9.384387	3	9.384387	31
17	9.40485	30	9.40485	25	9.40485	3	9.40485	38
16	9.425312	20	9.425312	23	9.425312	3	9.425312	34
10	9.445775	14	9.445775	14	9.445775	3	9.445775	24
16	9.466238	2	9.466238	14	9.466238	3	9.466238	14
16	9.486701	5	9.486701	16	9.486701	3	9.486701	5
11	9.507163	6	9.507163	15	9.507163	4	9.507163	11
10	9.527626	10	9.527626	19	9.527626	7	9.527626	8
5	9.548089	11	9.548089	30	9.548089	10	9.548089	13
7	9.568552	13	9.568552	34	9.568552	13	9.568552	18
3	9.589015	20	9.589015	27	9.589015	17	9.589015	21
3	9.609477	19	9.609477	27	9.609477	21	9.609477	20
8	9.62994	24	9.62994	31	9.62994	25	9.62994	17
10	9.650403	24	9.650403	24	9.650403	27	9.650403	11
9	9.670866	21	9.670866	22	9.670866	27	9.670866	0
10	9.691328	21	9.691328	19	9.691328	26	9.691328	4
10	9.711791	22	9.711791	7	9.711791	25	9.711791	10
15	9.732254	17	9.732254	6	9.732254	23	9.732254	15
15	9.752717	11	9.752717	17	9.752717	21	9.752717	14
12	9.77318	2	9.77318	20	9.77318	21	9.77318	16
9	9.793642	0	9.793642	14	9.793642	21	9.793642	26
2	9.814105	0	9.814105	25	9.814105	21	9.814105	29
6	9.834568	8	9.834568	22	9.834568	22	9.834568	31
5	9.855031	8	9.855031	26	9.855031	22	9.855031	28
0	9.875493	14	9.875493	30	9.875493	21	9.875493	16
2	9.895956	17	9.895956	27	9.895956	19	9.895956	25
15	9.916419	18	9.916419	21	9.916419	16	9.916419	22
26	9.936882	17	9.936882	20	9.936882	13	9.936882	20

36	9.957345	13	9.957345	31	9.957345	10	9.957345	13
41	9.977807	9	9.977807	30	9.977807	8	9.977807	12
51	9.99827	3	9.99827	30	9.99827	7	9.99827	7
59	10.01873	1	10.01873	32	10.01873	8	10.01873	14
65	10.0392	2	10.0392	25	10.0392	9	10.0392	20
63	10.05966	2	10.05966	27	10.05966	11	10.05966	19
46	10.08012	9	10.08012	20	10.08012	13	10.08012	12
52	10.10058	6	10.10058	15	10.10058	15	10.10058	22
45	10.12105	16	10.12105	5	10.12105	16	10.12105	22
35	10.14151	24	10.14151	3	10.14151	17	10.14151	38
44	10.16197	28	10.16197	13	10.16197	17	10.16197	39
51	10.18244	29	10.18244	14	10.18244	15	10.18244	42
88	10.2029	29	10.2029	20	10.2029	14	10.2029	37
142	10.22336	21	10.22336	19	10.22336	12	10.22336	55
216	10.24382	21	10.24382	17	10.24382	10	10.24382	70
299	10.26429	20	10.26429	12	10.26429	9	10.26429	69
376	10.28475	17	10.28475	5	10.28475	8	10.28475	56
478	10.30521	8	10.30521	9	10.30521	9	10.30521	60
581	10.32568	14	10.32568	6	10.32568	10	10.32568	63
711	10.34614	11	10.34614	8	10.34614	13	10.34614	80
874	10.3666	6	10.3666	10	10.3666	15	10.3666	74
1048	10.38706	4	10.38706	9	10.38706	18	10.38706	76
1249	10.40753	5	10.40753	3	10.40753	20	10.40753	66
1467	10.42799	2	10.42799	5	10.42799	22	10.42799	70
1675	10.44845	3	10.44845	7	10.44845	23	10.44845	69
1855	10.46891	11	10.46891	8	10.46891	24	10.46891	52
1976	10.48938	16	10.48938	9	10.48938	25	10.48938	38
2047	10.50984	16	10.50984	12	10.50984	25	10.50984	50
2030	10.5303	18	10.5303	9	10.5303	26	10.5303	56
1938	10.55077	15	10.55077	11	10.55077	28	10.55077	58
1777	10.57123	13	10.57123	3	10.57123	30	10.57123	61
1551	10.59169	16	10.59169	10	10.59169	31	10.59169	61
1292	10.61215	18	10.61215	7	10.61215	33	10.61215	44
1027	10.63262	15	10.63262	27	10.63262	33	10.63262	47
777	10.65308	11	10.65308	36	10.65308	34	10.65308	37
572	10.67354	10	10.67354	40	10.67354	33	10.67354	35
404	10.69401	6	10.69401	41	10.69401	32	10.69401	35
291	10.71447	4	10.71447	36	10.71447	30	10.71447	38
221	10.73493	17	10.73493	26	10.73493	28	10.73493	26
168	10.75539	27	10.75539	15	10.75539	26	10.75539	22
139	10.77586	40	10.77586	3	10.77586	23	10.77586	23
120	10.79632	54	10.79632	8	10.79632	21	10.79632	15
106	10.81678	62	10.81678	1	10.81678	18	10.81678	5
81	10.83724	61	10.83724	9	10.83724	15	10.83724	23
65	10.85771	47	10.85771	8	10.85771	13	10.85771	25
45	10.87817	30	10.87817	7	10.87817	11	10.87817	32
35	10.89863	24	10.89863	7	10.89863	12	10.89863	32
31	10.9191	20	10.9191	6	10.9191	13	10.9191	24
36	10.93956	17	10.93956	0	10.93956	16	10.93956	26
18	10.96002	20	10.96002	0	10.96002	20	10.96002	38
14	10.98048	18	10.98048	3	10.98048	25	10.98048	42
23	11.00095	30	11.00095	15	11.00095	29	11.00095	46
24	11.02141	36	11.02141	24	11.02141	31	11.02141	53
18	11.04187	35	11.04187	32	11.04187	33	11.04187	58
13	11.06233	27	11.06233	47	11.06233	32	11.06233	44
10	11.0828	14	11.0828	59	11.0828	30	11.0828	41
14	11.10326	20	11.10326	58	11.10326	27	11.10326	32
11	11.12372	28	11.12372	53	11.12372	23	11.12372	34

15	11.14419	28	11.14419	46	11.14419	21	11.14419	41
11	11.16465	32	11.16465	38	11.16465	20	11.16465	39
15	11.18511	29	11.18511	35	11.18511	20	11.18511	48
25	11.20557	35	11.20557	33	11.20557	23	11.20557	55
22	11.22604	41	11.22604	21	11.22604	28	11.22604	59
30	11.2465	41	11.2465	16	11.2465	33	11.2465	57
31	11.26696	40	11.26696	18	11.26696	37	11.26696	45
34	11.28743	26	11.28743	18	11.28743	40	11.28743	42
27	11.30789	32	11.30789	24	11.30789	40	11.30789	35
24	11.32835	40	11.32835	33	11.32835	37	11.32835	33
22	11.34881	44	11.34881	39	11.34881	32	11.34881	21
15	11.36928	35	11.36928	47	11.36928	26	11.36928	9
9	11.38974	35	11.38974	59	11.38974	21	11.38974	12
10	11.4102	33	11.4102	68	11.4102	17	11.4102	22
4	11.43066	41	11.43066	68	11.43066	16	11.43066	28
19	11.45113	44	11.45113	61	11.45113	17	11.45113	39
23	11.47159	43	11.47159	45	11.47159	21	11.47159	40
28	11.49205	40	11.49205	43	11.49205	25	11.49205	39
27	11.51252	38	11.51252	42	11.51252	30	11.51252	40
24	11.53298	46	11.53298	32	11.53298	32	11.53298	36
15	11.55344	51	11.55344	16	11.55344	33	11.55344	30
12	11.5739	48	11.5739	25	11.5739	32	11.5739	33
4	11.59437	58	11.59437	31	11.59437	30	11.59437	38
5	11.61483	55	11.61483	32	11.61483	27	11.61483	40
0	11.63529	63	11.63529	36	11.63529	24	11.63529	47
0	11.65576	59	11.65576	36	11.65576	23	11.65576	48
3	11.67622	46	11.67622	36	11.67622	23	11.67622	57
16	11.69668	50	11.69668	37	11.69668	25	11.69668	63
22	11.71714	45	11.71714	35	11.71714	28	11.71714	60
25	11.73761	46	11.73761	32	11.73761	30	11.73761	55
34	11.75807	37	11.75807	21	11.75807	33	11.75807	48
41	11.77853	24	11.77853	31	11.77853	34	11.77853	44
38	11.79899	24	11.79899	24	11.79899	35	11.79899	51
31	11.81946	19	11.81946	18	11.81946	35	11.81946	53
24	11.83992	28	11.83992	25	11.83992	34	11.83992	63
21	11.86038	26	11.86038	24	11.86038	32	11.86038	56
20	11.88085	27	11.88085	25	11.88085	31	11.88085	78
21	11.90131	35	11.90131	20	11.90131	30	11.90131	93
25	11.92177	30	11.92177	20	11.92177	29	11.92177	88
29	11.94223	31	11.94223	16	11.94223	29	11.94223	81
55	11.9627	25	11.9627	6	11.9627	29	11.9627	70
59	11.98316	11	11.98316	5	11.98316	30	11.98316	60
58	12.00362	11	12.00362	0	12.00362	31	12.00362	70
68	12.02409	9	12.02409	0	12.02409	32	12.02409	69
86	12.04455	19	12.04455	6	12.04455	33	12.04455	76
108	12.06501	27	12.06501	10	12.06501	33	12.06501	70
144	12.08547	28	12.08547	15	12.08547	33	12.08547	80
195	12.10594	23	12.10594	17	12.10594	32	12.10594	73
255	12.1264	35	12.1264	15	12.1264	30	12.1264	63
328	12.14686	40	12.14686	14	12.14686	27	12.14686	47
433	12.16732	48	12.16732	11	12.16732	24	12.16732	48
561	12.18779	46	12.18779	7	12.18779	22	12.18779	65
706	12.20825	42	12.20825	4	12.20825	21	12.20825	76
881	12.22871	38	12.22871	0	12.22871	22	12.22871	90
1065	12.24918	39	12.24918	0	12.24918	23	12.24918	107
1236	12.26964	32	12.26964	0	12.26964	25	12.26964	124
1399	12.2901	17	12.2901	4	12.2901	27	12.2901	127
1487	12.31056	9	12.31056	11	12.31056	28	12.31056	116

1502	12.33103	15	12.33103	14	12.33103	28	12.33103	103
1473	12.35149	10	12.35149	18	12.35149	26	12.35149	80
1389	12.37195	15	12.37195	24	12.37195	24	12.37195	82
1268	12.39242	11	12.39242	21	12.39242	20	12.39242	99
1104	12.41288	7	12.41288	25	12.41288	18	12.41288	87
917	12.43334	4	12.43334	24	12.43334	16	12.43334	88
744	12.4538	7	12.4538	20	12.4538	16	12.4538	91
574	12.47427	6	12.47427	11	12.47427	17	12.47427	113
438	12.49473	8	12.49473	17	12.49473	20	12.49473	112
305	12.51519	8	12.51519	22	12.51519	23	12.51519	105
209	12.53565	12	12.53565	22	12.53565	26	12.53565	101
164	12.55612	15	12.55612	24	12.55612	28	12.55612	102
124	12.57658	17	12.57658	27	12.57658	29	12.57658	125
101	12.59704	15	12.59704	26	12.59704	28	12.59704	143
100	12.61751	13	12.61751	32	12.61751	27	12.61751	137
93	12.63797	6	12.63797	25	12.63797	26	12.63797	151
108	12.65843	10	12.65843	21	12.65843	25	12.65843	145
114	12.67889	9	12.67889	13	12.67889	24	12.67889	130
127	12.69936	8	12.69936	12	12.69936	23	12.69936	122
161	12.71982	5	12.71982	17	12.71982	23	12.71982	120
202	12.74028	5	12.74028	18	12.74028	23	12.74028	123
251	12.76075	0	12.76075	18	12.76075	22	12.76075	113
292	12.78121	4	12.78121	16	12.78121	21	12.78121	125
330	12.80167	13	12.80167	22	12.80167	19	12.80167	128
360	12.82213	18	12.82213	18	12.82213	18	12.82213	135
385	12.8426	17	12.8426	16	12.8426	18	12.8426	142
400	12.86306	28	12.86306	13	12.86306	20	12.86306	127
404	12.88352	30	12.88352	22	12.88352	23	12.88352	125
384	12.90398	32	12.90398	28	12.90398	28	12.90398	153
366	12.92445	28	12.92445	38	12.92445	35	12.92445	166
337	12.94491	19	12.94491	39	12.94491	43	12.94491	181
290	12.96537	10	12.96537	32	12.96537	52	12.96537	175
237	12.98584	16	12.98584	23	12.98584	60	12.98584	171
175	13.0063	17	13.0063	25	13.0063	66	13.0063	170
114	13.02676	15	13.02676	28	13.02676	70	13.02676	175
75	13.04722	12	13.04722	44	13.04722	71	13.04722	170
45	13.06769	13	13.06769	50	13.06769	70	13.06769	157
23	13.08815	10	13.08815	68	13.08815	67	13.08815	174
10	13.10861	19	13.10861	70	13.10861	63	13.10861	194
2	13.12908	15	13.12908	75	13.12908	59	13.12908	199
13	13.14954	25	13.14954	71	13.14954	58	13.14954	209
14	13.17	34	13.17	69	13.17	59	13.17	202
22	13.19046	34	13.19046	71	13.19046	62	13.19046	201
28	13.21093	36	13.21093	79	13.21093	68	13.21093	210
30	13.23139	33	13.23139	85	13.23139	74	13.23139	218
28	13.25185	27	13.25185	89	13.25185	80	13.25185	219
22	13.27231	24	13.27231	108	13.27231	84	13.27231	223
15	13.29278	18	13.29278	122	13.29278	87	13.29278	239
16	13.31324	28	13.31324	128	13.31324	88	13.31324	251
1	13.3337	20	13.3337	137	13.3337	87	13.3337	242
11	13.35417	27	13.35417	138	13.35417	86	13.35417	242
14	13.37463	31	13.37463	143	13.37463	86	13.37463	260
23	13.39509	39	13.39509	145	13.39509	87	13.39509	290
21	13.41555	47	13.41555	149	13.41555	91	13.41555	307
23	13.43602	55	13.43602	151	13.43602	98	13.43602	324
23	13.45648	60	13.45648	154	13.45648	105	13.45648	326
16	13.47694	64	13.47694	180	13.47694	113	13.47694	334
18	13.49741	61	13.49741	198	13.49741	120	13.49741	348

16	13.51787	69	13.51787	203	13.51787	126	13.51787	348
13	13.53833	66	13.53833	204	13.53833	130	13.53833	335
20	13.55879	62	13.55879	210	13.55879	132	13.55879	349
28	13.57926	72	13.57926	224	13.57926	134	13.57926	366
43	13.59972	86	13.59972	239	13.59972	137	13.59972	391
46	13.62018	95	13.62018	259	13.62018	142	13.62018	408
62	13.64064	115	13.64064	288	13.64064	149	13.64064	428
74	13.66111	120	13.66111	305	13.66111	158	13.66111	438
72	13.68157	125	13.68157	323	13.68157	168	13.68157	463
84	13.70203	133	13.70203	344	13.70203	180	13.70203	485
85	13.7225	140	13.7225	355	13.7225	190	13.7225	489
75	13.74296	139	13.74296	365	13.74296	200	13.74296	494
65	13.76342	138	13.76342	379	13.76342	208	13.76342	511
66	13.78388	152	13.78388	396	13.78388	215	13.78388	516
75	13.80435	164	13.80435	418	13.80435	220	13.80435	528
73	13.82481	176	13.82481	416	13.82481	226	13.82481	539
89	13.84527	187	13.84527	424	13.84527	231	13.84527	564
102	13.86574	190	13.86574	431	13.86574	237	13.86574	589
116	13.8862	192	13.8862	439	13.8862	244	13.8862	621
134	13.90666	194	13.90666	455	13.90666	252	13.90666	633
157	13.92712	197	13.92712	475	13.92712	259	13.92712	665
168	13.94759	200	13.94759	502	13.94759	267	13.94759	696
183	13.96805	212	13.96805	522	13.96805	276	13.96805	724
199	13.98851	230	13.98851	557	13.98851	285	13.98851	768
212	14.00897	242	14.00897	591	14.00897	296	14.00897	810
220	14.02944	263	14.02944	584	14.02944	308	14.02944	841
228	14.0499	286	14.0499	606	14.0499	321	14.0499	882
234	14.07036	305	14.07036	618	14.07036	335	14.07036	899
242	14.09083	317	14.09083	641	14.09083	351	14.09083	910
242	14.11129	334	14.11129	666	14.11129	367	14.11129	915
253	14.13175	345	14.13175	695	14.13175	383	14.13175	944
260	14.15221	348	14.15221	736	14.15221	399	14.15221	955
265	14.17268	368	14.17268	769	14.17268	415	14.17268	985
266	14.19314	365	14.19314	798	14.19314	430	14.19314	1029
283	14.2136	377	14.2136	809	14.2136	445	14.2136	1073
291	14.23407	399	14.23407	808	14.23407	460	14.23407	1095
295	14.25453	416	14.25453	833	14.25453	473	14.25453	1140
304	14.27499	435	14.27499	848	14.27499	487	14.27499	1173
318	14.29545	457	14.29545	873	14.29545	499	14.29545	1183
323	14.31592	490	14.31592	908	14.31592	511	14.31592	1210
343	14.33638	503	14.33638	939	14.33638	523	14.33638	1242
361	14.35684	517	14.35684	973	14.35684	535	14.35684	1253
385	14.3773	538	14.3773	1005	14.3773	548	14.3773	1280
404	14.39777	535	14.39777	1030	14.39777	561	14.39777	1303
423	14.41823	546	14.41823	1051	14.41823	576	14.41823	1355
426	14.43869	552	14.43869	1064	14.43869	591	14.43869	1379
431	14.45916	560	14.45916	1074	14.45916	607	14.45916	1408
439	14.47962	570	14.47962	1096	14.47962	623	14.47962	1429
442	14.50008	586	14.50008	1099	14.50008	638	14.50008	1420
441	14.52054	600	14.52054	1112	14.52054	651	14.52054	1433
449	14.54101	609	14.54101	1121	14.54101	663	14.54101	1442
470	14.56147	627	14.56147	1135	14.56147	674	14.56147	1452
488	14.58193	650	14.58193	1154	14.58193	683	14.58193	1463
498	14.6024	655	14.6024	1177	14.6024	692	14.6024	1480
500	14.62286	660	14.62286	1192	14.62286	701	14.62286	1519
508	14.64332	672	14.64332	1206	14.64332	711	14.64332	1542
544	14.66378	684	14.66378	1222	14.66378	721	14.66378	1545
581	14.68425	680	14.68425	1254	14.68425	732	14.68425	1557

610	14.70471	681	14.70471	1272	14.70471	742	14.70471	1575
672	14.72517	684	14.72517	1264	14.72517	750	14.72517	1595
748	14.74563	682	14.74563	1263	14.74563	755	14.74563	1620
844	14.7661	690	14.7661	1259	14.7661	758	14.7661	1629
934	14.78656	701	14.78656	1252	14.78656	758	14.78656	1622
1022	14.80702	696	14.80702	1237	14.80702	756	14.80702	1618
1110	14.82749	702	14.82749	1218	14.82749	753	14.82749	1608
1164	14.84795	704	14.84795	1204	14.84795	751	14.84795	1592
1196	14.86841	699	14.86841	1195	14.86841	749	14.86841	1546
1197	14.88887	679	14.88887	1197	14.88887	749	14.88887	1505
1160	14.90934	674	14.90934	1191	14.90934	751	14.90934	1500
1101	14.9298	680	14.9298	1165	14.9298	752	14.9298	1475
1034	14.95026	654	14.95026	1144	14.95026	754	14.95026	1461
930	14.97073	649	14.97073	1131	14.97073	753	14.97073	1440
837	14.99119	638	14.99119	1134	14.99119	750	14.99119	1448
754	15.01165	638	15.01165	1124	15.01165	743	15.01165	1439
687	15.03211	641	15.03211	1115	15.03211	734	15.03211	1433
620	15.05258	633	15.05258	1089	15.05258	721	15.05258	1415
553	15.07304	621	15.07304	1076	15.07304	707	15.07304	1395
518	15.0935	599	15.0935	1069	15.0935	692	15.0935	1378
489	15.11396	591	15.11396	1055	15.11396	678	15.11396	1364
472	15.13443	578	15.13443	1030	15.13443	664	15.13443	1321
470	15.15489	554	15.15489	999	15.15489	652	15.15489	1303
446	15.17535	546	15.17535	972	15.17535	643	15.17535	1280
434	15.19582	526	15.19582	958	15.19582	636	15.19582	1282
428	15.21628	513	15.21628	938	15.21628	631	15.21628	1262
420	15.23674	483	15.23674	921	15.23674	628	15.23674	1261
417	15.2572	470	15.2572	891	15.2572	625	15.2572	1233
391	15.27767	455	15.27767	878	15.27767	621	15.27767	1212
370	15.29813	440	15.29813	858	15.29813	615	15.29813	1194
348	15.31859	428	15.31859	852	15.31859	605	15.31859	1157
338	15.33906	420	15.33906	830	15.33906	593	15.33906	1130
324	15.35952	419	15.35952	817	15.35952	577	15.35952	1110
299	15.37998	423	15.37998	794	15.37998	559	15.37998	1086
280	15.40044	428	15.40044	780	15.40044	541	15.40044	1074
289	15.42091	426	15.42091	777	15.42091	524	15.42091	1080
284	15.44137	419	15.44137	768	15.44137	509	15.44137	1072
278	15.46183	407	15.46183	757	15.46183	496	15.46183	1041
278	15.48229	390	15.48229	749	15.48229	485	15.48229	1010
272	15.50276	387	15.50276	735	15.50276	475	15.50276	1005
261	15.52322	371	15.52322	752	15.52322	465	15.52322	988
259	15.54368	357	15.54368	726	15.54368	456	15.54368	991
254	15.56415	335	15.56415	713	15.56415	447	15.56415	975
252	15.58461	327	15.58461	697	15.58461	439	15.58461	965
249	15.60507	321	15.60507	694	15.60507	432	15.60507	940
245	15.62553	320	15.62553	673	15.62553	427	15.62553	937
232	15.646	314	15.646	666	15.646	423	15.646	912
242	15.66646	302	15.66646	661	15.66646	421	15.66646	892
258	15.68692	309	15.68692	658	15.68692	418	15.68692	859
261	15.70739	318	15.70739	656	15.70739	414	15.70739	850
251	15.72785	317	15.72785	667	15.72785	408	15.72785	837
249	15.74831	321	15.74831	671	15.74831	401	15.74831	830
253	15.76877	309	15.76877	670	15.76877	393	15.76877	819
258	15.78924	302	15.78924	669	15.78924	386	15.78924	819
243	15.8097	293	15.8097	663	15.8097	379	15.8097	814
225	15.83016	292	15.83016	649	15.83016	374	15.83016	823
232	15.85062	278	15.85062	659	15.85062	370	15.85062	842
240	15.87109	286	15.87109	663	15.87109	368	15.87109	861

240	15.89155	290	15.89155	661	15.89155	366	15.89155	849
240	15.91201	296	15.91201	654	15.91201	364	15.91201	843
240	15.93248	296	15.93248	665	15.93248	360	15.93248	843
253	15.95294	297	15.95294	672	15.95294	356	15.95294	837
257	15.9734	299	15.9734	655	15.9734	351	15.9734	821
265	15.99386	292	15.99386	657	15.99386	346	15.99386	838
295	16.01433	291	16.01433	661	16.01433	341	16.01433	858
316	16.03479	293	16.03479	671	16.03479	337	16.03479	865
346	16.05525	294	16.05525	685	16.05525	336	16.05525	890
374	16.07572	319	16.07572	692	16.07572	336	16.07572	894
397	16.09618	323	16.09618	700	16.09618	339	16.09618	898
425	16.11664	329	16.11664	708	16.11664	343	16.11664	888
456	16.1371	328	16.1371	716	16.1371	348	16.1371	889
492	16.15757	342	16.15757	713	16.15757	353	16.15757	872
509	16.17803	347	16.17803	726	16.17803	357	16.17803	841
541	16.19849	342	16.19849	756	16.19849	360	16.19849	867
585	16.21895	352	16.21895	764	16.21895	362	16.21895	868
582	16.23942	361	16.23942	764	16.23942	362	16.23942	863
579	16.25988	382	16.25988	762	16.25988	362	16.25988	887
571	16.28034	393	16.28034	763	16.28034	363	16.28034	898
528	16.30081	390	16.30081	762	16.30081	363	16.30081	931
489	16.32127	389	16.32127	748	16.32127	365	16.32127	946
452	16.34173	393	16.34173	740	16.34173	368	16.34173	952
430	16.36219	389	16.36219	740	16.36219	371	16.36219	955
410	16.38266	381	16.38266	741	16.38266	374	16.38266	942
395	16.40312	399	16.40312	756	16.40312	376	16.40312	946
385	16.42358	385	16.42358	745	16.42358	377	16.42358	935
365	16.44405	374	16.44405	718	16.44405	377	16.44405	924
370	16.46451	380	16.46451	705	16.46451	375	16.46451	909
377	16.48497	366	16.48497	707	16.48497	372	16.48497	904
375	16.50543	362	16.50543	698	16.50543	368	16.50543	917
371	16.5259	353	16.5259	678	16.5259	364	16.5259	922
367	16.54636	350	16.54636	674	16.54636	361	16.54636	913
367	16.56682	349	16.56682	680	16.56682	359	16.56682	902
369	16.58728	359	16.58728	683	16.58728	357	16.58728	880
354	16.60775	367	16.60775	696	16.60775	357	16.60775	877
348	16.62821	348	16.62821	677	16.62821	358	16.62821	865
340	16.64867	336	16.64867	657	16.64867	358	16.64867	853
344	16.66914	335	16.66914	651	16.66914	359	16.66914	828
338	16.6896	319	16.6896	634	16.6896	359	16.6896	826
335	16.71006	316	16.71006	615	16.71006	359	16.71006	804
310	16.73052	313	16.73052	589	16.73052	359	16.73052	773
290	16.75099	312	16.75099	578	16.75099	357	16.75099	741
267	16.77145	310	16.77145	569	16.77145	352	16.77145	715
249	16.79191	308	16.79191	561	16.79191	346	16.79191	707
231	16.81238	293	16.81238	551	16.81238	336	16.81238	709
214	16.83284	285	16.83284	530	16.83284	324	16.83284	697
199	16.8533	258	16.8533	522	16.8533	309	16.8533	678
193	16.87376	249	16.87376	498	16.87376	294	16.87376	649
184	16.89423	245	16.89423	477	16.89423	279	16.89423	630
184	16.91469	247	16.91469	463	16.91469	266	16.91469	595
177	16.93515	238	16.93515	449	16.93515	257	16.93515	559
170	16.95561	225	16.95561	441	16.95561	250	16.95561	535
157	16.97608	218	16.97608	422	16.97608	245	16.97608	520
163	16.99654	211	16.99654	408	16.99654	242	16.99654	522
164	17.017	192	17.017	394	17.017	239	17.017	514
157	17.03747	190	17.03747	377	17.03747	234	17.03747	513
155	17.05793	170	17.05793	357	17.05793	227	17.05793	476

148	17.07839	157	17.07839	331	17.07839	218	17.07839	455
141	17.09885	148	17.09885	314	17.09885	206	17.09885	430
131	17.11932	150	17.11932	297	17.11932	192	17.11932	396
120	17.13978	131	17.13978	273	17.13978	177	17.13978	352
102	17.16024	112	17.16024	251	17.16024	163	17.16024	328
88	17.18071	114	17.18071	236	17.18071	150	17.18071	302
97	17.20117	98	17.20117	234	17.20117	139	17.20117	291
96	17.22163	85	17.22163	226	17.22163	130	17.22163	292
95	17.24209	95	17.24209	229	17.24209	124	17.24209	307
86	17.26256	84	17.26256	212	17.26256	120	17.26256	285
74	17.28302	65	17.28302	210	17.28302	117	17.28302	275
61	17.30348	54	17.30348	183	17.30348	115	17.30348	242
49	17.32394	63	17.32394	170	17.32394	113	17.32394	214
46	17.34441	58	17.34441	149	17.34441	109	17.34441	191
35	17.36487	52	17.36487	129	17.36487	104	17.36487	172
29	17.38533	55	17.38533	134	17.38533	96	17.38533	150
32	17.4058	37	17.4058	137	17.4058	87	17.4058	140
39	17.42626	31	17.42626	131	17.42626	78	17.42626	160
38	17.44672	34	17.44672	140	17.44672	71	17.44672	163
30	17.46718	22	17.46718	137	17.46718	65	17.46718	144
23	17.48765	13	17.48765	127	17.48765	62	17.48765	137
9	17.50811	11	17.50811	122	17.50811	63	17.50811	121
9	17.52857	13	17.52857	127	17.52857	65	17.52857	117
14	17.54904	15	17.54904	113	17.54904	68	17.54904	108
20	17.5695	9	17.5695	113	17.5695	71	17.5695	96
26	17.58996	10	17.58996	117	17.58996	72	17.58996	94
29	17.61042	11	17.61042	108	17.61042	70	17.61042	92
34	17.63089	9	17.63089	94	17.63089	66	17.63089	93
30	17.65135	18	17.65135	88	17.65135	59	17.65135	81
27	17.67181	26	17.67181	76	17.67181	52	17.67181	74
19	17.69227	32	17.69227	65	17.69227	44	17.69227	71
7	17.71274	33	17.71274	69	17.71274	38	17.71274	50
5	17.7332	29	17.7332	61	17.7332	33	17.7332	46
6	17.75366	21	17.75366	59	17.75366	31	17.75366	37
10	17.77413	11	17.77413	72	17.77413	30	17.77413	30
6	17.79459	5	17.79459	71	17.79459	31	17.79459	29
8	17.81505	2	17.81505	65	17.81505	33	17.81505	20
7	17.83551	0	17.83551	56	17.83551	34	17.83551	5
8	17.85598	5	17.85598	57	17.85598	36	17.85598	17
8	17.87644	5	17.87644	47	17.87644	37	17.87644	24
12	17.8969	9	17.8969	49	17.8969	38	17.8969	25
14	17.91737	11	17.91737	39	17.91737	39	17.91737	30
23	17.93783	14	17.93783	35	17.93783	39	17.93783	48
38	17.95829	14	17.95829	46	17.95829	40	17.95829	60
38	17.97875	8	17.97875	47	17.97875	41	17.97875	60
43	17.99922	11	17.99922	47	17.99922	41	17.99922	57
57	18.01968	11	18.01968	50	18.01968	39	18.01968	50
86	18.04014	12	18.04014	52	18.04014	37	18.04014	35
125	18.0606	13	18.0606	59	18.0606	33	18.0606	38
172	18.08107	5	18.08107	54	18.08107	29	18.08107	27
237	18.10153	12	18.10153	59	18.10153	24	18.10153	23
309	18.12199	12	18.12199	55	18.12199	20	18.12199	24
395	18.14246	14	18.14246	48	18.14246	16	18.14246	22
477	18.16292	16	18.16292	44	18.16292	14	18.16292	19
552	18.18338	15	18.18338	24	18.18338	13	18.18338	16
613	18.20384	17	18.20384	22	18.20384	13	18.20384	9
637	18.22431	12	18.22431	16	18.22431	15	18.22431	13
649	18.24477	19	18.24477	18	18.24477	16	18.24477	13

613	18.26523	19	18.26523	22	18.26523	17	18.26523	25
558	18.2857	17	18.2857	22	18.2857	17	18.2857	29
483	18.30616	28	18.30616	23	18.30616	17	18.30616	28
393	18.32662	26	18.32662	33	18.32662	16	18.32662	31
300	18.34708	33	18.34708	35	18.34708	14	18.34708	29
219	18.36755	33	18.36755	35	18.36755	13	18.36755	21
161	18.38801	27	18.38801	26	18.38801	12	18.38801	24
110	18.40847	29	18.40847	31	18.40847	12	18.40847	18
70	18.42893	26	18.42893	23	18.42893	13	18.42893	18
52	18.4494	28	18.4494	25	18.4494	15	18.4494	17
29	18.46986	31	18.46986	23	18.46986	19	18.46986	17
22	18.49032	31	18.49032	23	18.49032	22	18.49032	13
15	18.51079	33	18.51079	25	18.51079	26	18.51079	6
14	18.53125	26	18.53125	28	18.53125	28	18.53125	6
13	18.55171	36	18.55171	36	18.55171	29	18.55171	4
11	18.57217	38	18.57217	46	18.57217	28	18.57217	1
8	18.59264	43	18.59264	46	18.59264	27	18.59264	5
10	18.6131	52	18.6131	36	18.6131	25	18.6131	13
11	18.63356	48	18.63356	37	18.63356	23	18.63356	23
7	18.65403	41	18.65403	35	18.65403	22	18.65403	29
6	18.67449	32	18.67449	36	18.67449	21	18.67449	31
4	18.69495	16	18.69495	33	18.69495	21	18.69495	26
3	18.71541	16	18.71541	25	18.71541	22	18.71541	28
3	18.73588	19	18.73588	20	18.73588	22	18.73588	23
5	18.75634	30	18.75634	32	18.75634	21	18.75634	21
10	18.7768	36	18.7768	34	18.7768	20	18.7768	25
21	18.79726	52	18.79726	28	18.79726	19	18.79726	26
26	18.81773	55	18.81773	13	18.81773	18	18.81773	31
28	18.83819	59	18.83819	25	18.83819	17	18.83819	31
24	18.85865	52	18.85865	23	18.85865	17	18.85865	30
38	18.87912	35	18.87912	19	18.87912	19	18.87912	28
40	18.89958	26	18.89958	15	18.89958	21	18.89958	18
36	18.92004	31	18.92004	14	18.92004	24	18.92004	19
32	18.9405	24	18.9405	18	18.9405	27	18.9405	9
25	18.96097	17	18.96097	18	18.96097	29	18.96097	14
25	18.98143	14	18.98143	14	18.98143	30	18.98143	28
32	19.00189	20	19.00189	8	19.00189	30	19.00189	31
26	19.02235	23	19.02235	0	19.02235	29	19.02235	41
35	19.04282	29	19.04282	4	19.04282	26	19.04282	45
41	19.06328	22	19.06328	2	19.06328	23	19.06328	43
64	19.08374	26	19.08374	0	19.08374	20	19.08374	50
75	19.10421	29	19.10421	5	19.10421	17	19.10421	42
90	19.12467	30	19.12467	16	19.12467	14	19.12467	37
119	19.14513	24	19.14513	22	19.14513	11	19.14513	26
139	19.16559	17	19.16559	20	19.16559	9	19.16559	28
185	19.18606	13	19.18606	29	19.18606	8	19.18606	31
264	19.20652	9	19.20652	35	19.20652	7	19.20652	26
363	19.22698	7	19.22698	33	19.22698	7	19.22698	43
500	19.24745	11	19.24745	35	19.24745	8	19.24745	47
651	19.26791	7	19.26791	28	19.26791	8	19.26791	38
809	19.28837	24	19.28837	19	19.28837	8	19.28837	47
932	19.30883	31	19.30883	10	19.30883	8	19.30883	43
1021	19.3293	38	19.3293	16	19.3293	7	19.3293	40
1064	19.34976	32	19.34976	11	19.34976	6	19.34976	30
1042	19.37022	39	19.37022	10	19.37022	5	19.37022	21
979	19.39068	41	19.39068	18	19.39068	4	19.39068	12
874	19.41115	34	19.41115	19	19.41115	3	19.41115	2
731	19.43161	30	19.43161	22	19.43161	4	19.43161	9

575	19.45207	31	19.45207	22	19.45207	4	19.45207	11
442	19.47254	32	19.47254	20	19.47254	5	19.47254	12
331	19.493	41	19.493	15	19.493	7	19.493	26
226	19.51346	35	19.51346	7	19.51346	8	19.51346	30
156	19.53392	32	19.53392	9	19.53392	9	19.53392	33
114	19.55439	30	19.55439	9	19.55439	9	19.55439	25
88	19.57485	39	19.57485	11	19.57485	10	19.57485	31
74	19.59531	34	19.59531	11	19.59531	9	19.59531	32
54	19.61578	31	19.61578	6	19.61578	9	19.61578	32
34	19.63624	30	19.63624	13	19.63624	8	19.63624	26
12	19.6567	24	19.6567	15	19.6567	7	19.6567	24
8	19.67716	26	19.67716	13	19.67716	6	19.67716	27
5	19.69763	23	19.69763	20	19.69763	6	19.69763	23
3	19.71809	16	19.71809	22	19.71809	5	19.71809	16
6	19.73855	11	19.73855	20	19.73855	4	19.73855	20
11	19.75901	19	19.75901	28	19.75901	4	19.75901	18
14	19.77948	18	19.77948	25	19.77948	3	19.77948	27
15	19.79994	22	19.79994	21	19.79994	3	19.79994	33
12	19.8204	28	19.8204	23	19.8204	3	19.8204	32
13	19.84087	26	19.84087	26	19.84087	3	19.84087	29
10	19.86133	19	19.86133	25	19.86133	4	19.86133	28
7	19.88179	13	19.88179	15	19.88179	4	19.88179	24
2	19.90225	9	19.90225	23	19.90225	5	19.90225	9
6	19.92272	11	19.92272	26	19.92272	6	19.92272	6
2	19.94318	10	19.94318	23	19.94318	7	19.94318	11
7	19.96364	14	19.96364	28	19.96364	8	19.96364	18
13	19.98411	14	19.98411	26	19.98411	9	19.98411	25
13	20.00457	24	20.00457	26	20.00457	10	20.00457	37
15	20.02503	24	20.02503	37	20.02503	11	20.02503	37
16	20.04549	27	20.04549	38	20.04549	12	20.04549	40
14	20.06596	25	20.06596	34	20.06596	12	20.06596	36
8	20.08642	26	20.08642	29	20.08642	12	20.08642	21
6	20.10688	24	20.10688	31	20.10688	12	20.10688	10
4	20.12734	27	20.12734	27	20.12734	10	20.12734	15
4	20.14781	27	20.14781	19	20.14781	9	20.14781	20
11	20.16827	32	20.16827	18	20.16827	7	20.16827	25
12	20.18873	37	20.18873	15	20.18873	6	20.18873	29
11	20.2092	42	20.2092	9	20.2092	5	20.2092	34
15	20.22966	36	20.22966	13	20.22966	6	20.22966	38
15	20.25012	33	20.25012	15	20.25012	7	20.25012	39
13	20.27058	22	20.27058	26	20.27058	9	20.27058	31
9	20.29105	20	20.29105	34	20.29105	12	20.29105	15
5	20.31151	5	20.31151	38	20.31151	15	20.31151	19
2	20.33197	4	20.33197	34	20.33197	17	20.33197	27
6	20.35244	7	20.35244	38	20.35244	19	20.35244	25
4	20.3729	22	20.3729	40	20.3729	20	20.3729	12
5	20.39336	31	20.39336	36	20.39336	20	20.39336	15
0	20.41382	35	20.41382	26	20.41382	19	20.41382	19
8	20.43429	32	20.43429	29	20.43429	18	20.43429	22
14	20.45475	28	20.45475	30	20.45475	18	20.45475	30
17	20.47521	21	20.47521	20	20.47521	17	20.47521	32
15	20.49567	15	20.49567	17	20.49567	17	20.49567	25
17	20.51614	14	20.51614	31	20.51614	17	20.51614	30
21	20.5366	19	20.5366	38	20.5366	16	20.5366	30
19	20.55706	14	20.55706	67	20.55706	15	20.55706	29
15	20.57753	24	20.57753	82	20.57753	15	20.57753	24
16	20.59799	27	20.59799	88	20.59799	16	20.59799	38
8	20.61845	28	20.61845	89	20.61845	17	20.61845	36

23	20.63891	24	20.63891	86	20.63891	20	20.63891	41
29	20.65938	17	20.65938	65	20.65938	25	20.65938	52
27	20.67984	13	20.67984	41	20.67984	30	20.67984	50
21	20.7003	10	20.7003	44	20.7003	35	20.7003	40
27	20.72077	14	20.72077	48	20.72077	39	20.72077	31
29	20.74123	8	20.74123	52	20.74123	40	20.74123	17
39	20.76169	3	20.76169	73	20.76169	39	20.76169	17
56	20.78215	16	20.78215	85	20.78215	37	20.78215	29
66	20.80262	27	20.80262	104	20.80262	32	20.80262	53
73	20.82308	42	20.82308	115	20.82308	28	20.82308	55
78	20.84354	46	20.84354	135	20.84354	24	20.84354	66
88	20.864	44	20.864	146	20.864	22	20.864	79
89	20.88447	54	20.88447	160	20.88447	21	20.88447	82
92	20.90493	53	20.90493	169	20.90493	21	20.90493	86
103	20.92539	38	20.92539	153	20.92539	21	20.92539	79
120	20.94586	28	20.94586	144	20.94586	22	20.94586	77
152	20.96632	17	20.96632	128	20.96632	21	20.96632	94
188	20.98678	32	20.98678	133	20.98678	21	20.98678	104
218	21.00724	36	21.00724	143	21.00724	20	21.00724	114
257	21.02771	41	21.02771	147	21.02771	20	21.02771	113
277	21.04817	43	21.04817	183	21.04817	21	21.04817	117
309	21.06863	39	21.06863	221	21.06863	23	21.06863	120
317	21.0891	56	21.0891	248	21.0891	26	21.0891	121
317	21.10956	55	21.10956	268	21.10956	29	21.10956	132
318	21.13002	58	21.13002	256	21.13002	31	21.13002	134
301	21.15048	57	21.15048	254	21.15048	32	21.15048	152
270	21.17095	59	21.17095	247	21.17095	32	21.17095	177
231	21.19141	72	21.19141	255	21.19141	30	21.19141	183
200	21.21187	72	21.21187	245	21.21187	27	21.21187	193
188	21.23233	69	21.23233	236	21.23233	23	21.23233	213
167	21.2528	76	21.2528	251	21.2528	20	21.2528	242
158	21.27326	78	21.27326	273	21.27326	16	21.27326	254
163	21.29372	84	21.29372	276	21.29372	15	21.29372	261
169	21.31419	71	21.31419	278	21.31419	14	21.31419	285
186	21.33465	76	21.33465	282	21.33465	16	21.33465	292
202	21.35511	71	21.35511	287	21.35511	19	21.35511	296
203	21.37557	70	21.37557	299	21.37557	23	21.37557	298
204	21.39604	82	21.39604	319	21.39604	27	21.39604	290
222	21.4165	96	21.4165	335	21.4165	32	21.4165	282
245	21.43696	117	21.43696	346	21.43696	36	21.43696	297
264	21.45743	139	21.45743	370	21.45743	39	21.45743	327
277	21.47789	149	21.47789	393	21.47789	42	21.47789	355
295	21.49835	156	21.49835	413	21.49835	44	21.49835	380
300	21.51881	159	21.51881	420	21.51881	46	21.51881	430
315	21.53928	169	21.53928	438	21.53928	50	21.53928	448
327	21.55974	170	21.55974	445	21.55974	54	21.55974	470
331	21.5802	169	21.5802	459	21.5802	61	21.5802	476
346	21.60066	189	21.60066	478	21.60066	68	21.60066	495
376	21.62113	199	21.62113	500	21.62113	76	21.62113	496
403	21.64159	213	21.64159	513	21.64159	83	21.64159	504
439	21.66205	212	21.66205	532	21.66205	89	21.66205	523
462	21.68252	219	21.68252	562	21.68252	94	21.68252	545
481	21.70298	233	21.70298	594	21.70298	97	21.70298	574
500	21.72344	242	21.72344	616	21.72344	99	21.72344	620
522	21.7439	239	21.7439	642	21.7439	101	21.7439	666
557	21.76437	229	21.76437	678	21.76437	103	21.76437	691
599	21.78483	234	21.78483	725	21.78483	106	21.78483	728
647	21.80529	260	21.80529	785	21.80529	108	21.80529	780

719	21.82576	282	21.82576	841	21.82576	110	21.82576	820
809	21.84622	296	21.84622	874	21.84622	111	21.84622	863
919	21.86668	324	21.86668	901	21.86668	110	21.86668	896
1024	21.88714	367	21.88714	928	21.88714	109	21.88714	930
1168	21.90761	402	21.90761	946	21.90761	107	21.90761	977
1327	21.92807	431	21.92807	967	21.92807	107	21.92807	1009
1538	21.94853	447	21.94853	977	21.94853	111	21.94853	1037
1796	21.96899	458	21.96899	1000	21.96899	118	21.96899	1051
2038	21.98946	473	21.98946	1048	21.98946	129	21.98946	1085
2276	22.00992	492	22.00992	1089	22.00992	144	22.00992	1114
2473	22.03038	511	22.03038	1120	22.03038	160	22.03038	1170
2609	22.05085	527	22.05085	1173	22.05085	176	22.05085	1237
2661	22.07131	564	22.07131	1245	22.07131	189	22.07131	1300
2613	22.09177	604	22.09177	1306	22.09177	199	22.09177	1383
2515	22.11223	633	22.11223	1380	22.11223	206	22.11223	1442
2357	22.1327	673	22.1327	1451	22.1327	210	22.1327	1500
2256	22.15316	709	22.15316	1517	22.15316	213	22.15316	1572
2189	22.17362	719	22.17362	1570	22.17362	217	22.17362	1633
2183	22.19409	719	22.19409	1636	22.19409	223	22.19409	1681
2290	22.21455	737	22.21455	1679	22.21455	233	22.21455	1713
2494	22.23501	757	22.23501	1729	22.23501	247	22.23501	1788
2810	22.25547	773	22.25547	1797	22.25547	263	22.25547	1857
3214	22.27594	811	22.27594	1864	22.27594	281	22.27594	1918
3637	22.2964	853	22.2964	1943	22.2964	299	22.2964	1997
4027	22.31686	880	22.31686	2029	22.31686	316	22.31686	2046
4404	22.33732	909	22.33732	2106	22.33732	330	22.33732	2119
4706	22.35779	938	22.35779	2178	22.35779	342	22.35779	2202
4835	22.37825	947	22.37825	2232	22.37825	352	22.37825	2243
4772	22.39871	962	22.39871	2293	22.39871	359	22.39871	2280
4559	22.41918	992	22.41918	2343	22.41918	367	22.41918	2299
4224	22.43964	1013	22.43964	2357	22.43964	374	22.43964	2361
3825	22.4601	1023	22.4601	2379	22.4601	382	22.4601	2406
3395	22.48056	1052	22.48056	2409	22.48056	392	22.48056	2463
2987	22.50103	1097	22.50103	2470	22.50103	402	22.50103	2518
2629	22.52149	1122	22.52149	2515	22.52149	413	22.52149	2579
2397	22.54195	1151	22.54195	2587	22.54195	425	22.54195	2621
2237	22.56242	1179	22.56242	2642	22.56242	436	22.56242	2655
2133	22.58288	1210	22.58288	2691	22.58288	447	22.58288	2689
2043	22.60334	1220	22.60334	2726	22.60334	458	22.60334	2722
1989	22.6238	1230	22.6238	2744	22.6238	468	22.6238	2741
1948	22.64427	1236	22.64427	2713	22.64427	478	22.64427	2764
1927	22.66473	1230	22.66473	2709	22.66473	486	22.66473	2767
1908	22.68519	1233	22.68519	2698	22.68519	492	22.68519	2789
1901	22.70565	1237	22.70565	2704	22.70565	496	22.70565	2786
1880	22.72612	1234	22.72612	2693	22.72612	497	22.72612	2771
1867	22.74658	1228	22.74658	2705	22.74658	495	22.74658	2725
1850	22.76704	1221	22.76704	2694	22.76704	490	22.76704	2707
1814	22.78751	1219	22.78751	2687	22.78751	483	22.78751	2717
1765	22.80797	1190	22.80797	2674	22.80797	474	22.80797	2721
1706	22.82843	1191	22.82843	2646	22.82843	465	22.82843	2711
1651	22.84889	1182	22.84889	2588	22.84889	455	22.84889	2700
1607	22.86936	1176	22.86936	2543	22.86936	447	22.86936	2674
1585	22.88982	1175	22.88982	2499	22.88982	439	22.88982	2644
1559	22.91028	1163	22.91028	2447	22.91028	433	22.91028	2590
1536	22.93075	1147	22.93075	2410	22.93075	428	22.93075	2518
1528	22.95121	1124	22.95121	2349	22.95121	423	22.95121	2443
1496	22.97167	1089	22.97167	2288	22.97167	419	22.97167	2371
1458	22.99213	1055	22.99213	2211	22.99213	414	22.99213	2292

1431	23.0126	1020	23.0126	2166	23.0126	407	23.0126	2224
1380	23.03306	988	23.03306	2111	23.03306	399	23.03306	2158
1329	23.05352	946	23.05352	2034	23.05352	387	23.05352	2101
1286	23.07398	912	23.07398	1978	23.07398	374	23.07398	2042
1237	23.09445	893	23.09445	1919	23.09445	358	23.09445	1972
1189	23.11491	874	23.11491	1847	23.11491	341	23.11491	1911
1143	23.13537	846	23.13537	1804	23.13537	323	23.13537	1850
1109	23.15584	834	23.15584	1731	23.15584	305	23.15584	1777
1063	23.1763	808	23.1763	1668	23.1763	288	23.1763	1674
1020	23.19676	775	23.19676	1574	23.19676	273	23.19676	1580
993	23.21722	741	23.21722	1516	23.21722	259	23.21722	1501
945	23.23769	709	23.23769	1446	23.23769	248	23.23769	1442
913	23.25815	671	23.25815	1368	23.25815	237	23.25815	1376
878	23.27861	627	23.27861	1294	23.27861	228	23.27861	1325
832	23.29908	600	23.29908	1225	23.29908	219	23.29908	1270
801	23.31954	572	23.31954	1150	23.31954	211	23.31954	1230
760	23.34	557	23.34	1080	23.34	203	23.34	1182
725	23.36046	536	23.36046	1018	23.36046	195	23.36046	1129
680	23.38093	521	23.38093	964	23.38093	186	23.38093	1039
637	23.40139	477	23.40139	902	23.40139	177	23.40139	973
602	23.42185	464	23.42185	866	23.42185	168	23.42185	908
571	23.44231	442	23.44231	838	23.44231	158	23.44231	869
560	23.46278	407	23.46278	805	23.46278	147	23.46278	812
551	23.48324	369	23.48324	761	23.48324	136	23.48324	781
538	23.5037	348	23.5037	744	23.5037	125	23.5037	740
525	23.52417	331	23.52417	706	23.52417	115	23.52417	714
527	23.54463	324	23.54463	675	23.54463	104	23.54463	688
519	23.56509	304	23.56509	637	23.56509	95	23.56509	665
506	23.58555	294	23.58555	611	23.58555	86	23.58555	619
476	23.60602	281	23.60602	579	23.60602	78	23.60602	570
446	23.62648	286	23.62648	534	23.62648	70	23.62648	529
419	23.64694	265	23.64694	506	23.64694	62	23.64694	495
401	23.66741	255	23.66741	473	23.66741	55	23.66741	470
378	23.68787	236	23.68787	447	23.68787	49	23.68787	446
347	23.70833	220	23.70833	428	23.70833	43	23.70833	423
319	23.72879	210	23.72879	409	23.72879	37	23.72879	399
312	23.74926	197	23.74926	401	23.74926	32	23.74926	375
291	23.76972	179	23.76972	394	23.76972	27	23.76972	353
276	23.79018	166	23.79018	386	23.79018	23	23.79018	318
251	23.81064	160	23.81064	366	23.81064	21	23.81064	301
222	23.83111	150	23.83111	343	23.83111	19	23.83111	289
197	23.85157	154	23.85157	337	23.85157	19	23.85157	266
184	23.87203	152	23.87203	325	23.87203	19	23.87203	260
178	23.8925	148	23.8925	309	23.8925	20	23.8925	256
173	23.91296	141	23.91296	300	23.91296	20	23.91296	236
163	23.93342	123	23.93342	298	23.93342	21	23.93342	212
159	23.95388	107	23.95388	298	23.95388	20	23.95388	180
158	23.97435	95	23.97435	300	23.97435	19	23.97435	152
161	23.99481	85	23.99481	305	23.99481	18	23.99481	140
160	24.01527	82	24.01527	313	24.01527	17	24.01527	152
147	24.03574	81	24.03574	310	24.03574	17	24.03574	157
148	24.0562	88	24.0562	289	24.0562	17	24.0562	163
159	24.07666	72	24.07666	256	24.07666	18	24.07666	169
172	24.09712	64	24.09712	230	24.09712	20	24.09712	171
176	24.11759	57	24.11759	218	24.11759	23	24.11759	149
183	24.13805	37	24.13805	218	24.13805	25	24.13805	148
192	24.15851	43	24.15851	207	24.15851	26	24.15851	115
209	24.17897	51	24.17897	204	24.17897	25	24.17897	96

227	24.19944	63	24.19944	205	24.19944	23	24.19944	82
257	24.2199	77	24.2199	204	24.2199	20	24.2199	77
303	24.24036	82	24.24036	197	24.24036	16	24.24036	75
359	24.26083	91	24.26083	181	24.26083	12	24.26083	77
429	24.28129	93	24.28129	174	24.28129	9	24.28129	76
525	24.30175	88	24.30175	182	24.30175	5	24.30175	80
644	24.32221	79	24.32221	192	24.32221	3	24.32221	84
817	24.34268	69	24.34268	203	24.34268	2	24.34268	95
1034	24.36314	65	24.36314	194	24.36314	2	24.36314	85
1256	24.3836	50	24.3836	193	24.3836	3	24.3836	79
1482	24.40407	38	24.40407	196	24.40407	4	24.40407	61
1667	24.42453	28	24.42453	201	24.42453	5	24.42453	58
1808	24.44499	29	24.44499	187	24.44499	6	24.44499	50
1847	24.46545	31	24.46545	185	24.46545	7	24.46545	47
1805	24.48592	41	24.48592	185	24.48592	7	24.48592	44
1675	24.50638	47	24.50638	187	24.50638	7	24.50638	47
1474	24.52684	47	24.52684	175	24.52684	7	24.52684	45
1251	24.5473	52	24.5473	166	24.5473	7	24.5473	37
1018	24.56777	47	24.56777	146	24.56777	8	24.56777	29
797	24.58823	39	24.58823	144	24.58823	9	24.58823	18
608	24.60869	43	24.60869	152	24.60869	11	24.60869	15
446	24.62916	56	24.62916	142	24.62916	14	24.62916	18
344	24.64962	57	24.64962	134	24.64962	17	24.64962	21
253	24.67008	54	24.67008	148	24.67008	20	24.67008	24
200	24.69054	59	24.69054	165	24.69054	23	24.69054	24
163	24.71101	65	24.71101	171	24.71101	26	24.71101	30
130	24.73147	62	24.73147	149	24.73147	27	24.73147	36
107	24.75193	56	24.75193	144	24.75193	28	24.75193	37
90	24.7724	43	24.7724	125	24.7724	27	24.7724	44
84	24.79286	33	24.79286	116	24.79286	26	24.79286	40
65	24.81332	30	24.81332	101	24.81332	23	24.81332	42
47	24.83378	33	24.83378	82	24.83378	21	24.83378	38
40	24.85425	27	24.85425	73	24.85425	18	24.85425	27
28	24.87471	27	24.87471	76	24.87471	16	24.87471	14
24	24.89517	20	24.89517	90	24.89517	14	24.89517	11
25	24.91563	29	24.91563	92	24.91563	13	24.91563	15
22	24.9361	33	24.9361	76	24.9361	12	24.9361	20
25	24.95656	33	24.95656	72	24.95656	11	24.95656	20
30	24.97702	31	24.97702	67	24.97702	10	24.97702	22
24	24.99749	21	24.99749	49	24.99749	10	24.99749	17
16	25.01795	15	25.01795	44	25.01795	10	25.01795	21
10	25.03841	16	25.03841	44	25.03841	11	25.03841	21
12	25.05887	24	25.05887	36	25.05887	12	25.05887	25
10	25.07934	33	25.07934	44	25.07934	13	25.07934	30
12	25.0998	29	25.0998	49	25.0998	15	25.0998	31
15	25.12026	42	25.12026	47	25.12026	16	25.12026	35
17	25.14073	54	25.14073	34	25.14073	17	25.14073	34
17	25.16119	62	25.16119	27	25.16119	18	25.16119	26
16	25.18165	58	25.18165	37	25.18165	18	25.18165	21
9	25.20211	48	25.20211	43	25.20211	19	25.20211	13
7	25.22258	39	25.22258	55	25.22258	20	25.22258	10
10	25.24304	35	25.24304	60	25.24304	23	25.24304	12
9	25.2635	33	25.2635	60	25.2635	27	25.2635	13
3	25.28396	28	25.28396	64	25.28396	32	25.28396	17
9	25.30443	13	25.30443	48	25.30443	37	25.30443	16
12	25.32489	12	25.32489	37	25.32489	42	25.32489	15
13	25.34535	17	25.34535	29	25.34535	45	25.34535	22
13	25.36582	28	25.36582	28	25.36582	46	25.36582	18

7	25.38628	31	25.38628	38	25.38628	45	25.38628	26
6	25.40674	33	25.40674	54	25.40674	42	25.40674	28
13	25.4272	35	25.4272	58	25.4272	37	25.4272	26
19	25.44767	41	25.44767	61	25.44767	32	25.44767	25
23	25.46813	37	25.46813	59	25.46813	27	25.46813	16
22	25.48859	31	25.48859	51	25.48859	22	25.48859	19
24	25.50906	19	25.50906	35	25.50906	19	25.50906	15
22	25.52952	13	25.52952	24	25.52952	16	25.52952	7
16	25.54998	14	25.54998	14	25.54998	15	25.54998	4
6	25.57044	8	25.57044	9	25.57044	15	25.57044	8
4	25.59091	14	25.59091	4	25.59091	16	25.59091	26
8	25.61137	20	25.61137	16	25.61137	18	25.61137	34
31	25.63183	27	25.63183	18	25.63183	21	25.63183	37
54	25.65229	38	25.65229	28	25.65229	24	25.65229	38
85	25.67276	38	25.67276	31	25.67276	27	25.67276	30
147	25.69322	39	25.69322	37	25.69322	30	25.69322	28
216	25.71368	32	25.71368	39	25.71368	32	25.71368	20
311	25.73415	20	25.73415	28	25.73415	34	25.73415	6
424	25.75461	19	25.75461	26	25.75461	36	25.75461	0
576	25.77507	19	25.77507	39	25.77507	37	25.77507	0
741	25.79553	25	25.79553	37	25.79553	37	25.79553	6
901	25.816	42	25.816	44	25.816	38	25.816	7
1038	25.83646	51	25.83646	41	25.83646	37	25.83646	9
1107	25.85692	52	25.85692	47	25.85692	35	25.85692	11
1129	25.87739	50	25.87739	45	25.87739	34	25.87739	8
1097	25.89785	46	25.89785	50	25.89785	32	25.89785	14
988	25.91831	27	25.91831	46	25.91831	30	25.91831	16
844	25.93877	15	25.93877	36	25.93877	28	25.93877	11
683	25.95924	17	25.95924	31	25.95924	26	25.95924	13
543	25.9797	18	25.9797	32	25.9797	25	25.9797	16
404	26.00016	24	26.00016	26	26.00016	25	26.00016	20
293	26.02062	40	26.02062	23	26.02062	25	26.02062	19
212	26.04109	39	26.04109	21	26.04109	25	26.04109	22
140	26.06155	44	26.06155	33	26.06155	26	26.06155	21
97	26.08201	44	26.08201	41	26.08201	26	26.08201	17
70	26.10248	44	26.10248	67	26.10248	27	26.10248	15
59	26.12294	34	26.12294	78	26.12294	28	26.12294	11
53	26.1434	28	26.1434	85	26.1434	29	26.1434	6
61	26.16386	26	26.16386	68	26.16386	31	26.16386	9
62	26.18433	20	26.18433	56	26.18433	33	26.18433	12
70	26.20479	19	26.20479	58	26.20479	35	26.20479	5
72	26.22525	6	26.22525	60	26.22525	37	26.22525	4
84	26.24572	5	26.24572	61	26.24572	40	26.24572	17
86	26.26618	10	26.26618	65	26.26618	42	26.26618	22
94	26.28664	11	26.28664	62	26.28664	45	26.28664	25
105	26.3071	12	26.3071	65	26.3071	48	26.3071	21
120	26.32757	23	26.32757	61	26.32757	51	26.32757	20
135	26.34803	29	26.34803	64	26.34803	53	26.34803	22
143	26.36849	35	26.36849	44	26.36849	55	26.36849	24
159	26.38895	26	26.38895	47	26.38895	56	26.38895	29
183	26.40942	30	26.40942	63	26.40942	56	26.40942	29
211	26.42988	24	26.42988	80	26.42988	54	26.42988	29
287	26.45034	25	26.45034	87	26.45034	52	26.45034	36
375	26.47081	27	26.47081	88	26.47081	50	26.47081	40
489	26.49127	35	26.49127	82	26.49127	48	26.49127	37
672	26.51173	37	26.51173	73	26.51173	47	26.51173	43
912	26.53219	51	26.53219	74	26.53219	47	26.53219	47
1223	26.55266	48	26.55266	71	26.55266	47	26.55266	51

1615	26.57312	41	26.57312	56	26.57312	47	26.57312	59
2033	26.59358	35	26.59358	56	26.59358	48	26.59358	57
2424	26.61405	39	26.61405	58	26.61405	47	26.61405	55
2763	26.63451	27	26.63451	67	26.63451	47	26.63451	50
3012	26.65497	17	26.65497	75	26.65497	46	26.65497	48
3075	26.67543	13	26.67543	91	26.67543	46	26.67543	45
2971	26.6959	11	26.6959	102	26.6959	46	26.6959	52
2742	26.71636	13	26.71636	124	26.71636	48	26.71636	64
2391	26.73682	17	26.73682	137	26.73682	51	26.73682	65
1998	26.75728	15	26.75728	149	26.75728	54	26.75728	49
1627	26.77775	20	26.77775	149	26.77775	58	26.77775	45
1253	26.79821	37	26.79821	141	26.79821	61	26.79821	45
959	26.81867	38	26.81867	131	26.81867	63	26.81867	52
740	26.83914	46	26.83914	118	26.83914	64	26.83914	55
576	26.8596	44	26.8596	112	26.8596	65	26.8596	46
444	26.88006	35	26.88006	107	26.88006	65	26.88006	42
349	26.90052	32	26.90052	94	26.90052	64	26.90052	45
280	26.92099	40	26.92099	88	26.92099	64	26.92099	34
229	26.94145	42	26.94145	79	26.94145	63	26.94145	21
194	26.96191	35	26.96191	71	26.96191	63	26.96191	3
157	26.98238	39	26.98238	49	26.98238	63	26.98238	2
128	27.00284	46	27.00284	40	27.00284	63	27.00284	9
122	27.0233	38	27.0233	42	27.0233	62	27.0233	16
111	27.04376	38	27.04376	42	27.04376	61	27.04376	12
92	27.06423	33	27.06423	50	27.06423	59	27.06423	12
77	27.08469	26	27.08469	48	27.08469	56	27.08469	25
69	27.10515	17	27.10515	48	27.10515	52	27.10515	36
61	27.12561	22	27.12561	53	27.12561	48	27.12561	44
53	27.14608	20	27.14608	51	27.14608	45	27.14608	51
45	27.16654	18	27.16654	44	27.16654	42	27.16654	59
24	27.187	22	27.187	46	27.187	41	27.187	61
19	27.20747	16	27.20747	51	27.20747	42	27.20747	62
11	27.22793	15	27.22793	55	27.22793	46	27.22793	64
5	27.24839	15	27.24839	45	27.24839	52	27.24839	52
1	27.26885	11	27.26885	39	27.26885	58	27.26885	50
0	27.28932	10	27.28932	31	27.28932	64	27.28932	59
2	27.30978	11	27.30978	23	27.30978	69	27.30978	56
4	27.33024	27	27.33024	20	27.33024	72	27.33024	51
5	27.35071	42	27.35071	17	27.35071	73	27.35071	42
5	27.37117	52	27.37117	18	27.37117	72	27.37117	36
3	27.39163	57	27.39163	35	27.39163	70	27.39163	38
6	27.41209	57	27.41209	40	27.41209	67	27.41209	40
2	27.43256	57	27.43256	44	27.43256	62	27.43256	36
8	27.45302	47	27.45302	42	27.45302	58	27.45302	31
12	27.47348	22	27.47348	33	27.47348	54	27.47348	33
14	27.49394	25	27.49394	33	27.49394	50	27.49394	35
14	27.51441	25	27.51441	26	27.51441	46	27.51441	33
14	27.53487	22	27.53487	28	27.53487	44	27.53487	28
14	27.55533	27	27.55533	32	27.55533	43	27.55533	33
15	27.5758	30	27.5758	35	27.5758	45	27.5758	30
17	27.59626	28	27.59626	50	27.59626	48	27.59626	45
12	27.61672	27	27.61672	54	27.61672	54	27.61672	46
19	27.63718	23	27.63718	47	27.63718	61	27.63718	40
34	27.65765	20	27.65765	43	27.65765	68	27.65765	41
40	27.67811	13	27.67811	30	27.67811	75	27.67811	39
62	27.69857	23	27.69857	23	27.69857	81	27.69857	31
76	27.71904	23	27.71904	24	27.71904	84	27.71904	29
94	27.7395	21	27.7395	30	27.7395	86	27.7395	34

127	27.75996	21	27.75996	31	27.75996	85	27.75996	46
178	27.78042	18	27.78042	33	27.78042	83	27.78042	55
246	27.80089	16	27.80089	36	27.80089	79	27.80089	67
336	27.82135	20	27.82135	28	27.82135	76	27.82135	71
487	27.84181	24	27.84181	17	27.84181	71	27.84181	80
647	27.86227	21	27.86227	14	27.86227	68	27.86227	79
839	27.88274	22	27.88274	10	27.88274	64	27.88274	79
1040	27.9032	21	27.9032	0	27.9032	61	27.9032	69
1223	27.92366	20	27.92366	11	27.92366	57	27.92366	64
1388	27.94413	16	27.94413	25	27.94413	53	27.94413	61
1490	27.96459	7	27.96459	33	27.96459	50	27.96459	44
1509	27.98505	3	27.98505	36	27.98505	47	27.98505	43
1453	28.00551	3	28.00551	34	28.00551	44	28.00551	31
1331	28.02598	7	28.02598	28	28.02598	44	28.02598	35
1173	28.04644	7	28.04644	31	28.04644	45	28.04644	35
978	28.0669	0	28.0669	21	28.0669	48	28.0669	34
780	28.08737	10	28.08737	26	28.08737	53	28.08737	35
598	28.10783	16	28.10783	22	28.10783	58	28.10783	27
452	28.12829	21	28.12829	38	28.12829	62	28.12829	36
341	28.14875	14	28.14875	48	28.14875	64	28.14875	35
255	28.16922	22	28.16922	51	28.16922	64	28.16922	28
179	28.18968	28	28.18968	48	28.18968	62	28.18968	35
140	28.21014	27	28.21014	42	28.21014	59	28.21014	36
112	28.2306	30	28.2306	36	28.2306	55	28.2306	41
90	28.25107	26	28.25107	41	28.25107	51	28.25107	40
81	28.27153	18	28.27153	36	28.27153	50	28.27153	37
71	28.29199	30	28.29199	43	28.29199	50	28.29199	27
79	28.31246	29	28.31246	44	28.31246	52	28.31246	18
78	28.33292	26	28.33292	51	28.33292	55	28.33292	25
84	28.35338	17	28.35338	41	28.35338	58	28.35338	35
96	28.37384	22	28.37384	37	28.37384	59	28.37384	46
106	28.39431	18	28.39431	27	28.39431	59	28.39431	53
130	28.41477	7	28.41477	13	28.41477	58	28.41477	59
140	28.43523	7	28.43523	6	28.43523	54	28.43523	53
158	28.4557	0	28.4557	12	28.4557	51	28.4557	49
177	28.47616	4	28.47616	8	28.47616	47	28.47616	36
206	28.49662	13	28.49662	20	28.49662	44	28.49662	33
258	28.51708	12	28.51708	26	28.51708	44	28.51708	33
339	28.53755	11	28.53755	33	28.53755	45	28.53755	55
450	28.55801	20	28.55801	41	28.55801	47	28.55801	72
602	28.57847	30	28.57847	53	28.57847	50	28.57847	97
821	28.59893	32	28.59893	60	28.59893	53	28.59893	108
1071	28.6194	39	28.6194	58	28.6194	55	28.6194	104
1355	28.63986	43	28.63986	58	28.63986	55	28.63986	84
1670	28.66032	47	28.66032	48	28.66032	54	28.66032	78
1945	28.68079	53	28.68079	41	28.68079	52	28.68079	59
2171	28.70125	50	28.70125	42	28.70125	50	28.70125	50
2294	28.72171	38	28.72171	44	28.72171	47	28.72171	40
2309	28.74217	19	28.74217	42	28.74217	44	28.74217	51
2196	28.76264	24	28.76264	48	28.76264	42	28.76264	49
1991	28.7831	26	28.7831	51	28.7831	41	28.7831	56
1726	28.80356	18	28.80356	49	28.80356	39	28.80356	51
1405	28.82403	26	28.82403	42	28.82403	38	28.82403	39
1098	28.84449	25	28.84449	50	28.84449	36	28.84449	35
834	28.86495	31	28.86495	48	28.86495	34	28.86495	43
628	28.88541	30	28.88541	68	28.88541	31	28.88541	33
473	28.90588	28	28.90588	70	28.90588	28	28.90588	27
352	28.92634	12	28.92634	93	28.92634	26	28.92634	29

274	28.9468	9	28.9468	97	28.9468	23	28.9468	46
218	28.96726	21	28.96726	108	28.96726	21	28.96726	44
179	28.98773	21	28.98773	102	28.98773	20	28.98773	47
138	29.00819	20	29.00819	105	29.00819	20	29.00819	54
109	29.02865	32	29.02865	99	29.02865	21	29.02865	57
82	29.04912	37	29.04912	104	29.04912	24	29.04912	53
61	29.06958	41	29.06958	97	29.06958	27	29.06958	44
58	29.09004	52	29.09004	106	29.09004	31	29.09004	34
41	29.1105	52	29.1105	95	29.1105	34	29.1105	27
35	29.13097	40	29.13097	95	29.13097	36	29.13097	29
28	29.15143	42	29.15143	81	29.15143	37	29.15143	30
20	29.17189	40	29.17189	82	29.17189	36	29.17189	20
14	29.19236	33	29.19236	88	29.19236	34	29.19236	18
10	29.21282	25	29.21282	109	29.21282	31	29.21282	16
22	29.23328	23	29.23328	118	29.23328	28	29.23328	22
25	29.25374	25	29.25374	122	29.25374	26	29.25374	21
22	29.27421	34	29.27421	117	29.27421	26	29.27421	20
22	29.29467	45	29.29467	118	29.29467	27	29.29467	16
17	29.31513	44	29.31513	95	29.31513	29	29.31513	30
15	29.33559	34	29.33559	66	29.33559	33	29.33559	40
7	29.35606	26	29.35606	42	29.35606	37	29.35606	32
6	29.37652	23	29.37652	42	29.37652	40	29.37652	36
5	29.39698	23	29.39698	49	29.39698	43	29.39698	33
6	29.41745	11	29.41745	59	29.41745	45	29.41745	39
8	29.43791	10	29.43791	74	29.43791	46	29.43791	58
9	29.45837	21	29.45837	73	29.45837	47	29.45837	61
9	29.47883	33	29.47883	68	29.47883	46	29.47883	56
11	29.4993	33	29.4993	68	29.4993	46	29.4993	37
6	29.51976	38	29.51976	55	29.51976	46	29.51976	40
3	29.54022	38	29.54022	41	29.54022	46	29.54022	29
8	29.56068	33	29.56068	41	29.56068	48	29.56068	14
10	29.58115	25	29.58115	41	29.58115	50	29.58115	30
15	29.60161	32	29.60161	36	29.60161	53	29.60161	28
25	29.62207	29	29.62207	31	29.62207	55	29.62207	39
37	29.64254	28	29.64254	32	29.64254	57	29.64254	55
54	29.663	29	29.663	20	29.663	58	29.663	62
70	29.68346	22	29.68346	10	29.68346	58	29.68346	56
89	29.70392	14	29.70392	10	29.70392	57	29.70392	56
105	29.72439	16	29.72439	3	29.72439	55	29.72439	58
146	29.74485	17	29.74485	10	29.74485	53	29.74485	50
192	29.76531	8	29.76531	17	29.76531	50	29.76531	50
244	29.78578	10	29.78578	14	29.78578	47	29.78578	51
323	29.80624	27	29.80624	15	29.80624	43	29.80624	31
433	29.8267	38	29.8267	15	29.8267	40	29.8267	31
574	29.84716	45	29.84716	26	29.84716	36	29.84716	34
746	29.86763	45	29.86763	31	29.86763	32	29.86763	32
934	29.88809	36	29.88809	29	29.88809	30	29.88809	23
1116	29.90855	29	29.90855	34	29.90855	29	29.90855	30
1265	29.92901	23	29.92901	25	29.92901	29	29.92901	27
1367	29.94948	15	29.94948	30	29.94948	29	29.94948	18
1375	29.96994	13	29.96994	27	29.96994	31	29.96994	25
1319	29.9904	16	29.9904	28	29.9904	32	29.9904	23
1200	30.01087	24	30.01087	29	30.01087	33	30.01087	22
1038	30.03133	35	30.03133	27	30.03133	33	30.03133	27
859	30.05179	38	30.05179	34	30.05179	31	30.05179	36
682	30.07225	34	30.07225	32	30.07225	28	30.07225	55
523	30.09272	39	30.09272	22	30.09272	24	30.09272	57
396	30.11318	40	30.11318	22	30.11318	20	30.11318	61

300	30.13364	40	30.13364	14	30.13364	16	30.13364	58
226	30.15411	39	30.15411	14	30.15411	13	30.15411	54
169	30.17457	48	30.17457	20	30.17457	10	30.17457	55
140	30.19503	53	30.19503	28	30.19503	9	30.19503	50
117	30.21549	55	30.21549	22	30.21549	9	30.21549	36
99	30.23596	53	30.23596	17	30.23596	9	30.23596	29
85	30.25642	39	30.25642	25	30.25642	11	30.25642	32
74	30.27688	34	30.27688	33	30.27688	14	30.27688	35
61	30.29734	35	30.29734	40	30.29734	18	30.29734	26
59	30.31781	20	30.31781	48	30.31781	22	30.31781	19
51	30.33827	16	30.33827	42	30.33827	26	30.33827	31
52	30.35873	19	30.35873	39	30.35873	30	30.35873	35
43	30.3792	23	30.3792	43	30.3792	32	30.3792	40
39	30.39966	24	30.39966	38	30.39966	33	30.39966	46
44	30.42012	24	30.42012	34	30.42012	33	30.42012	40
58	30.44058	29	30.44058	38	30.44058	32	30.44058	52
63	30.46105	24	30.46105	58	30.46105	31	30.46105	51
81	30.48151	30	30.48151	70	30.48151	30	30.48151	39
110	30.50197	36	30.50197	80	30.50197	30	30.50197	28
140	30.52244	31	30.52244	79	30.52244	31	30.52244	10
172	30.5429	38	30.5429	61	30.5429	33	30.5429	24
220	30.56336	41	30.56336	55	30.56336	35	30.56336	18
275	30.58382	31	30.58382	55	30.58382	36	30.58382	27
334	30.60429	26	30.60429	51	30.60429	37	30.60429	32
398	30.62475	24	30.62475	65	30.62475	37	30.62475	33
457	30.64521	31	30.64521	68	30.64521	38	30.64521	36
502	30.66567	39	30.66567	77	30.66567	38	30.66567	41
571	30.68614	39	30.68614	76	30.68614	38	30.68614	38
679	30.7066	53	30.7066	88	30.7066	38	30.7066	34
804	30.72706	62	30.72706	79	30.72706	39	30.72706	23
996	30.74753	64	30.74753	69	30.74753	39	30.74753	25
1274	30.76799	52	30.76799	65	30.76799	40	30.76799	19
1635	30.78845	47	30.78845	58	30.78845	39	30.78845	18
2030	30.80891	51	30.80891	48	30.80891	38	30.80891	14
2407	30.82938	52	30.82938	52	30.82938	36	30.82938	15
2721	30.84984	69	30.84984	44	30.84984	34	30.84984	13
2934	30.8703	82	30.8703	49	30.8703	32	30.8703	25
3000	30.89077	74	30.89077	52	30.89077	31	30.89077	28
2918	30.91123	75	30.91123	65	30.91123	30	30.91123	36
2665	30.93169	78	30.93169	71	30.93169	29	30.93169	34
2324	30.95215	63	30.95215	85	30.95215	29	30.95215	50
1943	30.97262	45	30.97262	90	30.97262	30	30.97262	57
1546	30.99308	34	30.99308	86	30.99308	30	30.99308	56
1192	31.01354	19	31.01354	78	31.01354	29	31.01354	57
874	31.034	11	31.034	78	31.034	28	31.034	50
638	31.05447	13	31.05447	71	31.05447	25	31.05447	41
467	31.07493	21	31.07493	73	31.07493	22	31.07493	32
348	31.09539	14	31.09539	58	31.09539	19	31.09539	25
267	31.11586	17	31.11586	56	31.11586	15	31.11586	21
207	31.13632	35	31.13632	55	31.13632	12	31.13632	13
177	31.15678	39	31.15678	60	31.15678	10	31.15678	18
149	31.17724	41	31.17724	69	31.17724	8	31.17724	11
115	31.19771	38	31.19771	71	31.19771	8	31.19771	20
93	31.21817	33	31.21817	67	31.21817	10	31.21817	27
62	31.23863	38	31.23863	71	31.23863	12	31.23863	24
47	31.2591	42	31.2591	79	31.2591	16	31.2591	30
33	31.27956	42	31.27956	79	31.27956	20	31.27956	33
25	31.30002	30	31.30002	73	31.30002	24	31.30002	34

15	31.32048	33	31.32048	82	31.32048	28	31.32048	31
9	31.34095	32	31.34095	79	31.34095	31	31.34095	20
11	31.36141	27	31.36141	83	31.36141	32	31.36141	14
11	31.38187	23	31.38187	92	31.38187	32	31.38187	5
10	31.40233	29	31.40233	95	31.40233	31	31.40233	14
5	31.4228	36	31.4228	96	31.4228	29	31.4228	14
5	31.44326	44	31.44326	97	31.44326	26	31.44326	12
8	31.46372	42	31.46372	104	31.46372	24	31.46372	17
14	31.48419	34	31.48419	97	31.48419	22	31.48419	16
26	31.50465	31	31.50465	94	31.50465	20	31.50465	9
37	31.52511	26	31.52511	97	31.52511	19	31.52511	11
46	31.54557	19	31.54557	104	31.54557	19	31.54557	4
59	31.56604	10	31.56604	97	31.56604	20	31.56604	8
67	31.5865	12	31.5865	103	31.5865	20	31.5865	11
81	31.60696	23	31.60696	97	31.60696	20	31.60696	11
93	31.62743	38	31.62743	99	31.62743	20	31.62743	16
114	31.64789	45	31.64789	94	31.64789	19	31.64789	23
138	31.66835	48	31.66835	99	31.66835	18	31.66835	30
192	31.68881	44	31.68881	97	31.68881	17	31.68881	33
254	31.70928	36	31.70928	76	31.70928	16	31.70928	28
321	31.72974	32	31.72974	74	31.72974	15	31.72974	28
381	31.7502	27	31.7502	77	31.7502	15	31.7502	25
423	31.77066	20	31.77066	67	31.77066	17	31.77066	28
449	31.79113	17	31.79113	73	31.79113	19	31.79113	28
459	31.81159	21	31.81159	64	31.81159	22	31.81159	23
418	31.83205	36	31.83205	66	31.83205	25	31.83205	28
364	31.85252	37	31.85252	55	31.85252	28	31.85252	23
305	31.87298	44	31.87298	59	31.87298	30	31.87298	27
257	31.89344	38	31.89344	50	31.89344	30	31.89344	31
195	31.9139	37	31.9139	35	31.9139	29	31.9139	28
154	31.93437	44	31.93437	40	31.93437	27	31.93437	26
114	31.95483	40	31.95483	42	31.95483	24	31.95483	23
86	31.97529	36	31.97529	33	31.97529	22	31.97529	17
75	31.99576	26	31.99576	46	31.99576	20	31.99576	16
64	32.01622	24	32.01622	46	32.01622	19	32.01622	25
47	32.03668	20	32.03668	43	32.03668	19	32.03668	32
35	32.05714	12	32.05714	30	32.05714	20	32.05714	28
33	32.07761	12	32.07761	26	32.07761	20	32.07761	39
32	32.09807	5	32.09807	21	32.09807	20	32.09807	48
31	32.11853	8	32.11853	24	32.11853	18	32.11853	46
39	32.13899	5	32.13899	25	32.13899	15	32.13899	36
44	32.15946	6	32.15946	36	32.15946	12	32.15946	25
54	32.17992	5	32.17992	32	32.17992	9	32.17992	15
65	32.20038	9	32.20038	50	32.20038	7	32.20038	16
88	32.22085	9	32.22085	44	32.22085	6	32.22085	22
116	32.24131	11	32.24131	47	32.24131	6	32.24131	24
149	32.26177	11	32.26177	52	32.26177	8	32.26177	23
195	32.28223	11	32.28223	58	32.28223	11	32.28223	24
255	32.3027	11	32.3027	61	32.3027	15	32.3027	23
348	32.32316	9	32.32316	58	32.32316	19	32.32316	19
448	32.34362	0	32.34362	49	32.34362	22	32.34362	12
589	32.36409	4	32.36409	44	32.36409	24	32.36409	10
731	32.38455	5	32.38455	31	32.38455	25	32.38455	2
884	32.40501	4	32.40501	29	32.40501	25	32.40501	6
1023	32.42547	11	32.42547	17	32.42547	23	32.42547	7
1125	32.44594	18	32.44594	21	32.44594	21	32.44594	6
1180	32.4664	27	32.4664	29	32.4664	18	32.4664	16
1176	32.48686	30	32.48686	46	32.48686	14	32.48686	23

1113	32.50732	30	32.50732	50	32.50732	11	32.50732	29
1021	32.52779	32	32.52779	55	32.52779	8	32.52779	26
901	32.54825	31	32.54825	56	32.54825	6	32.54825	29
787	32.56871	34	32.56871	48	32.56871	5	32.56871	28
655	32.58918	28	32.58918	50	32.58918	4	32.58918	25
565	32.60964	21	32.60964	48	32.60964	4	32.60964	27
480	32.6301	19	32.6301	45	32.6301	5	32.6301	26
427	32.65056	12	32.65056	42	32.65056	7	32.65056	44
380	32.67103	11	32.67103	34	32.67103	9	32.67103	65
349	32.69149	0	32.69149	39	32.69149	12	32.69149	72
317	32.71195	1	32.71195	34	32.71195	15	32.71195	71
287	32.73242	5	32.73242	39	32.73242	18	32.73242	58
260	32.75288	12	32.75288	42	32.75288	21	32.75288	53
206	32.77334	14	32.77334	43	32.77334	22	32.77334	38
161	32.7938	12	32.7938	53	32.7938	23	32.7938	22
132	32.81427	11	32.81427	51	32.81427	22	32.81427	6
92	32.83473	12	32.83473	55	32.83473	20	32.83473	0
68	32.85519	15	32.85519	57	32.85519	18	32.85519	6
40	32.87565	17	32.87565	53	32.87565	15	32.87565	9
33	32.89612	16	32.89612	54	32.89612	12	32.89612	8
27	32.91658	23	32.91658	51	32.91658	11	32.91658	4
21	32.93704	23	32.93704	47	32.93704	10	32.93704	9
19	32.95751	23	32.95751	39	32.95751	11	32.95751	12
13	32.97797	16	32.97797	46	32.97797	14	32.97797	24
20	32.99843	11	32.99843	46	32.99843	17	32.99843	30
18	33.01889	11	33.01889	46	33.01889	20	33.01889	34
12	33.03936	3	33.03936	50	33.03936	24	33.03936	32
8	33.05982	13	33.05982	54	33.05982	27	33.05982	36
2	33.08028	20	33.08028	57	33.08028	30	33.08028	28
4	33.10075	21	33.10075	54	33.10075	32	33.10075	17
2	33.12121	27	33.12121	43	33.12121	33	33.12121	10
4	33.14167	26	33.14167	37	33.14167	34	33.14167	15
4	33.16213	30	33.16213	34	33.16213	33	33.16213	5
5	33.1826	28	33.1826	45	33.1826	31	33.1826	22
4	33.20306	28	33.20306	56	33.20306	28	33.20306	23
5	33.22352	17	33.22352	70	33.22352	25	33.22352	36
9	33.24398	11	33.24398	78	33.24398	22	33.24398	45
8	33.26445	19	33.26445	77	33.26445	19	33.26445	45
8	33.28491	21	33.28491	76	33.28491	16	33.28491	42
13	33.30537	18	33.30537	75	33.30537	14	33.30537	34
18	33.32584	14	33.32584	67	33.32584	13	33.32584	27
20	33.3463	18	33.3463	66	33.3463	12	33.3463	25
25	33.36676	25	33.36676	65	33.36676	12	33.36676	20
30	33.38722	27	33.38722	79	33.38722	13	33.38722	35
36	33.40769	20	33.40769	83	33.40769	13	33.40769	27
43	33.42815	18	33.42815	92	33.42815	14	33.42815	39
54	33.44861	19	33.44861	97	33.44861	16	33.44861	55
63	33.46908	20	33.46908	94	33.46908	17	33.46908	53
77	33.48954	21	33.48954	89	33.48954	19	33.48954	56
91	33.51	20	33.51	84	33.51	21	33.51	53
105	33.53046	21	33.53046	88	33.53046	22	33.53046	42
117	33.55093	27	33.55093	80	33.55093	24	33.55093	43
130	33.57139	24	33.57139	87	33.57139	25	33.57139	52
134	33.59185	21	33.59185	93	33.59185	25	33.59185	55
130	33.61231	12	33.61231	93	33.61231	24	33.61231	43
120	33.63278	19	33.63278	106	33.63278	23	33.63278	38
93	33.65324	18	33.65324	112	33.65324	20	33.65324	38
73	33.6737	19	33.6737	107	33.6737	18	33.6737	34

67	33.69417	17	33.69417	106	33.69417	15	33.69417	31
61	33.71463	18	33.71463	107	33.71463	13	33.71463	30
62	33.73509	14	33.73509	113	33.73509	12	33.73509	26
68	33.75555	14	33.75555	104	33.75555	12	33.75555	37
80	33.77602	9	33.77602	112	33.77602	13	33.77602	50
92	33.79648	11	33.79648	109	33.79648	15	33.79648	56
110	33.81694	9	33.81694	114	33.81694	17	33.81694	62
119	33.83741	15	33.83741	108	33.83741	20	33.83741	64
124	33.85787	16	33.85787	99	33.85787	22	33.85787	65
149	33.87833	23	33.87833	94	33.87833	23	33.87833	68
173	33.89879	24	33.89879	95	33.89879	23	33.89879	63
188	33.91926	22	33.91926	91	33.91926	22	33.91926	53
217	33.93972	21	33.93972	84	33.93972	20	33.93972	58
255	33.96018	23	33.96018	79	33.96018	18	33.96018	61
307	33.98064	24	33.98064	80	33.98064	17	33.98064	69
369	34.00111	20	34.00111	82	34.00111	16	34.00111	69
461	34.02157	20	34.02157	89	34.02157	16	34.02157	72
570	34.04203	17	34.04203	86	34.04203	16	34.04203	83
743	34.0625	16	34.0625	85	34.0625	17	34.0625	87
961	34.08296	20	34.08296	87	34.08296	18	34.08296	91
1203	34.10342	24	34.10342	82	34.10342	18	34.10342	89
1475	34.12388	25	34.12388	75	34.12388	17	34.12388	75
1741	34.14435	28	34.14435	75	34.14435	17	34.14435	69
1965	34.16481	25	34.16481	73	34.16481	15	34.16481	57
2109	34.18527	20	34.18527	79	34.18527	14	34.18527	57
2153	34.20574	14	34.20574	84	34.20574	14	34.20574	46
2104	34.2262	16	34.2262	83	34.2262	14	34.2262	49
1966	34.24666	9	34.24666	69	34.24666	15	34.24666	58
1782	34.26712	5	34.26712	72	34.26712	16	34.26712	59
1545	34.28759	5	34.28759	75	34.28759	17	34.28759	59
1308	34.30805	5	34.30805	66	34.30805	18	34.30805	65
1082	34.32851	11	34.32851	55	34.32851	18	34.32851	68
872	34.34897	14	34.34897	62	34.34897	17	34.34897	61
688	34.36944	16	34.36944	60	34.36944	17	34.36944	64
529	34.3899	14	34.3899	63	34.3899	17	34.3899	63
397	34.41036	12	34.41036	55	34.41036	17	34.41036	59
302	34.43083	13	34.43083	49	34.43083	17	34.43083	65
241	34.45129	11	34.45129	37	34.45129	18	34.45129	53
192	34.47175	11	34.47175	45	34.47175	20	34.47175	46
161	34.49221	13	34.49221	44	34.49221	23	34.49221	47
146	34.51268	19	34.51268	36	34.51268	25	34.51268	47
124	34.53314	31	34.53314	35	34.53314	26	34.53314	53
113	34.5536	33	34.5536	38	34.5536	28	34.5536	48
101	34.57407	30	34.57407	37	34.57407	28	34.57407	53
89	34.59453	32	34.59453	36	34.59453	27	34.59453	46
70	34.61499	22	34.61499	32	34.61499	24	34.61499	35
55	34.63545	26	34.63545	40	34.63545	21	34.63545	39
57	34.65592	18	34.65592	36	34.65592	18	34.65592	40
57	34.67638	19	34.67638	31	34.67638	15	34.67638	48
65	34.69684	29	34.69684	30	34.69684	13	34.69684	62
62	34.7173	31	34.7173	26	34.7173	12	34.7173	61
56	34.73777	38	34.73777	23	34.73777	12	34.73777	71
51	34.75823	42	34.75823	20	34.75823	13	34.75823	66
48	34.77869	42	34.77869	28	34.77869	16	34.77869	60
42	34.79916	43	34.79916	39	34.79916	18	34.79916	48
36	34.81962	32	34.81962	38	34.81962	19	34.81962	42
30	34.84008	26	34.84008	52	34.84008	20	34.84008	48
34	34.86054	13	34.86054	55	34.86054	19	34.86054	55

36	34.88101	10	34.88101	49	34.88101	18	34.88101	52
42	34.90147	7	34.90147	48	34.90147	16	34.90147	58
56	34.92193	0	34.92193	38	34.92193	15	34.92193	58
62	34.9424	2	34.9424	34	34.9424	14	34.9424	56
75	34.96286	5	34.96286	31	34.96286	15	34.96286	46
90	34.98332	5	34.98332	28	34.98332	17	34.98332	42
103	35.00378	2	35.00378	26	35.00378	19	35.00378	49
124	35.02425	5	35.02425	14	35.02425	21	35.02425	58
133	35.04471	2	35.04471	22	35.04471	23	35.04471	53
152	35.06517	5	35.06517	21	35.06517	24	35.06517	59
189	35.08564	13	35.08564	11	35.08564	24	35.08564	57
250	35.1061	12	35.1061	13	35.1061	24	35.1061	54
332	35.12656	21	35.12656	17	35.12656	23	35.12656	57
442	35.14702	27	35.14702	20	35.14702	24	35.14702	50
592	35.16748	30	35.16748	22	35.16748	25	35.16748	38
737	35.18795	30	35.18795	26	35.18795	28	35.18795	40
866	35.20841	22	35.20841	36	35.20841	32	35.20841	48
973	35.22887	29	35.22887	46	35.22887	36	35.22887	44
1021	35.24934	25	35.24934	46	35.24934	40	35.24934	46
1031	35.2698	25	35.2698	66	35.2698	41	35.2698	55
986	35.29026	28	35.29026	72	35.29026	41	35.29026	51
884	35.31073	25	35.31073	75	35.31073	37	35.31073	41
758	35.33119	26	35.33119	68	35.33119	32	35.33119	46
624	35.35165	25	35.35165	42	35.35165	26	35.35165	31
502	35.37211	24	35.37211	35	35.37211	19	35.37211	19
389	35.39258	18	35.39258	39	35.39258	13	35.39258	25
297	35.41304	10	35.41304	39	35.41304	9	35.41304	33
215	35.4335	19	35.4335	38	35.4335	7	35.4335	39
158	35.45396	14	35.45396	24	35.45396	7	35.45396	49
124	35.47443	24	35.47443	45	35.47443	8	35.47443	51
91	35.49489	30	35.49489	47	35.49489	11	35.49489	47
59	35.51535	31	35.51535	39	35.51535	14	35.51535	35
48	35.53582	28	35.53582	30	35.53582	18	35.53582	30
26	35.55628	29	35.55628	9	35.55628	22	35.55628	16
21	35.57674	26	35.57674	19	35.57674	26	35.57674	13
30	35.59721	23	35.59721	26	35.59721	30	35.59721	16
38	35.61767	22	35.61767	23	35.61767	35	35.61767	15
42	35.63813	19	35.63813	21	35.63813	38	35.63813	11
53	35.65859	19	35.65859	14	35.65859	41	35.65859	13
66	35.67905	27	35.67905	14	35.67905	42	35.67905	19
79	35.69952	24	35.69952	13	35.69952	42	35.69952	27
98	35.71998	15	35.71998	15	35.71998	40	35.71998	28
124	35.74044	17	35.74044	18	35.74044	36	35.74044	33
145	35.76091	9	35.76091	18	35.76091	32	35.76091	40
172	35.78137	15	35.78137	26	35.78137	27	35.78137	54
204	35.80183	18	35.80183	28	35.80183	23	35.80183	51
236	35.8223	30	35.8223	16	35.8223	19	35.8223	54
258	35.84276	32	35.84276	21	35.84276	17	35.84276	50
261	35.86322	41	35.86322	25	35.86322	15	35.86322	47
262	35.88368	34	35.88368	20	35.88368	15	35.88368	42
263	35.90414	35	35.90414	31	35.90414	15	35.90414	42
242	35.92461	28	35.92461	40	35.92461	15	35.92461	30
217	35.94507	29	35.94507	41	35.94507	16	35.94507	29
190	35.96553	27	35.96553	41	35.96553	16	35.96553	25
148	35.986	33	35.986	27	35.986	17	35.986	28
118	36.00646	32	36.00646	23	36.00646	17	36.00646	22
94	36.02692	40	36.02692	10	36.02692	17	36.02692	25
69	36.04739	33	36.04739	21	36.04739	16	36.04739	15

45	36.06785	30	36.06785	25	36.06785	16	36.06785	15
30	36.08831	17	36.08831	23	36.08831	15	36.08831	16
30	36.10877	22	36.10877	28	36.10877	16	36.10877	14
25	36.12924	14	36.12924	23	36.12924	17	36.12924	15
24	36.1497	9	36.1497	21	36.1497	18	36.1497	23
26	36.17016	9	36.17016	15	36.17016	20	36.17016	27
16	36.19062	17	36.19062	4	36.19062	23	36.19062	32
15	36.21109	24	36.21109	3	36.21109	25	36.21109	26
14	36.23155	24	36.23155	7	36.23155	26	36.23155	25
11	36.25201	25	36.25201	10	36.25201	27	36.25201	22
4	36.27248	26	36.27248	19	36.27248	26	36.27248	21
0	36.29294	23	36.29294	22	36.29294	25	36.29294	20
2	36.3134	22	36.3134	23	36.3134	23	36.3134	13
6	36.33387	15	36.33387	24	36.33387	22	36.33387	17
5	36.35433	3	36.35433	32	36.35433	20	36.35433	17
5	36.37479	1	36.37479	31	36.37479	19	36.37479	28
9	36.39525	4	36.39525	26	36.39525	19	36.39525	26
12	36.41571	6	36.41571	19	36.41571	19	36.41571	31
14	36.43618	5	36.43618	14	36.43618	19	36.43618	39
16	36.45664	10	36.45664	8	36.45664	19	36.45664	42
13	36.4771	10	36.4771	10	36.4771	19	36.4771	37
8	36.49757	9	36.49757	3	36.49757	19	36.49757	28
14	36.51803	6	36.51803	8	36.51803	17	36.51803	24
16	36.53849	5	36.53849	13	36.53849	16	36.53849	23
16	36.55896	4	36.55896	17	36.55896	13	36.55896	15
17	36.57942	5	36.57942	15	36.57942	11	36.57942	18
20	36.59988	3	36.59988	19	36.59988	9	36.59988	14
18	36.62034	7	36.62034	19	36.62034	7	36.62034	19
8	36.6408	7	36.6408	12	36.6408	7	36.6408	17
7	36.66127	6	36.66127	13	36.66127	7	36.66127	14
6	36.68173	5	36.68173	15	36.68173	8	36.68173	13
11	36.70219	2	36.70219	13	36.70219	11	36.70219	9
18	36.72266	0	36.72266	21	36.72266	13	36.72266	12
29	36.74312	2	36.74312	27	36.74312	16	36.74312	10
43	36.76358	1	36.76358	27	36.76358	19	36.76358	8
64	36.78405	0	36.78405	23	36.78405	21	36.78405	16
87	36.80451	0	36.80451	23	36.80451	21	36.80451	20
104	36.82497	5	36.82497	24	36.82497	21	36.82497	18
111	36.84543	9	36.84543	20	36.84543	20	36.84543	19
121	36.8659	11	36.8659	15	36.8659	18	36.8659	19
127	36.88636	13	36.88636	18	36.88636	16	36.88636	22
113	36.90682	11	36.90682	15	36.90682	15	36.90682	19
88	36.92728	12	36.92728	17	36.92728	13	36.92728	12
81	36.94775	11	36.94775	23	36.94775	13	36.94775	3
68	36.96821	5	36.96821	20	36.96821	12	36.96821	3
56	36.98867	3	36.98867	16	36.98867	13	36.98867	8
55	37.00914	5	37.00914	16	37.00914	13	37.00914	8
50	37.0296	12	37.0296	22	37.0296	13	37.0296	9
46	37.05006	19	37.05006	18	37.05006	14	37.05006	14
56	37.07053	23	37.07053	13	37.07053	13	37.07053	17
57	37.09099	24	37.09099	15	37.09099	13	37.09099	12
51	37.11145	22	37.11145	21	37.11145	14	37.11145	16
55	37.13191	20	37.13191	23	37.13191	14	37.13191	14
58	37.15237	11	37.15237	27	37.15237	16	37.15237	16
65	37.17284	8	37.17284	22	37.17284	17	37.17284	21
72	37.1933	4	37.1933	20	37.1933	19	37.1933	19
101	37.21376	4	37.21376	21	37.21376	21	37.21376	19
124	37.23423	14	37.23423	15	37.23423	22	37.23423	21

161	37.25469	24	37.25469	11	37.25469	21	37.25469	26
219	37.27515	27	37.27515	12	37.27515	20	37.27515	24
286	37.29562	23	37.29562	13	37.29562	17	37.29562	16
359	37.31608	24	37.31608	20	37.31608	14	37.31608	21
461	37.33654	20	37.33654	17	37.33654	12	37.33654	17
580	37.357	16	37.357	20	37.357	9	37.357	18
724	37.37746	16	37.37746	17	37.37746	8	37.37746	13
871	37.39793	17	37.39793	16	37.39793	9	37.39793	5
1036	37.41839	22	37.41839	18	37.41839	10	37.41839	3
1159	37.43885	27	37.43885	18	37.43885	12	37.43885	5
1227	37.45932	24	37.45932	21	37.45932	15	37.45932	5
1261	37.47978	19	37.47978	29	37.47978	16	37.47978	9
1260	37.50024	13	37.50024	32	37.50024	17	37.50024	10
1239	37.52071	14	37.52071	33	37.52071	17	37.52071	11
1237	37.54117	8	37.54117	25	37.54117	16	37.54117	11
1236	37.56163	4	37.56163	26	37.56163	14	37.56163	21
1257	37.58209	3	37.58209	15	37.58209	12	37.58209	34
1295	37.60256	12	37.60256	12	37.60256	10	37.60256	39
1325	37.62302	14	37.62302	18	37.62302	9	37.62302	42
1317	37.64348	14	37.64348	20	37.64348	8	37.64348	47
1257	37.66394	7	37.66394	17	37.66394	8	37.66394	45
1181	37.68441	12	37.68441	26	37.68441	8	37.68441	46
1082	37.70487	14	37.70487	25	37.70487	8	37.70487	40
954	37.72533	11	37.72533	25	37.72533	8	37.72533	29
818	37.7458	13	37.7458	18	37.7458	8	37.7458	19
683	37.76626	21	37.76626	17	37.76626	8	37.76626	20
561	37.78672	25	37.78672	8	37.78672	8	37.78672	22
453	37.80719	31	37.80719	14	37.80719	8	37.80719	15
346	37.82765	39	37.82765	17	37.82765	9	37.82765	13
260	37.84811	39	37.84811	16	37.84811	9	37.84811	20
197	37.86857	35	37.86857	13	37.86857	10	37.86857	20
163	37.88903	36	37.88903	28	37.88903	11	37.88903	16
134	37.9095	28	37.9095	37	37.9095	12	37.9095	24
110	37.92996	20	37.92996	49	37.92996	12	37.92996	26
88	37.95042	21	37.95042	50	37.95042	12	37.95042	22
80	37.97089	19	37.97089	47	37.97089	12	37.97089	21
66	37.99135	9	37.99135	41	37.99135	12	37.99135	18
60	38.01181	6	38.01181	34	38.01181	11	38.01181	14
44	38.03228	9	38.03228	23	38.03228	10	38.03228	15
34	38.05274	5	38.05274	11	38.05274	9	38.05274	10
37	38.0732	4	38.0732	3	38.0732	8	38.0732	8
43	38.09366	7	38.09366	17	38.09366	7	38.09366	3
49	38.11412	4	38.11412	23	38.11412	6	38.11412	7
62	38.13459	15	38.13459	33	38.13459	5	38.13459	12
67	38.15505	22	38.15505	35	38.15505	4	38.15505	20
75	38.17551	24	38.17551	40	38.17551	4	38.17551	21
88	38.19598	25	38.19598	46	38.19598	4	38.19598	24
100	38.21644	23	38.21644	41	38.21644	4	38.21644	24
114	38.2369	18	38.2369	36	38.2369	6	38.2369	26
150	38.25737	10	38.25737	23	38.25737	7	38.25737	25
191	38.27783	10	38.27783	17	38.27783	8	38.27783	25
239	38.29829	11	38.29829	16	38.29829	10	38.29829	20
307	38.31875	6	38.31875	17	38.31875	10	38.31875	15
386	38.33922	13	38.33922	21	38.33922	11	38.33922	19
455	38.35968	12	38.35968	22	38.35968	11	38.35968	17
514	38.38014	7	38.38014	26	38.38014	10	38.38014	15
565	38.4006	10	38.4006	25	38.4006	9	38.4006	14
591	38.42107	8	38.42107	17	38.42107	8	38.42107	11

595	38.44153	6	38.44153	12	38.44153	8	38.44153	11
580	38.46199	5	38.46199	10	38.46199	7	38.46199	16
543	38.48246	8	38.48246	11	38.48246	8	38.48246	15
482	38.50292	11	38.50292	13	38.50292	9	38.50292	18
413	38.52338	15	38.52338	16	38.52338	11	38.52338	22
343	38.54385	15	38.54385	23	38.54385	13	38.54385	27
270	38.56431	14	38.56431	25	38.56431	15	38.56431	26
210	38.58477	14	38.58477	25	38.58477	16	38.58477	23
161	38.60523	19	38.60523	18	38.60523	18	38.60523	26
132	38.62569	18	38.62569	9	38.62569	19	38.62569	26
113	38.64616	16	38.64616	11	38.64616	20	38.64616	23
110	38.66662	10	38.66662	16	38.66662	21	38.66662	33
112	38.68708	6	38.68708	20	38.68708	22	38.68708	37
108	38.70755	3	38.70755	25	38.70755	22	38.70755	40
94	38.72801	0	38.72801	23	38.72801	22	38.72801	35
90	38.74847	3	38.74847	36	38.74847	21	38.74847	37
91	38.76894	11	38.76894	37	38.76894	19	38.76894	25
90	38.7894	27	38.7894	32	38.7894	17	38.7894	20
96	38.80986	34	38.80986	31	38.80986	15	38.80986	23
122	38.83032	35	38.83032	28	38.83032	13	38.83032	24
156	38.85078	41	38.85078	29	38.85078	12	38.85078	33
202	38.87125	40	38.87125	33	38.87125	11	38.87125	40
250	38.89171	31	38.89171	36	38.89171	11	38.89171	47
305	38.91217	19	38.91217	37	38.91217	12	38.91217	41
362	38.93264	11	38.93264	35	38.93264	13	38.93264	37
419	38.9531	16	38.9531	43	38.9531	14	38.9531	41
461	38.97356	20	38.97356	37	38.97356	15	38.97356	31
487	38.99403	23	38.99403	26	38.99403	16	38.99403	38
491	39.01449	21	39.01449	34	39.01449	16	39.01449	36
499	39.03495	18	39.03495	39	39.03495	15	39.03495	44
496	39.05541	17	39.05541	42	39.05541	14	39.05541	46
479	39.07588	8	39.07588	52	39.07588	12	39.07588	37
466	39.09634	11	39.09634	51	39.09634	11	39.09634	38
455	39.1168	19	39.1168	51	39.1168	9	39.1168	33
454	39.13726	23	39.13726	50	39.13726	9	39.13726	40
449	39.15773	30	39.15773	51	39.15773	9	39.15773	42
423	39.17819	39	39.17819	39	39.17819	11	39.17819	33
400	39.19865	39	39.19865	22	39.19865	12	39.19865	38
364	39.21912	36	39.21912	24	39.21912	14	39.21912	34
328	39.23958	27	39.23958	23	39.23958	15	39.23958	32
283	39.26004	10	39.26004	14	39.26004	15	39.26004	25
225	39.28051	3	39.28051	20	39.28051	15	39.28051	15
184	39.30097	3	39.30097	13	39.30097	15	39.30097	16
152	39.32143	9	39.32143	18	39.32143	14	39.32143	19
124	39.34189	6	39.34189	18	39.34189	13	39.34189	18
94	39.36235	13	39.36235	22	39.36235	13	39.36235	16
65	39.38282	19	39.38282	21	39.38282	12	39.38282	13
62	39.40328	25	39.40328	14	39.40328	12	39.40328	20
55	39.42374	24	39.42374	13	39.42374	12	39.42374	16
45	39.44421	24	39.44421	14	39.44421	12	39.44421	15
35	39.46467	19	39.46467	8	39.46467	12	39.46467	14
26	39.48513	16	39.48513	13	39.48513	11	39.48513	15
28	39.5056	13	39.5056	12	39.5056	10	39.5056	16
29	39.52606	16	39.52606	17	39.52606	10	39.52606	17
31	39.54652	17	39.54652	21	39.54652	9	39.54652	19
27	39.56698	22	39.56698	22	39.56698	10	39.56698	25
34	39.58744	28	39.58744	26	39.58744	10	39.58744	33
41	39.60791	32	39.60791	28	39.60791	11	39.60791	35

36	39.62837	36	39.62837	26	39.62837	12	39.62837	29
33	39.64883	37	39.64883	26	39.64883	12	39.64883	31
29	39.6693	30	39.6693	15	39.6693	12	39.6693	21
26	39.68976	27	39.68976	19	39.68976	12	39.68976	18
22	39.71022	25	39.71022	25	39.71022	11	39.71022	16
21	39.73069	18	39.73069	30	39.73069	10	39.73069	20
34	39.75115	15	39.75115	33	39.75115	10	39.75115	24
34	39.77161	13	39.77161	29	39.77161	10	39.77161	26
37	39.79207	11	39.79207	27	39.79207	9	39.79207	31
32	39.81254	5	39.81254	24	39.81254	10	39.81254	30
24	39.833	8	39.833	23	39.833	10	39.833	24
24	39.85346	8	39.85346	16	39.85346	10	39.85346	20
21	39.87392	14	39.87392	10	39.87392	11	39.87392	11
17	39.89439	21	39.89439	15	39.89439	11	39.89439	3
18	39.91485	20	39.91485	16	39.91485	11	39.91485	8
24	39.93531	21	39.93531	13	39.93531	11	39.93531	8
31	39.95578	20	39.95578	12	39.95578	11	39.95578	6
28	39.97624	19	39.97624	6	39.97624	11	39.97624	11
25	39.9967	12	39.9967	6	39.9967	12	39.9967	21
17	40.01717	6	40.01717	9	40.01717	13	40.01717	25
15	40.03763	4	40.03763	18	40.03763	14	40.03763	26
15	40.05809	8	40.05809	21	40.05809	16	40.05809	19
13	40.07855	13	40.07855	24	40.07855	17	40.07855	19
16	40.09901	14	40.09901	23	40.09901	18	40.09901	16
24	40.11948	12	40.11948	21	40.11948	19	40.11948	18
28	40.13994	14	40.13994	23	40.13994	20	40.13994	13
34	40.1604	12	40.1604	24	40.1604	20	40.1604	13
33	40.18087	10	40.18087	24	40.18087	20	40.18087	16
29	40.20133	6	40.20133	26	40.20133	21	40.20133	15
21	40.22179	3	40.22179	19	40.22179	21	40.22179	17
23	40.24226	0	40.24226	27	40.24226	22	40.24226	16
16	40.26272	1	40.26272	31	40.26272	23	40.26272	15
20	40.28318	5	40.28318	40	40.28318	25	40.28318	14
26	40.30364	9	40.30364	37	40.30364	26	40.30364	21
31	40.3241	10	40.3241	43	40.3241	27	40.3241	24
44	40.34457	12	40.34457	44	40.34457	28	40.34457	21
46	40.36503	6	40.36503	42	40.36503	29	40.36503	18
53	40.38549	7	40.38549	41	40.38549	29	40.38549	18
53	40.40596	16	40.40596	32	40.40596	29	40.40596	12
60	40.42642	20	40.42642	22	40.42642	29	40.42642	15
60	40.44688	23	40.44688	23	40.44688	28	40.44688	18
59	40.46735	23	40.46735	24	40.46735	26	40.46735	21
68	40.48781	22	40.48781	23	40.48781	24	40.48781	13
65	40.50827	19	40.50827	17	40.50827	21	40.50827	20
63	40.52873	20	40.52873	24	40.52873	19	40.52873	21
75	40.5492	12	40.5492	30	40.5492	17	40.5492	26
77	40.56966	10	40.56966	32	40.56966	15	40.56966	27
101	40.59012	17	40.59012	36	40.59012	15	40.59012	29
121	40.61058	25	40.61058	39	40.61058	15	40.61058	27
139	40.63105	23	40.63105	36	40.63105	16	40.63105	27
142	40.65151	22	40.65151	35	40.65151	17	40.65151	25
157	40.67197	14	40.67197	31	40.67197	19	40.67197	22
161	40.69244	8	40.69244	30	40.69244	20	40.69244	11
147	40.7129	15	40.7129	45	40.7129	21	40.7129	15
130	40.73336	18	40.73336	63	40.73336	21	40.73336	16
115	40.75383	21	40.75383	70	40.75383	21	40.75383	23
95	40.77429	33	40.77429	62	40.77429	20	40.77429	22
77	40.79475	42	40.79475	61	40.79475	18	40.79475	17

63	40.81521	45	40.81521	56	40.81521	16	40.81521	14
53	40.83567	42	40.83567	49	40.83567	15	40.83567	14
49	40.85614	34	40.85614	51	40.85614	14	40.85614	11
57	40.8766	22	40.8766	50	40.8766	13	40.8766	15
61	40.89706	18	40.89706	64	40.89706	14	40.89706	17
63	40.91753	15	40.91753	71	40.91753	15	40.91753	23
56	40.93799	10	40.93799	74	40.93799	17	40.93799	25
49	40.95845	14	40.95845	72	40.95845	18	40.95845	27
38	40.97892	12	40.97892	69	40.97892	19	40.97892	26
33	40.99938	19	40.99938	75	40.99938	20	40.99938	22
32	41.01984	25	41.01984	76	41.01984	20	41.01984	12
31	41.0403	25	41.0403	86	41.0403	19	41.0403	14
32	41.06076	17	41.06076	102	41.06076	18	41.06076	16
29	41.08123	18	41.08123	101	41.08123	17	41.08123	21
25	41.10169	18	41.10169	99	41.10169	16	41.10169	27
18	41.12215	17	41.12215	81	41.12215	15	41.12215	21
12	41.14262	23	41.14262	80	41.14262	13	41.14262	27
9	41.16308	18	41.16308	74	41.16308	11	41.16308	29
9	41.18354	23	41.18354	73	41.18354	9	41.18354	30
15	41.20401	30	41.20401	82	41.20401	7	41.20401	34
12	41.22447	35	41.22447	91	41.22447	5	41.22447	28
12	41.24493	35	41.24493	91	41.24493	5	41.24493	33
14	41.26539	26	41.26539	98	41.26539	5	41.26539	39
17	41.28586	31	41.28586	101	41.28586	7	41.28586	37
16	41.30632	29	41.30632	100	41.30632	9	41.30632	34
23	41.32678	29	41.32678	102	41.32678	13	41.32678	27
34	41.34724	38	41.34724	112	41.34724	16	41.34724	27
37	41.36771	36	41.36771	109	41.36771	20	41.36771	18
41	41.38817	46	41.38817	105	41.38817	22	41.38817	11
49	41.40863	49	41.40863	94	41.40863	22	41.40863	9
44	41.4291	49	41.4291	77	41.4291	21	41.4291	6
46	41.44956	45	41.44956	49	41.44956	19	41.44956	12
42	41.47002	35	41.47002	42	41.47002	17	41.47002	17
36	41.49049	31	41.49049	36	41.49049	14	41.49049	13
34	41.51095	22	41.51095	31	41.51095	11	41.51095	12
38	41.53141	18	41.53141	38	41.53141	9	41.53141	18
36	41.55187	19	41.55187	51	41.55187	9	41.55187	23
32	41.57233	15	41.57233	56	41.57233	9	41.57233	32
31	41.5928	12	41.5928	63	41.5928	10	41.5928	39
39	41.61326	25	41.61326	69	41.61326	12	41.61326	46
30	41.63372	41	41.63372	72	41.63372	14	41.63372	48
36	41.65419	50	41.65419	74	41.65419	16	41.65419	44
35	41.67465	52	41.67465	66	41.67465	17	41.67465	39
36	41.69511	60	41.69511	66	41.69511	17	41.69511	26
45	41.71558	66	41.71558	66	41.71558	17	41.71558	23
50	41.73604	70	41.73604	65	41.73604	16	41.73604	24
68	41.7565	62	41.7565	66	41.7565	14	41.7565	20
86	41.77696	46	41.77696	56	41.77696	13	41.77696	27
115	41.79742	35	41.79742	57	41.79742	11	41.79742	28
140	41.81789	30	41.81789	58	41.81789	10	41.81789	27
171	41.83835	31	41.83835	50	41.83835	10	41.83835	21
213	41.85881	24	41.85881	36	41.85881	9	41.85881	12
233	41.87928	18	41.87928	19	41.87928	9	41.87928	9
243	41.89974	28	41.89974	17	41.89974	9	41.89974	4
244	41.9202	37	41.9202	22	41.9202	9	41.9202	6
235	41.94067	45	41.94067	32	41.94067	10	41.94067	0
229	41.96113	52	41.96113	31	41.96113	11	41.96113	0
212	41.98159	49	41.98159	32	41.98159	13	41.98159	8

197	42.00205	50	42.00205	40	42.00205	15	42.00205	9
178	42.02252	42	42.02252	53	42.02252	18	42.02252	13
168	42.04298	46	42.04298	56	42.04298	20	42.04298	18
153	42.06344	49	42.06344	54	42.06344	22	42.06344	25
132	42.0839	55	42.0839	59	42.0839	23	42.0839	31
117	42.10437	57	42.10437	63	42.10437	23	42.10437	33
97	42.12483	51	42.12483	75	42.12483	22	42.12483	28
72	42.14529	50	42.14529	69	42.14529	21	42.14529	21
53	42.16576	46	42.16576	53	42.16576	19	42.16576	21
40	42.18622	37	42.18622	46	42.18622	17	42.18622	19
40	42.20668	37	42.20668	44	42.20668	15	42.20668	10
44	42.22715	36	42.22715	48	42.22715	12	42.22715	3
44	42.24761	39	42.24761	43	42.24761	10	42.24761	3
45	42.26807	47	42.26807	32	42.26807	8	42.26807	11
42	42.28853	56	42.28853	31	42.28853	7	42.28853	16
40	42.30899	54	42.30899	38	42.30899	7	42.30899	17
34	42.32946	47	42.32946	38	42.32946	7	42.32946	15
33	42.34992	40	42.34992	31	42.34992	10	42.34992	14
32	42.37038	37	42.37038	35	42.37038	13	42.37038	13
33	42.39085	32	42.39085	41	42.39085	17	42.39085	11
47	42.41131	40	42.41131	46	42.41131	21	42.41131	7
61	42.43177	41	42.43177	50	42.43177	25	42.43177	0
67	42.45224	51	42.45224	38	42.45224	28	42.45224	0
70	42.4727	54	42.4727	33	42.4727	30	42.4727	12
70	42.49316	58	42.49316	29	42.49316	29	42.49316	18
83	42.51362	60	42.51362	36	42.51362	28	42.51362	21
108	42.53408	64	42.53408	33	42.53408	25	42.53408	22
122	42.55455	66	42.55455	29	42.55455	23	42.55455	17
124	42.57501	67	42.57501	39	42.57501	21	42.57501	17
136	42.59547	60	42.59547	38	42.59547	20	42.59547	14
166	42.61594	53	42.61594	35	42.61594	21	42.61594	6
206	42.6364	48	42.6364	36	42.6364	22	42.6364	0
259	42.65686	43	42.65686	30	42.65686	24	42.65686	4
323	42.67733	29	42.67733	35	42.67733	26	42.67733	20
414	42.69779	29	42.69779	24	42.69779	27	42.69779	25
523	42.71825	38	42.71825	23	42.71825	28	42.71825	34
645	42.73871	41	42.73871	24	42.73871	27	42.73871	38
738	42.75918	48	42.75918	16	42.75918	27	42.75918	36
796	42.77964	57	42.77964	23	42.77964	27	42.77964	34
848	42.8001	57	42.8001	16	42.8001	27	42.8001	25
876	42.82056	52	42.82056	11	42.82056	28	42.82056	11
880	42.84103	47	42.84103	21	42.84103	29	42.84103	0
851	42.86149	38	42.86149	36	42.86149	30	42.86149	4
792	42.88195	34	42.88195	39	42.88195	31	42.88195	7
739	42.90242	34	42.90242	37	42.90242	30	42.90242	6
671	42.92288	29	42.92288	43	42.92288	29	42.92288	17
601	42.94334	23	42.94334	55	42.94334	26	42.94334	20
532	42.96381	31	42.96381	55	42.96381	22	42.96381	25
459	42.98427	43	42.98427	56	42.98427	18	42.98427	27
412	43.00473	43	43.00473	53	43.00473	15	43.00473	24
376	43.02519	40	43.02519	44	43.02519	12	43.02519	15
359	43.04565	43	43.04565	42	43.04565	12	43.04565	12
331	43.06612	47	43.06612	39	43.06612	13	43.06612	13
327	43.08658	42	43.08658	28	43.08658	16	43.08658	25
355	43.10704	33	43.10704	18	43.10704	20	43.10704	30
402	43.12751	27	43.12751	16	43.12751	24	43.12751	38
470	43.14797	38	43.14797	13	43.14797	28	43.14797	40
569	43.16843	39	43.16843	9	43.16843	32	43.16843	38

697	43.1889	43	43.1889	7	43.1889	35	43.1889	44
868	43.20936	45	43.20936	9	43.20936	37	43.20936	41
1096	43.22982	44	43.22982	7	43.22982	37	43.22982	33
1364	43.25028	47	43.25028	4	43.25028	36	43.25028	26
1602	43.27074	54	43.27074	5	43.27074	34	43.27074	25
1839	43.29121	48	43.29121	5	43.29121	31	43.29121	28
2028	43.31167	40	43.31167	1	43.31167	27	43.31167	28
2161	43.33213	35	43.33213	14	43.33213	23	43.33213	30
2197	43.3526	29	43.3526	18	43.3526	19	43.3526	27
2179	43.37306	22	43.37306	22	43.37306	16	43.37306	15
2113	43.39352	26	43.39352	30	43.39352	14	43.39352	25
2022	43.41399	30	43.41399	35	43.41399	13	43.41399	24
1926	43.43445	31	43.43445	32	43.43445	14	43.43445	23
1817	43.45491	37	43.45491	36	43.45491	16	43.45491	14
1683	43.47537	42	43.47537	36	43.47537	19	43.47537	17
1557	43.49584	48	43.49584	24	43.49584	21	43.49584	19
1418	43.5163	43	43.5163	18	43.5163	22	43.5163	19
1287	43.53676	37	43.53676	23	43.53676	22	43.53676	22
1127	43.55722	39	43.55722	24	43.55722	20	43.55722	16
997	43.57769	43	43.57769	28	43.57769	17	43.57769	16
862	43.59815	45	43.59815	30	43.59815	14	43.59815	19
729	43.61861	42	43.61861	29	43.61861	11	43.61861	17
598	43.63908	36	43.63908	26	43.63908	9	43.63908	22
484	43.65954	32	43.65954	24	43.65954	9	43.65954	21
398	43.68	20	43.68	30	43.68	10	43.68	31
323	43.70047	14	43.70047	29	43.70047	13	43.70047	32
267	43.72093	8	43.72093	29	43.72093	17	43.72093	27
228	43.74139	8	43.74139	28	43.74139	21	43.74139	29
190	43.76185	14	43.76185	26	43.76185	24	43.76185	19
167	43.78231	12	43.78231	22	43.78231	26	43.78231	21
139	43.80278	11	43.80278	17	43.80278	27	43.80278	10
109	43.82324	9	43.82324	17	43.82324	27	43.82324	13
91	43.8437	16	43.8437	15	43.8437	26	43.8437	15
84	43.86417	18	43.86417	11	43.86417	26	43.86417	22
82	43.88463	20	43.88463	23	43.88463	26	43.88463	32
75	43.90509	23	43.90509	30	43.90509	28	43.90509	35
78	43.92556	28	43.92556	28	43.92556	30	43.92556	32
95	43.94602	29	43.94602	31	43.94602	33	43.94602	25
102	43.96648	29	43.96648	33	43.96648	36	43.96648	19
97	43.98694	24	43.98694	41	43.98694	39	43.98694	25
96	44.0074	17	44.0074	50	44.0074	42	44.0074	23
103	44.02787	9	44.02787	57	44.02787	43	44.02787	35
113	44.04833	9	44.04833	50	44.04833	42	44.04833	33
131	44.06879	8	44.06879	52	44.06879	41	44.06879	39
160	44.08926	6	44.08926	49	44.08926	39	44.08926	38
197	44.10972	18	44.10972	38	44.10972	36	44.10972	31
240	44.13018	30	44.13018	31	44.13018	34	44.13018	29
278	44.15065	43	44.15065	31	44.15065	32	44.15065	25
286	44.17111	48	44.17111	30	44.17111	30	44.17111	25
288	44.19157	46	44.19157	25	44.19157	29	44.19157	36
283	44.21203	40	44.21203	23	44.21203	28	44.21203	37
269	44.2325	29	44.2325	23	44.2325	28	44.2325	41
245	44.25296	22	44.25296	17	44.25296	28	44.25296	36
224	44.27342	12	44.27342	28	44.27342	28	44.27342	23
216	44.29388	0	44.29388	26	44.29388	28	44.29388	20
191	44.31435	4	44.31435	27	44.31435	28	44.31435	14
158	44.33481	12	44.33481	37	44.33481	28	44.33481	19
124	44.35527	17	44.35527	38	44.35527	29	44.35527	18

107	44.37574	21	44.37574	36	44.37574	30	44.37574	16
95	44.3962	23	44.3962	28	44.3962	32	44.3962	24
91	44.41666	24	44.41666	24	44.41666	34	44.41666	37
94	44.43713	26	44.43713	20	44.43713	36	44.43713	49
101	44.45759	22	44.45759	15	44.45759	37	44.45759	54
114	44.47805	15	44.47805	17	44.47805	37	44.47805	49
124	44.49851	12	44.49851	17	44.49851	36	44.49851	47
121	44.51897	11	44.51897	20	44.51897	34	44.51897	42
135	44.53944	10	44.53944	25	44.53944	32	44.53944	32
156	44.5599	9	44.5599	33	44.5599	30	44.5599	18
207	44.58036	6	44.58036	39	44.58036	28	44.58036	12
263	44.60083	6	44.60083	43	44.60083	26	44.60083	12
334	44.62129	6	44.62129	49	44.62129	26	44.62129	19
410	44.64175	8	44.64175	45	44.64175	27	44.64175	22
466	44.66222	10	44.66222	43	44.66222	28	44.66222	21
505	44.68268	14	44.68268	39	44.68268	29	44.68268	17
514	44.70314	18	44.70314	33	44.70314	30	44.70314	2
514	44.7236	22	44.7236	21	44.7236	30	44.7236	9
489	44.74406	22	44.74406	13	44.74406	31	44.74406	6
451	44.76453	18	44.76453	8	44.76453	31	44.76453	13
422	44.78499	13	44.78499	11	44.78499	30	44.78499	17
389	44.80545	10	44.80545	16	44.80545	30	44.80545	25
355	44.82592	6	44.82592	23	44.82592	30	44.82592	35
322	44.84638	4	44.84638	18	44.84638	29	44.84638	42
273	44.86684	2	44.86684	34	44.86684	28	44.86684	42
234	44.88731	2	44.88731	37	44.88731	26	44.88731	38
192	44.90777	3	44.90777	37	44.90777	23	44.90777	42
150	44.92823	3	44.92823	36	44.92823	20	44.92823	56
112	44.94869	5	44.94869	31	44.94869	17	44.94869	59
81	44.96916	5	44.96916	35	44.96916	13	44.96916	68
71	44.98962	6	44.98962	39	44.98962	10	44.98962	56
63	45.01008	7	45.01008	32	45.01008	9	45.01008	57
53	45.03054	13	45.03054	30	45.03054	8	45.03054	58
46	45.05101	19	45.05101	23	45.05101	8	45.05101	53
40	45.07147	26	45.07147	26	45.07147	10	45.07147	43
42	45.09193	31	45.09193	17	45.09193	12	45.09193	38
39	45.1124	29	45.1124	11	45.1124	14	45.1124	40
31	45.13286	29	45.13286	2	45.13286	15	45.13286	31
26	45.15332	28	45.15332	2	45.15332	17	45.15332	23
14	45.17379	24	45.17379	10	45.17379	17	45.17379	27
10	45.19425	17	45.19425	12	45.19425	18	45.19425	22
13	45.21471	13	45.21471	12	45.21471	18	45.21471	30
10	45.23517	10	45.23517	13	45.23517	19	45.23517	40
11	45.25563	10	45.25563	16	45.25563	20	45.25563	50
11	45.2761	18	45.2761	26	45.2761	22	45.2761	52
17	45.29656	19	45.29656	36	45.29656	23	45.29656	55
22	45.31702	14	45.31702	40	45.31702	24	45.31702	51
24	45.33749	16	45.33749	44	45.33749	25	45.33749	43
36	45.35795	18	45.35795	50	45.35795	24	45.35795	34
39	45.37841	22	45.37841	49	45.37841	23	45.37841	34
46	45.39888	24	45.39888	43	45.39888	21	45.39888	27
62	45.41934	29	45.41934	35	45.41934	19	45.41934	23
73	45.4398	30	45.4398	31	45.4398	18	45.4398	28
86	45.46026	26	45.46026	29	45.46026	18	45.46026	31
98	45.48072	37	45.48072	33	45.48072	20	45.48072	31
108	45.50119	38	45.50119	31	45.50119	22	45.50119	40
118	45.52165	31	45.52165	28	45.52165	25	45.52165	43
119	45.54211	26	45.54211	26	45.54211	27	45.54211	51

128	45.56258	23	45.56258	27	45.56258	28	45.56258	59
121	45.58304	14	45.58304	18	45.58304	27	45.58304	63
119	45.6035	9	45.6035	22	45.6035	24	45.6035	54
116	45.62397	13	45.62397	28	45.62397	20	45.62397	43
108	45.64443	12	45.64443	33	45.64443	16	45.64443	33
97	45.66489	7	45.66489	30	45.66489	12	45.66489	25
80	45.68535	26	45.68535	28	45.68535	9	45.68535	25
56	45.70582	31	45.70582	19	45.70582	8	45.70582	31
35	45.72628	33	45.72628	8	45.72628	9	45.72628	32
20	45.74674	36	45.74674	2	45.74674	11	45.74674	39
11	45.7672	32	45.7672	8	45.7672	14	45.7672	42
1	45.78767	23	45.78767	4	45.78767	17	45.78767	45
2	45.80813	19	45.80813	12	45.80813	20	45.80813	46
15	45.82859	15	45.82859	14	45.82859	22	45.82859	41
24	45.84906	6	45.84906	12	45.84906	24	45.84906	31
31	45.86952	3	45.86952	12	45.86952	24	45.86952	25
33	45.88998	6	45.88998	9	45.88998	23	45.88998	22
33	45.91045	7	45.91045	13	45.91045	22	45.91045	22
33	45.93091	13	45.93091	17	45.93091	20	45.93091	17
31	45.95137	18	45.95137	21	45.95137	18	45.95137	16
29	45.97183	17	45.97183	28	45.97183	17	45.97183	12
20	45.99229	18	45.99229	29	45.99229	15	45.99229	16
18	46.01276	22	46.01276	28	46.01276	15	46.01276	24
13	46.03322	33	46.03322	20	46.03322	14	46.03322	26
11	46.05368	39	46.05368	14	46.05368	15	46.05368	19
13	46.07415	43	46.07415	9	46.07415	17	46.07415	20
15	46.09461	41	46.09461	2	46.09461	20	46.09461	18
21	46.11507	38	46.11507	4	46.11507	24	46.11507	21
25	46.13554	33	46.13554	6	46.13554	28	46.13554	24
22	46.156	25	46.156	5	46.156	32	46.156	26
32	46.17646	13	46.17646	6	46.17646	35	46.17646	32
30	46.19692	4	46.19692	3	46.19692	36	46.19692	45
28	46.21738	7	46.21738	0	46.21738	35	46.21738	53
21	46.23785	14	46.23785	3	46.23785	32	46.23785	56
15	46.25831	18	46.25831	9	46.25831	27	46.25831	44
9	46.27877	22	46.27877	13	46.27877	21	46.27877	49
12	46.29924	21	46.29924	13	46.29924	15	46.29924	38
11	46.3197	14	46.3197	14	46.3197	10	46.3197	34
8	46.34016	15	46.34016	18	46.34016	7	46.34016	30
4	46.36063	18	46.36063	19	46.36063	7	46.36063	19
7	46.38109	9	46.38109	17	46.38109	8	46.38109	26
6	46.40155	13	46.40155	11	46.40155	11	46.40155	31
1	46.42201	25	46.42201	6	46.42201	14	46.42201	22
8	46.44248	24	46.44248	6	46.44248	18	46.44248	24
10	46.46294	29	46.46294	8	46.46294	21	46.46294	16
12	46.4834	32	46.4834	8	46.4834	24	46.4834	21
18	46.50386	35	46.50386	9	46.50386	25	46.50386	13
19	46.52433	33	46.52433	8	46.52433	24	46.52433	14
15	46.54479	32	46.54479	18	46.54479	23	46.54479	16
10	46.56525	24	46.56525	26	46.56525	20	46.56525	14
14	46.58572	13	46.58572	26	46.58572	17	46.58572	21
13	46.60618	20	46.60618	23	46.60618	14	46.60618	21
6	46.62664	22	46.62664	24	46.62664	12	46.62664	16
16	46.64711	12	46.64711	29	46.64711	10	46.64711	16
18	46.66757	11	46.66757	30	46.66757	9	46.66757	16
19	46.68803	19	46.68803	31	46.68803	9	46.68803	10
25	46.70849	31	46.70849	29	46.70849	11	46.70849	10
32	46.72895	33	46.72895	25	46.72895	13	46.72895	19

41	46.74942	30	46.74942	34	46.74942	16	46.74942	25
55	46.76988	28	46.76988	27	46.76988	20	46.76988	24
70	46.79034	27	46.79034	23	46.79034	24	46.79034	31
82	46.81081	23	46.81081	31	46.81081	28	46.81081	28
85	46.83127	19	46.83127	36	46.83127	30	46.83127	35
93	46.85173	12	46.85173	41	46.85173	31	46.85173	38
91	46.8722	8	46.8722	42	46.8722	30	46.8722	38
88	46.89266	12	46.89266	39	46.89266	28	46.89266	31
94	46.91312	19	46.91312	37	46.91312	26	46.91312	33
92	46.93358	20	46.93358	36	46.93358	23	46.93358	30
93	46.95404	15	46.95404	33	46.95404	21	46.95404	21
91	46.97451	19	46.97451	18	46.97451	19	46.97451	10
83	46.99497	20	46.99497	15	46.99497	17	46.99497	9
74	47.01543	20	47.01543	17	47.01543	15	47.01543	8
68	47.0359	29	47.0359	9	47.0359	13	47.0359	20
67	47.05636	36	47.05636	10	47.05636	12	47.05636	26
56	47.07682	36	47.07682	13	47.07682	10	47.07682	26
57	47.09729	44	47.09729	15	47.09729	9	47.09729	23
62	47.11775	48	47.11775	25	47.11775	9	47.11775	18
68	47.13821	45	47.13821	38	47.13821	9	47.13821	19
88	47.15867	36	47.15867	47	47.15867	11	47.15867	23
123	47.17914	33	47.17914	54	47.17914	13	47.17914	23
172	47.1996	27	47.1996	59	47.1996	15	47.1996	26
230	47.22006	26	47.22006	63	47.22006	18	47.22006	33
305	47.24052	23	47.24052	67	47.24052	21	47.24052	31
371	47.26099	22	47.26099	62	47.26099	22	47.26099	35
442	47.28145	22	47.28145	58	47.28145	23	47.28145	31
493	47.30191	29	47.30191	52	47.30191	24	47.30191	20
530	47.32238	30	47.32238	46	47.32238	23	47.32238	15
545	47.34284	31	47.34284	53	47.34284	22	47.34284	17
547	47.3633	27	47.3633	52	47.3633	22	47.3633	20
538	47.38377	21	47.38377	54	47.38377	21	47.38377	18
511	47.40423	21	47.40423	59	47.40423	20	47.40423	20
474	47.42469	22	47.42469	67	47.42469	20	47.42469	18
430	47.44515	11	47.44515	78	47.44515	20	47.44515	16
376	47.46561	10	47.46561	80	47.46561	20	47.46561	20
330	47.48608	17	47.48608	79	47.48608	20	47.48608	17
259	47.50654	20	47.50654	84	47.50654	20	47.50654	16
205	47.527	25	47.527	91	47.527	20	47.527	21
151	47.54747	22	47.54747	89	47.54747	19	47.54747	23
109	47.56793	29	47.56793	80	47.56793	19	47.56793	25
77	47.58839	34	47.58839	76	47.58839	19	47.58839	25
47	47.60886	38	47.60886	64	47.60886	18	47.60886	18
39	47.62932	33	47.62932	54	47.62932	18	47.62932	11
30	47.64978	22	47.64978	54	47.64978	18	47.64978	12
29	47.67024	21	47.67024	58	47.67024	17	47.67024	6
29	47.6907	22	47.6907	55	47.6907	16	47.6907	11
18	47.71117	14	47.71117	69	47.71117	15	47.71117	18
25	47.73163	10	47.73163	68	47.73163	15	47.73163	23
24	47.75209	16	47.75209	64	47.75209	14	47.75209	21
20	47.77256	27	47.77256	56	47.77256	15	47.77256	24
21	47.79302	33	47.79302	50	47.79302	16	47.79302	30
17	47.81348	37	47.81348	38	47.81348	19	47.81348	27
20	47.83395	40	47.83395	23	47.83395	21	47.83395	29
15	47.85441	38	47.85441	23	47.85441	22	47.85441	27
13	47.87487	28	47.87487	24	47.87487	23	47.87487	24
11	47.89533	34	47.89533	30	47.89533	22	47.89533	25
5	47.9158	33	47.9158	41	47.9158	21	47.9158	13

7	47.93626	27	47.93626	45	47.93626	19	47.93626	12
5	47.95672	29	47.95672	50	47.95672	16	47.95672	12
0	47.97718	29	47.97718	51	47.97718	15	47.97718	15
2	47.99765	34	47.99765	50	47.99765	14	47.99765	24
10	48.01811	38	48.01811	43	48.01811	14	48.01811	25
21	48.03857	37	48.03857	31	48.03857	14	48.03857	28
29	48.05904	35	48.05904	20	48.05904	14	48.05904	21
43	48.0795	28	48.0795	13	48.0795	14	48.0795	20
62	48.09996	23	48.09996	16	48.09996	13	48.09996	17
71	48.12043	27	48.12043	17	48.12043	11	48.12043	11
77	48.14089	26	48.14089	19	48.14089	10	48.14089	10
77	48.16135	27	48.16135	29	48.16135	8	48.16135	13
69	48.18181	38	48.18181	37	48.18181	8	48.18181	12
58	48.20227	37	48.20227	38	48.20227	9	48.20227	15
55	48.22274	39	48.22274	25	48.22274	11	48.22274	14
55	48.2432	37	48.2432	28	48.2432	14	48.2432	17
49	48.26366	37	48.26366	29	48.26366	18	48.26366	13
50	48.28413	26	48.28413	32	48.28413	21	48.28413	19
54	48.30459	19	48.30459	28	48.30459	25	48.30459	21
60	48.32505	34	48.32505	26	48.32505	27	48.32505	19
71	48.34552	33	48.34552	25	48.34552	28	48.34552	8
75	48.36598	32	48.36598	29	48.36598	27	48.36598	13
82	48.38644	35	48.38644	24	48.38644	26	48.38644	17
98	48.4069	35	48.4069	13	48.4069	23	48.4069	17
132	48.42736	38	48.42736	2	48.42736	19	48.42736	18
177	48.44783	39	48.44783	5	48.44783	15	48.44783	21
234	48.46829	36	48.46829	5	48.46829	11	48.46829	17
304	48.48875	31	48.48875	4	48.48875	8	48.48875	19
407	48.50922	32	48.50922	4	48.50922	5	48.50922	14
527	48.52968	27	48.52968	15	48.52968	4	48.52968	10
652	48.55014	22	48.55014	20	48.55014	3	48.55014	3
777	48.57061	18	48.57061	23	48.57061	4	48.57061	8
895	48.59107	14	48.59107	22	48.59107	6	48.59107	14
998	48.61153	14	48.61153	18	48.61153	8	48.61153	14
1102	48.63199	24	48.63199	14	48.63199	10	48.63199	21
1197	48.65246	34	48.65246	7	48.65246	12	48.65246	21
1296	48.67292	39	48.67292	4	48.67292	15	48.67292	29
1391	48.69338	38	48.69338	2	48.69338	17	48.69338	36
1489	48.71384	35	48.71384	9	48.71384	20	48.71384	40
1565	48.73431	31	48.73431	11	48.73431	22	48.73431	39
1611	48.75477	32	48.75477	22	48.75477	23	48.75477	44
1598	48.77523	33	48.77523	23	48.77523	23	48.77523	43
1534	48.7957	27	48.7957	31	48.7957	23	48.7957	38
1429	48.81616	21	48.81616	36	48.81616	21	48.81616	29
1307	48.83662	27	48.83662	39	48.83662	18	48.83662	22
1187	48.85709	24	48.85709	46	48.85709	15	48.85709	13
1054	48.87755	22	48.87755	48	48.87755	12	48.87755	10
929	48.89801	20	48.89801	44	48.89801	10	48.89801	11
810	48.91847	22	48.91847	39	48.91847	8	48.91847	11
689	48.93893	24	48.93893	27	48.93893	7	48.93893	8
566	48.9594	32	48.9594	18	48.9594	7	48.9594	20
437	48.97986	33	48.97986	9	48.97986	7	48.97986	24
330	49.00032	38	49.00032	15	49.00032	8	49.00032	21
231	49.02079	36	49.02079	18	49.02079	8	49.02079	22
164	49.04125	31	49.04125	22	49.04125	9	49.04125	19
119	49.06171	22	49.06171	22	49.06171	9	49.06171	20
76	49.08218	21	49.08218	23	49.08218	9	49.08218	12
55	49.10264	19	49.10264	25	49.10264	9	49.10264	14

40	49.1231	18	49.1231	20	49.1231	11	49.1231	9
24	49.14356	20	49.14356	9	49.14356	13	49.14356	11
11	49.16402	19	49.16402	4	49.16402	16	49.16402	14
7	49.18449	14	49.18449	12	49.18449	19	49.18449	18
0	49.20495	18	49.20495	14	49.20495	21	49.20495	22
0	49.22541	17	49.22541	17	49.22541	23	49.22541	29
5	49.24588	12	49.24588	19	49.24588	23	49.24588	28
27	49.26634	10	49.26634	18	49.26634	21	49.26634	26
55	49.2868	8	49.2868	23	49.2868	19	49.2868	26
104	49.30727	3	49.30727	29	49.30727	16	49.30727	25
163	49.32773	9	49.32773	30	49.32773	13	49.32773	19
213	49.34819	11	49.34819	18	49.34819	10	49.34819	19
260	49.36865	8	49.36865	26	49.36865	9	49.36865	10
285	49.38912	10	49.38912	34	49.38912	8	49.38912	17
286	49.40958	7	49.40958	35	49.40958	9	49.40958	21
285	49.43004	12	49.43004	37	49.43004	9	49.43004	19
273	49.4505	13	49.4505	34	49.4505	10	49.4505	16
256	49.47097	11	49.47097	27	49.47097	11	49.47097	13
218	49.49143	8	49.49143	19	49.49143	11	49.49143	11
195	49.51189	4	49.51189	17	49.51189	12	49.51189	11
165	49.53236	5	49.53236	9	49.53236	12	49.53236	8
125	49.55282	3	49.55282	4	49.55282	12	49.55282	9
91	49.57328	9	49.57328	12	49.57328	12	49.57328	11
54	49.59375	22	49.59375	18	49.59375	12	49.59375	22
24	49.61421	28	49.61421	21	49.61421	11	49.61421	27
9	49.63467	29	49.63467	25	49.63467	10	49.63467	29
0	49.65513	35	49.65513	26	49.65513	9	49.65513	26
0	49.67559	37	49.67559	25	49.67559	7	49.67559	20
0	49.69606	40	49.69606	22	49.69606	5	49.69606	11
11	49.71652	36	49.71652	17	49.71652	4	49.71652	5
18	49.73698	30	49.73698	13	49.73698	4	49.73698	3
40	49.75745	24	49.75745	25	49.75745	3	49.75745	5
78	49.77791	24	49.77791	25	49.77791	4	49.77791	11
120	49.79837	21	49.79837	28	49.79837	5	49.79837	11
171	49.81884	12	49.81884	32	49.81884	5	49.81884	17
212	49.8393	0	49.8393	39	49.8393	6	49.8393	19
243	49.85976	0	49.85976	43	49.85976	6	49.85976	18
248	49.88022	0	49.88022	49	49.88022	5	49.88022	14
218	49.90068	1	49.90068	46	49.90068	5	49.90068	9
165	49.92115	1	49.92115	41	49.92115	3	49.92115	6
115	49.94161	1	49.94161	31	49.94161	3	49.94161	6
62	49.96207	0	49.96207	23	49.96207	2	49.96207	0
62	49.98254	0	49.98254	23	49.98254	2	49.98254	0
62	50.003	0	50.003	23	50.003	2	50.003	0

Figure 2b

2 Theta	Intensity						
degree	a.u.						
	MOF-Th(Exp.)		ReO4-	1	Dichrolometh	2	Trichlorometh
2	34	4	0	4	5	4	0
2.020462	34	4.020462	0	4.020462	5	4.020462	0
2.040925	34	4.040925	0	4.040925	5	4.040925	0
2.061388	34	4.061388	23	4.061388	5	4.061388	0
2.081851	0	4.081851	0	4.081851	15	4.081851	59
2.102313	4	4.102313	43	4.102313	19	4.102313	80
2.122776	0	4.122776	113	4.122776	26	4.122776	62
2.143239	0	4.143239	7	4.143239	31	4.143239	38
2.163702	0	4.163702	20	4.163702	31	4.163702	41
2.184165	0	4.184165	20	4.184165	23	4.184165	32
2.204627	0	4.204627	0	4.204627	17	4.204627	0
2.22509	22	4.22509	0	4.22509	19	4.22509	73
2.245553	45	4.245553	0	4.245553	25	4.245553	65
2.266016	56	4.266016	4	4.266016	16	4.266016	0
2.286478	61	4.286478	105	4.286478	28	4.286478	22
2.306941	74	4.306941	0	4.306941	29	4.306941	16
2.327404	73	4.327404	0	4.327404	30	4.327404	48
2.347867	46	4.347867	18	4.347867	32	4.347867	45
2.36833	46	4.36833	4	4.36833	21	4.36833	49
2.388792	54	4.388792	20	4.388792	19	4.388792	0
2.409255	70	4.409255	83	4.409255	18	4.409255	15
2.429718	92	4.429718	10	4.429718	20	4.429718	43
2.450181	116	4.450181	0	4.450181	25	4.450181	33
2.470643	120	4.470643	18	4.470643	28	4.470643	11
2.491106	104	4.491106	0	4.491106	38	4.491106	57
2.511569	94	4.511569	16	4.511569	31	4.511569	0
2.532032	88	4.532032	0	4.532032	24	4.532032	0
2.552495	68	4.552495	14	4.552495	33	4.552495	23
2.572957	77	4.572957	24	4.572957	39	4.572957	0
2.59342	74	4.59342	6	4.59342	43	4.59342	2
2.613883	55	4.613883	0	4.613883	44	4.613883	0
2.634346	22	4.634346	111	4.634346	50	4.634346	31
2.654808	48	4.654808	9	4.654808	62	4.654808	0
2.675271	59	4.675271	0	4.675271	61	4.675271	0
2.695734	46	4.695734	0	4.695734	49	4.695734	37
2.716197	62	4.716197	47	4.716197	28	4.716197	4
2.73666	88	4.73666	71	4.73666	16	4.73666	0
2.757122	88	4.757122	52	4.757122	21	4.757122	0
2.777585	77	4.777585	31	4.777585	17	4.777585	81
2.798048	55	4.798048	0	4.798048	10	4.798048	43
2.818511	32	4.818511	6	4.818511	15	4.818511	0
2.838973	0	4.838973	29	4.838973	15	4.838973	0
2.859436	4	4.859436	0	4.859436	17	4.859436	64
2.879899	2	4.879899	0	4.879899	22	4.879899	65
2.900362	0	4.900362	18	4.900362	28	4.900362	0
2.920825	0	4.920825	71	4.920825	32	4.920825	4
2.941287	0	4.941287	0	4.941287	33	4.941287	21
2.96175	0	4.96175	94	4.96175	49	4.96175	12
2.982213	0	4.982213	34	4.982213	49	4.982213	67
3.002676	0	5.002676	26	5.002676	52	5.002676	0
3.023139	0	5.023139	2	5.023139	43	5.023139	9

3.043602	0	5.043602	73	5.043602	32	5.043602	30
3.064064	11	5.064064	73	5.064064	27	5.064064	29
3.084527	34	5.084527	9	5.084527	21	5.084527	35
3.10499	50	5.10499	0	5.10499	22	5.10499	0
3.125453	52	5.125453	14	5.125453	18	5.125453	0
3.145916	62	5.145916	8	5.145916	15	5.145916	7
3.166378	62	5.166378	5	5.166378	16	5.166378	0
3.186841	53	5.186841	0	5.186841	13	5.186841	0
3.207304	36	5.207304	44	5.207304	10	5.207304	39
3.227767	23	5.227767	0	5.227767	10	5.227767	93
3.248229	9	5.248229	4	5.248229	13	5.248229	7
3.268692	4	5.268692	38	5.268692	7	5.268692	0
3.289155	8	5.289155	0	5.289155	3	5.289155	0
3.309618	0	5.309618	0	5.309618	16	5.309618	0
3.330081	0	5.330081	34	5.330081	17	5.330081	0
3.350543	12	5.350543	38	5.350543	18	5.350543	0
3.371006	27	5.371006	0	5.371006	20	5.371006	40
3.391469	36	5.391469	0	5.391469	46	5.391469	18
3.411932	41	5.411932	69	5.411932	60	5.411932	96
3.432394	41	5.432394	2	5.432394	77	5.432394	31
3.452857	34	5.452857	0	5.452857	81	5.452857	52
3.47332	27	5.47332	1	5.47332	93	5.47332	53
3.493783	17	5.493783	23	5.493783	93	5.493783	136
3.514246	0	5.514246	0	5.514246	106	5.514246	126
3.534708	0	5.534708	0	5.534708	106	5.534708	87
3.555171	4	5.555171	42	5.555171	114	5.555171	106
3.575634	9	5.575634	1	5.575634	116	5.575634	109
3.596097	13	5.596097	7	5.596097	142	5.596097	56
3.616559	14	5.616559	0	5.616559	135	5.616559	0
3.637022	12	5.637022	59	5.637022	120	5.637022	85
3.657485	11	5.657485	79	5.657485	92	5.657485	28
3.677948	12	5.677948	79	5.677948	94	5.677948	0
3.698411	9	5.698411	48	5.698411	88	5.698411	72
3.718873	4	5.718873	6	5.718873	97	5.718873	37
3.739336	0	5.739336	51	5.739336	113	5.739336	169
3.759799	0	5.759799	26	5.759799	140	5.759799	161
3.780262	6	5.780262	0	5.780262	169	5.780262	52
3.800724	10	5.800724	38	5.800724	199	5.800724	106
3.821187	12	5.821187	77	5.821187	226	5.821187	151
3.84165	11	5.84165	0	5.84165	235	5.84165	210
3.862113	14	5.862113	0	5.862113	235	5.862113	125
3.882576	15	5.882576	1	5.882576	259	5.882576	224
3.903038	10	5.903038	0	5.903038	278	5.903038	225
3.923501	9	5.923501	71	5.923501	300	5.923501	245
3.943964	6	5.943964	71	5.943964	330	5.943964	235
3.964427	0	5.964427	92	5.964427	374	5.964427	269
3.984889	11	5.984889	64	5.984889	427	5.984889	263
4.005352	20	6.005352	82	6.005352	471	6.005352	316
4.025815	23	6.025815	92	6.025815	537	6.025815	463
4.046278	23	6.046278	152	6.046278	577	6.046278	461
4.066741	29	6.066741	172	6.066741	625	6.066741	478
4.087203	29	6.087203	240	6.087203	694	6.087203	572
4.107666	23	6.107666	154	6.107666	764	6.107666	646
4.128129	23	6.128129	266	6.128129	848	6.128129	778
4.148592	15	6.148592	257	6.148592	959	6.148592	972
4.169055	0	6.169055	315	6.169055	1105	6.169055	1107
4.189517	11	6.189517	311	6.189517	1296	6.189517	1271
4.20998	14	6.20998	360	6.20998	1505	6.20998	1462

4.230443	14	6.230443	421	6.230443	1748	6.230443	1727
4.250906	9	6.250906	593	6.250906	1997	6.250906	1960
4.271368	19	6.271368	626	6.271368	2278	6.271368	2253
4.291831	22	6.291831	663	6.291831	2598	6.291831	2757
4.312294	21	6.312294	911	6.312294	2941	6.312294	3350
4.332757	12	6.332757	1193	6.332757	3331	6.332757	3877
4.35322	12	6.35322	1213	6.35322	3808	6.35322	4538
4.373682	19	6.373682	1496	6.373682	4396	6.373682	5366
4.394145	34	6.394145	1670	6.394145	5117	6.394145	6543
4.414608	39	6.414608	1835	6.414608	5994	6.414608	7751
4.435071	38	6.435071	2218	6.435071	7016	6.435071	9106
4.455533	28	6.455533	2615	6.455533	8195	6.455533	10623
4.475996	31	6.475996	3135	6.475996	9559	6.475996	12096
4.496459	30	6.496459	3558	6.496459	11081	6.496459	12566
4.516922	23	6.516922	4584	6.516922	12575	6.516922	12163
4.537385	3	6.537385	5445	6.537385	13886	6.537385	10962
4.557847	13	6.557847	6548	6.557847	14733	6.557847	8756
4.57831	18	6.57831	7996	6.57831	14935	6.57831	6777
4.598773	19	6.598773	9682	6.598773	14405	6.598773	4961
4.619236	31	6.619236	11866	6.619236	13225	6.619236	3726
4.639698	40	6.639698	14497	6.639698	11503	6.639698	3007
4.660161	38	6.660161	16677	6.660161	9521	6.660161	2180
4.680624	38	6.680624	17941	6.680624	7606	6.680624	1794
4.701087	46	6.701087	17962	6.701087	5954	6.701087	1445
4.72155	45	6.72155	16427	6.72155	4657	6.72155	1341
4.742012	35	6.742012	12885	6.742012	3732	6.742012	1116
4.762475	36	6.762475	8870	6.762475	3057	6.762475	944
4.782938	27	6.782938	5857	6.782938	2564	6.782938	954
4.803401	29	6.803401	4053	6.803401	2196	6.803401	837
4.823863	37	6.823863	3000	6.823863	1923	6.823863	640
4.844326	34	6.844326	2385	6.844326	1695	6.844326	596
4.864789	22	6.864789	1941	6.864789	1499	6.864789	618
4.885252	21	6.885252	1628	6.885252	1374	6.885252	560
4.905715	22	6.905715	1478	6.905715	1241	6.905715	466
4.926177	20	6.926177	1186	6.926177	1149	6.926177	584
4.94664	18	6.94664	984	6.94664	1072	6.94664	392
4.967103	15	6.967103	944	6.967103	1006	6.967103	462
4.987566	20	6.987566	748	6.987566	941	6.987566	457
5.008028	24	7.008028	605	7.008028	882	7.008028	450
5.028491	24	7.028491	575	7.028491	854	7.028491	485
5.048954	14	7.048954	457	7.048954	822	7.048954	502
5.069417	7	7.069417	445	7.069417	797	7.069417	509
5.08988	20	7.08988	432	7.08988	786	7.08988	502
5.110342	33	7.110342	370	7.110342	754	7.110342	566
5.130805	35	7.130805	370	7.130805	767	7.130805	625
5.151268	47	7.151268	406	7.151268	794	7.151268	642
5.171731	56	7.171731	363	7.171731	840	7.171731	756
5.192193	64	7.192193	395	7.192193	916	7.192193	846
5.212656	64	7.212656	414	7.212656	1018	7.212656	909
5.233119	57	7.233119	401	7.233119	1155	7.233119	1175
5.253582	37	7.253582	439	7.253582	1323	7.253582	1386
5.274045	19	7.274045	436	7.274045	1526	7.274045	1658
5.294507	23	7.294507	490	7.294507	1767	7.294507	1855
5.31497	24	7.31497	561	7.31497	1984	7.31497	2211
5.335433	20	7.335433	557	7.335433	2220	7.335433	2827
5.355896	28	7.355896	652	7.355896	2472	7.355896	3076
5.376358	33	7.376358	775	7.376358	2798	7.376358	3711
5.396821	29	7.396821	915	7.396821	3290	7.396821	4689

5.417284	30	7.417284	953	7.417284	3907	7.417284	5292
5.437747	21	7.437747	1256	7.437747	4765	7.437747	6192
5.458209	14	7.458209	1525	7.458209	5963	7.458209	6756
5.478672	2	7.478672	1707	7.478672	7316	7.478672	6205
5.499135	11	7.499135	2104	7.499135	8537	7.499135	5330
5.519598	14	7.519598	2713	7.519598	9236	7.519598	3930
5.540061	25	7.540061	3337	7.540061	9226	7.540061	2228
5.560523	27	7.560523	4133	7.560523	8517	7.560523	1415
5.580986	27	7.580986	5504	7.580986	7275	7.580986	1036
5.601449	23	7.601449	7049	7.601449	5710	7.601449	718
5.621912	11	7.621912	8075	7.621912	4041	7.621912	561
5.642374	16	7.642374	8623	7.642374	2645	7.642374	606
5.662837	20	7.662837	8639	7.662837	1721	7.662837	304
5.6833	16	7.6833	7820	7.6833	1239	7.6833	333
5.703763	32	7.703763	5339	7.703763	1013	7.703763	314
5.724226	29	7.724226	2957	7.724226	881	7.724226	292
5.744688	52	7.744688	1726	7.744688	756	7.744688	260
5.765151	63	7.765151	1175	7.765151	648	7.765151	163
5.785614	85	7.785614	947	7.785614	569	7.785614	141
5.806077	106	7.806077	668	7.806077	490	7.806077	178
5.826539	120	7.826539	532	7.826539	441	7.826539	189
5.847002	146	7.847002	473	7.847002	398	7.847002	155
5.867465	167	7.867465	353	7.867465	360	7.867465	0
5.887928	174	7.887928	358	7.887928	355	7.887928	137
5.908391	181	7.908391	246	7.908391	324	7.908391	178
5.928853	183	7.928853	261	7.928853	310	7.928853	33
5.949316	205	7.949316	183	7.949316	301	7.949316	180
5.969779	221	7.969779	238	7.969779	279	7.969779	161
5.990242	261	7.990242	176	7.990242	258	7.990242	140
6.010705	305	8.010705	149	8.010705	236	8.010705	120
6.031167	374	8.031167	158	8.031167	229	8.031167	85
6.05163	449	8.05163	142	8.05163	215	8.05163	69
6.072093	556	8.072093	103	8.072093	207	8.072093	78
6.092556	660	8.092556	116	8.092556	205	8.092556	50
6.113018	771	8.113018	103	8.113018	185	8.113018	55
6.133481	919	8.133481	136	8.133481	173	8.133481	70
6.153944	1081	8.153944	116	8.153944	164	8.153944	105
6.174407	1252	8.174407	31	8.174407	152	8.174407	94
6.19487	1499	8.19487	88	8.19487	149	8.19487	117
6.215332	1808	8.215332	29	8.215332	137	8.215332	92
6.235795	2199	8.235795	121	8.235795	130	8.235795	136
6.256258	2670	8.256258	96	8.256258	124	8.256258	120
6.276721	3228	8.276721	90	8.276721	109	8.276721	76
6.297183	3791	8.297183	46	8.297183	99	8.297183	60
6.317646	4449	8.317646	71	8.317646	83	8.317646	78
6.338109	5269	8.338109	104	8.338109	81	8.338109	27
6.358572	6300	8.358572	61	8.358572	79	8.358572	0
6.379035	7694	8.379035	0	8.379035	86	8.379035	105
6.399497	9421	8.399497	75	8.399497	95	8.399497	3
6.41996	11396	8.41996	12	8.41996	92	8.41996	17
6.440423	13245	8.440423	32	8.440423	89	8.440423	43
6.460886	14555	8.460886	44	8.460886	94	8.460886	42
6.481348	14866	8.481348	27	8.481348	90	8.481348	0
6.501811	14041	8.501811	109	8.501811	98	8.501811	138
6.522274	12315	8.522274	72	8.522274	86	8.522274	32
6.542737	9948	8.542737	3	8.542737	76	8.542737	44
6.5632	7386	8.5632	16	8.5632	76	8.5632	51
6.583662	5088	8.583662	112	8.583662	85	8.583662	76

6.604125	3299	8.604125	85	8.604125	84	8.604125	24
6.624588	2211	8.624588	45	8.624588	89	8.624588	25
6.645051	1619	8.645051	0	8.645051	86	8.645051	0
6.665513	1337	8.665513	35	8.665513	89	8.665513	0
6.685976	1113	8.685976	12	8.685976	97	8.685976	35
6.706439	952	8.706439	0	8.706439	97	8.706439	0
6.726902	827	8.726902	0	8.726902	73	8.726902	0
6.747365	723	8.747365	38	8.747365	53	8.747365	0
6.767827	651	8.767827	12	8.767827	44	8.767827	0
6.78829	582	8.78829	33	8.78829	41	8.78829	25
6.808753	525	8.808753	8	8.808753	34	8.808753	29
6.829216	461	8.829216	0	8.829216	28	8.829216	0
6.849678	412	8.849678	32	8.849678	32	8.849678	0
6.870141	382	8.870141	0	8.870141	45	8.870141	24
6.890604	353	8.890604	24	8.890604	53	8.890604	91
6.911067	330	8.911067	7	8.911067	61	8.911067	104
6.93153	302	8.93153	14	8.93153	59	8.93153	4
6.951992	292	8.951992	0	8.951992	54	8.951992	0
6.972455	294	8.972455	0	8.972455	38	8.972455	51
6.992918	287	8.992918	7	8.992918	32	8.992918	59
7.013381	288	9.013381	0	9.013381	22	9.013381	34
7.033843	284	9.033843	9	9.033843	22	9.033843	0
7.054306	324	9.054306	16	9.054306	32	9.054306	78
7.074769	350	9.074769	12	9.074769	33	9.074769	139
7.095232	389	9.095232	43	9.095232	35	9.095232	38
7.115695	440	9.115695	0	9.115695	41	9.115695	5
7.136157	532	9.136157	0	9.136157	37	9.136157	64
7.15662	613	9.15662	42	9.15662	27	9.15662	24
7.177083	700	9.177083	0	9.177083	9	9.177083	8
7.197546	768	9.197546	0	9.197546	10	9.197546	43
7.218008	847	9.218008	2	9.218008	9	9.218008	65
7.238471	952	9.238471	0	9.238471	15	9.238471	51
7.258934	1098	9.258934	15	9.258934	9	9.258934	31
7.279397	1266	9.279397	10	9.279397	24	9.279397	27
7.29986	1528	9.29986	0	9.29986	35	9.29986	54
7.320322	1944	9.320322	79	9.320322	42	9.320322	18
7.340785	2502	9.340785	5	9.340785	42	9.340785	51
7.361248	3293	9.361248	25	9.361248	41	9.361248	0
7.381711	4365	9.381711	0	9.381711	34	9.381711	0
7.402173	5579	9.402173	0	9.402173	28	9.402173	45
7.422636	6672	9.422636	0	9.422636	21	9.422636	0
7.443099	7289	9.443099	0	9.443099	16	9.443099	28
7.463562	7230	9.463562	1	9.463562	7	9.463562	0
7.484025	6530	9.484025	39	9.484025	12	9.484025	0
7.504487	5356	9.504487	25	9.504487	11	9.504487	0
7.52495	3916	9.52495	0	9.52495	15	9.52495	16
7.545413	2450	9.545413	17	9.545413	14	9.545413	0
7.565876	1300	9.565876	29	9.565876	19	9.565876	0
7.586338	621	9.586338	55	9.586338	13	9.586338	7
7.606801	370	9.606801	8	9.606801	14	9.606801	17
7.627264	346	9.627264	0	9.627264	26	9.627264	51
7.647727	341	9.647727	12	9.647727	29	9.647727	27
7.66819	318	9.66819	17	9.66819	25	9.66819	3
7.688652	260	9.688652	7	9.688652	27	9.688652	113
7.709115	223	9.709115	0	9.709115	30	9.709115	0
7.729578	183	9.729578	14	9.729578	21	9.729578	9
7.750041	142	9.750041	0	9.750041	20	9.750041	35
7.770503	115	9.770503	41	9.770503	21	9.770503	0

7.790966	103	9.790966	0	9.790966	20	9.790966	26
7.811429	90	9.811429	28	9.811429	27	9.811429	0
7.831892	79	9.831892	28	9.831892	34	9.831892	0
7.852355	71	9.852355	0	9.852355	35	9.852355	0
7.872817	90	9.872817	0	9.872817	33	9.872817	0
7.89328	77	9.89328	0	9.89328	29	9.89328	28
7.913743	90	9.913743	20	9.913743	24	9.913743	16
7.934206	89	9.934206	4	9.934206	11	9.934206	0
7.954668	87	9.954668	0	9.954668	11	9.954668	106
7.975131	91	9.975131	9	9.975131	9	9.975131	67
7.995594	83	9.995594	49	9.995594	13	9.995594	0
8.016057	72	10.01606	10	10.01606	15	10.01606	113
8.03652	50	10.03652	60	10.03652	16	10.03652	12
8.056982	49	10.05698	0	10.05698	17	10.05698	42
8.077445	46	10.07745	0	10.07745	13	10.07745	47
8.097908	50	10.09791	0	10.09791	16	10.09791	16
8.118371	51	10.11837	3	10.11837	15	10.11837	129
8.138833	53	10.13883	17	10.13883	13	10.13883	94
8.159296	55	10.1593	26	10.1593	24	10.1593	0
8.179759	46	10.17976	0	10.17976	43	10.17976	58
8.200222	34	10.20022	26	10.20022	58	10.20022	45
8.220685	30	10.22069	36	10.22069	81	10.22069	160
8.241147	24	10.24115	60	10.24115	111	10.24115	107
8.26161	29	10.26161	50	10.26161	143	10.26161	160
8.282073	15	10.28207	33	10.28207	174	10.28207	106
8.302536	25	10.30254	50	10.30254	216	10.30254	278
8.322998	13	10.323	38	10.323	237	10.323	256
8.343461	24	10.34346	24	10.34346	273	10.34346	374
8.363924	29	10.36392	62	10.36392	324	10.36392	346
8.384387	27	10.38439	49	10.38439	377	10.38439	629
8.40485	27	10.40485	79	10.40485	438	10.40485	917
8.425312	32	10.42531	150	10.42531	552	10.42531	1177
8.445775	30	10.44578	162	10.44578	713	10.44578	1489
8.466238	24	10.46624	263	10.46624	914	10.46624	2039
8.486701	12	10.4867	237	10.4867	1195	10.4867	2564
8.507163	7	10.50716	356	10.50716	1555	10.50716	2862
8.527626	0	10.52763	460	10.52763	1990	10.52763	3059
8.548089	6	10.54809	592	10.54809	2430	10.54809	2498
8.568552	16	10.56855	740	10.56855	2798	10.56855	1927
8.589015	21	10.58902	1086	10.58902	2958	10.58902	1338
8.609477	23	10.60948	1392	10.60948	2897	10.60948	728
8.62994	19	10.62994	1679	10.62994	2629	10.62994	450
8.650403	23	10.6504	2266	10.6504	2188	10.6504	254
8.670866	18	10.67087	2829	10.67087	1662	10.67087	292
8.691328	17	10.69133	2931	10.69133	1155	10.69133	282
8.711791	11	10.71179	3026	10.71179	737	10.71179	91
8.732254	7	10.73225	2761	10.73225	464	10.73225	124
8.752717	17	10.75272	1974	10.75272	313	10.75272	116
8.77318	20	10.77318	1348	10.77318	246	10.77318	90
8.793642	21	10.79364	643	10.79364	191	10.79364	62
8.814105	23	10.81411	429	10.81411	158	10.81411	53
8.834568	21	10.83457	368	10.83457	128	10.83457	48
8.855031	23	10.85503	238	10.85503	95	10.85503	52
8.875493	16	10.87549	87	10.87549	84	10.87549	60
8.895956	16	10.89596	179	10.89596	70	10.89596	72
8.916419	10	10.91642	106	10.91642	56	10.91642	73
8.936882	8	10.93688	18	10.93688	34	10.93688	67
8.957345	3	10.95735	0	10.95735	30	10.95735	29

8.977807	2	10.97781	29	10.97781	21	10.97781	73
8.99827	2	10.99827	0	10.99827	20	10.99827	2
9.018733	1	11.01873	0	11.01873	25	11.01873	0
9.039196	5	11.0392	0	11.0392	32	11.0392	42
9.059658	7	11.05966	25	11.05966	33	11.05966	71
9.080121	6	11.08012	22	11.08012	36	11.08012	0
9.100584	9	11.10058	52	11.10058	33	11.10058	28
9.121047	4	11.12105	0	11.12105	28	11.12105	0
9.14151	10	11.14151	9	11.14151	14	11.14151	0
9.161972	14	11.16197	0	11.16197	8	11.16197	0
9.182435	17	11.18244	0	11.18244	5	11.18244	23
9.202898	20	11.2029	40	11.2029	5	11.2029	102
9.223361	20	11.22336	0	11.22336	6	11.22336	20
9.243823	23	11.24382	14	11.24382	10	11.24382	12
9.264286	22	11.26429	50	11.26429	17	11.26429	0
9.284749	18	11.28475	3	11.28475	20	11.28475	10
9.305212	13	11.30521	0	11.30521	20	11.30521	0
9.325675	4	11.32568	0	11.32568	18	11.32568	0
9.346137	6	11.34614	14	11.34614	12	11.34614	0
9.3666	0	11.3666	0	11.3666	8	11.3666	53
9.387063	0	11.38706	0	11.38706	9	11.38706	45
9.407526	0	11.40753	5	11.40753	4	11.40753	0
9.427988	6	11.42799	19	11.42799	0	11.42799	101
9.448451	10	11.44845	5	11.44845	0	11.44845	72
9.468914	21	11.46891	0	11.46891	0	11.46891	56
9.489377	30	11.48938	6	11.48938	10	11.48938	0
9.50984	40	11.50984	63	11.50984	16	11.50984	1
9.530302	40	11.5303	0	11.5303	21	11.5303	1
9.550765	40	11.55077	1	11.55077	20	11.55077	29
9.571228	41	11.57123	0	11.57123	22	11.57123	17
9.591691	36	11.59169	6	11.59169	19	11.59169	8
9.612153	30	11.61215	10	11.61215	16	11.61215	0
9.632616	28	11.63262	0	11.63262	10	11.63262	36
9.653079	24	11.65308	0	11.65308	8	11.65308	36
9.673542	28	11.67354	26	11.67354	0	11.67354	111
9.694005	32	11.69401	0	11.69401	7	11.69401	0
9.714467	31	11.71447	0	11.71447	7	11.71447	0
9.73493	31	11.73493	0	11.73493	6	11.73493	0
9.755393	34	11.75539	0	11.75539	0	11.75539	0
9.775856	35	11.77586	6	11.77586	5	11.77586	88
9.796318	40	11.79632	0	11.79632	7	11.79632	7
9.816781	40	11.81678	26	11.81678	12	11.81678	0
9.837244	39	11.83724	67	11.83724	17	11.83724	5
9.857707	36	11.85771	0	11.85771	15	11.85771	24
9.87817	31	11.87817	0	11.87817	17	11.87817	25
9.898632	22	11.89863	21	11.89863	19	11.89863	39
9.919095	17	11.9191	25	11.9191	18	11.9191	7
9.939558	22	11.93956	27	11.93956	16	11.93956	58
9.960021	19	11.96002	21	11.96002	15	11.96002	53
9.980483	13	11.98048	0	11.98048	26	11.98048	111
10.00095	28	12.00095	39	12.00095	24	12.00095	30
10.02141	34	12.02141	0	12.02141	33	12.02141	72
10.04187	47	12.04187	0	12.04187	42	12.04187	109
10.06233	55	12.06233	0	12.06233	48	12.06233	139
10.0828	56	12.0828	24	12.0828	64	12.0828	149
10.10326	66	12.10326	66	12.10326	77	12.10326	119
10.12372	83	12.12372	77	12.12372	91	12.12372	221
10.14419	90	12.14419	35	12.14419	119	12.14419	170

10.16465	92	12.16465	45	12.16465	152	12.16465	316
10.18511	86	12.18511	37	12.18511	193	12.18511	367
10.20557	110	12.20557	91	12.20557	235	12.20557	527
10.22604	128	12.22604	55	12.22604	295	12.22604	706
10.2465	152	12.2465	60	12.2465	368	12.2465	930
10.26696	177	12.26696	148	12.26696	457	12.26696	1240
10.28743	213	12.28743	112	12.28743	577	12.28743	1627
10.30789	279	12.30789	196	12.30789	729	12.30789	2113
10.32835	337	12.32835	242	12.32835	916	12.32835	2271
10.34881	431	12.34881	368	12.34881	1166	12.34881	2444
10.36928	562	12.36928	479	12.36928	1440	12.36928	2297
10.38974	708	12.38974	523	12.38974	1696	12.38974	1711
10.4102	961	12.4102	807	12.4102	1881	12.4102	1183
10.43066	1333	12.43066	996	12.43066	1922	12.43066	823
10.45113	1831	12.45113	1308	12.45113	1838	12.45113	577
10.47159	2382	12.47159	1745	12.47159	1630	12.47159	360
10.49205	2840	12.49205	2154	12.49205	1343	12.49205	311
10.51252	3059	12.51252	2349	12.51252	1005	12.51252	196
10.53298	2983	12.53298	2354	12.53298	695	12.53298	210
10.55344	2655	12.55344	2123	12.55344	473	12.55344	138
10.5739	2119	12.5739	1544	12.5739	317	12.5739	153
10.59437	1466	12.59437	954	12.59437	228	12.59437	111
10.61483	866	12.61483	640	12.61483	183	12.61483	35
10.63529	424	12.63529	319	12.63529	140	12.63529	87
10.65576	191	12.65576	262	12.65576	120	12.65576	117
10.67622	109	12.67622	174	12.67622	111	12.67622	146
10.69668	101	12.69668	180	12.69668	111	12.69668	199
10.71714	87	12.71714	90	12.71714	103	12.71714	167
10.73761	68	12.73761	187	12.73761	120	12.73761	259
10.75807	49	12.75807	136	12.75807	143	12.75807	340
10.77853	38	12.77853	105	12.77853	152	12.77853	353
10.79899	31	12.79899	133	12.79899	192	12.79899	433
10.81946	21	12.81946	126	12.81946	251	12.81946	656
10.83992	26	12.83992	110	12.83992	335	12.83992	753
10.86038	36	12.86038	194	12.86038	426	12.86038	874
10.88085	30	12.88085	190	12.88085	549	12.88085	967
10.90131	40	12.90131	202	12.90131	674	12.90131	973
10.92177	43	12.92177	263	12.92177	766	12.92177	726
10.94223	38	12.94223	356	12.94223	807	12.94223	532
10.9627	31	12.9627	396	12.9627	780	12.9627	356
10.98316	24	12.98316	453	12.98316	692	12.98316	234
11.00362	24	13.00362	694	13.00362	581	13.00362	152
11.02409	21	13.02409	800	13.02409	438	13.02409	191
11.04455	23	13.04455	890	13.04455	299	13.04455	186
11.06501	27	13.06501	1077	13.06501	181	13.06501	117
11.08547	27	13.08547	875	13.08547	115	13.08547	225
11.10594	31	13.10594	661	13.10594	80	13.10594	78
11.1264	27	13.1264	544	13.1264	59	13.1264	107
11.14686	19	13.14686	279	13.14686	42	13.14686	153
11.16732	5	13.16732	251	13.16732	34	13.16732	132
11.18779	5	13.18779	212	13.18779	28	13.18779	147
11.20825	7	13.20825	149	13.20825	22	13.20825	48
11.22871	2	13.22871	210	13.22871	15	13.22871	80
11.24918	0	13.24918	203	13.24918	10	13.24918	123
11.26964	0	13.26964	161	13.26964	12	13.26964	59
11.2901	2	13.2901	143	13.2901	8	13.2901	114
11.31056	11	13.31056	130	13.31056	10	13.31056	61
11.33103	15	13.33103	64	13.33103	9	13.33103	63

11.35149	30	13.35149	130	13.35149	5	13.35149	71
11.37195	39	13.37195	178	13.37195	7	13.37195	95
11.39242	38	13.39242	164	13.39242	9	13.39242	120
11.41288	41	13.41288	128	13.41288	12	13.41288	140
11.43334	39	13.43334	96	13.43334	19	13.43334	130
11.4538	33	13.4538	168	13.4538	21	13.4538	92
11.47427	27	13.47427	85	13.47427	20	13.47427	206
11.49473	25	13.49473	143	13.49473	15	13.49473	196
11.51519	22	13.51519	152	13.51519	14	13.51519	156
11.53565	25	13.53565	123	13.53565	9	13.53565	211
11.55612	30	13.55612	224	13.55612	5	13.55612	157
11.57658	32	13.57658	149	13.57658	3	13.57658	152
11.59704	30	13.59704	138	13.59704	2	13.59704	145
11.61751	39	13.61751	169	13.61751	7	13.61751	236
11.63797	37	13.63797	197	13.63797	6	13.63797	257
11.65843	37	13.65843	202	13.65843	10	13.65843	269
11.67889	32	13.67889	189	13.67889	12	13.67889	222
11.69936	23	13.69936	208	13.69936	9	13.69936	305
11.71982	13	13.71982	196	13.71982	12	13.71982	218
11.74028	15	13.74028	225	13.74028	13	13.74028	307
11.76075	16	13.76075	203	13.76075	11	13.76075	315
11.78121	30	13.78121	207	13.78121	10	13.78121	351
11.80167	36	13.80167	228	13.80167	10	13.80167	312
11.82213	37	13.82213	285	13.82213	13	13.82213	363
11.8426	35	13.8426	291	13.8426	10	13.8426	325
11.86306	47	13.86306	299	13.86306	10	13.86306	385
11.88352	49	13.88352	271	13.88352	11	13.88352	474
11.90398	54	13.90398	291	13.90398	7	13.90398	461
11.92445	49	13.92445	333	13.92445	8	13.92445	450
11.94491	56	13.94491	315	13.94491	9	13.94491	393
11.96537	68	13.96537	364	13.96537	10	13.96537	570
11.98584	79	13.98584	349	13.98584	14	13.98584	490
12.0063	84	14.0063	379	14.0063	23	14.0063	522
12.02676	83	14.02676	346	14.02676	24	14.02676	548
12.04722	101	14.04722	431	14.04722	23	14.04722	526
12.06769	122	14.06769	327	14.06769	24	14.06769	577
12.08815	147	14.08815	398	14.08815	26	14.08815	631
12.10861	183	14.10861	359	14.10861	27	14.10861	679
12.12908	220	14.12908	389	14.12908	26	14.12908	653
12.14954	275	14.14954	419	14.14954	21	14.14954	795
12.17	340	14.17	418	14.17	24	14.17	725
12.19046	428	14.19046	412	14.19046	21	14.19046	751
12.21093	557	14.21093	417	14.21093	23	14.21093	773
12.23139	733	14.23139	499	14.23139	15	14.23139	696
12.25185	976	14.25185	470	14.25185	14	14.25185	857
12.27231	1261	14.27231	510	14.27231	14	14.27231	884
12.29278	1565	14.29278	537	14.29278	13	14.29278	861
12.31324	1814	14.31324	535	14.31324	20	14.31324	960
12.3337	1919	14.3337	550	14.3337	21	14.3337	890
12.35417	1877	14.35417	580	14.35417	24	14.35417	955
12.37463	1671	14.37463	539	14.37463	31	14.37463	1031
12.39509	1365	14.39509	555	14.39509	33	14.39509	988
12.41555	1019	14.41555	539	14.41555	33	14.41555	963
12.43602	687	14.43602	594	14.43602	40	14.43602	1101
12.45648	432	14.45648	572	14.45648	45	14.45648	1045
12.47694	269	14.47694	702	14.47694	55	14.47694	1158
12.49741	201	14.49741	634	14.49741	58	14.49741	1106
12.51787	191	14.51787	680	14.51787	66	14.51787	1247

12.53833	174	14.53833	655	14.53833	72	14.53833	1112
12.55879	165	14.55879	748	14.55879	76	14.55879	1161
12.57926	160	14.57926	696	14.57926	73	14.57926	1158
12.59972	164	14.59972	625	14.59972	71	14.59972	1230
12.62018	172	14.62018	760	14.62018	72	14.62018	1153
12.64064	191	14.64064	605	14.64064	72	14.64064	1295
12.66111	211	14.66111	768	14.66111	78	14.66111	1338
12.68157	209	14.68157	730	14.68157	92	14.68157	1280
12.70203	224	14.70203	712	14.70203	107	14.70203	1413
12.7225	248	14.7225	725	14.7225	129	14.7225	1353
12.74296	298	14.74296	662	14.74296	154	14.74296	1419
12.76342	370	14.76342	667	14.76342	185	14.76342	1586
12.78388	484	14.78388	734	14.78388	231	14.78388	1707
12.80435	635	14.80435	685	14.80435	299	14.80435	1830
12.82481	813	14.82481	798	14.82481	377	14.82481	1892
12.84527	990	14.84527	800	14.84527	486	14.84527	2271
12.86574	1104	14.86574	835	14.86574	629	14.86574	2252
12.8862	1115	14.8862	902	14.8862	783	14.8862	2576
12.90666	1038	14.90666	949	14.90666	956	14.90666	2412
12.92712	895	14.92712	982	14.92712	1105	14.92712	2210
12.94759	723	14.94759	1100	14.94759	1195	14.94759	2116
12.96805	505	14.96805	1213	14.96805	1209	14.96805	1768
12.98851	324	14.98851	1406	14.98851	1145	14.98851	1478
13.00897	202	15.00897	1506	15.00897	1008	15.00897	1386
13.02944	149	15.02944	1679	15.02944	815	15.02944	1299
13.0499	145	15.0499	1855	15.0499	635	15.0499	1172
13.07036	156	15.07036	1939	15.07036	456	15.07036	1118
13.09083	156	15.09083	1697	15.09083	320	15.09083	998
13.11129	170	15.11129	1414	15.11129	240	15.11129	1042
13.13175	185	15.13175	1217	15.13175	187	15.13175	1101
13.15221	194	15.15221	1036	15.15221	159	15.15221	1070
13.17268	191	15.17268	764	15.17268	139	15.17268	996
13.19314	216	15.19314	773	15.19314	121	15.19314	966
13.2136	223	15.2136	701	15.2136	104	15.2136	1024
13.23407	250	15.23407	629	15.23407	89	15.23407	863
13.25453	260	15.25453	639	15.25453	82	15.25453	857
13.27499	248	15.27499	547	15.27499	68	15.27499	830
13.29545	240	15.29545	649	15.29545	63	15.29545	718
13.31592	228	15.31592	523	15.31592	57	15.31592	766
13.33638	209	15.33638	506	15.33638	42	15.33638	863
13.35684	196	15.35684	493	15.35684	39	15.35684	768
13.3773	212	15.3773	536	15.3773	28	15.3773	676
13.39777	229	15.39777	503	15.39777	22	15.39777	625
13.41823	253	15.41823	505	15.41823	11	15.41823	736
13.43869	273	15.43869	436	15.43869	10	15.43869	737
13.45916	280	15.45916	459	15.45916	6	15.45916	751
13.47962	273	15.47962	521	15.47962	7	15.47962	705
13.50008	291	15.50008	534	15.50008	11	15.50008	710
13.52054	293	15.52054	433	15.52054	13	15.52054	639
13.54101	299	15.54101	447	15.54101	12	15.54101	662
13.56147	306	15.56147	413	15.56147	9	15.56147	605
13.58193	328	15.58193	455	15.58193	8	15.58193	528
13.6024	347	15.6024	481	15.6024	4	15.6024	562
13.62286	372	15.62286	399	15.62286	1	15.62286	559
13.64332	378	15.64332	378	15.64332	6	15.64332	421
13.66378	387	15.66378	371	15.66378	5	15.66378	541
13.68425	391	15.68425	345	15.68425	1	15.68425	549
13.70471	414	15.70471	429	15.70471	10	15.70471	511

13.72517	440	15.72517	316	15.72517	15	15.72517	486
13.74563	459	15.74563	333	15.74563	17	15.74563	554
13.7661	482	15.7661	410	15.7661	15	15.7661	564
13.78656	511	15.78656	347	15.78656	19	15.78656	566
13.80702	545	15.80702	325	15.80702	15	15.80702	489
13.82749	567	15.82749	340	15.82749	18	15.82749	540
13.84795	586	15.84795	349	15.84795	14	15.84795	516
13.86841	599	15.86841	356	15.86841	11	15.86841	503
13.88887	605	15.88887	261	15.88887	6	15.88887	499
13.90934	619	15.90934	368	15.90934	13	15.90934	515
13.9298	642	15.9298	367	15.9298	11	15.9298	558
13.95026	658	15.95026	333	15.95026	10	15.95026	642
13.97073	684	15.97073	335	15.97073	8	15.97073	538
13.99119	703	15.99119	326	15.99119	7	15.99119	504
14.01165	730	16.01165	356	16.01165	1	16.01165	576
14.03211	762	16.03211	326	16.03211	7	16.03211	639
14.05258	791	16.05258	339	16.05258	15	16.05258	668
14.07304	811	16.07304	361	16.07304	24	16.07304	647
14.0935	834	16.0935	283	16.0935	42	16.0935	670
14.11396	876	16.11396	376	16.11396	56	16.11396	706
14.13443	911	16.13443	424	16.13443	85	16.13443	797
14.15489	957	16.15489	387	16.15489	111	16.15489	873
14.17535	1002	16.17535	375	16.17535	137	16.17535	1023
14.19582	1024	16.19582	381	16.19582	163	16.19582	1181
14.21628	1071	16.21628	412	16.21628	216	16.21628	1290
14.23674	1105	16.23674	360	16.23674	289	16.23674	1124
14.2572	1123	16.2572	416	16.2572	366	16.2572	1084
14.27767	1152	16.27767	463	16.27767	427	16.27767	930
14.29813	1188	16.29813	498	16.29813	477	16.29813	867
14.31859	1235	16.31859	648	16.31859	484	16.31859	765
14.33906	1249	16.33906	749	16.33906	466	16.33906	680
14.35952	1281	16.35952	834	16.35952	401	16.35952	676
14.37998	1301	16.37998	961	16.37998	316	16.37998	672
14.40044	1320	16.40044	947	16.40044	229	16.40044	713
14.42091	1362	16.42091	856	16.42091	167	16.42091	689
14.44137	1381	16.44137	659	16.44137	121	16.44137	614
14.46183	1405	16.46183	598	16.46183	94	16.46183	609
14.48229	1443	16.48229	496	16.48229	81	16.48229	685
14.50276	1461	16.50276	422	16.50276	79	16.50276	635
14.52322	1470	16.52322	387	16.52322	71	16.52322	629
14.54368	1484	16.54368	414	16.54368	56	16.54368	683
14.56415	1521	16.56415	312	16.56415	48	16.56415	550
14.58461	1535	16.58461	318	16.58461	52	16.58461	685
14.60507	1580	16.60507	282	16.60507	55	16.60507	712
14.62553	1623	16.62553	374	16.62553	67	16.62553	697
14.646	1658	16.646	335	16.646	91	16.646	745
14.66646	1703	16.66646	315	16.66646	114	16.66646	587
14.68692	1741	16.68692	288	16.68692	127	16.68692	651
14.70739	1759	16.70739	384	16.70739	141	16.70739	533
14.72785	1823	16.72785	339	16.72785	138	16.72785	551
14.74831	1917	16.74831	300	16.74831	126	16.74831	531
14.76877	2031	16.76877	372	16.76877	115	16.76877	507
14.78924	2185	16.78924	391	16.78924	94	16.78924	511
14.8097	2370	16.8097	386	16.8097	69	16.8097	545
14.83016	2546	16.83016	365	16.83016	55	16.83016	397
14.85062	2707	16.85062	342	16.85062	52	16.85062	449
14.87109	2806	16.87109	315	16.87109	35	16.87109	426
14.89155	2775	16.89155	274	16.89155	23	16.89155	344

14.91201	2655	16.91201	241	16.91201	26	16.91201	367
14.93248	2476	16.93248	279	16.93248	31	16.93248	341
14.95294	2246	16.95294	236	16.95294	39	16.95294	264
14.9734	2012	16.9734	185	16.9734	37	16.9734	342
14.99386	1838	16.99386	195	16.99386	31	16.99386	292
15.01433	1675	17.01433	236	17.01433	30	17.01433	382
15.03479	1584	17.03479	164	17.03479	28	17.03479	249
15.05525	1545	17.05525	133	17.05525	27	17.05525	246
15.07572	1501	17.07572	163	17.07572	18	17.07572	256
15.09618	1440	17.09618	122	17.09618	12	17.09618	160
15.11664	1409	17.11664	79	17.11664	10	17.11664	184
15.1371	1375	17.1371	89	17.1371	9	17.1371	168
15.15757	1343	17.15757	145	17.15757	10	17.15757	210
15.17803	1304	17.17803	144	17.17803	13	17.17803	129
15.19849	1282	17.19849	130	17.19849	16	17.19849	111
15.21895	1232	17.21895	134	17.21895	28	17.21895	75
15.23942	1220	17.23942	106	17.23942	31	17.23942	164
15.25988	1221	17.25988	104	17.25988	41	17.25988	146
15.28034	1187	17.28034	124	17.28034	42	17.28034	189
15.30081	1161	17.30081	105	17.30081	43	17.30081	88
15.32127	1145	17.32127	32	17.32127	38	17.32127	103
15.34173	1125	17.34173	80	17.34173	32	17.34173	95
15.36219	1082	17.36219	52	17.36219	25	17.36219	38
15.38266	1033	17.38266	91	17.38266	21	17.38266	109
15.40312	999	17.40312	77	17.40312	17	17.40312	61
15.42358	977	17.42358	23	17.42358	18	17.42358	110
15.44405	981	17.44405	22	17.44405	14	17.44405	50
15.46451	981	17.46451	48	17.46451	20	17.46451	132
15.48497	951	17.48497	43	17.48497	18	17.48497	36
15.50543	940	17.50543	0	17.50543	23	17.50543	12
15.5259	920	17.5259	72	17.5259	22	17.5259	22
15.54636	904	17.54636	64	17.54636	20	17.54636	0
15.56682	868	17.56682	52	17.56682	19	17.56682	0
15.58728	846	17.58728	3	17.58728	21	17.58728	0
15.60775	840	17.60775	0	17.60775	25	17.60775	0
15.62821	848	17.62821	48	17.62821	28	17.62821	0
15.64867	836	17.64867	15	17.64867	27	17.64867	48
15.66914	832	17.66914	61	17.66914	34	17.66914	28
15.6896	832	17.6896	27	17.6896	35	17.6896	48
15.71006	837	17.71006	14	17.71006	40	17.71006	0
15.73052	822	17.73052	43	17.73052	36	17.73052	1
15.75099	806	17.75099	0	17.75099	29	17.75099	0
15.77145	778	17.77145	17	17.77145	24	17.77145	0
15.79191	754	17.79191	0	17.79191	19	17.79191	0
15.81238	742	17.81238	0	17.81238	16	17.81238	36
15.83284	733	17.83284	44	17.83284	9	17.83284	14
15.8533	741	17.8533	12	17.8533	1	17.8533	5
15.87376	758	17.87376	61	17.87376	9	17.87376	70
15.89423	777	17.89423	13	17.89423	10	17.89423	3
15.91469	793	17.91469	64	17.91469	20	17.91469	4
15.93515	804	17.93515	0	17.93515	29	17.93515	0
15.95561	798	17.95561	0	17.95561	36	17.95561	20
15.97608	793	17.97608	0	17.97608	48	17.97608	0
15.99654	786	17.99654	13	17.99654	55	17.99654	56
16.017	789	18.017	0	18.017	60	18.017	53
16.03747	780	18.03747	0	18.03747	61	18.03747	89
16.05793	810	18.05793	58	18.05793	68	18.05793	105
16.07839	832	18.07839	71	18.07839	72	18.07839	58

16.09885	874	18.09885	19	18.09885	79	18.09885	146
16.11932	928	18.11932	25	18.11932	106	18.11932	242
16.13978	1013	18.13978	51	18.13978	149	18.13978	422
16.16024	1085	18.16024	40	18.16024	210	18.16024	511
16.18071	1180	18.18071	0	18.18071	276	18.18071	714
16.20117	1225	18.20117	14	18.20117	362	18.20117	940
16.22163	1241	18.22163	56	18.22163	476	18.22163	1048
16.24209	1202	18.24209	59	18.24209	593	18.24209	1150
16.26256	1143	18.26256	206	18.26256	701	18.26256	923
16.28302	1071	18.28302	197	18.28302	783	18.28302	727
16.30348	988	18.30348	362	18.30348	832	18.30348	463
16.32394	931	18.32394	475	18.32394	829	18.32394	404
16.34441	897	18.34441	705	18.34441	769	18.34441	206
16.36487	866	18.36487	830	18.36487	663	18.36487	214
16.38533	874	18.38533	994	18.38533	526	18.38533	118
16.4058	880	18.4058	1014	18.4058	401	18.4058	47
16.42626	894	18.42626	918	18.42626	296	18.42626	73
16.44672	867	18.44672	662	18.44672	201	18.44672	65
16.46718	863	18.46718	403	18.46718	144	18.46718	0
16.48765	857	18.48765	259	18.48765	111	18.48765	48
16.50811	864	18.50811	230	18.50811	91	18.50811	41
16.52857	879	18.52857	167	18.52857	72	18.52857	0
16.54904	889	18.54904	119	18.54904	48	18.54904	11
16.5695	897	18.5695	31	18.5695	38	18.5695	51
16.58996	904	18.58996	77	18.58996	29	18.58996	19
16.61042	927	18.61042	39	18.61042	26	18.61042	0
16.63089	914	18.63089	74	18.63089	19	18.63089	26
16.65135	878	18.65135	25	18.65135	13	18.65135	22
16.67181	860	18.67181	52	18.67181	18	18.67181	58
16.69227	823	18.69227	89	18.69227	16	18.69227	9
16.71274	802	18.71274	12	18.71274	12	18.71274	0
16.7332	768	18.7332	61	18.7332	10	18.7332	0
16.75366	737	18.75366	34	18.75366	9	18.75366	25
16.77413	708	18.77413	26	18.77413	11	18.77413	29
16.79459	669	18.79459	54	18.79459	9	18.79459	65
16.81505	659	18.81505	1	18.81505	18	18.81505	0
16.83551	640	18.83551	0	18.83551	22	18.83551	0
16.85598	610	18.85598	0	18.85598	20	18.85598	62
16.87644	588	18.87644	79	18.87644	25	18.87644	38
16.8969	564	18.8969	0	18.8969	28	18.8969	21
16.91737	536	18.91737	0	18.91737	23	18.91737	42
16.93783	512	18.93783	18	18.93783	25	18.93783	12
16.95829	495	18.95829	0	18.95829	24	18.95829	1
16.97875	477	18.97875	34	18.97875	22	18.97875	33
16.99922	462	18.99922	4	18.99922	20	18.99922	66
17.01968	444	19.01968	2	19.01968	22	19.01968	8
17.04014	427	19.04014	10	19.04014	13	19.04014	16
17.0606	390	19.0606	65	19.0606	9	19.0606	68
17.08107	365	19.08107	10	19.08107	19	19.08107	87
17.10153	333	19.10153	17	19.10153	23	19.10153	37
17.12199	309	19.12199	30	19.12199	28	19.12199	119
17.14246	294	19.14246	0	19.14246	43	19.14246	86
17.16292	285	19.16292	23	19.16292	67	19.16292	125
17.18338	265	19.18338	18	19.18338	91	19.18338	260
17.20384	264	19.20384	26	19.20384	118	19.20384	284
17.22431	246	19.22431	45	19.22431	141	19.22431	407
17.24477	226	19.24477	53	19.24477	163	19.24477	546
17.26523	196	19.26523	58	19.26523	224	19.26523	897

17.2857	183	19.2857	65	19.2857	306	19.2857	1141
17.30616	167	19.30616	67	19.30616	438	19.30616	1530
17.32662	160	19.32662	141	19.32662	617	19.32662	1948
17.34708	138	19.34708	173	19.34708	842	19.34708	2152
17.36755	127	19.36755	268	19.36755	1084	19.36755	1960
17.38801	116	19.38801	381	19.38801	1307	19.38801	1676
17.40847	116	19.40847	542	19.40847	1451	19.40847	1345
17.42893	127	19.42893	779	19.42893	1490	19.42893	971
17.4494	128	19.4494	1100	19.4494	1423	19.4494	651
17.46986	124	19.46986	1420	19.46986	1259	19.46986	376
17.49032	111	19.49032	1709	19.49032	1030	19.49032	272
17.51079	101	19.51079	1771	19.51079	793	19.51079	130
17.53125	84	19.53125	1548	19.53125	568	19.53125	168
17.55171	60	19.55171	1187	19.55171	397	19.55171	89
17.57217	45	19.57217	794	19.57217	272	19.57217	143
17.59264	29	19.59264	556	19.59264	199	19.59264	86
17.6131	17	19.6131	347	19.6131	140	19.6131	49
17.63356	24	19.63356	188	19.63356	105	19.63356	71
17.65403	22	19.65403	182	19.65403	80	19.65403	52
17.67449	15	19.67449	127	19.67449	50	19.67449	61
17.69495	1	19.69495	112	19.69495	38	19.69495	0
17.71541	5	19.71541	55	19.71541	27	19.71541	0
17.73588	5	19.73588	71	19.73588	23	19.73588	20
17.75634	8	19.75634	0	19.75634	19	19.75634	100
17.7768	8	19.7768	35	19.7768	18	19.7768	25
17.79726	7	19.79726	0	19.79726	22	19.79726	42
17.81773	2	19.81773	11	19.81773	19	19.81773	25
17.83819	3	19.83819	21	19.83819	19	19.83819	0
17.85865	0	19.85865	14	19.85865	18	19.85865	3
17.87912	4	19.87912	0	19.87912	14	19.87912	7
17.89958	8	19.89958	0	19.89958	14	19.89958	55
17.92004	14	19.92004	54	19.92004	11	19.92004	14
17.9405	20	19.9405	0	19.9405	6	19.9405	45
17.96097	24	19.96097	3	19.96097	5	19.96097	0
17.98143	29	19.98143	12	19.98143	11	19.98143	64
18.00189	39	20.00189	79	20.00189	11	20.00189	34
18.02235	57	20.02235	27	20.02235	13	20.02235	0
18.04282	91	20.04282	1	20.04282	15	20.04282	2
18.06328	111	20.06328	0	20.06328	9	20.06328	57
18.08374	177	20.08374	5	20.08374	9	20.08374	57
18.10421	284	20.10421	0	20.10421	16	20.10421	0
18.12467	456	20.12467	5	20.12467	16	20.12467	26
18.14513	707	20.14513	0	20.14513	15	20.14513	26
18.16559	984	20.16559	0	20.16559	19	20.16559	26
18.18606	1261	20.18606	20	20.18606	20	20.18606	48
18.20652	1457	20.20652	0	20.20652	24	20.20652	0
18.22698	1546	20.22698	7	20.22698	27	20.22698	61
18.24745	1477	20.24745	58	20.24745	24	20.24745	1
18.26791	1272	20.26791	0	20.26791	16	20.26791	26
18.28837	992	20.28837	0	20.28837	17	20.28837	4
18.30883	683	20.30883	0	20.30883	15	20.30883	35
18.3293	412	20.3293	46	20.3293	11	20.3293	50
18.34976	226	20.34976	0	20.34976	3	20.34976	17
18.37022	102	20.37022	9	20.37022	1	20.37022	0
18.39068	62	20.39068	13	20.39068	1	20.39068	50
18.41115	41	20.41115	38	20.41115	4	20.41115	0
18.43161	42	20.43161	18	20.43161	5	20.43161	0
18.45207	25	20.45207	24	20.45207	9	20.45207	0

18.47254	37	20.47254	18	20.47254	11	20.47254	0
18.493	36	20.493	43	20.493	15	20.493	26
18.51346	32	20.51346	43	20.51346	17	20.51346	0
18.53392	26	20.53392	0	20.53392	19	20.53392	10
18.55439	20	20.55439	13	20.55439	17	20.55439	62
18.57485	15	20.57485	12	20.57485	12	20.57485	65
18.59531	22	20.59531	21	20.59531	8	20.59531	15
18.61578	19	20.61578	9	20.61578	3	20.61578	12
18.63624	13	20.63624	20	20.63624	0	20.63624	27
18.6567	6	20.6567	13	20.6567	1	20.6567	0
18.67716	16	20.67716	0	20.67716	0	20.67716	0
18.69763	15	20.69763	39	20.69763	3	20.69763	44
18.71809	12	20.71809	20	20.71809	4	20.71809	55
18.73855	10	20.73855	30	20.73855	5	20.73855	66
18.75901	5	20.75901	15	20.75901	6	20.75901	27
18.77948	6	20.77948	16	20.77948	5	20.77948	71
18.79994	2	20.79994	49	20.79994	3	20.79994	94
18.8204	0	20.8204	12	20.8204	2	20.8204	55
18.84087	5	20.84087	0	20.84087	3	20.84087	51
18.86133	11	20.86133	0	20.86133	5	20.86133	69
18.88179	15	20.88179	0	20.88179	19	20.88179	42
18.90225	17	20.90225	25	20.90225	37	20.90225	30
18.92272	19	20.92272	0	20.92272	45	20.92272	105
18.94318	24	20.94318	47	20.94318	65	20.94318	80
18.96364	26	20.96364	0	20.96364	77	20.96364	160
18.98411	27	20.98411	63	20.98411	88	20.98411	257
19.00457	23	21.00457	51	21.00457	96	21.00457	269
19.02503	21	21.02503	60	21.02503	121	21.02503	365
19.04549	27	21.04549	14	21.04549	147	21.04549	430
19.06596	27	21.06596	32	21.06596	197	21.06596	494
19.08642	31	21.08642	90	21.08642	257	21.08642	566
19.10688	42	21.10688	116	21.10688	310	21.10688	556
19.12734	61	21.12734	167	21.12734	350	21.12734	489
19.14781	92	21.14781	189	21.14781	377	21.14781	328
19.16827	143	21.16827	218	21.16827	375	21.16827	345
19.18873	229	21.18873	237	21.18873	349	21.18873	235
19.2092	364	21.2092	380	21.2092	293	21.2092	130
19.22966	571	21.22966	478	21.22966	247	21.22966	95
19.25012	886	21.25012	439	21.25012	182	21.25012	190
19.27058	1288	21.27058	403	21.27058	140	21.27058	141
19.29105	1699	21.29105	279	21.29105	99	21.29105	186
19.31151	2052	21.31151	238	21.31151	79	21.31151	142
19.33197	2260	21.33197	118	21.33197	63	21.33197	222
19.35244	2273	21.35244	116	21.35244	52	21.35244	163
19.3729	2090	21.3729	59	21.3729	52	21.3729	173
19.39336	1766	21.39336	64	21.39336	51	21.39336	209
19.41382	1338	21.41382	68	21.41382	41	21.41382	263
19.43429	917	21.43429	51	21.43429	45	21.43429	248
19.45475	579	21.45475	64	21.45475	36	21.45475	245
19.47521	346	21.47521	90	21.47521	38	21.47521	270
19.49567	192	21.49567	89	21.49567	38	21.49567	260
19.51614	120	21.51614	86	21.51614	42	21.51614	285
19.5366	81	21.5366	85	21.5366	38	21.5366	334
19.55706	71	21.55706	55	21.55706	31	21.55706	260
19.57753	60	21.57753	39	21.57753	27	21.57753	336
19.59799	70	21.59799	118	21.59799	29	21.59799	336
19.61845	59	21.61845	111	21.61845	19	21.61845	391
19.63891	58	21.63891	94	21.63891	27	21.63891	412

19.65938	42	21.65938	132	21.65938	39	21.65938	427
19.67984	36	21.67984	89	21.67984	50	21.67984	522
19.7003	24	21.7003	90	21.7003	69	21.7003	516
19.72077	14	21.72077	67	21.72077	82	21.72077	482
19.74123	10	21.74123	96	21.74123	97	21.74123	477
19.76169	19	21.76169	192	21.76169	108	21.76169	538
19.78215	11	21.78215	135	21.78215	121	21.78215	541
19.80262	15	21.80262	133	21.80262	136	21.80262	681
19.82308	17	21.82308	192	21.82308	160	21.82308	687
19.84354	15	21.84354	165	21.84354	176	21.84354	745
19.864	9	21.864	202	21.864	212	21.864	842
19.88447	16	21.88447	162	21.88447	247	21.88447	963
19.90493	13	21.90493	179	21.90493	275	21.90493	1123
19.92539	10	21.92539	180	21.92539	323	21.92539	1316
19.94586	7	21.94586	210	21.94586	393	21.94586	1613
19.96632	5	21.96632	318	21.96632	477	21.96632	2072
19.98678	1	21.98678	334	21.98678	590	21.98678	2731
20.00724	0	22.00724	380	22.00724	777	22.00724	3403
20.02771	7	22.02771	440	22.02771	1043	22.02771	3818
20.04817	14	22.04817	578	22.04817	1341	22.04817	4206
20.06863	14	22.06863	685	22.06863	1692	22.06863	4261
20.0891	20	22.0891	881	22.0891	2015	22.0891	3662
20.10956	26	22.10956	1212	22.10956	2234	22.10956	3118
20.13002	29	22.13002	1797	22.13002	2360	22.13002	2586
20.15048	28	22.15048	2322	22.15048	2331	22.15048	2279
20.17095	26	22.17095	2792	22.17095	2153	22.17095	2136
20.19141	14	22.19141	3155	22.19141	1896	22.19141	2238
20.21187	7	22.21187	3108	22.21187	1636	22.21187	2286
20.23233	14	22.23233	2705	22.23233	1423	22.23233	2482
20.2528	17	22.2528	2197	22.2528	1300	22.2528	3115
20.27326	18	22.27326	1857	22.27326	1300	22.27326	3852
20.29372	23	22.29372	1340	22.29372	1450	22.29372	4749
20.31419	31	22.31419	1157	22.31419	1757	22.31419	6177
20.33465	29	22.33465	1085	22.33465	2222	22.33465	7553
20.35511	32	22.35511	1224	22.35511	2813	22.35511	8348
20.37557	23	22.37557	1297	22.37557	3533	22.37557	8634
20.39604	17	22.39604	1672	22.39604	4228	22.39604	8213
20.4165	25	22.4165	2216	22.4165	4786	22.4165	7183
20.43696	31	22.43696	2904	22.43696	5117	22.43696	5776
20.45743	35	22.45743	3802	22.45743	5140	22.45743	4708
20.47789	36	22.47789	5070	22.47789	4846	22.47789	3673
20.49835	32	22.49835	5856	22.49835	4280	22.49835	3063
20.51881	31	22.51881	6314	22.51881	3558	22.51881	2692
20.53928	20	22.53928	6184	22.53928	2778	22.53928	2415
20.55974	13	22.55974	4928	22.55974	2082	22.55974	2304
20.5802	0	22.5802	4025	22.5802	1546	22.5802	2217
20.60066	6	22.60066	2942	22.60066	1129	22.60066	2149
20.62113	16	22.62113	2153	22.62113	851	22.62113	2206
20.64159	22	22.64159	1496	22.64159	669	22.64159	2002
20.66205	24	22.66205	1128	22.66205	548	22.66205	2120
20.68252	23	22.68252	961	22.68252	467	22.68252	2030
20.70298	31	22.70298	872	22.70298	399	22.70298	1971
20.72344	34	22.72344	713	22.72344	347	22.72344	2050
20.7439	32	22.7439	669	22.7439	300	22.7439	1873
20.76437	23	22.76437	585	22.76437	259	22.76437	1890
20.78483	20	22.78483	613	22.78483	220	22.78483	1866
20.80529	25	22.80529	585	22.80529	202	22.80529	1820
20.82576	34	22.82576	490	22.82576	185	22.82576	1830

20.84622	35	22.84622	409	22.84622	166	22.84622	1801
20.86668	36	22.86668	494	22.86668	151	22.86668	1672
20.88714	48	22.88714	375	22.88714	131	22.88714	1730
20.90761	72	22.90761	368	22.90761	113	22.90761	1588
20.92807	91	22.92807	340	22.92807	101	22.92807	1704
20.94853	140	22.94853	403	22.94853	89	22.94853	1542
20.96899	185	22.96899	348	22.96899	77	22.96899	1595
20.98946	257	22.98946	288	22.98946	64	22.98946	1480
21.00992	335	23.00992	324	23.00992	58	23.00992	1415
21.03038	409	23.03038	325	23.03038	49	23.03038	1384
21.05085	446	23.05085	271	23.05085	52	23.05085	1340
21.07131	471	23.07131	326	23.07131	55	23.07131	1205
21.09177	451	23.09177	209	23.09177	49	23.09177	1246
21.11223	402	23.11223	226	23.11223	38	23.11223	1147
21.1327	333	23.1327	189	23.1327	41	23.1327	1182
21.15316	270	23.15316	191	23.15316	38	23.15316	1034
21.17362	192	23.17362	221	23.17362	39	23.17362	986
21.19409	159	23.19409	225	23.19409	33	23.19409	966
21.21455	142	23.21455	165	23.21455	30	23.21455	1078
21.23501	128	23.23501	111	23.23501	21	23.23501	877
21.25547	125	23.25547	197	23.25547	20	23.25547	833
21.27594	144	23.27594	138	23.27594	22	23.27594	832
21.2964	149	23.2964	106	23.2964	18	23.2964	703
21.31686	163	23.31686	93	23.31686	14	23.31686	728
21.33732	172	23.33732	95	23.33732	15	23.33732	617
21.35779	173	23.35779	64	23.35779	16	23.35779	730
21.37825	175	23.37825	61	23.37825	15	23.37825	641
21.39871	193	23.39871	84	23.39871	17	23.39871	622
21.41918	196	23.41918	65	23.41918	15	23.41918	546
21.43964	192	23.43964	28	23.43964	9	23.43964	549
21.4601	198	23.4601	80	23.4601	2	23.4601	499
21.48056	205	23.48056	39	23.48056	7	23.48056	475
21.50103	214	23.50103	52	23.50103	12	23.50103	535
21.52149	221	23.52149	0	23.52149	23	23.52149	437
21.54195	234	23.54195	34	23.54195	29	23.54195	544
21.56242	247	23.56242	40	23.56242	43	23.56242	536
21.58288	283	23.58288	0	23.58288	62	23.58288	540
21.60334	318	23.60334	31	23.60334	88	23.60334	527
21.6238	344	23.6238	57	23.6238	115	23.6238	455
21.64427	373	23.64427	87	23.64427	120	23.64427	422
21.66473	404	23.66473	120	23.66473	129	23.66473	344
21.68519	435	23.68519	89	23.68519	135	23.68519	360
21.70565	466	23.70565	136	23.70565	134	23.70565	252
21.72612	501	23.72612	185	23.72612	115	23.72612	243
21.74658	541	23.74658	211	23.74658	88	23.74658	313
21.76704	561	23.76704	156	23.76704	74	23.76704	217
21.78751	596	23.78751	158	23.78751	60	23.78751	184
21.80797	631	23.80797	83	23.80797	51	23.80797	187
21.82843	675	23.82843	83	23.82843	43	23.82843	171
21.84889	724	23.84889	26	23.84889	23	23.84889	153
21.86936	804	23.86936	2	23.86936	24	23.86936	123
21.88982	918	23.88982	0	23.88982	17	23.88982	175
21.91028	1120	23.91028	20	23.91028	16	23.91028	153
21.93075	1452	23.93075	0	23.93075	6	23.93075	114
21.95121	1911	23.95121	0	23.95121	3	23.95121	97
21.97167	2515	23.97167	3	23.97167	12	23.97167	150
21.99213	3167	23.99213	0	23.99213	20	23.99213	115
22.0126	3747	24.0126	8	24.0126	26	24.0126	81

22.03306	4151	24.03306	0	24.03306	25	24.03306	115
22.05352	4292	24.05352	7	24.05352	26	24.05352	139
22.07398	4147	24.07398	0	24.07398	27	24.07398	97
22.09445	3758	24.09445	53	24.09445	25	24.09445	142
22.11491	3246	24.11491	12	24.11491	29	24.11491	135
22.13537	2731	24.13537	26	24.13537	22	24.13537	144
22.15584	2319	24.15584	0	24.15584	17	24.15584	147
22.1763	2106	24.1763	8	24.1763	27	24.1763	102
22.19676	2121	24.19676	50	24.19676	46	24.19676	203
22.21722	2362	24.21722	0	24.21722	68	24.21722	173
22.23769	2893	24.23769	38	24.23769	90	24.23769	245
22.25815	3771	24.25815	32	24.25815	124	24.25815	266
22.27861	4985	24.27861	11	24.27861	159	24.27861	353
22.29908	6335	24.29908	72	24.29908	203	24.29908	375
22.31954	7550	24.31954	87	24.31954	243	24.31954	547
22.34	8419	24.34	63	24.34	283	24.34	692
22.36046	8740	24.36046	77	24.36046	350	24.36046	1094
22.38093	8451	24.38093	91	24.38093	441	24.38093	1607
22.40139	7621	24.40139	127	24.40139	591	24.40139	2146
22.42185	6370	24.42185	272	24.42185	805	24.42185	2945
22.44231	5043	24.44231	274	24.44231	1100	24.44231	3689
22.46278	3910	24.46278	478	24.46278	1483	24.46278	4351
22.48324	3092	24.48324	578	24.48324	1909	24.48324	4108
22.5037	2534	24.5037	915	24.5037	2288	24.5037	3757
22.52417	2214	24.52417	1351	24.52417	2574	24.52417	3030
22.54463	2078	24.54463	2000	24.54463	2724	24.54463	2224
22.56509	2019	24.56509	2660	24.56509	2685	24.56509	1673
22.58555	1985	24.58555	3399	24.58555	2463	24.58555	1073
22.60602	1931	24.60602	3841	24.60602	2121	24.60602	722
22.62648	1901	24.62648	3597	24.62648	1703	24.62648	479
22.64694	1893	24.64694	2963	24.64694	1295	24.64694	380
22.66741	1875	24.66741	2389	24.66741	943	24.66741	285
22.68787	1858	24.68787	1905	24.68787	671	24.68787	222
22.70833	1818	24.70833	1206	24.70833	469	24.70833	186
22.72879	1817	24.72879	767	24.72879	348	24.72879	158
22.74926	1811	24.74926	522	24.74926	274	24.74926	96
22.76972	1791	24.76972	352	24.76972	219	24.76972	88
22.79018	1766	24.79018	252	24.79018	184	24.79018	127
22.81064	1750	24.81064	205	24.81064	158	24.81064	95
22.83111	1732	24.83111	200	24.83111	130	24.83111	16
22.85157	1707	24.85157	156	24.85157	106	24.85157	128
22.87203	1663	24.87203	93	24.87203	82	24.87203	55
22.8925	1619	24.8925	39	24.8925	69	24.8925	35
22.91296	1570	24.91296	46	24.91296	60	24.91296	70
22.93342	1531	24.93342	44	24.93342	47	24.93342	54
22.95388	1477	24.95388	19	24.95388	31	24.95388	5
22.97435	1422	24.97435	26	24.97435	32	24.97435	7
22.99481	1392	24.99481	36	24.99481	30	24.99481	57
23.01527	1359	25.01527	40	25.01527	28	25.01527	0
23.03574	1304	25.03574	36	25.03574	28	25.03574	0
23.0562	1289	25.0562	53	25.0562	34	25.0562	1
23.07666	1247	25.07666	8	25.07666	36	25.07666	14
23.09712	1200	25.09712	0	25.09712	33	25.09712	0
23.11759	1165	25.11759	3	25.11759	32	25.11759	0
23.13805	1108	25.13805	0	25.13805	27	25.13805	0
23.15851	1043	25.15851	8	25.15851	19	25.15851	47
23.17897	982	25.17897	3	25.17897	22	25.17897	0
23.19944	932	25.19944	46	25.19944	16	25.19944	17

23.2199	875	25.2199	4	25.2199	10	25.2199	12
23.24036	825	25.24036	21	25.24036	9	25.24036	0
23.26083	794	25.26083	0	25.26083	3	25.26083	0
23.28129	757	25.28129	0	25.28129	1	25.28129	38
23.30175	732	25.30175	6	25.30175	0	25.30175	0
23.32221	693	25.32221	7	25.32221	6	25.32221	0
23.34268	655	25.34268	26	25.34268	9	25.34268	0
23.36314	616	25.36314	30	25.36314	9	25.36314	0
23.3836	573	25.3836	0	25.3836	11	25.3836	18
23.40407	536	25.40407	8	25.40407	11	25.40407	22
23.42453	500	25.42453	0	25.42453	10	25.42453	0
23.44499	470	25.44499	54	25.44499	5	25.44499	0
23.46545	450	25.46545	16	25.46545	5	25.46545	0
23.48592	446	25.48592	0	25.48592	11	25.48592	0
23.50638	455	25.50638	46	25.50638	11	25.50638	0
23.52684	454	25.52684	16	25.52684	17	25.52684	28
23.5473	462	25.5473	0	25.5473	16	25.5473	0
23.56777	465	25.56777	0	25.56777	18	25.56777	0
23.58823	443	25.58823	0	25.58823	17	25.58823	35
23.60869	421	25.60869	0	25.60869	14	25.60869	0
23.62916	377	25.62916	34	25.62916	19	25.62916	52
23.64962	341	25.64962	33	25.64962	24	25.64962	113
23.67008	298	25.67008	32	25.67008	45	25.67008	97
23.69054	259	25.69054	7	25.69054	62	25.69054	185
23.71101	225	25.71101	10	25.71101	86	25.71101	206
23.73147	203	25.73147	10	25.73147	116	25.73147	303
23.75193	174	25.75193	91	25.75193	153	25.75193	407
23.7724	161	25.7724	55	25.7724	200	25.7724	619
23.79286	136	25.79286	13	25.79286	261	25.79286	931
23.81332	127	25.81332	93	25.81332	349	25.81332	1113
23.83378	105	25.83378	98	25.83378	473	25.83378	1500
23.85425	107	25.85425	213	25.85425	624	25.85425	1768
23.87471	110	25.87471	233	25.87471	805	25.87471	1830
23.89517	106	25.89517	331	25.89517	980	25.89517	1807
23.91563	101	25.91563	451	25.91563	1144	25.91563	1711
23.9361	99	25.9361	595	25.9361	1238	25.9361	1404
23.95656	92	25.95656	852	25.95656	1266	25.95656	1151
23.97702	95	25.97702	1319	25.97702	1208	25.97702	847
23.99749	87	25.99749	1755	25.99749	1088	25.99749	662
24.01795	82	26.01795	1849	26.01795	921	26.01795	509
24.03841	72	26.03841	1549	26.03841	738	26.03841	351
24.05887	81	26.05887	1419	26.05887	565	26.05887	250
24.07934	97	26.07934	1143	26.07934	429	26.07934	143
24.0998	100	26.0998	839	26.0998	322	26.0998	132
24.12026	105	26.12026	569	26.12026	251	26.12026	113
24.14073	106	26.14073	344	26.14073	187	26.14073	120
24.16119	112	26.16119	235	26.16119	146	26.16119	84
24.18165	121	26.18165	164	26.18165	120	26.18165	69
24.20211	124	26.20211	148	26.20211	107	26.20211	85
24.22258	131	26.22258	138	26.22258	95	26.22258	27
24.24304	157	26.24304	125	26.24304	90	26.24304	48
24.2635	192	26.2635	119	26.2635	84	26.2635	47
24.28396	257	26.28396	111	26.28396	82	26.28396	28
24.30443	361	26.30443	61	26.30443	77	26.30443	89
24.32489	540	26.32489	75	26.32489	73	26.32489	136
24.34535	835	26.34535	61	26.34535	71	26.34535	88
24.36582	1265	26.36582	41	26.36582	77	26.36582	106
24.38628	1824	26.38628	68	26.38628	88	26.38628	180

24.40674	2397	26.40674	50	26.40674	103	26.40674	218
24.4272	2897	26.4272	124	26.4272	124	26.4272	270
24.44767	3214	26.44767	71	26.44767	140	26.44767	288
24.46813	3281	26.46813	92	26.46813	165	26.46813	330
24.48859	3090	26.48859	94	26.48859	198	26.48859	493
24.50906	2666	26.50906	128	26.50906	246	26.50906	652
24.52952	2105	26.52952	149	26.52952	306	26.52952	813
24.54998	1518	26.54998	157	26.54998	392	26.54998	1162
24.57044	1018	26.57044	174	26.57044	510	26.57044	1626
24.59091	659	26.59091	229	26.59091	663	26.59091	2464
24.61137	411	26.61137	277	26.61137	866	26.61137	3393
24.63183	275	26.63183	402	26.63183	1195	26.63183	4614
24.65229	198	26.65229	493	26.65229	1604	26.65229	5619
24.67276	163	26.67276	633	26.67276	2122	26.67276	5975
24.69322	137	26.69322	984	26.69322	2657	26.69322	5800
24.71368	114	26.71368	1374	26.71368	3127	26.71368	5084
24.73415	88	26.73415	1985	26.73415	3455	26.73415	4422
24.75461	59	26.75461	2804	26.75461	3592	26.75461	3527
24.77507	48	26.77507	3685	26.77507	3491	26.77507	2492
24.79553	35	26.79553	4483	26.79553	3187	26.79553	1790
24.816	28	26.816	5025	26.816	2720	26.816	1188
24.83646	25	26.83646	4798	26.83646	2212	26.83646	878
24.85692	22	26.85692	4248	26.85692	1695	26.85692	595
24.87739	17	26.87739	3393	26.87739	1272	26.87739	548
24.89785	6	26.89785	2738	26.89785	934	26.89785	434
24.91831	3	26.91831	1825	26.91831	680	26.91831	349
24.93877	0	26.93877	1283	26.93877	518	26.93877	311
24.95924	2	26.95924	859	26.95924	421	26.95924	322
24.9797	11	26.9797	656	26.9797	360	26.9797	183
25.00016	25	27.00016	462	27.00016	338	27.00016	155
25.02062	34	27.02062	372	27.02062	310	27.02062	163
25.04109	36	27.04109	339	27.04109	274	27.04109	58
25.06155	38	27.06155	319	27.06155	236	27.06155	91
25.08201	33	27.08201	249	27.08201	195	27.08201	104
25.10248	25	27.10248	228	27.10248	153	27.10248	81
25.12294	15	27.12294	251	27.12294	124	27.12294	45
25.1434	12	27.1434	158	27.1434	105	27.1434	62
25.16386	18	27.16386	119	27.16386	87	27.16386	23
25.18433	22	27.18433	127	27.18433	81	27.18433	19
25.20479	29	27.20479	38	27.20479	75	27.20479	49
25.22525	26	27.22525	59	27.22525	63	27.22525	55
25.24572	24	27.24572	34	27.24572	49	27.24572	7
25.26618	22	27.26618	37	27.26618	48	27.26618	25
25.28664	14	27.28664	5	27.28664	37	27.28664	0
25.3071	6	27.3071	0	27.3071	34	27.3071	0
25.32757	1	27.32757	64	27.32757	40	27.32757	0
25.34803	6	27.34803	10	27.34803	38	27.34803	0
25.36849	15	27.36849	5	27.36849	31	27.36849	52
25.38895	17	27.38895	54	27.38895	37	27.38895	25
25.40942	17	27.40942	28	27.40942	33	27.40942	0
25.42988	14	27.42988	13	27.42988	23	27.42988	30
25.45034	22	27.45034	23	27.45034	13	27.45034	15
25.47081	28	27.47081	0	27.47081	18	27.47081	1
25.49127	38	27.49127	0	27.49127	16	27.49127	66
25.51173	40	27.51173	5	27.51173	15	27.51173	0
25.53219	56	27.53219	39	27.53219	13	27.53219	19
25.55266	65	27.55266	33	27.55266	16	27.55266	39
25.57312	70	27.57312	65	27.57312	15	27.57312	19

25.59358	76	27.59358	0	27.59358	33	27.59358	0
25.61405	83	27.61405	38	27.61405	40	27.61405	0
25.63451	93	27.63451	15	27.63451	44	27.63451	16
25.65497	118	27.65497	27	27.65497	49	27.65497	85
25.67543	145	27.67543	18	27.67543	59	27.67543	24
25.6959	206	27.6959	0	27.6959	59	27.6959	63
25.71636	306	27.71636	0	27.71636	61	27.71636	113
25.73682	474	27.73682	1	27.73682	69	27.73682	166
25.75728	737	27.75728	39	27.75728	81	27.75728	139
25.77775	1136	27.77775	1	27.77775	98	27.77775	196
25.79821	1608	27.79821	53	27.79821	120	27.79821	289
25.81867	2056	27.81867	52	27.81867	152	27.81867	382
25.83914	2390	27.83914	46	27.83914	190	27.83914	478
25.8596	2566	27.8596	69	27.8596	255	27.8596	678
25.88006	2522	27.88006	86	27.88006	321	27.88006	977
25.90052	2271	27.90052	115	27.90052	431	27.90052	1326
25.92099	1875	27.92099	230	27.92099	583	27.92099	1777
25.94145	1395	27.94145	251	27.94145	784	27.94145	2269
25.96191	960	27.96191	323	27.96191	1046	27.96191	2618
25.98238	645	27.98238	476	27.98238	1351	27.98238	2719
26.00284	415	28.00284	639	28.00284	1636	28.00284	2618
26.0233	263	28.0233	969	28.0233	1885	28.0233	2147
26.04376	169	28.04376	1323	28.04376	2044	28.04376	1756
26.06423	140	28.06423	1989	28.06423	2096	28.06423	1409
26.08469	115	28.08469	2511	28.08469	2010	28.08469	1040
26.10515	97	28.10515	2905	28.10515	1825	28.10515	694
26.12561	94	28.12561	3089	28.12561	1556	28.12561	509
26.14608	87	28.14608	2671	28.14608	1257	28.14608	399
26.16654	87	28.16654	2188	28.16654	986	28.16654	274
26.187	87	28.187	1732	28.187	761	28.187	170
26.20747	73	28.20747	1234	28.20747	574	28.20747	178
26.22793	65	28.22793	914	28.22793	436	28.22793	185
26.24839	60	28.24839	573	28.24839	337	28.24839	117
26.26885	62	28.26885	388	28.26885	268	28.26885	75
26.28932	55	28.28932	339	28.28932	218	28.28932	0
26.30978	57	28.30978	245	28.30978	171	28.30978	64
26.33024	73	28.33024	193	28.33024	139	28.33024	59
26.35071	100	28.35071	130	28.35071	114	28.35071	46
26.37117	123	28.37117	115	28.37117	112	28.37117	68
26.39163	151	28.39163	67	28.39163	119	28.39163	66
26.41209	176	28.41209	92	28.41209	127	28.41209	82
26.43256	221	28.43256	75	28.43256	130	28.43256	73
26.45302	289	28.45302	107	28.45302	131	28.45302	137
26.47348	371	28.47348	75	28.47348	139	28.47348	160
26.49394	474	28.49394	109	28.49394	144	28.49394	163
26.51441	647	28.51441	114	28.51441	157	28.51441	226
26.53487	945	28.53487	71	28.53487	179	28.53487	256
26.55533	1485	28.55533	92	28.55533	216	28.55533	359
26.5758	2278	28.5758	193	28.5758	265	28.5758	449
26.59626	3235	28.59626	121	28.59626	318	28.59626	632
26.61672	4203	28.61672	142	28.61672	400	28.61672	945
26.63718	4992	28.63718	147	28.63718	502	28.63718	1414
26.65765	5523	28.65765	232	28.65765	648	28.65765	1981
26.67811	5620	28.67811	296	28.67811	858	28.67811	2732
26.69857	5248	28.69857	347	28.69857	1145	28.69857	3571
26.71904	4508	28.71904	498	28.71904	1564	28.71904	4103
26.7395	3548	28.7395	713	28.7395	2038	28.7395	4499
26.75996	2608	28.75996	964	28.75996	2520	28.75996	4114

26.78042	1814	28.78042	1342	28.78042	2920	28.78042	3533
26.80089	1204	28.80089	2014	28.80089	3173	28.80089	2969
26.82135	774	28.82135	2867	28.82135	3236	28.82135	2366
26.84181	502	28.84181	3684	28.84181	3096	28.84181	1671
26.86227	386	28.86227	3975	28.86227	2782	28.86227	1113
26.88274	323	28.88274	3710	28.88274	2341	28.88274	834
26.9032	290	28.9032	3197	28.9032	1865	28.9032	519
26.92366	274	28.92366	2683	28.92366	1444	28.92366	409
26.94413	246	28.94413	2114	28.94413	1081	28.94413	346
26.96459	206	28.96459	1529	28.96459	794	28.96459	185
26.98505	176	28.98505	1002	28.98505	580	28.98505	193
27.00551	146	29.00551	674	29.00551	425	29.00551	99
27.02598	116	29.02598	514	29.02598	321	29.02598	61
27.04644	91	29.04644	380	29.04644	256	29.04644	124
27.0669	72	29.0669	296	29.0669	214	29.0669	91
27.08737	61	29.08737	313	29.08737	189	29.08737	6
27.10783	53	29.10783	155	29.10783	167	29.10783	31
27.12829	45	29.12829	166	29.12829	141	29.12829	30
27.14875	35	29.14875	122	29.14875	125	29.14875	70
27.16922	24	29.16922	97	29.16922	95	29.16922	4
27.18968	15	29.18968	106	29.18968	75	29.18968	17
27.21014	11	29.21014	79	29.21014	58	29.21014	35
27.2306	9	29.2306	33	29.2306	44	29.2306	3
27.25107	2	29.25107	0	29.25107	30	29.25107	21
27.27153	11	29.27153	36	29.27153	37	29.27153	39
27.29199	7	29.29199	58	29.29199	48	29.29199	16
27.31246	14	29.31246	39	29.31246	45	29.31246	0
27.33292	18	29.33292	32	29.33292	43	29.33292	11
27.35338	19	29.35338	72	29.35338	40	29.35338	0
27.37384	19	29.37384	47	29.37384	32	29.37384	0
27.39431	16	29.39431	46	29.39431	29	29.39431	32
27.41477	12	29.41477	27	29.41477	20	29.41477	0
27.43523	10	29.43523	28	29.43523	20	29.43523	20
27.4557	5	29.4557	0	29.4557	23	29.4557	38
27.47616	6	29.47616	59	29.47616	33	29.47616	0
27.49662	2	29.49662	0	29.49662	30	29.49662	10
27.51708	11	29.51708	9	29.51708	21	29.51708	0
27.53755	21	29.53755	4	29.53755	19	29.53755	13
27.55801	23	29.55801	0	29.55801	15	29.55801	21
27.57847	33	29.57847	0	29.57847	20	29.57847	13
27.59893	38	29.59893	0	29.59893	21	29.59893	0
27.6194	44	29.6194	43	29.6194	29	29.6194	0
27.63986	58	29.63986	36	29.63986	41	29.63986	13
27.66032	61	29.66032	11	29.66032	56	29.66032	54
27.68079	81	29.68079	22	29.68079	54	29.68079	57
27.70125	102	29.70125	13	29.70125	58	29.70125	117
27.72171	122	29.72171	41	29.72171	66	29.72171	113
27.74217	151	29.74217	25	29.74217	79	29.74217	94
27.76264	192	29.76264	35	29.76264	92	29.76264	154
27.7831	248	29.7831	80	29.7831	115	29.7831	218
27.80356	343	29.80356	64	29.80356	147	29.80356	360
27.82403	497	29.82403	69	29.82403	186	29.82403	520
27.84449	749	29.84449	74	29.84449	233	29.84449	753
27.86495	1128	29.86495	88	29.86495	314	29.86495	1282
27.88541	1636	29.88541	114	29.88541	416	29.88541	1758
27.90588	2138	29.90588	161	29.90588	595	29.90588	2408
27.92634	2571	29.92634	225	29.92634	833	29.92634	3027
27.9468	2869	29.9468	348	29.9468	1120	29.9468	3119

27.96726	2983	29.96726	464	29.96726	1425	29.96726	3082
27.98773	2872	29.98773	611	29.98773	1686	29.98773	2822
28.00819	2549	30.00819	1030	30.00819	1875	30.00819	2287
28.02865	2082	30.02865	1476	30.02865	1948	30.02865	1906
28.04912	1584	30.04912	1777	30.04912	1903	30.04912	1400
28.06958	1155	30.06958	1880	30.06958	1743	30.06958	1021
28.09004	822	30.09004	2011	30.09004	1495	30.09004	628
28.1105	544	30.1105	1614	30.1105	1224	30.1105	443
28.13097	363	30.13097	1344	30.13097	946	30.13097	417
28.15143	245	30.15143	1238	30.15143	710	30.15143	277
28.17189	192	30.17189	924	30.17189	523	30.17189	224
28.19236	168	30.19236	624	30.19236	379	30.19236	128
28.21282	133	30.21282	406	30.21282	281	30.21282	155
28.23328	108	30.23328	268	30.23328	218	30.23328	115
28.25374	90	30.25374	149	30.25374	177	30.25374	108
28.27421	78	30.27421	173	30.27421	153	30.27421	64
28.29467	73	30.29467	178	30.29467	135	30.29467	23
28.31513	62	30.31513	67	30.31513	131	30.31513	21
28.33559	63	30.33559	112	30.33559	113	30.33559	71
28.35606	57	30.35606	53	30.35606	102	30.35606	55
28.37652	65	30.37652	101	30.37652	100	30.37652	43
28.39698	78	30.39698	97	30.39698	95	30.39698	61
28.41745	97	30.41745	60	30.41745	85	30.41745	73
28.43791	116	30.43791	22	30.43791	69	30.43791	63
28.45837	147	30.45837	21	30.45837	65	30.45837	127
28.47883	166	30.47883	51	30.47883	65	30.47883	127
28.4993	217	30.4993	100	30.4993	76	30.4993	94
28.51976	270	30.51976	44	30.51976	91	30.51976	179
28.54022	354	30.54022	89	30.54022	103	30.54022	181
28.56068	482	30.56068	37	30.56068	124	30.56068	247
28.58115	725	30.58115	41	30.58115	148	30.58115	318
28.60161	1134	30.60161	71	30.60161	178	30.60161	416
28.62207	1759	30.62207	85	30.62207	215	30.62207	601
28.64254	2553	30.64254	98	30.64254	254	30.64254	570
28.663	3424	30.663	130	30.663	323	30.663	649
28.68346	4169	30.68346	106	30.68346	382	30.68346	741
28.70392	4673	30.70392	248	30.70392	449	30.70392	713
28.72439	4827	30.72439	354	30.72439	512	30.72439	711
28.74485	4617	30.74485	448	30.74485	576	30.74485	888
28.76531	4065	30.76531	545	30.76531	617	30.76531	1030
28.78578	3299	30.78578	554	30.78578	695	30.78578	1299
28.80624	2488	30.80624	497	30.80624	798	30.80624	1811
28.8267	1756	30.8267	572	30.8267	958	30.8267	2460
28.84716	1197	30.84716	582	30.84716	1189	30.84716	3253
28.86763	801	30.86763	697	30.86763	1538	30.86763	3725
28.88809	516	30.88809	756	30.88809	1926	30.88809	3859
28.90855	333	30.90855	976	30.90855	2325	30.90855	3864
28.92901	243	30.92901	1332	30.92901	2681	30.92901	3338
28.94948	196	30.94948	2000	30.94948	2911	30.94948	2955
28.96994	166	30.96994	2784	30.96994	2982	30.96994	2449
28.9904	138	30.9904	3409	30.9904	2904	30.9904	1881
29.01087	125	31.01087	3856	31.01087	2650	31.01087	1421
29.03133	101	31.03133	3804	31.03133	2277	31.03133	963
29.05179	90	31.05179	3216	31.05179	1871	31.05179	635
29.07225	71	31.07225	2789	31.07225	1490	31.07225	501
29.09272	56	31.09272	2327	31.09272	1138	31.09272	389
29.11318	46	31.11318	1871	31.11318	847	31.11318	300
29.13364	38	31.13364	1265	31.13364	636	31.13364	202

29.15411	21	31.15411	828	31.15411	477	31.15411	204
29.17457	12	31.17457	542	31.17457	369	31.17457	94
29.19503	11	31.19503	430	31.19503	285	31.19503	124
29.21549	15	31.21549	297	31.21549	223	31.21549	60
29.23596	16	31.23596	269	31.23596	162	31.23596	54
29.25642	21	31.25642	168	31.25642	134	31.25642	61
29.27688	22	31.27688	113	31.27688	97	31.27688	88
29.29734	22	31.29734	120	31.29734	75	31.29734	53
29.31781	17	31.31781	117	31.31781	59	31.31781	65
29.33827	10	31.33827	91	31.33827	56	31.33827	100
29.35873	4	31.35873	49	31.35873	46	31.35873	100
29.3792	5	31.3792	48	31.3792	38	31.3792	32
29.39966	10	31.39966	0	31.39966	34	31.39966	0
29.42012	9	31.42012	18	31.42012	31	31.42012	0
29.44058	10	31.44058	0	31.44058	24	31.44058	57
29.46105	13	31.46105	41	31.46105	22	31.46105	8
29.48151	16	31.48151	0	31.48151	11	31.48151	15
29.50197	17	31.50197	25	31.50197	9	31.50197	12
29.52244	21	31.52244	0	31.52244	8	31.52244	39
29.5429	20	31.5429	0	31.5429	15	31.5429	22
29.56336	21	31.56336	6	31.56336	20	31.56336	76
29.58382	26	31.58382	0	31.58382	25	31.58382	21
29.60429	26	31.60429	0	31.60429	20	31.60429	60
29.62475	36	31.62475	1	31.62475	24	31.62475	45
29.64521	43	31.64521	15	31.64521	28	31.64521	77
29.66567	63	31.66567	0	31.66567	40	31.66567	141
29.68614	90	31.68614	0	31.68614	60	31.68614	187
29.7066	115	31.7066	18	31.7066	82	31.7066	316
29.72706	151	31.72706	0	31.72706	108	31.72706	398
29.74753	197	31.74753	12	31.74753	161	31.74753	576
29.76799	270	31.76799	33	31.76799	236	31.76799	701
29.78845	384	31.78845	69	31.78845	322	31.78845	920
29.80891	576	31.80891	90	31.80891	416	31.80891	896
29.82938	880	31.82938	137	31.82938	522	31.82938	835
29.84984	1292	31.84984	285	31.84984	616	31.84984	687
29.8703	1775	31.8703	423	31.8703	671	31.8703	681
29.89077	2216	31.89077	603	31.89077	695	31.89077	487
29.91123	2561	31.91123	754	31.91123	668	31.91123	362
29.93169	2742	31.93169	872	31.93169	600	31.93169	285
29.95215	2755	31.95215	779	31.95215	519	31.95215	183
29.97262	2549	31.97262	588	31.97262	419	31.97262	86
29.99308	2176	31.99308	561	31.99308	326	31.99308	82
30.01354	1726	32.01354	511	32.01354	243	32.01354	27
30.034	1292	32.034	377	32.034	183	32.034	47
30.05447	937	32.05447	224	32.05447	137	32.05447	37
30.07493	646	32.07493	133	32.07493	93	32.07493	37
30.09539	426	32.09539	97	32.09539	69	32.09539	25
30.11586	285	32.11586	59	32.11586	52	32.11586	72
30.13632	192	32.13632	38	32.13632	43	32.13632	54
30.15678	154	32.15678	0	32.15678	37	32.15678	54
30.17724	125	32.17724	18	32.17724	33	32.17724	93
30.19771	99	32.19771	25	32.19771	34	32.19771	51
30.21817	74	32.21817	0	32.21817	33	32.21817	140
30.23863	73	32.23863	36	32.23863	38	32.23863	117
30.2591	72	32.2591	8	32.2591	45	32.2591	162
30.27956	58	32.27956	15	32.27956	55	32.27956	177
30.30002	59	32.30002	0	32.30002	69	32.30002	210
30.32048	55	32.32048	73	32.32048	94	32.32048	258

30.34095	46	32.34095	99	32.34095	123	32.34095	371
30.36141	51	32.36141	100	32.36141	172	32.36141	532
30.38187	57	32.38187	71	32.38187	249	32.38187	749
30.40233	62	32.40233	123	32.40233	321	32.40233	1050
30.4228	60	32.4228	124	32.4228	424	32.4228	1427
30.44326	73	32.44326	205	32.44326	573	32.44326	1787
30.46372	79	32.46372	217	32.46372	769	32.46372	2222
30.48419	98	32.48419	352	32.48419	997	32.48419	2342
30.50465	126	32.50465	427	32.50465	1222	32.50465	2439
30.52511	188	32.52511	700	32.52511	1400	32.52511	2237
30.54557	268	32.54557	1016	32.54557	1505	32.54557	1964
30.56604	366	32.56604	1504	32.56604	1548	32.56604	1700
30.5865	464	32.5865	1965	32.5865	1509	32.5865	1539
30.60696	565	32.60696	2185	32.60696	1378	32.60696	1190
30.62743	648	32.62743	1956	32.62743	1207	32.62743	890
30.64789	717	32.64789	1704	32.64789	1032	32.64789	752
30.66835	738	32.66835	1455	32.66835	878	32.66835	685
30.68881	766	32.68881	1339	32.68881	756	32.68881	631
30.70928	808	32.70928	1036	32.70928	654	32.70928	594
30.72974	978	32.72974	763	32.72974	571	32.72974	454
30.7502	1265	32.7502	614	32.7502	512	32.7502	424
30.77066	1761	32.77066	537	32.77066	460	32.77066	325
30.79113	2501	32.79113	517	32.79113	417	32.79113	286
30.81159	3332	32.81159	501	32.81159	373	32.81159	224
30.83205	4095	32.83205	441	32.83205	320	32.83205	206
30.85252	4662	32.85252	335	32.85252	268	32.85252	104
30.87298	4927	32.87298	270	32.87298	216	32.87298	61
30.89344	4881	32.89344	229	32.89344	164	32.89344	61
30.9139	4492	32.9139	206	32.9139	123	32.9139	22
30.93437	3846	32.93437	143	32.93437	97	32.93437	21
30.95483	3044	32.95483	126	32.95483	71	32.95483	46
30.97529	2287	32.97529	67	32.97529	41	32.97529	47
30.99576	1674	32.99576	62	32.99576	32	32.99576	0
31.01622	1166	33.01622	57	33.01622	29	33.01622	0
31.03668	775	33.03668	6	33.03668	24	33.03668	39
31.05714	501	33.05714	0	33.05714	20	33.05714	5
31.07761	336	33.07761	49	33.07761	10	33.07761	30
31.09807	256	33.09807	3	33.09807	5	33.09807	0
31.11853	211	33.11853	8	33.11853	10	33.11853	0
31.13899	169	33.13899	0	33.13899	11	33.13899	0
31.15946	133	33.15946	0	33.15946	7	33.15946	0
31.17992	108	33.17992	11	33.17992	7	33.17992	9
31.20038	95	33.20038	0	33.20038	12	33.20038	0
31.22085	81	33.22085	18	33.22085	11	33.22085	0
31.24131	72	33.24131	0	33.24131	7	33.24131	12
31.26177	47	33.26177	22	33.26177	3	33.26177	0
31.28223	34	33.28223	0	33.28223	4	33.28223	0
31.3027	17	33.3027	0	33.3027	10	33.3027	0
31.32316	14	33.32316	0	33.32316	12	33.32316	39
31.34362	15	33.34362	0	33.34362	12	33.34362	8
31.36409	16	33.36409	3	33.36409	8	33.36409	17
31.38455	22	33.38455	0	33.38455	12	33.38455	1
31.40501	26	33.40501	7	33.40501	13	33.40501	3
31.42547	29	33.42547	1	33.42547	8	33.42547	0
31.44594	30	33.44594	0	33.44594	9	33.44594	0
31.4664	24	33.4664	8	33.4664	12	33.4664	49
31.48686	23	33.48686	2	33.48686	28	33.48686	90
31.50732	10	33.50732	4	33.50732	37	33.50732	122

31.52779	2	33.52779	14	33.52779	46	33.52779	93
31.54825	2	33.54825	0	33.54825	52	33.54825	165
31.56871	12	33.56871	0	33.56871	68	33.56871	184
31.58918	24	33.58918	53	33.58918	87	33.58918	181
31.60964	48	33.60964	42	33.60964	90	33.60964	208
31.6301	87	33.6301	38	33.6301	100	33.6301	184
31.65056	126	33.65056	88	33.65056	100	33.65056	185
31.67103	226	33.67103	157	33.67103	103	33.67103	148
31.69149	355	33.69149	103	33.69149	106	33.69149	81
31.71195	523	33.71195	142	33.71195	101	33.71195	38
31.73242	695	33.73242	68	33.73242	96	33.73242	53
31.75288	849	33.75288	93	33.75288	90	33.75288	56
31.77334	959	33.77334	56	33.77334	95	33.77334	95
31.7938	1006	33.7938	78	33.7938	80	33.7938	79
31.81427	975	33.81427	87	33.81427	68	33.81427	96
31.83473	865	33.83473	23	33.83473	60	33.83473	106
31.85519	699	33.85519	0	33.85519	42	33.85519	81
31.87565	546	33.87565	7	33.87565	37	33.87565	113
31.89612	390	33.89612	31	33.89612	38	33.89612	156
31.91658	272	33.91658	41	33.91658	48	33.91658	152
31.93704	175	33.93704	36	33.93704	61	33.93704	91
31.95751	102	33.95751	17	33.95751	88	33.95751	166
31.97797	60	33.97797	23	33.97797	119	33.97797	242
31.99843	42	33.99843	65	33.99843	148	33.99843	282
32.01889	27	34.01889	34	34.01889	188	34.01889	334
32.03936	19	34.03936	61	34.03936	229	34.03936	463
32.05982	12	34.05982	78	34.05982	272	34.05982	625
32.08028	6	34.08028	110	34.08028	334	34.08028	762
32.10075	0	34.10075	147	34.10075	408	34.10075	1130
32.12121	2	34.12121	193	34.12121	518	34.12121	1512
32.14167	13	34.14167	234	34.14167	682	34.14167	2301
32.16213	22	34.16213	261	34.16213	932	34.16213	2871
32.1826	38	34.1826	376	34.1826	1245	34.1826	3744
32.20306	54	34.20306	538	34.20306	1633	34.20306	4183
32.22352	73	34.22352	746	34.22352	2067	34.22352	4212
32.24398	108	34.24398	1236	34.24398	2463	34.24398	3856
32.26445	148	34.26445	1654	34.26445	2761	34.26445	3397
32.28491	187	34.28491	2482	34.28491	2926	34.28491	2975
32.30537	245	34.30537	3274	34.30537	2925	34.30537	2543
32.32584	346	34.32584	3816	34.32584	2797	34.32584	1939
32.3463	529	34.3463	3874	34.3463	2533	34.3463	1477
32.36676	821	34.36676	3435	34.36676	2195	34.36676	1021
32.38722	1210	34.38722	2870	34.38722	1791	34.38722	744
32.40769	1645	34.40769	2500	34.40769	1435	34.40769	575
32.42815	2027	34.42815	2228	34.42815	1120	34.42815	369
32.44861	2307	34.44861	1616	34.44861	827	34.44861	277
32.46908	2446	34.46908	1119	34.46908	596	34.46908	228
32.48954	2410	34.48954	716	34.48954	417	34.48954	222
32.51	2216	34.51	439	34.51	300	34.51	187
32.53046	1907	34.53046	344	34.53046	227	34.53046	168
32.55093	1567	34.55093	290	34.55093	176	34.55093	107
32.57139	1257	34.57139	220	34.57139	149	34.57139	88
32.59185	1029	34.59185	159	34.59185	109	34.59185	110
32.61231	867	34.61231	139	34.61231	100	34.61231	22
32.63278	742	34.63278	120	34.63278	80	34.63278	56
32.65324	649	34.65324	40	34.65324	57	34.65324	41
32.6737	600	34.6737	107	34.6737	54	34.6737	34
32.69417	556	34.69417	64	34.69417	43	34.69417	30

32.71463	504	34.71463	4	34.71463	36	34.71463	48
32.73509	443	34.73509	1	34.73509	19	34.73509	15
32.75555	367	34.75555	58	34.75555	21	34.75555	75
32.77602	275	34.77602	8	34.77602	25	34.77602	69
32.79648	210	34.79648	21	34.79648	16	34.79648	23
32.81694	144	34.81694	0	34.81694	17	34.81694	20
32.83741	101	34.83741	8	34.83741	19	34.83741	18
32.85787	71	34.85787	0	34.85787	14	34.85787	0
32.87833	57	34.87833	2	34.87833	21	34.87833	28
32.89879	44	34.89879	0	34.89879	20	34.89879	68
32.91926	34	34.91926	0	34.91926	19	34.91926	69
32.93972	31	34.93972	44	34.93972	22	34.93972	65
32.96018	28	34.96018	0	34.96018	34	34.96018	119
32.98064	20	34.98064	2	34.98064	44	34.98064	74
33.00111	11	35.00111	41	35.00111	53	35.00111	88
33.02157	2	35.02157	0	35.02157	71	35.02157	114
33.04203	4	35.04203	0	35.04203	83	35.04203	155
33.0625	10	35.0625	32	35.0625	91	35.0625	158
33.08296	11	35.08296	13	35.08296	104	35.08296	205
33.10342	10	35.10342	35	35.10342	121	35.10342	336
33.12388	6	35.12388	40	35.12388	147	35.12388	425
33.14435	6	35.14435	72	35.14435	182	35.14435	547
33.16481	7	35.16481	100	35.16481	235	35.16481	751
33.18527	5	35.18527	101	35.18527	307	35.18527	1043
33.20574	3	35.20574	133	35.20574	411	35.20574	1405
33.2262	6	35.2262	172	35.2262	541	35.2262	1723
33.24666	6	35.24666	251	35.24666	713	35.24666	1768
33.26712	5	35.26712	352	35.26712	876	35.26712	1767
33.28759	1	35.28759	478	35.28759	1011	35.28759	1755
33.30805	0	35.30805	748	35.30805	1129	35.30805	1494
33.32851	2	35.32851	987	35.32851	1192	35.32851	1294
33.34897	6	35.34897	1317	35.34897	1185	35.34897	1127
33.36944	11	35.36944	1463	35.36944	1118	35.36944	877
33.3899	11	35.3899	1388	35.3899	997	35.3899	654
33.41036	15	35.41036	1203	35.41036	847	35.41036	463
33.43083	33	35.43083	1109	35.43083	686	35.43083	315
33.45129	65	35.45129	873	35.45129	556	35.45129	217
33.47175	98	35.47175	750	35.47175	419	35.47175	184
33.49221	139	35.49221	623	35.49221	302	35.49221	180
33.51268	174	35.51268	424	35.51268	237	35.51268	93
33.53314	207	35.53314	267	35.53314	189	35.53314	106
33.5536	220	35.5536	195	35.5536	160	35.5536	104
33.57407	222	35.57407	130	35.57407	129	35.57407	96
33.59453	203	35.59453	67	35.59453	108	35.59453	98
33.61499	173	35.61499	109	35.61499	86	35.61499	78
33.63545	146	35.63545	90	35.63545	64	35.63545	56
33.65592	114	35.65592	58	35.65592	51	35.65592	87
33.67638	81	35.67638	29	35.67638	34	35.67638	91
33.69684	56	35.69684	28	35.69684	27	35.69684	97
33.7173	31	35.7173	56	35.7173	41	35.7173	138
33.73777	16	35.73777	27	35.73777	50	35.73777	111
33.75823	24	35.75823	70	35.75823	69	35.75823	154
33.77869	39	35.77869	90	35.77869	78	35.77869	171
33.79916	55	35.79916	73	35.79916	107	35.79916	308
33.81962	63	35.81962	59	35.81962	144	35.81962	330
33.84008	79	35.84008	86	35.84008	192	35.84008	423
33.86054	92	35.86054	98	35.86054	247	35.86054	519
33.88101	104	35.88101	97	35.88101	290	35.88101	515

33.90147	121	35.90147	166	35.90147	330	35.90147	501
33.92193	143	35.92193	238	35.92193	367	35.92193	399
33.9424	164	35.9424	331	35.9424	378	35.9424	366
33.96286	214	35.96286	392	35.96286	374	35.96286	355
33.98332	263	35.98332	475	35.98332	342	35.98332	241
34.00378	323	36.00378	448	36.00378	314	36.00378	173
34.02425	426	36.02425	407	36.02425	276	36.02425	130
34.04471	596	36.04471	374	36.04471	233	36.04471	89
34.06517	893	36.06517	298	36.06517	187	36.06517	38
34.08564	1363	36.08564	250	36.08564	136	36.08564	74
34.1061	2026	36.1061	229	36.1061	95	36.1061	20
34.12656	2758	36.12656	96	36.12656	73	36.12656	37
34.14702	3417	36.14702	98	36.14702	52	36.14702	50
34.16748	3916	36.16748	60	36.16748	38	36.16748	0
34.18795	4163	36.18795	48	36.18795	21	36.18795	14
34.20841	4156	36.20841	48	36.20841	26	36.20841	0
34.22887	3906	36.22887	10	36.22887	22	36.22887	20
34.24934	3406	36.24934	0	36.24934	19	36.24934	0
34.2698	2801	36.2698	0	36.2698	14	36.2698	23
34.29026	2206	36.29026	27	36.29026	9	36.29026	4
34.31073	1693	36.31073	0	36.31073	9	36.31073	0
34.33119	1257	36.33119	28	36.33119	10	36.33119	0
34.35165	874	36.35165	9	36.35165	6	36.35165	34
34.37211	575	36.37211	0	36.37211	5	36.37211	5
34.39258	378	36.39258	0	36.39258	0	36.39258	3
34.41304	276	36.41304	10	36.41304	4	36.41304	30
34.4335	239	36.4335	0	36.4335	2	36.4335	5
34.45396	197	36.45396	17	36.45396	4	36.45396	53
34.47443	168	36.47443	3	36.47443	5	36.47443	0
34.49489	141	36.49489	12	36.49489	4	36.49489	48
34.51535	116	36.51535	0	36.51535	8	36.51535	0
34.53582	100	36.53582	0	36.53582	8	36.53582	24
34.55628	75	36.55628	0	36.55628	5	36.55628	40
34.57674	63	36.57674	61	36.57674	7	36.57674	0
34.59721	53	36.59721	27	36.59721	9	36.59721	1
34.61767	40	36.61767	8	36.61767	9	36.61767	39
34.63813	33	36.63813	0	36.63813	9	36.63813	45
34.65859	23	36.65859	41	36.65859	11	36.65859	43
34.67905	14	36.67905	26	36.67905	9	36.67905	31
34.69952	7	36.69952	0	36.69952	4	36.69952	32
34.71998	12	36.71998	12	36.71998	0	36.71998	27
34.74044	12	36.74044	35	36.74044	2	36.74044	90
34.76091	11	36.76091	14	36.76091	10	36.76091	83
34.78137	18	36.78137	56	36.78137	21	36.78137	97
34.80183	17	36.80183	40	36.80183	41	36.80183	125
34.8223	21	36.8223	49	36.8223	62	36.8223	187
34.84276	22	36.84276	31	36.84276	90	36.84276	254
34.86322	18	36.86322	0	36.86322	123	36.86322	198
34.88368	11	36.88368	69	36.88368	149	36.88368	234
34.90414	8	36.90414	59	36.90414	168	36.90414	246
34.92461	20	36.92461	151	36.92461	176	36.92461	187
34.94507	26	36.94507	91	36.94507	183	36.94507	181
34.96553	42	36.96553	225	36.96553	181	36.96553	178
34.986	72	36.986	262	36.986	161	36.986	109
35.00646	94	37.00646	243	37.00646	143	37.00646	105
35.02692	126	37.02692	194	37.02692	123	37.02692	87
35.04739	152	37.04739	178	37.04739	111	37.04739	49
35.06785	192	37.06785	167	37.06785	93	37.06785	56

35.08831	266	37.08831	130	37.08831	82	37.08831	84
35.10877	381	37.10877	144	37.10877	66	37.10877	79
35.12924	576	37.12924	69	37.12924	59	37.12924	58
35.1497	848	37.1497	42	37.1497	52	37.1497	135
35.17016	1164	37.17016	70	37.17016	46	37.17016	130
35.19062	1454	37.19062	55	37.19062	45	37.19062	91
35.21109	1667	37.21109	57	37.21109	55	37.21109	118
35.23155	1780	37.23155	52	37.23155	70	37.23155	163
35.25201	1783	37.25201	28	37.25201	87	37.25201	193
35.27248	1674	37.27248	68	37.27248	102	37.27248	244
35.29294	1478	37.29294	83	37.29294	134	37.29294	293
35.3134	1225	37.3134	92	37.3134	161	37.3134	362
35.33387	969	37.33387	77	37.33387	203	37.33387	511
35.35433	748	37.35433	70	37.35433	253	37.35433	655
35.37479	553	37.37479	129	37.37479	317	37.37479	879
35.39525	380	37.39525	190	37.39525	412	37.39525	1298
35.41571	247	37.41571	212	37.41571	557	37.41571	1639
35.43618	157	37.43618	237	37.43618	749	37.43618	1999
35.45664	107	37.45664	299	37.45664	977	37.45664	2334
35.4771	76	37.4771	457	37.4771	1215	37.4771	2421
35.49757	67	37.49757	762	37.49757	1436	37.49757	2289
35.51803	56	37.51803	1066	37.51803	1599	37.51803	2128
35.53849	41	37.53849	1384	37.53849	1702	37.53849	1968
35.55896	39	37.55896	1739	37.55896	1750	37.55896	2145
35.57942	38	37.57942	1936	37.57942	1736	37.57942	2322
35.59988	33	37.59988	1771	37.59988	1709	37.59988	2264
35.62034	36	37.62034	1621	37.62034	1679	37.62034	2437
35.6408	42	37.6408	1533	37.6408	1685	37.6408	2578
35.66127	48	37.66127	1470	37.66127	1705	37.66127	2402
35.68173	68	37.68173	1499	37.68173	1728	37.68173	2359
35.70219	95	37.70219	1569	37.70219	1738	37.70219	2032
35.72266	140	37.72266	1496	37.72266	1696	37.72266	1786
35.74312	215	37.74312	1616	37.74312	1616	37.74312	1578
35.76358	325	37.76358	1701	37.76358	1504	37.76358	1368
35.78405	444	37.78405	1707	37.78405	1356	37.78405	1106
35.80451	543	37.80451	1437	37.80451	1194	37.80451	894
35.82497	614	37.82497	1240	37.82497	1012	37.82497	639
35.84543	654	37.84543	1137	37.84543	839	37.84543	478
35.8659	651	37.8659	1053	37.8659	680	37.8659	374
35.88636	609	37.88636	883	37.88636	558	37.88636	272
35.90682	526	37.90682	645	37.90682	451	37.90682	231
35.92728	433	37.92728	483	37.92728	354	37.92728	216
35.94775	338	37.94775	328	37.94775	276	37.94775	104
35.96821	262	37.96821	182	37.96821	217	37.96821	136
35.98867	191	37.98867	160	37.98867	170	37.98867	145
36.00914	126	38.00914	115	38.00914	139	38.00914	85
36.0296	78	38.0296	161	38.0296	110	38.0296	73
36.05006	56	38.05006	134	38.05006	77	38.05006	21
36.07053	38	38.07053	74	38.07053	62	38.07053	52
36.09099	29	38.09099	61	38.09099	65	38.09099	69
36.11145	24	38.11145	56	38.11145	58	38.11145	33
36.13191	16	38.13191	29	38.13191	52	38.13191	89
36.15237	13	38.15237	0	38.15237	52	38.15237	87
36.17284	12	38.17284	0	38.17284	50	38.17284	84
36.1933	12	38.1933	34	38.1933	52	38.1933	77
36.21376	14	38.21376	10	38.21376	63	38.21376	64
36.23423	16	38.23423	26	38.23423	68	38.23423	139
36.25469	17	38.25469	46	38.25469	71	38.25469	132

36.27515	15	38.27515	0	38.27515	84	38.27515	168
36.29562	11	38.29562	3	38.29562	96	38.29562	280
36.31608	10	38.31608	46	38.31608	110	38.31608	344
36.33654	6	38.33654	20	38.33654	134	38.33654	451
36.357	9	38.357	80	38.357	164	38.357	673
36.37746	10	38.37746	89	38.37746	220	38.37746	825
36.39793	13	38.39793	62	38.39793	296	38.39793	1038
36.41839	13	38.41839	122	38.41839	391	38.41839	1156
36.43885	12	38.43885	201	38.43885	485	38.43885	1088
36.45932	6	38.45932	278	38.45932	574	38.45932	1082
36.47978	7	38.47978	422	38.47978	636	38.47978	962
36.50024	3	38.50024	621	38.50024	668	38.50024	891
36.52071	9	38.52071	686	38.52071	673	38.52071	737
36.54117	8	38.54117	758	38.54117	635	38.54117	668
36.56163	12	38.56163	776	38.56163	569	38.56163	568
36.58209	24	38.58209	630	38.58209	510	38.58209	417
36.60256	31	38.60256	536	38.60256	445	38.60256	281
36.62302	38	38.62302	581	38.62302	374	38.62302	239
36.64348	43	38.64348	487	38.64348	301	38.64348	134
36.66394	44	38.66394	405	38.66394	249	38.66394	139
36.68441	46	38.68441	277	38.68441	189	38.68441	78
36.70487	53	38.70487	272	38.70487	151	38.70487	108
36.72533	64	38.72533	124	38.72533	122	38.72533	63
36.7458	86	38.7458	129	38.7458	102	38.7458	67
36.76626	135	38.76626	43	38.76626	83	38.76626	94
36.78672	193	38.78672	86	38.78672	82	38.78672	98
36.80719	248	38.80719	87	38.80719	72	38.80719	76
36.82765	290	38.82765	23	38.82765	61	38.82765	109
36.84811	311	38.84811	57	38.84811	52	38.84811	133
36.86857	314	38.86857	57	38.86857	68	38.86857	196
36.88903	289	38.88903	3	38.88903	84	38.88903	273
36.9095	258	38.9095	74	38.9095	102	38.9095	271
36.92996	206	38.92996	53	38.92996	134	38.92996	409
36.95042	165	38.95042	29	38.95042	185	38.95042	473
36.97089	131	38.97089	87	38.97089	225	38.97089	616
36.99135	109	38.99135	72	38.99135	287	38.99135	707
37.01181	84	39.01181	138	39.01181	362	39.01181	699
37.03228	64	39.03228	174	39.03228	441	39.03228	698
37.05274	53	39.05274	300	39.05274	493	39.05274	646
37.0732	55	39.0732	399	39.0732	542	39.0732	620
37.09366	58	39.09366	547	39.09366	569	39.09366	662
37.11412	67	39.11412	605	39.11412	562	39.11412	634
37.13459	73	39.13459	604	39.13459	555	39.13459	633
37.15505	86	39.15505	519	39.15505	547	39.15505	637
37.17551	105	39.17551	491	39.17551	518	39.17551	601
37.19598	128	39.19598	427	39.19598	499	39.19598	584
37.21644	140	39.21644	428	39.21644	487	39.21644	583
37.2369	175	39.2369	435	39.2369	459	39.2369	545
37.25737	212	39.25737	445	39.25737	420	39.25737	510
37.27783	277	39.27783	507	39.27783	388	39.27783	473
37.29829	392	39.29829	522	39.29829	352	39.29829	318
37.31875	574	39.31875	437	39.31875	310	39.31875	330
37.33922	877	39.33922	378	39.33922	280	39.33922	258
37.35968	1264	39.35968	301	39.35968	250	39.35968	215
37.38014	1670	39.38014	275	39.38014	209	39.38014	156
37.4006	2014	39.4006	273	39.4006	171	39.4006	81
37.42107	2247	39.42107	251	39.42107	135	39.42107	80
37.44153	2365	39.44153	164	39.44153	102	39.44153	76

37.46199	2356	39.46199	100	39.46199	77	39.46199	59
37.48246	2255	39.48246	123	39.48246	65	39.48246	44
37.50292	2115	39.50292	15	39.50292	60	39.50292	23
37.52338	2015	39.52338	0	39.52338	52	39.52338	41
37.54385	2061	39.54385	45	39.54385	50	39.54385	54
37.56431	2207	39.56431	0	39.56431	46	39.56431	0
37.58477	2397	39.58477	23	39.58477	37	39.58477	27
37.60523	2537	39.60523	26	39.60523	28	39.60523	10
37.62569	2595	39.62569	7	39.62569	18	39.62569	12
37.64616	2562	39.64616	24	39.64616	9	39.64616	19
37.66662	2458	39.66662	0	39.66662	3	39.66662	0
37.68708	2246	39.68708	0	39.68708	5	39.68708	0
37.70755	1962	39.70755	0	39.70755	8	39.70755	21
37.72801	1634	39.72801	4	39.72801	5	39.72801	0
37.74847	1329	39.74847	0	39.74847	9	39.74847	4
37.76894	1059	39.76894	31	39.76894	9	39.76894	15
37.7894	824	39.7894	0	39.7894	8	39.7894	57
37.80986	586	39.80986	3	39.80986	6	39.80986	21
37.83032	397	39.83032	7	39.83032	2	39.83032	44
37.85078	261	39.85078	17	39.85078	3	39.85078	14
37.87125	187	39.87125	0	39.87125	2	39.87125	9
37.89171	135	39.89171	0	39.89171	1	39.89171	31
37.91217	115	39.91217	0	39.91217	4	39.91217	1
37.93264	88	39.93264	27	39.93264	4	39.93264	0
37.9531	72	39.9531	0	39.9531	6	39.9531	51
37.97356	66	39.97356	1	39.97356	7	39.97356	0
37.99403	62	39.99403	0	39.99403	9	39.99403	0
38.01449	55	40.01449	0	40.01449	10	40.01449	0
38.03495	61	40.03495	5	40.03495	8	40.03495	8
38.05541	58	40.05541	18	40.05541	9	40.05541	0
38.07588	52	40.07588	1	40.07588	7	40.07588	2
38.09634	44	40.09634	0	40.09634	2	40.09634	28
38.1168	44	40.1168	0	40.1168	5	40.1168	15
38.13726	41	40.13726	18	40.13726	5	40.13726	0
38.15773	39	40.15773	31	40.15773	5	40.15773	16
38.17819	46	40.17819	0	40.17819	6	40.17819	0
38.19865	61	40.19865	0	40.19865	7	40.19865	16
38.21912	88	40.21912	0	40.21912	7	40.21912	0
38.23958	115	40.23958	9	40.23958	4	40.23958	18
38.26004	147	40.26004	23	40.26004	5	40.26004	5
38.28051	206	40.28051	23	40.28051	4	40.28051	28
38.30097	307	40.30097	9	40.30097	5	40.30097	42
38.32143	451	40.32143	5	40.32143	7	40.32143	0
38.34189	627	40.34189	19	40.34189	10	40.34189	13
38.36235	800	40.36235	0	40.36235	13	40.36235	0
38.38282	945	40.38282	0	40.38282	15	40.38282	0
38.40328	1041	40.40328	27	40.40328	15	40.40328	11
38.42374	1085	40.42374	0	40.42374	16	40.42374	54
38.44421	1047	40.44421	0	40.44421	18	40.44421	65
38.46467	955	40.46467	14	40.46467	15	40.46467	40
38.48513	825	40.48513	28	40.48513	18	40.48513	41
38.5056	685	40.5056	26	40.5056	20	40.5056	49
38.52606	558	40.52606	9	40.52606	22	40.52606	88
38.54652	444	40.54652	48	40.54652	35	40.54652	57
38.56698	334	40.56698	9	40.56698	45	40.56698	46
38.58744	242	40.58744	23	40.58744	52	40.58744	79
38.60791	165	40.60791	42	40.60791	60	40.60791	128
38.62837	109	40.62837	98	40.62837	81	40.62837	168

38.64883	75	40.64883	75	40.64883	101	40.64883	252
38.6693	58	40.6693	66	40.6693	119	40.6693	220
38.68976	47	40.68976	37	40.68976	141	40.68976	247
38.71022	43	40.71022	103	40.71022	155	40.71022	172
38.73069	45	40.73069	155	40.73069	168	40.73069	176
38.75115	54	40.75115	194	40.75115	169	40.75115	164
38.77161	71	40.77161	170	40.77161	164	40.77161	151
38.79207	87	40.79207	171	40.79207	148	40.79207	150
38.81254	103	40.81254	204	40.81254	135	40.81254	103
38.833	130	40.833	114	40.833	126	40.833	66
38.85346	188	40.85346	123	40.85346	111	40.85346	68
38.87392	281	40.87392	74	40.87392	99	40.87392	93
38.89439	407	40.89439	123	40.89439	85	40.89439	11
38.91485	554	40.91485	97	40.91485	70	40.91485	16
38.93531	677	40.93531	104	40.93531	54	40.93531	35
38.95578	790	40.95578	73	40.95578	37	40.95578	25
38.97624	853	40.97624	23	40.97624	27	40.97624	0
38.9967	876	40.9967	3	40.9967	15	40.9967	0
39.01717	848	41.01717	19	41.01717	13	41.01717	39
39.03763	804	41.03763	6	41.03763	10	41.03763	0
39.05809	763	41.05809	0	41.05809	7	41.05809	14
39.07855	735	41.07855	0	41.07855	6	41.07855	0
39.09901	726	41.09901	0	41.09901	6	41.09901	0
39.11948	718	41.11948	26	41.11948	4	41.11948	25
39.13994	679	41.13994	0	41.13994	2	41.13994	0
39.1604	630	41.1604	1	41.1604	5	41.1604	10
39.18087	577	41.18087	5	41.18087	4	41.18087	0
39.20133	526	41.20133	0	41.20133	4	41.20133	0
39.22179	459	41.22179	0	41.22179	5	41.22179	0
39.24226	399	41.24226	32	41.24226	6	41.24226	0
39.26272	344	41.26272	4	41.26272	1	41.26272	0
39.28318	282	41.28318	0	41.28318	7	41.28318	6
39.30364	238	41.30364	0	41.30364	8	41.30364	22
39.3241	185	41.3241	2	41.3241	11	41.3241	4
39.34457	124	41.34457	7	41.34457	14	41.34457	32
39.36503	75	41.36503	0	41.36503	12	41.36503	12
39.38549	46	41.38549	0	41.38549	17	41.38549	31
39.40596	32	41.40596	3	41.40596	15	41.40596	61
39.42642	24	41.42642	22	41.42642	20	41.42642	93
39.44688	14	41.44688	27	41.44688	25	41.44688	49
39.46735	10	41.46735	13	41.46735	28	41.46735	62
39.48781	8	41.48781	74	41.48781	36	41.48781	56
39.50827	14	41.50827	12	41.50827	33	41.50827	45
39.52873	18	41.52873	26	41.52873	29	41.52873	14
39.5492	20	41.5492	34	41.5492	27	41.5492	0
39.56966	21	41.56966	62	41.56966	21	41.56966	26
39.59012	18	41.59012	40	41.59012	20	41.59012	46
39.61058	27	41.61058	11	41.61058	10	41.61058	27
39.63105	25	41.63105	38	41.63105	10	41.63105	0
39.65151	22	41.65151	34	41.65151	10	41.65151	21
39.67197	17	41.67197	11	41.67197	11	41.67197	53
39.69244	15	41.69244	28	41.69244	10	41.69244	0
39.7129	14	41.7129	0	41.7129	3	41.7129	53
39.73336	14	41.73336	36	41.73336	0	41.73336	39
39.75383	12	41.75383	17	41.75383	9	41.75383	57
39.77429	6	41.77429	16	41.77429	16	41.77429	91
39.79475	0	41.79475	32	41.79475	19	41.79475	127
39.81521	4	41.81521	0	41.81521	24	41.81521	108

39.83567	3	41.83567	37	41.83567	36	41.83567	150
39.85614	5	41.85614	43	41.85614	61	41.85614	207
39.8766	6	41.8766	25	41.8766	83	41.8766	351
39.89706	6	41.89706	15	41.89706	121	41.89706	441
39.91753	9	41.91753	12	41.91753	160	41.91753	490
39.93799	11	41.93799	124	41.93799	210	41.93799	500
39.95845	12	41.95845	152	41.95845	264	41.95845	493
39.97892	10	41.97892	198	41.97892	305	41.97892	345
39.99938	7	41.99938	316	41.99938	327	41.99938	272
40.01984	10	42.01984	345	42.01984	335	42.01984	317
40.0403	8	42.0403	407	42.0403	335	42.0403	340
40.06076	4	42.06076	367	42.06076	318	42.06076	280
40.08123	0	42.08123	371	42.08123	283	42.08123	185
40.10169	1	42.10169	314	42.10169	244	42.10169	88
40.12215	4	42.12215	252	42.12215	195	42.12215	30
40.14262	8	42.14262	211	42.14262	150	42.14262	87
40.16308	11	42.16308	220	42.16308	110	42.16308	0
40.18354	12	42.18354	149	42.18354	78	42.18354	58
40.20401	11	42.20401	146	42.20401	49	42.20401	0
40.22447	10	42.22447	40	42.22447	28	42.22447	38
40.24493	7	42.24493	28	42.24493	19	42.24493	42
40.26539	4	42.26539	5	42.26539	12	42.26539	28
40.28586	0	42.28586	4	42.28586	8	42.28586	7
40.30632	1	42.30632	0	42.30632	5	42.30632	0
40.32678	4	42.32678	0	42.32678	2	42.32678	4
40.34724	12	42.34724	32	42.34724	0	42.34724	39
40.36771	20	42.36771	2	42.36771	0	42.36771	2
40.38817	39	42.38817	7	42.38817	4	42.38817	0
40.40863	45	42.40863	17	42.40863	8	42.40863	80
40.4291	56	42.4291	13	42.4291	9	42.4291	43
40.44956	63	42.44956	0	42.44956	8	42.44956	83
40.47002	63	42.47002	19	42.47002	11	42.47002	71
40.49049	62	42.49049	0	42.49049	11	42.49049	18
40.51095	75	42.51095	13	42.51095	9	42.51095	51
40.53141	97	42.53141	28	42.53141	17	42.53141	53
40.55187	126	42.55187	25	42.55187	22	42.55187	52
40.57233	160	42.57233	20	42.57233	34	42.57233	61
40.5928	202	42.5928	14	42.5928	48	42.5928	138
40.61326	228	42.61326	61	42.61326	70	42.61326	102
40.63372	244	42.63372	50	42.63372	84	42.63372	130
40.65419	247	42.65419	44	42.65419	107	42.65419	148
40.67465	233	42.67465	59	42.67465	124	42.67465	238
40.69511	206	42.69511	63	42.69511	140	42.69511	336
40.71558	187	42.71558	119	42.71558	162	42.71558	418
40.73604	158	42.73604	103	42.73604	202	42.73604	583
40.7565	126	42.7565	104	42.7565	262	42.7565	803
40.77696	99	42.77696	151	42.77696	350	42.77696	1015
40.79742	74	42.79742	179	42.79742	452	42.79742	1268
40.81789	55	42.81789	321	42.81789	587	42.81789	1489
40.83835	34	42.83835	350	42.83835	720	42.83835	1393
40.85881	25	42.85881	524	42.85881	852	42.85881	1301
40.87928	16	42.87928	689	42.87928	938	42.87928	1209
40.89974	9	42.89974	1006	42.89974	989	42.89974	1099
40.9202	14	42.9202	1155	42.9202	998	42.9202	1059
40.94067	8	42.94067	1280	42.94067	976	42.94067	974
40.96113	3	42.96113	1119	42.96113	922	42.96113	824
40.98159	5	42.98159	960	42.98159	841	42.98159	694
41.00205	0	43.00205	812	43.00205	748	43.00205	596

41.02252	1	43.02252	840	43.02252	664	43.02252	452
41.04298	6	43.04298	773	43.04298	574	43.04298	347
41.06344	13	43.06344	681	43.06344	499	43.06344	356
41.0839	16	43.0839	554	43.0839	421	43.0839	369
41.10437	17	43.10437	373	43.10437	365	43.10437	306
41.12483	13	43.12483	306	43.12483	320	43.12483	385
41.14529	11	43.14529	234	43.14529	307	43.14529	397
41.16576	11	43.16576	190	43.16576	313	43.16576	397
41.18622	8	43.18622	219	43.18622	335	43.18622	586
41.20668	7	43.20668	206	43.20668	372	43.20668	694
41.22715	9	43.22715	243	43.22715	440	43.22715	892
41.24761	16	43.24761	238	43.24761	527	43.24761	1210
41.26807	21	43.26807	290	43.26807	664	43.26807	1671
41.28853	25	43.28853	309	43.28853	857	43.28853	2277
41.30899	35	43.30899	476	43.30899	1116	43.30899	2501
41.32946	46	43.32946	678	43.32946	1410	43.32946	2920
41.34992	56	43.34992	854	43.34992	1741	43.34992	3109
41.37038	63	43.37038	1318	43.37038	2045	43.37038	2817
41.39085	62	43.39085	1826	43.39085	2248	43.39085	2567
41.41131	61	43.41131	2578	43.41131	2363	43.41131	2487
41.43177	51	43.43177	3023	43.43177	2409	43.43177	2421
41.45224	42	43.45224	3100	43.45224	2369	43.45224	2351
41.4727	33	43.4727	2800	43.4727	2276	43.4727	2285
41.49316	24	43.49316	2333	43.49316	2172	43.49316	2213
41.51362	29	43.51362	2168	43.51362	2067	43.51362	2008
41.53408	33	43.53408	2253	43.53408	1955	43.53408	1727
41.55455	30	43.55455	2399	43.55455	1840	43.55455	1555
41.57501	31	43.57501	2278	43.57501	1692	43.57501	1278
41.59547	25	43.59547	2179	43.59547	1498	43.59547	1158
41.61594	21	43.61594	1966	43.61594	1304	43.61594	980
41.6364	14	43.6364	1751	43.6364	1125	43.6364	841
41.65686	14	43.65686	1452	43.65686	959	43.65686	827
41.67733	19	43.67733	1200	43.67733	819	43.67733	626
41.69779	19	43.69779	1011	43.69779	703	43.69779	470
41.71825	37	43.71825	957	43.71825	599	43.71825	295
41.73871	54	43.73871	941	43.73871	505	43.73871	264
41.75918	75	43.75918	725	43.75918	421	43.75918	201
41.77964	118	43.77964	611	43.77964	347	43.77964	159
41.8001	182	43.8001	379	43.8001	276	43.8001	143
41.82056	270	43.82056	310	43.82056	209	43.82056	105
41.84103	356	43.84103	229	43.84103	162	43.84103	98
41.86149	446	43.86149	161	43.86149	132	43.86149	94
41.88195	506	43.88195	96	43.88195	121	43.88195	89
41.90242	534	43.90242	100	43.90242	100	43.90242	76
41.92288	534	43.92288	140	43.92288	88	43.92288	82
41.94334	504	43.94334	51	43.94334	82	43.94334	51
41.96381	448	43.96381	72	43.96381	76	43.96381	70
41.98427	388	43.98427	47	43.98427	71	43.98427	61
42.00473	333	44.00473	28	44.00473	57	44.00473	87
42.02519	283	44.02519	33	44.02519	59	44.02519	54
42.04565	221	44.04565	55	44.04565	62	44.04565	81
42.06612	177	44.06612	28	44.06612	67	44.06612	102
42.08658	129	44.08658	26	44.08658	76	44.08658	118
42.10704	96	44.10704	56	44.10704	82	44.10704	214
42.12751	62	44.12751	55	44.12751	108	44.12751	279
42.14797	42	44.14797	75	44.14797	144	44.14797	292
42.16843	31	44.16843	59	44.16843	185	44.16843	360
42.1889	25	44.1889	120	44.1889	230	44.1889	380

42.20936	24	44.20936	127	44.20936	277	44.20936	326
42.22982	23	44.22982	185	44.22982	330	44.22982	353
42.25028	14	44.25028	252	44.25028	354	44.25028	286
42.27074	15	44.27074	398	44.27074	358	44.27074	249
42.29121	13	44.29121	397	44.29121	349	44.29121	260
42.31167	9	44.31167	400	44.31167	334	44.31167	241
42.33213	0	44.33213	402	44.33213	317	44.33213	187
42.3526	5	44.3526	275	44.3526	295	44.3526	199
42.37306	17	44.37306	271	44.37306	267	44.37306	133
42.39352	23	44.39352	239	44.39352	237	44.39352	136
42.41399	38	44.41399	286	44.41399	201	44.41399	71
42.43445	41	44.43445	264	44.43445	158	44.43445	85
42.45491	53	44.45491	177	44.45491	110	44.45491	60
42.47537	56	44.47537	95	44.47537	76	44.47537	82
42.49584	56	44.49584	55	44.49584	57	44.49584	104
42.5163	55	44.5163	72	44.5163	55	44.5163	102
42.53676	56	44.53676	41	44.53676	63	44.53676	121
42.55722	73	44.55722	46	44.55722	71	44.55722	157
42.57769	78	44.57769	68	44.57769	88	44.57769	163
42.59815	96	44.59815	32	44.59815	106	44.59815	267
42.61861	134	44.61861	47	44.61861	136	44.61861	327
42.63908	185	44.63908	34	44.63908	162	44.63908	413
42.65954	266	44.65954	72	44.65954	206	44.65954	548
42.68	391	44.68	106	44.68	265	44.68	629
42.70047	596	44.70047	161	44.70047	339	44.70047	735
42.72093	843	44.72093	190	44.72093	418	44.72093	698
42.74139	1083	44.74139	346	44.74139	491	44.74139	676
42.76185	1312	44.76185	433	44.76185	539	44.76185	606
42.78231	1459	44.78231	609	44.78231	568	44.78231	547
42.80278	1522	44.80278	668	44.80278	569	44.80278	499
42.82324	1519	44.82324	715	44.82324	554	44.82324	502
42.8437	1433	44.8437	614	44.8437	511	44.8437	460
42.86417	1291	44.86417	494	44.86417	474	44.86417	391
42.88463	1130	44.88463	536	44.88463	420	44.88463	296
42.90509	989	44.90509	471	44.90509	373	44.90509	282
42.92556	830	44.92556	500	44.92556	332	44.92556	154
42.94602	692	44.94602	362	44.94602	290	44.94602	156
42.96648	578	44.96648	345	44.96648	241	44.96648	67
42.98694	454	44.98694	292	44.98694	188	44.98694	93
43.0074	354	45.0074	222	45.0074	135	45.0074	87
43.02787	319	45.02787	122	45.02787	101	45.02787	69
43.04833	294	45.04833	155	45.04833	77	45.04833	34
43.06879	291	45.06879	73	45.06879	64	45.06879	47
43.08926	307	45.08926	62	45.08926	42	45.08926	4
43.10972	343	45.10972	31	45.10972	29	45.10972	9
43.13018	399	45.13018	29	45.13018	30	45.13018	0
43.15065	521	45.15065	41	45.15065	22	45.15065	0
43.17111	697	45.17111	24	45.17111	22	45.17111	8
43.19157	987	45.19157	0	45.19157	15	45.19157	0
43.21203	1435	45.21203	0	45.21203	11	45.21203	0
43.2325	1984	45.2325	7	45.2325	10	45.2325	0
43.25296	2532	45.25296	0	45.25296	14	45.25296	14
43.27342	2963	45.27342	4	45.27342	15	45.27342	0
43.29388	3237	45.29388	7	45.29388	12	45.29388	0
43.31435	3326	45.31435	0	45.31435	8	45.31435	0
43.33481	3260	45.33481	0	45.33481	9	45.33481	31
43.35527	3117	45.35527	15	45.35527	6	45.35527	6
43.37574	2912	45.37574	3	45.37574	4	45.37574	23

43.3962	2744	45.3962	0	45.3962	7	45.3962	60
43.41666	2644	45.41666	6	45.41666	11	45.41666	59
43.43713	2566	45.43713	1	45.43713	14	45.43713	61
43.45759	2464	45.45759	29	45.45759	24	45.45759	128
43.47805	2284	45.47805	0	45.47805	32	45.47805	133
43.49851	2024	45.49851	27	45.49851	44	45.49851	212
43.51897	1758	45.51897	27	45.51897	62	45.51897	185
43.53944	1522	45.53944	35	45.53944	76	45.53944	169
43.5599	1320	45.5599	14	45.5599	95	45.5599	207
43.58036	1108	45.58036	81	45.58036	101	45.58036	187
43.60083	929	45.60083	58	45.60083	124	45.60083	112
43.62129	759	45.62129	115	45.62129	137	45.62129	174
43.64175	612	45.64175	164	45.64175	134	45.64175	115
43.66222	478	45.66222	143	45.66222	123	45.66222	174
43.68268	359	45.68268	175	45.68268	111	45.68268	132
43.70314	236	45.70314	136	45.70314	95	45.70314	57
43.7236	175	45.7236	96	45.7236	87	45.7236	122
43.74406	156	45.74406	85	45.74406	74	45.74406	28
43.76453	131	45.76453	132	45.76453	63	45.76453	31
43.78499	105	45.78499	61	45.78499	45	45.78499	28
43.80545	90	45.80545	103	45.80545	47	45.80545	0
43.82592	69	45.82592	49	45.82592	39	45.82592	2
43.84638	67	45.84638	46	45.84638	27	45.84638	21
43.86684	60	45.86684	57	45.86684	18	45.86684	7
43.88731	48	45.88731	64	45.88731	10	45.88731	0
43.90777	34	45.90777	0	45.90777	5	45.90777	0
43.92823	36	45.92823	1	45.92823	9	45.92823	0
43.94869	39	45.94869	0	45.94869	5	45.94869	19
43.96916	45	45.96916	0	45.96916	4	45.96916	0
43.98962	45	45.98962	11	45.98962	3	45.98962	12
44.01008	60	46.01008	46	46.01008	6	46.01008	55
44.03054	88	46.03054	35	46.03054	18	46.03054	38
44.05101	141	46.05101	8	46.05101	24	46.05101	0
44.07147	208	46.07147	26	46.07147	28	46.07147	26
44.09193	298	46.09193	46	46.09193	24	46.09193	43
44.1124	401	46.1124	40	46.1124	29	46.1124	12
44.13286	499	46.13286	25	46.13286	29	46.13286	66
44.15332	561	46.15332	38	46.15332	19	46.15332	65
44.17379	588	46.17379	14	46.17379	20	46.17379	14
44.19425	575	46.19425	9	46.19425	18	46.19425	55
44.21471	543	46.21471	16	46.21471	20	46.21471	24
44.23517	487	46.23517	49	46.23517	30	46.23517	0
44.25563	429	46.25563	38	46.25563	31	46.25563	0
44.2761	364	46.2761	54	46.2761	36	46.2761	0
44.29656	305	46.29656	33	46.29656	33	46.29656	0
44.31702	253	46.31702	18	46.31702	37	46.31702	6
44.33749	205	46.33749	30	46.33749	31	46.33749	7
44.35795	153	46.35795	26	46.35795	23	46.35795	23
44.37841	105	46.37841	3	46.37841	25	46.37841	24
44.39888	69	46.39888	13	46.39888	23	46.39888	0
44.41934	47	46.41934	38	46.41934	21	46.41934	0
44.4398	32	46.4398	0	46.4398	14	46.4398	0
44.46026	37	46.46026	6	46.46026	12	46.46026	0
44.48072	37	46.48072	0	46.48072	12	46.48072	24
44.50119	55	46.50119	0	46.50119	10	46.50119	24
44.52165	88	46.52165	0	46.52165	14	46.52165	26
44.54211	142	46.54211	15	46.54211	12	46.54211	0
44.56258	230	46.56258	40	46.56258	16	46.56258	0

44.58304	353	46.58304	29	46.58304	15	46.58304	5
44.6035	517	46.6035	16	46.6035	14	46.6035	37
44.62397	678	46.62397	23	46.62397	12	46.62397	3
44.64443	804	46.64443	38	46.64443	9	46.64443	17
44.66489	886	46.66489	0	46.66489	11	46.66489	0
44.68535	914	46.68535	0	46.68535	15	46.68535	15
44.70582	898	46.70582	0	46.70582	18	46.70582	24
44.72628	844	46.72628	0	46.72628	17	46.72628	2
44.74674	767	46.74674	41	46.74674	20	46.74674	11
44.7672	681	46.7672	54	46.7672	20	46.7672	59
44.78767	608	46.78767	25	46.78767	23	46.78767	73
44.80813	552	46.80813	17	46.80813	24	46.80813	114
44.82859	489	46.82859	39	46.82859	37	46.82859	149
44.84906	407	46.84906	66	46.84906	55	46.84906	108
44.86952	325	46.86952	18	46.86952	81	46.86952	167
44.88998	246	46.88998	35	46.88998	99	46.88998	170
44.91045	182	46.91045	77	46.91045	108	46.91045	105
44.93091	143	46.93091	110	46.93091	108	46.93091	144
44.95137	111	46.95137	106	46.95137	116	46.95137	106
44.97183	81	46.97183	140	46.97183	117	46.97183	126
44.99229	62	46.99229	144	46.99229	119	46.99229	121
45.01276	44	47.01276	107	47.01276	109	47.01276	102
45.03322	30	47.03322	109	47.03322	113	47.03322	36
45.05368	17	47.05368	68	47.05368	113	47.05368	78
45.07415	11	47.07415	100	47.07415	115	47.07415	35
45.09461	5	47.09461	86	47.09461	108	47.09461	52
45.11507	6	47.11507	73	47.11507	102	47.11507	55
45.13554	8	47.13554	65	47.13554	86	47.13554	86
45.156	8	47.156	66	47.156	77	47.156	106
45.17646	9	47.17646	64	47.17646	70	47.17646	81
45.19692	10	47.19692	32	47.19692	82	47.19692	126
45.21738	9	47.21738	58	47.21738	90	47.21738	153
45.23785	5	47.23785	68	47.23785	105	47.23785	229
45.25831	8	47.25831	77	47.25831	139	47.25831	353
45.27877	8	47.27877	62	47.27877	172	47.27877	350
45.29924	5	47.29924	23	47.29924	233	47.29924	589
45.3197	3	47.3197	169	47.3197	296	47.3197	755
45.34016	0	47.34016	161	47.34016	374	47.34016	843
45.36063	1	47.36063	266	47.36063	457	47.36063	826
45.38109	11	47.38109	279	47.38109	535	47.38109	831
45.40155	33	47.40155	495	47.40155	603	47.40155	765
45.42201	67	47.42201	626	47.42201	632	47.42201	702
45.44248	107	47.44248	773	47.44248	643	47.44248	641
45.46294	149	47.46294	843	47.46294	631	47.46294	592
45.4834	181	47.4834	796	47.4834	581	47.4834	573
45.50386	196	47.50386	612	47.50386	537	47.50386	444
45.52433	199	47.52433	508	47.52433	477	47.52433	409
45.54479	187	47.54479	492	47.54479	426	47.54479	332
45.56525	171	47.56525	464	47.56525	371	47.56525	178
45.58572	156	47.58572	507	47.58572	312	47.58572	127
45.60618	138	47.60618	452	47.60618	256	47.60618	79
45.62664	113	47.62664	318	47.62664	199	47.62664	46
45.64711	97	47.64711	251	47.64711	155	47.64711	33
45.66757	84	47.66757	152	47.66757	115	47.66757	62
45.68803	68	47.68803	98	47.68803	82	47.68803	38
45.70849	47	47.70849	41	47.70849	61	47.70849	0
45.72895	30	47.72895	72	47.72895	43	47.72895	17
45.74942	18	47.74942	62	47.74942	27	47.74942	24

45.76988	15	47.76988	41	47.76988	22	47.76988	0
45.79034	10	47.79034	48	47.79034	17	47.79034	28
45.81081	5	47.81081	50	47.81081	17	47.81081	0
45.83127	0	47.83127	27	47.83127	18	47.83127	0
45.85173	0	47.85173	7	47.85173	16	47.85173	18
45.8722	7	47.8722	0	47.8722	23	47.8722	0
45.89266	10	47.89266	0	47.89266	24	47.89266	4
45.91312	14	47.91312	9	47.91312	23	47.91312	0
45.93358	19	47.93358	3	47.93358	17	47.93358	0
45.95404	20	47.95404	0	47.95404	11	47.95404	19
45.97451	19	47.97451	0	47.97451	9	47.97451	4
45.99497	17	47.99497	0	47.99497	3	47.99497	25
46.01543	17	48.01543	36	48.01543	0	48.01543	27
46.0359	15	48.0359	0	48.0359	3	48.0359	22
46.05636	11	48.05636	0	48.05636	4	48.05636	0
46.07682	11	48.07682	17	48.07682	9	48.07682	14
46.09729	7	48.09729	0	48.09729	12	48.09729	49
46.11775	9	48.11775	10	48.11775	8	48.11775	51
46.13821	9	48.13821	0	48.13821	18	48.13821	103
46.15867	7	48.15867	10	48.15867	31	48.15867	40
46.17914	4	48.17914	43	48.17914	49	48.17914	72
46.1996	6	48.1996	54	48.1996	58	48.1996	58
46.22006	5	48.22006	60	48.22006	67	48.22006	93
46.24052	6	48.24052	51	48.24052	78	48.24052	117
46.26099	7	48.26099	81	48.26099	73	48.26099	56
46.28145	6	48.28145	83	48.28145	63	48.28145	53
46.30191	3	48.30191	70	48.30191	52	48.30191	65
46.32238	7	48.32238	71	48.32238	35	48.32238	23
46.34284	8	48.34284	38	48.34284	32	48.34284	89
46.3633	7	48.3633	103	48.3633	33	48.3633	62
46.38377	5	48.38377	68	48.38377	33	48.38377	64
46.40423	6	48.40423	38	48.40423	36	48.40423	81
46.42469	2	48.42469	47	48.42469	51	48.42469	115
46.44515	4	48.44515	64	48.44515	62	48.44515	133
46.46561	4	48.46561	59	48.46561	88	48.46561	169
46.48608	6	48.48608	77	48.48608	123	48.48608	239
46.50654	7	48.50654	46	48.50654	168	48.50654	285
46.527	10	48.527	115	48.527	213	48.527	480
46.54747	10	48.54747	99	48.54747	271	48.54747	566
46.56793	7	48.56793	186	48.56793	351	48.56793	736
46.58839	3	48.58839	171	48.58839	448	48.58839	889
46.60886	7	48.60886	237	48.60886	573	48.60886	1005
46.62932	3	48.62932	334	48.62932	715	48.62932	1140
46.64978	9	48.64978	542	48.64978	853	48.64978	1172
46.67024	16	48.67024	730	48.67024	996	48.67024	1256
46.6907	25	48.6907	870	48.6907	1116	48.6907	1373
46.71117	40	48.71117	1027	48.71117	1205	48.71117	1532
46.73163	56	48.73163	1059	48.73163	1285	48.73163	1861
46.75209	74	48.75209	1080	48.75209	1386	48.75209	2168
46.77256	101	48.77256	1073	48.77256	1503	48.77256	2333
46.79302	120	48.79302	1096	48.79302	1630	48.79302	2470
46.81348	140	48.81348	1351	48.81348	1758	48.81348	2419
46.83395	152	48.83395	1605	48.83395	1847	48.83395	2071
46.85441	155	48.85441	1956	48.85441	1866	48.85441	1912
46.87487	146	48.87487	2374	48.87487	1820	48.87487	1688
46.89533	130	48.89533	2355	48.89533	1732	48.89533	1515
46.9158	110	48.9158	2195	48.9158	1583	48.9158	1419
46.93626	91	48.93626	1800	48.93626	1421	48.93626	1218

46.95672	78	48.95672	1384	48.95672	1262	48.95672	1077
46.97718	73	48.97718	1296	48.97718	1099	48.97718	789
46.99765	67	48.99765	1260	48.99765	944	48.99765	574
47.01811	67	49.01811	1221	49.01811	807	49.01811	385
47.03857	66	49.03857	1040	49.03857	655	49.03857	268
47.05904	66	49.05904	854	49.05904	503	49.05904	200
47.0795	70	49.0795	658	49.0795	377	49.0795	91
47.09996	76	49.09996	428	49.09996	288	49.09996	78
47.12043	90	49.12043	307	49.12043	214	49.12043	45
47.14089	105	49.14089	219	49.14089	159	49.14089	35
47.16135	131	49.16135	127	49.16135	113	49.16135	0
47.18181	170	49.18181	80	49.18181	73	49.18181	0
47.20227	252	49.20227	34	49.20227	45	49.20227	0
47.22274	365	49.22274	68	49.22274	31	49.22274	0
47.2432	508	49.2432	33	49.2432	6	49.2432	30
47.26366	669	49.26366	35	49.26366	0	49.26366	2
47.28413	817	49.28413	37	49.28413	0	49.28413	29
47.30459	915	49.30459	0	49.30459	14	49.30459	119
47.32505	959	49.32505	0	49.32505	38	49.32505	171
47.34552	931	49.34552	0	49.34552	55	49.34552	201
47.36598	869	49.36598	67	49.36598	77	49.36598	358
47.38644	783	49.38644	66	49.38644	110	49.38644	414
47.4069	690	49.4069	105	49.4069	155	49.4069	528
47.42736	615	49.42736	131	49.42736	211	49.42736	569
47.44783	541	49.44783	176	49.44783	264	49.44783	515
47.46829	460	49.46829	362	49.46829	316	49.46829	434
47.48875	393	49.48875	470	49.48875	352	49.48875	374
47.50922	301	49.50922	566	49.50922	366	49.50922	362
47.52968	214	49.52968	588	49.52968	352	49.52968	316
47.55014	137	49.55014	548	49.55014	307	49.55014	335
47.57061	90	49.57061	432	49.57061	262	49.57061	197
47.59107	60	49.59107	300	49.59107	217	49.59107	196
47.61153	34	49.61153	261	49.61153	175	49.61153	58
47.63199	31	49.63199	281	49.63199	145	49.63199	30
47.65246	21	49.65246	325	49.65246	117	49.65246	26
47.67292	13	49.67292	235	49.67292	90	49.67292	5
47.69338	18	49.69338	161	49.69338	59	49.69338	0
47.71384	8	49.71384	39	49.71384	35	49.71384	0
47.73431	6	49.73431	63	49.73431	16	49.73431	8
47.75477	7	49.75477	1	49.75477	0	49.75477	0
47.77523	11	49.77523	0	49.77523	0	49.77523	2
47.7957	10	49.7957	0	49.7957	0	49.7957	34
47.81616	6	49.81616	0	49.81616	0	49.81616	150
47.83662	5	49.83662	0	49.83662	0	49.83662	281
47.85709	9	49.85709	0	49.85709	20	49.85709	385
47.87755	9	49.87755	0	49.87755	49	49.87755	427
47.89801	8	49.89801	0	49.89801	79	49.89801	376
47.91847	5	49.91847	0	49.91847	96	49.91847	382
47.93893	4	49.93893	68	49.93893	99	49.93893	231
47.9594	1	49.9594	29	49.9594	93	49.9594	50
47.97986	3	49.97986	5	49.97986	93	49.97986	39
48.00032	5	50.00032	0	50.00032	93	50.00032	0
48.02079	14						
48.04125	31						
48.06171	48						
48.08218	69						
48.10264	82						
48.1231	87						

48.14356	83
48.16402	77
48.18449	74
48.20495	70
48.22541	73
48.24588	77
48.26634	71
48.2868	69
48.30727	64
48.32773	68
48.34819	75
48.36865	88
48.38912	102
48.40958	141
48.43004	195
48.4505	284
48.47097	390
48.49143	560
48.51189	755
48.53236	983
48.55282	1185
48.57328	1351
48.59375	1469
48.61421	1575
48.63467	1685
48.65513	1876
48.67559	2145
48.69606	2489
48.71652	2791
48.73698	2989
48.75745	3029
48.77791	2935
48.79837	2697
48.81884	2390
48.8393	2078
48.85976	1815
48.88022	1600
48.90068	1402
48.92115	1177
48.94161	914
48.96207	668
48.98254	455
49.003	269
49.02346	146
49.04393	78
49.06439	42
49.08485	22
49.10531	10
49.12578	0
49.14624	0
49.1667	0
49.18716	0
49.20763	0
49.22809	11
49.24855	38
49.26902	95
49.28948	176
49.30994	303

49.3304	438
49.35087	560
49.37133	641
49.39179	667
49.41225	649
49.43272	596
49.45318	517
49.47364	439
49.49411	373
49.51457	327
49.53503	280
49.5555	228
49.57596	165
49.59642	102
49.61688	54
49.63734	14
49.65781	0
49.67827	0
49.69873	0
49.7192	40
49.73966	107
49.76012	219
49.78059	368
49.80105	521
49.82151	624
49.84197	673
49.86244	667
49.8829	596
49.90336	502
49.92382	346
49.94429	261
49.96475	184
49.98521	184
50.00568	184

3	Cycloohexane	4	Methanol	5	Water	6	Tetrahydrc	7	
4	0	4	0	4	0	4.2	0	4	
4.020462	0	4.020462	0	4.020462	0	4.220462	0	4.020462	
4.040925	0	4.040925	0	4.040925	42	4.240925	0	4.040925	
4.061388	0	4.061388	0	4.061388	0	4.261388	0	4.061388	
4.081851	0	4.081851	0	4.081851	0	4.281851	16	4.081851	
4.102313	0	4.102313	0	4.102313	0	4.302313	76	4.102313	
4.122776	17	4.122776	0	4.122776	34	4.322776	21	4.122776	
4.143239	73	4.143239	0	4.143239	33	4.343239	6	4.143239	
4.163702	0	4.163702	37	4.163702	0	4.363702	0	4.163702	
4.184165	0	4.184165	26	4.184165	0	4.384165	0	4.184165	
4.204627	90	4.204627	47	4.204627	18	4.404627	1	4.204627	
4.22509	80	4.22509	5	4.22509	0	4.42509	0	4.22509	
4.245553	128	4.245553	98	4.245553	63	4.445553	20	4.245553	
4.266016	0	4.266016	22	4.266016	20	4.466016	35	4.266016	
4.286478	0	4.286478	35	4.286478	0	4.486478	100	4.286478	
4.306941	0	4.306941	43	4.306941	0	4.506941	72	4.306941	
4.327404	54	4.327404	0	4.327404	0	4.527404	0	4.327404	
4.347867	53	4.347867	0	4.347867	0	4.547867	0	4.347867	
4.36833	4	4.36833	0	4.36833	0	4.56833	98	4.36833	
4.388792	22	4.388792	81	4.388792	75	4.588792	0	4.388792	
4.409255	103	4.409255	97	4.409255	86	4.609255	0	4.409255	
4.429718	0	4.429718	61	4.429718	72	4.629718	0	4.429718	
4.450181	0	4.450181	0	4.450181	27	4.650181	17	4.450181	
4.470643	46	4.470643	0	4.470643	37	4.670643	0	4.470643	
4.491106	59	4.491106	0	4.491106	15	4.691106	6	4.491106	
4.511569	22	4.511569	66	4.511569	0	4.711569	0	4.511569	
4.532032	4	4.532032	40	4.532032	0	4.732032	0	4.532032	
4.552495	72	4.552495	45	4.552495	7	4.752495	0	4.552495	
4.572957	55	4.572957	36	4.572957	72	4.772957	52	4.572957	
4.59342	46	4.59342	100	4.59342	27	4.79342	54	4.59342	
4.613883	18	4.613883	21	4.613883	17	4.813883	0	4.613883	
4.634346	11	4.634346	0	4.634346	30	4.834346	0	4.634346	
4.654808	35	4.654808	0	4.654808	40	4.854808	0	4.654808	
4.675271	76	4.675271	0	4.675271	13	4.875271	72	4.675271	
4.695734	5	4.695734	4	4.695734	44	4.895734	0	4.695734	
4.716197	49	4.716197	0	4.716197	23	4.916197	48	4.716197	
4.73666	60	4.73666	81	4.73666	59	4.93666	0	4.73666	
4.757122	0	4.757122	19	4.757122	0	4.957122	43	4.757122	
4.777585	13	4.777585	2	4.777585	0	4.977585	46	4.777585	
4.798048	7	4.798048	0	4.798048	62	4.998048	0	4.798048	
4.818511	53	4.818511	20	4.818511	0	5.018511	64	4.818511	
4.838973	43	4.838973	80	4.838973	74	5.038973	0	4.838973	
4.859436	33	4.859436	0	4.859436	0	5.059436	71	4.859436	
4.879899	38	4.879899	0	4.879899	0	5.079899	6	4.879899	
4.900362	18	4.900362	34	4.900362	27	5.100362	74	4.900362	
4.920825	90	4.920825	0	4.920825	11	5.120825	0	4.920825	
4.941287	0	4.941287	0	4.941287	23	5.141287	8	4.941287	
4.96175	0	4.96175	0	4.96175	0	5.16175	0	4.96175	
4.982213	0	4.982213	0	4.982213	103	5.182213	0	4.982213	
5.002676	15	5.002676	37	5.002676	0	5.202676	51	5.002676	
5.023139	19	5.023139	26	5.023139	0	5.223139	57	5.023139	

5.043602	69	5.043602	0	5.043602	35	5.243602	3	5.043602
5.064064	18	5.064064	82	5.064064	78	5.264064	0	5.064064
5.084527	25	5.084527	0	5.084527	0	5.284527	0	5.084527
5.10499	107	5.10499	0	5.10499	0	5.30499	0	5.10499
5.125453	17	5.125453	46	5.125453	63	5.325453	0	5.125453
5.145916	12	5.145916	60	5.145916	0	5.345916	18	5.145916
5.166378	0	5.166378	20	5.166378	65	5.366378	0	5.166378
5.186841	0	5.186841	0	5.186841	86	5.386841	0	5.186841
5.207304	0	5.207304	42	5.207304	63	5.407304	19	5.207304
5.227767	0	5.227767	68	5.227767	36	5.427767	18	5.227767
5.248229	0	5.248229	76	5.248229	68	5.448229	57	5.248229
5.268692	32	5.268692	0	5.268692	17	5.468692	41	5.268692
5.289155	41	5.289155	52	5.289155	0	5.489155	10	5.289155
5.309618	75	5.309618	57	5.309618	8	5.509618	13	5.309618
5.330081	0	5.330081	42	5.330081	8	5.530081	86	5.330081
5.350543	0	5.350543	0	5.350543	96	5.550543	59	5.350543
5.371006	56	5.371006	0	5.371006	76	5.571006	0	5.371006
5.391469	53	5.391469	19	5.391469	0	5.591469	23	5.391469
5.411932	0	5.411932	0	5.411932	0	5.611932	36	5.411932
5.432394	15	5.432394	28	5.432394	13	5.632394	12	5.432394
5.452857	83	5.452857	42	5.452857	10	5.652857	31	5.452857
5.47332	0	5.47332	0	5.47332	54	5.67332	0	5.47332
5.493783	11	5.493783	0	5.493783	16	5.693783	0	5.493783
5.514246	86	5.514246	74	5.514246	65	5.714246	0	5.514246
5.534708	0	5.534708	4	5.534708	22	5.734708	0	5.534708
5.555171	13	5.555171	24	5.555171	51	5.755171	65	5.555171
5.575634	0	5.575634	44	5.575634	24	5.775634	0	5.575634
5.596097	28	5.596097	0	5.596097	118	5.796097	29	5.596097
5.616559	126	5.616559	0	5.616559	155	5.816559	0	5.616559
5.637022	69	5.637022	7	5.637022	87	5.837022	0	5.637022
5.657485	65	5.657485	52	5.657485	52	5.857485	90	5.657485
5.677948	116	5.677948	0	5.677948	121	5.877948	0	5.677948
5.698411	112	5.698411	31	5.698411	111	5.898411	24	5.698411
5.718873	67	5.718873	0	5.718873	106	5.918873	4	5.718873
5.739336	25	5.739336	51	5.739336	178	5.939336	2	5.739336
5.759799	51	5.759799	76	5.759799	88	5.959799	78	5.759799
5.780262	116	5.780262	130	5.780262	105	5.980262	0	5.780262
5.800724	144	5.800724	44	5.800724	118	6.000724	49	5.800724
5.821187	160	5.821187	147	5.821187	231	6.021187	0	5.821187
5.84165	213	5.84165	105	5.84165	236	6.04165	96	5.84165
5.862113	173	5.862113	126	5.862113	229	6.062113	144	5.862113
5.882576	341	5.882576	176	5.882576	315	6.082576	109	5.882576
5.903038	280	5.903038	99	5.903038	253	6.103038	115	5.903038
5.923501	369	5.923501	176	5.923501	449	6.123501	119	5.923501
5.943964	386	5.943964	144	5.943964	383	6.143964	221	5.943964
5.964427	357	5.964427	64	5.964427	348	6.164427	213	5.964427
5.984889	370	5.984889	186	5.984889	473	6.184889	223	5.984889
6.005352	499	6.005352	226	6.005352	509	6.205352	222	6.005352
6.025815	531	6.025815	200	6.025815	513	6.225815	259	6.025815
6.046278	599	6.046278	167	6.046278	629	6.246278	321	6.046278
6.066741	595	6.066741	439	6.066741	829	6.266741	311	6.066741
6.087203	663	6.087203	401	6.087203	731	6.287203	421	6.087203
6.107666	805	6.107666	388	6.107666	849	6.307666	549	6.107666
6.128129	942	6.128129	418	6.128129	1140	6.328129	489	6.128129
6.148592	1074	6.148592	523	6.148592	1203	6.348592	643	6.148592
6.169055	1283	6.169055	622	6.169055	1431	6.369055	816	6.169055
6.189517	1436	6.189517	727	6.189517	1692	6.389517	936	6.189517
6.20998	1716	6.20998	860	6.20998	2101	6.40998	1100	6.20998

6.230443	2039	6.230443	983	6.230443	2445	6.430443	1317	6.230443
6.250906	2432	6.250906	1095	6.250906	2839	6.450906	1709	6.250906
6.271368	2991	6.271368	1224	6.271368	3317	6.471368	2065	6.271368
6.291831	3320	6.291831	1528	6.291831	4000	6.491831	2284	6.291831
6.312294	4101	6.312294	1775	6.312294	4855	6.512294	2751	6.312294
6.332757	4883	6.332757	2233	6.332757	5436	6.532757	3322	6.332757
6.35322	5677	6.35322	2686	6.35322	6592	6.55322	4020	6.35322
6.373682	6763	6.373682	3063	6.373682	7763	6.573682	4757	6.373682
6.394145	8004	6.394145	3572	6.394145	9738	6.594145	5605	6.394145
6.414608	9636	6.414608	4189	6.414608	12111	6.614608	6682	6.414608
6.435071	11339	6.435071	4730	6.435071	15009	6.635071	7651	6.435071
6.455533	13471	6.455533	5798	6.455533	18129	6.655533	9382	6.455533
6.475996	15742	6.475996	7000	6.475996	21211	6.675996	11344	6.475996
6.496459	17829	6.496459	8784	6.496459	23714	6.696459	13196	6.496459
6.516922	19762	6.516922	10396	6.516922	24684	6.716922	14909	6.516922
6.537385	19929	6.537385	12129	6.537385	22839	6.737385	16281	6.537385
6.557847	18440	6.557847	13518	6.557847	18897	6.757847	17013	6.557847
6.57831	15060	6.57831	14254	6.57831	13305	6.77831	16809	6.57831
6.598773	11320	6.598773	13795	6.598773	8979	6.798773	15616	6.598773
6.619236	8133	6.619236	12588	6.619236	6229	6.819236	13179	6.619236
6.639698	5819	6.639698	10011	6.639698	4695	6.839698	10781	6.639698
6.660161	4450	6.660161	7188	6.660161	3498	6.860161	8028	6.660161
6.680624	3513	6.680624	5307	6.680624	2828	6.880624	5851	6.680624
6.701087	2869	6.701087	3895	6.701087	2138	6.901087	4278	6.701087
6.72155	2330	6.72155	2942	6.72155	1948	6.92155	3102	6.72155
6.742012	1912	6.742012	2368	6.742012	1665	6.942012	2563	6.742012
6.762475	1613	6.762475	1875	6.762475	1393	6.962475	2059	6.762475
6.782938	1416	6.782938	1690	6.782938	1303	6.982938	1674	6.782938
6.803401	1224	6.803401	1258	6.803401	1054	7.003401	1478	6.803401
6.823863	1081	6.823863	1174	6.823863	1057	7.023863	1255	6.823863
6.844326	995	6.844326	1028	6.844326	877	7.044326	1031	6.844326
6.864789	998	6.864789	935	6.864789	945	7.064789	1038	6.864789
6.885252	818	6.885252	775	6.885252	837	7.085252	839	6.885252
6.905715	798	6.905715	701	6.905715	743	7.105715	689	6.905715
6.926177	703	6.926177	625	6.926177	586	7.126177	680	6.926177
6.94664	720	6.94664	624	6.94664	712	7.14664	598	6.94664
6.967103	679	6.967103	643	6.967103	623	7.167103	653	6.967103
6.987566	728	6.987566	531	6.987566	625	7.187566	603	6.987566
7.008028	528	7.008028	427	7.008028	671	7.208028	540	7.008028
7.028491	544	7.028491	439	7.028491	662	7.228491	529	7.028491
7.048954	602	7.048954	430	7.048954	633	7.248954	451	7.048954
7.069417	634	7.069417	423	7.069417	588	7.269417	554	7.069417
7.08988	674	7.08988	422	7.08988	590	7.28988	486	7.08988
7.110342	743	7.110342	446	7.110342	769	7.310342	465	7.110342
7.130805	717	7.130805	474	7.130805	802	7.330805	558	7.130805
7.151268	806	7.151268	456	7.151268	822	7.351268	670	7.151268
7.171731	915	7.171731	471	7.171731	896	7.371731	671	7.171731
7.192193	1015	7.192193	458	7.192193	1129	7.392193	670	7.192193
7.212656	1088	7.212656	614	7.212656	1264	7.412656	783	7.212656
7.233119	1248	7.233119	625	7.233119	1494	7.433119	940	7.233119
7.253582	1363	7.253582	578	7.253582	1713	7.453582	1109	7.253582
7.274045	1699	7.274045	760	7.274045	2063	7.474045	1371	7.274045
7.294507	2238	7.294507	777	7.294507	2301	7.494507	1638	7.294507
7.31497	2474	7.31497	971	7.31497	2846	7.51497	1658	7.31497
7.335433	2756	7.335433	1164	7.335433	3414	7.535433	1975	7.335433
7.355896	3406	7.355896	1440	7.355896	4403	7.555896	2479	7.355896
7.376358	4222	7.376358	1577	7.376358	5538	7.576358	2932	7.376358
7.396821	5169	7.396821	1953	7.396821	6386	7.596821	3719	7.396821

7.417284	6052	7.417284	2323	7.417284	7722	7.617284	4448	7.417284
7.437747	7426	7.437747	2740	7.437747	9229	7.637747	5223	7.437747
7.458209	9014	7.458209	3705	7.458209	10516	7.658209	6559	7.458209
7.478672	10779	7.478672	4879	7.478672	11730	7.678672	7707	7.478672
7.499135	11277	7.499135	6006	7.499135	10705	7.699135	8167	7.499135
7.519598	10426	7.519598	6867	7.519598	7917	7.719598	8839	7.519598
7.540061	7826	7.540061	7076	7.540061	5422	7.740061	8805	7.540061
7.560523	4955	7.560523	7379	7.560523	3702	7.760523	8115	7.560523
7.580986	3300	7.580986	6661	7.580986	2350	7.780986	7492	7.580986
7.601449	2193	7.601449	5395	7.601449	1616	7.801449	6877	7.601449
7.621912	1599	7.621912	3638	7.621912	1111	7.821912	4939	7.621912
7.642374	1181	7.642374	2020	7.642374	872	7.842374	3182	7.642374
7.662837	957	7.662837	1313	7.662837	705	7.862837	2089	7.662837
7.6833	883	7.6833	854	7.6833	607	7.8833	1193	7.6833
7.703763	631	7.703763	732	7.703763	527	7.903763	888	7.703763
7.724226	572	7.724226	553	7.724226	500	7.924226	674	7.724226
7.744688	414	7.744688	418	7.744688	435	7.944688	574	7.744688
7.765151	402	7.765151	439	7.765151	309	7.965151	572	7.765151
7.785614	372	7.785614	421	7.785614	230	7.985614	516	7.785614
7.806077	409	7.806077	335	7.806077	312	8.006077	380	7.806077
7.826539	322	7.826539	283	7.826539	258	8.026539	368	7.826539
7.847002	266	7.847002	319	7.847002	314	8.047002	285	7.847002
7.867465	241	7.867465	162	7.867465	246	8.067465	322	7.867465
7.887928	164	7.887928	147	7.887928	281	8.087928	148	7.887928
7.908391	186	7.908391	159	7.908391	141	8.108391	157	7.908391
7.928853	116	7.928853	177	7.928853	183	8.128853	140	7.928853
7.949316	187	7.949316	211	7.949316	244	8.149316	218	7.949316
7.969779	200	7.969779	103	7.969779	256	8.169779	132	7.969779
7.990242	99	7.990242	120	7.990242	184	8.190242	194	7.990242
8.010705	184	8.010705	60	8.010705	287	8.210705	148	8.010705
8.031167	189	8.031167	86	8.031167	219	8.231167	90	8.031167
8.05163	153	8.05163	114	8.05163	120	8.25163	71	8.05163
8.072093	111	8.072093	75	8.072093	168	8.272093	145	8.072093
8.092556	76	8.092556	57	8.092556	112	8.292556	134	8.092556
8.113018	79	8.113018	55	8.113018	132	8.313018	141	8.113018
8.133481	63	8.133481	63	8.133481	136	8.333481	149	8.133481
8.153944	57	8.153944	111	8.153944	109	8.353944	159	8.153944
8.174407	74	8.174407	81	8.174407	130	8.374407	79	8.174407
8.19487	89	8.19487	41	8.19487	154	8.39487	45	8.19487
8.215332	94	8.215332	1	8.215332	163	8.415332	97	8.215332
8.235795	43	8.235795	61	8.235795	103	8.435795	28	8.235795
8.256258	52	8.256258	2	8.256258	74	8.456258	48	8.256258
8.276721	52	8.276721	6	8.276721	123	8.476721	4	8.276721
8.297183	0	8.297183	65	8.297183	116	8.497183	18	8.297183
8.317646	55	8.317646	64	8.317646	81	8.517646	28	8.317646
8.338109	86	8.338109	50	8.338109	104	8.538109	96	8.338109
8.358572	27	8.358572	29	8.358572	141	8.558572	14	8.358572
8.379035	48	8.379035	23	8.379035	66	8.579035	36	8.379035
8.399497	32	8.399497	0	8.399497	33	8.599497	41	8.399497
8.41996	0	8.41996	45	8.41996	160	8.61996	97	8.41996
8.440423	48	8.440423	34	8.440423	93	8.640423	40	8.440423
8.460886	57	8.460886	30	8.460886	155	8.660886	8	8.460886
8.481348	60	8.481348	25	8.481348	35	8.681348	53	8.481348
8.501811	29	8.501811	0	8.501811	66	8.701811	33	8.501811
8.522274	45	8.522274	20	8.522274	76	8.722274	20	8.522274
8.542737	39	8.542737	12	8.542737	44	8.742737	69	8.542737
8.5632	55	8.5632	25	8.5632	92	8.7632	80	8.5632
8.583662	40	8.583662	0	8.583662	60	8.783662	29	8.583662

8.604125	0	8.604125	0	8.604125	144	8.804125	16	8.604125
8.624588	70	8.624588	43	8.624588	65	8.824588	0	8.624588
8.645051	30	8.645051	0	8.645051	75	8.845051	0	8.645051
8.665513	0	8.665513	24	8.665513	0	8.865513	89	8.665513
8.685976	57	8.685976	0	8.685976	53	8.885976	5	8.685976
8.706439	0	8.706439	2	8.706439	30	8.906439	70	8.706439
8.726902	0	8.726902	0	8.726902	19	8.926902	26	8.726902
8.747365	47	8.747365	0	8.747365	45	8.947365	33	8.747365
8.767827	20	8.767827	18	8.767827	47	8.967827	34	8.767827
8.78829	0	8.78829	0	8.78829	73	8.98829	0	8.78829
8.808753	0	8.808753	21	8.808753	32	9.008753	28	8.808753
8.829216	98	8.829216	39	8.829216	45	9.029216	22	8.829216
8.849678	40	8.849678	41	8.849678	83	9.049678	0	8.849678
8.870141	0	8.870141	0	8.870141	53	9.070141	13	8.870141
8.890604	16	8.890604	0	8.890604	89	9.090604	37	8.890604
8.911067	10	8.911067	19	8.911067	0	9.111067	2	8.911067
8.93153	0	8.93153	49	8.93153	0	9.13153	55	8.93153
8.951992	14	8.951992	0	8.951992	32	9.151992	24	8.951992
8.972455	0	8.972455	0	8.972455	26	9.172455	0	8.972455
8.992918	13	8.992918	27	8.992918	21	9.192918	0	8.992918
9.013381	33	9.013381	20	9.013381	37	9.213381	0	9.013381
9.033843	6	9.033843	0	9.033843	0	9.233843	0	9.033843
9.054306	36	9.054306	16	9.054306	0	9.254306	59	9.054306
9.074769	0	9.074769	30	9.074769	42	9.274769	29	9.074769
9.095232	45	9.095232	0	9.095232	1	9.295232	0	9.095232
9.115695	0	9.115695	0	9.115695	27	9.315695	0	9.115695
9.136157	0	9.136157	0	9.136157	60	9.336157	0	9.136157
9.15662	0	9.15662	16	9.15662	87	9.35662	44	9.15662
9.177083	23	9.177083	0	9.177083	13	9.377083	38	9.177083
9.197546	24	9.197546	8	9.197546	50	9.397546	0	9.197546
9.218008	5	9.218008	0	9.218008	10	9.418008	0	9.218008
9.238471	24	9.238471	0	9.238471	122	9.438471	0	9.238471
9.258934	13	9.258934	0	9.258934	23	9.458934	32	9.258934
9.279397	72	9.279397	0	9.279397	6	9.479397	0	9.279397
9.29986	32	9.29986	0	9.29986	1	9.49986	66	9.29986
9.320322	0	9.320322	0	9.320322	4	9.520322	55	9.320322
9.340785	0	9.340785	38	9.340785	0	9.540785	34	9.340785
9.361248	0	9.361248	0	9.361248	0	9.561248	76	9.361248
9.381711	14	9.381711	18	9.381711	76	9.581711	21	9.381711
9.402173	8	9.402173	0	9.402173	85	9.602173	7	9.402173
9.422636	27	9.422636	24	9.422636	22	9.622636	0	9.422636
9.443099	30	9.443099	0	9.443099	43	9.643099	10	9.443099
9.463562	65	9.463562	0	9.463562	2	9.663562	80	9.463562
9.484025	22	9.484025	32	9.484025	106	9.684025	50	9.484025
9.504487	62	9.504487	0	9.504487	14	9.704487	20	9.504487
9.52495	0	9.52495	0	9.52495	2	9.72495	33	9.52495
9.545413	19	9.545413	56	9.545413	12	9.745413	33	9.545413
9.565876	34	9.565876	0	9.565876	30	9.765876	52	9.565876
9.586338	29	9.586338	9	9.586338	0	9.786338	106	9.586338
9.606801	0	9.606801	0	9.606801	8	9.806801	0	9.606801
9.627264	0	9.627264	26	9.627264	42	9.827264	0	9.627264
9.647727	7	9.647727	19	9.647727	0	9.847727	0	9.647727
9.66819	4	9.66819	0	9.66819	8	9.86819	0	9.66819
9.688652	1	9.688652	13	9.688652	5	9.888652	17	9.688652
9.709115	92	9.709115	55	9.709115	0	9.909115	0	9.709115
9.729578	32	9.729578	25	9.729578	49	9.929578	0	9.729578
9.750041	48	9.750041	0	9.750041	0	9.950041	0	9.750041
9.770503	0	9.770503	0	9.770503	0	9.970503	0	9.770503

9.790966	0	9.790966	53	9.790966	0	9.990966	16	9.790966	
9.811429	0	9.811429	34	9.811429	27	10.01143	0	9.811429	
9.831892	37	9.831892	14	9.831892	7	10.03189	25	9.831892	
9.852355	0	9.852355	18	9.852355	0	10.05236	4	9.852355	
9.872817	128	9.872817	27	9.872817	5	10.07282	48	9.872817	
9.89328	89	9.89328	17	9.89328	3	10.09328	0	9.89328	
9.913743	7	9.913743	0	9.913743	27	10.11374	11	9.913743	
9.934206	0	9.934206	8	9.934206	1	10.13421	0	9.934206	
9.954668	34	9.954668	5	9.954668	38	10.15467	0	9.954668	
9.975131	49	9.975131	0	9.975131	59	10.17513	7	9.975131	
9.995594	35	9.995594	54	9.995594	5	10.19559	1	9.995594	
10.01606	44	10.01606	13	10.01606	40	10.21606	56	10.01606	
10.03652	37	10.03652	0	10.03652	0	10.23652	0	10.03652	
10.05698	49	10.05698	0	10.05698	43	10.25698	0	10.05698	
10.07745	36	10.07745	39	10.07745	81	10.27745	0	10.07745	
10.09791	40	10.09791	15	10.09791	64	10.29791	0	10.09791	
10.11837	0	10.11837	0	10.11837	38	10.31837	36	10.11837	
10.13883	61	10.13883	0	10.13883	87	10.33883	73	10.13883	
10.1593	28	10.1593	0	10.1593	33	10.3593	0	10.1593	
10.17976	68	10.17976	38	10.17976	21	10.37976	35	10.17976	
10.20022	67	10.20022	31	10.20022	75	10.40022	96	10.20022	
10.22069	167	10.22069	33	10.22069	95	10.42069	121	10.22069	
10.24115	124	10.24115	44	10.24115	127	10.44115	92	10.24115	
10.26161	172	10.26161	90	10.26161	134	10.46161	119	10.26161	
10.28207	214	10.28207	48	10.28207	153	10.48207	157	10.28207	
10.30254	221	10.30254	139	10.30254	240	10.50254	196	10.30254	
10.323	325	10.323	166	10.323	251	10.523	191	10.323	
10.34346	426	10.34346	143	10.34346	380	10.54346	302	10.34346	
10.36392	532	10.36392	226	10.36392	491	10.56392	347	10.36392	
10.38439	632	10.38439	269	10.38439	656	10.58439	432	10.38439	
10.40485	891	10.40485	382	10.40485	899	10.60485	593	10.40485	
10.42531	1077	10.42531	341	10.42531	1171	10.62531	694	10.42531	
10.44578	1356	10.44578	499	10.44578	1518	10.64578	1025	10.44578	
10.46624	1771	10.46624	671	10.46624	2099	10.66624	1335	10.46624	
10.4867	2308	10.4867	934	10.4867	2598	10.6867	1781	10.4867	
10.50716	2889	10.50716	1210	10.50716	3180	10.70716	2118	10.50716	
10.52763	3436	10.52763	1613	10.52763	3614	10.72763	2629	10.52763	
10.54809	3843	10.54809	1870	10.54809	3656	10.74809	2839	10.54809	
10.56855	3720	10.56855	2183	10.56855	3249	10.76855	2990	10.56855	
10.58902	3169	10.58902	2675	10.58902	2555	10.78902	2904	10.58902	
10.60948	2212	10.60948	2759	10.60948	1904	10.80948	2941	10.60948	
10.62994	1445	10.62994	2508	10.62994	1100	10.82994	2758	10.62994	
10.6504	907	10.6504	1973	10.6504	740	10.8504	2308	10.6504	
10.67087	597	10.67087	1407	10.67087	415	10.87087	1836	10.67087	
10.69133	448	10.69133	1000	10.69133	334	10.89133	1249	10.69133	
10.71179	393	10.71179	511	10.71179	224	10.91179	755	10.71179	
10.73225	227	10.73225	454	10.73225	162	10.93225	522	10.73225	
10.75272	207	10.75272	332	10.75272	99	10.95272	356	10.75272	
10.77318	152	10.77318	194	10.77318	67	10.97318	213	10.77318	
10.79364	97	10.79364	146	10.79364	73	10.99364	130	10.79364	
10.81411	96	10.81411	65	10.81411	119	11.01411	151	10.81411	
10.83457	70	10.83457	132	10.83457	14	11.03457	176	10.83457	
10.85503	38	10.85503	60	10.85503	0	11.05503	89	10.85503	
10.87549	85	10.87549	33	10.87549	0	11.07549	113	10.87549	
10.89596	28	10.89596	17	10.89596	52	11.09596	85	10.89596	
10.91642	22	10.91642	83	10.91642	0	11.11642	41	10.91642	
10.93688	63	10.93688	32	10.93688	0	11.13688	72	10.93688	
10.95735	67	10.95735	33	10.95735	0	11.15735	16	10.95735	

10.97781	0	10.97781	0	10.97781	3	11.17781	9	10.97781
10.99827	9	10.99827	0	10.99827	0	11.19827	0	10.99827
11.01873	65	11.01873	0	11.01873	70	11.21873	20	11.01873
11.0392	29	11.0392	0	11.0392	0	11.2392	11	11.0392
11.05966	41	11.05966	28	11.05966	0	11.25966	10	11.05966
11.08012	0	11.08012	31	11.08012	48	11.28012	0	11.08012
11.10058	12	11.10058	0	11.10058	0	11.30058	22	11.10058
11.12105	43	11.12105	26	11.12105	0	11.32105	25	11.12105
11.14151	29	11.14151	0	11.14151	0	11.34151	0	11.14151
11.16197	3	11.16197	0	11.16197	18	11.36197	3	11.16197
11.18244	0	11.18244	0	11.18244	29	11.38244	0	11.18244
11.2029	1	11.2029	0	11.2029	26	11.4029	0	11.2029
11.22336	0	11.22336	25	11.22336	0	11.42336	10	11.22336
11.24382	0	11.24382	3	11.24382	1	11.44382	3	11.24382
11.26429	0	11.26429	0	11.26429	6	11.46429	22	11.26429
11.28475	0	11.28475	0	11.28475	55	11.48475	0	11.28475
11.30521	22	11.30521	0	11.30521	0	11.50521	0	11.30521
11.32568	0	11.32568	0	11.32568	5	11.52568	0	11.32568
11.34614	15	11.34614	49	11.34614	0	11.54614	23	11.34614
11.3666	0	11.3666	36	11.3666	0	11.5666	26	11.3666
11.38706	0	11.38706	0	11.38706	0	11.58706	45	11.38706
11.40753	29	11.40753	13	11.40753	49	11.60753	61	11.40753
11.42799	0	11.42799	0	11.42799	0	11.62799	0	11.42799
11.44845	8	11.44845	0	11.44845	6	11.64845	19	11.44845
11.46891	75	11.46891	37	11.46891	27	11.66891	0	11.46891
11.48938	11	11.48938	0	11.48938	21	11.68938	0	11.48938
11.50984	0	11.50984	0	11.50984	0	11.70984	4	11.50984
11.5303	1	11.5303	0	11.5303	0	11.7303	0	11.5303
11.55077	0	11.55077	53	11.55077	6	11.75077	0	11.55077
11.57123	43	11.57123	15	11.57123	0	11.77123	24	11.57123
11.59169	0	11.59169	0	11.59169	0	11.79169	0	11.59169
11.61215	0	11.61215	0	11.61215	17	11.81215	6	11.61215
11.63262	0	11.63262	49	11.63262	0	11.83262	0	11.63262
11.65308	0	11.65308	0	11.65308	32	11.85308	10	11.65308
11.67354	15	11.67354	0	11.67354	26	11.87354	7	11.67354
11.69401	0	11.69401	37	11.69401	1	11.89401	12	11.69401
11.71447	3	11.71447	7	11.71447	15	11.91447	12	11.71447
11.73493	0	11.73493	0	11.73493	0	11.93493	14	11.73493
11.75539	23	11.75539	8	11.75539	0	11.95539	0	11.75539
11.77586	0	11.77586	8	11.77586	23	11.97586	0	11.77586
11.79632	0	11.79632	0	11.79632	22	11.99632	14	11.79632
11.81678	0	11.81678	0	11.81678	0	12.01678	12	11.81678
11.83724	0	11.83724	20	11.83724	0	12.03724	22	11.83724
11.85771	27	11.85771	17	11.85771	1	12.05771	26	11.85771
11.87817	0	11.87817	0	11.87817	30	12.07817	10	11.87817
11.89863	0	11.89863	19	11.89863	0	12.09863	11	11.89863
11.9191	13	11.9191	0	11.9191	0	12.1191	0	11.9191
11.93956	2	11.93956	30	11.93956	0	12.13956	0	11.93956
11.96002	30	11.96002	6	11.96002	48	12.16002	28	11.96002
11.98048	28	11.98048	0	11.98048	66	12.18048	42	11.98048
12.00095	14	12.00095	0	12.00095	77	12.20095	40	12.00095
12.02141	48	12.02141	0	12.02141	43	12.22141	30	12.02141
12.04187	36	12.04187	48	12.04187	61	12.24187	70	12.04187
12.06233	48	12.06233	0	12.06233	88	12.26233	29	12.06233
12.0828	40	12.0828	0	12.0828	89	12.2828	70	12.0828
12.10326	126	12.10326	0	12.10326	116	12.30326	43	12.10326
12.12372	128	12.12372	47	12.12372	204	12.32372	44	12.12372
12.14419	177	12.14419	116	12.14419	135	12.34419	108	12.14419

12.16465	224	12.16465	69	12.16465	279	12.36465	167	12.16465	
12.18511	316	12.18511	88	12.18511	269	12.38511	207	12.18511	
12.20557	352	12.20557	119	12.20557	447	12.40557	259	12.20557	
12.22604	525	12.22604	224	12.22604	554	12.42604	320	12.22604	
12.2465	720	12.2465	251	12.2465	738	12.4465	409	12.2465	
12.26696	1005	12.26696	332	12.26696	1127	12.46696	623	12.26696	
12.28743	1243	12.28743	524	12.28743	1477	12.48743	870	12.28743	
12.30789	1617	12.30789	728	12.30789	1991	12.50789	1194	12.30789	
12.32835	2103	12.32835	926	12.32835	2553	12.52835	1423	12.32835	
12.34881	2632	12.34881	1193	12.34881	3150	12.54881	1758	12.34881	
12.36928	3131	12.36928	1501	12.36928	3048	12.56928	2038	12.36928	
12.38974	3218	12.38974	1769	12.38974	2836	12.58974	2197	12.38974	
12.4102	2660	12.4102	2013	12.4102	2147	12.6102	2261	12.4102	
12.43066	1939	12.43066	2125	12.43066	1423	12.63066	2202	12.43066	
12.45113	1346	12.45113	2015	12.45113	811	12.65113	2020	12.45113	
12.47159	776	12.47159	1677	12.47159	502	12.67159	1650	12.47159	
12.49205	515	12.49205	1166	12.49205	308	12.69205	1268	12.49205	
12.51252	297	12.51252	719	12.51252	225	12.71252	849	12.51252	
12.53298	263	12.53298	395	12.53298	158	12.73298	602	12.53298	
12.55344	232	12.55344	330	12.55344	99	12.75344	378	12.55344	
12.5739	165	12.5739	208	12.5739	210	12.7739	230	12.5739	
12.59437	91	12.59437	258	12.59437	34	12.79437	192	12.59437	
12.61483	132	12.61483	129	12.61483	66	12.81483	106	12.61483	
12.63529	160	12.63529	87	12.63529	64	12.83529	165	12.63529	
12.65576	119	12.65576	83	12.65576	96	12.85576	106	12.65576	
12.67622	114	12.67622	11	12.67622	86	12.87622	92	12.67622	
12.69668	120	12.69668	69	12.69668	77	12.89668	133	12.69668	
12.71714	108	12.71714	67	12.71714	126	12.91714	99	12.71714	
12.73761	139	12.73761	59	12.73761	162	12.93761	88	12.73761	
12.75807	154	12.75807	136	12.75807	186	12.95807	126	12.75807	
12.77853	222	12.77853	84	12.77853	245	12.97853	209	12.77853	
12.79899	430	12.79899	215	12.79899	395	12.99899	229	12.79899	
12.81946	430	12.81946	169	12.81946	450	13.01946	299	12.81946	
12.83992	571	12.83992	216	12.83992	605	13.03992	381	12.83992	
12.86038	715	12.86038	355	12.86038	873	13.06038	569	12.86038	
12.88085	876	12.88085	471	12.88085	1079	13.08085	750	12.88085	
12.90131	899	12.90131	565	12.90131	1156	13.10131	899	12.90131	
12.92177	903	12.92177	760	12.92177	1091	13.12177	986	12.92177	
12.94223	794	12.94223	813	12.94223	923	13.14223	1013	12.94223	
12.9627	500	12.9627	871	12.9627	635	13.1627	1052	12.9627	
12.98316	330	12.98316	769	12.98316	390	13.18316	850	12.98316	
13.00362	236	13.00362	797	13.00362	218	13.20362	780	13.00362	
13.02409	174	13.02409	470	13.02409	165	13.22409	526	13.02409	
13.04455	92	13.04455	352	13.04455	35	13.24455	306	13.04455	
13.06501	51	13.06501	222	13.06501	83	13.26501	187	13.06501	
13.08547	53	13.08547	127	13.08547	48	13.28547	176	13.08547	
13.10594	0	13.10594	68	13.10594	67	13.30594	107	13.10594	
13.1264	61	13.1264	78	13.1264	41	13.3264	34	13.1264	
13.14686	12	13.14686	43	13.14686	28	13.34686	42	13.14686	
13.16732	0	13.16732	0	13.16732	0	13.36732	28	13.16732	
13.18779	20	13.18779	73	13.18779	0	13.38779	47	13.18779	
13.20825	0	13.20825	22	13.20825	0	13.40825	24	13.20825	
13.22871	0	13.22871	0	13.22871	13	13.42871	21	13.22871	
13.24918	11	13.24918	1	13.24918	0	13.44918	72	13.24918	
13.26964	0	13.26964	36	13.26964	23	13.46964	3	13.26964	
13.2901	0	13.2901	45	13.2901	0	13.4901	0	13.2901	
13.31056	4	13.31056	12	13.31056	0	13.51056	0	13.31056	
13.33103	0	13.33103	0	13.33103	0	13.53103	17	13.33103	

13.35149	29	13.35149	101	13.35149	35	13.55149	0	13.35149
13.37195	15	13.37195	59	13.37195	0	13.57195	15	13.37195
13.39242	22	13.39242	14	13.39242	19	13.59242	1	13.39242
13.41288	0	13.41288	49	13.41288	0	13.61288	6	13.41288
13.43334	42	13.43334	63	13.43334	0	13.63334	0	13.43334
13.4538	0	13.4538	14	13.4538	0	13.6538	39	13.4538
13.47427	15	13.47427	70	13.47427	0	13.67427	26	13.47427
13.49473	1	13.49473	112	13.49473	0	13.69473	49	13.49473
13.51519	40	13.51519	72	13.51519	29	13.71519	35	13.51519
13.53565	30	13.53565	15	13.53565	23	13.73565	0	13.53565
13.55612	0	13.55612	16	13.55612	30	13.75612	3	13.55612
13.57658	19	13.57658	113	13.57658	4	13.77658	11	13.57658
13.59704	0	13.59704	80	13.59704	0	13.79704	0	13.59704
13.61751	76	13.61751	42	13.61751	0	13.81751	17	13.61751
13.63797	29	13.63797	95	13.63797	39	13.83797	4	13.63797
13.65843	12	13.65843	79	13.65843	39	13.85843	22	13.65843
13.67889	0	13.67889	96	13.67889	33	13.87889	55	13.67889
13.69936	0	13.69936	62	13.69936	0	13.89936	39	13.69936
13.71982	0	13.71982	86	13.71982	29	13.91982	41	13.71982
13.74028	0	13.74028	75	13.74028	79	13.94028	18	13.74028
13.76075	0	13.76075	131	13.76075	15	13.96075	0	13.76075
13.78121	48	13.78121	146	13.78121	39	13.98121	0	13.78121
13.80167	42	13.80167	133	13.80167	1	14.00167	0	13.80167
13.82213	0	13.82213	190	13.82213	0	14.02213	13	13.82213
13.8426	3	13.8426	107	13.8426	107	14.0426	22	13.8426
13.86306	59	13.86306	153	13.86306	19	14.06306	35	13.86306
13.88352	33	13.88352	116	13.88352	62	14.08352	35	13.88352
13.90398	31	13.90398	174	13.90398	130	14.10398	17	13.90398
13.92445	75	13.92445	177	13.92445	101	14.12445	12	13.92445
13.94491	24	13.94491	128	13.94491	56	14.14491	0	13.94491
13.96537	24	13.96537	174	13.96537	96	14.16537	36	13.96537
13.98584	80	13.98584	224	13.98584	89	14.18584	31	13.98584
14.0063	31	14.0063	210	14.0063	150	14.2063	26	14.0063
14.02676	3	14.02676	250	14.02676	121	14.22676	90	14.02676
14.04722	44	14.04722	203	14.04722	126	14.24722	62	14.04722
14.06769	46	14.06769	222	14.06769	121	14.26769	70	14.06769
14.08815	84	14.08815	309	14.08815	71	14.28815	0	14.08815
14.10861	50	14.10861	289	14.10861	154	14.30861	40	14.10861
14.12908	27	14.12908	235	14.12908	147	14.32908	31	14.12908
14.14954	24	14.14954	269	14.14954	161	14.34954	0	14.14954
14.17	65	14.17	291	14.17	185	14.37	85	14.17
14.19046	67	14.19046	293	14.19046	130	14.39046	47	14.19046
14.21093	30	14.21093	304	14.21093	124	14.41093	9	14.21093
14.23139	77	14.23139	415	14.23139	136	14.43139	70	14.23139
14.25185	68	14.25185	295	14.25185	139	14.45185	61	14.25185
14.27231	127	14.27231	342	14.27231	245	14.47231	60	14.27231
14.29278	62	14.29278	379	14.29278	176	14.49278	14	14.29278
14.31324	43	14.31324	374	14.31324	181	14.51324	53	14.31324
14.3337	75	14.3337	383	14.3337	219	14.5337	81	14.3337
14.35417	87	14.35417	417	14.35417	229	14.55417	59	14.35417
14.37463	91	14.37463	414	14.37463	215	14.57463	78	14.37463
14.39509	69	14.39509	456	14.39509	223	14.59509	41	14.39509
14.41555	106	14.41555	381	14.41555	265	14.61555	94	14.41555
14.43602	90	14.43602	488	14.43602	182	14.63602	69	14.43602
14.45648	137	14.45648	344	14.45648	191	14.65648	88	14.45648
14.47694	137	14.47694	419	14.47694	241	14.67694	93	14.47694
14.49741	123	14.49741	455	14.49741	302	14.69741	86	14.49741
14.51787	109	14.51787	442	14.51787	187	14.71787	47	14.51787

14.53833	111	14.53833	492	14.53833	283	14.73833	81	14.53833	
14.55879	135	14.55879	378	14.55879	278	14.75879	85	14.55879	
14.57926	137	14.57926	402	14.57926	388	14.77926	103	14.57926	
14.59972	179	14.59972	489	14.59972	300	14.79972	76	14.59972	
14.62018	144	14.62018	530	14.62018	311	14.82018	100	14.62018	
14.64064	141	14.64064	554	14.64064	388	14.84064	107	14.64064	
14.66111	132	14.66111	503	14.66111	347	14.86111	143	14.66111	
14.68157	117	14.68157	529	14.68157	429	14.88157	125	14.68157	
14.70203	188	14.70203	520	14.70203	446	14.90203	118	14.70203	
14.7225	232	14.7225	550	14.7225	397	14.9225	145	14.7225	
14.74296	296	14.74296	544	14.74296	500	14.94296	219	14.74296	
14.76342	218	14.76342	551	14.76342	592	14.96342	268	14.76342	
14.78388	362	14.78388	668	14.78388	720	14.98388	324	14.78388	
14.80435	495	14.80435	642	14.80435	816	15.00435	425	14.80435	
14.82481	670	14.82481	796	14.82481	1043	15.02481	488	14.82481	
14.84527	860	14.84527	742	14.84527	1369	15.04527	632	14.84527	
14.86574	1286	14.86574	965	14.86574	1625	15.06574	830	14.86574	
14.8862	1398	14.8862	1026	14.8862	2017	15.0862	1058	14.8862	
14.90666	1633	14.90666	1249	14.90666	2230	15.10666	1240	14.90666	
14.92712	1851	14.92712	1492	14.92712	2125	15.12712	1431	14.92712	
14.94759	1585	14.94759	1405	14.94759	1727	15.14759	1516	14.94759	
14.96805	1415	14.96805	1481	14.96805	1300	15.16805	1489	14.96805	
14.98851	1007	14.98851	1445	14.98851	913	15.18851	1322	14.98851	
15.00897	691	15.00897	1348	15.00897	624	15.20897	1161	15.00897	
15.02944	496	15.02944	1137	15.02944	461	15.22944	847	15.02944	
15.0499	370	15.0499	927	15.0499	430	15.2499	651	15.0499	
15.07036	247	15.07036	723	15.07036	324	15.27036	476	15.07036	
15.09083	201	15.09083	477	15.09083	286	15.29083	301	15.09083	
15.11129	176	15.11129	547	15.11129	304	15.31129	258	15.11129	
15.13175	121	15.13175	454	15.13175	270	15.33175	203	15.13175	
15.15221	86	15.15221	485	15.15221	249	15.35221	184	15.15221	
15.17268	44	15.17268	425	15.17268	196	15.37268	96	15.17268	
15.19314	70	15.19314	421	15.19314	207	15.39314	125	15.19314	
15.2136	82	15.2136	330	15.2136	217	15.4136	88	15.2136	
15.23407	70	15.23407	377	15.23407	225	15.43407	81	15.23407	
15.25453	0	15.25453	335	15.25453	106	15.45453	82	15.25453	
15.27499	115	15.27499	392	15.27499	162	15.47499	79	15.27499	
15.29545	22	15.29545	335	15.29545	185	15.49545	76	15.29545	
15.31592	89	15.31592	374	15.31592	105	15.51592	0	15.31592	
15.33638	70	15.33638	393	15.33638	182	15.53638	1	15.33638	
15.35684	47	15.35684	283	15.35684	141	15.55684	34	15.35684	
15.3773	31	15.3773	235	15.3773	195	15.5773	27	15.3773	
15.39777	16	15.39777	282	15.39777	84	15.59777	38	15.39777	
15.41823	54	15.41823	221	15.41823	105	15.61823	63	15.41823	
15.43869	43	15.43869	273	15.43869	154	15.63869	29	15.43869	
15.45916	30	15.45916	188	15.45916	93	15.65916	20	15.45916	
15.47962	61	15.47962	248	15.47962	138	15.67962	58	15.47962	
15.50008	34	15.50008	285	15.50008	103	15.70008	56	15.50008	
15.52054	11	15.52054	218	15.52054	75	15.72054	41	15.52054	
15.54101	29	15.54101	250	15.54101	101	15.74101	0	15.54101	
15.56147	0	15.56147	234	15.56147	100	15.76147	21	15.56147	
15.58193	43	15.58193	223	15.58193	146	15.78193	40	15.58193	
15.6024	44	15.6024	200	15.6024	116	15.8024	16	15.6024	
15.62286	46	15.62286	208	15.62286	59	15.82286	0	15.62286	
15.64332	0	15.64332	147	15.64332	81	15.84332	0	15.64332	
15.66378	51	15.66378	241	15.66378	107	15.86378	0	15.66378	
15.68425	0	15.68425	165	15.68425	94	15.88425	23	15.68425	
15.70471	46	15.70471	238	15.70471	62	15.90471	32	15.70471	

15.72517	14	15.72517	241	15.72517	111	15.92517	16	15.72517
15.74563	21	15.74563	252	15.74563	79	15.94563	48	15.74563
15.7661	39	15.7661	291	15.7661	64	15.9661	57	15.7661
15.78656	0	15.78656	220	15.78656	82	15.98656	13	15.78656
15.80702	76	15.80702	182	15.80702	51	16.00702	36	15.80702
15.82749	23	15.82749	254	15.82749	51	16.02749	26	15.82749
15.84795	0	15.84795	256	15.84795	61	16.04795	8	15.84795
15.86841	7	15.86841	166	15.86841	85	16.06841	25	15.86841
15.88887	34	15.88887	225	15.88887	51	16.08887	0	15.88887
15.90934	0	15.90934	196	15.90934	23	16.10934	1	15.90934
15.9298	11	15.9298	208	15.9298	105	16.1298	51	15.9298
15.95026	10	15.95026	171	15.95026	88	16.15026	0	15.95026
15.97073	13	15.97073	240	15.97073	44	16.17073	0	15.97073
15.99119	17	15.99119	266	15.99119	112	16.19119	34	15.99119
16.01165	0	16.01165	255	16.01165	131	16.21165	9	16.01165
16.03211	46	16.03211	241	16.03211	153	16.23211	27	16.03211
16.05258	50	16.05258	282	16.05258	138	16.25258	36	16.05258
16.07304	88	16.07304	256	16.07304	126	16.27304	67	16.07304
16.0935	127	16.0935	310	16.0935	145	16.2935	13	16.0935
16.11396	119	16.11396	257	16.11396	227	16.31396	96	16.11396
16.13443	172	16.13443	259	16.13443	300	16.33443	111	16.13443
16.15489	175	16.15489	335	16.15489	361	16.35489	175	16.15489
16.17535	346	16.17535	304	16.17535	551	16.37535	200	16.17535
16.19582	448	16.19582	401	16.19582	741	16.39582	279	16.19582
16.21628	631	16.21628	490	16.21628	883	16.41628	389	16.21628
16.23674	729	16.23674	545	16.23674	979	16.43674	479	16.23674
16.2572	802	16.2572	627	16.2572	825	16.4572	515	16.2572
16.27767	714	16.27767	689	16.27767	703	16.47767	483	16.27767
16.29813	575	16.29813	737	16.29813	545	16.49813	599	16.29813
16.31859	346	16.31859	763	16.31859	353	16.51859	453	16.31859
16.33906	218	16.33906	646	16.33906	278	16.53906	375	16.33906
16.35952	183	16.35952	552	16.35952	234	16.55952	341	16.35952
16.37998	126	16.37998	459	16.37998	159	16.57998	236	16.37998
16.40044	80	16.40044	401	16.40044	208	16.60044	128	16.40044
16.42091	53	16.42091	329	16.42091	129	16.62091	81	16.42091
16.44137	114	16.44137	339	16.44137	195	16.64137	114	16.44137
16.46183	42	16.46183	352	16.46183	180	16.66183	107	16.46183
16.48229	118	16.48229	288	16.48229	153	16.68229	60	16.48229
16.50276	94	16.50276	283	16.50276	159	16.70276	52	16.50276
16.52322	60	16.52322	329	16.52322	168	16.72322	76	16.52322
16.54368	80	16.54368	261	16.54368	188	16.74368	60	16.54368
16.56415	51	16.56415	274	16.56415	163	16.76415	78	16.56415
16.58461	79	16.58461	310	16.58461	126	16.78461	89	16.58461
16.60507	108	16.60507	228	16.60507	263	16.80507	103	16.60507
16.62553	118	16.62553	278	16.62553	246	16.82553	116	16.62553
16.646	129	16.646	315	16.646	294	16.846	183	16.646
16.66646	154	16.66646	271	16.66646	280	16.86646	180	16.66646
16.68692	188	16.68692	332	16.68692	187	16.88692	184	16.68692
16.70739	179	16.70739	300	16.70739	268	16.90739	242	16.70739
16.72785	97	16.72785	328	16.72785	193	16.92785	199	16.72785
16.74831	146	16.74831	311	16.74831	166	16.94831	206	16.74831
16.76877	150	16.76877	291	16.76877	110	16.96877	149	16.76877
16.78924	92	16.78924	264	16.78924	150	16.98924	160	16.78924
16.8097	95	16.8097	219	16.8097	91	17.0097	95	16.8097
16.83016	29	16.83016	198	16.83016	63	17.03016	52	16.83016
16.85062	50	16.85062	199	16.85062	56	17.05062	45	16.85062
16.87109	116	16.87109	166	16.87109	76	17.07109	41	16.87109
16.89155	55	16.89155	131	16.89155	53	17.09155	76	16.89155

16.91201	49	16.91201	163	16.91201	46	17.11201	67	16.91201	
16.93248	25	16.93248	123	16.93248	65	17.13248	61	16.93248	
16.95294	19	16.95294	139	16.95294	104	17.15294	37	16.95294	
16.9734	27	16.9734	129	16.9734	84	17.1734	0	16.9734	
16.99386	0	16.99386	149	16.99386	16	17.19386	51	16.99386	
17.01433	0	17.01433	122	17.01433	101	17.21433	6	17.01433	
17.03479	0	17.03479	72	17.03479	135	17.23479	37	17.03479	
17.05525	7	17.05525	99	17.05525	51	17.25525	14	17.05525	
17.07572	32	17.07572	93	17.07572	28	17.27572	7	17.07572	
17.09618	6	17.09618	66	17.09618	32	17.29618	8	17.09618	
17.11664	5	17.11664	64	17.11664	55	17.31664	2	17.11664	
17.1371	48	17.1371	72	17.1371	48	17.3371	10	17.1371	
17.15757	46	17.15757	49	17.15757	5	17.35757	0	17.15757	
17.17803	0	17.17803	35	17.17803	43	17.37803	0	17.17803	
17.19849	28	17.19849	103	17.19849	33	17.39849	7	17.19849	
17.21895	8	17.21895	72	17.21895	21	17.41895	11	17.21895	
17.23942	8	17.23942	53	17.23942	0	17.43942	32	17.23942	
17.25988	28	17.25988	32	17.25988	8	17.45988	30	17.25988	
17.28034	0	17.28034	7	17.28034	0	17.48034	17	17.28034	
17.30081	7	17.30081	47	17.30081	0	17.50081	55	17.30081	
17.32127	0	17.32127	21	17.32127	19	17.52127	37	17.32127	
17.34173	4	17.34173	15	17.34173	21	17.54173	0	17.34173	
17.36219	0	17.36219	6	17.36219	0	17.56219	6	17.36219	
17.38266	0	17.38266	18	17.38266	0	17.58266	45	17.38266	
17.40312	10	17.40312	0	17.40312	46	17.60312	54	17.40312	
17.42358	14	17.42358	63	17.42358	0	17.62358	19	17.42358	
17.44405	15	17.44405	2	17.44405	0	17.64405	0	17.44405	
17.46451	0	17.46451	0	17.46451	18	17.66451	20	17.46451	
17.48497	4	17.48497	30	17.48497	0	17.68497	40	17.48497	
17.50543	31	17.50543	0	17.50543	0	17.70543	12	17.50543	
17.5259	47	17.5259	0	17.5259	1	17.7259	3	17.5259	
17.54636	32	17.54636	8	17.54636	0	17.74636	11	17.54636	
17.56682	10	17.56682	16	17.56682	29	17.76682	0	17.56682	
17.58728	46	17.58728	25	17.58728	4	17.78728	2	17.58728	
17.60775	13	17.60775	0	17.60775	0	17.80775	2	17.60775	
17.62821	17	17.62821	0	17.62821	0	17.82821	0	17.62821	
17.64867	0	17.64867	40	17.64867	0	17.84867	0	17.64867	
17.66914	8	17.66914	0	17.66914	16	17.86914	26	17.66914	
17.6896	14	17.6896	0	17.6896	22	17.8896	44	17.6896	
17.71006	5	17.71006	4	17.71006	15	17.91006	0	17.71006	
17.73052	0	17.73052	9	17.73052	50	17.93052	2	17.73052	
17.75099	0	17.75099	0	17.75099	0	17.95099	19	17.75099	
17.77145	12	17.77145	0	17.77145	0	17.97145	0	17.77145	
17.79191	2	17.79191	0	17.79191	0	17.99191	19	17.79191	
17.81238	20	17.81238	0	17.81238	21	18.01238	19	17.81238	
17.83284	48	17.83284	41	17.83284	0	18.03284	9	17.83284	
17.8533	0	17.8533	9	17.8533	0	18.0533	0	17.8533	
17.87376	0	17.87376	0	17.87376	0	18.07376	0	17.87376	
17.89423	47	17.89423	0	17.89423	0	18.09423	33	17.89423	
17.91469	30	17.91469	1	17.91469	0	18.11469	46	17.91469	
17.93515	51	17.93515	0	17.93515	1	18.13515	4	17.93515	
17.95561	0	17.95561	0	17.95561	15	18.15561	0	17.95561	
17.97608	7	17.97608	57	17.97608	16	18.17608	1	17.97608	
17.99654	26	17.99654	14	17.99654	0	18.19654	14	17.99654	
18.017	40	18.017	8	18.017	0	18.217	71	18.017	
18.03747	74	18.03747	4	18.03747	106	18.23747	62	18.03747	
18.05793	79	18.05793	10	18.05793	87	18.25793	46	18.05793	
18.07839	117	18.07839	12	18.07839	90	18.27839	82	18.07839	

18.09885	113	18.09885	88	18.09885	168	18.29885	116	18.09885	
18.11932	206	18.11932	96	18.11932	133	18.31932	122	18.11932	
18.13978	254	18.13978	107	18.13978	296	18.33978	216	18.13978	
18.16024	405	18.16024	168	18.16024	418	18.36024	336	18.16024	
18.18071	552	18.18071	174	18.18071	671	18.38071	479	18.18071	
18.20117	691	18.20117	370	18.20117	854	18.40117	602	18.20117	
18.22163	979	18.22163	452	18.22163	1036	18.42163	734	18.22163	
18.24209	1158	18.24209	583	18.24209	1322	18.44209	874	18.24209	
18.26256	1301	18.26256	695	18.26256	1336	18.46256	1038	18.26256	
18.28302	1280	18.28302	850	18.28302	1219	18.48302	1158	18.28302	
18.30348	1031	18.30348	1000	18.30348	978	18.50348	1188	18.30348	
18.32394	807	18.32394	877	18.32394	640	18.52394	1113	18.32394	
18.34441	579	18.34441	766	18.34441	441	18.54441	988	18.34441	
18.36487	359	18.36487	692	18.36487	306	18.56487	707	18.36487	
18.38533	227	18.38533	483	18.38533	227	18.58533	492	18.38533	
18.4058	182	18.4058	253	18.4058	80	18.6058	342	18.4058	
18.42626	132	18.42626	155	18.42626	127	18.62626	216	18.42626	
18.44672	96	18.44672	92	18.44672	53	18.64672	159	18.44672	
18.46718	50	18.46718	32	18.46718	39	18.66718	72	18.46718	
18.48765	23	18.48765	82	18.48765	62	18.68765	82	18.48765	
18.50811	64	18.50811	26	18.50811	40	18.70811	57	18.50811	
18.52857	0	18.52857	83	18.52857	14	18.72857	25	18.52857	
18.54904	0	18.54904	61	18.54904	53	18.74904	53	18.54904	
18.5695	0	18.5695	37	18.5695	34	18.7695	0	18.5695	
18.58996	2	18.58996	0	18.58996	25	18.78996	27	18.58996	
18.61042	20	18.61042	0	18.61042	68	18.81042	1	18.61042	
18.63089	8	18.63089	0	18.63089	0	18.83089	3	18.63089	
18.65135	0	18.65135	0	18.65135	0	18.85135	6	18.65135	
18.67181	8	18.67181	1	18.67181	65	18.87181	0	18.67181	
18.69227	14	18.69227	9	18.69227	0	18.89227	0	18.69227	
18.71274	27	18.71274	0	18.71274	19	18.91274	30	18.71274	
18.7332	36	18.7332	6	18.7332	40	18.9332	25	18.7332	
18.75366	0	18.75366	24	18.75366	6	18.95366	0	18.75366	
18.77413	2	18.77413	34	18.77413	0	18.97413	0	18.77413	
18.79459	0	18.79459	15	18.79459	3	18.99459	8	18.79459	
18.81505	31	18.81505	4	18.81505	0	19.01505	34	18.81505	
18.83551	0	18.83551	7	18.83551	0	19.03551	35	18.83551	
18.85598	20	18.85598	42	18.85598	0	19.05598	5	18.85598	
18.87644	5	18.87644	4	18.87644	81	19.07644	26	18.87644	
18.8969	18	18.8969	0	18.8969	11	19.0969	18	18.8969	
18.91737	35	18.91737	0	18.91737	11	19.11737	0	18.91737	
18.93783	34	18.93783	49	18.93783	54	19.13783	0	18.93783	
18.95829	23	18.95829	6	18.95829	49	19.15829	4	18.95829	
18.97875	0	18.97875	15	18.97875	14	19.17875	6	18.97875	
18.99922	0	18.99922	58	18.99922	80	19.19922	0	18.99922	
19.01968	54	19.01968	8	19.01968	35	19.21968	29	19.01968	
19.04014	55	19.04014	22	19.04014	73	19.24014	54	19.04014	
19.0606	40	19.0606	7	19.0606	47	19.2606	40	19.0606	
19.08107	71	19.08107	22	19.08107	14	19.28107	22	19.08107	
19.10153	78	19.10153	25	19.10153	116	19.30153	66	19.10153	
19.12199	96	19.12199	20	19.12199	49	19.32199	76	19.12199	
19.14246	81	19.14246	5	19.14246	106	19.34246	71	19.14246	
19.16292	125	19.16292	13	19.16292	119	19.36292	71	19.16292	
19.18338	120	19.18338	84	19.18338	180	19.38338	119	19.18338	
19.20384	177	19.20384	105	19.20384	256	19.40384	180	19.20384	
19.22431	263	19.22431	142	19.22431	257	19.42431	226	19.22431	
19.24477	370	19.24477	135	19.24477	419	19.44477	310	19.24477	
19.26523	490	19.26523	304	19.26523	573	19.46523	503	19.26523	

19.2857	713	19.2857	316	19.2857	1010	19.4857	667	19.2857	
19.30616	1080	19.30616	482	19.30616	1316	19.50616	958	19.30616	
19.32662	1390	19.32662	671	19.32662	1767	19.52662	1313	19.32662	
19.34708	1837	19.34708	962	19.34708	2155	19.54708	1702	19.34708	
19.36755	2114	19.36755	1143	19.36755	2327	19.56755	2136	19.36755	
19.38801	2052	19.38801	1446	19.38801	2062	19.58801	2413	19.38801	
19.40847	1841	19.40847	1582	19.40847	1647	19.60847	2442	19.40847	
19.42893	1344	19.42893	1558	19.42893	1207	19.62893	2436	19.42893	
19.4494	977	19.4494	1478	19.4494	754	19.6494	2112	19.4494	
19.46986	671	19.46986	1252	19.46986	481	19.66986	1704	19.46986	
19.49032	418	19.49032	866	19.49032	278	19.69032	1232	19.49032	
19.51079	248	19.51079	645	19.51079	263	19.71079	764	19.51079	
19.53125	228	19.53125	410	19.53125	170	19.73125	558	19.53125	
19.55171	144	19.55171	260	19.55171	147	19.75171	343	19.55171	
19.57217	152	19.57217	229	19.57217	127	19.77217	279	19.57217	
19.59264	79	19.59264	160	19.59264	131	19.79264	169	19.59264	
19.6131	20	19.6131	77	19.6131	57	19.8131	127	19.6131	
19.63356	120	19.63356	96	19.63356	50	19.83356	113	19.63356	
19.65403	60	19.65403	52	19.65403	76	19.85403	84	19.65403	
19.67449	17	19.67449	44	19.67449	43	19.87449	118	19.67449	
19.69495	29	19.69495	91	19.69495	42	19.89495	64	19.69495	
19.71541	0	19.71541	17	19.71541	0	19.91541	41	19.71541	
19.73588	39	19.73588	40	19.73588	14	19.93588	17	19.73588	
19.75634	37	19.75634	10	19.75634	46	19.95634	77	19.75634	
19.7768	0	19.7768	33	19.7768	44	19.9768	37	19.7768	
19.79726	0	19.79726	0	19.79726	83	19.99726	5	19.79726	
19.81773	12	19.81773	24	19.81773	45	20.01773	0	19.81773	
19.83819	11	19.83819	10	19.83819	7	20.03819	27	19.83819	
19.85865	0	19.85865	0	19.85865	0	20.05865	0	19.85865	
19.87912	3	19.87912	0	19.87912	11	20.07912	0	19.87912	
19.89958	0	19.89958	0	19.89958	0	20.09958	17	19.89958	
19.92004	8	19.92004	20	19.92004	36	20.12004	0	19.92004	
19.9405	22	19.9405	53	19.9405	2	20.1405	0	19.9405	
19.96097	36	19.96097	3	19.96097	19	20.16097	0	19.96097	
19.98143	7	19.98143	0	19.98143	28	20.18143	29	19.98143	
20.00189	32	20.00189	0	20.00189	38	20.20189	20	20.00189	
20.02235	28	20.02235	24	20.02235	0	20.22235	0	20.02235	
20.04282	0	20.04282	0	20.04282	0	20.24282	0	20.04282	
20.06328	0	20.06328	0	20.06328	12	20.26328	0	20.06328	
20.08374	2	20.08374	26	20.08374	34	20.28374	0	20.08374	
20.10421	19	20.10421	14	20.10421	29	20.30421	3	20.10421	
20.12467	0	20.12467	4	20.12467	0	20.32467	0	20.12467	
20.14513	27	20.14513	5	20.14513	10	20.34513	0	20.14513	
20.16559	29	20.16559	0	20.16559	29	20.36559	10	20.16559	
20.18606	12	20.18606	39	20.18606	32	20.38606	47	20.18606	
20.20652	0	20.20652	17	20.20652	17	20.40652	0	20.20652	
20.22698	0	20.22698	0	20.22698	13	20.42698	18	20.22698	
20.24745	5	20.24745	39	20.24745	0	20.44745	0	20.24745	
20.26791	43	20.26791	13	20.26791	23	20.46791	0	20.26791	
20.28837	0	20.28837	7	20.28837	18	20.48837	19	20.28837	
20.30883	0	20.30883	6	20.30883	1	20.50883	30	20.30883	
20.3293	4	20.3293	0	20.3293	0	20.5293	0	20.3293	
20.34976	0	20.34976	0	20.34976	6	20.54976	8	20.34976	
20.37022	5	20.37022	13	20.37022	0	20.57022	0	20.37022	
20.39068	48	20.39068	44	20.39068	4	20.59068	0	20.39068	
20.41115	27	20.41115	0	20.41115	0	20.61115	0	20.41115	
20.43161	0	20.43161	30	20.43161	0	20.63161	0	20.43161	
20.45207	3	20.45207	0	20.45207	0	20.65207	0	20.45207	

20.47254	0	20.47254	0	20.47254	0	20.67254	0	20.47254
20.493	0	20.493	0	20.493	48	20.693	23	20.493
20.51346	0	20.51346	21	20.51346	0	20.71346	0	20.51346
20.53392	0	20.53392	10	20.53392	26	20.73392	8	20.53392
20.55439	49	20.55439	3	20.55439	2	20.75439	0	20.55439
20.57485	8	20.57485	22	20.57485	0	20.77485	0	20.57485
20.59531	45	20.59531	23	20.59531	2	20.79531	22	20.59531
20.61578	0	20.61578	31	20.61578	27	20.81578	17	20.61578
20.63624	0	20.63624	0	20.63624	35	20.83624	0	20.63624
20.6567	0	20.6567	0	20.6567	12	20.8567	25	20.6567
20.67716	35	20.67716	11	20.67716	35	20.87716	0	20.67716
20.69763	8	20.69763	0	20.69763	3	20.89763	30	20.69763
20.71809	0	20.71809	0	20.71809	45	20.91809	0	20.71809
20.73855	0	20.73855	0	20.73855	23	20.93855	0	20.73855
20.75901	0	20.75901	53	20.75901	0	20.95901	0	20.75901
20.77948	0	20.77948	9	20.77948	0	20.97948	0	20.77948
20.79994	8	20.79994	69	20.79994	10	20.99994	8	20.79994
20.8204	0	20.8204	6	20.8204	9	21.0204	0	20.8204
20.84087	0	20.84087	15	20.84087	28	21.04087	0	20.84087
20.86133	1	20.86133	38	20.86133	0	21.06133	0	20.86133
20.88179	73	20.88179	5	20.88179	0	21.08179	25	20.88179
20.90225	28	20.90225	19	20.90225	54	21.10225	0	20.90225
20.92272	22	20.92272	56	20.92272	62	21.12272	36	20.92272
20.94318	25	20.94318	55	20.94318	88	21.14318	64	20.94318
20.96364	76	20.96364	22	20.96364	118	21.16364	29	20.96364
20.98411	112	20.98411	65	20.98411	112	21.18411	34	20.98411
21.00457	114	21.00457	161	21.00457	202	21.20457	40	21.00457
21.02503	187	21.02503	169	21.02503	211	21.22503	131	21.02503
21.04549	262	21.04549	226	21.04549	311	21.24549	177	21.04549
21.06596	400	21.06596	241	21.06596	456	21.26596	204	21.06596
21.08642	428	21.08642	293	21.08642	566	21.28642	287	21.08642
21.10688	468	21.10688	313	21.10688	574	21.30688	347	21.10688
21.12734	480	21.12734	377	21.12734	482	21.32734	399	21.12734
21.14781	435	21.14781	393	21.14781	374	21.34781	338	21.14781
21.16827	273	21.16827	353	21.16827	340	21.36827	344	21.16827
21.18873	209	21.18873	312	21.18873	200	21.38873	266	21.18873
21.2092	140	21.2092	262	21.2092	174	21.4092	191	21.2092
21.22966	113	21.22966	228	21.22966	168	21.42966	160	21.22966
21.25012	59	21.25012	172	21.25012	153	21.45012	141	21.25012
21.27058	25	21.27058	134	21.27058	121	21.47058	66	21.27058
21.29105	63	21.29105	118	21.29105	100	21.49105	61	21.29105
21.31151	30	21.31151	76	21.31151	29	21.51151	62	21.31151
21.33197	65	21.33197	70	21.33197	27	21.53197	26	21.33197
21.35244	48	21.35244	81	21.35244	41	21.55244	62	21.35244
21.3729	43	21.3729	134	21.3729	98	21.5729	69	21.3729
21.39336	18	21.39336	130	21.39336	36	21.59336	32	21.39336
21.41382	7	21.41382	92	21.41382	48	21.61382	43	21.41382
21.43429	57	21.43429	95	21.43429	91	21.63429	22	21.43429
21.45475	47	21.45475	135	21.45475	46	21.65475	48	21.45475
21.47521	60	21.47521	108	21.47521	116	21.67521	55	21.47521
21.49567	66	21.49567	114	21.49567	51	21.69567	34	21.49567
21.51614	42	21.51614	129	21.51614	35	21.71614	35	21.51614
21.5366	37	21.5366	109	21.5366	109	21.7366	71	21.5366
21.55706	46	21.55706	137	21.55706	33	21.75706	2	21.55706
21.57753	36	21.57753	122	21.57753	80	21.77753	22	21.57753
21.59799	48	21.59799	90	21.59799	104	21.79799	0	21.59799
21.61845	73	21.61845	150	21.61845	156	21.81845	37	21.61845
21.63891	81	21.63891	189	21.63891	132	21.83891	79	21.63891

21.65938	97	21.65938	147	21.65938	233	21.85938	56	21.65938
21.67984	106	21.67984	185	21.67984	152	21.87984	51	21.67984
21.7003	88	21.7003	207	21.7003	191	21.9003	104	21.7003
21.72077	145	21.72077	199	21.72077	233	21.92077	84	21.72077
21.74123	161	21.74123	243	21.74123	224	21.94123	132	21.74123
21.76169	127	21.76169	231	21.76169	257	21.96169	143	21.76169
21.78215	171	21.78215	244	21.78215	281	21.98215	108	21.78215
21.80262	214	21.80262	292	21.80262	285	22.00262	134	21.80262
21.82308	209	21.82308	293	21.82308	295	22.02308	164	21.82308
21.84354	280	21.84354	268	21.84354	312	22.04354	229	21.84354
21.864	283	21.864	367	21.864	363	22.064	191	21.864
21.88447	347	21.88447	421	21.88447	440	22.08447	324	21.88447
21.90493	358	21.90493	426	21.90493	541	22.10493	355	21.90493
21.92539	464	21.92539	490	21.92539	687	22.12539	386	21.92539
21.94586	633	21.94586	568	21.94586	834	22.14586	478	21.94586
21.96632	794	21.96632	730	21.96632	1135	22.16632	648	21.96632
21.98678	1076	21.98678	902	21.98678	1665	22.18678	901	21.98678
22.00724	1623	22.00724	1279	22.00724	2166	22.20724	1294	22.00724
22.02771	2120	22.02771	1588	22.02771	3019	22.22771	1848	22.02771
22.04817	2992	22.04817	2116	22.04817	3617	22.24817	2469	22.04817
22.06863	3457	22.06863	2533	22.06863	4009	22.26863	2967	22.06863
22.0891	3353	22.0891	2706	22.0891	3659	22.2891	3374	22.0891
22.10956	3080	22.10956	3090	22.10956	3248	22.30956	3542	22.10956
22.13002	2540	22.13002	3016	22.13002	2606	22.33002	3437	22.13002
22.15048	1970	22.15048	2673	22.15048	2004	22.35048	2880	22.15048
22.17095	1640	22.17095	2393	22.17095	1530	22.37095	2396	22.17095
22.19141	1276	22.19141	1974	22.19141	1377	22.39141	1901	22.19141
22.21187	1127	22.21187	1698	22.21187	1387	22.41187	1397	22.21187
22.23233	1173	22.23233	1481	22.23233	1398	22.43233	1314	22.23233
22.2528	1363	22.2528	1472	22.2528	1838	22.4528	1322	22.2528
22.27326	1662	22.27326	1595	22.27326	2246	22.47326	1392	22.27326
22.29372	2107	22.29372	1817	22.29372	3043	22.49372	1851	22.29372
22.31419	2871	22.31419	2280	22.31419	4263	22.51419	2328	22.31419
22.33465	3725	22.33465	2854	22.33465	5867	22.53465	3147	22.33465
22.35511	5017	22.35511	3791	22.35511	7530	22.55511	4181	22.35511
22.37557	6403	22.37557	4487	22.37557	8968	22.57557	5310	22.37557
22.39604	7151	22.39604	5214	22.39604	8726	22.59604	6275	22.39604
22.4165	6885	22.4165	5809	22.4165	7692	22.6165	6777	22.4165
22.43696	6124	22.43696	5942	22.43696	6339	22.63696	6934	22.43696
22.45743	4915	22.45743	5702	22.45743	4729	22.65743	6158	22.45743
22.47789	3649	22.47789	4839	22.47789	3422	22.67789	5201	22.47789
22.49835	2477	22.49835	4018	22.49835	2432	22.69835	4116	22.49835
22.51881	1779	22.51881	3087	22.51881	1661	22.71881	2916	22.51881
22.53928	1330	22.53928	2547	22.53928	1389	22.73928	2029	22.53928
22.55974	1072	22.55974	1944	22.55974	1096	22.75974	1401	22.55974
22.5802	847	22.5802	1541	22.5802	1006	22.7802	1023	22.5802
22.60066	677	22.60066	1369	22.60066	867	22.80066	735	22.60066
22.62113	601	22.62113	1209	22.62113	768	22.82113	580	22.62113
22.64159	554	22.64159	1182	22.64159	854	22.84159	556	22.64159
22.66205	467	22.66205	1063	22.66205	736	22.86205	437	22.66205
22.68252	445	22.68252	1035	22.68252	693	22.88252	343	22.68252
22.70298	383	22.70298	948	22.70298	717	22.90298	309	22.70298
22.72344	311	22.72344	938	22.72344	616	22.92344	231	22.72344
22.7439	365	22.7439	925	22.7439	622	22.9439	229	22.7439
22.76437	289	22.76437	897	22.76437	542	22.96437	180	22.76437
22.78483	318	22.78483	767	22.78483	550	22.98483	194	22.78483
22.80529	295	22.80529	770	22.80529	555	23.00529	163	22.80529
22.82576	297	22.82576	832	22.82576	615	23.02576	147	22.82576

22.84622	274	22.84622	770	22.84622	514	23.04622	125	22.84622
22.86668	256	22.86668	749	22.86668	517	23.06668	106	22.86668
22.88714	246	22.88714	747	22.88714	462	23.08714	95	22.88714
22.90761	187	22.90761	710	22.90761	453	23.10761	111	22.90761
22.92807	172	22.92807	739	22.92807	481	23.12807	87	22.92807
22.94853	205	22.94853	654	22.94853	457	23.14853	83	22.94853
22.96899	164	22.96899	605	22.96899	452	23.16899	82	22.96899
22.98946	178	22.98946	585	22.98946	341	23.18946	73	22.98946
23.00992	182	23.00992	598	23.00992	427	23.20992	41	23.00992
23.03038	239	23.03038	610	23.03038	344	23.23038	26	23.03038
23.05085	139	23.05085	576	23.05085	355	23.25085	53	23.05085
23.07131	131	23.07131	579	23.07131	311	23.27131	23	23.07131
23.09177	86	23.09177	573	23.09177	283	23.29177	69	23.09177
23.11223	134	23.11223	522	23.11223	335	23.31223	37	23.11223
23.1327	135	23.1327	428	23.1327	267	23.3327	21	23.1327
23.15316	119	23.15316	473	23.15316	268	23.35316	82	23.15316
23.17362	66	23.17362	366	23.17362	307	23.37362	37	23.17362
23.19409	109	23.19409	457	23.19409	238	23.39409	0	23.19409
23.21455	85	23.21455	415	23.21455	204	23.41455	0	23.21455
23.23501	84	23.23501	364	23.23501	222	23.43501	4	23.23501
23.25547	69	23.25547	370	23.25547	244	23.45547	17	23.25547
23.27594	65	23.27594	396	23.27594	191	23.47594	16	23.27594
23.2964	62	23.2964	274	23.2964	203	23.4964	50	23.2964
23.31686	35	23.31686	306	23.31686	195	23.51686	0	23.31686
23.33732	43	23.33732	309	23.33732	187	23.53732	10	23.33732
23.35779	39	23.35779	269	23.35779	114	23.55779	0	23.35779
23.37825	19	23.37825	222	23.37825	112	23.57825	22	23.37825
23.39871	39	23.39871	239	23.39871	124	23.59871	0	23.39871
23.41918	20	23.41918	267	23.41918	91	23.61918	0	23.41918
23.43964	22	23.43964	206	23.43964	95	23.63964	0	23.43964
23.4601	8	23.4601	214	23.4601	107	23.6601	25	23.4601
23.48056	43	23.48056	206	23.48056	137	23.68056	4	23.48056
23.50103	41	23.50103	166	23.50103	149	23.70103	44	23.50103
23.52149	31	23.52149	181	23.52149	162	23.72149	71	23.52149
23.54195	109	23.54195	217	23.54195	193	23.74195	34	23.54195
23.56242	172	23.56242	180	23.56242	272	23.76242	50	23.56242
23.58288	138	23.58288	175	23.58288	283	23.78288	62	23.58288
23.60334	221	23.60334	186	23.60334	315	23.80334	143	23.60334
23.6238	225	23.6238	306	23.6238	340	23.8238	164	23.6238
23.64427	217	23.64427	261	23.64427	303	23.84427	109	23.64427
23.66473	170	23.66473	273	23.66473	255	23.86473	216	23.66473
23.68519	117	23.68519	271	23.68519	204	23.88519	185	23.68519
23.70565	126	23.70565	206	23.70565	78	23.90565	119	23.70565
23.72612	92	23.72612	162	23.72612	104	23.92612	56	23.72612
23.74658	57	23.74658	146	23.74658	28	23.94658	54	23.74658
23.76704	8	23.76704	92	23.76704	82	23.96704	22	23.76704
23.78751	41	23.78751	33	23.78751	13	23.98751	0	23.78751
23.80797	25	23.80797	98	23.80797	55	24.00797	21	23.80797
23.82843	0	23.82843	63	23.82843	34	24.02843	7	23.82843
23.84889	0	23.84889	47	23.84889	0	24.04889	0	23.84889
23.86936	0	23.86936	32	23.86936	16	24.06936	0	23.86936
23.88982	0	23.88982	82	23.88982	38	24.08982	0	23.88982
23.91028	0	23.91028	136	23.91028	49	24.11028	0	23.91028
23.93075	0	23.93075	54	23.93075	6	24.13075	53	23.93075
23.95121	4	23.95121	38	23.95121	0	24.15121	0	23.95121
23.97167	0	23.97167	24	23.97167	35	24.17167	39	23.97167
23.99213	0	23.99213	39	23.99213	0	24.19213	24	23.99213
24.0126	31	24.0126	69	24.0126	29	24.2126	26	24.0126

24.03306	3	24.03306	32	24.03306	76	24.23306	10	24.03306	
24.05352	0	24.05352	35	24.05352	0	24.25352	24	24.05352	
24.07398	23	24.07398	68	24.07398	18	24.27398	51	24.07398	
24.09445	39	24.09445	61	24.09445	41	24.29445	9	24.09445	
24.11491	66	24.11491	38	24.11491	30	24.31491	25	24.11491	
24.13537	53	24.13537	73	24.13537	50	24.33537	70	24.13537	
24.15584	55	24.15584	127	24.15584	51	24.35584	30	24.15584	
24.1763	72	24.1763	70	24.1763	71	24.3763	56	24.1763	
24.19676	63	24.19676	54	24.19676	122	24.39676	29	24.19676	
24.21722	102	24.21722	120	24.21722	150	24.41722	97	24.21722	
24.23769	75	24.23769	137	24.23769	137	24.43769	135	24.23769	
24.25815	156	24.25815	142	24.25815	182	24.45815	134	24.25815	
24.27861	151	24.27861	101	24.27861	197	24.47861	98	24.27861	
24.29908	229	24.29908	185	24.29908	240	24.49908	200	24.29908	
24.31954	305	24.31954	223	24.31954	293	24.51954	250	24.31954	
24.34	410	24.34	289	24.34	529	24.54	251	24.34	
24.36046	603	24.36046	374	24.36046	713	24.56046	375	24.36046	
24.38093	804	24.38093	562	24.38093	1031	24.58093	413	24.38093	
24.40139	1164	24.40139	798	24.40139	1606	24.60139	705	24.40139	
24.42185	1708	24.42185	1163	24.42185	2294	24.62185	1013	24.42185	
24.44231	2377	24.44231	1593	24.44231	3261	24.64231	1548	24.44231	
24.46278	3347	24.46278	2235	24.46278	4292	24.66278	2018	24.46278	
24.48324	3824	24.48324	2797	24.48324	4527	24.68324	2566	24.48324	
24.5037	3925	24.5037	3275	24.5037	4190	24.7037	2947	24.5037	
24.52417	3489	24.52417	3539	24.52417	3570	24.72417	3273	24.52417	
24.54463	2960	24.54463	3401	24.54463	2883	24.74463	3090	24.54463	
24.56509	2352	24.56509	3033	24.56509	1975	24.76509	2795	24.56509	
24.58555	1629	24.58555	2598	24.58555	1398	24.78555	2217	24.58555	
24.60602	1190	24.60602	1974	24.60602	910	24.80602	1863	24.60602	
24.62648	764	24.62648	1547	24.62648	626	24.82648	1217	24.62648	
24.64694	509	24.64694	1045	24.64694	350	24.84694	869	24.64694	
24.66741	419	24.66741	690	24.66741	274	24.86741	553	24.66741	
24.68787	324	24.68787	476	24.68787	217	24.88787	381	24.68787	
24.70833	276	24.70833	358	24.70833	206	24.90833	313	24.70833	
24.72879	126	24.72879	325	24.72879	187	24.92879	198	24.72879	
24.74926	119	24.74926	244	24.74926	167	24.94926	192	24.74926	
24.76972	143	24.76972	150	24.76972	129	24.96972	143	24.76972	
24.79018	92	24.79018	132	24.79018	47	24.99018	110	24.79018	
24.81064	91	24.81064	156	24.81064	38	25.01064	98	24.81064	
24.83111	49	24.83111	109	24.83111	83	25.03111	122	24.83111	
24.85157	56	24.85157	80	24.85157	22	25.05157	42	24.85157	
24.87203	20	24.87203	51	24.87203	8	25.07203	90	24.87203	
24.8925	14	24.8925	46	24.8925	16	25.0925	33	24.8925	
24.91296	67	24.91296	20	24.91296	5	25.11296	28	24.91296	
24.93342	22	24.93342	86	24.93342	60	25.13342	42	24.93342	
24.95388	2	24.95388	26	24.95388	0	25.15388	0	24.95388	
24.97435	58	24.97435	0	24.97435	0	25.17435	2	24.97435	
24.99481	31	24.99481	42	24.99481	35	25.19481	28	24.99481	
25.01527	0	25.01527	52	25.01527	0	25.21527	16	25.01527	
25.03574	24	25.03574	57	25.03574	44	25.23574	9	25.03574	
25.0562	22	25.0562	51	25.0562	53	25.2562	13	25.0562	
25.07666	71	25.07666	5	25.07666	28	25.27666	16	25.07666	
25.09712	0	25.09712	32	25.09712	52	25.29712	27	25.09712	
25.11759	22	25.11759	7	25.11759	21	25.31759	20	25.11759	
25.13805	0	25.13805	16	25.13805	18	25.33805	0	25.13805	
25.15851	2	25.15851	57	25.15851	0	25.35851	3	25.15851	
25.17897	0	25.17897	49	25.17897	1	25.37897	0	25.17897	
25.19944	0	25.19944	114	25.19944	38	25.39944	0	25.19944	

25.2199	12	25.2199	0	25.2199	11	25.4199	20	25.2199
25.24036	8	25.24036	21	25.24036	17	25.44036	55	25.24036
25.26083	6	25.26083	9	25.26083	0	25.46083	42	25.26083
25.28129	0	25.28129	11	25.28129	0	25.48129	64	25.28129
25.30175	0	25.30175	0	25.30175	12	25.50175	36	25.30175
25.32221	0	25.32221	0	25.32221	26	25.52221	0	25.32221
25.34268	0	25.34268	0	25.34268	0	25.54268	4	25.34268
25.36314	17	25.36314	22	25.36314	0	25.56314	12	25.36314
25.3836	0	25.3836	2	25.3836	6	25.5836	0	25.3836
25.40407	24	25.40407	74	25.40407	27	25.60407	0	25.40407
25.42453	7	25.42453	25	25.42453	15	25.62453	27	25.42453
25.44499	2	25.44499	41	25.44499	8	25.64499	34	25.44499
25.46545	5	25.46545	21	25.46545	0	25.66545	38	25.46545
25.48592	11	25.48592	53	25.48592	22	25.68592	0	25.48592
25.50638	39	25.50638	40	25.50638	34	25.70638	26	25.50638
25.52684	0	25.52684	58	25.52684	30	25.72684	13	25.52684
25.5473	23	25.5473	10	25.5473	43	25.7473	6	25.5473
25.56777	20	25.56777	17	25.56777	30	25.76777	0	25.56777
25.58823	10	25.58823	33	25.58823	40	25.78823	48	25.58823
25.60869	84	25.60869	40	25.60869	55	25.80869	46	25.60869
25.62916	48	25.62916	43	25.62916	37	25.82916	98	25.62916
25.64962	86	25.64962	55	25.64962	59	25.84962	74	25.64962
25.67008	64	25.67008	113	25.67008	74	25.87008	130	25.67008
25.69054	80	25.69054	114	25.69054	112	25.89054	109	25.69054
25.71101	114	25.71101	111	25.71101	123	25.91101	199	25.71101
25.73147	117	25.73147	193	25.73147	213	25.93147	215	25.73147
25.75193	222	25.75193	187	25.75193	159	25.95193	362	25.75193
25.7724	341	25.7724	322	25.7724	406	25.9724	446	25.7724
25.79286	490	25.79286	479	25.79286	637	25.99286	603	25.79286
25.81332	670	25.81332	576	25.81332	959	26.01332	834	25.81332
25.83378	942	25.83378	782	25.83378	1368	26.03378	1123	25.83378
25.85425	1345	25.85425	1095	25.85425	1812	26.05425	1562	25.85425
25.87471	1684	25.87471	1401	25.87471	2373	26.07471	1909	25.87471
25.89517	1939	25.89517	1876	25.89517	2550	26.09517	2336	25.89517
25.91563	1735	25.91563	2073	25.91563	2211	26.11563	2574	25.91563
25.9361	1508	25.9361	2147	25.9361	1868	26.1361	2557	25.9361
25.95656	1255	25.95656	1955	25.95656	1525	26.15656	2443	25.95656
25.97702	1001	25.97702	1800	25.97702	1056	26.17702	2107	25.97702
25.99749	731	25.99749	1476	25.99749	706	26.19749	1719	25.99749
26.01795	472	26.01795	1191	26.01795	487	26.21795	1225	26.01795
26.03841	340	26.03841	812	26.03841	320	26.23841	949	26.03841
26.05887	261	26.05887	574	26.05887	224	26.25887	646	26.05887
26.07934	174	26.07934	406	26.07934	172	26.27934	452	26.07934
26.0998	169	26.0998	269	26.0998	120	26.2998	343	26.0998
26.12026	122	26.12026	251	26.12026	152	26.32026	249	26.12026
26.14073	70	26.14073	187	26.14073	93	26.34073	203	26.14073
26.16119	91	26.16119	159	26.16119	73	26.36119	187	26.16119
26.18165	49	26.18165	190	26.18165	75	26.38165	191	26.18165
26.20211	77	26.20211	139	26.20211	90	26.40211	125	26.20211
26.22258	68	26.22258	117	26.22258	48	26.42258	87	26.22258
26.24304	78	26.24304	77	26.24304	78	26.44304	126	26.24304
26.2635	61	26.2635	99	26.2635	57	26.4635	147	26.2635
26.28396	69	26.28396	99	26.28396	32	26.48396	111	26.28396
26.30443	94	26.30443	73	26.30443	83	26.50443	100	26.30443
26.32489	49	26.32489	90	26.32489	62	26.52489	79	26.32489
26.34535	81	26.34535	84	26.34535	126	26.54535	102	26.34535
26.36582	129	26.36582	108	26.36582	107	26.56582	150	26.36582
26.38628	131	26.38628	77	26.38628	135	26.58628	121	26.38628

26.40674	120	26.40674	102	26.40674	131	26.60674	166	26.40674
26.4272	157	26.4272	152	26.4272	192	26.6272	193	26.4272
26.44767	129	26.44767	105	26.44767	205	26.64767	267	26.44767
26.46813	242	26.46813	237	26.46813	211	26.66813	235	26.46813
26.48859	294	26.48859	236	26.48859	344	26.68859	257	26.48859
26.50906	385	26.50906	332	26.50906	381	26.70906	353	26.50906
26.52952	476	26.52952	391	26.52952	524	26.72952	464	26.52952
26.54998	583	26.54998	483	26.54998	633	26.74998	552	26.54998
26.57044	938	26.57044	602	26.57044	951	26.77044	742	26.57044
26.59091	1287	26.59091	809	26.59091	1432	26.79091	1010	26.59091
26.61137	1802	26.61137	1280	26.61137	2032	26.81137	1389	26.61137
26.63183	2455	26.63183	1647	26.63183	3018	26.83183	1976	26.63183
26.65229	3467	26.65229	2274	26.65229	4268	26.85229	2646	26.65229
26.67276	4287	26.67276	2956	26.67276	5514	26.87276	3633	26.67276
26.69322	4963	26.69322	3771	26.69322	6086	26.89322	4637	26.69322
26.71368	5039	26.71368	4349	26.71368	5667	26.91368	5324	26.71368
26.73415	4618	26.73415	4702	26.73415	4649	26.93415	5703	26.73415
26.75461	3974	26.75461	4539	26.75461	4146	26.95461	5528	26.75461
26.77507	3201	26.77507	4015	26.77507	3050	26.97507	4796	26.77507
26.79553	2427	26.79553	3388	26.79553	2022	26.99553	4058	26.79553
26.816	1760	26.816	2594	26.816	1319	27.016	3120	26.816
26.83646	1234	26.83646	1907	26.83646	853	27.03646	2304	26.83646
26.85692	860	26.85692	1405	26.85692	627	27.05692	1651	26.85692
26.87739	667	26.87739	984	26.87739	493	27.07739	1160	26.87739
26.89785	496	26.89785	717	26.89785	389	27.09785	884	26.89785
26.91831	416	26.91831	584	26.91831	342	27.11831	668	26.91831
26.93877	434	26.93877	433	26.93877	283	27.13877	568	26.93877
26.95924	301	26.95924	366	26.95924	291	27.15924	467	26.95924
26.9797	314	26.9797	325	26.9797	242	27.1797	361	26.9797
27.00016	224	27.00016	316	27.00016	159	27.20016	380	27.00016
27.02062	180	27.02062	264	27.02062	200	27.22062	323	27.02062
27.04109	149	27.04109	212	27.04109	145	27.24109	236	27.04109
27.06155	131	27.06155	170	27.06155	102	27.26155	227	27.06155
27.08201	80	27.08201	124	27.08201	35	27.28201	231	27.08201
27.10248	93	27.10248	49	27.10248	44	27.30248	118	27.10248
27.12294	7	27.12294	34	27.12294	48	27.32294	83	27.12294
27.1434	26	27.1434	66	27.1434	1	27.3434	108	27.1434
27.16386	0	27.16386	64	27.16386	0	27.36386	75	27.16386
27.18433	43	27.18433	56	27.18433	0	27.38433	69	27.18433
27.20479	0	27.20479	19	27.20479	11	27.40479	47	27.20479
27.22525	3	27.22525	0	27.22525	15	27.42525	61	27.22525
27.24572	23	27.24572	12	27.24572	3	27.44572	44	27.24572
27.26618	0	27.26618	14	27.26618	58	27.46618	52	27.26618
27.28664	12	27.28664	15	27.28664	19	27.48664	53	27.28664
27.3071	0	27.3071	6	27.3071	9	27.5071	5	27.3071
27.32757	0	27.32757	87	27.32757	0	27.52757	70	27.32757
27.34803	0	27.34803	16	27.34803	24	27.54803	13	27.34803
27.36849	8	27.36849	31	27.36849	0	27.56849	42	27.36849
27.38895	0	27.38895	0	27.38895	0	27.58895	24	27.38895
27.40942	0	27.40942	6	27.40942	0	27.60942	3	27.40942
27.42988	0	27.42988	0	27.42988	19	27.62988	6	27.42988
27.45034	10	27.45034	9	27.45034	20	27.65034	46	27.45034
27.47081	0	27.47081	0	27.47081	25	27.67081	23	27.47081
27.49127	35	27.49127	0	27.49127	0	27.69127	0	27.49127
27.51173	39	27.51173	0	27.51173	0	27.71173	0	27.51173
27.53219	8	27.53219	0	27.53219	43	27.73219	0	27.53219
27.55266	0	27.55266	0	27.55266	12	27.75266	6	27.55266
27.57312	15	27.57312	60	27.57312	0	27.77312	0	27.57312

27.59358	8	27.59358	0	27.59358	14	27.79358	20	27.59358
27.61405	0	27.61405	0	27.61405	42	27.81405	17	27.61405
27.63451	10	27.63451	0	27.63451	0	27.83451	51	27.63451
27.65497	41	27.65497	60	27.65497	17	27.85497	52	27.65497
27.67543	0	27.67543	27	27.67543	64	27.87543	16	27.67543
27.6959	17	27.6959	53	27.6959	32	27.8959	39	27.6959
27.71636	44	27.71636	34	27.71636	40	27.91636	55	27.71636
27.73682	117	27.73682	67	27.73682	129	27.93682	67	27.73682
27.75728	90	27.75728	72	27.75728	121	27.95728	89	27.75728
27.77775	143	27.77775	43	27.77775	147	27.97775	122	27.77775
27.79821	180	27.79821	148	27.79821	238	27.99821	209	27.79821
27.81867	213	27.81867	195	27.81867	222	28.01867	232	27.81867
27.83914	284	27.83914	236	27.83914	403	28.03914	287	27.83914
27.8596	415	27.8596	346	27.8596	426	28.0596	304	27.8596
27.88006	557	27.88006	410	27.88006	583	28.08006	419	27.88006
27.90052	775	27.90052	519	27.90052	902	28.10052	646	27.90052
27.92099	1137	27.92099	751	27.92099	1506	28.12099	855	27.92099
27.94145	1545	27.94145	1105	27.94145	1978	28.14145	1248	27.94145
27.96191	2252	27.96191	1429	27.96191	2763	28.16191	1735	27.96191
27.98238	2722	27.98238	1957	27.98238	3293	28.18238	2188	27.98238
28.00284	2965	28.00284	2246	28.00284	3119	28.20284	2579	28.00284
28.0233	2778	28.0233	2619	28.0233	2748	28.2233	2829	28.0233
28.04376	2397	28.04376	2666	28.04376	2526	28.24376	2913	28.04376
28.06423	2043	28.06423	2426	28.06423	1916	28.26423	2581	28.06423
28.08469	1720	28.08469	1982	28.08469	1417	28.28469	2193	28.08469
28.10515	1145	28.10515	1648	28.10515	978	28.30515	1730	28.10515
28.12561	842	28.12561	1312	28.12561	582	28.32561	1408	28.12561
28.14608	626	28.14608	925	28.14608	453	28.34608	1039	28.14608
28.16654	392	28.16654	684	28.16654	311	28.36654	723	28.16654
28.187	303	28.187	490	28.187	268	28.387	483	28.187
28.20747	261	28.20747	372	28.20747	180	28.40747	356	28.20747
28.22793	198	28.22793	325	28.22793	149	28.42793	312	28.22793
28.24839	155	28.24839	204	28.24839	143	28.44839	240	28.24839
28.26885	141	28.26885	173	28.26885	172	28.46885	170	28.26885
28.28932	135	28.28932	114	28.28932	111	28.48932	141	28.28932
28.30978	101	28.30978	146	28.30978	117	28.50978	141	28.30978
28.33024	93	28.33024	56	28.33024	76	28.53024	146	28.33024
28.35071	97	28.35071	77	28.35071	86	28.55071	72	28.35071
28.37117	107	28.37117	100	28.37117	67	28.57117	79	28.37117
28.39163	80	28.39163	27	28.39163	99	28.59163	87	28.39163
28.41209	109	28.41209	89	28.41209	59	28.61209	52	28.41209
28.43256	97	28.43256	110	28.43256	91	28.63256	159	28.43256
28.45302	135	28.45302	72	28.45302	105	28.65302	124	28.45302
28.47348	125	28.47348	103	28.47348	172	28.67348	125	28.47348
28.49394	106	28.49394	96	28.49394	123	28.69394	133	28.49394
28.51441	193	28.51441	91	28.51441	186	28.71441	155	28.51441
28.53487	270	28.53487	141	28.53487	289	28.73487	184	28.53487
28.55533	312	28.55533	160	28.55533	278	28.75533	211	28.55533
28.5758	378	28.5758	301	28.5758	412	28.7758	302	28.5758
28.59626	450	28.59626	335	28.59626	515	28.79626	331	28.59626
28.61672	679	28.61672	480	28.61672	735	28.81672	416	28.61672
28.63718	822	28.63718	577	28.63718	1008	28.83718	611	28.63718
28.65765	1201	28.65765	837	28.65765	1396	28.85765	907	28.65765
28.67811	1703	28.67811	1232	28.67811	2168	28.87811	1238	28.67811
28.69857	2487	28.69857	1738	28.69857	3295	28.89857	1831	28.69857
28.71904	3388	28.71904	2460	28.71904	4315	28.91904	2662	28.71904
28.7395	4066	28.7395	3146	28.7395	5034	28.9395	3611	28.7395
28.75996	4188	28.75996	3661	28.75996	4880	28.95996	4345	28.75996

28.78042	4168	28.78042	3970	28.78042	4124	28.98042	4678	28.78042
28.80089	3487	28.80089	3888	28.80089	3678	29.00089	4657	28.80089
28.82135	2988	28.82135	3495	28.82135	3122	29.02135	4096	28.82135
28.84181	2654	28.84181	2941	28.84181	2147	29.04181	3496	28.84181
28.86227	1807	28.86227	2384	28.86227	1487	29.06227	2777	28.86227
28.88274	1179	28.88274	1737	28.88274	995	29.08274	2042	28.88274
28.9032	855	28.9032	1273	28.9032	761	29.1032	1557	28.9032
28.92366	606	28.92366	907	28.92366	510	29.12366	1079	28.92366
28.94413	413	28.94413	600	28.94413	341	29.14413	712	28.94413
28.96459	311	28.96459	465	28.96459	283	29.16459	489	28.96459
28.98505	279	28.98505	362	28.98505	232	29.18505	352	28.98505
29.00551	247	29.00551	344	29.00551	146	29.20551	284	29.00551
29.02598	148	29.02598	239	29.02598	129	29.22598	223	29.02598
29.04644	184	29.04644	181	29.04644	134	29.24644	195	29.04644
29.0669	117	29.0669	142	29.0669	82	29.2669	163	29.0669
29.08737	101	29.08737	81	29.08737	48	29.28737	98	29.08737
29.10783	62	29.10783	107	29.10783	56	29.30783	104	29.10783
29.12829	58	29.12829	79	29.12829	27	29.32829	123	29.12829
29.14875	57	29.14875	47	29.14875	58	29.34875	14	29.14875
29.16922	83	29.16922	23	29.16922	4	29.36922	64	29.16922
29.18968	49	29.18968	53	29.18968	23	29.38968	102	29.18968
29.21014	41	29.21014	70	29.21014	13	29.41014	20	29.21014
29.2306	77	29.2306	25	29.2306	26	29.4306	61	29.2306
29.25107	56	29.25107	3	29.25107	0	29.45107	0	29.25107
29.27153	17	29.27153	39	29.27153	50	29.47153	43	29.27153
29.29199	0	29.29199	39	29.29199	26	29.49199	17	29.29199
29.31246	0	29.31246	3	29.31246	0	29.51246	0	29.31246
29.33292	35	29.33292	13	29.33292	25	29.53292	52	29.33292
29.35338	0	29.35338	52	29.35338	24	29.55338	0	29.35338
29.37384	32	29.37384	53	29.37384	24	29.57384	0	29.37384
29.39431	41	29.39431	0	29.39431	59	29.59431	15	29.39431
29.41477	27	29.41477	0	29.41477	0	29.61477	36	29.41477
29.43523	0	29.43523	10	29.43523	0	29.63523	0	29.43523
29.4557	34	29.4557	0	29.4557	20	29.6557	0	29.4557
29.47616	15	29.47616	0	29.47616	48	29.67616	26	29.47616
29.49662	0	29.49662	1	29.49662	30	29.69662	0	29.49662
29.51708	0	29.51708	3	29.51708	10	29.71708	0	29.51708
29.53755	30	29.53755	0	29.53755	0	29.73755	0	29.53755
29.55801	1	29.55801	9	29.55801	0	29.75801	6	29.55801
29.57847	10	29.57847	0	29.57847	0	29.77847	35	29.57847
29.59893	10	29.59893	43	29.59893	24	29.79893	0	29.59893
29.6194	41	29.6194	0	29.6194	26	29.8194	17	29.6194
29.63986	19	29.63986	7	29.63986	35	29.83986	10	29.63986
29.66032	17	29.66032	36	29.66032	27	29.86032	17	29.66032
29.68079	35	29.68079	61	29.68079	69	29.88079	3	29.68079
29.70125	52	29.70125	41	29.70125	62	29.90125	58	29.70125
29.72171	66	29.72171	65	29.72171	116	29.92171	9	29.72171
29.74217	113	29.74217	62	29.74217	75	29.94217	76	29.74217
29.76264	94	29.76264	63	29.76264	108	29.96264	111	29.76264
29.7831	160	29.7831	174	29.7831	205	29.9831	96	29.7831
29.80356	281	29.80356	110	29.80356	298	30.00356	189	29.80356
29.82403	348	29.82403	171	29.82403	321	30.02403	223	29.82403
29.84449	458	29.84449	323	29.84449	479	30.04449	351	29.84449
29.86495	662	29.86495	421	29.86495	737	30.06495	489	29.86495
29.88541	971	29.88541	546	29.88541	1055	30.08541	680	29.88541
29.90588	1290	29.90588	756	29.90588	1722	30.10588	978	29.90588
29.92634	1810	29.92634	1122	29.92634	2536	30.12634	1405	29.92634
29.9468	2247	29.9468	1437	29.9468	3323	30.1468	1763	29.9468

29.96726	2455	29.96726	1521	29.96726	3380	30.16726	2230	29.96726
29.98773	2509	29.98773	1770	29.98773	2876	30.18773	2546	29.98773
30.00819	2243	30.00819	1734	30.00819	2432	30.20819	2465	30.00819
30.02865	1880	30.02865	1613	30.02865	2206	30.22865	2274	30.02865
30.04912	1666	30.04912	1522	30.04912	1750	30.24912	1975	30.04912
30.06958	1265	30.06958	1202	30.06958	1071	30.26958	1561	30.06958
30.09004	904	30.09004	984	30.09004	711	30.29004	1285	30.09004
30.1105	522	30.1105	708	30.1105	417	30.3105	861	30.1105
30.13097	401	30.13097	506	30.13097	268	30.33097	590	30.13097
30.15143	309	30.15143	408	30.15143	197	30.35143	431	30.15143
30.17189	206	30.17189	228	30.17189	161	30.37189	287	30.17189
30.19236	142	30.19236	248	30.19236	163	30.39236	228	30.19236
30.21282	156	30.21282	148	30.21282	79	30.41282	179	30.21282
30.23328	107	30.23328	113	30.23328	69	30.43328	165	30.23328
30.25374	104	30.25374	104	30.25374	77	30.45374	70	30.25374
30.27421	131	30.27421	88	30.27421	83	30.47421	46	30.27421
30.29467	41	30.29467	126	30.29467	83	30.49467	73	30.29467
30.31513	66	30.31513	52	30.31513	73	30.51513	63	30.31513
30.33559	77	30.33559	76	30.33559	57	30.53559	57	30.33559
30.35606	24	30.35606	57	30.35606	16	30.55606	61	30.35606
30.37652	35	30.37652	49	30.37652	52	30.57652	76	30.37652
30.39698	38	30.39698	7	30.39698	51	30.59698	34	30.39698
30.41745	93	30.41745	24	30.41745	69	30.61745	91	30.41745
30.43791	26	30.43791	36	30.43791	47	30.63791	77	30.43791
30.45837	50	30.45837	68	30.45837	70	30.65837	77	30.45837
30.47883	60	30.47883	72	30.47883	70	30.67883	70	30.47883
30.4993	116	30.4993	44	30.4993	106	30.6993	88	30.4993
30.51976	113	30.51976	67	30.51976	117	30.71976	109	30.51976
30.54022	149	30.54022	142	30.54022	98	30.74022	114	30.54022
30.56068	190	30.56068	104	30.56068	158	30.76068	127	30.56068
30.58115	173	30.58115	163	30.58115	192	30.78115	158	30.58115
30.60161	264	30.60161	236	30.60161	338	30.80161	218	30.60161
30.62207	364	30.62207	250	30.62207	398	30.82207	317	30.62207
30.64254	452	30.64254	288	30.64254	541	30.84254	454	30.64254
30.663	635	30.663	400	30.663	630	30.863	548	30.663
30.68346	627	30.68346	524	30.68346	672	30.88346	646	30.68346
30.70392	627	30.70392	563	30.70392	637	30.90392	710	30.70392
30.72439	654	30.72439	603	30.72439	700	30.92439	687	30.72439
30.74485	663	30.74485	700	30.74485	734	30.94485	810	30.74485
30.76531	809	30.76531	724	30.76531	802	30.96531	861	30.76531
30.78578	906	30.78578	819	30.78578	1020	30.98578	889	30.78578
30.80624	1152	30.80624	980	30.80624	1459	31.00624	1003	30.80624
30.8267	1629	30.8267	1308	30.8267	1938	31.0267	1413	30.8267
30.84716	2137	30.84716	1760	30.84716	2835	31.04716	1907	30.84716
30.86763	2951	30.86763	2201	30.86763	3725	31.06763	2522	30.86763
30.88809	3633	30.88809	2957	30.88809	4436	31.08809	3464	30.88809
30.90855	3921	30.90855	3632	30.90855	4489	31.10855	4221	30.90855
30.92901	3818	30.92901	4151	30.92901	4018	31.12901	4832	30.92901
30.94948	3342	30.94948	4155	30.94948	3470	31.14948	4708	30.94948
30.96994	3077	30.96994	4027	30.96994	3070	31.16994	4493	30.96994
30.9904	2578	30.9904	3288	30.9904	2416	31.1904	3747	30.9904
31.01087	1874	31.01087	2782	31.01087	1700	31.21087	3189	31.01087
31.03133	1448	31.03133	2318	31.03133	1160	31.23133	2528	31.03133
31.05179	989	31.05179	1714	31.05179	758	31.25179	1975	31.05179
31.07225	649	31.07225	1230	31.07225	602	31.27225	1376	31.07225
31.09272	463	31.09272	914	31.09272	433	31.29272	948	31.09272
31.11318	349	31.11318	586	31.11318	344	31.31318	686	31.11318
31.13364	269	31.13364	476	31.13364	341	31.33364	480	31.13364

31.15411	210	31.15411	327	31.15411	239	31.35411	405	31.15411
31.17457	190	31.17457	207	31.17457	167	31.37457	202	31.17457
31.19503	107	31.19503	193	31.19503	157	31.39503	214	31.19503
31.21549	101	31.21549	189	31.21549	76	31.41549	234	31.21549
31.23596	107	31.23596	141	31.23596	60	31.43596	126	31.23596
31.25642	41	31.25642	61	31.25642	75	31.45642	67	31.25642
31.27688	46	31.27688	83	31.27688	61	31.47688	74	31.27688
31.29734	29	31.29734	38	31.29734	5	31.49734	70	31.29734
31.31781	12	31.31781	8	31.31781	56	31.51781	30	31.31781
31.33827	73	31.33827	31	31.33827	75	31.53827	4	31.33827
31.35873	19	31.35873	65	31.35873	7	31.55873	16	31.35873
31.3792	0	31.3792	33	31.3792	9	31.5792	0	31.3792
31.39966	0	31.39966	0	31.39966	0	31.59966	0	31.39966
31.42012	7	31.42012	0	31.42012	0	31.62012	0	31.42012
31.44058	8	31.44058	0	31.44058	0	31.64058	0	31.44058
31.46105	7	31.46105	14	31.46105	15	31.66105	0	31.46105
31.48151	0	31.48151	20	31.48151	43	31.68151	0	31.48151
31.50197	0	31.50197	0	31.50197	35	31.70197	0	31.50197
31.52244	0	31.52244	0	31.52244	27	31.72244	0	31.52244
31.5429	0	31.5429	0	31.5429	0	31.7429	0	31.5429
31.56336	22	31.56336	20	31.56336	8	31.76336	1	31.56336
31.58382	26	31.58382	0	31.58382	24	31.78382	1	31.58382
31.60429	0	31.60429	0	31.60429	0	31.80429	0	31.60429
31.62475	36	31.62475	0	31.62475	21	31.82475	0	31.62475
31.64521	52	31.64521	0	31.64521	51	31.84521	0	31.64521
31.66567	41	31.66567	42	31.66567	86	31.86567	2	31.66567
31.68614	101	31.68614	51	31.68614	161	31.88614	10	31.68614
31.7066	167	31.7066	92	31.7066	185	31.9066	90	31.7066
31.72706	214	31.72706	120	31.72706	313	31.92706	81	31.72706
31.74753	414	31.74753	204	31.74753	425	31.94753	176	31.74753
31.76799	498	31.76799	237	31.76799	634	31.96799	279	31.76799
31.78845	681	31.78845	336	31.78845	925	31.98845	475	31.78845
31.80891	858	31.80891	558	31.80891	923	32.00891	567	31.80891
31.82938	910	31.82938	609	31.82938	968	32.02938	739	31.82938
31.84984	778	31.84984	729	31.84984	801	32.04984	772	31.84984
31.8703	694	31.8703	671	31.8703	745	32.0703	770	31.8703
31.89077	521	31.89077	603	31.89077	665	32.09077	741	31.89077
31.91123	559	31.91123	505	31.91123	457	32.11123	553	31.91123
31.93169	388	31.93169	499	31.93169	360	32.13169	571	31.93169
31.95215	268	31.95215	364	31.95215	224	32.15215	377	31.95215
31.97262	115	31.97262	262	31.97262	178	32.17262	273	31.97262
31.99308	135	31.99308	175	31.99308	106	32.19308	183	31.99308
32.01354	88	32.01354	128	32.01354	56	32.21354	98	32.01354
32.034	56	32.034	53	32.034	48	32.234	90	32.034
32.05447	9	32.05447	38	32.05447	28	32.25447	15	32.05447
32.07493	21	32.07493	21	32.07493	33	32.27493	54	32.07493
32.09539	0	32.09539	40	32.09539	8	32.29539	0	32.09539
32.11586	23	32.11586	28	32.11586	48	32.31586	12	32.11586
32.13632	38	32.13632	98	32.13632	67	32.33632	43	32.13632
32.15678	22	32.15678	21	32.15678	36	32.35678	24	32.15678
32.17724	36	32.17724	49	32.17724	112	32.37724	27	32.17724
32.19771	58	32.19771	24	32.19771	82	32.39771	10	32.19771
32.21817	63	32.21817	8	32.21817	88	32.41817	56	32.21817
32.23863	76	32.23863	88	32.23863	100	32.43863	35	32.23863
32.2591	132	32.2591	100	32.2591	123	32.4591	74	32.2591
32.27956	106	32.27956	88	32.27956	182	32.47956	55	32.27956
32.30002	148	32.30002	120	32.30002	144	32.50002	109	32.30002
32.32048	173	32.32048	163	32.32048	261	32.52048	147	32.32048

32.34095	237	32.34095	223	32.34095	305	32.54095	197	32.34095	
32.36141	301	32.36141	275	32.36141	479	32.56141	254	32.36141	
32.38187	390	32.38187	399	32.38187	628	32.58187	406	32.38187	
32.40233	564	32.40233	561	32.40233	853	32.60233	497	32.40233	
32.4228	855	32.4228	783	32.4228	1374	32.6228	656	32.4228	
32.44326	1136	32.44326	1159	32.44326	1844	32.64326	958	32.44326	
32.46372	1472	32.46372	1475	32.46372	2534	32.66372	1442	32.46372	
32.48419	1765	32.48419	2076	32.48419	2968	32.68419	1927	32.48419	
32.50465	1908	32.50465	2619	32.50465	2758	32.70465	2462	32.50465	
32.52511	1711	32.52511	2942	32.52511	2480	32.72511	2830	32.52511	
32.54557	1551	32.54557	2919	32.54557	2211	32.74557	2743	32.54557	
32.56604	1376	32.56604	2677	32.56604	1948	32.76604	2448	32.56604	
32.5865	1235	32.5865	2441	32.5865	1605	32.7865	2255	32.5865	
32.60696	952	32.60696	2084	32.60696	1265	32.80696	1784	32.60696	
32.62743	792	32.62743	1761	32.62743	943	32.82743	1559	32.62743	
32.64789	648	32.64789	1381	32.64789	793	32.84789	1195	32.64789	
32.66835	590	32.66835	998	32.66835	816	32.86835	964	32.66835	
32.68881	554	32.68881	833	32.68881	692	32.88881	761	32.68881	
32.70928	530	32.70928	725	32.70928	637	32.90928	594	32.70928	
32.72974	452	32.72974	651	32.72974	557	32.92974	535	32.72974	
32.7502	421	32.7502	531	32.7502	553	32.9502	503	32.7502	
32.77066	327	32.77066	516	32.77066	438	32.97066	489	32.77066	
32.79113	302	32.79113	447	32.79113	443	32.99113	482	32.79113	
32.81159	209	32.81159	346	32.81159	244	33.01159	363	32.81159	
32.83205	194	32.83205	315	32.83205	208	33.03205	261	32.83205	
32.85252	130	32.85252	184	32.85252	163	33.05252	239	32.85252	
32.87298	79	32.87298	190	32.87298	60	33.07298	174	32.87298	
32.89344	55	32.89344	133	32.89344	89	33.09344	138	32.89344	
32.9139	80	32.9139	41	32.9139	49	33.1139	55	32.9139	
32.93437	73	32.93437	59	32.93437	59	33.13437	25	32.93437	
32.95483	0	32.95483	72	32.95483	30	33.15483	29	32.95483	
32.97529	40	32.97529	71	32.97529	7	33.17529	28	32.97529	
32.99576	9	32.99576	27	32.99576	22	33.19576	0	32.99576	
33.01622	24	33.01622	0	33.01622	0	33.21622	27	33.01622	
33.03668	0	33.03668	0	33.03668	41	33.23668	14	33.03668	
33.05714	19	33.05714	33	33.05714	36	33.25714	11	33.05714	
33.07761	33	33.07761	0	33.07761	0	33.27761	0	33.07761	
33.09807	6	33.09807	0	33.09807	35	33.29807	7	33.09807	
33.11853	0	33.11853	27	33.11853	26	33.31853	0	33.11853	
33.13899	15	33.13899	20	33.13899	30	33.33899	0	33.13899	
33.15946	33	33.15946	0	33.15946	0	33.35946	8	33.15946	
33.17992	7	33.17992	18	33.17992	15	33.37992	1	33.17992	
33.20038	0	33.20038	0	33.20038	8	33.40038	0	33.20038	
33.22085	0	33.22085	6	33.22085	53	33.42085	28	33.22085	
33.24131	0	33.24131	6	33.24131	23	33.44131	0	33.24131	
33.26177	16	33.26177	15	33.26177	0	33.46177	0	33.26177	
33.28223	0	33.28223	9	33.28223	7	33.48223	0	33.28223	
33.3027	0	33.3027	6	33.3027	50	33.5027	0	33.3027	
33.32316	0	33.32316	17	33.32316	50	33.52316	0	33.32316	
33.34362	2	33.34362	0	33.34362	0	33.54362	7	33.34362	
33.36409	0	33.36409	0	33.36409	0	33.56409	0	33.36409	
33.38455	19	33.38455	30	33.38455	10	33.58455	0	33.38455	
33.40501	0	33.40501	0	33.40501	0	33.60501	1	33.40501	
33.42547	29	33.42547	0	33.42547	42	33.62547	0	33.42547	
33.44594	42	33.44594	12	33.44594	2	33.64594	34	33.44594	
33.4664	24	33.4664	0	33.4664	3	33.6664	0	33.4664	
33.48686	55	33.48686	44	33.48686	66	33.68686	0	33.48686	
33.50732	96	33.50732	18	33.50732	54	33.70732	18	33.50732	

33.52779	77	33.52779	72	33.52779	109	33.72779	13	33.52779
33.54825	136	33.54825	90	33.54825	137	33.74825	86	33.54825
33.56871	147	33.56871	103	33.56871	199	33.76871	77	33.56871
33.58918	193	33.58918	168	33.58918	224	33.78918	144	33.58918
33.60964	149	33.60964	188	33.60964	191	33.80964	156	33.60964
33.6301	118	33.6301	175	33.6301	128	33.8301	182	33.6301
33.65056	108	33.65056	117	33.65056	183	33.85056	146	33.65056
33.67103	127	33.67103	135	33.67103	135	33.87103	128	33.67103
33.69149	100	33.69149	106	33.69149	101	33.89149	77	33.69149
33.71195	112	33.71195	84	33.71195	80	33.91195	88	33.71195
33.73242	68	33.73242	80	33.73242	60	33.93242	97	33.73242
33.75288	27	33.75288	94	33.75288	46	33.95288	25	33.75288
33.77334	35	33.77334	37	33.77334	56	33.97334	75	33.77334
33.7938	52	33.7938	39	33.7938	11	33.9938	57	33.7938
33.81427	52	33.81427	72	33.81427	26	34.01427	70	33.81427
33.83473	44	33.83473	79	33.83473	85	34.03473	88	33.83473
33.85519	31	33.85519	69	33.85519	69	34.05519	32	33.85519
33.87565	69	33.87565	43	33.87565	27	34.07565	47	33.87565
33.89612	68	33.89612	88	33.89612	120	34.09612	46	33.89612
33.91658	62	33.91658	120	33.91658	129	34.11658	77	33.91658
33.93704	122	33.93704	115	33.93704	136	34.13704	85	33.93704
33.95751	85	33.95751	90	33.95751	126	34.15751	88	33.95751
33.97797	160	33.97797	161	33.97797	180	34.17797	102	33.97797
33.99843	204	33.99843	208	33.99843	206	34.19843	169	33.99843
34.01889	238	34.01889	195	34.01889	291	34.21889	196	34.01889
34.03936	335	34.03936	266	34.03936	388	34.23936	253	34.03936
34.05982	418	34.05982	260	34.05982	437	34.25982	336	34.05982
34.08028	543	34.08028	424	34.08028	600	34.28028	430	34.08028
34.10075	684	34.10075	484	34.10075	767	34.30075	517	34.10075
34.12121	1035	34.12121	654	34.12121	1169	34.32121	754	34.12121
34.14167	1334	34.14167	969	34.14167	1686	34.34167	870	34.14167
34.16213	1987	34.16213	1360	34.16213	2470	34.36213	1346	34.16213
34.1826	2847	34.1826	1866	34.1826	3477	34.3826	1952	34.1826
34.20306	3551	34.20306	2504	34.20306	4311	34.40306	2770	34.20306
34.22352	4073	34.22352	3111	34.22352	4714	34.42352	3630	34.22352
34.24398	4068	34.24398	3558	34.24398	4324	34.44398	4229	34.24398
34.26445	3718	34.26445	3784	34.26445	3832	34.46445	4747	34.26445
34.28491	3342	34.28491	3602	34.28491	3268	34.48491	4367	34.28491
34.30537	2840	34.30537	3292	34.30537	2886	34.50537	4095	34.30537
34.32584	2561	34.32584	3001	34.32584	2502	34.52584	3518	34.32584
34.3463	1989	34.3463	2509	34.3463	1753	34.5463	2941	34.3463
34.36676	1357	34.36676	2136	34.36676	1168	34.56676	2474	34.36676
34.38722	928	34.38722	1563	34.38722	764	34.58722	1828	34.38722
34.40769	651	34.40769	1149	34.40769	557	34.60769	1288	34.40769
34.42815	448	34.42815	811	34.42815	429	34.62815	904	34.42815
34.44861	376	34.44861	602	34.44861	296	34.64861	636	34.44861
34.46908	229	34.46908	479	34.46908	254	34.66908	462	34.46908
34.48954	175	34.48954	350	34.48954	207	34.68954	330	34.48954
34.51	178	34.51	292	34.51	181	34.71	287	34.51
34.53046	128	34.53046	252	34.53046	122	34.73046	196	34.53046
34.55093	67	34.55093	166	34.55093	91	34.75093	155	34.55093
34.57139	35	34.57139	105	34.57139	97	34.77139	119	34.57139
34.59185	75	34.59185	92	34.59185	64	34.79185	56	34.59185
34.61231	49	34.61231	74	34.61231	57	34.81231	100	34.61231
34.63278	18	34.63278	64	34.63278	36	34.83278	64	34.63278
34.65324	26	34.65324	71	34.65324	119	34.85324	72	34.65324
34.6737	9	34.6737	40	34.6737	49	34.8737	42	34.6737
34.69417	14	34.69417	25	34.69417	18	34.89417	24	34.69417

34.71463	0	34.71463	33	34.71463	0	34.91463	4	34.71463	
34.73509	2	34.73509	27	34.73509	14	34.93509	23	34.73509	
34.75555	14	34.75555	45	34.75555	15	34.95555	9	34.75555	
34.77602	0	34.77602	35	34.77602	24	34.97602	29	34.77602	
34.79648	6	34.79648	46	34.79648	0	34.99648	47	34.79648	
34.81694	0	34.81694	51	34.81694	0	35.01694	0	34.81694	
34.83741	0	34.83741	0	34.83741	28	35.03741	10	34.83741	
34.85787	0	34.85787	29	34.85787	7	35.05787	36	34.85787	
34.87833	0	34.87833	56	34.87833	0	35.07833	0	34.87833	
34.89879	7	34.89879	23	34.89879	40	35.09879	6	34.89879	
34.91926	19	34.91926	52	34.91926	29	35.11926	4	34.91926	
34.93972	5	34.93972	33	34.93972	33	35.13972	11	34.93972	
34.96018	2	34.96018	42	34.96018	76	35.16018	0	34.96018	
34.98064	18	34.98064	64	34.98064	6	35.18064	52	34.98064	
35.00111	34	35.00111	39	35.00111	40	35.20111	61	35.00111	
35.02157	36	35.02157	80	35.02157	62	35.22157	36	35.02157	
35.04203	95	35.04203	78	35.04203	101	35.24203	73	35.04203	
35.0625	113	35.0625	88	35.0625	101	35.2625	81	35.0625	
35.08296	123	35.08296	171	35.08296	97	35.28296	99	35.08296	
35.10342	205	35.10342	200	35.10342	230	35.30342	134	35.10342	
35.12388	210	35.12388	255	35.12388	302	35.32388	132	35.12388	
35.14435	363	35.14435	288	35.14435	356	35.34435	231	35.14435	
35.16481	487	35.16481	358	35.16481	524	35.36481	272	35.16481	
35.18527	594	35.18527	517	35.18527	697	35.38527	383	35.18527	
35.20574	915	35.20574	704	35.20574	1125	35.40574	584	35.20574	
35.2262	1244	35.2262	873	35.2262	1523	35.4262	801	35.2262	
35.24666	1505	35.24666	1203	35.24666	1888	35.44666	1096	35.24666	
35.26712	1686	35.26712	1468	35.26712	1889	35.46712	1451	35.26712	
35.28759	1626	35.28759	1668	35.28759	1925	35.48759	1553	35.28759	
35.30805	1434	35.30805	1769	35.30805	1663	35.50805	1653	35.30805	
35.32851	1286	35.32851	1721	35.32851	1431	35.52851	1518	35.32851	
35.34897	1157	35.34897	1568	35.34897	1275	35.54897	1384	35.34897	
35.36944	968	35.36944	1331	35.36944	1025	35.56944	1197	35.36944	
35.3899	743	35.3899	1184	35.3899	784	35.5899	1123	35.3899	
35.41036	575	35.41036	976	35.41036	568	35.61036	908	35.41036	
35.43083	405	35.43083	740	35.43083	336	35.63083	751	35.43083	
35.45129	280	35.45129	598	35.45129	253	35.65129	453	35.45129	
35.47175	201	35.47175	456	35.47175	188	35.67175	376	35.47175	
35.49221	125	35.49221	308	35.49221	109	35.69221	224	35.49221	
35.51268	125	35.51268	223	35.51268	81	35.71268	153	35.51268	
35.53314	74	35.53314	177	35.53314	92	35.73314	111	35.53314	
35.5536	72	35.5536	115	35.5536	27	35.7536	116	35.5536	
35.57407	95	35.57407	83	35.57407	72	35.77407	62	35.57407	
35.59453	26	35.59453	106	35.59453	38	35.79453	48	35.59453	
35.61499	0	35.61499	147	35.61499	20	35.81499	44	35.61499	
35.63545	66	35.63545	90	35.63545	56	35.83545	35	35.63545	
35.65592	5	35.65592	47	35.65592	48	35.85592	61	35.65592	
35.67638	44	35.67638	64	35.67638	0	35.87638	56	35.67638	
35.69684	56	35.69684	81	35.69684	40	35.89684	27	35.69684	
35.7173	53	35.7173	56	35.7173	69	35.9173	49	35.7173	
35.73777	85	35.73777	51	35.73777	105	35.93777	39	35.73777	
35.75823	134	35.75823	102	35.75823	100	35.95823	74	35.75823	
35.77869	66	35.77869	95	35.77869	90	35.97869	79	35.77869	
35.79916	143	35.79916	118	35.79916	217	35.99916	142	35.79916	
35.81962	272	35.81962	244	35.81962	272	36.01962	187	35.81962	
35.84008	357	35.84008	290	35.84008	366	36.04008	250	35.84008	
35.86054	405	35.86054	363	35.86054	496	36.06054	357	35.86054	
35.88101	481	35.88101	509	35.88101	628	36.08101	458	35.88101	

35.90147	456	35.90147	484	35.90147	565	36.10147	486	35.90147
35.92193	409	35.92193	569	35.92193	530	36.12193	532	35.92193
35.9424	384	35.9424	529	35.9424	381	36.1424	498	35.9424
35.96286	328	35.96286	426	35.96286	362	36.16286	444	35.96286
35.98332	270	35.98332	418	35.98332	391	36.18332	384	35.98332
36.00378	221	36.00378	370	36.00378	261	36.20378	372	36.00378
36.02425	189	36.02425	320	36.02425	170	36.22425	284	36.02425
36.04471	151	36.04471	241	36.04471	116	36.24471	269	36.04471
36.06517	94	36.06517	169	36.06517	80	36.26517	112	36.06517
36.08564	19	36.08564	132	36.08564	44	36.28564	118	36.08564
36.1061	48	36.1061	83	36.1061	22	36.3061	71	36.1061
36.12656	54	36.12656	66	36.12656	18	36.32656	43	36.12656
36.14702	18	36.14702	56	36.14702	16	36.34702	52	36.14702
36.16748	0	36.16748	37	36.16748	15	36.36748	15	36.16748
36.18795	7	36.18795	36	36.18795	0	36.38795	0	36.18795
36.20841	0	36.20841	14	36.20841	15	36.40841	0	36.20841
36.22887	0	36.22887	0	36.22887	25	36.42887	36	36.22887
36.24934	0	36.24934	0	36.24934	0	36.44934	0	36.24934
36.2698	0	36.2698	0	36.2698	0	36.4698	11	36.2698
36.29026	6	36.29026	0	36.29026	11	36.49026	0	36.29026
36.31073	0	36.31073	0	36.31073	0	36.51073	6	36.31073
36.33119	13	36.33119	47	36.33119	0	36.53119	0	36.33119
36.35165	0	36.35165	24	36.35165	3	36.55165	0	36.35165
36.37211	8	36.37211	74	36.37211	0	36.57211	15	36.37211
36.39258	54	36.39258	42	36.39258	7	36.59258	0	36.39258
36.41304	5	36.41304	10	36.41304	0	36.61304	0	36.41304
36.4335	0	36.4335	20	36.4335	0	36.6335	19	36.4335
36.45396	0	36.45396	0	36.45396	34	36.65396	2	36.45396
36.47443	9	36.47443	0	36.47443	0	36.67443	0	36.47443
36.49489	0	36.49489	12	36.49489	26	36.69489	0	36.49489
36.51535	0	36.51535	37	36.51535	0	36.71535	0	36.51535
36.53582	0	36.53582	2	36.53582	0	36.73582	0	36.53582
36.55628	0	36.55628	0	36.55628	25	36.75628	0	36.55628
36.57674	0	36.57674	0	36.57674	0	36.77674	3	36.57674
36.59721	0	36.59721	30	36.59721	0	36.79721	0	36.59721
36.61767	34	36.61767	0	36.61767	0	36.81767	7	36.61767
36.63813	0	36.63813	46	36.63813	0	36.83813	22	36.63813
36.65859	0	36.65859	0	36.65859	21	36.85859	0	36.65859
36.67905	41	36.67905	13	36.67905	0	36.87905	0	36.67905
36.69952	54	36.69952	62	36.69952	50	36.89952	0	36.69952
36.71998	0	36.71998	31	36.71998	9	36.91998	31	36.71998
36.74044	32	36.74044	39	36.74044	0	36.94044	1	36.74044
36.76091	15	36.76091	14	36.76091	40	36.96091	0	36.76091
36.78137	49	36.78137	48	36.78137	61	36.98137	0	36.78137
36.80183	37	36.80183	53	36.80183	82	37.00183	68	36.80183
36.8223	93	36.8223	97	36.8223	116	37.0223	70	36.8223
36.84276	143	36.84276	161	36.84276	238	37.04276	78	36.84276
36.86322	194	36.86322	130	36.86322	261	37.06322	141	36.86322
36.88368	217	36.88368	177	36.88368	281	37.08368	168	36.88368
36.90414	237	36.90414	201	36.90414	274	37.10414	229	36.90414
36.92461	233	36.92461	235	36.92461	149	37.12461	221	36.92461
36.94507	237	36.94507	183	36.94507	131	37.14507	252	36.94507
36.96553	187	36.96553	196	36.96553	167	37.16553	191	36.96553
36.986	159	36.986	223	36.986	149	37.186	162	36.986
37.00646	201	37.00646	218	37.00646	111	37.20646	146	37.00646
37.02692	115	37.02692	149	37.02692	75	37.22692	196	37.02692
37.04739	78	37.04739	83	37.04739	42	37.24739	105	37.04739
37.06785	42	37.06785	68	37.06785	23	37.26785	90	37.06785

37.08831	57	37.08831	100	37.08831	37	37.28831	57	37.08831
37.10877	64	37.10877	80	37.10877	12	37.30877	37	37.10877
37.12924	44	37.12924	35	37.12924	42	37.32924	42	37.12924
37.1497	37	37.1497	55	37.1497	40	37.3497	21	37.1497
37.17016	49	37.17016	88	37.17016	52	37.37016	24	37.17016
37.19062	30	37.19062	67	37.19062	1	37.39062	45	37.19062
37.21109	92	37.21109	85	37.21109	99	37.41109	121	37.21109
37.23155	120	37.23155	90	37.23155	115	37.43155	67	37.23155
37.25201	160	37.25201	115	37.25201	98	37.45201	79	37.25201
37.27248	143	37.27248	142	37.27248	147	37.47248	97	37.27248
37.29294	173	37.29294	203	37.29294	239	37.49294	164	37.29294
37.3134	263	37.3134	205	37.3134	210	37.5134	175	37.3134
37.33387	342	37.33387	272	37.33387	312	37.53387	244	37.33387
37.35433	475	37.35433	323	37.35433	417	37.55433	337	37.35433
37.37479	565	37.37479	398	37.37479	562	37.57479	380	37.37479
37.39525	840	37.39525	573	37.39525	859	37.59525	550	37.39525
37.41571	1208	37.41571	772	37.41571	1304	37.61571	748	37.41571
37.43618	1612	37.43618	1063	37.43618	1763	37.63618	1031	37.43618
37.45664	2155	37.45664	1279	37.45664	2192	37.65664	1344	37.45664
37.4771	2348	37.4771	1666	37.4771	2298	37.6771	1874	37.4771
37.49757	2178	37.49757	1963	37.49757	2014	37.69757	2101	37.49757
37.51803	1953	37.51803	1994	37.51803	1779	37.71803	2175	37.51803
37.53849	1961	37.53849	1906	37.53849	1747	37.73849	2251	37.53849
37.55896	1990	37.55896	1812	37.55896	1823	37.75896	2012	37.55896
37.57942	1886	37.57942	1855	37.57942	1766	37.77942	1920	37.57942
37.59988	1918	37.59988	1799	37.59988	1741	37.79988	1967	37.59988
37.62034	1880	37.62034	1788	37.62034	2120	37.82034	1933	37.62034
37.6408	1983	37.6408	1918	37.6408	2305	37.8408	1893	37.6408
37.66127	2076	37.66127	1964	37.66127	2340	37.86127	2019	37.66127
37.68173	2022	37.68173	1959	37.68173	2224	37.88173	2032	37.68173
37.70219	1856	37.70219	1944	37.70219	1912	37.90219	2071	37.70219
37.72266	1681	37.72266	1915	37.72266	1618	37.92266	1974	37.72266
37.74312	1536	37.74312	1679	37.74312	1652	37.94312	1744	37.74312
37.76358	1453	37.76358	1484	37.76358	1366	37.96358	1552	37.76358
37.78405	1188	37.78405	1322	37.78405	1131	37.98405	1361	37.78405
37.80451	980	37.80451	1184	37.80451	777	38.00451	1332	37.80451
37.82497	715	37.82497	979	37.82497	662	38.02497	1020	37.82497
37.84543	470	37.84543	733	37.84543	429	38.04543	756	37.84543
37.8659	436	37.8659	539	37.8659	311	38.0659	560	37.8659
37.88636	317	37.88636	510	37.88636	242	38.08636	380	37.88636
37.90682	245	37.90682	314	37.90682	140	38.10682	374	37.90682
37.92728	211	37.92728	228	37.92728	119	38.12728	295	37.92728
37.94775	134	37.94775	226	37.94775	99	38.14775	172	37.94775
37.96821	102	37.96821	212	37.96821	124	38.16821	147	37.96821
37.98867	155	37.98867	167	37.98867	21	38.18867	150	37.98867
38.00914	79	38.00914	74	38.00914	37	38.20914	106	38.00914
38.0296	110	38.0296	121	38.0296	35	38.2296	75	38.0296
38.05006	85	38.05006	81	38.05006	34	38.25006	115	38.05006
38.07053	59	38.07053	77	38.07053	26	38.27053	152	38.07053
38.09099	32	38.09099	82	38.09099	62	38.29099	101	38.09099
38.11145	66	38.11145	70	38.11145	31	38.31145	0	38.11145
38.13191	64	38.13191	7	38.13191	18	38.33191	30	38.13191
38.15237	37	38.15237	72	38.15237	32	38.35237	38	38.15237
38.17284	0	38.17284	40	38.17284	59	38.37284	46	38.17284
38.1933	53	38.1933	76	38.1933	31	38.3933	62	38.1933
38.21376	60	38.21376	20	38.21376	2	38.41376	57	38.21376
38.23423	104	38.23423	76	38.23423	100	38.43423	32	38.23423
38.25469	119	38.25469	68	38.25469	28	38.45469	87	38.25469

38.27515	120	38.27515	113	38.27515	126	38.47515	117	38.27515
38.29562	196	38.29562	148	38.29562	177	38.49562	57	38.29562
38.31608	193	38.31608	162	38.31608	162	38.51608	132	38.31608
38.33654	230	38.33654	208	38.33654	272	38.53654	165	38.33654
38.357	336	38.357	238	38.357	392	38.557	183	38.357
38.37746	447	38.37746	343	38.37746	597	38.57746	306	38.37746
38.39793	594	38.39793	453	38.39793	832	38.59793	424	38.39793
38.41839	779	38.41839	591	38.41839	1032	38.61839	479	38.41839
38.43885	918	38.43885	738	38.43885	996	38.63885	716	38.43885
38.45932	888	38.45932	851	38.45932	1016	38.65932	909	38.45932
38.47978	787	38.47978	904	38.47978	824	38.67978	886	38.47978
38.50024	717	38.50024	900	38.50024	739	38.70024	876	38.50024
38.52071	653	38.52071	750	38.52071	748	38.72071	828	38.52071
38.54117	664	38.54117	725	38.54117	631	38.74117	712	38.54117
38.56163	509	38.56163	631	38.56163	495	38.76163	605	38.56163
38.58209	391	38.58209	626	38.58209	344	38.78209	607	38.58209
38.60256	295	38.60256	419	38.60256	259	38.80256	476	38.60256
38.62302	227	38.62302	393	38.62302	162	38.82302	362	38.62302
38.64348	154	38.64348	310	38.64348	132	38.84348	237	38.64348
38.66394	132	38.66394	183	38.66394	116	38.86394	244	38.66394
38.68441	143	38.68441	193	38.68441	98	38.88441	164	38.68441
38.70487	106	38.70487	150	38.70487	62	38.90487	133	38.70487
38.72533	56	38.72533	123	38.72533	72	38.92533	88	38.72533
38.7458	60	38.7458	104	38.7458	97	38.9458	87	38.7458
38.76626	55	38.76626	88	38.76626	79	38.96626	56	38.76626
38.78672	53	38.78672	126	38.78672	72	38.98672	73	38.78672
38.80719	42	38.80719	73	38.80719	51	39.00719	54	38.80719
38.82765	97	38.82765	104	38.82765	94	39.02765	93	38.82765
38.84811	76	38.84811	81	38.84811	81	39.04811	84	38.84811
38.86857	117	38.86857	135	38.86857	141	39.06857	106	38.86857
38.88903	115	38.88903	175	38.88903	107	39.08903	88	38.88903
38.9095	237	38.9095	152	38.9095	190	39.1095	127	38.9095
38.92996	278	38.92996	257	38.92996	279	39.12996	234	38.92996
38.95042	373	38.95042	336	38.95042	414	39.15042	179	38.95042
38.97089	457	38.97089	414	38.97089	554	39.17089	303	38.97089
38.99135	644	38.99135	499	38.99135	785	39.19135	413	38.99135
39.01181	664	39.01181	591	39.01181	766	39.21181	549	39.01181
39.03228	722	39.03228	650	39.03228	888	39.23228	597	39.03228
39.05274	717	39.05274	769	39.05274	797	39.25274	668	39.05274
39.0732	606	39.0732	703	39.0732	733	39.2732	679	39.0732
39.09366	698	39.09366	686	39.09366	695	39.29366	733	39.09366
39.11412	593	39.11412	676	39.11412	735	39.31412	646	39.11412
39.13459	595	39.13459	683	39.13459	767	39.33459	626	39.13459
39.15505	639	39.15505	706	39.15505	794	39.35505	643	39.15505
39.17551	546	39.17551	729	39.17551	786	39.37551	679	39.17551
39.19598	614	39.19598	681	39.19598	774	39.39598	735	39.19598
39.21644	525	39.21644	719	39.21644	728	39.41644	669	39.21644
39.2369	508	39.2369	708	39.2369	557	39.4369	708	39.2369
39.25737	421	39.25737	675	39.25737	527	39.45737	726	39.25737
39.27783	360	39.27783	625	39.27783	413	39.47783	561	39.27783
39.29829	365	39.29829	584	39.29829	392	39.49829	540	39.29829
39.31875	266	39.31875	449	39.31875	301	39.51875	398	39.31875
39.33922	215	39.33922	445	39.33922	214	39.53922	388	39.33922
39.35968	168	39.35968	345	39.35968	113	39.55968	313	39.35968
39.38014	64	39.38014	292	39.38014	124	39.58014	233	39.38014
39.4006	51	39.4006	197	39.4006	98	39.6006	135	39.4006
39.42107	58	39.42107	127	39.42107	62	39.62107	116	39.42107
39.44153	78	39.44153	160	39.44153	21	39.64153	92	39.44153

39.46199	69	39.46199	120	39.46199	16	39.66199	28	39.46199	
39.48246	60	39.48246	89	39.48246	4	39.68246	40	39.48246	
39.50292	8	39.50292	101	39.50292	0	39.70292	39	39.50292	
39.52338	3	39.52338	74	39.52338	7	39.72338	0	39.52338	
39.54385	0	39.54385	82	39.54385	12	39.74385	6	39.54385	
39.56431	32	39.56431	65	39.56431	83	39.76431	21	39.56431	
39.58477	39	39.58477	25	39.58477	49	39.78477	0	39.58477	
39.60523	21	39.60523	36	39.60523	32	39.80523	19	39.60523	
39.62569	12	39.62569	0	39.62569	43	39.82569	0	39.62569	
39.64616	24	39.64616	48	39.64616	0	39.84616	0	39.64616	
39.66662	2	39.66662	23	39.66662	43	39.86662	0	39.66662	
39.68708	15	39.68708	20	39.68708	4	39.88708	17	39.68708	
39.70755	31	39.70755	21	39.70755	0	39.90755	0	39.70755	
39.72801	7	39.72801	35	39.72801	0	39.92801	0	39.72801	
39.74847	3	39.74847	7	39.74847	28	39.94847	0	39.74847	
39.76894	0	39.76894	0	39.76894	0	39.96894	7	39.76894	
39.7894	7	39.7894	0	39.7894	3	39.9894	8	39.7894	
39.80986	16	39.80986	61	39.80986	0	40.00986	0	39.80986	
39.83032	19	39.83032	21	39.83032	24	40.03032	0	39.83032	
39.85078	0	39.85078	0	39.85078	0	40.05078	4	39.85078	
39.87125	1	39.87125	13	39.87125	14	40.07125	0	39.87125	
39.89171	16	39.89171	0	39.89171	0	40.09171	18	39.89171	
39.91217	12	39.91217	56	39.91217	4	40.11217	0	39.91217	
39.93264	48	39.93264	51	39.93264	0	40.13264	4	39.93264	
39.9531	0	39.9531	33	39.9531	0	40.1531	0	39.9531	
39.97356	9	39.97356	22	39.97356	0	40.17356	19	39.97356	
39.99403	0	39.99403	21	39.99403	0	40.19403	0	39.99403	
40.01449	0	40.01449	4	40.01449	7	40.21449	0	40.01449	
40.03495	0	40.03495	50	40.03495	26	40.23495	0	40.03495	
40.05541	58	40.05541	27	40.05541	0	40.25541	0	40.05541	
40.07588	72	40.07588	29	40.07588	0	40.27588	25	40.07588	
40.09634	9	40.09634	0	40.09634	0	40.29634	0	40.09634	
40.1168	39	40.1168	0	40.1168	18	40.3168	8	40.1168	
40.13726	30	40.13726	0	40.13726	0	40.33726	19	40.13726	
40.15773	0	40.15773	43	40.15773	53	40.35773	0	40.15773	
40.17819	16	40.17819	50	40.17819	0	40.37819	14	40.17819	
40.19865	0	40.19865	32	40.19865	0	40.39865	0	40.19865	
40.21912	0	40.21912	33	40.21912	0	40.41912	0	40.21912	
40.23958	11	40.23958	4	40.23958	0	40.43958	7	40.23958	
40.26004	0	40.26004	34	40.26004	10	40.46004	24	40.26004	
40.28051	5	40.28051	11	40.28051	18	40.48051	0	40.28051	
40.30097	0	40.30097	40	40.30097	18	40.50097	22	40.30097	
40.32143	0	40.32143	57	40.32143	10	40.52143	0	40.32143	
40.34189	10	40.34189	23	40.34189	0	40.54189	0	40.34189	
40.36235	31	40.36235	26	40.36235	19	40.56235	0	40.36235	
40.38282	33	40.38282	42	40.38282	10	40.58282	10	40.38282	
40.40328	18	40.40328	14	40.40328	9	40.60328	33	40.40328	
40.42374	68	40.42374	5	40.42374	25	40.62374	25	40.42374	
40.44421	44	40.44421	44	40.44421	36	40.64421	0	40.44421	
40.46467	35	40.46467	73	40.46467	77	40.66467	12	40.46467	
40.48513	79	40.48513	79	40.48513	72	40.68513	22	40.48513	
40.5056	39	40.5056	77	40.5056	53	40.7056	0	40.5056	
40.52606	66	40.52606	50	40.52606	71	40.72606	51	40.52606	
40.54652	77	40.54652	91	40.54652	58	40.74652	40	40.54652	
40.56698	49	40.56698	51	40.56698	69	40.76698	96	40.56698	
40.58744	87	40.58744	28	40.58744	72	40.78744	74	40.58744	
40.60791	122	40.60791	79	40.60791	114	40.80791	116	40.60791	
40.62837	146	40.62837	172	40.62837	195	40.82837	82	40.62837	

40.64883	182	40.64883	136	40.64883	177	40.84883	114	40.64883
40.6693	255	40.6693	211	40.6693	219	40.8693	149	40.6693
40.68976	271	40.68976	177	40.68976	299	40.88976	190	40.68976
40.71022	207	40.71022	242	40.71022	243	40.91022	277	40.71022
40.73069	218	40.73069	229	40.73069	181	40.93069	203	40.73069
40.75115	185	40.75115	213	40.75115	240	40.95115	201	40.75115
40.77161	152	40.77161	173	40.77161	154	40.97161	191	40.77161
40.79207	122	40.79207	146	40.79207	138	40.99207	128	40.79207
40.81254	110	40.81254	168	40.81254	59	41.01254	169	40.81254
40.833	127	40.833	133	40.833	86	41.033	112	40.833
40.85346	65	40.85346	88	40.85346	60	41.05346	99	40.85346
40.87392	79	40.87392	71	40.87392	31	41.07392	58	40.87392
40.89439	41	40.89439	65	40.89439	57	41.09439	61	40.89439
40.91485	18	40.91485	57	40.91485	35	41.11485	21	40.91485
40.93531	68	40.93531	39	40.93531	72	41.13531	84	40.93531
40.95578	26	40.95578	64	40.95578	14	41.15578	15	40.95578
40.97624	52	40.97624	13	40.97624	50	41.17624	0	40.97624
40.9967	60	40.9967	0	40.9967	22	41.1967	8	40.9967
41.01717	8	41.01717	0	41.01717	0	41.21717	15	41.01717
41.03763	26	41.03763	13	41.03763	21	41.23763	0	41.03763
41.05809	13	41.05809	38	41.05809	7	41.25809	0	41.05809
41.07855	27	41.07855	56	41.07855	8	41.27855	0	41.07855
41.09901	34	41.09901	0	41.09901	2	41.29901	0	41.09901
41.11948	20	41.11948	30	41.11948	19	41.31948	0	41.11948
41.13994	0	41.13994	11	41.13994	0	41.33994	0	41.13994
41.1604	0	41.1604	29	41.1604	0	41.3604	0	41.1604
41.18087	21	41.18087	0	41.18087	8	41.38087	1	41.18087
41.20133	0	41.20133	14	41.20133	0	41.40133	20	41.20133
41.22179	29	41.22179	15	41.22179	0	41.42179	14	41.22179
41.24226	26	41.24226	0	41.24226	42	41.44226	0	41.24226
41.26272	10	41.26272	0	41.26272	21	41.46272	0	41.26272
41.28318	0	41.28318	21	41.28318	25	41.48318	0	41.28318
41.30364	23	41.30364	0	41.30364	1	41.50364	0	41.30364
41.3241	28	41.3241	48	41.3241	17	41.5241	43	41.3241
41.34457	40	41.34457	0	41.34457	70	41.54457	0	41.34457
41.36503	57	41.36503	19	41.36503	53	41.56503	14	41.36503
41.38549	55	41.38549	27	41.38549	61	41.58549	26	41.38549
41.40596	93	41.40596	22	41.40596	73	41.60596	38	41.40596
41.42642	74	41.42642	61	41.42642	74	41.62642	61	41.42642
41.44688	26	41.44688	58	41.44688	54	41.64688	67	41.44688
41.46735	78	41.46735	68	41.46735	66	41.66735	49	41.46735
41.48781	78	41.48781	74	41.48781	12	41.68781	25	41.48781
41.50827	55	41.50827	17	41.50827	28	41.70827	58	41.50827
41.52873	41	41.52873	8	41.52873	81	41.72873	57	41.52873
41.5492	66	41.5492	42	41.5492	60	41.7492	36	41.5492
41.56966	84	41.56966	52	41.56966	22	41.76966	43	41.56966
41.59012	29	41.59012	2	41.59012	6	41.79012	24	41.59012
41.61058	1	41.61058	0	41.61058	31	41.81058	0	41.61058
41.63105	22	41.63105	2	41.63105	43	41.83105	22	41.63105
41.65151	21	41.65151	15	41.65151	16	41.85151	22	41.65151
41.67197	59	41.67197	17	41.67197	0	41.87197	0	41.67197
41.69244	31	41.69244	28	41.69244	0	41.89244	13	41.69244
41.7129	29	41.7129	4	41.7129	1	41.9129	0	41.7129
41.73336	26	41.73336	43	41.73336	18	41.93336	1	41.73336
41.75383	51	41.75383	46	41.75383	9	41.95383	0	41.75383
41.77429	34	41.77429	44	41.77429	0	41.97429	9	41.77429
41.79475	83	41.79475	9	41.79475	57	41.99475	53	41.79475
41.81521	112	41.81521	31	41.81521	104	42.01521	51	41.81521

41.83567	128	41.83567	57	41.83567	74	42.03567	28	41.83567
41.85614	183	41.85614	101	41.85614	165	42.05614	128	41.85614
41.8766	233	41.8766	164	41.8766	222	42.0766	120	41.8766
41.89706	288	41.89706	214	41.89706	328	42.09706	189	41.89706
41.91753	447	41.91753	363	41.91753	463	42.11753	226	41.91753
41.93799	502	41.93799	430	41.93799	576	42.13799	351	41.93799
41.95845	532	41.95845	469	41.95845	541	42.15845	348	41.95845
41.97892	464	41.97892	484	41.97892	425	42.17892	472	41.97892
41.99938	443	41.99938	445	41.99938	416	42.19938	451	41.99938
42.01984	365	42.01984	335	42.01984	357	42.21984	404	42.01984
42.0403	355	42.0403	431	42.0403	321	42.2403	335	42.0403
42.06076	336	42.06076	302	42.06076	340	42.26076	335	42.06076
42.08123	236	42.08123	301	42.08123	226	42.28123	257	42.08123
42.10169	196	42.10169	237	42.10169	171	42.30169	205	42.10169
42.12215	154	42.12215	222	42.12215	151	42.32215	215	42.12215
42.14262	70	42.14262	202	42.14262	75	42.34262	136	42.14262
42.16308	44	42.16308	108	42.16308	34	42.36308	80	42.16308
42.18354	125	42.18354	77	42.18354	55	42.38354	35	42.18354
42.20401	33	42.20401	28	42.20401	0	42.40401	88	42.20401
42.22447	0	42.22447	0	42.22447	8	42.42447	86	42.22447
42.24493	30	42.24493	15	42.24493	30	42.44493	16	42.24493
42.26539	18	42.26539	0	42.26539	0	42.46539	0	42.26539
42.28586	0	42.28586	26	42.28586	0	42.48586	0	42.28586
42.30632	23	42.30632	0	42.30632	13	42.50632	63	42.30632
42.32678	8	42.32678	0	42.32678	7	42.52678	0	42.32678
42.34724	22	42.34724	29	42.34724	0	42.54724	0	42.34724
42.36771	0	42.36771	0	42.36771	0	42.56771	24	42.36771
42.38817	15	42.38817	17	42.38817	0	42.58817	27	42.38817
42.40863	0	42.40863	0	42.40863	0	42.60863	19	42.40863
42.4291	36	42.4291	2	42.4291	7	42.6291	0	42.4291
42.44956	57	42.44956	24	42.44956	42	42.64956	0	42.44956
42.47002	34	42.47002	18	42.47002	17	42.67002	23	42.47002
42.49049	24	42.49049	0	42.49049	61	42.69049	21	42.49049
42.51095	38	42.51095	6	42.51095	34	42.71095	15	42.51095
42.53141	11	42.53141	64	42.53141	39	42.73141	0	42.53141
42.55187	57	42.55187	20	42.55187	68	42.75187	0	42.55187
42.57233	62	42.57233	80	42.57233	85	42.77233	28	42.57233
42.5928	95	42.5928	86	42.5928	12	42.7928	24	42.5928
42.61326	76	42.61326	59	42.61326	96	42.81326	27	42.61326
42.63372	96	42.63372	61	42.63372	120	42.83372	120	42.63372
42.65419	160	42.65419	99	42.65419	161	42.85419	76	42.65419
42.67465	182	42.67465	173	42.67465	106	42.87465	70	42.67465
42.69511	234	42.69511	181	42.69511	169	42.89511	105	42.69511
42.71558	275	42.71558	277	42.71558	249	42.91558	126	42.71558
42.73604	430	42.73604	312	42.73604	337	42.93604	198	42.73604
42.7565	554	42.7565	422	42.7565	525	42.9565	265	42.7565
42.77696	820	42.77696	554	42.77696	747	42.97696	364	42.77696
42.79742	1013	42.79742	779	42.79742	1091	42.99742	609	42.79742
42.81789	1284	42.81789	961	42.81789	1313	43.01789	704	42.81789
42.83835	1345	42.83835	1141	42.83835	1396	43.03835	902	42.83835
42.85881	1374	42.85881	1284	42.85881	1199	43.05881	1129	42.85881
42.87928	1227	42.87928	1305	42.87928	1086	43.07928	1152	42.87928
42.89974	1051	42.89974	1254	42.89974	975	43.09974	1153	42.89974
42.9202	1082	42.9202	1200	42.9202	917	43.1202	1019	42.9202
42.94067	918	42.94067	1082	42.94067	854	43.14067	881	42.94067
42.96113	857	42.96113	1093	42.96113	740	43.16113	854	42.96113
42.98159	693	42.98159	981	42.98159	691	43.18159	770	42.98159
43.00205	684	43.00205	854	43.00205	535	43.20205	708	43.00205

43.02252	496	43.02252	669	43.02252	344	43.22252	593	43.02252
43.04298	431	43.04298	602	43.04298	292	43.24298	495	43.04298
43.06344	370	43.06344	456	43.06344	239	43.26344	423	43.06344
43.0839	398	43.0839	437	43.0839	255	43.2839	313	43.0839
43.10437	353	43.10437	327	43.10437	286	43.30437	299	43.10437
43.12483	352	43.12483	352	43.12483	275	43.32483	297	43.12483
43.14529	356	43.14529	310	43.14529	317	43.34529	312	43.14529
43.16576	392	43.16576	368	43.16576	380	43.36576	302	43.16576
43.18622	502	43.18622	409	43.18622	453	43.38622	333	43.18622
43.20668	593	43.20668	505	43.20668	535	43.40668	368	43.20668
43.22715	706	43.22715	562	43.22715	692	43.42715	478	43.22715
43.24761	1026	43.24761	610	43.24761	965	43.44761	632	43.24761
43.26807	1304	43.26807	916	43.26807	1347	43.46807	840	43.26807
43.28853	1807	43.28853	1195	43.28853	1934	43.48853	1104	43.28853
43.30899	2313	43.30899	1554	43.30899	2714	43.50899	1514	43.30899
43.32946	2830	43.32946	1986	43.32946	3347	43.52946	1946	43.32946
43.34992	3068	43.34992	2559	43.34992	3406	43.54992	2481	43.34992
43.37038	2957	43.37038	2832	43.37038	3227	43.57038	2837	43.37038
43.39085	2806	43.39085	3002	43.39085	2693	43.59085	2949	43.39085
43.41131	2470	43.41131	2914	43.41131	2474	43.61131	2860	43.41131
43.43177	2452	43.43177	2851	43.43177	2548	43.63177	2646	43.43177
43.45224	2643	43.45224	2461	43.45224	2563	43.65224	2526	43.45224
43.4727	2356	43.4727	2583	43.4727	2691	43.6727	2456	43.4727
43.49316	2275	43.49316	2537	43.49316	2411	43.69316	2486	43.49316
43.51362	2240	43.51362	2515	43.51362	2206	43.71362	2423	43.51362
43.53408	2021	43.53408	2384	43.53408	1915	43.73408	2357	43.53408
43.55455	1626	43.55455	2195	43.55455	1597	43.75455	2099	43.55455
43.57501	1431	43.57501	1931	43.57501	1297	43.77501	1825	43.57501
43.59547	1254	43.59547	1669	43.59547	1159	43.79547	1633	43.59547
43.61594	1115	43.61594	1429	43.61594	1072	43.81594	1332	43.61594
43.6364	1017	43.6364	1204	43.6364	1025	43.8364	1175	43.6364
43.65686	867	43.65686	1040	43.65686	853	43.85686	1027	43.65686
43.67733	741	43.67733	1005	43.67733	641	43.87733	1000	43.67733
43.69779	551	43.69779	777	43.69779	498	43.89779	748	43.69779
43.71825	488	43.71825	669	43.71825	334	43.91825	605	43.71825
43.73871	394	43.73871	505	43.73871	277	43.93871	508	43.73871
43.75918	289	43.75918	404	43.75918	200	43.95918	382	43.75918
43.77964	216	43.77964	317	43.77964	216	43.97964	342	43.77964
43.8001	150	43.8001	178	43.8001	107	44.0001	213	43.8001
43.82056	131	43.82056	175	43.82056	81	44.02056	219	43.82056
43.84103	162	43.84103	156	43.84103	73	44.04103	149	43.84103
43.86149	109	43.86149	82	43.86149	65	44.06149	150	43.86149
43.88195	115	43.88195	96	43.88195	53	44.08195	103	43.88195
43.90242	101	43.90242	104	43.90242	85	44.10242	113	43.90242
43.92288	70	43.92288	57	43.92288	20	44.12288	78	43.92288
43.94334	74	43.94334	74	43.94334	37	44.14334	79	43.94334
43.96381	117	43.96381	57	43.96381	2	44.16381	51	43.96381
43.98427	134	43.98427	18	43.98427	0	44.18427	40	43.98427
44.00473	73	44.00473	37	44.00473	65	44.20473	78	44.00473
44.02519	81	44.02519	44	44.02519	40	44.22519	74	44.02519
44.04565	88	44.04565	40	44.04565	81	44.24565	23	44.04565
44.06612	57	44.06612	69	44.06612	72	44.26612	28	44.06612
44.08658	128	44.08658	108	44.08658	140	44.28658	30	44.08658
44.10704	181	44.10704	125	44.10704	139	44.30704	91	44.10704
44.12751	232	44.12751	158	44.12751	221	44.32751	127	44.12751
44.14797	264	44.14797	186	44.14797	340	44.34797	169	44.14797
44.16843	375	44.16843	266	44.16843	445	44.36843	238	44.16843
44.1889	478	44.1889	374	44.1889	564	44.3889	341	44.1889

44.20936	523	44.20936	403	44.20936	577	44.40936	361	44.20936
44.22982	467	44.22982	503	44.22982	399	44.42982	513	44.22982
44.25028	374	44.25028	492	44.25028	413	44.45028	467	44.25028
44.27074	363	44.27074	417	44.27074	387	44.47074	488	44.27074
44.29121	333	44.29121	442	44.29121	371	44.49121	424	44.29121
44.31167	348	44.31167	340	44.31167	271	44.51167	358	44.31167
44.33213	266	44.33213	325	44.33213	295	44.53213	387	44.33213
44.3526	259	44.3526	377	44.3526	263	44.5526	361	44.3526
44.37306	177	44.37306	317	44.37306	127	44.57306	268	44.37306
44.39352	160	44.39352	255	44.39352	140	44.59352	193	44.39352
44.41399	102	44.41399	201	44.41399	94	44.61399	186	44.41399
44.43445	126	44.43445	106	44.43445	78	44.63445	150	44.43445
44.45491	104	44.45491	105	44.45491	52	44.65491	94	44.45491
44.47537	100	44.47537	88	44.47537	108	44.67537	63	44.47537
44.49584	42	44.49584	46	44.49584	50	44.69584	85	44.49584
44.5163	69	44.5163	57	44.5163	74	44.7163	81	44.5163
44.53676	156	44.53676	59	44.53676	29	44.73676	71	44.53676
44.55722	152	44.55722	46	44.55722	105	44.75722	39	44.55722
44.57769	210	44.57769	89	44.57769	99	44.77769	49	44.57769
44.59815	201	44.59815	116	44.59815	127	44.79815	161	44.59815
44.61861	292	44.61861	118	44.61861	174	44.81861	175	44.61861
44.63908	410	44.63908	239	44.63908	401	44.83908	217	44.63908
44.65954	519	44.65954	314	44.65954	494	44.85954	272	44.65954
44.68	623	44.68	317	44.68	586	44.88	402	44.68
44.70047	827	44.70047	456	44.70047	710	44.90047	521	44.70047
44.72093	854	44.72093	538	44.72093	804	44.92093	674	44.72093
44.74139	870	44.74139	722	44.74139	737	44.94139	796	44.74139
44.76185	789	44.76185	717	44.76185	637	44.96185	803	44.76185
44.78231	727	44.78231	654	44.78231	560	44.98231	769	44.78231
44.80278	625	44.80278	686	44.80278	520	45.00278	691	44.80278
44.82324	581	44.82324	559	44.82324	597	45.02324	600	44.82324
44.8437	619	44.8437	503	44.8437	467	45.0437	547	44.8437
44.86417	460	44.86417	463	44.86417	398	45.06417	528	44.86417
44.88463	470	44.88463	426	44.88463	382	45.08463	475	44.88463
44.90509	400	44.90509	407	44.90509	258	45.10509	427	44.90509
44.92556	318	44.92556	343	44.92556	192	45.12556	302	44.92556
44.94602	155	44.94602	292	44.94602	157	45.14602	273	44.94602
44.96648	146	44.96648	154	44.96648	95	45.16648	165	44.96648
44.98694	122	44.98694	128	44.98694	95	45.18694	168	44.98694
45.0074	101	45.0074	99	45.0074	79	45.2074	140	45.0074
45.02787	137	45.02787	68	45.02787	100	45.22787	106	45.02787
45.04833	66	45.04833	101	45.04833	23	45.24833	107	45.04833
45.06879	50	45.06879	49	45.06879	72	45.26879	70	45.06879
45.08926	64	45.08926	11	45.08926	76	45.28926	21	45.08926
45.10972	5	45.10972	62	45.10972	24	45.30972	42	45.10972
45.13018	2	45.13018	40	45.13018	0	45.33018	0	45.13018
45.15065	0	45.15065	0	45.15065	0	45.35065	0	45.15065
45.17111	0	45.17111	0	45.17111	0	45.37111	0	45.17111
45.19157	10	45.19157	0	45.19157	20	45.39157	24	45.19157
45.21203	43	45.21203	0	45.21203	0	45.41203	6	45.21203
45.2325	12	45.2325	17	45.2325	53	45.4325	0	45.2325
45.25296	0	45.25296	0	45.25296	0	45.45296	0	45.25296
45.27342	0	45.27342	1	45.27342	0	45.47342	11	45.27342
45.29388	35	45.29388	0	45.29388	0	45.49388	8	45.29388
45.31435	9	45.31435	0	45.31435	14	45.51435	0	45.31435
45.33481	46	45.33481	0	45.33481	23	45.53481	0	45.33481
45.35527	19	45.35527	9	45.35527	0	45.55527	0	45.35527
45.37574	0	45.37574	15	45.37574	33	45.57574	0	45.37574

45.3962	2	45.3962	16	45.3962	32	45.5962	10	45.3962
45.41666	47	45.41666	20	45.41666	22	45.61666	17	45.41666
45.43713	41	45.43713	30	45.43713	118	45.63713	5	45.43713
45.45759	46	45.45759	0	45.45759	116	45.65759	0	45.45759
45.47805	73	45.47805	0	45.47805	124	45.67805	7	45.47805
45.49851	125	45.49851	5	45.49851	75	45.69851	63	45.49851
45.51897	134	45.51897	40	45.51897	162	45.71897	82	45.51897
45.53944	109	45.53944	74	45.53944	222	45.73944	157	45.53944
45.5599	161	45.5599	90	45.5599	233	45.7599	148	45.5599
45.58036	175	45.58036	108	45.58036	179	45.78036	153	45.58036
45.60083	187	45.60083	209	45.60083	209	45.80083	166	45.60083
45.62129	167	45.62129	177	45.62129	144	45.82129	169	45.62129
45.64175	110	45.64175	181	45.64175	178	45.84175	137	45.64175
45.66222	134	45.66222	123	45.66222	150	45.86222	93	45.66222
45.68268	138	45.68268	131	45.68268	181	45.88268	98	45.68268
45.70314	94	45.70314	126	45.70314	91	45.90314	143	45.70314
45.7236	112	45.7236	69	45.7236	122	45.9236	110	45.7236
45.74406	95	45.74406	92	45.74406	49	45.94406	96	45.74406
45.76453	50	45.76453	47	45.76453	76	45.96453	101	45.76453
45.78499	69	45.78499	46	45.78499	53	45.98499	60	45.78499
45.80545	7	45.80545	0	45.80545	29	46.00545	37	45.80545
45.82592	0	45.82592	0	45.82592	36	46.02592	17	45.82592
45.84638	30	45.84638	2	45.84638	17	46.04638	11	45.84638
45.86684	0	45.86684	0	45.86684	59	46.06684	16	45.86684
45.88731	38	45.88731	9	45.88731	0	46.08731	16	45.88731
45.90777	61	45.90777	0	45.90777	0	46.10777	11	45.90777
45.92823	1	45.92823	21	45.92823	15	46.12823	0	45.92823
45.94869	18	45.94869	0	45.94869	18	46.14869	0	45.94869
45.96916	9	45.96916	0	45.96916	43	46.16916	3	45.96916
45.98962	20	45.98962	8	45.98962	33	46.18962	0	45.98962
46.01008	28	46.01008	31	46.01008	62	46.21008	0	46.01008
46.03054	19	46.03054	0	46.03054	77	46.23054	21	46.03054
46.05101	49	46.05101	45	46.05101	28	46.25101	7	46.05101
46.07147	17	46.07147	17	46.07147	80	46.27147	17	46.07147
46.09193	30	46.09193	57	46.09193	55	46.29193	35	46.09193
46.1124	23	46.1124	12	46.1124	52	46.3124	42	46.1124
46.13286	33	46.13286	40	46.13286	59	46.33286	36	46.13286
46.15332	26	46.15332	27	46.15332	10	46.35332	0	46.15332
46.17379	58	46.17379	19	46.17379	19	46.37379	34	46.17379
46.19425	27	46.19425	10	46.19425	42	46.39425	38	46.19425
46.21471	3	46.21471	28	46.21471	82	46.41471	55	46.21471
46.23517	26	46.23517	48	46.23517	38	46.43517	30	46.23517
46.25563	51	46.25563	63	46.25563	25	46.45563	4	46.25563
46.2761	12	46.2761	44	46.2761	0	46.4761	23	46.2761
46.29656	33	46.29656	7	46.29656	0	46.49656	0	46.29656
46.31702	0	46.31702	0	46.31702	18	46.51702	0	46.31702
46.33749	31	46.33749	14	46.33749	10	46.53749	6	46.33749
46.35795	34	46.35795	0	46.35795	0	46.55795	13	46.35795
46.37841	0	46.37841	0	46.37841	0	46.57841	0	46.37841
46.39888	0	46.39888	19	46.39888	47	46.59888	0	46.39888
46.41934	7	46.41934	0	46.41934	0	46.61934	10	46.41934
46.4398	0	46.4398	26	46.4398	12	46.6398	10	46.4398
46.46026	0	46.46026	9	46.46026	0	46.66026	31	46.46026
46.48072	0	46.48072	9	46.48072	5	46.68072	0	46.48072
46.50119	9	46.50119	22	46.50119	12	46.70119	37	46.50119
46.52165	0	46.52165	13	46.52165	2	46.72165	13	46.52165
46.54211	34	46.54211	0	46.54211	0	46.74211	0	46.54211
46.56258	0	46.56258	0	46.56258	0	46.76258	2	46.56258

46.58304	0	46.58304	0	46.58304	14	46.78304	0	46.58304
46.6035	4	46.6035	0	46.6035	0	46.8035	0	46.6035
46.62397	0	46.62397	8	46.62397	24	46.82397	18	46.62397
46.64443	0	46.64443	0	46.64443	0	46.84443	23	46.64443
46.66489	81	46.66489	0	46.66489	35	46.86489	47	46.66489
46.68535	24	46.68535	8	46.68535	0	46.88535	5	46.68535
46.70582	14	46.70582	12	46.70582	18	46.90582	3	46.70582
46.72628	13	46.72628	0	46.72628	0	46.92628	0	46.72628
46.74674	4	46.74674	29	46.74674	0	46.94674	16	46.74674
46.7672	38	46.7672	15	46.7672	21	46.9672	13	46.7672
46.78767	88	46.78767	0	46.78767	49	46.98767	0	46.78767
46.80813	60	46.80813	36	46.80813	77	47.00813	19	46.80813
46.82859	35	46.82859	5	46.82859	84	47.02859	55	46.82859
46.84906	116	46.84906	80	46.84906	106	47.04906	38	46.84906
46.86952	125	46.86952	86	46.86952	125	47.06952	109	46.86952
46.88998	160	46.88998	99	46.88998	168	47.08998	153	46.88998
46.91045	132	46.91045	124	46.91045	150	47.11045	167	46.91045
46.93091	118	46.93091	126	46.93091	115	47.13091	122	46.93091
46.95137	81	46.95137	68	46.95137	80	47.15137	108	46.95137
46.97183	113	46.97183	55	46.97183	63	47.17183	119	46.97183
46.99229	77	46.99229	92	46.99229	87	47.19229	117	46.99229
47.01276	72	47.01276	78	47.01276	89	47.21276	78	47.01276
47.03322	59	47.03322	93	47.03322	71	47.23322	92	47.03322
47.05368	72	47.05368	45	47.05368	41	47.25368	64	47.05368
47.07415	111	47.07415	52	47.07415	71	47.27415	73	47.07415
47.09461	54	47.09461	86	47.09461	56	47.29461	82	47.09461
47.11507	79	47.11507	42	47.11507	94	47.31507	66	47.11507
47.13554	68	47.13554	91	47.13554	68	47.33554	36	47.13554
47.156	98	47.156	75	47.156	86	47.356	84	47.156
47.17646	112	47.17646	101	47.17646	49	47.37646	46	47.17646
47.19692	168	47.19692	64	47.19692	119	47.39692	72	47.19692
47.21738	197	47.21738	102	47.21738	127	47.41738	84	47.21738
47.23785	176	47.23785	109	47.23785	200	47.43785	113	47.23785
47.25831	212	47.25831	159	47.25831	215	47.45831	181	47.25831
47.27877	297	47.27877	246	47.27877	330	47.47877	195	47.27877
47.29924	480	47.29924	266	47.29924	471	47.49924	246	47.29924
47.3197	587	47.3197	417	47.3197	671	47.5197	397	47.3197
47.34016	783	47.34016	491	47.34016	836	47.54016	470	47.34016
47.36063	829	47.36063	609	47.36063	949	47.56063	594	47.36063
47.38109	855	47.38109	752	47.38109	937	47.58109	759	47.38109
47.40155	824	47.40155	762	47.40155	774	47.60155	774	47.40155
47.42201	723	47.42201	770	47.42201	696	47.62201	848	47.42201
47.44248	661	47.44248	704	47.44248	633	47.64248	740	47.44248
47.46294	601	47.46294	612	47.46294	567	47.66294	612	47.46294
47.4834	538	47.4834	553	47.4834	534	47.6834	578	47.4834
47.50386	526	47.50386	583	47.50386	488	47.70386	582	47.50386
47.52433	442	47.52433	479	47.52433	424	47.72433	554	47.52433
47.54479	340	47.54479	393	47.54479	297	47.74479	453	47.54479
47.56525	228	47.56525	317	47.56525	244	47.76525	368	47.56525
47.58572	190	47.58572	265	47.58572	143	47.78572	260	47.58572
47.60618	140	47.60618	142	47.60618	91	47.80618	202	47.60618
47.62664	82	47.62664	146	47.62664	41	47.82664	97	47.62664
47.64711	74	47.64711	92	47.64711	32	47.84711	86	47.64711
47.66757	66	47.66757	108	47.66757	7	47.86757	29	47.66757
47.68803	16	47.68803	64	47.68803	28	47.88803	53	47.68803
47.70849	0	47.70849	32	47.70849	51	47.90849	56	47.70849
47.72895	5	47.72895	40	47.72895	26	47.92895	10	47.72895
47.74942	22	47.74942	37	47.74942	0	47.94942	25	47.74942

47.76988	18	47.76988	12	47.76988	0	47.96988	8	47.76988
47.79034	0	47.79034	34	47.79034	0	47.99034	8	47.79034
47.81081	0	47.81081	28	47.81081	20	48.01081	10	47.81081
47.83127	50	47.83127	25	47.83127	46	48.03127	0	47.83127
47.85173	0	47.85173	0	47.85173	0	48.05173	32	47.85173
47.8722	0	47.8722	0	47.8722	0	48.0722	10	47.8722
47.89266	0	47.89266	0	47.89266	4	48.09266	0	47.89266
47.91312	6	47.91312	0	47.91312	11	48.11312	1	47.91312
47.93358	0	47.93358	7	47.93358	0	48.13358	16	47.93358
47.95404	13	47.95404	0	47.95404	0	48.15404	0	47.95404
47.97451	0	47.97451	0	47.97451	0	48.17451	0	47.97451
47.99497	0	47.99497	0	47.99497	0	48.19497	0	47.99497
48.01543	0	48.01543	0	48.01543	0	48.21543	4	48.01543
48.0359	0	48.0359	21	48.0359	2	48.2359	0	48.0359
48.05636	19	48.05636	5	48.05636	7	48.25636	0	48.05636
48.07682	13	48.07682	0	48.07682	32	48.27682	0	48.07682
48.09729	40	48.09729	19	48.09729	17	48.29729	0	48.09729
48.11775	77	48.11775	5	48.11775	56	48.31775	58	48.11775
48.13821	25	48.13821	38	48.13821	105	48.33821	20	48.13821
48.15867	27	48.15867	14	48.15867	130	48.35867	13	48.15867
48.17914	44	48.17914	94	48.17914	90	48.37914	80	48.17914
48.1996	62	48.1996	67	48.1996	131	48.3996	53	48.1996
48.22006	109	48.22006	83	48.22006	60	48.42006	72	48.22006
48.24052	56	48.24052	60	48.24052	76	48.44052	85	48.24052
48.26099	16	48.26099	48	48.26099	112	48.46099	39	48.26099
48.28145	47	48.28145	40	48.28145	56	48.48145	55	48.28145
48.30191	18	48.30191	48	48.30191	0	48.50191	70	48.30191
48.32238	39	48.32238	34	48.32238	71	48.52238	38	48.32238
48.34284	97	48.34284	61	48.34284	31	48.54284	43	48.34284
48.3633	24	48.3633	78	48.3633	73	48.5633	49	48.3633
48.38377	96	48.38377	58	48.38377	95	48.58377	52	48.38377
48.40423	121	48.40423	49	48.40423	126	48.60423	57	48.40423
48.42469	166	48.42469	24	48.42469	72	48.62469	67	48.42469
48.44515	113	48.44515	63	48.44515	141	48.64515	78	48.44515
48.46561	221	48.46561	116	48.46561	88	48.66561	98	48.46561
48.48608	203	48.48608	158	48.48608	155	48.68608	121	48.48608
48.50654	276	48.50654	202	48.50654	250	48.70654	143	48.50654
48.527	406	48.527	229	48.527	291	48.727	222	48.527
48.54747	517	48.54747	286	48.54747	523	48.74747	303	48.54747
48.56793	701	48.56793	376	48.56793	678	48.76793	363	48.56793
48.58839	958	48.58839	613	48.58839	950	48.78839	500	48.58839
48.60886	1083	48.60886	774	48.60886	1252	48.80886	656	48.60886
48.62932	1271	48.62932	977	48.62932	1341	48.82932	915	48.62932
48.64978	1425	48.64978	1122	48.64978	1444	48.84978	1023	48.64978
48.67024	1472	48.67024	1275	48.67024	1362	48.87024	1136	48.67024
48.6907	1460	48.6907	1260	48.6907	1263	48.8907	1209	48.6907
48.71117	1480	48.71117	1329	48.71117	1466	48.91117	1217	48.71117
48.73163	1848	48.73163	1391	48.73163	1770	48.93163	1271	48.73163
48.75209	2073	48.75209	1648	48.75209	2119	48.95209	1523	48.75209
48.77256	2200	48.77256	1887	48.77256	2472	48.97256	1689	48.77256
48.79302	2488	48.79302	2054	48.79302	2613	48.99302	2002	48.79302
48.81348	2503	48.81348	2375	48.81348	2539	49.01348	2262	48.81348
48.83395	2302	48.83395	2374	48.83395	2266	49.03395	2577	48.83395
48.85441	1912	48.85441	2276	48.85441	1719	49.05441	2356	48.85441
48.87487	1729	48.87487	1909	48.87487	1541	49.07487	2122	48.87487
48.89533	1544	48.89533	1735	48.89533	1507	49.09533	1841	48.89533
48.9158	1414	48.9158	1597	48.9158	1466	49.1158	1493	48.9158
48.93626	1294	48.93626	1359	48.93626	1331	49.13626	1407	48.93626

48.95672	1142	48.95672	1290	48.95672	1102	49.15672	1403	48.95672
48.97718	835	48.97718	1250	48.97718	792	49.17718	1244	48.97718
48.99765	698	48.99765	944	48.99765	552	49.19765	1009	48.99765
49.01811	556	49.01811	731	49.01811	483	49.21811	802	49.01811
49.03857	394	49.03857	595	49.03857	217	49.23857	601	49.03857
49.05904	242	49.05904	382	49.05904	166	49.25904	412	49.05904
49.0795	168	49.0795	307	49.0795	148	49.2795	296	49.0795
49.09996	197	49.09996	187	49.09996	22	49.29996	204	49.09996
49.12043	128	49.12043	153	49.12043	52	49.32043	111	49.12043
49.14089	67	49.14089	108	49.14089	10	49.34089	78	49.14089
49.16135	0	49.16135	43	49.16135	0	49.36135	81	49.16135
49.18181	0	49.18181	60	49.18181	0	49.38181	17	49.18181
49.20227	8	49.20227	27	49.20227	0	49.40227	67	49.20227
49.22274	9	49.22274	0	49.22274	16	49.42274	0	49.22274
49.2432	8	49.2432	0	49.2432	0	49.4432	14	49.2432
49.26366	23	49.26366	0	49.26366	7	49.46366	9	49.26366
49.28413	49	49.28413	17	49.28413	31	49.48413	17	49.28413
49.30459	38	49.30459	0	49.30459	57	49.50459	26	49.30459
49.32505	58	49.32505	0	49.32505	117	49.52505	51	49.32505
49.34552	153	49.34552	49	49.34552	211	49.54552	71	49.34552
49.36598	171	49.36598	118	49.36598	345	49.56598	83	49.36598
49.38644	312	49.38644	190	49.38644	448	49.58644	158	49.38644
49.4069	396	49.4069	309	49.4069	574	49.6069	217	49.4069
49.42736	528	49.42736	371	49.42736	615	49.62736	370	49.42736
49.44783	529	49.44783	464	49.44783	652	49.64783	408	49.44783
49.46829	469	49.46829	495	49.46829	566	49.66829	476	49.46829
49.48875	371	49.48875	478	49.48875	437	49.68875	449	49.48875
49.50922	424	49.50922	380	49.50922	363	49.70922	392	49.50922
49.52968	366	49.52968	299	49.52968	326	49.72968	361	49.52968
49.55014	301	49.55014	251	49.55014	341	49.75014	277	49.55014
49.57061	214	49.57061	297	49.57061	326	49.77061	259	49.57061
49.59107	241	49.59107	181	49.59107	212	49.79107	231	49.59107
49.61153	179	49.61153	225	49.61153	91	49.81153	172	49.61153
49.63199	110	49.63199	150	49.63199	91	49.83199	173	49.63199
49.65246	0	49.65246	103	49.65246	61	49.85246	98	49.65246
49.67292	70	49.67292	0	49.67292	31	49.87292	82	49.67292
49.69338	0	49.69338	19	49.69338	0	49.89338	76	49.69338
49.71384	0	49.71384	12	49.71384	0	49.91384	0	49.71384
49.73431	0	49.73431	0	49.73431	0	49.93431	0	49.73431
49.75477	0	49.75477	0	49.75477	0	49.95477	0	49.75477
49.77523	0	49.77523	0	49.77523	4	49.97523	0	49.77523
49.7957	0	49.7957	0	49.7957	44	49.9957	0	49.7957
49.81616	82	49.81616	2	49.81616	162	50.01616	9	49.81616
49.83662	143	49.83662	17	49.83662	230	50.03662	5	49.83662
49.85709	225	49.85709	53	49.85709	401	50.05709	52	49.85709
49.87755	351	49.87755	164	49.87755	618	50.07755	105	49.87755
49.89801	378	49.89801	236	49.89801	634	50.09801	191	49.89801
49.91847	374	49.91847	369	49.91847	513	50.11847	266	49.91847
49.93893	184	49.93893	288	49.93893	285	50.13893	331	49.93893
49.9594	4	49.9594	227	49.9594	160	50.1594	267	49.9594
49.97986	7	49.97986	107	49.97986	67	50.17986	102	49.97986
50.00032	0	50.00032	0	50.00032	0	50.20032	0	50.00032

Acetone	8	Ethanol
0	4.0001	32
65	4.020563	32
53	4.041026	67
42	4.061489	0
36	4.081951	41
0	4.102414	68
0	4.122877	7
67	4.14334	0
10	4.163802	4
90	4.184265	31
141	4.204728	70
0	4.225191	21
0	4.245654	23
30	4.266116	0
45	4.286579	9
123	4.307042	11
46	4.327505	0
26	4.347967	19
12	4.36843	0
31	4.388893	24
51	4.409356	0
101	4.429819	32
64	4.450281	81
54	4.470744	19
64	4.491207	0
0	4.51167	0
0	4.532132	0
0	4.552595	0
82	4.573058	0
111	4.593521	0
29	4.613984	40
79	4.634446	65
73	4.654909	12
166	4.675372	15
94	4.695835	1
41	4.716297	0
39	4.73676	51
119	4.757223	7
10	4.777686	6
0	4.798149	6
0	4.818611	9
0	4.839074	5
0	4.859537	0
9	4.88	0
9	4.900462	11
61	4.920925	16
62	4.941388	0
125	4.961851	38
52	4.982314	9
0	5.002776	0
16	5.023239	0

38	5.043702	42
15	5.064165	20
91	5.084627	0
0	5.10509	17
58	5.125553	0
17	5.146016	75
1	5.166479	0
5	5.186941	53
29	5.207404	0
0	5.227867	0
24	5.24833	0
12	5.268792	36
15	5.289255	0
0	5.309718	0
51	5.330181	10
41	5.350644	24
39	5.371106	59
0	5.391569	15
118	5.412032	63
66	5.432495	53
0	5.452957	20
0	5.47342	30
62	5.493883	0
0	5.514346	45
0	5.534809	39
0	5.555271	0
0	5.575734	20
0	5.596197	72
94	5.61666	0
0	5.637122	19
0	5.657585	64
18	5.678048	5
53	5.698511	48
32	5.718974	120
102	5.739436	89
0	5.759899	45
10	5.780362	112
0	5.800825	15
0	5.821287	22
47	5.84175	32
96	5.862213	82
77	5.882676	122
136	5.903139	23
141	5.923601	126
68	5.944064	120
62	5.964527	227
167	5.98499	102
175	6.005452	223
257	6.025915	141
246	6.046378	215
201	6.066841	217
296	6.087304	146
338	6.107766	275
502	6.128229	282
495	6.148692	482
666	6.169155	412
799	6.189617	458
1029	6.21008	585

1154	6.230543	685
1409	6.251006	802
1929	6.271469	933
2399	6.291931	1210
2910	6.312394	1191
3504	6.332857	1587
4287	6.35332	1959
5416	6.373782	2066
5924	6.394245	2697
6830	6.414708	2991
9042	6.435171	3447
11880	6.455634	4289
15482	6.476096	4905
19753	6.496559	6287
23159	6.517022	7542
27051	6.537485	8560
27011	6.557947	10596
20941	6.57841	11643
12391	6.598873	12305
6921	6.619336	10978
4291	6.639799	6558
3180	6.660261	3990
2414	6.680724	2701
1975	6.701187	1806
1646	6.72165	1468
1216	6.742112	1192
1116	6.762575	945
946	6.783038	874
819	6.803501	827
598	6.823964	659
548	6.844426	502
410	6.864889	599
434	6.885352	397
360	6.905815	371
278	6.926277	440
324	6.94674	241
311	6.967203	397
289	6.987666	207
313	7.008129	416
279	7.028591	269
289	7.049054	333
269	7.069517	332
309	7.08998	331
240	7.110442	334
306	7.130905	340
368	7.151368	257
347	7.171831	386
330	7.192294	394
475	7.212756	431
528	7.233219	466
615	7.253682	631
783	7.274145	609
929	7.294607	689
1042	7.31507	811
1268	7.335533	929
1638	7.355996	1245
2308	7.376459	1423
2721	7.396921	1804

3432	7.417384	1937
4910	7.437847	2621
6461	7.45831	3090
8337	7.478772	3857
10878	7.499235	5162
14364	7.519698	6080
14782	7.540161	7221
9892	7.560624	8335
4421	7.581086	9040
1875	7.601549	6441
1039	7.622012	2912
708	7.642475	1366
630	7.662937	1038
546	7.6834	622
430	7.703863	627
280	7.724326	504
331	7.744789	265
143	7.765251	470
193	7.785714	230
150	7.806177	300
136	7.82664	236
73	7.847102	261
87	7.867565	234
129	7.888028	219
78	7.908491	164
14	7.928953	219
75	7.949416	140
40	7.969879	177
24	7.990342	139
126	8.010804	191
32	8.031267	54
0	8.05173	212
69	8.072193	131
47	8.092655	154
32	8.113118	115
1	8.133581	72
54	8.154044	126
39	8.174507	87
0	8.194969	138
4	8.215432	126
38	8.235895	125
27	8.256358	16
71	8.27682	87
18	8.297283	64
11	8.317746	149
0	8.338209	61
0	8.358672	111
18	8.379134	32
0	8.399597	120
0	8.42006	21
0	8.440523	87
83	8.460985	126
32	8.481448	12
28	8.501911	133
11	8.522374	14
19	8.542837	56
0	8.563299	119
0	8.583762	36

4	8.604225	5
0	8.624688	52
22	8.64515	127
28	8.665613	38
0	8.686076	6
66	8.706539	11
0	8.727002	58
0	8.747464	118
0	8.767927	37
0	8.78839	14
0	8.808853	35
0	8.829315	33
0	8.849778	59
38	8.870241	8
0	8.890704	24
0	8.911167	21
0	8.931629	6
79	8.952092	89
0	8.972555	54
19	8.993018	75
1	9.01348	22
0	9.033943	91
4	9.054406	46
11	9.074869	29
0	9.095332	4
0	9.115794	10
35	9.136257	98
14	9.15672	76
2	9.177183	26
0	9.197645	64
0	9.218108	12
9	9.238571	66
0	9.259034	8
55	9.279497	0
47	9.299959	0
23	9.320422	43
13	9.340885	0
2	9.361348	47
63	9.38181	9
16	9.402273	0
74	9.422736	109
3	9.443199	74
0	9.463662	2
59	9.484124	51
55	9.504587	23
0	9.52505	55
0	9.545513	0
29	9.565975	69
76	9.586438	0
49	9.606901	0
6	9.627364	75
10	9.647827	57
40	9.668289	0
29	9.688752	0
0	9.709215	0
0	9.729678	20
12	9.75014	21
57	9.770603	17

39	9.791066	27
0	9.811529	4
25	9.831992	46
15	9.852454	0
0	9.872917	62
21	9.89338	0
68	9.913843	0
0	9.934305	0
0	9.954768	29
0	9.975231	28
31	9.995694	32
8	10.01616	30
6	10.03662	0
62	10.05708	0
0	10.07755	0
0	10.09801	24
0	10.11847	0
11	10.13893	52
32	10.1594	27
15	10.17986	61
78	10.20032	0
81	10.22078	61
87	10.24125	138
144	10.26171	44
101	10.28217	55
49	10.30264	90
166	10.3231	114
214	10.34356	122
213	10.36402	172
276	10.38449	330
364	10.40495	184
533	10.42541	353
569	10.44588	457
903	10.46634	597
1294	10.4868	789
1741	10.50726	934
2392	10.52773	1418
2961	10.54819	1568
3852	10.56865	1933
4128	10.58911	2428
3639	10.60958	2681
2258	10.63004	2610
1112	10.6505	2232
383	10.67097	1274
258	10.69143	562
107	10.71189	299
108	10.73235	191
160	10.75282	170
75	10.77328	71
67	10.79374	119
26	10.81421	56
40	10.83467	104
47	10.85513	25
0	10.87559	24
8	10.89606	44
48	10.91652	19
0	10.93698	0
0	10.95744	40

2	10.97791	50
0	10.99837	31
0	11.01883	24
9	11.0393	0
0	11.05976	38
25	11.08022	6
94	11.10068	0
34	11.12115	25
0	11.14161	30
11	11.16207	37
0	11.18254	10
7	11.203	4
0	11.22346	30
0	11.24392	18
35	11.26439	13
14	11.28485	0
1	11.30531	34
3	11.32577	0
0	11.34624	5
0	11.3667	22
0	11.38716	7
82	11.40763	5
40	11.42809	0
15	11.44855	0
0	11.46901	0
7	11.48948	0
28	11.50994	0
0	11.5304	0
25	11.55087	35
25	11.57133	2
44	11.59179	39
0	11.61225	0
0	11.63272	0
18	11.65318	0
0	11.67364	2
96	11.6941	0
2	11.71457	1
14	11.73503	53
15	11.75549	17
0	11.77596	0
0	11.79642	80
64	11.81688	0
81	11.83734	0
44	11.85781	71
28	11.87827	68
133	11.89873	48
44	11.9192	9
105	11.93966	49
0	11.96012	54
10	11.98058	0
39	12.00105	0
51	12.02151	6
91	12.04197	17
113	12.06243	99
98	12.0829	9
112	12.10336	34
115	12.12382	157
141	12.14429	75

161	12.16475	58
181	12.18521	97
296	12.20567	204
350	12.22614	162
336	12.2466	241
567	12.26706	353
640	12.28753	505
1019	12.30799	691
1358	12.32845	885
1711	12.34891	1043
2473	12.36938	1320
3036	12.38984	1651
3104	12.4103	1946
2348	12.43076	2155
1531	12.45123	1971
721	12.47169	1549
455	12.49215	841
319	12.51262	383
222	12.53308	199
226	12.55354	169
158	12.574	180
153	12.59447	140
152	12.61493	10
132	12.63539	59
184	12.65586	98
77	12.67632	0
156	12.69678	65
181	12.71724	86
227	12.73771	2
208	12.75817	66
243	12.77863	153
269	12.79909	121
403	12.81956	186
539	12.84002	172
678	12.86048	229
784	12.88095	321
1151	12.90141	427
1290	12.92187	526
1549	12.94233	638
1357	12.9628	765
1039	12.98326	702
632	13.00372	598
314	13.02419	372
303	13.04465	120
228	13.06511	86
187	13.08557	51
149	13.10604	32
166	13.1265	0
164	13.14696	11
182	13.16742	0
178	13.18789	5
258	13.20835	19
142	13.22881	34
213	13.24928	56
205	13.26974	0
175	13.2902	0
160	13.31066	55
203	13.33113	0

233	13.35159	35
216	13.37205	39
197	13.39252	20
196	13.41298	0
253	13.43344	68
184	13.4539	0
117	13.47437	20
277	13.49483	27
280	13.51529	10
280	13.53575	85
213	13.55622	93
206	13.57668	31
274	13.59714	1
280	13.61761	94
269	13.63807	138
271	13.65853	40
239	13.67899	29
264	13.69946	119
280	13.71992	100
307	13.74038	43
282	13.76085	78
382	13.78131	92
382	13.80177	80
333	13.82223	37
447	13.8427	129
435	13.86316	145
517	13.88362	114
478	13.90408	115
400	13.92455	135
512	13.94501	81
452	13.96547	155
504	13.98594	211
498	14.0064	130
532	14.02686	156
491	14.04732	206
581	14.06779	221
686	14.08825	182
563	14.10871	236
692	14.12918	212
556	14.14964	173
629	14.1701	251
692	14.19056	170
699	14.21103	297
654	14.23149	200
715	14.25195	260
713	14.27241	219
855	14.29288	273
832	14.31334	243
760	14.3338	314
812	14.35427	278
855	14.37473	236
865	14.39519	291
895	14.41565	305
967	14.43612	373
963	14.45658	281
997	14.47704	335
998	14.49751	414
985	14.51797	392

965	14.53843	386
1012	14.55889	373
1055	14.57936	433
1004	14.59982	374
1050	14.62028	311
1063	14.64074	404
1167	14.66121	413
1015	14.68167	430
1138	14.70213	383
1208	14.7226	405
1145	14.74306	431
1194	14.76352	523
1240	14.78398	446
1294	14.80445	615
1489	14.82491	608
1734	14.84537	703
2073	14.86584	841
2562	14.8863	1075
3344	14.90676	1231
3780	14.92722	1339
3794	14.94769	1913
3051	14.96815	2466
2329	14.98861	2230
1680	15.00907	1624
1403	15.02954	829
1084	15.05	563
1068	15.07046	497
1055	15.09093	470
864	15.11139	319
991	15.13185	332
866	15.15231	374
783	15.17278	276
905	15.19324	350
730	15.2137	438
835	15.23417	270
793	15.25463	193
731	15.27509	230
714	15.29555	245
705	15.31602	267
689	15.33648	247
590	15.35694	234
623	15.3774	206
641	15.39787	220
563	15.41833	175
515	15.43879	154
541	15.45926	226
534	15.47972	143
609	15.50018	150
529	15.52064	213
517	15.54111	224
567	15.56157	76
495	15.58203	79
569	15.6025	144
505	15.62296	230
434	15.64342	83
434	15.66388	45
465	15.68435	192
421	15.70481	182

380	15.72527	129
413	15.74573	73
401	15.7662	172
437	15.78666	129
458	15.80712	145
439	15.82759	127
440	15.84805	120
372	15.86851	98
473	15.88897	85
378	15.90944	48
415	15.9299	76
403	15.95036	85
451	15.97083	58
427	15.99129	89
356	16.01175	111
452	16.03221	94
373	16.05268	122
467	16.07314	99
472	16.0936	181
403	16.11407	132
474	16.13453	107
550	16.15499	133
547	16.17545	229
724	16.19592	259
871	16.21638	319
985	16.23684	371
1217	16.2573	383
1180	16.27777	659
1041	16.29823	710
850	16.31869	664
670	16.33916	539
548	16.35962	345
415	16.38008	227
506	16.40054	138
485	16.42101	164
515	16.44147	134
419	16.46193	208
502	16.4824	200
494	16.50286	43
402	16.52332	35
518	16.54378	193
421	16.56425	171
434	16.58471	118
522	16.60517	116
475	16.62563	142
509	16.6461	192
632	16.66656	176
789	16.68702	85
585	16.70749	265
566	16.72795	302
534	16.74841	228
420	16.76887	129
383	16.78934	129
379	16.8098	84
324	16.83026	31
327	16.85073	57
322	16.87119	53
295	16.89165	84

264	16.91211	39
287	16.93258	28
292	16.95304	16
249	16.9735	0
157	16.99396	16
235	17.01443	9
193	17.03489	47
207	17.05535	34
207	17.07582	1
174	17.09628	0
114	17.11674	0
148	17.1372	0
78	17.15767	0
187	17.17813	0
80	17.19859	0
28	17.21906	0
44	17.23952	0
51	17.25998	0
83	17.28044	0
32	17.30091	0
32	17.32137	0
89	17.34183	0
61	17.36229	0
59	17.38276	0
57	17.40322	0
50	17.42368	0
26	17.44415	0
46	17.46461	0
31	17.48507	0
40	17.50553	0
0	17.526	0
1	17.54646	0
33	17.56692	0
15	17.58739	0
33	17.60785	0
23	17.62831	0
0	17.64877	0
0	17.66924	0
0	17.6897	0
0	17.71016	0
0	17.73062	0
14	17.75109	0
0	17.77155	0
49	17.79201	0
0	17.81248	0
0	17.83294	0
0	17.8534	0
0	17.87386	0
0	17.89433	0
28	17.91479	0
0	17.93525	0
37	17.95572	0
27	17.97618	0
68	17.99664	0
0	18.0171	0
26	18.03757	0
15	18.05803	0
55	18.07849	0

22	18.09895	0
95	18.11942	0
134	18.13988	0
223	18.16034	23
350	18.18081	116
555	18.20127	199
772	18.22173	271
1115	18.24219	407
1310	18.26266	629
1520	18.28312	866
1385	18.30358	979
1024	18.32405	991
656	18.34451	937
381	18.36497	504
125	18.38543	202
86	18.4059	70
72	18.42636	1
29	18.44682	0
51	18.46728	0
0	18.48775	0
2	18.50821	2
41	18.52867	0
0	18.54914	0
18	18.5696	0
23	18.59006	0
1	18.61052	0
0	18.63099	0
0	18.65145	0
0	18.67191	0
9	18.69238	0
54	18.71284	0
1	18.7333	0
0	18.75376	0
4	18.77423	0
27	18.79469	0
0	18.81515	0
0	18.83561	0
0	18.85608	0
9	18.87654	0
0	18.897	0
1	18.91747	0
41	18.93793	0
1	18.95839	0
20	18.97885	0
0	18.99932	0
49	19.01978	0
31	19.04024	19
31	19.06071	0
4	19.08117	0
0	19.10163	0
91	19.12209	0
35	19.14256	0
112	19.16302	26
43	19.18348	64
71	19.20394	26
162	19.22441	33
255	19.24487	79
370	19.26533	135

582	19.2858	145
1039	19.30626	253
1453	19.32672	452
2137	19.34718	693
2869	19.36765	1024
3377	19.38811	1373
3170	19.40857	1942
2418	19.42904	2350
1713	19.4495	2229
1077	19.46996	1231
545	19.49042	571
260	19.51089	313
274	19.53135	141
132	19.55181	66
113	19.57227	178
98	19.59274	131
78	19.6132	38
69	19.63366	24
67	19.65413	43
104	19.67459	24
0	19.69505	59
41	19.71551	39
68	19.73598	3
13	19.75644	1
62	19.7769	49
0	19.79737	61
0	19.81783	0
6	19.83829	0
20	19.85875	0
86	19.87922	46
33	19.89968	17
5	19.92014	0
29	19.9406	24
26	19.96107	18
13	19.98153	22
0	20.00199	10
30	20.02246	5
0	20.04292	10
24	20.06338	22
32	20.08384	0
18	20.10431	30
22	20.12477	15
74	20.14523	26
42	20.1657	13
102	20.18616	27
0	20.20662	36
0	20.22708	19
0	20.24755	4
17	20.26801	60
40	20.28847	77
0	20.30893	36
4	20.3294	3
0	20.34986	0
0	20.37032	45
33	20.39079	0
35	20.41125	10
63	20.43171	56
0	20.45217	31

17	20.47264	50
0	20.4931	15
35	20.51356	21
4	20.53403	39
2	20.55449	28
30	20.57495	13
18	20.59541	0
1	20.61588	49
22	20.63634	46
0	20.6568	2
0	20.67726	66
16	20.69773	12
38	20.71819	30
52	20.73865	48
0	20.75912	50
13	20.77958	20
7	20.80004	22
23	20.8205	34
0	20.84097	85
13	20.86143	33
33	20.88189	19
40	20.90236	24
49	20.92282	19
12	20.94328	77
19	20.96374	52
44	20.98421	5
63	21.00467	102
102	21.02513	121
245	21.04559	154
366	21.06606	95
489	21.08652	180
554	21.10698	343
575	21.12745	400
515	21.14791	497
350	21.16837	450
301	21.18883	382
143	21.2093	209
77	21.22976	189
89	21.25022	128
35	21.27069	99
85	21.29115	48
89	21.31161	84
55	21.33207	126
129	21.35254	102
77	21.373	92
74	21.39346	76
148	21.41392	111
115	21.43439	90
172	21.45485	101
124	21.47531	159
166	21.49578	132
125	21.51624	111
136	21.5367	154
156	21.55716	146
182	21.57763	76
223	21.59809	143
209	21.61855	146
206	21.63902	147

160	21.65948	113
245	21.67994	193
255	21.7004	117
305	21.72087	216
273	21.74133	172
295	21.76179	219
327	21.78225	170
314	21.80272	200
455	21.82318	250
354	21.84364	254
419	21.86411	254
508	21.88457	240
479	21.90503	326
637	21.92549	309
729	21.94596	328
915	21.96642	458
1263	21.98688	588
1668	22.00735	702
2367	22.02781	910
3631	22.04827	1175
4426	22.06873	1725
5113	22.0892	2359
4928	22.10966	3143
3742	22.13012	3709
3208	22.15058	3349
2285	22.17105	2133
1721	22.19151	1264
1497	22.21197	889
1535	22.23244	844
1587	22.2529	830
1922	22.27336	896
2532	22.29382	1038
3594	22.31429	1341
5018	22.33475	1719
7299	22.35521	2345
9585	22.37568	3075
12107	22.39614	4580
12161	22.4166	6634
10014	22.43706	8593
8065	22.45753	8344
6071	22.47799	5526
4037	22.49845	2951
2609	22.51891	1983
1980	22.53938	1241
1862	22.55984	970
1610	22.5803	762
1494	22.60077	816
1412	22.62123	715
1440	22.64169	673
1334	22.66215	574
1269	22.68262	520
1335	22.70308	466
1291	22.72354	433
1218	22.74401	466
1219	22.76447	432
1118	22.78493	459
1220	22.80539	428
1133	22.82586	419

1158	22.84632	389
1134	22.86678	350
1109	22.88724	378
1082	22.90771	383
1092	22.92817	347
1035	22.94863	289
1025	22.9691	291
1008	22.98956	320
961	23.01002	307
879	23.03048	271
928	23.05095	259
846	23.07141	262
882	23.09187	265
803	23.11234	223
722	23.1328	239
756	23.15326	236
751	23.17372	171
651	23.19419	249
618	23.21465	186
588	23.23511	231
554	23.25557	212
533	23.27604	237
516	23.2965	197
485	23.31696	130
491	23.33743	88
392	23.35789	173
448	23.37835	162
351	23.39881	172
454	23.41928	187
286	23.43974	105
306	23.4602	65
289	23.48067	143
314	23.50113	146
279	23.52159	94
306	23.54205	108
260	23.56252	82
302	23.58298	155
405	23.60344	160
449	23.6239	229
401	23.64437	263
291	23.66483	262
261	23.68529	284
261	23.70576	239
201	23.72622	141
109	23.74668	89
180	23.76714	31
124	23.78761	70
139	23.80807	18
142	23.82853	72
114	23.849	55
102	23.86946	95
48	23.88992	56
102	23.91038	104
83	23.93085	47
85	23.95131	135
70	23.97177	23
43	23.99223	50
96	24.0127	12

65	24.03316	38
42	24.05362	57
40	24.07409	91
59	24.09455	113
51	24.11501	37
52	24.13547	32
56	24.15594	36
44	24.1764	63
73	24.19686	78
109	24.21733	98
113	24.23779	87
123	24.25825	93
127	24.27871	93
136	24.29918	88
202	24.31964	130
249	24.3401	170
327	24.36056	154
578	24.38103	239
865	24.40149	386
1357	24.42195	547
2087	24.44242	802
3224	24.46288	1145
4068	24.48334	1734
4761	24.5038	2470
4463	24.52427	3416
3429	24.54473	3999
2522	24.56519	3496
1952	24.58566	2125
1131	24.60612	1131
555	24.62658	510
344	24.64704	351
291	24.66751	248
209	24.68797	269
222	24.70843	157
147	24.72889	209
100	24.74936	84
87	24.76982	86
112	24.79028	50
85	24.81075	16
18	24.83121	87
35	24.85167	55
85	24.87213	90
14	24.8926	5
0	24.91306	75
19	24.93352	30
0	24.95398	52
54	24.97445	29
16	24.99491	25
2	25.01537	27
49	25.03584	0
2	25.0563	0
10	25.07676	28
32	25.09722	24
0	25.11769	35
0	25.13815	30
13	25.15861	0
19	25.17908	18
0	25.19954	3

5	25.22	0
23	25.24046	0
40	25.26093	13
0	25.28139	0
0	25.30185	16
0	25.32231	18
13	25.34278	0
6	25.36324	0
0	25.3837	0
30	25.40417	0
30	25.42463	0
0	25.44509	5
0	25.46555	0
0	25.48602	0
24	25.50648	0
18	25.52694	0
0	25.54741	13
60	25.56787	0
35	25.58833	5
10	25.60879	0
42	25.62926	23
40	25.64972	1
66	25.67018	44
104	25.69064	17
96	25.71111	32
90	25.73157	61
194	25.75203	99
258	25.7725	131
415	25.79296	147
648	25.81342	175
982	25.83388	326
1501	25.85435	410
2285	25.87481	593
3109	25.89527	979
3536	25.91574	1473
2709	25.9362	1788
2178	25.95666	1653
1752	25.97712	1170
1355	25.99759	548
679	26.01805	349
388	26.03851	222
248	26.05897	121
192	26.07944	103
121	26.0999	149
98	26.12036	115
112	26.14083	53
51	26.16129	37
58	26.18175	2
49	26.20221	48
47	26.22268	103
75	26.24314	29
32	26.2636	99
44	26.28407	13
0	26.30453	20
114	26.32499	0
101	26.34545	44
85	26.36592	49
84	26.38638	85

78	26.40684	92
92	26.4273	85
97	26.44777	15
204	26.46823	92
183	26.48869	138
193	26.50916	152
311	26.52962	171
441	26.55008	193
619	26.57054	336
837	26.59101	400
1391	26.61147	602
2072	26.63193	815
3441	26.6524	1300
5144	26.67286	1915
6771	26.69332	2805
7303	26.71378	4261
6294	26.73425	5609
5167	26.75471	6037
4225	26.77517	4609
3069	26.79563	2499
1957	26.8161	1541
1120	26.83656	882
693	26.85702	623
536	26.87749	194
453	26.89795	229
334	26.91841	328
293	26.93887	344
316	26.95934	337
211	26.9798	249
229	27.00026	144
199	27.02073	132
173	27.04119	121
148	27.06165	143
76	27.08211	189
46	27.10258	130
35	27.12304	43
30	27.1435	50
29	27.16396	12
28	27.18443	47
59	27.20489	85
32	27.22535	43
0	27.24582	30
14	27.26628	36
0	27.28674	27
33	27.3072	5
16	27.32767	0
0	27.34813	35
0	27.36859	23
33	27.38906	30
0	27.40952	27
27	27.42998	22
0	27.45044	44
0	27.47091	21
13	27.49137	10
15	27.51183	26
0	27.53229	39
0	27.55276	42
12	27.57322	20

0	27.59368	35
20	27.61415	19
1	27.63461	44
37	27.65507	19
14	27.67553	60
15	27.696	66
40	27.71646	61
75	27.73692	51
56	27.75739	39
121	27.77785	49
135	27.79831	101
215	27.81877	97
293	27.83924	163
354	27.8597	205
448	27.88016	266
667	27.90062	358
946	27.92109	460
1561	27.94155	592
2428	27.96201	884
3394	27.98248	1198
3992	28.00294	1785
3688	28.0234	2540
3090	28.04386	2997
2510	28.06433	2812
2141	28.08479	1930
1436	28.10525	1036
728	28.12572	613
456	28.14618	387
363	28.16664	239
264	28.1871	249
187	28.20757	168
137	28.22803	174
149	28.24849	144
116	28.26895	145
57	28.28942	156
70	28.30988	37
123	28.33034	65
28	28.35081	31
72	28.37127	13
0	28.39173	83
121	28.41219	87
61	28.43266	130
67	28.45312	51
95	28.47358	56
79	28.49405	17
174	28.51451	102
173	28.53497	80
223	28.55543	128
271	28.5759	183
337	28.59636	198
472	28.61682	249
699	28.63728	309
1072	28.65775	407
1614	28.67821	628
2718	28.69867	858
3916	28.71914	1275
5597	28.7396	2081
6744	28.76006	3183

5986	28.78052	4276
4539	28.80099	4984
3909	28.82145	4726
3307	28.84191	3008
2158	28.86238	1533
1229	28.88284	976
709	28.9033	569
423	28.92376	198
393	28.94423	151
274	28.96469	173
176	28.98515	178
167	29.00561	272
91	29.02608	153
120	29.04654	128
25	29.067	96
65	29.08747	25
76	29.10793	71
0	29.12839	28
27	29.14885	15
45	29.16932	75
35	29.18978	47
0	29.21024	36
27	29.23071	51
11	29.25117	50
0	29.27163	59
41	29.29209	17
27	29.31256	22
26	29.33302	36
12	29.35348	35
41	29.37394	71
9	29.39441	0
31	29.41487	0
1	29.43533	0
0	29.4558	0
0	29.47626	100
33	29.49672	86
0	29.51718	74
33	29.53765	18
30	29.55811	19
0	29.57857	13
0	29.59904	61
19	29.6195	28
31	29.63996	43
10	29.66042	62
58	29.68089	32
54	29.70135	26
94	29.72181	28
100	29.74227	69
130	29.76274	66
177	29.7832	107
197	29.80366	97
343	29.82413	157
376	29.84459	182
598	29.86505	247
1022	29.88551	344
1657	29.90598	478
2509	29.92644	694
3935	29.9469	1161

4921	29.96737	1843
4621	29.98783	2834
3570	30.00829	3762
2933	30.02875	3607
2685	30.04922	2185
2018	30.06968	1069
1197	30.09014	761
725	30.1106	393
416	30.13107	329
299	30.15153	248
194	30.17199	177
247	30.19246	174
170	30.21292	121
123	30.23338	136
74	30.25384	91
16	30.27431	66
75	30.29477	77
78	30.31523	78
37	30.3357	61
83	30.35616	81
41	30.37662	72
70	30.39708	53
118	30.41755	26
46	30.43801	78
102	30.45847	58
41	30.47893	96
52	30.4994	138
88	30.51986	94
124	30.54032	96
44	30.56079	86
164	30.58125	96
200	30.60171	112
330	30.62217	167
557	30.64264	253
629	30.6631	322
777	30.68356	412
767	30.70403	529
633	30.72449	704
729	30.74495	650
613	30.76541	491
872	30.78588	430
987	30.80634	505
1452	30.8268	664
2272	30.84726	875
3306	30.86773	1223
4755	30.88819	1688
5743	30.90865	2522
5849	30.92912	3620
4660	30.94958	4723
3856	30.97004	4828
3465	30.9905	3102
2811	31.01097	1758
1824	31.03143	985
965	31.05189	637
599	31.07236	569
398	31.09282	358
320	31.11328	280
284	31.13374	260

197	31.15421	164
160	31.17467	175
161	31.19513	183
54	31.21559	55
70	31.23606	107
52	31.25652	58
22	31.27698	76
28	31.29745	115
6	31.31791	54
16	31.33837	81
0	31.35883	33
25	31.3793	18
0	31.39976	79
34	31.42022	39
20	31.44069	48
0	31.46115	38
0	31.48161	30
0	31.50207	60
0	31.52254	34
0	31.543	37
67	31.56346	74
49	31.58392	17
52	31.60439	44
43	31.62485	68
24	31.64531	0
58	31.66578	29
127	31.68624	82
110	31.7067	97
188	31.72716	109
302	31.74763	171
455	31.76809	126
732	31.78855	248
1101	31.80902	386
1284	31.82948	548
1184	31.84994	843
926	31.8704	1009
792	31.89087	1007
714	31.91133	671
539	31.93179	293
295	31.95225	197
148	31.97272	79
101	31.99318	43
71	32.01364	82
62	32.0341	49
52	32.05457	96
39	32.07503	60
23	32.09549	20
18	32.11596	51
0	32.13642	24
0	32.15688	9
41	32.17735	39
48	32.19781	22
41	32.21827	52
46	32.23873	57
95	32.2592	29
82	32.27966	94
107	32.30012	84
139	32.32058	21

184	32.34105	89
247	32.36151	74
383	32.38197	113
402	32.40244	225
633	32.4229	271
1203	32.44336	379
1892	32.46383	570
2784	32.48429	811
3347	32.50475	1304
3087	32.52521	1747
2203	32.54567	2049
1966	32.56614	1688
1956	32.5866	965
1613	32.60706	558
1047	32.62753	376
718	32.64799	279
668	32.66845	256
605	32.68892	239
710	32.70938	358
603	32.72984	389
484	32.7503	456
444	32.77076	440
403	32.79123	415
324	32.81169	272
247	32.83215	126
110	32.85262	90
108	32.87308	88
16	32.89354	46
6	32.91401	42
62	32.93447	45
34	32.95493	11
12	32.97539	3
0	32.99586	8
33	33.01632	22
6	33.03678	0
37	33.05724	18
36	33.07771	53
53	33.09817	69
27	33.11863	50
0	33.1391	27
1	33.15956	0
0	33.18002	0
0	33.20049	0
0	33.22095	11
0	33.24141	0
38	33.26187	0
0	33.28233	38
0	33.3028	69
62	33.32326	35
0	33.34372	46
45	33.36419	0
3	33.38465	0
0	33.40511	9
21	33.42558	11
18	33.44604	43
18	33.4665	42
24	33.48696	34
57	33.50742	28

148	33.52789	72
139	33.54835	94
151	33.56881	80
247	33.58928	160
194	33.60974	176
174	33.6302	220
105	33.65067	236
119	33.67113	177
122	33.69159	125
76	33.71205	115
78	33.73252	94
49	33.75298	71
71	33.77344	54
10	33.7939	22
51	33.81437	35
21	33.83483	32
53	33.85529	69
17	33.87576	112
57	33.89622	72
42	33.91668	68
114	33.93715	82
141	33.95761	84
136	33.97807	138
189	33.99853	136
184	34.01899	141
260	34.03946	198
336	34.05992	273
462	34.08038	237
629	34.10085	314
895	34.12131	472
1322	34.14177	591
2019	34.16224	826
3060	34.1827	1215
4397	34.20316	1803
5347	34.22362	2641
5589	34.24408	3754
4647	34.26455	4802
3748	34.28501	5164
3309	34.30547	3794
3177	34.32594	2145
2452	34.3464	1309
1516	34.36686	869
932	34.38733	580
611	34.40779	414
406	34.42825	290
288	34.44871	278
218	34.46918	233
121	34.48964	170
138	34.5101	214
146	34.53056	190
152	34.55103	146
48	34.57149	87
67	34.59195	39
104	34.61242	96
17	34.63288	38
0	34.65334	25
0	34.67381	76
22	34.69427	76

9	34.71473	97
0	34.73519	117
0	34.75565	98
21	34.77612	42
11	34.79658	9
0	34.81704	7
0	34.83751	36
16	34.85797	32
0	34.87843	15
15	34.8989	71
0	34.91936	37
0	34.93982	75
45	34.96028	62
31	34.98074	75
0	35.00121	99
44	35.02167	32
62	35.04213	86
49	35.0626	113
59	35.08306	103
124	35.10352	115
132	35.12399	142
236	35.14445	163
330	35.16491	212
474	35.18537	280
751	35.20584	354
1192	35.2263	422
1776	35.24676	574
2236	35.26722	980
2168	35.28769	1361
1740	35.30815	1564
1443	35.32861	1700
1373	35.34908	1331
1299	35.36954	958
1031	35.39	570
637	35.41047	399
342	35.43093	270
231	35.45139	108
126	35.47185	93
116	35.49231	77
57	35.51278	112
52	35.53324	137
58	35.5537	121
50	35.57417	84
20	35.59463	84
39	35.61509	92
4	35.63556	21
5	35.65602	42
0	35.67648	89
45	35.69694	57
31	35.7174	103
28	35.73787	63
66	35.75833	42
102	35.77879	92
111	35.79926	113
258	35.81972	127
396	35.84018	170
597	35.86065	254
717	35.88111	358

677	35.90157	492
494	35.92203	584
463	35.9425	651
431	35.96296	523
399	35.98342	296
280	36.00388	208
157	36.02435	141
171	36.04481	107
51	36.06527	68
41	36.08574	38
25	36.1062	36
0	36.12666	27
12	36.14713	62
0	36.16759	44
3	36.18805	54
22	36.20851	27
0	36.22897	34
0	36.24944	39
0	36.2699	61
9	36.29036	30
31	36.31083	31
0	36.33129	60
5	36.35175	35
21	36.37222	26
17	36.39268	34
0	36.41314	19
0	36.4336	8
6	36.45406	4
3	36.47453	29
31	36.49499	3
1	36.51545	0
0	36.53592	48
0	36.55638	27
4	36.57684	70
55	36.59731	88
54	36.61777	70
45	36.63823	22
21	36.65869	70
18	36.67916	34
30	36.69962	11
55	36.72008	42
45	36.74054	37
30	36.76101	2
23	36.78147	81
53	36.80193	59
109	36.8224	89
177	36.84286	89
220	36.86332	144
309	36.88379	111
311	36.90425	181
280	36.92471	282
185	36.94517	298
167	36.96563	244
148	36.9861	202
173	37.00656	155
100	37.02702	83
46	37.04749	53
86	37.06795	46

29	37.08841	46
16	37.10888	50
37	37.12934	67
38	37.1498	70
41	37.17026	78
46	37.19072	94
89	37.21119	102
30	37.23165	81
72	37.25211	76
149	37.27258	132
158	37.29304	120
184	37.3135	158
188	37.33397	134
254	37.35443	223
426	37.37489	235
624	37.39535	357
865	37.41582	435
1437	37.43628	612
1968	37.45674	707
2473	37.4772	1153
2456	37.49767	1629
1978	37.51813	2020
1675	37.53859	2059
1745	37.55906	1499
1853	37.57952	1021
1992	37.59998	823
2010	37.62045	695
2262	37.64091	806
2852	37.66137	1145
3028	37.68183	1568
2716	37.70229	2057
2133	37.72276	2335
1791	37.74322	2011
1855	37.76368	1198
1615	37.78415	719
1277	37.80461	450
784	37.82507	365
467	37.84554	227
325	37.866	234
290	37.88646	239
193	37.90692	178
111	37.92738	173
107	37.94785	154
136	37.96831	58
87	37.98877	48
13	38.00924	97
67	38.0297	93
38	38.05016	94
25	38.07063	153
51	38.09109	87
50	38.11155	62
45	38.13201	74
42	38.15248	49
22	38.17294	31
15	38.1934	76
0	38.21386	85
61	38.23433	52
65	38.25479	107

130	38.27525	129
108	38.29572	104
123	38.31618	126
171	38.33664	144
311	38.35711	186
433	38.37757	185
648	38.39803	260
975	38.41849	410
1356	38.43895	586
1261	38.45942	789
1067	38.47988	1166
814	38.50034	1265
768	38.52081	999
778	38.54127	585
669	38.56173	405
490	38.5822	281
365	38.60266	183
159	38.62312	93
137	38.64358	158
108	38.66404	90
124	38.68451	120
85	38.70497	73
42	38.72543	86
61	38.7459	82
52	38.76636	144
98	38.78682	102
56	38.80729	101
39	38.82775	90
52	38.84821	89
60	38.86867	98
140	38.88914	132
157	38.9096	168
253	38.93006	154
320	38.95052	203
453	38.97099	264
711	38.99145	351
899	39.01191	403
933	39.03238	591
752	39.05284	811
642	39.0733	698
636	39.09377	554
699	39.11423	449
682	39.13469	297
714	39.15515	238
665	39.17561	332
762	39.19608	338
765	39.21654	391
582	39.237	461
462	39.25747	426
402	39.27793	385
415	39.29839	239
348	39.31886	182
325	39.33932	76
175	39.35978	101
126	39.38024	44
68	39.4007	77
51	39.42117	68
56	39.44163	60

38	39.46209	69
3	39.48256	103
12	39.50302	37
0	39.52348	13
53	39.54395	57
0	39.56441	46
17	39.58487	41
34	39.60533	20
7	39.6258	62
0	39.64626	73
0	39.66672	28
41	39.68718	68
25	39.70765	75
18	39.72811	34
0	39.74857	18
0	39.76904	24
0	39.7895	34
43	39.80996	9
8	39.83043	34
0	39.85089	46
0	39.87135	52
0	39.89181	19
35	39.91227	62
65	39.93274	65
15	39.9532	41
24	39.97366	21
14	39.99413	13
0	40.01459	9
0	40.03505	7
0	40.05552	3
63	40.07598	49
0	40.09644	48
1	40.1169	62
25	40.13736	51
0	40.15783	44
7	40.17829	34
0	40.19875	43
0	40.21922	25
0	40.23968	4
28	40.26014	44
12	40.28061	53
25	40.30107	36
1	40.32153	20
0	40.34199	68
0	40.36246	8
0	40.38292	33
12	40.40338	42
13	40.42384	23
26	40.44431	24
61	40.46477	29
50	40.48523	44
66	40.5057	41
78	40.52616	72
126	40.54662	60
64	40.56709	93
94	40.58755	92
107	40.60801	57
171	40.62847	94

251	40.64893	128
267	40.6694	157
321	40.68986	167
283	40.71032	289
220	40.73079	343
149	40.75125	307
164	40.77171	203
194	40.79218	143
177	40.81264	104
132	40.8331	69
105	40.85356	78
83	40.87402	53
40	40.89449	57
35	40.91495	50
18	40.93541	70
64	40.95588	72
20	40.97634	61
37	40.9968	85
27	41.01727	19
44	41.03773	42
0	41.05819	0
6	41.07865	56
38	41.09912	5
36	41.11958	48
13	41.14004	24
26	41.1605	83
52	41.18097	64
0	41.20143	53
0	41.22189	54
11	41.24236	40
0	41.26282	41
18	41.28328	51
55	41.30375	48
26	41.32421	42
82	41.34467	44
57	41.36513	53
77	41.38559	107
122	41.40606	79
133	41.42652	127
68	41.44698	147
57	41.46745	154
62	41.48791	44
53	41.50837	97
81	41.52884	73
77	41.5493	56
13	41.56976	25
0	41.59022	66
5	41.61068	58
34	41.63115	66
16	41.65161	50
36	41.67207	101
17	41.69254	78
22	41.713	54
33	41.73346	92
47	41.75393	84
87	41.77439	93
72	41.79485	135
24	41.81531	70

58	41.83578	70
123	41.85624	115
212	41.8767	169
288	41.89716	186
488	41.91763	264
628	41.93809	329
693	41.95855	427
609	41.97902	541
462	41.99948	534
318	42.01994	523
384	42.04041	402
371	42.06087	305
309	42.08133	177
220	42.10179	165
98	42.12225	153
69	42.14272	122
51	42.16318	72
14	42.18364	74
17	42.20411	80
43	42.22457	56
0	42.24503	66
0	42.2655	94
0	42.28596	88
18	42.30642	96
19	42.32688	118
0	42.34734	60
0	42.36781	72
16	42.38827	54
12	42.40873	94
24	42.4292	53
19	42.44966	41
2	42.47012	74
0	42.49059	119
22	42.51105	124
26	42.53151	137
33	42.55197	124
47	42.57244	139
46	42.5929	117
81	42.61336	91
36	42.63382	123
90	42.65429	126
106	42.67475	131
167	42.69521	169
249	42.71568	176
288	42.73614	216
425	42.7566	241
666	42.77707	356
1093	42.79753	520
1592	42.81799	641
1956	42.83845	972
1726	42.85891	1341
1177	42.87938	1654
991	42.89984	1592
1004	42.9203	1127
1146	42.94077	778
1045	42.96123	490
716	42.98169	435
448	43.00216	348

338	43.02262	293
266	43.04308	177
207	43.06354	208
230	43.084	156
150	43.10447	225
196	43.12493	258
244	43.14539	201
300	43.16586	256
310	43.18632	360
418	43.20678	325
538	43.22725	367
705	43.24771	402
1006	43.26817	511
1560	43.28863	768
2414	43.3091	973
3204	43.32956	1370
3667	43.35002	1983
3708	43.37048	2680
2784	43.39095	3302
2308	43.41141	3291
2341	43.43187	2507
2512	43.45234	1614
2910	43.4728	1232
2760	43.49326	1060
2715	43.51373	1009
2394	43.53419	1101
2028	43.55465	1418
1510	43.57511	1376
1231	43.59557	1307
1140	43.61604	962
1186	43.6365	690
1101	43.65696	487
819	43.67743	360
522	43.69789	360
379	43.71835	252
278	43.73882	194
160	43.75928	164
141	43.77974	202
143	43.8002	117
96	43.82066	126
44	43.84113	119
86	43.86159	145
84	43.88205	132
22	43.90252	144
42	43.92298	104
23	43.94344	79
28	43.96391	62
0	43.98437	112
19	44.00483	107
28	44.02529	89
34	44.04576	111
44	44.06622	145
75	44.08668	120
61	44.10714	120
188	44.12761	114
274	44.14807	190
355	44.16853	200
569	44.189	265

690	44.20946	328
625	44.22992	477
510	44.25039	528
369	44.27085	498
370	44.29131	443
325	44.31177	262
368	44.33223	269
251	44.3527	146
192	44.37316	172
172	44.39362	142
40	44.41409	112
68	44.43455	106
0	44.45501	133
51	44.47548	143
45	44.49594	61
44	44.5164	115
48	44.53686	122
67	44.55732	131
90	44.57779	83
135	44.59825	158
175	44.61871	167
297	44.63918	205
443	44.65964	234
580	44.6801	273
784	44.70057	419
1026	44.72103	509
1005	44.74149	782
750	44.76195	907
562	44.78242	904
518	44.80288	760
580	44.82334	476
651	44.8438	311
587	44.86427	295
457	44.88473	271
307	44.90519	189
186	44.92566	180
172	44.94612	286
102	44.96658	146
97	44.98705	78
84	45.00751	76
62	45.02797	94
46	45.04843	87
14	45.06889	33
39	45.08936	123
14	45.10982	119
0	45.13028	88
0	45.15075	36
0	45.17121	60
0	45.19167	90
0	45.21214	15
3	45.2326	39
19	45.25306	30
15	45.27352	51
0	45.29398	38
0	45.31445	25
7	45.33491	53
9	45.35537	29
7	45.37584	57

0	45.3963	65
0	45.41676	23
50	45.43723	49
4	45.45769	89
68	45.47815	78
143	45.49861	55
181	45.51908	75
249	45.53954	168
322	45.56	151
250	45.58046	206
254	45.60093	216
219	45.62139	200
204	45.64185	146
162	45.66232	141
185	45.68278	70
180	45.70324	89
99	45.72371	91
76	45.74417	69
69	45.76463	57
2	45.78509	49
47	45.80555	44
0	45.82602	25
0	45.84648	37
0	45.86694	49
17	45.88741	11
3	45.90787	30
0	45.92833	3
33	45.9488	55
41	45.96926	83
35	45.98972	39
45	46.01018	28
40	46.03064	27
55	46.05111	63
71	46.07157	8
89	46.09203	58
26	46.1125	69
61	46.13296	68
49	46.15342	53
40	46.17389	14
27	46.19435	52
15	46.21481	53
14	46.23527	13
30	46.25574	6
0	46.2762	24
10	46.29666	79
34	46.31712	33
0	46.33759	6
0	46.35805	42
25	46.37851	30
0	46.39898	28
0	46.41944	20
0	46.4399	25
45	46.46036	31
33	46.48083	0
0	46.50129	24
6	46.52175	59
14	46.54221	73
23	46.56268	57

0	46.58314	24
11	46.6036	62
34	46.62407	28
61	46.64453	4
0	46.66499	0
9	46.68546	14
6	46.70592	65
0	46.72638	43
0	46.74684	53
59	46.7673	60
6	46.78777	36
83	46.80823	39
130	46.82869	8
182	46.84916	78
198	46.86962	137
228	46.89008	174
215	46.91055	189
156	46.93101	174
133	46.95147	245
127	46.97193	147
133	46.9924	90
167	47.01286	26
91	47.03332	63
117	47.05378	119
83	47.07425	56
106	47.09471	83
89	47.11517	64
50	47.13564	95
47	47.1561	81
60	47.17656	27
107	47.19702	103
149	47.21749	113
177	47.23795	165
221	47.25841	128
340	47.27887	134
434	47.29934	241
608	47.3198	279
915	47.34026	424
1149	47.36073	553
1118	47.38119	739
911	47.40165	937
730	47.42212	967
642	47.44258	763
624	47.46304	531
700	47.4835	409
659	47.50396	263
443	47.52443	172
363	47.54489	130
215	47.56535	95
145	47.58582	113
137	47.60628	98
62	47.62674	55
43	47.64721	66
46	47.66767	48
44	47.68813	103
0	47.70859	28
34	47.72906	45
13	47.74952	55

1	47.76998	24
0	47.79044	56
44	47.81091	10
15	47.83137	79
0	47.85183	8
1	47.8723	56
0	47.89276	41
0	47.91322	37
0	47.93368	23
0	47.95415	21
0	47.97461	43
3	47.99507	46
41	48.01553	18
5	48.036	14
20	48.05646	27
33	48.07692	29
51	48.09739	79
63	48.11785	73
134	48.13831	94
129	48.15878	105
158	48.17924	110
150	48.1997	161
108	48.22016	197
122	48.24062	170
107	48.26109	105
79	48.28155	83
99	48.30201	58
81	48.32248	85
86	48.34294	77
87	48.3634	66
33	48.38387	71
58	48.40433	91
107	48.42479	46
134	48.44525	117
155	48.46572	165
221	48.48618	162
153	48.50664	161
271	48.5271	168
392	48.54757	242
561	48.56803	286
850	48.58849	410
1137	48.60896	465
1381	48.62942	647
1551	48.64988	927
1455	48.67034	1130
1306	48.69081	1156
1340	48.71127	1203
1569	48.73173	1114
2093	48.75219	1006
2740	48.77266	1074
3040	48.79312	1338
3165	48.81358	1857
2621	48.83405	2251
1917	48.85451	2524
1532	48.87497	2139
1421	48.89544	1541
1484	48.9159	948
1598	48.93636	735

1243	48.95682	517
893	48.97728	412
539	48.99775	321
348	49.01821	242
208	49.03867	200
174	49.05914	138
37	49.0796	151
90	49.10006	89
45	49.12053	57
47	49.14099	63
2	49.16145	38
0	49.18191	108
0	49.20238	71
0	49.22284	90
0	49.2433	68
0	49.26376	86
42	49.28423	91
19	49.30469	81
70	49.32515	78
124	49.34562	100
235	49.36608	167
419	49.38654	214
566	49.407	302
760	49.42747	463
719	49.44793	621
590	49.46839	791
395	49.48885	806
326	49.50932	597
352	49.52978	490
348	49.55024	283
380	49.57071	213
349	49.59117	88
182	49.61163	72
80	49.6321	117
0	49.65256	80
16	49.67302	41
0	49.69348	57
0	49.71394	96
0	49.73441	108
0	49.75487	123
0	49.77533	152
6	49.7958	153
76	49.81626	150
165	49.83672	243
379	49.85719	324
541	49.87765	442
663	49.89811	631
591	49.91857	738
348	49.93904	996
35	49.9595	1023
0	49.97996	808
0	50.00042	531

Figure 2c

2 Theta degree	Intensity a.u.							
	pH=1		pH=3		pH=5		pH=7	
5.0001	-51.7397	5.0001	-50.319	5.0001	-50.309	5.0001	-51.6914	5.0001
5.020563	-41.3933	5.020563	48.03387	5.020563	33.61959	5.020563	-39.9174	5.020563
5.041026	-125.041	5.041026	62.39245	5.041026	26.55541	5.041026	-9.13754	5.041026
5.061488	47.31651	5.061488	75.75674	5.061488	-8.5016	5.061488	-51.3517	5.061488
5.081951	-40.3201	5.081951	10.12676	5.081951	13.44857	5.081951	3.958377	5.081951
5.102414	-50.951	5.102414	-85.4975	5.102414	14.40592	5.102414	-11.7256	5.102414
5.122877	1.861175	5.122877	-15.3029	5.122877	16.37044	5.122877	12.59636	5.122877
5.14334	10.67898	5.14334	48.89749	5.14334	-43.6579	5.14334	-44.0758	5.14334
5.163802	-69.4975	5.163802	8.103559	5.163802	-49.679	5.163802	-92.742	5.163802
5.184265	-16.1021	5.184265	12.31536	5.184265	24.12723	5.184265	63.59776	5.184265
5.204728	102.2991	5.204728	29.53287	5.204728	91.94061	5.204728	14.9434	5.204728
5.225191	15.70592	5.225191	3.756113	5.225191	3.761171	5.225191	-50.705	5.225191
5.245653	-38.8816	5.245653	134.9851	5.245653	55.58891	5.245653	-30.4247	5.245653
5.266116	-60.4634	5.266116	68.21976	5.266116	-17.5762	5.266116	-14.1384	5.266116
5.286579	114.9605	5.286579	27.46017	5.286579	-7.73409	5.286579	-12.8461	5.286579
5.307042	15.38999	5.307042	33.7063	5.307042	68.11519	5.307042	-59.548	5.307042
5.327505	43.8252	5.327505	-8.04185	5.327505	126.9716	5.327505	46.75606	5.327505
5.347967	62.26609	5.347967	70.21572	5.347967	-17.1647	5.347967	31.06605	5.347967
5.36843	58.71265	5.36843	3.479019	5.36843	43.70607	5.36843	88.38197	5.36843
5.388893	-14.8351	5.388893	41.74804	5.388893	45.58405	5.388893	171.7038	5.388893
5.409356	71.6228	5.409356	51.02278	5.409356	94.46922	5.409356	-41.9684	5.409356
5.429818	0.086385	5.429818	61.30324	5.429818	149.3616	5.429818	20.36524	5.429818
5.450281	113.5556	5.450281	80.58942	5.450281	52.26107	5.450281	-25.2952	5.450281
5.470744	60.03059	5.470744	-4.11867	5.470744	33.16777	5.470744	-24.9496	5.470744
5.491207	97.51121	5.491207	79.17896	5.491207	97.08164	5.491207	41.40183	5.491207
5.51167	26.9975	5.51167	64.48232	5.51167	59.0027	5.51167	-20.2408	5.51167
5.532132	119.4895	5.532132	66.79139	5.532132	48.93093	5.532132	35.12251	5.532132
5.552595	43.98712	5.552595	69.10619	5.552595	36.86634	5.552595	10.49173	5.552595
5.573058	-18.5096	5.573058	29.42671	5.573058	62.80892	5.573058	129.8669	5.573058
5.593521	65.99944	5.593521	-4.24705	5.593521	89.75869	5.593521	24.24794	5.593521
5.613983	82.51411	5.613983	102.0849	5.613983	56.71563	5.613983	51.63494	5.613983
5.634446	13.03447	5.634446	63.4226	5.634446	54.67976	5.634446	-26.9721	5.634446
5.654909	151.5605	5.654909	109.766	5.654909	120.6511	5.654909	31.4267	5.654909
5.675372	30.0922	5.675372	77.11514	5.675372	7.629532	5.675372	2.831458	5.675372
5.695835	57.62957	5.695835	91.46999	5.695835	74.61519	5.695835	96.24214	5.695835
5.716297	39.17263	5.716297	36.83057	5.716297	131.608	5.716297	-9.34124	5.716297
5.73676	164.7214	5.73676	83.19687	5.73676	84.60804	5.73676	38.08129	5.73676
5.757223	63.27577	5.757223	-10.4311	5.757223	42.61523	5.757223	60.50975	5.757223
5.777686	1.835853	5.777686	87.94663	5.777686	86.62959	5.777686	114.9441	5.777686
5.798148	-49.5984	5.798148	167.3301	5.798148	111.6511	5.798148	92.38443	5.798148
5.818611	-17.4871	5.818611	279.7193	5.818611	114.6799	5.818611	173.8307	5.818611
5.839074	102.6298	5.839074	5.114189	5.839074	111.7158	5.839074	92.28281	5.839074
5.859537	38.75245	5.859537	-52.4852	5.859537	132.7589	5.859537	127.7409	5.859537
5.88	-10.1192	5.88	79.92117	5.88	212.8091	5.88	176.2049	5.88
5.900462	180.0147	5.900462	183.3333	5.900462	260.8665	5.900462	145.6748	5.900462
5.920925	242.1544	5.920925	149.7511	5.920925	185.9312	5.920925	97.15065	5.920925
5.941388	295.2997	5.941388	137.1746	5.941388	136.003	5.941388	107.6324	5.941388
5.961851	170.4507	5.961851	57.60381	5.961851	218.0819	5.961851	202.1201	5.961851
5.982313	221.6074	5.982313	131.0388	5.982313	155.1681	5.982313	165.6137	5.982313
6.002776	296.7697	6.002776	178.4795	6.002776	193.2614	6.002776	314.1133	6.002776
6.023239	423.9378	6.023239	216.9259	6.023239	221.3619	6.023239	295.6187	6.023239

6.043702	683.1115	6.043702	373.378	6.043702	266.4696	6.043702	499.1301	6.043702
6.064165	737.2909	6.064165	398.8359	6.064165	282.5845	6.064165	751.6474	6.064165
6.084627	862.476	6.084627	459.2994	6.084627	360.7065	6.084627	1141.171	6.084627
6.10509	966.6667	6.10509	545.7687	6.10509	420.8357	6.10509	1684.7	6.10509
6.125553	815.8631	6.125553	697.2438	6.125553	486.9721	6.125553	1815.235	6.125553
6.146016	1116.065	6.146016	934.7245	6.146016	548.1157	6.146016	1860.776	6.146016
6.166478	1389.273	6.166478	1125.211	6.166478	734.2665	6.166478	2009.323	6.166478
6.186941	1781.486	6.186941	1261.703	6.186941	967.4244	6.186941	2331.876	6.186941
6.207404	1824.705	6.207404	1629.201	6.207404	1348.589	6.207404	2249.434	6.207404
6.227867	2079.93	6.227867	1919.705	6.227867	1552.762	6.227867	2254.999	6.227867
6.24833	2154.161	6.24833	1927.214	6.24833	1765.941	6.24833	2552.57	6.24833
6.268792	1625.397	6.268792	2135.729	6.268792	1847.128	6.268792	2405.146	6.268792
6.289255	1501.639	6.289255	2180.25	6.289255	2150.322	6.289255	1660.729	6.289255
6.309718	937.8862	6.309718	1979.776	6.309718	2919.523	6.309718	1150.317	6.309718
6.330181	745.1394	6.330181	1477.309	6.330181	3841.731	6.330181	654.9115	6.330181
6.350643	451.3983	6.350643	1226.847	6.350643	4706.946	6.350643	376.5117	6.350643
6.371106	382.6628	6.371106	1144.39	6.371106	5007.169	6.371106	238.1179	6.371106
6.391569	337.933	6.391569	875.9398	6.391569	4852.398	6.391569	237.73	6.391569
6.412032	261.2089	6.412032	719.4949	6.412032	4583.635	6.412032	193.348	6.412032
6.432495	171.4904	6.432495	468.0558	6.432495	3563.879	6.432495	131.9719	6.432495
6.452957	206.7776	6.452957	345.6223	6.452957	2555.131	6.452957	176.6018	6.452957
6.47342	133.0706	6.47342	342.1946	6.47342	1482.389	6.47342	204.2375	6.47342
6.493883	137.3691	6.493883	236.7727	6.493883	883.6548	6.493883	187.8792	6.493883
6.514346	156.6734	6.514346	194.3564	6.514346	528.9276	6.514346	108.5269	6.514346
6.534808	75.98334	6.534808	234.9459	6.534808	460.2075	6.534808	2.180409	6.534808
6.555271	146.299	6.555271	185.5411	6.555271	285.4947	6.555271	122.8399	6.555271
6.575734	55.62024	6.575734	153.142	6.575734	260.789	6.575734	188.5053	6.575734
6.596197	127.9472	6.596197	180.7486	6.596197	243.0905	6.596197	178.1766	6.596197
6.61666	187.2798	6.61666	155.361	6.61666	178.3991	6.61666	148.8538	6.61666
6.637122	141.6182	6.637122	210.9791	6.637122	218.715	6.637122	137.537	6.637122
6.657585	230.9622	6.657585	185.6029	6.657585	190.038	6.657585	103.2261	6.657585
6.678048	62.31183	6.678048	126.2324	6.678048	202.3682	6.678048	83.9211	6.678048
6.698511	133.6672	6.698511	105.8676	6.698511	181.7056	6.698511	165.622	6.698511
6.718973	84.0282	6.718973	230.5086	6.718973	214.0502	6.718973	177.3289	6.718973
6.739436	84.39489	6.739436	220.1553	6.739436	48.40193	6.739436	138.0417	6.739436
6.759899	100.7673	6.759899	84.80769	6.759899	89.76085	6.759899	70.76039	6.759899
6.780362	143.1453	6.780362	100.4658	6.780362	108.1269	6.780362	180.485	6.780362
6.800825	50.52905	6.800825	161.1297	6.800825	227.5002	6.800825	118.2156	6.800825
6.821287	41.91845	6.821287	100.7993	6.821287	207.8807	6.821287	119.952	6.821287
6.84175	52.31353	6.84175	173.4746	6.84175	135.2683	6.84175	187.6944	6.84175
6.862213	67.71428	6.862213	169.1556	6.862213	257.6631	6.862213	139.4428	6.862213
6.882676	144.1207	6.882676	232.8423	6.882676	113.0651	6.882676	60.19702	6.882676
6.903138	82.53282	6.903138	198.5348	6.903138	194.4743	6.903138	166.9572	6.903138
6.923601	110.9506	6.923601	123.233	6.923601	125.8906	6.923601	134.7233	6.923601
6.944064	163.3741	6.944064	168.9369	6.944064	199.3142	6.944064	155.4953	6.944064
6.964527	105.8032	6.964527	174.6465	6.964527	213.7449	6.964527	290.2733	6.964527
6.98499	177.238	6.98499	167.3619	6.98499	148.1827	6.98499	317.0571	6.98499
7.005452	82.6785	7.005452	245.083	7.005452	243.6278	7.005452	475.8469	7.005452
7.025915	186.1247	7.025915	168.8098	7.025915	218.08	7.025915	484.6426	7.025915
7.046378	159.5765	7.046378	330.5423	7.046378	201.5395	7.046378	548.4442	7.046378
7.066841	292.034	7.066841	321.2806	7.066841	210.0061	7.066841	514.2518	7.066841
7.087303	257.4972	7.087303	442.0245	7.087303	270.4798	7.087303	477.0653	7.087303
7.107766	260.9661	7.107766	519.7742	7.107766	326.9608	7.107766	618.8847	7.107766
7.128229	408.4406	7.128229	506.5296	7.128229	432.4489	7.128229	935.71	7.128229
7.148692	392.9208	7.148692	649.2908	7.148692	550.9442	7.148692	1252.541	7.148692
7.169155	496.4067	7.169155	847.0576	7.169155	657.4467	7.169155	1257.378	7.169155
7.189617	465.8983	7.189617	921.8302	7.189617	1087.956	7.189617	1107.222	7.189617
7.21008	692.3956	7.21008	1105.609	7.21008	1339.473	7.21008	1182.071	7.21008

7.230543	862.8985	7.230543	1560.393	7.230543	1464.997	7.230543	1667.926	7.230543
7.251006	767.4071	7.251006	2094.182	7.251006	1549.528	7.251006	2460.786	7.251006
7.271468	727.9214	7.271468	2438.978	7.271468	1549.067	7.271468	2644.653	7.271468
7.291931	525.4413	7.291931	2507.779	7.291931	1581.612	7.291931	2302.526	7.291931
7.312394	384.9669	7.312394	2030.586	7.312394	1043.165	7.312394	1363.405	7.312394
7.332857	305.4983	7.332857	1498.399	7.332857	816.725	7.332857	699.2891	7.332857
7.35332	229.0352	7.35332	1067.217	7.35332	801.2921	7.35332	392.1796	7.35332
7.373782	135.5779	7.373782	749.041	7.373782	871.8663	7.373782	353.076	7.373782
7.394245	154.1262	7.394245	529.8708	7.394245	729.4478	7.394245	310.9783	7.394245
7.414708	172.6803	7.414708	417.7063	7.414708	527.0364	7.414708	144.8866	7.414708
7.435171	146.2399	7.435171	363.5475	7.435171	251.6322	7.435171	249.8008	7.435171
7.455633	72.80531	7.455633	239.3945	7.455633	273.2352	7.455633	205.7209	7.455633
7.476096	83.37634	7.476096	218.2472	7.476096	162.8454	7.476096	66.64689	7.476096
7.496559	172.9531	7.496559	217.1056	7.496559	133.4627	7.496559	106.5788	7.496559
7.517022	122.5355	7.517022	194.9698	7.517022	238.0872	7.517022	128.5167	7.517022
7.537485	54.12352	7.537485	115.8396	7.537485	169.7189	7.537485	119.4605	7.537485
7.557947	105.7173	7.557947	192.7152	7.557947	205.3578	7.557947	47.41023	7.557947
7.57841	7.316684	7.57841	213.5965	7.57841	90.00382	7.57841	27.36587	7.57841
7.598873	74.92178	7.598873	132.4835	7.598873	-3.34296	7.598873	82.32744	7.598873
7.619336	12.53255	7.619336	105.3763	7.619336	119.3175	7.619336	148.2949	7.619336
7.639798	-10.851	7.639798	118.2748	7.639798	138.985	7.639798	163.2683	7.639798
7.660261	38.77113	7.660261	26.17895	7.660261	98.6598	7.660261	31.24768	7.660261
7.680724	27.39893	7.680724	118.0889	7.680724	95.34174	7.680724	-8.76706	7.680724
7.701187	-46.9676	7.701187	156.0045	7.701187	50.03085	7.701187	109.2241	7.701187
7.72165	-0.32844	7.72165	29.92587	7.72165	71.72715	7.72165	89.22124	7.72165
7.742112	102.3164	7.742112	39.85296	7.742112	80.43062	7.742112	58.22427	7.742112
7.762575	37.9669	7.762575	89.78576	7.762575	76.14127	7.762575	70.23322	7.762575
7.783038	-45.3769	7.783038	70.72429	7.783038	81.85911	7.783038	-11.7519	7.783038
7.803501	-3.71506	7.803501	55.66854	7.803501	130.5841	7.803501	39.26891	7.803501
7.823963	-39.0475	7.823963	65.61852	7.823963	76.3163	7.823963	90.29563	7.823963
7.844426	14.62568	7.844426	169.5742	7.844426	39.05566	7.844426	41.32828	7.844426
7.864889	22.30457	7.864889	26.53563	7.864889	64.80221	7.864889	90.36685	7.864889
7.885352	-46.0109	7.885352	89.50276	7.885352	89.55593	7.885352	31.41135	7.885352
7.905815	-43.7317	7.905815	60.47563	7.905815	68.31683	7.905815	47.46177	7.905815
7.926277	84.55314	7.926277	77.45421	7.926277	118.0849	7.926277	105.5181	7.926277
7.94674	47.84366	7.94674	150.4385	7.94674	91.86016	7.94674	146.5804	7.94674
7.967203	117.1398	7.967203	-24.5715	7.967203	10.6426	7.967203	33.64857	7.967203
7.987666	52.44172	7.987666	-2.5757	7.987666	13.43221	7.987666	6.722681	7.987666
8.008128	-6.25074	8.008128	5.425773	8.008128	-24.771	8.008128	79.80272	8.008128
8.028591	62.06249	8.028591	14.43297	8.028591	-12.967	8.028591	41.88868	8.028591
8.049054	7.381382	8.049054	93.44589	8.049054	17.84411	8.049054	67.98056	8.049054
8.069517	70.70596	8.069517	-19.5355	8.069517	118.6624	8.069517	84.07837	8.069517
8.08998	66.0362	8.08998	16.48889	8.08998	34.48794	8.08998	24.1821	8.08998
8.110442	5.372131	8.110442	45.51898	8.110442	0.320624	8.110442	88.29176	8.110442
8.130905	33.71373	8.130905	79.55479	8.130905	28.16048	8.130905	-15.5927	8.130905
8.151368	-34.939	8.151368	33.59631	8.151368	40.00752	8.151368	29.52883	8.151368
8.171831	45.41397	8.171831	107.6436	8.171831	-9.13826	8.171831	23.65625	8.171831
8.192293	21.7726	8.192293	66.69654	8.192293	-40.2769	8.192293	87.7896	8.192293
8.212756	70.1369	8.212756	-4.24476	8.212756	82.59171	8.212756	-29.0711	8.212756
8.233219	96.50689	8.233219	-19.1803	8.233219	-50.5325	8.233219	81.07407	8.233219
8.253682	48.88255	8.253682	41.8898	8.253682	-13.6496	8.253682	-2.77482	8.253682
8.274145	78.26389	8.274145	66.96566	8.274145	36.24049	8.274145	36.38223	8.274145
8.294607	64.65089	8.294607	33.04725	8.294607	8.137774	8.294607	35.54519	8.294607
8.31507	64.04358	8.31507	50.13456	8.31507	14.04224	8.31507	66.71408	8.31507
8.335533	22.44195	8.335533	36.22759	8.335533	-14.0461	8.335533	92.88889	8.335533
8.355996	-20.154	8.355996	36.32634	8.355996	-6.1273	8.355996	-31.9304	8.355996
8.376458	22.2557	8.376458	80.43082	8.376458	36.7987	8.376458	-44.7437	8.376458
8.396921	35.6711	8.396921	32.54101	8.396921	17.73187	8.396921	9.230866	8.396921

8.417384	81.09216	8.417384	24.65693	8.417384	7.672225	8.417384	62.21137	8.417384
8.437847	61.51891	8.437847	-3.22143	8.437847	43.61976	8.437847	-113.802	8.437847
8.45831	108.9513	8.45831	-19.0941	8.45831	79.57447	8.45831	63.19015	8.45831
8.478772	-14.6106	8.478772	18.03903	8.478772	53.53635	8.478772	-1.81157	8.478772
8.499235	-43.1668	8.499235	-23.8222	8.499235	-35.4946	8.499235	7.192626	8.499235
8.519698	73.28265	8.519698	15.32237	8.519698	21.48167	8.519698	75.20275	8.519698
8.540161	-22.2622	8.540161	34.47262	8.540161	-37.5349	8.540161	-22.7812	8.540161
8.560623	87.19858	8.560623	-106.371	8.560623	2.455692	8.560623	-16.7592	8.560623
8.581086	-57.3349	8.581086	1.790295	8.581086	14.45347	8.581086	24.26866	8.581086
8.601549	39.13721	8.601549	76.95772	8.601549	-63.5416	8.601549	34.30247	8.601549
8.622012	25.61504	8.622012	-20.8691	8.622012	-6.52944	8.622012	4.342211	8.622012
8.642475	11.09855	8.642475	-20.6903	8.642475	4.489878	8.642475	31.38787	8.642475
8.662937	-11.4123	8.662937	-24.5057	8.662937	28.51637	8.662937	82.43946	8.662937
8.6834	-5.91741	8.6834	45.68462	8.6834	-51.45	8.6834	10.49697	8.6834
8.703863	-3.41688	8.703863	33.88065	8.703863	-12.4091	8.703863	-11.4396	8.703863
8.724326	55.08933	8.724326	15.08241	8.724326	57.63892	8.724326	71.62976	8.724326
8.744788	21.60122	8.744788	-41.7101	8.744788	9.69412	8.744788	39.70504	8.744788
8.765251	24.11878	8.765251	30.50308	8.765251	-43.2435	8.765251	151.7862	8.765251
8.785714	17.64202	8.785714	3.722005	8.785714	-13.4702	8.785714	119.8734	8.785714
8.806177	-29.8291	8.806177	80.94665	8.806177	-24.6897	8.806177	80.96642	8.806177
8.82664	-44.2945	8.82664	12.17702	8.82664	28.0979	8.82664	120.0654	8.82664
8.847102	24.64634	8.847102	-21.5869	8.847102	55.89272	8.847102	-17.8297	8.847102
8.867565	30.59283	8.867565	41.65492	8.867565	52.69472	8.867565	88.28111	8.867565
8.888028	-1.45501	8.888028	27.90245	8.888028	40.5039	8.888028	94.39786	8.888028
8.908491	-97.4972	8.908491	98.1557	8.908491	53.32026	8.908491	28.52052	8.908491
8.928953	1.786223	8.928953	69.41468	8.928953	-5.85621	8.928953	-58.3509	8.928953
8.949416	-5.92471	8.949416	-72.3206	8.949416	5.974504	8.949416	51.78363	8.949416
8.969879	-133.63	8.969879	1.949801	8.969879	-83.1876	8.969879	0.924067	8.969879
8.990342	7.670458	8.990342	16.22595	8.990342	84.65746	8.990342	-7.92957	8.990342
9.010805	51.97656	9.010805	-70.4922	9.010805	48.50971	9.010805	27.22271	9.010805
9.031267	18.28833	9.031267	28.7954	9.031267	26.36914	9.031267	7.380922	9.031267
9.05173	-23.3942	9.05173	-0.91129	9.05173	-62.7643	9.05173	-27.4549	9.05173
9.072193	29.9289	9.072193	63.38775	9.072193	77.10952	9.072193	46.71511	9.072193
9.092656	-33.7423	9.092656	-1.3075	9.092656	74.99048	9.092656	44.89109	9.092656
9.113118	-17.4078	9.113118	-63.997	9.113118	-25.1214	9.113118	-34.927	9.113118
9.133581	-14.0677	9.133581	2.214317	9.133581	16.77394	9.133581	99.26082	9.133581
9.154044	4.278169	9.154044	-10.5686	9.154044	36.67643	9.154044	13.45456	9.154044
9.174507	49.62968	9.174507	106.6542	9.174507	-6.4139	9.174507	-21.3458	9.174507
9.19497	47.98686	9.19497	-61.1173	9.19497	-104.497	9.19497	3.859833	9.19497
9.215432	-11.6503	9.215432	17.11688	9.215432	6.334764	9.215432	37.07135	9.215432
9.235895	132.7182	9.235895	-17.6432	9.235895	50.17375	9.235895	49.2888	9.235895
9.256358	84.09246	9.256358	98.6025	9.256358	-34.9801	9.256358	0.512161	9.256358
9.276821	-65.5276	9.276821	20.8539	9.276821	-30.1267	9.276821	-3.25855	9.276821
9.297283	39.85791	9.297283	75.11101	9.297283	-3.26622	9.297283	-54.0233	9.297283
9.317746	33.24915	9.317746	60.37384	9.317746	22.60149	9.317746	64.2178	9.317746
9.338209	77.64607	9.338209	25.64241	9.338209	17.47636	9.338209	-62.5351	9.338209
9.358672	89.04866	9.358672	10.91669	9.358672	-21.6416	9.358672	-33.2822	9.358672
9.379135	-6.54307	9.379135	20.19669	9.379135	-8.75234	9.379135	-43.0233	9.379135
9.399597	41.87087	9.399597	20.48242	9.399597	1.14407	9.399597	2.48542	9.399597
9.42006	18.2905	9.42006	172.7739	9.42006	63.04766	9.42006	33.00002	9.42006
9.440523	34.71579	9.440523	115.071	9.440523	-53.0416	9.440523	72.52053	9.440523
9.460986	-18.8532	9.460986	-43.6261	9.460986	19.87638	9.460986	82.04697	9.460986
9.481448	-43.4166	9.481448	47.68255	9.481448	8.801505	9.481448	14.57934	9.481448
9.501911	-20.1648	9.501911	8.996881	9.501911	42.73381	9.501911	52.11762	9.501911
9.522374	60.09275	9.522374	111.3169	9.522374	48.67329	9.522374	94.66184	9.522374
9.542837	27.35593	9.542837	163.6427	9.542837	-1.38005	9.542837	61.21197	9.542837
9.5633	20.62478	9.5633	17.97423	9.5633	-68.4262	9.5633	49.76802	9.5633
9.583762	110.8993	9.583762	-5.68855	9.583762	2.534808	9.583762	35.33001	9.583762

9.604225	-5.82048	9.604225	-6.3456	9.604225	-57.497	9.604225	-67.1021	9.604225
9.624688	90.46541	9.624688	-44.9969	9.624688	-1.65475	9.624688	38.47174	9.624688
9.645151	91.75697	9.645151	5.564136	9.645151	11.19467	9.645151	56.05149	9.645151
9.665613	4.054209	9.665613	6.130919	9.665613	13.05127	9.665613	14.63716	9.665613
9.686076	-7.64288	9.686076	24.70342	9.686076	-19.0849	9.686076	74.22876	9.686076
9.706539	114.6657	9.706539	-17.7183	9.706539	29.78601	9.706539	-64.1737	9.706539
9.727002	105.98	9.727002	-6.1344	9.727002	-35.3359	9.727002	10.42973	9.727002
9.747465	74.29992	9.747465	47.45528	9.747465	47.54946	9.747465	50.0391	9.747465
9.767927	65.62554	9.767927	62.05067	9.767927	81.44196	9.767927	3.65439	9.767927
9.78839	47.95683	9.78839	113.6518	9.78839	-36.6584	9.78839	13.27561	9.78839
9.808853	145.2938	9.808853	27.25863	9.808853	66.24847	9.808853	30.90274	9.808853
9.829316	101.6365	9.829316	-17.1288	9.829316	4.1625	9.829316	84.53581	9.829316
9.849778	69.98477	9.849778	61.48947	9.849778	-28.9163	9.849778	15.17479	9.849778
9.870241	91.33878	9.870241	20.11348	9.870241	-3.98791	9.870241	86.8197	9.870241
9.890704	64.69845	9.890704	66.74321	9.890704	18.94765	9.890704	42.47054	9.890704
9.911167	110.0638	9.911167	33.37866	9.911167	-41.1096	9.911167	2.127291	9.911167
9.93163	91.43483	9.93163	-20.9802	9.93163	-45.4439	9.93163	45.78997	9.93163
9.952092	44.81153	9.952092	12.66673	9.952092	45.22899	9.952092	37.45857	9.952092
9.972555	89.19391	9.972555	-10.6807	9.972555	6.909056	9.972555	142.1331	9.972555
9.993018	84.58197	9.993018	27.97768	9.993018	34.5963	9.993018	22.81355	9.993018
10.01348	175.9757	10.01348	108.6417	10.01348	-49.7093	10.01348	32.49992	10.01348
10.03394	126.3751	10.03394	46.31153	10.03394	6.992326	10.03394	118.1922	10.03394
10.05441	200.7802	10.05441	68.98704	10.05441	9.701105	10.05441	-28.1096	10.05441
10.07487	135.1909	10.07487	4.668269	10.07487	39.41706	10.07487	118.5946	10.07487
10.09533	268.6074	10.09533	-7.64478	10.09533	-2.8598	10.09533	68.30465	10.09533
10.1158	263.0295	10.1158	57.04789	10.1158	13.87051	10.1158	65.02065	10.1158
10.13626	283.4573	10.13626	121.7463	10.13626	43.60801	10.13626	123.7426	10.13626
10.15672	295.8907	10.15672	55.45041	10.15672	112.3527	10.15672	153.4704	10.15672
10.17718	475.3299	10.17718	133.1602	10.17718	12.10452	10.17718	191.2041	10.17718
10.19765	612.7747	10.19765	129.8758	10.19765	76.86355	10.19765	115.9438	10.19765
10.21811	593.2252	10.21811	144.5971	10.21811	157.6298	10.21811	272.6895	10.21811
10.23857	600.6814	10.23857	250.3241	10.23857	151.4031	10.23857	357.441	10.23857
10.25903	510.1432	10.25903	230.0568	10.25903	187.1837	10.25903	397.1984	10.25903
10.2795	518.6107	10.2795	202.7953	10.2795	216.9714	10.2795	421.9618	10.2795
10.29996	550.0839	10.29996	329.5395	10.29996	203.7664	10.29996	543.7311	10.29996
10.32042	403.5628	10.32042	299.2894	10.32042	126.5685	10.32042	494.5063	10.32042
10.34089	244.0474	10.34089	425.045	10.34089	205.3777	10.34089	464.2875	10.34089
10.36135	317.5376	10.36135	450.8063	10.36135	289.1942	10.36135	343.0746	10.36135
10.38181	174.0335	10.38181	486.5734	10.38181	388.0178	10.38181	374.8676	10.38181
10.40227	199.5351	10.40227	364.3462	10.40227	440.8486	10.40227	275.6665	10.40227
10.42274	150.0423	10.42274	182.1247	10.42274	375.6866	10.42274	174.4713	10.42274
10.4432	116.5553	10.4432	143.9089	10.4432	206.5318	10.4432	225.2821	10.4432
10.46366	99.07388	10.46366	88.69887	10.46366	209.3841	10.46366	169.0988	10.46366
10.48412	154.5982	10.48412	155.4945	10.48412	53.24363	10.48412	139.9214	10.48412
10.50459	166.1281	10.50459	118.2959	10.50459	29.11033	10.50459	86.74992	10.50459
10.52505	98.66376	10.52505	117.1031	10.52505	18.98421	10.52505	79.58438	10.52505
10.54551	190.2051	10.54551	101.9159	10.54551	16.86527	10.54551	119.4248	10.54551
10.56598	204.7521	10.56598	105.7345	10.56598	24.7535	10.56598	122.2711	10.56598
10.58644	157.3047	10.58644	65.55875	10.58644	11.64891	10.58644	168.1233	10.58644
10.6069	126.8631	10.6069	93.38876	10.6069	31.5515	10.6069	134.9814	10.6069
10.62736	200.4271	10.62736	67.2245	10.62736	34.46127	10.62736	42.84553	10.62736
10.64783	118.9968	10.64783	27.06595	10.64783	66.37822	10.64783	78.71553	10.64783
10.66829	118.5721	10.66829	52.91312	10.66829	53.30234	10.66829	107.5915	10.66829
10.68875	107.1532	10.68875	76.76602	10.68875	11.23365	10.68875	125.4733	10.68875
10.70922	102.7399	10.70922	62.62464	10.70922	-3.82787	10.70922	76.36107	10.70922
10.72968	119.3323	10.72968	12.48899	10.72968	16.11779	10.72968	180.2548	10.72968
10.75014	179.9304	10.75014	105.3591	10.75014	8.070623	10.75014	43.15438	10.75014
10.7706	171.5341	10.7706	156.2348	10.7706	33.03064	10.7706	184.0599	10.7706

10.79107	127.1436	10.79107	46.11635	10.79107	80.99783	10.79107	136.9714	10.79107
10.81153	220.7587	10.81153	137.0036	10.81153	43.97221	10.81153	21.88877	10.81153
10.83199	222.3794	10.83199	149.8965	10.83199	-73.0462	10.83199	161.8121	10.83199
10.85245	110.0059	10.85245	118.7952	10.85245	13.94248	10.85245	125.7413	10.85245
10.87292	145.638	10.87292	94.69962	10.87292	71.93839	10.87292	97.67648	10.87292
10.89338	228.2758	10.89338	85.60973	10.89338	28.94148	10.89338	148.6176	10.89338
10.91384	249.9193	10.91384	42.52558	10.91384	-0.04826	10.91384	100.5646	10.91384
10.93431	266.5685	10.93431	92.44714	10.93431	15.96918	10.93431	84.51749	10.93431
10.95477	126.2233	10.95477	71.37444	10.95477	-68.0062	10.95477	121.4763	10.95477
10.97523	218.8838	10.97523	76.30744	10.97523	126.0256	10.97523	75.44112	10.97523
10.99569	228.55	10.99569	58.24618	10.99569	38.06457	10.99569	140.4118	10.99569
11.01616	198.2219	11.01616	63.19063	11.01616	65.11073	11.01616	142.3884	11.01616
11.03662	195.8994	11.03662	124.1408	11.03662	53.16406	11.03662	13.37099	11.03662
11.05708	259.5826	11.05708	29.09671	11.05708	70.22456	11.05708	80.35946	11.05708
11.07755	148.2715	11.07755	156.0583	11.07755	71.29225	11.07755	73.35385	11.07755
11.09801	298.9661	11.09801	46.02568	11.09801	40.36712	11.09801	150.3542	11.09801
11.11847	160.6663	11.11847	68.99874	11.11847	100.4492	11.11847	122.3604	11.11847
11.13893	132.3723	11.13893	81.97753	11.13893	63.53839	11.13893	183.3726	11.13893
11.1594	112.0839	11.1594	142.9621	11.1594	108.6348	11.1594	178.3907	11.1594
11.17986	288.8011	11.17986	97.95228	11.17986	130.7384	11.17986	93.41467	11.17986
11.20032	153.5241	11.20032	19.94824	11.20032	17.84913	11.20032	170.4446	11.20032
11.22078	205.2527	11.22078	50.94991	11.22078	69.96706	11.22078	159.4805	11.22078
11.24125	176.987	11.24125	71.95731	11.24125	129.0922	11.24125	110.5222	11.24125
11.26171	297.727	11.26171	30.97044	11.26171	31.22447	11.26171	154.5699	11.26171
11.28217	251.4726	11.28217	92.98928	11.28217	72.36394	11.28217	147.6236	11.28217
11.30264	219.224	11.30264	95.01386	11.30264	18.51059	11.30264	64.68311	11.30264
11.3231	297.981	11.3231	137.0441	11.3231	28.66441	11.3231	248.7486	11.3231
11.34356	264.7437	11.34356	90.08015	11.34356	96.82542	11.34356	270.82	11.34356
11.36402	313.512	11.36402	102.1219	11.36402	27.9936	11.36402	161.8973	11.36402
11.38449	187.2861	11.38449	144.1693	11.38449	15.16896	11.38449	207.9805	11.38449
11.40495	292.0658	11.40495	40.22253	11.40495	15.3515	11.40495	203.0697	11.40495
11.42541	262.8511	11.42541	45.28143	11.42541	37.54122	11.42541	225.1648	11.42541
11.44588	167.6422	11.44588	117.346	11.44588	97.73811	11.44588	227.2658	11.44588
11.46634	262.439	11.46634	129.4164	11.46634	162.9422	11.46634	186.3728	11.46634
11.4868	291.2414	11.4868	101.4925	11.4868	75.15344	11.4868	173.4856	11.4868
11.50726	297.0495	11.50726	129.5742	11.50726	21.37187	11.50726	131.6044	11.50726
11.52773	192.8632	11.52773	138.6618	11.52773	104.5975	11.52773	213.7291	11.52773
11.54819	302.6827	11.54819	146.755	11.54819	69.83026	11.54819	172.8597	11.54819
11.56865	229.5078	11.56865	138.854	11.56865	148.0702	11.56865	177.9963	11.56865
11.58911	282.3386	11.58911	71.95864	11.58911	130.3174	11.58911	180.1388	11.58911
11.60958	262.175	11.60958	97.06905	11.60958	102.5717	11.60958	148.2872	11.60958
11.63004	310.0172	11.63004	172.1852	11.63004	72.83319	11.63004	300.4415	11.63004
11.6505	294.865	11.6505	148.307	11.6505	104.1019	11.6505	174.6018	11.6505
11.67097	265.7185	11.67097	114.4346	11.67097	88.37772	11.67097	180.7679	11.67097
11.69143	289.5777	11.69143	93.56788	11.69143	81.66076	11.69143	266.94	11.69143
11.71189	386.4426	11.71189	147.7069	11.71189	104.951	11.71189	205.118	11.71189
11.73235	299.3131	11.73235	139.8516	11.73235	89.24836	11.73235	240.302	11.73235
11.75282	217.1893	11.75282	156.0021	11.75282	50.55293	11.75282	164.4919	11.75282
11.77328	291.0712	11.77328	190.1583	11.77328	119.8647	11.77328	237.6877	11.77328
11.79374	457.9587	11.79374	173.3202	11.79374	169.1836	11.79374	171.8894	11.79374
11.81421	450.852	11.81421	201.4878	11.81421	185.5097	11.81421	185.097	11.81421
11.83467	311.7509	11.83467	77.66114	11.83467	188.843	11.83467	316.3105	11.83467
11.85513	308.6555	11.85513	137.8402	11.85513	75.18344	11.85513	216.53	11.85513
11.87559	300.5657	11.87559	167.025	11.87559	30.53108	11.87559	263.7554	11.87559
11.89606	416.4817	11.89606	150.2155	11.89606	125.8859	11.89606	249.9868	11.89606
11.91652	535.4033	11.91652	160.4118	11.91652	149.2479	11.91652	367.224	11.91652
11.93698	332.3306	11.93698	195.6137	11.93698	157.6171	11.93698	299.4672	11.93698
11.95744	361.2636	11.95744	200.8214	11.95744	173.9934	11.95744	252.7163	11.95744

11.97791	476.2022	11.97791	150.0348	11.97791	110.3769	11.97791	311.9713	11.97791
11.99837	518.1465	11.99837	263.2539	11.99837	164.7676	11.99837	338.2323	11.99837
12.01883	562.0965	12.01883	250.4788	12.01883	193.1655	12.01883	435.4991	12.01883
12.0393	651.0522	12.0393	312.7094	12.0393	314.5706	12.0393	411.7719	12.0393
12.05976	643.0136	12.05976	389.9456	12.05976	362.9828	12.05976	537.0507	12.05976
12.08022	694.9805	12.08022	432.1877	12.08022	451.4023	12.08022	633.3353	12.08022
12.10068	710.9532	12.10068	488.4354	12.10068	390.8289	12.10068	707.6259	12.10068
12.12115	587.9316	12.12115	530.6888	12.12115	373.2626	12.12115	644.9224	12.12115
12.14161	604.9157	12.14161	516.9481	12.14161	377.7036	12.14161	600.2248	12.14161
12.16207	482.9054	12.16207	570.213	12.16207	348.1517	12.16207	482.5331	12.16207
12.18254	538.9008	12.18254	453.4836	12.18254	359.607	12.18254	357.8474	12.18254
12.203	429.9019	12.203	455.7599	12.203	337.0695	12.203	341.1675	12.203
12.22346	417.9087	12.22346	350.042	12.22346	256.5392	12.22346	330.4937	12.22346
12.24392	410.9211	12.24392	201.3298	12.24392	203.016	12.24392	325.8257	12.24392
12.26439	400.9392	12.26439	250.6233	12.26439	180.5	12.26439	292.1636	12.26439
12.28485	375.963	12.28485	183.9225	12.28485	95.99124	12.28485	282.5075	12.28485
12.30531	346.9925	12.30531	203.2275	12.30531	196.4896	12.30531	263.8573	12.30531
12.32577	269.0276	12.32577	199.5382	12.32577	187.9952	12.32577	264.213	12.32577
12.34624	334.0684	12.34624	165.8546	12.34624	166.5079	12.34624	189.5747	12.34624
12.3667	404.1149	12.3667	171.1767	12.3667	144.0278	12.3667	113.9422	12.3667
12.38716	394.1671	12.38716	160.5046	12.38716	170.5549	12.38716	205.3157	12.38716
12.40763	381.2249	12.40763	87.83814	12.40763	125.0892	12.40763	275.6952	12.40763
12.42809	446.2884	12.42809	141.1774	12.42809	126.6306	12.42809	233.0805	12.42809
12.44855	399.3576	12.44855	154.5224	12.44855	138.1793	12.44855	239.4718	12.44855
12.46901	444.4325	12.46901	121.8732	12.46901	229.7351	12.46901	203.8689	12.46901
12.48948	389.513	12.48948	153.2296	12.48948	193.2981	12.48948	273.2721	12.48948
12.50994	500.5993	12.50994	170.5918	12.50994	169.8682	12.50994	217.6811	12.50994
12.5304	461.6912	12.5304	131.9597	12.5304	162.4455	12.5304	259.096	12.5304
12.55087	511.7887	12.55087	119.3334	12.55087	109.0301	12.55087	324.5169	12.55087
12.57133	531.892	12.57133	156.7127	12.57133	145.6218	12.57133	232.9437	12.57133
12.59179	523.0009	12.59179	130.0978	12.59179	267.2206	12.59179	340.3765	12.59179
12.61225	523.1155	12.61225	89.48859	12.61225	257.8267	12.61225	343.8151	12.61225
12.63272	466.2358	12.63272	165.8851	12.63272	219.4399	12.63272	250.2597	12.63272
12.65318	446.3618	12.65318	194.2873	12.65318	163.0603	12.65318	379.7102	12.65318
12.67364	416.4934	12.67364	146.6953	12.67364	226.6879	12.67364	309.1666	12.67364
12.6941	462.6307	12.6941	161.109	12.6941	244.3227	12.6941	264.6289	12.6941
12.71457	368.7737	12.71457	145.5284	12.71457	293.9646	12.71457	307.0972	12.71457
12.73503	342.9223	12.73503	207.9535	12.73503	203.6137	12.73503	272.5714	12.73503
12.75549	374.0767	12.75549	153.3844	12.75549	281.2701	12.75549	205.0515	12.75549
12.77596	368.2367	12.77596	143.821	12.77596	74.93352	12.77596	234.5376	12.77596
12.79642	279.4024	12.79642	93.26325	12.79642	142.6042	12.79642	248.0295	12.79642
12.81688	204.5738	12.81688	93.71127	12.81688	141.282	12.81688	167.5274	12.81688
12.83734	349.7508	12.83734	117.165	12.83734	144.967	12.83734	177.0312	12.83734
12.85781	308.9335	12.85781	129.6245	12.85781	81.6592	12.85781	240.541	12.85781
12.87827	385.1219	12.87827	149.0897	12.87827	60.35857	12.87827	276.0566	12.87827
12.89873	375.316	12.89873	132.5606	12.89873	91.06512	12.89873	145.5782	12.89873
12.9192	312.5157	12.9192	129.0372	12.9192	75.77884	12.9192	100.1057	12.9192
12.93966	307.7212	12.93966	156.5195	12.93966	102.4997	12.93966	169.6391	12.93966
12.96012	315.9323	12.96012	52.00762	12.96012	95.22783	12.96012	183.1785	12.96012
12.98058	273.149	12.98058	63.50142	12.98058	109.9631	12.98058	163.7237	12.98058
13.00105	344.3715	13.00105	112.0009	13.00105	99.70552	13.00105	206.2749	13.00105
13.02151	236.5996	13.02151	137.5062	13.02151	71.45514	13.02151	159.8321	13.02151
13.04197	328.8334	13.04197	92.01714	13.04197	63.21193	13.04197	243.3951	13.04197
13.06243	273.0729	13.06243	61.53383	13.06243	91.9759	13.06243	116.9641	13.06243
13.0829	308.3181	13.0829	80.05624	13.0829	68.74705	13.0829	177.539	13.0829
13.10336	323.5689	13.10336	91.58437	13.10336	15.52538	13.10336	191.1198	13.10336
13.12382	296.8254	13.12382	41.11822	13.12382	90.31088	13.12382	158.7065	13.12382
13.14429	286.0876	13.14429	22.65779	13.14429	47.10357	13.14429	170.2992	13.14429

13.16475	344.3554	13.16475	109.2031	13.16475	119.9034	13.16475	260.8977	13.16475
13.18521	265.629	13.18521	78.75411	13.18521	45.71047	13.18521	141.5023	13.18521
13.20567	268.9082	13.20567	0.310852	13.20567	41.52469	13.20567	230.1127	13.20567
13.22614	254.1931	13.22614	54.87332	13.22614	46.34609	13.22614	88.72904	13.22614
13.2466	290.4836	13.2466	79.44151	13.2466	82.17466	13.2466	178.3513	13.2466
13.26706	291.7799	13.26706	19.01541	13.26706	17.01042	13.26706	121.9795	13.26706
13.28753	301.0818	13.28753	-1.40496	13.28753	99.85335	13.28753	159.6136	13.28753
13.30799	321.3894	13.30799	73.1804	13.30799	56.70346	13.30799	202.2537	13.30799
13.32845	315.7027	13.32845	32.77147	13.32845	10.56075	13.32845	129.8997	13.32845
13.34891	274.0216	13.34891	-23.6317	13.34891	60.42521	13.34891	132.5516	13.34891
13.36938	259.3462	13.36938	73.9708	13.36938	26.29686	13.36938	130.2094	13.36938
13.38984	261.6765	13.38984	93.57903	13.38984	9.175678	13.38984	164.8731	13.38984
13.4103	326.0125	13.4103	46.193	13.4103	41.06168	13.4103	163.5428	13.4103
13.43076	211.3542	13.43076	26.81269	13.43076	71.95486	13.43076	181.2184	13.43076
13.45123	265.7015	13.45123	34.4381	13.45123	18.85522	13.45123	148.8999	13.45123
13.47169	277.0545	13.47169	56.06923	13.47169	75.76275	13.47169	162.5873	13.47169
13.49215	193.4132	13.49215	46.70609	13.49215	3.677463	13.49215	113.2807	13.49215
13.51262	198.7775	13.51262	9.348662	13.51262	56.59935	13.51262	150.98	13.51262
13.53308	230.1476	13.53308	111.997	13.53308	64.52843	13.53308	84.68517	13.53308
13.55354	210.5233	13.55354	1.650983	13.55354	64.46467	13.55354	103.3963	13.55354
13.574	164.9046	13.574	10.31073	13.574	49.4081	13.574	55.11335	13.574
13.59447	217.2917	13.59447	15.97619	13.59447	66.3587	13.59447	75.83633	13.59447
13.61493	171.6844	13.61493	40.64738	13.61493	48.31648	13.61493	85.56523	13.61493
13.63539	191.0829	13.63539	28.32429	13.63539	23.28145	13.63539	143.3	13.63539
13.65586	228.4869	13.65586	33.00692	13.65586	-4.74642	13.65586	109.0408	13.65586
13.67632	250.8967	13.67632	75.69528	13.67632	34.2329	13.67632	98.78747	13.67632
13.69678	240.3121	13.69678	26.38936	13.69678	50.2194	13.69678	76.54006	13.69678
13.71724	255.7333	13.71724	52.08916	13.71724	44.21307	13.71724	78.29858	13.71724
13.73771	178.1601	13.73771	-5.20532	13.73771	46.21392	13.73771	46.06302	13.73771
13.75817	153.5925	13.75817	14.50592	13.75817	27.22195	13.75817	130.8334	13.75817
13.77863	183.0307	13.77863	39.22289	13.77863	48.23716	13.77863	77.60967	13.77863
13.79909	183.4745	13.79909	-31.0544	13.79909	34.25954	13.79909	45.39188	13.79909
13.81956	199.924	13.81956	27.67399	13.81956	9.289106	13.81956	66.18002	13.81956
13.84002	178.3792	13.84002	55.40813	13.84002	34.32585	13.84002	97.97408	13.84002
13.86048	191.84	13.86048	-14.852	13.86048	11.36977	13.86048	96.77406	13.86048
13.88095	213.3065	13.88095	-7.10644	13.88095	-10.5791	13.88095	128.58	13.88095
13.90141	125.7787	13.90141	54.64486	13.90141	-20.5209	13.90141	13.39179	13.90141
13.92187	106.2566	13.92187	-4.59812	13.92187	34.5446	13.92187	72.20954	13.92187
13.94233	122.7402	13.94233	-2.83537	13.94233	30.61723	13.94233	75.03322	13.94233
13.9628	204.2294	13.9628	-10.0669	13.9628	41.69704	13.9628	143.8628	13.9628
13.98326	177.7243	13.98326	-7.29271	13.98326	6.784034	13.98326	72.69833	13.98326
14.00372	162.2249	14.00372	45.4872	14.00372	-40.1218	14.00372	14.53978	14.00372
14.02419	149.7312	14.02419	-2.72717	14.02419	50.97955	14.02419	79.38715	14.02419
14.04465	187.2431	14.04465	-37.9358	14.04465	5.088071	14.04465	41.24044	14.04465
14.06511	55.76071	14.06511	22.86127	14.06511	-31.7962	14.06511	27.09966	14.06511
14.08557	169.284	14.08557	56.66407	14.08557	-5.62563	14.08557	94.9648	14.08557
14.10604	188.813	14.10604	24.47259	14.10604	6.552138	14.10604	90.83586	14.10604
14.1265	148.3476	14.1265	-8.71316	14.1265	21.73709	14.1265	23.71285	14.1265
14.14696	75.88792	14.14696	-7.89319	14.14696	23.92921	14.14696	1.595756	14.14696
14.16742	104.4339	14.16742	-9.0675	14.16742	-12.8715	14.16742	79.48459	14.16742
14.18789	172.9856	14.18789	-34.2361	14.18789	-14.665	14.18789	109.3793	14.18789
14.20835	124.5429	14.20835	28.60105	14.20835	39.54867	14.20835	50.28003	14.20835
14.22881	215.1059	14.22881	10.4439	14.22881	-7.23049	14.22881	101.1866	14.22881
14.24928	91.67464	14.24928	-12.7075	14.24928	-40.0025	14.24928	145.0992	14.24928
14.26974	135.249	14.26974	14.14679	14.26974	63.23272	14.26974	110.0176	14.26974
14.2902	95.82907	14.2902	51.00681	14.2902	26.4751	14.2902	4.941978	14.2902
14.31066	180.4148	14.31066	65.87256	14.31066	33.72465	14.31066	-13.1277	14.31066
14.33113	141.0062	14.33113	-52.256	14.33113	53.98138	14.33113	31.8085	14.33113

14.35159	107.6033	14.35159	15.62122	14.35159	37.24529	14.35159	-30.2494	14.35159
14.37205	92.20603	14.37205	-38.4959	14.37205	45.51638	14.37205	-16.3013	14.37205
14.39252	58.81446	14.39252	-14.7499	14.39252	-6.20536	14.39252	107.6527	14.39252
14.41298	105.4286	14.41298	3.001794	14.41298	7.080088	14.41298	7.612612	14.41298
14.43344	110.0483	14.43344	22.75921	14.43344	5.37271	14.43344	34.57845	14.43344
14.4539	123.6738	14.4539	23.52235	14.4539	119.6725	14.4539	27.55021	14.4539
14.47437	186.3049	14.47437	-0.70879	14.47437	64.97949	14.47437	68.52789	14.47437
14.49483	153.9418	14.49483	15.06579	14.49483	22.29364	14.49483	50.5115	14.49483
14.51529	143.5842	14.51529	54.84609	14.51529	64.61498	14.51529	113.501	14.51529
14.53575	159.2324	14.53575	12.63212	14.53575	90.94349	14.53575	106.4965	14.53575
14.55622	138.8862	14.55622	75.42387	14.55622	126.2792	14.55622	153.4979	14.55622
14.57668	150.5458	14.57668	118.2213	14.57668	183.6221	14.57668	201.5052	14.57668
14.59714	192.211	14.59714	161.0245	14.59714	238.9721	14.59714	293.5184	14.59714
14.61761	213.8818	14.61761	251.8335	14.61761	290.3293	14.61761	275.5375	14.61761
14.63807	130.5584	14.63807	257.6481	14.63807	327.6937	14.63807	411.5626	14.63807
14.65853	183.2406	14.65853	409.4684	14.65853	353.0653	14.65853	533.5936	14.65853
14.67899	153.9285	14.67899	474.2945	14.67899	380.4441	14.67899	459.6305	14.67899
14.69946	221.6221	14.69946	464.1263	14.69946	363.83	14.69946	428.6734	14.69946
14.71992	129.3213	14.71992	418.9639	14.71992	280.2231	14.71992	331.7221	14.71992
14.74038	108.0262	14.74038	325.8071	14.74038	250.6234	14.74038	206.7768	14.74038
14.76085	25.73683	14.76085	192.6561	14.76085	196.0309	14.76085	40.83744	14.76085
14.78131	63.45311	14.78131	118.5108	14.78131	207.4455	14.78131	74.90398	14.78131
14.80177	97.17506	14.80177	130.3712	14.80177	85.86738	14.80177	23.97644	14.80177
14.82223	30.9027	14.82223	-28.7627	14.82223	14.29638	14.82223	-25.9452	14.82223
14.8427	6.636001	14.8427	14.10919	14.8427	-22.2674	14.8427	-5.86087	14.8427
14.86316	58.37499	14.86316	2.986774	14.86316	26.17593	14.86316	72.22936	14.86316
14.88362	10.11964	14.88362	17.87008	14.88362	-7.37353	14.88362	50.32551	14.88362
14.90408	5.869976	14.90408	3.759108	14.90408	1.084194	14.90408	38.42759	14.90408
14.92455	27.62599	14.92455	-25.3461	14.92455	15.54909	14.92455	46.53559	14.92455
14.94501	32.38767	14.94501	34.55433	14.94501	-44.9788	14.94501	27.64951	14.94501
14.96547	62.15504	14.96547	10.46053	14.96547	8.500423	14.96547	-0.23064	14.96547
14.98594	82.92808	14.98594	-0.62756	14.98594	5.986856	14.98594	19.89513	14.98594
15.0064	12.70679	15.0064	-11.7099	15.0064	-10.5195	15.0064	13.02682	15.0064
15.02686	1.491183	15.02686	20.21344	15.02686	-18.0187	15.02686	-14.8356	15.02686
15.04732	31.28125	15.04732	42.14253	15.04732	-18.5108	15.04732	-30.692	15.04732
15.06779	46.077	15.06779	-7.92267	15.06779	11.00437	15.06779	-24.768	15.06779
15.08825	-18.1216	15.08825	-27.9821	15.08825	-41.4733	15.08825	-1.8381	15.08825
15.10871	43.68551	15.10871	-17.5713	15.10871	27.05619	15.10871	-14.9023	15.10871
15.12918	61.49828	15.12918	17.84519	15.12918	-9.40713	15.12918	-24.9605	15.12918
15.14964	92.31673	15.14964	-21.7326	15.14964	22.13673	15.14964	11.98721	15.14964
15.1701	16.14086	15.1701	87.69541	15.1701	-5.31223	15.1701	-16.0592	15.1701
15.19056	-25.0293	15.19056	-12.8709	15.19056	-1.75402	15.19056	-11.0996	15.19056
15.21103	11.80613	15.21103	47.56852	15.21103	4.811377	15.21103	-18.1342	15.21103
15.23149	-11.3527	15.23149	9.013659	15.23149	11.38395	15.23149	5.837215	15.23149
15.25195	44.49411	15.25195	9.464519	15.25195	-5.0363	15.25195	8.814526	15.25195
15.27241	30.34662	15.27241	32.9211	15.27241	10.55062	15.27241	34.79776	15.27241
15.29288	53.2048	15.29288	7.383405	15.29288	36.14473	15.29288	-7.21309	15.29288
15.31334	62.06866	15.31334	-16.1486	15.31334	-24.254	15.31334	29.782	15.31334
15.3338	-0.06181	15.3338	-6.67482	15.3338	10.35448	15.3338	-39.217	15.3338
15.35427	-12.1866	15.35427	-6.19535	15.35427	-7.02989	15.35427	22.78993	15.35427
15.37473	8.694291	15.37473	-14.7102	15.37473	-43.4071	15.37473	27.80278	15.37473
15.39519	-21.4191	15.39519	-1.21924	15.39519	31.22293	15.39519	-14.1784	15.39519
15.41565	-29.5269	15.41565	-8.7226	15.41565	-24.1399	15.41565	25.84625	15.41565
15.43612	20.37101	15.43612	15.77976	15.43612	-28.4955	15.43612	1.876869	15.43612
15.45658	41.2746	15.45658	64.28784	15.45658	23.86029	15.45658	1.913414	15.45658
15.47704	25.18387	15.47704	-39.1983	15.47704	40.2233	15.47704	10.95588	15.47704
15.49751	35.09881	15.49751	-9.67882	15.49751	3.593482	15.49751	-37.9957	15.49751
15.51797	20.01943	15.51797	3.846428	15.51797	30.97085	15.51797	-11.0651	15.51797

15.53843	-14.0543	15.53843	-45.6226	15.53843	17.35539	15.53843	1.87155	15.53843
15.55889	49.87769	15.55889	16.9141	15.55889	-35.2529	15.55889	26.81407	15.55889
15.57936	61.81534	15.57936	12.45651	15.57936	46.14601	15.57936	5.762521	15.57936
15.59982	-29.2413	15.59982	-36.9954	15.59982	30.55209	15.59982	-26.2831	15.59982
15.62028	10.70767	15.62028	-11.84	15.62028	-24.0347	15.62028	49.67719	15.62028
15.64074	-29.3377	15.64074	-7.67891	15.64074	93.38578	15.64074	-0.3566	15.64074
15.66121	47.6227	15.66121	52.48789	15.66121	-3.18661	15.66121	3.615545	15.66121
15.68167	-39.4113	15.68167	-19.3396	15.68167	42.24818	15.68167	55.59361	15.68167
15.70213	1.560428	15.70213	-6.16134	15.70213	21.69015	15.70213	20.5776	15.70213
15.7226	55.53781	15.7226	-3.97738	15.7226	10.1393	15.7226	-41.4325	15.7226
15.74306	41.52087	15.74306	-15.7877	15.74306	-27.4044	15.74306	21.56334	15.74306
15.76352	42.5096	15.76352	-15.5923	15.76352	-17.0131	15.76352	20.5651	15.76352
15.78398	3.504009	15.78398	25.60886	15.78398	49.38538	15.78398	-33.4272	15.78398
15.80445	-45.4959	15.80445	9.81572	15.80445	30.79102	15.80445	18.58639	15.80445
15.82491	-3.18088	15.82491	-5.9717	15.82491	52.20384	15.82491	17.60592	15.82491
15.84537	75.13982	15.84537	41.2466	15.84537	-6.37616	15.84537	-4.36863	15.84537
15.86584	47.4662	15.86584	-6.52937	15.86584	11.05102	15.86584	-28.3373	15.86584
15.8863	82.79826	15.8863	-47.2996	15.8863	64.48537	15.8863	4.400999	15.8863
15.90676	43.13599	15.90676	24.93584	15.90676	43.92691	15.90676	63.14518	15.90676
15.92722	83.47939	15.92722	32.17703	15.92722	66.37563	15.92722	41.89528	15.92722
15.94769	97.82848	15.94769	36.42395	15.94769	116.8315	15.94769	56.6513	15.94769
15.96815	92.18324	15.96815	53.67658	15.96815	134.2946	15.96815	95.41325	15.96815
15.98861	72.54367	15.98861	120.9349	15.98861	137.7648	15.98861	81.18112	15.98861
16.00907	69.90978	16.00907	60.19902	16.00907	132.2423	16.00907	118.9549	16.00907
16.02954	25.28157	16.02954	132.4688	16.02954	99.72686	16.02954	80.73463	16.02954
16.05	47.65903	16.05	98.74435	16.05	110.2186	16.05	66.52027	16.05
16.07046	11.04217	16.07046	131.0256	16.07046	159.7176	16.07046	8.311839	16.07046
16.09093	39.43099	16.09093	50.31257	16.09093	137.2237	16.09093	51.10933	16.09093
16.11139	49.82548	16.11139	112.6053	16.11139	126.7371	16.11139	-12.0873	16.11139
16.13185	44.22565	16.13185	70.90367	16.13185	66.25755	16.13185	-10.2779	16.13185
16.15231	8.631493	16.15231	8.207807	16.15231	42.78523	16.15231	32.53733	16.15231
16.17278	15.04301	16.17278	60.51767	16.17278	14.32008	16.17278	70.35851	16.17278
16.19324	16.46021	16.19324	1.833246	16.19324	-24.1379	16.19324	28.18562	16.19324
16.2137	53.88308	16.2137	3.15455	16.2137	17.41132	16.2137	-33.9814	16.2137
16.23417	30.31163	16.23417	17.48158	16.23417	17.9677	16.23417	28.8576	16.23417
16.25463	-68.2541	16.25463	-12.1857	16.25463	45.53127	16.25463	-0.29752	16.25463
16.27509	2.185759	16.27509	-27.8472	16.27509	24.10201	16.27509	-5.44672	16.27509
16.29555	74.63133	16.29555	26.49699	16.29555	-6.32007	16.29555	2.41	16.29555
16.31602	22.08259	16.31602	44.8469	16.31602	8.265027	16.31602	10.27265	16.31602
16.33648	22.53952	16.33648	3.202538	16.33648	41.8573	16.33648	23.14122	16.33648
16.35694	-25.9979	16.35694	41.5639	16.35694	-3.54324	16.35694	21.01571	16.35694
16.3774	16.47041	16.3774	14.93098	16.3774	48.06339	16.3774	39.89613	16.3774
16.39787	62.94437	16.39787	-14.6962	16.39787	19.6772	16.39787	57.78247	16.39787
16.41833	18.424	16.41833	37.68231	16.41833	31.29819	16.41833	-19.3253	16.41833
16.43879	29.90931	16.43879	75.06656	16.43879	41.92636	16.43879	66.57291	16.43879
16.45926	16.4003	16.45926	52.45653	16.45926	45.56171	16.45926	75.47703	16.45926
16.47972	16.89696	16.47972	28.85222	16.47972	35.20423	16.47972	49.38706	16.47972
16.50018	-28.6007	16.50018	61.25364	16.50018	22.85393	16.50018	4.303017	16.50018
16.52064	-19.0687	16.52064	-3.33922	16.52064	-5.48919	16.52064	33.2249	16.52064
16.54111	14.46892	16.54111	26.07364	16.54111	3.17487	16.54111	54.1527	16.54111
16.56157	-33.9878	16.56157	11.49222	16.56157	2.846107	16.56157	22.08643	16.56157
16.58203	39.56125	16.58203	30.91653	16.58203	-25.4755	16.58203	33.02608	16.58203
16.6025	39.11593	16.6025	14.34655	16.6025	-26.9697	16.6025	-7.02835	16.6025
16.62296	29.67628	16.62296	41.7823	16.62296	36.54325	16.62296	24.92315	16.62296
16.64342	-16.7577	16.64342	18.22377	16.64342	15.06338	16.64342	39.88057	16.64342
16.66388	30.81401	16.66388	48.67097	16.66388	5.590684	16.66388	-42.1561	16.66388
16.68435	21.3914	16.68435	39.12388	16.68435	51.12517	16.68435	-29.1868	16.68435
16.70481	54.97445	16.70481	-0.41748	16.70481	21.66683	16.70481	52.78838	16.70481

16.72527	36.56319	16.72527	-0.95312	16.72527	23.21568	16.72527	3.769494	16.72527
16.74573	12.1576	16.74573	47.51697	16.74573	24.7717	16.74573	31.75653	16.74573
16.7662	-23.2423	16.7662	29.99277	16.7662	24.3349	16.7662	43.7495	16.7662
16.78666	15.36345	16.78666	22.4743	16.78666	-3.09473	16.78666	19.74838	16.78666
16.80712	2.974885	16.80712	40.96155	16.80712	-9.51717	16.80712	21.75319	16.80712
16.82759	1.591999	16.82759	1.454524	16.82759	-8.93244	16.82759	-3.23608	16.82759
16.84805	-9.78521	16.84805	2.953218	16.84805	35.65947	16.84805	76.78058	16.84805
16.86851	75.84325	16.86851	33.45763	16.86851	39.25856	16.86851	14.80316	16.86851
16.88897	69.4774	16.88897	15.96778	16.88897	-9.13517	16.88897	104.8317	16.88897
16.90944	26.11722	16.90944	17.48364	16.90944	-12.5217	16.90944	50.86609	16.90944
16.9299	9.762716	16.9299	-43.9948	16.9299	28.0989	16.9299	8.906435	16.9299
16.95036	130.4139	16.95036	-18.8638	16.95036	43.7267	16.95036	-6.04729	16.95036
16.97083	90.07074	16.97083	28.27291	16.97083	-21.6383	16.97083	-43.9951	16.97083
16.99129	2.73326	16.99129	48.41533	16.99129	-10.9962	16.99129	4.644567	16.99129
17.01175	96.40146	17.01175	-0.43652	17.01175	1.653178	17.01175	55.29015	17.01175
17.03221	38.07534	17.03221	50.71735	17.03221	5.309692	17.03221	32.94167	17.03221
17.05268	57.75489	17.05268	27.87694	17.05268	-8.02661	17.05268	-26.4009	17.05268
17.07314	17.44012	17.07314	22.04225	17.07314	22.64426	17.07314	15.26246	17.07314
17.0936	28.13102	17.0936	-12.7867	17.0936	28.32231	17.0936	-11.0683	17.0936
17.11406	58.82761	17.11406	22.39005	17.11406	7.007535	17.11406	9.60694	17.11406
17.13453	-4.47014	17.13453	-2.42747	17.13453	15.69994	17.13453	51.28807	17.13453
17.15499	-5.76221	17.15499	20.76073	17.15499	29.39953	17.15499	49.97512	17.15499
17.17545	22.95141	17.17545	13.95466	17.17545	28.10629	17.17545	82.66809	17.17545
17.19592	-11.3293	17.19592	53.15431	17.19592	-1.17977	17.19592	29.36699	17.19592
17.21638	-27.6043	17.21638	15.35968	17.21638	12.54135	17.21638	2.071812	17.21638
17.23684	-19.5718	17.23684	-11.4292	17.23684	-2.73036	17.23684	-2.21744	17.23684
17.2573	-23.5335	17.2573	30.78759	17.2573	-25.9949	17.2573	39.49923	17.2573
17.27777	29.51045	17.27777	34.01012	17.27777	2.441327	17.27777	40.22182	17.27777
17.29823	10.56006	17.29823	25.23838	17.29823	50.88472	17.29823	20.95033	17.29823
17.31869	-11.3846	17.31869	36.47237	17.31869	-0.66472	17.31869	-20.3152	17.31869
17.33916	-40.3237	17.33916	-24.2879	17.33916	-11.207	17.33916	5.425131	17.33916
17.35962	37.74297	17.35962	46.9575	17.35962	45.25795	17.35962	-25.8286	17.35962
17.38008	12.81529	17.38008	20.20865	17.38008	5.730047	17.38008	-0.07638	17.38008
17.40054	68.89329	17.40054	-10.5345	17.40054	50.20933	17.40054	15.68176	17.40054
17.42101	112.977	17.42101	-20.2719	17.42101	40.69578	17.42101	-4.55419	17.42101
17.44147	95.06632	17.44147	-21.0036	17.44147	74.18942	17.44147	-3.78421	17.44147
17.46193	112.1613	17.46193	-15.7295	17.46193	-12.3098	17.46193	-4.00831	17.46193
17.48239	168.262	17.48239	3.550224	17.48239	31.19822	17.48239	22.77352	17.48239
17.50286	219.3684	17.50286	25.83571	17.50286	28.71339	17.50286	48.56127	17.50286
17.52332	201.4805	17.52332	-41.8731	17.52332	-37.7643	17.52332	15.35494	17.52332
17.54378	307.5982	17.54378	16.42384	17.54378	0.765265	17.54378	-3.84546	17.54378
17.56425	314.7216	17.56425	13.72649	17.56425	31.30197	17.56425	-12.0399	17.56425
17.58471	311.8507	17.58471	20.03486	17.58471	12.84585	17.58471	-0.2285	17.58471
17.60517	285.9854	17.60517	25.34896	17.60517	30.39691	17.60517	-3.41113	17.60517
17.62563	244.1259	17.62563	48.66877	17.62563	31.95515	17.62563	-0.58784	17.62563
17.6461	204.272	17.6461	5.994311	17.6461	-17.4794	17.6461	5.241373	17.6461
17.66656	120.4238	17.66656	-16.6744	17.66656	25.09316	17.66656	48.07651	17.66656
17.68702	33.58125	17.68702	-9.33744	17.68702	57.67294	17.68702	3.917571	17.68702
17.70749	49.74439	17.70749	-23.9947	17.70749	41.25989	17.70749	10.76456	17.70749
17.72795	37.9132	17.72795	10.32311	17.72795	-25.146	17.72795	29.61746	17.72795
17.74841	49.0877	17.74841	8.64668	17.74841	69.45532	17.74841	-0.52371	17.74841
17.76887	-3.73214	17.76887	60.97597	17.76887	30.06381	17.76887	34.34105	17.76887
17.78934	-31.5463	17.78934	-17.689	17.78934	28.67947	17.78934	18.21173	17.78934
17.8098	62.64523	17.8098	10.65173	17.8098	-9.69768	17.8098	50.08833	17.8098
17.83026	36.84243	17.83026	25.99818	17.83026	60.93234	17.83026	41.97085	17.83026
17.85072	91.0453	17.85072	-25.6496	17.85072	69.56953	17.85072	34.8593	17.85072
17.87119	99.25385	17.87119	38.70827	17.87119	51.21391	17.87119	98.75367	17.87119
17.89165	178.4681	17.89165	58.0719	17.89165	104.8655	17.89165	46.65397	17.89165

17.91211	187.688	17.91211	47.44125	17.91211	136.5242	17.91211	133.5602	17.91211
17.93258	238.9136	17.93258	65.81632	17.93258	239.1901	17.93258	169.4723	17.93258
17.95304	244.1448	17.95304	82.19711	17.95304	295.8632	17.95304	164.3904	17.95304
17.9735	223.3817	17.9735	114.5836	17.9735	221.5435	17.9735	222.3144	17.9735
17.99396	230.6243	17.99396	165.9759	17.99396	425.2309	17.99396	287.2443	17.99396
18.01443	163.8726	18.01443	222.3738	18.01443	430.9255	18.01443	378.1801	18.01443
18.03489	160.1266	18.03489	271.7775	18.03489	443.6274	18.03489	296.1219	18.03489
18.05535	130.3862	18.05535	273.1869	18.05535	371.3363	18.05535	237.0696	18.05535
18.07582	90.65153	18.07582	329.6021	18.07582	287.0525	18.07582	182.0232	18.07582
18.09628	104.9225	18.09628	246.0229	18.09628	191.7758	18.09628	137.9827	18.09628
18.11674	44.19918	18.11674	148.4495	18.11674	199.5063	18.11674	109.9482	18.11674
18.1372	47.48152	18.1372	133.8818	18.1372	163.244	18.1372	60.91953	18.1372
18.15767	-37.2305	18.15767	60.31977	18.15767	70.98889	18.15767	44.89683	18.15767
18.17813	34.06323	18.17813	23.76351	18.17813	83.74094	18.17813	8.880055	18.17813
18.19859	-39.6374	18.19859	34.21297	18.19859	35.50017	18.19859	22.8692	18.19859
18.21905	-16.3324	18.21905	31.66816	18.21905	48.26658	18.21905	-19.1357	18.21905
18.23952	-22.0216	18.23952	11.12906	18.23952	44.04016	18.23952	-17.1347	18.23952
18.25998	6.294753	18.25998	-41.4043	18.25998	53.82092	18.25998	1.872183	18.25998
18.28044	-22.3832	18.28044	3.06804	18.28044	20.60886	18.28044	26.88502	18.28044
18.30091	-24.0554	18.30091	-11.4539	18.30091	45.40398	18.30091	37.90379	18.30091
18.32137	-3.722	18.32137	3.029907	18.32137	4.206278	18.32137	11.92847	18.32137
18.34183	15.6171	18.34183	-12.4806	18.34183	-28.9842	18.34183	2.959085	18.34183
18.36229	1.961876	18.36229	19.01466	18.36229	60.83241	18.36229	-3.00438	18.36229
18.38276	58.31233	18.38276	-13.4844	18.38276	11.65624	18.38276	0.038077	18.38276
18.40322	18.66846	18.40322	3.02231	18.40322	42.48725	18.40322	6.086458	18.40322
18.42368	70.03027	18.42368	-38.4653	18.42368	-10.6746	18.42368	31.14076	18.42368
18.44415	10.39775	18.44415	9.052846	18.44415	-17.8292	18.44415	67.20099	18.44415
18.46461	78.77091	18.46461	-17.4233	18.46461	54.02334	18.46461	2.267142	18.46461
18.48507	91.14973	18.48507	-10.8937	18.48507	27.88307	18.48507	27.33922	18.48507
18.50553	92.53425	18.50553	60.64157	18.50553	68.74997	18.50553	43.41721	18.50553
18.526	53.92444	18.526	-4.81742	18.526	8.624044	18.526	-26.4989	18.526
18.54646	28.3203	18.54646	-18.2707	18.54646	-34.4947	18.54646	7.590982	18.54646
18.56692	18.72183	18.56692	-17.7182	18.56692	-8.04185	18.56692	6.686751	18.56692
18.58738	14.12905	18.58738	1.839972	18.58738	3.418185	18.58738	-7.21156	18.58738
18.60785	53.54194	18.60785	-7.59612	18.60785	25.8854	18.60785	-24.1039	18.60785
18.62831	-12.0395	18.62831	0.973509	18.62831	-32.6402	18.62831	-8.55836	18.62831
18.64877	-23.6153	18.64877	8.548861	18.64877	9.841348	18.64877	5.993149	18.64877
18.66924	20.81467	18.66924	24.12993	18.66924	1.330092	18.66924	21.55058	18.66924
18.6897	56.25026	18.6897	-13.2833	18.6897	31.82601	18.6897	-24.8861	18.6897
18.71016	40.69154	18.71016	-20.6908	18.71016	64.32912	18.71016	30.68321	18.71016
18.73062	-52.8615	18.73062	-3.09251	18.73062	-27.1606	18.73062	15.25841	18.73062
18.75109	49.59111	18.75109	-28.4885	18.75109	9.35685	18.75109	9.839537	18.75109
18.77155	-9.95059	18.77155	11.12114	18.77155	-13.1185	18.77155	50.42659	18.77155
18.79201	-21.4866	18.79201	49.73655	18.79201	-8.5867	18.79201	1.019556	18.79201
18.81248	10.98304	18.81248	24.35768	18.81248	17.95229	18.81248	25.61845	18.81248
18.83294	42.45837	18.83294	2.984531	18.83294	60.49846	18.83294	50.22327	18.83294
18.8534	25.93937	18.8534	35.61711	18.8534	35.05181	18.8534	51.83401	18.8534
18.87386	17.42605	18.87386	16.2554	18.87386	-7.38767	18.87386	85.45068	18.87386
18.89433	76.91841	18.89433	54.89942	18.89433	45.18004	18.89433	84.07327	18.89433
18.91479	57.41644	18.91479	17.54916	18.91479	95.75492	18.91479	48.70178	18.91479
18.93525	62.92015	18.93525	16.20463	18.93525	38.33698	18.93525	76.33621	18.93525
18.95571	90.42954	18.95571	34.86581	18.95571	37.92622	18.95571	70.97657	18.95571
18.97618	150.9446	18.97618	36.53272	18.97618	26.52263	18.97618	87.62285	18.97618
18.99664	223.4653	18.99664	84.20535	18.99664	106.1262	18.99664	97.27506	18.99664
19.0171	267.9918	19.0171	105.8837	19.0171	97.737	19.0171	96.93319	19.0171
19.03757	335.5238	19.03757	109.5678	19.03757	137.355	19.03757	185.5972	19.03757
19.05803	365.0616	19.05803	103.2576	19.05803	195.9801	19.05803	237.2672	19.05803
19.07849	436.605	19.07849	152.9531	19.07849	249.6124	19.07849	239.9431	19.07849

19.09895	366.1542	19.09895	226.6543	19.09895	264.2519	19.09895	406.6249	19.09895
19.11942	335.709	19.11942	302.3613	19.11942	395.8985	19.11942	509.3127	19.11942
19.13988	269.2694	19.13988	361.074	19.13988	451.5524	19.13988	554.0063	19.13988
19.16034	211.8356	19.16034	354.7924	19.16034	411.2134	19.16034	453.7059	19.16034
19.18081	134.4074	19.18081	244.5165	19.18081	364.8816	19.18081	289.4115	19.18081
19.20127	69.9849	19.20127	232.2464	19.20127	278.557	19.20127	199.1229	19.20127
19.22173	64.56809	19.22173	201.982	19.22173	259.2395	19.22173	135.8403	19.22173
19.24219	64.15693	19.24219	138.7233	19.24219	163.9293	19.24219	56.56355	19.24219
19.26266	4.751461	19.26266	101.4703	19.26266	134.6262	19.26266	57.29277	19.26266
19.28312	1.351665	19.28312	87.22303	19.28312	63.33026	19.28312	5.027899	19.28312
19.30358	71.95754	19.30358	70.9815	19.30358	106.0415	19.30358	-4.23104	19.30358
19.32404	-2.4309	19.32404	57.74569	19.32404	42.75998	19.32404	-7.48406	19.32404
19.34451	63.18633	19.34451	14.5156	19.34451	34.4856	19.34451	-1.73116	19.34451
19.36497	20.80924	19.36497	49.29123	19.36497	47.2184	19.36497	23.02767	19.36497
19.38543	12.43783	19.38543	34.07258	19.38543	33.95838	19.38543	9.79242	19.38543
19.4059	45.07209	19.4059	-23.1403	19.4059	35.70554	19.4059	14.5631	19.4059
19.42636	35.71203	19.42636	11.65246	19.42636	37.45988	19.42636	-5.66031	19.42636
19.44682	15.35764	19.44682	62.45098	19.44682	3.221391	19.44682	19.12222	19.44682
19.46728	17.00893	19.46728	-14.7448	19.46728	0.990084	19.46728	-14.0893	19.46728
19.48775	50.6659	19.48775	3.065192	19.48775	11.76596	19.48775	10.70503	19.48775
19.50821	57.32854	19.50821	7.880881	19.50821	24.549	19.50821	-23.4947	19.50821
19.52867	7.996856	19.52867	40.70229	19.52867	0.339232	19.52867	-18.7013	19.52867
19.54914	18.67085	19.54914	29.52943	19.54914	18.13664	19.54914	-15.902	19.54914
19.5696	43.35052	19.5696	-27.6377	19.5696	11.94122	19.5696	3.903199	19.5696
19.59006	16.03587	19.59006	19.20086	19.59006	-4.24702	19.59006	13.71434	19.59006
19.61052	-13.2731	19.61052	-19.9548	19.61052	43.57192	19.61052	29.5314	19.61052
19.63099	-24.5764	19.63099	-19.1048	19.63099	19.39804	19.63099	-6.64561	19.63099
19.65145	3.660368	19.65145	5.750926	19.65145	0.231338	19.65145	19.1833	19.65145
19.67191	-12.0972	19.67191	25.61239	19.67191	37.07181	19.67191	-7.98187	19.67191
19.69237	45.15096	19.69237	15.47958	19.69237	64.91946	19.69237	-8.14111	19.69237
19.71284	35.40476	19.71284	-5.64751	19.71284	15.7743	19.71284	-25.2944	19.71284
19.7333	8.664248	19.7333	-9.76887	19.7333	10.63631	19.7333	53.55818	19.7333
19.75376	25.92941	19.75376	-1.88451	19.75376	-29.4945	19.75376	-9.5833	19.75376
19.77423	15.20024	19.77423	-37.9944	19.77423	-9.79342	19.77423	-10.7188	19.77423
19.79469	-18.5232	19.79469	2.725232	19.79469	-2.08515	19.79469	-20.8485	19.79469
19.81515	5.758945	19.81515	-39.5494	19.81515	14.63029	19.81515	42.02783	19.81515
19.83561	-15.9532	19.83561	63.18174	19.83561	-16.6471	19.83561	-17.09	19.83561
19.85608	46.34035	19.85608	-15.0814	19.85608	-0.91728	19.85608	26.7982	19.85608
19.87654	-7.36043	19.87654	-9.33887	19.87654	28.8197	19.87654	5.692264	19.87654
19.897	25.94446	19.897	12.40941	19.897	34.56385	19.897	31.59226	19.897
19.91747	1.255031	19.91747	-13.8366	19.91747	30.31519	19.91747	17.49817	19.91747
19.93793	17.57128	19.93793	-11.0769	19.93793	-17.9263	19.93793	30.41001	19.93793
19.95839	40.8932	19.95839	7.688576	19.95839	27.8394	19.95839	-9.67223	19.95839
19.97885	11.2208	19.97885	27.45975	19.97885	4.612268	19.97885	50.25146	19.97885
19.99932	7.554071	19.99932	12.23664	19.99932	40.39232	19.99932	17.18107	19.99932
20.01978	25.89302	20.01978	46.01925	20.01978	6.179543	20.01978	23.1166	20.01978
20.04024	-10.7624	20.04024	-14.1924	20.04024	51.97395	20.04024	-4.94195	20.04024
20.0607	-18.412	20.0607	14.60164	20.0607	24.77553	20.0607	-39.9946	20.0607
20.08117	32.94393	20.08117	-13.5986	20.08117	6.584293	20.08117	5.823938	20.08117
20.10163	-16.6944	20.10163	22.20692	20.10163	-4.59977	20.10163	-6.35164	20.10163
20.12209	-14.3271	20.12209	14.01815	20.12209	28.22335	20.12209	3.478716	20.12209
20.14256	6.045923	20.14256	41.83509	20.14256	42.05365	20.14256	35.31499	20.14256
20.16302	-14.5754	20.16302	18.65776	20.16302	8.89112	20.16302	38.15719	20.16302
20.18348	45.80897	20.18348	27.48616	20.18348	-16.2642	20.18348	45.00531	20.18348
20.20394	45.199	20.20394	-1.67973	20.20394	25.5876	20.20394	29.85935	20.20394
20.22441	-18.4053	20.22441	10.16011	20.22441	26.44661	20.22441	50.71932	20.22441
20.24487	49.9961	20.24487	-42.9943	20.24487	42.3128	20.24487	1.585213	20.24487
20.26533	17.40317	20.26533	-9.62792	20.26533	34.18617	20.26533	10.45703	20.26533

20.2858	15.81591	20.2858	25.74423	20.2858	13.06671	20.2858	-3.66523	20.2858
20.30626	26.23432	20.30626	-12.8779	20.30626	39.95443	20.30626	-22.7816	20.30626
20.32672	63.65842	20.32672	-36.4943	20.32672	-8.15067	20.32672	-25.9945	20.32672
20.34718	-3.91182	20.34718	13.28991	20.34718	-22.2486	20.34718	23.54676	20.34718
20.36765	39.52363	20.36765	13.07986	20.36765	-9.98957	20.36765	-7.90605	20.36765
20.38811	19.96475	20.38811	45.87554	20.38811	47.27663	20.38811	22.64707	20.38811
20.40857	-11.5885	20.40857	62.67694	20.40857	10.55002	20.40857	-29.7939	20.40857
20.42903	34.86402	20.42903	29.48406	20.42903	45.83058	20.42903	-8.22892	20.42903
20.4495	28.32217	20.4495	-3.7031	20.4495	17.11832	20.4495	40.34197	20.4495
20.46996	34.786	20.46996	30.11547	20.46996	94.41324	20.46996	-4.08122	20.46996
20.49042	-25.7445	20.49042	-8.06024	20.49042	18.71533	20.49042	17.50152	20.49042
20.51089	-24.2693	20.51089	26.76977	20.51089	44.02461	20.51089	13.09018	20.51089
20.53135	8.501663	20.53135	52.6055	20.53135	6.341058	20.53135	10.68477	20.53135
20.55181	-14.7217	20.55181	41.44696	20.55181	48.66469	20.55181	22.28527	20.55181
20.57227	-27.9393	20.57227	11.29414	20.57227	20.9955	20.57227	43.8917	20.57227
20.59274	62.84868	20.59274	3.147036	20.59274	25.33348	20.59274	5.504056	20.59274
20.6132	29.64237	20.6132	-26.9943	20.6132	23.67865	20.6132	33.12233	20.6132
20.63366	34.44173	20.63366	-2.64809	20.63366	30.03099	20.63366	-52.2535	20.63366
20.65413	29.24677	20.65413	1.703878	20.65413	14.39051	20.65413	5.376658	20.65413
20.67459	52.05749	20.67459	-0.93843	20.67459	53.75721	20.67459	25.01271	20.67459
20.69505	39.87389	20.69505	-17.575	20.69505	64.13109	20.69505	-8.34532	20.69505
20.71551	45.69596	20.71551	12.79413	20.71551	48.51214	20.71551	18.30257	20.71551
20.73598	69.52371	20.73598	49.16898	20.73598	69.90038	20.73598	25.95639	20.73598
20.75644	101.3571	20.75644	75.54957	20.75644	34.29579	20.75644	28.61613	20.75644
20.7769	119.1962	20.7769	9.935872	20.7769	73.69838	20.7769	31.2818	20.7769
20.79736	98.041	20.79736	55.3279	20.79736	79.10815	20.79736	25.95338	20.79736
20.81783	84.89145	20.81783	82.72565	20.81783	44.52509	20.81783	92.6309	20.81783
20.83829	66.74758	20.83829	45.12912	20.83829	59.94922	20.83829	120.3143	20.83829
20.85875	18.60938	20.85875	81.53832	20.85875	64.38052	20.85875	122.0037	20.85875
20.87922	26.47686	20.87922	94.95323	20.87922	83.819	20.87922	72.69898	20.87922
20.89968	54.35002	20.89968	63.37387	20.89968	85.26466	20.89968	125.4002	20.89968
20.92014	59.22885	20.92014	47.80023	20.92014	116.7175	20.92014	56.1073	20.92014
20.9406	39.11336	20.9406	73.23232	20.9406	76.17751	20.9406	24.82036	20.9406
20.96107	16.00354	20.96107	34.67012	20.96107	88.64471	20.96107	25.53933	20.96107
20.98153	-46.1006	20.98153	-4.88635	20.98153	56.11908	20.98153	11.26423	20.98153
21.00199	-11.1991	21.00199	52.5629	21.00199	39.60063	21.00199	-16.0049	21.00199
21.02246	7.708152	21.02246	2.017869	21.02246	73.08936	21.02246	32.7318	21.02246
21.04292	17.62104	21.04292	9.478563	21.04292	34.58527	21.04292	-23.5255	21.04292
21.06338	0.539607	21.06338	-1.05502	21.06338	8.088349	21.06338	28.22306	21.06338
21.08384	2.463848	21.08384	16.41712	21.08384	8.598612	21.08384	10.97758	21.08384
21.10431	43.39376	21.10431	11.89498	21.10431	16.11605	21.10431	8.738021	21.10431
21.12477	33.32936	21.12477	21.37857	21.12477	19.64067	21.12477	35.50438	21.12477
21.14523	52.27063	21.14523	13.86787	21.14523	9.172469	21.14523	32.27667	21.14523
21.16569	19.21758	21.16569	32.3629	21.16569	-1.28856	21.16569	-6.94512	21.16569
21.18616	-12.8298	21.18616	15.86365	21.18616	23.2576	21.18616	9.839015	21.18616
21.20662	1.128496	21.20662	-23.6299	21.20662	-39.1891	21.20662	-32.3709	21.20662
21.22708	1.09247	21.22708	46.88232	21.22708	27.37144	21.22708	25.42505	21.22708
21.24755	67.06212	21.24755	18.40024	21.24755	18.93913	21.24755	-7.77304	21.24755
21.26801	-9.96255	21.26801	41.92388	21.26801	18.51399	21.26801	-19.9652	21.26801
21.28847	2.018451	21.28847	11.45324	21.28847	26.09604	21.28847	3.848535	21.28847
21.30893	-39.9949	21.30893	0.988324	21.30893	14.68526	21.30893	-15.3318	21.30893
21.3294	-1.14188	21.3294	39.52913	21.3294	27.28166	21.3294	43.49381	21.3294
21.34986	4.716785	21.34986	-20.9243	21.34986	13.88524	21.34986	39.32533	21.34986
21.37032	10.58113	21.37032	6.627913	21.37032	25.496	21.37032	7.162774	21.37032
21.39079	34.45115	21.39079	-3.81411	21.39079	16.11393	21.39079	-1.99386	21.39079
21.41125	-59.6732	21.41125	8.749582	21.41125	47.73905	21.41125	11.85543	21.41125
21.43171	17.20821	21.43171	89.319	21.43171	86.37134	21.43171	39.71065	21.43171
21.45217	46.09525	21.45217	68.89414	21.45217	34.01081	21.45217	44.57179	21.45217

21.47264	63.98798	21.47264	57.47501	21.47264	-38.3425	21.47264	54.43885	21.47264
21.4931	37.88638	21.4931	43.06159	21.4931	27.31129	21.4931	49.31183	21.4931
21.51356	29.79045	21.51356	28.6539	21.51356	105.9723	21.51356	77.19074	21.51356
21.53402	46.7002	21.53402	65.25193	21.53402	9.640471	21.53402	82.07558	21.53402
21.55449	48.61563	21.55449	84.85568	21.55449	76.31583	21.55449	72.96633	21.55449
21.57495	97.53674	21.57495	38.46516	21.57495	44.99837	21.57495	86.86301	21.57495
21.59541	123.4635	21.59541	109.0804	21.59541	56.68809	21.59541	80.76561	21.59541
21.61588	101.396	21.61588	74.70127	21.61588	78.38499	21.61588	63.67414	21.61588
21.63634	139.3341	21.63634	76.32791	21.63634	69.08906	21.63634	103.5886	21.63634
21.6568	141.2779	21.6568	92.96027	21.6568	87.80031	21.6568	147.509	21.6568
21.67726	244.2274	21.67726	149.5984	21.67726	99.51874	21.67726	259.4353	21.67726
21.69773	303.1826	21.69773	217.2422	21.69773	193.2444	21.69773	299.3675	21.69773
21.71819	432.1434	21.71819	192.8917	21.71819	197.9771	21.71819	353.3056	21.71819
21.73865	435.1099	21.73865	270.547	21.73865	315.7171	21.73865	435.2497	21.73865
21.75912	534.0821	21.75912	311.2079	21.75912	394.4642	21.75912	506.1997	21.75912
21.77958	615.0599	21.77958	382.8746	21.77958	504.2186	21.77958	642.1556	21.77958
21.80004	521.0435	21.80004	521.5471	21.80004	592.98	21.80004	699.1174	21.80004
21.8205	536.0327	21.8205	622.2252	21.8205	698.7487	21.8205	774.0852	21.8205
21.84097	457.0276	21.84097	680.9091	21.84097	682.5246	21.84097	751.0589	21.84097
21.86143	441.0282	21.86143	685.5986	21.86143	753.3076	21.86143	702.0385	21.86143
21.88189	382.0344	21.88189	712.2939	21.88189	626.0978	21.88189	555.024	21.88189
21.90235	381.0464	21.90235	690.995	21.90235	535.8952	21.90235	459.0155	21.90235
21.92282	452.0639	21.92282	562.7017	21.92282	564.6998	21.92282	381.0128	21.92282
21.94328	471.0872	21.94328	438.4142	21.94328	428.5115	21.94328	391.0161	21.94328
21.96374	543.1162	21.96374	437.1324	21.96374	422.3305	21.96374	340.0254	21.96374
21.98421	615.1508	21.98421	404.8563	21.98421	523.1566	21.98421	362.0405	21.98421
22.00467	689.1911	22.00467	368.586	22.00467	605.9899	22.00467	446.0616	22.00467
22.02513	887.2371	22.02513	390.3214	22.02513	661.8303	22.02513	536.0886	22.02513
22.04559	1003.289	22.04559	512.0624	22.04559	811.6779	22.04559	608.1215	22.04559
22.06606	1042.346	22.06606	589.8093	22.06606	1237.533	22.06606	827.1603	22.06606
22.08652	1150.409	22.08652	663.5618	22.08652	1537.395	22.08652	1058.205	22.08652
22.10698	1185.478	22.10698	884.32	22.10698	1878.264	22.10698	1511.256	22.10698
22.12745	964.5521	22.12745	1111.084	22.12745	2215.14	22.12745	1716.312	22.12745
22.14791	793.6321	22.14791	1381.854	22.14791	2249.024	22.14791	1768.375	22.14791
22.16837	696.7178	22.16837	1628.629	22.16837	2022.915	22.16837	1839.443	22.16837
22.18883	503.8092	22.18883	1651.41	22.18883	1807.812	22.18883	1538.518	22.18883
22.2093	401.9063	22.2093	1443.197	22.2093	1534.717	22.2093	1186.598	22.2093
22.22976	214.009	22.22976	1190.99	22.22976	1210.63	22.22976	886.6843	22.22976
22.25022	215.1175	22.25022	990.788	22.25022	920.5491	22.25022	588.7765	22.25022
22.27068	148.2316	22.27068	688.592	22.27068	627.4757	22.27068	330.8745	22.27068
22.29115	104.3513	22.29115	473.4018	22.29115	411.4095	22.29115	222.9785	22.29115
22.31161	138.4768	22.31161	325.2173	22.31161	339.3504	22.31161	163.0885	22.31161
22.33207	46.6079	22.33207	201.0385	22.33207	203.2986	22.33207	105.2043	22.33207
22.35254	42.74469	22.35254	126.8654	22.35254	165.2539	22.35254	63.32609	22.35254
22.373	83.88717	22.373	150.6981	22.373	109.2164	22.373	71.45379	22.373
22.39346	28.03532	22.39346	99.53643	22.39346	63.18604	22.39346	49.5874	22.39346
22.41392	34.18914	22.41392	53.38052	22.41392	58.16288	22.41392	72.72694	22.41392
22.43439	39.34864	22.43439	2.230335	22.43439	65.1469	22.43439	18.87241	22.43439
22.45485	-21.4862	22.45485	37.08587	22.45485	76.13811	22.45485	25.0238	22.45485
22.47531	54.68468	22.47531	49.94712	22.47531	22.13648	22.47531	7.181106	22.47531
22.49578	15.86121	22.49578	3.814103	22.49578	23.14204	22.49578	22.34434	22.49578
22.51624	7.043413	22.51624	45.68681	22.51624	-4.84523	22.51624	41.5135	22.51624
22.5367	12.2313	22.5367	33.56523	22.5367	37.17469	22.5367	-6.31142	22.5367
22.55716	-6.57515	22.55716	13.44937	22.55716	-18.7982	22.55716	29.86958	22.55716
22.57763	2.62409	22.57763	34.33924	22.57763	22.23605	22.57763	2.056512	22.57763
22.59809	15.829	22.59809	3.23483	22.59809	4.277497	22.59809	-14.7506	22.59809
22.61855	-24.9604	22.61855	23.13614	22.61855	16.32612	22.61855	-5.55186	22.61855
22.63901	2.255853	22.63901	-22.9568	22.63901	-1.61807	22.63901	48.65284	22.63901

22.65948	9.477793	22.65948	9.955934	22.65948	56.44491	22.65948	8.863459	22.65948
22.67994	20.70541	22.67994	56.87441	22.67994	29.51507	22.67994	-5.92	22.67994
22.7004	18.9387	22.7004	3.798615	22.7004	4.592409	22.7004	23.30247	22.7004
22.72087	15.17767	22.72087	-3.27146	22.72087	-5.32307	22.72087	19.53087	22.72087
22.74133	11.42231	22.74133	12.66419	22.74133	24.76862	22.74133	6.76518	22.74133
22.76179	0.672634	22.76179	-25.3944	22.76179	-13.1325	22.76179	-24.9946	22.76179
22.78225	40.92863	22.78225	9.552644	22.78225	-15.0265	22.78225	-6.02829	22.78225
22.80272	7.190303	22.80272	-36.4945	22.80272	44.08678	22.80272	-5.05608	22.80272
22.82318	-22.5423	22.82318	-8.62118	22.82318	27.20718	22.82318	-14.0779	22.82318
22.84364	-24.2693	22.84364	-13.7421	22.84364	34.33477	22.84364	-22.0939	22.84364
22.86411	-5.42031	22.86411	25.1427	22.86411	17.46953	22.86411	3.896104	22.86411
22.88457	16.43438	22.88457	16.03323	22.88457	12.61148	22.88457	-12.108	22.88457
22.90503	-21.7053	22.90503	-61.0705	22.90503	-6.2394	22.90503	16.89384	22.90503
22.92549	-20.8392	22.92549	27.83144	22.92549	-21.0831	22.92549	-16.0984	22.92549
22.94596	11.03251	22.94596	11.73914	22.94596	-27.9196	22.94596	-10.0847	22.94596
22.96642	-16.0901	22.96642	-26.3474	22.96642	38.25103	22.96642	-23.0651	22.96642
22.98688	-1.20703	22.98688	6.571688	22.98688	-17.5711	22.98688	-8.18868	22.98688
23.00734	-11.3183	23.00734	-2.50345	23.00734	-10.3861	23.00734	45.69368	23.00734
23.02781	-17.4239	23.02781	5.427129	23.02781	24.80606	23.02781	26.58197	23.02781
23.04827	-11.5238	23.04827	49.36343	23.04827	-34.9946	23.04827	3.476187	23.04827
23.06873	19.38201	23.06873	-12.6945	23.06873	-23.9946	23.06873	3.376323	23.06873
23.0892	-15.7065	23.0892	-35.7468	23.0892	35.70841	23.0892	-4.71762	23.0892
23.10966	-12.7894	23.10966	19.20668	23.10966	-11.5814	23.10966	46.19437	23.10966
23.13012	35.1334	23.13012	2.165873	23.13012	60.1359	23.13012	51.11227	23.13012
23.15058	14.06188	23.15058	36.13079	23.15058	-17.1396	23.15058	-11.9639	23.15058
23.17105	13.99603	23.17105	-34.8986	23.17105	16.59211	23.17105	28.96586	23.17105
23.19151	29.93586	23.19151	-26.9222	23.19151	16.33098	23.19151	4.901535	23.19151
23.21197	5.881372	23.21197	37.05987	23.21197	14.07702	23.21197	8.843136	23.21197
23.23244	65.83256	23.23244	-19.9523	23.23244	-8.16975	23.23244	-23.2093	23.23244
23.2529	23.78942	23.2529	10.0412	23.2529	-15.4093	23.2529	45.74411	23.2529
23.27336	43.75195	23.27336	59.04045	23.27336	2.358237	23.27336	21.70348	23.27336
23.29382	46.72017	23.29382	-20.9546	23.29382	13.133	23.29382	30.66877	23.29382
23.31429	52.69405	23.31429	-12.9439	23.31429	2.914937	23.31429	51.63999	23.31429
23.33475	85.67362	23.33475	21.07253	23.33475	9.704054	23.33475	12.61713	23.33475
23.35521	43.65886	23.35521	78.09467	23.35521	45.50035	23.35521	61.6002	23.35521
23.37567	46.64978	23.37567	39.12253	23.37567	33.30382	23.37567	44.58918	23.37567
23.39614	51.64637	23.39614	53.15612	23.39614	10.11447	23.39614	41.5841	23.39614
23.4166	39.64864	23.4166	20.19542	23.4166	51.9323	23.4166	11.58493	23.4166
23.43706	48.65659	23.43706	14.24045	23.43706	20.75731	23.43706	64.59169	23.43706
23.45753	-12.3298	23.45753	66.2912	23.45753	32.5895	23.45753	14.60437	23.45753
23.47799	-24.3105	23.47799	25.34767	23.47799	45.42886	23.47799	-12.377	23.47799
23.49845	9.195527	23.49845	49.40987	23.49845	25.2754	23.49845	-6.35249	23.49845
23.51891	27.70722	23.51891	26.47778	23.51891	-14.8709	23.51891	20.67796	23.51891
23.53938	-32.7754	23.53938	-4.44858	23.53938	3.990025	23.53938	19.71433	23.53938
23.55984	-8.25236	23.55984	43.63078	23.55984	7.858101	23.55984	-30.2434	23.55984
23.5803	29.27636	23.5803	-18.2841	23.5803	-15.2666	23.5803	4.804857	23.5803
23.60077	35.81076	23.60077	-21.1933	23.60077	-5.38421	23.60077	-10.141	23.60077
23.62123	31.35084	23.62123	-16.0968	23.62123	-29.4946	23.62123	-6.08093	23.62123
23.64169	35.89659	23.64169	-27.9945	23.64169	6.365032	23.64169	2.985066	23.64169
23.66215	22.44802	23.66215	16.70189	23.66215	35.23184	23.66215	6.056983	23.66215
23.68262	-36.9949	23.68262	-43.596	23.68262	28.10583	23.68262	11.13482	23.68262
23.70308	-21.4801	23.70308	-12.8881	23.70308	-18.013	23.70308	11.21859	23.70308
23.72354	-17.9597	23.72354	30.82554	23.72354	-17.1247	23.72354	-38.6917	23.72354
23.744	-24.4336	23.744	28.54486	23.744	-13.2291	23.744	-0.59612	23.744
23.76447	-8.90298	23.76447	15.26991	23.76447	21.67355	23.76447	-37.4946	23.76447
23.78493	53.6333	23.78493	28.00068	23.78493	10.58343	23.78493	0.939162	23.78493
23.80539	-12.8247	23.80539	4.737173	23.80539	-12.4995	23.80539	58.37883	23.80539
23.82586	0.722888	23.82586	-6.52061	23.82586	4.424718	23.82586	-26.1756	23.82586

23.84632	35.2762	23.84632	-9.77268	23.84632	18.35613	23.84632	-5.72406	23.84632
23.86678	89.83518	23.86678	34.98098	23.86678	9.294719	23.86678	14.73338	23.86678
23.88724	45.39984	23.88724	5.740367	23.88724	26.24049	23.88724	45.19674	23.88724
23.90771	12.97018	23.90771	32.50547	23.90771	39.19343	23.90771	-4.33398	23.90771
23.92817	30.54619	23.92817	31.2763	23.92817	42.15356	23.92817	20.14123	23.92817
23.94863	72.12788	23.94863	14.05285	23.94863	32.12086	23.94863	30.62237	23.94863
23.9691	112.7152	23.9691	63.83512	23.9691	81.09534	23.9691	59.10942	23.9691
23.98956	86.30829	23.98956	59.62311	23.98956	102.077	23.98956	24.6024	23.98956
24.01002	102.907	24.01002	123.4168	24.01002	128.0658	24.01002	113.1013	24.01002
24.03048	194.5114	24.03048	110.2163	24.03048	65.06185	24.03048	78.60613	24.03048
24.05095	207.1215	24.05095	101.0214	24.05095	134.065	24.05095	88.11688	24.05095
24.07141	249.7372	24.07141	132.8323	24.07141	140.0754	24.07141	67.63355	24.07141
24.09187	357.3586	24.09187	155.6489	24.09187	172.093	24.09187	139.1561	24.09187
24.11233	338.9857	24.11233	138.4712	24.11233	192.1177	24.11233	178.6847	24.11233
24.1328	443.6185	24.1328	231.2993	24.1328	281.1496	24.1328	220.2191	24.1328
24.15326	526.257	24.15326	315.1331	24.15326	297.1887	24.15326	304.7595	24.15326
24.17372	638.9011	24.17372	374.9726	24.17372	505.235	24.17372	460.3058	24.17372
24.19419	655.5509	24.19419	478.8178	24.19419	597.2884	24.19419	622.858	24.19419
24.21465	552.2064	24.21465	642.6687	24.21465	769.349	24.21465	769.4161	24.21465
24.23511	599.8676	24.23511	780.5254	24.23511	908.4168	24.23511	935.9802	24.23511
24.25557	533.5344	24.25557	944.3878	24.25557	1001.492	24.25557	946.5502	24.25557
24.27604	403.2069	24.27604	908.2559	24.27604	1007.574	24.27604	991.1261	24.27604
24.2965	281.885	24.2965	932.1297	24.2965	939.6633	24.2965	814.7079	24.2965
24.31696	194.5689	24.31696	851.0092	24.31696	863.7598	24.31696	586.2957	24.31696
24.33743	142.2584	24.33743	748.8945	24.33743	765.8635	24.33743	459.8893	24.33743
24.35789	111.9537	24.35789	559.7855	24.35789	600.9743	24.35789	253.4889	24.35789
24.37835	76.65455	24.37835	424.6822	24.37835	403.0924	24.37835	187.0945	24.37835
24.39881	29.36111	24.39881	237.5847	24.39881	306.2176	24.39881	182.7059	24.39881
24.41928	29.07335	24.41928	221.4928	24.41928	218.35	24.41928	99.32329	24.41928
24.43974	18.79127	24.43974	176.4067	24.43974	191.4896	24.43974	70.94658	24.43974
24.4602	-14.4851	24.4602	112.3263	24.4602	96.63633	24.4602	52.5758	24.4602
24.48066	-13.7559	24.48066	81.25166	24.48066	115.7903	24.48066	41.21094	24.48066
24.50113	78.97908	24.50113	72.1827	24.50113	60.95138	24.50113	5.852009	24.50113
24.52159	-10.2803	24.52159	42.11948	24.52159	50.11967	24.52159	24.499	24.52159
24.54205	8.465997	24.54205	48.06197	24.54205	66.29514	24.54205	75.15191	24.54205
24.56252	45.21797	24.56252	75.01019	24.56252	25.47779	24.56252	17.81075	24.56252
24.58298	12.97562	24.58298	44.96413	24.58298	51.66761	24.58298	-7.5245	24.58298
24.60344	42.73895	24.60344	14.9238	24.60344	18.86462	24.60344	13.14619	24.60344
24.6239	10.50795	24.6239	56.88918	24.6239	47.0688	24.6239	-20.1772	24.6239
24.64437	21.28263	24.64437	3.860287	24.64437	45.28016	24.64437	-29.4947	24.64437
24.66483	34.06298	24.66483	-14.1629	24.66483	-24.5013	24.66483	20.99677	24.66483
24.68529	8.849013	24.68529	3.819669	24.68529	36.72442	24.68529	-10.5059	24.68529
24.70576	2.640721	24.70576	12.80794	24.70576	15.95731	24.70576	20.99742	24.70576
24.72622	-17.5619	24.72622	14.80194	24.72622	29.19738	24.72622	7.506636	24.72622
24.74668	-24.7588	24.74668	-6.19834	24.74668	-45.5554	24.74668	24.02177	24.74668
24.76714	7.869641	24.76714	4.807101	24.76714	-8.30094	24.76714	-16.4572	24.76714
24.78761	20.5038	24.78761	20.81826	24.78761	-33.0393	24.78761	9.069817	24.78761
24.80807	16.14363	24.80807	-10.1649	24.80807	28.22946	24.80807	-17.3973	24.80807
24.82853	75.78913	24.82853	18.85776	24.82853	-28.4946	24.82853	8.141553	24.82853
24.84899	45.44032	24.84899	-24.1139	24.84899	-28.3778	24.84899	-18.3137	24.84899
24.86946	29.09717	24.86946	-7.07986	24.86946	55.74622	24.86946	52.23698	24.86946
24.88992	28.75971	24.88992	-22.0401	24.88992	16.87739	24.88992	8.793585	24.88992
24.91038	53.42792	24.91038	-46.9946	24.91038	-10.9843	24.91038	25.35611	24.91038
24.93085	51.10181	24.93085	21.78316	24.93085	22.16126	24.93085	-6.07544	24.93085
24.95131	55.78137	24.95131	21.56663	24.95131	7.313958	24.95131	-8.50107	24.95131
24.97177	77.46661	24.97177	-37.6442	24.97177	-4.52616	24.97177	3.079222	24.97177
24.99223	157.1575	24.99223	-13.8493	24.99223	0.640896	24.99223	-18.3346	24.99223
25.0127	180.8541	25.0127	12.95136	25.0127	3.815132	25.0127	-5.74242	25.0127

25.03316	182.5564	25.03316	-19.2423	25.03316	10.99655	25.03316	-60.1444	25.03316
25.05362	195.2643	25.05362	34.5698	25.05362	13.18514	25.05362	13.45964	25.05362
25.07409	235.978	25.07409	8.387601	25.07409	36.38091	25.07409	18.06955	25.07409
25.09455	281.6972	25.09455	18.21112	25.09455	27.58386	25.09455	23.68538	25.09455
25.11501	312.4222	25.11501	-32.9596	25.11501	7.793985	25.11501	-0.69286	25.11501
25.13547	323.1529	25.13547	-6.12466	25.13547	18.01129	25.13547	22.93482	25.13547
25.15594	381.8892	25.15594	-13.284	25.15594	28.23577	25.15594	38.56843	25.15594
25.1764	372.6312	25.1764	7.562443	25.1764	3.467435	25.1764	-21.792	25.1764
25.19686	346.3789	25.19686	-72.5854	25.19686	15.70628	25.19686	5.853408	25.19686
25.21732	308.1322	25.21732	26.27244	25.21732	23.95229	25.21732	37.50478	25.21732
25.23779	229.8913	25.23779	-8.86399	25.23779	23.20549	25.23779	-17.8379	25.23779
25.25825	191.656	25.25825	-51.9947	25.25825	33.46586	25.25825	16.82531	25.25825
25.27871	213.4263	25.27871	9.6488	25.27871	49.73341	25.27871	0.494452	25.27871
25.29918	122.2024	25.29918	-29.702	25.29918	17.00815	25.29918	1.169522	25.29918
25.31964	129.9841	25.31964	0.952932	25.31964	53.29005	25.31964	17.85052	25.31964
25.3401	121.7716	25.3401	55.61358	25.3401	29.57914	25.3401	-18.4626	25.3401
25.36056	104.5646	25.36056	-23.72	25.36056	27.87541	25.36056	-23.7697	25.36056
25.38103	101.3634	25.38103	44.95205	25.38103	65.17885	25.38103	7.564219	25.38103
25.40149	43.16784	25.40149	51.62986	25.40149	26.48947	25.40149	14.90409	25.40149
25.42195	113.978	25.42195	64.3134	25.42195	3.807271	25.42195	31.24988	25.42195
25.44242	156.7937	25.44242	105.0027	25.44242	51.13225	25.44242	55.6016	25.44242
25.46288	176.6152	25.46288	48.69765	25.46288	79.4644	25.46288	80.95924	25.46288
25.48334	172.4424	25.48334	103.3984	25.48334	46.80374	25.48334	66.32281	25.48334
25.5038	256.2752	25.5038	126.1048	25.5038	50.15025	25.5038	124.6923	25.5038
25.52427	382.1137	25.52427	119.8169	25.52427	60.50394	25.52427	83.0677	25.52427
25.54473	400.9579	25.54473	119.5348	25.54473	117.8648	25.54473	120.449	25.54473
25.56519	485.8077	25.56519	179.2584	25.56519	187.2329	25.56519	240.8363	25.56519
25.58565	561.6632	25.58565	166.9877	25.58565	197.6081	25.58565	174.2295	25.58565
25.60612	550.5244	25.60612	144.7228	25.60612	215.9905	25.60612	255.6286	25.60612
25.62658	539.3913	25.62658	194.4635	25.62658	302.3801	25.62658	288.0336	25.62658
25.64704	477.2639	25.64704	190.21	25.64704	393.7768	25.64704	194.4446	25.64704
25.66751	415.1421	25.66751	302.9622	25.66751	440.1808	25.66751	181.8614	25.66751
25.68797	334.026	25.68797	223.7201	25.68797	511.5919	25.68797	160.2842	25.68797
25.70843	187.9156	25.70843	278.4838	25.70843	453.0102	25.70843	135.713	25.70843
25.72889	142.8108	25.72889	194.2532	25.72889	375.4356	25.72889	109.1476	25.72889
25.74936	50.71176	25.74936	199.0283	25.74936	353.8683	25.74936	86.58817	25.74936
25.76982	46.61837	25.76982	191.8091	25.76982	297.3081	25.76982	29.03467	25.76982
25.79028	68.53066	25.79028	156.5956	25.79028	249.7551	25.79028	-14.5129	25.79028
25.81075	54.44862	25.81075	61.38788	25.81075	157.2093	25.81075	39.94542	25.81075
25.83121	45.37225	25.83121	100.1859	25.83121	52.67068	25.83121	21.40969	25.83121
25.85167	44.30157	25.85167	55.98957	25.85167	58.13922	25.85167	5.879872	25.85167
25.87213	5.23656	25.87213	12.799	25.87213	73.61494	25.87213	23.35598	25.87213
25.8926	18.17723	25.8926	32.61416	25.8926	43.09784	25.8926	-3.16199	25.8926
25.91306	84.12357	25.91306	30.43503	25.91306	23.58792	25.91306	-11.674	25.91306
25.93352	58.07559	25.93352	5.261624	25.93352	42.08517	25.93352	8.819852	25.93352
25.95398	29.03329	25.95398	34.09395	25.95398	62.5896	25.95398	41.31966	25.95398
25.97445	7.996657	25.97445	47.93198	25.97445	2.101212	25.97445	18.82538	25.97445
25.99491	6.965705	25.99491	35.77575	25.99491	24.62	25.99491	4.337033	25.99491
26.01537	22.94043	26.01537	35.62523	26.01537	51.14597	26.01537	-24.1454	26.01537
26.03584	55.92083	26.03584	5.480441	26.03584	9.679111	26.03584	-28.1697	26.03584
26.0563	65.90691	26.0563	53.34137	26.0563	37.21944	26.0563	40.81191	26.0563
26.07676	75.89866	26.07676	57.20802	26.07676	82.76694	26.07676	13.79944	26.07676
26.09722	96.89609	26.09722	95.0804	26.09722	125.3216	26.09722	39.7929	26.09722
26.11769	90.89919	26.11769	56.9585	26.11769	164.8835	26.11769	79.79228	26.11769
26.13815	69.90797	26.13815	143.8423	26.13815	164.4525	26.13815	107.7976	26.13815
26.15861	30.92243	26.15861	115.7319	26.15861	203.0287	26.15861	82.80882	26.15861
26.17908	72.94256	26.17908	101.6271	26.17908	205.6121	26.17908	167.826	26.17908
26.19954	112.9684	26.19954	183.5281	26.19954	218.2027	26.19954	115.8491	26.19954

26.22	181.9999	26.22	113.4348	26.22	210.8004	26.22	154.8781	26.22
26.24046	247.037	26.24046	144.3472	26.24046	125.4054	26.24046	129.913	26.24046
26.26093	315.0799	26.26093	197.2654	26.26093	228.0175	26.26093	125.9538	26.26093
26.28139	312.1284	26.28139	157.1893	26.28139	185.6367	26.28139	236.0006	26.28139
26.30185	400.1826	26.30185	200.1189	26.30185	193.2632	26.30185	314.0533	26.30185
26.32231	520.2424	26.32231	268.0542	26.32231	278.8969	26.32231	402.1119	26.32231
26.34278	574.308	26.34278	332.9952	26.34278	337.5377	26.34278	509.1765	26.34278
26.36324	682.3792	26.36324	444.942	26.36324	492.1857	26.36324	689.2469	26.36324
26.3837	780.4561	26.3837	648.8945	26.3837	705.8408	26.3837	919.3233	26.3837
26.40417	789.5386	26.40417	742.8527	26.40417	1069.503	26.40417	1192.406	26.40417
26.42463	753.6269	26.42463	980.8167	26.42463	1270.173	26.42463	1289.494	26.42463
26.44509	649.7208	26.44509	1224.786	26.44509	1501.849	26.44509	1388.588	26.44509
26.46555	631.8204	26.46555	1285.762	26.46555	1613.533	26.46555	1278.688	26.46555
26.48602	529.9257	26.48602	1368.743	26.48602	1573.224	26.48602	1147.794	26.48602
26.50648	430.0367	26.50648	1302.73	26.50648	1444.923	26.50648	974.906	26.50648
26.52694	357.1533	26.52694	1182.722	26.52694	1146.628	26.52694	757.0239	26.52694
26.54741	298.2756	26.54741	905.7204	26.54741	913.3406	26.54741	637.1476	26.54741
26.56787	333.4036	26.56787	754.7244	26.56787	662.0604	26.56787	441.2773	26.56787
26.58833	270.5372	26.58833	605.7341	26.58833	464.7874	26.58833	301.413	26.58833
26.60879	333.6765	26.60879	376.7495	26.60879	383.5215	26.60879	260.5545	26.60879
26.62926	360.8216	26.62926	250.7707	26.62926	216.2628	26.62926	165.702	26.62926
26.64972	318.9723	26.64972	169.7976	26.64972	135.0113	26.64972	111.8554	26.64972
26.67018	448.1286	26.67018	115.8302	26.67018	164.767	26.67018	92.01468	26.67018
26.69064	393.2906	26.69064	116.8685	26.69064	111.5298	26.69064	114.1799	26.69064
26.71111	393.4583	26.71111	87.91252	26.71111	145.2999	26.71111	68.35107	26.71111
26.73157	364.6317	26.73157	108.9623	26.73157	60.07706	26.73157	77.52816	26.73157
26.75203	322.8108	26.75203	65.01778	26.75203	104.8614	26.75203	33.71117	26.75203
26.7725	265.9955	26.7725	36.07899	26.7725	64.65299	26.7725	76.90011	26.7725
26.79296	192.186	26.79296	57.14592	26.79296	84.45174	26.79296	53.09496	26.79296
26.81342	156.3821	26.81342	55.21858	26.81342	98.25765	26.81342	36.29574	26.81342
26.83388	149.5838	26.83388	77.29695	26.83388	39.07074	26.83388	31.50244	26.83388
26.85435	53.79129	26.85435	5.381053	26.85435	14.89102	26.85435	24.71507	26.85435
26.87481	39.00441	26.87481	-41.5291	26.87481	17.71847	26.87481	-6.06638	26.87481
26.89527	49.22321	26.89527	42.56642	26.89527	31.55309	26.89527	13.15809	26.89527
26.91574	57.44768	26.91574	-21.3323	26.91574	31.3949	26.91574	-6.61151	26.91574
26.9362	30.67783	26.9362	36.77468	26.9362	11.24389	26.9362	-15.3752	26.9362
26.95666	-8.08634	26.95666	-22.1126	26.95666	14.10005	26.95666	13.86706	26.95666
26.97712	42.15516	26.97712	31.00582	26.97712	-13.0366	26.97712	6.115222	26.97712
26.99759	-31.5977	26.99759	-23.87	26.99759	-24.1661	26.99759	3.369313	26.99759
27.01805	20.6552	27.01805	56.25986	27.01805	-17.0663	27.01805	37.62933	27.01805
27.03851	17.91373	27.03851	5.39546	27.03851	39.04067	27.03851	1.895265	27.03851
27.05897	29.17794	27.05897	14.53678	27.05897	-32.8452	27.05897	20.16713	27.05897
27.07944	63.44782	27.07944	-20.3162	27.07944	3.276146	27.07944	35.44491	27.07944
27.0999	18.72338	27.0999	-10.1634	27.0999	33.40465	27.0999	-2.27138	27.0999
27.12036	-28.9954	27.12036	25.99509	27.12036	16.54034	27.12036	41.01825	27.12036
27.14083	-17.9857	27.14083	-1.8407	27.14083	2.683196	27.14083	-18.6862	27.14083
27.16129	19.02972	27.16129	17.32924	27.16129	7.833235	27.16129	-10.3847	27.16129
27.18175	43.05078	27.18175	-33.4951	27.18175	21.99045	27.18175	3.922684	27.18175
27.20221	-0.92249	27.20221	-16.4034	27.20221	28.15485	27.20221	32.23601	27.20221
27.22268	-1.89007	27.22268	5.694072	27.22268	57.32642	27.22268	-3.44474	27.22268
27.24314	-13.852	27.24314	9.797246	27.24314	13.50518	27.24314	42.88043	27.24314
27.2636	26.19179	27.2636	0.906142	27.2636	17.69111	27.2636	-9.78847	27.2636
27.28407	28.24123	27.28407	-16.9792	27.28407	70.88422	27.28407	26.54854	27.28407
27.30453	39.29635	27.30453	35.1411	27.30453	27.0845	27.30453	26.89149	27.30453
27.32499	-3.64286	27.32499	45.26717	27.32499	53.29197	27.32499	0.240353	27.32499
27.34545	-6.57639	27.34545	-24.601	27.34545	46.50661	27.34545	19.59514	27.34545
27.36592	66.49576	27.36592	-0.46354	27.36592	37.72843	27.36592	-12.0441	27.36592
27.38638	29.57359	27.38638	-35.3203	27.38638	99.95743	27.38638	27.32249	27.38638

27.40684	36.65709	27.40684	4.82864	27.40684	68.19361	27.40684	-16.3049	27.40684
27.4273	92.74626	27.4273	27.98332	27.4273	40.43696	27.4273	16.07353	27.4273
27.44777	78.84111	27.44777	46.14371	27.44777	-0.3125	27.44777	80.45794	27.44777
27.46823	150.9416	27.46823	-4.69017	27.46823	17.94521	27.46823	109.8483	27.46823
27.48869	148.0479	27.48869	-14.5183	27.48869	-20.7899	27.48869	104.2445	27.48869
27.50916	224.1597	27.50916	61.65924	27.50916	86.48217	27.50916	123.6467	27.50916
27.52962	220.2773	27.52962	47.84252	27.52962	81.76141	27.52962	117.0548	27.52962
27.55008	335.4005	27.55008	86.03153	27.55008	103.0478	27.55008	139.4688	27.55008
27.57054	480.5294	27.57054	117.2263	27.57054	129.3414	27.57054	146.8888	27.57054
27.59101	479.664	27.59101	93.42671	27.59101	120.6422	27.59101	189.3146	27.59101
27.61147	532.8043	27.61147	89.63289	27.61147	134.9502	27.61147	179.7464	27.61147
27.63193	688.9502	27.63193	118.8448	27.63193	194.2653	27.63193	290.1841	27.63193
27.6524	801.1018	27.6524	153.0624	27.6524	270.5876	27.6524	326.6278	27.6524
27.67286	950.2591	27.67286	227.2858	27.67286	298.9171	27.67286	426.0774	27.67286
27.69332	937.4221	27.69332	292.5148	27.69332	421.2538	27.69332	545.5328	27.69332
27.71378	1024.591	27.71378	341.7496	27.71378	530.5977	27.71378	760.9943	27.71378
27.73425	1048.765	27.73425	397.9901	27.73425	574.9487	27.73425	778.4616	27.73425
27.75471	1028.945	27.75471	423.2363	27.75471	607.3069	27.75471	769.9349	27.75471
27.77517	986.1307	27.77517	512.4883	27.77517	605.6723	27.77517	697.4141	27.77517
27.79563	796.322	27.79563	444.746	27.79563	617.0449	27.79563	573.8991	27.79563
27.8161	765.519	27.8161	457.0094	27.8161	583.4246	27.8161	487.3902	27.8161
27.83656	538.7218	27.83656	368.2785	27.83656	522.8115	27.83656	375.8871	27.83656
27.85702	520.9302	27.85702	303.5533	27.85702	384.2056	27.85702	246.39	27.85702
27.87749	351.1442	27.87749	229.8339	27.87749	316.6069	27.87749	110.8988	27.87749
27.89795	282.3639	27.89795	188.1202	27.89795	252.0153	27.89795	152.4135	27.89795
27.91841	148.5893	27.91841	122.4122	27.91841	198.431	27.91841	121.9342	27.91841
27.93887	80.82041	27.93887	123.7099	27.93887	125.8538	27.93887	83.46073	27.93887
27.95934	83.05717	27.95934	118.0134	27.95934	91.28375	27.95934	26.99322	27.95934
27.9798	22.2996	27.9798	26.32256	27.9798	49.72092	27.9798	23.53164	27.9798
28.00026	32.54771	28.00026	39.63746	28.00026	89.16527	28.00026	45.07598	28.00026
28.02073	34.80149	28.02073	22.95808	28.02073	29.61679	28.02073	16.62624	28.02073
28.04119	90.06095	28.04119	-2.71557	28.04119	42.07549	28.04119	57.18243	28.04119
28.06165	47.32609	28.06165	-5.38351	28.06165	57.54137	28.06165	77.74454	28.06165
28.08211	76.5969	28.08211	9.954281	28.08211	48.01443	28.08211	-4.68743	28.08211
28.10258	144.8734	28.10258	26.29779	28.10258	65.49467	28.10258	66.88653	28.10258
28.12304	105.1556	28.12304	86.64703	28.12304	53.98208	28.12304	67.46641	28.12304
28.1435	88.4434	28.1435	48.00198	28.1435	67.47667	28.1435	34.05221	28.1435
28.16396	86.73692	28.16396	14.36266	28.16396	71.97844	28.16396	87.64394	28.16396
28.18443	109.0361	28.18443	38.72906	28.18443	19.48739	28.18443	78.24159	28.18443
28.20489	146.341	28.20489	30.10118	28.20489	68.00352	28.20489	107.8452	28.20489
28.22535	116.6515	28.22535	-12.521	28.22535	69.52682	28.22535	143.4547	28.22535
28.24582	242.9677	28.24582	49.86259	28.24582	136.0573	28.24582	167.0701	28.24582
28.26628	291.2896	28.26628	85.25188	28.26628	77.59496	28.26628	164.6914	28.26628
28.28674	291.6172	28.28674	23.64689	28.28674	79.1398	28.28674	222.3187	28.28674
28.3072	430.9505	28.3072	74.04762	28.3072	189.6918	28.3072	247.9519	28.3072
28.32767	441.2894	28.32767	102.4541	28.32767	153.251	28.32767	196.591	28.32767
28.34813	588.634	28.34813	156.8663	28.34813	192.8174	28.34813	301.236	28.34813
28.36859	722.9843	28.36859	163.2842	28.36859	195.3909	28.36859	364.887	28.36859
28.38906	794.3402	28.38906	229.7078	28.38906	252.9717	28.38906	472.5439	28.38906
28.40952	1036.702	28.40952	317.1371	28.40952	392.5596	28.40952	594.2067	28.40952
28.42998	1215.069	28.42998	390.5722	28.42998	541.1547	28.42998	913.8754	28.42998
28.45044	1403.442	28.45044	513.013	28.45044	774.757	28.45044	1125.55	28.45044
28.47091	1486.821	28.47091	703.4595	28.47091	944.3664	28.47091	1320.231	28.47091
28.49137	1483.205	28.49137	857.9117	28.49137	1124.983	28.49137	1393.917	28.49137
28.51183	1326.595	28.51183	902.3697	28.51183	1071.607	28.51183	1428.61	28.51183
28.53229	1097.991	28.53229	1091.833	28.53229	1175.238	28.53229	1372.308	28.53229
28.55276	854.3922	28.55276	1028.303	28.55276	1123.876	28.55276	1066.012	28.55276
28.57322	718.7993	28.57322	908.7779	28.57322	959.5212	28.57322	918.7224	28.57322

28.59368	420.212	28.59368	755.2587	28.59368	814.1738	28.59368	602.4385	28.59368
28.61415	351.6304	28.61415	632.7453	28.61415	619.8334	28.61415	428.1606	28.61415
28.63461	214.0544	28.63461	447.2376	28.63461	419.5003	28.63461	317.8885	28.63461
28.65507	204.4842	28.65507	338.7356	28.65507	397.1744	28.65507	177.6224	28.65507
28.67553	133.9196	28.67553	224.2393	28.67553	253.8556	28.67553	186.3622	28.67553
28.696	106.3607	28.696	139.7488	28.696	219.544	28.696	84.10797	28.696
28.71646	110.8075	28.71646	116.264	28.71646	105.2396	28.71646	111.8596	28.71646
28.73692	61.25993	28.73692	77.78489	28.73692	68.94235	28.73692	59.61721	28.73692
28.75739	86.71806	28.75739	75.31152	28.75739	87.65228	28.75739	8.380722	28.75739
28.77785	59.18186	28.77785	-19.1561	28.77785	67.3694	28.77785	58.15015	28.77785
28.79831	52.65134	28.79831	18.38193	28.79831	61.09369	28.79831	18.92551	28.79831
28.81877	31.12649	28.81877	4.92572	28.81877	53.82517	28.81877	27.70678	28.81877
28.83924	15.60732	28.83924	15.47523	28.83924	22.56381	28.83924	8.493985	28.83924
28.8597	27.09383	28.8597	13.03047	28.8597	22.30964	28.8597	4.287109	28.8597
28.88016	35.58601	28.88016	35.59143	28.88016	32.06265	28.88016	-4.91384	28.88016
28.90062	41.08387	28.90062	14.15811	28.90062	6.822834	28.90062	38.89113	28.90062
28.92109	118.5874	28.92109	-12.2695	28.92109	-16.4098	28.92109	40.70202	28.92109
28.94155	40.09662	28.94155	-7.69136	28.94155	23.36474	28.94155	43.51884	28.94155
28.96201	74.6115	28.96201	-12.1075	28.96201	-15.8535	28.96201	28.34158	28.96201
28.98248	142.1321	28.98248	23.48206	28.98248	11.93535	28.98248	-18.8298	28.98248
29.00294	63.65831	29.00294	57.07735	29.00294	7.73143	29.00294	-29.9952	29.00294
29.0234	129.1902	29.0234	15.67836	29.0234	-31.4653	29.0234	19.61815	29.0234
29.04386	101.7278	29.04386	8.285098	29.04386	9.345116	29.04386	32.23738	29.04386
29.06433	106.2711	29.06433	-4.10244	29.06433	-15.8373	29.06433	7.862536	29.06433
29.08479	98.82003	29.08479	-59.4843	29.08479	-13.0125	29.08479	70.49361	29.08479
29.10525	91.37466	29.10525	16.13964	29.10525	18.81948	29.10525	67.13062	29.10525
29.12572	108.935	29.12572	11.76927	29.12572	-12.3414	29.12572	-80.2264	29.12572
29.14618	51.50092	29.14618	-35.5954	29.14618	-31.4951	29.14618	-13.5776	29.14618
29.16664	92.07257	29.16664	41.04569	29.16664	13.32033	29.16664	-10.9228	29.16664
29.1871	-20.3501	29.1871	34.69248	29.1871	-21.8571	29.1871	23.73787	29.1871
29.20757	-61.7671	29.20757	12.345	29.20757	12.97264	29.20757	-5.59551	29.20757
29.22803	43.82158	29.22803	31.00323	29.22803	5.809566	29.22803	-25.923	29.22803
29.24849	49.41593	29.24849	-9.33281	29.24849	-2.34633	29.24849	-4.30645	29.24849
29.26895	20.01596	29.26895	19.33688	29.26895	-44.4951	29.26895	-5.684	29.26895
29.28942	68.62167	29.28942	-35.9877	29.28942	3.301091	29.28942	55.94436	29.28942
29.30988	19.23305	29.30988	13.69341	29.30988	-16.8956	29.30988	-1.42135	29.30988
29.33034	-10.1499	29.33034	73.38026	29.33034	39.91492	29.33034	50.21886	29.33034
29.35081	43.47285	29.35081	23.07283	29.35081	24.7326	29.35081	10.865	29.35081
29.37127	6.101257	29.37127	14.77112	29.37127	6.557461	29.37127	27.51705	29.37127
29.39173	59.73534	29.39173	-7.52486	29.39173	52.3895	29.39173	27.17504	29.39173
29.41219	44.37511	29.41219	12.18488	29.41219	85.22871	29.41219	107.8389	29.41219
29.43266	-42.9795	29.43266	18.90034	29.43266	20.07511	29.43266	105.5088	29.43266
29.45312	36.67166	29.45312	-22.3785	29.45312	90.92868	29.45312	97.18453	29.45312
29.47358	65.32845	29.47358	40.34842	29.47358	74.78944	29.47358	130.8662	29.47358
29.49405	115.9909	29.49405	45.08105	29.49405	124.6574	29.49405	99.5538	29.49405
29.51451	100.6591	29.51451	110.8194	29.51451	80.53247	29.51451	215.2473	29.51451
29.53497	111.3329	29.53497	110.5635	29.53497	104.4148	29.53497	131.9468	29.53497
29.55543	195.0124	29.55543	206.3133	29.55543	106.3042	29.55543	179.6521	29.55543
29.5759	304.6976	29.5759	242.0688	29.5759	231.2009	29.5759	203.3634	29.5759
29.59636	384.3884	29.59636	172.83	29.59636	300.1047	29.59636	231.0806	29.59636
29.61682	441.0849	29.61682	273.597	29.61682	499.0157	29.61682	211.8038	29.61682
29.63728	424.7871	29.63728	339.3697	29.63728	781.9338	29.63728	281.5328	29.63728
29.65775	385.495	29.65775	372.1481	29.65775	985.8592	29.65775	273.2678	29.65775
29.67821	394.2086	29.67821	427.9322	29.67821	927.7917	29.67821	427.0087	29.67821
29.69867	418.9278	29.69867	564.722	29.69867	916.7314	29.69867	397.7556	29.69867
29.71914	248.6527	29.71914	758.5176	29.71914	1047.678	29.71914	448.5083	29.71914
29.7396	254.3833	29.7396	829.3189	29.7396	1037.632	29.7396	372.267	29.7396
29.76006	146.1195	29.76006	910.1259	29.76006	1048.594	29.76006	341.0316	29.76006

29.78052	166.8615	29.78052	770.9387	29.78052	903.5621	29.78052	362.8022	29.78052
29.80099	98.60909	29.80099	585.7571	29.80099	765.5377	29.80099	316.5786	29.80099
29.82145	111.3624	29.82145	511.5813	29.82145	583.5204	29.82145	227.361	29.82145
29.84191	59.12133	29.84191	417.4112	29.84191	436.5104	29.84191	157.1493	29.84191
29.86238	22.88597	29.86238	367.2469	29.86238	328.5075	29.86238	148.9435	29.86238
29.88284	19.65629	29.88284	225.0882	29.88284	213.5119	29.88284	76.74364	29.88284
29.9033	32.43228	29.9033	83.93529	29.9033	130.5234	29.9033	60.5497	29.9033
29.92376	31.21395	29.92376	136.7881	29.92376	62.54203	29.92376	4.361693	29.92376
29.94423	-16.9987	29.94423	49.6466	29.94423	74.56788	29.94423	51.17961	29.94423
29.96469	28.79431	29.96469	16.51084	29.96469	42.6009	29.96469	82.00345	29.96469
29.98515	-33.407	29.98515	27.38081	29.98515	46.64111	29.98515	16.83321	29.98515
30.00561	33.39738	30.00561	50.25649	30.00561	40.68849	30.00561	52.66889	30.00561
30.02608	13.20742	30.02608	-8.8621	30.02608	25.74305	30.02608	21.5105	30.02608
30.04654	-22.9769	30.04654	-0.97497	30.04654	39.80479	30.04654	41.35803	30.04654
30.067	25.84455	30.067	41.91788	30.067	43.87371	30.067	-1.78851	30.067
30.08747	-3.32837	30.08747	15.81646	30.08747	31.94981	30.08747	14.07087	30.08747
30.10793	-35.4956	30.10793	18.72075	30.10793	6.033081	30.10793	-7.06383	30.10793
30.12839	4.009321	30.12839	28.63077	30.12839	-21.8765	30.12839	21.8074	30.12839
30.14885	29.51994	30.14885	-6.45349	30.14885	55.22117	30.14885	25.68455	30.14885
30.16932	39.03624	30.16932	61.46798	30.16932	97.32597	30.16932	64.56762	30.16932
30.18978	43.55821	30.18978	15.39516	30.18978	-0.56204	30.18978	6.456615	30.18978
30.21024	65.08585	30.21024	41.32807	30.21024	19.55713	30.21024	32.35154	30.21024
30.23071	61.61918	30.23071	76.2667	30.23071	36.68347	30.23071	89.25238	30.23071
30.25117	71.15817	30.25117	65.21105	30.25117	37.81699	30.25117	57.15915	30.25117
30.27163	105.7029	30.27163	79.16113	30.27163	20.95769	30.27163	102.0718	30.27163
30.29209	119.2532	30.29209	55.11692	30.29209	63.10557	30.29209	58.99045	30.29209
30.31256	157.8092	30.31256	94.07845	30.31256	43.26062	30.31256	100.915	30.31256
30.33302	230.3709	30.33302	90.04569	30.33302	37.42286	30.33302	150.8454	30.33302
30.35348	161.9383	30.35348	150.0186	30.35348	50.59227	30.35348	146.7818	30.35348
30.37394	227.5114	30.37394	146.9973	30.37394	141.7689	30.37394	216.7241	30.37394
30.39441	200.0901	30.39441	170.9818	30.39441	178.9526	30.39441	300.6724	30.39441
30.41487	338.6745	30.41487	175.9719	30.41487	146.1436	30.41487	381.6265	30.41487
30.43533	372.2646	30.43533	218.9677	30.43533	185.3417	30.43533	422.5866	30.43533
30.4558	383.8604	30.4558	250.9693	30.4558	236.547	30.4558	463.5526	30.4558
30.47626	369.4618	30.47626	207.9766	30.47626	225.7595	30.47626	370.5245	30.47626
30.49672	498.0689	30.49672	273.9896	30.49672	273.9792	30.49672	389.5024	30.49672
30.51718	580.6817	30.51718	336.0084	30.51718	331.206	30.51718	361.4861	30.51718
30.53765	656.3002	30.53765	331.0328	30.53765	341.44	30.53765	378.4758	30.53765
30.55811	793.9243	30.55811	398.063	30.55811	462.6812	30.55811	488.4715	30.55811
30.57857	907.5541	30.57857	511.0989	30.57857	631.9296	30.57857	536.473	30.57857
30.59904	951.1896	30.59904	601.1406	30.59904	827.1851	30.59904	646.4805	30.59904
30.6195	1021.831	30.6195	766.1879	30.6195	893.4478	30.6195	752.4938	30.6195
30.63996	957.4777	30.63996	1026.241	30.63996	1042.718	30.63996	861.5132	30.63996
30.66042	857.1301	30.66042	1124.3	30.66042	1091.995	30.66042	893.5384	30.66042
30.68089	723.7883	30.68089	1402.364	30.68089	1021.279	30.68089	957.5696	30.68089
30.70135	631.4522	30.70135	1278.435	30.70135	1017.571	30.70135	906.6066	30.70135
30.72181	482.1218	30.72181	1283.51	30.72181	972.8691	30.72181	779.6497	30.72181
30.74227	455.797	30.74227	1197.592	30.74227	885.1749	30.74227	615.6985	30.74227
30.76274	282.4779	30.76274	961.6796	30.76274	726.4879	30.76274	459.7534	30.76274
30.7832	257.1645	30.7832	851.7728	30.7832	577.8081	30.7832	375.8142	30.7832
30.80366	151.8568	30.80366	636.8716	30.80366	498.1354	30.80366	276.8809	30.80366
30.82413	115.5547	30.82413	483.9762	30.82413	339.4699	30.82413	181.9535	30.82413
30.84459	132.2583	30.84459	271.0865	30.84459	304.8116	30.84459	138.0321	30.84459
30.86505	101.9676	30.86505	262.2025	30.86505	152.1605	30.86505	78.11655	30.86505
30.88551	85.68253	30.88551	202.3242	30.88551	93.51653	30.88551	55.20694	30.88551
30.90598	75.40317	30.90598	163.4517	30.90598	145.8797	30.90598	12.30325	30.90598
30.92644	77.12948	30.92644	129.5849	30.92644	95.25015	30.92644	-22.5945	30.92644
30.9469	39.86147	30.9469	82.72382	30.9469	75.62773	30.9469	38.51366	30.9469

30.96737	-5.40087	30.96737	85.86845	30.96737	60.01249	30.96737	23.62775	30.96737
30.98783	34.34248	30.98783	19.01881	30.98783	7.404427	30.98783	50.74776	30.98783
31.00829	-32.9085	31.00829	55.17489	31.00829	10.80354	31.00829	8.87369	31.00829
31.02875	-1.15381	31.02875	60.33669	31.02875	13.20983	31.02875	21.00555	31.02875
31.04922	5.606557	31.04922	51.50422	31.04922	59.6233	31.04922	30.14333	31.04922
31.06968	13.3726	31.06968	54.67746	31.06968	36.04395	31.06968	64.28703	31.06968
31.09014	-29.8557	31.09014	60.85643	31.09014	-27.5282	31.09014	7.43666	31.09014
31.1106	1.921723	31.1106	16.04112	31.1106	3.906784	31.1106	45.59221	31.1106
31.13107	-31.2952	31.13107	56.23153	31.13107	-68.651	31.13107	-18.2463	31.13107
31.15153	57.49355	31.15153	123.4277	31.15153	10.79833	31.15153	13.92108	31.15153
31.17199	-8.71203	31.17199	17.62952	31.17199	-25.7451	31.17199	6.094406	31.17199
31.19246	53.08808	31.19246	7.837099	31.19246	9.718585	31.19246	58.27365	31.19246
31.21292	31.89386	31.21292	-6.9496	31.21292	-15.8105	31.21292	39.45882	31.21292
31.23338	11.70531	31.23338	59.26942	31.23338	8.667555	31.23338	13.64991	31.23338
31.25384	-16.4776	31.25384	62.49417	31.25384	39.15281	31.25384	-5.15307	31.25384
31.27431	10.34525	31.27431	29.72464	31.27431	-17.3548	31.27431	69.04987	31.27431
31.29477	5.173734	31.29477	9.960829	31.29477	2.144845	31.29477	6.258728	31.29477
31.31523	38.00789	31.31523	51.20274	31.31523	11.65163	31.31523	-3.52649	31.31523
31.3357	56.84773	31.3357	71.45038	31.3357	61.1656	31.3357	56.69422	31.3357
31.35616	12.69324	31.35616	99.70374	31.35616	31.68674	31.35616	24.92086	31.35616
31.37662	47.54443	31.37662	74.96281	31.37662	26.21506	31.37662	31.15341	31.37662
31.39708	52.4013	31.39708	111.2276	31.39708	75.75056	31.39708	38.39189	31.39708
31.41755	106.2638	31.41755	92.49814	31.41755	4.293238	31.41755	80.63629	31.41755
31.43801	74.13205	31.43801	104.7744	31.43801	72.84309	31.43801	41.88662	31.43801
31.45847	174.006	31.45847	90.05636	31.45847	72.40013	31.45847	152.1429	31.45847
31.47893	156.8855	31.47893	113.344	31.47893	68.96434	31.47893	102.405	31.47893
31.4994	105.7708	31.4994	85.63746	31.4994	52.53573	31.4994	214.6731	31.4994
31.51986	116.6617	31.51986	173.9366	31.51986	172.1143	31.51986	252.9472	31.51986
31.54032	123.5583	31.54032	182.2415	31.54032	180.7	31.54032	297.2271	31.54032
31.56079	132.4606	31.56079	195.552	31.56079	278.293	31.56079	412.513	31.56079
31.58125	142.3685	31.58125	247.8683	31.58125	265.8931	31.58125	405.8047	31.58125
31.60171	111.2821	31.60171	277.1904	31.60171	277.5004	31.60171	371.1025	31.60171
31.62217	99.20143	31.62217	288.5181	31.62217	293.1148	31.62217	313.4061	31.62217
31.64264	73.1264	31.64264	283.8516	31.64264	257.7365	31.64264	333.7157	31.64264
31.6631	55.05706	31.6631	252.1908	31.6631	207.3653	31.6631	294.0312	31.6631
31.68356	-12.0066	31.68356	205.5357	31.68356	174.0013	31.68356	244.3526	31.68356
31.70403	63.9354	31.70403	136.8863	31.70403	105.6444	31.70403	132.6799	31.70403
31.72449	-5.11692	31.72449	98.24268	31.72449	59.29479	31.72449	151.0132	31.72449
31.74495	-20.1636	31.74495	83.60477	31.74495	12.95231	31.74495	69.35233	31.74495
31.76541	13.79548	31.76541	51.97257	31.76541	59.61702	31.76541	62.69743	31.76541
31.78588	67.76019	31.78588	61.34609	31.78588	22.2889	31.78588	48.04846	31.78588
31.80634	29.73058	31.80634	41.72534	31.80634	9.967965	31.80634	46.40541	31.80634
31.8268	-11.2934	31.8268	47.11031	31.8268	29.65421	31.8268	38.76828	31.8268
31.84726	17.68838	31.84726	-13.499	31.84726	52.34763	31.84726	30.13707	31.84726
31.86773	63.6758	31.86773	142.8974	31.86773	93.04823	31.86773	52.51179	31.86773
31.88819	42.66889	31.88819	52.29956	31.88819	84.756	31.88819	42.89243	31.88819
31.90865	25.66766	31.90865	80.70741	31.90865	23.47095	31.90865	18.279	31.90865
31.92912	53.6721	31.92912	100.121	31.92912	22.19309	31.92912	41.67149	31.92912
31.94958	68.68222	31.94958	80.54031	31.94958	99.92239	31.94958	72.0699	31.94958
31.97004	102.698	31.97004	141.9653	31.97004	56.65888	31.97004	46.47424	31.97004
31.9905	115.7195	31.9905	180.3961	31.9905	67.40255	31.9905	66.8845	31.9905
32.01097	139.7466	32.01097	153.8326	32.01097	67.1534	32.01097	112.3007	32.01097
32.03143	174.7795	32.03143	248.2748	32.03143	93.91142	32.03143	147.7228	32.03143
32.05189	190.818	32.05189	311.7227	32.05189	82.67662	32.05189	161.1508	32.05189
32.07236	186.8622	32.07236	290.1763	32.07236	102.449	32.07236	250.5848	32.07236
32.09282	293.912	32.09282	339.6357	32.09282	98.22855	32.09282	237.0246	32.09282
32.11328	322.9675	32.11328	310.1007	32.11328	129.0153	32.11328	237.4704	32.11328
32.13374	384.0287	32.13374	317.5715	32.13374	118.8092	32.13374	236.9222	32.13374

32.15421	473.0956	32.15421	266.0481	32.15421	138.6103	32.15421	263.3798	32.15421
32.17467	541.1682	32.17467	401.5303	32.17467	201.4186	32.17467	345.8434	32.17467
32.19513	683.2464	32.19513	338.0183	32.19513	275.234	32.19513	400.3129	32.19513
32.21559	577.3303	32.21559	343.512	32.21559	308.0566	32.21559	400.7883	32.21559
32.23606	502.4199	32.23606	348.0114	32.23606	550.8865	32.23606	366.2697	32.23606
32.25652	476.5152	32.25652	429.5165	32.25652	649.7235	32.25652	404.7569	32.25652
32.27698	437.6161	32.27698	461.0274	32.27698	635.5676	32.27698	307.2501	32.27698
32.29745	404.7227	32.29745	381.544	32.29745	712.4189	32.29745	303.7492	32.29745
32.31791	278.835	32.31791	336.0663	32.31791	650.2775	32.31791	258.2542	32.31791
32.33837	198.953	32.33837	365.5943	32.33837	592.1431	32.33837	236.7652	32.33837
32.35883	135.0766	32.35883	383.1281	32.35883	562.016	32.35883	188.2821	32.35883
32.3793	240.2059	32.3793	324.6675	32.3793	501.8961	32.3793	146.8049	32.3793
32.39976	171.3409	32.39976	246.2127	32.39976	394.7833	32.39976	133.3336	32.39976
32.42022	106.4816	32.42022	206.7637	32.42022	332.6777	32.42022	146.8683	32.42022
32.44069	150.628	32.44069	161.3203	32.44069	305.5793	32.44069	130.4088	32.44069
32.46115	94.77999	32.46115	227.8827	32.46115	203.4881	32.46115	128.9553	32.46115
32.48161	43.93769	32.48161	138.4507	32.48161	271.404	32.48161	105.5078	32.48161
32.50207	110.1011	32.50207	194.0246	32.50207	184.3271	32.50207	122.0661	32.50207
32.52254	84.27012	32.52254	157.6041	32.52254	189.2574	32.52254	26.63037	32.52254
32.543	3.444852	32.543	140.1893	32.543	90.19489	32.543	67.20056	32.543
32.56346	28.62526	32.56346	127.7803	32.56346	116.1395	32.56346	33.77668	32.56346
32.58392	-1.18866	32.58392	63.377	32.58392	74.09139	32.58392	80.35872	32.58392
32.60439	0.003097	32.60439	55.97942	32.60439	74.0504	32.60439	67.94669	32.60439
32.62485	57.20053	32.62485	54.58756	32.62485	70.01659	32.62485	-12.4594	32.62485
32.64531	-28.5964	32.64531	37.20142	32.64531	47.98996	32.64531	-18.8596	32.64531
32.66578	-26.3876	32.66578	41.82101	32.66578	71.97051	32.66578	14.74611	32.66578
32.68624	10.82689	32.68624	92.44631	32.68624	59.95823	32.68624	46.35777	32.68624
32.7067	-29.953	32.7067	18.07734	32.7067	8.953129	32.7067	22.97535	32.7067
32.72716	-6.72716	32.72716	65.7141	32.72716	-3.04479	32.72716	-3.40115	32.72716
32.74763	-82.4957	32.74763	14.35657	32.74763	46.96447	32.74763	-8.77172	32.74763
32.76809	7.700088	32.76809	-35.9952	32.76809	48.98091	32.76809	-3.13637	32.76809
32.78855	-63.0985	32.78855	-13.8804	32.78855	13.00452	32.78855	-25.4951	32.78855
32.80902	32.10862	32.80902	22.24023	32.80902	3.035315	32.80902	2.6912	32.80902
32.82948	-10.6786	32.82948	21.36655	32.82948	59.07329	32.82948	29.88342	32.82948
32.84994	0.539862	32.84994	34.49859	32.84994	65.11844	32.84994	25.08157	32.84994
32.8704	57.764	32.8704	72.63635	32.8704	2.170765	32.8704	48.28563	32.8704
32.89087	-46.0062	32.89087	8.779828	32.89087	-23.7697	32.89087	36.49563	32.89087
32.91133	-35.7707	32.91133	-47.071	32.91133	-23.7697	32.91133	24.71154	32.91133
32.93179	49.47046	32.93179	0.08396	32.93179	11.04067	32.93179	24.93338	32.93179
32.95225	12.71729	32.95225	20.24461	32.95225	2.858238	32.95225	32.16114	32.95225
32.97272	-19.0302	32.97272	38.41098	32.97272	3.682988	32.97272	-27.6052	32.97272
32.99318	9.228	32.99318	-22.4169	32.99318	3.514917	32.99318	-4.36557	32.99318
33.01364	28.49187	33.01364	10.76089	33.01364	-21.646	33.01364	11.87996	33.01364
33.03411	10.76141	33.03411	1.944431	33.03411	36.20031	33.03411	-16.8686	33.03411
33.05457	-19.9634	33.05457	23.13369	33.05457	-13.9462	33.05457	20.3888	33.05457
33.07503	49.31752	33.07503	49.32867	33.07503	47.91441	33.07503	-15.3479	33.07503
33.09549	-21.3959	33.09549	33.52938	33.09549	-2.21777	33.09549	50.92133	33.09549
33.11596	-9.10366	33.11596	39.73581	33.11596	14.65723	33.11596	7.196475	33.11596
33.13642	-1.80573	33.13642	20.94796	33.13642	-38.4606	33.13642	-20.5225	33.13642
33.15688	-18.5021	33.15688	2.165833	33.15688	-5.57124	33.15688	9.764544	33.15688
33.17735	6.807146	33.17735	0.389428	33.17735	2.325292	33.17735	-8.94254	33.17735
33.19781	38.1221	33.19781	56.61874	33.19781	-2.771	33.19781	24.35631	33.19781
33.21827	40.44273	33.21827	44.85379	33.21827	3.139891	33.21827	25.66107	33.21827
33.23873	59.76903	33.23873	12.09455	33.23873	-23.942	33.23873	90.97176	33.23873
33.2592	36.10101	33.2592	7.341031	33.2592	49.9832	33.2592	50.28838	33.2592
33.27966	74.43867	33.27966	30.59324	33.27966	40.91563	33.27966	24.61091	33.27966
33.30012	-11.218	33.30012	2.851166	33.30012	70.85523	33.30012	61.93937	33.30012
33.32058	27.13101	33.32058	31.11482	33.32058	28.80201	33.32058	104.2738	33.32058

33.34105	38.4857	33.34105	76.38419	33.34105	84.75597	33.34105	23.61406	33.34105
33.36151	31.84606	33.36151	62.65929	33.36151	103.7171	33.36151	74.96029	33.36151
33.38197	-13.7879	33.38197	71.9401	33.38197	67.68542	33.38197	23.31245	33.38197
33.40244	-14.4162	33.40244	83.22665	33.40244	21.66091	33.40244	54.67053	33.40244
33.4229	17.96121	33.4229	75.51891	33.4229	86.64358	33.4229	37.03452	33.4229
33.44336	-23.6557	33.44336	55.8169	33.44336	23.63343	33.44336	15.40445	33.44336
33.46382	5.733013	33.46382	57.1206	33.46382	75.63045	33.46382	-41.2197	33.46382
33.48429	-18.8726	33.48429	73.43003	33.48429	45.63466	33.48429	-2.83793	33.48429
33.50475	30.52753	33.50475	-4.25482	33.50475	25.64604	33.50475	30.54976	33.50475
33.52521	-34.0667	33.52521	38.06606	33.52521	76.6646	33.52521	1.94338	33.52521
33.54568	-10.6553	33.54568	47.39265	33.54568	25.69034	33.54568	31.34292	33.54568
33.56614	38.76187	33.56614	40.72497	33.56614	87.72326	33.56614	25.74839	33.56614
33.5866	64.18467	33.5866	24.06301	33.5866	24.76335	33.5866	34.15978	33.5866
33.60706	31.61314	33.60706	51.40678	33.60706	46.81063	33.60706	26.57709	33.60706
33.62753	81.0473	33.62753	120.7563	33.62753	56.86508	33.62753	133.0003	33.62753
33.64799	7.487124	33.64799	87.11147	33.64799	65.92671	33.64799	109.4295	33.64799
33.66845	66.93263	33.66845	96.4724	33.66845	52.99552	33.66845	159.8646	33.66845
33.68891	115.3838	33.68891	126.8391	33.68891	39.0715	33.68891	125.3056	33.68891
33.70938	178.8407	33.70938	133.2114	33.70938	61.15467	33.70938	120.7525	33.70938
33.72984	112.3032	33.72984	174.5895	33.72984	92.24501	33.72984	181.2054	33.72984
33.7503	236.7714	33.7503	231.9733	33.7503	101.3425	33.7503	154.6641	33.7503
33.77077	271.2453	33.77077	181.3629	33.77077	142.4472	33.77077	294.1288	33.77077
33.79123	396.7249	33.79123	232.7581	33.79123	207.5591	33.79123	374.5995	33.79123
33.81169	479.2101	33.81169	320.1591	33.81169	209.6782	33.81169	318.076	33.81169
33.83215	504.701	33.83215	351.5659	33.83215	245.8044	33.83215	390.5585	33.83215
33.85262	584.1976	33.85262	402.9783	33.85262	316.9378	33.85262	508.0468	33.85262
33.87308	688.6999	33.87308	447.3964	33.87308	487.0784	33.87308	543.5412	33.87308
33.89354	789.2078	33.89354	565.8203	33.89354	730.2262	33.89354	720.0414	33.89354
33.91401	821.7214	33.91401	777.2499	33.91401	917.3811	33.91401	951.5476	33.91401
33.93447	812.2407	33.93447	950.6852	33.93447	1134.543	33.93447	1081.06	33.93447
33.95493	764.7657	33.95493	1163.126	33.95493	1346.713	33.95493	1106.578	33.95493
33.97539	688.2963	33.97539	1306.573	33.97539	1570.889	33.97539	1288.102	33.97539
33.99586	651.8326	33.99586	1365.026	33.99586	1614.073	33.99586	1177.631	33.99586
34.01632	571.3746	34.01632	1316.484	34.01632	1634.264	34.01632	1116.167	34.01632
34.03678	487.9223	34.03678	1371.948	34.03678	1689.462	34.03678	1043.709	34.03678
34.05724	286.4757	34.05724	1063.417	34.05724	1450.667	34.05724	815.2566	34.05724
34.07771	259.0347	34.07771	1006.893	34.07771	1317.879	34.07771	603.8101	34.07771
34.09817	207.5994	34.09817	801.3738	34.09817	1041.099	34.09817	543.3696	34.09817
34.11863	118.1698	34.11863	645.8606	34.11863	790.3254	34.11863	372.935	34.11863
34.1391	83.74585	34.1391	521.3531	34.1391	634.5593	34.1391	256.5063	34.1391
34.15956	209.3276	34.15956	400.8514	34.15956	501.8004	34.15956	212.0836	34.15956
34.18002	134.915	34.18002	273.3554	34.18002	307.0486	34.18002	90.66673	34.18002
34.20048	121.5081	34.20048	179.8651	34.20048	207.3041	34.20048	66.25583	34.20048
34.22095	82.10683	34.22095	165.3806	34.22095	195.5667	34.22095	84.85085	34.22095
34.24141	121.7113	34.24141	98.9017	34.24141	57.83649	34.24141	73.45178	34.24141
34.26187	70.32138	34.26187	55.42859	34.26187	63.11347	34.26187	71.05865	34.26187
34.28234	40.93717	34.28234	39.96118	34.28234	56.39763	34.28234	39.67143	34.28234
34.3028	24.55864	34.3028	26.4995	34.3028	47.68895	34.3028	-7.70986	34.3028
34.32326	76.18578	34.32326	76.04355	34.32326	54.98747	34.32326	-6.08523	34.32326
34.34372	22.8186	34.34372	1.593315	34.34372	49.29315	34.34372	-14.4547	34.34372
34.36419	30.45709	34.36419	18.1488	34.36419	73.60602	34.36419	15.18181	34.36419
34.38465	17.10126	34.38465	6.710015	34.38465	10.92606	34.38465	-42.1758	34.38465
34.40511	-28.2489	34.40511	9.276948	34.40511	-13.7467	34.40511	30.47253	34.40511
34.42557	-19.8543	34.42557	52.84961	34.42557	42.58769	34.42557	10.12678	34.42557
34.44604	25.54604	34.44604	24.42798	34.44604	-17.0707	34.44604	61.78696	34.44604
34.4665	-13.048	34.4665	48.01208	34.4665	38.27803	34.4665	19.45305	34.4665
34.48696	62.36368	34.48696	-3.3981	34.48696	7.633961	34.48696	20.12507	34.48696
34.50743	44.78101	34.50743	44.19745	34.50743	-1.00293	34.50743	46.80302	34.50743

34.52789	-3.79598	34.52789	22.79872	34.52789	-11.6326	34.52789	-24.5131	34.52789
34.54835	-27.3673	34.54835	11.40571	34.54835	45.74484	34.54835	21.17667	34.54835
34.56881	30.06707	34.56881	97.01842	34.56881	48.12949	34.56881	-14.1276	34.56881
34.58928	8.507105	34.58928	71.63686	34.58928	59.52131	34.58928	11.57403	34.58928
34.60974	32.95282	34.60974	65.26101	34.60974	10.92032	34.60974	-24.7184	34.60974
34.6302	-42.5958	34.6302	75.89088	34.6302	61.3265	34.6302	-36.1478	34.6302
34.65067	8.861276	34.65067	52.52649	34.65067	15.73986	34.65067	36.42871	34.65067
34.67113	-3.67598	34.67113	38.16781	34.67113	57.1604	34.67113	43.01116	34.67113
34.69159	39.79244	34.69159	37.81486	34.69159	42.58812	34.69159	26.59953	34.69159
34.71205	63.26653	34.71205	16.46763	34.71205	24.02301	34.71205	51.19383	34.71205
34.73252	59.7463	34.73252	106.1261	34.73252	-6.53492	34.73252	55.79404	34.73252
34.75298	94.23175	34.75298	54.79033	34.75298	47.91434	34.75298	34.40019	34.75298
34.77344	163.7229	34.77344	80.46027	34.77344	68.37077	34.77344	95.01225	34.77344
34.7939	172.2197	34.7939	58.13592	34.7939	20.83438	34.7939	98.63024	34.7939
34.81437	214.7222	34.81437	103.8173	34.81437	54.30516	34.81437	109.2542	34.81437
34.83483	234.2303	34.83483	154.5044	34.83483	87.78313	34.83483	88.88399	34.83483
34.85529	242.7441	34.85529	133.1972	34.85529	85.26827	34.85529	159.5197	34.85529
34.87576	332.2636	34.87576	199.8958	34.87576	143.7606	34.87576	271.1614	34.87576
34.89622	390.7888	34.89622	188.6	34.89622	153.2601	34.89622	211.809	34.89622
34.91668	541.3196	34.91668	178.31	34.91668	84.76677	34.91668	284.4626	34.91668
34.93714	625.8562	34.93714	294.0258	34.93714	175.2806	34.93714	361.122	34.93714
34.95761	694.3984	34.95761	315.7472	34.95761	275.8017	34.95761	333.7874	34.95761
34.97807	810.9463	34.97807	428.4743	34.97807	340.3299	34.97807	446.4587	34.97807
34.99853	767.4999	34.99853	464.2072	34.99853	487.8653	34.99853	488.1359	34.99853
35.019	789.0591	35.019	560.9458	35.019	651.4078	35.019	447.8191	35.019
35.03946	684.624	35.03946	549.6901	35.03946	662.9576	35.03946	458.5081	35.03946
35.05992	565.1946	35.05992	544.4402	35.05992	643.5145	35.05992	424.2031	35.05992
35.08038	508.7709	35.08038	572.196	35.08038	725.0786	35.08038	295.9041	35.08038
35.10085	374.3528	35.10085	477.9575	35.10085	592.6499	35.10085	235.6109	35.10085
35.12131	253.9404	35.12131	376.7247	35.12131	502.2283	35.12131	234.3237	35.12131
35.14177	228.5337	35.14177	323.4976	35.14177	412.814	35.14177	146.0423	35.14177
35.16223	55.13269	35.16223	258.2763	35.16223	335.4068	35.16223	103.767	35.16223
35.1827	102.7373	35.1827	170.0606	35.1827	228.0068	35.1827	72.49748	35.1827
35.20316	64.34766	35.20316	133.8508	35.20316	169.614	35.20316	20.23394	35.20316
35.22362	115.9637	35.22362	143.6466	35.22362	98.2283	35.22362	48.97633	35.22362
35.24409	-15.4147	35.24409	77.44812	35.24409	67.84983	35.24409	49.72463	35.24409
35.26455	49.21268	35.26455	36.25539	35.26455	33.47854	35.26455	14.47886	35.26455
35.28501	38.84571	35.28501	38.06839	35.28501	13.11443	35.28501	71.23901	35.28501
35.30547	32.48441	35.30547	9.8871	35.30547	68.75748	35.30547	7.005084	35.30547
35.32594	22.12879	35.32594	62.71154	35.32594	25.40773	35.32594	-0.22292	35.32594
35.3464	24.77885	35.3464	40.5417	35.3464	52.06515	35.3464	22.555	35.3464
35.36686	54.43458	35.36686	8.377579	35.36686	-12.2703	35.36686	32.33885	35.36686
35.38733	12.09599	35.38733	17.21918	35.38733	12.40152	35.38733	2.128618	35.38733
35.40779	47.76307	35.40779	37.06651	35.40779	52.08048	35.40779	45.92431	35.40779
35.42825	36.43583	35.42825	50.91956	35.42825	82.7666	35.42825	59.72593	35.42825
35.44871	49.11427	35.44871	46.77833	35.44871	1.459915	35.44871	80.53346	35.44871
35.46918	42.79838	35.46918	19.64282	35.46918	-16.8396	35.46918	46.34693	35.46918
35.48964	18.48817	35.48964	41.51304	35.48964	53.86807	35.48964	100.1663	35.48964
35.5101	91.18363	35.5101	71.38898	35.5101	38.58291	35.5101	117.9916	35.5101
35.53056	80.88477	35.53056	107.2706	35.53056	52.30494	35.53056	149.8229	35.53056
35.55103	88.59159	35.55103	115.158	35.55103	45.03414	35.55103	102.66	35.55103
35.57149	121.3041	35.57149	72.05113	35.57149	82.77052	35.57149	191.5031	35.57149
35.59195	107.0223	35.59195	75.94995	35.59195	128.5141	35.59195	205.3521	35.59195
35.61242	132.7461	35.61242	119.8545	35.61242	153.2648	35.61242	223.207	35.61242
35.63288	129.4756	35.63288	128.7648	35.63288	128.0227	35.63288	243.0679	35.63288
35.65334	116.2108	35.65334	151.6808	35.65334	176.7878	35.65334	207.9346	35.65334
35.6738	119.9517	35.6738	157.6025	35.6738	130.5601	35.6738	141.8073	35.6738
35.69427	55.69825	35.69427	99.52992	35.69427	146.3395	35.69427	122.686	35.69427

35.71473	142.4505	35.71473	165.4631	35.71473	169.1262	35.71473	114.5705	35.71473
35.73519	67.20837	35.73519	127.402	35.73519	148.92	35.73519	112.461	35.73519
35.75566	53.97195	35.75566	56.34657	35.75566	69.72095	35.75566	49.35737	35.75566
35.77612	40.74121	35.77612	29.2969	35.77612	111.5291	35.77612	57.25968	35.77612
35.79658	82.51614	35.79658	43.25295	35.79658	69.34444	35.79658	95.16792	35.79658
35.81704	45.29675	35.81704	35.21472	35.81704	66.16696	35.81704	52.08208	35.81704
35.83751	76.08303	35.83751	22.18222	35.83751	38.99666	35.83751	61.00216	35.83751
35.85797	11.87499	35.85797	70.15543	35.85797	-20.1665	35.85797	39.92817	35.85797
35.87843	62.67263	35.87843	34.13437	35.87843	16.67758	35.87843	1.860103	35.87843
35.89889	11.47594	35.89889	-8.88097	35.89889	55.52881	35.89889	35.79796	35.89889
35.91936	29.28493	35.91936	29.10942	35.91936	44.38722	35.91936	22.74174	35.91936
35.93982	37.09959	35.93982	48.10553	35.93982	4.252804	35.93982	6.691439	35.93982
35.96028	12.91993	35.96028	-13.8926	35.96028	11.12557	35.96028	37.64706	35.96028
35.98075	-2.25405	35.98075	50.1149	35.98075	-29.9945	35.98075	-16.3914	35.98075
36.00121	5.577643	36.00121	-0.87182	36.00121	-11.4018	36.00121	56.57608	36.00121
36.02167	25.41501	36.02167	-13.8528	36.02167	-19.802	36.02167	36.54948	36.02167
36.04213	-11.7419	36.04213	25.17189	36.04213	10.80497	36.04213	4.5288	36.04213
36.0626	-10.8932	36.0626	5.202331	36.0626	10.41915	36.0626	25.51404	36.0626
36.08306	39.96117	36.08306	25.23849	36.08306	19.04051	36.08306	-6.49479	36.08306
36.10352	-27.1788	36.10352	-29.7196	36.10352	-8.33096	36.10352	-12.4977	36.10352
36.12399	-27.313	36.12399	-23.672	36.12399	-13.6952	36.12399	-24.4947	36.12399
36.14445	-0.69002	36.14445	-18.6187	36.14445	-11.0524	36.14445	12.23572	36.14445
36.16491	80.93863	36.16491	15.44037	36.16491	24.59771	36.16491	8.972058	36.16491
36.18537	-42.427	36.18537	-41.4949	36.18537	7.254955	36.18537	11.71432	36.18537
36.20584	47.21296	36.20584	-15.346	36.20584	12.91938	36.20584	32.4625	36.20584
36.2263	88.85864	36.2263	12.80851	36.2263	16.59098	36.2263	7.216606	36.2263
36.24676	40.51	36.24676	-16.0312	36.24676	0.269763	36.24676	87.97664	36.24676
36.26722	25.16704	36.26722	6.134759	36.26722	9.955722	36.26722	31.74259	36.26722
36.28769	0.829746	36.28769	26.30647	36.28769	29.64886	36.28769	13.51447	36.28769
36.30815	35.49813	36.30815	15.4839	36.30815	-31.6508	36.30815	56.29227	36.30815
36.32861	30.17219	36.32861	51.66706	36.32861	-8.94333	36.32861	-8.92401	36.32861
36.34908	17.85193	36.34908	46.85593	36.34908	54.77134	36.34908	17.86564	36.34908
36.36954	52.53735	36.36954	30.05053	36.36954	27.49319	36.36954	67.66121	36.36954
36.39	22.22844	36.39	51.25085	36.39	20.22222	36.39	76.4627	36.39
36.41046	5.925204	36.41046	53.4569	36.41046	-3.04158	36.41046	75.27012	36.41046
36.43093	32.62765	36.43093	36.66867	36.43093	33.70181	36.43093	3.083457	36.43093
36.45139	60.33577	36.45139	13.88616	36.45139	-11.5476	36.45139	8.90272	36.45139
36.47185	75.04956	36.47185	45.10937	36.47185	3.210111	36.47185	57.72791	36.47185
36.49232	85.76904	36.49232	-13.6617	36.49232	-10.025	36.49232	55.55902	36.49232
36.51278	22.49418	36.51278	0.572956	36.51278	16.74713	36.51278	50.39605	36.51278
36.53324	58.22501	36.53324	41.81334	36.53324	70.52641	36.53324	48.23901	36.53324
36.5537	37.96151	36.5537	52.05943	36.5537	60.31286	36.5537	51.08789	36.5537
36.57417	66.70368	36.57417	47.31126	36.57417	23.10649	36.57417	80.9427	36.57417
36.59463	36.45153	36.59463	54.5688	36.59463	20.9073	36.59463	107.8034	36.59463
36.61509	58.20506	36.61509	49.83207	36.61509	45.71529	36.61509	42.67008	36.61509
36.63555	41.96427	36.63555	40.10106	36.63555	102.5304	36.63555	121.5426	36.63555
36.65602	35.72915	36.65602	81.37577	36.65602	159.3528	36.65602	117.4212	36.65602
36.67648	38.4997	36.67648	113.6562	36.67648	120.1823	36.67648	62.30557	36.67648
36.69694	36.27594	36.69694	118.9424	36.69694	104.019	36.69694	84.19592	36.69694
36.71741	-16.9422	36.71741	91.23424	36.71741	98.8629	36.71741	115.0922	36.71741
36.73787	65.84543	36.73787	80.53185	36.73787	94.71395	36.73787	78.99438	36.73787
36.75833	50.6387	36.75833	89.83517	36.75833	52.57219	36.75833	93.9025	36.75833
36.77879	45.43763	36.77879	64.14421	36.77879	5.437603	36.77879	86.81654	36.77879
36.79926	49.24225	36.79926	112.459	36.79926	27.3102	36.79926	31.7365	36.79926
36.81972	27.05253	36.81972	60.77947	36.81972	35.18996	36.81972	35.66238	36.81972
36.84018	53.8685	36.84018	89.10568	36.84018	65.07691	36.84018	11.59419	36.84018
36.86065	51.69014	36.86065	69.43761	36.86065	44.97104	36.86065	27.53192	36.86065
36.88111	26.51746	36.88111	99.77528	36.88111	50.87234	36.88111	69.47558	36.88111

36.90157	52.35046	36.90157	77.11865	36.90157	26.78083	36.90157	16.42516	36.90157
36.92203	68.18913	36.92203	43.46775	36.92203	-7.30351	36.92203	36.38067	36.92203
36.9425	45.03347	36.9425	34.82258	36.9425	-15.3807	36.9425	120.3421	36.9425
36.96296	70.8835	36.96296	101.1831	36.96296	53.54934	36.96296	81.30944	36.96296
36.98342	83.7392	36.98342	143.5494	36.98342	57.48654	36.98342	160.2827	36.98342
37.00388	178.6006	37.00388	193.9214	37.00388	85.43091	37.00388	154.2619	37.00388
37.02435	251.4676	37.02435	227.2991	37.02435	56.38246	37.02435	210.247	37.02435
37.04481	172.3403	37.04481	232.6825	37.04481	116.3412	37.04481	180.2381	37.04481
37.06527	260.2188	37.06527	270.0717	37.06527	171.3071	37.06527	291.235	37.06527
37.08574	340.1028	37.08574	286.4666	37.08574	188.2802	37.08574	319.2379	37.08574
37.1062	416.9926	37.1062	295.8672	37.1062	213.2605	37.1062	320.2467	37.1062
37.12666	489.888	37.12666	295.2735	37.12666	299.2479	37.12666	403.2615	37.12666
37.14712	602.7891	37.14712	324.6855	37.14712	300.2425	37.14712	472.2821	37.14712
37.16759	622.6959	37.16759	386.1033	37.16759	438.2443	37.16759	584.3087	37.16759
37.18805	702.6084	37.18805	518.5268	37.18805	540.2533	37.18805	677.3412	37.18805
37.20851	688.5265	37.20851	496.956	37.20851	571.2695	37.20851	831.3797	37.20851
37.22898	695.4503	37.22898	602.391	37.22898	734.2928	37.22898	852.424	37.22898
37.24944	670.3798	37.24944	718.8316	37.24944	761.3233	37.24944	923.4743	37.24944
37.2699	617.315	37.2699	788.278	37.2699	781.361	37.2699	901.5305	37.2699
37.29036	715.2558	37.29036	803.7301	37.29036	750.4058	37.29036	734.5927	37.29036
37.31083	689.2023	37.31083	751.1879	37.31083	762.4579	37.31083	652.6607	37.31083
37.33129	566.1545	37.33129	742.6515	37.33129	805.5171	37.33129	823.7347	37.33129
37.35175	598.1124	37.35175	749.1207	37.35175	801.5835	37.35175	799.8146	37.35175
37.37221	585.0759	37.37221	737.5958	37.37221	843.6571	37.37221	794.9004	37.37221
37.39268	489.0452	37.39268	736.0765	37.39268	808.7379	37.39268	729.9921	37.39268
37.41314	473.0201	37.41314	802.5629	37.41314	907.8258	37.41314	834.0898	37.41314
37.4336	384.0006	37.4336	766.0551	37.4336	848.9209	37.4336	844.1934	37.4336
37.45407	314.9869	37.45407	760.5529	37.45407	791.0232	37.45407	778.3029	37.45407
37.47453	297.9788	37.47453	730.0565	37.47453	733.1327	37.47453	717.4183	37.47453
37.49499	235.9764	37.49499	680.5659	37.49499	583.2493	37.49499	615.5397	37.49499
37.51545	198.9797	37.51545	629.0809	37.51545	452.3731	37.51545	515.6669	37.51545
37.53592	182.9887	37.53592	527.6017	37.53592	483.5042	37.53592	394.8002	37.53592
37.55638	139.0033	37.55638	374.1282	37.55638	278.6423	37.55638	377.9393	37.55638
37.57684	111.0236	37.57684	367.6604	37.57684	214.7877	37.57684	258.0844	37.57684
37.59731	79.04959	37.59731	316.1983	37.59731	212.9402	37.59731	122.2353	37.59731
37.61777	-14.9187	37.61777	189.742	37.61777	127.1	37.61777	138.3922	37.61777
37.63823	21.11859	37.63823	147.2914	37.63823	71.26686	37.63823	78.55505	37.63823
37.65869	52.1616	37.65869	94.84648	37.65869	32.44093	37.65869	42.72379	37.65869
37.67916	32.21029	37.67916	84.40731	37.67916	78.62219	37.67916	77.89847	37.67916
37.69962	-3.73535	37.69962	43.97386	37.69962	53.81062	37.69962	60.07906	37.69962
37.72008	50.3247	37.72008	78.54613	37.72008	73.00623	37.72008	26.26558	37.72008
37.74054	47.39041	37.74054	18.12412	37.74054	29.20902	37.74054	-8.54198	37.74054
37.76101	-0.53819	37.76101	-0.29216	37.76101	-13.581	37.76101	59.65638	37.76101
37.78147	45.53888	37.78147	17.29728	37.78147	-31.3639	37.78147	32.86067	37.78147
37.80193	75.62162	37.80193	59.89244	37.80193	34.86045	37.80193	18.07088	37.80193
37.8224	62.71004	37.8224	67.49332	37.8224	12.09195	37.8224	40.28702	37.8224
37.84286	28.80414	37.84286	68.09993	37.84286	-0.66937	37.84286	44.50908	37.84286
37.86332	44.90392	37.86332	22.71226	37.86332	-5.42352	37.86332	-9.26294	37.86332
37.88378	82.00937	37.88378	35.33031	37.88378	-3.17048	37.88378	57.97096	37.88378
37.90425	-5.87951	37.90425	71.95408	37.90425	23.08973	37.90425	30.21079	37.90425
37.92471	5.237295	37.92471	11.58357	37.92471	26.35712	37.92471	34.45654	37.92471
37.94517	40.35977	37.94517	22.21879	37.94517	11.63169	37.94517	81.70822	37.94517
37.96564	77.48793	37.96564	25.85973	37.96564	-21.0866	37.96564	61.96582	37.96564
37.9861	111.6218	37.9861	77.50639	37.9861	81.20237	37.9861	18.22934	37.9861
38.00656	95.76127	38.00656	101.1588	38.00656	34.49847	38.00656	102.4988	38.00656
38.02702	84.90645	38.02702	68.81688	38.02702	-7.19825	38.02702	76.77416	38.02702
38.04749	94.05731	38.04749	152.4807	38.04749	67.11221	38.04749	63.05545	38.04749
38.06795	164.2139	38.06795	107.1503	38.06795	21.42985	38.06795	83.34267	38.06795

38.08841	163.3761	38.08841	150.8255	38.08841	83.75467	38.08841	150.6358	38.08841
38.10887	173.5439	38.10887	133.5065	38.10887	143.0867	38.10887	155.9349	38.10887
38.12934	291.7175	38.12934	205.1933	38.12934	117.4258	38.12934	209.2399	38.12934
38.1498	225.8967	38.1498	196.8857	38.1498	154.7722	38.1498	234.5508	38.1498
38.17026	280.0817	38.17026	218.5838	38.17026	207.1257	38.17026	211.8676	38.17026
38.19073	289.2722	38.19073	282.2878	38.19073	200.4864	38.19073	272.1903	38.19073
38.21119	263.4685	38.21119	277.9973	38.21119	325.8543	38.21119	253.519	38.21119
38.23165	225.6705	38.23165	360.7127	38.23165	299.2294	38.23165	207.8536	38.23165
38.25211	197.8781	38.25211	318.4337	38.25211	219.6116	38.25211	215.1942	38.25211
38.27258	142.0914	38.27258	270.1605	38.27258	202.001	38.27258	206.5406	38.27258
38.29304	153.3104	38.29304	225.893	38.29304	201.3976	38.29304	178.893	38.29304
38.3135	99.53502	38.3135	270.6312	38.3135	178.8014	38.3135	162.2513	38.3135
38.33397	100.7653	38.33397	199.3752	38.33397	189.2124	38.33397	150.6155	38.33397
38.35443	111.0013	38.35443	137.1248	38.35443	113.6305	38.35443	44.98565	38.35443
38.37489	16.24302	38.37489	137.8802	38.37489	145.0558	38.37489	98.36172	38.37489
38.39535	32.49038	38.39535	146.6413	38.39535	67.4883	38.39535	23.74371	38.39535
38.41582	73.74341	38.41582	115.4081	38.41582	99.92797	38.41582	46.13163	38.41582
38.43628	19.00211	38.43628	91.1807	38.43628	40.37481	38.43628	87.52547	38.43628
38.45674	19.2665	38.45674	85.95897	38.45674	48.82884	38.45674	17.92523	38.45674
38.4772	69.53655	38.4772	78.74297	38.4772	42.29004	38.4772	24.33091	38.4772
38.49767	45.81229	38.49767	46.53269	38.49767	17.75842	38.49767	54.74252	38.49767
38.51813	53.0937	38.51813	59.32813	38.51813	15.23398	38.51813	18.16005	38.51813
38.53859	2.38079	38.53859	73.1293	38.53859	36.71671	38.53859	-0.41649	38.53859
38.55906	61.67355	38.55906	30.93618	38.55906	62.20663	38.55906	34.01289	38.55906
38.57952	42.97199	38.57952	44.74879	38.57952	66.70372	38.57952	34.44819	38.57952
38.59998	100.2761	38.59998	111.5671	38.59998	24.20799	38.59998	81.88941	38.59998
38.62044	27.5859	38.62044	137.3912	38.62044	76.71944	38.62044	100.3366	38.62044
38.64091	124.9014	38.64091	164.2209	38.64091	80.23807	38.64091	89.78964	38.64091
38.66137	169.2225	38.66137	111.0565	38.66137	116.7639	38.66137	112.2486	38.66137
38.68183	177.5493	38.68183	124.8977	38.68183	69.29686	38.68183	169.7135	38.68183
38.7023	177.8818	38.7023	200.7446	38.7023	132.837	38.7023	130.1844	38.7023
38.72276	231.22	38.72276	212.5973	38.72276	155.3844	38.72276	220.6612	38.72276
38.74322	197.5639	38.74322	243.4557	38.74322	189.9389	38.74322	227.1438	38.74322
38.76368	243.9134	38.76368	309.3198	38.76368	204.5006	38.76368	295.6325	38.76368
38.78415	230.2686	38.78415	307.1896	38.78415	147.0695	38.78415	309.127	38.78415
38.80461	211.6295	38.80461	399.0652	38.80461	202.6455	38.80461	259.6275	38.80461
38.82507	183.996	38.82507	282.9464	38.82507	211.2287	38.82507	249.1339	38.82507
38.84553	198.3682	38.84553	327.8334	38.84553	230.8192	38.84553	216.6461	38.84553
38.866	173.7462	38.866	329.7262	38.866	172.4167	38.866	202.1644	38.866
38.88646	256.1297	38.88646	325.6246	38.88646	169.0215	38.88646	161.6885	38.88646
38.90692	217.519	38.90692	244.5288	38.90692	200.6334	38.90692	174.2186	38.90692
38.92739	256.9139	38.92739	254.4386	38.92739	187.2526	38.92739	188.7546	38.92739
38.94785	240.3145	38.94785	257.3542	38.94785	161.8789	38.94785	202.2965	38.94785
38.96831	121.7208	38.96831	161.2756	38.96831	176.5123	38.96831	89.84438	38.96831
38.98877	125.1328	38.98877	150.2027	38.98877	153.153	38.98877	98.39816	38.98877
39.00924	144.5504	39.00924	174.1354	39.00924	234.8008	39.00924	172.9579	39.00924
39.0297	96.97374	39.0297	114.0739	39.0297	159.4558	39.0297	103.5235	39.0297
39.05016	84.40273	39.05016	150.0181	39.05016	179.118	39.05016	65.09501	39.05016
39.07063	48.8374	39.07063	60.96809	39.07063	203.7874	39.07063	59.67248	39.07063
39.09109	40.27774	39.09109	113.9238	39.09109	116.464	39.09109	47.25587	39.09109
39.11155	41.72376	39.11155	96.88515	39.11155	119.1477	39.11155	68.84518	39.11155
39.13201	81.17546	39.13201	131.8522	39.13201	98.83859	39.13201	68.44041	39.13201
39.15248	27.63283	39.15248	109.8251	39.15248	62.53667	39.15248	-2.95843	39.15248
39.17294	-17.9041	39.17294	42.80364	39.17294	25.24193	39.17294	60.64866	39.17294
39.1934	44.5646	39.1934	30.78792	39.1934	29.95437	39.1934	78.26166	39.1934
39.21386	17.039	39.21386	11.77792	39.21386	33.67398	39.21386	-9.11941	39.21386
39.23433	8.51908	39.23433	12.77364	39.23433	30.40078	39.23433	-35.4946	39.23433
39.25479	10.00483	39.25479	2.775089	39.25479	29.13475	39.25479	9.501879	39.25479

39.27525	60.49626	39.27525	42.78226	39.27525	22.8759	39.27525	2.50424	39.27525
39.29572	5.993368	39.29572	-2.20485	39.29572	10.62423	39.29572	-13.4875	39.29572
39.31618	-20.5039	39.31618	64.81376	39.31618	11.37974	39.31618	-9.47327	39.31618
39.33664	-48.9954	39.33664	67.83809	39.33664	-41.8576	39.33664	13.54686	39.33664
39.3571	20.36661	39.3571	33.86815	39.3571	18.91229	39.3571	73.57291	39.3571
39.37757	58.7343	39.37757	43.90393	39.37757	15.68933	39.37757	-29.3951	39.37757
39.39803	17.10765	39.39803	-7.05457	39.39803	-22.5265	39.39803	21.6428	39.39803
39.41849	0.486686	39.41849	19.99265	39.41849	20.26495	39.41849	3.686621	39.41849
39.43896	33.8714	39.43896	45.0456	39.43896	5.063521	39.43896	-7.26363	39.43896
39.45942	-25.7382	39.45942	17.10426	39.45942	35.86928	39.45942	67.79204	39.45942
39.47988	7.657844	39.47988	35.16866	39.47988	40.68221	39.47988	6.853636	39.47988
39.50034	46.05958	39.50034	23.23877	39.50034	42.50232	39.50034	-10.0788	39.50034
39.52081	6.466997	39.52081	24.3146	39.52081	37.32961	39.52081	4.994597	39.52081
39.54127	34.88009	39.54127	27.39616	39.54127	-2.83592	39.54127	26.07396	39.54127
39.56173	5.298855	39.56173	-14.5166	39.56173	-21.9943	39.56173	39.15925	39.56173
39.58219	17.7233	39.58219	15.57644	39.58219	3.098666	39.58219	21.25046	39.58219
39.60266	0.153417	39.60266	20.67516	39.60266	34.19879	39.60266	1.3476	39.60266
39.62312	14.58921	39.62312	18.77961	39.62312	34.30609	39.62312	-3.54934	39.62312
39.64358	-25.9693	39.64358	8.889779	39.64358	-8.57943	39.64358	-8.44036	39.64358
39.66405	16.47783	39.66405	-25.9943	39.66405	13.54222	39.66405	12.67455	39.66405
39.68451	17.93066	39.68451	-9.32174	39.68451	12.67106	39.68451	25.79538	39.68451
39.70497	-38.6108	39.70497	-2.64342	39.70497	13.80707	39.70497	11.92213	39.70497
39.72543	51.85333	39.72543	63.04062	39.72543	25.95026	39.72543	-25.9452	39.72543
39.7459	3.323184	39.7459	16.73037	39.7459	5.100634	39.7459	-1.80659	39.7459
39.76636	33.79871	39.76636	6.425854	39.76636	39.25818	39.76636	25.33793	39.76636
39.78682	-6.72008	39.78682	-33.8729	39.78682	11.42291	39.78682	23.48838	39.78682
39.80729	33.7668	39.80729	30.83398	39.80729	10.59481	39.80729	-5.35525	39.80729
39.82775	35.25935	39.82775	30.54663	39.82775	26.77389	39.82775	53.80704	39.82775
39.84821	11.75759	39.84821	34.265	39.84821	8.960155	39.84821	-28.0247	39.84821
39.86867	32.2615	39.86867	36.9891	39.86867	53.15359	39.86867	76.1494	39.86867
39.88914	0.771083	39.88914	9.718911	39.88914	45.35421	39.88914	-15.6705	39.88914
39.9096	-20.7137	39.9096	30.45445	39.9096	36.562	39.9096	-1.48455	39.9096
39.93006	55.80728	39.93006	8.195708	39.93006	-4.22302	39.93006	57.70736	39.93006
39.95052	41.3339	39.95052	45.94269	39.95052	-6.00087	39.95052	5.905196	39.95052
39.97099	32.86619	39.97099	10.6954	39.97099	-21.7715	39.97099	-22.891	39.97099
39.99145	33.40415	39.99145	16.45382	39.99145	2.835217	39.99145	-8.26199	39.99145
40.01191	74.94779	40.01191	26.21797	40.01191	34.44915	40.01191	25.37299	40.01191
40.03238	36.49712	40.03238	31.98784	40.03238	-17.9297	40.03238	3.013898	40.03238
40.05284	87.05211	40.05284	32.76344	40.05284	36.69856	40.05284	32.66073	40.05284
40.0733	28.61278	40.0733	41.54475	40.0733	-28.666	40.0733	25.31348	40.0733
40.09376	-5.82087	40.09376	27.33179	40.09376	28.97668	40.09376	40.97215	40.09376
40.11423	37.75115	40.11423	39.12455	40.11423	1.626504	40.11423	47.63675	40.11423
40.13469	51.32885	40.13469	43.92304	40.13469	26.28351	40.13469	62.30727	40.13469
40.15515	21.91223	40.15515	31.72724	40.15515	23.94769	40.15515	-25.0163	40.15515
40.17562	52.50128	40.17562	38.53717	40.17562	30.61905	40.17562	33.66609	40.17562
40.19608	41.096	40.19608	29.35282	40.19608	38.29759	40.19608	30.35438	40.19608
40.21654	46.69641	40.21654	66.17419	40.21654	33.98331	40.21654	55.0486	40.21654
40.237	64.30249	40.237	14.00128	40.237	53.67621	40.237	20.74874	40.237
40.25747	18.91425	40.25747	82.8341	40.25747	73.37628	40.25747	80.4548	40.25747
40.27793	47.53168	40.27793	46.67264	40.27793	61.08353	40.27793	70.16679	40.27793
40.29839	100.1548	40.29839	64.5169	40.29839	34.79796	40.29839	62.88469	40.29839
40.31885	54.78357	40.31885	52.36689	40.31885	84.51957	40.31885	29.60853	40.31885
40.33932	50.41803	40.33932	32.22259	40.33932	24.24836	40.33932	23.33828	40.33932
40.35978	80.05817	40.35978	66.08402	40.35978	85.98432	40.35978	59.07396	40.35978
40.38024	78.70398	40.38024	71.95117	40.38024	60.72747	40.38024	31.81557	40.38024
40.40071	131.3555	40.40071	65.82404	40.40071	85.47778	40.40071	106.5631	40.40071
40.42117	68.01264	40.42117	43.70264	40.42117	114.2353	40.42117	96.31654	40.42117
40.44163	78.67548	40.44163	85.58695	40.44163	50.99997	40.44163	66.07592	40.44163

40.46209	43.344	40.46209	96.477	40.46209	111.7718	40.46209	105.8412	40.46209
40.48256	45.01819	40.48256	103.3728	40.48256	152.5509	40.48256	111.6124	40.48256
40.50302	89.69806	40.50302	81.27424	40.50302	136.3371	40.50302	89.38958	40.50302
40.52348	30.38361	40.52348	90.18145	40.52348	75.13046	40.52348	48.17265	40.52348
40.54395	-7.92517	40.54395	72.09438	40.54395	73.93102	40.54395	85.96164	40.54395
40.56441	13.77173	40.56441	55.01303	40.56441	60.73877	40.56441	34.75655	40.56441
40.58487	-44.5257	40.58487	35.93741	40.58487	85.5537	40.58487	23.55739	40.58487
40.60533	21.18256	40.60533	6.867499	40.60533	68.3758	40.60533	34.36415	40.60533
40.6258	-14.1035	40.6258	-13.1967	40.6258	74.20508	40.6258	27.17684	40.6258
40.64626	-3.38391	40.64626	50.74486	40.64626	48.04154	40.64626	37.99545	40.64626
40.66672	-27.6586	40.66672	30.69212	40.66672	43.88518	40.66672	19.81998	40.66672
40.68718	-6.98056	40.68718	47.64511	40.68718	-6.26401	40.68718	32.65043	40.68718
40.70765	-5.2968	40.70765	-15.3962	40.70765	27.59399	40.70765	22.48681	40.70765
40.72811	2.392631	40.72811	18.56824	40.72811	7.459158	40.72811	22.32911	40.72811
40.74857	-1.91226	40.74857	-20.4616	40.74857	12.33151	40.74857	13.17734	40.74857
40.76904	-4.21148	40.76904	39.51427	40.76904	56.21104	40.76904	-5.96851	40.76904
40.7895	-7.50502	40.7895	1.495867	40.7895	43.09774	40.7895	-23.1084	40.7895
40.80996	-8.79289	40.80996	32.48319	40.80996	21.99163	40.80996	3.529004	40.80996
40.83042	-17.0751	40.83042	16.47623	40.83042	48.89269	40.83042	3.172371	40.83042
40.85089	5.648415	40.85089	4.474991	40.85089	-7.19907	40.85089	8.821662	40.85089
40.87135	-8.62242	40.87135	-8.52052	40.87135	-27.2836	40.87135	-4.52312	40.87135
40.89181	-7.88758	40.89181	-7.51031	40.89181	32.63895	40.89181	15.13801	40.89181
40.91228	37.85294	40.91228	-46.4944	40.91228	-7.43128	40.91228	-1.19493	40.91228
40.93274	-20.4009	40.93274	8.103943	40.93274	-32.4943	40.93274	-14.5219	40.93274
40.9532	-24.649	40.9532	19.70799	40.9532	7.727227	40.9532	18.15697	40.9532
40.97366	-15.8915	40.97366	9.317763	40.97366	30.95595	40.97366	-10.1582	40.97366
40.99413	-23.1282	40.99413	-11.0667	40.99413	33.19186	40.99413	-5.46745	40.99413
41.01459	23.64066	41.01459	61.55447	41.01459	43.43494	41.01459	15.22923	41.01459
41.03505	-11.5848	41.03505	17.18141	41.03505	24.6852	41.03505	5.931835	41.03505
41.05551	63.19548	41.05551	15.81407	41.05551	41.94264	41.05551	11.64036	41.05551
41.07598	-15.0186	41.07598	5.452455	41.07598	30.20726	41.07598	14.35481	41.07598
41.09644	-0.22699	41.09644	41.09656	41.09644	28.47906	41.09644	1.075181	41.09644
41.1169	43.57029	41.1169	30.74639	41.1169	39.75803	41.1169	-7.19852	41.1169
41.13737	28.37324	41.13737	64.40194	41.13737	56.04419	41.13737	4.533696	41.13737
41.15783	-12.8181	41.15783	35.06321	41.15783	24.33752	41.15783	8.271838	41.15783
41.17829	-9.00382	41.17829	13.7302	41.17829	65.63802	41.17829	36.0159	41.17829
41.19875	11.81616	41.19875	11.40292	41.19875	66.94571	41.19875	9.765894	41.19875
41.21922	66.64182	41.21922	69.08136	41.21922	61.26057	41.21922	18.52181	41.21922
41.23968	11.47315	41.23968	68.76552	41.23968	21.58262	41.23968	17.28364	41.23968
41.26014	-31.6898	41.26014	-9.5446	41.26014	56.91184	41.26014	79.0514	41.26014
41.28061	-4.84715	41.28061	-19.849	41.28061	53.24824	41.28061	-23.1749	41.28061
41.30107	-14.9988	41.30107	-1.14766	41.30107	59.59182	41.30107	-6.39531	41.30107
41.32153	-1.14475	41.32153	19.55939	41.32153	31.94257	41.32153	4.390223	41.32153
41.34199	-18.285	41.34199	-16.7278	41.34199	4.300508	41.34199	-20.8183	41.34199
41.36246	-0.41964	41.36246	-24.0093	41.36246	30.66562	41.36246	-4.02095	41.36246
41.38292	-11.5486	41.38292	35.71487	41.38292	23.03791	41.38292	51.78235	41.38292
41.40338	5.328172	41.40338	-22.5552	41.40338	24.41738	41.40338	16.59158	41.40338
41.42384	-7.78941	41.42384	-20.8195	41.42384	21.80403	41.42384	26.40673	41.42384
41.44431	-6.90131	41.44431	25.92186	41.44431	44.19785	41.44431	2.227796	41.44431
41.46477	-22.0075	41.46477	15.66897	41.46477	25.59885	41.46477	38.05479	41.46477
41.48523	33.89191	41.48523	16.4218	41.48523	44.00703	41.48523	-5.11229	41.48523
41.5057	47.79704	41.5057	5.180349	41.5057	43.42239	41.5057	56.72655	41.5057
41.52616	45.70784	41.52616	-8.05538	41.52616	0.844933	41.52616	84.57131	41.52616
41.54662	87.62431	41.54662	23.71462	41.54662	20.27465	41.54662	74.42201	41.54662
41.56708	121.5465	41.56708	60.49034	41.56708	47.71154	41.56708	90.27861	41.56708
41.58755	111.4743	41.58755	9.271777	41.58755	74.15562	41.58755	54.14115	41.58755
41.60801	123.4078	41.60801	37.05894	41.60801	102.6069	41.60801	35.00961	41.60801
41.62847	91.34699	41.62847	45.85183	41.62847	73.06529	41.62847	47.88399	41.62847

41.64894	122.2918	41.64894	64.65043	41.64894	168.5309	41.64894	123.7643	41.64894
41.6694	101.2424	41.6694	72.45477	41.6694	215.0037	41.6694	125.6505	41.6694
41.68986	109.1986	41.68986	117.2648	41.68986	165.4836	41.68986	147.5427	41.68986
41.71032	110.1605	41.71032	83.08059	41.71032	222.9708	41.71032	154.4408	41.71032
41.73079	66.12804	41.73079	129.9021	41.73079	181.4651	41.73079	111.3447	41.73079
41.75125	115.1013	41.75125	154.7293	41.75125	215.9666	41.75125	97.25467	41.75125
41.77171	85.08019	41.77171	109.5622	41.77171	226.4753	41.77171	78.17052	41.77171
41.79217	78.06478	41.79217	137.4009	41.79217	225.9911	41.79217	107.0923	41.79217
41.81264	80.05505	41.81264	100.2453	41.81264	129.5142	41.81264	71.01998	41.81264
41.8331	48.05099	41.8331	147.0954	41.8331	137.0444	41.8331	107.9536	41.8331
41.85356	35.05261	41.85356	70.95123	41.85356	108.5818	41.85356	34.89314	41.85356
41.87403	10.05991	41.87403	66.81279	41.87403	91.12634	41.87403	24.8386	41.87403
41.89449	8.072878	41.89449	90.68006	41.89449	94.67808	41.89449	44.78999	41.89449
41.91495	86.09152	41.91495	33.55306	41.91495	44.237	41.91495	15.74729	41.91495
41.93541	15.11585	41.93541	-1.56822	41.93541	41.8031	41.93541	11.71053	41.93541
41.95588	21.14585	41.95588	7.316222	41.95588	28.37639	41.95588	7.679685	41.95588
41.97634	24.18153	41.97634	-6.79361	41.97634	-24.0432	41.97634	-23.3452	41.97634
41.9968	120.2229	41.9968	-6.89773	41.9968	12.54448	41.9968	-11.4859	41.9968
42.01727	31.26991	42.01727	4.003881	42.01727	10.13929	42.01727	-13.6207	42.01727
42.03773	87.32262	42.03773	-16.0888	42.03773	45.74128	42.03773	19.2505	42.03773
42.05819	45.38099	42.05819	16.82427	42.05819	46.35046	42.05819	21.12759	42.05819
42.07865	108.4451	42.07865	5.743042	42.07865	42.96681	42.07865	-3.98939	42.07865
42.09912	38.51479	42.09912	27.66754	42.09912	39.59033	42.09912	40.89954	42.09912
42.11958	46.5902	42.11958	30.59776	42.11958	50.22104	42.11958	13.79441	42.11958
42.14004	39.67128	42.14004	-10.4663	42.14004	59.85892	42.14004	48.69519	42.14004
42.1605	65.75804	42.1605	52.47537	42.1605	1.50398	42.1605	-19.3981	42.1605
42.18097	86.85048	42.18097	36.42276	42.18097	71.15622	42.18097	35.51453	42.18097
42.20143	54.9486	42.20143	20.37587	42.20143	60.81564	42.20143	46.43309	42.20143
42.22189	113.0524	42.22189	27.3347	42.22189	62.48223	42.22189	13.35757	42.22189
42.24236	67.16185	42.24236	22.29925	42.24236	88.15601	42.24236	57.28797	42.24236
42.26282	148.277	42.26282	5.269529	42.26282	38.83696	42.26282	11.2243	42.26282
42.28328	177.3978	42.28328	-33.7545	42.28328	18.52509	42.28328	31.16655	42.28328
42.30374	182.5243	42.30374	71.22725	42.30374	63.2204	42.30374	-5.88528	42.30374
42.32421	224.6565	42.32421	50.2147	42.32421	99.92288	42.32421	16.06882	42.32421
42.34467	241.7943	42.34467	7.207859	42.34467	64.63255	42.34467	57.02884	42.34467
42.36513	240.9379	42.36513	59.20675	42.36513	106.3494	42.36513	51.99478	42.36513
42.3856	190.0871	42.3856	51.21136	42.3856	79.07341	42.3856	77.96665	42.3856
42.40606	249.2419	42.40606	75.22169	42.40606	92.80461	42.40606	98.94444	42.40606
42.42652	294.4025	42.42652	109.2377	42.42652	78.54299	42.42652	25.92815	42.42652
42.44698	337.5687	42.44698	140.2595	42.44698	107.2885	42.44698	60.91779	42.44698
42.46745	431.7406	42.46745	138.287	42.46745	98.04128	42.46745	116.9134	42.46745
42.48791	501.9182	42.48791	146.3203	42.48791	148.8012	42.48791	193.9148	42.48791
42.50837	449.1014	42.50837	216.3592	42.50837	189.5683	42.50837	251.9222	42.50837
42.52883	529.2904	42.52883	241.4039	42.52883	212.3425	42.52883	267.9356	42.52883
42.5493	508.485	42.5493	220.4542	42.5493	206.124	42.5493	356.9548	42.5493
42.56976	472.6853	42.56976	290.5103	42.56976	274.9126	42.56976	394.98	42.56976
42.59022	389.8912	42.59022	310.5722	42.59022	321.7084	42.59022	521.0111	42.59022
42.61069	373.1029	42.61069	360.6397	42.61069	455.5114	42.61069	586.0482	42.61069
42.63115	340.3202	42.63115	351.713	42.63115	399.3216	42.63115	546.0911	42.63115
42.65161	357.5432	42.65161	342.792	42.65161	396.1389	42.65161	423.14	42.65161
42.67207	287.7719	42.67207	342.8767	42.67207	376.9634	42.67207	341.1948	42.67207
42.69254	198.0062	42.69254	253.9672	42.69254	384.7951	42.69254	355.2555	42.69254
42.713	247.2462	42.713	236.0634	42.713	384.634	42.713	285.3221	42.713
42.73346	169.4919	42.73346	250.1653	42.73346	355.48	42.73346	347.3947	42.73346
42.75393	206.7433	42.75393	238.2729	42.75393	321.3333	42.75393	253.4732	42.75393
42.77439	278.0003	42.77439	182.3862	42.77439	245.1937	42.77439	218.5576	42.77439
42.79485	210.263	42.79485	136.5052	42.79485	236.0612	42.79485	161.6479	42.79485
42.81531	181.5314	42.81531	124.63	42.81531	219.936	42.81531	195.7442	42.81531

42.83578	262.8055	42.83578	173.7605	42.83578	192.818	42.83578	259.8463	42.83578
42.85624	221.0853	42.85624	75.89676	42.85624	171.7071	42.85624	252.9545	42.85624
42.8767	266.3707	42.8767	133.0387	42.8767	182.6034	42.8767	320.0685	42.8767
42.89716	351.6618	42.89716	159.1864	42.89716	229.5069	42.89716	424.1884	42.89716
42.91763	438.9586	42.91763	203.3398	42.91763	288.4175	42.91763	482.3143	42.91763
42.93809	499.261	42.93809	187.4989	42.93809	224.3353	42.93809	577.4461	42.93809
42.95855	579.5692	42.95855	258.6637	42.95855	344.2603	42.95855	598.5839	42.95855
42.97902	685.883	42.97902	290.8343	42.97902	457.1925	42.97902	552.7275	42.97902
42.99948	802.2025	42.99948	313.0106	42.99948	547.1319	42.99948	575.8771	42.99948
43.01994	917.5276	43.01994	342.1925	43.01994	628.0784	43.01994	698.0325	43.01994
43.0404	1089.858	43.0404	490.3803	43.0404	698.0322	43.0404	815.194	43.0404
43.06087	1123.195	43.06087	542.5737	43.06087	812.993	43.06087	1024.361	43.06087
43.08133	1220.537	43.08133	601.7729	43.08133	1032.961	43.08133	1057.535	43.08133
43.10179	1208.885	43.10179	743.9778	43.10179	1233.936	43.10179	1223.714	43.10179
43.12226	1104.239	43.12226	843.1884	43.12226	1381.919	43.12226	1298.899	43.12226
43.14272	1060.598	43.14272	946.4047	43.14272	1447.908	43.14272	1406.09	43.14272
43.16318	995.9627	43.16318	1056.627	43.16318	1389.905	43.16318	1300.287	43.16318
43.18364	871.3333	43.18364	1025.855	43.18364	1278.909	43.18364	1136.49	43.18364
43.20411	892.7095	43.20411	939.0881	43.20411	1182.92	43.20411	1102.699	43.20411
43.22457	843.0914	43.22457	913.3273	43.22457	1080.939	43.22457	1054.913	43.22457
43.24503	808.479	43.24503	911.5723	43.24503	1070.964	43.24503	1072.134	43.24503
43.26549	640.8723	43.26549	838.8229	43.26549	1004.997	43.26549	1104.36	43.26549
43.28596	508.2712	43.28596	802.0793	43.28596	941.0367	43.28596	905.593	43.28596
43.30642	464.6759	43.30642	755.3414	43.30642	777.0838	43.30642	791.8314	43.30642
43.32688	419.0862	43.32688	639.6093	43.32688	747.138	43.32688	663.0757	43.32688
43.34735	381.5021	43.34735	566.8828	43.34735	635.1994	43.34735	503.3261	43.34735
43.36781	310.9238	43.36781	512.1621	43.36781	484.268	43.36781	407.5822	43.36781
43.38827	300.3511	43.38827	413.4471	43.38827	384.3437	43.38827	376.8444	43.38827
43.40873	190.7841	43.40873	302.7378	43.40873	332.4266	43.40873	352.1124	43.40873
43.4292	215.2228	43.4292	320.0343	43.4292	262.5168	43.4292	242.3864	43.4292
43.44966	164.6672	43.44966	241.3364	43.44966	242.614	43.44966	192.6662	43.44966
43.47012	120.1172	43.47012	194.6443	43.47012	194.7185	43.47012	156.9521	43.47012
43.49059	65.57294	43.49059	165.9579	43.49059	158.8302	43.49059	122.2438	43.49059
43.51105	67.03433	43.51105	71.27729	43.51105	131.949	43.51105	76.54144	43.51105
43.53151	56.50139	43.53151	114.6023	43.53151	90.07497	43.53151	23.84502	43.53151
43.55197	43.97413	43.55197	37.93313	43.55197	59.20815	43.55197	61.15452	43.55197
43.57244	32.45255	43.57244	54.26963	43.57244	43.3485	43.57244	71.46995	43.57244
43.5929	47.93664	43.5929	36.61186	43.5929	15.49604	43.5929	56.7913	43.5929
43.61336	73.42641	43.61336	3.959808	43.61336	76.65075	43.61336	47.11857	43.61336
43.63382	63.92186	43.63382	47.31348	43.63382	-12.1874	43.63382	52.45177	43.63382
43.65429	7.422977	43.65429	22.67287	43.65429	-10.0183	43.65429	40.79089	43.65429
43.67475	67.92978	43.67475	17.03799	43.67475	8.157954	43.67475	81.13593	43.67475
43.69521	38.44225	43.69521	-10.5912	43.69521	30.34138	43.69521	60.4869	43.69521
43.71568	-29.0396	43.71568	24.78539	43.71568	3.531982	43.71568	21.84379	43.71568
43.73614	35.48423	43.73614	34.16767	43.73614	5.729763	43.73614	48.20661	43.73614
43.7566	41.01373	43.7566	-9.44433	43.7566	29.93472	43.7566	35.57534	43.7566
43.77706	8.548907	43.77706	38.9494	43.77706	10.14686	43.77706	21.95001	43.77706
43.79753	15.08976	43.79753	34.34885	43.79753	67.36617	43.79753	16.33059	43.79753
43.81799	4.636293	43.81799	46.75403	43.81799	74.59267	43.81799	70.7171	43.81799
43.83845	86.1885	43.83845	79.16492	43.83845	67.82634	43.83845	57.10953	43.83845
43.85892	28.74638	43.85892	39.58154	43.85892	65.06719	43.85892	98.50789	43.85892
43.87938	106.3099	43.87938	89.00388	43.87938	37.31522	43.87938	109.9122	43.87938
43.89984	137.8792	43.89984	66.43194	43.89984	86.57043	43.89984	160.3224	43.89984
43.9203	123.4541	43.9203	89.86572	43.9203	92.83281	43.9203	177.7385	43.9203
43.94077	99.03468	43.94077	92.30522	43.94077	94.10237	43.94077	120.1605	43.94077
43.96123	36.62094	43.96123	109.7505	43.96123	147.3791	43.96123	143.5885	43.96123
43.98169	71.21288	43.98169	95.2014	43.98169	137.663	43.98169	142.0224	43.98169
44.00215	84.8105	44.00215	129.6581	44.00215	159.9541	44.00215	163.4622	44.00215

44.02262	59.41379	44.02262	81.12047	44.02262	197.2524	44.02262	102.908	44.02262
44.04308	16.02276	44.04308	172.5886	44.04308	156.5579	44.04308	104.3596	44.04308
44.06354	55.63741	44.06354	114.0624	44.06354	169.8705	44.06354	114.8172	44.06354
44.08401	8.257727	44.08401	88.54199	44.08401	138.1903	44.08401	73.28074	44.08401
44.10447	105.8837	44.10447	94.02728	44.10447	96.51729	44.10447	110.7502	44.10447
44.12493	54.5154	44.12493	55.51828	44.12493	132.8515	44.12493	101.2255	44.12493
44.14539	113.1527	44.14539	63.01501	44.14539	68.1928	44.14539	46.70682	44.14539
44.16586	87.79577	44.16586	85.51746	44.16586	16.54132	44.16586	45.19403	44.16586
44.18632	81.44447	44.18632	77.02564	44.18632	44.89702	44.18632	46.68716	44.18632
44.20678	51.09885	44.20678	57.53953	44.20678	45.2599	44.20678	5.18621	44.20678
44.22725	53.75891	44.22725	55.05915	44.22725	35.62996	44.22725	40.69119	44.22725
44.24771	49.42464	44.24771	71.58449	44.24771	45.0072	44.24771	16.20209	44.24771
44.26817	94.09605	44.26817	42.11555	44.26817	35.39161	44.26817	66.71891	44.26817
44.28863	127.7731	44.28863	6.652335	44.28863	19.7832	44.28863	72.24166	44.28863
44.3091	122.4559	44.3091	77.19484	44.3091	-4.81803	44.3091	49.77033	44.3091
44.32956	185.1443	44.32956	74.74307	44.32956	69.58791	44.32956	53.30492	44.32956
44.35002	116.8384	44.35002	106.297	44.35002	119.001	44.35002	54.84544	44.35002
44.37048	177.5382	44.37048	105.8567	44.37048	93.42135	44.37048	124.3919	44.37048
44.39095	194.2437	44.39095	137.4221	44.39095	80.84883	44.39095	105.9442	44.39095
44.41141	243.9548	44.41141	177.9932	44.41141	135.2835	44.41141	125.5025	44.41141
44.43187	234.6716	44.43187	209.5701	44.43187	190.7253	44.43187	208.0667	44.43187
44.45234	255.3941	44.45234	254.1526	44.45234	297.1743	44.45234	218.6369	44.45234
44.4728	243.1223	44.4728	328.7409	44.4728	375.6306	44.4728	288.213	44.4728
44.49326	235.8561	44.49326	303.3349	44.49326	436.0939	44.49326	248.7949	44.49326
44.51372	224.5957	44.51372	356.9346	44.51372	447.5645	44.51372	245.3828	44.51372
44.53419	201.3409	44.53419	359.5401	44.53419	382.0422	44.53419	276.9767	44.53419
44.55465	192.0917	44.55465	383.1513	44.55465	334.5271	44.55465	208.5764	44.55465
44.57511	174.8483	44.57511	290.7682	44.57511	277.0192	44.57511	218.1821	44.57511
44.59558	120.6105	44.59558	269.3908	44.59558	262.5185	44.59558	165.7937	44.59558
44.61604	80.3784	44.61604	243.0191	44.61604	306.0249	44.61604	196.4112	44.61604
44.6365	86.15199	44.6365	187.6532	44.6365	247.5385	44.6365	129.0347	44.6365
44.65696	84.93124	44.65696	168.293	44.65696	159.0593	44.65696	107.664	44.65696
44.67743	104.7162	44.67743	122.9385	44.67743	201.5873	44.67743	128.2993	44.67743
44.69789	43.50677	44.69789	143.5897	44.69789	116.1225	44.69789	51.94053	44.69789
44.71835	32.30306	44.71835	135.2467	44.71835	88.66478	44.71835	43.58767	44.71835
44.73881	62.10502	44.73881	54.90935	44.73881	46.21429	44.73881	39.24073	44.73881
44.75928	50.91265	44.75928	46.57775	44.75928	37.77099	44.75928	16.89972	44.75928
44.77974	29.72596	44.77974	111.2519	44.77974	67.33485	44.77974	7.564631	44.77974
44.8002	-26.4551	44.8002	34.93171	44.8002	55.9059	44.8002	67.23547	44.8002
44.82067	17.36961	44.82067	17.61727	44.82067	3.484122	44.82067	12.91222	44.82067
44.84113	6.199946	44.84113	38.30856	44.84113	66.06953	44.84113	8.594901	44.84113
44.86159	12.03596	44.86159	28.00557	44.86159	-43.3379	44.86159	-1.7165	44.86159
44.88205	5.877653	44.88205	-5.2917	44.88205	55.26186	44.88205	50.97803	44.88205
44.90252	-33.275	44.90252	24.41676	44.90252	-15.1312	44.90252	9.678483	44.90252
44.92298	37.57806	44.92298	-18.8691	44.92298	-16.5171	44.92298	34.38486	44.92298
44.94344	13.43678	44.94344	-15.1492	44.94344	-10.8958	44.94344	-6.90284	44.94344
44.96391	-26.6988	44.96391	24.57645	44.96391	32.73268	44.96391	6.815376	44.96391
44.98437	2.171252	44.98437	6.307796	44.98437	22.36833	44.98437	5.539521	44.98437
45.00483	46.047	45.00483	16.04486	45.00483	1.011159	45.00483	-4.73041	45.00483
45.02529	64.92842	45.02529	-38.2123	45.02529	10.66117	45.02529	-30.9944	45.02529
45.04576	75.81553	45.04576	0.536161	45.04576	11.31835	45.04576	5.622198	45.04576
45.06622	3.708302	45.06622	-15.7096	45.06622	15.98271	45.06622	13.24474	45.06622
45.08668	50.60675	45.08668	53.05035	45.08668	-3.34575	45.08668	66.8732	45.08668
45.10714	82.51089	45.10714	-19.184	45.10714	9.332971	45.10714	-6.49241	45.10714
45.12761	68.42069	45.12761	-4.41258	45.12761	15.01887	45.12761	21.1479	45.12761
45.14807	61.33617	45.14807	32.36455	45.14807	0.711943	45.14807	0.794133	45.14807
45.16853	59.25733	45.16853	17.14739	45.16853	1.412196	45.16853	16.44629	45.16853
45.189	82.18416	45.189	52.93596	45.189	-1.88037	45.189	44.10437	45.189

45.20946	67.11667	45.20946	38.73025	45.20946	29.83424	45.20946	9.768376	45.20946
45.22992	102.0549	45.22992	16.53026	45.22992	70.55602	45.22992	5.438304	45.22992
45.25038	45.99872	45.25038	37.33599	45.25038	110.285	45.25038	15.11416	45.25038
45.27085	36.94826	45.27085	37.14745	45.27085	118.0211	45.27085	64.79593	45.27085
45.29131	65.90347	45.29131	37.96462	45.29131	76.76446	45.29131	65.48363	45.29131
45.31177	103.8644	45.31177	111.7875	45.31177	144.515	45.31177	59.17725	45.31177
45.33224	73.83093	45.33224	84.61615	45.33224	71.27264	45.33224	85.87679	45.33224
45.3527	69.80318	45.3527	60.45049	45.3527	101.0375	45.3527	81.58226	45.3527
45.37316	70.78109	45.37316	100.2906	45.37316	99.80953	45.37316	83.29366	45.37316
45.39362	33.76469	45.39362	43.13635	45.39362	126.5887	45.39362	62.01097	45.39362
45.41409	36.75396	45.41409	59.98786	45.41409	109.3751	45.41409	60.73421	45.41409
45.43455	33.74891	45.43455	54.8451	45.43455	105.1687	45.43455	54.46337	45.43455
45.45501	-4.25047	45.45501	46.70805	45.45501	56.96945	45.45501	53.19846	45.45501
45.47547	77.75583	45.47547	46.57673	45.47547	56.77738	45.47547	13.93947	45.47547
45.49594	8.767807	45.49594	39.45113	45.49594	55.59248	45.49594	-10.3136	45.49594
45.5164	-13.2145	45.5164	80.33125	45.5164	19.41476	45.5164	27.43926	45.5164
45.53686	11.80879	45.53686	36.2171	45.53686	-4.75578	45.53686	1.198035	45.53686
45.55733	-17.1622	45.55733	9.108668	45.55733	10.08086	45.55733	22.96274	45.55733
45.57779	-14.1275	45.57779	-36.994	45.57779	11.92468	45.57779	24.73336	45.57779
45.59825	14.91283	45.59825	-8.6914	45.59825	-3.22433	45.59825	-0.49009	45.59825
45.61871	17.95886	45.61871	14.61696	45.61871	14.63385	45.61871	22.29239	45.61871
45.63918	-10.9894	45.63918	15.93105	45.63918	37.4992	45.63918	37.08078	45.63918
45.65964	57.06796	45.65964	52.25085	45.65964	-17.6283	45.65964	-16.1249	45.65964
45.6801	17.13102	45.6801	13.57638	45.6801	-21.7486	45.6801	49.67535	45.6801
45.70057	-4.80024	45.70057	-7.09237	45.70057	-8.45868	45.70057	15.48152	45.70057
45.72103	12.27417	45.72103	5.244607	45.72103	6.838383	45.72103	28.29361	45.72103
45.74149	38.35426	45.74149	-20.4127	45.74149	22.14262	45.74149	19.11162	45.74149
45.76195	71.44003	45.76195	19.93572	45.76195	-42.546	45.76195	50.93556	45.76195
45.78242	38.53147	45.78242	25.28986	45.78242	14.77264	45.78242	16.76542	45.78242
45.80288	39.62859	45.80288	5.649727	45.80288	16.09841	45.80288	13.6012	45.80288
45.82334	33.73138	45.82334	-2.98469	45.82334	-20.5686	45.82334	46.44291	45.82334
45.8438	-1.16015	45.8438	-35.6134	45.8438	14.7715	45.8438	40.29054	45.8438
45.86427	27.954	45.86427	13.76365	45.86427	26.11881	45.86427	62.1441	45.86427
45.88473	41.07383	45.88473	41.1464	45.88473	40.47329	45.88473	-9.99643	45.88473
45.90519	10.19932	45.90519	22.53488	45.90519	24.83496	45.90519	42.86898	45.90519
45.92566	15.3305	45.92566	31.92908	45.92566	5.203802	45.92566	-1.2597	45.92566
45.94612	12.46735	45.94612	32.329	45.94612	-16.4202	45.94612	23.61755	45.94612
45.96658	21.60988	45.96658	-11.2654	45.96658	-33.037	45.96658	-30.4993	45.96658
45.98704	49.75808	45.98704	10.146	45.98704	20.3534	45.98704	-21.6102	45.98704
46.00751	-45.088	46.00751	1.563086	46.00751	-6.24904	46.00751	23.00122	46.00751
46.02797	25.07152	46.02797	-8.01411	46.02797	25.15569	46.02797	12.61855	46.02797
46.04843	-20.7632	46.04843	3.414425	46.04843	25.56761	46.04843	-31.7582	46.04843
46.0689	16.40766	46.0689	-50.1513	46.0689	-16.0133	46.0689	9.870967	46.0689
46.08936	-47.4158	46.08936	16.28865	46.08936	-21.587	46.08936	35.50606	46.08936
46.10982	-7.2335	46.10982	-29.2656	46.10982	-39.0837	46.10982	16.14708	46.10982
46.13028	-13.0456	46.13028	10.18577	46.13028	40.42687	46.13028	7.794025	46.13028
46.15075	20.14806	46.15075	1.642914	46.15075	-4.05541	46.15075	20.44689	46.15075
46.17121	26.34735	46.17121	8.105779	46.17121	21.46949	46.17121	-6.89432	46.17121
46.19167	37.55231	46.19167	-14.4256	46.19167	-10.9984	46.19167	24.7704	46.19167
46.21213	20.76295	46.21213	-2.95133	46.21213	26.54082	46.21213	-4.55897	46.21213
46.2326	39.97927	46.2326	16.52871	46.2326	-2.91275	46.2326	26.11759	46.2326
46.25306	66.20126	46.25306	-5.98554	46.25306	8.640862	46.25306	12.80008	46.25306
46.27352	-19.5711	46.27352	-5.49406	46.27352	-6.79835	46.27352	-29.5115	46.27352
46.29399	-25.3377	46.29399	4.003136	46.29399	50.76962	46.29399	0.182813	46.29399
46.31445	13.71805	46.31445	-24.4939	46.31445	-7.65524	46.31445	61.88307	46.31445
46.33491	1.779493	46.33491	-3.79138	46.33491	40.92709	46.33491	4.589245	46.33491
46.35537	11.84661	46.35537	-2.0831	46.35537	9.516588	46.35537	23.30135	46.35537
46.37584	71.91941	46.37584	1.630901	46.37584	19.11327	46.37584	-24.9806	46.37584

46.3963	-4.00212	46.3963	23.35063	46.3963	-19.2829	46.3963	6.743318	46.3963
46.41676	-1.91797	46.41676	13.07608	46.41676	-1.67184	46.41676	-7.52681	46.41676
46.43723	39.17186	46.43723	31.80725	46.43723	-7.05363	46.43723	-7.79102	46.43723
46.45769	78.26736	46.45769	29.54414	46.45769	25.57177	46.45769	40.9507	46.45769
46.47815	50.36853	46.47815	5.286753	46.47815	-10.7957	46.47815	45.69834	46.47815
46.49861	45.47539	46.49861	48.03509	46.49861	54.84409	46.49861	33.45191	46.49861
46.51908	11.58792	46.51908	46.78915	46.51908	9.491014	46.51908	42.2114	46.51908
46.53954	7.706121	46.53954	49.54893	46.53954	46.14512	46.53954	8.976809	46.53954
46.56	82.83	46.56	12.31444	46.56	16.8064	46.56	60.74814	46.56
46.58046	45.95956	46.58046	23.08567	46.58046	33.47487	46.58046	72.52541	46.58046
46.60093	119.0948	46.60093	10.86261	46.60093	77.15051	46.60093	20.30858	46.60093
46.62139	52.23571	46.62139	58.64528	46.62139	64.83332	46.62139	51.09769	46.62139
46.64185	34.38229	46.64185	42.43368	46.64185	15.52332	46.64185	76.89272	46.64185
46.66232	96.53455	46.66232	35.2278	46.66232	62.22049	46.66232	101.6937	46.66232
46.68278	70.69249	46.68278	56.02763	46.68278	109.9248	46.68278	53.50055	46.68278
46.70324	100.8561	46.70324	73.83319	46.70324	71.63638	46.70324	34.31335	46.70324
46.7237	111.0254	46.7237	55.64447	46.7237	23.35508	46.7237	65.13207	46.7237
46.74417	59.20037	46.74417	19.46148	46.74417	100.081	46.74417	76.95672	46.74417
46.76463	83.38101	46.76463	58.28421	46.76463	29.81403	46.76463	47.78729	46.76463
46.78509	60.56733	46.78509	43.11266	46.78509	25.55428	46.78509	72.62378	46.78509
46.80555	52.75932	46.80555	74.94683	46.80555	34.3017	46.80555	45.4662	46.80555
46.82602	60.957	46.82602	40.78672	46.82602	92.0563	46.82602	67.31454	46.82602
46.84648	93.16035	46.84648	56.63234	46.84648	62.81808	46.84648	99.1688	46.84648
46.86694	53.36937	46.86694	60.48368	46.86694	108.587	46.86694	97.02898	46.86694
46.88741	108.5841	46.88741	62.34074	46.88741	62.36317	46.88741	33.8951	46.88741
46.90787	78.80444	46.90787	72.20352	46.90787	101.1465	46.90787	94.76713	46.90787
46.92833	125.0305	46.92833	86.07203	46.92833	78.93697	46.92833	140.6451	46.92833
46.94879	115.2622	46.94879	97.94626	46.94879	77.73463	46.94879	103.529	46.94879
46.96926	75.49963	46.96926	116.8262	46.96926	124.5395	46.96926	111.4188	46.96926
46.98972	184.7427	46.98972	160.7119	46.98972	135.3515	46.98972	157.3145	46.98972
47.01018	199.9915	47.01018	182.6033	47.01018	155.1707	47.01018	251.2162	47.01018
47.03065	246.2459	47.03065	214.5004	47.03065	203.9971	47.03065	284.1237	47.03065
47.05111	238.506	47.05111	197.4032	47.05111	220.8307	47.05111	293.0372	47.05111
47.07157	281.7718	47.07157	223.3118	47.07157	264.6714	47.07157	291.9566	47.07157
47.09203	294.0433	47.09203	223.2261	47.09203	392.5193	47.09203	399.882	47.09203
47.1125	263.3204	47.1125	278.1461	47.1125	484.3744	47.1125	340.8133	47.1125
47.13296	247.6032	47.13296	303.0718	47.13296	508.2367	47.13296	364.7505	47.13296
47.15342	260.8917	47.15342	301.0033	47.15342	533.1061	47.15342	369.6936	47.15342
47.17388	177.1859	47.17388	334.9404	47.17388	459.9828	47.17388	377.6426	47.17388
47.19435	195.4857	47.19435	256.8833	47.19435	420.8665	47.19435	317.5976	47.19435
47.21481	201.7912	47.21481	256.832	47.21481	412.7575	47.21481	330.5584	47.21481
47.23527	189.1024	47.23527	243.7863	47.23527	336.6557	47.23527	334.5253	47.23527
47.25574	91.4193	47.25574	203.7464	47.25574	335.561	47.25574	262.498	47.25574
47.2762	98.74184	47.2762	217.7121	47.2762	288.4736	47.2762	193.4767	47.2762
47.29666	71.07007	47.29666	166.6837	47.29666	266.3933	47.29666	180.4612	47.29666
47.31712	31.40397	47.31712	111.6609	47.31712	220.3201	47.31712	132.4517	47.31712
47.33759	60.74355	47.33759	40.64383	47.33759	125.2542	47.33759	77.44816	47.33759
47.35805	75.0888	47.35805	26.63251	47.35805	113.1954	47.35805	40.45051	47.35805
47.37851	22.43973	47.37851	80.62691	47.37851	62.14384	47.37851	30.45878	47.37851
47.39898	-10.2037	47.39898	59.62703	47.39898	67.09943	47.39898	41.47298	47.39898
47.41944	6.15861	47.41944	26.63287	47.41944	62.06219	47.41944	-13.5069	47.41944
47.4399	3.526567	47.4399	46.64443	47.4399	14.03214	47.4399	-11.4809	47.4399
47.46036	-25.0998	47.46036	13.66172	47.46036	25.00926	47.46036	-1.44889	47.46036
47.48083	-12.1938	47.48083	-7.31528	47.48083	12.99356	47.48083	-18.411	47.48083
47.50129	11.71779	47.50129	-12.2865	47.50129	13.98504	47.50129	-14.3672	47.50129
47.52175	15.63511	47.52175	-8.25209	47.52175	28.9837	47.52175	8.68255	47.52175
47.54221	2.558093	47.54221	6.788082	47.54221	4.989533	47.54221	-11.2618	47.54221
47.56268	33.48676	47.56268	-24.166	47.56268	-4.99745	47.56268	-13.2002	47.56268

47.58314	28.4211	47.58314	3.885599	47.58314	-21.9773	47.58314	-14.1327	47.58314
47.6036	-37.6389	47.6036	-14.0571	47.6036	-38.5358	47.6036	0.940734	47.6036
47.62407	5.306804	47.62407	-49.994	47.62407	24.91277	47.62407	-47.9799	47.62407
47.64453	20.25817	47.64453	14.26679	47.64453	-12.6315	47.64453	18.10537	47.64453
47.66499	40.21522	47.66499	9.533292	47.66499	32.8315	47.66499	5.196568	47.66499
47.68545	22.17794	47.68545	-2.19448	47.68545	-0.69836	47.68545	-13.7063	47.68545
47.70592	49.14634	47.70592	11.08347	47.70592	-2.22105	47.70592	0.396741	47.70592
47.72638	12.12041	47.72638	-22.6329	47.72638	-13.7366	47.72638	-26.4943	47.72638
47.74684	47.10016	47.74684	24.65653	47.74684	9.755117	47.74684	-0.11837	47.74684
47.76731	58.08558	47.76731	5.951651	47.76731	-15.746	47.76731	15.26346	47.76731
47.78777	30.07668	47.78777	15.25249	47.78777	-10.24	47.78777	-9.34878	47.78777
47.80823	19.07346	47.80823	11.55905	47.80823	3.273199	47.80823	70.04491	47.80823
47.82869	-4.92409	47.82869	8.871333	47.82869	-17.2064	47.82869	20.44451	47.82869
47.84916	51.08405	47.84916	-16.8107	47.84916	5.321145	47.84916	25.85004	47.84916
47.86962	58.09785	47.86962	38.51307	47.86962	53.85589	47.86962	51.2615	47.86962
47.89008	33.11733	47.89008	32.84252	47.89008	31.3978	47.89008	35.67888	47.89008
47.91054	6.142492	47.91054	15.17769	47.91054	11.9469	47.91054	48.10217	47.91054
47.93101	47.17333	47.93101	38.51859	47.93101	41.50317	47.93101	106.5314	47.93101
47.95147	71.20984	47.95147	42.8652	47.95147	77.06663	47.95147	36.96655	47.95147
47.97193	12.25203	47.97193	18.21754	47.97193	112.6373	47.97193	27.40762	47.97193
47.9924	94.29989	47.9924	20.5756	47.9924	60.21507	47.9924	58.85461	47.9924
48.01286	87.35342	48.01286	56.93939	48.01286	65.80006	48.01286	10.30753	48.01286
48.03332	106.4126	48.03332	38.30889	48.03332	52.39222	48.03332	82.76637	48.03332
48.05378	96.47753	48.05378	47.68412	48.05378	58.99156	48.05378	52.23113	48.05378
48.07425	83.5481	48.07425	65.06507	48.07425	46.59808	48.07425	58.70182	48.07425
48.09471	71.62434	48.09471	59.45174	48.09471	76.21178	48.09471	52.17843	48.09471
48.11517	134.7063	48.11517	-3.15586	48.11517	51.83266	48.11517	87.66097	48.11517
48.13564	120.7939	48.13564	36.24226	48.13564	96.46072	48.13564	62.14943	48.13564
48.1561	115.8871	48.1561	55.6461	48.1561	111.096	48.1561	44.64381	48.1561
48.17656	77.98608	48.17656	127.0557	48.17656	126.7384	48.17656	140.1441	48.17656
48.19702	154.0907	48.19702	65.47095	48.19702	149.388	48.19702	122.6503	48.19702
48.21749	244.201	48.21749	122.892	48.21749	119.0447	48.21749	139.1625	48.21749
48.23795	260.317	48.23795	121.3187	48.23795	154.7087	48.23795	243.6806	48.23795
48.25841	310.4386	48.25841	108.7511	48.25841	169.3798	48.25841	202.2046	48.25841
48.27887	356.566	48.27887	158.1893	48.27887	167.0581	48.27887	270.7345	48.27887
48.29934	420.699	48.29934	205.6332	48.29934	233.7436	48.29934	338.2703	48.29934
48.3198	465.8376	48.3198	280.0828	48.3198	223.4362	48.3198	356.8121	48.3198
48.34026	502.982	48.34026	342.5382	48.34026	323.1361	48.34026	604.3598	48.34026
48.36073	508.1321	48.36073	373.9992	48.36073	448.8431	48.36073	721.9134	48.36073
48.38119	663.2878	48.38119	428.466	48.38119	471.5573	48.38119	885.473	48.38119
48.40165	620.4492	48.40165	542.9385	48.40165	501.2787	48.40165	1175.038	48.40165
48.42211	686.6162	48.42211	605.4167	48.42211	542.0072	48.42211	1294.61	48.42211
48.44258	688.7889	48.44258	702.9007	48.44258	550.7429	48.44258	1381.187	48.44258
48.46304	661.9673	48.46304	769.3904	48.46304	521.4858	48.46304	1342.77	48.46304
48.4835	757.1514	48.4835	755.8858	48.4835	617.236	48.4835	1217.359	48.4835
48.50397	784.3412	48.50397	787.3869	48.50397	665.9932	48.50397	1398.955	48.50397
48.52443	791.5367	48.52443	784.8937	48.52443	750.7576	48.52443	1409.556	48.52443
48.54489	776.7378	48.54489	915.4063	48.54489	865.5292	48.54489	1594.162	48.54489
48.56535	639.9446	48.56535	880.9246	48.56535	907.308	48.56535	1380.775	48.56535
48.58582	650.157	48.58582	1009.449	48.58582	1005.094	48.58582	1286.394	48.58582
48.60628	478.3752	48.60628	986.9783	48.60628	1022.887	48.60628	1108.019	48.60628
48.62674	438.599	48.62674	799.5138	48.62674	954.6875	48.62674	858.6495	48.62674
48.6472	439.8285	48.6472	770.0549	48.6472	813.4951	48.6472	752.286	48.6472
48.66767	405.0637	48.66767	677.6019	48.66767	714.3097	48.66767	630.9285	48.66767
48.68813	336.3045	48.68813	588.1545	48.68813	599.1316	48.68813	586.5768	48.68813
48.70859	264.5511	48.70859	508.7128	48.70859	536.9606	48.70859	419.2312	48.70859
48.72906	224.8033	48.72906	508.2769	48.72906	521.7969	48.72906	341.8914	48.72906
48.74952	242.0612	48.74952	375.8467	48.74952	499.6403	48.74952	251.5576	48.74952

48.76998	129.3247	48.76998	360.4222	48.76998	363.4908	48.76998	202.2297	48.76998
48.79044	118.594	48.79044	210.0034	48.79044	285.3486	48.79044	169.9077	48.79044
48.81091	135.8689	48.81091	164.5904	48.81091	270.2135	48.81091	49.59161	48.81091
48.83137	92.14945	48.83137	140.183	48.83137	165.0856	48.83137	28.28147	48.83137
48.85183	148.4357	48.85183	65.78143	48.85183	134.9649	48.85183	95.97724	48.85183
48.8723	106.7276	48.8723	70.38556	48.8723	20.85141	48.8723	45.67895	48.8723
48.89276	82.02526	48.89276	46.9954	48.89276	74.74506	48.89276	19.38657	48.89276
48.91322	102.3286	48.91322	56.61097	48.91322	81.64589	48.91322	3.100122	48.91322
48.93368	129.6375	48.93368	44.23225	48.93368	30.55389	48.93368	0.819595	48.93368
48.95415	121.9522	48.95415	45.85926	48.95415	106.4691	48.95415	50.54499	48.95415
48.97461	94.27248	48.97461	29.492	48.97461	42.39144	48.97461	51.27631	48.97461
48.99507	160.5985	48.99507	27.13045	48.99507	43.32098	48.99507	69.01356	48.99507
49.01553	141.9301	49.01553	25.77463	49.01553	65.2577	49.01553	73.75672	49.01553
49.036	101.2675	49.036	63.42453	49.036	56.20159	49.036	69.50581	49.036
49.05646	149.6105	49.05646	108.0801	49.05646	68.15266	49.05646	114.2608	49.05646
49.07692	184.9592	49.07692	110.7415	49.07692	74.11092	49.07692	90.02176	49.07692
49.09739	153.3136	49.09739	78.40856	49.09739	106.0763	49.09739	111.7886	49.09739
49.11785	259.6736	49.11785	122.0813	49.11785	126.049	49.11785	161.5614	49.11785
49.13831	239.0394	49.13831	135.7599	49.13831	98.02875	49.13831	255.3401	49.13831
49.15877	279.4108	49.15877	148.4441	49.15877	137.0157	49.15877	225.1247	49.15877
49.17924	264.7878	49.17924	135.134	49.17924	201.0099	49.17924	267.9153	49.17924
49.1997	285.1706	49.1997	153.8297	49.1997	222.0112	49.1997	309.7118	49.1997
49.22016	190.5591	49.22016	198.5311	49.22016	263.0197	49.22016	325.5142	49.22016
49.24063	214.9532	49.24063	232.2383	49.24063	215.0354	49.24063	207.3225	49.24063
49.26109	191.3529	49.26109	195.9511	49.26109	197.0582	49.26109	215.1367	49.26109
49.28155	215.7584	49.28155	231.6697	49.28155	155.0882	49.28155	224.9569	49.28155
49.30201	239.1695	49.30201	185.394	49.30201	158.1255	49.30201	137.783	49.30201
49.32248	135.5863	49.32248	147.124	49.32248	195.1698	49.32248	198.615	49.32248
49.34294	174.0088	49.34294	163.8597	49.34294	160.2214	49.34294	159.453	49.34294
49.3634	153.437	49.3634	117.6012	49.3634	137.2802	49.3634	113.2968	49.3634
49.38386	150.8709	49.38386	90.34833	49.38386	123.3461	49.38386	115.1466	49.38386
49.40433	118.3104	49.40433	160.1012	49.40433	131.4192	49.40433	76.00234	49.40433
49.42479	139.7556	49.42479	106.8598	49.42479	107.4995	49.42479	62.86398	49.42479
49.44525	164.2064	49.44525	87.6242	49.44525	50.58693	49.44525	100.7315	49.44525
49.46572	185.663	49.46572	66.39426	49.46572	63.68157	49.46572	66.60502	49.46572
49.48618	176.1252	49.48618	109.1701	49.48618	16.78338	49.48618	76.48442	49.48618
49.50664	168.5931	49.50664	131.9516	49.50664	72.89238	49.50664	103.3698	49.50664
49.5271	203.0667	49.5271	90.7388	49.5271	47.00855	49.5271	135.261	49.5271
49.54757	191.5459	49.54757	100.5318	49.54757	122.1319	49.54757	171.1582	49.54757
49.56803	261.0309	49.56803	146.3304	49.56803	132.2624	49.56803	227.0613	49.56803
49.58849	348.5215	49.58849	176.1348	49.58849	124.4001	49.58849	253.9703	49.58849
49.60896	363.0177	49.60896	175.945	49.60896	173.545	49.60896	298.8853	49.60896
49.62942	354.5197	49.62942	243.7608	49.62942	195.6971	49.62942	507.8061	49.62942
49.64988	337.0273	49.64988	219.5824	49.64988	302.8563	49.64988	452.7329	49.64988
49.67034	329.5406	49.67034	253.4097	49.67034	379.0227	49.67034	470.6656	49.67034
49.69081	276.0596	49.69081	284.2427	49.69081	361.1964	49.69081	437.6043	49.69081
49.71127	292.5843	49.71127	296.0814	49.71127	440.3771	49.71127	441.5488	49.71127
49.73173	228.1146	49.73173	269.9259	49.73173	351.5651	49.73173	354.4993	49.73173
49.75219	213.6506	49.75219	251.7761	49.75219	350.7602	49.75219	320.4557	49.75219
49.77266	149.1923	49.77266	205.632	49.77266	300.9625	49.77266	247.4181	49.77266
49.79312	186.7397	49.79312	138.4936	49.79312	234.172	49.79312	235.3863	49.79312
49.81358	138.2927	49.81358	226.3609	49.81358	212.3887	49.81358	240.3605	49.81358
49.83405	121.8514	49.83405	142.234	49.83405	239.6125	49.83405	198.3406	49.83405
49.85451	103.4158	49.85451	106.1128	49.85451	183.8435	49.85451	120.3266	49.85451
49.87497	74.98591	49.87497	75.99731	49.87497	136.0817	49.87497	77.31859	49.87497
49.89543	11.56165	49.89543	45.88754	49.89543	25.32712	49.89543	77.31647	49.89543
49.9159	68.14307	49.9159	38.7835	49.9159	48.57968	49.9159	9.320264	49.9159
49.93636	46.73017	49.93636	5.685179	49.93636	52.83942	49.93636	11.32999	49.93636

49.95682	50.32295	49.95682	18.59258	49.95682	46.10633	49.95682	0.34563	49.95682
49.97729	104.9214	49.97729	-39.4943	49.97729	-10.6196	49.97729	-9.6328	49.97729
49.99775	-24.4745	49.99775	-22	49.99775	-22.3383	49.99775	-22.6053	49.99775

pH=9		pH=12	2 Theta degree	Intensity a.u. Experiment	
-52.7447	5.0001	-51.2933	2		8.5
-63.9976	5.020563	60.27568	2.020462		8.5
-3.13145	5.041026	-11.1501	2.040925		8.5
-24.2594	5.061488	-48.5705	2.061388		8.5
-30.3814	5.081951	2.014386	2.081851		0
-51.4976	5.102414	-23.3954	2.102313		1
18.24357	5.122877	14.20003	2.122776		0
-9.00938	5.14334	90.80081	2.143239		0
17.74358	5.163802	-45.5931	2.163702		0
-69.4976	5.184265	-21.9817	2.184165		0
-18.9296	5.204728	14.63497	2.204627		0
105.6443	5.225191	-20.743	2.22509		5.5
23.2241	5.245653	-37.1157	2.245553		11.25
-6.1902	5.266116	61.51685	2.266016		14
15.4014	5.286579	28.15476	2.286478		15.25
63.9989	5.307042	35.79796	2.306941		18.5
-19.3977	5.327505	4.446465	2.327404		18.25
137.2116	5.347967	57.10028	2.347867		11.5
-21.1732	5.36843	-28.2406	2.36833		11.5
51.44796	5.388893	-7.5762	2.388792		13.5
-86.925	5.409356	69.09352	2.409255		17.5
9.707906	5.429818	39.76855	2.429718		23
144.3467	5.450281	-71.5511	2.450181		29
59.99147	5.470744	108.1345	2.470643		30
21.64211	5.491207	-36.1746	2.491106		26
34.29865	5.51167	-9.47833	2.511569		23.5
-53.0389	5.532132	17.22321	2.532032		22
108.6294	5.552595	-50.07	2.552495		17
183.3037	5.573058	-45.4584	2.572957		19.25
-19.0161	5.593521	-25.8415	2.59342		18.5
98.66992	5.613983	28.78066	2.613883		13.75
9.361888	5.634446	-62.5919	2.634346		5.5
125.0598	5.654909	81.04092	2.654808		12
41.76353	5.675372	89.679	2.675271		14.75
169.4732	5.695835	41.32239	2.695734		11.5
-14.8112	5.716297	-30.0289	2.716197		15.5
83.91028	5.73676	44.62507	2.73666		22
43.63767	5.757223	-81.7156	2.757122		22
231.371	5.777686	14.94896	2.777585		19.25
141.1102	5.798148	97.61887	2.798048		13.75
72.85526	5.818611	14.29407	2.818511		8
127.6063	5.839074	119.9746	2.838973		0
58.36317	5.859537	107.6604	2.859436		1
67.12598	5.88	-12.6485	2.879899		0.5
31.8947	5.900462	-0.95208	2.900362		0
57.66932	5.920925	53.74964	2.920825		0
145.4498	5.941388	8.456662	2.941287		0
-7.76373	5.961851	233.169	2.96175		0
204.0286	5.982313	133.8866	2.982213		0
261.8268	6.002776	106.6096	3.002676		0
230.631	6.023239	161.3378	3.023139		0

296.441	6.043702	154.0713	3.043602	0
309.257	6.064165	181.8102	3.064064	2.75
451.0788	6.084627	175.5543	3.084527	8.5
379.9066	6.10509	337.3038	3.10499	12.5
596.7402	6.125553	388.0585	3.125453	13
829.5798	6.146016	429.8186	3.145916	15.5
1032.425	6.166478	445.5839	3.166378	15.5
1329.277	6.186941	420.3546	3.186841	13.25
1985.134	6.207404	473.1306	3.207304	9
2478.997	6.227867	565.9118	3.227767	5.75
2198.866	6.24833	801.6984	3.248229	2.25
1335.741	6.268792	1129.49	3.268692	1
905.622	6.289255	1470.287	3.289155	2
812.5089	6.309718	1590.09	3.309618	0
867.4016	6.330181	1351.898	3.330081	0
1274.3	6.350643	899.7108	3.350543	3
1487.205	6.371106	603.5292	3.371006	6.75
1814.115	6.391569	491.3529	3.391469	9
1804.031	6.412032	399.1819	3.411932	10.25
1541.954	6.432495	352.0162	3.432394	10.25
1257.882	6.452957	324.8558	3.452857	8.5
809.8157	6.47342	308.7007	3.47332	6.75
420.7556	6.493883	292.5509	3.493783	4.25
282.7015	6.514346	229.4064	3.514246	0
256.6532	6.534808	128.2672	3.534708	0
189.6109	6.555271	236.1333	3.555171	1
141.5744	6.575734	227.0047	3.575634	2.25
157.5439	6.596197	142.8815	3.596097	3.25
59.5192	6.61666	85.76349	3.616559	3.5
196.5005	6.637122	103.6508	3.637022	3
94.48762	6.657585	72.54345	3.657485	2.75
138.4807	6.678048	102.4414	3.677948	3
96.47965	6.698511	126.3446	3.698411	2.25
94.48452	6.718973	108.2532	3.718873	1
147.4953	6.739436	118.167	3.739336	0
139.512	6.759899	107.0862	3.759799	0
153.5346	6.780362	78.01061	3.780262	1.5
161.563	6.800825	107.9404	3.800724	2.5
108.5974	6.821287	0.875423	3.821187	3
130.6377	6.84175	136.8158	3.84165	2.75
81.68393	6.862213	49.76145	3.862113	3.5
109.736	6.882676	62.71241	3.882576	3.75
98.79404	6.903138	48.66869	3.903038	2.5
122.8579	6.923601	45.63026	3.923501	2.25
164.9278	6.944064	81.59714	3.943964	1.5
146.0035	6.964527	137.5693	3.964427	0
205.0851	6.98499	110.5468	3.984889	2.75
220.1726	7.005452	150.5296	4.005352	5
219.2661	7.025915	39.51767	4.025815	5.75
223.3654	7.046378	166.5111	4.046278	5.75
346.4706	7.066841	173.5098	4.066741	7.25
420.5818	7.087303	268.5138	4.087203	7.25
451.6988	7.107766	174.5231	4.107666	5.75
453.8217	7.128229	207.5377	4.128129	5.75
620.9506	7.148692	213.5576	4.148592	3.75
753.0854	7.169155	418.5828	4.169055	0
982.226	7.189617	512.6133	4.189517	2.75
840.3726	7.21008	624.6491	4.20998	3.5

845.5251	7.230543	877.6902	4.230443	3.5
711.6834	7.251006	834.7366	4.250906	2.25
309.8477	7.271468	887.7884	4.271368	4.75
142.0179	7.291931	683.8454	4.291831	5.5
128.194	7.312394	564.9077	4.312294	5.25
101.376	7.332857	543.9754	4.332757	3
112.5639	7.35332	700.0483	4.35322	3
36.75765	7.373782	670.1266	4.373682	4.75
54.95735	7.394245	340.2101	4.394145	8.5
44.16295	7.414708	211.299	4.414608	9.75
90.37446	7.435171	200.3931	4.435071	9.5
114.5919	7.455633	141.4926	4.455533	7
67.81519	7.476096	91.59731	4.475996	7.75
47.0444	7.496559	102.7074	4.496459	7.5
-32.7205	7.517022	99.82274	4.516922	5.75
-22.4794	7.537485	60.94341	4.537385	0.75
133.7675	7.557947	121.0694	4.557847	3.25
51.02032	7.57841	25.20065	4.57831	4.5
50.27905	7.598873	90.33723	4.598773	4.75
-15.4563	7.619336	140.4791	4.619236	7.75
78.81424	7.639798	136.6263	4.639698	10
14.09069	7.660261	53.77877	4.660161	9.5
20.37305	7.680724	75.93656	4.680624	9.5
109.6613	7.701187	43.09965	4.701087	11.5
-2.04454	7.72165	61.26805	4.72155	11.25
53.25553	7.742112	85.44174	4.742012	8.75
55.5615	7.762575	-15.3793	4.762475	9
25.87337	7.783038	90.80505	4.782938	6.75
61.19115	7.803501	8.994657	4.803401	7.25
69.51483	7.823963	60.18957	4.823863	9.25
110.8444	7.844426	9.38978	4.844326	8.5
65.1799	7.864889	28.5953	4.864789	5.5
45.52129	7.885352	31.80612	4.885252	5.25
26.86859	7.905815	85.02224	4.905715	5.5
6.22179	7.926277	71.24367	4.926177	5
116.5809	7.94674	-57.5296	4.94664	4.5
58.9459	7.967203	14.70243	4.967103	3.75
68.31681	7.987666	53.93976	4.987566	5
128.6936	8.008128	66.1824	5.008028	6
7.076348	8.028591	-39.5697	5.028491	6
2.464972	8.049054	71.68359	5.048954	3.5
104.8595	8.069517	78.94214	5.069417	1.75
-15.7401	8.08998	-18.794	5.08988	5
-21.3337	8.110442	24.47514	5.110342	8.25
73.07851	8.130905	75.7496	5.130805	8.75
-66.5034	8.151368	11.02936	5.151268	11.75
46.92069	8.171831	25.31442	5.171731	14
4.350643	8.192293	12.60479	5.192193	16
71.7865	8.212756	-32.0995	5.212656	16
43.22826	8.233219	29.20143	5.233119	14.25
23.67592	8.253682	-7.49229	5.253582	9.25
-33.8705	8.274145	74.81928	5.274045	4.75
22.58895	8.294607	41.13617	5.294507	5.75
126.0543	8.31507	44.45835	5.31497	6
18.5256	8.335533	80.78584	5.335433	5
-64.9972	8.355996	-10.8814	5.355896	7
-17.9669	8.376458	70.45673	5.376358	8.25
-4.93069	8.396921	69.80013	5.396821	7.25

21.11143	8.417384	-2.85117	5.417284	7.5
42.15945	8.437847	-54.4972	5.437747	5.25
0.213378	8.45831	2.622642	5.458209	3.5
17.27321	8.478772	18.74776	5.478672	0.5
27.33894	8.499235	33.87818	5.499135	2.75
-1.58942	8.519698	-17.9861	5.519598	3.5
141.4881	8.540161	-42.8451	5.540061	6.25
-49.4284	8.560623	52.30125	5.560523	6.75
81.66091	8.581086	24.45289	5.580986	6.75
-75.2438	8.601549	33.60982	5.601449	5.75
74.85732	8.622012	-55.2279	5.621912	2.75
79.96439	8.642475	38.9396	5.642374	4
40.07735	8.662937	57.11244	5.662837	5
-15.8038	8.6834	-2.70941	5.6833	4
37.32099	8.703863	58.47404	5.703763	8
-33.5483	8.724326	20.66279	5.724226	7.25
76.58825	8.744788	-34.1432	5.744688	13
48.73073	8.765251	-13.9438	5.765151	15.75
105.8791	8.785714	22.26087	5.785614	21.25
20.03341	8.806177	36.47084	5.806077	26.5
-43.8064	8.82664	38.6861	5.826539	30
-39.4541	8.847102	0.906677	5.847002	36.5
-21.0958	8.867565	29.13255	5.867465	41.75
93.2683	8.888028	13.36373	5.887928	43.5
37.63833	8.908491	53.60021	5.908391	45.25
-72.9857	8.928953	87.842	5.928853	45.75
-15.6039	8.949416	-8.91092	5.949316	51.25
55.78387	8.969879	17.34148	5.969779	55.25
29.17752	8.990342	71.59917	5.990242	65.25
105.5771	9.010805	-31.1378	6.010705	76.25
44.98254	9.031267	64.13046	6.031167	93.5
26.3939	9.05173	-59.5959	6.05163	112.25
3.81117	9.072193	-31.317	6.072093	139
28.23434	9.092656	-11.0328	6.092556	165
-2.33658	9.113118	39.2567	6.113018	192.75
-24.9016	9.133581	2.551512	6.133481	229.75
120.5393	9.154044	28.85163	6.153944	270.25
18.98607	9.174507	34.15705	6.174407	313
-19.5612	9.19497	51.46778	6.19487	374.75
30.89736	9.215432	3.783806	6.215332	452
78.36186	9.235895	-4.89486	6.235795	549.75
126.8323	9.256358	19.43177	6.256258	667.5
70.30856	9.276821	57.76371	6.276721	807
27.79078	9.297283	-19.8991	6.297183	947.75
14.27889	9.317746	78.4435	6.317646	1112.25
23.77291	9.338209	43.79134	6.338109	1317.25
-25.7272	9.358672	13.14449	6.358572	1575
-30.2213	9.379135	-61.4971	6.379035	1923.5
49.29039	9.399597	-12.2815	6.399497	2355.25
102.808	9.42006	-14.0607	6.41996	2849
55.33156	9.440523	64.16545	6.440423	3311.25
57.861	9.460986	-53.6031	6.460886	3638.75
41.39635	9.481448	41.63364	6.481348	3716.5
30.9376	9.501911	70.87569	6.501811	3510.25
61.48475	9.522374	115.123	6.522274	3078.75
12.03781	9.542837	31.37569	6.542737	2487
78.59677	9.5633	14.63365	6.5632	1846.5
-23.8384	9.583762	-44.1031	6.583662	1272

19.7324	9.604225	14.16547	6.604125	824.75
22.30908	9.624688	-5.56066	6.624588	552.75
51.89165	9.645151	10.71851	6.645051	404.75
34.48013	9.665613	-43.997	6.665513	334.25
-63.9255	9.686076	-10.4932	6.685976	278.25
86.67481	9.706539	52.01587	6.706439	238
48.281	9.727002	95.53027	6.726902	206.75
58.89309	9.747465	20.04998	6.747365	180.75
58.51109	9.767927	43.57499	6.767827	162.75
34.135	9.78839	64.1053	6.78829	145.5
30.76481	9.808853	52.64091	6.808753	131.25
51.40052	9.829316	43.18183	6.829216	115.25
-12.9579	9.849778	66.72805	6.849678	103
-13.3103	9.870241	-2.72043	6.870141	95.5
11.34308	9.890704	117.8364	6.890604	88.25
147.0024	9.911167	149.3985	6.911067	82.5
60.66763	9.93163	147.966	6.93153	75.5
18.33877	9.952092	55.5387	6.951992	73
90.01581	9.972555	85.11674	6.972455	73.5
136.6987	9.993018	64.70008	6.992918	71.75
122.3876	10.01348	136.2887	7.013381	72
76.08234	10.03394	15.88267	7.033843	71
133.783	10.05441	72.48193	7.054306	81
163.4896	10.07487	42.08648	7.074769	87.5
78.20202	10.09533	61.69634	7.095232	97.25
130.9204	10.1158	98.3115	7.115695	110
158.6447	10.13626	111.932	7.136157	133
207.3748	10.15672	86.55773	7.15662	153.25
157.1109	10.17718	96.1888	7.177083	175
171.8529	10.19765	155.8252	7.197546	192
332.6008	10.21811	94.46685	7.218008	211.75
434.3546	10.23857	160.1138	7.238471	238
476.1143	10.25903	124.7661	7.258934	274.5
499.8799	10.2795	157.4237	7.279397	316.5
457.6514	10.29996	231.0866	7.29986	382
295.4288	10.32042	304.7548	7.320322	486
200.2121	10.34089	256.4283	7.340785	625.5
148.0013	10.36135	296.1071	7.361248	823.25
100.7964	10.38181	223.7912	7.381711	1091.25
96.59742	10.40227	185.4806	7.402173	1394.75
141.4043	10.42274	223.1753	7.422636	1668
163.2172	10.4432	113.8753	7.443099	1822.25
152.0359	10.46366	96.58061	7.463562	1807.5
138.8605	10.48412	37.29123	7.484025	1632.5
124.6911	10.50459	122.0071	7.504487	1339
70.52752	10.52505	54.72837	7.52495	979
60.36987	10.54551	66.4549	7.545413	612.5
75.21812	10.56598	89.18672	7.565876	325
142.0723	10.58644	14.92386	7.586338	155.25
104.9323	10.6069	42.66629	7.606801	92.5
101.7983	10.62736	83.41403	7.627264	86.5
117.6702	10.64783	98.16708	7.647727	85.25
123.5479	10.66829	70.92542	7.66819	79.5
79.43161	10.68875	61.68906	7.688652	65
142.3212	10.70922	74.45802	7.709115	55.75
85.21667	10.72968	57.23227	7.729578	45.75
75.11806	10.75014	105.0118	7.750041	35.5
136.0253	10.7706	75.79669	7.770503	28.75

157.9385	10.79107	79.58685	7.790966	25.75
99.85764	10.81153	129.3823	7.811429	22.5
53.78264	10.83199	130.1831	7.831892	19.75
186.7135	10.85245	67.98917	7.852355	17.75
67.65035	10.87292	65.80054	7.872817	22.5
128.5931	10.89338	152.6172	7.89328	19.25
130.5417	10.91384	120.4392	7.913743	22.5
65.4962	10.93431	145.2665	7.934206	22.25
150.4566	10.95477	99.09908	7.954668	21.75
152.423	10.97523	142.937	7.975131	22.75
99.39519	10.99569	132.7802	7.995594	20.75
77.37333	11.01616	84.62866	8.016057	18
203.3574	11.03662	158.4825	8.03652	12.5
111.3473	11.05708	166.3416	8.056982	12.25
94.34316	11.07755	84.20597	8.077445	11.5
76.34491	11.09801	143.0757	8.097908	12.5
78.35257	11.11847	189.9507	8.118371	12.75
93.36613	11.13893	147.831	8.138833	13.25
184.3856	11.1594	115.7166	8.159296	13.75
135.411	11.17986	176.6076	8.179759	11.5
156.4422	11.20032	55.50378	8.200222	8.5
132.4794	11.22078	46.40531	8.220685	7.5
103.5225	11.24125	81.31214	8.241147	6
161.5715	11.26171	108.2243	8.26161	7.25
202.6264	11.28217	130.1417	8.282073	3.75
194.6871	11.30264	165.0645	8.302536	6.25
169.7538	11.3231	116.9925	8.322998	3.25
136.8264	11.34356	124.9259	8.343461	6
161.9049	11.36402	107.8645	8.363924	7.25
125.9893	11.38449	266.8085	8.384387	6.75
248.0797	11.40495	227.7577	8.40485	6.75
185.1759	11.42541	181.7123	8.425312	8
187.278	11.44588	194.6721	8.445775	7.5
132.386	11.46634	140.6373	8.466238	6
102.4999	11.4868	104.6078	8.486701	3
223.6198	11.50726	187.5835	8.507163	1.75
219.7455	11.52773	248.5646	8.527626	0
138.8771	11.54819	136.551	8.548089	1.5
186.0147	11.56865	216.5427	8.568552	4
172.1581	11.58911	119.5397	8.589015	5.25
206.3075	11.60958	192.542	8.609477	5.75
125.4627	11.63004	145.5495	8.62994	4.75
271.6239	11.6505	143.5624	8.650403	5.75
179.7909	11.67097	191.5806	8.670866	4.5
199.9639	11.69143	179.6041	8.691328	4.25
181.1428	11.71189	220.633	8.711791	2.75
224.3275	11.73235	265.6671	8.732254	1.75
193.5182	11.75282	147.7065	8.752717	4.25
189.7148	11.77328	140.7512	8.77318	5
174.9173	11.79374	111.8012	8.793642	5.25
196.1257	11.81421	193.8565	8.814105	5.75
220.34	11.83467	198.9171	8.834568	5.25
271.5602	11.85513	222.9831	8.855031	5.75
256.7863	11.87559	221.0543	8.875493	4
226.0183	11.89606	232.1308	8.895956	4
294.2562	11.91652	258.2126	8.916419	2.5
251.5	11.93698	137.2998	8.936882	2
360.7497	11.95744	174.3922	8.957345	0.75

403.0053	11.97791	186.49	8.977807	0.5
377.2668	11.99837	197.593	8.99827	0.5
428.5343	12.01883	174.7014	9.018733	0.25
431.8076	12.0393	182.815	9.039196	1.25
495.0868	12.05976	355.934	9.059658	1.75
554.3719	12.08022	299.0583	9.080121	1.5
448.663	12.10068	384.1878	9.100584	2.25
391.96	12.12115	406.3227	9.121047	1
419.2628	12.14161	536.4628	9.14151	2.5
340.5716	12.16207	508.6083	9.161972	3.5
247.8862	12.18254	471.7591	9.182435	4.25
269.2068	12.203	434.9152	9.202898	5
311.5332	12.22346	391.0766	9.223361	5
219.8656	12.24392	329.2433	9.243823	5.75
236.2039	12.26439	301.4152	9.264286	5.5
255.5481	12.28485	224.5925	9.284749	4.5
253.8981	12.30531	249.7751	9.305212	3.25
243.2541	12.32577	239.963	9.325675	1
190.616	12.34624	308.1562	9.346137	1.5
148.9838	12.3667	253.3547	9.3666	0
212.3575	12.38716	268.5585	9.387063	0
307.7371	12.40763	181.7676	9.407526	0
220.1226	12.42809	197.9821	9.427988	1.5
256.514	12.44855	281.2018	9.448451	2.5
311.9113	12.46901	189.4268	9.468914	5.25
382.3145	12.48948	216.6571	9.489377	7.5
408.7237	12.50994	185.8927	9.50984	10
433.1387	12.5304	215.1337	9.530302	10
418.5596	12.55087	233.3799	9.550765	10
316.9865	12.57133	189.6314	9.571228	10.25
299.4192	12.59179	178.8883	9.591691	9
335.8578	12.61225	338.1504	9.612153	7.5
358.3024	12.63272	295.4178	9.632616	7
425.7528	12.65318	203.6906	9.653079	6
360.2092	12.67364	175.9687	9.673542	7
260.6714	12.6941	301.252	9.694005	8
210.1396	12.71457	299.5407	9.714467	7.75
238.6136	12.73503	265.8346	9.73493	7.75
164.0936	12.75549	211.1339	9.755393	8.5
186.5795	12.77596	191.4385	9.775856	8.75
190.0712	12.79642	116.7483	9.796318	10
197.5689	12.81688	204.0635	9.816781	10
188.0725	12.83734	111.384	9.837244	9.75
222.582	12.85781	165.7097	9.857707	9
204.0974	12.87827	169.0408	9.87817	7.75
222.6187	12.89873	191.3772	9.898632	5.5
234.1459	12.9192	162.7189	9.919095	4.25
109.679	12.93966	185.0659	9.939558	5.5
119.218	12.96012	181.4182	9.960021	4.75
170.7629	12.98058	183.7758	9.980483	3.25
177.3137	13.00105	181.1387	10.00095	7
140.8704	13.02151	230.5069	10.02141	8.5
123.433	13.04197	105.8804	10.04187	11.75
218.0016	13.06243	174.2592	10.06233	13.75
172.576	13.0829	171.6433	10.0828	14
157.1563	13.10336	104.0327	10.10326	16.5
133.7426	13.12382	71.42746	10.12372	20.75
195.3347	13.14429	172.8275	10.14419	22.5

144.9327	13.16475	93.2328	10.16465	23
151.5367	13.18521	112.6434	10.18511	21.5
114.1465	13.20567	107.0594	10.20557	27.5
153.7623	13.22614	47.4806	10.22604	32
136.384	13.2466	134.9071	10.2465	38
92.01152	13.26706	157.339	10.26696	44.25
116.645	13.28753	121.7761	10.28743	53.25
145.2844	13.30799	88.21858	10.30789	69.75
139.9296	13.32845	119.6663	10.32835	84.25
134.5808	13.34891	117.1194	10.34881	107.75
158.2379	13.36938	121.5777	10.36928	140.5
141.9009	13.38984	73.04139	10.38974	177
129.5698	13.4103	93.51035	10.4102	240.25
101.2446	13.43076	63.98462	10.43066	333.25
124.9253	13.45123	81.46419	10.45113	457.75
131.6119	13.47169	114.9491	10.47159	595.5
83.30437	13.49215	106.4392	10.49205	710
195.0028	13.51262	118.9347	10.51252	764.75
169.7071	13.53308	81.43549	10.53298	745.75
100.4173	13.55354	113.9416	10.55344	663.75
167.1334	13.574	49.45297	10.5739	529.75
131.8555	13.59447	84.96966	10.59437	366.5
123.5834	13.61493	53.49165	10.61483	216.5
155.3172	13.63539	-21.981	10.63529	106
116.057	13.65586	67.55155	10.65576	47.75
105.8026	13.67632	43.08946	10.67622	27.25
157.5541	13.69678	109.6327	10.69668	25.25
163.3116	13.71724	64.18118	10.71714	21.75
-1.92506	13.73771	62.73499	10.73761	17
91.84419	13.75817	72.2941	10.75807	12.25
185.6194	13.77863	81.85852	10.77853	9.5
134.4004	13.79909	116.4282	10.79899	7.75
121.1874	13.81956	108.0033	10.81946	5.25
128.9803	13.84002	20.5836	10.83992	6.5
78.77904	13.86048	124.1692	10.86038	9
79.58372	13.88095	104.7602	10.88085	7.5
68.3943	13.90141	59.3564	10.90131	10
36.21079	13.92187	63.95794	10.92177	10.75
30.03318	13.94233	70.56478	10.94223	9.5
-22.1385	13.9628	46.17693	10.9627	7.75
85.69567	13.98326	32.79438	10.98316	6
71.53577	14.00372	39.41713	11.00362	6
113.3818	14.02419	67.04519	11.02409	5.25
20.23369	14.04465	29.67855	11.04455	5.75
59.0915	14.06511	18.31721	11.06501	6.75
57.95522	14.08557	73.96118	11.08547	6.75
-4.17516	14.10604	20.61045	11.10594	7.75
1.700369	14.1265	-5.73498	11.1264	6.75
23.5818	14.14696	3.924896	11.14686	4.75
35.46913	14.16742	60.59007	11.16732	1.25
1.36237	14.18789	85.26055	11.18779	1.25
40.26151	14.20835	47.93634	11.20825	1.75
72.16656	14.22881	23.61743	11.22871	0.5
45.07751	14.24928	3.303818	11.24918	0
11.99436	14.26974	-65.0045	11.26964	0
-0.08288	14.2902	40.69251	11.2901	0.5
-50.1542	14.31066	51.39481	11.31056	2.75
31.78034	14.33113	-18.8976	11.33103	3.75

27.72081	14.35159	-11.1847	11.35149	7.5
89.66718	14.37205	31.53353	11.37195	9.75
26.61946	14.39252	60.25704	11.39242	9.5
-19.4224	14.41298	-17.0141	11.41288	10.25
47.54173	14.43344	-32.28	11.43334	9.75
50.51171	14.4539	1.308658	11.4538	8.25
115.4876	14.47437	-5.09736	11.47427	6.75
63.4694	14.49483	5.501933	11.49473	6.25
104.4571	14.51529	62.10653	11.51519	5.5
122.4507	14.53575	13.71642	11.53565	6.25
117.4502	14.55622	44.33162	11.55612	7.5
146.4556	14.57668	3.95212	11.57658	8
158.4669	14.59714	45.57793	11.59704	7.5
108.4842	14.61761	119.209	11.61751	9.75
154.5073	14.63807	146.8454	11.63797	9.25
224.5363	14.65853	27.48716	11.65843	9.25
109.5712	14.67899	32.13417	11.67889	8
163.6121	14.69946	140.7865	11.69936	5.75
24.65881	14.71992	115.4441	11.71982	3.25
-27.2885	14.74038	94.10705	11.74028	3.75
43.77	14.76085	97.77528	11.76075	4
24.83445	14.78131	109.4488	11.78121	7.5
61.9048	14.80177	107.1276	11.80167	9
9.981059	14.82223	44.81178	11.82213	9.25
23.06322	14.8427	-12.4988	11.8426	8.75
19.15129	14.86316	81.19597	11.86306	11.75
-4.75475	14.88362	64.89602	11.88352	12.25
-11.6549	14.90408	-64.3986	11.90398	13.5
33.45091	14.92455	44.31202	11.92445	12.25
67.56258	14.94501	25.02798	11.94491	14
29.68017	14.96547	-9.25076	11.96537	17
4.80366	14.98594	-62.5242	11.98584	19.75
35.93305	15.0064	12.20767	12.0063	21
7.06835	15.02686	-52.0552	12.02676	20.75
-19.7904	15.04732	19.68732	12.04722	25.25
19.35666	15.06779	36.43509	12.06769	30.5
41.50966	15.08825	-18.8118	12.08815	36.75
-53.3314	15.10871	-30.0534	12.10861	45.75
-14.1666	15.12918	29.71024	12.12908	55
-53.9959	15.14964	-22.5208	12.14954	68.75
14.37626	15.1701	28.25352	12.17	85
32.75432	15.19056	3.033116	12.19046	107
-9.86173	15.21103	-2.18199	12.21093	139.25
-7.47186	15.23149	28.60822	12.23139	183.25
44.9239	15.25195	25.40372	12.25185	244
55.32558	15.27241	-1.79547	12.27231	315.25
54.73315	15.29288	27.01064	12.29278	391.25
56.14663	15.31334	33.82205	12.31324	453.5
16.56601	15.3338	-3.36123	12.3337	479.75
32.9913	15.35427	14.46079	12.35417	469.25
8.422485	15.37473	0.288112	12.37463	417.75
81.85958	15.39519	44.12074	12.39509	341.25
4.302577	15.41565	-16.0413	12.41555	254.75
4.751478	15.43612	27.8019	12.43602	171.75
33.20628	15.45658	1.650435	12.45648	108
-22.333	15.47704	-61.4957	12.47694	67.25
-17.8664	15.49751	-1.10446	12.49741	50.25
-29.3939	15.51797	50.29211	12.51787	47.75

-25.1986	15.53843	32.69399	12.53833	43.5
-8.99737	15.55889	-12.8988	12.55879	41.25
32.20974	15.57936	24.51364	12.57926	40
0.42275	15.59982	-21.0686	12.59972	41
25.64167	15.62028	-24.6455	12.62018	43
64.86649	15.64074	-16.2171	12.64064	47.75
-0.90279	15.66121	-49.7834	12.66111	52.75
3.333839	15.68167	24.65558	12.68157	52.25
-21.4236	15.70213	-21.9001	12.70203	56
2.824806	15.7226	-3.45052	12.7225	62
62.07915	15.74306	-32.9956	12.74296	74.5
-18.6606	15.76352	3.268159	12.76342	92.5
6.605537	15.78398	-13.4628	12.78388	121
39.87759	15.80445	-2.18839	12.80435	158.75
56.15554	15.82491	-17.9087	12.82481	203.25
36.4394	15.84537	48.37627	12.84527	247.5
51.72916	15.86584	10.66656	12.86574	276
20.02483	15.8863	37.96215	12.8862	278.75
61.32641	15.90676	6.263044	12.90666	259.5
139.6339	15.92722	46.56924	12.92712	223.75
167.9473	15.94769	64.88074	12.94759	180.75
188.2665	15.96815	62.19754	12.96805	126.25
186.5917	15.98861	37.51965	12.98851	81
97.92281	16.00907	96.84705	13.00897	50.5
123.2598	16.02954	84.17976	13.02944	37.25
53.60271	16.05	106.5178	13.0499	36.25
-11.0485	16.07046	66.8611	13.07036	39
36.30621	16.09093	76.20972	13.09083	39
5.666823	16.11139	14.56364	13.11129	42.5
8.033337	16.13185	26.92287	13.13175	46.25
56.40575	16.15231	33.2874	13.15221	48.5
23.78408	16.17278	-7.34276	13.17268	47.75
25.1683	16.19324	-14.9676	13.19314	54
-6.44157	16.2137	51.41281	13.2136	55.75
62.95446	16.23417	-25.2014	13.23407	62.5
27.3564	16.25463	-16.8104	13.25453	65
-0.23576	16.27509	-10.4141	13.27499	62
70.17799	16.29555	-6.0124	13.29545	60
72.59763	16.31602	41.39456	13.31592	57
14.02318	16.33648	-27.1932	13.33638	52.25
28.45464	16.35694	-17.7756	13.35684	49
33.892	16.3774	67.64724	13.3773	53
18.33526	16.39787	9.07541	13.39777	57.25
53.78443	16.41833	10.50888	13.41823	63.25
41.2395	16.43879	13.94766	13.43869	68.25
74.70048	16.45926	14.39173	13.45916	70
-13.8326	16.47972	17.84111	13.47962	68.25
0.64014	16.50018	34.2958	13.50008	72.75
-49.8812	16.52064	42.75578	13.52054	73.25
11.60342	16.54111	-9.77893	13.54101	74.75
53.09391	16.56157	17.69166	13.56147	76.5
12.59031	16.58203	38.16756	13.58193	82
-9.90739	16.6025	6.648759	13.6024	86.75
3.60082	16.62296	-26.8647	13.62286	93
-1.88507	16.64342	-3.37293	13.64332	94.5
11.63495	16.66388	12.12417	13.66378	96.75
38.16086	16.68435	28.62659	13.68425	97.75
-14.3073	16.70481	-17.8657	13.70471	103.5

-15.7696	16.72527	73.64732	13.72517	110
57.77404	16.74573	-23.8344	13.74563	114.75
3.323574	16.7662	-16.3107	13.7661	120.5
-46.121	16.78666	-6.78181	13.78656	127.75
32.44035	16.80712	5.75242	13.80702	136.25
0.007597	16.82759	20.29195	13.82749	141.75
0.580746	16.84805	-2.16321	13.84795	146.5
-10.8402	16.86851	25.38693	13.86841	149.75
-36.2552	16.88897	-26.0576	13.88887	151.25
27.33562	16.90944	-45.0271	13.90934	154.75
-4.06762	16.9299	59.00875	13.9298	160.5
-4.46495	16.95036	-25.9501	13.95026	164.5
15.14362	16.97083	-25.9037	13.97073	171
-2.2419	16.99129	2.854668	13.99119	175.75
6.378479	17.01175	-34.3817	14.01165	182.5
-27.9952	17.03221	6.387249	14.03211	190.5
-6.13875	17.05268	-9.83851	14.05258	197.75
7.723643	17.07314	35.94104	14.07304	202.75
25.59194	17.0936	74.72589	14.0935	208.5
-6.53386	17.11406	0.516048	14.11396	219
-14.6538	17.13453	18.31151	14.13443	227.75
45.23225	17.15499	17.11227	14.15489	239.25
3.124161	17.17545	-5.08167	14.17535	250.5
4.021976	17.19592	-16.2703	14.19582	256
38.9257	17.21638	-16.4536	14.21628	267.75
42.83532	17.23684	33.36834	14.23674	276.25
17.75085	17.2573	33.19562	14.2572	280.75
2.672276	17.27777	19.0282	14.27767	288
87.59961	17.29823	20.86608	14.29813	297
65.53285	17.31869	13.70926	14.31859	308.75
8.471992	17.33916	-17.4422	14.33906	312.25
0.417039	17.35962	-9.58846	14.35952	320.25
-25.632	17.38008	-9.72936	14.37998	325.25
-1.13825	17.40054	-1.86496	14.40044	330
31.36141	17.42101	15.00474	14.42091	340.5
-24.133	17.44147	45.87974	14.44137	345.25
42.37845	17.46193	8.760051	14.46183	351.25
31.89582	17.48239	-4.35434	14.48229	360.75
36.4191	17.50286	-15.4634	14.50276	365.25
27.94828	17.52332	-2.56721	14.52322	367.5
36.48336	17.54378	13.33431	14.54368	371
-33.9756	17.56425	12.24114	14.56415	380.25
-19.4288	17.58471	13.15326	14.58461	383.75
-6.87596	17.60517	-12.9293	14.60507	395
10.68275	17.62563	-49.0066	14.62553	405.75
10.24735	17.6461	44.92146	14.646	414.5
17.81786	17.66656	7.854797	14.66646	425.75
39.39427	17.68702	3.793438	14.68692	435.25
41.97659	17.70749	64.73738	14.70739	439.75
-7.43519	17.72795	10.68663	14.72785	455.75
52.15893	17.74841	50.64118	14.74831	479.25
20.75896	17.76887	-1.39896	14.76877	507.75
21.36489	17.78934	-22.4338	14.78924	546.25
-12.0233	17.8098	9.536654	14.8097	592.5
65.59447	17.83026	-35.4876	14.83016	636.5
24.21811	17.85072	19.49348	14.85062	676.75
-17.1523	17.87119	104.4799	14.87109	701.5
69.48311	17.89165	115.4715	14.89155	693.75

95.12447	17.91211	67.46851	14.91201	663.75
58.77173	17.93258	83.47078	14.93248	619
131.4249	17.95304	104.4784	14.95294	561.5
169.084	17.9735	101.4912	14.9734	503
154.7489	17.99396	138.5094	14.99386	459.5
157.4198	18.01443	148.5329	15.01433	418.75
164.0966	18.03489	249.5617	15.03479	396
110.7793	18.05535	243.5958	15.05525	386.25
49.46785	18.07582	228.6352	15.07572	375.25
42.16234	18.09628	255.6799	15.09618	360
-52.1373	18.11674	143.7299	15.11664	352.25
17.56903	18.1372	78.78525	15.1371	343.75
-1.71877	18.15767	53.84586	15.15757	335.75
-20.0007	18.17813	22.91178	15.17803	326
45.72335	18.19859	-1.017	15.19849	320.5
-6.54674	18.21905	-13.9405	15.21895	308
32.18908	18.23952	-17.8587	15.23942	305
48.9308	18.25998	59.22847	15.25988	305.25
-13.3216	18.28044	-12.6791	15.28034	296.75
18.43195	18.30091	-35.5814	15.30081	290.25
28.19139	18.32137	33.52168	15.32127	286.25
-21.0433	18.34183	-29.37	15.34173	281.25
-0.27204	18.36229	13.74367	15.36219	270.5
-18.4949	18.38276	2.862614	15.38266	258.25
-22.7118	18.40322	24.98687	15.40312	249.75
-26.9229	18.42368	-9.88358	15.42358	244.25
44.87197	18.44415	-22.7487	15.44405	245.25
5.672726	18.46461	6.391437	15.46451	245.25
-4.52061	18.48507	29.5369	15.48497	237.75
-3.70804	18.50553	-15.3123	15.50543	235
3.110429	18.526	17.84374	15.5259	230
-17.0652	18.54646	-29.9949	15.54636	226
-9.23492	18.56692	-17.5081	15.56682	217
24.60127	18.58738	49.9839	15.58728	211.5
17.44335	18.60785	8.481251	15.60775	210
8.291346	18.62831	7.983905	15.62821	212
-0.85476	18.64877	-5.50814	15.64867	209
-47.995	18.66924	-52.9949	15.66914	208
1.68071	18.6897	6.469137	15.6896	208
43.36229	18.71016	-17.0615	15.71006	209.25
-5.95024	18.73062	0.413076	15.73052	205.5
44.74315	18.75109	28.893	15.75099	201.5
-2.55757	18.77155	23.37823	15.77145	194.5
-0.85238	18.79201	15.86876	15.79191	188.5
39.85872	18.81248	3.364593	15.81238	185.5
24.57572	18.83294	23.86573	15.83284	183.25
11.29862	18.8534	62.37217	15.8533	185.25
88.02743	18.87386	-4.11609	15.87376	189.5
63.76214	18.89433	44.40096	15.89423	194.25
90.50275	18.91479	30.92331	15.91469	198.25
131.2493	18.93525	42.45096	15.93515	201
179.0017	18.95571	24.98392	15.95561	199.5
249.76	18.97618	8.522176	15.97608	198.25
186.5242	18.99664	4.065737	15.99654	196.5
249.2944	19.0171	95.6146	16.017	197.25
203.0704	19.03757	82.16877	16.03747	195
241.8524	19.05803	114.7282	16.05793	202.5
207.6402	19.07849	138.293	16.07839	208

270.434	19.09895	178.8631	16.09885	218.5
246.2336	19.11942	232.4385	16.11932	232
168.0392	19.13988	318.0192	16.13978	253.25
167.8506	19.16034	252.6051	16.16024	271.25
85.66798	19.18081	283.1964	16.18071	295
37.49125	19.20127	213.793	16.20117	306.25
29.32042	19.22173	219.3949	16.22163	310.25
20.1555	19.24219	132.0021	16.24209	300.5
7.996474	19.26266	74.61462	16.26256	285.75
44.84336	19.28312	89.23243	16.28302	267.75
-37.3039	19.30358	20.85554	16.30348	247
4.554836	19.32404	26.48395	16.32394	232.75
-16.5806	19.34451	42.11767	16.34441	224.25
1.289931	19.36497	-21.2433	16.36487	216.5
12.16633	19.38543	30.40101	16.38533	218.5
-11.9514	19.4059	7.05063	16.4058	220
10.93685	19.42636	22.70556	16.42626	223.5
25.83097	19.44682	-11.6342	16.44672	216.75
23.73098	19.46728	36.03132	16.46718	215.75
24.63691	19.48775	10.70216	16.48765	214.25
2.548732	19.50821	-14.6217	16.50811	216
-18.5335	19.52867	10.05974	16.52857	219.75
21.3901	19.54914	-9.25351	16.54904	222.25
10.31963	19.5696	-16.5615	16.5695	224.25
-13.7449	19.59006	-9.86411	16.58996	226
46.19642	19.61052	27.83855	16.61042	231.75
-5.85633	19.63099	-0.4535	16.63089	228.5
44.09683	19.65145	-33.7402	16.65135	219.5
-6.94412	19.67191	8.97833	16.67181	215
-23.9792	19.69237	16.7022	16.69227	205.75
-1.59862	19.71284	42.43137	16.71274	200.5
15.78783	19.7333	-3.83416	16.7332	192
-1.81982	19.75376	-1.09438	16.75366	184.25
9.578432	19.77423	43.6507	16.77413	177
3.982589	19.79469	16.40108	16.79459	167.25
35.39265	19.81515	7.15677	16.81505	164.75
20.80862	19.83561	25.91776	16.83551	160
75.23048	19.85608	47.68405	16.85598	152.5
9.658256	19.87654	35.45565	16.87644	147
21.09193	19.897	2.232548	16.8969	141
-8.46849	19.91747	-5.98525	16.91737	134
59.977	19.93793	37.80225	16.93783	128
44.42839	19.95839	4.595064	16.95829	123.75
-0.11432	19.97885	22.39318	16.97875	119.25
17.34887	19.99932	0.19659	16.99922	115.5
31.81797	20.01978	-24.9947	17.01968	111
8.292976	20.04024	0.529931	17.04014	106.75
39.77388	20.0607	24.05986	17.0606	97.5
16.2607	20.08117	12.59509	17.08107	91.25
25.75341	20.10163	3.135619	17.10153	83.25
10.25203	20.12209	-8.31855	17.12199	77.25
-6.24345	20.14256	68.23259	17.14246	73.5
10.26698	20.16302	42.78904	17.16292	71.25
-16.2167	20.18348	10.35078	17.18338	66.25
5.305544	20.20394	-6.08217	17.20384	66
-7.16632	20.22441	-4.50982	17.22431	61.5
1.367725	20.24487	13.06783	17.24477	56.5
35.90767	20.26533	47.65079	17.26523	49

-16.5465	20.2858	8.239052	17.2857	45.75
-31.9947	20.30626	-5.16739	17.30616	41.75
16.47645	20.32672	12.43148	17.32662	40
-5.04646	20.34718	-41.9643	17.34708	34.5
2.436523	20.36765	31.64512	17.36755	31.75
8.925413	20.38811	53.2599	17.38801	29
10.42021	20.40857	13.87998	17.40847	29
14.92091	20.42903	-24.4946	17.42893	31.75
19.42751	20.4495	-13.6534	17.4494	32
21.94001	20.46996	13.19313	17.46986	31
28.45842	20.49042	26.04497	17.49032	27.75
-14.0173	20.51089	34.90211	17.51079	25.25
24.51295	20.53135	-20.2354	17.53125	21
24.04907	20.55181	3.632306	17.55171	15
69.5911	20.57227	-39.4946	17.57217	11.25
25.13903	20.59274	-2.82207	17.59264	7.25
55.69286	20.6132	16.85581	17.6131	4.25
11.2526	20.63366	42.53899	17.63356	6
-2.18176	20.65413	24.22747	17.65403	5.5
50.38979	20.67459	-0.07874	17.67449	3.75
58.96723	20.69505	19.62034	17.69495	0.25
5.550585	20.71551	5.324737	17.71541	1.25
-1.86016	20.73598	21.03443	17.73588	1.25
58.735	20.75644	0.74943	17.75634	2
93.33607	20.7769	12.46973	17.7768	2
53.94303	20.79736	67.19534	17.79726	1.75
107.5559	20.81783	71.92625	17.81773	0.5
137.1747	20.83829	82.66245	17.83819	0.75
94.79936	20.85875	69.40397	17.85865	0
112.4299	20.87922	66.15079	17.87912	1
66.06643	20.89968	24.90291	17.89958	2
58.70882	20.92014	52.66033	17.92004	3.5
40.35712	20.9406	43.42305	17.9405	5
-22.9887	20.96107	31.19108	17.96097	6
32.67142	20.98153	70.96442	17.98143	7.25
-48.6626	21.00199	54.74305	18.00189	9.75
-4.99067	21.02246	-3.47301	18.02235	14.25
-14.3129	21.04292	-29.6838	18.04282	22.75
9.370868	21.06338	8.110778	18.06328	27.75
29.06049	21.08384	43.91063	18.08374	44.25
10.75602	21.10431	-17.2842	18.10421	71
32.45745	21.12477	-4.47377	18.12467	114
23.16478	21.14523	17.34199	18.14513	176.75
-10.122	21.16569	1.16305	18.16559	246
32.59716	21.18616	-40.0106	18.18606	315.25
17.3222	21.20662	5.821081	18.20652	364.25
-16.9468	21.22708	4.658051	18.22698	386.5
17.79001	21.24755	66.50032	18.24745	369.25
11.53276	21.26801	-9.6521	18.26791	318
54.28143	21.28847	-0.79922	18.28837	248
20.03599	21.30893	28.05896	18.30883	170.75
-15.2035	21.3294	-31.0776	18.3293	103
-16.4372	21.34986	45.79124	18.34976	56.5
15.33511	21.37032	24.66533	18.37022	25.5
51.11329	21.39079	-27.4553	18.39068	15.5
34.89737	21.41125	-12.5706	18.41115	10.25
61.68736	21.43171	10.31942	18.43161	10.5
16.48325	21.45217	22.21473	18.45207	6.25

63.28505	21.47264	29.11533	18.47254	9.25
61.09275	21.4931	2.021245	18.493	9
37.90635	21.51356	31.93246	18.51346	8
36.72586	21.53402	-2.15103	18.53392	6.5
13.55127	21.55449	-15.2292	18.55439	5
31.38258	21.57495	50.69792	18.57485	3.75
92.2198	21.59541	47.63034	18.59531	5.5
77.06293	21.61588	29.56807	18.61578	4.75
71.91195	21.63634	72.51111	18.63624	3.25
126.7669	21.6568	116.4594	18.6567	1.5
153.6277	21.67726	91.41308	18.67716	4
251.4945	21.69773	134.372	18.69763	3.75
270.3671	21.71819	111.3363	18.71809	3
339.2456	21.73865	223.3058	18.73855	2.5
398.1301	21.75912	109.2807	18.75901	1.25
472.0204	21.77958	207.2608	18.77948	1.5
429.9167	21.80004	235.2463	18.79994	0.5
472.8189	21.8205	340.237	18.8204	0
429.7269	21.84097	469.2331	18.84087	1.25
358.6409	21.86143	457.2345	18.86133	2.75
277.5608	21.88189	501.2412	18.88179	3.75
309.4865	21.90235	431.2531	18.90225	4.25
180.4182	21.92282	384.2704	18.92272	4.75
272.3558	21.94328	276.293	18.94318	6
286.2993	21.96374	190.3208	18.96364	6.5
466.2487	21.98421	242.354	18.98411	6.75
522.204	22.00467	138.3925	19.00457	5.75
689.1652	22.02513	176.4363	19.02503	5.25
940.1323	22.04559	212.4854	19.04549	6.75
1206.105	22.06606	286.5398	19.06596	6.75
1484.084	22.08652	384.5995	19.08642	7.75
1648.069	22.10698	473.6645	19.10688	10.5
1516.06	22.12745	630.7348	19.12734	15.25
1167.056	22.14791	713.8104	19.14781	23
952.0589	22.16837	832.8914	19.16827	35.75
675.0673	22.18883	864.9775	19.18873	57.25
462.0817	22.2093	713.0691	19.2092	91
307.1019	22.22976	663.1659	19.22966	142.75
183.1281	22.25022	569.268	19.25012	221.5
149.1601	22.27068	446.3755	19.27058	322
98.19806	22.29115	295.4882	19.29105	424.75
76.24191	22.31161	191.6062	19.31151	513
70.29167	22.33207	129.7296	19.33197	565
68.34734	22.35254	83.8582	19.35244	568.25
61.4089	22.373	65.99215	19.3729	522.5
19.47637	22.39346	36.1314	19.39336	441.5
28.54974	22.41392	59.27595	19.41382	334.5
9.629022	22.43439	39.4258	19.43429	229.25
3.714204	22.45485	33.58096	19.45475	144.75
39.80529	22.47531	-2.25858	19.47521	86.5
-13.0977	22.49578	18.90718	19.49567	48
-46.9948	22.51624	61.07825	19.51614	30
13.76417	22.5367	12.25462	19.5366	20.25
-7.47093	22.55716	51.43629	19.55706	17.75
-3.70012	22.57763	27.62327	19.57753	15
-6.92341	22.59809	16.81555	19.59799	17.5
-30.1408	22.61855	-30.9869	19.61845	14.75
46.64772	22.63901	-4.78399	19.63891	14.5

51.44214	22.65948	26.4242	19.65938	10.5
-1.75753	22.67994	48.63769	19.67984	9
38.0487	22.7004	17.85649	19.7003	6
27.86083	22.72087	38.08059	19.72077	3.5
-29.3211	22.74133	37.30999	19.74123	2.5
-2.49719	22.76179	26.54469	19.76169	4.75
-0.66735	22.78225	25.7847	19.78215	2.75
-30.8316	22.80272	21.03001	19.80262	3.75
21.01005	22.82318	3.280619	19.82308	4.25
-15.1424	22.84364	4.536536	19.84354	3.75
9.711068	22.86411	33.79776	19.864	2.25
42.57043	22.88457	0.064278	19.88447	4
76.4357	22.90503	27.3361	19.90493	3.25
4.306871	22.92549	43.61323	19.92539	2.5
13.18395	22.94596	12.89566	19.94586	1.75
11.06693	22.96642	38.1834	19.96632	1.25
15.95581	22.98688	16.47644	19.98678	0.25
-18.1494	23.00734	-29.2252	20.00724	0
-24.2487	23.02781	39.07842	20.02771	1.75
-4.95529	23.04827	58.38737	20.04817	3.5
-31.656	23.06873	30.70162	20.06863	3.5
18.64928	23.0892	-0.97883	20.0891	5
17.96042	23.10966	-3.65397	20.10956	6.5
17.27746	23.13012	-23.3238	20.13002	7.25
22.60041	23.15058	2.596083	20.15048	7
25.92926	23.17105	6.521277	20.17095	6.5
4.264007	23.19151	36.45178	20.19141	3.5
15.60466	23.21197	41.38758	20.21187	1.75
41.95123	23.23244	30.32868	20.23233	3.5
0.30369	23.2529	27.27509	20.2528	4.25
3.662059	23.27336	25.2268	20.27326	4.5
17.02633	23.29382	47.18381	20.29372	5.75
33.39651	23.31429	87.14613	20.31419	7.75
29.77259	23.33475	3.113743	20.33465	7.25
77.15457	23.35521	4.086665	20.35511	8
28.54246	23.37567	26.06489	20.37557	5.75
30.93626	23.39614	30.04842	20.39604	4.25
15.33595	23.4166	7.037251	20.4165	6.25
17.74155	23.43706	44.03138	20.43696	7.75
-26.8469	23.45753	-1.96918	20.45743	8.75
1.570461	23.47799	12.03556	20.47789	9
-10.0062	23.49845	18.04561	20.49835	8
6.422988	23.51891	1.060955	20.51881	7.75
30.85811	23.53938	44.0816	20.53928	5
-3.70087	23.55984	31.10756	20.55974	3.25
1.746057	23.5803	46.13882	20.5802	0
-6.80111	23.60077	55.17537	20.60066	1.5
10.65762	23.62123	3.217238	20.62113	4
1.122262	23.64169	9.264403	20.64159	5.5
22.5928	23.66215	-23.6831	20.66205	6
-1.93075	23.68262	34.37465	20.68252	5.75
18.5516	23.70308	102.4377	20.70298	7.75
0.039855	23.72354	15.5061	20.72344	8.5
34.53401	23.744	61.57978	20.7439	8
15.03408	23.76447	2.658764	20.76437	5.75
6.540041	23.78493	49.74305	20.78483	5
30.05191	23.80539	20.83264	20.80529	6.25
41.56968	23.82586	40.92754	20.82576	8.5

13.09336	23.84632	36.02773	20.84622	8.75
-2.37706	23.86678	40.13323	20.86668	9
24.15843	23.88724	12.24404	20.88714	12
15.69982	23.90771	54.36015	20.90761	18
28.24711	23.92817	65.48155	20.92807	22.75
38.80031	23.94863	10.60827	20.94853	35
59.35941	23.9691	20.74028	20.96899	46.25
55.92441	23.98956	62.8776	20.98946	64.25
59.49532	24.01002	29.02022	21.00992	83.75
90.07214	24.03048	96.16814	21.03038	102.25
129.6548	24.05095	118.3214	21.05085	111.5
150.2435	24.07141	65.4799	21.07131	117.75
166.838	24.09187	80.64375	21.09177	112.75
297.4384	24.11233	104.8129	21.11223	100.5
339.0447	24.1328	163.9873	21.1327	83.25
471.657	24.15326	250.1671	21.15316	67.5
586.2751	24.17372	233.3521	21.17362	48
662.8992	24.19419	318.5425	21.19409	39.75
688.5291	24.21465	459.7381	21.21455	35.5
594.165	24.23511	604.9391	21.23501	32
589.8067	24.25557	681.1453	21.25547	31.25
502.4544	24.27604	788.3569	21.27594	36
361.1079	24.2965	885.5737	21.2964	37.25
289.7674	24.31696	981.7959	21.31686	40.75
175.4328	24.33743	786.0234	21.33732	43
149.1041	24.35789	651.2562	21.35779	43.25
49.78124	24.37835	510.4943	21.37825	43.75
81.46432	24.39881	343.7376	21.39871	48.25
76.15331	24.41928	236.9863	21.41918	49
34.84819	24.43974	188.2403	21.43964	48
45.54899	24.4602	59.4996	21.4601	49.5
29.25569	24.48066	43.76419	21.48056	51.25
-17.0317	24.50113	61.03409	21.50103	53.5
-6.31321	24.52159	23.30929	21.52149	55.25
-5.5888	24.54205	1.589789	21.54195	58.5
-11.8585	24.56252	49.8756	21.56242	61.75
28.87773	24.58298	43.16671	21.58288	70.75
44.61985	24.60344	18.46312	21.60334	79.5
20.36788	24.6239	-3.23517	21.6238	86
9.121806	24.64437	-13.9281	21.64427	93.25
-15.1184	24.66483	5.384173	21.66473	101
30.64738	24.68529	-14.2982	21.68519	108.75
-2.58098	24.70576	-27.9753	21.70565	116.5
-12.8034	24.72622	21.35296	21.72612	125.25
-25.02	24.74668	9.686492	21.74658	135.25
0.973676	24.76714	32.02533	21.76704	140.25
31.97325	24.78761	-23.6305	21.78751	149
32.97873	24.80807	46.71891	21.80797	157.75
-42.0099	24.82853	30.07366	21.82843	168.75
-2.99261	24.84899	38.43371	21.84889	181
28.03058	24.86946	-8.20094	21.86936	201
28.05967	24.88992	-3.83028	21.88982	229.5
-15.9053	24.91038	-18.4543	21.91028	280
2.135562	24.93085	38.92694	21.93075	363
29.18237	24.95131	12.31351	21.95121	477.75
14.23507	24.97177	1.705375	21.97167	628.75
-9.70632	24.99223	-3.89745	21.99213	791.75
6.358199	25.0127	-30.495	22.0126	936.75

4.428617	25.03316	4.370481	22.03306	1037.75
-31.4951	25.05362	-46.7588	22.05352	1073
-3.67214	25.07409	44.11731	22.07398	1036.75
11.15669	25.09455	48.99868	22.09445	939.5
53.99143	25.11501	-1.11465	22.11491	811.5
39.83206	25.13547	2.777329	22.13537	682.75
33.6786	25.15594	-10.3254	22.15584	579.75
5.531048	25.1764	-8.42281	22.1763	526.5
28.3894	25.19686	-6.51493	22.19676	530.25
52.25365	25.21732	-46.6017	22.21722	590.5
-38.8762	25.23779	18.31676	22.23769	723.25
20.99987	25.25825	-24.7595	22.25815	942.75
79.88183	25.27871	-21.8304	22.27861	1246.25
67.7697	25.29918	30.10405	22.29908	1583.75
78.66347	25.31964	14.04375	22.31954	1887.5
5.563143	25.3401	3.988758	22.34	2104.75
89.46872	25.36056	-8.06093	22.36046	2185
109.3802	25.38103	0.894681	22.38093	2112.75
160.2976	25.40149	5.855598	22.40139	1905.25
263.2209	25.42195	60.82182	22.42185	1592.5
434.1501	25.44242	51.79334	22.44231	1260.75
473.0852	25.46288	108.7702	22.46278	977.5
588.0262	25.48334	108.7523	22.48324	773
537.9731	25.5038	87.73972	22.5037	633.5
491.9259	25.52427	143.7325	22.52417	553.5
484.8846	25.54473	87.7305	22.54463	519.5
397.8492	25.56519	134.7338	22.56509	504.75
405.8198	25.58565	164.7425	22.58555	496.25
335.7962	25.60612	170.7564	22.60602	482.75
347.7785	25.62658	154.7757	22.62648	475.25
298.7668	25.64704	227.8002	22.64694	473.25
195.7609	25.66751	189.8301	22.66741	468.75
144.7609	25.68797	175.8653	22.68787	464.5
120.7669	25.70843	167.9057	22.70833	454.5
128.7787	25.72889	116.9515	22.72879	454.25
89.79646	25.74936	137.0025	22.74926	452.75
68.82012	25.76982	73.05892	22.76972	447.75
41.84969	25.79028	67.1206	22.79018	441.5
-3.11485	25.81075	87.18758	22.81064	437.5
41.92652	25.83121	41.25986	22.83111	433
32.97379	25.85167	40.33744	22.85157	426.75
-10.973	25.87213	22.42033	22.87203	415.75
16.08605	25.8926	-16.4915	22.8925	404.75
25.15103	25.91306	62.60202	22.91296	392.5
72.22192	25.93352	27.70082	22.93342	382.75
25.29871	25.95398	20.80492	22.95388	369.25
-0.6186	25.97445	-22.0857	22.97435	355.5
-10.53	25.99491	45.02903	22.99481	348
50.56451	26.01537	-17.851	23.01527	339.75
30.66492	26.03584	15.27435	23.03574	326
-42.2288	26.0563	-8.59503	23.0562	322.25
50.88344	26.07676	-16.4591	23.07666	311.75
55.00156	26.09722	0.682108	23.09712	300
-6.87442	26.11769	37.82863	23.11759	291.25
2.25551	26.13815	44.98046	23.13805	277
43.39134	26.15861	25.13759	23.15851	260.75
69.53307	26.17908	69.30003	23.17897	245.5
81.68071	26.19954	2.467766	23.19944	233

127.8343	26.22	27.64081	23.2199	218.75
212.9937	26.24046	110.8192	23.24036	206.25
193.1591	26.26093	131.0028	23.26083	198.5
338.3303	26.28139	124.1918	23.28129	189.25
393.5075	26.30185	115.386	23.30175	183
347.6905	26.32231	116.5856	23.32221	173.25
470.8795	26.34278	241.7904	23.34268	163.75
558.0743	26.36324	317.0006	23.36314	154
609.2751	26.3837	336.216	23.3836	143.25
786.4818	26.40417	377.4368	23.40407	134
886.6944	26.42463	500.6629	23.42453	125
792.9129	26.44509	517.8943	23.44499	117.5
661.1373	26.46555	656.1309	23.46545	112.5
614.3676	26.48602	678.3729	23.48592	111.5
452.6038	26.50648	644.6202	23.50638	113.75
346.8459	26.52694	661.8728	23.52684	113.5
161.0939	26.54741	581.1307	23.5473	115.5
149.3478	26.56787	423.3939	23.56777	116.25
85.60758	26.58833	361.6624	23.58823	110.75
100.8733	26.60879	197.9362	23.60869	105.25
75.14491	26.62926	115.2153	23.62916	94.25
51.42244	26.64972	94.49969	23.64962	85.25
59.70586	26.67018	107.7894	23.67008	74.5
37.9952	26.69064	73.08441	23.69054	64.75
68.29043	26.71111	102.3847	23.71101	56.25
33.59157	26.73157	48.69035	23.73147	50.75
19.89861	26.75203	-11.9987	23.75193	43.5
12.21156	26.7725	44.31749	23.7724	40.25
31.53041	26.79296	26.63902	23.79286	34
6.855166	26.81342	56.96586	23.81332	31.75
-26.8142	26.83388	51.29799	23.83378	26.25
-15.8898	26.85435	32.63543	23.85425	26.75
20.04056	26.87481	57.97817	23.87471	27.5
51.97678	26.89527	18.32621	23.89517	26.5
89.91891	26.91574	29.67956	23.91563	25.25
42.86694	26.9362	40.03821	23.9361	24.75
-34.1791	26.95666	12.40216	23.95656	23
14.78071	26.97712	92.77142	23.97702	23.75
55.74645	26.99759	47.14598	23.99749	21.75
38.7181	27.01805	17.52584	24.01795	20.5
36.69564	27.03851	-13.089	24.03841	18
48.6791	27.05897	20.30148	24.05887	20.25
9.668456	27.07944	4.697248	24.07934	24.25
24.66372	27.0999	9.098323	24.0998	25
35.66488	27.12036	-30.4953	24.12026	26.25
49.67195	27.14083	-8.74221	24.14073	26.5
51.68493	27.16129	1.016183	24.16119	28
18.7038	27.18175	36.77988	24.18165	30.25
6.728581	27.20221	37.54888	24.20211	31
-1.24074	27.22268	15.32318	24.22258	32.75
45.79585	27.24314	53.10279	24.24304	39.25
21.83835	27.2636	26.8877	24.2635	48
17.88674	27.28407	-11.3221	24.28396	64.25
26.94104	27.30453	12.47342	24.30443	90.25
49.00125	27.32499	24.27424	24.32489	135
59.06735	27.34545	87.08036	24.34535	208.75
29.13936	27.36592	60.89179	24.36582	316.25
-11.7827	27.38638	27.70851	24.38628	456

31.3011	27.40684	30.53054	24.40674	599.25
36.39082	27.4273	21.35787	24.4272	724.25
42.48645	27.44777	68.19051	24.44767	803.5
75.58798	27.46823	62.02845	24.46813	820.25
40.69542	27.48869	52.87169	24.48859	772.5
109.8088	27.50916	42.72024	24.50906	666.5
86.92799	27.52962	85.57409	24.52952	526.25
135.0531	27.55008	98.43324	24.54998	379.5
205.1842	27.57054	59.2977	24.57044	254.5
257.3211	27.59101	115.1675	24.59091	164.75
174.464	27.61147	144.0425	24.61137	102.75
267.6128	27.63193	126.9229	24.63183	68.75
316.7674	27.6524	157.8085	24.65229	49.5
457.928	27.67286	177.6995	24.67276	40.75
490.0945	27.69332	291.5958	24.69322	34.25
528.2668	27.71378	417.4974	24.71368	28.5
500.4451	27.73425	405.4042	24.73415	22
465.6293	27.75471	428.3164	24.75461	14.75
363.8194	27.77517	498.2339	24.77507	12
305.0154	27.79563	544.1567	24.79553	8.75
174.2173	27.8161	458.0848	24.816	7
169.4251	27.83656	428.0182	24.83646	6.25
38.63881	27.85702	340.9569	24.85692	5.5
38.85841	27.87749	242.9009	24.87739	4.25
67.08392	27.89795	208.8502	24.89785	1.5
55.31534	27.91841	96.80481	24.91831	0.75
20.55266	27.93887	116.7647	24.93877	0
23.79589	27.95934	101.7299	24.95924	0.5
63.04501	27.9798	31.70046	24.9797	2.75
35.30005	28.00026	116.6763	25.00016	6.25
48.56098	28.02073	33.6574	25.02062	8.5
27.82782	28.04119	52.64383	25.04109	9
70.10056	28.06165	34.63556	25.06155	9.5
36.37921	28.08211	74.63259	25.08201	8.25
100.6638	28.10258	41.63493	25.10248	6.25
-2.04578	28.12304	31.64257	25.12294	3.75
58.25058	28.1435	58.65551	25.1434	3
51.55284	28.16396	39.67375	25.16386	4.5
107.861	28.18443	102.6973	25.18433	5.5
83.17508	28.20489	64.72615	25.20479	7.25
106.495	28.22535	40.76031	25.22525	6.5
75.82093	28.24582	80.79977	25.24572	6
96.15271	28.26628	57.84453	25.26618	5.5
160.4904	28.28674	151.8946	25.28664	3.5
227.834	28.3072	174.95	25.3071	1.5
226.1835	28.32767	165.0106	25.32757	0.25
291.5389	28.34813	121.0766	25.34803	1.5
312.9002	28.36859	211.1479	25.36849	3.75
335.2674	28.38906	338.2245	25.38895	4.25
435.6405	28.40952	261.3063	25.40942	4.25
524.0195	28.42998	330.3935	25.42988	3.5
645.4044	28.45044	414.486	25.45034	5.5
678.7952	28.47091	469.5838	25.47081	7
774.192	28.49137	614.6869	25.49127	9.5
647.5946	28.51183	629.7953	25.51173	10
530.0031	28.53229	744.909	25.53219	14
464.4176	28.55276	822.028	25.55266	16.25
398.8379	28.57322	761.1523	25.57312	17.5

275.2642	28.59368	692.2819	25.59358	19
188.6963	28.61415	549.4169	25.61405	20.75
131.1344	28.63461	436.5571	25.63451	23.25
77.57831	28.65507	314.7026	25.65497	29.5
82.02817	28.67553	214.8534	25.67543	36.25
38.48393	28.696	192.0095	25.6959	51.5
30.9456	28.71646	142.171	25.71636	76.5
73.41317	28.73692	53.3377	25.73682	118.5
-24.1134	28.75739	64.50974	25.75728	184.25
3.366027	28.77785	77.68707	25.77775	284
-1.14869	28.79831	32.86971	25.79821	402
4.342497	28.81877	33.05766	25.81867	514
34.83959	28.83924	73.25091	25.83914	597.5
80.34258	28.8597	90.44946	25.8596	641.5
0.851481	28.88016	83.65331	25.88006	630.5
60.36628	28.90062	14.86247	25.90052	567.75
68.88699	28.92109	66.07694	25.92099	468.75
72.4136	28.94155	20.2967	25.94145	348.75
-3.05389	28.96201	13.52177	25.96191	240
2.484532	28.98248	-22.2479	25.98238	161.25
12.02885	29.00294	52.98781	26.00284	103.75
25.57908	29.0234	30.22879	26.0233	65.75
23.13521	29.04386	59.47506	26.04376	42.25
28.69724	29.06433	46.72665	26.06423	35
-2.73482	29.08479	-41.0165	26.08469	28.75
39.83902	29.10525	3.245723	26.10515	24.25
18.41877	29.12572	11.51322	26.12561	23.5
-28.9956	29.14618	49.78601	26.14608	21.75
-17.5439	29.16664	38.06411	26.16654	21.75
31.91367	29.1871	78.34751	26.187	21.75
18.37716	29.20757	83.63622	26.20747	18.25
59.84655	29.22803	57.93022	26.22793	16.25
33.32184	29.24849	93.22953	26.24839	15
-11.197	29.26895	37.53415	26.26885	15.5
69.29014	29.28942	38.84407	26.28932	13.75
19.78314	29.30988	74.15929	26.30978	14.25
112.282	29.33034	32.47981	26.33024	18.25
66.78686	29.35081	61.80563	26.35071	25
27.29758	29.37127	19.13677	26.37117	30.75
53.81419	29.39173	65.4732	26.39163	37.75
58.33672	29.41219	26.81493	26.41209	44
68.86514	29.43266	49.16197	26.43256	55.25
88.39948	29.45312	72.51431	26.45302	72.25
47.93971	29.47358	93.87196	26.47348	92.75
115.4858	29.49405	58.23491	26.49394	118.5
81.0379	29.51451	45.60316	26.51441	161.75
99.59584	29.53497	-3.02329	26.53487	236.25
120.1597	29.55543	60.35557	26.55533	371.25
142.7294	29.5759	73.73973	26.5758	569.5
195.3051	29.59636	110.1292	26.59626	808.75
226.8867	29.61682	61.52396	26.61672	1050.75
293.4741	29.63728	100.924	26.63718	1248
387.0675	29.65775	141.3294	26.65765	1380.75
419.6668	29.67821	181.7401	26.67811	1405
456.2719	29.69867	136.1561	26.69857	1312
392.883	29.71914	206.5773	26.71904	1127
358.5	29.7396	244.0039	26.7395	887
282.1229	29.76006	304.4358	26.75996	652

179.7517	29.78052	361.873	26.78042	453.5
182.3864	29.80099	385.3155	26.80089	301
131.027	29.82145	392.7633	26.82135	193.5
84.67351	29.84191	381.2164	26.84181	125.5
82.32592	29.86238	247.6748	26.86227	96.5
56.98423	29.88284	253.1385	26.88274	80.75
16.64845	29.9033	188.6075	26.9032	72.5
-1.68143	29.92376	142.0818	26.92366	68.5
68.99459	29.94423	140.5615	26.94413	61.5
15.67652	29.96469	138.0464	26.96459	51.5
51.36435	29.98515	81.53661	26.98505	44
70.05809	30.00561	124.0321	27.00551	36.5
-1.24227	30.02608	84.53296	27.02598	29
72.46327	30.04654	59.03909	27.04644	22.75
18.17472	30.067	99.55053	27.0669	18
23.89207	30.08747	79.06727	27.08737	15.25
74.61533	30.10793	75.58931	27.10783	13.25
103.3445	30.12839	43.11665	27.12829	11.25
22.07955	30.14885	91.6493	27.14875	8.75
77.82052	30.16932	56.18725	27.16922	6
131.5674	30.18978	65.73051	27.18968	3.75
29.32016	30.21024	60.27906	27.21014	2.75
65.07884	30.23071	54.83293	27.2306	2.25
144.8434	30.25117	84.39209	27.25107	0.5
38.61391	30.27163	53.95656	27.27153	2.75
74.3903	30.29209	103.5263	27.29199	1.75
111.1726	30.31256	105.1014	27.31246	3.5
156.9608	30.33302	72.68178	27.33292	4.5
99.75489	30.35348	104.2675	27.35338	4.75
200.5549	30.37394	135.8584	27.37384	4.75
170.3608	30.39441	159.4547	27.39431	4
289.1726	30.41487	301.0563	27.41477	3
257.9903	30.43533	232.6632	27.43523	2.5
317.814	30.4558	306.2754	27.4557	1.25
291.6435	30.47626	371.8929	27.47616	1.5
410.4789	30.49672	460.5157	27.49662	0.5
428.3203	30.51718	413.1438	27.51708	2.75
515.1675	30.53765	454.7772	27.53755	5.25
570.0206	30.55811	539.4159	27.55801	5.75
712.8797	30.57857	501.0599	27.57847	8.25
843.7446	30.59904	532.7092	27.59893	9.5
947.6155	30.6195	630.3639	27.6194	11
991.4922	30.63996	673.0238	27.63986	14.5
967.3749	30.66042	852.689	27.66032	15.25
771.2634	30.68089	966.3596	27.68079	20.25
553.1579	30.70135	1037.035	27.70125	25.5
591.0583	30.72181	1080.717	27.72171	30.5
341.9646	30.74227	925.403	27.74217	37.75
230.8767	30.76274	722.0947	27.76264	48
187.7948	30.7832	640.7917	27.7831	62
177.7188	30.80366	530.4941	27.80356	85.75
134.6487	30.82413	413.2018	27.82403	124.25
86.5845	30.84459	242.9147	27.84449	187.25
75.5262	30.86505	150.633	27.86495	282
80.47381	30.88551	144.3565	27.88541	409
117.4273	30.90598	125.0854	27.90588	534.5
76.38672	30.92644	115.8195	27.92634	642.75
84.35204	30.9469	100.559	27.9468	717.25

4.323255	30.96737	87.30379	27.96726	745.75
50.30038	30.98783	8.053862	27.98773	718
22.2834	31.00829	56.80924	28.00819	637.25
65.27233	31.02875	68.56992	28.02865	520.5
10.26717	31.04922	27.33591	28.04912	396
53.2679	31.06968	29.1072	28.06958	288.75
19.27455	31.09014	18.88379	28.09004	205.5
55.28709	31.1106	29.66568	28.1105	136
33.30554	31.13107	96.45288	28.13097	90.75
0.329893	31.15153	64.24538	28.15143	61.25
62.36015	31.17199	43.04319	28.17189	48
-4.60369	31.19246	32.84629	28.19236	42
29.43838	31.21292	58.65471	28.21282	33.25
83.48634	31.23338	43.46842	28.23328	27
38.54022	31.25384	80.28743	28.25374	22.5
37.6	31.27431	53.11175	28.27421	19.5
83.66567	31.29477	44.94138	28.29467	18.25
9.737258	31.31523	-1.2237	28.31513	15.5
40.81475	31.3357	30.61653	28.33559	15.75
81.89814	31.35616	40.46206	28.35606	14.25
56.98743	31.37662	11.3129	28.37652	16.25
34.08263	31.39708	59.16904	28.39698	19.5
133.1837	31.41755	65.03048	28.41745	24.25
133.2907	31.43801	90.89722	28.43791	29
161.4037	31.45847	65.76927	28.45837	36.75
165.5225	31.47893	86.64662	28.47883	41.5
137.6472	31.4994	97.52927	28.4993	54.25
233.7778	31.51986	136.4172	28.51976	67.5
226.9143	31.54032	156.3105	28.54022	88.5
190.0568	31.56079	183.2091	28.56068	120.5
173.2051	31.58125	184.1129	28.58115	181.25
147.3593	31.60171	208.0221	28.60161	283.5
96.51949	31.62217	191.9366	28.62207	439.75
105.6855	31.64264	205.8563	28.64254	638.25
75.85748	31.6631	241.7814	28.663	856
43.03534	31.68356	175.7118	28.68346	1042.25
22.21909	31.70403	125.6475	28.70392	1168.25
40.40876	31.72449	87.58848	28.72439	1206.75
72.60432	31.74495	52.53477	28.74485	1154.25
82.80579	31.76541	94.48636	28.76531	1016.25
56.01317	31.78588	74.44326	28.78578	824.75
41.22644	31.80634	51.40546	28.80624	622
-29.5544	31.8268	31.37296	28.8267	439
78.67071	31.84726	51.34577	28.84716	299.25
72.9017	31.86773	51.32388	28.86763	200.25
70.13859	31.88819	-11.6927	28.88809	129
94.38139	31.90865	41.29601	28.90855	83.25
106.6301	31.92912	73.29003	28.92901	60.75
74.88469	31.94958	52.28935	28.94948	49
158.1452	31.97004	30.29398	28.96994	41.5
202.4116	31.9905	94.30391	28.9904	34.5
283.6839	32.01097	74.31914	29.01087	31.25
411.9622	32.03143	126.3397	29.03133	25.25
647.2463	32.05189	137.3655	29.05179	22.5
741.5363	32.07236	145.3967	29.07225	17.75
790.8322	32.09282	104.4331	29.09272	14
652.1341	32.11328	142.4748	29.11318	11.5
642.4418	32.13374	148.5219	29.13364	9.5

654.7554	32.15421	224.5742	29.15411	5.25
629.075	32.17467	211.6319	29.17457	3
616.4005	32.19513	288.6949	29.19503	2.75
528.7318	32.21559	193.7631	29.21549	3.75
529.069	32.23606	316.8367	29.23596	4
398.4122	32.25652	272.9156	29.25642	5.25
326.7613	32.27698	296.9997	29.27688	5.5
317.1163	32.29745	285.0892	29.29734	5.5
256.4771	32.31791	200.184	29.31781	4.25
247.8439	32.33837	219.2841	29.33827	2.5
184.2166	32.35883	155.3895	29.35873	1
148.5952	32.3793	148.5002	29.3792	1.25
176.9797	32.39976	143.6161	29.39966	2.5
142.3701	32.42022	119.7374	29.42012	2.25
119.7664	32.44069	79.86403	29.44058	2.5
103.1686	32.46115	73.99593	29.46105	3.25
142.5767	32.48161	67.13313	29.48151	4
78.99068	32.50207	100.2756	29.50197	4.25
38.41059	32.52254	84.42345	29.52244	5.25
76.8364	32.543	70.57656	29.5429	5
35.26813	32.56346	95.73498	29.56336	5.25
46.70575	32.58392	83.8987	29.58382	6.5
34.14928	32.60439	114.0677	29.60429	6.5
23.59871	32.62485	8.242047	29.62475	9
62.05405	32.64531	-9.57832	29.64521	10.75
36.51529	32.66578	15.60661	29.66567	15.75
-12.0176	32.68624	-35.2032	29.68614	22.5
51.45548	32.7067	-9.00762	29.7066	28.75
-2.06557	32.72716	10.19322	29.72706	37.75
-24.5807	32.74763	38.39936	29.74753	49.25
17.91005	32.76809	-7.38919	29.76799	67.5
60.40671	32.78855	28.82756	29.78845	96
61.90928	32.80902	-1.95038	29.80891	144
-9.58225	32.82948	-13.723	29.82938	220
-5.06788	32.84994	17.50963	29.84984	323
-23.5476	32.8704	-4.2524	29.8703	443.75
39.97858	32.89087	22.99086	29.89077	554
64.51067	32.91133	-18.7606	29.91123	640.25
60.04866	32.93179	-2.5067	29.93169	685.5
40.59256	32.95225	-0.24752	29.95215	688.75
-31.8576	32.97272	-15.983	29.97262	637.25
-28.3019	32.99318	-0.71326	29.99308	544
20.92645	33.01364	4.561824	30.01354	431.5
31.16075	33.03411	-51.1578	30.034	323
0.400947	33.05457	29.1279	30.05447	234.25
27.64705	33.07503	45.4189	30.07493	161.5
44.89906	33.09549	13.7152	30.09539	106.5
84.15698	33.11596	39.0168	30.11586	71.25
-46.5792	33.13642	-6.6763	30.13632	48
-11.3095	33.15688	-1.3641	30.15678	38.5
15.96613	33.17735	-8.04659	30.17724	31.25
25.24766	33.19781	-3.72377	30.19771	24.75
50.53509	33.21827	1.604344	30.21817	18.5
9.828426	33.23873	35.93776	30.23863	18.25
49.12767	33.2592	43.27649	30.2591	18
53.43281	33.27966	29.62051	30.27956	14.5
64.74386	33.30012	-9.03016	30.30002	14.75
9.060807	33.32058	-37.6755	30.32048	13.75

66.38366	33.34105	36.68441	30.34095	11.5
65.71242	33.36151	4.049643	30.36141	12.75
40.04709	33.38197	27.42018	30.38187	14.25
37.38765	33.40244	8.796027	30.40233	15.5
0.73412	33.4229	59.17717	30.4228	15
48.08649	33.44336	59.56363	30.44326	18.25
24.44477	33.46382	47.95538	30.46372	19.75
33.80896	33.48429	34.35244	30.48419	24.5
1.17904	33.50475	35.7548	30.50465	31.5
14.55503	33.52521	56.16246	30.52511	47
32.93692	33.54568	19.57542	30.54557	67
60.32472	33.56614	38.99369	30.56604	91.5
44.71842	33.5866	-51.5827	30.5865	116
-5.88197	33.60706	17.84614	30.60696	141.25
60.52354	33.62753	44.28032	30.62743	162
67.93495	33.64799	-62.2802	30.64789	179.25
39.35227	33.66845	31.16458	30.66835	184.5
49.77549	33.68891	9.614671	30.68881	191.5
87.20461	33.70938	22.07006	30.70928	202
145.6396	33.72984	54.53075	30.72974	244.5
140.0806	33.7503	99.99675	30.7502	316.25
164.5274	33.77077	51.46805	30.77066	440.25
209.9801	33.79123	100.9447	30.79113	625.25
236.4388	33.81169	111.4266	30.81159	833
350.9034	33.83215	214.9138	30.83205	1023.75
459.3738	33.85262	219.4063	30.85252	1165.5
639.8502	33.87308	220.9041	30.87298	1231.75
779.3324	33.89354	241.4072	30.89344	1220.25
959.8206	33.91401	355.9156	30.9139	1123
1087.315	33.93447	505.4294	30.93437	961.5
1228.815	33.95493	546.9484	30.95483	761
1064.32	33.97539	697.4727	30.97529	571.75
974.8323	33.99586	843.0024	30.99576	418.5
726.35	34.01632	841.5373	31.01622	291.5
612.8735	34.03678	899.0775	31.03668	193.75
499.403	34.05724	773.6231	31.05714	125.25
362.9384	34.07771	677.174	31.07761	84
198.4797	34.09817	565.7301	31.09807	64
155.0269	34.11863	483.2915	31.11853	52.75
101.58	34.1391	354.8583	31.13899	42.25
50.13903	34.15956	342.4304	31.15946	33.25
61.70395	34.18002	172.0077	31.17992	27
79.27477	34.20048	42.59038	31.20038	23.75
20.85149	34.22095	72.17835	31.22085	20.25
42.43411	34.24141	25.77162	31.24131	18
53.02264	34.26187	29.37019	31.26177	11.75
42.61708	34.28234	30.97407	31.28223	8.5
23.21741	34.3028	43.58325	31.3027	4.25
24.82365	34.32326	19.19774	31.32316	3.5
-6.5642	34.34372	-5.18248	31.34362	3.75
15.05385	34.36419	2.442614	31.36409	4
20.6778	34.38465	-18.927	31.38455	5.5
37.30766	34.40511	3.708704	31.40501	6.5
-8.05658	34.42557	20.34971	31.42547	7.25
14.58508	34.44604	-5.00399	31.44594	7.5
14.23265	34.4665	13.64761	31.4664	6
20.88612	34.48696	12.30452	31.48686	5.75
23.5455	34.50743	23.96674	31.50732	2.5

12.21078	34.52789	-10.3657	31.52779	0.5
50.88196	34.54835	12.30707	31.54825	0.5
59.55905	34.56881	34.98519	31.56871	3
53.24204	34.58928	-11.3314	31.58918	6
1.930936	34.60974	25.35734	31.60964	12
-7.37427	34.6302	-36.9486	31.6301	21.75
43.32644	34.65067	10.75071	31.65056	31.5
-41.967	34.67113	14.45535	31.67103	56.5
33.74556	34.69159	-34.8347	31.69149	88.75
14.46397	34.71205	7.880531	31.71195	130.75
11.18829	34.73252	8.601078	31.73242	173.75
45.91851	34.75298	33.32693	31.75288	212.25
58.65464	34.77344	-35.9419	31.77334	239.75
83.39667	34.7939	-27.2055	31.7938	251.5
36.1446	34.81437	37.5363	31.81427	243.75
91.89844	34.83483	97.28336	31.83473	216.25
70.65818	34.85529	105.0357	31.85519	174.75
194.4238	34.87576	132.7934	31.87565	136.5
187.1954	34.89622	123.5564	31.89612	97.5
275.9728	34.91668	142.3246	31.91658	68
402.7562	34.93714	104.0982	31.93704	43.75
444.5454	34.95761	127.8771	31.95751	25.5
528.3406	34.97807	264.6613	31.97797	15
671.1417	34.99853	268.4508	31.99843	10.5
663.9487	35.019	306.2455	32.01889	6.75
464.7615	35.03946	434.0457	32.03936	4.75
469.5803	35.05992	477.851	32.05982	3
397.405	35.08038	511.6617	32.08028	1.5
329.2356	35.10085	471.4778	32.10075	0
241.0721	35.12131	364.2991	32.12121	0.5
160.9145	35.14177	292.1257	32.14167	3.25
108.7628	35.16223	283.9576	32.16213	5.5
69.61698	35.1827	271.7948	32.1826	9.5
86.47709	35.20316	137.6373	32.20306	13.5
-11.6569	35.22362	109.4851	32.22352	18.25
26.21501	35.24409	144.3383	32.24398	27
14.09283	35.26455	92.19669	32.26445	37
38.97656	35.28501	7.060419	32.28491	46.75
51.86618	35.30547	85.92945	32.30537	61.25
21.76171	35.32594	-23.1962	32.32584	86.5
51.66315	35.3464	1.68343	32.3463	132.25
0.570487	35.36686	-13.4316	32.36676	205.25
45.48373	35.38733	16.45862	32.38722	302.5
3.402876	35.40779	41.35417	32.40769	411.25
61.32793	35.42825	-27.745	32.42815	506.75
33.25888	35.44871	-27.8388	32.44861	576.75
30.19574	35.46918	9.848482	32.46908	611.5
52.1385	35.48964	11.54109	32.48954	602.5
79.08717	35.5101	-34.761	32.51	554
19.04174	35.53056	88.94222	32.53046	476.75
118.0022	35.55103	44.65073	32.55093	391.75
139.9686	35.57149	31.36455	32.57139	314.25
151.9409	35.59195	73.08367	32.59185	257.25
89.91905	35.61242	82.8081	32.61231	216.75
105.9031	35.63288	83.53783	32.63278	185.5
86.89314	35.65334	61.27286	32.65324	162.25
83.88903	35.6738	82.01319	32.6737	150
120.8908	35.69427	91.75884	32.69417	139

109.8985	35.71473	29.50978	32.71463	126
88.91215	35.73519	67.26602	32.73509	110.75
-9.06834	35.75566	39.02757	32.75555	91.75
32.95708	35.77612	73.79442	32.77602	68.75
-16.0116	35.79658	31.56657	32.79648	52.5
27.02562	35.81704	1.344029	32.81694	36
16.06875	35.83751	8.126789	32.83741	25.25
-10.8822	35.85797	-4.08515	32.85787	17.75
46.17271	35.87843	8.708219	32.87833	14.25
23.23356	35.89889	4.506888	32.89879	11
20.3003	35.91936	-44.6891	32.91926	8.5
-26.6271	35.93982	-1.87986	32.93972	7.75
-70.7517	35.96028	54.93472	32.96018	7
50.12957	35.98075	-19.2454	32.98064	5
-20.9833	36.00121	-11.4202	33.00111	2.75
84.90981	36.02167	-23.5897	33.02157	0.5
13.80879	36.04213	6.246063	33.04203	1
-5.28633	36.0626	4.087157	33.0625	2.5
10.62445	36.08306	-30.0664	33.08296	2.75
-13.4589	36.10352	22.78526	33.10342	2.5
19.46373	36.12399	12.64226	33.12388	1.5
33.39223	36.14445	-38.4954	33.14435	1.5
-10.6734	36.16491	-11.7015	33.16481	1.75
42.26693	36.18537	11.09775	33.18527	1.25
7.213138	36.20584	13.9023	33.20574	0.75
-28.8348	36.2263	20.71215	33.2262	1.5
1.123264	36.24676	-25.4727	33.24666	1.5
13.08718	36.26722	19.34776	33.26712	1.25
9.057006	36.28769	-3.82648	33.28759	0.25
25.03273	36.30815	-34.9954	33.30805	0
40.01436	36.32861	4.062215	33.32851	0.5
5.001897	36.34908	1.125147	33.34897	1.5
-9.00466	36.36954	18.19338	33.36944	2.75
56.99468	36.39	9.266919	33.3899	2.75
-22.0001	36.41046	-23.6542	33.41036	3.75
-2.98893	36.43093	53.42991	33.43083	8.25
-0.97187	36.45139	-25.4806	33.45129	16.25
66.05109	36.47185	24.6141	33.47175	24.5
14.07995	36.49232	20.71416	33.49221	34.75
40.11471	36.51278	16.81951	33.51268	43.5
-26.8446	36.53324	36.93017	33.53314	51.75
49.20195	36.5537	9.046136	33.5536	55
112.2544	36.57417	92.1674	33.57407	55.5
84.31281	36.59463	3.293971	33.59453	50.75
108.3771	36.61509	25.42584	33.61499	43.25
60.44729	36.63555	77.56302	33.63545	36.5
19.52338	36.65602	41.7055	33.65592	28.5
111.6054	36.67648	87.85328	33.67638	20.25
104.6933	36.69694	88.00636	33.69684	14
30.78708	36.71741	97.16475	33.7173	7.75
57.88679	36.73787	76.32845	33.73777	4
-6.0076	36.75833	87.49744	33.75823	6
-5.89609	36.77879	128.6717	33.77869	9.75
40.22134	36.79926	55.85133	33.79916	13.75
-16.6553	36.81972	67.03624	33.81962	15.75
55.47389	36.84018	134.2264	33.84008	19.75
94.60902	36.86065	3.421955	33.86054	23
44.75005	36.88111	104.6228	33.88101	26

36.89699	36.90157	67.82888	33.90147	30.25
-10.9502	36.92203	3.040303	33.92193	35.75
19.20858	36.9425	108.257	33.9424	41
112.3732	36.96296	29.47905	33.96286	53.5
113.5438	36.98342	123.7064	33.98332	65.75
113.7202	37.00388	52.93901	34.00378	80.75
115.9026	37.02435	140.1769	34.02425	106.5
179.0909	37.04481	121.4202	34.04471	149
165.285	37.06527	159.6687	34.06517	223.25
205.4851	37.08574	226.9226	34.08564	340.75
248.6911	37.1062	241.1817	34.1061	506.5
306.903	37.12666	270.4462	34.12656	689.5
375.1208	37.14712	316.7159	34.14702	854.25
375.3445	37.16759	265.991	34.16748	979
417.574	37.18805	352.2713	34.18795	1040.75
516.8096	37.20851	392.557	34.20841	1039
472.0509	37.22898	502.848	34.22887	976.5
500.2982	37.24944	373.1442	34.24934	851.5
350.5515	37.2699	535.4458	34.2698	700.25
348.8106	37.29036	489.7527	34.29026	551.5
397.0756	37.31083	544.0649	34.31073	423.25
369.3465	37.33129	480.3824	34.33119	314.25
524.6234	37.35175	470.7051	34.35165	218.5
459.9061	37.37221	413.0332	34.37211	143.75
465.1947	37.39268	392.3666	34.39258	94.5
428.4892	37.41314	421.7053	34.41304	69
438.7897	37.4336	423.0493	34.4335	59.75
359.096	37.45407	427.3986	34.45396	49.25
299.4083	37.47453	449.7532	34.47443	42
250.7264	37.49499	354.1131	34.49489	35.25
258.0505	37.51545	415.4783	34.51535	29
177.3804	37.53592	330.8488	34.53582	25
136.7163	37.55638	311.2246	34.55628	18.75
93.05806	37.57684	233.6058	34.57674	15.75
34.40573	37.59731	206.9922	34.59721	13.25
54.7593	37.61777	208.3839	34.61767	10
3.118781	37.63823	127.7809	34.63813	8.25
9.484163	37.65869	117.1833	34.65859	5.75
32.85545	37.67916	94.5909	34.67905	3.5
40.23264	37.69962	30.00384	34.69952	1.75
-9.38427	37.72008	45.42208	34.71998	3
-66.9953	37.74054	23.84563	34.74044	3
10.22177	37.76101	65.27447	34.76091	2.75
-41.5553	37.78147	84.70862	34.78137	4.5
6.673561	37.80193	16.14808	34.80183	4.25
46.90831	37.8224	0.592831	34.8223	5.25
-40.851	37.84286	12.04289	34.84276	5.5
35.39552	37.86332	18.49825	34.86322	4.5
13.64799	37.88378	20.95892	34.88368	2.75
-33.0936	37.90425	1.424887	34.90414	2
49.17062	37.92471	21.89616	34.92461	5
28.4408	37.94517	57.37273	34.94507	6.5
53.71687	37.96564	42.85461	34.96553	10.5
5.998855	37.9861	-12.6582	34.986	18
46.28674	38.00656	-14.1657	35.00646	23.5
43.58053	38.02702	91.33206	35.02692	31.5
37.88022	38.04749	20.83516	35.04739	38
71.18582	38.06795	109.3436	35.06785	48

104.4973	38.08841	101.8572	35.08831	66.5
93.81473	38.10887	132.3763	35.10877	95.25
118.138	38.12934	80.90055	35.12924	144
151.4672	38.1498	107.4302	35.1497	212
119.8024	38.17026	187.9651	35.17016	291
168.1434	38.19073	130.5053	35.19062	363.5
129.4903	38.21119	206.0508	35.21109	416.75
102.8431	38.23165	218.6016	35.23155	445
149.2019	38.25211	177.1577	35.25201	445.75
94.56651	38.27258	212.7192	35.27248	418.5
146.937	38.29304	199.2859	35.29294	369.5
33.31349	38.3135	161.8579	35.3134	306.25
6.695834	38.33397	146.4353	35.33387	242.25
23.08409	38.35443	164.0179	35.35433	187
34.47824	38.37489	99.60583	35.37479	138.25
29.8783	38.39535	49.19907	35.39525	95
32.28426	38.41582	55.79762	35.41571	61.75
16.69613	38.43628	27.40147	35.43618	39.25
28.1139	38.45674	64.01062	35.45664	26.75
12.53758	38.4772	21.62508	35.4771	19
17.96715	38.49767	5.244839	35.49757	16.75
70.40263	38.51813	40.8699	35.51803	14
26.84402	38.53859	74.50027	35.53849	10.25
65.29131	38.55906	46.13594	35.55896	9.75
41.74451	38.57952	31.77691	35.57942	9.5
68.20361	38.59998	40.42318	35.59988	8.25
65.6686	38.62044	46.07476	35.62034	9
122.1395	38.64091	65.73164	35.6408	10.5
149.6163	38.66137	154.3938	35.66127	12
175.099	38.68183	65.06131	35.68173	17
220.5876	38.7023	53.7341	35.70219	23.75
260.0822	38.72276	132.4122	35.72266	35
286.5826	38.74322	173.0956	35.74312	53.75
314.0889	38.76368	135.7843	35.76358	81.25
341.6012	38.78415	186.4783	35.78405	111
351.1193	38.80461	175.1776	35.80451	135.75
308.6433	38.82507	189.8822	35.82497	153.5
274.1733	38.84553	167.5921	35.84543	163.5
257.7091	38.866	200.3073	35.8659	162.75
239.2509	38.88646	153.0279	35.88636	152.25
228.7985	38.90692	169.7537	35.90682	131.5
215.3521	38.92739	113.4848	35.92728	108.25
162.9116	38.94785	75.22123	35.94775	84.5
134.4769	38.96831	133.963	35.96821	65.5
116.0482	38.98877	142.71	35.98867	47.75
79.62537	39.00924	120.4623	36.00914	31.5
82.20845	39.0297	95.21997	36.0296	19.5
13.79744	39.05016	80.98292	36.05006	14
21.39232	39.07063	126.7512	36.07053	9.5
8.993114	39.09109	113.5247	36.09099	7.25
98.59981	39.11155	87.30357	36.11145	6
53.21241	39.13201	47.08772	36.13191	4
1.830911	39.15248	14.87718	36.15237	3.25
1.455317	39.17294	58.67194	36.17284	3
3.085628	39.1934	71.47201	36.1933	3
49.72184	39.21386	27.27738	36.21376	3.5
2.36396	39.23433	8.088049	36.23423	4
13.01198	39.25479	-26.096	36.25469	4.25

47.66591	39.27525	-24.9724	36.27515	3.75
23.32574	39.29572	4.1564	36.29562	2.75
30.99147	39.31618	29.29054	36.31608	2.5
0.663108	39.33664	-14.57	36.33654	1.5
-40.6594	39.3571	41.57474	36.357	2.25
12.0241	39.37757	-7.27521	36.37746	2.5
16.71344	39.39803	-44.1199	36.39793	3.25
41.4087	39.41849	12.04081	36.41839	3.25
37.10985	39.43896	15.20677	36.43885	3
14.81692	39.45942	41.37803	36.45932	1.5
-4.47012	39.47988	13.5546	36.47978	1.75
41.24875	39.50034	-10.2635	36.50024	0.75
-16.0265	39.52081	-3.07635	36.52071	2.25
27.7042	39.54127	5.116123	36.54117	2
10.44078	39.56173	-34.6861	36.56163	3
-21.8167	39.58219	30.51699	36.58209	6
70.93165	39.60266	13.72537	36.60256	7.75
-38.3141	39.62312	13.93906	36.62302	9.5
-25.5539	39.64358	37.15806	36.64348	10.75
2.651271	39.66405	8.382353	36.66394	11
-10.1377	39.68451	31.61195	36.68441	11.5
-5.92075	39.70497	11.84686	36.70487	13.25
-46.6979	39.72543	35.08706	36.72533	16
11.53085	39.7459	9.332569	36.7458	21.5
12.76551	39.76636	19.58338	36.76626	33.75
25.00607	39.78682	27.8395	36.78672	48.25
19.25253	39.80729	31.10091	36.80719	62
-27.4951	39.82775	27.36763	36.82765	72.5
-18.9907	39.84821	11.63966	36.84811	77.75
4.519639	39.86867	37.91699	36.86857	78.5
32.03587	39.88914	38.19962	36.88903	72.25
24.558	39.9096	10.48755	36.9095	64.5
6.086028	39.93006	-11.2192	36.92996	51.5
72.61997	39.95052	48.07933	36.95042	41.25
30.15981	39.97099	41.38317	36.97089	32.75
-8.29445	39.99145	28.69232	36.99135	27.25
-5.7428	40.01191	-1.99324	37.01181	21
45.81475	40.03238	91.32652	37.03228	16
24.37821	40.05284	21.65157	37.05274	13.25
7.947573	40.0733	-24.0181	37.0732	13.75
26.52284	40.09376	3.317592	37.09366	14.5
30.10401	40.11423	9.658557	37.11412	16.75
16.69108	40.13469	-37.9952	37.13459	18.25
-52.7159	40.15515	-9.32964	37.15505	21.5
70.88294	40.17562	18.34121	37.17551	26.25
35.48772	40.19608	22.01735	37.19598	32
66.0984	40.21654	3.698799	37.21644	35
48.715	40.237	7.38555	37.2369	43.75
44.33749	40.25747	46.07761	37.25737	53
36.96589	40.27793	27.77496	37.27783	69.25
26.6002	40.29839	55.47762	37.29829	98
16.2404	40.31885	25.18559	37.31875	143.5
33.88652	40.33932	12.89885	37.33922	219.25
38.53853	40.35978	53.61742	37.35968	316
50.19645	40.38024	62.3413	37.38014	417.5
86.86028	40.40071	105.0705	37.4006	503.5
92.53	40.42117	33.80495	37.42107	561.75
75.20563	40.44163	92.54473	37.44153	591.25

59.88717	40.46209	93.28982	37.46199	589
42.5746	40.48256	114.0402	37.48246	563.75
9.267945	40.50302	61.7959	37.50292	528.75
15.96719	40.52348	49.5569	37.52338	503.75
68.67234	40.54395	25.3232	37.54385	515.25
52.3834	40.56441	-8.90521	37.56431	551.75
-8.89965	40.58487	35.8717	37.58477	599.25
42.82322	40.60533	53.65391	37.60523	634.25
19.55198	40.6258	-4.55858	37.62569	648.75
-18.7134	40.64626	10.23423	37.64616	640.5
8.027223	40.66672	28.03235	37.66662	614.5
24.7737	40.68718	-2.16423	37.68708	561.5
3.526081	40.70765	48.64449	37.70755	490.5
-13.7156	40.72811	18.45852	37.72801	408.5
-48.9514	40.74857	6.277846	37.74847	332.25
31.81865	40.76904	2.102479	37.76894	264.75
60.59464	40.7895	48.93242	37.7894	206
43.37655	40.80996	42.76765	37.80986	146.5
-14.8357	40.83042	20.60819	37.83032	99.25
28.95806	40.85089	47.45404	37.85078	65.25
-0.24233	40.87135	-7.69481	37.87125	46.75
11.56319	40.89181	26.16164	37.89171	33.75
76.3746	40.91228	47.02339	37.91217	28.75
-5.80807	40.93274	25.89045	37.93264	22
-3.98484	40.9532	73.76281	37.9531	18
-4.15571	40.97366	36.64047	37.97356	16.5
7.679322	40.99413	13.52344	37.99403	15.5
21.52026	41.01459	6.411711	38.01449	13.75
-19.6329	41.03505	2.305284	38.03495	15.25
7.219851	41.05551	-17.7958	38.05541	14.5
-8.9215	41.07598	-30.8917	38.07588	13
38.94306	41.09644	52.01782	38.09634	11
27.81352	41.1169	36.93261	38.1168	11
-2.31012	41.13737	9.852695	38.13726	10.25
-4.42786	41.15783	28.77809	38.15773	9.75
-6.53969	41.17829	45.70878	38.17819	11.5
59.35439	41.19875	4.644779	38.19865	15.25
6.254367	41.21922	35.58608	38.21912	22
-43.8398	41.23968	67.53268	38.23958	28.75
20.07204	41.26014	3.484592	38.26004	36.75
-14.0103	41.28061	6.441802	38.28051	51.5
37.91332	41.30107	-27.5957	38.30097	76.75
-0.15719	41.32153	-2.62787	38.32143	112.75
-9.22179	41.34199	54.34525	38.34189	156.75
-12.2805	41.36246	39.32367	38.36235	200
2.66673	41.38292	-16.6926	38.38282	236.25
26.61984	41.40338	4.29643	38.40328	260.25
17.57886	41.42384	-22.7092	38.42374	271.25
53.54378	41.44431	-18.7096	38.44421	261.75
41.51461	41.46477	22.29534	38.46467	238.75
5.491333	41.48523	-30.6944	38.48513	206.25
61.47397	41.5057	-12.6789	38.5056	171.25
37.4625	41.52616	-22.658	38.52606	139.5
27.45694	41.54662	28.36812	38.54652	111
75.45728	41.56708	28.39957	38.56698	83.5
82.46353	41.58755	65.43633	38.58744	60.5
90.47569	41.60801	17.47839	38.60791	41.25
69.49374	41.62847	49.52576	38.62837	27.25

102.5177	41.64894	49.57842	38.64883	18.75
132.5476	41.6694	54.63639	38.6693	14.5
151.5833	41.68986	68.69966	38.68976	11.75
110.625	41.71032	40.76824	38.71022	10.75
66.67258	41.73079	114.8421	38.73069	11.25
81.72605	41.75125	74.9213	38.75115	13.5
85.78544	41.77171	58.00579	38.77161	17.75
34.85072	41.79217	84.09557	38.79207	21.75
73.92191	41.81264	108.1907	38.81254	25.75
68.99901	41.8331	109.2911	38.833	32.5
40.08201	41.85356	53.39676	38.85346	47
-14.8291	41.87403	57.50776	38.87392	70.25
47.26572	41.89449	19.62406	38.89439	101.75
24.36643	41.91495	72.74567	38.91485	138.5
22.47304	41.93541	3.87258	38.93531	169.25
25.58556	41.95588	-42.9952	38.95578	197.5
2.703977	41.97634	-2.85193	38.97624	213.25
35.8283	41.9968	43.29665	38.9967	219
48.95853	42.01727	-35.5495	39.01717	212
41.09467	42.03773	-10.3903	39.03763	201
66.2367	42.05819	-2.2258	39.05809	190.75
75.38464	42.07865	-7.05601	39.07855	183.75
69.53849	42.09912	41.11908	39.09901	181.5
75.69824	42.11958	-28.7005	39.11948	179.5
77.86389	42.14004	7.485179	39.13994	169.75
76.03545	42.1605	-12.3238	39.1604	157.5
147.2129	42.18097	-10.1275	39.18087	144.25
38.39627	42.20143	-25.9259	39.20133	131.5
74.58553	42.22189	69.28101	39.22179	114.75
72.78071	42.24236	34.49323	39.24226	99.75
94.98178	42.26282	5.710744	39.26272	86
78.18877	42.28328	36.93357	39.28318	70.5
53.40165	42.30374	22.16169	39.30364	59.5
93.62044	42.32421	49.39512	39.3241	46.25
79.84512	42.34467	7.633849	39.34457	31
37.07572	42.36513	-33.1221	39.36503	18.75
28.31222	42.3856	27.12722	39.38549	11.5
41.55462	42.40606	82.38186	39.40596	8
157.8029	42.42652	88.64181	39.42642	6
117.0571	42.44698	34.90705	39.44688	3.5
188.3173	42.46745	42.1776	39.46735	2.5
273.5833	42.48791	63.45345	39.48781	2
386.8552	42.50837	17.73461	39.50827	3.5
500.133	42.52883	91.02107	39.52873	4.5
566.4167	42.5493	126.3128	39.5492	5
623.7064	42.56976	147.6099	39.56966	5.25
575.002	42.59022	201.9123	39.59012	4.5
545.3033	42.61069	180.2199	39.61058	6.75
488.6107	42.63115	241.5329	39.63105	6.25
476.924	42.65161	184.8512	39.65151	5.5
392.2431	42.67207	284.1748	39.67197	4.25
398.5682	42.69254	247.5036	39.69244	3.75
265.8991	42.713	228.8378	39.7129	3.5
258.236	42.73346	168.1773	39.73336	3.5
197.5788	42.75393	182.5221	39.75383	3
135.9274	42.77439	191.8722	39.77429	1.5
172.282	42.79485	136.2276	39.79475	0
149.6425	42.81531	128.5883	39.81521	1

118.0089	42.83578	127.9543	39.83567	0.75
106.3812	42.85624	126.3256	39.85614	1.25
140.7594	42.8767	161.7023	39.8766	1.5
185.1434	42.89716	185.0842	39.89706	1.5
183.5335	42.91763	168.4714	39.91753	2.25
285.9294	42.93809	173.8639	39.93799	2.75
317.3312	42.95855	220.2617	39.95845	3
410.7389	42.97902	256.6649	39.97892	2.5
492.1525	42.99948	311.0733	39.99938	1.75
745.572	43.01994	422.487	40.01984	2.5
799.9974	43.0404	372.9061	40.0403	2
1031.429	43.06087	423.3304	40.06076	1
1082.866	43.08133	558.7601	40.08123	0
1169.309	43.10179	537.195	40.10169	0.25
1124.758	43.12226	624.6353	40.12215	1
926.2131	43.14272	748.0808	40.14262	2
997.674	43.16318	717.5316	40.16308	2.75
882.1407	43.18364	742.9878	40.18354	3
954.6133	43.20411	694.4493	40.20401	2.75
938.0919	43.22457	646.916	40.22447	2.5
832.5764	43.24503	608.3881	40.24493	1.75
669.0668	43.26549	589.8655	40.26539	1
583.563	43.28596	587.3481	40.28586	0
525.0652	43.30642	558.8361	40.30632	0.25
473.5733	43.32688	575.3294	40.32678	1
449.0873	43.34735	485.828	40.34724	3
381.6071	43.36781	335.3319	40.36771	5
320.1329	43.38827	343.8411	40.38817	9.75
293.6646	43.40873	253.3556	40.40863	11.25
164.2022	43.4292	224.8754	40.4291	14
114.7457	43.44966	211.4005	40.44956	15.75
105.2951	43.47012	131.9309	40.47002	15.75
97.85043	43.49059	174.4666	40.49049	15.5
74.41164	43.51105	83.00757	40.51095	18.75
21.97875	43.53151	26.55388	40.53141	24.25
85.55177	43.55197	34.1055	40.55187	31.5
36.1307	43.57244	74.66242	40.57233	40
51.71552	43.5929	27.22464	40.5928	50.5
-1.69374	43.61336	69.79216	40.61326	57
3.902891	43.63382	23.36499	40.63372	61
3.505431	43.65429	29.94312	40.65419	61.75
60.11387	43.67475	-16.4734	40.67465	58.25
52.72822	43.69521	78.11529	40.69511	51.5
24.34847	43.71568	4.709331	40.71558	46.75
48.97463	43.73614	43.30867	40.73604	39.5
23.60669	43.7566	16.91332	40.7565	31.5
78.24465	43.77706	29.52327	40.77696	24.75
62.88852	43.79753	38.13852	40.79742	18.5
100.5383	43.81799	69.75908	40.81789	13.75
80.19396	43.83845	47.38494	40.83835	8.5
120.8555	43.85892	89.0161	40.85881	6.25
77.52302	43.87938	91.65257	40.87928	4
129.1964	43.89984	54.29433	40.89974	2.25
107.8757	43.9203	76.9414	40.9202	3.5
154.5609	43.94077	73.59377	40.94067	2
188.252	43.96123	133.2515	40.96113	0.75
115.949	43.98169	96.91444	40.98159	1.25
157.6519	44.00215	104.5827	41.00205	0

126.3607	44.02262	177.2563	41.02252	0.25
132.0754	44.04308	103.9352	41.04298	1.5
81.79604	44.06354	101.6194	41.06344	3.25
73.52255	44.08401	106.3089	41.0839	4
25.25498	44.10447	61.00368	41.10437	4.25
55.99331	44.12493	106.7038	41.12483	3.25
56.73754	44.14539	14.40919	41.14529	2.75
36.48768	44.16586	8.119899	41.16576	2.75
10.24372	44.18632	64.83591	41.18622	2
27.00566	44.20678	28.55723	41.20668	1.75
30.77351	44.22725	7.283844	41.22715	2.25
47.54726	44.24771	18.01576	41.24761	4
9.326916	44.26817	9.752989	41.26807	5.25
115.1125	44.28863	16.49552	41.28853	6.25
60.90394	44.3091	69.24335	41.30899	8.75
68.70131	44.32956	19.99648	41.32946	11.5
86.50458	44.35002	96.75491	41.34992	14
125.3138	44.37048	56.51866	41.37038	15.75
184.1288	44.39095	62.2877	41.39085	15.5
156.9498	44.41141	67.06204	41.41131	15.25
188.7767	44.43187	93.8417	41.43177	12.75
221.6095	44.45234	70.62665	41.45224	10.5
238.4482	44.4728	136.4169	41.4727	8.25
233.2928	44.49326	142.2125	41.49316	6
175.1433	44.51372	137.0133	41.51362	7.25
173.9997	44.53419	187.8195	41.53408	8.25
179.862	44.55465	188.631	41.55455	7.5
99.73022	44.57511	145.4477	41.57501	7.75
101.6043	44.59558	140.2698	41.59547	6.25
122.4844	44.61604	109.0972	41.61594	5.25
115.3703	44.6365	72.92986	41.6364	3.5
84.26212	44.65696	98.76784	41.65686	3.5
34.15984	44.67743	61.61113	41.67733	4.75
38.06348	44.69789	31.45972	41.69779	4.75
64.97302	44.71835	79.31361	41.71825	9.25
67.88847	44.73881	72.17281	41.73871	13.5
24.80982	44.75928	18.0373	41.75918	18.75
49.73707	44.77974	94.9071	41.77964	29.5
-9.32977	44.8002	29.78221	41.8001	45.5
-1.39071	44.82067	-19.3374	41.82056	67.5
-11.4457	44.84113	3.548331	41.84103	89
-39.4949	44.86159	44.43935	41.86149	111.5
11.63797	44.88205	26.33566	41.88195	126.5
46.77673	44.90252	-26.7627	41.90242	133.5
35.92139	44.92298	-4.85579	41.92288	133.5
13.07195	44.94344	23.05644	41.94334	126
36.22842	44.96391	-12.026	41.96381	112
-0.60921	44.98437	-21.1032	41.98427	97
40.55906	45.00483	-11.1751	42.00473	83.25
13.73324	45.02529	-7.24163	42.02519	70.75
34.91332	45.04576	-4.30288	42.04565	55.25
20.09931	45.06622	-33.3588	42.06612	44.25
-9.7088	45.08668	51.59051	42.08658	32.25
-0.51101	45.10714	-2.45484	42.10704	24
26.69269	45.12761	-39.4949	42.12751	15.5
-6.09771	45.14807	3.22425	42.14797	10.5
49.1178	45.16853	4.948683	42.16843	7.75
37.33921	45.189	-23.3216	42.1889	6.25

15.56652	45.20946	35.41346	42.20936	6
56.79974	45.22992	14.1538	42.22982	5.75
105.0389	45.25038	-20.1006	42.25028	3.5
75.28388	45.27085	6.650398	42.27074	3.75
88.53481	45.29131	-24.5933	42.29121	3.25
56.79165	45.31177	21.16821	42.31167	2.25
134.0544	45.33224	88.93507	42.33213	0
84.32302	45.3527	71.70722	42.3526	1.25
15.59757	45.37316	40.48469	42.37306	4.25
64.87801	45.39362	98.26746	42.39352	5.75
22.16437	45.41409	37.05553	42.41399	9.5
79.45663	45.43455	20.8489	42.43445	10.25
58.75478	45.45501	57.64758	42.45491	13.25
65.05885	45.47547	88.45156	42.47537	14
31.36882	45.49594	36.26085	42.49584	14
57.68469	45.5164	52.07543	42.5163	13.75
-15.9935	45.53686	33.89532	42.53676	14
10.33414	45.55733	33.72051	42.55722	18.25
18.66773	45.57779	8.551009	42.57769	19.5
16.00721	45.59825	34.38681	42.59815	24
14.3526	45.61871	-9.77209	42.61861	33.5
52.7039	45.63918	19.07432	42.63908	46.25
-4.9389	45.65964	5.926024	42.65954	66.5
38.4242	45.6801	50.78304	42.68	97.75
44.79321	45.70057	72.64535	42.70047	149
11.16812	45.72103	6.512969	42.72093	210.75
56.54893	45.74149	42.38589	42.74139	270.75
31.93565	45.76195	-1.73589	42.76185	328
24.32827	45.78242	40.14764	42.78231	364.75
45.7268	45.80288	20.03647	42.80278	380.5
-6.86877	45.82334	-41.0694	42.82324	379.75
31.54156	45.8438	9.830041	42.8437	358.25
-4.0422	45.86427	20.73478	42.86417	322.75
19.37994	45.88473	17.64482	42.88463	282.5
-39.192	45.90519	6.560169	42.90509	247.25
25.24193	45.92566	66.48082	42.92556	207.5
-6.31821	45.94612	36.40677	42.94602	173
8.127543	45.96658	26.33802	42.96648	144.5
-46.4208	45.98704	-0.72542	42.98694	113.5
33.03677	46.00751	12.21645	43.0074	88.5
4.500237	46.02797	10.16361	43.02787	79.75
-12.0304	46.04843	-8.88392	43.04833	73.5
-0.55511	46.0689	51.07385	43.06879	72.75
6.926066	46.08936	8.036922	43.08926	76.75
4.413151	46.10982	-23.9947	43.10972	85.75
-17.0939	46.13028	-8.15089	43.13018	99.75
17.40503	46.15075	-17.3018	43.15065	130.25
-23.0902	46.17121	42.55264	43.17111	174.25
2.420526	46.19167	-5.58764	43.19157	246.75
28.93713	46.21213	24.27739	43.21203	358.75
10.45964	46.2326	3.147717	43.2325	496
-51.012	46.25306	8.023347	43.25296	633
5.522363	46.27352	28.90428	43.27342	740.75
-23.9374	46.29399	52.79052	43.29388	809.25
-14.3597	46.31445	1.682058	43.31435	831.5
29.22391	46.33491	18.5789	43.33481	815
49.81343	46.35537	4.481047	43.35527	779.25
53.40886	46.37584	14.3885	43.37574	728

61.01018	46.3963	-29.6988	43.3962	686
9.617414	46.41676	-7.7807	43.41666	661
41.23055	46.43723	10.14266	43.43713	641.5
21.84959	46.45769	-19.9287	43.45759	616
36.47453	46.47815	-24.9947	43.47805	571
19.10538	46.49861	20.804	43.49851	506
11.74213	46.51908	32.60802	43.51897	439.5
17.38479	46.53954	40.41734	43.53944	380.5
36.03334	46.56	33.23196	43.5599	330
121.6878	46.58046	50.05188	43.58036	277
44.34817	46.60093	59.87711	43.60083	232.25
82.01444	46.62139	17.70764	43.62129	189.75
44.68662	46.64185	87.54348	43.64175	153
78.36469	46.66232	108.3846	43.66222	119.5
32.04868	46.68278	74.23106	43.68268	89.75
25.73856	46.70324	76.0828	43.70314	59
14.43435	46.7237	48.93985	43.7236	43.75
30.13605	46.74417	108.8022	43.74406	39
34.84364	46.76463	41.66985	43.76453	32.75
51.55715	46.78509	39.54281	43.78499	26.25
37.27655	46.80555	22.42106	43.80545	22.5
-17.9981	46.82602	74.30463	43.82592	17.25
46.73307	46.84648	91.19349	43.84638	16.75
27.47019	46.86694	51.08766	43.86684	15
43.21321	46.88741	100.9871	43.88731	12
44.96214	46.90787	75.89191	43.90777	8.5
82.71697	46.92833	106.802	43.92823	9
71.4777	46.94879	102.7174	43.94869	9.75
121.2443	46.96926	72.63805	43.96916	11.25
141.0169	46.98972	70.56404	43.98962	11.25
150.7953	47.01018	50.49533	44.01008	15
193.5797	47.03065	94.43192	44.03054	22
247.3699	47.05111	114.3738	44.05101	35.25
255.1661	47.07157	126.321	44.07147	52
293.9681	47.09203	166.2735	44.09193	74.5
294.7761	47.1125	212.2313	44.1124	100.25
265.59	47.13296	269.1944	44.13286	124.75
240.4097	47.15342	302.1628	44.15332	140.25
135.2354	47.17388	285.1366	44.17379	147
184.067	47.19435	360.1156	44.19425	143.75
193.9045	47.21481	327.0999	44.21471	135.75
122.7479	47.23527	257.0895	44.23517	121.75
118.5972	47.25574	197.0845	44.25563	107.25
124.4523	47.2762	204.0847	44.2761	91
91.31345	47.29666	214.0902	44.29656	76.25
14.18045	47.31712	240.1011	44.31702	63.25
59.05335	47.33759	174.1172	44.33749	51.25
-0.06784	47.35805	115.1386	44.35795	38.25
-1.18313	47.37851	51.16539	44.37841	26.25
-38.2925	47.39898	105.1974	44.39888	17.25
29.60402	47.41944	19.2348	44.41934	11.75
29.50644	47.4399	24.27745	44.4398	8
-31.5852	47.46036	16.32541	44.46026	9.25
-22.671	47.48083	33.37867	44.48072	9.25
2.24914	47.50129	-17.5628	44.50119	13.75
20.17518	47.52175	16.5011	44.52165	22
13.10713	47.54221	17.57028	44.54211	35.5
44.04498	47.56268	-47.3552	44.56258	57.5

-12.0113	47.58314	2.724529	44.58304	88.25
-18.0616	47.6036	0.80961	44.6035	129.25
10.89395	47.62407	-18.1	44.62397	169.5
-0.14459	47.64453	-21.0043	44.64443	201
0.822777	47.66499	6.096672	44.66489	221.5
-27.204	47.68545	-10.797	44.68535	228.5
28.77523	47.70592	-23.6854	44.70582	224.5
-18.2397	47.72638	-23.0162	44.72628	211
-24.2487	47.74684	-0.34172	44.74674	191.75
2.544853	47.76731	2.338101	44.7672	170.25
-4.65568	47.78777	26.02322	44.78767	152
26.14969	47.80823	-20.2864	44.80813	138
47.96097	47.82869	17.40937	44.82859	122.25
32.77815	47.84916	35.1104	44.84906	101.75
42.60123	47.86962	0.816729	44.86952	81.25
53.43022	47.89008	-3.47164	44.88998	61.5
75.26511	47.91054	34.2453	44.91045	45.5
66.1059	47.93101	73.96755	44.93091	35.75
67.95261	47.95147	113.6951	44.95137	27.75
77.80521	47.97193	124.4279	44.97183	20.25
85.66371	47.9924	69.16608	44.99229	15.5
54.52813	48.01286	96.90954	45.01276	11
5.398439	48.03332	111.6583	45.03322	7.5
-7.72534	48.05378	129.4124	45.05368	4.25
24.15678	48.07425	115.1717	45.07415	2.75
-7.95519	48.09471	96.93639	45.09461	1.25
60.93874	48.11517	120.7064	45.11507	1.5
7.83857	48.13564	133.4816	45.13554	2
28.74431	48.1561	97.2622	45.156	2
106.656	48.17656	121.0481	45.17646	2.25
61.57349	48.19702	119.8393	45.19692	2.5
50.49694	48.21749	151.6357	45.21738	2.25
98.4263	48.23795	131.4375	45.23785	1.25
149.3616	48.25841	131.2446	45.25831	2
189.3027	48.27887	175.057	45.27877	2
225.2498	48.29934	210.8747	45.29924	1.25
310.2028	48.3198	177.6977	45.3197	0.75
298.1616	48.34026	296.526	45.34016	0
348.1264	48.36073	331.3596	45.36063	0.25
371.0971	48.38119	416.1985	45.38109	2.75
446.0737	48.40165	366.0428	45.40155	8.25
389.0562	48.42211	496.8923	45.42201	16.75
519.0446	48.44258	570.7471	45.44248	26.75
518.0388	48.46304	642.6072	45.46294	37.25
651.0391	48.4835	662.4727	45.4834	45.25
711.0452	48.50397	691.3434	45.50386	49
698.0571	48.52443	659.2194	45.52433	49.75
783.0751	48.54489	680.1007	45.54479	46.75
693.0989	48.56535	755.9874	45.56525	42.75
647.1286	48.58582	658.8793	45.58572	39
554.1642	48.60628	749.7766	45.60618	34.5
476.2058	48.62674	773.6791	45.62664	28.25
368.2532	48.6472	612.587	45.64711	24.25
381.3065	48.66767	605.5001	45.66757	21
309.3658	48.68813	449.4185	45.68803	17
235.4309	48.70859	362.3423	45.70849	11.75
208.5019	48.72906	299.2714	45.72895	7.5
128.5789	48.74952	310.2058	45.74942	4.5

107.6617	48.76998	293.1454	45.76988	3.75
54.7505	48.79044	218.0904	45.79034	2.5
54.84517	48.81091	151.0407	45.81081	1.25
54.94573	48.83137	143.9962	45.83127	0
38.0522	48.85183	62.95713	45.85173	0
58.16457	48.8723	5.923311	45.8722	1.75
22.28285	48.89276	-10.1052	45.89266	2.5
30.40703	48.91322	-32.1284	45.91312	3.5
16.53712	48.93368	33.85368	45.93358	4.75
12.67311	48.95415	56.84108	45.95404	5
14.815	48.97461	36.83378	45.97451	4.75
14.9628	48.99507	33.83178	45.99497	4.25
50.1165	49.01553	97.83509	46.01543	4.25
83.2761	49.036	50.8437	46.0359	3.75
57.44161	49.05646	89.85761	46.05636	2.75
120.613	49.07692	67.87683	46.07682	2.75
161.7903	49.09739	162.9014	46.09729	1.75
197.9736	49.11785	131.9312	46.11775	2.25
202.1627	49.13831	172.9663	46.13821	2.25
172.3577	49.15877	216.0067	46.15867	1.75
186.5586	49.17924	250.0525	46.17914	1
146.7655	49.1997	287.1035	46.1996	1.5
173.9782	49.22016	304.1598	46.22006	1.25
108.1969	49.24063	347.2215	46.24052	1.5
118.4214	49.26109	409.2884	46.26099	1.75
53.65186	49.28155	342.3607	46.28145	1.5
122.8882	49.30201	336.4382	46.30191	0.75
77.13047	49.32248	261.5211	46.32238	1.75
53.37863	49.34294	201.6092	46.34284	2
18.6327	49.3634	135.7027	46.3633	1.75
53.89267	49.38386	155.8014	46.38377	1.25
69.15855	49.40433	249.9055	46.40423	1.5
41.43033	49.42479	109.0149	46.42469	0.5
2.708005	49.44525	107.1295	46.44515	1
37.99159	49.46572	66.24953	46.46561	1
72.28108	49.48618	56.3748	46.48608	1.5
100.5765	49.50664	75.50539	46.50654	1.75
145.8778	49.5271	94.64127	46.527	2.5
195.185	49.54757	61.78246	46.54747	2.5
213.4981	49.56803	105.9289	46.56793	1.75
206.8171	49.58849	150.0807	46.58839	0.75
264.142	49.60896	144.2378	46.60886	1.75
301.4728	49.62942	202.4002	46.62932	0.75
299.8095	49.64988	180.5679	46.64978	2.25
260.1522	49.67034	272.7409	46.67024	4
264.5007	49.69081	260.9193	46.6907	6.25
229.8551	49.71127	363.1029	46.71117	10
191.2155	49.73173	348.2918	46.73163	14
149.5817	49.75219	394.486	46.75209	18.5
223.9538	49.77266	254.6855	46.77256	25.25
111.3319	49.79312	214.8904	46.79302	30
107.7158	49.81358	166.1005	46.81348	35
64.1057	49.83405	210.3159	46.83395	38
38.50146	49.85451	108.5366	46.85441	38.75
15.90312	49.87497	130.7627	46.87487	36.5
25.31069	49.89543	72.99402	46.89533	32.5
6.724163	49.9159	86.23067	46.9158	27.5
27.14354	49.93636	38.47261	46.93626	22.75

-39.4312	49.95682	29.71986	46.95672	19.5
-24	49.97729	-16.0276	46.97718	18.25
-24.7386	49.99775	-23.7697	46.99765	16.75
			47.01811	16.75
			47.03857	16.5
			47.05904	16.5
			47.0795	17.5
			47.09996	19
			47.12043	22.5
			47.14089	26.25
			47.16135	32.75
			47.18181	42.5
			47.20227	63
			47.22274	91.25
			47.2432	127
			47.26366	167.25
			47.28413	204.25
			47.30459	228.75
			47.32505	239.75
			47.34552	232.75
			47.36598	217.25
			47.38644	195.75
			47.4069	172.5
			47.42736	153.75
			47.44783	135.25
			47.46829	115
			47.48875	98.25
			47.50922	75.25
			47.52968	53.5
			47.55014	34.25
			47.57061	22.5
			47.59107	15
			47.61153	8.5
			47.63199	7.75
			47.65246	5.25
			47.67292	3.25
			47.69338	4.5
			47.71384	2
			47.73431	1.5
			47.75477	1.75
			47.77523	2.75
			47.7957	2.5
			47.81616	1.5
			47.83662	1.25
			47.85709	2.25
			47.87755	2.25
			47.89801	2
			47.91847	1.25
			47.93893	1
			47.9594	0.25
			47.97986	0.75
			48.00032	1.25
			48.02079	3.5
			48.04125	7.75
			48.06171	12
			48.08218	17.25
			48.10264	20.5
			48.1231	21.75

48.14356	20.75
48.16402	19.25
48.18449	18.5
48.20495	17.5
48.22541	18.25
48.24588	19.25
48.26634	17.75
48.2868	17.25
48.30727	16
48.32773	17
48.34819	18.75
48.36865	22
48.38912	25.5
48.40958	35.25
48.43004	48.75
48.4505	71
48.47097	97.5
48.49143	140
48.51189	188.75
48.53236	245.75
48.55282	296.25
48.57328	337.75
48.59375	367.25
48.61421	393.75
48.63467	421.25
48.65513	469
48.67559	536.25
48.69606	622.25
48.71652	697.75
48.73698	747.25
48.75745	757.25
48.77791	733.75
48.79837	674.25
48.81884	597.5
48.8393	519.5
48.85976	453.75
48.88022	400
48.90068	350.5
48.92115	294.25
48.94161	228.5
48.96207	167
48.98254	113.75
49.003	67.25
49.02346	36.5
49.04393	19.5
49.06439	10.5
49.08485	5.5
49.10531	2.5
49.12578	0
49.14624	0
49.1667	0
49.18716	0
49.20763	0
49.22809	2.75
49.24855	9.5
49.26902	23.75
49.28948	44
49.30994	75.75

49.3304	109.5
49.35087	140
49.37133	160.25
49.39179	166.75
49.41225	162.25
49.43272	149
49.45318	129.25
49.47364	109.75
49.49411	93.25
49.51457	81.75
49.53503	70
49.5555	57
49.57596	41.25
49.59642	25.5
49.61688	13.5
49.63734	3.5
49.65781	0
49.67827	0
49.69873	0
49.7192	10
49.73966	26.75
49.76012	54.75
49.78059	92
49.80105	130.25
49.82151	156
49.84197	168.25
49.86244	166.75
49.8829	149
49.90336	125.5
49.92382	86.5
49.94429	65.25
49.96475	46
49.98521	46
50.00568	46

Figure 4a

Pressure kPa	Quantity Adsorbed for N2 cm\+(3) / g N2
5.45E-04	0.37174
5.91E-04	1.1216
4.89E-04	2.3715
4.21E-04	4.89
4.96E-04	9.8793
5.00E-04	22.652
5.01E-04	45.678
4.84E-04	58.534
9.06E-04	71.478
0.00198	84.287
0.00382	97.197
0.0067	109.9
0.01134	122.53
0.01808	135.11
0.02624	147.47
0.03562	159.46
0.04763	171.24
0.0652	182.51
0.09561	193.47
0.15308	203.74
0.32653	216.24
0.52977	222.78
1.6107	235.55
2.0103	237.76
6.9775	249.48
8.7096	251.46
10.861	253.36
13.943	255.43
17.801	257.36
21.321	258.67
25.15	259.78
29.683	260.79
33.872	261.58
36.472	261.99
38.493	262.27
40.47	262.49
42.499	262.67
44.511	262.85
46.511	263.04
48.509	263.21
50.524	263.36
52.521	263.53
54.503	263.69
56.421	263.83
58.523	263.9
60.512	264.02
62.527	264.07
64.5	264.11
66.507	264.22
68.48	264.22
70.412	264.27

PSD

Pore Width nm	Pore Volume cm\+(3) / g
0.4422	0.0010206
0.475	0.0021781
0.5104	0.0042991
0.5483	0.0078475
0.5891	0.013248
0.6329	0.020683
0.68	0.029861
0.7305	0.039868
0.7849	0.049221
0.8433	0.056192
0.906	0.059315
0.9733	0.057893
1.0457	0.052244
1.1235	0.043589
1.2071	0.033623
1.2968	0.023977
1.3933	0.015806
1.4969	0.0096327
1.6082	0.0054265
1.7278	0.0028258
1.8563	0.0013601
1.9944	6.05E-04
2.1427	2.49E-04
2.302	9.46E-05
2.4732	3.32E-05
2.6572	1.08E-05
2.8548	3.23E-06
3.0671	8.97E-07
3.2952	2.30E-07
3.5402	5.44E-08
3.8035	1.19E-08
4.0864	2.41E-09
4.3903	4.50E-10
4.7168	7.76E-11
5.0676	1.24E-11
5.4444	1.82E-12
5.8493	2.48E-13
6.2844	3.13E-14
6.7517	3.63E-15
7.2538	3.90E-16
7.7933	3.87E-17
8.3729	3.55E-18
8.9956	3.01E-19
9.6646	2.36E-20
10.383	0
11.156	0
11.985	0
12.876	0
13.834	0
14.863	0
15.968	0
17.156	0

72.442	264.28	18.432	0
74.391	264.28	19.802	0
76.405	264.31	21.275	0
78.386	264.32	22.857	0
80.377	264.31	24.557	0
82.381	264.28	26.384	0
84.389	264.33	28.346	0
86.414	264.31	30.454	0
88.401	264.29	32.719	0
90.437	264.26	35.152	0
92.46	264.27	37.766	0
94.433	264.3	40.575	0
96.434	264.37	43.592	0
98.541	264.64	46.834	0
		50.317	0
		54.059	0
		58.08	0
		62.399	0
		67.039	0
		72.025	0
		77.382	0
		83.136	0
		89.319	0
		95.962	0
		103.1	0
		110.77	0
		119	0
		127.85	0
		137.36	0
		147.58	0
		158.55	0
		170.34	0
		183.01	0
		196.62	0
		211.25	0
		226.96	0
		243.83	0
		261.97	0
		281.45	0
		302.38	0
		324.87	0
		349.03	0
		374.99	0
		402.88	0
		432.84	0
		465.03	0

Figure 4b

Pressure kPa	Uptake cm\+(3) / g, STP C2H6 (GCMC-298K)	cm\+(3) / g C2H4 (GCMC-298K)	Pressure kPa	Uptake cm\+(3) / g, STP C2H6 (298K)	kPa
1.00E-03	0.004491922	0.001763643	1.8656	3.5964	1.959
2.56507692	10.59535012	4.293970397	2.5611	4.9554	2.6501
5.12915385	18.46785986	8.290735481	4.3396	8.4824	4.5573
7.69323077	24.65384518	11.96924195	4.9305	9.6701	5.0468
10.2573077	30.79529468	15.78028698	5.1122	10.036	7.0774
12.8213846	34.61635336	18.87132391	6.922	13.445	7.562
15.3854615	38.44238858	22.34130028	7.4677	14.507	9.6528
17.9495385	42.05677129	25.62417405	7.6172	14.803	10.084
20.5136154	45.78560677	28.48036426	9.4282	18.154	12.579
23.0776923	48.8458643	31.31100924	9.9695	19.173	15.119
25.6417692	51.21941822	34.02004337	10.118	19.52	17.615
28.2058462	54.51929427	36.47239456	12.721	23.963	20.222
30.7699231	57.18027372	39.05467335	15.194	28.275	22.631
33.334	60.17415768	41.27338707	17.696	32.501	25.112
35.8980769	61.53556829	42.36050592	20.185	36.585	27.595
38.4621539	66.27250911	45.65894729	22.647	40.414	30.093
41.0262308	65.42775087	47.16482941	25.079	43.746	32.592
43.5903077	69.69990576	49.92439114	27.728	47.298	35.079
46.1543846	70.50941568	50.77299029	30.231	50.545	37.587
48.7184615	73.15448016	53.28168068	32.741	53.732	40.071
51.2825385	74.89715285	55.06356069	35.231	56.837	42.559
53.8466154	76.39039425	55.09902908	37.733	59.781	45.051
56.4106923	77.93959604	58.05130411	40.225	62.574	47.533
58.9747692	83.57389768	59.89262119	42.73	65.316	50.013
61.5388462	82.62140768	61.61840047	45.233	67.944	52.562
64.1029231	84.98237225	62.56275701	47.711	70.391	55.049
66.667	84.21189866	64.56175376	50.212	72.738	57.543
69.2310769	87.12866464	64.82043804	52.72	75.046	60.072
71.7951539	91.9041564	66.5309319	55.31	77.373	62.532
74.3592308	90.55929337	67.73181191	57.51	79.035	65.093
76.9233077	91.61787877	70.02258652	59.983	80.811	67.552
79.4873846	94.12552478	70.59462265	62.493	82.514	70.11
82.0514615	98.03626472	70.20869305	65.016	84.208	72.636
84.6155385	97.06381565	73.30548406	67.53	85.723	75.209
87.1796154	99.68381109	75.35744378	70.057	87.204	77.652
89.7436923	99.60925806	75.86944396	72.564	88.634	80.166
92.3077692	101.009659	77.72183442	75.081	90	82.725
94.8718462	103.5817945	78.58461019	77.61	91.292	85.18
97.4359231	101.5372045	79.60924547	80.121	92.5	87.696
100	106.5768471	80.18992345	82.637	93.655	90.182
			85.152	94.751	92.753
			87.666	95.787	95.258
			90.187	96.748	97.688
			92.707	97.687	100.29
			95.206	98.568	
			97.724	99.393	
			100.23	100.18	

exp-c2h4
cm\+(3) / g
C2H4 (298K)

3.3838
4.4358
7.3618
8.1099
10.999
11.692
14.49
15.097
18.192
21.245
24.151
27.098
29.654
32.225
34.718
37.131
39.432
41.667
43.843
45.942
47.948
49.918
51.801
53.648
55.495
57.187
58.859
60.479
62.042
63.572
65.048
66.501
67.876
69.264
70.545
71.79
73.057
74.226
75.367
76.505
77.592
78.661
79.649
80.674

Figure 4c

Pressure kPa	Uptake cm\+(3) / C2H6 (298K)	kPa	exp-c2h4 cm\+(3) / C2H4 (298	Pressure kPa Fugacity	exp-c2h2 cm\+(3) / g, STP C2H2 (298K)		Pressure bar p/p0
1.8656	3.5964	1.959	3.3838	1.2191	3.8935	0.8701	0.01841
2.5611	4.9554	2.6501	4.4358	1.7898	5.25	1.353	0.02527
4.3396	8.4824	4.5573	7.3618	2.1384	6.0535	1.8747	0.04283
4.9305	9.6701	5.0468	8.1099	2.4467	8.8251	2.3577	0.04866
5.1122	10.036	7.0774	10.999	2.5424	10.1	2.5686	0.05045
6.922	13.445	7.562	11.692	3.6126	12.727	3.7733	0.06831
7.4677	14.507	9.6528	14.49	4.2524	13.719	4.4994	0.0737
7.6172	14.803	10.084	15.097	4.6442	16.177	4.9983	0.07517
9.4282	18.154	12.579	18.192	4.8816	17.028	6.4786	0.09305
9.9695	19.173	15.119	21.245	5.0282	19.979	7.1706	0.09839
10.118	19.52	17.615	24.151	6.2589	22.524	7.5847	0.09985
12.721	23.963	20.222	27.098	6.9342	25.253	9.158	
15.194	28.275	22.631	29.654	7.2958	27.737	9.8055	
17.696	32.501	25.112	32.225	7.5911	29.997	10.038	
20.185	36.585	27.595	34.718	8.8723	32.336	12.638	
22.647	40.414	30.093	37.131	9.5422	34.593	15.199	
25.079	43.746	32.592	39.432	9.8851	36.984	17.935	
27.728	47.298	35.079	41.667	10.054	38.628	20.133	
30.231	50.545	37.587	43.843	12.022	41.035	22.6	
32.741	53.732	40.071	45.942	12.687	42.702	25.041	
35.231	56.837	42.559	47.948	14.887	44.513	27.588	
37.733	59.781	45.051	49.918	15.153	46.37	30.287	
40.225	62.574	47.533	51.801	17.458	48.164	32.826	
42.73	65.316	50.013	53.648	17.665	49.942	35.36	
45.233	67.944	52.562	55.495	20.045	51.762	37.844	
47.711	70.391	55.049	57.187	22.657	53.485	40.311	
50.212	72.738	57.543	58.859	25.152	55.137	42.867	
52.72	75.046	60.072	60.479	27.853	56.842	45.358	
55.31	77.373	62.532	62.042	30.26	58.513	47.83	
57.51	79.035	65.093	63.572	32.782	60.164	50.395	
59.983	80.811	67.552	65.048	35.27	61.741	52.876	
62.493	82.514	70.11	66.501	37.796	63.176	55.366	
65.016	84.208	72.636	67.876	40.306	64.706	57.824	
67.53	85.723	75.209	69.264	42.507	66.211	60.011	
70.057	87.204	77.652	70.545	44.999	67.668	62.478	
72.564	88.634	80.166	71.79	47.553	69.092	64.986	
75.081	90	82.725	73.057	50.068	70.615	67.503	
77.61	91.292	85.18	74.226	52.597	72.032	70.029	
80.121	92.5	87.696	75.367	55.127	73.425	72.554	
82.637	93.655	90.182	76.505	57.655	74.778	75.075	
85.152	94.751	92.753	77.592	60.186	76.142	77.597	
87.666	95.787	95.258	78.661	62.699	77.412	80.111	
90.187	96.748	97.688	79.649	65.217	78.652	82.635	
92.707	97.687	100.29	80.674	67.727	79.882	85.131	
95.206	98.568			70.252	81.075	87.665	
97.724	99.393			72.757		90.263	
100.23	100.18			75.271		92.779	
				77.782		95.27	
				80.29		97.698	
				82.806		100.2	
				85.306			

87.82
90.33
92.824
95.333
97.831
100.34

Uptake		exp-c2h4		exp-c2h2
cm\+(3) / g, bar		cm\+(3) / g		Va/cm^3(STP) g^-1
C2H6 (298K)	p/p0	C2H4 (298K)	p/p0	C2H2 (298K)
3.5964	0.01933	3.3838	0.01413	3.8935
4.9554	0.02615	4.4358	0.02147	5.25
8.4824	0.04498	7.3618	0.02566	6.0535
9.6701	0.04981	8.1099	0.04183	8.8251
10.036	0.06985	10.999	0.04993	10.1
13.445	0.07463	11.692	0.06785	12.727
14.507	0.09526	14.49	0.07508	13.719
14.803	0.09952	15.097	0.09389	16.177
18.154	0.1241	18.192	0.09961	17.028
19.173	0.1492	21.245	0.125	19.979
19.52	0.1738	24.151	0.1485	22.524

Figure 4d

mol/kg	kJ/mol		
uptake	Qst C2H4	Load mol/	Qst C2H6
0.2	26.14689	0.2	28.59688
0.4	26.11827	0.4	28.59133
0.6	26.08973	0.6	28.58581
0.8	26.06129	0.8	28.58032
1	26.03294	1	28.57485
1.2	26.00468	1.2	28.56941
1.4	25.97651	1.4	28.564
1.6	25.94844	1.6	28.55862
1.8	25.92045	1.8	28.55326
2	25.89256	2	28.54792
2.2	25.86476	2.2	28.54262
2.4	25.83704	2.4	28.53733
2.6	25.80942	2.6	28.53208
2.8	25.78188	2.8	28.52684
3	25.75444	3	28.52163
3.2	25.72708	3.2	28.51645
3.4	25.69981	3.4	28.51129
3.6	25.67263	3.6	28.50615
		3.8	28.50104
		4	28.49595
		4.2	28.49088
		4.4	28.48583
		4.6	28.4808
		4.8	28.4758
		5	28.47082
		5.2	28.46586
		5.4	28.46092
		5.6	28.456
		5.8	28.4511
		6	28.44622

Figure 4e

kPa	C2H4	C2H6	Sads
1	0.039542	0.030746	0.777552982
1.0476	0.041416	0.032447	0.783441182
1.0975	0.043377	0.034241	0.789381469
1.1498	0.04543	0.036133	0.795355492
1.2045	0.047579	0.038128	0.801361945
1.2619	0.049827	0.040232	0.807433721
1.3219	0.052181	0.04245	0.813514498
1.3849	0.054644	0.04479	0.819669131
1.4508	0.05722	0.047256	0.825865082
1.5199	0.059917	0.049857	0.832101073
1.5923	0.062737	0.052598	0.83838883
1.6681	0.065688	0.055487	0.844705273
1.7475	0.068774	0.058533	0.851091982
1.8307	0.072003	0.061742	0.857492049
1.9179	0.075379	0.065124	0.863954152
2.0092	0.078909	0.068688	0.870471049
2.1049	0.0826	0.072442	0.877021792
2.2051	0.08646	0.076398	0.883622484
2.3101	0.090493	0.080563	0.890267756
2.4201	0.09471	0.08495	0.89694858
2.5354	0.099117	0.08957	0.90367949
2.6561	0.10372	0.094434	0.910470497
2.7826	0.10853	0.099554	0.917294757
2.9151	0.11356	0.10494	0.92409299
3.0539	0.11881	0.11062	0.931066409
3.1993	0.12429	0.11658	0.937967656
3.3516	0.13001	0.12286	0.94500423
3.5112	0.13598	0.12946	0.952051772
3.6784	0.14222	0.13641	0.959147799
3.8535	0.14872	0.14371	0.966312534
4.037	0.15551	0.15138	0.973442222
4.2292	0.16259	0.15944	0.980626115
4.4306	0.16997	0.16792	0.987939048
4.6416	0.17767	0.17681	0.995159565
4.8626	0.18569	0.18616	1.0025311
5.0941	0.19405	0.19597	1.009894357
5.3367	0.20276	0.20626	1.017261787
5.5908	0.21182	0.21706	1.024737985
5.857	0.22126	0.22839	1.032224532
6.1359	0.23108	0.24027	1.039769777
6.4281	0.2413	0.25271	1.047285537
6.7342	0.25193	0.26576	1.054896201
7.0548	0.26298	0.27941	1.062476234
7.3907	0.27446	0.29371	1.070137725
7.7426	0.28639	0.30868	1.07783093
8.1113	0.29878	0.32433	1.085514425
8.4975	0.31163	0.34069	1.093251612
8.9022	0.32497	0.35779	1.100993938
9.326	0.3388	0.37566	1.10879575
9.7701	0.35313	0.3943	1.11658596
10.235	0.36798	0.41376	1.124408935
10.723	0.38335	0.43405	1.132255119

11.233	0.39926	0.45519	1.140084156
11.768	0.41571	0.47722	1.147963725
12.328	0.43271	0.50015	1.155854961
12.916	0.45028	0.524	1.163720352
13.53	0.46841	0.54879	1.171601802
14.175	0.48711	0.57454	1.179487179
14.85	0.50639	0.60128	1.187385217
15.557	0.52625	0.62901	1.195268409
16.298	0.54669	0.65775	1.203149866
17.074	0.56772	0.68751	1.211001902
17.886	0.58933	0.71831	1.218858704
18.738	0.61152	0.75016	1.226713762
19.63	0.6343	0.78305	1.234510484
20.565	0.65764	0.81699	1.242305821
21.544	0.68155	0.85199	1.25007703
22.57	0.70602	0.88803	1.25779723
23.645	0.73104	0.92512	1.265484789
24.771	0.75659	0.96324	1.273133401
25.95	0.78267	1.0024	1.280744119
27.186	0.80924	1.0425	1.288245761
28.48	0.83631	1.0837	1.295811362
29.836	0.86384	1.1258	1.303250602
31.257	0.89182	1.1688	1.310578368
32.745	0.92022	1.2128	1.317945709
34.305	0.94902	1.2576	1.325156477
35.938	0.97818	1.3032	1.332270134
37.649	1.0077	1.3496	1.339287486
39.442	1.0375	1.3968	1.346313253
41.32	1.0676	1.4447	1.353222181
43.288	1.0979	1.4932	1.360051006
45.349	1.1285	1.5422	1.366592822
47.508	1.1592	1.5918	1.373188406
49.77	1.19	1.6419	1.379747899
52.14	1.221	1.6923	1.385995086
54.623	1.252	1.7431	1.392252396
57.224	1.283	1.7941	1.398363211
59.948	1.3141	1.8453	1.404231033
62.803	1.3451	1.8966	1.410006691
65.793	1.376	1.9479	1.415625
68.926	1.4067	1.9993	1.421269638
72.208	1.4373	2.0505	1.426633271
75.646	1.4678	2.1015	1.431734569
79.248	1.498	2.1523	1.436782377
83.022	1.5279	2.2028	1.44171739
86.975	1.5575	2.2529	1.446484751
91.116	1.5869	2.3025	1.450942088
95.455	1.6159	2.3516	1.455288075
100	1.6445	2.4001	1.459470964

Figure 4f

Fe2(O2)(dobdc)

	Selectivity	mmol/g<br>Uptake for C2H6
Fe2(O2)(dobdc)	4.4	3.3
MAF-49	2.7	1.7
ZIF-8	1.994	3.5
ZIF-7	1.75	2.24
ZIF-3	2.22	6
PCN-245	1.75	3.3
MIL-142A	1.5	3.8
IRMOF-8	1.6	4.8
PCN-250	1.9	5.2
Ni(bdc)(ted)0.5	2	5
Cu(Qc)2	3.75	1.85
Zn-atz-ipa	2	1.76
MUF-15	1.95	4.7
Our work	1.46	4.47

Figure 5a

EXP10/90	C2H6	C2H4
20	0	0
21	0	0
22	0	0
23	0	0
24	0	0
25	0	0.671934
26	0	0.915227
27	0	1.045917
29	0	1.101083
30	0	1.108692
31	0.00158	1.116203
32	0.015277	1.119725
33	0.069361	1.10941
35	0.237206	1.089678
37	0.511704	1.057407
39	0.73286	1.031406
41	0.831318	1.019831
43	0.903215	1.011379
45	0.970208	1.010464
48	0.989611	1.004126
51	1.003307	1.010088
53	1.00559	1.004583
55	1.006731	1.01027
57	1	1

Figure 5b

EXP10/90	C2H6	C2H4	Simulated	C2H6	C2H4
20	0	0	24.038333	2.67E-05	
21	0	0	24.083333	3.31E-05	
22	0	0	24.128333	4.11E-05	
23	0	0	24.173333	5.12E-05	
24	0	0	24.218333	6.38E-05	
25	0	0.671934	24.263333	7.95E-05	
26	0	0.915227	24.308333	9.91E-05	
27	0	1.045917	24.353333	1.24E-04	
29	0	1.101083	24.398333	1.54E-04	
30	0	1.108692	24.443333	1.93E-04	
31	0.00158	1.116203	24.488333	2.41E-04	
32	0.015277	1.119725	24.533333	3.01E-04	
33	0.069361	1.10941	24.578333	3.76E-04	
35	0.237206	1.089678	24.623333	4.70E-04	
37	0.511704	1.057407	24.668333	5.88E-04	
39	0.73286	1.031406	24.713333	7.34E-04	
41	0.831318	1.019831	24.756667	9.18E-04	
43	0.903215	1.011379	24.801667	0.001148	
45	0.970208	1.010464	24.846667	0.001435	
48	0.989611	1.004126	24.891667	0.001793	
51	1.003307	1.010088	24.936667	0.002242	
53	1.00559	1.004583	24.981667	0.002802	
55	1.006731	1.01027	25.026667	0.003502	1.76E-06
57	1	1	25.071667	0.004376	1.90E-06
			25.116667	0.005469	2.07E-06
			25.161667	0.006833	2.27E-06
			25.206667	0.008538	2.50E-06
			25.251667	0.010666	2.78E-06
			25.296667	0.013322	3.11E-06
			25.341667	0.016637	3.51E-06
			25.386667	0.020772	4.00E-06
			25.431667	0.025926	4.58E-06
			25.478333	0.032346	5.30E-06
			25.523333	0.040338	6.18E-06
			25.568333	0.050273	7.28E-06
			25.615	0.062605	8.63E-06
			25.661667	0.077892	1.03E-05
			25.708333	0.096796	1.25E-05
			25.755	0.120107	1.52E-05
			25.803333	0.148748	1.86E-05
			25.851667	0.183778	2.30E-05
			25.901667	0.226359	2.86E-05
			25.951667	0.277708	3.58E-05
			26.005	0.339042	4.51E-05
			26.06	0.411222	5.71E-05
			26.118333	0.494661	7.26E-05
			26.181667	0.588671	9.28E-05
			26.25	0.690802	1.19E-04
			26.328333	0.79475	1.53E-04
			26.415	0.888925	1.96E-04
			26.511667	0.963363	2.49E-04
			26.605	1.009941	3.05E-04

26.703333	1.038873	3.68E-04
26.808333	1.056106	4.43E-04
26.92	1.065741	5.33E-04
27.04	1.070779	6.44E-04
27.17	1.073257	7.81E-04
27.306667	1.074385	9.50E-04
27.45	1.074853	0.001159
27.6	1.075046	0.001416
27.756667	1.075101	0.001732
27.92	1.075101	0.002122
28.088333	1.075074	0.0026
28.265	1.075046	0.003188
28.446667	1.074991	0.00391
28.635	1.074908	0.004797
28.83	1.074826	0.005885
29.031667	1.074743	0.00722
29.24	1.074606	0.008857
29.455	1.074468	0.010864
29.678333	1.074275	0.013322
29.91	1.074055	0.016333
30.15	1.07378	0.020016
30.398333	1.073449	0.024518
30.656667	1.073036	0.030018
30.925	1.072541	0.036728
31.203333	1.071935	0.044904
31.493333	1.071192	0.054856
31.796667	1.070284	0.066944
32.113333	1.06921	0.081604
32.445	1.067861	0.099341
32.793333	1.066264	0.120744
33.16	1.064365	0.146491
33.548333	1.062053	0.17734
33.961667	1.0593	0.214129
34.401667	1.056024	0.257742
34.876667	1.052197	0.309052
35.39	1.04771	0.368886
35.953333	1.042535	0.437713
36.578333	1.036671	0.515632
37.283333	1.030174	0.601777
38.1	1.02321	0.693918
39.075	1.016135	0.787645
40.296667	1.009473	0.87545
41.945	1.004077	0.946482
44.468333	1.000857	0.988576
49.408333	1.000031	0.99975
66.701667	1.000003	0.999998
379.85	1.000003	0.999998
1666.6667	1.000003	0.999998

Figure 5c

Cyc1			Cyc2			Cyc3	
	C2H4	C2H6		C2H4	C2H6		C2H4
28.5	0	0	28.5	0	0	28.5	0
29	0	0	29	0	0	29	0
29.5	0	0	29.5	0	0	29.5	0
30	1.32894	0	30	1.32894	0	30	1.32894
30.5	10.74188	0	30.5	5.276558	0	30.5	8.015803
31	49.9122	0	31	45.3468	0	31	35.3029
31.5	89.56102	0	31.5	86.82177	0	31.5	81.79982
32	95.53117	0	32	97.81387	0	32	95.98771
32.5	99.11326	0	32.5	99.5698	0	32.5	98.65672
33	99.53468	0	33	99.99122	0	33	99.53468
33.5	99.67515	0	33.5	99.99493	0	33.5	99.11326
34	99.42932	0	34	99.25373	0	34	98.34065
34.5	95.32046	4.679543	34.5	93.49429	6.505707	34.5	93.03775
35	81.27305	18.72695	35	79.44688	20.55312	35	78.07726
35.5	69.04156	30.95844	35.5	67.22564	32.77436	35.5	65.85601
36	61.73398	38.26602	36	59.46444	40.53556	36	59.0079
36.5	55.71408	44.28592	36.5	53.42406	46.57594	36.5	53.42406
37	52.46269	47.53731	37	51.56277	48.43723	37	50.64969
37.5	50.7887	49.2113	37.5	50.33363	49.66637	37.5	50.7887
38	50.04829	49.95171	38	50.04829	49.95171	38	50.04829
38.5	50	50	38.5	50	50	38.5	50
39	50	50	39	50	50	39	50
39.5	50	50	39.5	50	50	39.5	50

	Cyc4			Cyc5		
C2H6		C2H4	C2H6		C2H4	C2H6
0	28.5	0	0	28.5	0	0
0	29	0	0	29	0	0
0	29.5	0	0	29.5	0	0
0	30	0	0	30	0.886743	0
0	30.5	2.993854	0	30.5	9.385426	0
0	31	44.43371	0	31	43.52063	0
0	31.5	84.53907	0	31.5	79.97366	0
0	32	94.61809	0	32	95.53117	0
0	32.5	97.74363	0	32.5	98.65672	0
0	33	98.1753	0	33	99.07814	0
0	33.5	98.20749	0	33.5	98.65672	0
0	34	96.51449	3.485514	34	98.34065	0
6.962248	34.5	92.58121	7.418788	34.5	92.58121	7.418788
21.92274	35	81.72959	18.27041	35	79.90342	20.09658
34.14399	35.5	70.42142	29.57858	35.5	69.50834	30.49166
40.9921	36	63.11677	36.88323	36	60.37752	39.62248
46.57594	36.5	57.07638	42.92362	36.5	54.79368	45.20632
49.35031	37	53.38894	46.61106	37	52.01932	47.98068
49.2113	37.5	51.24671	48.75329	37.5	50.33363	49.66637
49.95171	38	50.04829	49.95171	38	50.04829	49.95171
50	38.5	50	50	38.5	50	50
50	39	50	50	39	50	50
50	39.5	50	50	39.5	50	50

Figure 5d

	C2H2	C2H4	C2H6
20	0	0	0
21	0	0	0
22	0	0	0
23	0	0	0
24	0	0	0.053234
25	0	0	0.517442
26	0	0	0.957887
27	0	0	1.072608
28	0	0	1.109892
29	0	0	1.122183
30	0	0	1.129968
31	0	0	1.132016
32	0	0	1.135704
33	0	0.021276	1.125461
35	0.008165	0.167954	1.105795
37	0.218759	0.511706	1.078753
39	0.493269	0.866111	1.052531
41	0.660843	0.95379	1.02508
43	0.775563	0.977963	1.006234
45	0.874305	0.991484	1.005004
47	0.946825	1.005414	1.004126
49	0.976734	1.003307	1.010088
51	0.989435	1.00559	1.004583
53	0.995991	1.006731	1.01027
55	1	1	1
57	1	1	1
60	1	1	1

Supplementary Figure 3

2 Theta degree a.u.	Intensity a.u.		
MOF-Th(Exp.)		Simulated A	
34	4	0	3
34	4.02	0	3.020462
34	4.04	0	3.040925
34	4.06	0	3.061388
0	4.08	0	3.081851
4	4.1	0	3.102313
0	4.12	0	3.122776
0	4.14	0	3.143239
0	4.16	0	3.163702
0	4.18	0	3.184165
0	4.2	0	3.204627
22	4.22	0	3.22509
45	4.24	0	3.245553
56	4.26	0	3.266016
61	4.28	0	3.286478
74	4.3	0	3.306941
73	4.32	0	3.327404
46	4.34	0	3.347867
46	4.36	0	3.36833
54	4.38	0	3.388792
70	4.4	0	3.409255
92	4.42	0	3.429718
116	4.44	0	3.450181
120	4.46	0	3.470643
104	4.48	0	3.491106
94	4.5	0	3.511569
88	4.52	0	3.532032
68	4.54	0	3.552495
77	4.56	0	3.572957
74	4.58	0	3.59342
55	4.6	0	3.613883
22	4.62	0	3.634346
48	4.64	0	3.654808
59	4.66	0	3.675271
46	4.68	0	3.695734
62	4.7	0	3.716197
88	4.72	0	3.73666
88	4.74	0	3.757122
77	4.76	0	3.777585
55	4.78	0	3.798048
32	4.8	0	3.818511
0	4.82	0	3.838973
4	4.84	0	3.859436
2	4.86	0	3.879899
0	4.88	0	3.900362
0	4.9	0	3.920825
0	4.92	0	3.941287
0	4.94	19.97874	3.96175
0	4.96	20.5456	3.982213
0	4.98	21.1372	4.002676
0	5	21.7544	4.023139

0	5.02	22.3992	4.043602
11	5.04	23.073	4.064064
34	5.06	23.7776	4.084527
50	5.08	24.5152	4.10499
52	5.1	25.2874	4.125453
62	5.12	26.0968	4.145916
62	5.14	26.9456	4.166378
53	5.16	27.8364	4.186841
36	5.18	28.7722	4.207304
23	5.2	29.756	4.227767
9	5.22	30.791	4.248229
4	5.24	31.881	4.268692
8	5.26	33.0298	4.289155
0	5.28	34.2418	4.309618
0	5.3	35.5216	4.330081
12	5.32	36.8746	4.350543
27	5.34	38.3064	4.371006
36	5.36	39.823	4.391469
41	5.38	41.4316	4.411932
41	5.4	43.1394	4.432394
34	5.42	44.955	4.452857
27	5.44	46.8876	4.47332
17	5.46	48.9474	4.493783
0	5.48	51.1458	4.514246
0	5.5	53.4956	4.534708
4	5.52	56.0108	4.555171
9	5.54	58.7076	4.575634
13	5.56	61.6036	4.596097
14	5.58	64.719	4.616559
12	5.6	68.0766	4.637022
11	5.62	71.7018	4.657485
12	5.64	75.6242	4.677948
9	5.66	79.877	4.698411
4	5.68	84.498	4.718873
0	5.7	89.5314	4.739336
0	5.72	95.0272	4.759799
6	5.74	101.044	4.780262
10	5.76	107.6496	4.800724
12	5.78	114.923	4.821187
11	5.8	122.9574	4.84165
14	5.82	131.8622	4.862113
15	5.84	141.7678	4.882576
10	5.86	152.8292	4.903038
9	5.88	165.2328	4.923501
6	5.9	179.2042	4.943964
0	5.92	201.226	4.964427
11	5.94	219.394	4.984889
20	5.96	240.168	5.005352
23	5.98	264.068	5.025815
23	6	291.754	5.046278
29	6.02	324.07	5.066741
29	6.04	362.102	5.087203
23	6.06	407.28	5.107666
23	6.08	461.494	5.128129
15	6.1	527.306	5.148592
0	6.12	608.24	5.169055
11	6.14	709.24	5.189517
14	6.16	837.41	5.20998

14	6.18	1003.266	5.230443
9	6.2	1223.022	5.250906
19	6.22	1523.558	5.271368
22	6.24	1954.154	5.291831
21	6.26	2612.52	5.312294
12	6.28	3688.6	5.332757
12	6.3	5504.58	5.35322
19	6.32	8484.08	5.373682
34	6.34	12899.5	5.394145
39	6.36	17899.46	5.414608
38	6.38	20000	5.435071
28	6.4	16854.3	5.455533
31	6.42	11785	5.475996
30	6.44	7688.7	5.496459
23	6.46	5011.04	5.516922
3	6.48	3399.14	5.537385
13	6.5	2442.1	5.557847
18	6.52	1849.116	5.57831
19	6.54	1455.49	5.598773
31	6.56	1177.786	5.619236
40	6.58	973.444	5.639698
38	6.6	818.704	5.660161
38	6.62	698.946	5.680624
46	6.64	604.592	5.701087
45	6.66	529.124	5.72155
35	6.68	467.984	5.742012
36	6.7	417.918	5.762475
27	6.72	376.556	5.782938
29	6.74	342.146	5.803401
37	6.76	313.37	5.823863
34	6.78	289.232	5.844326
22	6.8	268.966	5.864789
21	6.82	251.99	5.885252
22	6.84	237.848	5.905715
20	6.86	226.202	5.926177
18	6.88	216.794	5.94664
15	6.9	209.442	5.967103
20	6.92	204.026	5.987566
24	6.94	200.488	6.008028
24	6.96	198.8302	6.028491
14	6.98	199.1158	6.048954
7	7	201.48	6.069417
20	7.02	206.138	6.08988
33	7.04	213.42	6.110342
35	7.06	223.788	6.130805
47	7.08	237.898	6.151268
56	7.1	256.676	6.171731
64	7.12	281.432	6.192193
64	7.14	314.06	6.212656
57	7.16	357.344	6.233119
37	7.18	415.52	6.253582
19	7.2	495.402	6.274045
23	7.22	609	6.294507
24	7.24	779.612	6.31497
20	7.26	1053.572	6.335433
28	7.28	1514.926	6.355896
33	7.3	2288.1	6.376358
29	7.32	3495.7	6.396821

30	7.34	5071.64	6.417284
21	7.36	6259.42	6.437747
14	7.38	5875.88	6.458209
2	7.4	4358.58	6.478672
11	7.42	2902.2	6.499135
14	7.44	1890.92	6.519598
25	7.46	1267.158	6.540061
27	7.48	897.022	6.560523
27	7.5	672.172	6.580986
23	7.52	526.542	6.601449
11	7.54	425.624	6.621912
16	7.56	352.1	6.642374
20	7.58	296.682	6.662837
16	7.6	253.85	6.6833
32	7.62	220.064	6.703763
29	7.64	192.9406	6.724226
52	7.66	170.8284	6.744688
63	7.68	152.5552	6.765151
85	7.7	137.2716	6.785614
106	7.72	124.3498	6.806077
120	7.74	113.3192	6.826539
146	7.76	103.8204	6.847002
167	7.78	95.5758	6.867465
174	7.8	88.3682	6.887928
181	7.82	82.026	6.908391
183	7.84	57.0968	6.928853
205	7.86	52.6162	6.949316
221	7.88	48.6416	6.969779
261	7.9	45.0996	6.990242
305	7.92	41.93	7.010705
374	7.94	39.0822	7.031167
449	7.96	36.5144	7.05163
556	7.98	34.1912	7.072093
660	8	32.0822	7.092556
771	8.02	30.1624	7.113018
919	8.04	28.4094	7.133481
1081	8.06	26.8046	7.153944
1252	8.08	25.332	7.174407
1499	8.1	23.9772	7.19487
1808	8.12	22.7282	7.215332
2199	8.14	21.5742	7.235795
2670	8.16	20.5056	7.256258
3228	8.18	19.51458	7.276721
3791	8.2	18.59354	7.297183
4449	8.22	17.73614	7.317646
5269	8.24	16.93666	7.338109
6300	8.26	16.18996	7.358572
7694	8.28	15.49154	7.379035
9421	8.3	14.83732	7.399497
11396	8.32	14.22366	7.41996
13245	8.34	13.64724	7.440423
14555	8.36	13.10514	7.460886
14866	8.38	12.5947	7.481348
14041	8.4	12.11348	7.501811
12315	8.42	11.6593	7.522274
9948	8.44	11.23018	7.542737
7386	8.46	10.8243	7.5632
5088	8.48	10.44002	7.583662

3299	8.5	10.07584	7.604125
2211	8.52	9.73036	7.624588
1619	8.54	9.40236	7.645051
1337	8.56	9.09064	7.665513
1113	8.58	8.79418	7.685976
952	8.6	8.51198	7.706439
827	8.62	8.24312	7.726902
723	8.64	7.98682	7.747365
651	8.66	7.74226	7.767827
582	8.68	7.50878	7.78829
525	8.7	7.28568	7.808753
461	8.72	7.07238	7.829216
412	8.74	6.86832	7.849678
382	8.76	6.67294	7.870141
353	8.78	6.4858	7.890604
330	8.8	6.3064	7.911067
302	8.82	6.13436	7.93153
292	8.84	0	7.951992
294	8.86	0	7.972455
287	8.88	0	7.992918
288	8.9	0	8.013381
284	8.92	0	8.033843
324	8.94	0	8.054306
350	8.96	0	8.074769
389	8.98	0.37564	8.095232
440	9	0.386254	8.115695
532	9.02	0.397324	8.136157
613	9.04	0.408876	8.15662
700	9.06	0.420942	8.177083
768	9.08	0.433546	8.197546
847	9.1	0.446728	8.218008
952	9.12	0.460518	8.238471
1098	9.14	0.474956	8.258934
1266	9.16	0.490084	8.279397
1528	9.18	0.505946	8.29986
1944	9.2	0.52259	8.320322
2502	9.22	0.54007	8.340785
3293	9.24	0.55844	8.361248
4365	9.26	0.577764	8.381711
5579	9.28	0.598106	8.402173
6672	9.3	0.619542	8.422636
7289	9.32	0.64215	8.443099
7230	9.34	0.666018	8.463562
6530	9.36	0.691242	8.484025
5356	9.38	0.717924	8.504487
3916	9.4	0.74618	8.52495
2450	9.42	0.776136	8.545413
1300	9.44	0.807932	8.565876
621	9.46	0.841722	8.586338
370	9.48	0.877672	8.606801
346	9.5	0.915974	8.627264
341	9.52	0.956838	8.647727
318	9.54	1.000494	8.66819
260	9.56	1.047204	8.688652
223	9.58	1.09726	8.709115
183	9.6	1.150988	8.729578
142	9.62	1.208756	8.750041
115	9.64	1.270978	8.770503

103	9.66	1.338122	8.790966
90	9.68	1.410724	8.811429
79	9.7	1.48939	8.831892
71	9.72	1.574812	8.852355
90	9.74	1.667784	8.872817
77	9.76	1.769224	8.89328
90	9.78	1.880188	8.913743
89	9.8	2.0019	8.934206
87	9.82	2.1358	8.954668
91	9.84	2.28358	8.975131
83	9.86	2.44718	8.995594
72	9.88	2.62896	9.016057
50	9.9	2.8317	9.03652
49	9.92	3.05876	9.056982
46	9.94	3.31416	9.077445
50	9.96	3.60282	9.097908
51	9.98	3.9307	9.118371
53	10	4.30526	9.138833
55	10.02	4.7357	9.159296
46	10.04	5.23368	9.179759
34	10.06	5.814	9.200222
30	10.08	6.49572	9.220685
24	10.1	7.30372	9.241147
29	10.12	8.27086	9.26161
15	10.14	9.44138	9.282073
25	10.16	10.8758	9.302536
13	10.18	12.65848	9.322998
24	10.2	14.90984	9.343461
29	10.22	17.80626	9.363924
27	10.24	21.6158	9.384387
27	10.26	26.7716	9.40485
32	10.28	34.0426	9.425312
30	10.3	44.93	9.445775
24	10.32	62.406	9.466238
12	10.34	91.7858	9.486701
7	10.36	140.742	9.507163
0	10.38	216.324	9.527626
6	10.4	312.22	9.548089
16	10.42	377.642	9.568552
21	10.44	345.214	9.589015
23	10.46	251.648	9.609477
19	10.48	166.0402	9.62994
23	10.5	107.5668	9.650403
18	10.52	71.7584	9.670866
17	10.54	50.5446	9.691328
11	10.56	37.6218	9.711791
7	10.58	29.2174	9.732254
17	10.6	23.3786	9.752717
20	10.62	19.12294	9.77318
21	10.64	15.91892	9.793642
23	10.66	13.44794	9.814105
21	10.68	11.5044	9.834568
23	10.7	9.94964	9.855031
16	10.72	8.68738	9.875493
16	10.74	7.64916	9.895956
10	10.76	7.44352	9.916419
8	10.78	6.73554	9.936882
3	10.8	6.13838	9.957345

2	10.82	5.63102	9.977807
2	10.84	5.19722	9.99827
1	10.86	4.82434	10.01873
5	10.88	4.50234	10.0392
7	10.9	4.22322	10.05966
6	10.92	3.98058	10.08012
9	10.94	3.76918	10.10058
4	10.96	3.58474	10.12105
10	10.98	3.42378	10.14151
14	11	3.2834	10.16197
17	11.02	3.16116	10.18244
20	11.04	3.0551	10.2029
20	11.06	2.96354	10.22336
23	11.08	2.88504	10.24382
22	11.1	2.81846	10.26429
18	11.12	2.76282	10.28475
13	11.14	2.7173	10.30521
4	11.16	2.68124	10.32568
6	11.18	2.65408	10.34614
0	11.2	2.6354	10.3666
0	11.22	2.62486	10.38706
0	11.24	2.6222	10.40753
6	11.26	2.62728	10.42799
10	11.28	2.64	10.44845
21	11.3	2.66034	10.46891
30	11.32	3.42564	10.48938
40	11.34	3.4822	10.50984
40	11.36	3.54766	10.5303
40	11.38	3.62242	10.55077
41	11.4	3.7069	10.57123
36	11.42	3.8016	10.59169
30	11.44	3.90718	10.61215
28	11.46	4.02436	10.63262
24	11.48	4.15404	10.65308
28	11.5	4.29722	10.67354
32	11.52	4.45512	10.69401
31	11.54	4.6291	10.71447
31	11.56	4.82076	10.73493
34	11.58	5.03196	10.75539
35	11.6	5.26492	10.77586
40	11.62	5.52216	10.79632
40	11.64	5.80668	10.81678
39	11.66	6.12198	10.83724
36	11.68	6.47222	10.85771
31	11.7	6.86238	10.87817
22	11.72	7.29834	10.89863
17	11.74	7.78724	10.9191
22	11.76	8.33768	10.93956
19	11.78	8.9602	10.96002
13	11.8	9.6677	10.98048
28	11.82	10.47628	11.00095
34	11.84	11.406	11.02141
47	11.86	12.48236	11.04187
55	11.88	13.73796	11.06233
56	11.9	14.85582	11.0828
66	11.92	16.6198	11.10326
83	11.94	18.73508	11.12372
90	11.96	21.3028	11.14419

92	11.98	24.4632	11.16465
86	12	28.4144	11.18511
110	12.02	33.445	11.20557
128	12.04	39.9866	11.22604
152	12.06	48.7236	11.2465
177	12.08	60.8352	11.26696
213	12.1	78.5724	11.28743
279	12.12	106.4248	11.30789
337	12.14	152.7448	11.32835
431	12.16	230.478	11.34881
562	12.18	353.89	11.36928
708	12.2	522.624	11.38974
961	12.22	672.078	11.4102
1333	12.24	665.904	11.43066
1831	12.26	511.704	11.45113
2382	12.28	345.892	11.47159
2840	12.3	226.696	11.49205
3059	12.32	152.4144	11.51252
2983	12.34	108.6206	11.53298
2655	12.36	82.6624	11.55344
2119	12.38	66.5158	11.5739
1466	12.4	55.92	11.59437
866	12.42	48.7598	11.61483
424	12.44	43.9458	11.63529
191	12.46	40.8736	11.65576
109	12.48	39.1988	11.67622
101	12.5	38.7418	11.69668
87	12.52	39.4486	11.71714
68	12.54	41.3776	11.73761
49	12.56	44.712	11.75807
38	12.58	49.7968	11.77853
31	12.6	57.2266	11.79899
21	12.62	68.0468	11.81946
26	12.64	84.2438	11.83992
36	12.66	109.8074	11.86038
30	12.68	152.4436	11.88085
40	12.7	224.932	11.90131
43	12.72	343.37	11.92177
38	12.74	515.76	11.94223
31	12.76	700.122	11.9627
24	12.78	755.602	11.98316
24	12.8	618.582	12.00362
21	12.82	427.608	12.02409
23	12.84	279.08	12.04455
27	12.86	183.296	12.06501
27	12.88	125.8412	12.08547
31	12.9	91.5622	12.10594
27	12.92	70.0886	12.1264
19	12.94	55.66	12.14686
5	12.96	45.3724	12.16732
5	12.98	37.7324	12.18779
7	13	31.8948	12.20825
2	13.02	27.3344	12.22871
0	13.04	23.7046	12.24918
0	13.06	20.7684	12.26964
2	13.08	18.35922	12.2901
11	13.1	16.35764	12.31056
15	13.12	14.676	12.33103

30	13.14	13.24902	12.35149
39	13.16	12.02726	12.37195
38	13.18	10.97274	12.39242
41	13.2	10.0559	12.41288
39	13.22	9.25344	12.43334
33	13.24	8.54682	12.4538
27	13.26	7.92112	12.47427
25	13.28	7.36424	12.49473
22	13.3	6.86628	12.51519
25	13.32	7.4293	12.53565
30	13.34	7.05458	12.55612
32	13.36	6.71936	12.57658
30	13.38	6.41892	12.59704
39	13.4	6.14928	12.61751
37	13.42	5.90706	12.63797
37	13.44	5.6894	12.65843
32	13.46	5.49388	12.67889
23	13.48	5.31838	12.69936
13	13.5	5.1611	12.71982
15	13.52	5.02052	12.74028
16	13.54	4.89532	12.76075
30	13.56	4.78434	12.78121
36	13.58	4.68662	12.80167
37	13.6	4.60136	12.82213
35	13.62	4.52782	12.8426
47	13.64	4.46544	12.86306
49	13.66	4.41372	12.88352
54	13.68	4.37228	12.90398
49	13.7	4.34084	12.92445
56	13.72	3.67962	12.94491
68	13.74	3.68432	12.96537
79	13.76	3.69792	12.98584
84	13.78	3.72046	13.0063
83	13.8	3.75204	13.02676
101	13.82	3.79284	13.04722
122	13.84	3.84312	13.06769
147	13.86	3.90324	13.08815
183	13.88	3.97358	13.10861
220	13.9	4.05472	13.12908
275	13.92	4.14722	13.14954
340	13.94	4.25182	13.17
428	13.96	4.3694	13.19046
557	13.98	4.50094	13.21093
733	14	4.64756	13.23139
976	14.02	4.81064	13.25185
1261	14.04	4.9917	13.27231
1565	14.06	5.19252	13.29278
1814	14.08	5.41518	13.31324
1919	14.1	5.6621	13.3337
1877	14.12	5.93606	13.35417
1671	14.14	6.24032	13.37463
1365	14.16	6.57876	13.39509
1019	14.18	6.95584	13.41555
687	14.2	7.37692	13.43602
432	14.22	7.8483	13.45648
269	14.24	7.64968	13.47694
201	14.26	8.26532	13.49741
191	14.28	8.9581	13.51787

174	14.3	9.74142	13.53833
165	14.32	10.63168	13.55879
160	14.34	11.64918	13.57926
164	14.36	12.81924	13.59972
172	14.38	14.17372	13.62018
191	14.4	15.7533	13.64064
211	14.42	17.61032	13.66111
209	14.44	19.8132	13.68157
224	14.46	22.4526	13.70203
248	14.48	25.6504	13.7225
298	14.5	29.5742	13.74296
370	14.52	34.4572	13.76342
484	14.54	40.6342	13.78388
635	14.56	48.5956	13.80435
813	14.58	59.0912	13.82481
990	14.6	73.3406	13.84527
1104	14.62	93.5318	13.86574
1115	14.64	124.2276	13.8862
1038	14.66	173.3632	13.90666
895	14.68	256.11	13.92712
723	14.7	393.43	13.94759
505	14.72	603.036	13.96805
324	14.74	860.934	13.98851
202	14.76	1017.42	14.00897
149	14.78	906.406	14.02944
145	14.8	651.562	14.0499
156	14.82	428.16	14.07036
156	14.84	277.8	14.09083
170	14.86	186.264	14.11129
185	14.88	132.031	14.13175
194	14.9	98.832	14.15221
191	14.92	77.1034	14.17268
216	14.94	61.9334	14.19314
223	14.96	50.842	14.2136
250	14.98	42.4752	14.23407
260	15	36.0142	14.25453
248	15.02	30.9286	14.27499
240	15.04	26.8588	14.29545
228	15.06	23.7094	14.31592
209	15.08	20.998	14.33638
196	15.1	18.74548	14.35684
212	15.12	16.85594	14.3773
229	15.14	15.25736	14.39777
253	15.16	13.89484	14.41823
273	15.18	12.72588	14.43869
280	15.2	11.71726	14.45916
273	15.22	10.8427	14.47962
291	15.24	10.08128	14.50008
293	15.26	9.41614	14.52054
299	15.28	8.83362	14.54101
306	15.3	8.32262	14.56147
328	15.32	7.874	14.58193
347	15.34	7.4803	14.6024
372	15.36	7.13534	14.62286
378	15.38	6.83406	14.64332
387	15.4	6.57232	14.66378
391	15.42	6.34676	14.68425
414	15.44	6.15466	14.70471

440	15.46	5.99396	14.72517
459	15.48	5.86308	14.74563
482	15.5	5.76096	14.7661
511	15.52	5.68706	14.78656
545	15.54	5.64128	14.80702
567	15.56	5.62408	14.82749
586	15.58	5.63638	14.84795
599	15.6	5.67978	14.86841
605	15.62	5.75654	14.88887
619	15.64	5.86976	14.90934
642	15.66	6.02356	14.9298
658	15.68	6.22328	14.95026
684	15.7	6.47586	14.97073
703	15.72	6.7903	14.99119
730	15.74	7.17834	15.01165
762	15.76	7.65526	15.03211
791	15.78	8.24138	15.05258
811	15.8	8.96386	15.07304
834	15.82	9.85958	15.0935
876	15.84	10.97956	15.11396
911	15.86	12.39572	15.13443
957	15.88	14.21182	15.15489
1002	15.9	16.5821	15.17535
1024	15.92	19.7464	15.19582
1071	15.94	24.1074	15.21628
1105	15.96	30.414	15.23674
1123	15.98	40.1572	15.2572
1152	16	56.1982	15.27767
1188	16.02	83.2524	15.29813
1235	16.04	127.205	15.31859
1249	16.06	190.7946	15.33906
1281	16.08	257.95	15.35952
1301	16.1	276.712	15.37998
1320	16.12	225.794	15.40044
1362	16.14	156.5012	15.42091
1381	16.16	103.0784	15.44137
1405	16.18	68.8712	15.46183
1443	16.2	48.5652	15.48229
1461	16.22	35.6878	15.50276
1470	16.24	28.522	15.52322
1484	16.26	24.011	15.54368
1521	16.28	21.1528	15.56415
1535	16.3	19.46912	15.58461
1580	16.32	18.74188	15.60507
1623	16.34	18.91586	15.62553
1658	16.36	20.0904	15.646
1703	16.38	22.5948	15.66646
1741	16.4	27.1948	15.68692
1759	16.42	35.4394	15.70739
1823	16.44	49.948	15.72785
1917	16.46	74.069	15.74831
2031	16.48	109.5764	15.76877
2185	16.5	148.1374	15.78924
2370	16.52	160.6244	15.8097
2546	16.54	132.5014	15.83016
2707	16.56	92.2664	15.85062
2806	16.58	60.707	15.87109
2775	16.6	40.2582	15.89155

2655	16.62	27.9418	15.91201
2476	16.64	20.5638	15.93248
2246	16.66	16.17046	15.95294
2012	16.68	13.04326	15.9734
1838	16.7	10.80332	15.99386
1675	16.72	9.13162	16.01433
1584	16.74	7.84784	16.03479
1545	16.76	6.83992	16.05525
1501	16.78	6.03388	16.07572
1440	16.8	5.37904	16.09618
1409	16.82	4.83972	16.11664
1375	16.84	4.39028	16.1371
1343	16.86	4.01186	16.15757
1304	16.88	3.69044	16.17803
1282	16.9	3.41528	16.19849
1232	16.92	3.1782	16.21895
1220	16.94	2.97278	16.23942
1221	16.96	2.79394	16.25988
1187	16.98	2.63766	16.28034
1161	17	2.5007	16.30081
1145	17.02	2.38042	16.32127
1125	17.04	2.2747	16.34173
1082	17.06	2.18178	16.36219
1033	17.08	2.10018	16.38266
999	17.1	2.02872	16.40312
977	17.12	1.966402	16.42358
981	17.14	1.912402	16.44405
981	17.16	1.866046	16.46451
951	17.18	1.826776	16.48497
940	17.2	1.794142	16.50543
920	17.22	1.767792	16.5259
904	17.24	1.74745	16.54636
868	17.26	1.73292	16.56682
846	17.28	1.724074	16.58728
840	17.3	1.720856	16.60775
848	17.32	1.723268	16.62821
836	17.34	1.731386	16.64867
832	17.36	1.745348	16.66914
832	17.38	1.765366	16.6896
837	17.4	1.791726	16.71006
822	17.42	1.824798	16.73052
806	17.44	1.86505	16.75099
778	17.46	1.913054	16.77145
754	17.48	1.969504	16.79191
742	17.5	2.03524	16.81238
733	17.52	2.11128	16.83284
741	17.54	2.19882	16.8533
758	17.56	2.03378	16.87376
777	17.58	2.15612	16.89423
793	17.6	2.29504	16.91469
804	17.62	2.45312	16.93515
798	17.64	2.63358	16.95561
793	17.66	2.84026	16.97608
786	17.68	3.078	16.99654
789	17.7	3.35284	17.017
780	17.72	3.67232	17.03747
810	17.74	4.0461	17.05793
832	17.76	4.91378	17.07839

874	17.78	5.44922	17.09885
928	17.8	6.08954	17.11932
1013	17.82	6.86356	17.13978
1085	17.84	7.81066	17.16024
1180	17.86	8.98574	17.18071
1225	17.88	10.46698	17.20117
1241	17.9	12.36872	17.22163
1202	17.92	14.86434	17.24209
1143	17.94	18.23272	17.26256
1071	17.96	22.9664	17.28302
988	17.98	29.8702	17.30348
931	18	41.1532	17.32394
897	18.02	60.0852	17.34441
866	18.04	91.686	17.36487
874	18.06	140.7676	17.38533
880	18.08	204.072	17.4058
894	18.1	249.948	17.42626
867	18.12	232.1	17.44672
863	18.14	170.9174	17.46718
857	18.16	113.3914	17.48765
864	18.18	73.7458	17.50811
879	18.2	49.3918	17.52857
889	18.22	34.9774	17.54904
897	18.24	26.2374	17.5695
904	18.26	20.5908	17.58996
927	18.28	16.69524	17.61042
914	18.3	13.87656	17.63089
878	18.32	11.77196	17.65135
860	18.34	10.16534	17.67181
823	18.36	8.91774	17.69227
802	18.38	7.93582	17.71274
768	18.4	7.15498	17.7332
737	18.42	6.5295	17.75366
708	18.44	6.02642	17.77413
669	18.46	5.62164	17.79459
659	18.48	5.29722	17.81505
640	18.5	5.0398	17.83551
610	18.52	4.8393	17.85598
588	18.54	4.68818	17.87644
564	18.56	4.5808	17.8969
536	18.58	4.51312	17.91737
512	18.6	4.4823	17.93783
495	18.62	4.48658	17.95829
477	18.64	4.5252	17.97875
462	18.66	4.59822	17.99922
444	18.68	4.7066	18.01968
427	18.7	4.85222	18.04014
390	18.72	5.0379	18.0606
365	18.74	5.2676	18.08107
333	18.76	5.5466	18.10153
309	18.78	5.88172	18.12199
294	18.8	6.28184	18.14246
285	18.82	6.75828	18.16292
265	18.84	7.32572	18.18338
264	18.86	8.0031	18.20384
246	18.88	8.81524	18.22431
226	18.9	9.79492	18.24477
196	18.92	10.98602	18.26523

183	18.94	12.44824	18.2857
167	18.96	14.2642	18.30616
160	18.98	16.55064	18.32662
138	19	19.47646	18.34708
127	19.02	23.2934	18.36755
116	19.04	28.3956	18.38801
116	19.06	35.4496	18.40847
127	19.08	45.7064	18.42893
128	19.1	61.6606	18.4494
124	19.12	88.0614	18.46986
111	19.14	132.6026	18.49032
101	19.16	204.574	18.51079
84	19.18	307.168	18.53125
60	19.2	410.644	18.55171
45	19.22	430.324	18.57217
29	19.24	343.52	18.59264
17	19.26	234.512	18.6131
24	19.28	152.2448	18.63356
22	19.3	99.8282	18.65403
15	19.32	68.5364	18.67449
1	19.34	49.8456	18.69495
5	19.36	38.0816	18.71541
5	19.38	30.1422	18.73588
8	19.4	24.469	18.75634
8	19.42	20.2544	18.7768
7	19.44	17.03674	18.79726
2	19.46	14.5266	18.81773
3	19.48	12.6242	18.83819
0	19.5	11.0175	18.85865
4	19.52	9.70328	18.87912
8	19.54	8.61514	18.89958
14	19.56	7.70434	18.92004
20	19.58	6.69628	18.9405
24	19.6	6.04642	18.96097
29	19.62	5.48872	18.98143
39	19.64	5.00676	19.00189
57	19.66	4.5876	19.02235
91	19.68	4.22098	19.04282
111	19.7	3.89862	19.06328
177	19.72	3.61384	19.08374
284	19.74	3.36118	19.10421
456	19.76	3.1361	19.12467
707	19.78	2.93492	19.14513
984	19.8	2.7545	19.16559
1261	19.82	2.59224	19.18606
1457	19.84	2.44594	19.20652
1546	19.86	2.31374	19.22698
1477	19.88	2.19404	19.24745
1272	19.9	2.0855	19.26791
992	19.92	1.986936	19.28837
683	19.94	1.897368	19.30883
412	19.96	1.81593	19.3293
226	19.98	1.741876	19.34976
102	20	1.674566	19.37022
62	20.02	1.613448	19.39068
41	20.04	1.558046	19.41115
42	20.06	1.507948	19.43161
25	20.08	1.46281	19.45207

37	20.1	1.422334	19.47254
36	20.12	1.386278	19.493
32	20.14	1.354442	19.51346
26	20.16	1.32667	19.53392
20	20.18	1.302848	19.55439
15	20.2	1.282902	19.57485
22	20.22	1.266796	19.59531
19	20.24	1.254542	19.61578
13	20.26	1.24619	19.63624
6	20.28	1.241836	19.6567
16	20.3	1.241632	19.67716
15	20.32	1.245782	19.69763
12	20.34	1.254558	19.71809
10	20.36	1.26831	19.73855
5	20.38	1.287472	19.75901
6	20.4	1.312586	19.77948
2	20.42	1.344322	19.79994
0	20.44	1.383508	19.8204
5	20.46	1.995518	19.84087
11	20.48	2.06884	19.86133
15	20.5	2.15414	19.88179
17	20.52	2.25346	19.90225
19	20.54	2.3693	19.92272
24	20.56	2.50486	19.94318
26	20.58	2.66424	19.96364
27	20.6	2.85276	19.98411
23	20.62	3.07736	20.00457
21	20.64	3.34734	20.02503
27	20.66	3.67524	20.04549
27	20.68	4.0784	20.06596
31	20.7	4.17312	20.08642
42	20.72	4.82146	20.10688
61	20.74	5.65636	20.12734
92	20.76	6.75894	20.14781
143	20.78	9.7531	20.16827
229	20.8	11.95796	20.18873
364	20.82	15.32406	20.2092
571	20.84	20.818	20.22966
886	20.86	30.0372	20.25012
1288	20.88	44.9838	20.27058
1699	20.9	66.6228	20.29105
2052	20.92	89.6162	20.31151
2260	20.94	96.3726	20.33197
2273	20.96	79.3068	20.35244
2090	20.98	55.7556	20.3729
1766	21	37.5398	20.39336
1338	21.02	25.8692	20.41382
917	21.04	18.94274	20.43429
579	21.06	14.88382	20.45475
346	21.08	12.41234	20.47521
192	21.1	10.81898	20.49567
120	21.12	9.74698	20.51614
81	21.14	9.01326	20.5366
71	21.16	8.51478	20.55706
60	21.18	8.18806	20.57753
70	21.2	7.99184	20.59799
59	21.22	7.89856	20.61845
58	21.24	7.88964	20.63891

42	21.26	7.95252	20.65938
36	21.28	8.07894	20.67984
24	21.3	8.26374	20.7003
14	21.32	8.5042	20.72077
10	21.34	8.79952	20.74123
19	21.36	9.15056	20.76169
11	21.38	9.55978	20.78215
15	21.4	10.03118	20.80262
17	21.42	10.57034	20.82308
15	21.44	11.1847	20.84354
9	21.46	11.88376	20.864
16	21.48	12.6796	20.88447
13	21.5	13.58744	20.90493
10	21.52	14.62642	20.92539
7	21.54	15.8209	20.94586
5	21.56	17.20198	20.96632
1	21.58	18.80986	20.98678
0	21.6	20.6972	21.00724
7	21.62	22.9338	21.02771
14	21.64	25.614	21.04817
14	21.66	28.8682	21.06863
20	21.68	32.8798	21.0891
26	21.7	37.914	21.10956
29	21.72	44.3686	21.13002
28	21.74	52.8758	21.15048
26	21.76	64.5362	21.17095
14	21.78	81.4658	21.19141
7	21.8	107.855	21.21187
14	21.82	151.309	21.23233
17	21.84	223.136	21.2528
18	21.86	334.646	21.27326
23	21.88	480.604	21.29372
31	21.9	593.978	21.31419
29	21.92	566.28	21.33465
32	21.94	433.992	21.35511
23	21.96	306.824	21.37557
17	21.98	221.44	21.39604
25	22	173.4848	21.4165
31	22.02	151.477	21.43696
35	22.04	146.6472	21.45743
36	22.06	154.7686	21.47789
32	22.08	176.0066	21.49835
31	22.1	215.354	21.51881
20	22.12	284.9	21.53928
13	22.14	406.7	21.55974
0	22.16	611.56	21.5802
6	22.18	923.906	21.60066
16	22.2	1300.706	21.62113
22	22.22	1509.084	21.64159
24	22.24	1322.156	21.66205
23	22.26	943.89	21.68252
31	22.28	620.78	21.70298
34	22.3	405.104	21.72344
32	22.32	274.054	21.7439
23	22.34	196.1788	21.76437
20	22.36	148.1748	21.78483
25	22.38	116.4958	21.80529
34	22.4	94.1216	21.82576

35	22.42	77.7248	21.84622
36	22.44	65.2806	21.86668
48	22.46	55.6136	21.88714
72	22.48	47.9578	21.90761
91	22.5	41.7936	21.92807
140	22.52	36.7578	21.94853
185	22.54	32.591	21.96899
257	22.56	29.104	21.98946
335	22.58	26.1566	22.00992
409	22.6	23.6424	22.03038
446	22.62	21.4806	22.05085
471	22.64	19.60814	22.07131
451	22.66	17.97544	22.09177
402	22.68	16.54326	22.11223
333	22.7	15.2801	22.1327
270	22.72	14.16042	22.15316
192	22.74	13.1634	22.17362
159	22.76	12.27188	22.19409
142	22.78	11.47172	22.21455
128	22.8	10.75102	22.23501
125	22.82	10.09992	22.25547
144	22.84	9.51	22.27594
149	22.86	9.65256	22.2964
163	22.88	9.18392	22.31686
172	22.9	8.7591	22.33732
173	22.92	8.37378	22.35779
175	22.94	8.02434	22.37825
193	22.96	7.70766	22.39871
196	22.98	7.42114	22.41918
192	23	7.16268	22.43964
198	23.02	6.93054	22.4601
205	23.04	6.72346	22.48056
214	23.06	6.54052	22.50103
221	23.08	6.3813	22.52149
234	23.1	6.24586	22.54195
247	23.12	6.13486	22.56242
283	23.14	6.04966	22.58288
318	23.16	5.99268	22.60334
344	23.18	5.96754	22.6238
373	23.2	5.97988	22.64427
404	23.22	6.03814	22.66473
435	23.24	6.15512	22.68519
466	23.26	6.3509	22.70565
501	23.28	6.65882	22.72612
541	23.3	7.1399	22.74658
561	23.32	7.91512	22.76704
596	23.34	9.22132	22.78751
631	23.36	11.4661	22.80797
675	23.38	14.65636	22.82843
724	23.4	20.291	22.84889
804	23.42	26.8936	22.86936
918	23.44	30.1464	22.88982
1120	23.46	26.4868	22.91028
1452	23.48	19.89048	22.93075
1911	23.5	14.4265	22.95121
2515	23.52	10.84304	22.97167
3167	23.54	8.709	22.99213
3747	23.56	7.48048	23.0126

4151	23.58	6.76384	23.03306
4292	23.6	6.33376	23.05352
4147	23.62	6.07632	23.07398
3758	23.64	5.9344	23.09445
3246	23.66	5.8773	23.11491
2731	23.68	4.43692	23.13537
2319	23.7	4.54202	23.15584
2106	23.72	4.69584	23.1763
2121	23.74	4.89554	23.19676
2362	23.76	5.14062	23.21722
2893	23.78	5.4325	23.23769
3771	23.8	5.77436	23.25815
4985	23.82	6.171	23.27861
6335	23.84	6.6291	23.29908
7550	23.86	7.1574	23.31954
8419	23.88	7.7671	23.34
8740	23.9	8.47242	23.36046
8451	23.92	9.29156	23.38093
7621	23.94	10.24766	23.40139
6370	23.96	11.37058	23.42185
5043	23.98	12.6991	23.44231
3910	24	14.2843	23.46278
3092	24.02	16.19424	23.48324
2534	24.04	18.52122	23.5037
2214	24.06	21.3926	23.52417
2078	24.08	24.9878	23.54463
2019	24.1	29.5658	23.56509
1985	24.12	35.5104	23.58555
1931	24.14	43.419	23.60602
1901	24.16	54.2924	23.62648
1893	24.18	69.9884	23.64694
1875	24.2	94.2052	23.66741
1858	24.22	134.0566	23.68787
1818	24.24	201.322	23.70833
1817	24.26	311.094	23.72879
1811	24.28	470.18	23.74926
1791	24.3	640.106	23.76972
1766	24.32	691.196	23.79018
1750	24.34	565.234	23.81064
1732	24.36	389.822	23.83111
1707	24.38	253.492	23.85157
1663	24.4	165.6572	23.87203
1619	24.42	113.044	23.8925
1570	24.44	81.7188	23.91296
1531	24.46	62.15	23.93342
1477	24.48	49.0446	23.95388
1422	24.5	39.7358	23.97435
1392	24.52	32.853	23.99481
1359	24.54	27.6214	24.01527
1304	24.56	23.56	24.03574
1289	24.58	20.3494	24.0562
1247	24.6	17.7646	24.07666
1200	24.62	15.6456	24.09712
1165	24.64	13.88988	24.11759
1108	24.66	12.42598	24.13805
1043	24.68	11.19616	24.15851
982	24.7	10.15472	24.17897
932	24.72	9.2661	24.19944

875	24.74	8.50276	24.2199
825	24.76	7.84322	24.24036
794	24.78	7.27062	24.26083
757	24.8	6.77148	24.28129
732	24.82	6.335	24.30175
693	24.84	5.95242	24.32221
655	24.86	5.61656	24.34268
616	24.88	5.32156	24.36314
573	24.9	5.03726	24.3836
536	24.92	4.81098	24.40407
500	24.94	4.61344	24.42453
470	24.96	4.44192	24.44499
450	24.98	4.29424	24.46545
446	25	4.1686	24.48592
455	25.02	4.06366	24.50638
454	25.04	3.97834	24.52684
462	25.06	3.91194	24.5473
465	25.08	4.8324	24.56777
443	25.1	4.83024	24.58823
421	25.12	4.84778	24.60869
377	25.14	4.88552	24.62916
341	25.16	4.9444	24.64962
298	25.18	5.02564	24.67008
259	25.2	5.13102	24.69054
225	25.22	5.26274	24.71101
203	25.24	5.42372	24.73147
174	25.26	5.6176	24.75193
161	25.28	5.84898	24.7724
136	25.3	6.12376	24.79286
127	25.32	6.4493	24.81332
105	25.34	6.86688	24.83378
107	25.36	7.32614	24.85425
110	25.38	7.87388	24.87471
106	25.4	8.53108	24.89517
101	25.42	9.32576	24.91563
99	25.44	10.29598	24.9361
92	25.46	11.4944	24.95656
95	25.48	12.99526	24.97702
87	25.5	14.90594	24.99749
82	25.52	17.38656	25.01795
72	25.54	20.6874	25.03841
81	25.56	25.2328	25.05887
97	25.58	31.8188	25.07934
100	25.6	42.033	25.0998
105	25.62	58.8942	25.12026
106	25.64	87.2804	25.14073
112	25.66	133.0536	25.16119
121	25.68	198.1278	25.18165
124	25.7	263.34	25.20211
131	25.72	275.062	25.22258
157	25.74	219.932	25.24304
192	25.76	151.366	25.2635
257	25.78	99.1588	25.28396
361	25.8	66.4592	25.30443
540	25.82	47.0496	25.32489
835	25.84	35.566	25.34535
1265	25.86	28.4488	25.36582
1824	25.88	23.7566	25.38628

2397	25.9	20.516	25.40674
2897	25.92	18.22462	25.4272
3214	25.94	16.59682	25.44767
3281	25.96	15.45626	25.46813
3090	25.98	14.68888	25.48859
2666	26	14.21958	25.50906
2105	26.02	13.9993	25.52952
1518	26.04	13.99716	25.54998
1018	26.06	14.1957	25.57044
659	26.08	14.58848	25.59091
411	26.1	15.17828	25.61137
275	26.12	15.97696	25.63183
198	26.14	17.00598	25.65229
163	26.16	18.29768	25.67276
137	26.18	19.89796	25.69322
114	26.2	21.8702	25.71368
88	26.22	24.301	25.73415
59	26.24	27.3098	25.75461
48	26.26	31.0626	25.77507
35	26.28	35.7938	25.79553
28	26.3	41.8416	25.816
25	26.32	49.706	25.83646
22	26.34	60.1582	25.85692
17	26.36	75.1178	25.87739
6	26.38	95.6406	25.89785
3	26.4	126.9622	25.91831
0	26.42	178.0696	25.93877
2	26.44	264.324	25.95924
11	26.46	406.208	25.9797
25	26.48	617.798	26.00016
34	26.5	861.742	26.02062
36	26.52	973.64	26.04109
38	26.54	829.596	26.06155
33	26.56	583.764	26.08201
25	26.58	382.316	26.10248
15	26.6	250.09	26.12294
12	26.62	170.5708	26.1434
18	26.64	123.7448	26.16386
22	26.66	95.3544	26.18433
29	26.68	77.4592	26.20479
26	26.7	66.3584	26.22525
24	26.72	60.5894	26.24572
22	26.74	59.5326	26.26618
14	26.76	61.7286	26.28664
6	26.78	62.0326	26.3071
1	26.8	54.8686	26.32757
6	26.82	43.8418	26.34803
15	26.84	34.321	26.36849
17	26.86	27.5178	26.38895
17	26.88	22.9032	26.40942
14	26.9	19.73558	26.42988
22	26.92	17.45244	26.45034
28	26.94	15.7136	26.47081
38	26.96	14.33382	26.49127
40	26.98	13.2111	26.51173
56	27	12.28386	26.53219
65	27.02	11.51122	26.55266
70	27.04	10.86436	26.57312

76	27.06	10.32226	26.59358
83	27.08	9.86926	26.61405
93	27.1	9.49358	26.63451
118	27.12	9.97604	26.65497
145	27.14	9.75282	26.67543
206	27.16	9.5873	26.6959
306	27.18	9.47608	26.71636
474	27.2	9.15936	26.73682
737	27.22	9.1581	26.75728
1136	27.24	9.20764	26.77775
1608	27.26	9.30912	26.79821
2056	27.28	9.46472	26.81867
2390	27.3	9.67788	26.83914
2566	27.32	9.95336	26.8596
2522	27.34	10.29752	26.88006
2271	27.36	10.71866	26.90052
1875	27.38	11.2274	26.92099
1395	27.4	11.83736	26.94145
960	27.42	12.56596	26.96191
645	27.44	13.43554	26.98238
415	27.46	14.475	27.00284
263	27.48	15.7221	27.0233
169	27.5	17.22662	27.04376
140	27.52	19.05528	27.06423
115	27.54	21.2986	27.08469
97	27.56	24.0822	27.10515
94	27.58	27.583	27.12561
87	27.6	32.0572	27.14608
87	27.62	37.8872	27.16654
87	27.64	45.6708	27.187
73	27.66	56.421	27.20747
65	27.68	72.0398	27.22793
60	27.7	96.3206	27.24839
62	27.72	136.4768	27.26885
55	27.74	204.166	27.28932
57	27.76	313.404	27.30978
73	27.78	468.776	27.33024
100	27.8	624.516	27.35071
123	27.82	652.438	27.37117
151	27.84	520.484	27.39163
176	27.86	356.324	27.41209
221	27.88	232.836	27.43256
289	27.9	154.3254	27.45302
371	27.92	107.5718	27.47348
474	27.94	79.7478	27.49394
647	27.96	62.339	27.51441
945	27.98	49.7602	27.53487
1485	28	41.5824	27.55533
2278	28.02	35.6344	27.5758
3235	28.04	31.229	27.59626
4203	28.06	27.9388	27.61672
4992	28.08	25.484	27.63718
5523	28.1	23.678	27.65765
5620	28.12	22.394	27.67811
5248	28.14	21.548	27.69857
4508	28.16	21.0862	27.71904
3548	28.18	20.9782	27.7395
2608	28.2	21.214	27.75996

1814	28.22	21.8018	27.78042
1204	28.24	22.7382	27.80089
774	28.26	24.136	27.82135
502	28.28	26.0406	27.84181
386	28.3	28.5672	27.86227
323	28.32	32.361	27.88274
290	28.34	36.7254	27.9032
274	28.36	42.4898	27.92366
246	28.38	50.2138	27.94413
206	28.4	60.8052	27.96459
176	28.42	75.8828	27.98505
146	28.44	98.6098	28.00551
116	28.46	135.2534	28.02598
91	28.48	197.0136	28.04644
72	28.5	300.034	28.0669
61	28.52	459.086	28.08737
53	28.54	660.588	28.10783
45	28.56	797.166	28.12829
35	28.58	727.772	28.14875
24	28.6	530.792	28.16922
15	28.62	351.042	28.18968
11	28.64	228.356	28.21014
9	28.66	153.2336	28.2306
2	28.68	108.7108	28.25107
11	28.7	81.5662	28.27153
7	28.72	63.8958	28.29199
14	28.74	51.61	28.31246
18	28.76	42.6506	28.33292
19	28.78	35.9032	28.35338
19	28.8	30.7002	28.37384
16	28.82	26.61	28.39431
12	28.84	23.3416	28.41477
10	28.86	20.6934	28.43523
5	28.88	18.52144	28.4557
6	28.9	16.7218	28.47616
2	28.92	15.21752	28.49662
11	28.94	13.95088	28.51708
21	28.96	12.87804	28.53755
23	28.98	11.96518	28.55801
33	29	11.18602	28.57847
38	29.02	10.51994	28.59893
44	29.04	10.04218	28.6194
58	29.06	9.5594	28.63986
61	29.08	9.15036	28.66032
81	29.1	8.80696	28.68079
102	29.12	8.52288	28.70125
122	29.14	8.29322	28.72171
151	29.16	8.11436	28.74217
192	29.18	7.9838	28.76264
248	29.2	7.90004	28.7831
343	29.22	7.86258	28.80356
497	29.24	7.87186	28.82403
749	29.26	8.80648	28.84449
1128	29.28	8.93942	28.86495
1636	29.3	8.5122	28.88541
2138	29.32	8.77806	28.90588
2571	29.34	9.11142	28.92634
2869	29.36	9.5216	28.9468

2983	29.38	10.02072	28.96726
2872	29.4	10.62438	28.98773
2549	29.42	11.35298	29.00819
2082	29.44	12.2331	29.02865
1584	29.46	13.29994	29.04912
1155	29.48	14.60056	29.06958
822	29.5	16.19886	29.09004
544	29.52	18.1831	29.1105
363	29.54	20.6776	29.13097
245	29.56	23.8614	29.15143
192	29.58	28.0004	29.17189
168	29.6	33.5062	29.19236
133	29.62	41.0664	29.21282
108	29.64	51.9512	29.23328
90	29.66	68.6852	29.25374
78	29.68	96.146	29.27421
73	29.7	142.4858	29.29467
62	29.72	218.146	29.31513
63	29.74	328.92	29.33559
57	29.76	450.056	29.35606
65	29.78	492.214	29.37652
78	29.8	407.752	29.39698
97	29.82	283.774	29.41745
116	29.84	186.0544	29.43791
147	29.86	122.7994	29.45837
166	29.88	84.8948	29.47883
217	29.9	62.4132	29.4993
270	29.92	48.4762	29.51976
354	29.94	39.237	29.54022
482	29.96	32.7544	29.56068
725	29.98	28.0348	29.58115
1134	30	24.5198	29.60161
1759	30.02	21.8656	29.62207
2553	30.04	19.09216	29.64254
3424	30.06	17.57806	29.663
4169	30.08	16.44302	29.68346
4673	30.1	15.6147	29.70392
4827	30.12	15.0432	29.72439
4617	30.14	14.6949	29.74485
4065	30.16	14.67252	29.76531
3299	30.18	14.72156	29.78578
2488	30.2	14.9614	29.80624
1756	30.22	15.40056	29.8267
1197	30.24	16.05844	29.84716
801	30.26	16.96806	29.86763
516	30.28	18.18116	29.88809
333	30.3	19.77764	29.90855
243	30.32	21.885	29.92901
196	30.34	24.7236	29.94948
166	30.36	28.7084	29.96994
138	30.38	34.6372	29.9904
125	30.4	43.9028	30.01087
101	30.42	58.4796	30.03133
90	30.44	80.1382	30.05179
71	30.46	107.1276	30.07225
56	30.48	126.5816	30.09272
46	30.5	123.212	30.11318
38	30.52	107.8648	30.13364

21	30.54	97.5898	30.15411
12	30.56	97.8086	30.17457
11	30.58	109.742	30.19503
15	30.6	136.2826	30.21549
16	30.62	185.2428	30.23596
21	30.64	271.084	30.25642
22	30.66	412.512	30.27688
22	30.68	618.222	30.29734
17	30.7	835.812	30.31781
10	30.72	896.026	30.33827
4	30.74	729.3	30.35873
5	30.76	502.602	30.3792
10	30.78	327.51	30.39966
9	30.8	214.906	30.42012
10	30.82	147.4412	30.44058
13	30.84	107.1922	30.46105
16	30.86	82.2396	30.48151
17	30.88	65.296	30.50197
21	30.9	53.227	30.52244
20	30.92	44.2808	30.5429
21	30.94	37.4636	30.56336
26	30.96	32.1568	30.58382
26	30.98	27.9518	30.60429
36	31	24.5692	30.62475
43	31.02	21.8128	30.64521
63	31.04	19.54204	30.66567
90	31.06	17.65398	30.68614
115	31.08	16.12678	30.7066
151	31.1	14.7955	30.72706
197	31.12	13.6694	30.74753
270	31.14	12.7149	30.76799
384	31.16	11.90616	30.78845
576	31.18	11.22328	30.80891
880	31.2	10.65114	30.82938
1292	31.22	10.17854	30.84984
1775	31.24	9.3248	30.8703
2216	31.26	9.04344	30.89077
2561	31.28	8.84674	30.91123
2742	31.3	8.7361	30.93169
2755	31.32	8.71654	30.95215
2549	31.34	8.79752	30.97262
2176	31.36	8.99432	30.99308
1726	31.38	9.33028	31.01354
1292	31.4	9.84036	31.034
937	31.42	10.57714	31.05447
646	31.44	11.62136	31.07493
426	31.46	13.1027	31.09539
285	31.48	15.24766	31.11586
192	31.5	18.4922	31.13632
154	31.52	23.707	31.15678
125	31.54	32.502	31.17724
99	31.56	47.343	31.19771
74	31.58	70.8726	31.21817
73	31.6	102.7012	31.23863
72	31.62	129.7754	31.2591
58	31.64	126.6732	31.27956
59	31.66	96.9612	31.30002
55	31.68	66.0976	31.32048

46	31.7	44.1314	31.34095
51	31.72	30.4762	31.36141
57	31.74	22.4046	31.38187
62	31.76	17.58134	31.40233
60	31.78	14.5417	31.4228
73	31.8	12.51104	31.44326
79	31.82	11.10294	31.46372
98	31.84	10.11496	31.48419
126	31.86	9.4308	31.50465
188	31.88	8.97912	31.52511
268	31.9	8.71562	31.54557
366	31.92	8.6952	31.56604
464	31.94	8.74364	31.5865
565	31.96	8.84974	31.60696
648	31.98	9.19654	31.62743
717	32	9.71044	31.64789
738	32.02	10.41896	31.66835
766	32.04	11.36456	31.68881
808	32.06	12.61036	31.70928
978	32.08	14.24998	31.72974
1265	32.1	16.42394	31.7502
1761	32.12	19.3508	31.77066
2501	32.14	23.3934	31.79113
3332	32.16	29.2168	31.81159
4095	32.18	37.2736	31.83205
4662	32.2	51.8824	31.85252
4927	32.22	76.5214	31.87298
4881	32.24	116.9752	31.89344
4492	32.26	177.0972	31.9139
3846	32.28	245.832	31.93437
3044	32.3	276.244	31.95483
2287	32.32	234.862	31.97529
1674	32.34	166.1486	31.99576
1166	32.36	110.5482	32.01622
775	32.38	74.7242	32.03668
501	32.4	54.1912	32.05714
336	32.42	43.8182	32.07761
256	32.44	40.454	32.09807
211	32.46	42.9698	32.11853
169	32.48	51.008	32.13899
133	32.5	61.9114	32.15946
108	32.52	66.1418	32.17992
95	32.54	56.4402	32.20038
81	32.56	42.1	32.22085
72	32.58	29.6926	32.24131
47	32.6	21.3762	32.26177
34	32.62	16.17986	32.28223
17	32.64	12.93924	32.3027
14	32.66	10.81792	32.32316
15	32.68	9.33402	32.34362
16	32.7	8.2381	32.36409
22	32.72	7.39968	32.38455
26	32.74	6.74388	32.40501
29	32.76	6.2234	32.42547
30	32.78	5.80904	32.44594
24	32.8	5.47364	32.4664
23	32.82	5.20392	32.48686
10	32.84	4.98846	32.50732

2	32.86	4.8189	32.52779
2	32.88	4.68916	32.54825
12	32.9	4.59484	32.56871
24	32.92	4.53284	32.58918
48	32.94	4.50124	32.60964
87	32.96	4.49904	32.6301
126	32.98	4.52616	32.65056
226	33	4.58346	32.67103
355	33.02	4.67284	32.69149
523	33.04	4.7973	32.71195
695	33.06	4.96138	32.73242
849	33.08	5.17146	32.75288
959	33.1	5.43646	32.77334
1006	33.12	5.64902	32.7938
975	33.14	6.06952	32.81427
865	33.16	6.59996	32.83473
699	33.18	7.277	32.85519
546	33.2	8.15596	32.87565
390	33.22	9.3247	32.89612
272	33.24	10.9359	32.91658
175	33.26	13.2824	32.93704
102	33.28	16.94732	32.95751
60	33.3	23.0046	32.97797
42	33.32	33.0896	32.99843
27	33.34	48.9322	33.01889
19	33.36	70.1822	33.03936
12	33.38	87.983	33.05982
6	33.4	85.6784	33.08028
0	33.42	66.2196	33.10075
2	33.44	46.3034	33.12121
13	33.46	32.306	33.14167
22	33.48	23.7748	33.16213
38	33.5	18.91606	33.1826
54	33.52	16.21032	33.20306
73	33.54	14.71294	33.22352
108	33.56	13.93072	33.24398
148	33.58	13.6242	33.26445
187	33.6	14.00088	33.28491
245	33.62	14.36038	33.30537
346	33.64	14.99442	33.32584
529	33.66	15.90146	33.3463
821	33.68	17.09942	33.36676
1210	33.7	18.62546	33.38722
1645	33.72	20.5384	33.40769
2027	33.74	22.9246	33.42815
2307	33.76	25.6426	33.44861
2446	33.78	29.4026	33.46908
2410	33.8	34.1854	33.48954
2216	33.82	40.3608	33.51
1907	33.84	48.4896	33.53046
1567	33.86	59.4694	33.55093
1257	33.88	74.8706	33.57139
1029	33.9	97.725	33.59185
867	33.92	134.0694	33.61231
742	33.94	194.95	33.63278
649	33.96	296.972	33.65324
600	33.98	457.112	33.6737
556	34	669.54	33.69417

504	34.02	839.096	33.71463
443	34.04	801.328	33.73509
367	34.06	599.778	33.75555
275	34.08	399.648	33.77602
210	34.1	259.21	33.79648
144	34.12	172.3234	33.81694
101	34.14	120.9042	33.83741
71	34.16	89.9824	33.85787
57	34.18	70.3412	33.87833
44	34.2	57.1438	33.89879
34	34.22	47.831	33.91926
31	34.24	40.7396	33.93972
28	34.26	34.5932	33.96018
20	34.28	29.4468	33.98064
11	34.3	25.35	34.00111
2	34.32	22.1316	34.02157
4	34.34	19.58326	34.04203
10	34.36	17.53404	34.0625
11	34.38	15.86006	34.08296
10	34.4	14.47576	34.10342
6	34.42	13.32188	34.12388
6	34.44	12.3559	34.14435
7	34.46	11.54622	34.16481
5	34.48	10.86894	34.18527
3	34.5	10.3058	34.20574
6	34.52	9.84288	34.2262
6	34.54	9.4697	34.24666
5	34.56	9.17866	34.26712
1	34.58	8.9646	34.28759
0	34.6	8.82462	34.30805
2	34.62	8.75784	34.32851
6	34.64	8.7656	34.34897
11	34.66	8.85154	34.36944
11	34.68	9.02192	34.3899
15	34.7	9.28616	34.41036
33	34.72	9.65764	34.43083
65	34.74	10.15494	34.45129
98	34.76	10.80354	34.47175
139	34.78	11.63842	34.49221
174	34.8	12.70812	34.51268
207	34.82	14.08062	34.53314
220	34.84	15.8531	34.5536
222	34.86	18.16742	34.57407
203	34.88	21.1586	34.59453
173	34.9	25.3256	34.61499
146	34.92	31.1752	34.63545
114	34.94	39.8314	34.65592
81	34.96	53.5366	34.67638
56	34.98	76.4552	34.69684
31	35	115.016	34.7173
16	35.02	176.2384	34.73777
24	35.04	259.67	34.75823
39	35.06	332.578	34.77869
55	35.08	327.69	34.79916
63	35.1	250.64	34.81962
79	35.12	168.7452	34.84008
92	35.14	110.0002	34.86054
104	35.16	73.3192	34.88101

121	35.18	51.5488	34.90147
143	35.2	38.4656	34.92193
164	35.22	30.1336	34.9424
214	35.24	24.508	34.96286
263	35.26	20.4478	34.98332
323	35.28	17.44838	35.00378
426	35.3	15.1892	35.02425
596	35.32	13.46892	35.04471
893	35.34	12.15556	35.06517
1363	35.36	11.16148	35.08564
2026	35.38	10.42902	35.1061
2758	35.4	9.92246	35.12656
3417	35.42	9.62356	35.14702
3916	35.44	9.52988	35.16748
4163	35.46	9.65592	35.18795
4156	35.48	10.03714	35.20841
3906	35.5	9.92902	35.22887
3406	35.52	11.09146	35.24934
2801	35.54	12.9129	35.2698
2206	35.56	15.83684	35.29026
1693	35.58	20.7438	35.31073
1257	35.6	29.1834	35.33119
874	35.62	43.322	35.35165
575	35.64	64.9492	35.37211
378	35.66	91.3998	35.39258
276	35.68	106.9088	35.41304
239	35.7	94.7694	35.4335
197	35.72	68.3188	35.45396
168	35.74	45.3494	35.47443
141	35.76	29.92	35.49489
116	35.78	20.5134	35.51535
100	35.8	15.23626	35.53582
75	35.82	11.79282	35.55628
63	35.84	9.52258	35.57674
53	35.86	7.92632	35.59721
40	35.88	6.7517	35.61767
33	35.9	5.86044	35.63813
23	35.92	5.16874	35.65859
14	35.94	4.62212	35.67905
7	35.96	4.18368	35.69952
12	35.98	3.8277	35.71998
12	36	3.93346	35.74044
11	36.02	3.70352	35.76091
18	36.04	3.51506	35.78137
17	36.06	3.36076	35.80183
21	36.08	3.23506	35.8223
22	36.1	3.13378	35.84276
18	36.12	3.0537	35.86322
11	36.14	2.9924	35.88368
8	36.16	2.94806	35.90414
20	36.18	2.9194	35.92461
26	36.2	2.90554	35.94507
42	36.22	2.90596	35.96553
72	36.24	2.9206	35.986
94	36.26	2.94964	36.00646
126	36.28	2.99376	36.02692
152	36.3	3.05398	36.04739
192	36.32	3.1319	36.06785

266	36.34	3.22972	36.08831
381	36.36	3.35052	36.10877
576	36.38	3.49848	36.12924
848	36.4	3.67928	36.1497
1164	36.42	3.90082	36.17016
1454	36.44	4.17414	36.19062
1667	36.46	4.515	36.21109
1780	36.48	4.94654	36.23155
1783	36.5	5.5039	36.25201
1674	36.52	6.24394	36.27248
1478	36.54	7.26808	36.29294
1225	36.56	8.4586	36.3134
969	36.58	10.84634	36.33387
748	36.6	14.79828	36.35433
553	36.62	21.2956	36.37479
380	36.64	31.1938	36.39525
247	36.66	43.4862	36.41571
157	36.68	51.3526	36.43618
107	36.7	46.7218	36.45664
76	36.72	35.0146	36.4771
67	36.74	24.6524	36.49757
56	36.76	17.75186	36.51803
41	36.78	13.8389	36.53849
39	36.8	11.59568	36.55896
38	36.82	10.40446	36.57942
33	36.84	9.81378	36.59988
36	36.86	9.59632	36.62034
42	36.88	9.64416	36.6408
48	36.9	9.90642	36.66127
68	36.92	10.36174	36.68173
95	36.94	11.00752	36.70219
140	36.96	11.8562	36.72266
215	36.98	12.93486	36.74312
325	37	14.28732	36.76358
444	37.02	15.97894	36.78405
543	37.04	18.10506	36.80451
614	37.06	20.8052	36.82497
654	37.08	24.2884	36.84543
651	37.1	28.88	36.8659
609	37.12	35.1304	36.88636
526	37.14	43.9642	36.90682
433	37.16	57.6214	36.92728
338	37.18	79.826	36.94775
262	37.2	116.6954	36.96821
191	37.22	175.4528	36.98867
126	37.24	258.018	37.00914
78	37.26	339.238	37.0296
56	37.28	353.186	37.05006
38	37.3	288.772	37.07053
29	37.32	212.838	37.09099
24	37.34	162.0354	37.11145
16	37.36	140.41	37.13191
13	37.38	146.607	37.15237
12	37.4	182.2288	37.17284
12	37.42	251.31	37.1933
14	37.44	348.662	37.21376
16	37.46	426.884	37.23423
17	37.48	405.37	37.25469

15	37.5	304.912	37.27515
11	37.52	205.658	37.29562
10	37.54	135.8934	37.31608
6	37.56	92.3926	37.33654
9	37.58	66.3454	37.357
10	37.6	50.3978	37.37746
13	37.62	40.0018	37.39793
13	37.64	32.7596	37.41839
12	37.66	27.4626	37.43885
6	37.68	23.4622	37.45932
7	37.7	20.3722	37.47978
3	37.72	17.94532	37.50024
9	37.74	16.01436	37.52071
8	37.76	14.46394	37.54117
12	37.78	13.21248	37.56163
24	37.8	12.20166	37.58209
31	37.82	11.38948	37.60256
38	37.84	10.74586	37.62302
43	37.86	10.24988	37.64348
44	37.88	9.88794	37.66394
46	37.9	9.65276	37.68441
53	37.92	9.54312	37.70487
64	37.94	9.56412	37.72533
86	37.96	9.72816	37.7458
135	37.98	10.05702	37.76626
193	38	10.58514	37.78672
248	38.02	11.36554	37.80719
290	38.04	12.47956	37.82765
311	38.06	14.05564	37.84811
314	38.08	16.3095	37.86857
289	38.1	19.64022	37.88903
258	38.12	24.842	37.9095
206	38.14	33.4584	37.92996
165	38.16	48.0532	37.95042
131	38.18	72.1268	37.97089
109	38.2	107.748	37.99135
84	38.22	147.7514	38.01181
64	38.24	163.789	38.03228
53	38.26	137.8322	38.05274
55	38.28	97.1018	38.0732
58	38.3	64.4224	38.09366
67	38.32	43.1322	38.11412
73	38.34	30.3548	38.13459
86	38.36	22.8028	38.15505
105	38.38	18.1637	38.17551
128	38.4	15.1341	38.19598
140	38.42	13.05682	38.21644
175	38.44	11.59942	38.2369
212	38.46	10.57978	38.25737
277	38.48	9.8905	38.27783
392	38.5	9.4672	38.29829
574	38.52	9.27384	38.31875
877	38.54	9.29598	38.33922
1264	38.56	9.53794	38.35968
1670	38.58	10.0239	38.38014
2014	38.6	10.80242	38.4006
2247	38.62	11.95724	38.42107
2365	38.64	13.63036	38.44153

2356	38.66	16.07688	38.46199
2255	38.68	19.79428	38.48246
2115	38.7	25.7754	38.50292
2015	38.72	35.8358	38.52338
2061	38.74	52.7002	38.54385
2207	38.76	78.8402	38.56431
2397	38.78	113.8872	38.58477
2537	38.8	141.8844	38.60523
2595	38.82	136.1494	38.62569
2562	38.84	104.2004	38.64616
2458	38.86	73.125	38.66662
2246	38.88	52.6704	38.68708
1962	38.9	42.5176	38.70755
1634	38.92	40.902	38.72801
1329	38.94	46.4746	38.74847
1059	38.96	59.2242	38.76894
824	38.98	73.909	38.7894
586	39	77.0054	38.80986
397	39.02	62.9112	38.83032
261	39.04	44.3742	38.85078
187	39.06	30.0364	38.87125
135	39.08	20.6984	38.89171
115	39.1	14.97732	38.91217
88	39.12	11.45536	38.93264
72	39.14	9.16632	38.9531
66	39.16	7.57182	38.97356
62	39.18	6.39728	38.99403
55	39.2	5.49864	39.01449
61	39.22	4.7926	39.03495
58	39.24	4.22632	39.05541
52	39.26	3.7643	39.07588
44	39.28	3.3818	39.09634
44	39.3	3.06114	39.1168
41	39.32	2.78938	39.13726
39	39.34	2.55688	39.15773
46	39.36	2.35632	39.17819
61	39.38	2.18204	39.19865
88	39.4	2.02966	39.21912
115	39.42	1.895678	39.23958
147	39.44	1.777318	39.26004
206	39.46	1.67233	39.28051
307	39.48	1.578882	39.30097
451	39.5	1.495472	39.32143
627	39.52	1.420858	39.34189
800	39.54	1.354012	39.36235
945	39.56	1.294078	39.38282
1041	39.58	1.240338	39.40328
1085	39.6	1.192194	39.42374
1047	39.62	1.149148	39.44421
955	39.64	1.110786	39.46467
825	39.66	1.076766	39.48513
685	39.68	1.046812	39.5056
558	39.7	0.865592	39.52606
444	39.72	0.847328	39.54652
334	39.74	0.852472	39.56698
242	39.76	0.84154	39.58744
165	39.78	0.83399	39.60791
109	39.8	0.829874	39.62837

75	39.82	0.829306	39.64883
58	39.84	0.83246	39.6693
47	39.86	0.83959	39.68976
43	39.88	0.851036	39.71022
45	39.9	0.867248	39.73069
54	39.92	0.888812	39.75115
71	39.94	0.91649	39.77161
87	39.96	0.951266	39.79207
103	39.98	0.99443	39.81254
130	40	1.04767	39.833
188	40.02	1.113242	39.85346
281	40.04	1.194192	39.87392
407	40.06	1.294722	39.89439
554	40.08	1.420752	39.91485
677	40.1	1.580892	39.93531
790	40.12	1.788246	39.95578
853	40.14	2.06442	39.97624
876	40.16	2.4488	39.9967
848	40.18	3.01792	40.01717
804	40.2	3.91408	40.03763
763	40.22	5.36286	40.05809
735	40.24	7.63002	40.07855
726	40.26	10.78814	40.09901
718	40.28	13.89246	40.11948
679	40.3	14.68956	40.13994
630	40.32	12.85412	40.1604
577	40.34	10.87378	40.18087
526	40.36	10.20812	40.20133
459	40.38	11.3488	40.22179
399	40.4	14.79172	40.24226
344	40.42	21.2208	40.26272
282	40.44	30.868	40.28318
238	40.46	40.9192	40.30364
185	40.48	43.2864	40.3241
124	40.5	34.999	40.34457
75	40.52	24.1564	40.36503
46	40.54	15.865	40.38549
32	40.56	10.54918	40.40596
24	40.58	7.3651	40.42642
14	40.6	5.46246	40.44688
10	40.62	4.26794	40.46735
8	40.64	3.46562	40.48781
14	40.66	2.89634	40.50827
18	40.68	2.47778	40.52873
20	40.7	2.1631	40.5492
21	40.72	1.92311	40.56966
18	40.74	1.738592	40.59012
27	40.76	1.596528	40.61058
25	40.78	1.48794	40.63105
22	40.8	1.406604	40.65151
17	40.82	1.348254	40.67197
15	40.84	1.310078	40.69244
14	40.86	1.290424	40.7129
14	40.88	1.288654	40.73336
12	40.9	1.305104	40.75383
6	40.92	1.341168	40.77429
0	40.94	1.399522	40.79475
4	40.96	1.484542	40.81521

3	40.98	1.60303	40.83567
5	41	1.76547	40.85614
6	41.02	1.98842	40.8766
6	41.04	2.29968	40.89706
9	41.06	2.75066	40.91753
11	41.08	3.44348	40.93799
12	41.1	4.5765	40.95845
10	41.12	6.48454	40.97892
7	41.14	9.60424	40.99938
10	41.16	14.44158	41.01984
8	41.18	19.6022	41.0403
4	41.2	21.6794	41.06076
0	41.22	18.39282	41.08123
1	41.24	13.24218	41.10169
4	41.26	9.13518	41.12215
8	41.28	6.49276	41.14262
11	41.3	4.94704	41.16308
12	41.32	4.07966	41.18354
11	41.34	3.59772	41.20401
10	41.36	3.3379	41.22447
7	41.38	3.2202	41.24493
4	41.4	3.20716	41.26539
0	41.42	3.28204	41.28586
1	41.44	3.43984	41.30632
4	41.46	3.68426	41.32678
12	41.48	4.02716	41.34724
20	41.5	4.49004	41.36771
39	41.52	5.10748	41.38817
45	41.54	5.93348	41.40863
56	41.56	7.05464	41.4291
63	41.58	8.61952	41.44956
63	41.6	10.90894	41.47002
62	41.62	14.48416	41.49049
75	41.64	20.412	41.51095
97	41.66	30.4126	41.53141
126	41.68	47.1036	41.55187
160	41.7	70.0088	41.57233
202	41.72	92.7972	41.5928
228	41.74	96.5762	41.61326
244	41.76	77.023	41.63372
247	41.78	52.9562	41.65419
233	41.8	34.9278	41.67465
206	41.82	23.49	41.69511
187	41.84	16.91762	41.71558
158	41.86	12.88866	41.73604
126	41.88	10.3797	41.7565
99	41.9	8.71374	41.77696
74	41.92	7.55124	41.79742
55	41.94	6.675	41.81789
34	41.96	6.06808	41.83835
25	41.98	5.62544	41.85881
16	42	5.30596	41.87928
9	42.02	5.08186	41.89974
14	42.04	4.93412	41.9202
8	42.06	4.84966	41.94067
3	42.08	4.81956	41.96113
5	42.1	4.83796	41.98159
0	42.12	4.90138	42.00205

1	42.14	5.00824	42.02252
6	42.16	5.15864	42.04298
13	42.18	5.35434	42.06344
16	42.2	5.59868	42.0839
17	42.22	5.89688	42.10437
13	42.24	6.25628	42.12483
11	42.26	6.68694	42.14529
11	42.28	7.20238	42.16576
8	42.3	7.82076	42.18622
7	42.32	8.5666	42.20668
9	42.34	9.4734	42.22715
16	42.36	10.58758	42.24761
21	42.38	11.97472	42.26807
25	42.4	13.72962	42.28853
35	42.42	15.99356	42.30899
46	42.44	18.98722	42.32946
56	42.46	23.084	42.34992
63	42.48	28.984	42.37038
62	42.5	38.0824	42.39085
61	42.52	53.1376	42.41131
51	42.54	78.228	42.43177
42	42.56	118.6132	42.45224
33	42.58	175.9968	42.4727
24	42.6	233.6	42.49316
29	42.62	244.304	42.51362
33	42.64	196.206	42.53408
30	42.66	136.2208	42.55455
31	42.68	91.2536	42.57501
25	42.7	62.898	42.59547
21	42.72	46.308	42.61594
14	42.74	36.78	42.6364
14	42.76	31.2002	42.65686
19	42.78	27.8836	42.67733
19	42.8	26.0052	42.69779
37	42.82	25.1648	42.71825
54	42.84	25.1674	42.73871
75	42.86	25.9344	42.75918
118	42.88	27.4716	42.77964
182	42.9	29.8632	42.8001
270	42.92	33.2834	42.82056
356	42.94	38.0294	42.84103
446	42.96	44.5922	42.86149
506	42.98	53.8194	42.88195
534	43	67.3084	42.90242
534	43.02	88.2474	42.92288
504	43.04	122.7364	42.94334
448	43.06	180.8994	42.96381
388	43.08	274.778	42.98427
333	43.1	409.74	43.00473
283	43.12	550.256	43.02519
221	43.14	586.92	43.04565
177	43.16	481.46	43.06612
129	43.18	343.388	43.08658
96	43.2	242.974	43.10704
62	43.22	188.9106	43.12751
42	43.24	174.7156	43.14797
31	43.26	193.6166	43.16843
25	43.28	236.296	43.1889

24	43.3	272.286	43.20936
23	43.32	254.19	43.22982
14	43.34	193.0094	43.25028
15	43.36	133.6046	43.27074
13	43.38	91.5948	43.29121
9	43.4	65.0166	43.31167
0	43.42	48.7062	43.33213
5	43.44	38.4068	43.3526
17	43.46	31.4782	43.37306
23	43.48	26.515	43.39352
38	43.5	22.7974	43.41399
41	43.52	19.93156	43.43445
53	43.54	17.67924	43.45491
56	43.56	15.88494	43.47537
56	43.58	14.44304	43.49584
55	43.6	13.28008	43.5163
56	43.62	12.34444	43.53676
73	43.64	11.60006	43.55722
78	43.66	11.02244	43.57769
96	43.68	10.59648	43.59815
134	43.7	10.31522	43.61861
185	43.72	10.17978	43.63908
266	43.74	10.20026	43.65954
391	43.76	10.398	43.68
596	43.78	10.8098	43.70047
843	43.8	11.49578	43.72093
1083	43.82	12.55494	43.74139
1312	43.84	14.1604	43.76185
1459	43.86	16.64346	43.78231
1522	43.88	20.724	43.80278
1519	43.9	27.5526	43.82324
1433	43.92	39.1916	43.8437
1291	43.94	57.9926	43.86417
1130	43.96	84.4782	43.88463
989	43.98	110.0494	43.90509
830	44	112.7836	43.92556
692	44.02	89.4592	43.94602
578	44.04	61.9674	43.96648
454	44.06	41.6232	43.98694
354	44.08	28.7876	44.0074
319	44.1	20.9626	44.02787
294	44.12	16.48306	44.04833
291	44.14	13.73038	44.06879
307	44.16	11.95854	44.08926
343	44.18	10.79924	44.10972
399	44.2	10.06822	44.13018
521	44.22	9.66928	44.15065
697	44.24	9.55478	44.17111
987	44.26	9.7106	44.19157
1435	44.28	10.15204	44.21203
1984	44.3	10.92666	44.2325
2532	44.32	12.12604	44.25296
2963	44.34	13.91528	44.27342
3237	44.36	16.60604	44.29388
3326	44.38	20.8276	44.31435
3260	44.4	27.8032	44.33481
3117	44.42	39.6744	44.35527
2912	44.44	59.3606	44.37574

2744	44.46	89.0786	44.3962
2644	44.48	124.4028	44.41666
2566	44.5	142.9932	44.43713
2464	44.52	124.8022	44.45759
2284	44.54	90.0344	44.47805
2024	44.56	61.159	44.49851
1758	44.58	42.852	44.51897
1522	44.6	32.7464	44.53944
1320	44.62	29.4762	44.5599
1108	44.64	30.2776	44.58036
929	44.66	31.8026	44.60083
759	44.68	29.2836	44.62129
612	44.7	22.9614	44.64175
478	44.72	16.80344	44.66222
359	44.74	12.34614	44.68268
236	44.76	9.42644	44.70314
175	44.78	7.33006	44.7236
156	44.8	6.09584	44.74406
131	44.82	5.232	44.76453
105	44.84	4.59508	44.78499
90	44.86	4.10938	44.80545
69	44.88	3.73234	44.82592
67	44.9	3.4378	44.84638
60	44.92	3.20866	44.86684
48	44.94	3.03346	44.88731
34	44.96	2.9047	44.90777
36	44.98	2.8178	44.92823
39	45	2.77054	44.94869
45	45.02	2.7629	44.96916
45	45.04	2.79706	44.98962
60	45.06	2.8778	45.01008
88	45.08	3.0131	45.03054
141	45.1	3.21544	45.05101
208	45.12	3.50376	45.07147
298	45.14	3.907	45.09193
401	45.16	4.47074	45.1124
499	45.18	5.30176	45.13286
561	45.2	6.48216	45.15332
588	45.22	8.32342	45.17379
575	45.24	11.37286	45.19425
543	45.26	16.55224	45.21471
487	45.28	25.058	45.23517
429	45.3	37.607	45.25563
364	45.32	51.5784	45.2761
305	45.34	56.9222	45.29656
253	45.36	47.596	45.31702
205	45.38	33.305	45.33749
153	45.4	21.9014	45.35795
105	45.42	14.47744	45.37841
69	45.44	10.01064	45.39888
47	45.46	7.35224	45.41934
32	45.48	5.59416	45.4398
37	45.5	4.49422	45.46026
37	45.52	3.71716	45.48072
55	45.54	3.14634	45.50119
88	45.56	2.71668	45.52165
142	45.58	2.3885	45.54211
230	45.6	2.13628	45.56258

353	45.62	1.943624	45.58304
517	45.64	1.80031	45.6035
678	45.66	1.701206	45.62397
804	45.68	1.815002	45.64443
886	45.7	1.81681	45.66489
914	45.72	1.892104	45.68535
898	45.74	2.08586	45.70582
844	45.76	2.47348	45.72628
767	45.78	3.14734	45.74674
681	45.8	4.13992	45.7672
608	45.82	5.15458	45.78767
552	45.84	5.33708	45.80813
489	45.86	4.4456	45.82859
407	45.88	3.3306	45.84906
325	45.9	2.49576	45.86952
246	45.92	1.980142	45.88998
182	45.94	1.703004	45.91045
143	45.96	1.447622	45.93091
111	45.98	1.41516	45.95137
81	46	1.373946	45.97183
62	46.02	1.265572	45.99229
44	46.04	1.139968	46.01276
30	46.06	1.044268	46.03322
17	46.08	0.983804	46.05368
11	46.1	0.950562	46.07415
5	46.12	0.936106	46.09461
6	46.14	0.934564	46.11507
8	46.16	0.923136	46.13554
8	46.18	0.939742	46.156
9	46.2	0.96371	46.17646
10	46.22	0.994946	46.19692
9	46.24	1.033742	46.21738
5	46.26	1.08072	46.23785
8	46.28	1.136842	46.25831
8	46.3	1.203474	46.27877
5	46.32	1.282468	46.29924
3	46.34	1.376322	46.3197
0	46.36	1.488408	46.34016
1	46.38	1.62332	46.36063
11	46.4	1.787408	46.38109
33	46.42	1.98963	46.40155
67	46.44	2.2429	46.42201
107	46.46	2.56646	46.44248
149	46.48	2.99042	46.46294
181	46.5	3.58488	46.4834
196	46.52	4.40866	46.50386
199	46.54	5.67196	46.52433
187	46.56	7.73606	46.54479
171	46.58	11.17588	46.56525
156	46.6	16.66196	46.58572
138	46.62	24.3364	46.60618
113	46.64	31.7238	46.62664
97	46.66	32.5756	46.64711
84	46.68	26.0466	46.66757
68	46.7	18.38176	46.68803
47	46.72	12.7761	46.70849
30	46.74	9.32528	46.72895
18	46.76	7.386	46.74942

15	46.78	6.36076	46.76988
10	46.8	5.80994	46.79034
5	46.82	5.64262	46.81081
0	46.84	5.71236	46.83127
0	46.86	5.9857	46.85173
7	46.88	6.46114	46.8722
10	46.9	7.16162	46.89266
14	46.92	8.13576	46.91312
19	46.94	9.4662	46.93358
20	46.96	11.52704	46.95404
19	46.98	14.07874	46.97451
17	47	17.78324	46.99497
17	47.02	23.4974	47.01543
15	47.04	32.8872	47.0359
11	47.06	48.7692	47.05636
11	47.08	74.8216	47.07682
7	47.1	113.7308	47.09729
9	47.12	156.9482	47.11775
9	47.14	174.2628	47.13821
7	47.16	146.2516	47.15867
4	47.18	102.3104	47.17914
6	47.2	67.0524	47.1996
5	47.22	44.0686	47.22006
6	47.24	30.2516	47.24052
7	47.26	22.0524	47.26099
6	47.28	16.97318	47.28145
3	47.3	13.60476	47.30191
7	47.32	11.2297	47.32238
8	47.34	9.4921	47.34284
7	47.36	8.1839	47.3633
5	47.38	7.17962	47.38377
6	47.4	6.39698	47.40423
2	47.42	5.78002	47.42469
4	47.44	5.28972	47.44515
4	47.46	4.8979	47.46561
6	47.48	4.58512	47.48608
7	47.5	4.33628	47.50654
10	47.52	4.14044	47.527
10	47.54	3.9895	47.54747
7	47.56	3.8775	47.56793
3	47.58	3.8002	47.58839
7	47.6	3.75468	47.60886
3	47.62	3.7393	47.62932
9	47.64	3.75354	47.64978
16	47.66	3.79804	47.67024
25	47.68	3.8749	47.6907
40	47.7	3.98788	47.71117
56	47.72	4.14318	47.73163
74	47.74	4.35044	47.75209
101	47.76	4.76146	47.77256
120	47.78	5.13266	47.79302
140	47.8	5.64336	47.81348
152	47.82	6.3854	47.83395
155	47.84	7.53714	47.85441
146	47.86	9.40256	47.87487
130	47.88	12.38596	47.89533
110	47.9	16.76364	47.9158
91	47.92	21.794	47.93626

78	47.94	24.2402	47.95672
73	47.96	21.705	47.97718
67	47.98	17.24144	47.99765
67	48	13.70838	48.01811
66	48.02	11.59192	48.03857
66	48.04	10.5766	48.05904
70	48.06	10.28284	48.0795
76	48.08	10.44708	48.09996
90	48.1	10.93224	48.12043
105	48.12	11.6874	48.14089
131	48.14	12.68218	48.16135
170	48.16	14.00836	48.18181
252	48.18	15.69754	48.20227
365	48.2	18.04798	48.22274
508	48.22	20.8166	48.2432
669	48.24	24.4334	48.26366
817	48.26	29.2902	48.28413
915	48.28	36.104	48.30459
959	48.3	46.2792	48.32505
931	48.32	62.473	48.34552
869	48.34	89.022	48.36598
783	48.36	131.3006	48.38644
690	48.38	191.7658	48.4069
615	48.4	255.736	48.42736
541	48.42	277.338	48.44783
460	48.44	241.168	48.46829
393	48.46	195.8712	48.48875
301	48.48	175.4978	48.50922
214	48.5	190.2136	48.52968
137	48.52	244.272	48.55014
90	48.54	335.114	48.57061
60	48.56	427.406	48.59107
34	48.58	435.548	48.61153
31	48.6	343.88	48.63199
21	48.62	235.92	48.65246
13	48.64	155.5956	48.67292
18	48.66	104.477	48.69338
8	48.68	73.7758	48.71384
6	48.7	55.2216	48.73431
7	48.72	43.3834	48.75477
11	48.74	35.3062	48.77523
10	48.76	29.5002	48.7957
6	48.78	25.1852	48.81616
5	48.8	21.9108	48.83662
9	48.82	19.3942	48.85709
9	48.84	17.4486	48.87755
8	48.86	15.94856	48.89801
5	48.88	14.80944	48.91847
4	48.9	13.9757	48.93893
1	48.92	13.41408	48.9594
3	48.94	13.11032	48.97986
5	48.96	13.06848	49.00032
14	48.98	13.46498	49.02079
31	49	14.05322	49.04125
48	49.02	15.07348	49.06171
69	49.04	16.68994	49.08218
82	49.06	19.21136	49.10264
87	49.08	23.2574	49.1231

83	49.1	30.0486	49.14356
77	49.12	41.6978	49.16402
74	49.14	61.0742	49.18449
70	49.16	90.308	49.20495
73	49.18	124.8302	49.22541
77	49.2	142.3696	49.24588
71	49.22	123.6948	49.26634
69	49.24	89.0824	49.2868
64	49.26	60.1774	49.30727
68	49.28	41.1212	49.32773
75	49.3	29.7036	49.34819
88	49.32	23.0754	49.36865
102	49.34	19.16586	49.38912
141	49.36	16.79268	49.40958
195	49.38	15.36762	49.43004
284	49.4	14.59354	49.4505
390	49.42	14.38012	49.47097
560	49.44	14.77182	49.49143
755	49.46	15.58384	49.51189
983	49.48	17.0109	49.53236
1185	49.5	19.24224	49.55282
1351	49.52	22.6432	49.57328
1469	49.54	27.948	49.59375
1575	49.56	36.6386	49.61421
1685	49.58	51.4442	49.63467
1876	49.6	76.4122	49.65513
2145	49.62	115.677	49.67559
2489	49.64	167.5454	49.69606
2791	49.66	208.178	49.71652
2989	49.68	197.6932	49.73698
3029	49.7	148.0244	49.75745
2935	49.72	99.2842	49.77791
2697	49.74	65.1932	49.79837
2390	49.76	44.1076	49.81884
2078	49.78	31.5994	49.8393
1815	49.8	24.0266	49.85976
1600	49.82	19.14946	49.88022
1402	49.84	15.79312	49.90068
1177	49.86	13.36936	49.92115
914	49.88	11.32978	49.94161
668	49.9	9.96508	49.96207
455	49.92	8.91548	49.98254
269	49.94	8.10206	50.003
146	49.96	7.47096	
78	49.98	6.98482	
42	50	6.6177	
22	50.02	6.35178	
10	50.04	6.1753	
0	50.06	5.68348	
0	50.08	5.67998	
0	50.1	5.75728	
0	50.12	5.92086	
0	50.14	6.1811	
11	50.16	6.55446	
38	50.18	7.06572	
95	50.2	7.79644	
176	50.22	8.71166	
303	50.24	9.93634	

	438	50.26	11.59684
	560	50.28	13.904
	641	50.3	17.24606
	667	50.32	22.3942
	649	50.34	30.846
	596	50.36	45.1316
	517	50.38	68.557
	439	50.4	103.1742
	373	50.42	142.04
	327	50.44	157.6772
	280	50.46	132.6032
	228	50.48	93.2048
	165	50.5	61.6146
	102	50.52	41.073
	54	50.54	28.795
	14	50.56	21.5976
	0	50.58	17.24442
	0	50.6	14.47934
	0	50.62	12.6755
	40	50.64	11.5253
	107	50.66	10.84746
	219	50.68	10.6279
	368	50.7	10.81972
	521	50.72	11.46496
	624	50.74	12.6829
	673	50.76	14.73406
	667	50.78	18.16758
	596	50.8	24.0632
	502	50.82	34.2294
	346	50.84	50.9692
	261	50.86	75.5
	184	50.88	102.1012
	184	50.9	110.8492
	184	50.92	91.958
--		50.94	65.048
--		50.96	44.4414
--		50.98	32.0246
--		51	26.0184
--		51.02	24.1912
--		51.04	23.7158
--		51.06	21.249
--		51.08	16.79606
--		51.1	12.65092
--		51.12	9.65486
--		51.14	7.46718
--		51.16	6.16852
--		51.18	5.29202
--		51.2	4.6706
--		51.22	4.21178
--		51.24	3.86588
--		51.26	3.60452
--		51.28	3.41032
--		51.3	3.27232
--		51.32	3.18392
--		51.34	3.14182
--		51.36	3.14564
--		51.38	3.24094
--		51.4	3.3486

--	51.42	3.52064
--	51.44	3.77316
--	51.46	4.13146
--	51.48	4.63604
--	51.5	5.35648
--	51.52	6.42396
--	51.54	8.10074
--	51.56	10.88674
--	51.58	15.59302
--	51.6	23.2012
--	51.62	34.0578
--	51.64	45.0542
--	51.66	47.2654
--	51.68	38.219
--	51.7	26.7988
--	51.72	18.21242
--	51.74	12.80674
--	51.76	9.66744
--	51.78	7.90004
--	51.8	6.91298
--	51.82	6.38988
--	51.84	6.24912
--	51.86	6.29776
--	51.88	6.5856
--	51.9	6.9814
--	51.92	7.84532
--	51.94	9.13094
--	51.96	11.04504
--	51.98	14.0027
--	52	19.03722
--	52.02	27.2458
--	52.04	40.9938
--	52.06	62.2972
--	52.08	89.4634
--	52.1	108.2846
--	52.12	99.435
--	52.14	72.9252
--	52.16	48.5112
--	52.18	31.7988
--	52.2	21.5566
--	52.22	15.49188
--	52.24	11.80498
--	52.26	9.4159
--	52.28	7.76498
--	52.3	6.57084
--	52.32	5.68162
--	52.34	5.00656
--	52.36	4.38312
--	52.38	3.98354
--	52.4	3.6739
--	52.42	3.4355
--	52.44	3.25524
--	52.46	3.12404
--	52.48	3.03574
--	52.5	2.9866
--	52.52	2.97486
--	52.54	2.9888
--	52.56	3.1478

--	52.58	3.26044
--	52.6	3.42496
--	52.62	3.65312
--	52.64	3.96292
--	52.66	4.38198
--	52.68	4.9548
--	52.7	5.75852
--	52.72	6.94098
--	52.74	8.79722
--	52.76	11.87584
--	52.78	17.02618
--	52.8	25.1816
--	52.82	36.3092
--	52.84	46.1492
--	52.86	45.781
--	52.88	35.7788
--	52.9	25.072
--	52.92	17.44524
--	52.94	12.7815
--	52.96	10.14076
--	52.98	8.70298
--	53	7.9533
--	53.02	7.93276
--	53.04	7.91346
--	53.06	8.14534
--	53.08	8.62006
--	53.1	9.36056
--	53.12	10.42446
--	53.14	11.90712
--	53.16	14.05522
--	53.18	17.27284
--	53.2	22.3512
--	53.22	30.5618
--	53.24	43.447
--	53.26	61.6104
--	53.28	80.5048
--	53.3	87.3434
--	53.32	79.9424
--	53.34	73.2918
--	53.36	77.9082
--	53.38	98.4368
--	53.4	136.7484
--	53.42	185.1782
--	53.44	210.486
--	53.46	182.7144
--	53.48	130.65
--	53.5	86.8124
--	53.52	57.665
--	53.54	39.9638
--	53.56	29.4354
--	53.58	22.8436
--	53.6	18.5828
--	53.62	15.62142
--	53.64	13.4894
--	53.66	11.92828
--	53.68	10.78234
--	53.7	9.95286
--	53.72	9.4068

--	53.74	9.0457
--	53.76	8.87824
--	53.78	8.89932
--	53.8	9.11938
--	53.82	9.56718
--	53.84	10.29694
--	53.86	11.40432
--	53.88	13.06462
--	53.9	15.62352
--	53.92	19.77414
--	53.94	26.7848
--	53.96	38.5558
--	53.98	57.016
--	54	81.3972
--	54.02	100.6294
--	54.04	96.0976
--	54.06	73.28
--	54.08	50.9124
--	54.1	35.4426
--	54.12	26.1296
--	54.14	20.9286
--	54.16	18.2505
--	54.18	16.9202
--	54.2	16.52048
--	54.22	16.84598
--	54.24	17.8496
--	54.26	19.59068
--	54.28	22.236
--	54.3	26.1072
--	54.32	31.8274
--	54.34	40.5624
--	54.36	54.8566
--	54.38	79.0472
--	54.4	119.4354
--	54.42	181.7376
--	54.44	260.442
--	54.46	313.314
--	54.48	286.08
--	54.5	210.412
--	54.52	142.6392
--	54.54	98.418
--	54.56	74.5482
--	54.58	64.3676
--	54.6	59.9134
--	54.62	52.4778
--	54.64	41.1506
--	54.66	30.7978
--	54.68	23.2688
--	54.7	18.1897
--	54.72	14.7891
--	54.74	12.43366
--	54.76	10.71752
--	54.78	9.34818
--	54.8	8.32744
--	54.82	7.51528
--	54.84	6.8637
--	54.86	6.45724
--	54.88	6.04336

--	54.9	5.52462
--	54.92	5.29032
--	54.94	5.1347
--	54.96	5.05918
--	54.98	5.07132
--	55	5.18714
--	55.02	5.43624
--	55.04	5.87428
--	55.06	6.61232
--	55.08	7.87436
--	55.1	10.07108
--	55.12	13.81806
--	55.14	19.73962
--	55.16	27.5834
--	55.18	33.7528
--	55.2	32.215
--	55.22	24.7604
--	55.24	17.46002
--	55.26	12.3984
--	55.28	9.33314
--	55.3	7.7207
--	55.32	6.78146
--	55.34	6.30434
--	55.36	6.1269
--	55.38	6.18158
--	55.4	6.4519
--	55.42	6.95582
--	55.44	7.74658
--	55.46	8.93026
--	55.48	10.71548
--	55.5	13.43394
--	55.52	18.10168
--	55.54	26.0268
--	55.56	39.0412
--	55.58	58.2764
--	55.6	79.8592
--	55.62	88.5632
--	55.64	74.8672
--	55.66	53.521
--	55.68	36.8598
--	55.7	26.8506
--	55.72	22.1044
--	55.74	20.5598
--	55.76	19.41182
--	55.78	16.4966
--	55.8	12.85324
--	55.82	9.91262
--	55.84	7.90476
--	55.86	6.6145
--	55.88	5.7949
--	55.9	5.26722
--	55.92	4.92608
--	55.94	4.42202
--	55.96	4.32152
--	55.98	4.3083
--	56	4.37642
--	56.02	4.5448
--	56.04	4.78322

--	56.06	5.12246
--	56.08	5.5833
--	56.1	6.17096
--	56.12	6.9875
--	56.14	8.07918
--	56.16	9.56338
--	56.18	11.64272
--	56.2	14.70328
--	56.22	19.51612
--	56.24	27.5254
--	56.26	40.9962
--	56.28	62.4902
--	56.3	92.2234
--	56.32	119.5632
--	56.34	120.243
--	56.36	93.5986
--	56.38	63.8988
--	56.4	42.3436
--	56.42	28.9068
--	56.44	21.128
--	56.46	16.58184
--	56.48	13.88716
--	56.5	12.27772
--	56.52	11.38994
--	56.54	11.06222
--	56.56	11.24214
--	56.58	12.10056
--	56.6	13.4847
--	56.62	15.82546
--	56.64	19.75146
--	56.66	26.472
--	56.68	38.1168
--	56.7	57.2694
--	56.72	85.231
--	56.74	115.1798
--	56.76	124.19
--	56.78	101.8526
--	56.8	70.7076
--	56.82	46.4742
--	56.84	30.8396
--	56.86	21.4566
--	56.88	15.85644
--	56.9	12.34872
--	56.92	9.9942
--	56.94	8.31938
--	56.96	7.08068
--	56.98	6.14036
--	57	5.41288
--	57.02	4.84198
--	57.04	4.38932
--	57.06	4.0282
--	57.08	3.65656
--	57.1	3.42954
--	57.12	3.25218
--	57.14	3.11756
--	57.16	3.02148
--	57.18	2.96166
--	57.2	2.9377

--	57.22	2.94172
--	57.24	2.99754
--	57.26	3.10314
--	57.28	3.27192
--	57.3	3.5271
--	57.32	3.9118
--	57.34	4.51206
--	57.36	5.49652
--	57.38	7.15526
--	57.4	9.87924
--	57.42	13.95012
--	57.44	18.71048
--	57.46	21.0902
--	57.48	18.58558
--	57.5	14.03478
--	57.52	10.31552
--	57.54	7.95596
--	57.56	6.65574
--	57.58	6.0382
--	57.6	5.83648
--	57.62	5.90714
--	57.64	6.19154
--	57.66	6.71812
--	57.68	7.43584
--	57.7	8.43692
--	57.72	9.8237
--	57.74	11.78704
--	57.76	14.70162
--	57.78	19.29088
--	57.8	26.788
--	57.82	38.739
--	57.84	56.601
--	57.86	77.6432
--	57.88	89.5672
--	57.9	83.1784
--	57.92	71.5722
--	57.94	68.4976
--	57.96	78.9626
--	57.98	103.713
--	58	135.5446
--	58.02	149.0984
--	58.04	126.1054
--	58.06	89.0976
--	58.08	58.987
--	58.1	39.1508
--	58.12	27.0872
--	58.14	19.83318
--	58.16	15.27508
--	58.18	12.20744
--	58.2	10.01482
--	58.22	8.26252
--	58.24	7.01302
--	58.26	6.03442
--	58.28	5.25416
--	58.3	4.62244
--	58.32	4.10424
--	58.34	3.67436
--	58.36	3.3143

--	58.38	3.01032
--	58.4	2.75204
--	58.42	2.5316
--	58.44	2.34314
--	58.46	2.1826
--	58.48	2.0477
--	58.5	1.938024
--	58.52	1.854346
--	58.54	1.796012
--	58.56	1.75166
--	58.58	1.690456
--	58.6	1.6015
--	58.62	1.513424
--	58.64	1.558134
--	58.66	1.51169
--	58.68	1.4821
--	58.7	1.46652
--	58.72	1.463362
--	58.74	1.472264
--	58.76	1.49381
--	58.78	1.541942
--	58.8	1.593892
--	58.82	1.664944
--	58.84	1.759356
--	58.86	1.883162
--	58.88	2.04504
--	58.9	2.25766
--	58.92	2.52172
--	58.94	2.90126
--	58.96	3.41946
--	58.98	4.14196
--	59	5.19518
--	59.02	6.8628
--	59.04	9.67462
--	59.06	14.40656
--	59.08	21.838
--	59.1	31.6506
--	59.12	39.3476
--	59.14	37.3966
--	59.16	28.0392
--	59.18	18.85136
--	59.2	12.42786
--	59.22	8.45968
--	59.24	6.1109
--	59.26	4.69392
--	59.28	3.78584
--	59.3	3.1649
--	59.32	2.8238
--	59.34	2.4257
--	59.36	2.18434
--	59.38	2.00252
--	59.4	1.86535
--	59.42	1.76266
--	59.44	1.687368
--	59.46	1.634488
--	59.48	1.46354
--	59.5	1.449698
--	59.52	1.450536

--	59.54	1.465052
--	59.56	1.492764
--	59.58	1.533648
--	59.6	1.588112
--	59.62	1.656996
--	59.64	1.741606
--	59.66	1.843786
--	59.68	1.966014
--	59.7	2.16462
--	59.72	2.33924
--	59.74	2.54714
--	59.76	2.79574
--	59.78	3.09486
--	59.8	3.45776
--	59.82	3.90256
--	59.84	4.45456
--	59.86	5.14962
--	59.88	6.04022
--	59.9	7.20554
--	59.92	8.77244
--	59.94	10.96346
--	59.96	14.20908
--	59.98	19.36362
--	60	27.976
--	60.02	42.3396
--	60.04	64.7216
--	60.06	93.9362
--	60.08	116.1304
--	60.1	109.5768
--	60.12	82.3594
--	60.14	56.6538
--	60.16	39.8154
--	60.18	31.0136
--	60.2	27.4882
--	60.22	25.3788
--	60.24	21.395
--	60.26	16.443
--	60.28	12.39392
--	60.3	9.5984
--	60.32	7.77938
--	60.34	6.60512
--	60.36	5.8313
--	60.38	5.31136
--	60.4	4.98774
--	60.42	4.7773
--	60.44	4.67908
--	60.46	4.68326
--	60.48	4.78864
--	60.5	5.00232
--	60.52	5.34006
--	60.54	5.82918
--	60.56	6.51322
--	60.58	7.46114
--	60.6	8.74886
--	60.62	10.65414
--	60.64	13.5329
--	60.66	18.16988
--	60.68	25.9848

--	60.7	39.0678
--	60.72	59.4472
--	60.74	85.8758
--	60.76	105.3802
--	60.78	98.417
--	60.8	73.0386
--	60.82	48.9346
--	60.84	32.3092
--	60.86	22.1356
--	60.88	16.18182
--	60.9	12.66046
--	60.92	10.49256
--	60.94	9.19384
--	60.96	8.36716
--	60.98	7.95928
--	61	7.93212
--	61.02	8.3182
--	61.04	9.25094
--	61.06	11.0482
--	61.08	14.33858
--	61.1	20.1296
--	61.12	29.5778
--	61.14	42.8236
--	61.16	55.1912
--	61.18	55.6632
--	61.2	43.546
--	61.22	29.8446
--	61.24	19.80712
--	61.26	13.4717
--	61.28	9.6865
--	61.3	7.4038
--	61.32	5.94692
--	61.34	4.95178
--	61.36	4.23576
--	61.38	3.70324
--	61.4	3.29888
--	61.42	2.98782
--	61.44	2.78046
--	61.46	2.59544
--	61.48	2.45462
--	61.5	2.35086
--	61.52	2.27948
--	61.54	2.2379
--	61.56	2.11546
--	61.58	2.13552
--	61.6	2.18818
--	61.62	2.27882
--	61.64	2.41648
--	61.66	2.6157
--	61.68	2.88846
--	61.7	3.3037
--	61.72	3.93514
--	61.74	4.95392
--	61.76	6.6647
--	61.78	9.5038
--	61.8	13.86262
--	61.82	19.34094
--	61.84	22.996

--	61.86	21.09
--	61.88	15.88382
--	61.9	11.21752
--	61.92	8.1104
--	61.94	6.2999
--	61.96	5.33846
--	61.98	4.88436
--	62	4.76896
--	62.02	4.8501
--	62.04	5.12704
--	62.06	5.6111
--	62.08	6.34898
--	62.1	7.44046
--	62.12	9.08932
--	62.14	11.70292
--	62.16	16.01428
--	62.18	23.0974
--	62.2	33.9942
--	62.22	48.0816
--	62.24	58.7914
--	62.26	57.145
--	62.28	48.071
--	62.3	42.3288
--	62.32	44.2362
--	62.34	54.2002
--	62.36	68.3422
--	62.38	74.0462
--	62.4	62.4288
--	62.42	44.3242
--	62.44	29.6704
--	62.46	20.0206
--	62.48	14.14484
--	62.5	10.61822
--	62.52	8.39956
--	62.54	6.9176
--	62.56	5.8757
--	62.58	5.1219
--	62.6	4.57186
--	62.62	4.1751
--	62.64	3.90072
--	62.66	3.67928
--	62.68	3.60802
--	62.7	3.63546
--	62.72	3.77376
--	62.74	4.04972
--	62.76	4.51596
--	62.78	5.27832
--	62.8	6.55304
--	62.82	8.74852
--	62.84	12.50828
--	62.86	18.56252
--	62.88	26.9804
--	62.9	34.7534
--	62.92	35.0054
--	62.94	27.3798
--	62.96	18.85062
--	62.98	12.64252
--	63	8.75634

--	63.02	6.46826
--	63.04	5.13668
--	63.06	4.32286
--	63.08	3.8159
--	63.1	3.50952
--	63.12	3.35462
--	63.14	3.33282
--	63.16	3.44936
--	63.18	3.74002
--	63.2	4.29232
--	63.22	5.27668
--	63.24	6.95836
--	63.26	9.62742
--	63.28	13.26532
--	63.3	16.55146
--	63.32	16.95502
--	63.34	15.18234
--	63.36	14.2597
--	63.38	15.77126
--	63.4	20.085
--	63.42	26.0866
--	63.44	29.2336
--	63.46	25.3898
--	63.48	18.31098
--	63.5	12.34806
--	63.52	8.37186
--	63.54	5.94576
--	63.56	4.49964
--	63.58	3.59896
--	63.6	3.00424
--	63.62	2.58988
--	63.64	2.29216
--	63.66	2.0761
--	63.68	1.920962
--	63.7	1.813998
--	63.72	1.698436
--	63.74	1.670586
--	63.76	1.67972
--	63.78	1.729534
--	63.8	1.828924
--	63.82	1.995936
--	63.84	2.20148
--	63.86	2.65434
--	63.88	3.42596
--	63.9	4.72818
--	63.92	6.78388
--	63.94	9.5409
--	63.96	11.8566
--	63.98	11.54348
--	64	9.04836
--	64.02	6.52646
--	64.04	4.78756
--	64.06	3.77214
--	64.08	3.25344
--	64.1	3.04358
--	64.12	3.03226
--	64.14	3.17212
--	64.16	3.45614

--	64.18	3.9058
--	64.2	4.57374
--	64.22	5.56594
--	64.24	7.09802
--	64.26	9.5992
--	64.28	13.8255
--	64.3	20.8362
--	64.32	31.516
--	64.34	44.6076
--	64.36	52.5116
--	64.38	47.1168
--	64.4	34.9302
--	64.42	24.8998
--	64.44	19.22374
--	64.46	17.2384
--	64.48	17.08562
--	64.5	15.8312
--	64.52	12.5368
--	64.54	9.0764
--	64.56	6.49912
--	64.58	4.79732
--	64.6	3.72094
--	64.62	3.01342
--	64.64	2.52774
--	64.66	2.17594
--	64.68	1.911498
--	64.7	1.708636
--	64.72	1.551856
--	64.74	1.431278
--	64.76	1.340576
--	64.78	1.275986
--	64.8	1.223434
--	64.82	1.208534
--	64.84	1.22151
--	64.86	1.269356
--	64.88	1.366858
--	64.9	1.518694
--	64.92	1.838768
--	64.94	2.4012
--	64.96	3.33374
--	64.98	4.70008
--	65	6.22034
--	65.02	6.67292
--	65.04	5.60232
--	65.06	4.10912
--	65.08	2.96112
--	65.1	2.26216
--	65.12	1.860782
--	65.14	1.659188
--	65.16	1.579448
--	65.18	1.582218
--	65.2	1.653866
--	65.22	1.796856
--	65.24	2.02748
--	65.26	2.38204
--	65.28	2.93614
--	65.3	3.84376
--	65.32	5.38694

--	65.34	7.98572
--	65.36	12.05864
--	65.38	17.40004
--	65.4	21.4864
--	65.42	20.2706
--	65.44	15.13198
--	65.46	10.17124
--	65.48	6.72014
--	65.5	4.59282
--	65.52	3.33472
--	65.54	2.5759
--	65.56	2.09004
--	65.58	1.758906
--	65.6	1.523228
--	65.62	1.351406
--	65.64	1.23388
--	65.66	1.141004
--	65.68	1.073866
--	65.7	1.027354
--	65.72	0.998144
--	65.74	0.984206
--	65.76	0.98455
--	65.78	0.999104
--	65.8	1.028726
--	65.82	1.02584
--	65.84	1.094244
--	65.86	1.188514
--	65.88	1.317564
--	65.9	1.496656
--	65.92	1.75442
--	65.94	2.1479
--	65.96	2.78614
--	65.98	3.83746
--	66	5.60618
--	66.02	8.04944
--	66.04	10.59326
--	66.06	11.27814
--	66.08	9.4108
--	66.1	6.93794
--	66.12	5.09206
--	66.14	3.98626
--	66.16	3.41804
--	66.18	3.19722
--	66.2	3.19952
--	66.22	3.36786
--	66.24	3.69118
--	66.26	4.19076
--	66.28	4.92164
--	66.3	5.99488
--	66.32	7.63584
--	66.34	10.29226
--	66.36	14.75214
--	66.38	22.1206
--	66.4	33.3276
--	66.42	47.0934
--	66.44	55.5788
--	66.46	50.4098
--	66.48	38.462

--	66.5	29.1624
--	66.52	25.0294
--	66.54	25.3504
--	66.56	27.2202
--	66.58	25.6614
--	66.6	20.0044
--	66.62	14.13064
--	66.64	9.88142
--	66.66	7.12326
--	66.68	5.4142
--	66.7	4.33576
--	66.72	3.6178
--	66.74	3.1126
--	66.76	2.74382
--	66.78	2.4701
--	66.8	2.26708
--	66.82	2.1196
--	66.84	2.0183
--	66.86	1.958132
--	66.88	1.917298
--	66.9	1.939162
--	66.92	2.01014
--	66.94	2.14616
--	66.96	2.3808
--	66.98	2.78268
--	67	3.47656
--	67.02	4.67324
--	67.04	6.49418
--	67.06	8.84608
--	67.08	10.57102
--	67.1	9.9856
--	67.12	7.84586
--	67.14	5.8672
--	67.16	4.57996
--	67.18	3.87626
--	67.2	3.58014
--	67.22	3.54972
--	67.24	3.71026
--	67.26	4.04084
--	67.28	4.55898
--	67.3	5.318
--	67.32	6.42488
--	67.34	8.09376
--	67.36	10.75806
--	67.38	15.22748
--	67.4	22.7576
--	67.42	34.73
--	67.44	51.1004
--	67.46	65.5804
--	67.48	64.9922
--	67.5	50.2096
--	67.52	34.4944
--	67.54	23.55
--	67.56	17.24122
--	67.58	13.9807
--	67.6	11.81258
--	67.62	9.5088
--	67.64	7.36036

--	67.66	5.73984
--	67.68	4.63202
--	67.7	3.87808
--	67.72	3.365
--	67.74	3.00796
--	67.76	2.75674
--	67.78	2.58356
--	67.8	2.47324
--	67.82	2.41792
--	67.84	2.41478
--	67.86	2.46534
--	67.88	2.57578
--	67.9	2.7068
--	67.92	2.98318
--	67.94	3.38698
--	67.96	3.98064
--	67.98	4.88598
--	68	6.347
--	68.02	8.8133
--	68.04	12.9678
--	68.06	19.56786
--	68.08	28.363
--	68.1	35.6336
--	68.12	34.465
--	68.14	26.2398
--	68.16	17.8931
--	68.18	12.01936
--	68.2	8.41352
--	68.22	6.32924
--	68.24	5.13988
--	68.26	4.45996
--	68.28	4.0941
--	68.3	3.95676
--	68.32	4.02208
--	68.34	4.30812
--	68.36	4.89004
--	68.38	5.94442
--	68.4	7.81472
--	68.42	11.0408
--	68.44	16.22112
--	68.46	23.3458
--	68.48	29.7244
--	68.5	29.7162
--	68.52	23.777
--	68.54	17.76884
--	68.56	14.3136
--	68.58	13.47226
--	68.6	14.1176
--	68.62	13.86456
--	68.64	11.38766
--	68.66	8.34388
--	68.68	5.9601
--	68.7	4.38464
--	68.72	3.40072
--	68.74	2.78478
--	68.76	2.38258
--	68.78	2.1066
--	68.8	1.911532

--	68.82	1.773572
--	68.84	1.679414
--	68.86	1.62146
--	68.88	1.595868
--	68.9	1.601788
--	68.92	1.641334
--	68.94	1.720238
--	68.96	1.787882
--	68.98	1.991542
--	69	2.31162
--	69.02	2.8361
--	69.04	3.72542
--	69.06	5.19334
--	69.08	7.45228
--	69.1	10.2891
--	69.12	12.17746
--	69.14	11.21726
--	69.16	8.5803
--	69.18	6.29694
--	69.2	4.77256
--	69.22	3.93254
--	69.24	3.55278
--	69.26	3.46656
--	69.28	3.58628
--	69.3	3.88546
--	69.32	4.3803
--	69.34	5.12748
--	69.36	6.24646
--	69.38	7.98366
--	69.4	10.8328
--	69.42	15.66922
--	69.44	23.7292
--	69.46	36.0682
--	69.48	51.3146
--	69.5	60.696
--	69.52	54.274
--	69.54	39.209
--	69.56	25.945
--	69.58	16.97296
--	69.6	11.53284
--	69.62	8.3155
--	69.64	6.35396
--	69.66	5.0788
--	69.68	4.22202
--	69.7	3.58802
--	69.72	3.12056
--	69.74	2.77172
--	69.76	2.51084
--	69.78	2.31782
--	69.8	2.1794
--	69.82	2.08712
--	69.84	2.0361
--	69.86	2.02448
--	69.88	2.05324
--	69.9	2.12662
--	69.92	2.25284
--	69.94	2.44602
--	69.96	2.7298

--	69.98	3.11914
--	70	3.74804
--	70.02	4.7495
--	70.04	6.43416
--	70.06	9.27354
--	70.08	13.83228
--	70.1	20.258
--	70.12	26.4694
--	70.14	27.1754
--	70.16	21.5892
--	70.18	15.0035
--	70.2	10.13764
--	70.22	7.08994
--	70.24	5.31354
--	70.26	4.29732
--	70.28	3.70892
--	70.3	3.37132
--	70.32	3.20012
--	70.34	3.15746
--	70.36	3.23062
--	70.38	3.426
--	70.4	3.7728
--	70.42	4.33186
--	70.44	5.20196
--	70.46	6.50788
--	70.48	8.32724
--	70.5	10.45978
--	70.52	12.52096
--	70.54	15.25174
--	70.56	20.4262
--	70.58	29.615
--	70.6	43.2836
--	70.62	57.4576
--	70.64	60.4594
--	70.66	48.634
--	70.68	33.4806
--	70.7	21.9738
--	70.72	14.62662
--	70.74	10.24322
--	70.76	7.63698
--	70.78	6.01346
--	70.8	4.9372
--	70.82	4.19032
--	70.84	3.66144
--	70.86	3.28894
--	70.88	3.03682
--	70.9	2.88474
--	70.92	2.82374
--	70.94	2.85506
--	70.96	2.933
--	70.98	3.20654
--	71	3.68016
--	71.02	4.48876
--	71.04	5.89484
--	71.06	8.37366
--	71.08	12.37154
--	71.1	18.14962
--	71.12	24.1382

--	71.14	25.5512
--	71.16	20.7446
--	71.18	14.44998
--	71.2	9.64582
--	71.22	6.57722
--	71.24	4.7535
--	71.26	3.6815
--	71.28	3.03032
--	71.3	2.62
--	71.32	2.36336
--	71.34	2.22078
--	71.36	2.17876
--	71.38	2.24598
--	71.4	2.46142
--	71.42	2.90934
--	71.44	3.72518
--	71.46	5.05904
--	71.48	6.90262
--	71.5	8.56646
--	71.52	8.69868
--	71.54	7.57978
--	71.56	6.77244
--	71.58	7.02702
--	71.6	8.37512
--	71.62	10.01432
--	71.64	10.11028
--	71.66	8.116
--	71.68	5.75992
--	71.7	3.97354
--	71.72	2.82678
--	71.74	2.13184
--	71.76	1.708668
--	71.78	1.43776
--	71.8	1.253512
--	71.82	1.12266
--	71.84	1.027792
--	71.86	0.958934
--	71.88	0.909946
--	71.9	0.87698
--	71.92	0.857728
--	71.94	0.851084
--	71.96	0.857054
--	71.98	0.87706
--	72	0.911518
--	72.02	1.004438
--	72.04	1.11184
--	72.06	1.287296
--	72.08	1.560074
--	72.1	1.933852
--	72.12	2.23568
--	72.14	2.30988
--	72.16	2.09016
--	72.18	1.85096
--	72.2	1.72282
--	72.22	1.711098
--	72.24	1.794636
--	72.26	1.957942
--	72.28	2.19902

--	72.3	2.53032
--	72.32	2.97976
--	72.34	3.5974
--	72.36	4.4752
--	72.38	5.79428
--	72.4	7.91278
--	72.42	11.46506
--	72.44	17.3528
--	72.46	26.3684
--	72.48	37.6136
--	72.5	44.8888
--	72.52	40.8296
--	72.54	30.457
--	72.56	21.6452
--	72.58	16.49468
--	72.6	14.34548
--	72.62	13.46068
--	72.64	11.6607
--	72.66	8.9417
--	72.68	6.54108
--	72.7	4.85094
--	72.72	3.75424
--	72.74	3.05584
--	72.76	2.6016
--	72.78	2.29588
--	72.8	2.08678
--	72.82	1.947342
--	72.84	1.863376
--	72.86	1.827906
--	72.88	1.838964
--	72.9	1.899078
--	72.92	2.01586
--	72.94	2.20394
--	72.96	2.4896
--	72.98	2.9223
--	73	3.59446
--	73.02	4.73758
--	73.04	6.64616
--	73.06	9.82134
--	73.08	14.65528
--	73.1	20.5396
--	73.12	23.9508
--	73.14	21.2664
--	73.16	15.5711
--	73.18	10.5956
--	73.2	7.29918
--	73.22	5.34604
--	73.24	4.25356
--	73.26	3.66478
--	73.28	3.37834
--	73.3	3.30288
--	73.32	3.41144
--	73.34	3.72208
--	73.36	4.30788
--	73.38	5.33952
--	73.4	7.15318
--	73.42	10.2903
--	73.44	15.38242

--	73.46	22.5502
--	73.48	29.4032
--	73.5	29.9924
--	73.52	23.5952
--	73.54	16.15106
--	73.56	10.6555
--	73.58	7.18152
--	73.6	5.11042
--	73.62	3.89004
--	73.64	3.10432
--	73.66	2.57222
--	73.68	2.19242
--	73.7	1.912058
--	73.72	1.700622
--	73.74	1.538932
--	73.76	1.414278
--	73.78	1.318
--	73.8	1.24407
--	73.82	1.188266
--	73.84	1.147628
--	73.86	1.120134
--	73.88	1.104504
--	73.9	1.100106
--	73.92	1.10695
--	73.94	1.125848
--	73.96	1.158856
--	73.98	1.168976
--	74	1.301706
--	74.02	1.428106
--	74.04	1.624498
--	74.06	1.917206
--	74.08	2.29862
--	74.1	2.62114
--	74.12	2.65584
--	74.14	2.45882
--	74.16	2.28218
--	74.18	2.21762
--	74.2	2.26124
--	74.22	2.3928
--	74.24	2.59922
--	74.26	2.8796
--	74.28	3.24516
--	74.3	3.71968
--	74.32	4.34458
--	74.34	5.19394
--	74.36	6.40784
--	74.38	8.2462
--	74.4	11.13424
--	74.42	15.61982
--	74.44	22.054
--	74.46	29.394
--	74.48	33.8752
--	74.5	34.454
--	74.52	36.6418
--	74.54	45.12
--	74.56	61.3842
--	74.58	81.3644
--	74.6	89.643

--	74.62	75.5486
--	74.64	53.188
--	74.66	35.12
--	74.68	23.285
--	74.7	16.13952
--	74.72	11.88642
--	74.74	9.25212
--	74.76	7.51416
--	74.78	6.30624
--	74.8	5.44226
--	74.82	4.81994
--	74.84	4.37892
--	74.86	4.08332
--	74.88	3.91414
--	74.9	3.8664
--	74.92	3.95014
--	74.94	4.19756
--	74.96	4.7438
--	74.98	5.59942
--	75	7.1776
--	75.02	9.90484
--	75.04	14.262
--	75.06	20.1406
--	75.08	25.038
--	75.1	24.3014
--	75.12	18.81268
--	75.14	13.23858
--	75.16	9.34442
--	75.18	7.00244
--	75.2	5.71806
--	75.22	5.07896
--	75.24	4.83942
--	75.26	4.8891
--	75.28	5.20458
--	75.3	5.82764
--	75.32	6.88406
--	75.34	8.65428
--	75.36	11.69274
--	75.38	16.9176
--	75.4	25.4676
--	75.42	37.833
--	75.44	50.7404
--	75.46	54.005
--	75.48	43.901
--	75.5	30.5422
--	75.52	20.415
--	75.54	14.10168
--	75.56	10.48388
--	75.58	8.26734
--	75.6	6.527
--	75.62	5.15388
--	75.64	4.1462
--	75.66	3.4266
--	75.68	2.90976
--	75.7	2.52966
--	75.72	2.24178
--	75.74	2.01812
--	75.76	1.841396

--	75.78	1.700402
--	75.8	1.58751
--	75.82	1.49732
--	75.84	1.425936
--	75.86	1.37053
--	75.88	1.32906
--	75.9	1.300094
--	75.92	1.282678
--	75.94	1.254682
--	75.96	1.27427
--	75.98	1.290684
--	76	1.318644
--	76.02	1.359088
--	76.04	1.41341
--	76.06	1.399884
--	76.08	1.490798
--	76.1	1.60375
--	76.12	1.74363
--	76.14	1.91708
--	76.16	2.13324
--	76.18	2.40486
--	76.2	2.75008
--	76.22	3.19536
--	76.24	3.78048
--	76.26	4.5685
--	76.28	5.66834
--	76.3	7.28824
--	76.32	9.84354
--	76.34	14.1062
--	76.36	21.2706
--	76.38	32.6466
--	76.4	48.1772
--	76.42	61.8446
--	76.44	61.113
--	76.46	46.8574
--	76.48	31.6282
--	76.5	20.7182
--	76.52	13.9488
--	76.54	9.96318
--	76.56	7.5321
--	76.58	5.9252
--	76.6	4.80832
--	76.62	4.01554
--	76.64	3.44436
--	76.66	3.02884
--	76.68	2.72602
--	76.7	2.5083
--	76.72	2.35836
--	76.74	2.26592
--	76.76	2.22584
--	76.78	2.23724
--	76.8	2.30344
--	76.82	2.43284
--	76.84	2.6406
--	76.86	2.9525
--	76.88	3.41352
--	76.9	4.137
--	76.92	5.1859

--	76.94	7.02298
--	76.96	10.1494
--	76.98	15.27674
--	77	22.8056
--	77.02	31.098
--	77.04	34.024
--	77.06	28.3064
--	77.08	19.85716
--	77.1	13.19422
--	77.12	8.89352
--	77.14	6.338
--	77.16	4.85288
--	77.18	3.97236
--	77.2	3.44012
--	77.22	3.1343
--	77.24	3.00514
--	77.26	3.04778
--	77.28	3.30464
--	77.3	3.8898
--	77.32	5.01718
--	77.34	6.98564
--	77.36	10.0218
--	77.38	13.65444
--	77.4	15.53044
--	77.42	13.54166
--	77.44	9.80438
--	77.46	6.6605
--	77.48	4.57156
--	77.5	3.33198
--	77.52	2.58
--	77.54	2.11838
--	77.56	1.817598
--	77.58	1.611702
--	77.6	1.46742
--	77.62	1.366654
--	77.64	1.298716
--	77.66	1.257084
--	77.68	1.237914
--	77.7	1.23929
--	77.72	1.260864
--	77.74	1.303786
--	77.76	1.370856
--	77.78	1.46696
--	77.8	1.599954
--	77.82	1.782278
--	77.84	2.03438
--	77.86	2.41578
--	77.88	2.95208
--	77.9	3.80688
--	77.92	5.1758
--	77.94	7.55058
--	77.96	11.22772
--	77.98	15.9707
--	78	19.41944
--	78.02	18.15546
--	78.04	13.701
--	78.06	9.53788
--	78.08	6.71968

--	78.1	5.05898
--	78.12	4.16658
--	78.14	3.73694
--	78.16	3.59378
--	78.18	3.65958
--	78.2	3.91874
--	78.22	4.40294
--	78.24	5.20668
--	78.26	6.53626
--	78.28	8.77848
	78.3	12.5224
	78.32	18.38926
	78.34	26.2454
	78.36	32.944
	78.38	32.9472
	78.4	28.0134
	78.42	24.4668
	78.44	24.9766
	78.46	29.2168
	78.48	33.4656
	78.5	31.426
	78.52	23.9108
	78.54	16.475
	78.56	11.21254
	78.58	7.91438
	78.6	5.93252
	78.62	4.72502
	78.64	3.95384
	78.66	3.44002
	78.68	3.09416
	78.7	2.87034
	78.72	2.74438
	78.74	2.7051
	78.76	2.75142
	78.78	2.89222
	78.8	3.14964
	78.82	3.58982
	78.84	4.26068
	78.86	5.35398
	78.88	7.24654
	78.9	10.46824
	78.92	15.6277
	78.94	22.7444
	78.96	29.1782
	78.98	29.1272
	79	22.6664
	79.02	15.60022
	79.04	10.50884
	79.06	7.35562
	79.08	5.53418
	79.1	4.50828
	79.12	3.94104
	79.14	3.66192
	79.16	3.60156
	79.18	3.75132
	79.2	4.15874
	79.22	4.95674
	79.24	6.41624

79.26	8.97558
79.28	13.14228
79.3	18.99338
79.32	24.538
79.34	25.0302
79.36	20.1876
79.38	14.903
79.4	11.61118
79.42	10.35814
79.44	10.13346
79.46	9.24014
79.48	7.24784
79.5	5.27338
79.52	3.82862
79.54	2.8822
79.56	2.2808
79.58	1.890442
79.6	1.624128
79.62	1.433636
79.64	1.293684
79.66	1.190516
79.68	1.116204
79.7	1.066234
79.72	1.038524
79.74	1.033178
79.76	1.05281
79.78	1.103916
79.8	1.200512
79.82	1.371946
79.84	1.646366
79.86	2.17104
79.88	3.02266
79.9	4.22418
79.92	5.38736
79.94	5.52776
79.96	4.50818
79.98	3.28674
80	2.4261

Supplementary Figure 5

sample		Soaked in methanol 3 days		Soaked in methanol 1 days	
20.95907	99.99996	21.37442	100.00002	18.22796	100
20.96234	99.99697	21.38841	99.9955	18.23086	100.0007
20.96235	99.99277	21.4086	99.98623	18.23195	100.00026
20.96072	99.99311	21.4249	99.97775	18.23699	100.0007
20.95943	99.99494	21.44142	99.96949	18.24182	100.00131
20.96258	99.99411	21.46043	99.96162	18.24313	100.00227
20.95716	99.99461	21.47806	99.95548	18.24092	100.00393
20.95847	99.99459	21.49535	99.95001	18.24274	100.00567
20.96368	99.98715	21.50876	99.94418	18.24437	100.00654
20.95929	99.9822	21.5282	99.93735	18.25089	100.00785
20.96126	99.98774	21.54245	99.92864	18.25348	100.00681
20.95986	99.99053	21.55872	99.92363	18.25519	100.00777
20.95736	99.99713	21.57351	99.91825	18.25876	100.01143
20.95522	100.01206	21.58853	99.91713	18.25979	100.01763
20.95784	100.02241	21.60484	99.91913	18.26031	100.02688
20.95934	100.02791	21.62532	99.91835	18.26205	100.03255
20.96154	100.03299	21.6403	99.91741	18.26394	100.03874
20.96103	100.03201	21.6564	99.91358	18.26814	100.04398
20.95986	100.02798	21.67139	99.90958	18.266	100.04817
20.96036	100.0235	21.68609	99.90371	18.2657	100.05105
20.95914	100.01761	21.69914	99.89894	18.26986	100.05235
20.95706	100.01361	21.71342	99.89609	18.27073	100.05148
20.96232	100.00827	21.72587	99.89072	18.2738	100.04965
20.95586	100.00341	21.7408	99.88503	18.27751	100.04852
20.95925	100.00053	21.75299	99.87779	18.27769	100.04834
20.96195	99.9973	21.76675	99.86907	18.2787	100.04869
20.96224	99.9932	21.78228	99.85971	18.2819	100.04878
20.95784	99.99014	21.7969	99.85248	18.28236	100.04773
20.96262	99.98822	21.8144	99.84429	18.28658	100.04625
20.96297	99.98707	21.82314	99.8354	18.28475	100.04555
20.9611	99.98648	21.83363	99.82706	18.28554	100.0445
20.95916	99.98588	21.84865	99.81864	18.28749	100.04433
20.96474	99.98539	21.85988	99.81047	18.29201	100.04468
20.95988	99.98597	21.87212	99.80332	18.29086	100.04502
20.9567	99.98651	21.87957	99.79502	18.29146	100.04625
20.96025	99.98535	21.89638	99.78595	18.29507	100.04712
20.96157	99.98916	21.90767	99.77809	18.29622	100.04703
20.95652	99.98967	21.91671	99.77101	18.29769	100.0466
20.95612	99.98588	21.93127	99.76379	18.29994	100.0459
20.95602	99.98378	21.94219	99.75701	18.30109	100.04537
20.96154	99.98247	21.95344	99.74866	18.30399	100.04581
20.95856	99.98082	21.96496	99.74086	18.30612	100.04546
20.96411	99.98113	21.97054	99.73426	18.30582	100.04555
20.9616	99.98032	21.98212	99.72833	18.30759	100.04537
20.96478	99.97796	21.98967	99.72368	18.30708	100.0452
20.96286	99.97564	22	99.71813	18.30942	100.04476
20.96875	99.9762	22.01338	99.71204	18.30599	100.04433
20.96416	99.9757	22.0217	99.70444	18.31001	100.04642
20.96475	99.97639	22.03131	99.69734	18.31128	100.04712
20.9692	99.97924	22.04068	99.69003	18.31091	100.04852
20.96662	99.97568	22.0491	99.6827	18.31093	100.04886
20.96314	99.97522	22.05722	99.67578	18.31273	100.04747

20.96131	99.97131	22.06653	99.66872	18.31682	100.04686
20.96584	99.96596	22.07547	99.66152	18.31534	100.0459
20.97208	99.96579	22.08232	99.6549	18.31775	100.04564
20.97164	99.96772	22.09364	99.64955	18.31957	100.04529
20.97179	99.97029	22.09844	99.64155	18.32178	100.04529
20.97428	99.96945	22.11118	99.63421	18.32393	100.04511
20.97871	99.96882	22.11775	99.62669	18.32426	100.04555
20.98331	99.96383	22.12414	99.61864	18.32918	100.04485
20.98356	99.96003	22.13225	99.61092	18.33388	100.04459
20.99137	99.95588	22.14199	99.60179	18.33665	100.0445
20.9961	99.95392	22.15016	99.59212	18.34265	100.04433
21.00531	99.95553	22.15724	99.58319	18.34747	100.04363
21.01448	99.95292	22.16232	99.57653	18.35563	100.04249
21.01693	99.94881	22.1685	99.57038	18.35992	100.04171
21.03066	99.94608	22.17445	99.56573	18.36903	100.04066
21.03968	99.93941	22.18594	99.55975	18.37726	100.04127
21.04498	99.93542	22.18785	99.55266	18.38265	100.04127
21.05654	99.93538	22.19642	99.54458	18.39355	100.04171
21.07229	99.93329	22.20528	99.53603	18.4052	100.0418
21.08333	99.93095	22.21286	99.52699	18.41653	100.04101
21.0985	99.92865	22.22083	99.51836	18.42638	100.04075
21.11392	99.92945	22.22844	99.51094	18.43251	100.04005
21.13196	99.93016	22.22976	99.50364	18.44606	100.03979
21.15226	99.93163	22.23995	99.49745	18.46088	100.03927
21.16754	99.93083	22.2469	99.48988	18.47627	100.03831
21.18822	99.92752	22.25281	99.48326	18.49519	100.03708
21.20952	99.92423	22.25179	99.47473	18.50786	100.0356
21.23216	99.92206	22.26144	99.466	18.52727	100.03386
21.25212	99.92324	22.2665	99.45768	18.55062	100.03255
21.27751	99.91985	22.27717	99.44987	18.57081	100.03185
21.30309	99.91666	22.28666	99.44434	18.5882	100.03071
21.33031	99.91903	22.29652	99.43768	18.60798	100.03037
21.35282	99.91787	22.3019	99.43023	18.63162	100.03037
21.3857	99.91646	22.31476	99.42198	18.6584	100.03002
21.41442	99.91777	22.32533	99.41305	18.67718	100.02914
21.44292	99.91227	22.33366	99.40513	18.70156	100.02792
21.47298	99.90513	22.34299	99.39798	18.73171	100.0267
21.50628	99.90183	22.35509	99.39036	18.75384	100.02504
21.53896	99.89962	22.36574	99.38227	18.78555	100.02443
21.56765	99.89547	22.37713	99.37476	18.81346	100.02373
21.60227	99.89203	22.38828	99.36828	18.84494	100.02243
21.63458	99.89062	22.40547	99.36137	18.87216	100.02085
21.67377	99.89148	22.4192	99.35325	18.90338	100.01981
21.71283	99.88904	22.43612	99.34371	18.93775	100.01824
21.74617	99.88755	22.451	99.33454	18.9724	100.01658
21.78518	99.88671	22.46803	99.32582	19.0058	100.01527
21.82527	99.87496	22.4839	99.31834	19.03813	100.01414
21.86951	99.87013	22.50152	99.31139	19.07561	100.01309
21.90937	99.86772	22.51608	99.30284	19.11099	100.01222
21.94854	99.86525	22.53656	99.294	19.14287	100.01204
21.98883	99.86375	22.55504	99.28396	19.18077	100.01047
22.03602	99.85936	22.57494	99.2761	19.22061	100.00899
22.06877	99.85598	22.59014	99.26815	19.25122	100.00716
22.11072	99.84875	22.62155	99.26061	19.29112	100.00506
22.15768	99.84587	22.63591	99.25344	19.33618	100.00297
22.19787	99.84127	22.66069	99.24601	19.37565	100.00113
22.24707	99.83395	22.68914	99.23805	19.41908	99.99983
22.29146	99.83096	22.7146	99.22985	19.45927	99.99895

22.33841	99.82667	22.7397	99.22331	19.5025	99.99834
22.38772	99.82558	22.76822	99.21341	19.54805	99.99686
22.42994	99.82902	22.79516	99.20291	19.58815	99.99407
22.48035	99.82837	22.82682	99.19288	19.63201	99.99171
22.52842	99.82625	22.86148	99.18351	19.6761	99.98988
22.56776	99.82359	22.89094	99.17424	19.72706	99.98857
22.62004	99.82125	22.91792	99.16612	19.76139	99.98866
22.67	99.81579	22.95006	99.15774	19.81004	99.98726
22.72096	99.81148	22.98257	99.14684	19.8533	99.9849
22.77168	99.81097	23.01322	99.13737	19.902	99.98141
22.81956	99.80367	23.04587	99.12692	19.94904	99.97871
22.87436	99.79697	23.07358	99.11807	20.00074	99.97679
22.92504	99.79342	23.11208	99.10956	20.04693	99.9747
22.96936	99.79	23.14794	99.10126	20.09575	99.97339
23.02223	99.78979	23.18522	99.09203	20.14757	99.97129
23.07747	99.79101	23.22235	99.08269	20.19062	99.96833
23.11949	99.79291	23.26333	99.07346	20.2489	99.96562
23.1712	99.78734	23.30146	99.06392	20.29125	99.9637
23.22379	99.78158	23.33995	99.05386	20.34328	99.96178
23.27154	99.77752	23.38461	99.04311	20.38492	99.96004
23.32993	99.77168	23.42173	99.03195	20.43553	99.95768
23.3767	99.76772	23.46045	99.02119	20.49056	99.95524
23.4319	99.76609	23.49719	99.01285	20.53763	99.95279
23.48386	99.76224	23.53662	99.00438	20.5848	99.95061
23.5408	99.75556	23.57933	98.99698	20.6368	99.94922
23.58719	99.75251	23.62217	98.98758	20.68352	99.9473
23.64174	99.74711	23.6691	98.9793	20.731	99.94555
23.68197	99.7437	23.71194	98.97023	20.78537	99.9432
23.73504	99.74001	23.75109	98.95971	20.83311	99.93979
23.78448	99.74091	23.79918	98.94857	20.87735	99.93656
23.83816	99.74278	23.84823	98.93767	20.92883	99.93281
23.8857	99.7405	23.88952	98.92722	20.9758	99.92923
23.94136	99.73455	23.93588	98.91669	21.03294	99.92635
23.99747	99.72601	23.97326	98.90915	21.08044	99.92409
24.04334	99.71384	24.02008	98.89962	21.12101	99.92173
24.09542	99.70607	24.06804	98.88927	21.17284	99.9185
24.14562	99.70242	24.11438	98.8795	21.22134	99.91536
24.19483	99.70093	24.16408	98.86972	21.27665	99.91178
24.24664	99.69961	24.21133	98.8602	21.32293	99.90803
24.29466	99.69509	24.25658	98.85015	21.37186	99.90576
24.33768	99.69151	24.30462	98.83951	21.42384	99.90332
24.39117	99.68981	24.35255	98.82908	21.46792	99.90105
24.43842	99.68849	24.40406	98.81725	21.52459	99.89843
24.4878	99.68598	24.45123	98.80555	21.56926	99.89424
24.53506	99.68122	24.49283	98.79628	21.61801	99.89049
24.58997	99.67171	24.54063	98.78342	21.66854	99.88674
24.6371	99.66196	24.59188	98.77264	21.7161	99.88369
24.68672	99.65635	24.64274	98.76256	21.76592	99.88133
24.7376	99.65871	24.69028	98.75255	21.81416	99.87836
24.78306	99.65931	24.74096	98.74314	21.85542	99.87505
24.83281	99.65547	24.78834	98.73295	21.90094	99.87138
24.8712	99.65355	24.83526	98.72075	21.95192	99.86676
24.91827	99.64381	24.88567	98.70967	21.9951	99.86388
24.96989	99.63518	24.93742	98.69943	22.04519	99.86109
25.01655	99.62567	24.9832	98.68916	22.09506	99.85768
25.06631	99.62479	25.02062	98.68156	22.13778	99.85489
25.10901	99.62558	25.07363	98.67169	22.18089	99.85062
25.15956	99.61835	25.12599	98.66066	22.23213	99.8466

25.2057	99.61772	25.17436	98.64746	22.27794	99.84285
25.24624	99.61325	25.22461	98.63557	22.32194	99.83892
25.29207	99.59949	25.26831	98.62362	22.36664	99.83412
25.34073	99.59242	25.31808	98.61176	22.4126	99.8295
25.3776	99.58803	25.36774	98.6011	22.4491	99.82583
25.42082	99.5826	25.4168	98.589	22.49629	99.82165
25.46465	99.58085	25.46093	98.57662	22.53905	99.81781
25.51245	99.57819	25.51314	98.56457	22.58514	99.81414
25.5531	99.57417	25.55248	98.5541	22.63215	99.80995
25.59906	99.56332	25.5976	98.54277	22.6775	99.80542
25.64642	99.55582	25.64337	98.53125	22.7187	99.80105
25.68768	99.55178	25.69465	98.51955	22.7608	99.79652
25.73223	99.55092	25.7435	98.50863	22.8043	99.79268
25.7733	99.54817	25.7907	98.49696	22.853	99.78831
25.81192	99.54189	25.8403	98.48599	22.88886	99.78395
25.8475	99.53824	25.88535	98.47444	22.9359	99.77994
25.8919	99.53288	25.9278	98.4635	22.9804	99.77566
25.93453	99.53174	25.97713	98.45138	23.02136	99.77112
25.97688	99.5287	26.02583	98.43746	23.06163	99.7665
26.01913	99.52426	26.0575	98.42667	23.09442	99.76275
26.0613	99.51345	26.1073	98.41268	23.1337	99.75795
26.09798	99.50202	26.1541	98.39855	23.17547	99.75376
26.14162	99.49387	26.1976	98.38506	23.2177	99.74896
26.18032	99.48956	26.24382	98.37127	23.2592	99.74416
26.22133	99.48897	26.2938	98.35727	23.29978	99.73849
26.26405	99.48791	26.33784	98.34341	23.34075	99.73317
26.30901	99.48563	26.3784	98.33046	23.38044	99.72793
26.34969	99.4826	26.42851	98.3173	23.42277	99.72322
26.38338	99.47712	26.47588	98.30382	23.46202	99.71842
26.42318	99.46686	26.51922	98.29144	23.50371	99.71414
26.46518	99.45847	26.55333	98.28176	23.53795	99.70978
26.49906	99.44701	26.59748	98.26882	23.57996	99.70437
26.54278	99.43925	26.64182	98.25683	23.62312	99.69992
26.58269	99.43282	26.6886	98.24486	23.65835	99.69416
26.61611	99.42899	26.72903	98.23196	23.69619	99.69024
26.65686	99.42474	26.77297	98.21881	23.73468	99.68596
26.7018	99.41778	26.82045	98.20543	23.77395	99.68107
26.73859	99.4113	26.86004	98.19169	23.81225	99.67636
26.77571	99.40299	26.90343	98.17845	23.85116	99.67139
26.8065	99.39647	26.94268	98.1667	23.89086	99.66528
26.8492	99.3892	26.98544	98.15408	23.92661	99.65943
26.88435	99.38517	27.01996	98.14286	23.96919	99.65402
26.92348	99.37999	27.06361	98.12871	24.01309	99.64774
26.9636	99.37784	27.10179	98.11445	24.04727	99.64268
27.00346	99.37632	27.14574	98.10097	24.08814	99.63736
27.04295	99.37494	27.18684	98.089	24.12711	99.63238
27.08154	99.37445	27.22754	98.07558	24.15754	99.62776
27.12348	99.36887	27.26994	98.06102	24.19186	99.62366
27.16114	99.36135	27.30726	98.0471	24.23013	99.61816
27.199	99.34679	27.35094	98.03262	24.27014	99.61258
27.22873	99.33483	27.3895	98.01899	24.308	99.60638
27.26521	99.32385	27.43423	98.0063	24.34584	99.59896
27.30154	99.31085	27.4633	97.99451	24.38194	99.59242
27.33964	99.304	27.5035	97.97946	24.41974	99.58518
27.38362	99.29874	27.54694	97.96485	24.45918	99.57942
27.41928	99.29211	27.58849	97.95021	24.49654	99.57479
27.45546	99.29182	27.63017	97.93653	24.53714	99.56965
27.49674	99.28558	27.6669	97.92415	24.57261	99.56502

27.52954	99.28287	27.7086	97.91072	24.61482	99.55926
27.56818	99.28269	27.74735	97.89657	24.65216	99.55359
27.60763	99.27344	27.78736	97.88187	24.69081	99.54766
27.63818	99.26755	27.827	97.86549	24.72571	99.54085
27.67656	99.25932	27.8699	97.85084	24.75858	99.53562
27.71692	99.24741	27.89893	97.83975	24.79557	99.52951
27.7555	99.23921	27.94221	97.82551	24.83384	99.52305
27.79428	99.23301	27.98222	97.81222	24.8694	99.51712
27.83	99.22911	28.02033	97.79726	24.90616	99.51066
27.86499	99.21907	28.05568	97.78231	24.95044	99.50525
27.90877	99.21321	28.0969	97.76712	24.99106	99.49897
27.9486	99.20996	28.1373	97.75167	25.02411	99.49321
27.98375	99.20307	28.17504	97.7372	25.06255	99.48736
28.02045	99.2027	28.2139	97.72193	25.10304	99.47977
28.06349	99.19682	28.25349	97.70728	25.14026	99.47358
28.09874	99.19113	28.29124	97.69241	25.1761	99.46686
28.13191	99.18764	28.32409	97.68084	25.2194	99.46092
28.16584	99.17758	28.36215	97.6664	25.2533	99.45482
28.2083	99.1667	28.40014	97.65094	25.29226	99.4481
28.24833	99.15562	28.43934	97.63635	25.32525	99.44295
28.28796	99.14317	28.47425	97.62031	25.36423	99.4364
28.33148	99.13329	28.51887	97.60323	25.40039	99.42925
28.3663	99.12952	28.55011	97.5886	25.43936	99.42279
28.41002	99.1249	28.59124	97.5748	25.47492	99.41616
28.44839	99.11743	28.62842	97.56004	25.50994	99.40944
28.48425	99.11137	28.67007	97.54569	25.55058	99.40325
28.52268	99.10323	28.70928	97.5301	25.5871	99.39653
28.55518	99.09927	28.73674	97.51733	25.62734	99.39051
28.59426	99.09659	28.77672	97.50292	25.6677	99.38379
28.63156	99.09183	28.81758	97.48754	25.70596	99.37724
28.67482	99.09251	28.85409	97.47149	25.74402	99.37044
28.72002	99.08612	28.89689	97.45561	25.78189	99.36302
28.75584	99.07974	28.9336	97.43952	25.81185	99.35735
28.7967	99.07887	28.97102	97.42476	25.85295	99.35098
28.83695	99.07389	29.0086	97.41039	25.89328	99.34452
28.87738	99.06803	29.04775	97.39593	25.93234	99.3385
28.91709	99.05803	29.08328	97.38209	25.9704	99.33239
28.95346	99.04079	29.12386	97.36877	26.00953	99.32524
28.98643	99.02732	29.15638	97.35705	26.04806	99.318
29.02885	99.01702	29.1961	97.34054	26.09019	99.30997
29.066	99.01095	29.23199	97.32366	26.12863	99.3022
29.10786	99.01224	29.26828	97.30648	26.17157	99.29479
29.14737	99.01161	29.31189	97.29042	26.2089	99.2878
29.19044	99.00091	29.34818	97.27587	26.24764	99.28074
29.23155	98.9942	29.3842	97.26021	26.28875	99.27349
29.26834	98.98476	29.42737	97.24389	26.3256	99.26651
29.30486	98.97393	29.46619	97.22762	26.37012	99.25953
29.34971	98.96312	29.50182	97.21	26.40163	99.25412
29.39078	98.95596	29.53765	97.1946	26.44222	99.24723
29.42759	98.94738	29.57303	97.18305	26.48818	99.23973
29.47116	98.94075	29.61428	97.16865	26.52534	99.23196
29.51183	98.9363	29.65286	97.15364	26.56428	99.22446
29.55234	98.92459	29.68683	97.13643	26.60652	99.21695
29.59468	98.91436	29.7307	97.11876	26.64773	99.20962
29.63889	98.90238	29.76783	97.10069	26.68932	99.20238
29.67645	98.89479	29.80591	97.08336	26.72554	99.1947
29.72092	98.89611	29.84522	97.06733	26.77063	99.18676
29.76054	98.89658	29.88331	97.05044	26.81244	99.17891

29.80162	98.89235	29.92261	97.03299	26.85428	99.1714
29.84586	98.8828	29.96634	97.01744	26.89588	99.1632
29.88959	98.86147	29.99596	97.00475	26.93851	99.15552
29.93106	98.84088	30.03685	96.98835	26.97944	99.14802
29.9639	98.82426	30.07575	96.97332	27.00763	99.14165
30.00458	98.81035	30.11504	96.95791	27.05202	99.13406
30.04292	98.80401	30.15958	96.94192	27.09363	99.12585
30.08485	98.80431	30.1988	96.92775	27.13302	99.11791
30.1285	98.80363	30.23813	96.9125	27.17559	99.11032
30.16797	98.80376	30.27752	96.89635	27.21944	99.10291
30.2101	98.80211	30.31994	96.88064	27.26167	99.0961
30.25168	98.79581	30.35856	96.86278	27.30414	99.08868
30.29446	98.79156	30.39529	96.84662	27.34362	99.08039
30.33737	98.78678	30.42791	96.83427	27.38713	99.07228
30.38141	98.772	30.46404	96.819	27.42814	99.06355
30.41524	98.75961	30.50894	96.8042	27.46771	99.055
30.45518	98.74599	30.55081	96.78891	27.50856	99.04689
30.49674	98.73267	30.59364	96.77096	27.54387	99.0406
30.5353	98.72755	30.63284	96.75278	27.58806	99.03293
30.57982	98.7208	30.6775	96.73428	27.6273	99.02525
30.62184	98.71093	30.71764	96.71512	27.66623	99.01678
30.66731	98.70298	30.75404	96.69803	27.70917	99.00806
30.70798	98.69619	30.79625	96.68123	27.75136	98.99881
30.75068	98.6875	30.83727	96.66508	27.79155	98.99026
30.79329	98.6782	30.87256	96.65271	27.83482	98.98188
30.83814	98.67042	30.90789	96.63681	27.87603	98.97333
30.86882	98.66223	30.9508	96.61926	27.91438	98.96539
30.91423	98.65293	30.9917	96.60164	27.95877	98.9564
30.95984	98.64177	31.03424	96.58433	28.00305	98.94759
30.99888	98.63317	31.07524	96.56733	28.04248	98.93904
31.04036	98.62408	31.11968	96.55046	28.08197	98.92987
31.08178	98.60934	31.15202	96.53308	28.12401	98.92097
31.12676	98.60019	31.19747	96.51466	28.16017	98.91487
31.17069	98.58848	31.24335	96.4958	28.20409	98.90684
31.20962	98.57484	31.28333	96.47772	28.24956	98.89829
31.25531	98.57102	31.3135	96.46419	28.28705	98.89
31.29841	98.56898	31.35614	96.44772	28.32622	98.88066
31.33394	98.567	31.39851	96.43086	28.37276	98.87115
31.37136	98.56206	31.44316	96.41257	28.4136	98.8619
31.41871	98.55482	31.48156	96.39397	28.45458	98.85291
31.46005	98.54967	31.52342	96.375	28.49426	98.84436
31.49984	98.53743	31.56475	96.35611	28.53642	98.83572
31.54355	98.51892	31.60864	96.33853	28.5831	98.82726
31.5871	98.50164	31.6485	96.32069	28.62089	98.81792
31.62986	98.49174	31.69278	96.30283	28.6645	98.8085
31.67366	98.47818	31.73092	96.2849	28.71175	98.79934
31.71754	98.46836	31.7649	96.27056	28.75	98.79009
31.75731	98.46147	31.808	96.25197	28.7856	98.78302
31.8019	98.44806	31.85358	96.23353	28.82818	98.77377
31.84336	98.43515	31.88918	96.21569	28.87284	98.76409
31.87685	98.42917	31.93395	96.1991	28.90951	98.75423
31.92168	98.42115	31.97637	96.18212	28.95652	98.74471
31.96527	98.41298	32.01795	96.16524	28.99888	98.73555
32.00561	98.39868	32.05972	96.14751	29.0419	98.72648
32.05169	98.38995	32.09917	96.12859	29.08335	98.71766
32.08832	98.38519	32.14112	96.10935	29.12029	98.70894
32.12866	98.3759	32.18544	96.08988	29.16332	98.69934
32.17202	98.36821	32.22123	96.07449	29.20718	98.68992

32.21388	98.36201	32.2625	96.05481	29.24986	98.68023
32.256	98.3531	32.30256	96.03717	29.29258	98.66976
32.29742	98.34158	32.34386	96.01868	29.3252	98.66182
32.3351	98.33324	32.38756	96.00032	29.36904	98.65205
32.3769	98.32279	32.43083	95.98129	29.40694	98.6421
32.41616	98.31028	32.47004	95.96202	29.44952	98.63259
32.45795	98.29672	32.51327	95.94356	29.49222	98.62325
32.4985	98.28945	32.55369	95.92639	29.53504	98.61365
32.54196	98.28163	32.60211	95.90918	29.5804	98.60406
32.58256	98.27007	32.64478	95.89058	29.61874	98.59446
32.62103	98.26092	32.67728	95.87641	29.65827	98.58434
32.6669	98.25114	32.72049	95.85825	29.69957	98.57421
32.70561	98.23965	32.7627	95.84035	29.73952	98.56418
32.75076	98.22832	32.80107	95.82249	29.78091	98.55423
32.78356	98.2204	32.84675	95.80357	29.82602	98.54428
32.82142	98.21242	32.89286	95.78405	29.86634	98.53416
32.86224	98.19577	32.93315	95.7641	29.9068	98.52439
32.90812	98.17968	32.976	95.7441	29.94454	98.51593
32.94904	98.16118	33.02232	95.72439	29.98886	98.50633
32.99453	98.14008	33.06246	95.70355	30.02599	98.49603
33.02856	98.1327	33.09914	95.68366	30.07	98.48643
33.07181	98.13171	33.13601	95.66885	30.1124	98.47657
33.10922	98.13084	33.18018	95.64938	30.15242	98.46601
33.15095	98.13044	33.22269	95.6309	30.19384	98.45554
33.19458	98.12643	33.26399	95.61108	30.23298	98.44394
33.23456	98.11799	33.30432	95.59042	30.2734	98.43277
33.27504	98.09809	33.3457	95.57013	30.3202	98.42195
33.31627	98.07791	33.39176	95.55079	30.35698	98.41122
33.35881	98.057	33.42966	95.53277	30.40211	98.40162
33.40394	98.0343	33.47418	95.5152	30.44049	98.39176
33.4455	98.02275	33.51428	95.49691	30.48494	98.38199
33.4876	98.01114	33.55718	95.47807	30.52445	98.37256
33.52859	98.00381	33.59204	95.46183	30.55226	98.36418
33.56824	97.99906	33.6326	95.44209	30.59735	98.35328
33.60867	97.98455	33.67478	95.42168	30.64048	98.3422
33.6507	97.97222	33.71696	95.40236	30.68076	98.33059
33.69503	97.95878	33.7589	95.38252	30.72575	98.31916
33.73556	97.94655	33.80084	95.36235	30.76434	98.30808
33.7703	97.9448	33.8432	95.34289	30.80568	98.29726
33.80934	97.94327	33.88584	95.32176	30.84519	98.28679
33.85225	97.93687	33.93044	95.30155	30.88821	98.27632
33.8948	97.92202	33.96926	95.28127	30.93084	98.26532
33.93544	97.90729	34.01785	95.26143	30.97072	98.25407
33.97985	97.89295	34.05158	95.24597	31.0137	98.2429
34.0225	97.87752	34.08822	95.22653	31.05272	98.23199
34.06591	97.86799	34.13141	95.20605	31.08919	98.22379
34.10161	97.85675	34.17374	95.18586	31.1291	98.21314
34.14465	97.84254	34.21738	95.165	31.17328	98.20319
34.18522	97.83106	34.25878	95.1446	31.2126	98.19185
34.22045	97.81981	34.30028	95.1246	31.2532	98.18007
34.2657	97.81126	34.339	95.10492	31.29586	98.16838
34.30199	97.806	34.38546	95.08495	31.33203	98.15669
34.34378	97.79442	34.42674	95.06457	31.3755	98.14569
34.38366	97.78453	34.47044	95.04405	31.41664	98.13487
34.42738	97.76848	34.50296	95.02709	31.45542	98.12388
34.46734	97.75133	34.54248	95.00567	31.50192	98.11201
34.50878	97.73655	34.58256	94.98511	31.53482	98.10084
34.55025	97.72318	34.6236	94.96574	31.57871	98.08959

34.58762	97.7028	34.6672	94.94598	31.61869	98.07859
34.63104	97.68335	34.70845	94.927	31.66316	98.06725
34.66085	97.6659	34.75248	94.90654	31.69702	98.05782
34.70344	97.64662	34.7912	94.88467	31.7333	98.04535
34.73854	97.64101	34.83093	94.86331	31.777	98.03243
34.7817	97.63272	34.87716	94.84161	31.81788	98.02022
34.82361	97.63084	34.91845	94.82002	31.85628	98.00774
34.86438	97.62558	34.95078	94.80323	31.89995	97.99587
34.90676	97.61103	34.9927	94.78275	31.94175	97.98374
34.9475	97.60308	35.02875	94.76232	31.97934	97.97179
34.9887	97.59412	35.07224	94.74179	32.01487	97.96001
35.03058	97.57921	35.11419	94.72136	32.06144	97.94788
35.06847	97.56945	35.15996	94.69992	32.10095	97.93601
35.10436	97.55856	35.20134	94.6783	32.14514	97.92441
35.1455	97.53379	35.23625	94.65833	32.18555	97.91175
35.18678	97.51348	35.27875	94.6371	32.22314	97.89945
35.22554	97.50042	35.32041	94.61602	32.266	97.88802
35.26838	97.48306	35.36116	94.59439	32.29822	97.87772
35.30977	97.4704	35.39428	94.57637	32.34024	97.8656
35.34758	97.46357	35.4384	94.55471	32.38137	97.85277
35.39209	97.45074	35.47954	94.53346	32.42098	97.83968
35.43187	97.43728	35.51924	94.51187	32.46835	97.82712
35.47286	97.42103	35.5607	94.49052	32.5103	97.81525
35.50906	97.40284	35.60246	94.46941	32.55026	97.80373
35.55539	97.38625	35.64258	94.44732	32.59123	97.79178
35.59428	97.36558	35.68503	94.42532	32.63105	97.7793
35.62724	97.35992	35.72617	94.40284	32.67454	97.76673
35.6656	97.35274	35.76544	94.38024	32.7181	97.75399
35.71041	97.34031	35.80633	94.35774	32.75525	97.7409
35.75316	97.32359	35.84259	94.34011	32.80026	97.72808
35.79222	97.30643	35.88202	94.31881	32.83091	97.71752
35.83412	97.29501	35.92375	94.29741	32.87407	97.70487
35.87414	97.2805	35.96436	94.2762	32.91706	97.69222
35.91536	97.26824	36.0062	94.25489	32.95622	97.67913
35.95756	97.25747	36.04738	94.23232	32.99666	97.66647
35.99786	97.24059	36.08656	94.21037	33.03864	97.65347
36.03879	97.22019	36.13	94.18849	33.07754	97.64038
36.0718	97.21325	36.16782	94.16683	33.1211	97.62764
36.11393	97.20031	36.21188	94.14522	33.16162	97.61447
36.15217	97.18455	36.25472	94.12166	33.20148	97.60129
36.19676	97.16722	36.28921	94.1023	33.24406	97.5882
36.23694	97.14698	36.32783	94.07838	33.2843	97.57503
36.28038	97.13419	36.37154	94.05425	33.32589	97.56177
36.31968	97.1187	36.40798	94.0314	33.36644	97.54841
36.36331	97.10693	36.45297	94.00906	33.40487	97.53515
36.40778	97.09928	36.48968	93.98711	33.44272	97.52486
36.44463	97.08535	36.533	93.96523	33.48359	97.51159
36.48965	97.06619	36.5685	93.94286	33.52633	97.49868
36.51947	97.04975	36.60983	93.92085	33.56145	97.48489
36.56049	97.02728	36.64899	93.89829	33.60236	97.47145
36.60393	97.00454	36.69413	93.87619	33.64622	97.45845
36.64126	96.99126	36.73023	93.85782	33.68396	97.44519
36.68431	96.98335	36.76785	93.83509	33.72822	97.43219
36.72555	96.97134	36.81292	93.8126	33.76546	97.41866
36.76191	96.96031	36.8529	93.7896	33.80614	97.40514
36.80511	96.94239	36.89377	93.76632	33.8511	97.39126
36.84278	96.92353	36.93244	93.74327	33.88922	97.37756
36.88813	96.9083	36.97968	93.71933	33.92856	97.36282

36.92935	96.89365	37.0203	93.6948	33.96735	97.34886
36.96112	96.88598	37.06039	93.67241	34.01139	97.33524
36.99934	96.87523	37.09921	93.64967	34.04591	97.32469
37.0468	96.85887	37.13908	93.62634	34.08454	97.31107
37.08668	96.84176	37.17095	93.60791	34.12604	97.29676
37.13067	96.82301	37.21847	93.58319	34.16609	97.28254
37.16978	96.80643	37.25535	93.55764	34.20696	97.26806
37.20965	96.79033	37.30028	93.53368	34.24639	97.25506
37.25101	96.7757	37.33909	93.51073	34.28539	97.24109
37.29194	96.76194	37.37775	93.48868	34.32782	97.2267
37.3382	96.73994	37.42217	93.46614	34.37097	97.21247
37.37845	96.72408	37.46033	93.44359	34.41197	97.1979
37.42234	96.7082	37.5028	93.42021	34.45649	97.18359
37.46589	96.68272	37.5447	93.39581	34.49384	97.16919
37.4939	96.66591	37.58396	93.37174	34.53866	97.15427
37.53602	96.65686	37.61917	93.35113	34.57312	97.1418
37.57953	96.64384	37.66006	93.32588	34.60809	97.12731
37.62056	96.62782	37.7035	93.29981	34.65056	97.11283
37.65903	96.62031	37.74304	93.27415	34.69416	97.0993
37.70303	96.60406	37.78224	93.24925	34.73127	97.0849
37.74504	96.58222	37.82661	93.22404	34.7753	97.06972
37.78476	96.55924	37.86567	93.19951	34.81335	97.05524
37.823	96.5397	37.90548	93.17563	34.85657	97.0397
37.86766	96.52301	37.9488	93.15207	34.897	97.02583
37.90992	96.50316	37.98678	93.12954	34.93943	97.01169
37.94142	96.49346	38.02652	93.10717	34.9785	96.9966
37.9825	96.48085	38.06122	93.08775	35.01689	96.98107
38.02277	96.46362	38.10515	93.0631	35.05744	96.96554
38.06362	96.44335	38.1489	93.03685	35.10083	96.94992
38.10628	96.42411	38.18674	93.0094	35.14104	96.93465
38.14818	96.40762	38.22488	92.98318	35.17329	96.9226
38.1866	96.39202	38.26946	92.95727	35.21578	96.90751
38.22888	96.37386	38.31416	92.93178	35.25668	96.89268
38.26794	96.35714	38.35363	92.90701	35.30183	96.87758
38.31452	96.34035	38.3948	92.88232	35.3394	96.86231
38.35464	96.31619	38.43541	92.85727	35.3776	96.84625
38.38616	96.30406	38.47694	92.8318	35.42544	96.83098
38.42786	96.29826	38.51085	92.81141	35.46162	96.81528
38.4703	96.28398	38.5503	92.78534	35.50303	96.79992
38.50829	96.26763	38.5931	92.75867	35.54124	96.785
38.54976	96.25335	38.63617	92.73302	35.5824	96.76955
38.5927	96.23548	38.67581	92.70821	35.62858	96.75402
38.63519	96.20993	38.714	92.68325	35.66516	96.73849
38.67798	96.18996	38.7551	92.6588	35.7092	96.72226
38.71705	96.17225	38.79944	92.63345	35.74828	96.70603
38.75563	96.14839	38.83838	92.60651	35.78353	96.69338
38.79424	96.12944	38.88237	92.58025	35.82263	96.6775
38.82936	96.1117	38.92106	92.5545	35.86641	96.66179
38.8705	96.09091	38.95139	92.53413	35.90338	96.64582
38.91362	96.07752	38.99772	92.50952	35.94519	96.63003
38.95781	96.06639	39.03595	92.48482	35.99284	96.61397
38.9989	96.05107	39.07658	92.45692	36.03215	96.59801
39.039	96.02963	39.11319	92.42902	36.07216	96.58195
39.08162	96.0039	39.15926	92.40064	36.11504	96.5659
39.1214	95.98747	39.20264	92.37199	36.15586	96.54958
39.16372	95.97518	39.24185	92.34575	36.19766	96.53265
39.21	95.96057	39.28469	92.31966	36.23665	96.51529
39.2475	95.9508	39.32442	92.29327	36.28113	96.49818

39.29187	95.92867	39.36702	92.26723	36.31138	96.48544
39.33396	95.90371	39.39967	92.24556	36.35596	96.47017
39.36144	95.89231	39.43959	92.21903	36.39488	96.45508
39.40253	95.86966	39.47839	92.19198	36.43529	96.43929
39.4461	95.8459	39.5237	92.16481	36.47861	96.42227
39.4882	95.83002	39.559	92.13816	36.51998	96.40499
39.52681	95.80604	39.60125	92.11044	36.5605	96.3878
39.56556	95.79089	39.64453	92.08396	36.60306	96.37053
39.60918	95.77722	39.6834	92.05687	36.6457	96.35369
39.65246	95.75474	39.72498	92.02984	36.68352	96.33719
39.69547	95.73558	39.76553	92.00256	36.72458	96.32
39.7341	95.70923	39.8103	91.97474	36.76862	96.30308
39.77708	95.68082	39.83972	91.95217	36.80818	96.28632
39.8054	95.66664	39.88205	91.92338	36.85092	96.26913
39.84961	95.65048	39.9248	91.89478	36.89148	96.25255
39.89218	95.6305	39.9675	91.86726	36.92392	96.23938
39.9338	95.61515	40.0066	91.84111	36.96358	96.2221
39.97175	95.60145	40.05119	91.81355	37.00295	96.205
40.01459	95.58869	40.09266	91.78541	37.0466	96.18763
40.05439	95.57552	40.13226	91.75596	37.0861	96.17001
40.09948	95.54951	40.17212	91.7262	37.13269	96.15308
40.13928	95.52997	40.21365	91.69833	37.17178	96.13545
40.1802	95.50895	40.25488	91.67078	37.21523	96.118
40.2174	95.48665	40.28674	91.64924	37.25726	96.10012
40.25358	95.47379	40.3291	91.62198	37.299	96.08223
40.29596	95.45206	40.37053	91.59354	37.33884	96.06504
40.33469	95.42583	40.41324	91.56492	37.37802	96.04732
40.37533	95.40162	40.45194	91.53549	37.41983	96.0304
40.41594	95.38411	40.492	91.50564	37.46292	96.01277
40.45747	95.36684	40.53168	91.47712	37.50256	95.99514
40.5009	95.34701	40.57737	91.44828	37.53925	95.9811
40.54434	95.33365	40.61563	91.41851	37.57783	95.96382
40.58748	95.31477	40.65942	91.38808	37.61758	95.9455
40.62461	95.29478	40.69924	91.35786	37.65616	95.92717
40.66391	95.27475	40.73006	91.33358	37.69689	95.9092
40.69662	95.24959	40.77075	91.30446	37.73998	95.89009
40.73723	95.23038	40.81224	91.27628	37.78094	95.87246
40.7827	95.20927	40.84933	91.24789	37.8218	95.8544
40.8248	95.17766	40.89153	91.21849	37.86154	95.83642
40.86175	95.16044	40.9324	91.18969	37.90324	95.81888
40.9052	95.13123	40.97204	91.16062	37.94242	95.80135
40.94617	95.1103	41.01631	91.13104	37.98025	95.78302
40.986	95.09991	41.05881	91.10236	38.02376	95.764
41.02024	95.08613	41.09809	91.07234	38.06048	95.74943
41.06544	95.07604	41.14094	91.04242	38.10267	95.73023
41.1107	95.0548	41.1727	91.01734	38.1447	95.71217
41.15088	95.03439	41.2147	90.98569	38.18408	95.69367
41.19101	95.00705	41.25219	90.95414	38.22321	95.67474
41.223	94.98679	41.29906	90.9232	38.26399	95.65563
41.26568	94.9738	41.34047	90.89324	38.30364	95.63652
41.30869	94.95424	41.3788	90.86339	38.34456	95.61811
41.34772	94.93292	41.42208	90.83364	38.38779	95.59952
41.386	94.91625	41.45982	90.80241	38.4303	95.58067
41.4318	94.89392	41.50184	90.77126	38.47091	95.56191
41.47168	94.86702	41.54223	90.74147	38.50913	95.54306
41.51008	94.84643	41.58175	90.71181	38.55126	95.52395
41.55054	94.82619	41.61484	90.68838	38.59094	95.50537
41.59588	94.79618	41.65576	90.65819	38.63271	95.486

41.63934	94.77199	41.69678	90.62621	38.66287	95.47073
41.6676	94.75154	41.73767	90.5952	38.70504	95.45205
41.71191	94.72681	41.77912	90.56332	38.74706	95.43312
41.75242	94.70901	41.8202	90.53219	38.7857	95.41436
41.79559	94.6897	41.85694	90.50254	38.8332	95.39525
41.83396	94.67299	41.89698	90.47069	38.87008	95.37501
41.874	94.65421	41.94588	90.43914	38.908	95.35433
41.91488	94.62883	41.98285	90.40759	38.95132	95.33434
41.9555	94.60741	42.02284	90.37473	38.98924	95.31462
41.99692	94.59011	42.05644	90.34882	39.0325	95.29595
42.03906	94.57078	42.0974	90.31874	39.07223	95.27737
42.07912	94.54821	42.13568	90.28918	39.1134	95.25799
42.1189	94.52585	42.17609	90.25905	39.15286	95.23827
42.15722	94.50277	42.21814	90.22826	39.19472	95.21812
42.19809	94.47698	42.25932	90.19633	39.23318	95.19805
42.24043	94.44432	42.3006	90.16312	39.27268	95.18208
42.28144	94.42349	42.34102	90.12972	39.31072	95.16184
42.32402	94.39888	42.38246	90.09715	39.35325	95.14194
42.36372	94.3717	42.4225	90.06527	39.39594	95.12179
42.4056	94.35803	42.46313	90.0335	39.43312	95.10163
42.44794	94.34497	42.49497	90.00766	39.47522	95.08147
42.48639	94.32813	42.53384	89.97643	39.5175	95.0614
42.53194	94.30628	42.57322	89.94286	39.55836	95.04203
42.56291	94.28617	42.61442	89.90931	39.59562	95.02179
42.60433	94.25726	42.65396	89.87581	39.63614	95.00172
42.64188	94.22718	42.69188	89.84112	39.67791	94.9813
42.68165	94.20089	42.73765	89.80791	39.71838	94.9601
42.72662	94.17861	42.77408	89.77314	39.76226	94.93933
42.7669	94.15899	42.81564	89.74166	39.79	94.92275
42.81078	94.13784	42.85776	89.70929	39.83324	94.90207
42.85261	94.11697	42.8983	89.67652	39.87354	94.88148
42.8934	94.09429	42.92828	89.6509	39.91485	94.86071
42.93392	94.06767	42.9707	89.6166	39.95428	94.83933
42.9756	94.04263	43.01076	89.58288	39.995	94.81857
43.01688	94.01945	43.05396	89.54922	40.0362	94.79675
43.05566	93.99069	43.09594	89.51583	40.07883	94.77607
43.09034	93.97085	43.13604	89.48226	40.12097	94.75565
43.1286	93.94527	43.17687	89.44791	40.15794	94.73427
43.17108	93.91496	43.21756	89.41474	40.19676	94.71386
43.20984	93.88908	43.25686	89.38099	40.23774	94.69274
43.25332	93.86219	43.30044	89.34723	40.27466	94.6711
43.29385	93.8359	43.34174	89.31321	40.31954	94.64946
43.33664	93.81179	43.37691	89.28514	40.35939	94.62808
43.37796	93.79307	43.4179	89.25068	40.39668	94.61046
43.4195	93.77375	43.45672	89.21626	40.43525	94.58969
43.45888	93.75033	43.49917	89.18364	40.47769	94.56875
43.50019	93.72491	43.53911	89.14999	40.51495	94.54711
43.53494	93.70395	43.57802	89.11587	40.56281	94.5259
43.57503	93.68727	43.6214	89.08127	40.60198	94.50357
43.61716	93.66398	43.66141	89.04567	40.64477	94.4814
43.65773	93.63853	43.70517	89.01065	40.68342	94.45906
43.70089	93.61702	43.74496	88.97521	40.7269	94.43725
43.73752	93.58905	43.78485	88.94085	40.76712	94.41561
43.7814	93.56376	43.81832	88.91274	40.81378	94.39353
43.82084	93.54222	43.86023	88.87773	40.8518	94.37172
43.8653	93.51134	43.90088	88.84239	40.89106	94.34991
43.90472	93.47932	43.9414	88.80596	40.93474	94.32678
43.9424	93.45151	43.98515	88.76922	40.97867	94.30453

43.97762	93.43865	44.0198	88.73353	41.01032	94.28647
44.01828	93.42798	44.05896	88.69903	41.04975	94.26431
44.0592	93.41437	44.10078	88.66403	41.0958	94.24328
44.09919	93.38783	44.1423	88.6298	41.1343	94.22251
44.1473	93.35955	44.18773	88.59472	41.17774	94.20131
44.18416	93.33391	44.22719	88.55897	41.21755	94.17871
44.22417	93.30254	44.25896	88.53101	41.25471	94.15585
44.2679	93.27756	44.30023	88.4955	41.29711	94.13141
44.30825	93.25408	44.33618	88.45856	41.33938	94.10759
44.34633	93.22516	44.38052	88.42188	41.38198	94.08473
44.38666	93.19686	44.42119	88.38488	41.41986	94.06161
44.4212	93.17809	44.46072	88.3484	41.46161	94.03953
44.4647	93.15051	44.4969	88.3128	41.50372	94.01737
44.50165	93.11623	44.53874	88.27596	41.53475	93.99913
44.54523	93.09114	44.58093	88.23918	41.57774	93.97662
44.58966	93.07871	44.6255	88.20226	41.61621	93.95402
44.62965	93.06388	44.66804	88.166	41.65684	93.93107
44.66903	93.04631	44.70022	88.13708	41.69682	93.90812
44.7111	93.02259	44.74176	88.10111	41.7374	93.88474
44.7533	92.99138	44.77903	88.06462	41.77509	93.8617
44.79582	92.96618	44.81563	88.02723	41.81558	93.83884
44.83645	92.93398	44.8573	87.99091	41.86074	93.81563
44.87734	92.90418	44.89534	87.95431	41.90414	93.79259
44.91602	92.88124	44.93425	87.91598	41.94332	93.76912
44.9487	92.85671	44.97476	87.87978	41.98516	93.74539
44.9903	92.83115	45.0178	87.84245	42.02598	93.7213
45.03008	92.81172	45.05929	87.80515	42.0685	93.69766
45.0721	92.78323	45.10061	87.76883	42.10734	93.67322
45.10913	92.75507	45.13011	87.73788	42.14248	93.65446
45.15306	92.72899	45.17526	87.69971	42.17984	93.63143
45.19508	92.70514	45.2131	87.66095	42.21764	93.60804
45.23612	92.68108	45.25253	87.62423	42.2636	93.58544
45.27701	92.65828	45.29074	87.58693	42.3049	93.56162
45.31994	92.63001	45.33275	87.55028	42.34432	93.5378
45.36406	92.59892	45.37206	87.51378	42.38368	93.51354
45.39545	92.57668	45.41244	87.47489	42.42683	93.4899
45.43864	92.5472	45.45421	87.4364	42.46844	93.46616
45.47746	92.52292	45.4959	87.39642	42.5062	93.44234
45.51729	92.49687	45.53482	87.35764	42.54386	93.418
45.55354	92.47467	45.56638	87.32682	42.58444	93.39269
45.59838	92.44197	45.61358	87.28775	42.62684	93.36739
45.64116	92.40483	45.65081	87.24951	42.66748	93.34226
45.67836	92.37925	45.69058	87.20998	42.70714	93.31756
45.72284	92.3487	45.7317	87.17083	42.7442	93.29758
45.76606	92.32595	45.77182	87.13129	42.78265	93.27411
45.80767	92.30154	45.81549	87.09293	42.82468	93.24924
45.83854	92.27863	45.85512	87.0529	42.86218	93.2242
45.87935	92.25215	45.89564	87.01433	42.90343	93.19968
45.92096	92.22401	45.93844	86.97614	42.945	93.17464
45.95929	92.19794	45.98224	86.93633	42.98488	93.14994
46.00286	92.17683	46.00616	86.90491	43.02536	93.12455
46.04376	92.14535	46.04572	86.86534	43.06288	93.09863
46.08576	92.11789	46.08908	86.82566	43.1065	93.07272
46.124	92.09548	46.1289	86.78518	43.14821	93.04724
46.1687	92.07332	46.16711	86.74547	43.19046	93.02255
46.21315	92.05473	46.20896	86.70503	43.22873	92.99829
46.25285	92.02659	46.25016	86.66506	43.26484	92.9777
46.28556	92.00144	46.28932	86.62538	43.30796	92.95274

46.3273	91.96786	46.3315	86.58602	43.34414	92.92805
46.36921	91.93494	46.37292	86.54646	43.38902	92.90344
46.40872	91.90292	46.41524	86.50664	43.4265	92.87901
46.45354	91.87924	46.4427	86.47399	43.46996	92.85353
46.4949	91.85424	46.48676	86.43346	43.50402	92.82779
46.5394	91.82513	46.53016	86.39324	43.54842	92.80196
46.57959	91.79518	46.5683	86.35277	43.59116	92.77665
46.62136	91.76451	46.60478	86.31343	43.62946	92.75161
46.6602	91.72898	46.64772	86.27251	43.66703	92.72587
46.70219	91.69597	46.68739	86.23169	43.71176	92.69952
46.74113	91.67247	46.72719	86.19021	43.75086	92.67317
46.78608	91.65116	46.76708	86.14884	43.78912	92.64716
46.81708	91.63642	46.81146	86.10938	43.83313	92.62116
46.85847	91.61528	46.85248	86.06846	43.86918	92.60005
46.8997	91.59189	46.88398	86.03592	43.90824	92.57413
46.9419	91.56536	46.92607	85.99383	43.95127	92.54769
46.98496	91.53758	46.9685	85.95067	43.98922	92.52178
47.0239	91.50645	47.00508	85.90804	44.03375	92.49525
47.06467	91.47809	47.04562	85.86644	44.0743	92.46872
47.1025	91.45279	47.08768	85.82539	44.11059	92.44185
47.14762	91.42293	47.12787	85.78411	44.15189	92.41506
47.18922	91.3993	47.16604	85.74276	44.19421	92.38914
47.23083	91.37541	47.21154	85.69948	44.23724	92.36288
47.26463	91.34807	47.25146	85.65784	44.27553	92.33635
47.3114	91.31342	47.28559	85.61644	44.31758	92.30904
47.35536	91.28278	47.32274	85.58301	44.3525	92.28112
47.39248	91.25152	47.36504	85.54181	44.39384	92.2532
47.43512	91.22946	47.40406	85.49958	44.43461	92.22597
47.4766	91.20861	47.44199	85.45663	44.47108	92.20442
47.51904	91.18445	47.4848	85.41352	44.51448	92.1772
47.55809	91.15058	47.52779	85.37137	44.5522	92.14936
47.59784	91.10696	47.56838	85.3283	44.59366	92.12135
47.64281	91.06985	47.6106	85.28595	44.6372	92.09343
47.6813	91.03713	47.65	85.24448	44.67507	92.06638
47.71418	91.01762	47.68974	85.20139	44.7166	92.04029
47.75762	90.99655	47.72996	85.15835	44.75582	92.01324
47.79946	90.96982	47.76422	85.12432	44.79781	91.98601
47.84124	90.94473	47.80246	85.07996	44.83638	91.95818
47.88198	90.91819	47.83992	85.03588	44.87614	91.92904
47.92359	90.89606	47.88493	84.99273	44.91948	91.90172
47.96116	90.876	47.92297	84.94836	44.95866	91.87424
48.00237	90.84118	47.9618	84.90489	44.99457	91.85173
48.04422	90.80946	48.00128	84.86154	45.02787	91.82415
48.08888	90.77732	48.04197	84.81907	45.0701	91.79623
48.1256	90.74033	48.08329	84.77699	45.11042	91.76831
48.16444	90.72375	48.12172	84.73355	45.14788	91.741
48.20184	90.70345	48.16834	84.68956	45.19231	91.71307
48.2443	90.67469	48.19611	84.65398	45.23068	91.68489
48.28442	90.64403	48.23909	84.60902	45.27212	91.65653
48.32656	90.61795	48.28137	84.56447	45.31256	91.62782
48.36938	90.58332	48.32208	84.51951	45.35398	91.59981
48.4079	90.54531	48.3593	84.47501	45.39602	91.57102
48.4525	90.51286	48.40188	84.43046	45.43738	91.54231
48.49603	90.47979	48.44326	84.38814	45.478	91.5143
48.53687	90.44606	48.48874	84.3459	45.51946	91.48551
48.57722	90.41718	48.5251	84.30178	45.5591	91.45715
48.61612	90.39281	48.56461	84.25764	45.59284	91.43446
48.65966	90.36595	48.60838	84.21139	45.63602	91.40497

48.69609	90.34301	48.64137	84.17337	45.67475	91.37556
48.7331	90.31677	48.67414	84.12836	45.71394	91.34773
48.7752	90.29448	48.72322	84.0836	45.75676	91.31928
48.81864	90.2626	48.76324	84.03963	45.80217	91.2911
48.85804	90.23271	48.80371	83.99542	45.83978	91.26265
48.89765	90.19819	48.84208	83.95075	45.883	91.23342
48.94087	90.15711	48.88493	83.90589	45.92046	91.20427
48.98054	90.12407	48.92446	83.86082	45.96219	91.17531
49.02546	90.09024	48.96798	83.81638	46.00566	91.14625
49.06617	90.06407	49.00712	83.77178	46.04785	91.11614
49.1072	90.04722	49.04739	83.72748	46.08806	91.08578
49.13828	90.03066	49.081	83.69068	46.12944	91.05594
49.18103	90.00621	49.1226	83.64476	46.1694	91.02653
49.21968	89.97814	49.16192	83.59955	46.20234	91.00323
49.26622	89.94762	49.20176	83.55221	46.24575	90.97383
49.3005	89.91386	49.24119	83.50679	46.27977	90.9446
49.3481	89.8797	49.28334	83.46131	46.32466	90.91467
49.3927	89.8503	49.32103	83.41449	46.3654	90.88465
49.42996	89.81865	49.364	83.36895	46.40963	90.85525
49.47368	89.78216	49.4032	83.32323	46.44812	90.82462
49.51262	89.75059	49.44464	83.27693	46.48834	90.79382
49.55629	89.71647	49.48371	83.23209	46.52846	90.76336
49.5894	89.69223	49.51809	83.19614	46.56568	90.73343
49.63194	89.66266	49.55824	83.14964	46.60966	90.70333
49.67106	89.63002	49.59964	83.1032	46.65152	90.67401
49.716	89.59718	49.63934	83.05659	46.69289	90.64391
49.76086	89.56498	49.68377	83.00882	46.7242	90.61948
49.8007	89.54331	49.71998	82.96194	46.76425	90.58867
49.83953	89.51857	49.76372	82.91634	46.80265	90.5577
49.88422	89.49531	49.80246	82.869	46.84709	90.52724
49.91975	89.4731	49.84021	82.82239	46.88583	90.49662
49.96028	89.4401	49.88237	82.77501	46.92597	90.46634
50.00494	89.41235	49.92114	82.7275	46.97193	90.43606
50.03496	89.38874	49.9493	82.68993	47.00861	90.40543
50.07698	89.35775	49.99604	82.64351	47.05277	90.37446
50.1175	89.32956	50.035	82.59763	47.09205	90.34383
50.15967	89.29118	50.07576	82.55077	47.12582	90.31294
50.20512	89.25709	50.11713	82.50336	47.17261	90.28205
50.24656	89.22673	50.15746	82.45621	47.21409	90.25125
50.2885	89.18797	50.19726	82.40831	47.25485	90.21975
50.33159	89.15537	50.23778	82.35982	47.29532	90.1879
50.37235	89.13234	50.28098	82.3119	47.33149	90.16198
50.41388	89.10225	50.3236	82.2645	47.37029	90.1297
50.45874	89.07144	50.364	82.21791	47.4108	90.09785
50.49446	89.04418	50.39053	82.18101	47.44925	90.06635
50.53653	89.01146	50.43546	82.13439	47.49118	90.03511
50.57026	88.97712	50.47564	82.08652	47.53028	90.00274
50.61178	88.94132	50.5146	82.03741	47.57324	89.9708
50.65328	88.91664	50.5579	81.98881	47.61187	89.93887
50.69515	88.88951	50.5982	81.93917	47.64708	89.9078
50.73718	88.86749	50.63985	81.89182	47.69159	89.87779
50.78105	88.83949	50.68122	81.84434	47.7341	89.84629
50.82646	88.80526	50.72431	81.79523	47.7728	89.81426
50.86546	88.77641	50.76348	81.74818	47.81711	89.78207
50.90394	88.74027	50.80693	81.6991	47.8536	89.74891
50.951	88.70851	50.84185	81.65975	47.8993	89.71636
50.9888	88.67504	50.877	81.61218	47.92862	89.69106
51.02746	88.64784	50.92053	81.56415	47.97296	89.6586

51.0725	88.62373	50.96348	81.5162	48.01322	89.62718
51.11234	88.60118	50.99824	81.4679	48.05338	89.59612
51.15522	88.57779	51.04574	81.41998	48.09074	89.56445
51.19537	88.54731	51.08646	81.37116	48.13593	89.53268
51.23794	88.51264	51.12418	81.32289	48.177	89.50031
51.2767	88.4808	51.16786	81.27351	48.21541	89.46811
51.3215	88.44932	51.21054	81.22376	48.25238	89.43565
51.3642	88.42558	51.2502	81.174	48.29562	89.40258
51.40281	88.40163	51.285	81.13356	48.33426	89.36969
51.4412	88.37176	51.32634	81.08545	48.37594	89.33601
51.47613	88.34957	51.3651	81.03649	48.42068	89.30241
51.5182	88.31474	51.40738	80.98825	48.44816	89.27632
51.56368	88.27981	51.44621	80.93976	48.49186	89.24377
51.60496	88.24847	51.48712	80.88971	48.52989	89.21062
51.6498	88.21798	51.52496	80.84065	48.56984	89.17816
51.69093	88.18661	51.56682	80.79279	48.61159	89.14456
51.73138	88.14804	51.61102	80.74536	48.6574	89.11132
51.77372	88.11581	51.6557	80.69767	48.6926	89.07886
51.81994	88.09217	51.69274	80.64916	48.7326	89.04614
51.86082	88.06183	51.72511	80.60851	48.7753	89.01368
51.90159	88.03235	51.7701	80.55767	48.81954	88.98017
51.93444	88.00927	51.81192	80.50748	48.86228	88.94658
51.97982	87.97172	51.84834	80.45813	48.901	88.91193
52.02144	87.93363	51.88896	80.40941	48.93817	88.87712
52.06581	87.90092	51.92989	80.36022	48.9823	88.84318
52.1023	87.86705	51.97384	80.31085	49.02392	88.80915
52.14828	87.83186	52.01508	80.26087	49.05664	88.78227
52.18981	87.7994	52.05502	80.21113	49.09664	88.74868
52.23519	87.77215	52.0924	80.16039	49.1402	88.71438
52.27874	87.75253	52.13736	80.11004	49.17933	88.68062
52.32076	87.7316	52.172	80.07107	49.223	88.64615
52.36426	87.71144	52.2119	80.0213	49.25814	88.61308
52.40926	87.68525	52.252	79.97249	49.30208	88.57975
52.44961	87.65054	52.29228	79.92444	49.34141	88.54528
52.4841	87.62035	52.33196	79.87354	49.37773	88.51125
52.52416	87.58368	52.3744	79.82344	49.42056	88.47652
52.5677	87.5497	52.41597	79.77309	49.46233	88.44197
52.61068	87.51854	52.45665	79.72196	49.50124	88.40776
52.656	87.49192	52.49729	79.67269	49.5457	88.37382
52.69864	87.46255	52.53871	79.62318	49.58868	88.33996
52.74324	87.43878	52.57764	79.57268	49.62858	88.30541
52.78734	87.40957	52.6107	79.53316	49.65754	88.27757
52.82925	87.37963	52.65118	79.48377	49.70359	88.24311
52.86989	87.35337	52.69782	79.43321	49.74503	88.20847
52.91295	87.32662	52.73746	79.38357	49.78653	88.17426
52.94664	87.3074	52.77804	79.33272	49.82356	88.13988
52.9898	87.27939	52.8176	79.28063	49.86414	88.10463
53.02961	87.25336	52.85907	79.23016	49.90535	88.06964
53.07405	87.22519	52.90215	79.17851	49.94446	88.03456
53.1185	87.19456	52.94168	79.12774	49.98122	88.00044
53.15838	87.16409	52.98042	79.07826	50.0213	87.96563
53.19904	87.13186	53.02081	79.02735	50.06328	87.93151
53.24412	87.10127	53.0556	78.98816	50.10568	87.89713
53.2867	87.06908	53.09434	78.93905	50.14597	87.8624
53.32851	87.04373	53.14216	78.8893	50.17877	87.835
53.37243	87.0219	53.18222	78.83958	50.22042	87.79958
53.40892	86.99994	53.22226	78.78799	50.26324	87.76467
53.45098	86.97171	53.26552	78.73675	50.30824	87.72925

53.49418	86.94478	53.30436	78.68544	50.34637	87.69434
53.53656	86.91257	53.34903	78.63586	50.38909	87.65787
53.5808	86.88502	53.38734	78.58709	50.42954	87.62157
53.61895	86.85672	53.42885	78.5386	50.47108	87.5858
53.66512	86.82618	53.46988	78.48853	50.51352	87.55002
53.71142	86.79703	53.50301	78.44693	50.55176	87.51477
53.75626	86.7664	53.5459	78.39503	50.59254	87.47899
53.79932	86.74325	53.5866	78.34378	50.63453	87.44374
53.8407	86.71582	53.6302	78.29314	50.67532	87.40788
53.87734	86.69238	53.66771	78.24306	50.71726	87.37219
53.91729	86.66372	53.71039	78.19305	50.75986	87.33589
53.95714	86.63105	53.74978	78.14239	50.79231	87.30692
54.00299	86.60448	53.79594	78.09184	50.83305	87.27045
54.04615	86.58186	53.83863	78.04212	50.87587	87.23478
54.09075	86.56279	53.88033	77.99402	50.91546	87.20024
54.13535	86.54118	53.9206	77.94379	50.95437	87.16498
54.17527	86.51758	53.95162	77.90405	51.00188	87.12946
54.21944	86.49441	53.99905	77.85332	51.04041	87.09251
54.26127	86.46633	54.03915	77.80052	51.08316	87.05571
54.30586	86.43763	54.0816	77.7498	51.12334	87.01899
54.35229	86.40707	54.12478	77.69852	51.16384	86.98327
54.39377	86.37644	54.16744	77.64792	51.20658	86.94807
54.42792	86.35653	54.2064	77.59788	51.24412	86.91222
54.46884	86.33106	54.25112	77.54761	51.28534	86.87605
54.51135	86.30992	54.29242	77.49811	51.32646	86.83964
54.55664	86.2867	54.32972	77.44948	51.36688	86.8037
54.60137	86.25501	54.37518	77.39965	51.40139	86.77523
54.64564	86.22696	54.4059	77.35918	51.44662	86.73923
54.6892	86.19881	54.4475	77.30918	51.48828	86.70303
54.73916	86.17352	54.4914	77.25849	51.52666	86.6661
54.77888	86.15372	54.53356	77.20791	51.57212	86.62895
54.82209	86.13172	54.57449	77.1588	51.6105	86.59267
54.86611	86.10919	54.61484	77.11063	51.65234	86.55632
54.90008	86.09066	54.65957	77.06133	51.69148	86.52061
54.94482	86.06436	54.70028	77.01249	51.72911	86.48489
54.98904	86.04187	54.74268	76.96348	51.77041	86.44826
55.03495	86.02436	54.7835	76.91339	51.81272	86.41112
55.07752	86.00169	54.82848	76.86355	51.85523	86.37461
55.12383	85.97689	54.8602	76.82412	51.89558	86.33801
55.16875	85.95419	54.9034	76.77365	51.92858	86.30917
55.20888	85.92793	54.94566	76.72422	51.9719	86.27296
55.25443	85.90507	54.98734	76.67629	52.01352	86.23598
55.29759	85.88658	55.0294	76.62789	52.05531	86.1985
55.34188	85.86315	55.0687	76.58021	52.0958	86.16107
55.37455	85.84365	55.11291	76.53156	52.13838	86.12418
55.4159	85.82062	55.15551	76.48244	52.1781	86.08704
55.45987	85.79885	55.1953	76.43362	52.21897	86.05099
55.51188	85.77801	55.23914	76.385	52.26272	86.0152
55.55139	85.75965	55.28418	76.33527	52.3061	85.97893
55.59768	85.73967	55.31794	76.29549	52.34926	85.94352
55.64399	85.71302	55.35909	76.2451	52.38472	85.907
55.68879	85.69239	55.40574	76.19666	52.42942	85.86983
55.73298	85.66827	55.44838	76.15047	52.472	85.83343
55.77761	85.64544	55.48974	76.10518	52.51336	85.79629
55.82386	85.6288	55.5351	76.06056	52.54465	85.7661
55.8572	85.61138	55.57761	76.01392	52.58549	85.72921
55.9008	85.59199	55.61788	75.9651	52.62622	85.69213
55.94754	85.57431	55.66025	75.91581	52.66782	85.65516

55.99246	85.55636	55.70437	75.86651	52.71235	85.61876
56.03642	85.53632	55.74615	75.8178	52.75142	85.58196
56.08308	85.51628	55.78032	75.7789	52.7913	85.54495
56.12641	85.49936	55.821	75.7321	52.83701	85.50807
56.1727	85.48064	55.86579	75.68559	52.87592	85.47113
56.21698	85.46144	55.9145	75.63906	52.91922	85.43469
56.26331	85.44347	55.95478	75.59303	52.96066	85.39818
56.30449	85.42331	55.99913	75.5463	53.00129	85.3612
56.34876	85.40268	56.04211	75.49911	53.04392	85.32455
56.39222	85.38903	56.08393	75.45335	53.08494	85.28734
56.4319	85.37703	56.12467	75.4064	53.12683	85.24966
56.47636	85.35312	56.16977	75.36042	53.15852	85.22039
56.52038	85.33926	56.21301	75.31614	53.20159	85.18433
56.56328	85.32587	56.25062	75.27935	53.24294	85.14827
56.605	85.3091	56.29121	75.23382	53.28654	85.11285
56.65476	85.2929	56.33722	75.18871	53.32554	85.07709
56.70144	85.27314	56.38164	75.14304	53.3699	85.04048
56.74656	85.2498	56.42329	75.09672	53.41414	85.0032
56.79407	85.22785	56.46615	75.05275	53.45524	84.96641
56.84072	85.21335	56.50957	75.00577	53.49589	84.9297
56.88592	85.19912	56.55429	74.9589	53.5377	84.89328
56.92082	85.18855	56.60047	74.91264	53.58356	84.85719
56.96664	85.16821	56.64034	74.86749	53.62402	84.82018
57.00904	85.15166	56.68495	74.82383	53.66287	84.78296
57.05868	85.13915	56.71258	74.78867	53.69734	84.75335
57.10623	85.12742	56.76328	74.74429	53.74097	84.71678
57.15046	85.11353	56.80476	74.69964	53.7816	84.68054
57.1937	85.09859	56.84848	74.65538	53.8252	84.64436
57.2405	85.07975	56.89514	74.61149	53.86574	84.60787
57.289	85.06199	56.93845	74.56811	53.91129	84.57048
57.33374	85.04977	56.97965	74.5243	53.95187	84.53335
57.38028	85.03492	57.02627	74.48006	53.99137	84.4976
57.41394	85.02198	57.07037	74.43548	54.03468	84.4619
57.4626	85.0036	57.11377	74.39327	54.07765	84.42621
57.5041	84.98191	57.1592	74.35088	54.11975	84.39086
57.55407	84.96941	57.19374	74.31623	54.16222	84.35485
57.5995	84.95687	57.2378	74.27359	54.20436	84.31857
57.6438	84.94539	57.28378	74.23149	54.24773	84.28291
57.68889	84.93587	57.32918	74.18825	54.28906	84.24673
57.73399	84.92358	57.37383	74.14688	54.32389	84.21802
57.78098	84.90971	57.41798	74.10511	54.36452	84.18138
57.82625	84.89716	57.46174	74.06259	54.4089	84.14487
57.87268	84.88341	57.50818	74.02225	54.45232	84.10902
57.9104	84.87036	57.55176	73.98134	54.49699	84.07311
57.95364	84.85699	57.59842	73.94102	54.53824	84.0372
57.99826	84.83882	57.6376	73.90111	54.5801	84.00239
58.04772	84.82359	57.67578	73.86886	54.6245	83.9673
58.09266	84.81374	57.7212	73.82809	54.66612	83.93204
58.13625	84.80577	57.7667	73.78947	54.70986	83.8968
58.18545	84.80521	57.8084	73.75024	54.75488	83.86041
58.22894	84.79756	57.85334	73.71074	54.79916	83.82479
58.27722	84.79169	57.89887	73.67436	54.83914	83.78979
58.32481	84.77623	57.9432	73.63598	54.88394	83.75518
58.37056	84.75426	57.98962	73.59791	54.929	83.72093
58.41697	84.74409	58.03579	73.56021	54.96287	83.69253
58.45906	84.73157	58.07993	73.52011	55.00379	83.65641
58.4978	84.7239	58.12358	73.4815	55.04919	83.62097
58.54444	84.71843	58.1537	73.45135	55.09193	83.58593

58.58886	84.70662	58.20332	73.41276	55.13515	83.55113
58.63132	84.6981	58.25035	73.37591	55.17754	83.51673
58.68189	84.69002	58.2983	73.33877	55.22283	83.48281
58.7271	84.67848	58.34082	73.30188	55.26781	83.44873
58.77135	84.66887	58.39012	73.26611	55.31338	83.41512
58.81688	84.65593	58.43453	73.2309	55.35504	83.38084
58.859	84.64479	58.4795	73.19628	55.4002	83.34652
58.90636	84.63435	58.52587	73.16225	55.44132	83.3124
58.95206	84.62581	58.57066	73.12817	55.48526	83.27829
58.9953	84.62257	58.61436	73.09265	55.52009	83.25156
59.03615	84.61242	58.65285	73.06617	55.56841	83.21742
59.08632	84.60195	58.70308	73.03213	55.60476	83.18355
59.1359	84.59483	58.74887	72.9996	55.65106	83.1492
59.17826	84.58418	58.7955	72.96796	55.69746	83.11593
59.2185	84.57153	58.8447	72.93492	55.73984	83.08308
59.26068	84.55921	58.8864	72.90358	55.7781	83.04997
59.30602	84.55169	58.93134	72.87167	55.82168	83.01752
59.3545	84.54353	58.9802	72.83988	55.86462	82.98368
59.40198	84.53893	59.02671	72.80874	55.90967	82.94955
59.44928	84.5401	59.07164	72.77864	55.95559	82.91595
59.4833	84.53738	59.12024	72.74964	56.0019	82.88257
59.52605	84.53063	59.15926	72.72699	56.04353	82.84993
59.57666	84.52304	59.20704	72.6981	56.09192	82.81769
59.622	84.51632	59.25628	72.67076	56.13628	82.78571
59.66514	84.51073	59.30093	72.64303	56.17125	82.75998
59.71235	84.50193	59.34859	72.61637	56.21402	82.7279
59.75559	84.49219	59.39449	72.59149	56.2577	82.69498
59.80004	84.47898	59.43816	72.5643	56.30214	82.66256
59.84476	84.46402	59.48948	72.538	56.34894	82.63057
59.89283	84.44896	59.53499	72.51256	56.3931	82.59913
59.93829	84.43761	59.58382	72.48753	56.43664	82.5692
59.97399	84.43489	59.63126	72.46424	56.48322	82.53804
60.0196	84.43415	59.6747	72.44557	56.52969	82.50736
60.06092	84.43271	59.71633	72.42221	56.57112	82.47661
60.1114	84.43289	59.76889	72.39867	56.6147	82.44583
60.15678	84.42594	59.81546	72.37531	56.66384	82.4157
60.20069	84.41323	59.864	72.35309	56.70343	82.38522
60.24392	84.40682	59.90979	72.33225	56.74955	82.35459
60.2894	84.40359	59.95967	72.31121	56.79444	82.32421
60.33468	84.40085	60.0077	72.29227	56.83222	82.29965
60.3816	84.39922	60.0583	72.27353	56.87657	82.26943
60.42662	84.39674	60.10525	72.25379	56.91552	82.23993
60.47268	84.3868	60.15134	72.23738	56.96441	82.20967
60.5141	84.37776	60.19773	72.22455	57.01106	82.1807
60.55097	84.37223	60.24754	72.20739	57.05292	82.15125
60.59581	84.36254	60.29516	72.1905	57.09795	82.12254
60.63976	84.35452	60.34049	72.17363	57.14382	82.09435
60.68671	84.35064	60.38884	72.15588	57.1907	82.06585
60.72778	84.34567	60.43745	72.14021	57.234	82.03715
60.7752	84.33688	60.48797	72.12521	57.2814	82.00792
60.8225	84.33086	60.53997	72.11195	57.32688	81.9794
60.86643	84.32864	60.59032	72.09736	57.3747	81.95128
60.91058	84.31882	60.63755	72.08229	57.40429	81.92962
60.95805	84.31173	60.6883	72.06813	57.44886	81.90223
61.00202	84.3084	60.72907	72.05541	57.49492	81.87528
61.03665	84.30724	60.77453	72.04155	57.54218	81.84817
61.08662	84.30682	60.82795	72.02879	57.59127	81.82132
61.13124	84.30346	60.87491	72.01641	57.63758	81.7952

61.1761	84.29761	60.9229	72.00432	57.68307	81.76866
61.21559	84.288	60.97127	71.99183	57.73218	81.7425
61.25994	84.27785	61.02021	71.97992	57.77665	81.71668
61.30579	84.27366	61.0714	71.96726	57.82235	81.69068
61.34536	84.26981	61.1215	71.95513	57.86658	81.66517
61.38819	84.26319	61.1687	71.94492	57.91318	81.63946
61.43712	84.25706	61.22064	71.93552	57.95876	81.61461
61.4836	84.25789	61.25635	71.92841	58.00492	81.58995
61.5215	84.25793	61.30787	71.92032	58.054	81.56556
61.56574	84.25654	61.3537	71.91106	58.09028	81.54617
61.60692	84.25152	61.4072	71.90098	58.13672	81.5219
61.65535	84.24598	61.45469	71.89118	58.187	81.4981
61.69703	84.24094	61.50258	71.88254	58.23157	81.47438
61.73988	84.23543	61.5547	71.8736	58.27683	81.45159
61.78599	84.23144	61.60415	71.86566	58.32454	81.42877
61.8295	84.22539	61.65582	71.85776	58.368	81.40528
61.87621	84.215	61.70306	71.84897	58.41796	81.38303
61.92044	84.20693	61.75064	71.84081	58.46382	81.3609
61.9659	84.19604	61.7905	71.83296	58.5114	81.33846
61.99906	84.18591	61.83781	71.82467	58.55831	81.31706
62.04511	84.18084	61.89068	71.81668	58.60451	81.29572
62.0874	84.17476	61.93651	71.80821	58.6505	81.27515
62.13366	84.17676	61.98772	71.80139	58.69996	81.25495
62.17418	84.18124	62.0364	71.79385	58.75092	81.23485
62.2182	84.1822	62.08179	71.78547	58.78688	81.21893
62.26548	84.17872	62.12868	71.77842	58.83108	81.19832
62.30845	84.17122	62.18128	71.7716	58.8784	81.17857
62.34994	84.16596	62.23048	71.76453	58.92546	81.15834
62.39301	84.15583	62.27546	71.75912	58.97394	81.13899
62.44262	84.15163	62.31028	71.75516	59.0207	81.11977
62.48238	84.14683	62.3593	71.74921	59.07063	81.10063
62.52926	84.13991	62.40744	71.7428	59.11889	81.08246
62.56345	84.13622	62.45506	71.73618	59.1627	81.06297
62.60467	84.12866	62.5025	71.72821	59.2114	81.04412
62.64954	84.12303	62.55349	71.72085	59.25579	81.02515
62.69055	84.1197	62.5993	71.71415	59.30844	81.00674
62.73726	84.11713	62.64882	71.70815	59.35586	80.99051
62.78202	84.11365	62.69555	71.7023	59.39156	80.9775
62.82358	84.11194	62.7407	71.69668	59.43894	80.96126
62.87294	84.11098	62.789	71.69215	59.48655	80.94513
62.91452	84.10727	62.82641	71.68837	59.53428	80.92804
62.96324	84.10682	62.87402	71.68305	59.57851	80.91056
63.00508	84.10412	62.92309	71.67782	59.62844	80.89382
63.03584	84.10151	62.96812	71.67232	59.67482	80.87675
63.08108	84.09807	63.01789	71.667	59.72372	80.86071
63.12528	84.09297	63.06586	71.66257	59.76899	80.84569
63.16988	84.09331	63.11226	71.65878	59.81888	80.8306
63.21292	84.09338	63.16068	71.65497	59.86832	80.81627
63.25639	84.08614	63.20592	71.64887	59.91272	80.80111
63.30085	84.08249	63.2558	71.64359	59.95958	80.78573
63.34416	84.0754	63.30478	71.63698	60.00654	80.77056
63.3899	84.06084	63.34081	71.63132	60.05616	80.75608
63.43094	84.05316	63.38478	71.62699	60.09168	80.74469
63.47288	84.04667	63.42954	71.62298	60.14139	80.73007
63.50851	84.03961	63.47451	71.61924	60.1889	80.71595
63.5526	84.03666	63.52469	71.61631	60.23499	80.70163
63.5965	84.03759	63.5669	71.61137	60.28	80.68754
63.64172	84.04206	63.61389	71.606	60.33074	80.67421

63.68375	84.0483	63.65954	71.60194	60.37888	80.661
63.72506	84.04245	63.7078	71.59659	60.4278	80.6475
63.77094	84.03571	63.75774	71.59208	60.4728	80.63424
63.81272	84.0288	63.80012	71.58726	60.51746	80.62066
63.857	84.01987	63.83814	71.58337	60.56924	80.60791
63.89965	84.01835	63.88694	71.57869	60.61785	80.59561
63.93994	84.01847	63.92887	71.57662	60.65955	80.58339
63.9775	84.01241	63.97414	71.57294	60.7073	80.57161
64.02152	84.0112	64.02282	71.56936	60.75253	80.55888
64.06518	84.01002	64.06695	71.56577	60.79197	80.54905
64.10784	84.00917	64.11145	71.55947	60.836	80.53672
64.15006	84.00963	64.15757	71.55509	60.88821	80.52452
64.19786	84.00578	64.20764	71.55083	60.92949	80.5122
64.2398	84.00413	64.25351	71.54778	60.98136	80.50026
64.27868	84.00602	64.295	71.54796	61.02407	80.4894
64.32516	84.00704	64.33404	71.54587	61.07402	80.47817
64.36676	84.01095	64.37815	71.54247	61.11876	80.46759
64.41455	84.01591	64.42461	71.53983	61.16566	80.45622
64.45615	84.01169	64.47012	71.53447	61.2117	80.44431
64.49889	84.00021	64.5153	71.52981	61.25702	80.4331
64.53428	83.99057	64.55938	71.52689	61.30322	80.42227
64.57668	83.97998	64.605	71.52299	61.35289	80.41249
64.62132	83.96837	64.65211	71.52045	61.39132	80.40354
64.66566	83.97391	64.69526	71.51856	61.4369	80.39298
64.70691	83.98313	64.74047	71.51522	61.4834	80.38257
64.75006	83.98487	64.78892	71.5117	61.5323	80.37175
64.7908	83.98758	64.82599	71.50866	61.57818	80.3623
64.83271	83.98616	64.86759	71.50497	61.62441	80.35264
64.87911	83.97314	64.91545	71.50364	61.66761	80.34233
64.92109	83.96348	64.95832	71.5018	61.7176	80.33172
64.96754	83.96133	65.00354	71.49834	61.76574	80.32117
64.99474	83.95837	65.04648	71.49546	61.8091	80.31078
65.04336	83.95909	65.093	71.49111	61.85776	80.30167
65.089	83.95596	65.13382	71.48741	61.90498	80.29261
65.12871	83.95379	65.18098	71.48482	61.9457	80.28315
65.17261	83.94662	65.22494	71.48175	61.99624	80.27369
65.2164	83.93681	65.27138	71.47756	62.04332	80.26288
65.25615	83.9365	65.30708	71.47577	62.07874	80.25511
65.30413	83.934	65.3511	71.47381	62.12371	80.24518
65.34759	83.93384	65.3948	71.47068	62.16961	80.23621
65.38984	83.93591	65.4386	71.47068	62.21505	80.2279
65.43329	83.93443	65.48047	71.47281	62.26342	80.21891
65.47082	83.93157	65.52708	71.47228	62.3085	80.21022
65.51234	83.92494	65.57175	71.47102	62.35586	80.20006
65.5554	83.91771	65.62018	71.46736	62.40193	80.19067
65.59907	83.91432	65.66345	71.46018	62.45082	80.18184
65.64016	83.91607	65.70484	71.45568	62.49938	80.17376
65.68365	83.91585	65.7498	71.45313	62.5414	80.16572
65.72744	83.91448	65.78626	71.45169	62.5862	80.15802
65.7732	83.91407	65.82916	71.45037	62.63818	80.15007
65.81558	83.91037	65.87012	71.44752	62.68322	80.14176
65.85676	83.90648	65.91486	71.44394	62.72902	80.13356
65.90132	83.90105	65.96125	71.44127	62.766	80.12705
65.93791	83.89595	66.00444	71.43948	62.8119	80.11829
65.97714	83.88977	66.0457	71.43767	62.85642	80.10967
66.02031	83.88135	66.09766	71.43658	62.9025	80.10173
66.06482	83.88059	66.1416	71.43414	62.94782	80.09327
66.1072	83.87992	66.17958	71.4295	62.99632	80.08577

66.14942	83.8753	66.223	71.42685	63.04242	80.07742
66.19268	83.87417	66.26124	71.42435	63.0886	80.06962
66.23506	83.87357	66.30362	71.42176	63.13256	80.0616
66.27844	83.87229	66.34753	71.41993	63.17776	80.05364
66.32421	83.8706	66.38999	71.4175	63.22394	80.04694
66.36399	83.86796	66.4354	71.41512	63.26695	80.03927
66.40579	83.86225	66.47716	71.41352	63.3094	80.03165
66.44531	83.85946	66.52265	71.41213	63.34911	80.02529
66.48042	83.85818	66.56764	71.41082	63.39459	80.0174
66.52322	83.85413	66.61028	71.40966	63.4375	80.00966
66.566	83.85588	66.65392	71.4068	63.48666	80.00239
66.61166	83.85368	66.69922	71.40376	63.52943	79.99469
66.65372	83.85055	66.7354	71.40246	63.56926	79.98651
66.6946	83.85427	66.77516	71.4003	63.61713	79.97894
66.73984	83.85691	66.81821	71.39751	63.66241	79.97131
66.78259	83.85864	66.8641	71.39579	63.70665	79.96511
66.82698	83.86036	66.90538	71.39336	63.75284	79.95914
66.8664	83.85781	66.9498	71.3927	63.79392	79.95198
66.91334	83.85068	66.98945	71.39189	63.83933	79.94537
66.94887	83.84175	67.03515	71.38999	63.88563	79.93786
66.99098	83.83664	67.07701	71.38867	63.92719	79.93011
67.03284	83.83059	67.11953	71.38387	63.97511	79.92309
67.07476	83.82916	67.1645	71.38192	64.00434	79.91721
67.1215	83.83558	67.19794	71.38066	64.05024	79.91091
67.15902	83.83434	67.2393	71.37728	64.0968	79.90447
67.1997	83.83468	67.28311	71.37638	64.1423	79.89816
67.24574	83.83391	67.32762	71.37466	64.19001	79.89157
67.29101	83.83131	67.37395	71.37231	64.23669	79.88465
67.33178	83.83208	67.41259	71.37195	64.2768	79.87781
67.37343	83.82782	67.4561	71.37047	64.32289	79.87006
67.40947	83.82807	67.49966	71.36789	64.37074	79.8637
67.45028	83.82728	67.542	71.36576	64.41568	79.85698
67.49304	83.82574	67.5853	71.3627	64.45894	79.8503
67.53663	83.82764	67.63595	71.36086	64.50361	79.8445
67.57764	83.829	67.66686	71.35996	64.54529	79.83802
67.62356	83.83115	67.71293	71.35916	64.59192	79.83176
67.66726	83.83143	67.75228	71.35774	64.6347	79.82539
67.71011	83.82886	67.794	71.35522	64.67437	79.8208
67.75271	83.81821	67.83688	71.35304	64.71832	79.81362
67.79274	83.80384	67.88105	71.35122	64.75984	79.80656
67.83506	83.79241	67.9249	71.34834	64.80514	79.7998
67.86969	83.78851	67.96975	71.3448	64.85	79.79317
67.91158	83.79297	68.0132	71.34137	64.89404	79.78728
67.95415	83.79564	68.0556	71.33784	64.94241	79.78074
67.99586	83.79629	68.0993	71.33617	64.98443	79.77444
68.03943	83.79806	68.13762	71.33613	65.03061	79.76765
68.08352	83.79443	68.18165	71.33441	65.07468	79.76132
68.12294	83.79058	68.2222	71.33365	65.11781	79.75509
68.16919	83.7886	68.26285	71.33394	65.162	79.74901
68.20918	83.78486	68.30893	71.33418	65.20291	79.74308
68.24941	83.78731	68.35074	71.33452	65.2409	79.73819
68.2886	83.78756	68.39505	71.33367	65.28526	79.73238
68.33515	83.78956	68.43824	71.33203	65.32836	79.72667
68.37692	83.78934	68.47962	71.32969	65.37382	79.72049
68.41028	83.78196	68.52311	71.32669	65.41653	79.71392
68.45393	83.77768	68.56757	71.32525	65.46198	79.70716
68.49753	83.77234	68.60398	71.32511	65.50146	79.70056
68.53943	83.76339	68.64711	71.32438	65.54732	79.69468

68.58374	83.75785	68.68811	71.32586	65.59079	79.6894
68.62478	83.75717	68.732	71.32409	65.64074	79.68372
68.66511	83.76057	68.77466	71.32014	65.68134	79.67794
68.70764	83.76242	68.81449	71.31712	65.72224	79.67171
68.75241	83.76069	68.8606	71.313	65.76505	79.665
68.7964	83.757	68.90328	71.31312	65.80979	79.65954
68.8347	83.74979	68.94688	71.31246	65.85731	79.65413
68.87024	83.75004	68.98559	71.31133	65.89024	79.64961
68.91576	83.74984	69.02753	71.31005	65.93194	79.64472
68.95814	83.75065	69.06025	71.30788	65.97903	79.63968
69.00002	83.75597	69.10608	71.30591	66.02005	79.63486
69.04485	83.75348	69.1514	71.30432	66.0627	79.62992
69.08548	83.75437	69.19534	71.30342	66.10438	79.62423
69.12715	83.75362	69.2403	71.3014	66.14813	79.61771
69.16726	83.75143	69.2812	71.3001	66.19488	79.61127
69.21694	83.75303	69.32345	71.2989	66.23822	79.6053
69.25708	83.7498	69.36761	71.29631	66.27857	79.59944
69.29944	83.75203	69.40772	71.29601	66.31914	79.59355
69.33344	83.75296	69.45356	71.29584	66.36639	79.58793
69.37609	83.74703	69.49308	71.29494	66.40749	79.58207
69.417	83.74682	69.52803	71.29538	66.44841	79.57668
69.45569	83.74182	69.5721	71.29429	66.49634	79.57204
69.50066	83.7388	69.61218	71.29252	66.53026	79.56813
69.54243	83.74228	69.65591	71.29136	66.56994	79.56322
69.58711	83.73976	69.70154	71.28918	66.61634	79.55822
69.6247	83.73598	69.74021	71.287	66.66018	79.55293
69.66844	83.72744	69.7817	71.28515	66.7019	79.54786
69.70884	83.72265	69.82642	71.28411	66.74487	79.54213
69.75379	83.72613	69.86606	71.28443	66.78974	79.53687
69.78875	83.73047	69.90855	71.28566	66.83004	79.53134
69.82759	83.73332	69.95578	71.28492	66.8749	79.52549
69.86942	83.72822	69.98785	71.28422	66.91938	79.5203
69.91157	83.71966	70.03012	71.28247	66.96089	79.51536
69.95499	83.71615	70.07374	71.27859	67.00459	79.51036
69.99716	83.71647	70.11592	71.27646	67.05071	79.50524
70.04562	83.71638	70.15766	71.27494	67.08189	79.50097
70.08509	83.71718	70.19812	71.27301	67.12574	79.49593
70.1302	83.71403	70.24071	71.272	67.16785	79.49042
70.17444	83.70998	70.28241	71.2693	67.21142	79.48488
70.21668	83.7121	70.32669	71.26864	67.25618	79.47998
70.25718	83.71211	70.36834	71.26824	67.29941	79.47483
70.3021	83.71015	70.41024	71.26799	67.34126	79.47052
70.33811	83.7091	70.44744	71.26907	67.38238	79.46598
70.37944	83.70611	70.48744	71.26686	67.42876	79.46095
70.42331	83.70437	70.52962	71.26381	67.4685	79.45575
70.46618	83.69932	70.57106	71.26095	67.51415	79.44975
70.50821	83.6946	70.6136	71.25809	67.55268	79.4449
70.54924	83.69274	70.65692	71.25664	67.59561	79.4401
70.58882	83.69375	70.70009	71.25629	67.64164	79.43475
70.63135	83.6981	70.74311	71.25654	67.68387	79.43001
70.6751	83.69732	70.78266	71.25784	67.71759	79.42592
70.71622	83.69279	70.82249	71.25803	67.76313	79.4209
70.75968	83.69167	70.86598	71.25704	67.80264	79.41611
70.78979	83.68686	70.90432	71.256	67.84458	79.41164
70.83782	83.68197	70.94342	71.25345	67.88924	79.40685
70.87772	83.67822	70.98834	71.25084	67.93442	79.40194
70.91796	83.66993	71.02688	71.25008	67.97462	79.39708
70.966	83.6717	71.07081	71.24866	68.0197	79.39187

71.00584	83.67433	71.11157	71.24792	68.06255	79.38719
71.04636	83.67415	71.15634	71.24687	68.10425	79.38236
71.0925	83.6779	71.19828	71.2453	68.15032	79.37722
71.13422	83.66952	71.2395	71.24536	68.19006	79.37138
71.176	83.66814	71.28174	71.24495	68.22906	79.3661
71.22045	83.67519	71.32558	71.24341	68.27343	79.3608
71.25585	83.67872	71.36098	71.24275	68.31793	79.3556
71.29345	83.68522	71.40142	71.23983	68.35316	79.35214
71.33416	83.68683	71.4468	71.23623	68.39584	79.34745
71.37728	83.68155	71.48578	71.23755	68.43609	79.34262
71.42418	83.67496	71.52528	71.23699	68.48349	79.33737
71.46474	83.666	71.56997	71.23707	68.52347	79.33239
71.50818	83.66092	71.61536	71.23612	68.5691	79.32721
71.5529	83.6605	71.65653	71.2325	68.61118	79.32288
71.59213	83.65874	71.69816	71.23126	68.64916	79.31861
71.63497	83.65548	71.7425	71.23081	68.69944	79.31449
71.67519	83.65548	71.78472	71.23014	68.73952	79.30985
71.7108	83.65663	71.819	71.23048	68.77968	79.30516
71.75012	83.65644	71.8603	71.23009	68.8257	79.3009
71.79296	83.66003	71.9044	71.22862	68.86394	79.29672
71.83624	83.66378	71.94722	71.22815	68.90231	79.29347
71.88202	83.65953	71.98876	71.22773	68.94564	79.28865
71.92138	83.65504	72.03228	71.22645	68.98614	79.28381
71.96343	83.64803	72.0704	71.22455	69.0298	79.27829
72.00855	83.64202	72.11889	71.22201	69.071	79.27244
72.0478	83.63957	72.15994	71.22036	69.11306	79.26707
72.09184	83.63805	72.20194	71.2196	69.1583	79.26247
72.1353	83.63455	72.24376	71.21804	69.20043	79.25802
72.17862	83.63259	72.28026	71.21812	69.24322	79.25423
72.22016	83.6316	72.31821	71.21649	69.2905	79.24988
72.25756	83.62795	72.36444	71.21317	69.33014	79.24517
72.2949	83.63223	72.4003	71.21199	69.37566	79.24019
72.3381	83.63868	72.44444	71.21313	69.41806	79.23487
72.38174	83.63715	72.48422	71.21279	69.45894	79.23051
72.42361	83.63445	72.52553	71.21303	69.49639	79.22578
72.4649	83.6279	72.56938	71.21274	69.53461	79.22264
72.5091	83.61564	72.60718	71.21029	69.57847	79.2189
72.55156	83.61154	72.65412	71.20986	69.62012	79.21474
72.59424	83.61498	72.69913	71.2085	69.66381	79.20947
72.63544	83.61826	72.73042	71.20768	69.70694	79.20467
72.67369	83.62566	72.77304	71.20582	69.75055	79.19933
72.70929	83.62671	72.81792	71.20473	69.79039	79.19433
72.75269	83.61787	72.85956	71.2033	69.83535	79.1906
72.79148	83.61286	72.90343	71.20277	69.87852	79.18611
72.8353	83.60656	72.94253	71.20327	69.91812	79.18211
72.87812	83.60447	72.98311	71.20192	69.96381	79.17795
72.92408	83.61468	73.02796	71.20125	70.005	79.17343
72.96772	83.61909	73.066	71.20073	70.04897	79.16844
73.00968	83.61681	73.11132	71.19784	70.08878	79.16372
73.05269	83.6147	73.1543	71.19702	70.13563	79.15907
73.094	83.60853	73.18768	71.19596	70.16846	79.1555
73.13772	83.60766	73.23128	71.19628	70.21112	79.15089
73.16959	83.6084	73.2767	71.19714	70.25498	79.1466
73.21168	83.61054	73.31418	71.19565	70.29576	79.14267
73.25656	83.612	73.35587	71.19489	70.3397	79.13815
73.29902	83.60928	73.39603	71.19368	70.38	79.13457
73.341	83.6059	73.44184	71.19066	70.4174	79.13085
73.37951	83.60089	73.48345	71.18992	70.46396	79.12618

73.4212	83.5933	73.52756	71.18909	70.50785	79.12227
73.46666	83.59149	73.5672	71.18628	70.55121	79.11797
73.5061	83.59542	73.60866	71.18626	70.59356	79.11366
73.5457	83.5994	73.64148	71.18589	70.63391	79.10942
73.59189	83.60297	73.6855	71.18519	70.6824	79.10473
73.62469	83.60358	73.72589	71.18655	70.71448	79.10118
73.66582	83.60038	73.76944	71.18542	70.7586	79.09635
73.71006	83.59605	73.81045	71.18318	70.8016	79.09173
73.75018	83.59568	73.85494	71.18278	70.8428	79.08691
73.79337	83.59184	73.89961	71.1802	70.88549	79.08239
73.83691	83.58794	73.93915	71.17958	70.92784	79.07764
73.87844	83.58351	73.98525	71.17974	70.9726	79.07313
73.92245	83.57763	74.025	71.17793	71.01399	79.06891
73.96503	83.57019	74.06688	71.17621	71.0555	79.0648
74.00796	83.56584	74.09885	71.17662	71.09705	79.06155
74.04796	83.56537	74.14328	71.17692	71.1435	79.05762
74.09156	83.57229	74.1859	71.17667	71.18765	79.05357
74.13306	83.57986	74.22871	71.1757	71.22809	79.04874
74.16538	83.58245	74.26952	71.17401	71.26843	79.04341
74.21254	83.57947	74.31109	71.17292	71.30944	79.03888
74.25044	83.57436	74.35603	71.17219	71.34762	79.03517
74.29834	83.57409	74.39586	71.17187	71.38955	79.03152
74.34045	83.574	74.4347	71.17166	71.43174	79.02765
74.37866	83.57829	74.47734	71.17243	71.4725	79.02389
74.41953	83.57764	74.51944	71.17239	71.51207	79.01999
74.46172	83.57137	74.5551	71.17135	71.55172	79.01552
74.50532	83.56778	74.59524	71.1705	71.59836	79.01132
74.54864	83.56443	74.63903	71.16734	71.64468	79.00715
74.58416	83.56101	74.6829	71.16396	71.6879	79.00354
74.62045	83.56006	74.72346	71.16377	71.72574	78.99954
74.65898	83.55882	74.7653	71.16244	71.76425	78.9952
74.70324	83.55534	74.80752	71.16321	71.80788	78.99027
74.74954	83.55583	74.85034	71.16377	71.85062	78.98483
74.79274	83.55353	74.89343	71.1628	71.89545	78.98004
74.83491	83.5491	74.93087	71.16074	71.93605	78.97636
74.87337	83.54489	74.97431	71.15693	71.97214	78.97371
74.91712	83.54346	75.00589	71.15559	72.01171	78.97007
74.964	83.54554	75.04988	71.15377	72.0525	78.96656
75.00463	83.55252	75.09235	71.15348	72.09599	78.96207
75.04747	83.55597	75.13256	71.15346	72.13756	78.95783
75.08325	83.5549	75.17697	71.15357	72.18109	78.95366
75.12256	83.55012	75.21811	71.15323	72.22124	78.94958
75.16084	83.54234	75.26171	71.15245	72.26556	78.94535
75.2067	83.54491	75.30295	71.15079	72.3071	78.94085
75.25201	83.54073	75.34419	71.14893	72.34903	78.93657
75.29215	83.53286	75.39153	71.14663	72.38978	78.93232
75.33208	83.53041	75.43622	71.14561	72.43276	78.9285
75.37511	83.52135	75.46874	71.14576	72.47466	78.9244
75.41443	83.52239	75.51176	71.14576	72.50772	78.92108
75.4607	83.52572	75.54884	71.14635	72.5522	78.91702
75.50244	83.52619	75.5949	71.1445	72.59632	78.91256
75.53447	83.52814	75.63715	71.14397	72.63821	78.90849
75.57806	83.52518	75.68099	71.14221	72.6813	78.90467
75.61936	83.52449	75.72201	71.13948	72.71887	78.90057
75.66031	83.52143	75.76364	71.1389	72.7604	78.89651
75.70852	83.51496	75.80512	71.13776	72.80681	78.89194
75.75232	83.51455	75.8479	71.13673	72.84582	78.88714
75.78922	83.51258	75.88882	71.13697	72.88724	78.88221

75.82954	83.51515	75.9215	71.13658	72.93162	78.87772
75.87278	83.51836	75.96552	71.13573	72.975	78.87441
75.91522	83.51591	76.00508	71.13378	73.01878	78.8707
75.95605	83.51634	76.04995	71.13384	73.06013	78.86669
76.0008	83.51891	76.09045	71.13349	73.10306	78.8632
76.04538	83.51877	76.1332	71.13222	73.13366	78.85987
76.07795	83.51902	76.17486	71.13186	73.17912	78.85592
76.11966	83.52334	76.21656	71.12952	73.2176	78.85262
76.16558	83.52212	76.25932	71.12835	73.26211	78.8489
76.20415	83.51634	76.30212	71.12635	73.30484	78.8446
76.24675	83.51293	76.34358	71.12576	73.34509	78.84065
76.28603	83.5095	76.3773	71.12573	73.39078	78.83601
76.33056	83.50793	76.41969	71.12342	73.43153	78.83127
76.37504	83.51102	76.4616	71.12349	73.47482	78.82736
76.41766	83.51132	76.50478	71.12351	73.5162	78.82335
76.45586	83.50895	76.54454	71.12281	73.55876	78.81959
76.49792	83.50351	76.58881	71.12446	73.59958	78.81607
76.53052	83.49645	76.63134	71.12639	73.64291	78.81195
76.5729	83.49241	76.67411	71.12478	73.68509	78.80787
76.61665	83.49437	76.71924	71.12397	73.72651	78.80394
76.65656	83.49441	76.75722	71.12175	73.75863	78.80082
76.69981	83.50206	76.80201	71.11775	73.80094	78.79707
76.74366	83.50582	76.83325	71.11641	73.84652	78.79326
76.78472	83.50526	76.87387	71.11715	73.8905	78.78937
76.82406	83.50171	76.92038	71.11663	73.93054	78.78535
76.8658	83.49542	76.95938	71.11666	73.9729	78.78155
76.90707	83.49326	77.00126	71.11739	74.01402	78.77756
76.95212	83.48944	77.04231	71.11619	74.0556	78.77385
76.9886	83.48878	77.0856	71.11746	74.10009	78.77014
77.02874	83.49155	77.12906	71.11986	74.14185	78.76621
77.06908	83.49358	77.16879	71.12305	74.18042	78.76178
77.1105	83.49374	77.21108	71.12421	74.22534	78.75752
77.15268	83.48775	77.25134	71.12419	74.26739	78.75279
77.19778	83.48741	77.28956	71.12325	74.30224	78.74896
77.23872	83.48656	77.32911	71.12008	74.34442	78.7454
77.2804	83.48076	77.37168	71.11962	74.38782	78.74183
77.32608	83.48017	77.41269	71.11884	74.42824	78.73858
77.36593	83.47975	77.45692	71.12092	74.47242	78.73512
77.40834	83.47715	77.49716	71.12251	74.51352	78.73114
77.44211	83.47568	77.5398	71.12475	74.56128	78.72679
77.48382	83.47627	77.58374	71.1248	74.60084	78.72241
77.52813	83.47613	77.62276	71.12332	74.64186	78.71834
77.57005	83.47072	77.66497	71.12195	74.68426	78.7146
77.61226	83.47019	77.70785	71.1173	74.73018	78.71123
77.65338	83.47491	77.74008	71.11611	74.7711	78.70787
77.69838	83.47736	77.78288	71.11323	74.80972	78.70387
77.73847	83.48078	77.8241	71.11155	74.85171	78.69946
77.78136	83.47787	77.86411	71.10899	74.89692	78.69514
77.82382	83.47298	77.9104	71.10863	74.92813	78.69185
77.86765	83.46738	77.95482	71.10664	74.97375	78.68821
77.90831	83.46356	77.99395	71.10589	75.01297	78.68461
77.94947	83.46689	78.033	71.10657	75.05551	78.68073
77.98275	83.46836	78.0783	71.10524	75.09915	78.67603
78.02543	83.47194	78.11909	71.10506	75.13876	78.67158
78.06879	83.47234	78.15878	71.10385	75.18431	78.66736
78.10964	83.46598	78.191	71.10429	75.22456	78.66315
78.15254	83.46292	78.23775	71.10381	75.2707	78.65964
78.19432	83.45766	78.27788	71.10476	75.31181	78.65592

78.23437	83.44878	78.32158	71.10165	75.35337	78.65212
78.28038	83.44615	78.36509	71.09667	75.39586	78.64836
78.32232	83.44307	78.40774	71.09244	75.4396	78.64425
78.36309	83.44374	78.45131	71.09047	75.48132	78.64006
78.40825	83.44587	78.49329	71.09162	75.52206	78.63662
78.43829	83.4496	78.53049	71.09309	75.55475	78.63395
78.481	83.45015	78.57451	71.09277	75.60178	78.6307
78.52398	83.43936	78.6152	71.09129	75.64022	78.62729
78.5686	83.4355	78.65168	71.08924	75.68491	78.62253
78.60825	83.433	78.68732	71.08797	75.7266	78.61795
78.6498	83.43159	78.73368	71.0891	75.76575	78.61334
78.69258	83.43152	78.77403	71.08797	75.8104	78.60871
78.73816	83.43689	78.82056	71.08715	75.85326	78.60568
78.77656	83.44106	78.86142	71.08636	75.896	78.60216
78.81902	83.43976	78.90372	71.08431	75.93598	78.59872
78.86093	83.44402	78.94371	71.08312	75.97824	78.59634
78.89956	83.444	78.98555	71.08275	76.02086	78.59257
78.93987	83.43477	79.02682	71.08222	76.05994	78.58889
78.98344	83.43807	79.06919	71.08233	76.09736	78.5861
79.02182	83.4366	79.10159	71.08082	76.13769	78.58168
79.06472	83.43133	79.14457	71.08052	76.17732	78.57741
79.10239	83.43328	79.18769	71.08147	76.22009	78.57405
79.148	83.43036	79.22903	71.08389	76.26589	78.57066
79.18989	83.42824	79.2693	71.08542	76.3053	78.567
79.22969	83.42905	79.31448	71.08486	76.34795	78.56306
79.27134	83.4272	79.3535	71.08251	76.39039	78.55887
79.3167	83.41996	79.3948	71.0802	76.43381	78.55466
79.35176	83.41975	79.4362	71.07952	76.474	78.55078
79.39257	83.41673	79.4774	71.07765	76.51954	78.54654
79.42974	83.41524	79.51944	71.07745	76.5621	78.54234
79.47529	83.41211	79.555	71.07567	76.60654	78.53789
79.5189	83.40524	79.59629	71.07249	76.64456	78.53331
79.56099	83.40416	79.64015	71.07178	76.68456	78.52976
79.60316	83.40736	79.68109	71.06952	76.7218	78.52717
79.64497	83.41633	79.72043	71.06584	76.76488	78.52371
79.68165	83.4218	79.76278	71.06457	76.8065	78.52072
79.72741	83.4198	79.8059	71.06353	76.84585	78.51742
79.77353	83.41026	79.849	71.06277	76.8872	78.51383
79.818	83.39939	79.89021	71.06399	76.93282	78.50949
79.85803	83.39551	79.9333	71.06433	76.97531	78.50525
79.89153	83.39605	79.97376	71.06307	77.01438	78.5011
79.9321	83.40377	80.00655	71.06336	77.05679	78.49641
79.97718	83.40863	80.05024	71.06404	77.09799	78.49367
80.01592	83.40672	80.09421	71.06307	77.1427	78.49042
80.06074	83.40723	80.1357	71.06198	77.18432	78.48686
80.10095	83.40527	80.17628	71.06065	77.22588	78.48357
80.14125	83.40322	80.22022	71.05816	77.26515	78.47919
80.18413	83.40755	80.266	71.05853	77.30616	78.47512
80.22819	83.41096	80.30581	71.05725	77.34402	78.47232
80.26915	83.41053	80.34834	71.05537	77.38372	78.46849
80.31414	83.40927	80.3898	71.05369	77.42615	78.4647
80.34919	83.40698	80.4323	71.05174	77.4675	78.46067
80.39081	83.40173	80.46781	71.05204	77.50988	78.4565
80.43168	83.39734	80.5101	71.05394	77.5519	78.45262
80.47551	83.39354	80.54872	71.05515	77.5942	78.44873
80.51765	83.39338	80.59303	71.05578	77.63813	78.44546
80.5597	83.39288	80.63744	71.05704	77.67798	78.44171
80.60035	83.38894	80.68012	71.05564	77.71729	78.43766

80.6417	83.38685	80.72116	71.05422	77.76274	78.43368
80.68613	83.38407	80.76196	71.05324	77.8042	78.42965
80.72488	83.38138	80.80528	71.05106	77.85182	78.42569
80.76212	83.38233	80.84729	71.04926	77.88644	78.42273
80.80121	83.38114	80.88766	71.04796	77.92484	78.41936
80.84546	83.37999	80.92194	71.04528	77.96895	78.41574
80.88834	83.37667	80.96413	71.0428	78.01259	78.41294
80.92902	83.37012	81.00579	71.04242	78.05139	78.40979
80.96915	83.37129	81.04438	71.04231	78.095	78.40615
81.00982	83.37476	81.09112	71.04276	78.13472	78.40266
81.0536	83.37141	81.13647	71.04315	78.17611	78.39835
81.0949	83.36493	81.1754	71.04274	78.21958	78.3941
81.13584	83.35536	81.21876	71.04238	78.26535	78.39022
81.1822	83.34618	81.26178	71.04384	78.30509	78.38588
81.21985	83.34496	81.30499	71.04243	78.34592	78.38212
81.25261	83.3484	81.34372	71.04145	78.38816	78.37886
81.2967	83.35268	81.38147	71.0407	78.43068	78.37555
81.33503	83.3554	81.41801	71.038	78.47284	78.37276
81.37915	83.3523	81.45818	71.03722	78.50522	78.37
81.42162	83.34784	81.5021	71.0359	78.55106	78.36626
81.46218	83.3531	81.54584	71.03513	78.59234	78.36268
81.50491	83.35873	81.58639	71.03377	78.63528	78.35851
81.5485	83.35994	81.62694	71.03296	78.67645	78.35532
81.58691	83.36213	81.66924	71.0323	78.71842	78.3519
81.63187	83.3579	81.71478	71.03107	78.76366	78.34822
81.67059	83.35037	81.75353	71.03011	78.80332	78.3445
81.71772	83.35207	81.79438	71.0313	78.8465	78.34035
81.75922	83.3575	81.82836	71.03307	78.8891	78.33636
81.7934	83.35827	81.87001	71.03241	78.92966	78.3324
81.8385	83.36153	81.91426	71.0317	78.97343	78.32869
81.88347	83.36357	81.95387	71.03017	79.0127	78.3242
81.92374	83.36186	81.99901	71.02655	79.05321	78.32021
81.96455	83.35918	82.04324	71.02676	79.09832	78.31614
82.0092	83.35544	82.08295	71.02598	79.131	78.31323
82.0481	83.35772	82.1241	71.0247	79.175	78.31025
82.09347	83.35788	82.16664	71.02494	79.21772	78.30692
82.13425	83.3572	82.20746	71.02408	79.25728	78.30319
82.181	83.35482	82.24945	71.02427	79.30005	78.29908
82.22339	83.3523	82.28504	71.02364	79.34086	78.29501
82.25728	83.35049	82.32612	71.02152	79.38384	78.29114
82.29803	83.34704	82.36809	71.02054	79.42429	78.2879
82.34149	83.34936	82.41121	71.01953	79.46662	78.28462
82.38495	83.35379	82.45256	71.01952	79.50662	78.28099
82.42555	83.35773	82.49395	71.0199	79.54995	78.27773
82.46776	83.35962	82.53563	71.02084	79.59109	78.27373
82.5072	83.35799	82.57837	71.01978	79.63099	78.2703
82.55282	83.35203	82.61906	71.01778	79.66376	78.26802
82.59494	83.34539	82.66056	71.01691	79.7077	78.26415
82.63556	83.34271	82.70312	71.01407	79.74843	78.26082
82.67538	83.33927	82.73301	71.01466	79.79468	78.25694
82.71532	83.3366	82.7811	71.01628	79.83629	78.25275
82.7529	83.33447	82.82179	71.01447	79.87979	78.24916
82.79425	83.33142	82.86293	71.01293	79.91993	78.24552
82.83651	83.32887	82.9045	71.01139	79.96422	78.24174
82.8827	83.32919	82.94758	71.00896	80.00493	78.23842
82.92676	83.3332	82.98993	71.00836	80.05253	78.23455
82.96551	83.3374	83.03334	71.00831	80.08946	78.23053
83.00799	83.33755	83.0746	71.00737	80.13458	78.22739

83.0466	83.33044	83.11736	71.00751	80.1769	78.22377
83.09132	83.32066	83.15976	71.00837	80.21843	78.22054
83.1336	83.31432	83.19668	71.00714	80.25605	78.21728
83.16938	83.31269	83.23676	71.00628	80.2934	78.21454
83.21522	83.31718	83.27616	71.0064	80.33874	78.21066
83.25331	83.3219	83.31666	71.00534	80.37881	78.2068
83.29738	83.31716	83.35962	71.00485	80.42262	78.20307
83.34137	83.30934	83.40172	71.004	80.4624	78.19952
83.38131	83.30219	83.44605	71.00171	80.50566	78.19564
83.42399	83.30167	83.48884	71.00074	80.54776	78.19245
83.46669	83.30637	83.53012	71.00122	80.58955	78.18888
83.50553	83.31358	83.57043	71.00127	80.63313	78.18532
83.54731	83.31186	83.61126	71.00074	80.6729	78.18233
83.59056	83.30699	83.64336	71.00007	80.70918	78.17897
83.6321	83.30493	83.68676	70.99951	80.7572	78.1757
83.67387	83.30761	83.72738	70.99783	80.80102	78.17167
83.7091	83.31549	83.77064	70.99628	80.84218	78.16773
83.75189	83.31786	83.81098	70.99577	80.88446	78.16355
83.78928	83.31123	83.85492	70.99519	80.91578	78.16051
83.83205	83.3019	83.89648	70.99473	80.95842	78.15732
83.87696	83.29386	83.94091	70.99505	81.00391	78.15405
83.91812	83.28845	83.98239	70.99539	81.04425	78.15048
83.9604	83.29243	84.02672	70.99507	81.08691	78.14689
84.00478	83.29301	84.0657	70.99487	81.12507	78.14333
84.05049	83.28507	84.09832	70.99437	81.16946	78.13894
84.08901	83.28114	84.14232	70.99208	81.21106	78.1355
84.1322	83.26999	84.18437	70.98909	81.2505	78.13204
84.16398	83.26823	84.22652	70.98658	81.29372	78.12808
84.20728	83.26814	84.2618	70.9863	81.33487	78.1252
84.24545	83.26302	84.30407	70.98687	81.37836	78.12184
84.291	83.26134	84.34842	70.98754	81.41825	78.11826
84.33292	83.25378	84.39141	70.98747	81.4519	78.11562
84.37326	83.25076	84.43225	70.98526	81.49292	78.11242
84.41576	83.25152	84.47676	70.98361	81.53206	78.10876
84.45912	83.25402	84.5186	70.98171	81.57588	78.10472
84.50232	83.25815	84.55116	70.98097	81.61565	78.10053
84.54212	83.26219	84.59371	70.98186	81.6597	78.09562
84.5853	83.26798	84.63856	70.98235	81.70175	78.09149
84.61714	83.27336	84.67837	70.98253	81.74438	78.08779
84.66	83.28045	84.71976	70.98316	81.7799	78.08432
84.70131	83.28629	84.76191	70.98316	81.82203	78.08159
84.742	83.29104	84.80316	70.98273	81.86919	78.07854
84.78798	83.29093	84.84641	70.98374	81.91096	78.07496
84.82853	83.28844	84.88732	70.98449	81.95362	78.07178
84.87445	83.2846	84.93179	70.98336	81.99656	78.06757
84.91159	83.2776	84.97031	70.982	82.03761	78.06337
84.95503	83.26754	85.00555	70.98044	82.07	78.06064
84.99422	83.25962	85.04166	70.97731	82.11218	78.05693
85.03878	83.25992	85.08859	70.97471	82.15598	78.05357
85.07332	83.26313	85.12781	70.97278	82.20109	78.05009
85.11334	83.27408	85.16704	70.9706	82.24179	78.0468
85.15445	83.28482	85.21038	70.96981	82.28222	78.04344
85.19318	83.2834	85.25644	70.96997	82.32184	78.04032
85.23831	83.28104	85.29542	70.97075	82.36632	78.0378
85.28069	83.27725	85.33862	70.97266	82.40675	78.03431
85.32679	83.27391	85.38019	70.97334	82.44996	78.03109
85.36762	83.26849	85.42382	70.97285	82.49285	78.0273
85.40905	83.26584	85.45594	70.97217	82.5365	78.02318

85.45449	83.26241	85.49778	70.97015	82.57826	78.01934
85.49556	83.26347	85.54224	70.96886	82.61922	78.01544
85.53598	83.26221	85.58418	70.96859	82.66056	78.0119
85.57644	83.25713	85.62691	70.96759	82.69476	78.00913
85.6066	83.2576	85.66856	70.9677	82.73666	78.00546
85.65219	83.25187	85.712	70.9671	82.78299	78.00145
85.69429	83.25026	85.75218	70.9663	82.8252	77.9972
85.73874	83.2543	85.79648	70.96697	82.86788	77.99263
85.77886	83.25317	85.83454	70.96636	82.90874	77.98906
85.81947	83.2495	85.87882	70.9657	82.94944	77.9864
85.86145	83.24395	85.91306	70.96498	82.98982	77.9836
85.90313	83.23995	85.95712	70.96358	83.03242	77.98121
85.94795	83.23931	85.99689	70.96157	83.07531	77.9785
85.99114	83.24446	86.03869	70.96028	83.11903	77.9753
86.03466	83.25142	86.08246	70.95942	83.15688	77.97195
86.063	83.25367	86.12345	70.95954	83.20421	77.96846
86.1045	83.25341	86.16509	70.95978	83.23475	77.96576
86.1472	83.25076	86.20715	70.95838	83.27658	77.9621
86.19262	83.24893	86.24736	70.95886	83.32014	77.9588
86.23484	83.24721	86.29259	70.95765	83.36132	77.95536
86.27715	83.24439	86.33572	70.95731	83.4047	77.95171
86.3207	83.24232	86.36726	70.95862	83.44624	77.94741
86.35995	83.24478	86.40725	70.95836	83.49313	77.94353
86.40031	83.2478	86.44968	70.95809	83.53078	77.93987
86.44425	83.24911	86.49478	70.95836	83.56831	77.9365
86.4832	83.24972	86.53522	70.95793	83.61224	77.93368
86.51415	83.25147	86.57785	70.95692	83.65501	77.93041
86.56106	83.25052	86.62172	70.95625	83.69922	77.92725
86.60062	83.24362	86.66101	70.95467	83.74028	77.92383
86.64544	83.23507	86.70328	70.95305	83.7826	77.9197
86.68403	83.2226	86.74334	70.95148	83.82528	77.91616
86.72742	83.2082	86.78759	70.95097	83.85868	77.9131
86.76905	83.19912	86.82172	70.95019	83.90097	77.90839
86.80929	83.20342	86.86486	70.9507	83.94429	77.90487
86.8523	83.21286	86.90262	70.95117	83.98608	77.90143
86.89538	83.21692	86.94772	70.95053	84.02603	77.89792
86.93801	83.22161	86.98813	70.95044	84.06968	77.8953
86.97214	83.22055	87.03351	70.94936	84.11291	77.8919
87.01278	83.21157	87.07386	70.9493	84.15559	77.88839
87.05634	83.20683	87.11447	70.94862	84.19649	77.88462
87.09676	83.20858	87.15839	70.94845	84.24086	77.8809
87.13938	83.21299	87.20025	70.94811	84.28288	77.87738
87.18276	83.21516	87.24261	70.94724	84.3233	77.8739
87.22572	83.21689	87.27485	70.94588	84.3635	77.8703
87.26587	83.21889	87.3193	70.94518	84.40532	77.86725
87.30782	83.21796	87.35931	70.94406	84.44793	77.86461
87.35155	83.22059	87.40225	70.94177	84.47906	77.86204
87.3934	83.22348	87.44476	70.94075	84.52222	77.85895
87.43526	83.22482	87.48456	70.93819	84.56363	77.85545
87.47762	83.22267	87.52986	70.9371	84.60853	77.85179
87.51319	83.22047	87.57042	70.93669	84.6497	77.84797
87.55112	83.21816	87.61276	70.93615	84.695	77.84441
87.59203	83.21706	87.65251	70.9364	84.73508	77.84067
87.63594	83.21593	87.69778	70.93652	84.77916	77.83705
87.67931	83.21312	87.73044	70.9368	84.81939	77.83348
87.72224	83.20455	87.77011	70.93807	84.8613	77.82982
87.76444	83.19097	87.81055	70.93878	84.90688	77.8265
87.80656	83.18397	87.8536	70.93687	84.94668	77.82298

87.85022	83.17439	87.8941	70.93596	84.98626	77.81961
87.88999	83.16708	87.93672	70.93402	85.0235	77.81706
87.93144	83.17081	87.97954	70.93221	85.06168	77.81382
87.96532	83.16974	88.02091	70.93102	85.10528	77.81019
88.00616	83.17339	88.06201	70.92906	85.14447	77.80655
88.04826	83.18225	88.10504	70.92812	85.18725	77.80307
88.09288	83.18193	88.14696	70.92817	85.22844	77.79998
88.13382	83.17457	88.18137	70.92774	85.27191	77.79702
88.17762	83.16955	88.22519	70.92663	85.31524	77.79424
88.2193	83.16609	88.26812	70.92567	85.35681	77.79104
88.26246	83.16696	88.31205	70.92427	85.39982	77.78723
88.30394	83.17313	88.352	70.92484	85.43981	77.78348
88.34657	83.17509	88.39408	70.9251	85.48144	77.77975
88.38455	83.1793	88.43266	70.92485	85.52289	77.77648
88.42093	83.18326	88.47759	70.92336	85.56178	77.77296
88.46388	83.18881	88.52155	70.92162	85.60681	77.76968
88.5058	83.18713	88.56124	70.92088	85.63878	77.76703
88.5468	83.17874	88.60199	70.92069	85.67972	77.76344
88.59242	83.1708	88.63789	70.92139	85.72254	77.75984
88.63328	83.15958	88.67781	70.92139	85.763	77.7567
88.67247	83.15298	88.72122	70.9206	85.80214	77.75341
88.71625	83.15137	88.76319	70.91911	85.84576	77.74967
88.75851	83.15296	88.80336	70.91776	85.88634	77.74668
88.80109	83.15509	88.84924	70.91705	85.92735	77.74359
88.84524	83.15496	88.89102	70.91635	85.97113	77.7409
88.87434	83.15508	88.93224	70.91541	86.01489	77.73817
88.91875	83.15109	88.97501	70.91489	86.05486	77.73493
88.95789	83.14635	89.01766	70.91336	86.09511	77.73128
88.99926	83.14718	89.05897	70.91215	86.13828	77.72752
89.04141	83.15572	89.09018	70.91277	86.1788	77.72409
89.08574	83.16305	89.13478	70.91139	86.21934	77.72084
89.12872	83.16892	89.17559	70.91062	86.25551	77.71787
89.16778	83.17513	89.21589	70.91144	86.29559	77.71444
89.21078	83.16855	89.25848	70.90989	86.33808	77.71025
89.25611	83.16346	89.30151	70.90999	86.38165	77.70658
89.29762	83.15987	89.34295	70.90945	86.42225	77.70306
89.34101	83.15646	89.38328	70.90795	86.46637	77.69911
89.38048	83.15871	89.42725	70.9078	86.50732	77.69612
89.41669	83.15454	89.46375	70.90718	86.54903	77.69282
89.45824	83.15311	89.50705	70.90619	86.58868	77.68973
89.50064	83.15649	89.54218	70.90619	86.62958	77.68647
89.54559	83.15712	89.58641	70.90567	86.67453	77.6832
89.58671	83.16045	89.62313	70.90466	86.71806	77.6795
89.62901	83.16576	89.66719	70.90487	86.76216	77.67622
89.67187	83.166	89.70594	70.90472	86.78976	77.674
89.70897	83.16408	89.74976	70.90487	86.83391	77.67048
89.75484	83.16092	89.78954	70.90476	86.88042	77.66717
89.79699	83.14907	89.83705	70.9035	86.92041	77.66408
89.83887	83.13907	89.87403	70.90258	86.96441	77.66043
89.87192	83.13345	89.91874	70.90113	87.01001	77.65768
89.91572	83.13046	89.96359	70.89986	87.05001	77.65455
89.95819	83.13269	89.99656	70.90004	87.09055	77.65115
89.99843	83.13171	90.0397	70.89992	87.13136	77.64733
90.04131	83.12888	90.08001	70.89917	87.17545	77.64365
90.08259	83.12244	90.1217	70.89987	87.21884	77.63987
90.12543	83.11545	90.16112	70.89868	87.25876	77.63644
90.16636	83.11299	90.20672	70.8966	87.30428	77.63351
90.20731	83.11124	90.25051	70.89478	87.34649	77.63049

90.25108	83.10416	90.29202	70.89288	87.38449	77.62796
90.29454	83.09738	90.33387	70.89229	87.41898	77.62566
90.32728	83.0933	90.37542	70.89291	87.46462	77.62244
90.36822	83.09131	90.41699	70.89359	87.5066	77.61858
90.41338	83.09299	90.45106	70.89399	87.54996	77.61492
90.45538	83.09794	90.49476	70.89355	87.59051	77.61086
90.49518	83.10216	90.53699	70.89236	87.63368	77.60747
90.53586	83.09786	90.57724	70.89042	87.67543	77.60415
90.57424	83.09537	90.619	70.88833	87.71859	77.60062
90.62039	83.09195	90.66194	70.88793	87.75936	77.59778
90.66496	83.09003	90.70374	70.88786	87.80392	77.59492
90.70822	83.09536	90.74616	70.8868	87.84605	77.59264
90.74559	83.10023	90.78832	70.88682	87.88516	77.58951
90.78024	83.1076	90.82764	70.88629	87.92676	77.58606
90.82257	83.11214	90.86822	70.88457	87.97257	77.58286
90.865	83.1091	90.90594	70.88513	88.01652	77.57953
90.90762	83.10873	90.94652	70.88419	88.04601	77.57756
90.95014	83.10862	90.98453	70.88239	88.09069	77.57399
90.99049	83.1091	91.02891	70.88352	88.13306	77.57044
91.03353	83.11077	91.07209	70.88419	88.17462	77.5668
91.07709	83.10582	91.11416	70.88355	88.21406	77.56287
91.1166	83.09677	91.15218	70.88338	88.25821	77.55953
91.15924	83.08698	91.19764	70.88261	88.30431	77.55629
91.2007	83.08203	91.23856	70.88097	88.34455	77.55327
91.24278	83.08425	91.27665	70.88097	88.38795	77.55021
91.28564	83.0891	91.32018	70.87989	88.43141	77.54754
91.32014	83.09553	91.35582	70.87956	88.47442	77.54438
91.36066	83.10181	91.39806	70.87909	88.51612	77.54079
91.40194	83.10277	91.43781	70.87927	88.55919	77.53765
91.44251	83.10094	91.47934	70.87976	88.59184	77.53492
91.48575	83.09225	91.52601	70.87831	88.63989	77.53155
91.52897	83.08668	91.56722	70.87685	88.67831	77.52849
91.57228	83.08588	91.60871	70.87589	88.72071	77.5255
91.61349	83.0827	91.65056	70.87595	88.76399	77.52244
91.65571	83.08616	91.68824	70.87593	88.80575	77.519
91.69855	83.08653	91.73399	70.87571	88.84855	77.51554
91.74035	83.08325	91.77853	70.87467	88.88938	77.51209
91.77207	83.08495	91.80866	70.87363	88.93413	77.50865
91.81515	83.08134	91.85068	70.87239	88.98024	77.50537
91.85559	83.07208	91.89514	70.87239	89.02124	77.50238
91.90178	83.06462	91.9343	70.87178	89.06172	77.49945
91.94337	83.0594	91.97519	70.87157	89.10569	77.49682
91.98344	83.05546	92.01551	70.87049	89.14598	77.49372
92.02801	83.05801	92.05616	70.86903	89.18941	77.4906
92.07339	83.06371	92.09951	70.86825	89.22059	77.48784
92.11368	83.06703	92.1439	70.86662	89.26405	77.48461
92.15558	83.07182	92.18613	70.86618	89.30561	77.48094
92.20191	83.07886	92.23001	70.86591	89.34705	77.47766
92.23211	83.08467	92.26291	70.86589	89.38936	77.47373
92.27795	83.0924	92.30238	70.866	89.43329	77.4703
92.31886	83.09641	92.34872	70.86663	89.46944	77.46744
92.36166	83.08976	92.38864	70.86602	89.51626	77.46423
92.40156	83.07835	92.43101	70.86474	89.55704	77.46083
92.44434	83.06406	92.47199	70.86404	89.59976	77.45717
92.48749	83.05132	92.51814	70.86251	89.64299	77.4536
92.52798	83.04788	92.5575	70.86175	89.6853	77.45074
92.57078	83.05225	92.5965	70.86051	89.73153	77.44854
92.61384	83.06301	92.63959	70.85929	89.77099	77.44536

92.65188	83.07225	92.68014	70.85808	89.80982	77.4425
92.68668	83.07936	92.71235	70.8576	89.84462	77.43957
92.73118	83.08547	92.75819	70.85713	89.88566	77.4358
92.77168	83.07967	92.80066	70.85694	89.93257	77.43309
92.81525	83.07503	92.84397	70.85613	89.97306	77.42995
92.85766	83.0736	92.88501	70.8551	90.01665	77.42714
92.90242	83.07099	92.92741	70.85453	90.05909	77.42426
92.94076	83.07204	92.97118	70.85333	90.09774	77.42034
92.98087	83.073	93.01086	70.85325	90.13964	77.41662
93.02528	83.07003	93.05181	70.85348	90.18246	77.41395
93.06944	83.06521	93.09316	70.85619	90.22553	77.41089
93.11172	83.06409	93.13495	70.85804	90.2672	77.40856
93.15094	83.06591	93.16915	70.85706	90.30907	77.40584
93.19361	83.06897	93.21369	70.85637	90.35125	77.40208
93.22887	83.07048	93.25522	70.85224	90.38349	77.39913
93.2691	83.06857	93.29824	70.84746	90.42438	77.39521
93.31126	83.06497	93.34299	70.84657	90.47068	77.39174
93.35456	83.06117	93.38278	70.8469	90.50905	77.38851
93.39559	83.05845	93.42518	70.84763	90.55234	77.3857
93.43652	83.05796	93.46634	70.849	90.59562	77.3829
93.47908	83.06036	93.50644	70.85074	90.63859	77.37997
93.52262	83.05782	93.54839	70.84952	90.68066	77.37715
93.56332	83.05714	93.58776	70.84815	90.72171	77.37431
93.60703	83.05411	93.62442	70.84762	90.76431	77.37118
93.64676	83.04943	93.66556	70.84574	90.80891	77.36846
93.67894	83.05198	93.70551	70.84388	90.84894	77.36502
93.72074	83.05158	93.74522	70.84371	90.88672	77.3616
93.76336	83.05567	93.78888	70.84364	90.93281	77.35864
93.80561	83.05727	93.83105	70.84468	90.97619	77.35596
93.84851	83.05426	93.87598	70.84571	91.01008	77.35423
93.89051	83.05308	93.91472	70.84497	91.04955	77.35158
93.93043	83.05273	93.95781	70.84279	91.09134	77.34926
93.97297	83.0548	93.99993	70.84063	91.13419	77.34636
94.01434	83.05532	94.04058	70.83938	91.17397	77.34342
94.05519	83.05769	94.07649	70.83982	91.21966	77.34036
94.09944	83.05597	94.11958	70.84022	91.26306	77.33668
94.13312	83.05138	94.16028	70.83881	91.30532	77.33327
94.17681	83.05288	94.19912	70.83727	91.34697	77.32979
94.21444	83.05012	94.24391	70.83571	91.38728	77.32668
94.25701	83.04169	94.28618	70.83514	91.43055	77.32341
94.29829	83.03858	94.33156	70.83636	91.47374	77.32008
94.34094	83.03765	94.36757	70.83516	91.51375	77.31631
94.38165	83.03284	94.40981	70.83395	91.55728	77.31333
94.42532	83.03129	94.45405	70.83253	91.59564	77.31071
94.46332	83.02747	94.49805	70.83004	91.62841	77.30819
94.50564	83.0213	94.53257	70.82869	91.67188	77.30514
94.55103	83.0206	94.57434	70.82841	91.71359	77.30176
94.58324	83.02432	94.61474	70.82788	91.75719	77.29883
94.62612	83.03006	94.65738	70.82827	91.79759	77.29579
94.66599	83.0331	94.69781	70.82911	91.83889	77.29292
94.70924	83.03297	94.73991	70.82957	91.87922	77.28986
94.75234	83.03302	94.7825	70.82882	91.92255	77.28659
94.79172	83.0323	94.82075	70.82834	91.96424	77.28359
94.83495	83.03102	94.86155	70.82909	92.00519	77.28066
94.87518	83.0304	94.90797	70.82768	92.04784	77.27759
94.91925	83.03198	94.94749	70.82713	92.09015	77.27446
94.95929	83.02998	94.97984	70.82524	92.13119	77.27097
95.0009	83.0269	95.0263	70.82272	92.16493	77.26864

95.04522	83.02637	95.06897	70.82123	92.20708	77.26589
95.08655	83.02698	95.11126	70.82017	92.24984	77.26299
95.11897	83.02567	95.15157	70.81936	92.29322	77.26034
95.16446	83.02391	95.19324	70.81928	92.33624	77.25714
95.20576	83.02266	95.23398	70.81928	92.37603	77.2541
95.24936	83.01895	95.27858	70.82085	92.41874	77.25123
95.29119	83.0156	95.32138	70.82304	92.45999	77.2484
95.33253	83.01085	95.36049	70.82248	92.5	77.24545
95.37368	83.00974	95.40265	70.82103	92.54619	77.24184
95.41659	83.00966	95.43492	70.81913	92.58728	77.23869
95.45532	83.01144	95.47746	70.81713	92.62891	77.23531
95.49681	83.01664	95.52051	70.81527	92.66925	77.23253
95.54359	83.01653	95.56406	70.81496	92.71041	77.23016
95.57449	83.00799	95.60219	70.81763	92.75528	77.22678
95.61791	82.99599	95.64518	70.81769	92.78692	77.22431
95.65516	82.98776	95.68875	70.81776	92.82882	77.22049
95.69843	82.9822	95.72969	70.81793	92.86725	77.21751
95.74585	82.98025	95.77211	70.81616	92.90924	77.21466
95.78762	82.982	95.8161	70.81442	92.95124	77.21214
95.82728	82.98067	95.8546	70.81469	92.99353	77.20958
95.86853	82.97506	95.8908	70.81345	93.03594	77.20647
95.91387	82.97573	95.93234	70.81081	93.07843	77.20293
95.95449	82.96811	95.97608	70.80877	93.11548	77.19947
95.99584	82.95954	96.01619	70.80733	93.15939	77.19669
96.0271	82.96135	96.05762	70.80648	93.20382	77.19392
96.07326	82.95822	96.0984	70.80506	93.24618	77.19126
96.11413	82.96491	96.14111	70.80498	93.29074	77.18847
96.15694	82.97638	96.18449	70.80708	93.32908	77.18471
96.19707	82.98345	96.22348	70.81006	93.36968	77.18117
96.24071	82.98729	96.268	70.81199	93.40378	77.17879
96.28368	82.98628	96.30421	70.81234	93.44642	77.17602
96.32953	82.98284	96.34144	70.81084	93.4867	77.17335
96.36899	82.98342	96.38222	70.8065	93.52875	77.17008
96.40828	82.98682	96.42446	70.80393	93.57031	77.16645
96.45176	82.98911	96.46559	70.80371	93.61093	77.16318
96.48624	82.98667	96.50996	70.80299	93.65297	77.1602
96.52966	82.98339	96.55196	70.80206	93.69342	77.15744
96.56969	82.97935	96.59211	70.79974	93.73716	77.15475
96.61364	82.97433	96.63492	70.79859	93.77734	77.152
96.65649	82.97769	96.67389	70.79791	93.82153	77.14913
96.69504	82.97807	96.72122	70.79927	93.86156	77.14591
96.73361	82.97351	96.76389	70.80101	93.90574	77.14309
96.77719	82.97122	96.79504	70.80157	93.94103	77.14106
96.8237	82.96484	96.83434	70.80048	93.98069	77.13792
96.8638	82.95913	96.87751	70.79745	94.02491	77.13543
96.90652	82.95628	96.91898	70.79583	94.06636	77.13287
96.94934	82.95226	96.95929	70.79409	94.10695	77.12937
96.99243	82.9512	97.00176	70.79302	94.14624	77.12646
97.02446	82.94998	97.04908	70.79319	94.18947	77.12318
97.06501	82.94473	97.08947	70.793	94.23072	77.12003
97.10886	82.94596	97.13119	70.79358	94.27375	77.11653
97.14932	82.94552	97.17194	70.79296	94.31415	77.11361
97.18961	82.94127	97.21139	70.79296	94.35725	77.11105
97.23121	82.93955	97.24549	70.79314	94.39794	77.10836
97.27945	82.93614	97.28945	70.79283	94.44232	77.10586
97.3183	82.93476	97.33301	70.79355	94.48272	77.10276
97.35782	82.93682	97.37371	70.79279	94.52525	77.09927
97.40224	82.93894	97.41381	70.79249	94.55882	77.09635

97.44138	82.94241	97.45736	70.79196	94.60087	77.09331
97.48116	82.9432	97.50046	70.79091	94.64322	77.0901
97.51932	82.94296	97.54341	70.79073	94.68352	77.08745
97.5633	82.94691	97.58438	70.78952	94.72805	77.08496
97.60288	82.94813	97.62432	70.78651	94.76505	77.08265
97.64657	82.94663	97.66431	70.78581	94.80672	77.07976
97.68709	82.93974	97.69962	70.78716	94.8483	77.07706
97.72844	82.93211	97.74476	70.78722	94.88824	77.07428
97.77445	82.92122	97.78656	70.78652	94.93406	77.07068
97.81524	82.91378	97.82726	70.78675	94.97785	77.06762
97.85715	82.91244	97.86715	70.78185	95.01811	77.06445
97.90215	82.91054	97.91	70.7789	95.05918	77.06158
97.9377	82.91586	97.95429	70.77882	95.103	77.05902
97.97564	82.91655	97.99674	70.77801	95.14579	77.05656
98.01632	82.91769	98.03751	70.77892	95.17965	77.05431
98.06007	82.91805	98.0787	70.7806	95.2198	77.05154
98.10268	82.91283	98.11969	70.78066	95.26063	77.04817
98.14216	82.90872	98.15384	70.77973	95.30188	77.04452
98.18876	82.90453	98.19616	70.78066	95.34259	77.04115
98.23046	82.90709	98.24074	70.77973	95.38638	77.03813
98.27181	82.91519	98.28022	70.77753	95.42831	77.0359
98.31172	82.92048	98.32284	70.77634	95.47251	77.03322
98.35278	82.92257	98.36402	70.77499	95.51142	77.0311
98.38457	82.91879	98.40831	70.77418	95.55587	77.02816
98.42869	82.91075	98.45047	70.77368	95.59671	77.02515
98.46918	82.90465	98.49236	70.77215	95.64114	77.02248
98.51382	82.90584	98.53612	70.77155	95.68032	77.01966
98.55532	82.9069	98.57592	70.77058	95.7173	77.01756
98.59809	82.915	98.60856	70.77077	95.75775	77.01492
98.63808	82.91936	98.65045	70.77229	95.79778	77.01252
98.68347	82.91903	98.69268	70.77156	95.84147	77.00933
98.72706	82.92193	98.73224	70.77023	95.88201	77.00541
98.76694	82.91766	98.77332	70.77023	95.92528	77.00189
98.81066	82.91361	98.81775	70.76942	95.97024	76.99938
98.85369	82.91092	98.85929	70.76851	96.0107	76.99676
98.89351	82.91213	98.90131	70.7692	96.05072	76.99484
98.92736	82.9141	98.94126	70.76906	96.09222	76.99235
98.96776	82.91325	98.98384	70.76849	96.13438	76.98923
99.01244	82.9132	99.02689	70.76875	96.17984	76.98663
99.05219	82.91156	99.06231	70.76772	96.22437	76.98361
99.09476	82.90966	99.10261	70.76586	96.26447	76.98096
99.13692	82.90842	99.14181	70.76369	96.30491	76.97798
99.17618	82.907	99.18546	70.76257	96.33898	76.97565
99.22071	82.90768	99.23088	70.76339	96.3805	76.9728
99.26298	82.90374	99.26955	70.76388	96.42241	76.96966
99.30449	82.90408	99.31215	70.76407	96.46453	76.96653
99.34344	82.91087	99.35061	70.76407	96.50642	76.96347
99.37902	82.90933	99.39288	70.7631	96.55259	76.96046
99.41768	82.90591	99.43644	70.76131	96.59768	76.95741
99.45852	82.90544	99.47949	70.76075	96.63605	76.95442
99.50181	82.90958	99.51149	70.76063	96.67451	76.95131
99.5442	82.91427	99.55228	70.76097	96.71881	76.94831
99.58676	82.915	99.59976	70.75988	96.76032	76.94582
99.62782	82.91685	99.63928	70.75981	96.80258	76.94368
99.67132	82.91368	99.68075	70.75896	96.84449	76.94095
99.71049	82.90857	99.72232	70.75666	96.88845	76.93845
99.75475	82.90573	99.76372	70.75787	96.93068	76.93562
99.7959	82.90316	99.80535	70.75758	96.96151	76.93325

99.82762	82.8994	99.84799	70.75584	97.00295	76.93087
99.86936	82.89677	99.88805	70.75523	97.04636	76.92818
99.91214	82.89772	99.93169	70.75366	97.08746	76.92545
99.95647	82.90222	99.96753	70.75243	97.13038	76.9226
99.99656	82.90281	100.0083	70.75192	97.17291	76.91952
100.0388	82.90015	100.0501	70.75095	97.21782	76.91696
100.0819	82.89948	100.0927	70.7503	97.25705	76.91405
100.1231	82.89514	100.1322	70.75165	97.30043	76.91075
100.1631	82.89394	100.1732	70.75086	97.34181	76.90779
100.2108	82.89516	100.2132	70.75076	97.38349	76.905
100.2515	82.89478	100.2545	70.75056	97.42372	76.90258
100.286	82.89413	100.2993	70.75045	97.46722	76.90062
100.3254	82.89402	100.3425	70.75111	97.49856	76.89894
100.3686	82.8892	100.3802	70.7499	97.54344	76.89649
100.4095	82.88822	100.4193	70.74876	97.58482	76.89363
100.4536	82.88192	100.4612	70.74748	97.62253	76.89029
100.494	82.87777	100.5004	70.74881	97.66578	76.88729
100.5361	82.87687	100.5398	70.74952	97.7097	76.88431
100.5819	82.87151	100.5856	70.74921	97.75284	76.88211
100.6216	82.87408	100.6283	70.74967	97.79632	76.8796
100.6631	82.87433	100.6693	70.74537	97.83608	76.87719
100.7061	82.87018	100.7116	70.74473	97.87086	76.87407
100.7476	82.86913	100.7519	70.7475	97.91175	76.87053
100.7881	82.87025	100.7956	70.7451	97.95864	76.86809
100.8215	82.86981	100.8362	70.74325	98.00251	76.86508
100.8625	82.87139	100.8711	70.74136	98.04447	76.86233
100.905	82.87389	100.9126	70.73836	98.08566	76.85987
100.9483	82.87614	100.9531	70.73655	98.11786	76.85784
100.9912	82.87434	100.9936	70.73702	98.16172	76.85515
101.0306	82.87351	101.0366	70.73793	98.20262	76.85262
101.0764	82.87659	101.0778	70.7364	98.24102	76.84995
101.1131	82.87629	101.1207	70.73526	98.28409	76.84704
101.1573	82.87637	101.1606	70.7343	98.32712	76.84426
101.1975	82.87368	101.2026	70.73315	98.36757	76.84178
101.2389	82.87182	101.2433	70.73502	98.41163	76.83983
101.2718	82.87056	101.2859	70.73616	98.45244	76.83723
101.3154	82.86373	101.321	70.73524	98.49384	76.83486
101.357	82.862	101.3627	70.73446	98.53619	76.83237
101.4	82.86293	101.4057	70.73207	98.578	76.82878
101.4409	82.86042	101.4469	70.72983	98.61863	76.82565
101.4811	82.85923	101.4901	70.73175	98.66168	76.82262
101.5235	82.85664	101.5304	70.73221	98.70245	76.819
101.5654	82.84964	101.5747	70.73241	98.73319	76.81704
101.6061	82.84549	101.6164	70.73272	98.78272	76.81406
101.6505	82.84272	101.6604	70.72972	98.82391	76.81195
101.6927	82.84485	101.7004	70.72821	98.86462	76.80996
101.7267	82.84624	101.7421	70.72743	98.90436	76.80767
101.7672	82.84263	101.7766	70.72545	98.94632	76.80517
101.8075	82.83941	101.819	70.7257	98.99032	76.80182
101.852	82.83705	101.864	70.72688	99.03159	76.79859
101.8937	82.83265	101.9037	70.72617	99.07297	76.79577
101.9374	82.83082	101.9463	70.72573	99.11524	76.79327
101.979	82.83071	101.9876	70.72357	99.15968	76.79051
102.0237	82.83119	102.0302	70.72072	99.2	76.78788
102.065	82.83136	102.0711	70.7198	99.24262	76.78492
102.1092	82.83366	102.1145	70.7198	99.27489	76.78291
102.15	82.8338	102.158	70.72148	99.3158	76.78025
102.1857	82.83239	102.1973	70.72374	99.36032	76.77786

102.2268	82.83266	102.2316	70.72429	99.40459	76.77551
102.2692	82.83113	102.2724	70.72322	99.44679	76.77277
102.3145	82.83233	102.3166	70.72126	99.48778	76.7702
102.3521	82.83299	102.3582	70.71904	99.5313	76.76696
102.3958	82.82912	102.3994	70.72033	99.57184	76.76444
102.4404	82.82486	102.4428	70.72062	99.61041	76.76198
102.48	82.81922	102.4847	70.72079	99.65682	76.75982
102.5254	82.81715	102.5274	70.72042	99.69756	76.75784
102.5637	82.82073	102.5664	70.71816	99.73805	76.75506
102.6054	82.81776	102.6089	70.71508	99.78282	76.75228
102.6505	82.81795	102.6513	70.71286	99.82726	76.7492
102.6897	82.81981	102.6833	70.71229	99.86634	76.7468
102.7251	82.81982	102.7262	70.71098	99.90195	76.74491
102.7688	82.8194	102.77	70.71068	99.94457	76.74275
102.8104	82.81643	102.812	70.71303	99.98804	76.74064
102.8523	82.81407	102.8545	70.71257	100.0286	76.73797
102.8934	82.8112	102.8946	70.71147	100.0671	76.73546
102.9339	82.81045	102.9375	70.71033	100.1126	76.73287
102.9773	82.80933	102.9793	70.70824	100.1554	76.7301
103.0209	82.80382	103.0199	70.70747	100.1968	76.72782
103.0633	82.80144	103.0652	70.7086	100.2396	76.72579
103.1038	82.80266	103.1037	70.70956	100.2839	76.72328
103.1467	82.80986	103.1373	70.70856	100.3252	76.72077
103.1798	82.819	103.1816	70.70922	100.3684	76.71797
103.22	82.81838	103.2257	70.70767	100.4121	76.71521
103.2622	82.81526	103.2637	70.70595	100.4523	76.71228
103.3061	82.81187	103.3034	70.70514	100.4958	76.70938
103.3474	82.80494	103.3462	70.70194	100.5326	76.70724
103.3872	82.80447	103.3909	70.69934	100.572	76.70451
103.4341	82.80902	103.433	70.69659	100.6139	76.70192
103.4741	82.81047	103.4716	70.69527	100.6559	76.6999
103.5147	82.80554	103.5135	70.69592	100.6974	76.69714
103.557	82.79725	103.5572	70.6974	100.7409	76.69454
103.5996	82.79237	103.5868	70.69944	100.7838	76.69234
103.6336	82.78886	103.6306	70.70012	100.8244	76.68945
103.6742	82.78284	103.6727	70.70037	100.8681	76.68685
103.7132	82.78196	103.7166	70.69958	100.9111	76.68395
103.7575	82.77904	103.7575	70.69794	100.9531	76.68124
103.8017	82.78027	103.7993	70.69845	100.9967	76.67881
103.8406	82.78847	103.8423	70.69924	101.0352	76.67647
103.8826	82.79451	103.8839	70.69922	101.0724	76.67437
103.9221	82.79345	103.9236	70.69929	101.1139	76.67151
103.964	82.79297	103.9666	70.69755	101.1576	76.66911
104.0087	82.78797	104.0103	70.69503	101.2004	76.66633
104.0494	82.78361	104.0404	70.69393	101.2429	76.66413
104.0847	82.78592	104.0822	70.69197	101.2846	76.66159
104.1259	82.78673	104.1282	70.69339	101.3271	76.65937
104.1699	82.78988	104.1681	70.69596	101.3717	76.65704
104.2127	82.79068	104.2125	70.69669	101.4113	76.65477
104.2516	82.78884	104.2502	70.69585	101.4536	76.65269
104.2923	82.78565	104.2938	70.69317	101.5003	76.65021
104.3327	82.78064	104.3379	70.6895	101.5389	76.64811
104.3747	82.7794	104.3774	70.68712	101.5834	76.64554
104.4163	82.77734	104.4221	70.68663	101.6253	76.64292
104.4592	82.77347	104.4625	70.68571	101.665	76.63995
104.503	82.76988	104.4969	70.68448	101.7004	76.638
104.5421	82.76976	104.5361	70.68479	101.7436	76.63523
104.5852	82.77213	104.5801	70.6853	101.7825	76.63253

104.6137	82.77297	104.6206	70.6838	101.8278	76.63014
104.6608	82.77209	104.6637	70.68315	101.8694	76.62766
104.7031	82.76829	104.7054	70.68073	101.9134	76.62551
104.7432	82.76293	104.7481	70.68082	101.9516	76.62328
104.788	82.76041	104.7881	70.68152	101.9914	76.62139
104.8298	82.76043	104.8307	70.68114	102.0336	76.61886
104.874	82.76243	104.8723	70.68167	102.0772	76.61703
104.9136	82.76527	104.9134	70.68119	102.1198	76.61532
104.9548	82.76482	104.9505	70.67999	102.1642	76.61288
104.9988	82.76478	104.9898	70.67955	102.2053	76.61068
105.043	82.76259	105.0348	70.68012	102.2505	76.60811
105.0748	82.76097	105.0717	70.67843	102.2919	76.605
105.1184	82.7568	105.115	70.67962	102.3205	76.60271
105.1602	82.75094	105.1594	70.67972	102.3631	76.6005
105.2033	82.74689	105.1994	70.67939	102.4038	76.59734
105.2438	82.74719	105.2402	70.67907	102.4491	76.59495
105.2831	82.7478	105.2854	70.6792	102.4919	76.59288
105.3268	82.74733	105.3278	70.6787	102.5314	76.5904
105.3699	82.74815	105.3658	70.67792	102.5739	76.5883
105.4127	82.74222	105.3995	70.67741	102.6175	76.58617
105.4544	82.73901	105.4436	70.6766	102.6585	76.5836
105.4976	82.73844	105.4856	70.68	102.7014	76.58125
105.5294	82.73943	105.5242	70.68064	102.7442	76.57898
105.5722	82.74105	105.566	70.68079	102.7846	76.57573
105.6141	82.73498	105.6095	70.68006	102.824	76.57315
105.6586	82.73175	105.6527	70.67565	102.8592	76.57107
105.6978	82.72869	105.6925	70.67414	102.9005	76.56882
105.7412	82.72879	105.7338	70.67367	102.9446	76.56674
105.7832	82.73327	105.7758	70.67159	102.9847	76.56473
105.8249	82.73886	105.8185	70.66952	103.0253	76.56214
105.8666	82.74115	105.8535	70.66988	103.0707	76.55941
105.9093	82.73435	105.8956	70.6693	103.1118	76.55682
105.9514	82.72855	105.9344	70.66778	103.1509	76.55409
105.9867	82.72443	105.9788	70.66764	103.1953	76.5513
106.027	82.72233	106.0237	70.66625	103.2362	76.54837
106.0686	82.7219	106.0614	70.66603	103.2792	76.54585
106.1081	82.72339	106.1033	70.66681	103.3182	76.54367
106.1511	82.72281	106.144	70.66569	103.3576	76.54172
106.1926	82.71598	106.1875	70.66413	103.3986	76.53945
106.2357	82.7142	106.2279	70.66373	103.4406	76.53721
106.2783	82.71321	106.2712	70.66199	103.4722	76.53529
106.322	82.71339	106.3055	70.66148	103.5135	76.53264
106.3621	82.71598	106.3485	70.66216	103.5549	76.53068
106.4027	82.71478	106.3863	70.66124	103.5959	76.52806
106.4448	82.71728	106.4306	70.66112	103.6383	76.52573
106.4852	82.71979	106.4697	70.66246	103.6791	76.52328
106.518	82.71902	106.5117	70.66298	103.7196	76.52083
106.5621	82.71578	106.5528	70.66322	103.7634	76.51824
106.6029	82.70745	106.596	70.66359	103.8072	76.51507
106.6452	82.69952	106.6428	70.66177	103.8447	76.51232
106.6882	82.69284	106.6817	70.66134	103.8864	76.50916
106.7281	82.69176	106.721	70.66114	103.9252	76.50705
106.7708	82.68903	106.7568	70.65938	103.965	76.50514
106.8128	82.68932	106.7993	70.65976	104.0083	76.50314
106.8547	82.68926	106.8414	70.65996	104.0526	76.50098
106.8988	82.68871	106.8808	70.6583	104.0821	76.49934
106.9404	82.69313	106.9233	70.65797	104.1234	76.49694
106.972	82.69311	106.9643	70.65761	104.165	76.49432

107.0163	82.6935	107.009	70.65565	104.2071	76.49176
107.0622	82.69462	107.0475	70.65415	104.2446	76.48912
107.1035	82.69387	107.0906	70.65369	104.29	76.4864
107.1424	82.6933	107.1318	70.65239	104.332	76.48381
107.184	82.68978	107.1761	70.65187	104.3706	76.48134
107.2282	82.6882	107.2089	70.65144	104.4132	76.47878
107.2696	82.69033	107.2484	70.64876	104.4562	76.47638
107.3121	82.69473	107.288	70.64853	104.4994	76.47398
107.3549	82.69902	107.329	70.64938	104.5407	76.47253
107.398	82.69805	107.375	70.64926	104.5772	76.47049
107.4282	82.69857	107.4173	70.65219	104.6115	76.46898
107.4716	82.69422	107.4572	70.65151	104.6559	76.46676
107.5145	82.68784	107.4978	70.64977	104.695	76.46408
107.555	82.68575	107.5426	70.64904	104.7393	76.46147
107.5963	82.68626	107.5841	70.64586	104.7789	76.45907
107.6403	82.68717	107.6234	70.64296	104.819	76.45682
107.6837	82.68681	107.6618	70.64308	104.8627	76.45476
107.7277	82.68607	107.704	70.64223	104.901	76.45265
107.7713	82.68484	107.7443	70.64065	104.9462	76.45034
107.8115	82.68225	107.7864	70.64161	104.9846	76.44826
107.8512	82.67783	107.8274	70.63893	105.0234	76.44566
107.8861	82.67964	107.8684	70.63689	105.0669	76.44359
107.9266	82.68097	107.9127	70.63741	105.1075	76.44096
107.9707	82.68141	107.9543	70.63686	105.1509	76.43906
108.0128	82.68175	107.9944	70.63725	105.1917	76.43731
108.0532	82.67726	108.0366	70.63761	105.2258	76.4354
108.094	82.67281	108.0788	70.63689	105.2693	76.43328
108.1425	82.66796	108.1136	70.63621	105.3134	76.43114
108.1824	82.665	108.1542	70.63551	105.3542	76.4289
108.2262	82.65977	108.198	70.63452	105.3936	76.42672
108.2654	82.65622	108.2352	70.63398	105.4343	76.42492
108.3086	82.6544	108.2838	70.63448	105.4768	76.42219
108.348	82.65233	108.3254	70.63426	105.5214	76.4197
108.392	82.65582	108.3654	70.63575	105.5612	76.41742
108.4244	82.65514	108.4061	70.63694	105.6041	76.41495
108.4645	82.65013	108.4506	70.63544	105.6473	76.41281
108.5052	82.64588	108.4907	70.63418	105.6861	76.41033
108.5502	82.64248	108.535	70.63092	105.7262	76.40772
108.595	82.64338	108.5673	70.62831	105.7714	76.40543
108.6363	82.64513	108.6117	70.62936	105.8142	76.40344
108.6773	82.64194	108.6532	70.63017	105.8469	76.40158
108.7187	82.63888	108.6966	70.63001	105.8911	76.39995
108.759	82.63796	108.7365	70.62917	105.9336	76.39761
108.8034	82.63444	108.7783	70.62791	105.974	76.39546
108.8435	82.63905	108.8185	70.62649	106.0133	76.39303
108.8777	82.64566	108.8624	70.62621	106.0556	76.39041
108.9187	82.64718	108.9063	70.62682	106.0979	76.38783
108.9631	82.64803	108.9491	70.62639	106.1416	76.3858
109.0036	82.6459	108.9894	70.62794	106.1832	76.38362
109.0481	82.64278	109.0215	70.62768	106.2244	76.38101
109.0902	82.6368	109.0635	70.62567	106.2672	76.3793
109.1273	82.62924	109.1041	70.62574	106.3112	76.37649
109.171	82.6298	109.1478	70.62265	106.3546	76.37438
109.2133	82.63257	109.1876	70.62098	106.3854	76.3729
109.2538	82.63512	109.2301	70.62325	106.4301	76.37122
109.2956	82.63743	109.2703	70.62149	106.4701	76.36913
109.3308	82.63764	109.314	70.62132	106.5132	76.36732
109.37	82.63752	109.3572	70.62117	106.5549	76.36485

109.4154	82.63077	109.3975	70.61876	106.5984	76.36184
109.4578	82.62396	109.4406	70.61902	106.6439	76.35906
109.5015	82.61661	109.4763	70.61836	106.6856	76.35612
109.5414	82.60707	109.5193	70.61655	106.7293	76.35394
109.5824	82.60175	109.5572	70.6159	106.7717	76.35171
109.6265	82.60209	109.6038	70.61401	106.8149	76.34971
109.666	82.60634	109.6432	70.61291	106.8598	76.34775
109.7074	82.61303	109.684	70.61372	106.9012	76.34595
109.7512	82.61706	109.7269	70.61413	106.9443	76.34408
109.7821	82.61685	109.7676	70.61396	106.9825	76.34229
109.826	82.61615	109.8096	70.61464	107.0151	76.3408
109.8688	82.61114	109.8496	70.6143	107.0621	76.33868
109.9086	82.61003	109.8888	70.61329	107.1035	76.33667
109.9482	82.61644	109.9227	70.61442	107.1482	76.33458
109.9913	82.6201	109.9683	70.61348	107.1871	76.33239
110.0326	82.62186	110.0068	70.6136	107.2286	76.32998
110.0752	82.62368	110.051	70.61499	107.2755	76.32763
110.1168	82.6198	110.0907	70.61498	107.3173	76.32537
110.1605	82.61398	110.1325	70.61401	107.3592	76.32292
110.2025	82.61381	110.1748	70.61254	107.4002	76.32098
110.2401	82.60999	110.2205	70.61036	107.4414	76.31891
110.2819	82.60418	110.2662	70.6075	107.4835	76.31688
110.3194	82.60206	110.3037	70.60774	107.5284	76.3149
110.3586	82.59605	110.3436	70.6067	107.5708	76.31311
110.4016	82.58974	110.3799	70.60503	107.6142	76.31099
110.4417	82.5898	110.4207	70.60375	107.645	76.30916
110.4852	82.58942	110.4649	70.60261	107.6894	76.30753
110.5243	82.5901	110.5066	70.6023	107.7286	76.30569
110.5681	82.58879	110.5497	70.60358	107.7716	76.30401
110.6097	82.59003	110.5912	70.60307	107.8161	76.30233
110.6528	82.59141	110.632	70.60216	107.8575	76.30077
110.6957	82.58995	110.6752	70.60271	107.8986	76.29828
110.7371	82.59073	110.7184	70.60133	107.9434	76.29525
110.771	82.59179	110.759	70.6021	107.9894	76.2932
110.8139	82.59479	110.8016	70.60479	108.0272	76.29109
110.8555	82.59671	110.8353	70.60237	108.0719	76.28954
110.8953	82.59257	110.8787	70.6023	108.1134	76.28817
110.9394	82.5885	110.9232	70.6031	108.1587	76.28597
110.98	82.58312	110.9643	70.60058	108.1904	76.28406
111.0222	82.57574	111.0033	70.60053	108.2357	76.28107
111.0655	82.57519	111.0454	70.59926	108.2786	76.27898
111.1062	82.5769	111.0898	70.59638	108.3221	76.27699
111.1451	82.57719	111.1309	70.59349	108.3622	76.27489
111.1907	82.57807	111.1691	70.59169	108.4027	76.27315
111.2204	82.57811	111.2148	70.591	108.45	76.27094
111.2633	82.57645	111.2534	70.59203	108.488	76.26865
111.3068	82.57219	111.2866	70.59232	108.5295	76.26666
111.3486	82.56943	111.3314	70.59106	108.5722	76.26458
111.3888	82.5739	111.372	70.59145	108.6136	76.26221
111.4316	82.57137	111.4148	70.59114	108.6585	76.26007
111.4753	82.56984	111.4547	70.59001	108.7005	76.25776
111.519	82.57188	111.4966	70.59244	108.7432	76.25554
111.5575	82.57261	111.5383	70.59069	108.7843	76.25399
111.6005	82.57182	111.5821	70.58845	108.8192	76.25257
111.6453	82.56832	111.6238	70.58965	108.8615	76.25041
111.6741	82.56839	111.666	70.58742	108.9024	76.24864
111.7174	82.56077	111.7077	70.58726	108.9464	76.24675
111.7601	82.55475	111.7408	70.58857	108.9878	76.24482

111.8024	82.55357	111.7802	70.58746	109.03	76.24278
111.8428	82.55246	111.8225	70.58578	109.0723	76.24027
111.884	82.55401	111.8638	70.58581	109.1205	76.23749
111.9299	82.55486	111.9044	70.5835	109.1598	76.23502
111.9732	82.55004	111.9482	70.58266	109.2004	76.23316
112.0122	82.5411	111.9911	70.58282	109.2438	76.23164
112.0514	82.53877	112.0332	70.58165	109.2852	76.2303
112.0936	82.53652	112.0749	70.5816	109.3284	76.22838
112.1336	82.54122	112.1166	70.5816	109.3737	76.2262
112.1782	82.54749	112.158	70.58281	109.4142	76.22399
112.2122	82.54675	112.1905	70.58333	109.452	76.22259
112.2522	82.54431	112.232	70.58317	109.4902	76.22077
112.2955	82.54536	112.2764	70.58159	109.534	76.21862
112.3394	82.54012	112.317	70.57969	109.5724	76.21626
112.3785	82.53501	112.3608	70.57816	109.6166	76.21406
112.4203	82.53136	112.4012	70.57786	109.6594	76.21196
112.4648	82.52588	112.4418	70.57989	109.6974	76.21044
112.5052	82.52325	112.484	70.57906	109.7413	76.20894
112.5495	82.52351	112.526	70.5777	109.7828	76.20707
112.5909	82.52626	112.5686	70.57711	109.8269	76.20514
112.6312	82.52586	112.6092	70.57712	109.8667	76.20268
112.6659	82.52772	112.6432	70.57736	109.9087	76.20016
112.708	82.5321	112.6821	70.57894	109.9488	76.19774
112.7503	82.53323	112.7266	70.58114	109.9842	76.19599
112.7891	82.52859	112.7674	70.57809	110.0264	76.194
112.8332	82.52841	112.8077	70.57665	110.0646	76.1925
112.8762	82.52563	112.8531	70.5755	110.1066	76.19022
112.9188	82.5208	112.8935	70.57263	110.149	76.18837
112.9584	82.5216	112.9354	70.57126	110.1909	76.1869
113	82.52148	112.9766	70.57049	110.2317	76.18531
113.0401	82.51544	113.0202	70.56997	110.2711	76.18414
113.0817	82.51061	113.0598	70.57137	110.3101	76.18202
113.1139	82.50999	113.0947	70.57166	110.3584	76.17974
113.1565	82.51091	113.1345	70.57063	110.4013	76.1775
113.1983	82.51667	113.1772	70.56887	110.4395	76.17568
113.2432	82.51726	113.2164	70.56623	110.4826	76.17367
113.2849	82.51785	113.2589	70.56428	110.5242	76.17127
113.3248	82.51534	113.3008	70.5629	110.5623	76.16931
113.3713	82.50727	113.3428	70.56193	110.5963	76.16724
113.4134	82.50189	113.3836	70.56093	110.6372	76.16536
113.4581	82.50388	113.4292	70.56105	110.6846	76.16365
113.4998	82.50252	113.4681	70.56124	110.7209	76.16154
113.5416	82.49883	113.5082	70.56256	110.7649	76.15928
113.5742	82.50065	113.5451	70.56319	110.806	76.15678
113.6167	82.50014	113.585	70.56228	110.846	76.15438
113.661	82.49814	113.6307	70.56301	110.8878	76.1524
113.7022	82.50102	113.671	70.56136	110.9281	76.15034
113.745	82.50317	113.7094	70.55916	110.969	76.1486
113.7858	82.5033	113.7529	70.55945	111.0102	76.14723
113.8282	82.50205	113.7961	70.55725	111.0543	76.14493
113.8723	82.49956	113.8356	70.55713	111.0928	76.14284
113.9122	82.49832	113.8768	70.55868	111.1364	76.1408
113.956	82.49789	113.9217	70.55824	111.1732	76.13867
113.9987	82.4911	113.9635	70.55957	111.2048	76.13704
114.0416	82.48778	113.9942	70.56105	111.2509	76.13507
114.0854	82.48601	114.0356	70.56101	111.2896	76.13345
114.1192	82.48209	114.0783	70.55969	111.3364	76.13122
114.1638	82.48059	114.1232	70.56035	111.3776	76.12946

114.2064	82.48036	114.1646	70.55782	111.4184	76.12788
114.2455	82.47854	114.2074	70.55605	111.4626	76.12588
114.2854	82.47855	114.2495	70.55734	111.505	76.12382
114.3251	82.47696	114.292	70.55644	111.5436	76.12159
114.3692	82.47609	114.3334	70.5565	111.5852	76.11933
114.4136	82.47406	114.3756	70.55574	111.6278	76.11737
114.4528	82.47209	114.4121	70.55446	111.6698	76.11573
114.4955	82.47376	114.449	70.5535	111.7122	76.11413
114.5379	82.47661	114.4945	70.55144	111.7464	76.11252
114.5707	82.47835	114.5366	70.54982	111.7874	76.11055
114.6118	82.47742	114.5783	70.54813	111.8269	76.1091
114.6522	82.47492	114.6214	70.54613	111.8679	76.10745
114.6955	82.47174	114.6641	70.5457	111.9118	76.10575
114.7377	82.46893	114.7028	70.54689	111.9555	76.10414
114.781	82.47074	114.7462	70.54856	111.995	76.10145
114.8226	82.47093	114.7853	70.54698	112.0354	76.09892
114.8648	82.46954	114.8271	70.54613	112.0783	76.0973
114.9064	82.47181	114.8698	70.5431	112.1187	76.0953
114.9484	82.47113	114.9052	70.53895	112.1606	76.09307
114.9889	82.47079	114.9454	70.53815	112.2042	76.09157
115.0244	82.46842	114.9911	70.53708	112.2446	76.0893
115.0679	82.46309	115.0283	70.53665	112.2871	76.08761
115.1096	82.46204	115.0686	70.54013	112.3309	76.08617
115.1519	82.45976	115.1098	70.54208	112.364	76.08466
115.1916	82.4611	115.1523	70.54415	112.4046	76.0831
115.2324	82.46544	115.1931	70.54401	112.4489	76.08099
115.2775	82.46312	115.2352	70.54219	112.4855	76.07929
115.3173	82.46111	115.2813	70.53963	112.5252	76.07746
115.3564	82.46006	115.3196	70.53875	112.568	76.07565
115.4002	82.45759	115.3533	70.5388	112.6104	76.07312
115.443	82.45448	115.3932	70.53742	112.653	76.07138
115.4764	82.44968	115.4332	70.53731	112.6924	76.06935
115.5197	82.44978	115.4786	70.53647	112.7356	76.06751
115.5596	82.45012	115.5179	70.53541	112.7778	76.06595
115.6016	82.44428	115.5616	70.53621	112.8231	76.0633
115.6442	82.44122	115.605	70.53675	112.8639	76.06122
115.6836	82.43789	115.643	70.53587	112.9058	76.05891
115.7272	82.43431	115.6825	70.53683	112.9468	76.0573
115.768	82.43316	115.7287	70.53626	112.9816	76.05645
115.808	82.43433	115.7696	70.53664	113.025	76.055
115.8531	82.43823	115.803	70.53943	113.0633	76.05321
115.8929	82.43771	115.8469	70.53888	113.1085	76.05089
115.9372	82.43182	115.8873	70.5385	113.1496	76.04841
115.979	82.43061	115.9299	70.53727	113.1895	76.04657
116.0111	82.42609	115.9741	70.53505	113.2321	76.04459
116.0507	82.42048	116.0159	70.53423	113.2742	76.04322
116.0951	82.42268	116.0562	70.53262	113.3162	76.04146
116.136	82.42438	116.1005	70.53112	113.3582	76.0401
116.1806	82.42403	116.1428	70.52901	113.4001	76.03876
116.2217	82.42396	116.181	70.5273	113.4416	76.03698
116.2658	82.42074	116.2241	70.52675	113.4833	76.03524
116.3084	82.4149	116.2556	70.52713	113.5176	76.03395
116.3522	82.4112	116.3007	70.52569	113.5591	76.03202
116.3899	82.40709	116.346	70.52618	113.6011	76.03042
116.4318	82.40448	116.3906	70.526	113.6435	76.02928
116.465	82.40438	116.43	70.52527	113.683	76.02715
116.5065	82.40438	116.4691	70.52508	113.7274	76.02568
116.5475	82.40046	116.5125	70.5255	113.7692	76.02383

116.5892	82.4011	116.554	70.52614	113.8111	76.02195
116.6262	82.40023	116.5977	70.52554	113.8515	76.01986
116.6718	82.39859	116.6383	70.52511	113.8948	76.01755
116.7133	82.40181	116.6825	70.5234	113.9388	76.01546
116.7543	82.40146	116.7144	70.52226	113.9767	76.01361
116.7972	82.40286	116.7551	70.52145	114.0201	76.01228
116.839	82.40144	116.7977	70.52033	114.0629	76.01057
116.8804	82.39394	116.8427	70.51922	114.1077	76.00911
116.9146	82.39151	116.8867	70.51756	114.1422	76.00774
116.9554	82.38808	116.9279	70.51633	114.181	76.00561
116.9973	82.3834	116.9676	70.51628	114.225	76.00379
117.0405	82.38659	117.0085	70.51513	114.2646	76.00222
117.0843	82.39064	117.0513	70.51607	114.3099	76.00022
117.127	82.39404	117.09	70.51697	114.352	75.99879
117.1693	82.39491	117.1344	70.51508	114.3932	75.99685
117.2108	82.39263	117.1692	70.51572	114.4346	75.99486
117.2523	82.38731	117.2115	70.51692	114.4758	75.99297
117.2936	82.38199	117.2528	70.51564	114.5182	75.99075
117.3323	82.37818	117.292	70.51648	114.5592	75.98885
117.3674	82.37675	117.3353	70.51658	114.6041	75.98612
117.409	82.37713	117.3768	70.51339	114.6448	75.98368
117.4491	82.37742	117.4211	70.51187	114.6869	75.98179
117.494	82.37753	117.4612	70.51198	114.7265	75.98021
117.5332	82.3741	117.5019	70.51184	114.7624	75.97919
117.5742	82.37165	117.5456	70.51188	114.8052	75.97784
117.619	82.37037	117.5896	70.51238	114.8486	75.97596
117.6594	82.36676	117.6201	70.5129	114.8915	75.97411
117.6996	82.36669	117.6618	70.51333	114.9338	75.97245
117.7426	82.36402	117.7032	70.51105	114.9734	75.97086
117.7842	82.36273	117.7422	70.50799	115.0156	75.96947
117.8218	82.36673	117.7873	70.50443	115.0605	75.96755
117.8667	82.36613	117.8273	70.50111	115.1024	75.96536
117.8994	82.36792	117.8695	70.50204	115.1445	75.96364
117.9426	82.36914	117.9133	70.5045	115.1888	75.9622
117.9858	82.36752	117.9541	70.50443	115.2304	75.96084
118.0268	82.36867	117.9937	70.50477	115.2706	75.95973
118.0718	82.3712	118.0392	70.50608	115.3032	75.95839
118.1108	82.37166	118.0729	70.50434	115.345	75.95629
118.153	82.36623	118.1128	70.50293	115.3868	75.95481
118.1954	82.36078	118.1574	70.50189	115.4324	75.95317
118.2355	82.35929	118.1975	70.49931	115.472	75.9518
118.2782	82.35505	118.2426	70.49824	115.5148	75.94987
118.3233	82.35138	118.2822	70.49892	115.5604	75.9479
118.3519	82.34851	118.3243	70.49772	115.6004	75.94617
118.3939	82.34429	118.3609	70.49763	115.6412	75.94433
118.4351	82.34129	118.4034	70.49818	115.6858	75.94244
118.4773	82.33794	118.4465	70.49748	115.7246	75.94042
118.5199	82.34182	118.488	70.49899	115.7697	75.93842
118.5657	82.34065	118.5206	70.49869	115.8088	75.93657
118.6024	82.33609	118.5619	70.49791	115.8516	75.93518
118.6478	82.33989	118.6011	70.49719	115.8946	75.93382
118.6853	82.34288	118.6484	70.49717	115.9287	75.93239
118.7298	82.33925	118.6904	70.49719	115.9705	75.93087
118.771	82.33582	118.7319	70.49725	116.0141	75.92952
118.8058	82.33801	118.7733	70.49817	116.0529	75.92754
118.8475	82.33562	118.8161	70.49619	116.0991	75.92553
118.8885	82.33527	118.8582	70.49603	116.1398	75.92337
118.9286	82.33462	118.8983	70.49404	116.1804	75.92112

118.9728	82.3287	118.9408	70.49242	116.2232	75.91965
119.0144	82.32647	118.9756	70.49496	116.2668	75.91827
119.057	82.32319	119.0133	70.49361	116.3105	75.91724
119.0987	82.32098	119.0564	70.49225	116.3518	75.91613
119.1406	82.32095	119.098	70.4925	116.3939	75.91457
119.1819	82.31873	119.1421	70.48988	116.4348	75.91268
119.2233	82.31358	119.1824	70.48753	116.4795	75.91057
119.2591	82.31208	119.2261	70.48653	116.5226	75.90832
119.2981	82.31324	119.2673	70.4856	116.5524	75.90669
119.3418	82.30949	119.3091	70.48805	116.5967	75.90542
119.3833	82.30744	119.3494	70.48925	116.6393	75.9038
119.425	82.31225	119.3897	70.49046	116.6804	75.90217
119.4677	82.31024	119.4242	70.4908	116.7186	75.90029
119.5106	82.30543	119.469	70.48969	116.7594	75.89866
119.5535	82.30147	119.5083	70.48767	116.8026	75.89697
119.5954	82.29478	119.5518	70.48632	116.8429	75.89517
119.635	82.28903	119.5886	70.48463	116.8844	75.89354
119.6769	82.28732	119.6364	70.48158	116.9288	75.89168
119.7193	82.2869	119.676	70.48224	116.9686	75.88988
119.7601	82.28778	119.7172	70.48131	117.0108	75.88841
119.7926	82.28704	119.7575	70.48157	117.0534	75.88652
119.8338	82.28341	119.7998	70.48293	117.0867	75.88442
119.8757	82.28063	119.8427	70.484	117.1296	75.8821
119.9192	82.2785	119.8759	70.48741	117.1669	75.88005
119.9662	82.27748	119.9215	70.48845	117.2119	75.87846
120.0056	82.27791	119.9601	70.48648	117.2539	75.87702
120.0459	82.28215	120.0012	70.48376	117.2956	75.87616
120.0894	82.2801	120.0449	70.48149	117.3369	75.87452
120.1311	82.27726	120.0867	70.48175	117.377	75.87267
120.1708	82.27577	120.129	70.48134	117.4181	75.87074
120.2133	82.27444	120.1688	70.48187	117.4625	75.86934
120.2479	82.27479	120.2126	70.48028	117.5052	75.86822
120.2879	82.27515	120.2519	70.47773	117.5432	75.86697
120.3305	82.27605	120.2956	70.4785	117.5851	75.86626
120.3708	82.27504	120.3248	70.47757	117.6287	75.86405
120.4146	82.27163	120.3692	70.47706	117.667	75.86148
120.4545	82.26737	120.4112	70.47689	117.703	75.86
120.4963	82.26324	120.4535	70.47405	117.7444	75.85764
120.5391	82.25788	120.4934	70.47191	117.7863	75.85567
120.5832	82.25534	120.5363	70.47117	117.8287	75.85407
120.6251	82.25487	120.5808	70.46943	117.8708	75.85241
120.6692	82.25395	120.6222	70.47056	117.9123	75.85093
120.7037	82.25249	120.6665	70.47122	117.954	75.84972
120.7421	82.25246	120.7043	70.47012	117.9927	75.84878
120.7842	82.2558	120.7462	70.47101	118.0384	75.84785
120.8252	82.25583	120.7759	70.46984	118.0827	75.84641
120.8644	82.25628	120.8206	70.47018	118.1198	75.84416
120.91	82.25805	120.8601	70.47096	118.1644	75.84218
120.9522	82.25398	120.9046	70.47006	118.2069	75.84019
120.994	82.25078	120.9441	70.46921	118.2466	75.83836
121.0347	82.25145	120.9898	70.46738	118.2876	75.8363
121.0782	82.24854	121.0306	70.46647	118.3226	75.83491
121.1205	82.24463	121.0737	70.46623	118.3636	75.83313
121.1563	82.24295	121.1144	70.46754	118.4083	75.83128
121.1978	82.24082	121.1536	70.46957	118.4475	75.83033
121.2402	82.2398	121.1976	70.47029	118.4876	75.82895
121.2816	82.2413	121.233	70.46947	118.5286	75.82711
121.3239	82.24168	121.2739	70.46779	118.5702	75.82553

121.3672	82.23969	121.3181	70.46388	118.6128	75.82421
121.4049	82.2358	121.3578	70.46209	118.6549	75.82249
121.4464	82.23122	121.3981	70.46156	118.6986	75.82117
121.4918	82.22788	121.4416	70.46106	118.7397	75.81947
121.5342	82.22471	121.4835	70.46105	118.7816	75.81742
121.5738	82.22172	121.5236	70.45899	118.8195	75.81608
121.6149	82.21876	121.5639	70.45773	118.8552	75.81463
121.6572	82.21507	121.6074	70.4581	118.8939	75.81339
121.691	82.2159	121.6498	70.45836	118.9386	75.81211
121.7313	82.21431	121.6858	70.45909	118.9782	75.81062
121.776	82.21333	121.7269	70.46166	119.021	75.80926
121.8168	82.21755	121.7673	70.46137	119.0614	75.80785
121.8594	82.2198	121.8089	70.45986	119.1047	75.80664
121.8982	82.21949	121.849	70.45781	119.145	75.80435
121.9416	82.21865	121.8932	70.45353	119.1882	75.80277
121.9878	82.21926	121.9376	70.45042	119.2307	75.80098
122.0298	82.22034	121.9769	70.44963	119.272	75.79924
122.0677	82.21779	122.0195	70.44938	119.3138	75.79793
122.1107	82.21612	122.0611	70.44919	119.3534	75.79595
122.1444	82.21383	122.103	70.45022	119.3945	75.79442
122.1868	82.20647	122.1379	70.44965	119.4382	75.79277
122.2305	82.20496	122.1793	70.45071	119.471	75.79145
122.2674	82.20591	122.2255	70.45217	119.5108	75.78964
122.3125	82.20506	122.264	70.45108	119.5557	75.78773
122.354	82.20632	122.3064	70.45051	119.5972	75.78576
122.3961	82.20466	122.3457	70.44788	119.638	75.78413
122.4393	82.19978	122.3893	70.44541	119.6781	75.78266
122.4804	82.19863	122.4313	70.44619	119.7213	75.78087
122.5206	82.20126	122.4731	70.44592	119.7619	75.77895
122.5691	82.20205	122.5139	70.44482	119.8048	75.77688
122.5988	82.20321	122.5577	70.44409	119.8445	75.77545
122.6412	82.19965	122.589	70.44172	119.8877	75.77465
122.6802	82.19189	122.631	70.44253	119.9306	75.77452
122.724	82.18499	122.6732	70.44448	119.9704	75.77396
122.7657	82.18028	122.7145	70.44303	120.0133	75.77299
122.8088	82.18	122.7594	70.44288	120.0568	75.77188
122.8514	82.18048	122.7986	70.44106	120.0892	75.76981
122.8906	82.17808	122.84	70.43749	120.1299	75.7676
122.9354	82.17815	122.8821	70.4373	120.173	75.76518
122.9752	82.18006	122.9231	70.4367	120.2152	75.76293
123.0132	82.17837	122.9659	70.43493	120.2529	75.76136
123.0488	82.17853	123.0068	70.43716	120.298	75.76027
123.0939	82.18157	123.0401	70.43733	120.3404	75.75958
123.134	82.18178	123.0834	70.43591	120.3797	75.75794
123.1769	82.17761	123.1232	70.43579	120.4203	75.75626
123.2201	82.17709	123.1667	70.43331	120.466	75.75458
123.2625	82.17679	123.2072	70.4316	120.5039	75.7533
123.3008	82.17461	123.2498	70.43431	120.5477	75.75211
123.3434	82.17203	123.2952	70.43515	120.5942	75.75109
123.3842	82.16971	123.3336	70.43537	120.6229	75.75009
123.425	82.16439	123.3736	70.43588	120.6685	75.74808
123.4701	82.15492	123.4181	70.4332	120.7116	75.74652
123.5101	82.15026	123.458	70.43271	120.7499	75.74438
123.5565	82.14616	123.4946	70.43352	120.7912	75.74262
123.5868	82.14357	123.5356	70.43341	120.8364	75.7411
123.6274	82.14333	123.5782	70.43309	120.8748	75.73976
123.6673	82.14419	123.6192	70.4343	120.9166	75.73868
123.7096	82.14815	123.6626	70.43249	120.9574	75.73739

123.7548	82.15086	123.7029	70.43145	121.0004	75.73643
123.7984	82.14919	123.7472	70.43057	121.0431	75.73507
123.8416	82.14842	123.7886	70.42617	121.0852	75.7336
123.8819	82.14457	123.8331	70.42595	121.1255	75.73175
123.9247	82.13703	123.8703	70.42757	121.1709	75.72944
123.9639	82.13414	123.9126	70.42828	121.2108	75.72736
124.0075	82.13361	123.9439	70.42955	121.2457	75.72608
124.0376	82.13282	123.987	70.4322	121.2878	75.72507
124.0805	82.13228	124.0288	70.42966	121.3295	75.72384
124.1191	82.13268	124.0711	70.4289	121.3731	75.72249
124.1676	82.1314	124.1149	70.4276	121.4128	75.72106
124.2108	82.12825	124.1552	70.42375	121.4547	75.71969
124.2513	82.12619	124.1952	70.42604	121.4979	75.71786
124.2897	82.123	124.2382	70.42581	121.5398	75.71627
124.3326	82.1184	124.2776	70.42571	121.5797	75.7153
124.377	82.11832	124.3205	70.426	121.6212	75.71363
124.4154	82.11804	124.3641	70.42355	121.6676	75.71193
124.4565	82.11897	124.392	70.42199	121.7064	75.70988
124.4911	82.12049	124.4381	70.42377	121.7487	75.70707
124.5343	82.1162	124.4786	70.42464	121.7908	75.70509
124.5769	82.11132	124.5198	70.42527	121.8326	75.70402
124.6177	82.10841	124.5649	70.42571	121.8669	75.70351
124.6614	82.10921	124.609	70.4231	121.912	75.70287
124.7029	82.11103	124.6497	70.4211	121.9518	75.70151
124.7451	82.11053	124.6916	70.41942	121.9944	75.70041
124.788	82.11357	124.7342	70.41794	122.0364	75.6989
124.828	82.11114	124.7728	70.41883	122.0807	75.69746
124.8722	82.10538	124.8164	70.41952	122.1197	75.69593
124.9119	82.10742	124.8508	70.41836	122.1626	75.6942
124.947	82.10749	124.8927	70.41801	122.2039	75.69301
124.9881	82.10689	124.9327	70.41857	122.2481	75.69147
125.0314	82.10644	124.9756	70.41798	122.2912	75.69019
125.0737	82.10536	125.0175	70.41804	122.328	75.6888
125.1134	82.10012	125.058	70.41983	122.3741	75.68675
125.156	82.09624	125.1042	70.41948	122.406	75.68568
125.1956	82.09757	125.1453	70.41863	122.4454	75.6841
125.2403	82.09623	125.1883	70.41875	122.4854	75.68329
125.284	82.09308	125.2282	70.41603	122.5304	75.68162
125.3245	82.08864	125.2698	70.4131	122.5716	75.68029
125.3663	82.08597	125.3032	70.41098	122.6148	75.67844
125.4077	82.08382	125.3451	70.40881	122.6568	75.67628
125.447	82.08708	125.3884	70.41089	122.6983	75.67468
125.4843	82.09276	125.429	70.41324	122.744	75.67294
125.5267	82.09377	125.4711	70.41355	122.784	75.67167
125.5674	82.09138	125.5134	70.41473	122.8249	75.6696
125.6092	82.08783	125.5546	70.41246	122.8657	75.66849
125.6522	82.07985	125.5941	70.4091	122.9108	75.667
125.6938	82.07277	125.6393	70.40696	122.9496	75.66545
125.7341	82.07035	125.6781	70.40402	122.9943	75.66387
125.7766	82.07052	125.7201	70.40223	123.0278	75.66228
125.817	82.06974	125.7513	70.40276	123.0696	75.6604
125.8609	82.06734	125.7953	70.40404	123.1115	75.65853
125.9004	82.06586	125.8368	70.40507	123.1544	75.6581
125.9373	82.06305	125.8788	70.40816	123.1956	75.65669
125.9819	82.06091	125.919	70.40793	123.2388	75.65547
126.0199	82.05986	125.9615	70.40784	123.2788	75.65412
126.0615	82.0605	126.0039	70.40775	123.3246	75.65316
126.1037	82.05683	126.0466	70.40395	123.3662	75.65165

126.1453	82.0507	126.0904	70.40162	123.4091	75.65003
126.186	82.04987	126.1301	70.40204	123.4512	75.64872
126.2292	82.04767	126.1727	70.4022	123.4934	75.64627
126.2712	82.04599	126.2075	70.40344	123.5337	75.6447
126.3127	82.04784	126.249	70.40429	123.5758	75.64349
126.3559	82.05257	126.2911	70.40246	123.6177	75.64238
126.3912	82.05157	126.3314	70.40005	123.653	75.64131
126.4323	82.0481	126.3722	70.39794	123.6941	75.63994
126.4731	82.04875	126.4138	70.39777	123.7364	75.6386
126.517	82.04566	126.4548	70.3978	123.7773	75.63718
126.557	82.04431	126.4975	70.39817	123.8223	75.63614
126.5964	82.04441	126.5422	70.3974	123.8616	75.63518
126.6381	82.04482	126.5834	70.39729	123.9028	75.63372
126.68	82.04281	126.6238	70.39494	123.9442	75.63261
126.7245	82.03766	126.6562	70.39303	123.9862	75.63195
126.766	82.03873	126.7013	70.39287	124.028	75.63084
126.809	82.03586	126.7404	70.39022	124.0714	75.62988
126.8392	82.02891	126.7827	70.39005	124.1172	75.62838
126.8841	82.0255	126.8247	70.39148	124.1598	75.62606
126.9256	82.02354	126.8652	70.39109	124.1961	75.62449
126.9682	82.02209	126.9038	70.39373	124.2352	75.6229
127.0099	82.02493	126.95	70.39535	124.2766	75.62169
127.0497	82.01984	126.992	70.39439	124.3198	75.62057
127.0917	82.01192	127.0298	70.3959	124.3602	75.61973
127.1357	82.00721	127.0721	70.39594	124.4036	75.61853
127.1766	82.00861	127.1079	70.39434	124.4459	75.61701
127.2189	82.0184	127.1487	70.39388	124.4832	75.61531
127.2616	82.02049	127.1899	70.39114	124.5261	75.61384
127.3014	82.01946	127.233	70.387	124.5705	75.61195
127.3431	82.01615	127.2752	70.38565	124.61	75.61024
127.3781	82.0135	127.3137	70.38456	124.6545	75.60872
127.4173	82.01175	127.3564	70.38485	124.6937	75.60749
127.4573	82.01252	127.3975	70.38589	124.7346	75.60661
127.5028	82.01278	127.4394	70.3847	124.7786	75.60501
127.5447	82.00726	127.4818	70.38364	124.8098	75.60414
127.5846	82.00413	127.5283	70.38272	124.854	75.60245
127.628	82.00341	127.5606	70.3814	124.8964	75.6013
127.6677	82.00407	127.6003	70.38174	124.9405	75.6005
127.707	82.00175	127.6392	70.38358	124.9807	75.59921
127.7503	82.00008	127.6829	70.38398	125.0234	75.59788
127.7915	81.9993	127.7268	70.38458	125.0638	75.59627
127.8285	81.9987	127.7654	70.38487	125.107	75.59481
127.8681	81.99587	127.8086	70.38296	125.1482	75.59288
127.909	81.99762	127.8476	70.38197	125.1924	75.59132
127.9523	81.99967	127.8898	70.38069	125.2332	75.58924
127.9934	81.99279	127.9346	70.37875	125.2744	75.58765
128.0377	81.99087	127.9756	70.37963	125.3153	75.58628
128.0796	81.99517	128.0101	70.37887	125.3565	75.58561
128.1184	81.99387	128.0513	70.37747	125.3966	75.58462
128.1624	81.98965	128.0936	70.37901	125.432	75.58348
128.2055	81.98752	128.1341	70.38008	125.4726	75.5825
128.2479	81.98089	128.1778	70.38006	125.5134	75.58102
128.2818	81.97697	128.2194	70.38136	125.5554	75.57964
128.321	81.97563	128.26	70.37817	125.6029	75.57808
128.3632	81.9772	128.3004	70.37603	125.6416	75.57627
128.4057	81.97967	128.3418	70.37521	125.6848	75.57416
128.4465	81.97648	128.3829	70.37236	125.7235	75.57301
128.4888	81.97698	128.4246	70.37183	125.7648	75.57184

128.5325	81.97767	128.4581	70.37035	125.8084	75.57111
128.5729	81.97334	128.4996	70.36805	125.8485	75.57057
128.6141	81.9699	128.5447	70.36834	125.8898	75.56878
128.6574	81.97009	128.5869	70.37011	125.9347	75.5669
128.6956	81.96876	128.6284	70.37046	125.9684	75.56556
128.7286	81.96591	128.6692	70.37098	126.0098	75.56365
128.7728	81.96417	128.7119	70.36986	126.054	75.56219
128.8134	81.96459	128.7529	70.36688	126.0906	75.56094
128.8551	81.96073	128.7946	70.36629	126.1316	75.55933
128.8975	81.96154	128.8372	70.36715	126.175	75.55796
128.9366	81.96008	128.8765	70.36732	126.216	75.55636
128.9794	81.95385	128.9094	70.36863	126.2587	75.55518
129.0212	81.95065	128.9525	70.36856	126.2974	75.55401
129.0645	81.94853	128.9939	70.36645	126.3406	75.55267
129.1059	81.95006	129.0355	70.3676	126.388	75.55167
129.1472	81.9542	129.079	70.36568	126.4211	75.55034
129.1914	81.95303	129.12	70.36379	126.4663	75.54947
129.2336	81.95196	129.1605	70.36452	126.5096	75.54832
129.2651	81.95459	129.204	70.36314	126.5489	75.54701
129.3099	81.95135	129.2446	70.3627	126.5829	75.54578
129.3508	81.95439	129.2872	70.36485	126.6241	75.54401
129.3928	81.956	129.329	70.36473	126.6664	75.54256
129.4291	81.95431	129.3633	70.36387	126.7069	75.54122
129.4726	81.94961	129.4038	70.36481	126.7484	75.54035
129.5144	81.94168	129.445	70.36391	126.7897	75.53912
129.5587	81.93867	129.4852	70.36479	126.8311	75.53779
129.6041	81.93351	129.5259	70.36449	126.8715	75.53569
129.6447	81.9353	129.5681	70.36276	126.9143	75.53406
129.689	81.94223	129.6126	70.36311	126.9569	75.53289
129.7202	81.94368	129.6516	70.36234	126.9994	75.53117
129.7617	81.93914	129.6959	70.36131	127.0379	75.53053
129.8025	81.93713	129.7359	70.36124	127.0766	75.52935
129.8468	81.93084	129.779	70.35937	127.1201	75.52781
129.8904	81.92362	129.8121	70.35677	127.1647	75.52674
129.9293	81.91924	129.8527	70.35592	127.1956	75.52503
129.9734	81.91548	129.8958	70.35553	127.2375	75.52336
130.0148	81.91279	129.9355	70.35582	127.2746	75.52171
130.0557	81.90759	129.9794	70.35539	127.3194	75.51974
130.0966	81.90676	130.0202	70.35488	127.3626	75.51869
130.1398	81.90583	130.0615	70.35601	127.4031	75.51747
130.1719	81.90356	130.1033	70.35641	127.4443	75.51651
130.2135	81.90087	130.1426	70.357	127.4824	75.51524
130.2565	81.89948	130.186	70.3555	127.522	75.51411
130.3009	81.89863	130.2274	70.35344	127.5632	75.51266
130.3392	81.89726	130.26	70.35154	127.6049	75.51151
130.383	81.89789	130.3026	70.34943	127.6483	75.5108
130.4256	81.89945	130.3438	70.34975	127.6931	75.50949
130.4669	81.89663	130.3823	70.35097	127.7237	75.50827
130.5052	81.89137	130.4263	70.35094	127.7634	75.50648
130.5458	81.89154	130.4648	70.35277	127.8074	75.50457
130.5894	81.89183	130.5094	70.3525	127.8472	75.50294
130.6259	81.89224	130.5488	70.35321	127.8887	75.50137
130.6672	81.8929	130.5886	70.35449	127.9316	75.49993
130.7076	81.89099	130.6322	70.35154	127.9723	75.49877
130.7505	81.88164	130.6712	70.34999	128.0138	75.49753
130.7942	81.87525	130.7105	70.34935	128.0547	75.49641
130.8348	81.87085	130.7523	70.34634	128.0982	75.49514
130.8754	81.86568	130.7953	70.34598	128.1383	75.49415

130.9152	81.86753	130.837	70.34745	128.1804	75.49293
130.9573	81.86799	130.8797	70.34504	128.2208	75.49175
130.9981	81.86755	130.9198	70.34447	128.2601	75.4902
131.0434	81.86939	130.9628	70.34247	128.2998	75.48833
131.0848	81.87003	131.0059	70.34097	128.3374	75.48771
131.1248	81.86894	131.0456	70.34094	128.3774	75.48597
131.1577	81.86975	131.0864	70.3416	128.419	75.48494
131.1993	81.86928	131.1266	70.34346	128.4591	75.48388
131.2444	81.86481	131.1622	70.34643	128.5021	75.48256
131.2855	81.86469	131.2039	70.34549	128.5411	75.4811
131.3239	81.86242	131.2442	70.34268	128.5836	75.47949
131.3665	81.85977	131.286	70.34118	128.626	75.47827
131.4074	81.85713	131.3287	70.33803	128.6656	75.47647
131.4489	81.85354	131.3708	70.33619	128.7087	75.476
131.4923	81.84933	131.4132	70.33769	128.7499	75.47466
131.534	81.84608	131.4539	70.33854	128.7918	75.47325
131.5745	81.84452	131.4976	70.33646	128.8329	75.47198
131.6074	81.84508	131.5365	70.3376	128.8732	75.46999
131.6462	81.84286	131.576	70.3369	128.9152	75.46863
131.6873	81.84435	131.6111	70.33582	128.9486	75.46763
131.7326	81.84658	131.6524	70.3359	128.991	75.4658
131.7728	81.84765	131.6941	70.33378	129.0312	75.46394
131.8149	81.85254	131.7363	70.33307	129.0747	75.46332
131.8557	81.85278	131.7794	70.33279	129.1151	75.46193
131.8994	81.85016	131.8215	70.33161	129.157	75.46123
131.9413	81.8434	131.8626	70.33239	129.1987	75.46102
131.9837	81.83843	131.9045	70.33044	129.2399	75.46021
132.0223	81.83982	131.9455	70.32896	129.2836	75.45938
132.0586	81.83845	131.9877	70.32891	129.3239	75.4582
132.0995	81.8372	132.028	70.32856	129.3666	75.45616
132.1381	81.83576	132.0632	70.32937	129.4062	75.45465
132.1826	81.83099	132.1054	70.33007	129.4512	75.45434
132.2269	81.8323	132.1484	70.33106	129.4846	75.45349
132.2686	81.83509	132.1886	70.33174	129.5281	75.45231
132.3098	81.83536	132.2301	70.33238	129.5686	75.45018
132.3483	81.83227	132.2726	70.33089	129.6119	75.44804
132.39	81.82769	132.3134	70.33123	129.656	75.44609
132.4316	81.82443	132.3545	70.33385	129.6998	75.44598
132.4764	81.82286	132.3956	70.3336	129.7424	75.44589
132.5084	81.82232	132.4354	70.33411	129.7837	75.44431
132.5517	81.81843	132.4766	70.33099	129.8279	75.4431
132.5908	81.81543	132.5098	70.3267	129.8705	75.44156
132.6328	81.81445	132.5499	70.32483	129.9122	75.44036
132.6731	81.81237	132.5924	70.32523	129.9537	75.43955
132.7154	81.81319	132.6358	70.32473	129.9954	75.4383
132.7573	81.81282	132.6768	70.32727	130.0414	75.43631
132.7986	81.81185	132.7195	70.32756	130.0852	75.43509
132.8423	81.81292	132.765	70.32581	130.1173	75.43397
132.8819	81.81488	132.8029	70.32721	130.1595	75.43291
132.9253	81.8152	132.8447	70.32699	130.2051	75.43227
132.9654	81.81713	132.8862	70.32593	130.2466	75.43133
133.0064	81.81636	132.928	70.3263	130.287	75.42989
133.0404	81.81364	132.9625	70.32549	130.3319	75.42871
133.0818	81.81497	133.0024	70.32419	130.3731	75.42741
133.1258	81.81425	133.05	70.32729	130.4148	75.4256
133.1654	81.81269	133.0911	70.32721	130.457	75.42446
133.2078	81.81056	133.1312	70.32549	130.5043	75.42312
133.2501	81.80373	133.1765	70.32316	130.5449	75.42195

133.2911	81.79695	133.2159	70.31758	130.5861	75.42091
133.3332	81.79312	133.2571	70.31584	130.6309	75.41992
133.3744	81.7903	133.3026	70.31412	130.6718	75.41883
133.4184	81.7918	133.3444	70.31308	130.7152	75.41804
133.4585	81.79606	133.3855	70.3138	130.7467	75.4171
133.493	81.79539	133.4205	70.31332	130.7846	75.41569
133.5355	81.79557	133.4616	70.31373	130.8311	75.41418
133.5758	81.79881	133.5032	70.31443	130.8744	75.41259
133.6184	81.79357	133.5451	70.31373	130.9184	75.41103
133.6602	81.79337	133.5861	70.31213	130.961	75.40986
133.7023	81.79097	133.6278	70.3101	131.0011	75.40854
133.7436	81.78591	133.6734	70.3082	131.0459	75.40722
133.7854	81.78511	133.7117	70.30705	131.0872	75.40608
133.8253	81.78296	133.755	70.30687	131.1309	75.40494
133.8665	81.78516	133.7958	70.30802	131.1726	75.4042
133.9107	81.78835	133.8392	70.3071	131.2154	75.40267
133.9387	81.78778	133.8702	70.30666	131.2558	75.40164
133.9835	81.7843	133.9114	70.30635	131.2935	75.40056
134.0217	81.77941	133.9534	70.30635	131.335	75.39877
134.0676	81.77446	133.9945	70.30621	131.3744	75.39728
134.1075	81.77058	134.034	70.30589	131.416	75.39515
134.1504	81.77241	134.0752	70.30589	131.4564	75.39375
134.1937	81.7785	134.1183	70.30531	131.5001	75.39296
134.2322	81.78186	134.1602	70.3054	131.5406	75.39241
134.2723	81.78368	134.2024	70.30566	131.5863	75.39204
134.319	81.77639	134.2424	70.30543	131.6278	75.39184
134.3603	81.76739	134.2885	70.30514	131.6729	75.39016
134.3928	81.76048	134.3193	70.30653	131.7148	75.38879
134.434	81.75282	134.3632	70.3071	131.7569	75.38724
134.4743	81.75563	134.4049	70.30652	131.8007	75.38542
134.5192	81.75666	134.4464	70.30598	131.8406	75.38477
134.5585	81.75629	134.4879	70.30397	131.8822	75.38381
134.6032	81.75669	134.527	70.30125	131.9147	75.38316
134.6462	81.75889	134.5672	70.29923	131.9557	75.38187
134.6853	81.76054	134.6099	70.29703	131.9983	75.38039
134.7257	81.75993	134.6546	70.29671	132.0394	75.37865
134.7681	81.7547	134.6923	70.29741	132.0807	75.37735
134.8123	81.74739	134.7336	70.29767	132.1273	75.37625
134.8509	81.74346	134.764	70.29875	132.1697	75.37499
134.8918	81.7429	134.8061	70.29862	132.211	75.37399
134.923	81.74585	134.8472	70.2971	132.2524	75.37285
134.9671	81.74489	134.8906	70.29787	132.2923	75.3714
135.0092	81.73878	134.9342	70.29735	132.3346	75.37077
135.0519	81.73476	134.9764	70.29664	132.3801	75.3696
135.0923	81.73475	135.0145	70.29712	132.4199	75.36895
135.1356	81.73737	135.0588	70.2983	132.4609	75.36843
135.1729	81.73899	135.1022	70.2977	132.5005	75.36674
135.2158	81.73828	135.1446	70.29671	132.5372	75.36569
135.2576	81.73584	135.1864	70.2953	132.5778	75.36398
135.3016	81.73589	135.2149	70.29311	132.6204	75.36217
135.342	81.73764	135.2593	70.29197	132.6623	75.36092
135.3764	81.73781	135.301	70.2921	132.7	75.35989
135.418	81.73473	135.3411	70.29233	132.7466	75.35834
135.461	81.72983	135.3801	70.29123	132.785	75.35691
135.5018	81.73057	135.4238	70.28916	132.8273	75.35558
135.545	81.73234	135.4681	70.28888	132.8646	75.35473
135.5911	81.73517	135.509	70.28788	132.9104	75.35423
135.6334	81.74036	135.5513	70.28754	132.9504	75.35329

135.6718	81.73688	135.5945	70.2888	132.9916	75.35227
135.7151	81.73619	135.635	70.28872	133.0323	75.35045
135.756	81.73071	135.6681	70.28984	133.0667	75.34935
135.7992	81.72419	135.708	70.29061	133.1074	75.34779
135.8352	81.72474	135.7503	70.29085	133.1493	75.34629
135.8757	81.72083	135.7898	70.29071	133.191	75.34549
135.9159	81.72146	135.8347	70.28892	133.2306	75.34416
135.9591	81.72111	135.8761	70.28809	133.2739	75.34297
135.9992	81.71985	135.9154	70.28817	133.314	75.34153
136.0413	81.71478	135.9586	70.28727	133.3556	75.34043
136.0808	81.71176	135.9995	70.2887	133.396	75.33887
136.1278	81.70965	136.0426	70.28892	133.4363	75.33783
136.1711	81.70302	136.0796	70.28717	133.4757	75.3375
136.2106	81.70587	136.1142	70.28676	133.517	75.33614
136.2518	81.70978	136.1556	70.28584	133.5584	75.33557
136.2844	81.71108	136.1991	70.2848	133.5986	75.33416
136.3293	81.71308	136.2411	70.28452	133.6372	75.33231
136.3685	81.71047	136.283	70.28317	133.673	75.33102
136.4122	81.70715	136.3226	70.28206	133.7122	75.32919
136.4532	81.70571	136.3666	70.28029	133.7576	75.32744
136.4955	81.70228	136.407	70.27948	133.7988	75.32629
136.5394	81.70625	136.4484	70.2797	133.8408	75.32511
136.578	81.70768	136.492	70.2785	133.8798	75.32405
136.6241	81.70738	136.5345	70.27858	133.9208	75.32325
136.6622	81.70857	136.5694	70.27847	133.9639	75.32201
136.7056	81.70653	136.6089	70.27817	134.0039	75.32073
136.7511	81.70222	136.6487	70.27786	134.0425	75.31904
136.7914	81.69331	136.6901	70.2774	134.0803	75.31765
136.8227	81.69011	136.7296	70.27781	134.1237	75.3164
136.8633	81.69044	136.7747	70.27715	134.167	75.31541
136.9073	81.68696	136.8147	70.27703	134.2076	75.31443
136.948	81.68818	136.8562	70.27729	134.2476	75.31272
136.9918	81.68835	136.8961	70.27729	134.2792	75.31137
137.0323	81.68713	136.9378	70.27736	134.319	75.30976
137.0745	81.68764	136.9818	70.27718	134.3643	75.30876
137.1172	81.68306	137.0173	70.27715	134.4043	75.30794
137.1593	81.67961	137.0572	70.27712	134.447	75.30784
137.2012	81.67373	137.0983	70.27729	134.489	75.30727
137.2434	81.66703	137.1402	70.27719	134.5309	75.30619
137.2769	81.67027	137.1813	70.27753	134.5711	75.30487
137.317	81.67533	137.2222	70.27744	134.6146	75.30346
137.3594	81.67822	137.2646	70.27658	134.6543	75.30182
137.4018	81.68042	137.3064	70.27698	134.6974	75.30089
137.444	81.67754	137.3465	70.27731	134.7357	75.30029
137.4828	81.67716	137.3908	70.2772	134.7798	75.29912
137.5264	81.67377	137.4325	70.27761	134.8116	75.29885
137.5687	81.66906	137.4679	70.27781	134.853	75.29744
137.6098	81.66933	137.5066	70.27614	134.8961	75.29588
137.6535	81.66551	137.548	70.27504	134.9379	75.29408
137.6941	81.66172	137.5888	70.27355	134.9783	75.29271
137.7271	81.66329	137.632	70.27205	135.0171	75.29169
137.7665	81.66502	137.6712	70.2726	135.0627	75.29086
137.8128	81.66808	137.7114	70.27219	135.0989	75.28976
137.8521	81.66754	137.7568	70.27275	135.1447	75.2883
137.8932	81.66322	137.7966	70.27284	135.1813	75.28701
137.9376	81.6566	137.8388	70.27238	135.2222	75.28585
137.9759	81.65134	137.8782	70.27142	135.2648	75.28551
138.0206	81.649	137.913	70.27062	135.3082	75.28469

138.0631	81.65287	137.9552	70.27002	135.3471	75.28367
138.1034	81.65304	137.9951	70.26925	135.3876	75.28268
138.1426	81.65178	138.039	70.26855	135.4182	75.28149
138.1799	81.64956	138.0812	70.26769	135.4659	75.28005
138.2205	81.64653	138.1247	70.26616	135.5061	75.27922
138.2629	81.64332	138.1669	70.26443	135.5491	75.27815
138.3047	81.63646	138.2084	70.26294	135.5909	75.27701
138.3466	81.63626	138.2475	70.2615	135.6344	75.27578
138.3894	81.63105	138.2916	70.26108	135.675	75.27447
138.4303	81.62936	138.3346	70.26074	135.715	75.27302
138.4716	81.63368	138.369	70.26074	135.7525	75.27162
138.5116	81.63351	138.4088	70.26131	135.8008	75.27058
138.5554	81.63411	138.4522	70.26211	135.8427	75.2685
138.5983	81.63145	138.4914	70.26238	135.8807	75.2669
138.6393	81.6261	138.5326	70.26238	135.9225	75.266
138.681	81.61937	138.5778	70.26142	135.9654	75.26483
138.7141	81.61461	138.6164	70.25952	136.005	75.26408
138.7519	81.61455	138.6593	70.25747	136.0396	75.26377
138.7937	81.61503	138.6984	70.25611	136.083	75.26267
138.8354	81.61434	138.7377	70.25585	136.1262	75.26174
138.8747	81.61774	138.7811	70.25568	136.168	75.26118
138.9205	81.61755	138.8136	70.25646	136.2096	75.26032
138.964	81.61662	138.8564	70.25709	136.2515	75.25913
139.0048	81.61629	138.9008	70.25722	136.2925	75.25761
139.0454	81.61741	138.9419	70.25603	136.332	75.25597
139.0883	81.6171	138.9844	70.25428	136.3759	75.25396
139.1298	81.61479	139.0241	70.25314	136.4173	75.25264
139.1626	81.61722	139.0672	70.25221	136.4571	75.25168
139.2037	81.62061	139.1099	70.25176	136.5031	75.25082
139.2447	81.62075	139.151	70.25096	136.5406	75.25063
139.2879	81.61556	139.1934	70.24956	136.5746	75.24981
139.3287	81.61088	139.2339	70.24968	136.6162	75.24874
139.3739	81.60858	139.2675	70.25026	136.6584	75.24692
139.4136	81.60861	139.31	70.25013	136.6984	75.24531
139.4528	81.6112	139.3519	70.25116	136.7419	75.24374
139.4963	81.61239	139.3971	70.24988	136.7826	75.24254
139.5381	81.61012	139.4377	70.24861	136.8269	75.24155
139.579	81.6042	139.4792	70.24924	136.8682	75.24083
139.617	81.60113	139.5237	70.24911	136.9079	75.24004
139.6595	81.60359	139.5631	70.25005	136.9502	75.2384
139.6977	81.60151	139.6043	70.25072	136.9907	75.23746
139.7414	81.60276	139.6456	70.2493	137.0309	75.23601
139.7808	81.60655	139.6882	70.24765	137.0743	75.23539
139.824	81.60626	139.7261	70.24665	137.1164	75.23492
139.8677	81.60777	139.7652	70.24526	137.1552	75.23433
139.9074	81.60259	139.8049	70.24429	137.1911	75.23342
139.9481	81.60158	139.8464	70.24283	137.2341	75.23233
139.9906	81.60029	139.8883	70.2414	137.2764	75.23102
140.032	81.59775	139.9304	70.24104	137.3218	75.22914
140.0651	81.59914	139.9694	70.2415	137.3638	75.22786
140.105	81.60108	140.0106	70.2418	137.4049	75.22605
140.1498	81.59608	140.0564	70.24249	137.4476	75.22499
140.1909	81.58789	140.0982	70.24151	137.4876	75.22398
140.2316	81.5846	140.1354	70.24041	137.5297	75.22268
140.2751	81.5803	140.1699	70.23971	137.5729	75.22132
140.317	81.58257	140.2107	70.23885	137.6159	75.22012
140.3589	81.58575	140.2567	70.23881	137.6576	75.21887
140.3972	81.58641	140.2946	70.23946	137.7	75.21838

140.438	81.58273	140.3379	70.24139	137.7418	75.21806
140.4791	81.57546	140.3803	70.24099	137.7835	75.21695
140.5246	81.57571	140.4206	70.24221	137.8201	75.21669
140.5666	81.57656	140.4626	70.24203	137.8612	75.21519
140.6015	81.57712	140.5056	70.24113	137.9045	75.21382
140.6424	81.57701	140.5464	70.24194	137.9448	75.21238
140.6851	81.57138	140.5851	70.24024	137.9898	75.21098
140.7278	81.56086	140.6198	70.23989	138.0294	75.20907
140.7676	81.5541	140.6598	70.23865	138.0744	75.20757
140.8089	81.55264	140.7046	70.23854	138.1158	75.20609
140.8488	81.55307	140.7464	70.23756	138.1588	75.20494
140.8899	81.55721	140.7884	70.23716	138.2	75.205
140.9336	81.56097	140.8284	70.23713	138.2413	75.20375
140.9745	81.56125	140.8701	70.23695	138.2816	75.20305
141.0174	81.56031	140.9115	70.2381	138.3259	75.2017
141.0548	81.55575	140.9545	70.2376	138.3588	75.201
141.0917	81.55367	140.9977	70.23697	138.4021	75.19955
141.1331	81.55587	141.0408	70.23563	138.4465	75.19868
141.1737	81.55632	141.0722	70.23396	138.4883	75.19814
141.2188	81.55734	141.1117	70.23317	138.5296	75.19697
141.2582	81.55499	141.1518	70.2314	138.5719	75.19591
141.3011	81.55123	141.1922	70.23024	138.6116	75.19458
141.342	81.54399	141.2338	70.23059	138.6561	75.19339
141.382	81.54256	141.2768	70.22845	138.6984	75.19244
141.4247	81.53871	141.3181	70.22897	138.7386	75.19246
141.4642	81.53482	141.3618	70.22863	138.7835	75.19177
141.4966	81.53436	141.4013	70.22726	138.8248	75.19095
141.5384	81.53445	141.447	70.22759	138.8683	75.18973
141.584	81.53396	141.486	70.22726	138.9103	75.18855
141.6218	81.52889	141.5212	70.22673	138.9498	75.18768
141.6654	81.52634	141.5624	70.22685	138.9834	75.18708
141.7077	81.52442	141.6032	70.22673	139.0248	75.18568
141.7472	81.52184	141.6425	70.22562	139.0698	75.18386
141.7912	81.52617	141.6849	70.22436	139.1111	75.18276
141.8323	81.52391	141.7269	70.22332	139.1502	75.18124
141.8708	81.52441	141.7682	70.22204	139.1925	75.18059
141.914	81.52424	141.8118	70.22179	139.2352	75.17976
141.9452	81.52021	141.8522	70.22144	139.28	75.17938
141.9864	81.52402	141.8928	70.22055	139.3241	75.17876
142.03	81.52814	141.9346	70.22081	139.3642	75.17819
142.071	81.52856	141.9698	70.22066	139.4051	75.17692
142.1133	81.528	142.0127	70.21966	139.4486	75.17519
142.155	81.52706	142.0537	70.22009	139.4914	75.17437
142.1976	81.51796	142.095	70.22007	139.5306	75.17344
142.244	81.51129	142.1355	70.2196	139.5734	75.17305
142.2834	81.51299	142.1761	70.21965	139.6074	75.17201
142.3237	81.51558	142.2175	70.21879	139.6487	75.17105
142.3639	81.5194	142.2605	70.21781	139.6902	75.16913
142.4064	81.51756	142.3042	70.21784	139.7356	75.1678
142.4467	81.51695	142.3433	70.21867	139.7756	75.16684
142.4807	81.51443	142.3813	70.21882	139.8144	75.16539
142.5223	81.51414	142.4212	70.21886	139.859	75.16439
142.563	81.5165	142.4636	70.21707	139.8995	75.16299
142.6056	81.51623	142.5025	70.21481	139.9376	75.16163
142.6493	81.51095	142.5454	70.2148	139.9806	75.16
142.6894	81.50352	142.5875	70.215	140.0209	75.15894
142.7345	81.50126	142.6276	70.21506	140.063	75.15739
142.7765	81.49748	142.6682	70.21512	140.1063	75.15634

142.819	81.49719	142.7095	70.21297	140.1383	75.15571
142.8575	81.50002	142.7485	70.21142	140.1772	75.15418
142.8976	81.50088	142.7949	70.21158	140.2205	75.15308
142.9329	81.4995	142.8344	70.21169	140.2621	75.15234
142.9718	81.49774	142.8699	70.21226	140.3031	75.15146
143.0129	81.49572	142.9128	70.21331	140.3404	75.15037
143.0562	81.49578	142.9549	70.21318	140.3835	75.14936
143.0968	81.49805	142.9955	70.2116	140.4251	75.14815
143.1402	81.49569	143.0384	70.2113	140.4639	75.14698
143.1804	81.48954	143.0787	70.20998	140.5076	75.14638
143.2208	81.4835	143.1193	70.20945	140.5479	75.14643
143.2632	81.47639	143.1638	70.21194	140.5898	75.14516
143.3094	81.4759	143.2062	70.21245	140.6308	75.14355
143.3504	81.47852	143.246	70.21213	140.6728	75.14185
143.3838	81.47754	143.2916	70.21155	140.7104	75.13932
143.4255	81.47439	143.3229	70.20923	140.7428	75.13802
143.4697	81.47271	143.363	70.20653	140.7882	75.13653
143.5116	81.46929	143.4062	70.20596	140.8285	75.13578
143.5511	81.46831	143.446	70.2057	140.8705	75.135
143.5906	81.47072	143.4877	70.20557	140.9123	75.13412
143.6367	81.47144	143.5295	70.20562	140.9531	75.13338
143.6769	81.4702	143.5705	70.20407	140.9958	75.13223
143.7198	81.46751	143.6134	70.20464	141.0349	75.1309
143.7607	81.469	143.6536	70.20475	141.0789	75.12995
143.8042	81.46632	143.6954	70.20424	141.1196	75.12934
143.8368	81.46435	143.7401	70.20434	141.1616	75.12877
143.8779	81.46239	143.7734	70.20273	141.2032	75.12818
143.9208	81.46136	143.8147	70.20153	141.2472	75.12737
143.9624	81.46293	143.8541	70.2006	141.2864	75.12626
144.0018	81.46335	143.8966	70.20168	141.3292	75.12481
144.0436	81.46266	143.9402	70.20216	141.365	75.12336
144.0847	81.45944	143.9821	70.20144	141.4031	75.12222
144.1249	81.45545	144.0214	70.20325	141.4428	75.12126
144.1671	81.45006	144.0644	70.20144	141.4866	75.12018
144.2086	81.45038	144.1055	70.20015	141.5278	75.11958
144.2507	81.45063	144.1476	70.20261	141.569	75.11902
144.2962	81.44847	144.1894	70.20252	141.611	75.11816
144.3416	81.45107	144.2224	70.20197	141.652	75.11732
144.3734	81.45487	144.2634	70.202	141.6941	75.11636
144.4151	81.45252	144.3071	70.19945	141.7319	75.11527
144.4558	81.44817	144.3484	70.19869	141.7758	75.11402
144.4999	81.44557	144.3859	70.20033	141.8177	75.11302
144.5432	81.43887	144.4287	70.1987	141.8582	75.11168
144.5838	81.43522	144.4706	70.19671	141.8902	75.11018
144.6261	81.43312	144.5129	70.19432	141.9327	75.10862
144.6665	81.4368	144.5558	70.19069	141.9716	75.10686
144.7075	81.44132	144.5969	70.19114	142.0161	75.10563
144.7504	81.44229	144.639	70.19189	142.0554	75.10494
144.7902	81.44279	144.6718	70.19146	142.0996	75.10426
144.8265	81.44218	144.7115	70.19069	142.1416	75.10362
144.8638	81.4374	144.7553	70.19071	142.1827	75.10302
144.9052	81.43307	144.7949	70.19024	142.2244	75.10232
144.9476	81.43142	144.8362	70.19077	142.2665	75.10172
144.991	81.42928	144.8786	70.19118	142.3074	75.10084
145.0326	81.42712	144.9198	70.19098	142.3492	75.09907
145.0726	81.42403	144.9625	70.1912	142.3933	75.09785
145.1162	81.42379	145.0032	70.19032	142.4344	75.09683
145.1612	81.4209	145.0434	70.19003	142.4748	75.09558

145.2012	81.41565	145.086	70.19029	142.5076	75.09535
145.2426	81.41207	145.1206	70.18916	142.548	75.09461
145.2761	81.41015	145.1594	70.18858	142.5921	75.09318
145.3168	81.40866	145.2006	70.18749	142.6319	75.09206
145.356	81.41148	145.2441	70.18713	142.673	75.09072
145.3987	81.41381	145.2871	70.18694	142.7183	75.08952
145.4404	81.41701	145.3277	70.18598	142.76	75.08875
145.4834	81.41257	145.3678	70.18522	142.802	75.08816
145.5236	81.40888	145.4107	70.18433	142.8435	75.08738
145.5686	81.40847	145.451	70.18332	142.8851	75.08652
145.6099	81.40459	145.4949	70.18362	142.9261	75.08513
145.6509	81.40544	145.5376	70.18339	142.9706	75.08379
145.6938	81.40469	145.5692	70.18226	143.011	75.08281
145.7268	81.4068	145.611	70.18255	143.0538	75.08146
145.7668	81.40669	145.652	70.18268	143.0932	75.0805
145.8096	81.4057	145.694	70.18292	143.1288	75.0798
145.8518	81.40068	145.7344	70.18449	143.1727	75.07912
145.8934	81.39505	145.7754	70.18378	143.2164	75.07815
145.9352	81.38964	145.8179	70.18396	143.2574	75.07728
145.9783	81.38992	145.8593	70.18441	143.2956	75.07618
146.0181	81.39383	145.9017	70.18183	143.334	75.0749
146.0606	81.39052	145.9435	70.18022	143.3759	75.07378
146.1008	81.39026	145.9837	70.17803	143.4213	75.07259
146.1407	81.38646	146.0194	70.17626	143.4626	75.07152
146.1816	81.3842	146.06	70.17597	143.5074	75.07048
146.225	81.38889	146.1028	70.17497	143.549	75.06995
146.2606	81.3882	146.145	70.17336	143.5934	75.06945
146.3013	81.38889	146.1897	70.17303	143.6302	75.0681
146.3417	81.38529	146.2288	70.17363	143.6613	75.06734
146.3852	81.37825	146.2688	70.17346	143.7052	75.06635
146.4228	81.37647	146.3146	70.17516	143.7497	75.06526
146.4664	81.37464	146.3531	70.17668	143.7908	75.06497
146.5059	81.37192	146.3974	70.17469	143.8342	75.06404
146.5471	81.37131	146.4374	70.17523	143.8756	75.06266
146.5928	81.37155	146.4698	70.17538	143.9208	75.06179
146.6343	81.36949	146.5115	70.17377	143.9593	75.06083
146.6761	81.36847	146.5533	70.17452	144.0014	75.06023
146.7092	81.3688	146.5966	70.17602	144.044	75.05933
146.7508	81.36905	146.6382	70.17438	144.0874	75.05793
146.7942	81.36638	146.6813	70.17121	144.1276	75.05639
146.8362	81.3658	146.7188	70.16992	144.171	75.05483
146.8758	81.36366	146.7629	70.16684	144.2133	75.05389
146.9169	81.35997	146.8028	70.16776	144.2549	75.05299
146.9571	81.35766	146.8453	70.16983	144.2876	75.05244
147.0005	81.35574	146.8868	70.16836	144.3283	75.05117
147.0429	81.35791	146.923	70.16888	144.3717	75.05008
147.0858	81.36043	146.9639	70.16864	144.4102	75.04915
147.1272	81.36029	147.0049	70.16726	144.4549	75.04784
147.159	81.36084	147.0468	70.16796	144.4954	75.0475
147.2023	81.36315	147.0889	70.16999	144.5402	75.04616
147.2446	81.35875	147.1322	70.16884	144.578	75.04523
147.2871	81.35516	147.1725	70.16841	144.6191	75.04471
147.3284	81.35551	147.2162	70.16648	144.6619	75.04348
147.3705	81.35519	147.2579	70.16319	144.7053	75.04205
147.4119	81.35684	147.2994	70.16367	144.7464	75.03969
147.454	81.36352	147.3362	70.16377	144.7873	75.03776
147.4938	81.36654	147.3697	70.16346	144.8296	75.03634
147.5362	81.36226	147.4131	70.16264	144.8745	75.03594

147.58	81.35561	147.4567	70.16071	144.906	75.03545
147.6119	81.34785	147.4953	70.15813	144.9455	75.03496
147.6538	81.34464	147.5367	70.15762	144.9895	75.03417
147.6956	81.34389	147.578	70.15749	145.0309	75.03307
147.7339	81.34175	147.6208	70.15704	145.074	75.03254
147.7764	81.34297	147.6609	70.15744	145.1115	75.03176
147.8207	81.34056	147.7018	70.15769	145.1586	75.03051
147.862	81.33408	147.7441	70.15765	145.1986	75.02885
147.9038	81.3347	147.785	70.15878	145.2399	75.0275
147.9467	81.33735	147.8181	70.15817	145.2814	75.02638
147.9843	81.33524	147.8619	70.15677	145.3252	75.0254
148.0276	81.33649	147.9014	70.15704	145.366	75.02537
148.0707	81.33368	147.9428	70.15564	145.4076	75.02487
148.1114	81.32704	147.984	70.15532	145.4406	75.02425
148.1454	81.32613	148.0267	70.15581	145.4832	75.02362
148.1867	81.32569	148.071	70.15435	145.5243	75.02272
148.2265	81.32949	148.1087	70.15392	145.5653	75.02169
148.2695	81.33286	148.151	70.15461	145.6054	75.02036
148.3117	81.32981	148.1952	70.15399	145.6481	75.01916
148.3506	81.32599	148.2379	70.154	145.6899	75.01802
148.3915	81.31867	148.2704	70.1545	145.7324	75.01699
148.4334	81.3121	148.3128	70.15279	145.7756	75.01626
148.4779	81.31167	148.3549	70.15066	145.817	75.01573
148.5161	81.31241	148.3962	70.15326	145.8598	75.01485
148.5581	81.31254	148.4366	70.15382	145.8987	75.0137
148.5914	81.31023	148.4782	70.15343	145.9381	75.012
148.6348	81.30993	148.5238	70.15438	145.9835	75.00995
148.6758	81.31091	148.5604	70.14988	146.0231	75.00822
148.721	81.30904	148.6027	70.14747	146.0582	75.00699
148.7617	81.30897	148.6434	70.14637	146.1009	75.00649
148.8048	81.30347	148.6842	70.14594	146.144	75.00603
148.8432	81.29774	148.7212	70.14801	146.182	75.0062
148.8879	81.29651	148.7601	70.15069	146.2252	75.00574
148.9317	81.2973	148.8026	70.15122	146.2673	75.00448
148.9719	81.29842	148.8435	70.15075	146.3116	75.00324
149.0137	81.29586	148.8814	70.1517	146.3533	75.00179
149.0448	81.29447	148.9292	70.14834	146.3975	75.0004
149.0869	81.29068	148.9732	70.146	146.4376	74.99942
149.1281	81.28802	149.0124	70.14587	146.4784	74.99859
149.1741	81.287	149.0519	70.14198	146.5194	74.99699
149.2156	81.28884	149.095	70.14065	146.5592	74.996
149.2547	81.2918	149.138	70.14167	146.5986	74.99493
149.2988	81.29011	149.1693	70.14118	146.6421	74.99421
149.338	81.28626	149.2118	70.14079	146.674	74.99368
149.382	81.28441	149.2558	70.14116	146.7182	74.99304
149.4231	81.2837	149.2959	70.14085	146.7579	74.99211
149.4642	81.28279	149.3377	70.14017	146.801	74.99106
149.499	81.28322	149.3778	70.13951	146.8421	74.99035
149.5437	81.28245	149.4211	70.13758	146.8799	74.98904
149.584	81.27997	149.463	70.13862	146.9229	74.98828
149.6258	81.27841	149.4978	70.14151	146.9633	74.98733
149.6686	81.28	149.5395	70.14077	147.0067	74.98586
149.7062	81.28001	149.5847	70.14114	147.0495	74.98444
149.7485	81.27667	149.6185	70.14012	147.0911	74.98305
149.7916	81.27443	149.6612	70.13545	147.1321	74.9818
149.8339	81.27453	149.708	70.13523	147.1775	74.98134
149.8744	81.27426	149.7449	70.13748	147.2093	74.98063
149.9128	81.27225	149.7854	70.13661	147.25	74.97981

149.9597	81.27167	149.8265	70.13694	147.2928	74.9783
150.0006	81.27283	149.8702	70.13541	147.3369	74.97648
150.0318	81.27103	149.9092	70.13312	147.3767	74.97511
150.0769	81.27076	149.9518	70.1346	147.4201	74.97427
150.1157	81.27	149.9967	70.13441	147.4609	74.97345
150.1585	81.26629	150.0376	70.13402	147.5046	74.97303
150.1983	81.26221	150.072	70.13462	147.5462	74.97209
150.2384	81.25769	150.114	70.13274	147.585	74.9709
150.2817	81.25643	150.1526	70.13128	147.6283	74.97026
150.3242	81.25829	150.1953	70.13392	147.671	74.96864
150.3688	81.25524	150.2372	70.1353	147.7106	74.96774
150.409	81.25447	150.2771	70.13525	147.7491	74.96669
150.4472	81.25515	150.3204	70.13521	147.7924	74.96481
150.4848	81.25177	150.3635	70.13189	147.8261	74.964
150.5274	81.25107	150.4028	70.12969	147.8653	74.96277
150.5699	81.25236	150.4448	70.13073	147.9081	74.96155
150.6095	81.2526	150.486	70.13112	147.9496	74.9605
150.6502	81.25121	150.5197	70.13079	147.9932	74.95931
150.6919	81.24914	150.5615	70.13057	148.0341	74.95839
150.7326	81.24811	150.6038	70.12892	148.0783	74.95761
150.7708	81.24517	150.6453	70.12791	148.121	74.95661
150.8134	81.24149	150.6865	70.12765	148.1626	74.95572
150.8536	81.24387	150.7279	70.12614	148.2041	74.95454
150.8966	81.24622	150.7689	70.12575	148.2436	74.95352
150.9291	81.24704	150.8094	70.12595	148.2848	74.95183
150.9704	81.24835	150.8518	70.12557	148.3251	74.95098
151.0121	81.24504	150.8952	70.12482	148.368	74.95029
151.0534	81.2382	150.9372	70.12453	148.4096	74.94978
151.0952	81.23415	150.971	70.12255	148.4414	74.94976
151.1379	81.23385	151.0132	70.12112	148.4824	74.94916
151.1776	81.23462	151.0563	70.12121	148.5276	74.94788
151.2202	81.23279	151.0962	70.12071	148.5658	74.94623
151.2606	81.23103	151.1398	70.12034	148.6075	74.94474
151.3051	81.23103	151.1786	70.12119	148.6462	74.94383
151.3462	81.23013	151.218	70.1221	148.6889	74.9431
151.3775	81.22971	151.263	70.12304	148.7309	74.942
151.4226	81.22941	151.3031	70.12456	148.7748	74.94155
151.4629	81.22729	151.3438	70.12319	148.8157	74.94026
151.5062	81.21965	151.3825	70.12246	148.8577	74.939
151.5458	81.21809	151.4204	70.12303	148.897	74.93778
151.5919	81.21645	151.4596	70.12126	148.9392	74.93692
151.6314	81.21312	151.5057	70.11971	148.9722	74.9368
151.673	81.21157	151.5436	70.11744	149.0128	74.93601
151.7149	81.21023	151.5869	70.11319	149.0553	74.93584
151.7573	81.20842	151.6264	70.11062	149.0963	74.93502
151.8016	81.20772	151.6676	70.11131	149.14	74.93397
151.8407	81.20969	151.7109	70.11179	149.1801	74.933
151.8848	81.21167	151.7531	70.11152	149.2202	74.93178
151.9176	81.21413	151.7926	70.11151	149.2628	74.93092
151.9587	81.2122	151.8374	70.11151	149.3035	74.92943
151.9999	81.20794	151.869	70.11285	149.3454	74.92868
152.0424	81.2016	151.9135	70.11502	149.3864	74.92737
152.0856	81.19516	151.9539	70.11625	149.4269	74.92575
152.1268	81.19205	151.9973	70.11597	149.471	74.92485
152.1659	81.19266	152.0379	70.11519	149.5115	74.92387
152.2065	81.19639	152.0794	70.1138	149.5519	74.92264
152.2476	81.1956	152.1225	70.11395	149.5826	74.92212
152.2926	81.1925	152.1614	70.11463	149.6276	74.92087

152.3344	81.19296	152.2037	70.11262	149.6673	74.91951
152.3678	81.19025	152.2466	70.11114	149.7062	74.91894
152.4109	81.18654	152.2889	70.10958	149.7528	74.91764
152.4497	81.18904	152.3204	70.10768	149.7899	74.9167
152.4915	81.18917	152.3628	70.10625	149.8341	74.91565
152.5351	81.1866	152.4041	70.10515	149.8741	74.91451
152.5776	81.18961	152.4453	70.10354	149.918	74.91333
152.6187	81.1897	152.4842	70.10272	149.9595	74.91195
152.6593	81.18793	152.5284	70.10185	149.9966	74.91103
152.7027	81.1882	152.5716	70.10227	150.0396	74.91052
152.7434	81.18287	152.6136	70.10446	150.0797	74.90996
152.7848	81.18019	152.653	70.10509	150.1198	74.90933
152.8182	81.17845	152.6933	70.10536	150.1639	74.90848
152.8598	81.1759	152.7368	70.10555	150.1956	74.90721
152.9033	81.17376	152.7685	70.10392	150.2405	74.90632
152.9436	81.17233	152.8066	70.10199	150.2792	74.90507
152.9825	81.17154	152.8479	70.10406	150.3225	74.90395
153.0262	81.16938	152.8915	70.10313	150.3661	74.90318
153.0676	81.17009	152.9339	70.10279	150.4061	74.90239
153.1118	81.16686	152.9747	70.10301	150.4484	74.90178
153.1494	81.16191	153.0169	70.10003	150.4894	74.90098
153.1929	81.15994	153.0602	70.09894	150.5302	74.89978
153.2339	81.15948	153.1038	70.09859	150.5712	74.89882
153.2686	81.15708	153.1476	70.09747	150.6134	74.8978
153.31	81.15658	153.188	70.09834	150.6548	74.89692
153.3514	81.15759	153.2173	70.10012	150.6969	74.8962
153.3942	81.15616	153.2578	70.09878	150.7317	74.89591
153.4339	81.15451	153.303	70.09784	150.7736	74.89486
153.4769	81.15775	153.3459	70.09689	150.8151	74.89389
153.521	81.15894	153.3878	70.09468	150.8556	74.89246
153.5616	81.15679	153.4264	70.09482	150.8951	74.89116
153.6022	81.15706	153.469	70.09736	150.9346	74.88975
153.6452	81.15629	153.5094	70.09771	150.9795	74.88867
153.6865	81.15529	153.5544	70.09677	151.0193	74.88762
153.7263	81.15326	153.5927	70.09701	151.0606	74.88616
153.7668	81.15303	153.6349	70.09349	151.1018	74.88529
153.8012	81.15225	153.6686	70.0918	151.1459	74.88403
153.8423	81.15143	153.71	70.09277	151.1856	74.88304
153.8835	81.14775	153.7509	70.09215	151.2264	74.88151
153.9224	81.1502	153.7922	70.09212	151.2691	74.8804
153.9644	81.14955	153.8343	70.09385	151.3121	74.87957
154.0076	81.14154	153.8774	70.09311	151.3447	74.87869
154.0483	81.14205	153.919	70.09202	151.3841	74.87824
154.0886	81.14029	153.9587	70.09178	151.4263	74.87749
154.1295	81.13697	154.0018	70.09081	151.4683	74.87643
154.1731	81.13232	154.0422	70.09048	151.508	74.87546
154.2167	81.12748	154.0849	70.09075	151.5497	74.87417
154.2533	81.12515	154.1178	70.09209	151.5901	74.87258
154.294	81.1239	154.16	70.09054	151.6358	74.87153
154.3335	81.12312	154.2021	70.08855	151.6766	74.87034
154.378	81.12653	154.246	70.08857	151.7154	74.86959
154.4177	81.12736	154.2866	70.08711	151.7592	74.86902
154.461	81.12211	154.3291	70.08624	151.802	74.86842
154.5023	81.12341	154.3731	70.08656	151.8404	74.86771
154.5435	81.12105	154.4137	70.08558	151.882	74.86688
154.584	81.11634	154.4542	70.08579	151.9237	74.86658
154.6256	81.11354	154.4945	70.08507	151.9551	74.86552
154.6686	81.10784	154.5373	70.085	151.9985	74.86445

154.7046	81.10458	154.5684	70.08501	152.0403	74.86301
154.744	81.10558	154.6117	70.0821	152.0828	74.86143
154.788	81.10566	154.6534	70.08103	152.1233	74.8597
154.8287	81.10813	154.6942	70.0798	152.163	74.85811
154.8674	81.10829	154.7329	70.0791	152.2071	74.85694
154.9126	81.101	154.7776	70.08046	152.2456	74.85664
154.9552	81.09493	154.8206	70.0801	152.2881	74.85675
154.9946	81.09471	154.8598	70.07955	152.3305	74.85633
155.0395	81.09491	154.9035	70.07931	152.3698	74.85602
155.0813	81.09651	154.9431	70.07724	152.4154	74.85448
155.1191	81.09952	154.9836	70.07648	152.4566	74.85281
155.1518	81.09932	155.0169	70.07704	152.4881	74.85213
155.1929	81.09791	155.0594	70.07562	152.5288	74.85088
155.2364	81.09475	155.1008	70.076	152.573	74.85061
155.2738	81.0922	155.1437	70.07724	152.6141	74.85039
155.3133	81.0893	155.1867	70.07592	152.6541	74.84925
155.3545	81.08636	155.2232	70.07471	152.6994	74.84899
155.3997	81.08396	155.2671	70.07373	152.7401	74.84903
155.4418	81.08456	155.3074	70.07245	152.7842	74.8479
155.4844	81.08136	155.346	70.07169	152.8261	74.84688
155.5249	81.07901	155.3896	70.0737	152.8632	74.84507
155.5707	81.07993	155.4336	70.07325	152.9043	74.84243
155.6116	81.07927	155.4641	70.07232	152.9471	74.84062
155.6521	81.07943	155.5084	70.07244	152.9906	74.83935
155.6867	81.07964	155.5532	70.07043	153.0294	74.83797
155.7265	81.07866	155.5932	70.06997	153.0737	74.83699
155.7704	81.07249	155.6326	70.07024	153.1073	74.83642
155.8123	81.07167	155.672	70.06913	153.15	74.83596
155.8514	81.07221	155.7135	70.06781	153.1915	74.83546
155.8966	81.07306	155.7556	70.06625	153.2328	74.83441
155.938	81.07449	155.7986	70.06418	153.2746	74.83329
155.9802	81.07493	155.8396	70.06138	153.3168	74.83267
156.0223	81.07372	155.8829	70.06004	153.3626	74.83176
156.064	81.0716	155.9182	70.06012	153.4004	74.8306
156.1046	81.0681	155.958	70.06127	153.445	74.82984
156.1394	81.06536	156.0014	70.06329	153.485	74.82843
156.1811	81.06591	156.0424	70.06389	153.5244	74.82734
156.2224	81.06284	156.0796	70.0634	153.567	74.82637
156.2634	81.06286	156.1247	70.06127	153.6082	74.82571
156.3064	81.06062	156.1664	70.05852	153.6518	74.82467
156.3452	81.05119	156.2069	70.05758	153.6938	74.82348
156.3902	81.04704	156.2507	70.05591	153.7259	74.82278
156.4294	81.04656	156.2919	70.05617	153.7667	74.82134
156.4714	81.04902	156.3337	70.05718	153.8111	74.81995
156.5118	81.05162	156.3686	70.05863	153.8484	74.81943
156.5544	81.05431	156.4079	70.0607	153.8926	74.81828
156.5881	81.05631	156.4503	70.06242	153.9364	74.81785
156.6283	81.05617	156.4907	70.06317	153.9778	74.8172
156.6688	81.05414	156.5326	70.06171	154.0153	74.81625
156.7114	81.04993	156.5733	70.05989	154.0593	74.81535
156.7562	81.04645	156.62	70.05751	154.0981	74.81399
156.7939	81.04582	156.6598	70.05696	154.1401	74.81322
156.8364	81.0423	156.7015	70.05835	154.1808	74.81246
156.8767	81.04122	156.7396	70.05882	154.2218	74.81232
156.9203	81.04035	156.7844	70.05778	154.255	74.81194
156.959	81.03237	156.8171	70.05813	154.3005	74.81085
156.9975	81.02865	156.86	70.05704	154.3418	74.80934
157.0326	81.03104	156.9016	70.05469	154.3854	74.80785

157.0754	81.03058	156.9399	70.05503	154.4249	74.80631
157.1188	81.02887	156.9854	70.0537	154.4681	74.80493
157.1581	81.02858	157.0258	70.05286	154.5105	74.80336
157.1982	81.02776	157.0681	70.05258	154.5532	74.80233
157.2438	81.02784	157.1107	70.05194	154.5946	74.80125
157.2847	81.02951	157.1477	70.05135	154.6365	74.80035
157.3262	81.03041	157.1903	70.05118	154.6788	74.79977
157.368	81.02875	157.2354	70.05002	154.7189	74.79882
157.4106	81.02399	157.2677	70.04905	154.761	74.79812
157.451	81.01921	157.3091	70.04793	154.8	74.79773
157.4914	81.01919	157.3495	70.04751	154.8428	74.79736
157.5315	81.01785	157.3905	70.04685	154.8776	74.79664
157.5678	81.01435	157.4372	70.04657	154.9174	74.79562
157.6114	81.01319	157.4762	70.04584	154.9602	74.79434
157.65	81.01379	157.518	70.04383	155.0026	74.79342
157.6924	81.01407	157.5583	70.04464	155.0444	74.79242
157.7337	81.01385	157.6001	70.04446	155.083	74.79177
157.7772	81.01097	157.6414	70.04557	155.1246	74.79098
157.8193	81.00918	157.684	70.04506	155.1669	74.78993
157.862	81.00564	157.719	70.04378	155.2093	74.78905
157.9032	81.00156	157.7606	70.04295	155.2479	74.78811
157.947	81.00437	157.8011	70.04165	155.2908	74.78723
157.986	81.00202	157.844	70.04283	155.3356	74.78591
158.0198	81.00005	157.8818	70.04363	155.3788	74.78531
158.0614	81.00002	157.9236	70.04538	155.4205	74.78413
158.1036	80.99876	157.9667	70.04395	155.4586	74.78284
158.1459	80.99621	158.0102	70.04157	155.4904	74.78209
158.1885	80.99463	158.05	70.03915	155.5331	74.78039
158.2289	80.99498	158.0906	70.03574	155.5772	74.77951
158.2709	80.99413	158.1321	70.03531	155.6182	74.77866
158.3138	80.9913	158.1663	70.0354	155.6581	74.77728
158.3549	80.99204	158.2067	70.0361	155.6997	74.77636
158.4	80.99288	158.2488	70.03706	155.7438	74.77516
158.4375	80.99024	158.2892	70.03725	155.7827	74.77391
158.4708	80.98812	158.3326	70.03802	155.8258	74.77333
158.5146	80.98637	158.3718	70.03888	155.8685	74.77229
158.5543	80.9847	158.4122	70.03919	155.9092	74.77124
158.599	80.98446	158.4571	70.03816	155.9524	74.77053
158.6377	80.98502	158.4985	70.0373	155.997	74.76984
158.6807	80.98241	158.5402	70.03483	156.0275	74.76976
158.7232	80.97657	158.5793	70.03354	156.0716	74.76856
158.7649	80.97048	158.6142	70.03418	156.1081	74.76747
158.807	80.96988	158.6559	70.03238	156.152	74.76605
158.848	80.96874	158.6978	70.03196	156.1944	74.76463
158.8886	80.96786	158.7372	70.03143	156.2333	74.76345
158.9216	80.96753	158.7803	70.02993	156.2774	74.76224
158.9634	80.96858	158.8223	70.02974	156.3163	74.76113
159.0076	80.96936	158.8644	70.02819	156.3583	74.75979
159.0476	80.96971	158.9056	70.02657	156.4014	74.75925
159.0894	80.96693	158.9444	70.02568	156.4414	74.75853
159.1323	80.96583	158.9856	70.0258	156.483	74.75803
159.1706	80.96536	159.0298	70.02516	156.525	74.75716
159.2136	80.96283	159.0649	70.0252	156.568	74.75598
159.2578	80.96437	159.1051	70.02447	156.6091	74.75462
159.3001	80.96055	159.1479	70.02381	156.6403	74.75386
159.3435	80.95495	159.1887	70.024	156.6814	74.75304
159.386	80.94805	159.2292	70.02517	156.7228	74.75201
159.4231	80.94261	159.2733	70.02567	156.7624	74.75113

159.4581	80.94085	159.3152	70.02439	156.808	74.74961
159.4992	80.93958	159.3548	70.02367	156.8516	74.74834
159.5405	80.94004	159.399	70.0226	156.8908	74.74747
159.5801	80.9429	159.4392	70.02181	156.934	74.74712
159.6244	80.94048	159.4824	70.02102	156.9763	74.74621
159.6631	80.93544	159.5166	70.02032	157.0181	74.74506
159.7096	80.93677	159.5577	70.01986	157.0604	74.74345
159.7488	80.93445	159.598	70.01996	157.1024	74.74207
159.7874	80.93312	159.6411	70.01889	157.1445	74.74172
159.8314	80.93446	159.6811	70.01791	157.1883	74.74102
159.8734	80.93342	159.7251	70.01674	157.2299	74.74056
159.9058	80.93461	159.7669	70.01498	157.2628	74.73975
159.9485	80.93735	159.8066	70.01529	157.3049	74.73838
159.9884	80.9358	159.8511	70.01605	157.3486	74.73723
160.0297	80.93364	159.8906	70.01567	157.3911	74.73608
160.0721	80.93206	159.9309	70.01481	157.4312	74.73491
160.1146	80.9294	159.9631	70.01327	157.4721	74.73436
160.1576	80.92755	160.0091	70.01153	157.5154	74.73398
160.197	80.92746	160.0504	70.01048	157.5572	74.73287
160.2411	80.92662	160.0904	70.00888	157.5953	74.73174
160.2806	80.92242	160.1299	70.01057	157.6369	74.73033
160.3215	80.92092	160.1735	70.01135	157.6814	74.72885
160.3543	80.92047	160.2129	70.01105	157.7225	74.72772
160.3981	80.91866	160.2562	70.01104	157.7639	74.72703
160.4375	80.91816	160.2976	70.01022	157.7976	74.72585
160.4798	80.91738	160.3382	70.00913	157.8404	74.72482
160.5222	80.91448	160.3819	70.00841	157.8812	74.72432
160.5652	80.90981	160.4149	70.00831	157.9218	74.72356
160.607	80.90701	160.4582	70.00638	157.9642	74.72252
160.6479	80.91081	160.4973	70.00512	158.0053	74.72161
160.6863	80.90756	160.5391	70.00352	158.0456	74.72078
160.7313	80.90362	160.5791	70.00368	158.0879	74.71988
160.7719	80.90021	160.623	70.00694	158.1274	74.71892
160.805	80.89599	160.664	70.00671	158.1698	74.71792
160.8483	80.89727	160.7052	70.006	158.2096	74.71695
160.8931	80.90012	160.7462	70.00628	158.2535	74.71584
160.9332	80.90164	160.7869	70.00246	158.2946	74.71527
160.9716	80.90188	160.8301	70.00023	158.3331	74.71438
161.0166	80.89942	160.8622	70.00084	158.3756	74.71353
161.0568	80.89992	160.9027	69.99858	158.408	74.7132
161.0972	80.90362	160.9454	69.9961	158.4494	74.71216
161.1399	80.90364	160.9851	69.99816	158.489	74.7112
161.1813	80.8997	161.0284	69.99822	158.533	74.70966
161.2231	80.89291	161.0664	69.99863	158.5731	74.70822
161.2653	80.88377	161.1094	69.99962	158.614	74.70734
161.3069	80.87712	161.1498	69.99678	158.6569	74.70654
161.3378	80.87609	161.1922	69.99636	158.6962	74.706
161.3801	80.87821	161.2383	69.99718	158.7383	74.70452
161.4231	80.87927	161.279	69.99633	158.7785	74.70314
161.464	80.87982	161.314	69.99607	158.8179	74.70195
161.5064	80.87767	161.3565	69.99523	158.858	74.70084
161.5476	80.87366	161.3961	69.99242	158.8996	74.70015
161.5889	80.8701	161.4396	69.99011	158.9387	74.6992
161.6311	80.8706	161.4829	69.99018	158.9811	74.69773
161.6729	80.87066	161.5244	69.98923	159.0139	74.69652
161.7149	80.86795	161.5667	69.98822	159.0532	74.69516
161.7544	80.87013	161.6083	69.98745	159.0964	74.69391
161.7866	80.86499	161.6502	69.98793	159.1379	74.69368

161.8315	80.86471	161.6906	69.98918	159.1791	74.69322
161.8724	80.86786	161.7316	69.98872	159.2207	74.69243
161.9126	80.86224	161.7644	69.9894	159.2592	74.69148
161.9558	80.86425	161.8062	69.98901	159.2994	74.69046
161.9957	80.86134	161.8476	69.98733	159.3425	74.68953
162.0362	80.85577	161.8902	69.98694	159.3839	74.68894
162.0813	80.85451	161.9334	69.98665	159.4263	74.68793
162.1256	80.8564	161.9757	69.98575	159.4668	74.68668
162.1649	80.85884	162.0154	69.98401	159.5094	74.6849
162.2046	80.85821	162.0559	69.9837	159.5398	74.68361
162.2403	80.85961	162.0981	69.98298	159.5803	74.68299
162.2786	80.85972	162.1392	69.98123	159.6238	74.6823
162.322	80.85035	162.1784	69.9807	159.6647	74.6812
162.3648	80.84561	162.2126	69.97982	159.7055	74.6803
162.4037	80.84769	162.2553	69.97876	159.747	74.67942
162.443	80.84595	162.2988	69.97899	159.788	74.67871
162.4861	80.84853	162.3388	69.97878	159.8308	74.67781
162.5247	80.84874	162.3812	69.9805	159.871	74.67672
162.5705	80.84005	162.4233	69.98361	159.9125	74.67579
162.61	80.83461	162.4638	69.98298	159.9544	74.67404
162.6545	80.83075	162.5065	69.98307	159.9925	74.67293
162.6883	80.83027	162.5492	69.98055	160.0331	74.67196
162.728	80.83236	162.5885	69.97571	160.0757	74.67073
162.7696	80.83321	162.6286	69.97491	160.1206	74.66937
162.8079	80.83256	162.6632	69.9738	160.1534	74.66793
162.8489	80.83776	162.7045	69.97296	160.1973	74.66688
162.8922	80.8381	162.745	69.97389	160.2362	74.66586
162.9328	80.83275	162.786	69.97372	160.2759	74.66518
162.9745	80.82923	162.8291	69.97186	160.3181	74.6647
163.0126	80.81907	162.8708	69.97036	160.3565	74.66436
163.056	80.81362	162.9102	69.96883	160.3981	74.66446
163.0995	80.81052	162.952	69.96728	160.4432	74.66359
163.1429	80.81306	162.9948	69.96789	160.4849	74.66286
163.1872	80.82137	163.0351	69.96721	160.5269	74.66183
163.2162	80.82626	163.0779	69.966	160.566	74.65977
163.261	80.82589	163.1117	69.9675	160.61	74.65867
163.3019	80.81917	163.1538	69.96796	160.6527	74.65761
163.3463	80.80984	163.1972	69.96747	160.6922	74.65665
163.3854	80.80426	163.2338	69.96799	160.7352	74.65534
163.4281	80.80534	163.2774	69.96669	160.769	74.65447
163.4692	80.80893	163.3177	69.96574	160.8095	74.65333
163.5112	80.8143	163.3628	69.96667	160.8538	74.65155
163.5525	80.81327	163.4022	69.96593	160.8946	74.65001
163.5949	80.80935	163.4417	69.9654	160.9335	74.6484
163.6396	80.80736	163.4824	69.96554	160.9758	74.64788
163.6682	80.80449	163.5253	69.96469	161.0193	74.6471
163.7133	80.79985	163.5619	69.96428	161.0615	74.64649
163.753	80.7963	163.6005	69.96305	161.1024	74.64615
163.7969	80.79444	163.6432	69.9606	161.1479	74.64553
163.841	80.79367	163.6864	69.95897	161.1858	74.64501
163.8809	80.79332	163.7275	69.95844	161.2287	74.64389
163.9239	80.7963	163.7682	69.95793	161.2703	74.64246
163.9646	80.80049	163.8126	69.95926	161.3028	74.64155
164.007	80.79804	163.8536	69.96014	161.3459	74.64016
164.0456	80.79366	163.8932	69.9616	161.3886	74.63882
164.0896	80.79258	163.9328	69.96335	161.4303	74.63846
164.1246	80.79197	163.975	69.96186	161.4721	74.63783
164.1667	80.78771	164.0106	69.9595	161.5135	74.63706

164.2084	80.78544	164.0498	69.95759	161.5588	74.63663
164.2491	80.78397	164.0962	69.95564	161.5986	74.63523
164.293	80.77907	164.1359	69.95525	161.6422	74.63331
164.3348	80.77211	164.1772	69.95508	161.6786	74.63157
164.378	80.77506	164.2162	69.95396	161.7242	74.63001
164.4186	80.77711	164.2598	69.95286	161.7678	74.62867
164.4626	80.78013	164.3	69.95338	161.8108	74.62772
164.5038	80.78555	164.341	69.95165	161.8492	74.62671
164.5456	80.78276	164.3824	69.95047	161.8961	74.62622
164.5794	80.77869	164.4251	69.94921	161.9293	74.6257
164.6213	80.77243	164.4594	69.94656	161.9704	74.62494
164.6603	80.7679	164.5017	69.94655	162.0088	74.62435
164.7022	80.76888	164.5418	69.94649	162.0548	74.62284
164.7429	80.77478	164.5848	69.94472	162.0966	74.6216
164.786	80.77446	164.6269	69.94436	162.1389	74.6204
164.8274	80.77206	164.6659	69.94348	162.1824	74.61959
164.869	80.76798	164.7101	69.94376	162.2249	74.6188
164.9106	80.75776	164.7532	69.94397	162.2653	74.61767
164.9501	80.75376	164.793	69.9424	162.3076	74.61643
164.992	80.75309	164.8336	69.94117	162.3518	74.61506
165.032	80.75192	164.8759	69.94284	162.3945	74.61365
165.0754	80.75215	164.9094	69.94233	162.4342	74.61271
165.1068	80.75189	164.9495	69.94211	162.4756	74.61167
165.1491	80.75118	164.9918	69.94446	162.5181	74.61067
165.1905	80.74919	165.032	69.94269	162.5514	74.61013
165.2306	80.74998	165.0746	69.94068	162.5908	74.60947
165.2722	80.74998	165.1162	69.94103	162.633	74.60853
165.3158	80.74924	165.1626	69.93993	162.6764	74.60724
165.3587	80.74791	165.2004	69.93855	162.7192	74.6069
165.3998	80.74374	165.2415	69.93875	162.7609	74.60594
165.4449	80.74083	165.2838	69.93741	162.8026	74.60495
165.4835	80.74017	165.3248	69.93517	162.8435	74.60414
165.5252	80.74098	165.3591	69.93487	162.8825	74.60272
165.5575	80.74172	165.4002	69.93433	162.9233	74.60129
165.6002	80.74067	165.4412	69.9334	162.9651	74.59985
165.6412	80.739	165.4834	69.93378	163.0089	74.59888
165.6836	80.73527	165.5253	69.93311	163.0503	74.59702
165.7245	80.7325	165.566	69.93083	163.0858	74.59611
165.7654	80.73239	165.6108	69.93068	163.1248	74.59531
165.8094	80.73289	165.6479	69.93025	163.1668	74.59395
165.8511	80.7317	165.6909	69.92806	163.2083	74.59333
165.8921	80.73186	165.7331	69.92776	163.2504	74.5925
165.9325	80.72972	165.773	69.92621	163.2911	74.59164
165.9742	80.72533	165.8088	69.92474	163.3344	74.59117
166.0087	80.72582	165.8516	69.92515	163.3732	74.59069
166.0502	80.72639	165.892	69.92449	163.4121	74.58972
166.0902	80.72556	165.9325	69.92366	163.456	74.58892
166.1335	80.72754	165.9717	69.9238	163.4981	74.58751
166.1732	80.72839	166.0123	69.92308	163.5378	74.58584
166.2152	80.72033	166.0551	69.92317	163.5776	74.58467
166.2592	80.71667	166.0973	69.92353	163.6226	74.58355
166.3004	80.71324	166.1379	69.92247	163.6641	74.58276
166.3418	80.70611	166.1799	69.92126	163.6971	74.58201
166.3858	80.70666	166.2206	69.92115	163.7378	74.58112
166.425	80.70317	166.2555	69.92077	163.7774	74.57986
166.459	80.69916	166.2981	69.92161	163.8217	74.57875
166.499	80.69507	166.3377	69.92161	163.8642	74.57735
166.545	80.69603	166.3814	69.92051	163.9052	74.57586

166.5862	80.6983	166.4228	69.91967	163.9473	74.57462
166.6271	80.69969	166.4637	69.9173	163.9891	74.57329
166.6694	80.70317	166.5041	69.91707	164.0318	74.57245
166.7096	80.70506	166.5465	69.91655	164.0709	74.57162
166.7498	80.70266	166.5892	69.91434	164.1127	74.57104
166.7915	80.69626	166.6307	69.91346	164.1537	74.57032
166.8331	80.69404	166.674	69.91248	164.193	74.56974
166.8741	80.69023	166.7069	69.91171	164.2361	74.56929
166.9164	80.6882	166.7483	69.91134	164.2789	74.56801
166.9606	80.69248	166.787	69.91301	164.3097	74.56741
166.9908	80.69344	166.8308	69.91241	164.3516	74.56618
167.034	80.68637	166.8745	69.91172	164.3949	74.56445
167.0736	80.68423	166.9157	69.91144	164.4377	74.5637
167.1142	80.6822	166.9573	69.90907	164.4775	74.5625
167.1585	80.67861	166.9973	69.90823	164.5226	74.56202
167.1966	80.67882	167.0413	69.90841	164.5641	74.56141
167.236	80.67901	167.0828	69.90752	164.6065	74.56093
167.2805	80.67861	167.1248	69.90735	164.6472	74.56
167.3224	80.67522	167.1559	69.90684	164.6888	74.55886
167.3651	80.67359	167.1969	69.90459	164.7359	74.5574
167.4081	80.67585	167.2388	69.90334	164.7753	74.55587
167.4399	80.67646	167.2839	69.90183	164.8168	74.55473
167.4819	80.67926	167.3236	69.9004	164.8552	74.5533
167.5217	80.68154	167.3666	69.90109	164.8928	74.55259
167.5614	80.6792	167.4074	69.90237	164.933	74.55096
167.6059	80.67527	167.4492	69.90292	164.9751	74.55002
167.6454	80.6695	167.493	69.90408	165.0192	74.5487
167.6872	80.66739	167.5318	69.90285	165.0608	74.54802
167.7307	80.66419	167.5764	69.90024	165.1036	74.54762
167.7746	80.66259	167.6065	69.90012	165.1408	74.547
167.8138	80.66096	167.6473	69.89824	165.1856	74.54606
167.8569	80.658	167.6906	69.89733	165.2261	74.54452
167.893	80.65683	167.7339	69.89742	165.2682	74.54343
167.9348	80.65068	167.775	69.89647	165.3103	74.54152
167.9748	80.64658	167.8145	69.89619	165.3541	74.54041
168.0189	80.6488	167.8574	69.89644	165.3961	74.53951
168.0577	80.65009	167.8989	69.8951	165.4376	74.53848
168.0973	80.64718	167.9418	69.89301	165.4722	74.53789
168.1416	80.64753	167.9832	69.89074	165.5106	74.53728
168.1817	80.64024	168.0237	69.88895	165.5517	74.53661
168.2248	80.63216	168.0555	69.88959	165.5988	74.53545
168.2662	80.63188	168.1002	69.88981	165.6407	74.53396
168.3082	80.63294	168.14	69.89011	165.682	74.53335
168.341	80.6336	168.1841	69.89053	165.7275	74.53252
168.3841	80.63475	168.2248	69.89032	165.7709	74.53083
168.4263	80.63403	168.2658	69.88956	165.8116	74.52966
168.4678	80.6378	168.3084	69.89011	165.8531	74.52789
168.5103	80.64099	168.347	69.88767	165.8953	74.52639
168.5504	80.64083	168.3882	69.88469	165.9378	74.52604
168.5896	80.64175	168.4318	69.88347	165.9817	74.52573
168.6307	80.63453	168.4728	69.88098	166.0237	74.52494
168.6783	80.62823	168.507	69.8806	166.0657	74.52412
168.7163	80.62216	168.547	69.88024	166.096	74.52294
168.7598	80.61961	168.5882	69.87945	166.1406	74.52192
168.8019	80.62122	168.6324	69.87918	166.1834	74.52114
168.846	80.61991	168.6772	69.88041	166.224	74.52047
168.8771	80.62201	168.7158	69.88006	166.2658	74.51987
168.9186	80.61651	168.7568	69.87845	166.3079	74.51842

168.9609	80.60702	168.7999	69.87814	166.3493	74.517
169.002	80.6041	168.842	69.87762	166.3923	74.51523
169.0442	80.60084	168.883	69.87652	166.4328	74.5141
169.0861	80.60356	168.9249	69.87572	166.4763	74.5135
169.1245	80.61053	168.957	69.8744	166.516	74.51301
169.17	80.60798	169.0001	69.87126	166.5632	74.51165
169.2108	80.60334	169.0435	69.87005	166.6058	74.51023
169.2549	80.60262	169.0821	69.86901	166.638	74.50898
169.2944	80.59725	169.1238	69.87011	166.6789	74.50734
169.3299	80.59573	169.1656	69.8698	166.7233	74.50583
169.3701	80.59498	169.208	69.86901	166.7634	74.50476
169.4117	80.59036	169.2471	69.8715	166.8053	74.50376
169.4496	80.58631	169.288	69.86932	166.8449	74.50269
169.4933	80.58205	169.3306	69.86763	166.8885	74.50245
169.5339	80.58121	169.3705	69.86907	166.9303	74.50138
169.5767	80.58213	169.407	69.86772	166.9709	74.50028
169.6205	80.58232	169.4459	69.86615	167.0121	74.49844
169.6637	80.58488	169.4869	69.86748	167.056	74.4968
169.7011	80.58699	169.5289	69.86558	167.0966	74.49556
169.7449	80.58734	169.568	69.86443	167.1354	74.49426
169.7767	80.58811	169.6106	69.86577	167.1797	74.49317
169.8182	80.58359	169.6528	69.86435	167.2246	74.49245
169.8586	80.58058	169.6935	69.86247	167.2579	74.49142
169.9004	80.57869	169.7342	69.86076	167.2973	74.49008
169.9358	80.57795	169.7765	69.85879	167.3359	74.48958
169.9843	80.57956	169.82	69.85821	167.3801	74.48831
170.0246	80.57539	169.8526	69.85878	167.4234	74.48771
170.0662	80.5696	169.8926	69.85759	167.4646	74.48745
170.1086	80.56491	169.9356	69.85725	167.5045	74.48666
170.1493	80.56242	169.9776	69.85679	167.543	74.48528
170.1883	80.56148	170.0187	69.85573	167.5848	74.48358
170.2231	80.56454	170.0588	69.85522	167.6276	74.48166
170.2632	80.56399	170.0997	69.85488	167.6656	74.48012
170.3063	80.56393	170.1419	69.85327	167.7104	74.47933
170.3461	80.56502	170.1847	69.85407	167.7518	74.47873
170.3874	80.56332	170.2258	69.85441	167.7926	74.47826
170.4329	80.56273	170.2684	69.85413	167.8344	74.47747
170.4758	80.56235	170.3023	69.85431	167.8664	74.4768
170.5152	80.56216	170.3448	69.85209	167.9123	74.47576
170.5553	80.55935	170.3844	69.85113	167.9508	74.475
170.5966	80.55254	170.428	69.85012	167.9906	74.47396
170.6405	80.54589	170.4694	69.8492	168.0297	74.4729
170.6842	80.54426	170.51	69.84969	168.076	74.47153
170.7239	80.54605	170.5526	69.85039	168.1171	74.47026
170.7566	80.54993	170.5899	69.84969	168.1572	74.46959
170.8009	80.55174	170.6337	69.85042	168.1968	74.46907
170.8409	80.55204	170.6768	69.84819	168.239	74.46809
170.8827	80.55108	170.7148	69.84485	168.2829	74.46605
170.9276	80.54976	170.7515	69.84494	168.3235	74.46411
170.9696	80.54945	170.7945	69.84378	168.3605	74.46194
171.0092	80.54758	170.8326	69.84255	168.3983	74.46101
171.0517	80.54387	170.8766	69.84284	168.4391	74.45974
171.0922	80.54158	170.919	69.8411	168.479	74.45862
171.1349	80.54091	170.9572	69.83898	168.5224	74.45729
171.1758	80.54192	171.0003	69.8383	168.5646	74.45617
171.2099	80.54106	171.0394	69.83639	168.6083	74.4553
171.2509	80.54145	171.0853	69.83475	168.6488	74.45365
171.2913	80.54119	171.127	69.83429	168.688	74.45249

171.3333	80.53824	171.1694	69.83386	168.7296	74.45089
171.3748	80.53632	171.2008	69.83372	168.7695	74.45023
171.4166	80.53401	171.2414	69.83463	168.8117	74.44959
171.4592	80.53292	171.2866	69.83333	168.8537	74.44922
171.4998	80.53205	171.3291	69.83368	168.8992	74.44863
171.5399	80.53008	171.3677	69.83468	168.9388	74.44713
171.583	80.52824	171.4103	69.8332	168.9809	74.44626
171.623	80.52879	171.4489	69.8332	169.0129	74.44489
171.6577	80.52737	171.4909	69.83354	169.0569	74.44322
171.7013	80.52388	171.5352	69.83179	169.1	74.44214
171.74	80.52275	171.5756	69.83053	169.1408	74.44154
171.7815	80.52025	171.6167	69.82948	169.1811	74.44071
171.8216	80.51933	171.6528	69.82743	169.2224	74.44032
171.8627	80.51914	171.6946	69.82646	169.2647	74.43934
171.9053	80.51932	171.7352	69.82713	169.3079	74.43763
171.9482	80.51716	171.7762	69.82634	169.3465	74.43649
171.9912	80.51612	171.8185	69.8248	169.3918	74.43486
172.0292	80.51719	171.8614	69.82332	169.4322	74.43401
172.0703	80.51824	171.9011	69.82209	169.4737	74.43265
172.1044	80.51863	171.943	69.82251	169.5154	74.43128
172.1482	80.51587	171.9815	69.82297	169.5565	74.43039
172.1887	80.51389	172.0262	69.82265	169.5986	74.42931
172.231	80.51082	172.0685	69.82057	169.6341	74.42859
172.271	80.5081	172.1043	69.81868	169.6719	74.42776
172.3122	80.50562	172.1432	69.81737	169.7122	74.42662
172.3542	80.50517	172.1803	69.81649	169.755	74.42532
172.3939	80.50576	172.2261	69.81691	169.7954	74.42448
172.4368	80.50363	172.2675	69.81614	169.8403	74.42324
172.4788	80.50463	172.3086	69.81452	169.8804	74.42227
172.5212	80.50487	172.3488	69.81124	169.9211	74.42046
172.5639	80.50246	172.3939	69.80963	169.9645	74.41866
172.6042	80.50063	172.4354	69.80903	170.0068	74.41834
172.6377	80.49963	172.4764	69.80849	170.0461	74.41757
172.6803	80.49442	172.518	69.80951	170.0859	74.41722
172.72	80.48996	172.5526	69.80939	170.1284	74.41709
172.7606	80.48561	172.5936	69.80873	170.1619	74.41591
172.7996	80.48183	172.6361	69.80748	170.201	74.41432
172.8422	80.48185	172.6764	69.80701	170.2432	74.41271
172.8848	80.4832	172.7149	69.8058	170.2848	74.41104
172.9261	80.48547	172.7581	69.80517	170.3247	74.40959
172.967	80.48348	172.7974	69.80433	170.3673	74.40811
173.0088	80.47968	172.8406	69.80275	170.406	74.40736
173.0491	80.47583	172.8829	69.80257	170.447	74.40617
173.0882	80.47393	172.9228	69.8026	170.4895	74.40497
173.1295	80.47525	172.9658	69.80216	170.5306	74.40367
173.1702	80.47759	172.9992	69.80273	170.5675	74.40217
173.2123	80.47936	173.044	69.80321	170.6093	74.40053
173.2528	80.47633	173.084	69.80177	170.6512	74.39894
173.2935	80.46809	173.1249	69.80145	170.6923	74.39761
173.3398	80.46339	173.1649	69.7995	170.7305	74.39617
173.3802	80.46089	173.207	69.79689	170.7639	74.39539
173.4255	80.45917	173.2484	69.79512	170.8058	74.39438
173.464	80.45896	173.2888	69.7943	170.843	74.39335
173.5082	80.45735	173.3307	69.7952	170.8871	74.39178
173.5386	80.45749	173.3715	69.79752	170.9295	74.39079
173.5805	80.45663	173.4104	69.79801	170.969	74.38962
173.6259	80.45702	173.4479	69.79754	171.008	74.38858
173.6655	80.45808	173.4885	69.79679	171.0492	74.38772

173.7076	80.45476	173.531	69.79273	171.092	74.38622
173.7495	80.45276	173.5716	69.79003	171.131	74.38478
173.7919	80.455	173.614	69.78857	171.1732	74.38319
173.8332	80.4576	173.6561	69.78661	171.2141	74.38147
173.8745	80.45463	173.6984	69.78569	171.2552	74.38045
173.916	80.45134	173.7392	69.78561	171.2964	74.37961
173.9592	80.44872	173.7813	69.78524	171.3357	74.37882
173.9891	80.44594	173.8246	69.78536	171.3675	74.37879
174.0338	80.44503	173.8654	69.78497	171.4081	74.37744
174.0773	80.44263	173.9004	69.78451	171.4516	74.37628
174.1167	80.44166	173.9407	69.78442	171.4919	74.37576
174.1556	80.44032	173.9817	69.78218	171.5336	74.3741
174.2004	80.43718	174.0215	69.78068	171.575	74.37282
174.241	80.43689	174.0632	69.78051	171.6172	74.37126
174.2837	80.4384	174.1056	69.77902	171.6528	74.36874
174.3235	80.43922	174.1471	69.77857	171.6993	74.36724
174.3658	80.43697	174.1886	69.77947	171.7412	74.36614
174.408	80.43837	174.2341	69.77637	171.7819	74.36474
174.4513	80.43884	174.2736	69.77415	171.8215	74.36337
174.4953	80.4346	174.3151	69.77458	171.8635	74.36238
174.5276	80.43387	174.349	69.77318	171.8996	74.36131
174.5666	80.43366	174.3877	69.77318	171.9405	74.36003
174.6089	80.4284	174.4276	69.77528	171.9822	74.35957
174.6516	80.42247	174.4684	69.77254	172.0235	74.35844
174.6934	80.41912	174.5119	69.77073	172.0646	74.35765
174.7337	80.42016	174.5538	69.77016	172.1074	74.35703
174.7728	80.4235	174.5961	69.76706	172.1518	74.35578
174.8176	80.42265	174.6353	69.76819	172.189	74.35464
174.8611	80.42361	174.6809	69.76785	172.2322	74.35296
174.8999	80.42121	174.7199	69.7662	172.2768	74.35137
174.9416	80.41798	174.7608	69.76606	172.3164	74.34991
174.975	80.41942	174.7941	69.76492	172.3581	74.34879
175.0142	80.42091	174.8346	69.76376	172.4007	74.34757
175.0618	80.42118	174.8762	69.76301	172.4408	74.34648
175.1014	80.41904	174.9216	69.76196	172.483	74.34535
175.1402	80.41503	174.9636	69.76051	172.5176	74.34517
175.1852	80.41443	175.0019	69.7602	172.5612	74.3441
175.2238	80.4084	175.0455	69.76028	172.6035	74.3427
175.2643	80.40591	175.0879	69.76152	172.6453	74.34176
175.305	80.40443	175.1325	69.7624	172.6854	74.34027
175.3484	80.40169	175.1692	69.76092	172.727	74.3393
175.3917	80.40382	175.2127	69.75983	172.7694	74.338
175.4246	80.40136	175.2474	69.75874	172.8103	74.33676
175.4628	80.3987	175.2899	69.75584	172.8495	74.33484
175.5024	80.39551	175.3313	69.75501	172.8919	74.33305
175.546	80.39038	175.3729	69.75444	172.9346	74.33163
175.5861	80.38757	175.4149	69.75319	172.9752	74.33004
175.6281	80.38668	175.4557	69.7551	173.0194	74.3287
175.673	80.38549	175.4956	69.75548	173.057	74.32773
175.7126	80.3864	175.539	69.75353	173.1008	74.32674
175.7556	80.38612	175.5804	69.75227	173.1316	74.32602
175.7963	80.38629	175.6207	69.74895	173.1741	74.32481
175.8399	80.38592	175.6578	69.74697	173.2178	74.32298
175.8706	80.38485	175.6948	69.74824	173.256	74.32179
175.9137	80.38219	175.7387	69.74792	173.2975	74.3204
175.9544	80.3774	175.7794	69.74746	173.338	74.31949
175.994	80.37602	175.8224	69.74725	173.3776	74.3189
176.0375	80.37646	175.8604	69.74504	173.4211	74.31774

176.0794	80.37793	175.9032	69.74314	173.4645	74.31629
176.1195	80.37629	175.9441	69.74212	173.5064	74.31478
176.1605	80.37487	175.9855	69.74038	173.5444	74.31319
176.2018	80.37167	176.0271	69.73806	173.5879	74.31229
176.2458	80.36701	176.0698	69.73868	173.627	74.3113
176.286	80.36734	176.1086	69.73856	173.6629	74.31013
176.3275	80.36538	176.1434	69.73726	173.7045	74.30882
176.3685	80.36466	176.186	69.73875	173.7447	74.30741
176.4034	80.36316	176.226	69.73799	173.7873	74.3061
176.4448	80.36119	176.2686	69.73529	173.8289	74.30496
176.4858	80.3607	176.3088	69.73543	173.871	74.30356
176.5274	80.35994	176.3516	69.73265	173.9111	74.30211
176.5682	80.35645	176.394	69.73149	173.9545	74.30064
176.6136	80.35547	176.4336	69.73067	173.9921	74.29961
176.6558	80.35579	176.4741	69.72847	174.0342	74.29896
176.6972	80.35075	176.5174	69.72922	174.0765	74.29778
176.7384	80.34738	176.5579	69.72904	174.1206	74.29664
176.7784	80.34717	176.594	69.72778	174.1608	74.29519
176.8218	80.34656	176.6337	69.72793	174.2035	74.29366
176.8522	80.34686	176.6757	69.72745	174.2466	74.29229
176.8977	80.34467	176.7165	69.72536	174.2807	74.29125
176.9392	80.34204	176.7582	69.72512	174.3224	74.28993
176.9808	80.33585	176.7987	69.72418	174.3626	74.28813
177.0223	80.3301	176.8424	69.72091	174.4059	74.28669
177.0638	80.32754	176.8855	69.71832	174.4508	74.28614
177.1057	80.32554	176.9231	69.7159	174.4912	74.28496
177.1463	80.32259	176.9656	69.71431	174.5312	74.28448
177.1875	80.31624	177.0097	69.71447	174.5756	74.28368
177.2306	80.32008	177.0401	69.7155	174.6153	74.28196
177.2701	80.32475	177.0818	69.71629	174.6577	74.28066
177.3048	80.32628	177.1284	69.71711	174.6966	74.27939
177.3466	80.32823	177.1678	69.71716	174.742	74.27807
177.3907	80.32989	177.2068	69.71632	174.7851	74.27609
177.4326	80.3287	177.2477	69.71693	174.8254	74.27474
177.47	80.32745	177.2884	69.71493	174.8677	74.27292
177.509	80.32619	177.3349	69.71309	174.903	74.27172
177.556	80.32506	177.3773	69.71232	174.9434	74.27056
177.5942	80.32135	177.4175	69.70932	174.9858	74.26942
177.6353	80.3198	177.4579	69.70824	175.0274	74.2687
177.6773	80.32055	177.492	69.7083	175.069	74.26717
177.7206	80.3194	177.5341	69.70689	175.1115	74.26547
177.7547	80.3189	177.5748	69.70573	175.1539	74.26395
177.7961	80.31456	177.6167	69.70658	175.1945	74.26255
177.8379	80.31466	177.6573	69.7061	175.2358	74.26135
177.8797	80.31182	177.7012	69.70588	175.2804	74.26087
177.9221	80.30586	177.7405	69.70612	175.3229	74.26028
177.9612	80.30545	177.7793	69.70441	175.3646	74.25837
178.0011	80.30239	177.8195	69.70436	175.409	74.2573
178.0414	80.3013	177.862	69.70428	175.4418	74.2566
178.0828	80.30402	177.9078	69.70332	175.4823	74.25551
178.1264	80.30356	177.9394	69.70227	175.5264	74.25507
178.1686	80.30255	177.9824	69.70089	175.5659	74.25404
178.2108	80.30326	178.0243	69.69954	175.6086	74.25237
178.2515	80.30584	178.0666	69.69799	175.6509	74.25026
178.2843	80.3059	178.1067	69.69649	175.6951	74.24774
178.3279	80.30696	178.1496	69.69583	175.7338	74.24597
178.3694	80.30808	178.1899	69.69561	175.7784	74.24456
178.413	80.30631	178.2328	69.69494	175.8179	74.24331

178.4556	80.30175	178.2736	69.69557	175.8578	74.24251
178.4981	80.29609	178.316	69.69484	175.9029	74.2412
178.5388	80.29317	178.3618	69.69312	175.9422	74.23978
178.579	80.29099	178.3921	69.69283	175.9861	74.2386
178.622	80.28995	178.4334	69.69346	176.0287	74.23679
178.661	80.28929	178.4726	69.69328	176.063	74.23551
178.705	80.28929	178.5152	69.69177	176.1035	74.23466
178.7322	80.28686	178.5565	69.69103	176.1447	74.23377
178.7758	80.28151	178.5981	69.68903	176.1875	74.23258
178.8201	80.27863	178.64	69.68711	176.2274	74.23104
178.8608	80.27618	178.6822	69.68709	176.2704	74.22946
178.9027	80.27181	178.7235	69.68518	176.3114	74.22768
178.9445	80.26857	178.7661	69.68253	176.3519	74.22652
178.9849	80.26917	178.8051	69.68216	176.3947	74.22562
179.0266	80.26656	178.8411	69.68131	176.4363	74.22445
179.0684	80.26704	178.8837	69.68097	176.4777	74.22327
179.1091	80.26695	178.9244	69.68225	176.5182	74.22235
179.1506	80.26948	178.9642	69.68082	176.5587	74.22139
179.1845	80.27256	179.0078	69.67932	176.6028	74.21986
179.2294	80.2678	179.0496	69.67858	176.6433	74.21836
179.2664	80.27062	179.0885	69.67782	176.6751	74.21708
179.3101	80.26717	179.1299	69.6776	176.7191	74.21573
179.3504	80.26035	179.171	69.67735	176.7605	74.21392
179.3926	80.25914	179.2142	69.67742	176.8034	74.2129
179.4331	80.25964	179.2573	69.67664	176.8411	74.21176
179.4772	80.25924	179.2897	69.67729	176.8847	74.21077
179.5199	80.25469	179.3324	69.67591	176.9252	74.21042
179.5616	80.25304	179.3769	69.674	176.968	74.20884
179.6022	80.25101	179.4166	69.6713	177.0109	74.20779
179.6346	80.24867	179.4551	69.66698	177.053	74.20604
179.677	80.24987	179.4992	69.66577	177.0935	74.20427
179.7218	80.24905	179.5401	69.66323	177.1361	74.2031
179.7624	80.247	179.5808	69.66258	177.1738	74.20183
179.8036	80.24676	179.6225	69.66287	177.2102	74.20073
179.8453	80.24372	179.6634	69.66356	177.2502	74.19888
179.8883	80.24259	179.7059	69.66332	177.2912	74.19752
179.9279	80.24144	179.7373	69.66241	177.3335	74.19561
179.9691	80.23878	179.7782	69.66143	177.3729	74.19473
180.0137	80.2381	179.8234	69.65864	177.4162	74.19359
180.0538	80.23596	179.8643	69.6583	177.4584	74.1924
180.095	80.22986	179.9057	69.65884	177.4984	74.19139
180.1372	80.22598	179.9472	69.65872	177.5383	74.18993
180.1702	80.225	179.9891	69.66022	177.5792	74.18891
180.2097	80.22468	180.0307	69.65898	177.6203	74.1872
180.2537	80.22882	180.0718	69.65786	177.6652	74.18534
180.2924	80.22816	180.113	69.65728	177.7056	74.18336
180.3367	80.22636	180.156	69.65473	177.7444	74.18197
180.3728	80.22351	180.1912	69.65374	177.7885	74.18113
180.4147	80.2175	180.2306	69.65335	177.8207	74.18065
180.4573	80.21722	180.2724	69.65198	177.8621	74.17991
180.5008	80.21948	180.3152	69.65071	177.9051	74.17871
180.5444	80.21998	180.3561	69.65097	177.9463	74.1774
180.5866	80.22239	180.3938	69.64992	177.986	74.17605
180.6182	80.22521	180.4389	69.6499	178.0269	74.17436
180.6581	80.22581	180.4813	69.64915	178.0716	74.17325
180.7004	80.22173	180.5206	69.64735	178.1123	74.17246
180.7436	80.217	180.5606	69.64673	178.1578	74.17085
180.7841	80.20892	180.6008	69.64491	178.1966	74.16947

180.8242	80.2005	180.6375	69.6435	178.2408	74.16782
180.8691	80.19857	180.6812	69.64237	178.281	74.16577
180.9102	80.20218	180.7227	69.6403	178.3252	74.16395
180.9504	80.2033	180.7636	69.63856	178.3678	74.16338
180.9913	80.20287	180.8048	69.63837	178.4062	74.16277
181.0345	80.20384	180.8468	69.63787	178.4425	74.16189
181.066	80.20235	180.8879	69.63758	178.4822	74.16066
181.1103	80.19924	180.9297	69.6367	178.5241	74.15916
181.1482	80.19646	180.9734	69.63534	178.5686	74.15789
181.1918	80.19415	181.0128	69.63466	178.6076	74.15759
181.2361	80.19048	181.0563	69.63354	178.6494	74.15653
181.2767	80.1892	181.0892	69.63265	178.692	74.15484
181.3166	80.19103	181.1289	69.63199	178.7342	74.1522
181.3596	80.1898	181.17	69.63181	178.7742	74.14973
181.4017	80.18817	181.2123	69.63183	178.817	74.14859
181.4442	80.18597	181.2544	69.63212	178.8596	74.14761
181.4852	80.18383	181.2974	69.63258	178.9006	74.14709
181.5205	80.18358	181.339	69.63149	178.9413	74.1456
181.5625	80.18194	181.38	69.63016	178.9772	74.14414
181.6034	80.1784	181.4198	69.62896	179.0182	74.14238
181.6456	80.17733	181.4614	69.62701	179.0579	74.14037
181.6865	80.17585	181.5051	69.62589	179.1019	74.13911
181.7278	80.17631	181.536	69.626	179.1424	74.13759
181.7696	80.1737	181.5791	69.62646	179.1858	74.13709
181.8113	80.174	181.6192	69.62612	179.2272	74.13671
181.8539	80.17382	181.6609	69.62598	179.2696	74.1356
181.8922	80.1682	181.6986	69.62505	179.3107	74.13464
181.9358	80.17105	181.7394	69.62251	179.3511	74.13298
181.9764	80.16922	181.7866	69.62138	179.394	74.13126
182.0196	80.16712	181.8245	69.61952	179.4342	74.12944
182.0536	80.16681	181.8655	69.61757	179.476	74.12741
182.0942	80.16119	181.9092	69.61779	179.5192	74.12537
182.1337	80.15792	181.9504	69.61756	179.5647	74.12359
182.1747	80.15479	181.9818	69.61886	179.5953	74.12234
182.2206	80.15396	182.0272	69.61967	179.6378	74.12167
182.2624	80.15615	182.0678	69.62061	179.6788	74.1215
182.3011	80.15491	182.111	69.61934	179.7191	74.12067
182.3447	80.15366	182.1508	69.6174	179.7618	74.12004
182.3833	80.14889	182.1924	69.6168	179.8058	74.1186
182.4277	80.14303	182.2367	69.61458	179.8465	74.11757
182.4684	80.14392	182.2762	69.61452	179.8891	74.11791
182.5037	80.14199	182.317	69.61348	179.9305	74.11637
182.5458	80.14366	182.3611	69.61162	179.9722	74.11387
182.5855	80.14915	182.4041	69.61099	180.0144	74.10951
182.6261	80.14745	182.4361	69.60986	180.0562	74.10435
182.6705	80.14795	182.4764	69.60764	180.0995	74.10007
182.7114	80.14529	182.5181	69.60667	180.1414	74.09721
182.7549	80.13865	182.5599	69.6052	180.1841	74.09579
182.793	80.13619	182.6027	69.60324	180.2178	74.0938
182.8338	80.13399	182.6444	69.60242	180.2544	74.09321
182.8775	80.1336	182.6847	69.60155	180.2994	74.09145
182.9195	80.13208	182.7279	69.60108	180.3422	74.09019
182.9497	80.13066	182.7664	69.6012	180.3838	74.08776
182.9937	80.13045	182.8105	69.60168	180.4256	74.08562
183.0355	80.12731	182.8494	69.60099	180.4652	74.08361
183.0784	80.13118	182.8846	69.60002	180.5058	74.08151
183.117	80.13156	182.9258	69.59874	180.5488	74.08034
183.157	80.12687	182.9687	69.59721	180.5886	74.07923

183.1997	80.12645	183.01	69.59637	180.6318	74.07866
183.2418	80.12301	183.0513	69.59573	180.6746	74.07716
183.2812	80.12065	183.0935	69.59506	180.716	74.07624
183.3247	80.12074	183.1332	69.59466	180.752	74.07504
183.3632	80.11665	183.1778	69.5939	180.7907	74.07373
183.3977	80.11634	183.2159	69.59258	180.8337	74.07203
183.4398	80.11654	183.2583	69.59128	180.8751	74.07056
183.4809	80.11404	183.302	69.58952	180.9167	74.0693
183.5246	80.11339	183.3332	69.58723	180.9567	74.068
183.5696	80.11395	183.3762	69.58587	180.9983	74.06756
183.6088	80.11021	183.4168	69.58486	181.0394	74.06648
183.6526	80.1053	183.4601	69.5842	181.0814	74.06531
183.6916	80.10412	183.5026	69.5848	181.1249	74.06337
183.733	80.10069	183.5424	69.58458	181.1668	74.06169
183.7776	80.0993	183.5817	69.58451	181.2062	74.06003
183.8186	80.09751	183.6271	69.58374	181.2497	74.05857
183.8605	80.09588	183.6715	69.58247	181.2911	74.05726
183.9023	80.09127	183.7092	69.58173	181.3318	74.05594
183.933	80.08544	183.7488	69.5805	181.3642	74.05553
183.9754	80.08625	183.7846	69.57988	181.4019	74.05416
184.0149	80.08832	183.8258	69.57906	181.4485	74.05288
184.0572	80.0847	183.867	69.57911	181.4868	74.0522
184.1018	80.08282	183.9067	69.57826	181.5286	74.05145
184.141	80.08247	183.947	69.57695	181.5696	74.05023
184.1794	80.0777	183.9887	69.57626	181.614	74.04916
184.2229	80.08051	184.0312	69.57513	181.6561	74.04728
184.2682	80.08495	184.0738	69.57475	181.6968	74.04513
184.3095	80.08428	184.1144	69.57521	181.7422	74.0434
184.3504	80.08355	184.1563	69.57464	181.7843	74.04153
184.3777	80.08355	184.2014	69.57333	181.8246	74.03966
184.4231	80.08092	184.2353	69.57303	181.8644	74.03775
184.4672	80.07654	184.2765	69.57229	181.9061	74.03658
184.5058	80.07143	184.3151	69.5716	181.9463	74.03557
184.549	80.06921	184.3552	69.57122	181.9824	74.03497
184.5879	80.06497	184.3988	69.57044	182.0225	74.03414
184.6311	80.05999	184.4406	69.5688	182.0675	74.03281
184.6721	80.0618	184.4836	69.56761	182.1084	74.03159
184.7124	80.06317	184.5228	69.56703	182.1483	74.03047
184.7563	80.06471	184.5649	69.56674	182.1911	74.02839
184.7973	80.06554	184.6089	69.56681	182.2296	74.02663
184.8269	80.06469	184.6523	69.56593	182.2704	74.02536
184.8719	80.05905	184.6843	69.56473	182.3116	74.02403
184.9164	80.0523	184.7254	69.56287	182.353	74.02301
184.9552	80.04979	184.768	69.56107	182.3967	74.02173
184.9974	80.04635	184.8082	69.56002	182.4374	74.02003
185.0399	80.04839	184.8509	69.55943	182.4802	74.01794
185.0815	80.0493	184.8936	69.55894	182.5111	74.01659
185.1238	80.05083	184.9314	69.55873	182.5533	74.01494
185.1626	80.05276	184.9755	69.55893	182.5948	74.01407
185.2078	80.04619	185.0136	69.55839	182.6372	74.01221
185.2491	80.04176	185.0588	69.55759	182.6785	74.01102
185.28	80.03984	185.0994	69.55744	182.7201	74.01028
185.3241	80.03833	185.133	69.55649	182.7656	74.00822
185.3668	80.03637	185.1744	69.55524	182.8077	74.00743
185.4065	80.03167	185.2181	69.55463	182.8455	74.00555
185.4491	80.0294	185.2586	69.55342	182.8864	74.00331
185.4921	80.02858	185.3012	69.5523	182.9283	74.00146
185.5308	80.02988	185.3416	69.55167	182.9721	74.00021

185.5745	80.0327	185.3836	69.55042	183.011	73.99923
185.6149	80.03296	185.4249	69.54912	183.0553	73.99812
185.6538	80.03079	185.4669	69.54822	183.0984	73.99705
185.6968	80.02835	185.5078	69.54783	183.129	73.99555
185.7383	80.02963	185.5476	69.54761	183.1681	73.99462
185.7789	80.02971	185.5844	69.54731	183.2098	73.99356
185.813	80.02528	185.6248	69.54693	183.2506	73.99258
185.8544	80.01917	185.667	69.54653	183.2916	73.99096
185.8946	80.01517	185.7085	69.54512	183.3339	73.98953
185.9366	80.0135	185.7476	69.54397	183.377	73.98818
185.979	80.01569	185.7916	69.54264	183.4184	73.98655
186.02	80.0221	185.8329	69.5411	183.4627	73.98578
186.06	80.02274	185.8734	69.54021	183.5014	73.98398
186.1044	80.01878	185.9168	69.53938	183.5452	73.98149
186.1436	80.01355	185.9583	69.53882	183.5853	73.97969
186.1835	80.00885	186.001	69.53825	183.6268	73.97824
186.2258	80.00814	186.0349	69.538	183.6687	73.9774
186.2623	80.01008	186.0761	69.53723	183.7114	73.97653
186.3061	80.00874	186.1171	69.53656	183.7395	73.97567
186.3462	80.00851	186.1563	69.53534	183.7834	73.9738
186.3898	80.00583	186.1973	69.53382	183.8241	73.97201
186.4294	80.00077	186.2385	69.53327	183.8656	73.97055
186.4728	80.00339	186.2809	69.532	183.91	73.96917
186.515	79.9973	186.3247	69.53125	183.9521	73.96801
186.5567	79.99447	186.3647	69.53079	183.9919	73.96631
186.5971	79.99323	186.4059	69.53014	184.036	73.96499
186.6399	79.99235	186.448	69.52956	184.0759	73.96356
186.6806	79.99387	186.4829	69.52948	184.1188	73.96236
186.717	79.99189	186.5226	69.52861	184.1584	73.9606
186.7566	79.99146	186.5638	69.52704	184.1991	73.95962
186.7962	79.98481	186.6034	69.52654	184.2394	73.95866
186.8404	79.9808	186.6452	69.52532	184.2748	73.95733
186.8841	79.98115	186.6877	69.52474	184.3176	73.95622
186.925	79.98526	186.7307	69.52403	184.3592	73.95487
186.9636	79.98961	186.7705	69.52371	184.4017	73.95381
187.0067	79.99002	186.8134	69.52371	184.4433	73.9526
187.0464	79.98946	186.8556	69.52271	184.4837	73.95107
187.0901	79.98335	186.899	69.52145	184.5287	73.94957
187.1316	79.97617	186.9305	69.52043	184.5699	73.94761
187.1647	79.97576	186.972	69.51897	184.6131	73.94638
187.2064	79.97802	187.0139	69.51833	184.6545	73.94509
187.248	79.97762	187.0557	69.51891	184.6951	73.94383
187.2882	79.97605	187.0959	69.51875	184.7379	73.94234
187.3316	79.97707	187.1362	69.51816	184.7793	73.94034
187.3732	79.97055	187.1788	69.51728	184.8187	73.93962
187.4137	79.96404	187.2211	69.51612	184.861	73.93801
187.4542	79.95916	187.2628	69.51535	184.8904	73.93671
187.4978	79.95607	187.3064	69.51448	184.934	73.93542
187.5383	79.95574	187.3464	69.51291	184.9772	73.93336
187.5819	79.95632	187.3791	69.51231	185.0171	73.93193
187.6203	79.95776	187.4205	69.51082	185.0578	73.93093
187.6657	79.95268	187.4625	69.5098	185.0983	73.92974
187.6963	79.94916	187.5054	69.50934	185.1392	73.9286
187.7391	79.94624	187.5466	69.50804	185.181	73.92741
187.7822	79.94508	187.5877	69.50695	185.2256	73.92554
187.8239	79.94523	187.6296	69.50516	185.2663	73.92357
187.8642	79.9435	187.6751	69.50409	185.3084	73.92181
187.9044	79.94024	187.7145	69.50494	185.3498	73.91994

187.9471	79.94001	187.7548	69.50543	185.3907	73.91906
187.9875	79.93972	187.7988	69.50588	185.4305	73.91878
188.0298	79.94209	187.8329	69.50564	185.4752	73.91821
188.0718	79.94359	187.8701	69.50344	185.5091	73.91718
188.1106	79.94235	187.9102	69.50133	185.5502	73.91559
188.1472	79.94368	187.9529	69.50042	185.5926	73.9137
188.1868	79.93834	187.9929	69.50036	185.6352	73.91196
188.2298	79.93538	188.0342	69.49994	185.6756	73.91045
188.2712	79.9358	188.0765	69.49965	185.7168	73.90903
188.3126	79.93464	188.1174	69.49861	185.7589	73.90739
188.3528	79.93216	188.1609	69.49724	185.8011	73.90568
188.3942	79.92716	188.2028	69.49613	185.8422	73.90463
188.4353	79.92622	188.2426	69.49501	185.8835	73.90303
188.478	79.9259	188.2794	69.49383	185.9222	73.90193
188.5185	79.93059	188.3187	69.49366	185.9642	73.90007
188.5602	79.93296	188.3599	69.49383	186.0071	73.89803
188.5923	79.92794	188.4026	69.49273	186.0414	73.897
188.6336	79.92312	188.444	69.49229	186.0833	73.89519
188.6772	79.91747	188.4845	69.49041	186.1236	73.89434
188.7186	79.91306	188.5299	69.48889	186.1668	73.89375
188.7615	79.91365	188.5689	69.4886	186.2075	73.89235
188.8026	79.91193	188.6115	69.48873	186.2501	73.89097
188.8418	79.91001	188.6544	69.48904	186.2899	73.88988
188.8817	79.90925	188.6974	69.48908	186.3308	73.88791
188.9264	79.91004	188.7301	69.48853	186.3763	73.88653
188.9661	79.90938	188.7695	69.48721	186.4164	73.88507
189.011	79.90483	188.8126	69.4855	186.4572	73.88322
189.0407	79.90301	188.8553	69.48382	186.4972	73.88198
189.0816	79.90412	188.8993	69.48245	186.5418	73.88141
189.1254	79.90481	188.9363	69.48122	186.5808	73.88089
189.1666	79.90345	188.9795	69.48083	186.6229	73.87931
189.2086	79.9021	189.0225	69.48097	186.6575	73.87903
189.2486	79.89479	189.0636	69.48136	186.7013	73.87781
189.2901	79.89022	189.1031	69.48117	186.7393	73.87537
189.3317	79.88762	189.1447	69.47986	186.7799	73.87416
189.3739	79.88994	189.1766	69.47939	186.8219	73.87269
189.4129	79.89473	189.2219	69.478	186.8639	73.87062
189.4546	79.89236	189.2625	69.47665	186.9069	73.86915
189.4939	79.89395	189.3049	69.47628	186.948	73.8679
189.5404	79.89479	189.348	69.47474	186.9894	73.86622
189.5731	79.89166	189.3882	69.47421	187.0309	73.86547
189.6156	79.88784	189.4282	69.47355	187.0711	73.86438
189.6569	79.88558	189.4706	69.47249	187.1144	73.86303
189.6992	79.88121	189.5097	69.47224	187.1563	73.86126
189.7384	79.87308	189.5514	69.4709	187.1977	73.85886
189.7798	79.86899	189.5933	69.47019	187.2384	73.85762
189.823	79.86749	189.6287	69.47006	187.2704	73.85624
189.864	79.87047	189.6709	69.46904	187.3114	73.85533
189.9051	79.87346	189.7112	69.46762	187.3545	73.85484
189.9492	79.87352	189.7525	69.4666	187.3965	73.85371
189.9868	79.8712	189.7955	69.46549	187.4356	73.85196
190.0241	79.86524	189.8358	69.46466	187.476	73.85042
190.0631	79.86232	189.8774	69.46489	187.5198	73.84862
190.1048	79.8629	189.9223	69.46502	187.5604	73.84667
190.1446	79.86627	189.9589	69.46476	187.6031	73.84617
190.1888	79.86722	190.002	69.46485	187.645	73.84469
190.2297	79.86459	190.0457	69.46447	187.6854	73.84341
190.2707	79.86103	190.0786	69.4628	187.7267	73.8424

190.3117	79.8554	190.12	69.46236	187.7668	73.84111
190.3546	79.85451	190.1617	69.46083	187.8005	73.84001
190.395	79.8529	190.202	69.45879	187.8462	73.83917
190.437	79.85226	190.2428	69.45815	187.8839	73.83773
190.472	79.8521	190.2834	69.45651	187.9267	73.83597
190.5141	79.85058	190.3268	69.455	187.967	73.83442
190.5532	79.85124	190.3683	69.45417	188.0076	73.83243
190.5954	79.85047	190.4108	69.45343	188.05	73.83168
190.637	79.84977	190.4533	69.45309	188.0905	73.83016
190.678	79.84699	190.4949	69.45297	188.1334	73.8288
190.7184	79.84406	190.5286	69.45214	188.1756	73.82774
190.7581	79.84192	190.5696	69.45107	188.2158	73.82586
190.8004	79.84017	190.6102	69.45016	188.2596	73.82474
190.8459	79.84056	190.6516	69.44842	188.298	73.82346
190.8871	79.84151	190.6929	69.44805	188.3392	73.82215
190.9188	79.84164	190.7322	69.44888	188.3808	73.82097
190.96	79.84104	190.7756	69.44849	188.414	73.81966
191.0053	79.84056	190.8181	69.44854	188.4601	73.81781
191.045	79.83938	190.8607	69.44776	188.503	73.81593
191.0858	79.83471	190.9008	69.44549	188.5428	73.81468
191.1291	79.82954	190.942	69.44499	188.5831	73.8136
191.1724	79.82553	190.9748	69.44395	188.6231	73.81319
191.2146	79.8186	191.017	69.44171	188.6654	73.81266
191.2552	79.81798	191.0589	69.4406	188.7094	73.81134
191.2951	79.82175	191.1	69.43821	188.7512	73.80954
191.3384	79.82071	191.1395	69.4367	188.7914	73.8074
191.3811	79.8178	191.1823	69.43612	188.8335	73.80549
191.4216	79.81734	191.225	69.43646	188.8746	73.80413
191.4518	79.8171	191.2639	69.43623	188.9146	73.80301
191.4961	79.81455	191.3051	69.43631	188.9597	73.80152
191.5391	79.81428	191.3499	69.43643	189.0009	73.80001
191.5814	79.81727	191.3924	69.43371	189.0313	73.79891
191.6206	79.81846	191.4236	69.43329	189.0739	73.79704
191.6621	79.81797	191.4655	69.43294	189.1128	73.79593
191.7048	79.81715	191.5081	69.43263	189.1556	73.79505
191.7416	79.81013	191.5472	69.43242	189.1977	73.7937
191.7877	79.80354	191.5892	69.43251	189.2411	73.79282
191.8301	79.79976	191.6316	69.43196	189.2789	73.79131
191.8716	79.79984	191.6724	69.43112	189.322	73.7892
191.9064	79.80255	191.7149	69.43053	189.3622	73.78784
191.945	79.8039	191.7548	69.42852	189.404	73.78628
191.9886	79.80133	191.797	69.42755	189.4452	73.78528
192.0316	79.80218	191.8378	69.42806	189.4854	73.78425
192.0734	79.80076	191.8715	69.4277	189.527	73.78305
192.1141	79.79384	191.9151	69.4279	189.5608	73.78198
192.1577	79.79329	191.9534	69.42757	189.6046	73.781
192.1978	79.7914	191.995	69.42642	189.6425	73.78032
192.2398	79.78726	192.0392	69.426	189.6852	73.77906
192.2803	79.7855	192.0816	69.42426	189.7277	73.77792
192.325	79.78406	192.1238	69.42256	189.7681	73.77658
192.3552	79.78443	192.1659	69.42089	189.8127	73.77516
192.3974	79.78456	192.206	69.41945	189.8524	73.77322
192.4383	79.78477	192.2461	69.42028	189.8944	73.77209
192.4793	79.78282	192.2895	69.42082	189.9334	73.77023
192.5214	79.77958	192.3233	69.42043	189.9756	73.76883
192.5636	79.7768	192.363	69.41945	190.0174	73.76823
192.6052	79.77595	192.4092	69.41908	190.063	73.76713
192.6482	79.77395	192.4468	69.41833	190.1042	73.7665

192.6924	79.77056	192.4926	69.41697	190.1434	73.76532
192.7329	79.76855	192.5319	69.41767	190.1786	73.76377
192.772	79.76553	192.5756	69.41658	190.2187	73.7622
192.8024	79.76404	192.6156	69.41404	190.2599	73.76108
192.8472	79.76369	192.6588	69.41298	190.3002	73.75928
192.8875	79.7626	192.7001	69.41015	190.3396	73.75838
192.9285	79.76228	192.7398	69.40879	190.3822	73.75687
192.9708	79.76163	192.7728	69.40943	190.4231	73.75533
193.0141	79.76222	192.8121	69.40883	190.4662	73.75388
193.0552	79.76241	192.8536	69.40886	190.5068	73.75265
193.0975	79.76237	192.8988	69.40935	190.5497	73.75129
193.1388	79.75867	192.9395	69.40794	190.5929	73.7498
193.1803	79.75361	192.9804	69.40764	190.6338	73.74839
193.2197	79.74791	193.018	69.40789	190.6756	73.74676
193.2612	79.74549	193.0649	69.40584	190.7163	73.74596
193.3059	79.74366	193.108	69.40407	190.7549	73.74476
193.3396	79.74472	193.1498	69.40296	190.7914	73.74409
193.3798	79.74705	193.1914	69.40211	190.8324	73.74247
193.4228	79.74165	193.223	69.40175	190.8754	73.7409
193.4635	79.73863	193.2659	69.40146	190.9156	73.7393
193.5058	79.73802	193.3087	69.40079	190.9557	73.73792
193.5486	79.73659	193.3464	69.39812	191.0011	73.73737
193.5912	79.73632	193.3902	69.3967	191.042	73.73589
193.6314	79.73751	193.4306	69.39625	191.0846	73.73505
193.6711	79.73776	193.4685	69.39615	191.1254	73.73408
193.7153	79.73575	193.5136	69.39724	191.1644	73.73323
193.7534	79.73515	193.5546	69.39644	191.2076	73.73225
193.7862	79.73383	193.5955	69.39598	191.2502	73.73048
193.8299	79.72818	193.6381	69.3962	191.2892	73.72862
193.8729	79.72094	193.6723	69.39448	191.3221	73.72674
193.9156	79.71737	193.7154	69.39344	191.3662	73.725
193.9561	79.71831	193.7564	69.39354	191.4088	73.72412
193.9989	79.71759	193.7979	69.39223	191.451	73.72299
194.0372	79.71557	193.8392	69.39192	191.4931	73.72163
194.079	79.71691	193.8792	69.39153	191.5319	73.72006
194.1222	79.7187	193.9205	69.38949	191.5752	73.71848
194.1619	79.71716	193.9616	69.388	191.617	73.71688
194.2033	79.71504	194.005	69.38713	191.6579	73.71567
194.2377	79.71342	194.0456	69.38591	191.6995	73.71476
194.2773	79.70796	194.088	69.38541	191.7429	73.71347
194.3174	79.70663	194.123	69.38417	191.7861	73.71301
194.3603	79.70592	194.1637	69.38209	191.8228	73.71217
194.4016	79.70246	194.2051	69.38098	191.8645	73.71141
194.4448	79.69939	194.2476	69.38018	191.9048	73.71039
194.4869	79.69943	194.2854	69.38008	191.938	73.70862
194.5274	79.70128	194.3285	69.38066	191.9826	73.70676
194.5688	79.70663	194.3697	69.3802	192.0246	73.70572
194.6103	79.70898	194.4125	69.37898	192.0666	73.70445
194.6522	79.70508	194.4539	69.37896	192.109	73.70376
194.6844	79.70322	194.4942	69.37788	192.1474	73.7034
194.7246	79.7019	194.5348	69.37672	192.1896	73.70192
194.768	79.69912	194.5678	69.37708	192.2308	73.70026
194.8109	79.69985	194.6118	69.37673	192.2725	73.69851
194.8501	79.70094	194.6523	69.3762	192.3161	73.69654
194.8948	79.69929	194.6935	69.37771	192.3578	73.6949
194.9366	79.69609	194.736	69.37733	192.3984	73.69414
194.9772	79.69203	194.7781	69.37679	192.4403	73.69314
195.0191	79.68905	194.8183	69.37574	192.4824	73.69235

195.0612	79.68633	194.8604	69.37416	192.5243	73.69143
195.1021	79.68522	194.9027	69.37202	192.5566	73.69027
195.1428	79.68329	194.9451	69.37013	192.6016	73.68867
195.1848	79.6797	194.9834	69.36928	192.6397	73.68722
195.2167	79.67753	195.0184	69.36838	192.6829	73.68541
195.2578	79.67896	195.0651	69.36846	192.7248	73.6837
195.2978	79.67963	195.1058	69.36776	192.7647	73.68327
195.3393	79.67713	195.1467	69.36695	192.805	73.68204
195.383	79.67547	195.1852	69.36634	192.8498	73.68109
195.4247	79.66846	195.2264	69.36578	192.8952	73.68066
195.4672	79.66355	195.2687	69.36643	192.9316	73.67899
195.5096	79.6596	195.3124	69.36818	192.9738	73.67743
195.5476	79.65994	195.3544	69.36711	193.0166	73.67605
195.5923	79.66506	195.395	69.36656	193.0562	73.67439
195.6322	79.66557	195.4353	69.3658	193.0861	73.6729
195.6651	79.66733	195.4724	69.36305	193.1281	73.6714
195.7075	79.66765	195.5131	69.36205	193.171	73.66993
195.75	79.66379	195.5531	69.36112	193.2102	73.66928
195.7922	79.65999	195.594	69.35871	193.255	73.66874
195.8343	79.657	195.6365	69.35736	193.2968	73.66825
195.8753	79.65392	195.6776	69.35629	193.338	73.66784
195.9159	79.6546	195.7201	69.35483	193.3792	73.66634
195.9585	79.65375	195.7629	69.3551	193.4205	73.66451
195.9992	79.65499	195.8032	69.35498	193.4621	73.66372
196.0409	79.65597	195.8444	69.35457	193.5038	73.66202
196.0822	79.65047	195.8836	69.35595	193.5451	73.66021
196.1137	79.64747	195.9181	69.35534	193.5848	73.65932
196.1572	79.64631	195.9581	69.35367	193.6294	73.65793
196.1986	79.64537	195.9992	69.35366	193.6705	73.65734
196.2404	79.64108	196.0398	69.35169	193.7037	73.6568
196.2834	79.63727	196.0877	69.35016	193.7453	73.65635
196.3228	79.63867	196.128	69.35059	193.7866	73.65495
196.3662	79.63375	196.1695	69.35009	193.8288	73.65286
196.4082	79.62943	196.2131	69.34862	193.8695	73.65088
196.4494	79.63134	196.2549	69.34844	193.9108	73.64829
196.4905	79.62938	196.2951	69.34626	193.9524	73.64669
196.5317	79.62886	196.3388	69.34399	193.9957	73.64509
196.5646	79.63001	196.3724	69.34378	194.0352	73.64405
196.6075	79.63091	196.4139	69.34265	194.0749	73.64371
196.6499	79.62825	196.4528	69.34259	194.1205	73.64281
196.6908	79.62358	196.4939	69.34366	194.1617	73.64201
196.7328	79.61992	196.5334	69.34382	194.2022	73.64087
196.7712	79.61716	196.5804	69.34367	194.2444	73.63897
196.8159	79.61746	196.6224	69.34297	194.286	73.63751
196.8564	79.61718	196.6614	69.34305	194.3205	73.63664
196.8989	79.61697	196.7013	69.34239	194.3636	73.63511
196.9395	79.61888	196.744	69.34228	194.4022	73.63391
196.9802	79.61744	196.7853	69.34133	194.4449	73.63274
197.0226	79.61488	196.8173	69.33832	194.4857	73.63149
197.0631	79.61469	196.8587	69.33629	194.5268	73.63049
197.0983	79.61266	196.9036	69.33465	194.5706	73.62927
197.1403	79.60821	196.9431	69.33324	194.6106	73.62773
197.1831	79.60423	196.9886	69.33375	194.6518	73.62653
197.2212	79.6024	197.0291	69.33288	194.6915	73.62532
197.2639	79.59699	197.0713	69.33258	194.7345	73.62453
197.3058	79.59397	197.1119	69.33314	194.7766	73.62333
197.3477	79.59599	197.1516	69.33221	194.8166	73.62177
197.388	79.59219	197.1951	69.33249	194.8484	73.62097

197.4329	79.58788	197.2373	69.33184	194.8894	73.62025
197.4738	79.58343	197.2724	69.33043	194.9291	73.61895
197.5136	79.57623	197.3142	69.32939	194.9734	73.61785
197.5484	79.57749	197.3511	69.32743	195.0145	73.61682
197.5897	79.58323	197.3938	69.32617	195.0514	73.61536
197.6316	79.58457	197.4345	69.32479	195.0944	73.61495
197.6742	79.58333	197.4756	69.32315	195.1367	73.61495
197.7147	79.57783	197.518	69.32212	195.1789	73.61453
197.7555	79.56896	197.5591	69.32189	195.2239	73.61336
197.7968	79.56561	197.6028	69.32113	195.2666	73.61194
197.8365	79.56793	197.6436	69.32059	195.3066	73.60952
197.8784	79.56529	197.686	69.32131	195.3474	73.60772
197.9163	79.56529	197.7196	69.32165	195.3904	73.60628
197.9589	79.56407	197.7609	69.32203	195.4297	73.60472
197.993	79.5616	197.8011	69.32097	195.4617	73.60403
198.0356	79.5623	197.8397	69.31852	195.5079	73.60241
198.0759	79.56008	197.881	69.31574	195.5492	73.60106
198.117	79.56299	197.9238	69.31499	195.59	73.59996
198.1586	79.56458	197.9657	69.31608	195.6311	73.59928
198.2037	79.56054	198.0092	69.31667	195.6745	73.59784
198.2438	79.56307	198.0487	69.3153	195.7163	73.59683
198.2832	79.55853	198.09	69.31235	195.756	73.59542
198.3237	79.55016	198.1325	69.31021	195.7944	73.59443
198.3666	79.55027	198.1667	69.30964	195.838	73.59354
198.4111	79.54652	198.2076	69.3102	195.8828	73.59242
198.4427	79.54266	198.2498	69.31058	195.9225	73.59157
198.4847	79.54169	198.294	69.31158	195.9625	73.58979
198.5267	79.54595	198.3303	69.31086	196.005	73.58856
198.5691	79.54841	198.3722	69.30925	196.0475	73.58719
198.6105	79.54787	198.414	69.30873	196.0786	73.58646
198.6522	79.54987	198.4592	69.3069	196.1193	73.58496
198.694	79.54575	198.4978	69.30609	196.1634	73.58367
198.734	79.54283	198.5384	69.30628	196.2043	73.58262
198.7767	79.54476	198.5801	69.30641	196.2437	73.5812
198.8203	79.54324	198.6153	69.3071	196.2876	73.58044
198.862	79.54441	198.6603	69.30724	196.3301	73.5796
198.9038	79.54413	198.7023	69.30652	196.3716	73.57797
198.9445	79.53746	198.7406	69.3049	196.4119	73.57687
198.9746	79.53624	198.7815	69.30342	196.4558	73.57523
199.0192	79.53186	198.8252	69.30155	196.4997	73.57431
199.0632	79.52778	198.8674	69.30065	196.5391	73.57418
199.1008	79.52806	198.9062	69.30068	196.5812	73.57335
199.1434	79.52622	198.9482	69.29892	196.612	73.57258
199.1851	79.52693	198.9924	69.2978	196.6571	73.57112
199.2288	79.52466	199.0326	69.29744	196.6978	73.57054
199.2697	79.52463	199.0614	69.29564	196.7401	73.56913
199.3092	79.52436	199.106	69.29466	196.782	73.56738
199.3526	79.5195	199.1498	69.29473	196.8212	73.5656
199.3931	79.51638	199.1878	69.29307	196.8642	73.56362
199.4279	79.51141	199.231	69.29294	196.9079	73.56215
199.4696	79.51099	199.273	69.29205	196.9455	73.56183
199.5112	79.50893	199.3151	69.2915	196.9892	73.56161
199.554	79.50489	199.3556	69.29089	197.0276	73.56106
199.5943	79.50555	199.3978	69.29075	197.0735	73.56058
199.637	79.5028	199.4367	69.29109	197.1176	73.55863
199.6797	79.50712	199.4816	69.29012	197.1537	73.55708
199.7225	79.50887	199.5148	69.28931	197.1956	73.55523
199.7638	79.51295	199.5555	69.28793	197.2269	73.55356

199.8064	79.50785	199.5968	69.28621	197.2705	73.55218
199.8467	79.50076	199.6368	69.28559	197.3115	73.55209
199.8799	79.50083	199.681	69.28495	197.3528	73.55065
199.9198	79.49701	199.7234	69.2843	197.3957	73.54992
199.9628	79.49596	199.767	69.28419	197.4365	73.54961
200.0026	79.49607	199.8079	69.28466	197.4811	73.54797
200.0452	79.49266	199.8472	69.28468	197.5212	73.54766
200.0851	79.4847	199.8903	69.28276	197.5616	73.54726
200.1269	79.48015	199.9314	69.28134	197.6022	73.54545
200.1706	79.47908	199.9631	69.28008	197.6439	73.54397
200.2109	79.48081	200.0038	69.27823	197.6872	73.54188
200.2522	79.4865	200.0434	69.27711	197.7284	73.53962
200.2932	79.48561	200.0871	69.27696	197.7706	73.5386
200.3281	79.48493	200.1304	69.27673	197.8102	73.53727
200.3682	79.4812	200.1711	69.27567	197.8462	73.53635
200.4092	79.47847	200.2048	69.27671	197.888	73.53541
200.4514	79.48535	200.252	69.27576	197.9282	73.53435
200.4924	79.48648	200.2924	69.27309	197.9697	73.53359
200.5364	79.48708	200.3336	69.27253	198.0117	73.53272
200.5783	79.48148	200.3751	69.27033	198.0522	73.53158
200.6203	79.47574	200.41	69.27119	198.0956	73.53011
200.6606	79.47418	200.4501	69.27299	198.1342	73.52939
200.7033	79.47059	200.4938	69.27311	198.1766	73.52874
200.7477	79.47544	200.5353	69.27274	198.2172	73.52748
200.7885	79.47375	200.576	69.27092	198.26	73.52713
200.8335	79.47044	200.6158	69.26938	198.3003	73.52534
200.8622	79.46674	200.6588	69.26875	198.3424	73.52373
200.9058	79.46094	200.6996	69.26796	198.3787	73.52283
200.9456	79.46072	200.7389	69.2675	198.4184	73.52182
200.9876	79.45663	200.7836	69.26638	198.4605	73.521
201.0286	79.46016	200.8267	69.26681	198.5013	73.51969
201.0708	79.46087	200.8591	69.26722	198.543	73.51903
201.1124	79.45893	200.8986	69.26573	198.5819	73.51826
201.1572	79.455	200.9409	69.26537	198.6263	73.51728
201.1983	79.44872	200.9825	69.26241	198.6685	73.51619
201.2362	79.44845	201.0226	69.26181	198.712	73.51528
201.2797	79.44648	201.067	69.26193	198.7496	73.51396
201.3132	79.44796	201.1111	69.26042	198.793	73.51249
201.3558	79.45211	201.1519	69.26079	198.8347	73.51118
201.3967	79.44767	201.1944	69.25988	198.8726	73.50991
201.4413	79.4392	201.2369	69.25945	198.9174	73.50873
201.4839	79.43305	201.2766	69.26069	198.9566	73.50764
201.5228	79.43027	201.313	69.26062	198.9912	73.50703
201.5656	79.43126	201.3531	69.25976	199.0333	73.5056
201.6077	79.43322	201.3932	69.25745	199.0763	73.50401
201.65	79.43288	201.4333	69.25664	199.1162	73.50263
201.691	79.43348	201.4811	69.25475	199.1578	73.5015
201.7314	79.4335	201.5228	69.25467	199.1987	73.50012
201.7651	79.42884	201.5626	69.25572	199.2408	73.49874
201.8064	79.42446	201.6025	69.25462	199.2832	73.49761
201.8444	79.42052	201.6468	69.25472	199.3272	73.49614
201.8859	79.41742	201.684	69.25346	199.3654	73.49573
201.9277	79.41768	201.7274	69.25233	199.4075	73.49447
201.9729	79.42094	201.76	69.25231	199.4473	73.4931
202.0125	79.42362	201.8049	69.25164	199.4906	73.49146
202.0552	79.41615	201.8463	69.25078	199.5312	73.4891
202.0952	79.41563	201.8861	69.25244	199.5734	73.48776
202.1381	79.41451	201.9262	69.25153	199.608	73.4871

202.179	79.41299	201.9711	69.25133	199.6465	73.48743
202.2106	79.41812	202.0123	69.25134	199.6903	73.48675
202.2519	79.41816	202.055	69.24865	199.7329	73.4858
202.2946	79.41778	202.0958	69.24777	199.7712	73.48499
202.3368	79.41831	202.1387	69.24692	199.811	73.48347
202.3784	79.41248	202.1803	69.24548	199.8517	73.48313
202.4186	79.41529	202.2129	69.2444	199.8919	73.48271
202.4616	79.41245	202.2547	69.2437	199.9362	73.4816
202.5019	79.40644	202.3	69.24193	199.9767	73.48072
202.542	79.40762	202.3401	69.24091	200.0201	73.47894
202.5836	79.40389	202.3819	69.24017	200.0594	73.47768
202.6273	79.40598	202.4209	69.23848	200.102	73.47725
202.6666	79.40612	202.4638	69.23772	200.1341	73.47595
202.7088	79.4007	202.5021	69.23658	200.1787	73.47467
202.7424	79.3976	202.5433	69.2358	200.2191	73.47343
202.7826	79.39098	202.5858	69.236	200.2634	73.47145
202.8239	79.38537	202.6256	69.23551	200.3009	73.47036
202.8681	79.38863	202.6601	69.23484	200.3411	73.47004
202.9078	79.38771	202.7029	69.23511	200.3844	73.46929
202.9522	79.39301	202.7436	69.233	200.4264	73.46913
202.9932	79.39221	202.7827	69.23133	200.4656	73.46831
203.0316	79.38941	202.8244	69.23139	200.5104	73.46742
203.0744	79.39005	202.8676	69.23073	200.5528	73.4666
203.1192	79.3811	202.9103	69.2314	200.5928	73.46493
203.1602	79.37747	202.9534	69.23182	200.6365	73.46341
203.1917	79.37515	202.9923	69.2304	200.6786	73.46225
203.2332	79.36865	203.0341	69.22746	200.7217	73.46156
203.2746	79.37219	203.0746	69.22621	200.7538	73.46042
203.3196	79.37252	203.1088	69.22486	200.7967	73.45936
203.3603	79.37049	203.1516	69.22327	200.8386	73.45837
203.4016	79.36599	203.1925	69.22337	200.8775	73.45683
203.4432	79.35684	203.2317	69.22275	200.9205	73.45589
203.4835	79.35877	203.2722	69.22192	200.9618	73.45517
203.5254	79.35534	203.3145	69.22085	201.0031	73.45363
203.5647	79.35971	203.3566	69.2201	201.0454	73.45133
203.6064	79.36701	203.398	69.21864	201.0867	73.44991
203.642	79.36308	203.4391	69.21837	201.1286	73.44896
203.6842	79.36161	203.4822	69.21816	201.1692	73.44857
203.7256	79.35923	203.5263	69.21751	201.2096	73.44803
203.7673	79.35294	203.5574	69.21816	201.2541	73.44713
203.8096	79.34957	203.5994	69.21758	201.292	73.44572
203.8514	79.34371	203.641	69.21671	201.3374	73.44416
203.8913	79.34036	203.6834	69.2168	201.3695	73.44374
203.9329	79.33906	203.7218	69.21508	201.4131	73.44253
203.975	79.33819	203.7666	69.21368	201.4526	73.44138
204.0154	79.34564	203.8064	69.21279	201.495	73.44115
204.0598	79.34919	203.846	69.21066	201.5328	73.44081
204.09	79.34801	203.8862	69.21076	201.575	73.43993
204.1308	79.34516	203.9313	69.21063	201.6202	73.43894
204.1734	79.33632	203.9713	69.21001	201.66	73.4376
204.2165	79.33371	204.0054	69.21073	201.7002	73.43542
204.2593	79.33377	204.0457	69.21126	201.7421	73.43454
204.2981	79.33228	204.0888	69.20926	201.785	73.43396
204.3406	79.33691	204.1302	69.20785	201.8256	73.43245
204.3822	79.33302	204.1699	69.20591	201.8673	73.43204
204.4224	79.3281	204.2114	69.20407	201.8994	73.43164
204.4629	79.32489	204.2544	69.2044	201.941	73.43039
204.5058	79.32333	204.2982	69.20467	201.9845	73.42962

204.5459	79.32199	204.3372	69.20403	202.0262	73.42822
204.585	79.3151	204.3806	69.20334	202.0678	73.42681
204.6185	79.31367	204.4223	69.20207	202.1092	73.42549
204.6621	79.31843	204.4543	69.2009	202.1488	73.42357
204.7061	79.32104	204.4966	69.20168	202.1912	73.42314
204.7472	79.32668	204.5386	69.20111	202.2357	73.42294
204.7884	79.32794	204.5788	69.20071	202.2754	73.42122
204.8258	79.32094	204.6204	69.20048	202.3178	73.42015
204.8697	79.31513	204.6654	69.19948	202.3627	73.41835
204.9155	79.30963	204.7055	69.19898	202.4	73.41636
204.9549	79.3094	204.7492	69.1988	202.4458	73.41533
204.9969	79.30727	204.789	69.19911	202.4858	73.41406
205.0383	79.30595	204.8303	69.19814	202.5166	73.41341
205.0729	79.30432	204.8696	69.1966	202.5606	73.41233
205.1137	79.29845	204.906	69.19465	202.6008	73.41088
205.1558	79.29879	204.9446	69.19258	202.6434	73.41012
205.1968	79.29743	204.9891	69.18997	202.6844	73.40879
205.2393	79.29393	205.0281	69.1895	202.7272	73.40764
205.2804	79.29607	205.07	69.18942	202.7684	73.407
205.3211	79.29037	205.1136	69.18984	202.8116	73.40661
205.3612	79.28676	205.1539	69.18914	202.8506	73.40641
205.4054	79.29116	205.1961	69.1895	202.8938	73.40557
205.4456	79.29328	205.2395	69.19005	202.9359	73.40441
205.4902	79.29605	205.2789	69.18815	202.9766	73.40372
205.5201	79.29136	205.3216	69.18809	203.0204	73.40243
205.5626	79.28426	205.3573	69.18643	203.0622	73.40133
205.6044	79.27965	205.3944	69.18449	203.1024	73.40012
205.6474	79.27424	205.4402	69.185	203.1362	73.39884
205.6876	79.27643	205.4802	69.18505	203.178	73.39718
205.7273	79.27575	205.5219	69.18429	203.2205	73.39546
205.769	79.27204	205.564	69.185	203.2596	73.3943
205.8144	79.26974	205.6034	69.18394	203.3009	73.39381
205.8499	79.26489	205.6456	69.18251	203.3432	73.39294
205.8936	79.26785	205.6864	69.18246	203.3879	73.39205
205.9358	79.26767	205.7303	69.18015	203.4285	73.39104
205.967	79.26806	205.77	69.17917	203.4685	73.38945
206.0071	79.27011	205.804	69.179	203.5106	73.38835
206.0521	79.27052	205.8466	69.17806	203.5512	73.38727
206.092	79.27368	205.8901	69.17626	203.5941	73.38692
206.134	79.27083	205.9297	69.17399	203.6352	73.38614
206.1777	79.26511	205.9705	69.17169	203.668	73.38475
206.2196	79.25704	206.0099	69.17067	203.709	73.38368
206.2613	79.24881	206.0537	69.17096	203.7504	73.38231
206.3041	79.24612	206.0942	69.17112	203.7909	73.38106
206.3451	79.25059	206.1384	69.17101	203.8348	73.38007
206.3831	79.25714	206.1796	69.17036	203.8756	73.37929
206.427	79.25652	206.2221	69.16906	203.9164	73.37833
206.468	79.25537	206.2518	69.16762	203.9599	73.37727
206.499	79.25304	206.294	69.16643	203.9977	73.37643
206.5426	79.24526	206.3331	69.16549	204.043	73.37575
206.5849	79.24126	206.3769	69.16445	204.0825	73.37513
206.627	79.2416	206.4186	69.16389	204.1248	73.3743
206.668	79.24278	206.4605	69.16329	204.1683	73.3735
206.7091	79.24574	206.5019	69.16212	204.2072	73.37247
206.751	79.24377	206.5455	69.16111	204.2487	73.37096
206.7924	79.24126	206.5862	69.16033	204.2816	73.36956
206.8341	79.23496	206.6293	69.15928	204.3268	73.36791
206.8748	79.22807	206.6676	69.1582	204.3645	73.36647

206.9198	79.23046	206.6994	69.15682	204.408	73.36541
206.9531	79.23059	206.7438	69.15586	204.4494	73.36463
206.9952	79.22795	206.7854	69.15523	204.4904	73.36412
207.0401	79.22808	206.827	69.15468	204.533	73.3633
207.0779	79.22257	206.8665	69.15474	204.5744	73.36221
207.1166	79.22032	206.9072	69.1541	204.6146	73.36121
207.1594	79.21872	206.9498	69.15325	204.6585	73.36108
207.2009	79.21918	206.9932	69.15235	204.6963	73.36012
207.2453	79.2205	207.0356	69.15066	204.738	73.35907
207.2836	79.21735	207.0734	69.15023	204.7814	73.35802
207.3277	79.2155	207.1165	69.1503	204.8209	73.35642
207.3684	79.21179	207.1488	69.14867	204.8611	73.3554
207.3987	79.21122	207.19	69.15038	204.8944	73.35465
207.4424	79.2097	207.2323	69.15147	204.9344	73.3533
207.4845	79.2143	207.2745	69.15038	204.9782	73.35172
207.5274	79.21806	207.3142	69.15026	205.0207	73.34967
207.5694	79.2139	207.3599	69.14765	205.0614	73.34766
207.6094	79.21374	207.4022	69.14421	205.1024	73.34677
207.648	79.20831	207.4436	69.14261	205.1457	73.34626
207.6931	79.20439	207.4844	69.14131	205.1892	73.3458
207.7355	79.20373	207.5256	69.1404	205.2271	73.34536
207.7773	79.2006	207.5687	69.1401	205.2682	73.34444
207.8197	79.20149	207.6024	69.1391	205.3086	73.34304
207.8532	79.19968	207.6446	69.13871	205.3539	73.3421
207.8921	79.19483	207.6854	69.13848	205.3935	73.34146
207.9331	79.19305	207.7258	69.13762	205.428	73.3406
207.9738	79.18907	207.7698	69.13694	205.4714	73.34073
208.0172	79.19073	207.8092	69.13765	205.5136	73.34013
208.0594	79.19238	207.8515	69.13693	205.5525	73.33924
208.0997	79.19557	207.892	69.13665	205.5972	73.33891
208.1422	79.19653	207.937	69.13794	205.6392	73.3374
208.1839	79.18807	207.9742	69.13638	205.6802	73.33576
208.224	79.18368	208.0176	69.13603	205.7236	73.3351
208.2665	79.17699	208.0504	69.13551	205.7645	73.33353
208.3066	79.17433	208.0887	69.13296	205.8068	73.33217
208.3474	79.17338	208.1333	69.13167	205.8484	73.33154
208.3802	79.17433	208.1742	69.13057	205.8877	73.33031
208.4239	79.17467	208.2172	69.12847	205.9295	73.32939
208.4664	79.17266	208.2595	69.1287	205.9712	73.32815
208.5059	79.16969	208.3002	69.12833	206.0149	73.32637
208.5494	79.16544	208.3411	69.12727	206.0453	73.32469
208.5906	79.16508	208.382	69.12799	206.0877	73.32308
208.6344	79.15777	208.4219	69.12649	206.1297	73.32118
208.6759	79.15794	208.4631	69.12494	206.1734	73.32074
208.7181	79.16316	208.4979	69.12464	206.2127	73.32039
208.7582	79.16551	208.539	69.12497	206.2572	73.31982
208.8006	79.17233	208.5798	69.12439	206.2939	73.32018
208.8323	79.16988	208.6216	69.12445	206.3397	73.31885
208.8719	79.16789	208.6627	69.12321	206.3801	73.31786
208.916	79.16541	208.7051	69.12163	206.4242	73.31616
208.958	79.15924	208.7457	69.12076	206.4597	73.31395
208.9971	79.15888	208.7869	69.12001	206.5029	73.31305
209.0392	79.15481	208.8282	69.12017	206.5488	73.31224
209.0807	79.1531	208.8702	69.11987	206.5898	73.3118
209.1234	79.15257	208.9125	69.11884	206.6321	73.31125
209.1639	79.14949	208.947	69.11726	206.663	73.31037
209.2057	79.14561	208.9851	69.11629	206.7065	73.30831
209.248	79.14223	209.0272	69.11519	206.7446	73.30695

209.279	79.14076	209.0693	69.11449	206.788	73.30655
209.3231	79.13977	209.1124	69.11444	206.8288	73.30621
209.3659	79.13814	209.1539	69.11279	206.8696	73.30584
209.405	79.13673	209.1922	69.11061	206.9122	73.30461
209.448	79.1326	209.2347	69.10928	206.9541	73.30303
209.487	79.13518	209.278	69.10864	206.999	73.30166
209.5316	79.1376	209.3209	69.1087	207.0377	73.30035
209.5729	79.13636	209.3613	69.10931	207.0766	73.30002
209.6117	79.13593	209.3974	69.10864	207.1212	73.29898
209.6547	79.13197	209.4378	69.10804	207.1606	73.29772
209.6935	79.12673	209.4776	69.10742	207.1955	73.29718
209.7295	79.12346	209.5192	69.1069	207.2372	73.2969
209.772	79.12196	209.5635	69.1063	207.2766	73.29577
209.814	79.12377	209.6039	69.10551	207.3206	73.2947
209.8558	79.12801	209.6469	69.10376	207.3587	73.29359
209.8942	79.12459	209.6902	69.10374	207.4019	73.29199
209.9378	79.12575	209.7298	69.10335	207.4429	73.29069
209.9763	79.12246	209.7714	69.10166	207.485	73.28944
210.0228	79.11415	209.8123	69.10105	207.5262	73.28883
210.0633	79.11043	209.8466	69.09873	207.57	73.28687
210.1024	79.10793	209.8862	69.0959	207.6107	73.28588
210.147	79.10835	209.9274	69.0958	207.6511	73.28563
210.1885	79.10882	209.969	69.0959	207.6919	73.28466
210.2285	79.11363	210.0139	69.09645	207.7346	73.28439
210.2635	79.11321	210.0564	69.0973	207.7743	73.28324
210.3047	79.10777	210.0951	69.09607	207.8079	73.28222
210.3451	79.10249	210.1356	69.09389	207.8506	73.28085
210.387	79.10047	210.1799	69.0927	207.8912	73.27982
210.4293	79.09821	210.2206	69.09093	207.9316	73.27908
210.468	79.09367	210.2606	69.09061	207.9767	73.27811
210.5116	79.09524	210.2959	69.09026	208.0147	73.27695
210.5531	79.09469	210.3379	69.0888	208.0592	73.27613
210.5942	79.09564	210.3783	69.08775	208.1021	73.2751
210.6353	79.09582	210.4201	69.08725	208.1434	73.27391
210.6787	79.09358	210.4617	69.08677	208.1854	73.27322
210.7134	79.09178	210.5028	69.08721	208.2267	73.27198
210.7529	79.08634	210.5432	69.08672	208.2674	73.27079
210.7937	79.08196	210.5874	69.08578	208.3088	73.26985
210.8375	79.07707	210.6299	69.0852	208.3508	73.2691
210.8822	79.07536	210.6701	69.08434	208.3906	73.2679
210.9212	79.07204	210.711	69.08311	208.4234	73.26701
210.9637	79.06889	210.7454	69.08218	208.4667	73.26612
211.0043	79.07347	210.7866	69.08125	208.5069	73.26421
211.0456	79.0728	210.8283	69.07887	208.5476	73.26268
211.0875	79.07204	210.8652	69.07831	208.59	73.26163
211.1281	79.07044	210.9086	69.07774	208.6303	73.26067
211.1631	79.06868	210.9484	69.07724	208.6712	73.26034
211.2037	79.06467	210.989	69.07768	208.7114	73.25982
211.2438	79.06508	211.0306	69.07768	208.7547	73.25977
211.2879	79.06832	211.0755	69.07724	208.7962	73.25909
211.3286	79.06472	211.1173	69.07647	208.8388	73.25767
211.3706	79.06091	211.1606	69.07597	208.878	73.25658
211.4111	79.05289	211.193	69.07462	208.9212	73.25517
211.4551	79.04698	211.2354	69.07338	208.9532	73.2542
211.4936	79.04664	211.2749	69.07212	208.995	73.2532
211.5356	79.04787	211.3166	69.06986	209.0371	73.25253
211.5792	79.04837	211.3589	69.06892	209.0787	73.2514
211.6154	79.04859	211.403	69.06772	209.1233	73.24982

211.6535	79.05163	211.4436	69.06704	209.1631	73.2487
211.6974	79.05011	211.4858	69.06725	209.2019	73.2475
211.736	79.05152	211.5262	69.06694	209.2479	73.24647
211.7784	79.053	211.5683	69.0668	209.2881	73.24624
211.8196	79.04853	211.6093	69.06777	209.3297	73.24581
211.8608	79.0479	211.6406	69.0676	209.3719	73.24479
211.9032	79.04425	211.682	69.06702	209.4098	73.24394
211.9455	79.04393	211.726	69.0664	209.4541	73.243
211.9863	79.044	211.7666	69.06486	209.4944	73.24212
212.0278	79.04461	211.8102	69.06281	209.5401	73.24103
212.0684	79.04408	211.8488	69.06237	209.5731	73.24008
212.1094	79.03965	211.8844	69.06255	209.6123	73.23862
212.1414	79.03732	211.9315	69.06147	209.6568	73.23796
212.1819	79.03206	211.9727	69.06136	209.6974	73.23688
212.2233	79.03044	212.0142	69.05997	209.7415	73.23591
212.2685	79.02952	212.0596	69.05815	209.7818	73.23476
212.3098	79.02942	212.0916	69.0572	209.8226	73.23293
212.348	79.02772	212.1332	69.05508	209.8654	73.23188
212.3902	79.02609	212.1777	69.05329	209.909	73.23039
212.4318	79.02139	212.2161	69.05215	209.9477	73.22886
212.474	79.01954	212.2579	69.0507	209.9917	73.22742
212.5173	79.01863	212.3022	69.05056	210.031	73.22667
212.5584	79.01491	212.342	69.0505	210.07	73.22652
212.5911	79.01565	212.3827	69.04932	210.1151	73.22585
212.6324	79.01054	212.4268	69.04811	210.1579	73.22517
212.6734	79.00741	212.468	69.04834	210.1906	73.22439
212.7175	79.00733	212.5106	69.04757	210.2322	73.22334
212.7574	79.00561	212.5457	69.04657	210.2722	73.22204
212.8006	79.005	212.5844	69.04699	210.3148	73.22126
212.841	78.99791	212.6258	69.04535	210.3549	73.21978
212.8845	78.99261	212.6683	69.04344	210.3944	73.21865
212.9235	78.98842	212.7118	69.04342	210.4356	73.21799
212.9677	78.98586	212.752	69.04233	210.4777	73.21734
213.0074	78.98755	212.7938	69.04242	210.5211	73.21642
213.0404	78.98701	212.8364	69.04258	210.5594	73.21554
213.0824	78.98242	212.8767	69.04115	210.6013	73.21475
213.1245	78.97972	212.919	69.04058	210.6433	73.21343
213.1639	78.97408	212.96	69.04008	210.6853	73.21264
213.2056	78.96717	212.991	69.03865	210.7196	73.21169
213.2478	78.96394	213.0343	69.03833	210.7598	73.21099
213.291	78.96042	213.0732	69.03781	210.7983	73.20971
213.3364	78.95902	213.1152	69.036	210.8418	73.20892
213.3759	78.95923	213.1552	69.0343	210.8843	73.20879
213.4162	78.96332	213.1956	69.03335	210.9254	73.20758
213.4584	78.96293	213.2388	69.03212	210.9664	73.20681
213.493	78.96153	213.281	69.03154	211.007	73.20556
213.5338	78.96187	213.325	69.03154	211.0484	73.20409
213.5746	78.95746	213.3656	69.03083	211.0895	73.20348
213.6191	78.95158	213.4066	69.03013	211.1345	73.20259
213.6567	78.95147	213.4388	69.02909	211.1779	73.20169
213.7021	78.95245	213.4817	69.02861	211.2182	73.20038
213.7408	78.95424	213.521	69.02763	211.258	73.19826
213.7812	78.95609	213.5636	69.02702	211.299	73.1971
213.8234	78.95295	213.6031	69.02619	211.3313	73.19626
213.8658	78.94723	213.6473	69.02538	211.3715	73.19552
213.9078	78.94403	213.6895	69.02583	211.4128	73.19538
213.9478	78.94034	213.7303	69.02479	211.455	73.19484
213.9907	78.93821	213.7713	69.02386	211.4953	73.19399

214.0224	78.93803	213.8105	69.02308	211.5367	73.19296
214.0669	78.93629	213.8543	69.02118	211.5806	73.19216
214.1062	78.93527	213.888	69.02093	211.6217	73.19069
214.1506	78.93429	213.9282	69.02087	211.6655	73.18909
214.1915	78.93316	213.9697	69.01974	211.7041	73.18726
214.2318	78.93296	214.0114	69.01894	211.7467	73.18565
214.2732	78.93401	214.0542	69.01758	211.7886	73.18462
214.3166	78.93751	214.0977	69.01612	211.8305	73.18429
214.3553	78.93894	214.1374	69.01522	211.8714	73.18383
214.3961	78.93931	214.1775	69.01422	211.912	73.18328
214.4381	78.9394	214.2223	69.01318	211.944	73.18279
214.4719	78.93696	214.263	69.01204	211.9846	73.18076
214.5116	78.93463	214.3055	69.01204	212.0277	73.17963
214.5542	78.92953	214.336	69.01229	212.0704	73.17788
214.5961	78.92562	214.3799	69.01212	212.1108	73.17577
214.6392	78.92209	214.4228	69.01267	212.1535	73.17491
214.6784	78.91881	214.4621	69.01238	212.1972	73.17336
214.7207	78.91633	214.5036	69.01037	212.2367	73.17252
214.7616	78.91642	214.5421	69.00925	212.2766	73.17177
214.8005	78.91773	214.5853	69.00751	212.3184	73.17072
214.8455	78.918	214.6275	69.00638	212.3621	73.16982
214.8856	78.91778	214.67	69.00556	212.4039	73.16864
214.9177	78.91527	214.7104	69.00391	212.4458	73.16804
214.962	78.91333	214.7514	69.00294	212.4798	73.16742
215.0039	78.91	214.7852	69.0018	212.5201	73.16749
215.0438	78.90685	214.8275	69.00065	212.5614	73.16749
215.088	78.90677	214.8686	68.99984	212.6017	73.16666
215.1295	78.90668	214.9082	68.9997	212.6418	73.16567
215.1699	78.90455	214.9524	68.9977	212.6875	73.16389
215.2094	78.90312	214.9934	68.99727	212.7296	73.16204
215.253	78.90127	215.0327	68.99735	212.7693	73.16101
215.295	78.89825	215.0766	68.9965	212.811	73.16049
215.3353	78.89784	215.1196	68.99662	212.8532	73.15942
215.3675	78.89811	215.1596	68.99522	212.8965	73.15861
215.4088	78.89992	215.2007	68.99273	212.9341	73.15739
215.4516	78.89958	215.2324	68.99125	212.9766	73.15601
215.4908	78.89753	215.2777	68.98977	213.017	73.15474
215.5336	78.89593	215.3174	68.98872	213.0616	73.15369
215.5758	78.89246	215.3606	68.98885	213.0958	73.15294
215.618	78.89109	215.402	68.98875	213.1349	73.15162
215.6603	78.88864	215.444	68.98821	213.1748	73.15142
215.7006	78.88829	215.485	68.9874	213.217	73.15115
215.7435	78.88649	215.5251	68.98641	213.2606	73.15046
215.7863	78.87927	215.5692	68.98479	213.3012	73.14996
215.8267	78.87835	215.6093	68.9837	213.3448	73.14891
215.869	78.87493	215.6519	68.98314	213.3825	73.14711
215.9042	78.86903	215.6831	68.98215	213.425	73.14604
215.943	78.869	215.7266	68.98153	213.4688	73.14534
215.9836	78.86934	215.7647	68.98101	213.5102	73.14372
216.0259	78.87163	215.8082	68.97978	213.5512	73.14254
216.0706	78.87825	215.8483	68.97912	213.5936	73.14129
216.1085	78.87506	215.8896	68.97914	213.6348	73.14015
216.149	78.87345	215.9307	68.9783	213.6766	73.13903
216.1918	78.87044	215.9746	68.97837	213.7111	73.13839
216.2328	78.86501	216.011	68.97772	213.7523	73.13755
216.2754	78.86632	216.0554	68.97578	213.7922	73.13663
216.3158	78.86507	216.0944	68.97462	213.8342	73.13526
216.3473	78.86388	216.1302	68.97312	213.8738	73.13396

216.3902	78.86274	216.1713	68.97093	213.9174	73.13302
216.4322	78.86109	216.2135	68.96956	213.9575	73.13142
216.4772	78.85752	216.2548	68.96789	213.9994	73.13054
216.5201	78.8518	216.2964	68.96616	214.0434	73.13022
216.5595	78.84501	216.3394	68.96528	214.0834	73.12865
216.601	78.84326	216.3779	68.9646	214.1272	73.12785
216.6422	78.84197	216.419	68.96459	214.1665	73.12691
216.6825	78.84401	216.4616	68.96443	214.2102	73.12551
216.7226	78.84806	216.5054	68.96339	214.2449	73.12467
216.7622	78.84828	216.5451	68.96297	214.2851	73.12321
216.7949	78.84849	216.5799	68.96257	214.3283	73.12215
216.8407	78.84735	216.6192	68.96132	214.369	73.122
216.8824	78.8451	216.6611	68.95981	214.4105	73.12157
216.9213	78.84472	216.7042	68.95849	214.4522	73.12037
216.9642	78.84173	216.7442	68.95688	214.4937	73.11915
217.007	78.83925	216.7833	68.95584	214.5355	73.11732
217.0474	78.83699	216.8288	68.95518	214.5776	73.1157
217.089	78.83264	216.8682	68.9545	214.6188	73.11509
217.1311	78.83066	216.9113	68.95282	214.6623	73.11439
217.1736	78.8266	216.9515	68.95225	214.7021	73.11349
217.2156	78.82193	216.9952	68.9515	214.7443	73.11254
217.2458	78.81871	217.0302	68.94995	214.7867	73.1113
217.2885	78.81495	217.0729	68.94983	214.8244	73.11041
217.332	78.81565	217.1145	68.94843	214.8623	73.10965
217.3727	78.81304	217.1546	68.94711	214.9038	73.10882
217.4144	78.81008	217.1935	68.94689	214.9445	73.10822
217.4556	78.81092	217.2367	68.94611	214.985	73.1069
217.4961	78.81133	217.2796	68.94464	215.0256	73.10527
217.5393	78.81074	217.3226	68.94398	215.0678	73.10398
217.5813	78.8101	217.3642	68.94223	215.1122	73.10248
217.622	78.80928	217.4066	68.93991	215.153	73.10119
217.6641	78.80782	217.4463	68.9394	215.194	73.10027
217.707	78.80631	217.4831	68.93817	215.235	73.09915
217.749	78.80649	217.5234	68.9372	215.2742	73.09851
217.7811	78.80628	217.5654	68.93712	215.3175	73.09828
217.824	78.80262	217.6072	68.93577	215.3606	73.09767
217.8646	78.7998	217.6472	68.93519	215.4015	73.09671
217.9068	78.7985	217.6912	68.93417	215.444	73.09556
217.9469	78.79855	217.7314	68.93291	215.4725	73.09478
217.9902	78.7988	217.774	68.93226	215.5152	73.09342
218.0306	78.79753	217.8142	68.93134	215.5575	73.09202
218.074	78.79954	217.8568	68.93076	215.5978	73.09133
218.1156	78.79945	217.897	68.93023	215.6403	73.09019
218.1564	78.79269	217.9334	68.92904	215.6807	73.08953
218.1986	78.78977	217.9734	68.9273	215.7201	73.08881
218.2304	78.78915	218.0128	68.92587	215.7653	73.08722
218.2714	78.78055	218.0567	68.92517	215.8082	73.08644
218.3113	78.77491	218.0968	68.92404	215.8466	73.08587
218.3537	78.77617	218.1384	68.92281	215.8918	73.08534
218.397	78.77344	218.1791	68.92162	215.9333	73.08458
218.4379	78.77316	218.2199	68.91926	215.974	73.08336
218.4798	78.77552	218.2625	68.91741	216.007	73.08221
218.522	78.77942	218.3047	68.91717	216.0512	73.08089
218.5629	78.78026	218.3462	68.91675	216.089	73.07987
218.6062	78.77481	218.3775	68.91784	216.1314	73.0791
218.6459	78.77308	218.4205	68.92011	216.1691	73.07819
218.6803	78.77193	218.4605	68.91907	216.2122	73.07701
218.7225	78.76881	218.5059	68.91798	216.2528	73.07632

218.7619	78.7712	218.5477	68.91559	216.297	73.07515
218.8049	78.77127	218.5903	68.91297	216.3373	73.07418
218.8452	78.77	218.6318	68.91118	216.3789	73.07349
218.8888	78.76833	218.6703	68.90942	216.4192	73.07208
218.9314	78.76691	218.7126	68.90714	216.4644	73.07069
218.9719	78.76768	218.7535	68.90555	216.5039	73.06898
219.0148	78.76512	218.798	68.90458	216.5485	73.06796
219.0571	78.76012	218.827	68.90389	216.5906	73.06686
219.0985	78.75787	218.8701	68.90359	216.6256	73.06608
219.1314	78.75709	218.9137	68.9024	216.6648	73.0656
219.1713	78.75421	218.9548	68.90175	216.707	73.06459
219.2124	78.75281	218.9953	68.90203	216.7461	73.06363
219.2545	78.75131	219.038	68.90239	216.7896	73.06288
219.2958	78.74986	219.0788	68.90153	216.8269	73.06201
219.3406	78.75098	219.1187	68.90165	216.8692	73.06074
219.3805	78.74838	219.16	68.90123	216.912	73.05953
219.4221	78.74481	219.2011	68.89866	216.9533	73.05777
219.4634	78.74281	219.2427	68.89789	216.9956	73.05622
219.5056	78.73899	219.273	68.89723	217.0345	73.05547
219.547	78.73771	219.3177	68.8962	217.0783	73.05478
219.5898	78.73931	219.3604	68.89532	217.1186	73.05441
219.6287	78.7375	219.3997	68.89456	217.1635	73.0534
219.663	78.73677	219.4444	68.893	217.2042	73.05216
219.7048	78.73437	219.4853	68.8912	217.2386	73.05118
219.746	78.73316	219.5276	68.89072	217.2776	73.05024
219.7873	78.73164	219.5675	68.88924	217.3184	73.04986
219.8307	78.72792	219.6067	68.88829	217.3593	73.04942
219.8726	78.73022	219.6495	68.88634	217.3995	73.04883
219.9168	78.73108	219.6922	68.88455	217.4449	73.048
219.9556	78.72926	219.7236	68.88385	217.482	73.04703
219.9975	78.72663	219.7646	68.88326	217.526	73.0459
220.0394	78.71987	219.8055	68.88243	217.5683	73.04463
220.0817	78.71436	219.8499	68.88208	217.6097	73.0433
220.113	78.71224	219.8904	68.88225	217.6512	73.04195
220.1533	78.70903	219.9313	68.88135	217.6914	73.04082
220.1946	78.70629	219.9761	68.88075	217.7328	73.03987
220.2364	78.70331	220.0138	68.87991	217.7674	73.03879
220.2783	78.69953	220.055	68.87829	217.8086	73.0378
220.3188	78.69456	220.0984	68.87721	217.8508	73.03689
220.3622	78.69106	220.1398	68.87581	217.8898	73.03567
220.4018	78.68815	220.1729	68.87467	217.9329	73.03459
220.4428	78.68817	220.2129	68.87237	217.974	73.03311
220.4879	78.69231	220.2541	68.87037	218.0132	73.03175
220.5266	78.69331	220.2995	68.86831	218.0578	73.03058
220.5594	78.69526	220.3384	68.86693	218.103	73.02991
220.5997	78.69576	220.3828	68.86656	218.1443	73.02926
220.643	78.69177	220.4218	68.86619	218.1838	73.02811
220.6834	78.68845	220.4647	68.8663	218.2213	73.02724
220.7265	78.68692	220.5032	68.86591	218.2673	73.02582
220.7697	78.6873	220.5449	68.86511	218.3074	73.02439
220.8102	78.68805	220.586	68.8638	218.3491	73.02375
220.853	78.68666	220.6202	68.86286	218.381	73.02289
220.8956	78.68783	220.6622	68.86156	218.4214	73.02239
220.9386	78.68296	220.7061	68.8611	218.4625	73.02202
220.9798	78.67784	220.7486	68.85971	218.5052	73.02091
221.0137	78.67704	220.7884	68.85946	218.5474	73.01974
221.0516	78.67041	220.8319	68.85888	218.5865	73.01851
221.0939	78.67025	220.8719	68.85709	218.6292	73.01696

221.1354	78.67049	220.9156	68.85631	218.6711	73.01547
221.1754	78.66895	220.9582	68.8559	218.713	73.01388
221.2165	78.67368	220.9991	68.85468	218.7551	73.01295
221.2596	78.67124	221.0405	68.85335	218.7954	73.01228
221.2994	78.66601	221.0754	68.85349	218.837	73.01218
221.342	78.66786	221.1148	68.85228	218.8805	73.01199
221.3849	78.66877	221.1584	68.85105	218.9188	73.01091
221.4268	78.66682	221.1989	68.85016	218.9635	73.00934
221.4692	78.66851	221.2412	68.84865	218.9951	73.00817
221.5119	78.66693	221.2836	68.8478	219.036	73.00682
221.5459	78.66301	221.3241	68.84681	219.0766	73.00536
221.5871	78.66048	221.3666	68.8457	219.1176	73.00446
221.6258	78.65751	221.4096	68.84526	219.1594	73.00323
221.667	78.66111	221.4497	68.84401	219.203	73.00248
221.7113	78.6616	221.4917	68.84255	219.2445	73.00195
221.7519	78.65791	221.5258	68.84271	219.2832	73.00166
221.793	78.65486	221.567	68.84305	219.3242	73.00102
221.834	78.65208	221.6064	68.84106	219.3662	72.99925
221.8786	78.65042	221.648	68.83987	219.4108	72.99768
221.9186	78.64835	221.6904	68.83971	219.4518	72.99599
221.957	78.64875	221.7325	68.83709	219.4948	72.99436
221.993	78.64701	221.7738	68.83598	219.5294	72.99355
222.0357	78.64354	221.8144	68.8341	219.5709	72.99249
222.0761	78.63846	221.8574	68.83095	219.6103	72.99172
222.1164	78.63819	221.8986	68.82893	219.6514	72.99085
222.1604	78.6386	221.9387	68.82782	219.694	72.98989
222.1993	78.63465	221.9725	68.82766	219.7383	72.98867
222.2412	78.63184	222.0141	68.82754	219.7801	72.98771
222.2834	78.62871	222.0575	68.82796	219.8209	72.98714
222.3256	78.6209	222.0991	68.82706	219.8617	72.98635
222.3664	78.61675	222.1396	68.82534	219.9026	72.9853
222.4066	78.61827	222.1806	68.82364	219.9437	72.98386
222.4447	78.61999	222.2186	68.82111	219.9873	72.98256
222.4857	78.62458	222.2634	68.82026	220.0302	72.9811
222.5284	78.6266	222.3024	68.81967	220.0716	72.98059
222.5687	78.63024	222.3458	68.81936	220.1118	72.97951
222.6083	78.62779	222.3894	68.81931	220.1456	72.97879
222.647	78.62538	222.4186	68.81884	220.1875	72.97785
222.6926	78.62427	222.4593	68.81789	220.2289	72.97691
222.734	78.62016	222.5016	68.81665	220.2709	72.97612
222.7752	78.61969	222.543	68.81572	220.3106	72.97475
222.8164	78.6156	222.5843	68.81413	220.351	72.97427
222.8556	78.60756	222.6255	68.81347	220.3942	72.97316
222.8877	78.60788	222.667	68.8139	220.4362	72.97203
222.934	78.60221	222.7098	68.81207	220.479	72.97112
222.9732	78.59742	222.75	68.81104	220.5198	72.96973
223.0171	78.59763	222.7923	68.80933	220.562	72.96852
223.0582	78.59499	222.8366	68.80607	220.6015	72.96721
223.099	78.59804	222.8675	68.80498	220.6421	72.96621
223.1426	78.59678	222.91	68.80448	220.6843	72.9652
223.1838	78.59562	222.9539	68.80273	220.726	72.96448
223.2241	78.59094	222.9938	68.80324	220.7576	72.9638
223.2656	78.58458	223.0376	68.8035	220.8008	72.96284
223.3062	78.5846	223.0789	68.80356	220.8406	72.96179
223.3481	78.58567	223.1208	68.80344	220.8819	72.96012
223.3915	78.58698	223.1592	68.80205	220.9232	72.95927
223.4238	78.58908	223.203	68.80089	220.9669	72.95764
223.466	78.58714	223.2417	68.79806	221.0073	72.95676

223.5079	78.58107	223.2837	68.79588	221.0502	72.9563
223.5459	78.57546	223.3193	68.79426	221.091	72.95483
223.5896	78.5697	223.3591	68.793	221.131	72.95431
223.6316	78.56602	223.4012	68.79322	221.1737	72.95328
223.6705	78.56007	223.443	68.79356	221.2147	72.95229
223.7158	78.55917	223.4848	68.79328	221.2566	72.95101
223.757	78.5606	223.527	68.79201	221.2897	72.95002
223.7976	78.55971	223.5686	68.78982	221.3304	72.94901
223.8392	78.56155	223.6066	68.7883	221.371	72.94771
223.8709	78.56303	223.6503	68.78683	221.4112	72.94717
223.9131	78.56171	223.6905	68.78443	221.4561	72.94611
223.9551	78.55752	223.7318	68.78266	221.497	72.94508
223.9974	78.55516	223.7642	68.78122	221.5393	72.94391
224.0384	78.55378	223.8066	68.77954	221.5809	72.9432
224.0813	78.55061	223.8507	68.77952	221.6224	72.94294
224.1194	78.54713	223.89	68.7787	221.6652	72.94172
224.158	78.54689	223.931	68.7777	221.7075	72.94045
224.1996	78.54743	223.975	68.77777	221.7503	72.93847
224.2416	78.54439	224.0137	68.77729	221.7924	72.93665
224.285	78.54176	224.0575	68.77671	221.8322	72.93586
224.3183	78.54103	224.0972	68.77671	221.8746	72.93517
224.3582	78.54263	224.1392	68.77538	221.9063	72.93496
224.4	78.54355	224.1817	68.77442	221.9509	72.93411
224.4416	78.54228	224.212	68.77442	221.993	72.93354
224.4839	78.54222	224.2558	68.77269	222.0338	72.93232
224.5245	78.53428	224.2967	68.77173	222.0758	72.93092
224.5655	78.52659	224.3402	68.7708	222.1158	72.92975
224.608	78.52458	224.3808	68.76906	222.1582	72.92872
224.651	78.52646	224.4214	68.76811	222.2002	72.92708
224.6925	78.52819	224.4627	68.76756	222.243	72.92584
224.7332	78.52774	224.5066	68.76624	222.2823	72.92467
224.7664	78.52825	224.547	68.76453	222.324	72.92263
224.8062	78.52198	224.5892	68.76302	222.3652	72.92173
224.8482	78.516	224.6295	68.7603	222.4069	72.92031
224.8896	78.51298	224.6627	68.75913	222.448	72.91997
224.9304	78.51148	224.7039	68.75843	222.4882	72.91967
224.9714	78.51336	224.7455	68.75681	222.525	72.91866
225.0138	78.51069	224.7881	68.75623	222.5642	72.91791
225.0585	78.50972	224.8306	68.75521	222.6062	72.91651
225.1022	78.50739	224.8738	68.7542	222.6489	72.91489
225.1425	78.50182	224.9148	68.75487	222.6917	72.91417
225.185	78.50081	224.9559	68.75411	222.7332	72.91372
225.2213	78.49666	225.0006	68.75317	222.7748	72.91342
225.2666	78.49577	225.0417	68.75214	222.8142	72.91281
225.299	78.4918	225.0792	68.75112	222.857	72.91142
225.3398	78.48767	225.1132	68.74922	222.9003	72.91035
225.38	78.4905	225.1553	68.74877	222.9399	72.90845
225.4219	78.49055	225.1947	68.74885	222.9807	72.90708
225.468	78.49278	225.2388	68.74709	223.0219	72.90646
225.507	78.49173	225.28	68.74629	223.0552	72.90539
225.5487	78.48952	225.3213	68.74526	223.0977	72.90475
225.5908	78.48576	225.3635	68.74442	223.137	72.90359
225.6312	78.4846	225.4037	68.74365	223.1804	72.90185
225.6741	78.4831	225.4475	68.74267	223.2232	72.90053
225.7154	78.48064	225.4876	68.74042	223.2636	72.8992
225.7502	78.47893	225.5267	68.73872	223.3049	72.89848
225.7904	78.47909	225.5614	68.73686	223.3482	72.89738
225.8291	78.48148	225.6024	68.73478	223.3881	72.89669

225.8738	78.48291	225.6485	68.73544	223.4302	72.89584
225.9136	78.48266	225.6888	68.73468	223.4713	72.8949
225.9572	78.48304	225.728	68.73406	223.5111	72.89457
226.0022	78.47807	225.7711	68.73381	223.5545	72.89357
226.0442	78.47222	225.8138	68.73191	223.5962	72.89257
226.0825	78.46931	225.8556	68.73042	223.6365	72.89139
226.1234	78.46493	225.8968	68.72934	223.6703	72.89026
226.1646	78.46056	225.9373	68.72798	223.7114	72.88868
226.1989	78.45617	225.9798	68.72694	223.7524	72.88731
226.2404	78.45701	226.0112	68.72604	223.7961	72.88662
226.2833	78.45527	226.0545	68.72422	223.8399	72.88519
226.3241	78.45294	226.0958	68.72236	223.8779	72.88451
226.3654	78.45335	226.1389	68.7209	223.9176	72.88379
226.4047	78.45239	226.1774	68.71896	223.9588	72.88292
226.4478	78.45364	226.2187	68.71822	224.001	72.88191
226.4893	78.44994	226.2626	68.71913	224.0429	72.88069
226.5299	78.44757	226.3039	68.71808	224.0842	72.87995
226.5711	78.44663	226.3484	68.71692	224.1254	72.87846
226.6149	78.4402	226.388	68.71608	224.1659	72.87776
226.649	78.43824	226.4308	68.7133	224.2093	72.87674
226.6908	78.44059	226.464	68.71271	224.2483	72.87546
226.7294	78.4395	226.5046	68.71294	224.2803	72.87419
226.7744	78.43794	226.548	68.71119	224.3234	72.87306
226.8142	78.43894	226.588	68.71013	224.3666	72.87201
226.8566	78.44062	226.631	68.70843	224.4094	72.87028
226.8986	78.43867	226.673	68.70576	224.4511	72.86904
226.937	78.43471	226.7131	68.70501	224.4929	72.86777
226.9818	78.43144	226.7511	68.70525	224.5314	72.86617
227.022	78.42585	226.7951	68.70463	224.57	72.86498
227.065	78.41936	226.8357	68.70473	224.6144	72.86433
227.1066	78.41451	226.8779	68.70467	224.6566	72.86343
227.147	78.41505	226.9113	68.70264	224.6994	72.86325
227.1792	78.41637	226.9539	68.70138	224.7405	72.86325
227.2224	78.41561	226.9976	68.70019	224.7827	72.86218
227.2639	78.41524	227.0373	68.69804	224.8126	72.86138
227.3058	78.41426	227.0789	68.69692	224.8576	72.85948
227.3466	78.41092	227.1226	68.69566	224.9	72.85785
227.3893	78.40896	227.1636	68.69436	224.9418	72.85637
227.4286	78.40618	227.2033	68.69358	224.9819	72.85561
227.469	78.40479	227.2468	68.69183	225.0216	72.85536
227.512	78.40129	227.2864	68.69123	225.0625	72.8543
227.5548	78.40018	227.3248	68.6903	225.107	72.85278
227.593	78.40227	227.3602	68.68793	225.1468	72.85135
227.626	78.39921	227.4002	68.68668	225.1908	72.84964
227.6686	78.39503	227.4441	68.68501	225.231	72.84837
227.7105	78.39149	227.4838	68.68204	225.2727	72.84847
227.7532	78.39022	227.5254	68.68097	225.3131	72.84769
227.7962	78.38729	227.5683	68.68064	225.3546	72.84667
227.8385	78.38851	227.6086	68.67988	225.3949	72.84541
227.8788	78.39011	227.6497	68.67901	225.4289	72.84451
227.9204	78.38826	227.6927	68.67761	225.47	72.8433
227.9602	78.38512	227.7317	68.67531	225.5108	72.84261
227.9998	78.38474	227.7756	68.6747	225.5551	72.84164
228.0442	78.38235	227.8071	68.67521	225.5972	72.84025
228.076	78.37856	227.8495	68.67473	225.6374	72.83871
228.1189	78.37806	227.8896	68.67473	225.6791	72.83723
228.1629	78.37795	227.9328	68.67427	225.7222	72.83624
228.2024	78.37694	227.9728	68.67245	225.7611	72.83519

228.2454	78.37536	228.0154	68.67224	225.8054	72.83423
228.2873	78.37439	228.0553	68.67099	225.8457	72.83314
228.3247	78.36987	228.0946	68.66908	225.889	72.83239
228.3676	78.36476	228.1387	68.6687	225.9306	72.83118
228.4098	78.36142	228.1801	68.6676	225.9717	72.83023
228.4518	78.36151	228.2213	68.66666	226.0139	72.82962
228.4922	78.36061	228.2558	68.66587	226.0461	72.82903
228.5258	78.35821	228.2998	68.66407	226.09	72.82837
228.567	78.35401	228.3388	68.66157	226.1301	72.8274
228.6059	78.35412	228.381	68.66029	226.171	72.82657
228.651	78.35359	228.4278	68.65863	226.2128	72.8249
228.6932	78.34971	228.4629	68.65736	226.2539	72.82301
228.7344	78.34867	228.5067	68.65762	226.2987	72.82162
228.7738	78.3483	228.5476	68.65765	226.3364	72.82053
228.8166	78.34579	228.5915	68.65613	226.3752	72.82014
228.859	78.34452	228.6332	68.65498	226.42	72.81965
228.899	78.34457	228.6737	68.65334	226.4638	72.81869
228.9404	78.34214	228.709	68.65134	226.5078	72.81784
228.9823	78.33723	228.751	68.65077	226.545	72.81685
229.0252	78.33362	228.791	68.6497	226.5807	72.81615
229.0592	78.3338	228.8317	68.64813	226.6194	72.81555
229.1025	78.3318	228.8768	68.64689	226.6615	72.81449
229.1424	78.32869	228.9144	68.64599	226.7037	72.81317
229.1855	78.33026	228.9597	68.64483	226.745	72.8111
229.223	78.33158	228.999	68.6435	226.786	72.80928
229.267	78.33056	229.0368	68.64332	226.8274	72.80783
229.3078	78.32952	229.0807	68.64168	226.8668	72.80651
229.3496	78.32809	229.1217	68.6428	226.9099	72.80636
229.393	78.32673	229.1551	68.64321	226.9512	72.80564
229.435	78.32287	229.1956	68.64217	226.9916	72.80505
229.473	78.31682	229.2383	68.64149	227.0358	72.80385
229.5096	78.31197	229.2798	68.63967	227.0752	72.8021
229.5478	78.30823	229.3219	68.63764	227.1214	72.80058
229.5913	78.30211	229.3626	68.63644	227.1616	72.79875
229.6298	78.2984	229.4038	68.63539	227.1965	72.79774
229.6728	78.3012	229.446	68.63384	227.2374	72.79745
229.7147	78.29939	229.4888	68.6344	227.2769	72.79751
229.7578	78.29736	229.5293	68.63377	227.3198	72.79735
229.8001	78.29802	229.571	68.63331	227.3611	72.79664
229.8401	78.29856	229.6052	68.6329	227.4025	72.795
229.8827	78.29787	229.6444	68.63104	227.444	72.79383
229.9229	78.29718	229.6882	68.62888	227.4852	72.79178
229.9588	78.29702	229.73	68.62899	227.5263	72.79026
230.0008	78.29611	229.7723	68.62885	227.5691	72.78938
230.0405	78.29526	229.813	68.62706	227.6115	72.78825
230.0842	78.28988	229.8562	68.62655	227.6535	72.78684
230.1272	78.28752	229.896	68.62481	227.6924	72.78506
230.1659	78.28211	229.9384	68.62343	227.7354	72.7835
230.2082	78.27328	229.9814	68.62169	227.7774	72.78184
230.2519	78.27485	230.0224	68.61966	227.81	72.78112
230.296	78.275	230.0564	68.61761	227.8525	72.78035
230.3336	78.27229	230.0963	68.61559	227.8942	72.77925
230.3782	78.27233	230.1378	68.61503	227.9354	72.77828
230.4112	78.27124	230.1816	68.61437	227.9788	72.77704
230.4506	78.26963	230.2222	68.61507	228.0195	72.77629
230.492	78.26662	230.2625	68.61297	228.0584	72.77568
230.532	78.26595	230.3058	68.61072	228.0986	72.77454
230.5756	78.26452	230.3502	68.60962	228.1431	72.77367

230.6182	78.26442	230.3898	68.60755	228.1818	72.77249
230.6593	78.26615	230.4326	68.6076	228.224	72.77114
230.6988	78.2621	230.4722	68.60883	228.2662	72.76981
230.742	78.25773	230.507	68.60915	228.3082	72.76906
230.7842	78.25048	230.547	68.6067	228.3406	72.76829
230.8265	78.24698	230.5873	68.60542	228.381	72.76725
230.8673	78.24709	230.6281	68.60327	228.4227	72.76589
230.909	78.2444	230.669	68.60131	228.467	72.76406
230.9402	78.24512	230.7131	68.60029	228.509	72.76227
230.9808	78.24642	230.7544	68.59807	228.5488	72.7617
231.0247	78.2469	230.7954	68.59722	228.5924	72.7611
231.0652	78.24622	230.837	68.59683	228.6344	72.76049
231.1053	78.24442	230.8756	68.59538	228.6695	72.75969
231.1466	78.24179	230.9203	68.59397	228.7132	72.75879
231.1886	78.23654	230.9515	68.59167	228.7523	72.75796
231.231	78.23007	230.9954	68.58786	228.7944	72.75691
231.272	78.22697	231.0342	68.58686	228.8374	72.75569
231.3152	78.22598	231.0752	68.58615	228.8802	72.754
231.3586	78.22385	231.1179	68.5851	228.9206	72.75298
231.3892	78.22396	231.1597	68.58434	228.9528	72.75243
231.432	78.22595	231.2014	68.58348	228.9927	72.75123
231.4746	78.22335	231.2433	68.58195	229.0378	72.75039
231.5157	78.22002	231.287	68.58119	229.0814	72.74905
231.5576	78.21805	231.3247	68.57977	229.1215	72.74732
231.5978	78.21733	231.3694	68.57837	229.1627	72.74651
231.6375	78.21437	231.4013	68.57787	229.2047	72.74602
231.6822	78.21208	231.4414	68.57767	229.2464	72.74457
231.7232	78.21272	231.4818	68.57648	229.2886	72.74338
231.7621	78.21219	231.525	68.57577	229.3285	72.74239
231.8064	78.20846	231.567	68.5747	229.3685	72.74103
231.8388	78.20569	231.6094	68.574	229.4099	72.74023
231.8788	78.20431	231.6526	68.57335	229.4506	72.7398
231.9226	78.2033	231.6917	68.57205	229.4934	72.73903
231.9641	78.20159	231.7307	68.57102	229.538	72.73838
232.0039	78.20133	231.7768	68.56898	229.5698	72.7382
232.0464	78.20184	231.8184	68.56796	229.6105	72.73727
232.0878	78.19594	231.8496	68.56749	229.6545	72.73584
232.1312	78.19125	231.8892	68.56676	229.6952	72.73439
232.1739	78.18912	231.9318	68.56575	229.7378	72.73235
232.2146	78.18692	231.973	68.56382	229.7797	72.73069
232.2566	78.18721	232.0143	68.56178	229.8197	72.7293
232.2886	78.1843	232.0572	68.55915	229.8606	72.72842
232.3288	78.18248	232.0959	68.55701	229.9043	72.72723
232.3709	78.18177	232.1372	68.55587	229.9455	72.72635
232.4125	78.17851	232.1803	68.55564	229.988	72.72569
232.4536	78.17927	232.2232	68.55452	230.026	72.72474
232.4921	78.17933	232.2644	68.55391	230.0714	72.72386
232.5323	78.17674	232.2975	68.55355	230.1007	72.72328
232.5776	78.17417	232.3375	68.55189	230.1437	72.72231
232.6202	78.17039	232.3786	68.5511	230.1858	72.72008
232.6595	78.1687	232.4224	68.54979	230.2264	72.71954
232.7014	78.16594	232.4652	68.54921	230.2682	72.7181
232.7452	78.16415	232.5074	68.54917	230.3122	72.71643
232.7869	78.16223	232.5494	68.54817	230.3536	72.71612
232.818	78.1619	232.5904	68.54757	230.3958	72.71449
232.8597	78.1599	232.6296	68.54572	230.4339	72.71409
232.9037	78.1568	232.6734	68.54193	230.4762	72.71321
232.943	78.15776	232.7144	68.54	230.5203	72.7118

232.9821	78.15678	232.7499	68.53837	230.5624	72.71091
233.0234	78.15358	232.7902	68.5366	230.5991	72.7095
233.0687	78.15197	232.8306	68.53617	230.6406	72.70828
233.1086	78.1502	232.872	68.53573	230.6831	72.70743
233.1499	78.1448	232.9146	68.5351	230.7169	72.70651
233.1925	78.14387	232.9541	68.53401	230.7589	72.70536
233.2333	78.14286	232.998	68.53308	230.8006	72.70467
233.265	78.14008	233.0367	68.53096	230.8446	72.70294
233.31	78.13874	233.0774	68.52877	230.885	72.70139
233.3495	78.13919	233.1194	68.52683	230.9298	72.70009
233.3909	78.1369	233.159	68.52526	230.9687	72.6988
233.433	78.13491	233.1964	68.52479	231.0104	72.69805
233.4756	78.13345	233.2376	68.52483	231.0525	72.6968
233.5173	78.12784	233.2792	68.52489	231.0938	72.69501
233.5613	78.12573	233.319	68.52489	231.1348	72.69392
233.6028	78.1223	233.3622	68.52417	231.1769	72.69321
233.6429	78.12117	233.4043	68.52203	231.219	72.69231
233.6815	78.12353	233.4405	68.5214	231.261	72.69118
233.7142	78.12338	233.4869	68.52021	231.3013	72.69012
233.7554	78.1204	233.5274	68.51798	231.3318	72.68873
233.797	78.12025	233.5688	68.51653	231.3745	72.68736
233.838	78.11593	233.6102	68.5143	231.4162	72.68672
233.8789	78.10958	233.6448	68.51232	231.4594	72.68544
233.9194	78.10436	233.6864	68.51	231.4993	72.68427
233.9605	78.10078	233.726	68.50797	231.5409	72.68293
234.0036	78.09986	233.77	68.50657	231.584	72.68128
234.045	78.09791	233.8121	68.50472	231.6268	72.67985
234.085	78.1001	233.8531	68.50378	231.665	72.67873
234.1252	78.10045	233.8963	68.50361	231.7079	72.67767
234.1606	78.09849	233.9358	68.50264	231.7492	72.67695
234.2038	78.09625	233.9754	68.50286	231.7894	72.67648
234.2438	78.09412	234.0172	68.5017	231.8316	72.67523
234.289	78.09178	234.061	68.49997	231.8636	72.67423
234.3303	78.08632	234.0956	68.49907	231.9066	72.67284
234.3744	78.08178	234.1374	68.49684	231.9487	72.67087
234.4161	78.07965	234.1765	68.4954	231.9913	72.66968
234.459	78.07647	234.2195	68.49422	232.0342	72.66904
234.4993	78.07278	234.2599	68.49282	232.0754	72.6683
234.5394	78.07294	234.3018	68.49126	232.1142	72.66731
234.5798	78.07408	234.347	68.49045	232.1555	72.66621
234.619	78.06914	234.3851	68.4898	232.1942	72.66601
234.6585	78.06726	234.4265	68.48813	232.2417	72.66577
234.6928	78.06734	234.4705	68.48623	232.2802	72.6651
234.7373	78.06317	234.5129	68.48384	232.3248	72.6639
234.7793	78.06203	234.5459	68.48265	232.3662	72.66222
234.8224	78.06184	234.5852	68.48158	232.4071	72.66081
234.864	78.06141	234.6276	68.48159	232.449	72.66003
234.9034	78.06091	234.6678	68.48136	232.481	72.65948
234.9434	78.05869	234.7102	68.48136	232.5238	72.65876
234.9852	78.05537	234.7534	68.48021	232.5679	72.65781
235.0261	78.05339	234.7946	68.47908	232.6072	72.65633
235.0678	78.05077	234.8364	68.47594	232.6435	72.65536
235.1076	78.04695	234.878	68.47275	232.6866	72.65417
235.1426	78.04674	234.9158	68.4708	232.7287	72.65282
235.1845	78.04287	234.9588	68.46933	232.7725	72.65158
235.2279	78.03902	234.9943	68.46994	232.8154	72.65021
235.2688	78.03613	235.0346	68.46881	232.8566	72.64826
235.307	78.03589	235.0769	68.46767	232.8957	72.64678

235.3496	78.03621	235.1192	68.46684	232.937	72.64578
235.3949	78.03474	235.1597	68.46454	232.9776	72.64516
235.4348	78.0358	235.2014	68.46253	233.0208	72.64476
235.477	78.03361	235.2433	68.45964	233.061	72.64401
235.5194	78.02975	235.2859	68.45587	233.096	72.6427
235.5598	78.02597	235.3268	68.45314	233.1375	72.64154
235.5943	78.02214	235.3683	68.45139	233.1797	72.63964
235.6368	78.01996	235.4126	68.44923	233.2221	72.63806
235.6774	78.02034	235.444	68.44813	233.2651	72.63765
235.7185	78.01963	235.486	68.44677	233.3058	72.63683
235.7581	78.01749	235.5285	68.44549	233.3443	72.6362
235.8016	78.01156	235.5682	68.44525	233.3882	72.63545
235.8445	78.00824	235.6088	68.44433	233.4291	72.63425
235.8856	78.00711	235.6515	68.44379	233.4694	72.63261
235.9271	78.00771	235.6928	68.44316	233.5142	72.63131
235.9655	78.0047	235.7337	68.44229	233.5545	72.63011
236.0104	78.00173	235.7725	68.44178	233.5938	72.62882
236.0421	78.00222	235.8121	68.44055	233.6298	72.62771
236.0837	77.99892	235.8581	68.43963	233.6701	72.62609
236.127	77.99775	235.8894	68.43939	233.7128	72.62486
236.1678	77.99544	235.9276	68.43738	233.7553	72.62342
236.2098	77.99283	235.97	68.43604	233.7965	72.62294
236.2511	77.98922	236.0123	68.43435	233.837	72.62324
236.293	77.98729	236.0589	68.4334	233.8791	72.62301
236.3336	77.98887	236.0986	68.43295	233.9203	72.62182
236.3779	77.9842	236.1427	68.43181	233.9618	72.62078
236.4158	77.98171	236.1808	68.43104	234.0029	72.61923
236.4582	77.98146	236.2218	68.42886	234.0465	72.61712
236.5014	77.9802	236.2648	68.42755	234.0855	72.61575
236.5438	77.97982	236.3038	68.42687	234.1283	72.61397
236.5746	77.97735	236.3386	68.4261	234.1679	72.61227
236.618	77.97585	236.38	68.42461	234.2089	72.61141
236.657	77.97433	236.4207	68.42259	234.2446	72.6105
236.7021	77.97085	236.4635	68.42056	234.2852	72.60945
236.7423	77.96824	236.506	68.41903	234.3261	72.60806
236.7848	77.96868	236.5483	68.41845	234.3705	72.60637
236.8216	77.96614	236.5912	68.41827	234.4113	72.60549
236.8677	77.96128	236.6306	68.41722	234.4546	72.6043
236.9084	77.95939	236.6726	68.41494	234.4948	72.60391
236.952	77.95705	236.7142	68.41343	234.5374	72.60291
236.9934	77.95361	236.759	68.4123	234.5786	72.60162
237.027	77.95119	236.7912	68.41138	234.6186	72.60064
237.0687	77.95101	236.8322	68.41085	234.6607	72.59936
237.1073	77.95004	236.8739	68.41017	234.7025	72.59844
237.1513	77.94851	236.9152	68.4093	234.7451	72.59714
237.192	77.94538	236.9532	68.40877	234.7893	72.59586
237.2356	77.94342	236.998	68.40787	234.83	72.59401
237.2782	77.94186	237.0407	68.40661	234.8644	72.59277
237.3184	77.93773	237.0806	68.40434	234.9025	72.59165
237.3594	77.93305	237.1204	68.40286	234.9412	72.59053
237.4014	77.93166	237.1642	68.40189	234.983	72.58952
237.4394	77.93046	237.2027	68.40078	235.0263	72.58841
237.4745	77.92906	237.2383	68.40045	235.0666	72.58743
237.5172	77.93131	237.28	68.39937	235.107	72.58666
237.5593	77.93113	237.3236	68.39791	235.1499	72.58587
237.6018	77.92349	237.363	68.39687	235.1931	72.58483
237.6419	77.91804	237.4038	68.39641	235.2355	72.58388
237.6854	77.91455	237.4453	68.39548	235.2734	72.58196

237.7252	77.91216	237.4845	68.39461	235.3148	72.58074
237.7697	77.91288	237.5263	68.39323	235.356	72.57968
237.8089	77.91108	237.5687	68.39147	235.392	72.57819
237.8528	77.91314	237.6104	68.38978	235.4318	72.57733
237.8934	77.91277	237.6559	68.38841	235.4716	72.57689
237.9268	77.91125	237.6867	68.38759	235.516	72.57594
237.9703	77.91101	237.7305	68.38664	235.555	72.5753
238.0091	77.90805	237.7702	68.38615	235.5991	72.57393
238.0492	77.90292	237.8123	68.38554	235.6402	72.57184
238.0892	77.89983	237.8521	68.38371	235.6832	72.57117
238.1332	77.89675	237.893	68.38215	235.7238	72.57062
238.1734	77.89484	237.935	68.37983	235.7647	72.5702
238.2186	77.89286	237.9736	68.37844	235.8088	72.56905
238.2592	77.89097	238.0153	68.37802	235.8497	72.56703
238.2992	77.88899	238.058	68.37684	235.8915	72.56504
238.3426	77.88423	238.0997	68.37619	235.9323	72.56342
238.3838	77.88282	238.1325	68.37439	235.9703	72.56245
238.425	77.88356	238.1766	68.37249	236.005	72.5615
238.4588	77.88357	238.2165	68.37092	236.0472	72.56065
238.5002	77.88037	238.259	68.37025	236.0871	72.55921
238.5431	77.87983	238.3007	68.37052	236.1294	72.55853
238.5831	77.87764	238.3434	68.37031	236.1713	72.55839
238.6254	77.87609	238.3853	68.36884	236.2136	72.55712
238.6649	77.87547	238.4272	68.36689	236.2554	72.55626
238.708	77.87362	238.4681	68.36499	236.295	72.55459
238.7474	77.87138	238.51	68.362	236.3373	72.55249
238.7908	77.86624	238.552	68.36057	236.3785	72.55143
238.8321	77.86451	238.5844	68.35947	236.423	72.55027
238.8715	77.86542	238.6273	68.3575	236.4634	72.54892
238.9032	77.86348	238.669	68.35733	236.5058	72.54763
238.9464	77.85843	238.7101	68.35746	236.545	72.54632
238.989	77.8591	238.7521	68.35683	236.5887	72.54553
239.0303	77.85617	238.7931	68.35563	236.621	72.54492
239.0732	77.85035	238.8366	68.35321	236.6616	72.54374
239.1121	77.85121	238.8762	68.35134	236.7059	72.54314
239.1537	77.85121	238.9178	68.35059	236.7464	72.54179
239.1958	77.8498	238.9594	68.35004	236.7862	72.54061
239.2367	77.84717	239.0036	68.34984	236.8301	72.53924
239.2813	77.8426	239.0394	68.34827	236.8703	72.53749
239.324	77.83983	239.081	68.34673	236.9148	72.53623
239.356	77.83779	239.1182	68.34452	236.9532	72.53458
239.3949	77.83426	239.162	68.34245	236.9948	72.53362
239.4382	77.83396	239.2016	68.34061	237.0385	72.53219
239.4783	77.83097	239.2466	68.33823	237.081	72.53062
239.5222	77.82477	239.2871	68.33675	237.1224	72.52986
239.5641	77.82297	239.3282	68.33581	237.155	72.52883
239.6038	77.82049	239.372	68.33503	237.1975	72.52832
239.6449	77.8168	239.4118	68.33379	237.2383	72.52742
239.687	77.81714	239.4516	68.33334	237.282	72.52626
239.7282	77.81506	239.4846	68.33202	237.3255	72.52556
239.7691	77.81469	239.5266	68.33056	237.3655	72.52403
239.8024	77.81506	239.5681	68.32956	237.4067	72.52289
239.8436	77.81125	239.6101	68.32813	237.4486	72.52188
239.8839	77.81088	239.652	68.32627	237.4905	72.52027
239.9236	77.80969	239.6924	68.32327	237.5312	72.5188
239.9668	77.80555	239.7338	68.32059	237.5764	72.51726
240.0096	77.80515	239.7738	68.31831	237.6156	72.51665
240.0536	77.8032	239.8156	68.31719	237.6556	72.51563

240.0898	77.80056	239.8545	68.31865	237.6986	72.51443
240.131	77.79989	239.898	68.31955	237.74	72.51354
240.1735	77.79419	239.9309	68.3201	237.7715	72.51199
240.2207	77.79065	239.972	68.31932	237.8124	72.51024
240.2617	77.78809	240.0125	68.31707	237.8554	72.50927
240.304	77.78284	240.0545	68.31504	237.8977	72.50833
240.3322	77.78358	240.0951	68.31196	237.9405	72.50704
240.3741	77.78361	240.139	68.30982	237.9811	72.50614
240.419	77.78225	240.1796	68.30839	238.0235	72.50525
240.4602	77.78391	240.222	68.30705	238.0614	72.50433
240.499	77.78302	240.2606	68.30637	238.104	72.50349
240.5422	77.77696	240.3048	68.30584	238.1453	72.502
240.5872	77.77402	240.3465	68.30635	238.1907	72.50047
240.6267	77.77067	240.3798	68.30614	238.2306	72.49913
240.6673	77.76863	240.4198	68.30545	238.2736	72.49755
240.7091	77.76553	240.4633	68.3043	238.3136	72.49637
240.748	77.76065	240.5028	68.30234	238.3564	72.49534
240.7836	77.75832	240.545	68.3007	238.3911	72.49425
240.8244	77.75678	240.5855	68.29884	238.4307	72.49368
240.8693	77.7547	240.6262	68.29727	238.474	72.4927
240.9101	77.75533	240.6696	68.29526	238.5134	72.491
240.9525	77.75375	240.7102	68.29235	238.5563	72.48959
240.99	77.74881	240.7558	68.28989	238.5964	72.48712
241.0287	77.75033	240.7976	68.28839	238.6403	72.48476
241.0707	77.75126	240.8288	68.2872	238.6814	72.48363
241.114	77.75123	240.8726	68.28602	238.7222	72.4821
241.156	77.74661	240.9131	68.28577	238.765	72.481
241.1978	77.74186	240.9527	68.28495	238.8058	72.4808
241.2286	77.73855	240.9947	68.28339	238.8466	72.47987
241.2711	77.73276	241.0383	68.2832	238.8894	72.47914
241.3152	77.73242	241.0793	68.28121	238.9178	72.4787
241.3569	77.73559	241.121	68.27921	238.9611	72.47757
241.398	77.73485	241.1635	68.27747	239.0028	72.47672
241.442	77.73034	241.2008	68.27558	239.046	72.4757
241.4804	77.72698	241.2429	68.27386	239.0879	72.47488
241.5231	77.72318	241.2755	68.27253	239.1292	72.4743
241.5671	77.72011	241.3186	68.27083	239.1684	72.47334
241.6082	77.71926	241.3637	68.26897	239.212	72.47131
241.6472	77.71901	241.4044	68.26769	239.2541	72.46976
241.6838	77.71697	241.4444	68.26648	239.2939	72.46864
241.7237	77.71516	241.4871	68.2658	239.3346	72.46773
241.7668	77.71374	241.5297	68.26418	239.376	72.46713
241.807	77.71184	241.5711	68.26275	239.4184	72.46601
241.8505	77.70966	241.6134	68.2614	239.4603	72.46461
241.8899	77.7025	241.6535	68.26019	239.5015	72.46278
241.9322	77.69926	241.6961	68.25935	239.533	72.46172
241.9744	77.6984	241.7297	68.25779	239.5762	72.46056
242.015	77.69521	241.7722	68.25548	239.6162	72.45916
242.0553	77.6959	241.8128	68.25368	239.6598	72.45748
242.0982	77.69765	241.8533	68.25232	239.7006	72.45601
242.1392	77.69463	241.8972	68.25266	239.7432	72.45526
242.1785	77.69306	241.9386	68.25319	239.7808	72.4546
242.217	77.69125	241.9816	68.25079	239.824	72.45387
242.2538	77.6877	242.0219	68.24908	239.8674	72.45309
242.2945	77.68566	242.0642	68.24742	239.9094	72.45185
242.338	77.68401	242.1023	68.2465	239.9498	72.45048
242.3824	77.68282	242.1439	68.24799	239.9904	72.44958
242.4227	77.68136	242.1815	68.24795	240.0333	72.44806

242.462	77.67832	242.2208	68.24649	240.0746	72.44658
242.5044	77.67573	242.2606	68.24457	240.1172	72.44499
242.5467	77.67459	242.3018	68.24123	240.1494	72.44383
242.5889	77.67179	242.3422	68.23782	240.19	72.44282
242.6323	77.67112	242.3844	68.23559	240.2314	72.44265
242.6616	77.67105	242.427	68.23348	240.2732	72.44141
242.7026	77.66953	242.4692	68.23247	240.3172	72.43977
242.7413	77.66677	242.5107	68.23274	240.3574	72.43878
242.783	77.66399	242.5546	68.23206	240.3973	72.43621
242.8245	77.66266	242.5942	68.22987	240.439	72.43516
242.8665	77.65977	242.6271	68.22896	240.4784	72.43401
242.9096	77.65529	242.6695	68.22751	240.521	72.43241
242.9496	77.65247	242.7101	68.22552	240.564	72.43124
242.992	77.65125	242.7532	68.22432	240.6052	72.42974
243.0332	77.64923	242.792	68.22285	240.6463	72.42882
243.075	77.64789	242.8313	68.22091	240.6804	72.42764
243.1094	77.649	242.8768	68.21921	240.7209	72.42638
243.1504	77.64677	242.9182	68.21837	240.7608	72.42496
243.1899	77.64277	242.9602	68.2171	240.8031	72.42401
243.2334	77.63753	243.0017	68.2156	240.8444	72.42267
243.2774	77.63185	243.0464	68.21474	240.8865	72.42115
243.3209	77.62563	243.0789	68.21409	240.9242	72.41968
243.3599	77.62035	243.1172	68.21302	240.9702	72.41789
243.4032	77.62212	243.1613	68.21341	241.012	72.41659
243.4441	77.6234	243.2028	68.21117	241.0513	72.41583
243.4857	77.62071	243.2446	68.20984	241.0945	72.41473
243.5272	77.61959	243.2838	68.20802	241.1364	72.41394
243.5596	77.61736	243.3268	68.20462	241.1772	72.41281
243.6008	77.6158	243.3692	68.20221	241.2215	72.41133
243.6427	77.61481	243.4093	68.19985	241.2639	72.41063
243.686	77.61381	243.4498	68.19847	241.2968	72.40951
243.7282	77.61382	243.4918	68.19831	241.3384	72.40848
243.77	77.61049	243.5234	68.19857	241.3784	72.40726
243.8117	77.60654	243.5645	68.19847	241.416	72.40577
243.8539	77.60504	243.6088	68.19723	241.461	72.40422
243.8942	77.60377	243.6494	68.1956	241.5014	72.40306
243.9347	77.60229	243.6916	68.19338	241.5434	72.40197
243.9782	77.60023	243.7312	68.19117	241.5846	72.40084
244.0176	77.59833	243.7725	68.189	241.6288	72.39951
244.0575	77.59339	243.8113	68.18694	241.6703	72.39838
244.0908	77.5897	243.855	68.18651	241.711	72.39734
244.1333	77.58623	243.894	68.18536	241.7536	72.39622
244.1738	77.58279	243.9372	68.1843	241.7961	72.39492
244.2146	77.57941	243.9731	68.18348	241.8389	72.39326
244.2572	77.57528	244.0142	68.1815	241.8821	72.392
244.3008	77.57382	244.0576	68.18033	241.915	72.39068
244.3392	77.57403	244.0997	68.17885	241.9559	72.3896
244.3821	77.5713	244.1381	68.17655	241.9969	72.38895
244.4229	77.57124	244.181	68.17428	242.0376	72.3874
244.4641	77.57033	244.2214	68.17174	242.0822	72.38616
244.5058	77.56682	244.2653	68.16859	242.1235	72.38407
244.5403	77.56681	244.3022	68.167	242.1625	72.3817
244.5828	77.56484	244.3453	68.1671	242.2042	72.37997
244.6226	77.56233	244.3864	68.16518	242.2442	72.37879
244.6649	77.55995	244.423	68.1646	242.2897	72.37764
244.7072	77.55643	244.4634	68.16457	242.3305	72.37665
244.7486	77.55457	244.506	68.16205	242.3738	72.37531
244.7886	77.55167	244.5476	68.15973	242.4145	72.37391

244.83	77.54736	244.5887	68.15945	242.4483	72.37297
244.8699	77.54687	244.6292	68.1569	242.4899	72.37225
244.9127	77.5459	244.6703	68.15411	242.5332	72.37184
244.9528	77.54153	244.7142	68.15339	242.5722	72.37048
244.9887	77.54046	244.7551	68.15217	242.6164	72.3688
245.0309	77.54047	244.7981	68.15046	242.6574	72.36729
245.0697	77.53866	244.8383	68.14872	242.6982	72.36549
245.113	77.53766	244.8738	68.14761	242.7396	72.36419
245.1572	77.53708	244.9164	68.14556	242.7799	72.36352
245.1974	77.53583	244.9575	68.14436	242.8225	72.3618
245.237	77.532	244.9987	68.14465	242.8652	72.36065
245.2775	77.5285	245.0407	68.14378	242.9077	72.35981
245.3194	77.52858	245.0828	68.14224	242.9499	72.35894
245.3623	77.52476	245.1222	68.14063	242.9891	72.35833
245.4028	77.51844	245.1679	68.1379	243.0328	72.3567
245.4351	77.51737	245.2037	68.1371	243.0653	72.35527
245.4743	77.51533	245.2471	68.13472	243.1074	72.35361
245.516	77.51318	245.29	68.13194	243.1483	72.35216
245.559	77.51295	245.3225	68.13121	243.1888	72.35061
245.603	77.51244	245.3632	68.12856	243.2337	72.34981
245.6412	77.51111	245.4034	68.12635	243.2738	72.34896
245.682	77.50883	245.4452	68.12585	243.3153	72.34767
245.7274	77.50363	245.4879	68.12358	243.3588	72.34733
245.769	77.50085	245.5316	68.12185	243.4007	72.34593
245.8102	77.49862	245.5722	68.12091	243.4417	72.34446
245.8536	77.49476	245.6144	68.11956	243.4799	72.34259
245.8965	77.49633	245.6566	68.11806	243.523	72.34075
245.9387	77.49737	245.697	68.11713	243.5634	72.33904
245.9722	77.49452	245.7402	68.11485	243.6082	72.33764
246.0114	77.4926	245.774	68.11224	243.6504	72.3365
246.0518	77.48805	245.8157	68.11214	243.6828	72.33573
246.0937	77.48196	245.854	68.1107	243.7237	72.33484
246.1356	77.47955	245.8973	68.10896	243.7676	72.33338
246.1746	77.47799	245.9355	68.10818	243.8084	72.33194
246.2178	77.48006	245.9792	68.10646	243.8521	72.33058
246.2606	77.48087	246.018	68.10444	243.8926	72.32873
246.2997	77.47753	246.0632	68.10266	243.9365	72.32752
246.3432	77.47401	246.1056	68.10068	243.9792	72.32672
246.385	77.47264	246.147	68.0993	244.0198	72.32546
246.4186	77.47061	246.1875	68.09887	244.0615	72.32453
246.4608	77.46856	246.2195	68.09832	244.1027	72.32276
246.5012	77.46461	246.2629	68.09747	244.147	72.32129
246.5426	77.45864	246.3042	68.09523	244.1877	72.31979
246.5842	77.45345	246.3464	68.09281	244.2216	72.31822
246.6265	77.4516	246.389	68.08936	244.2619	72.31747
246.6664	77.45064	246.4303	68.08832	244.3037	72.31641
246.706	77.44833	246.4702	68.08655	244.3452	72.31491
246.7478	77.44622	246.5128	68.08481	244.3886	72.31347
246.7928	77.44516	246.5557	68.08442	244.4296	72.31155
246.8315	77.44524	246.5984	68.08148	244.4715	72.30991
246.8662	77.44479	246.6358	68.07964	244.5088	72.30843
246.9072	77.44307	246.67	68.07887	244.5506	72.30754
246.9499	77.44238	246.7133	68.07711	244.593	72.30628
246.9908	77.43951	246.754	68.07491	244.6362	72.30491
247.0309	77.43362	246.7937	68.07307	244.6778	72.30335
247.0741	77.43286	246.8382	68.07026	244.7201	72.30147
247.115	77.43047	246.8789	68.06949	244.761	72.30047
247.1572	77.42573	246.9219	68.06968	244.8022	72.29895

247.1987	77.42305	246.9664	68.06929	244.8362	72.29767
247.2405	77.42027	247.0063	68.069	244.8778	72.29655
247.2804	77.41693	247.0481	68.06782	244.9169	72.29525
247.3129	77.41479	247.091	68.06541	244.9594	72.29375
247.3552	77.41515	247.1224	68.06392	245.0024	72.29236
247.3976	77.41636	247.1626	68.06271	245.044	72.29104
247.4381	77.4131	247.2046	68.05933	245.0844	72.28946
247.4816	77.40828	247.2452	68.05742	245.1266	72.28769
247.522	77.40695	247.287	68.05489	245.1677	72.28639
247.5658	77.40466	247.3291	68.05133	245.2085	72.28497
247.6043	77.40264	247.3697	68.04998	245.2502	72.28325
247.647	77.39923	247.4108	68.04836	245.2904	72.28243
247.6889	77.39666	247.4526	68.0462	245.333	72.28162
247.7289	77.3941	247.494	68.04501	245.3755	72.28063
247.7719	77.38996	247.5367	68.04436	245.4165	72.28014
247.8134	77.39201	247.5694	68.04216	245.4495	72.27943
247.8464	77.39279	247.6106	68.04039	245.4907	72.2781
247.8881	77.38857	247.6512	68.03802	245.5305	72.27599
247.9293	77.38841	247.694	68.03465	245.5727	72.27409
247.972	77.38943	247.736	68.03325	245.6154	72.27183
248.0141	77.38847	247.7758	68.03387	245.6578	72.27055
248.0538	77.38695	247.8158	68.03398	245.6985	72.26943
248.0966	77.38199	247.8579	68.03301	245.7397	72.26767
248.1377	77.37604	247.8994	68.03135	245.783	72.26679
248.1814	77.37118	247.9429	68.02823	245.8227	72.26504
248.22	77.36914	247.982	68.02565	245.866	72.26334
248.2616	77.36693	248.0156	68.02422	245.9065	72.26211
248.2938	77.36754	248.0568	68.02139	245.9474	72.26049
248.3392	77.36358	248.098	68.02042	245.9796	72.25893
248.3805	77.35998	248.1407	68.01911	246.0214	72.25762
248.4214	77.36141	248.1827	68.01776	246.0611	72.25614
248.4646	77.35691	248.2241	68.01648	246.1064	72.25531
248.5081	77.35452	248.2679	68.01449	246.1497	72.25463
248.5482	77.35179	248.3088	68.01266	246.19	72.25327
248.5914	77.34567	248.3523	68.01087	246.2334	72.252
248.6305	77.34386	248.3953	68.00887	246.2708	72.24999
248.6709	77.34076	248.4341	68.00602	246.31	72.24836
248.7094	77.33986	248.4685	68.00458	246.3546	72.24756
248.7445	77.33982	248.5068	68.00207	246.398	72.2463
248.7876	77.33593	248.5496	68.00068	246.4378	72.24479
248.8296	77.33695	248.5924	67.99939	246.4781	72.24293
248.87	77.3368	248.6345	67.99739	246.5232	72.24088
248.9135	77.33205	248.678	67.99614	246.5644	72.23889
248.953	77.33084	248.7181	67.99535	246.596	72.2375
248.9974	77.32619	248.76	67.9926	246.6392	72.23635
249.0394	77.32239	248.8014	67.98946	246.6799	72.23564
249.0804	77.319	248.8433	67.9881	246.7196	72.2352
249.1208	77.31682	248.8888	67.98537	246.7599	72.23379
249.1638	77.31784	248.9218	67.98357	246.8026	72.23242
249.1966	77.31728	248.965	67.98246	246.8454	72.23076
249.2361	77.31458	249.0056	67.98004	246.8851	72.22866
249.2783	77.31463	249.0452	67.97695	246.9271	72.2274
249.3197	77.31306	249.0898	67.97574	246.9713	72.22586
249.3582	77.30662	249.1288	67.97436	247.0139	72.22434
249.4039	77.30239	249.1729	67.97289	247.0572	72.22313
249.444	77.30159	249.2129	67.97116	247.0936	72.22198
249.4867	77.29893	249.2562	67.96833	247.1366	72.22027
249.5291	77.2988	249.2943	67.96547	247.1798	72.21896

249.5683	77.30169	249.3385	67.96401	247.2142	72.21726
249.6069	77.30122	249.3712	67.96287	247.254	72.21478
249.6468	77.29793	249.4143	67.96218	247.2966	72.21294
249.6878	77.29304	249.4568	67.96191	247.3406	72.21126
249.7202	77.29222	249.4975	67.96	247.3809	72.20973
249.7642	77.29031	249.5366	67.9573	247.4234	72.20903
249.8056	77.28578	249.5792	67.95562	247.4684	72.20826
249.8485	77.28402	249.6221	67.95307	247.5077	72.20695
249.8917	77.28249	249.661	67.95103	247.5498	72.20596
249.9302	77.27904	249.7044	67.95045	247.5886	72.20366
249.9734	77.27666	249.7453	67.9486	247.6311	72.20248
250.0159	77.27517	249.7856	67.94569	247.6741	72.20105
250.0588	77.27302	249.8192	67.94415	247.716	72.19873
250.0986	77.27046	249.8619	67.9414	247.7498	72.19786
250.1406	77.26639	249.9024	67.93945	247.7927	72.19653
250.1733	77.26513	249.9482	67.93913	247.8342	72.19398
250.2153	77.26305	249.9894	67.93744	247.8742	72.19273
250.2566	77.26005	250.0266	67.93527	247.9122	72.19203
250.2985	77.26025	250.0691	67.93288	247.9567	72.19013
250.3401	77.26	250.1104	67.93018	247.9955	72.18913
250.3798	77.25573	250.157	67.9287	248.0402	72.18744
250.4254	77.25188	250.1967	67.92698	248.0804	72.18564
250.4635	77.24557	250.2366	67.92474	248.1216	72.18406
250.5068	77.2412	250.2681	67.92378	248.1662	72.18203
250.5487	77.2385	250.31	67.92168	248.2085	72.18026
250.5898	77.2334	250.3516	67.92006	248.2504	72.17875
250.622	77.23299	250.3922	67.91866	248.2894	72.17707
250.6614	77.23174	250.4342	67.91624	248.332	72.17562
250.7045	77.22705	250.4751	67.91288	248.364	72.17508
250.7455	77.22696	250.518	67.91052	248.4067	72.174
250.7867	77.22718	250.5589	67.90781	248.45	72.17238
250.8304	77.22283	250.6016	67.90497	248.4897	72.17079
250.8735	77.22253	250.6422	67.90345	248.532	72.16873
250.9138	77.22039	250.6851	67.9014	248.5725	72.16654
250.953	77.21575	250.718	67.9	248.6151	72.16517
250.9969	77.21202	250.7604	67.89771	248.6567	72.16391
251.038	77.20925	250.8014	67.89496	248.6989	72.16315
251.0708	77.20847	250.8447	67.89347	248.7416	72.1617
251.1126	77.20581	250.8862	67.8925	248.7842	72.16027
251.1549	77.20421	250.9248	67.89164	248.824	72.15817
251.196	77.20415	250.9661	67.88998	248.8672	72.15597
251.238	77.20127	251.0083	67.88738	248.9089	72.15407
251.2793	77.19843	251.0486	67.88269	248.9524	72.15229
251.3216	77.19657	251.0939	67.8799	248.9827	72.15126
251.3644	77.19495	251.1337	67.88005	249.0249	72.14936
251.4076	77.19402	251.1652	67.87905	249.0657	72.14827
251.4462	77.19226	251.2089	67.87765	249.1087	72.14724
251.4876	77.18891	251.2515	67.87761	249.1523	72.14584
251.53	77.18597	251.2925	67.87579	249.1934	72.14442
251.5706	77.18343	251.3342	67.87305	249.2352	72.14288
251.6024	77.17927	251.3775	67.87107	249.2774	72.14036
251.6433	77.17979	251.4182	67.86788	249.3197	72.13844
251.685	77.17944	251.4584	67.86367	249.3599	72.13688
251.7266	77.17662	251.4995	67.86163	249.4022	72.1347
251.7686	77.17605	251.5394	67.85969	249.4438	72.1333
251.8106	77.17365	251.584	67.85758	249.486	72.1317
251.8514	77.1699	251.6179	67.85681	249.517	72.13029
251.8938	77.16366	251.6596	67.85441	249.561	72.12858

251.9342	77.16132	251.7003	67.85189	249.6025	72.127
251.9748	77.16068	251.7434	67.85035	249.6438	72.12534
252.0188	77.1578	251.7839	67.84722	249.6875	72.12351
252.053	77.15549	251.8295	67.8441	249.7266	72.12187
252.0913	77.15459	251.8707	67.84195	249.7697	72.11981
252.1347	77.15319	251.9108	67.83771	249.8113	72.11767
252.178	77.15083	251.9512	67.835	249.8539	72.11632
252.2205	77.14769	251.9905	67.83408	249.8939	72.11465
252.26	77.14603	252.0367	67.83166	249.9363	72.11322
252.303	77.14266	252.0683	67.831	249.9793	72.11173
252.3453	77.1408	252.1102	67.82937	250.0213	72.10989
252.3853	77.14181	252.1522	67.82469	250.0642	72.10854
252.428	77.13825	252.1937	67.82127	250.1008	72.10689
252.4678	77.13528	252.2371	67.81958	250.1386	72.10581
252.5024	77.13336	252.2761	67.81803	250.178	72.10441
252.5437	77.13001	252.32	67.81912	250.2194	72.1028
252.5848	77.12832	252.3617	67.81849	250.2625	72.10097
252.6286	77.12399	252.4006	67.81573	250.3048	72.09903
252.6694	77.11856	252.4417	67.81304	250.346	72.09742
252.7122	77.11473	252.4821	67.80994	250.3857	72.09609
252.7547	77.10943	252.5141	67.80878	250.4266	72.09475
252.7962	77.1096	252.5544	67.80685	250.4705	72.09346
252.8385	77.1091	252.5983	67.80501	250.512	72.0917
252.8772	77.10722	252.6392	67.80259	250.5555	72.08943
252.9192	77.10296	252.6797	67.79998	250.5956	72.08741
252.9552	77.09908	252.72	67.7982	250.6364	72.08518
252.9958	77.09713	252.7615	67.79603	250.6797	72.08304
253.0383	77.09689	252.8018	67.79457	250.7228	72.08099
253.0792	77.0966	252.8469	67.79284	250.7539	72.07915
253.1184	77.09465	252.8869	67.79003	250.7962	72.07762
253.163	77.09184	252.931	67.78775	250.8381	72.07596
253.2034	77.08629	252.9629	67.7857	250.8773	72.0743
253.2449	77.08419	253.0033	67.78069	250.9184	72.07281
253.2869	77.08403	253.0484	67.77861	250.9618	72.07098
253.3276	77.08185	253.0892	67.77655	251.0005	72.0698
253.3713	77.07928	253.1314	67.77371	251.0438	72.06837
253.4124	77.07956	253.1741	67.77296	251.088	72.06688
253.4538	77.07859	253.2148	67.76964	251.1258	72.06484
253.4868	77.07669	253.2556	67.76577	251.1696	72.06235
253.5263	77.07456	253.2975	67.76269	251.2112	72.05983
253.5676	77.07345	253.3374	67.75831	251.2547	72.05795
253.6089	77.07076	253.3795	67.75456	251.2884	72.05662
253.6506	77.06468	253.4148	67.75306	251.3305	72.05461
253.691	77.06228	253.4568	67.75103	251.3701	72.05287
253.7352	77.06015	253.4963	67.75011	251.4113	72.05108
253.7761	77.05684	253.5388	67.75088	251.4552	72.04935
253.82	77.05566	253.5829	67.74933	251.4963	72.04779
253.8594	77.05365	253.6238	67.74701	251.5398	72.04623
253.9012	77.05267	253.667	67.7449	251.5823	72.04444
253.9296	77.05044	253.7046	67.74142	251.6236	72.04246
253.9717	77.04569	253.7482	67.73956	251.6641	72.04076
254.0136	77.0443	253.79	67.73805	251.7066	72.03899
254.0556	77.04089	253.8321	67.73515	251.7493	72.03696
254.0968	77.03638	253.8639	67.73294	251.7924	72.03504
254.1399	77.03539	253.9066	67.73043	251.835	72.03312
254.1813	77.03259	253.9483	67.72681	251.8756	72.03139
254.2228	77.02888	253.9884	67.72298	251.9072	72.03043
254.2637	77.02567	254.0306	67.7216	251.949	72.02883

254.3059	77.02258	254.0734	67.71721	251.9912	72.02744
254.3447	77.02245	254.1162	67.71384	252.0346	72.02549
254.3798	77.02215	254.1583	67.71092	252.0757	72.0236
254.4215	77.01852	254.2008	67.706	252.118	72.02151
254.462	77.01915	254.2416	67.70331	252.1591	72.01919
254.5065	77.01741	254.283	67.70179	252.1994	72.01687
254.5492	77.0119	254.3171	67.69963	252.2434	72.01425
254.5884	77.00757	254.3584	67.69775	252.2863	72.01205
254.6282	77.00287	254.3984	67.69568	252.3274	72.01039
254.6712	76.99689	254.4404	67.69229	252.3691	72.00846
254.7131	76.99332	254.4846	67.69096	252.41	72.00651
254.7522	76.99248	254.527	67.68855	252.4538	72.00487
254.7932	76.99255	254.5644	67.6861	252.4966	72.00319
254.8296	76.99148	254.6067	67.68459	252.5293	72.00242
254.87	76.98979	254.6485	67.68308	252.5704	72.00138
254.907	76.98993	254.6895	67.68117	252.6131	72.00025
254.9519	76.98494	254.7324	67.6786	252.6551	71.99865
254.9912	76.98079	254.7632	67.67635	252.6944	71.99639
255.0333	76.97866	254.8077	67.67112	252.7369	71.99422
255.0794	76.97539	254.8495	67.66773	252.7773	71.99193
255.1193	76.97226	254.8898	67.66535	252.8198	71.9896
255.1624	76.97229	254.9326	67.66195	252.8647	71.98759
255.2039	76.97027	254.9716	67.65894	252.9058	71.98537
255.2425	76.9676	255.0144	67.65524	252.9475	71.98249
255.2852	76.96397	255.0566	67.65197	252.9878	71.98085
255.3256	76.96076	255.0998	67.65066	253.0303	71.9791
255.3602	76.95888	255.142	67.64886	253.0624	71.97748
255.4013	76.95579	255.1832	67.64653	253.106	71.97628
255.4439	76.95392	255.2161	67.64558	253.1469	71.97434
255.4864	76.95152	255.2592	67.64222	253.1896	71.97251
255.529	76.95001	255.2958	67.63808	253.2307	71.97046
255.5701	76.94655	255.341	67.63546	253.2749	71.96832
255.6118	76.94291	255.3808	67.6312	253.3154	71.96621
255.6553	76.93942	255.4244	67.62686	253.356	71.96403
255.6934	76.93384	255.4658	67.62559	253.3973	71.96173
255.7354	76.92631	255.5074	67.62359	253.4402	71.95986
255.7794	76.92571	255.5512	67.62094	253.4816	71.95746
255.8126	76.9251	255.5908	67.61906	253.5224	71.95576
255.8547	76.91777	255.6331	67.61515	253.5656	71.95358
255.895	76.91421	255.664	67.61248	253.6062	71.95136
255.937	76.91085	255.7112	67.61017	253.6504	71.94971
255.9782	76.90887	255.7525	67.60721	253.6823	71.94768
256.0192	76.90669	255.7915	67.60385	253.7252	71.94551
256.0601	76.90557	255.8352	67.60225	253.7631	71.94389
256.1007	76.90091	255.8764	67.59906	253.8063	71.94212
256.1436	76.89356	255.9193	67.59534	253.8484	71.94
256.185	76.89065	255.9605	67.59376	253.8905	71.93866
256.229	76.88919	256.0016	67.59015	253.9304	71.9368
256.262	76.88803	256.044	67.58706	253.9726	71.93483
256.3019	76.88324	256.0873	67.58469	254.0145	71.93286
256.3448	76.88001	256.1146	67.58147	254.0568	71.93062
256.3884	76.87586	256.1572	67.57708	254.0981	71.92844
256.4293	76.87245	256.2012	67.57484	254.1397	71.92649
256.471	76.86945	256.2417	67.5726	254.1825	71.92424
256.5121	76.86548	256.2834	67.57133	254.2253	71.92223
256.552	76.86287	256.3239	67.56981	254.2682	71.91959
256.5935	76.85896	256.3668	67.56676	254.2976	71.91715
256.6368	76.85746	256.4089	67.56451	254.3413	71.91483

256.68	76.85454	256.4482	67.56254	254.3813	71.91238
256.7128	76.8506	256.4916	67.55902	254.4256	71.9105
256.7524	76.84761	256.532	67.55591	254.4651	71.90911
256.7902	76.8435	256.5652	67.5542	254.5081	71.90711
256.8332	76.84241	256.6076	67.55067	254.549	71.90507
256.8785	76.83791	256.6489	67.54794	254.5902	71.90319
256.9163	76.83589	256.6932	67.54573	254.6332	71.90098
256.9606	76.83491	256.7356	67.54199	254.6767	71.89856
257.0001	76.83061	256.7784	67.53682	254.718	71.89651
257.042	76.83066	256.8198	67.53307	254.757	71.89386
257.0856	76.82658	256.8634	67.52842	254.7962	71.89172
257.1265	76.82161	256.9037	67.52503	254.8329	71.8902
257.1667	76.81892	256.944	67.52164	254.8726	71.8876
257.2121	76.81467	256.9873	67.51782	254.9146	71.88617
257.2434	76.81302	257.0237	67.51631	254.9575	71.88377
257.2881	76.8128	257.0614	67.51396	254.9987	71.88154
257.3256	76.81068	257.1064	67.51207	255.038	71.87941
257.3702	76.81127	257.1418	67.50889	255.0825	71.87703
257.4098	76.81227	257.1844	67.50419	255.1228	71.8749
257.4531	76.80879	257.227	67.50014	255.1664	71.87269
257.4918	76.80643	257.2665	67.49746	255.2069	71.87038
257.5356	76.80351	257.307	67.49667	255.2489	71.86773
257.5752	76.79988	257.3512	67.49566	255.292	71.86535
257.6152	76.79708	257.393	67.49338	255.3327	71.86298
257.658	76.7913	257.4358	67.48945	255.3754	71.86065
257.6915	76.78855	257.4684	67.48529	255.4172	71.85851
257.7326	76.78672	257.5112	67.48118	255.448	71.85708
257.7748	76.78197	257.5516	67.47645	255.4918	71.85504
257.817	76.78081	257.5941	67.47202	255.5314	71.85263
257.8575	76.78061	257.6348	67.46907	255.576	71.85037
257.9011	76.77965	257.6768	67.46531	255.6189	71.84707
257.9432	76.77771	257.719	67.46149	255.66	71.84388
257.985	76.77657	257.7622	67.45967	255.7034	71.84124
258.0253	76.77611	257.8086	67.45729	255.7453	71.8382
258.068	76.77222	257.8455	67.45538	255.786	71.83621
258.1061	76.76971	257.8882	67.45365	255.8291	71.83464
258.1409	76.76811	257.9191	67.45074	255.8702	71.83269
258.1836	76.76183	257.9619	67.44672	255.912	71.83043
258.2252	76.75732	258.0039	67.44314	255.9542	71.82805
258.2683	76.75439	258.0475	67.43937	255.9934	71.82499
258.31	76.75186	258.0892	67.4356	256.0356	71.82226
258.3473	76.75024	258.1289	67.43191	256.0699	71.82049
258.3902	76.74913	258.1706	67.42706	256.1121	71.81741
258.433	76.74666	258.2123	67.42307	256.1537	71.8152
258.4722	76.74479	258.2544	67.421	256.1989	71.81289
258.5139	76.74282	258.2958	67.4168	256.24	71.81101
258.5559	76.73938	258.3347	67.41339	256.2812	71.80895
258.5885	76.73746	258.3718	67.41177	256.3249	71.80642
258.6289	76.73645	258.4125	67.40727	256.3649	71.80355
258.6724	76.73332	258.4506	67.404	256.4051	71.80061
258.7154	76.73092	258.4972	67.40139	256.4498	71.79804
258.7584	76.72923	258.5391	67.39651	256.4905	71.79567
258.7997	76.72776	258.5796	67.39274	256.5316	71.79352
258.838	76.72768	258.6208	67.38897	256.5718	71.79124
258.8783	76.72451	258.6614	67.38577	256.605	71.78925
258.9199	76.7219	258.7055	67.38376	256.6472	71.78689
258.964	76.72031	258.7453	67.37942	256.6907	71.78496
259.0086	76.71827	258.7902	67.37585	256.7324	71.7825

259.0482	76.71623	258.821	67.37327	256.7746	71.78035
259.0855	76.71301	258.8603	67.37047	256.8137	71.77757
259.1226	76.71027	258.9039	67.36839	256.8565	71.77483
259.1658	76.70476	258.9468	67.36595	256.9001	71.77192
259.2074	76.70078	258.988	67.36106	256.9419	71.7685
259.2488	76.69766	259.0308	67.35563	256.9828	71.76592
259.2887	76.69206	259.0726	67.35058	257.0241	71.76274
259.3298	76.68764	259.1146	67.34618	257.0632	71.75935
259.3743	76.68492	259.1555	67.34264	257.1066	71.75681
259.4128	76.68379	259.1975	67.34049	257.15	71.75391
259.4526	76.6826	259.239	67.33752	257.19	71.75127
259.4958	76.68059	259.2735	67.33445	257.2214	71.74986
259.5365	76.68047	259.3156	67.33206	257.2638	71.74747
259.5706	76.67909	259.3562	67.32826	257.305	71.74482
259.6121	76.67831	259.3978	67.32338	257.353	71.74185
259.6536	76.67664	259.4384	67.32039	257.39	71.73888
259.6975	76.6739	259.4824	67.31758	257.4333	71.73609
259.738	76.66956	259.5249	67.31391	257.4748	71.73354
259.779	76.66464	259.5674	67.31037	257.5201	71.73142
259.8204	76.65997	259.6092	67.30605	257.5617	71.72889
259.8614	76.65324	259.653	67.30208	257.6036	71.7267
259.9023	76.64997	259.6948	67.29686	257.6434	71.7237
259.9451	76.64673	259.7236	67.29289	257.6861	71.72071
259.989	76.64314	259.7659	67.28724	257.7295	71.71806
260.0217	76.64048	259.8084	67.28281	257.768	71.71491
260.0642	76.63786	259.8531	67.27898	257.8093	71.71199
260.106	76.63662	259.8927	67.27591	257.8469	71.70969
260.1487	76.63882	259.9368	67.27353	257.8896	71.70632
260.188	76.64021	259.978	67.26927	257.9312	71.70289
260.231	76.63796	260.0194	67.26415	257.9716	71.70026
260.2727	76.63233	260.0606	67.26037	258.0113	71.69766
260.3134	76.62593	260.1042	67.25486	258.0582	71.69594
260.357	76.62243	260.1463	67.24967	258.0996	71.69375
260.3963	76.61791	260.1774	67.24761	258.1416	71.69089
260.4394	76.61631	260.2194	67.24395	258.1816	71.68787
260.4715	76.61782	260.2567	67.24169	258.2256	71.68428
260.5128	76.61645	260.3042	67.23849	258.2656	71.68133
260.5538	76.61423	260.3435	67.23388	258.3058	71.6785
260.5972	76.61377	260.3861	67.23023	258.3513	71.67555
260.6378	76.60922	260.4267	67.2273	258.382	71.67306
260.6797	76.60395	260.4674	67.22347	258.4268	71.66978
260.7237	76.60119	260.5133	67.22025	258.466	71.66663
260.7631	76.59715	260.5532	67.21683	258.5106	71.66379
260.8046	76.59171	260.5963	67.21119	258.5523	71.66066
260.8455	76.58776	260.6294	67.20777	258.5937	71.65864
260.8887	76.58288	260.6687	67.20437	258.6358	71.65574
260.9312	76.57953	260.7113	67.19938	258.6788	71.6528
260.9713	76.57742	260.7524	67.19439	258.7203	71.64998
261.0047	76.57519	260.795	67.19015	258.7617	71.64627
261.0482	76.57587	260.836	67.18476	258.8032	71.64312
261.087	76.57389	260.8795	67.18111	258.843	71.64027
261.1308	76.56786	260.9212	67.17827	258.8842	71.63728
261.1718	76.56402	260.9655	67.17341	258.9275	71.63426
261.2124	76.55874	261.006	67.16997	258.9674	71.63163
261.2531	76.55473	261.0497	67.16561	259.0022	71.62886
261.297	76.55265	261.0848	67.16022	259.041	71.62571
261.3376	76.55165	261.125	67.15559	259.085	71.62244
261.3792	76.54926	261.1692	67.15167	259.1277	71.61874

261.4208	76.54455	261.2104	67.14627	259.1712	71.6158
261.4552	76.54208	261.2514	67.14334	259.2142	71.61258
261.4932	76.54111	261.2924	67.13969	259.2535	71.6093
261.535	76.53837	261.3327	67.13534	259.297	71.6057
261.5785	76.53491	261.3772	67.13005	259.3371	71.60188
261.6207	76.53188	261.419	67.12417	259.3796	71.59807
261.6639	76.52864	261.4603	67.11955	259.4206	71.59473
261.7064	76.52396	261.501	67.11444	259.4624	71.59165
261.7476	76.52223	261.535	67.11175	259.5051	71.5885
261.789	76.52083	261.577	67.10795	259.5454	71.58519
261.8291	76.51611	261.6176	67.10433	259.5883	71.58178
261.8697	76.51179	261.6618	67.09975	259.6214	71.57927
261.9054	76.50831	261.7027	67.09428	259.6637	71.57595
261.9468	76.50705	261.7451	67.09075	259.7064	71.57297
261.989	76.50604	261.7844	67.0859	259.7494	71.56978
262.0283	76.50118	261.8274	67.08136	259.7894	71.56634
262.0701	76.4975	261.8706	67.07715	259.8309	71.56346
262.1133	76.49563	261.91	67.07012	259.8739	71.56023
262.1574	76.494	261.9516	67.06395	259.9153	71.55757
262.1982	76.4951	261.9881	67.06052	259.9569	71.55458
262.2368	76.49517	262.0295	67.05535	260.002	71.55044
262.2783	76.49181	262.0723	67.05187	260.0438	71.54625
262.32	76.48624	262.1152	67.04957	260.084	71.54189
262.3517	76.47961	262.1576	67.04422	260.1256	71.53771
262.397	76.47448	262.1998	67.03993	260.1595	71.53484
262.4392	76.46727	262.2412	67.03668	260.2026	71.5316
262.4805	76.46284	262.2838	67.03066	260.2458	71.52809
262.5196	76.46051	262.3242	67.02577	260.2876	71.52449
262.5628	76.46003	262.3664	67.0207	260.3307	71.52081
262.6049	76.4602	262.4074	67.01407	260.3698	71.51687
262.644	76.45772	262.4416	67.00994	260.4105	71.5131
262.6852	76.45416	262.482	67.00609	260.4525	71.50866
262.7258	76.45085	262.5242	67.00102	260.4942	71.50448
262.7691	76.44647	262.5658	66.99629	260.536	71.50104
262.8107	76.44375	262.6101	66.99004	260.5774	71.49779
262.8507	76.44092	262.6512	66.98438	260.6194	71.49463
262.8838	76.43728	262.6921	66.98159	260.6623	71.49117
262.9235	76.43528	262.7362	66.97857	260.7026	71.48729
262.9643	76.42971	262.7772	66.97493	260.7433	71.48349
263.0036	76.4261	262.8169	66.96996	260.777	71.4812
263.0492	76.42474	262.8599	66.96449	260.8202	71.4777
263.0907	76.42367	262.8902	66.95947	260.862	71.47407
263.1335	76.41859	262.9332	66.95482	260.903	71.46998
263.1734	76.41308	262.9736	66.9501	260.9437	71.4655
263.215	76.40663	263.0185	66.94353	260.9891	71.46177
263.2573	76.39882	263.0594	66.93839	261.0281	71.45848
263.2989	76.39225	263.0998	66.93439	261.0718	71.45524
263.3332	76.39097	263.1388	66.92856	261.114	71.45208
263.3748	76.38893	263.1821	66.92347	261.1552	71.44879
263.4191	76.38275	263.2256	66.91802	261.2014	71.44486
263.461	76.38158	263.2659	66.9127	261.2405	71.44067
263.5015	76.38282	263.3086	66.91094	261.2839	71.43612
263.5427	76.38191	263.3387	66.90781	261.323	71.43112
263.5839	76.37849	263.3825	66.90239	261.3656	71.42697
263.6277	76.37523	263.4246	66.89595	261.3997	71.42371
263.67	76.37066	263.4686	66.89046	261.4406	71.41984
263.7087	76.36686	263.5096	66.88457	261.4804	71.41683
263.7507	76.36214	263.5514	66.88067	261.524	71.41285

263.7878	76.35957	263.5942	66.87678	261.5666	71.40888
263.8303	76.35771	263.6378	66.86998	261.6082	71.40429
263.8705	76.35272	263.6764	66.86516	261.6517	71.39962
263.9142	76.34615	263.7189	66.85941	261.6917	71.39485
263.955	76.34301	263.7615	66.85342	261.7345	71.39067
263.9949	76.3377	263.7968	66.84886	261.7758	71.38652
264.0405	76.33337	263.838	66.84265	261.8171	71.38162
264.0818	76.33252	263.8787	66.83769	261.8584	71.37692
264.122	76.33061	263.9231	66.83473	261.9005	71.37192
264.1624	76.32535	263.9606	66.83025	261.9332	71.36899
264.2041	76.31695	264.004	66.82494	261.9787	71.36488
264.2373	76.31369	264.0427	66.81994	262.0203	71.36136
264.28	76.31297	264.0844	66.8144	262.0612	71.35767
264.3207	76.31098	264.1302	66.8093	262.1025	71.35351
264.3628	76.31278	264.1737	66.80403	262.1408	71.34949
264.4056	76.31077	264.2165	66.79683	262.1789	71.3449
264.4467	76.30556	264.2503	66.79088	262.2209	71.34081
264.4895	76.30229	264.2936	66.78555	262.2632	71.33611
264.5302	76.296	264.3323	66.77959	262.3059	71.33192
264.5719	76.29336	264.3745	66.77448	262.3498	71.32802
264.6128	76.28982	264.4181	66.76967	262.3873	71.32331
264.6533	76.28253	264.4589	66.76388	262.4298	71.31869
264.6958	76.2776	264.5012	66.7584	262.471	71.31368
264.7392	76.27209	264.5432	66.75468	262.5119	71.30843
264.7728	76.267	264.584	66.7481	262.5447	71.3042
264.8154	76.26348	264.6287	66.74228	262.5886	71.29929
264.8575	76.25987	264.6685	66.73712	262.6299	71.29466
264.8988	76.25809	264.7044	66.72997	262.6719	71.28974
264.9394	76.25729	264.7465	66.72346	262.7126	71.28529
264.9819	76.25799	264.7856	66.7176	262.756	71.28089
265.0229	76.25738	264.8279	66.71226	262.7952	71.27652
265.0678	76.25318	264.8707	66.70833	262.8379	71.27211
265.1066	76.24866	264.9093	66.7034	262.8828	71.26693
265.1483	76.24243	264.9535	66.69754	262.9246	71.26138
265.1906	76.23676	264.9967	66.69174	262.9674	71.2563
265.2238	76.23447	265.0394	66.68615	263.0081	71.25156
265.2681	76.23364	265.08	66.67856	263.0519	71.24673
265.3064	76.2332	265.1226	66.67188	263.0918	71.24187
265.35	76.23201	265.1547	66.66697	263.133	71.2361
265.388	76.22903	265.1967	66.65961	263.168	71.23164
265.4328	76.22214	265.2411	66.65458	263.2095	71.22624
265.4717	76.21731	265.2818	66.64923	263.2515	71.22112
265.5135	76.21174	265.3248	66.64102	263.2934	71.21596
265.5564	76.20742	265.3661	66.63493	263.3327	71.21105
265.597	76.2067	265.4102	66.62952	263.3758	71.20578
265.6386	76.20231	265.4504	66.6252	263.4155	71.20099
265.6738	76.20016	265.4904	66.62319	263.458	71.19626
265.7148	76.19893	265.5337	66.61753	263.4992	71.19119
265.7558	76.19406	265.5715	66.60974	263.5418	71.18626
265.799	76.18724	265.6072	66.60394	263.5831	71.18133
265.8393	76.18147	265.6478	66.59514	263.6268	71.176
265.8827	76.17701	265.6926	66.58761	263.6668	71.17064
265.922	76.17403	265.7344	66.58432	263.7009	71.1664
265.9631	76.17292	265.7775	66.57805	263.7432	71.16073
266.0082	76.16805	265.8178	66.57141	263.784	71.15508
266.0484	76.16567	265.8581	66.56656	263.8264	71.14929
266.091	76.16185	265.9015	66.55949	263.8664	71.14427
266.1218	76.15866	265.9456	66.55304	263.9116	71.1389

266.1639	76.15607	265.9865	66.54781	263.9542	71.13372
266.2059	76.1518	266.028	66.54152	263.9945	71.12802
266.2448	76.14318	266.0639	66.5368	264.0344	71.12225
266.2906	76.13346	266.1038	66.5323	264.0781	71.11678
266.3309	76.12895	266.1429	66.52639	264.1172	71.11061
266.3691	76.12436	266.1877	66.51949	264.159	71.10468
266.4124	76.12324	266.2314	66.51248	264.2017	71.09835
266.4557	76.12338	266.2695	66.50586	264.2444	71.09197
266.4955	76.11844	266.313	66.50114	264.286	71.08654
266.5405	76.11328	266.3562	66.49642	264.3203	71.08252
266.5822	76.10828	266.3948	66.49022	264.3633	71.07699
266.6246	76.10047	266.439	66.48488	264.4044	71.07084
266.6562	76.09318	266.4808	66.47715	264.4447	71.06416
266.698	76.09045	266.5145	66.47075	264.4859	71.05723
266.7385	76.08796	266.5568	66.46411	264.5295	71.05091
266.7819	76.08501	266.6011	66.45539	264.5709	71.04512
266.8248	76.08819	266.6399	66.44774	264.6123	71.03924
266.8646	76.08177	266.6844	66.44213	264.6539	71.03349
266.9069	76.07287	266.7247	66.43583	264.6948	71.02728
266.9478	76.0663	266.768	66.42978	264.7374	71.02058
266.9914	76.05835	266.8128	66.4245	264.7774	71.01442
267.0332	76.05627	266.8546	66.4156	264.8222	71.00826
267.0716	76.05495	266.8955	66.4085	264.8644	71.00223
267.1062	76.05263	266.938	66.40179	264.9043	70.99599
267.147	76.0525	266.9725	66.39396	264.9394	70.99108
267.1887	76.04759	267.0132	66.38765	264.981	70.98506
267.2272	76.04191	267.056	66.38084	265.0217	70.97848
267.2714	76.04011	267.0962	66.37286	265.0661	70.97222
267.3137	76.03664	267.1375	66.36738	265.1064	70.96522
267.3555	76.03474	267.1805	66.36107	265.1496	70.95753
267.3978	76.03202	267.2225	66.35428	265.1908	70.95039
267.4397	76.02739	267.2637	66.34851	265.2332	70.94402
267.4808	76.02278	267.306	66.34232	265.2758	70.93794
267.5271	76.01685	267.3481	66.3359	265.32	70.93247
267.5579	76.01026	267.388	66.33044	265.3608	70.92665
267.5986	76.00374	267.4233	66.32504	265.4062	70.92022
267.6404	76.00016	267.4654	66.31757	265.4492	70.91399
267.681	75.99603	267.5088	66.31102	265.4809	70.90836
267.7272	75.99281	267.5505	66.30174	265.5216	70.90169
267.7638	75.99386	267.5932	66.29401	265.5652	70.89474
267.804	75.98983	267.6352	66.28585	265.6076	70.8888
267.8472	75.98283	267.6759	66.27677	265.6513	70.88209
267.8896	75.9783	267.7191	66.2704	265.6902	70.8754
267.9312	75.97253	267.7608	66.26535	265.7325	70.86873
267.9748	75.96959	267.8018	66.25857	265.7756	70.86104
268.0104	75.96681	267.845	66.25214	265.8181	70.85381
268.0514	75.96502	267.88	66.24763	265.8628	70.8465
268.0894	75.96142	267.9199	66.23973	265.906	70.83954
268.1344	75.95215	267.9638	66.23169	265.9481	70.83238
268.1758	75.94462	268.006	66.22548	265.9877	70.82522
268.2169	75.94201	268.047	66.21826	266.0318	70.81841
268.2578	75.93873	268.0878	66.20978	266.0762	70.81088
268.2976	75.93486	268.1309	66.2047	266.1091	70.80521
268.3406	75.93268	268.1744	66.19731	266.1505	70.79842
268.3855	75.92742	268.215	66.18972	266.193	70.79163
268.4275	75.92258	268.2585	66.18315	266.2346	70.78542
268.4691	75.91523	268.2996	66.1745	266.2784	70.77848
268.5143	75.90785	268.334	66.16808	266.321	70.77144

268.5431	75.90601	268.3764	66.16063	266.3629	70.76417
268.587	75.89865	268.4188	66.15232	266.4041	70.75642
268.6274	75.89319	268.4618	66.14405	266.4492	70.74886
268.6701	75.89091	268.5061	66.13746	266.4895	70.74179
268.7135	75.88691	268.5482	66.12964	266.532	70.73384
268.7553	75.88394	268.5913	66.12105	266.5724	70.72572
268.797	75.87962	268.6316	66.11284	266.6142	70.71783
268.8386	75.87761	268.6719	66.10466	266.6582	70.70898
268.8794	75.87195	268.7178	66.09862	266.7002	70.70076
268.923	75.86443	268.7576	66.0935	266.7328	70.69408
268.9636	75.85982	268.7917	66.08763	266.7768	70.6863
268.996	75.85456	268.8326	66.0794	266.8178	70.67896
269.0371	75.8461	268.8745	66.07075	266.8604	70.67166
269.081	75.8364	268.917	66.06159	266.904	70.66413
269.1215	75.8298	268.9548	66.05409	266.9443	70.65641
269.1625	75.82223	268.9985	66.04807	266.9857	70.64827
269.203	75.81678	269.04	66.03987	267.029	70.63988
269.2459	75.81561	269.0823	66.03167	267.0722	70.63175
269.2886	75.81404	269.1244	66.02563	267.1139	70.624
269.3283	75.81306	269.1677	66.01821	267.1597	70.61659
269.372	75.81018	269.2108	66.01035	267.1988	70.60907
269.4134	75.80604	269.2452	66.00379	267.2415	70.60169
269.445	75.80502	269.2912	65.99409	267.272	70.59524
269.4895	75.80024	269.3285	65.98394	267.3158	70.58669
269.5308	75.79533	269.371	65.97658	267.36	70.57789
269.5716	75.79029	269.4137	65.96805	267.4028	70.56943
269.6155	75.78168	269.4573	65.96029	267.4458	70.56081
269.6554	75.77352	269.4982	65.95296	267.4864	70.5525
269.6944	75.76719	269.5409	65.9419	267.5286	70.54419
269.7401	75.76218	269.583	65.93255	267.5715	70.53559
269.78	75.75779	269.6245	65.92534	267.6158	70.52683
269.8225	75.75425	269.664	65.91653	267.6564	70.51806
269.8614	75.75203	269.7005	65.90924	267.7006	70.50939
269.894	75.7495	269.7426	65.90229	267.7444	70.50067
269.9385	75.74598	269.786	65.89262	267.7869	70.49203
269.9804	75.74193	269.8275	65.88339	267.8284	70.48275
270.0229	75.73479	269.869	65.8766	267.8714	70.47353
270.0644	75.72887	269.9118	65.86908	267.9035	70.467
270.1068	75.72242	269.9531	65.86204	267.9478	70.4583
270.149	75.71922	269.9974	65.85404	267.9896	70.45007
270.1897	75.71366	270.0406	65.84502	268.0318	70.44264
270.2309	75.70575	270.0792	65.83579	268.0762	70.43363
270.2746	75.6988	270.124	65.82537	268.117	70.42407
270.3165	75.69294	270.1584	65.81709	268.1592	70.41446
270.3594	75.691	270.1982	65.80968	268.2032	70.40536
270.4	75.68762	270.2402	65.80104	268.245	70.39565
270.4338	75.68318	270.2817	65.79217	268.2867	70.3869
270.4752	75.67516	270.3259	65.78446	268.3298	70.37777
270.5156	75.66737	270.3667	65.77592	268.3722	70.36776
270.5578	75.659	270.4109	65.76652	268.4135	70.35865
270.6009	75.65427	270.4497	65.7576	268.4555	70.34948
270.646	75.6523	270.4911	65.74715	268.4972	70.34039
270.6853	75.64867	270.5346	65.73619	268.5335	70.33295
270.7266	75.64475	270.577	65.72844	268.5756	70.32397
270.7714	75.64097	270.6099	65.72105	268.6161	70.31445
270.8099	75.63416	270.6498	65.71082	268.6602	70.30455
270.8525	75.6264	270.6935	65.70366	268.7035	70.29521
270.8852	75.62278	270.7375	65.69586	268.7454	70.28445

270.928	75.62034	270.7798	65.68688	268.7878	70.27432
270.9698	75.6178	270.821	65.67826	268.8279	70.26438
271.0099	75.61249	270.8639	65.6677	268.8724	70.2538
271.0519	75.60828	270.9069	65.65552	268.9156	70.24398
271.0935	75.60529	270.9485	65.64665	268.9559	70.23409
271.1378	75.59571	270.9891	65.63836	269.0001	70.22457
271.1806	75.58719	271.0315	65.63084	269.0432	70.21467
271.2199	75.58222	271.068	65.6245	269.0752	70.20692
271.262	75.57602	271.1094	65.61564	269.1198	70.19712
271.3054	75.57241	271.1446	65.6069	269.1637	70.18684
271.3381	75.57009	271.1896	65.59717	269.2048	70.17634
271.378	75.56583	271.227	65.58674	269.2508	70.16585
271.4211	75.55999	271.2691	65.5763	269.2926	70.15527
271.4636	75.55212	271.3148	65.56575	269.3365	70.14445
271.5033	75.54673	271.355	65.55372	269.38	70.13391
271.5461	75.53869	271.396	65.54369	269.4203	70.12274
271.5888	75.52983	271.4408	65.53517	269.4632	70.11165
271.6278	75.52187	271.484	65.52641	269.5061	70.10076
271.6686	75.5146	271.5197	65.51927	269.5498	70.09015
271.7108	75.51161	271.5622	65.51136	269.5915	70.07923
271.7525	75.50835	271.6062	65.50013	269.635	70.06841
271.784	75.50368	271.6441	65.48897	269.6782	70.05783
271.8265	75.49981	271.6896	65.48019	269.7105	70.04897
271.871	75.49253	271.7309	65.46948	269.7552	70.03859
271.9128	75.48598	271.7736	65.46119	269.7976	70.02797
271.9549	75.48026	271.8164	65.45261	269.8416	70.01715
271.9971	75.47514	271.8596	65.44212	269.882	70.0063
272.0362	75.46873	271.9009	65.43165	269.9262	69.9953
272.0804	75.46043	271.9432	65.42142	269.9715	69.98375
272.1226	75.45786	271.9779	65.41373	270.0135	69.97172
272.163	75.45212	272.0216	65.40359	270.0581	69.95972
272.2081	75.44827	272.061	65.39335	270.1022	69.94769
272.2473	75.4426	272.1039	65.38099	270.1457	69.93611
272.2908	75.43401	272.1472	65.36806	270.1899	69.92459
272.3225	75.42745	272.1902	65.35663	270.2328	69.91222
272.3652	75.41924	272.2322	65.34706	270.2735	69.90047
272.405	75.41418	272.2754	65.3382	270.3193	69.88857
272.448	75.40978	272.3164	65.32893	270.3542	69.87946
272.4898	75.40634	272.3598	65.31898	270.3979	69.86827
272.5345	75.40315	272.4029	65.30889	270.4388	69.85667
272.575	75.39893	272.435	65.29987	270.4779	69.8448
272.6166	75.39092	272.4795	65.28779	270.5178	69.83275
272.6588	75.38468	272.5197	65.27732	270.5638	69.82109
272.6987	75.38012	272.5618	65.26552	270.6038	69.80907
272.7397	75.3738	272.6033	65.25441	270.6489	69.79677
272.7746	75.36966	272.6472	65.24476	270.6946	69.78386
272.8167	75.36263	272.6905	65.23565	270.7367	69.77037
272.8591	75.35673	272.7338	65.22596	270.7782	69.75715
272.9009	75.35045	272.7758	65.21601	270.8239	69.74434
272.9417	75.34352	272.82	65.20771	270.8663	69.73174
272.9832	75.33881	272.8589	65.19629	270.9018	69.72212
273.0254	75.33263	272.8947	65.18655	270.946	69.7102
273.0675	75.32404	272.9387	65.17638	270.9902	69.69718
273.1102	75.31727	272.9838	65.16528	271.0313	69.68453
273.1498	75.31383	273.0246	65.15654	271.072	69.67136
273.1928	75.30802	273.065	65.14782	271.1204	69.65786
273.2253	75.303	273.1062	65.1366	271.164	69.64463
273.2699	75.29636	273.152	65.12449	271.2079	69.63064

273.3093	75.28885	273.1953	65.11178	271.2534	69.61776
273.3528	75.27898	273.2374	65.09882	271.2962	69.60502
273.3953	75.26933	273.2795	65.0883	271.3404	69.59191
273.4381	75.26579	273.3228	65.07831	271.3855	69.57891
273.477	75.26152	273.3539	65.06852	271.4304	69.56524
273.5201	75.25736	273.3984	65.05671	271.471	69.55204
273.5632	75.25159	273.4398	65.04649	271.5168	69.5389
273.6056	75.24374	273.4844	65.03503	271.5522	69.52823
273.6504	75.23702	273.5253	65.02375	271.5963	69.51491
273.68	75.23054	273.569	65.01239	271.6367	69.50116
273.721	75.22366	273.6106	64.99883	271.6828	69.48704
273.7656	75.21784	273.6516	64.98681	271.7262	69.47302
273.8063	75.20753	273.697	64.97648	271.7708	69.45942
273.8478	75.19751	273.7383	64.96591	271.8108	69.44585
273.8902	75.18968	273.78	64.95688	271.8567	69.43203
273.9284	75.18278	273.813	64.94807	271.9006	69.41839
273.9712	75.17464	273.8578	64.93556	271.9453	69.40387
274.016	75.16816	273.8986	64.92345	271.9884	69.38949
274.0561	75.15949	273.9408	64.91229	272.029	69.37564
274.102	75.15219	273.9835	64.90057	272.0759	69.36162
274.1429	75.14837	274.0241	64.8882	272.1175	69.34748
274.1853	75.14116	274.0672	64.87618	272.1625	69.33327
274.2168	75.1383	274.1084	64.86239	272.198	69.32228
274.2569	75.1351	274.1513	64.84991	272.2395	69.30889
274.2993	75.12845	274.1947	64.83897	272.2809	69.29544
274.3401	75.1209	274.2392	64.82688	272.3261	69.28143
274.383	75.11602	274.2762	64.8175	272.3698	69.2674
274.4265	75.11039	274.3184	64.80818	272.4134	69.25262
274.4682	75.10154	274.3619	64.79455	272.4562	69.23728
274.5085	75.09596	274.4003	64.78176	272.499	69.22193
274.5524	75.09251	274.4432	64.77049	272.5412	69.20755
274.5941	75.08567	274.4866	64.75465	272.584	69.19392
274.6365	75.0788	274.5271	64.74346	272.6301	69.18074
274.6703	75.07547	274.5702	64.73373	272.6724	69.16776
274.7107	75.06765	274.613	64.71988	272.7157	69.15327
274.753	75.058	274.6556	64.70903	272.7506	69.14092
274.7956	75.05127	274.6985	64.6978	272.795	69.12624
274.8359	75.04631	274.7325	64.68764	272.8383	69.1115
274.8784	75.03876	274.7722	64.67676	272.8791	69.0972
274.9194	75.03335	274.8137	64.66606	272.9219	69.08333
274.9615	75.02576	274.8578	64.65483	272.9639	69.06888
275.0026	75.01531	274.8995	64.64266	273.0105	69.05468
275.0473	75.00706	274.9443	64.62963	273.0506	69.03987
275.0886	74.99718	274.988	64.61617	273.0946	69.025
275.122	74.99264	275.0312	64.60477	273.1359	69.0104
275.1623	74.98816	275.073	64.59291	273.178	68.99576
275.2045	74.98029	275.1131	64.58114	273.2222	68.98099
275.248	74.97566	275.1584	64.56921	273.2683	68.96612
275.289	74.9707	275.1908	64.55882	273.3106	68.95171
275.3332	74.96244	275.2329	64.54514	273.354	68.93713
275.3734	74.95978	275.2758	64.53351	273.3882	68.9258
275.4158	74.95331	275.319	64.52274	273.431	68.91178
275.4574	74.94553	275.3628	64.50982	273.475	68.8973
275.4995	74.93903	275.4028	64.4973	273.5154	68.88154
275.5425	74.9299	275.4438	64.48618	273.5586	68.86628
275.5758	74.92324	275.4831	64.4736	273.601	68.8505
275.6177	74.91263	275.5254	64.46168	273.6435	68.83475
275.6554	74.90275	275.5676	64.45094	273.6873	68.81997

275.6999	74.89346	275.6136	64.43918	273.7346	68.80455
275.7414	74.8868	275.6447	64.43023	273.7797	68.78923
275.7846	74.88008	275.6904	64.41958	273.823	68.77328
275.8256	74.87358	275.7315	64.40717	273.8658	68.75635
275.8675	74.86548	275.7743	64.39592	273.9082	68.73985
275.9052	74.85613	275.813	64.38684	273.9522	68.7243
275.9461	74.84831	275.8575	64.37425	273.9962	68.70889
275.9884	74.84252	275.899	64.36283	274.0317	68.69681
276.0328	74.84008	275.94	64.35121	274.0737	68.68143
276.0758	74.8332	275.9807	64.33691	274.1192	68.66555
276.1088	74.82554	276.0225	64.32542	274.1641	68.64898
276.15	74.81829	276.0646	64.31366	274.2104	68.63215
276.192	74.81013	276.0966	64.30355	274.2536	68.61501
276.2339	74.80147	276.1373	64.29164	274.2988	68.59666
276.2768	74.79411	276.1808	64.28205	274.3449	68.57901
276.3174	74.78796	276.2211	64.27213	274.3895	68.56133
276.3577	74.77881	276.2646	64.2621	274.4346	68.54479
276.4009	74.77058	276.3045	64.25163	274.4796	68.52783
276.4404	74.76505	276.345	64.23908	274.5246	68.51035
276.4826	74.75666	276.3886	64.22834	274.571	68.49238
276.526	74.74939	276.428	64.21955	274.609	68.47643
276.5598	74.74031	276.4677	64.20762	274.6559	68.45739
276.6016	74.73296	276.513	64.19735	274.7042	68.43822
276.6431	74.7259	276.5448	64.18751	274.7478	68.41859
276.6856	74.71564	276.5847	64.17195	274.7946	68.40014
276.7285	74.70803	276.6252	64.15954	274.8428	68.38113
276.7715	74.70131	276.6656	64.14715	274.8896	68.36228
276.8136	74.69279	276.7078	64.13593	274.936	68.34368
276.856	74.68277	276.7467	64.12667	274.9861	68.32485
276.8961	74.67909	276.7892	64.11829	275.0311	68.30572
276.9406	74.67398	276.8284	64.10909	275.0774	68.28661
276.9817	74.66894	276.8718	64.10183	275.1244	68.26771
277.0162	74.6628	276.9127	64.09242	275.1723	68.2488
277.0596	74.65217	276.9534	64.08179	275.2189	68.23013
277.1012	74.64102	276.986	64.0742	275.2666	68.21155
277.143	74.62868	277.0266	64.0629	275.302	68.19688
277.1879	74.62108	277.0646	64.05136	275.3476	68.17807
277.2272	74.61559	277.1012	64.0414	275.3953	68.16005
277.2685	74.60926	277.1414	64.03357	275.4393	68.14139
277.3119	74.60125	277.1825	64.02229	275.4877	68.12259
277.3572	74.59241	277.2212	64.01194	275.5322	68.10482
277.3978	74.58307	277.2615	64.00267	275.574	68.08749
277.4382	74.57468	277.3052	63.99161	275.62	68.07107
277.4747	74.56911	277.3475	63.98281	275.6619	68.05491
277.515	74.56104	277.3883	63.97533	275.7073	68.03873
277.5576	74.55328	277.42	63.96797	275.749	68.02233
277.598	74.54324	277.459	63.95778	275.7911	68.00623
277.6416	74.53229	277.4973	63.94942	275.8349	67.99069
277.68	74.52713	277.5391	63.93879	275.8728	67.97425
277.723	74.51784	277.581	63.92938	275.9182	67.95791
277.7666	74.50974	277.6187	63.92098	275.9497	67.9451
277.8076	74.5028	277.6606	63.91075	275.9915	67.92894
277.8508	74.49181	277.7009	63.90117	276.0332	67.91399
277.8951	74.48359	277.7406	63.89119	276.072	67.90016
277.9331	74.47462	277.7793	63.88084	276.1098	67.88629
277.9756	74.46788	277.82	63.87323	276.1521	67.87266
278.0116	74.46313	277.8528	63.86815	276.1896	67.85949
278.0546	74.45586	277.8939	63.85984	276.2278	67.84551

278.0944	74.44864	277.9346	63.85103	276.2666	67.83202
278.1363	74.43902	277.975	63.84156	276.3059	67.81937
278.1784	74.42681	278.0136	63.83124	276.3462	67.80626
278.2216	74.41594	278.054	63.82355	276.3832	67.79318
278.2618	74.4083	278.0942	63.81598	276.4222	67.78025
278.3061	74.40028	278.1362	63.80611	276.4536	67.77036
278.346	74.3928	278.1748	63.79878	276.4898	67.75778
278.3889	74.38771	278.215	63.78899	276.5288	67.74583
278.43	74.38106	278.2552	63.77884	276.5638	67.73331
278.465	74.37243	278.2878	63.77414	276.6032	67.72098
278.5073	74.36226	278.3282	63.7666	276.6378	67.70944
278.5504	74.35119	278.3689	63.75836	276.6755	67.69848
278.5905	74.33935	278.4097	63.75257	276.7138	67.68683
278.6332	74.32854	278.4485	63.74762	276.75	67.67505
278.6758	74.31908	278.4883	63.73914	276.7872	67.66379
278.7175	74.31428	278.5258	63.7305	276.8246	67.65238
278.7595	74.30941	278.5715	63.72166	276.8596	67.64286
278.8022	74.303	278.6109	63.70964	276.899	67.63328
278.8437	74.29727	278.6487	63.70208	276.9319	67.62324
278.8855	74.28513	278.6878	63.69486	276.9722	67.6132
278.9179	74.27307	278.723	63.68779	276.9985	67.60513
278.96	74.26141	278.7632	63.6815	277.0393	67.59501
279.002	74.25211	278.8056	63.67509	277.073	67.58464
279.0442	74.24585	278.845	63.66662	277.1094	67.57391
279.0865	74.24035	278.8848	63.65926	277.1468	67.56344
279.1284	74.23259	278.9244	63.65052	277.184	67.55358
279.1688	74.22678	278.965	63.64111	277.22	67.54393
279.2114	74.21909	279.0035	63.63462	277.2548	67.53487
279.257	74.20863	279.0428	63.62979	277.2937	67.52597
279.2966	74.2001	279.084	63.62241	277.3284	67.51653
279.3382	74.19177	279.1262	63.61429	277.3625	67.50733
279.3711	74.1845	279.1584	63.60797	277.3998	67.49874
279.4165	74.17343	279.1963	63.59999	277.4372	67.49068
279.4587	74.16406	279.2356	63.59341	277.4754	67.48245
279.5018	74.15508	279.2784	63.58707	277.513	67.47421
279.5416	74.14314	279.315	63.58097	277.5439	67.46707
279.583	74.13211	279.3582	63.57282	277.5809	67.45813
279.6272	74.12576	279.3992	63.56454	277.6169	67.44933
279.6696	74.11583	279.439	63.55786	277.6552	67.44097
279.711	74.10548	279.4797	63.55277	277.6925	67.43271
279.7509	74.09902	279.5186	63.5474	277.7291	67.42339
279.794	74.09	279.5607	63.53961	277.767	67.41508
279.8339	74.07987	279.5939	63.5332	277.803	67.40643
279.8772	74.07297	279.6337	63.52252	277.8386	67.39808
279.91	74.0661	279.6747	63.51184	277.877	67.39022
279.9517	74.05455	279.7129	63.50564	277.9162	67.38194
279.9954	74.04329	279.7541	63.50177	277.9514	67.37386
280.0356	74.03157	279.7946	63.49639	277.9878	67.36633
280.0776	74.02217	279.8344	63.48989	278.0186	67.36043
280.1198	74.01469	279.8755	63.4824	278.0576	67.35324
280.163	74.00814	279.918	63.47413	278.0952	67.34655
280.203	74.00079	279.958	63.46733	278.132	67.33924
280.245	73.99379	279.9959	63.46191	278.17	67.33254
280.2849	73.98499	280.0288	63.45745	278.2075	67.32465
280.3228	73.97514	280.0711	63.45063	278.2434	67.3166
280.3576	73.9692	280.1104	63.44511	278.2759	67.30936
280.4009	73.95862	280.1512	63.43875	278.3152	67.30184
280.4435	73.94622	280.1918	63.43162	278.3541	67.2953

280.4858	73.93751	280.2318	63.42511	278.3927	67.28846
280.5263	73.9258	280.2683	63.41676	278.4313	67.28143
280.568	73.91338	280.3104	63.41105	278.4675	67.27464
280.6106	73.90709	280.3528	63.40689	278.5068	67.26745
280.6515	73.89737	280.3918	63.40122	278.5442	67.26062
280.6948	73.88883	280.4314	63.39575	278.5754	67.25457
280.7378	73.88017	280.464	63.39035	278.613	67.24785
280.7772	73.87026	280.5092	63.38299	278.6509	67.24109
280.8132	73.86324	280.5492	63.37585	278.692	67.23546
280.8565	73.85222	280.59	63.37049	278.7305	67.22982
280.8965	73.84094	280.6299	63.36283	278.7694	67.22349
280.9387	73.83338	280.6701	63.35818	278.8088	67.21729
280.9775	73.82528	280.7106	63.35401	278.8481	67.21073
281.0244	73.81591	280.7486	63.347	278.886	67.20427
281.0666	73.80663	280.7907	63.34187	278.9248	67.19799
281.107	73.79493	280.8311	63.33669	278.9632	67.1916
281.1465	73.78265	280.873	63.32892	279.0024	67.18521
281.1898	73.77081	280.9042	63.32432	279.0388	67.1791
281.2335	73.76149	280.9436	63.31975	279.0792	67.1721
281.2674	73.75583	280.9888	63.31179	279.1196	67.16597
281.3066	73.7469	281.027	63.30696	279.1496	67.16066
281.351	73.73688	281.0657	63.30456	279.1874	67.1542
281.3976	73.73265	281.1079	63.29781	279.2253	67.14809
281.4352	73.72427	281.148	63.29202	279.2652	67.14206
281.478	73.71265	281.191	63.28599	279.3062	67.13616
281.5225	73.70347	281.2303	63.27798	279.3455	67.13029
281.5614	73.69203	281.2713	63.27411	279.3856	67.12464
281.6074	73.67972	281.3118	63.26819	279.4256	67.11862
281.6474	73.6692	281.3429	63.26123	279.4673	67.11291
281.689	73.66228	281.3864	63.25469	279.5061	67.10775
281.7316	73.65573	281.4256	63.2485	279.5448	67.10241
281.773	73.64969	281.4649	63.24066	279.5815	67.09682
281.8055	73.64406	281.5077	63.23763	279.6196	67.09131
281.8466	73.6352	281.5463	63.23519	279.6534	67.08625
281.8884	73.6248	281.5879	63.22845	279.6916	67.08019
281.9308	73.61042	281.6288	63.22459	279.7286	67.07469
281.9732	73.59873	281.672	63.21945	279.7707	67.0683
282.0176	73.59027	281.7124	63.21151	279.81	67.06164
282.0568	73.5783	281.7527	63.20612	279.8506	67.05551
282.1004	73.57125	281.7842	63.20198	279.8902	67.0493
282.1444	73.56348	281.8273	63.19387	279.9303	67.04358
282.1834	73.55079	281.8686	63.18755	279.9707	67.03813
282.2242	73.54162	281.9072	63.18218	280.0078	67.03294
282.2598	73.53313	281.9488	63.17628	280.0462	67.02785
282.3004	73.52139	281.9894	63.17336	280.0881	67.0236
282.3447	73.51148	282.0323	63.16784	280.1287	67.01861
282.3864	73.50116	282.0718	63.1625	280.1672	67.01351
282.428	73.49276	282.1115	63.15849	280.21	67.00857
282.4702	73.4865	282.1519	63.15063	280.2422	67.0037
282.5122	73.47779	282.1931	63.14466	280.2807	66.99937
282.5554	73.46976	282.2249	63.1416	280.3204	66.99468
282.5938	73.46075	282.268	63.13681	280.3611	66.99006
282.638	73.44934	282.3086	63.13164	280.3986	66.98553
282.6794	73.43968	282.3502	63.12707	280.4412	66.98017
282.7128	73.43301	282.3918	63.12069	280.4839	66.97567
282.754	73.42355	282.4315	63.11454	280.5237	66.9703
282.7988	73.41201	282.4722	63.11061	280.562	66.96477
282.8389	73.40128	282.5159	63.10481	280.6018	66.95906

282.8796	73.39167	282.5548	63.09853	280.6418	66.95345
282.9214	73.38301	282.5951	63.09382	280.681	66.94862
282.9642	73.37304	282.6388	63.0894	280.7236	66.94399
283.0065	73.36575	282.6724	63.08692	280.763	66.93976
283.0483	73.35704	282.7123	63.08586	280.8056	66.93545
283.0877	73.34634	282.7534	63.08373	280.8384	66.93065
283.1284	73.33535	282.7944	63.0784	280.876	66.9252
283.1629	73.32767	282.8336	63.07309	280.9185	66.91964
283.207	73.31714	282.8762	63.06718	280.9581	66.91384
283.2481	73.30387	282.9178	63.06075	280.9987	66.90896
283.2898	73.29632	282.9586	63.057	281.0398	66.90465
283.3335	73.28886	282.9992	63.05268	281.0793	66.90084
283.3743	73.28098	283.039	63.04714	281.1196	66.89644
283.4188	73.27276	283.0782	63.0414	281.1618	66.89229
283.4557	73.26188	283.1101	63.03522	281.2006	66.88735
283.4988	73.2523	283.1544	63.03006	281.2422	66.88245
283.542	73.24206	283.1972	63.02685	281.2813	66.87791
283.5847	73.23173	283.2354	63.02297	281.3252	66.8729
283.626	73.22644	283.2768	63.01948	281.3565	66.8689
283.6696	73.21944	283.3163	63.01715	281.3952	66.86388
283.7016	73.21108	283.3603	63.01198	281.4369	66.85876
283.7478	73.20327	283.3956	63.00693	281.4766	66.85426
283.7871	73.19387	283.435	63.00254	281.518	66.85002
283.8298	73.18134	283.4754	62.99678	281.5588	66.84617
283.8738	73.17056	283.5172	62.99148	281.5972	66.8416
283.9143	73.16169	283.5519	62.9877	281.639	66.83701
283.955	73.15203	283.5912	62.98223	281.6792	66.83221
283.9972	73.14328	283.6332	62.97585	281.7186	66.82643
284.037	73.1345	283.6767	62.96994	281.7582	66.82107
284.08	73.12437	283.716	62.96392	281.8	66.81622
284.1252	73.11408	283.7575	62.95795	281.8414	66.8118
284.156	73.10735	283.8005	62.95214	281.8816	66.80809
284.2	73.09758	283.8385	62.948	281.9212	66.80397
284.2434	73.08989	283.8804	62.94403	281.9529	66.80104
284.2824	73.07976	283.9228	62.9412	281.9938	66.79677
284.3229	73.07054	283.9629	62.93644	282.0339	66.79153
284.3676	73.06364	283.9969	62.93089	282.0754	66.78751
284.4094	73.05572	284.0376	62.92565	282.1172	66.78298
284.4522	73.04564	284.078	62.91963	282.1592	66.77905
284.4935	73.03508	284.1182	62.916	282.201	66.77578
284.5303	73.0242	284.1589	62.91249	282.2404	66.77219
284.5723	73.01151	284.2006	62.90764	282.2806	66.76834
284.6053	73.00505	284.2428	62.90494	282.3218	66.76463
284.6456	72.99601	284.2832	62.90087	282.3604	66.76201
284.6878	72.9861	284.3242	62.89642	282.4024	66.75836
284.7305	72.97675	284.3665	62.89189	282.443	66.756
284.7706	72.9699	284.4067	62.88514	282.483	66.75277
284.8132	72.96588	284.439	62.87945	282.5239	66.74792
284.8564	72.95999	284.478	62.87395	282.5568	66.74387
284.8992	72.95063	284.5191	62.86924	282.5982	66.73725
284.9411	72.9407	284.5614	62.86535	282.641	66.73078
284.9856	72.93064	284.6026	62.86175	282.6824	66.72549
285.0258	72.92098	284.6446	62.85731	282.719	66.72128
285.059	72.91519	284.684	62.85327	282.76	66.71807
285.1018	72.90915	284.7274	62.84903	282.8001	66.71378
285.1424	72.89935	284.7648	62.8448	282.8444	66.70963
285.1836	72.88808	284.8064	62.83934	282.8836	66.70504
285.2258	72.87674	284.8507	62.83331	282.9258	66.70063

285.2666	72.86844	284.8834	62.82864	282.9664	66.69663
285.3096	72.86087	284.9227	62.82227	283.0064	66.69293
285.3486	72.84848	284.9646	62.81797	283.0493	66.68911
285.3924	72.83862	285.0046	62.81365	283.0818	66.68576
285.4342	72.82867	285.046	62.80818	283.1213	66.68146
285.4754	72.81782	285.0895	62.80373	283.1637	66.67715
285.516	72.81155	285.1318	62.79974	283.205	66.67302
285.5542	72.8052	285.1713	62.79626	283.2455	66.66935
285.5884	72.79674	285.215	62.79305	283.2874	66.66633
285.6313	72.78755	285.2549	62.78993	283.3258	66.66293
285.6726	72.78318	285.2947	62.78557	283.3668	66.65964
285.7149	72.77732	285.3284	62.78212	283.4077	66.6557
285.757	72.7722	285.3709	62.77744	283.4507	66.65101
285.7983	72.76432	285.4127	62.77308	283.492	66.64684
285.8404	72.75494	285.4548	62.76948	283.5315	66.64267
285.8796	72.74729	285.493	62.76571	283.573	66.63912
285.9201	72.73893	285.5345	62.7628	283.6148	66.63575
285.9628	72.73038	285.5762	62.75987	283.6558	66.63229
286.003	72.72315	285.617	62.75725	283.6881	66.62924
286.0362	72.71545	285.6608	62.75484	283.7278	66.62543
286.0774	72.7059	285.7006	62.75193	283.7695	66.62152
286.1176	72.69691	285.7426	62.74924	283.8106	66.61686
286.1618	72.68734	285.7746	62.7462	283.8489	66.61295
286.2002	72.6761	285.8158	62.74354	283.8918	66.60897
286.241	72.66923	285.8596	62.7399	283.9316	66.60563
286.2837	72.66415	285.8985	62.73605	283.971	66.60293
286.3236	72.65471	285.9412	62.73044	284.0136	66.60003
286.3662	72.64633	285.9803	62.72508	284.0547	66.59673
286.408	72.63571	286.023	62.72068	284.0968	66.59318
286.4475	72.62671	286.0645	62.7179	284.1371	66.58903
286.4772	72.62006	286.1093	62.71686	284.1777	66.58494
286.5182	72.61415	286.1469	62.71395	284.2162	66.58066
286.562	72.61059	286.1884	62.71092	284.2565	66.57688
286.6042	72.60326	286.2215	62.70638	284.2911	66.57424
286.6416	72.59428	286.2607	62.70046	284.332	66.57052
286.6838	72.58599	286.3039	62.69486	284.373	66.56713
286.7258	72.57774	286.3432	62.69147	284.4148	66.56356
286.7674	72.56893	286.3886	62.68791	284.4577	66.5599
286.8094	72.5602	286.4283	62.68512	284.4965	66.55693
286.8506	72.55407	286.4691	62.68265	284.5388	66.55307
286.893	72.54524	286.51	62.67769	284.5788	66.5496
286.9248	72.53647	286.548	62.67447	284.6206	66.54616
286.9656	72.52773	286.5926	62.66984	284.662	66.54286
287.0064	72.52121	286.634	62.66649	284.7046	66.53939
287.0461	72.51316	286.6666	62.66354	284.7418	66.53536
287.0889	72.50494	286.7085	62.66001	284.7845	66.53125
287.1306	72.49828	286.7486	62.65655	284.818	66.52736
287.1691	72.49079	286.7908	62.65372	284.8598	66.52374
287.2094	72.48136	286.8336	62.65001	284.8985	66.52082
287.2512	72.47351	286.8739	62.64559	284.9391	66.51712
287.2918	72.46883	286.9144	62.64167	284.982	66.51359
287.3323	72.46	286.9573	62.63703	285.0214	66.51063
287.3754	72.45279	286.9956	62.63382	285.0632	66.50683
287.416	72.4458	287.038	62.63047	285.104	66.50427
287.4505	72.43652	287.0774	62.62771	285.1474	66.50159
287.4904	72.42591	287.112	62.62455	285.19	66.49819
287.5318	72.41523	287.155	62.62062	285.2266	66.49501
287.5723	72.40802	287.1965	62.61629	285.2703	66.49124

287.6146	72.40217	287.2356	62.61305	285.3115	66.4881
287.6535	72.39794	287.2754	62.60952	285.355	66.4844
287.6949	72.39343	287.318	62.60555	285.3942	66.48184
287.7373	72.38628	287.3575	62.60173	285.4285	66.47916
287.7756	72.37838	287.4022	62.59759	285.4689	66.47612
287.8189	72.36858	287.4447	62.59371	285.512	66.47277
287.8602	72.35873	287.4819	62.59049	285.5519	66.46896
287.891	72.35093	287.5242	62.58782	285.5913	66.46564
287.9327	72.34014	287.5588	62.58517	285.6322	66.46209
287.9738	72.33125	287.5981	62.58351	285.6734	66.45906
288.0149	72.3247	287.6408	62.58073	285.7156	66.45535
288.0554	72.31434	287.6811	62.57701	285.7551	66.45176
288.0942	72.30673	287.7244	62.57206	285.7972	66.44758
288.1366	72.30206	287.764	62.566	285.8396	66.44406
288.179	72.29498	287.8059	62.56227	285.8819	66.44082
288.2166	72.28787	287.848	62.55974	285.9226	66.43785
288.2565	72.28271	287.8875	62.55769	285.9646	66.43465
288.2996	72.27476	287.9296	62.55554	286.0066	66.43134
288.3359	72.26871	287.9719	62.55254	286.042	66.42907
288.3749	72.26038	288.0047	62.55026	286.0814	66.42627
288.4146	72.25268	288.0469	62.54816	286.1196	66.42385
288.4556	72.24476	288.0852	62.54588	286.1564	66.421
288.4988	72.23618	288.128	62.54305	286.198	66.41829
288.5368	72.22855	288.1695	62.53956	286.2375	66.41485
288.5772	72.22277	288.2114	62.53576	286.2782	66.41169
288.6184	72.21337	288.2529	62.53167	286.3207	66.40863
288.6586	72.20238	288.2938	62.52806	286.363	66.40604
288.701	72.19701	288.3346	62.52488	286.4028	66.40311
288.7429	72.19033	288.3744	62.52262	286.4468	66.39945
288.7746	72.18421	288.4166	62.51997	286.4868	66.39504
288.8096	72.1776	288.4516	62.51707	286.5326	66.39026
288.8514	72.17165	288.4908	62.51272	286.5654	66.38647
288.8923	72.16367	288.5341	62.50788	286.6057	66.38252
288.9348	72.15501	288.5776	62.50319	286.6437	66.37919
288.9772	72.14878	288.6181	62.50013	286.6858	66.37637
289.0174	72.141	288.6563	62.49781	286.7278	66.37405
289.0593	72.13301	288.6984	62.49462	286.7696	66.37178
289.1026	72.12545	288.7385	62.49165	286.8094	66.36955
289.1434	72.11747	288.779	62.48852	286.8499	66.36636
289.1815	72.11152	288.8216	62.48593	286.8908	66.36358
289.2275	72.10581	288.866	62.48231	286.9341	66.3603
289.2651	72.10187	288.8989	62.48021	286.9753	66.35712
289.2969	72.09883	288.94	62.47768	287.0175	66.35401
289.3386	72.08998	288.9828	62.47389	287.0586	66.35037
289.3819	72.08078	289.023	62.47168	287.1004	66.34723
289.4216	72.07258	289.064	62.46976	287.1387	66.34402
289.4643	72.06431	289.1067	62.4662	287.1719	66.34154
289.5066	72.05533	289.1446	62.46227	287.2158	66.33888
289.5444	72.0498	289.1888	62.45969	287.2556	66.33728
289.588	72.04295	289.2302	62.45597	287.2959	66.33529
289.6272	72.03381	289.2719	62.45327	287.337	66.3336
289.6679	72.02841	289.3126	62.45133	287.3805	66.33074
289.71	72.02166	289.3465	62.44792	287.4202	66.32705
289.744	72.01605	289.3862	62.44493	287.4626	66.32337
289.7852	72.00803	289.4273	62.44305	287.5029	66.31983
289.8242	71.99919	289.469	62.43954	287.5461	66.31672
289.8665	71.99116	289.5109	62.43765	287.5844	66.3133
289.9054	71.98105	289.5512	62.43495	287.6297	66.31081

289.949	71.97173	289.5939	62.43081	287.667	66.30781
289.9916	71.96499	289.6339	62.42743	287.7106	66.30492
290.0328	71.9569	289.6772	62.42285	287.7522	66.30266
290.0725	71.94763	289.7156	62.41785	287.7873	66.30034
290.1128	71.94159	289.7566	62.41428	287.828	66.29766
290.1512	71.93667	289.7912	62.41299	287.8683	66.29607
290.187	71.93009	289.8342	62.41071	287.908	66.29351
290.2304	71.92196	289.877	62.40921	287.9478	66.29088
290.2687	71.91439	289.9174	62.40743	287.9932	66.28786
290.3115	71.90556	289.9574	62.40448	288.0344	66.28372
290.3524	71.89869	289.9998	62.40183	288.0736	66.27957
290.394	71.89241	290.0416	62.39864	288.114	66.2757
290.434	71.88688	290.0818	62.39609	288.157	66.27263
290.4734	71.88057	290.1224	62.39544	288.1994	66.2699
290.5158	71.87236	290.1638	62.39298	288.2411	66.26753
290.557	71.86589	290.2055	62.39079	288.281	66.26528
290.5954	71.85901	290.2414	62.38908	288.3168	66.26348
290.6302	71.85312	290.2832	62.3847	288.3566	66.26157
290.6686	71.84489	290.3271	62.38016	288.3972	66.25957
290.7113	71.83863	290.363	62.37728	288.4368	66.25676
290.7515	71.83075	290.4016	62.3737	288.478	66.25345
290.7938	71.8221	290.4447	62.37023	288.5202	66.24967
290.8367	71.81608	290.486	62.36917	288.5614	66.2467
290.8761	71.80966	290.5261	62.36615	288.6015	66.24393
290.9183	71.80274	290.5666	62.36179	288.6428	66.24062
290.9606	71.79791	290.6076	62.35688	288.6856	66.23769
290.9989	71.7914	290.6496	62.35136	288.7264	66.23402
291.04	71.78142	290.6842	62.34667	288.767	66.23102
291.0817	71.77097	290.7262	62.3436	288.8086	66.22789
291.1223	71.76359	290.7695	62.33979	288.8507	66.22405
291.1564	71.75824	290.8087	62.33669	288.8928	66.22181
291.197	71.75172	290.8475	62.3358	288.926	66.22001
291.2394	71.74625	290.8891	62.33417	288.9662	66.21851
291.2781	71.74154	290.9324	62.33341	289.0089	66.21769
291.3204	71.73431	290.9724	62.33122	289.0504	66.21596
291.363	71.72538	291.0139	62.33054	289.094	66.21335
291.4038	71.7202	291.0548	62.32997	289.1338	66.20978
291.4442	71.71301	291.0964	62.32836	289.1735	66.20685
291.484	71.70532	291.1292	62.32556	289.218	66.20409
291.5294	71.69958	291.1737	62.32186	289.259	66.20161
291.568	71.69471	291.2123	62.31788	289.3002	66.19951
291.6002	71.6895	291.2538	62.31397	289.3396	66.19705
291.6412	71.68158	291.295	62.31443	289.3809	66.19397
291.6833	71.67439	291.3378	62.31485	289.4207	66.19156
291.7276	71.66696	291.3796	62.31117	289.4651	66.18887
291.769	71.65864	291.4194	62.30749	289.5048	66.1864
291.8089	71.64884	291.4606	62.30334	289.5375	66.1839
291.85	71.63928	291.5048	62.29718	289.5781	66.1812
291.8887	71.63186	291.5456	62.29423	289.6201	66.17842
291.9297	71.62532	291.5778	62.29351	289.6602	66.17596
291.9708	71.61893	291.6202	62.29105	289.704	66.17334
292.0127	71.61689	291.66	62.28869	289.7425	66.17053
292.0449	71.61248	291.7026	62.28551	289.786	66.16793
292.0888	71.6027	291.7426	62.28157	289.8291	66.16487
292.1268	71.59492	291.787	62.28007	289.8686	66.16241
292.168	71.58697	291.8272	62.27726	289.9104	66.15956
292.2106	71.57631	291.8668	62.27668	289.9534	66.15743
292.2508	71.57181	291.9092	62.27557	289.9939	66.1548

292.2928	71.56982	291.9516	62.27135	290.0354	66.15279
292.3338	71.56353	291.9912	62.26646	290.0695	66.15099
292.3764	71.55881	292.024	62.26395	290.1109	66.14865
292.418	71.55283	292.0678	62.2637	290.1524	66.14633
292.4573	71.54479	292.1089	62.26206	290.1954	66.1439
292.4894	71.53718	292.1502	62.26211	290.2358	66.14124
292.5305	71.52831	292.191	62.25901	290.2772	66.13874
292.57	71.51846	292.2328	62.25351	290.3181	66.13648
292.6134	71.50899	292.274	62.25209	290.3586	66.13371
292.654	71.50077	292.3155	62.24882	290.4032	66.13068
292.6944	71.49442	292.356	62.24772	290.4412	66.12683
292.7366	71.48864	292.3978	62.24751	290.4838	66.12292
292.7774	71.47927	292.4368	62.24461	290.5237	66.11942
292.8184	71.47198	292.4711	62.24277	290.5676	66.11611
292.8611	71.46856	292.5135	62.24018	290.6069	66.1136
292.8998	71.46025	292.557	62.23371	290.6482	66.11134
292.9372	71.45056	292.5962	62.22807	290.6831	66.10973
292.9763	71.44677	292.639	62.2258	290.7216	66.10777
293.0117	71.44259	292.6796	62.2234	290.7615	66.10601
293.0549	71.43668	292.7201	62.22057	290.8034	66.10396
293.0944	71.43349	292.7642	62.21919	290.8475	66.10156
293.1354	71.42757	292.8038	62.2159	290.8876	66.09948
293.1777	71.41691	292.845	62.21403	290.9278	66.09741
293.2212	71.4089	292.8874	62.21173	290.969	66.09483
293.2632	71.40263	292.919	62.20758	291.0095	66.09221
293.2993	71.39778	292.9629	62.20571	291.0489	66.08879
293.343	71.39358	293.0028	62.20232	291.0932	66.08563
293.3852	71.38774	293.0444	62.20002	291.1355	66.08303
293.4272	71.38402	293.0863	62.19985	291.1762	66.08098
293.4581	71.37873	293.129	62.1989	291.2188	66.07973
293.4998	71.37022	293.1707	62.19709	291.2598	66.07807
293.5413	71.36315	293.2104	62.19607	291.2896	66.07692
293.5832	71.35754	293.2532	62.19403	291.3336	66.0748
293.6249	71.35133	293.2938	62.1899	291.3752	66.07248
293.6653	71.34489	293.3347	62.18695	291.4167	66.0699
293.7042	71.33768	293.3666	62.18404	291.4609	66.067
293.7469	71.32729	293.4085	62.1798	291.502	66.06394
293.7866	71.31668	293.4488	62.17985	291.5425	66.06049
293.8258	71.31005	293.489	62.17852	291.5848	66.05755
293.8685	71.30389	293.532	62.1749	291.6253	66.05479
293.904	71.29761	293.5763	62.17508	291.6666	66.05315
293.9469	71.29318	293.6149	62.17133	291.7113	66.05191
293.9844	71.28904	293.6585	62.16719	291.7501	66.04952
294.0286	71.28435	293.6996	62.16622	291.7919	66.04704
294.0692	71.27731	293.7412	62.16172	291.8256	66.04494
294.1086	71.26969	293.7829	62.15763	291.8675	66.0423
294.1498	71.26205	293.8169	62.1577	291.9072	66.04064
294.1894	71.25426	293.8578	62.15611	291.9509	66.03867
294.2309	71.24802	293.8972	62.1531	291.9928	66.03656
294.274	71.24322	293.9416	62.15131	292.0314	66.03411
294.3132	71.23529	293.9832	62.1456	292.0716	66.03157
294.3482	71.22749	294.025	62.14228	292.1148	66.02964
294.3875	71.2245	294.0644	62.14393	292.1578	66.02743
294.4308	71.22219	294.1057	62.14395	292.1974	66.02481
294.4702	71.21716	294.149	62.14211	292.239	66.02161
294.5114	71.21073	294.19	62.13978	292.2814	66.01982
294.5519	71.20138	294.2324	62.13668	292.3226	66.0179
294.5958	71.19138	294.2646	62.13347	292.3632	66.01611

294.6361	71.18313	294.3062	62.13112	292.4062	66.01478
294.6787	71.17568	294.3478	62.1281	292.4356	66.01299
294.718	71.17223	294.3875	62.12486	292.4802	66.01055
294.7591	71.16808	294.4298	62.11977	292.5213	66.00885
294.8037	71.16087	294.4726	62.11785	292.5646	66.00695
294.8402	71.15608	294.5111	62.11876	292.6052	66.00468
294.874	71.15217	294.5541	62.11348	292.6448	66.00239
294.9177	71.14524	294.5942	62.11006	292.688	65.99962
294.9594	71.13798	294.6389	62.11044	292.7297	65.99777
295.0002	71.13293	294.679	62.1066	292.7722	65.99638
295.0419	71.12532	294.7138	62.10388	292.8121	65.99418
295.0837	71.11642	294.7578	62.10203	292.8516	65.99241
295.1242	71.10813	294.7944	62.09695	292.8968	65.98971
295.1633	71.09994	294.8356	62.09247	292.9404	65.98631
295.2068	71.09151	294.8795	62.09371	292.9795	65.98365
295.2454	71.08394	294.9182	62.09456	293.0198	65.98178
295.2893	71.07782	294.962	62.09329	293.0531	65.98026
295.3215	71.0745	295.002	62.09307	293.0965	65.97878
295.3642	71.0707	295.0446	62.08996	293.138	65.97773
295.4033	71.06451	295.0841	62.08978	293.1778	65.97443
295.4462	71.06083	295.1262	62.0883	293.2175	65.97131
295.4859	71.05602	295.1578	62.08566	293.2594	65.96786
295.532	71.04883	295.2023	62.08312	293.3013	65.96453
295.5683	71.0448	295.2432	62.0793	293.3454	65.96207
295.609	71.03942	295.2842	62.07579	293.3863	65.95911
295.651	71.0313	295.3268	62.07378	293.4246	65.95693
295.6923	71.028	295.3673	62.07315	293.4675	65.95458
295.733	71.02402	295.409	62.07051	293.5092	65.95206
295.7666	71.01856	295.4515	62.07054	293.5509	65.95057
295.8067	71.01038	295.4926	62.07014	293.585	65.95022
295.8474	71.00201	295.5332	62.0669	293.625	65.94892
295.8927	70.9932	295.5749	62.0644	293.6657	65.94731
295.931	70.98497	295.609	62.06123	293.7011	65.94583
295.9744	70.98079	295.6494	62.05772	293.7398	65.9429
296.013	70.97584	295.6913	62.05652	293.7805	65.94078
296.0556	70.96952	295.7338	62.05742	293.8191	65.93976
296.0977	70.96272	295.7736	62.054	293.8626	65.93757
296.1363	70.95449	295.8161	62.05153	293.9074	65.93491
296.178	70.94911	295.8575	62.05122	293.9465	65.93129
296.212	70.94558	295.8995	62.0476	293.9867	65.9281
296.2537	70.9426	295.938	62.04577	294.0302	65.92537
296.2925	70.94163	295.9823	62.04575	294.0708	65.92321
296.334	70.93691	296.022	62.04212	294.1135	65.92214
296.3732	70.92714	296.0564	62.0405	294.1562	65.92049
296.4161	70.91936	296.0965	62.04069	294.1888	65.9194
296.4564	70.91597	296.1358	62.03812	294.2294	65.91802
296.4984	70.91083	296.1792	62.03783	294.2728	65.9163
296.5404	70.90745	296.2204	62.03655	294.3128	65.91401
296.5806	70.90327	296.2627	62.0323	294.353	65.91248
296.6228	70.89548	296.3046	62.0304	294.394	65.91079
296.6648	70.88529	296.3484	62.0259	294.4366	65.91007
296.7036	70.87738	296.3867	62.02057	294.4769	65.90873
296.7368	70.87273	296.4288	62.01974	294.518	65.90714
296.7783	70.86465	296.4712	62.01628	294.5626	65.90499
296.8213	70.85897	296.5032	62.01186	294.6036	65.90211
296.8575	70.85377	296.5439	62.01031	294.644	65.90045
296.8988	70.84737	296.5865	62.00887	294.6886	65.89818
296.9398	70.84325	296.6283	62.00932	294.7295	65.8962

296.9823	70.83822	296.6689	62.01147	294.7716	65.89375
297.0233	70.83352	296.7094	62.01191	294.8021	65.89122
297.0628	70.82461	296.7506	62.00941	294.8454	65.88818
297.1072	70.8189	296.7946	62.00558	294.8885	65.88538
297.1502	70.81491	296.836	62.0027	294.9275	65.88342
297.1838	70.81105	296.8762	61.99786	294.9719	65.88149
297.2216	70.80659	296.9191	61.99602	295.0125	65.88035
297.2625	70.80307	296.9518	61.99646	295.054	65.87881
297.3028	70.79691	296.9944	61.99189	295.0944	65.87695
297.3454	70.78963	297.0346	61.9908	295.1367	65.87572
297.3852	70.78554	297.0774	61.9922	295.1786	65.87454
297.4259	70.78101	297.119	61.98802	295.2201	65.87311
297.4698	70.77677	297.1614	61.98712	295.2637	65.87147
297.5094	70.76975	297.2002	61.98482	295.3044	65.86941
297.5478	70.76427	297.2425	61.97952	295.337	65.86663
297.59	70.75832	297.2825	61.9785	295.3791	65.86348
297.6236	70.75106	297.3231	61.9774	295.4213	65.86052
297.664	70.74165	297.3709	61.9762	295.4612	65.85775
297.7054	70.73698	297.399	61.97534	295.5035	65.85525
297.7475	70.73152	297.4424	61.97514	295.5441	65.85305
297.7879	70.7261	297.4838	61.9741	295.5884	65.8506
297.8304	70.72539	297.5263	61.97309	295.6278	65.84837
297.8704	70.72198	297.5678	61.97326	295.669	65.8461
297.9092	70.71603	297.6084	61.96975	295.7123	65.84463
297.9532	70.70898	297.6425	61.96599	295.7502	65.84329
297.9966	70.70501	297.6849	61.96318	295.7928	65.84153
298.0368	70.70058	297.725	61.95812	295.8332	65.83997
298.0703	70.69529	297.7665	61.95768	295.8752	65.83744
298.1137	70.68958	297.8098	61.95628	295.9177	65.83511
298.1519	70.68543	297.8437	61.95442	295.95	65.8331
298.1906	70.67893	297.8832	61.95548	295.99	65.83064
298.235	70.67202	297.9224	61.95538	296.0288	65.82876
298.2786	70.66604	297.9678	61.95369	296.0721	65.82639
298.3184	70.66021	298.007	61.95322	296.1136	65.82422
298.3578	70.65482	298.0494	61.95109	296.1535	65.82245
298.3996	70.64902	298.0884	61.948	296.1966	65.82063
298.4435	70.64362	298.13	61.94706	296.2367	65.81925
298.4826	70.63437	298.1716	61.94759	296.2793	65.81742
298.5235	70.62935	298.2144	61.94275	296.3224	65.81561
298.5668	70.62472	298.256	61.93976	296.3628	65.81365
298.5997	70.62172	298.2885	61.93863	296.403	65.81269
298.6416	70.61852	298.33	61.9335	296.4426	65.81236
298.6816	70.61283	298.3717	61.93226	296.4843	65.8114
298.7232	70.61001	298.4132	61.93209	296.5247	65.81053
298.763	70.60613	298.4576	61.92925	296.5606	65.80892
298.8012	70.60037	298.498	61.92993	296.6024	65.80686
298.8436	70.59498	298.5374	61.92946	296.6425	65.80505
298.8864	70.58889	298.578	61.92789	296.6856	65.80297
298.9268	70.58131	298.6188	61.92896	296.7275	65.80126
298.968	70.57799	298.6606	61.92743	296.768	65.79901
299.0072	70.57649	298.7049	61.92433	296.8096	65.79676
299.0405	70.57348	298.7361	61.92186	296.8546	65.79437
299.081	70.56805	298.7782	61.91702	296.8941	65.79206
299.1237	70.56274	298.8184	61.91104	296.9328	65.79039
299.1633	70.55701	298.861	61.90885	296.9746	65.78954
299.2056	70.55148	298.9001	61.90852	297.0163	65.78878
299.2487	70.54746	298.942	61.90637	297.0564	65.78793
299.2879	70.54618	298.9878	61.90746	297.0906	65.78658

299.3288	70.54228	299.0259	61.90632	297.1348	65.78468
299.3717	70.53488	299.0698	61.90583	297.1747	65.78227
299.413	70.52838	299.11	61.9045	297.2154	65.77979
299.4562	70.52453	299.1526	61.90232	297.2535	65.7778
299.4891	70.52097	299.1865	61.90099	297.2992	65.77581
299.5275	70.51501	299.2251	61.89699	297.3366	65.77366
299.5686	70.51494	299.2684	61.89567	297.382	65.77163
299.6114	70.51232	299.3097	61.89206	297.4228	65.76977
299.6514	70.50792	299.3516	61.89008	297.4639	65.76742
299.6926	70.50258	299.3927	61.88918	297.5039	65.76599
299.7323	70.49616	299.4341	61.88588	297.5455	65.7644
299.7739	70.4874	299.4774	61.88562	297.5862	65.76385
299.8162	70.47952	299.5186	61.88531	297.6278	65.76287
299.8586	70.47553	299.5573	61.88337	297.6718	65.76173
299.8982	70.47035	299.5991	61.88279	297.7037	65.76087
299.9293	70.46634	299.6317	61.88163	297.7449	65.75838
299.9718	70.4607	299.6734	61.8788	297.7843	65.75552
300.0124	70.45397	299.7166	61.8768	297.8291	65.75258
300.054	70.4487	299.7575	61.87485	297.8716	65.7502
300.0958	70.44509	299.8009	61.87297	297.9108	65.74831
300.1365	70.4418	299.8418	61.87117	297.9522	65.74682
300.178	70.44086	299.8828	61.87088	297.9957	65.74602
300.2204	70.4383	299.9277	61.86817	298.038	65.74531
300.2636	70.43321	299.9694	61.86787	298.0779	65.7443
300.3076	70.42964	300.0089	61.86661	298.12	65.74266
300.3473	70.42318	300.052	61.86357	298.1606	65.74025
300.3867	70.41797	300.0845	61.86523	298.2043	65.73772
300.4292	70.41695	300.1267	61.86749	298.2438	65.73531
300.4628	70.41426	300.1672	61.86785	298.2833	65.73281
300.503	70.41063	300.21	61.86776	298.3202	65.7311
300.5427	70.40669	300.2504	61.86851	298.361	65.72978
300.5857	70.40211	300.291	61.86529	298.4036	65.72859
300.6276	70.39765	300.3352	61.86272	298.4436	65.72821
300.6663	70.39079	300.3738	61.86034	298.4846	65.72838
300.7088	70.38721	300.417	61.85518	298.5284	65.72716
300.7495	70.38101	300.459	61.85214	298.5656	65.72555
300.7916	70.37274	300.5009	61.84897	298.6104	65.72373
300.8322	70.36886	300.533	61.84754	298.6505	65.72231
300.8746	70.36528	300.5752	61.84836	298.6924	65.72091
300.9076	70.36134	300.6156	61.84685	298.734	65.72015
300.9466	70.35879	300.6582	61.84493	298.7752	65.71861
300.9879	70.35516	300.6965	61.84767	298.817	65.71678
301.032	70.35141	300.7383	61.84701	298.8505	65.71517
301.069	70.34702	300.7821	61.84394	298.8911	65.71205
301.1124	70.33868	300.8213	61.84309	298.9322	65.70983
301.1508	70.33484	300.864	61.83977	298.9745	65.7073
301.1908	70.32967	300.9073	61.83406	299.0146	65.70496
301.2327	70.325	300.9475	61.83372	299.0547	65.7033
301.2718	70.32235	300.9824	61.83288	299.0976	65.70197
301.3129	70.31493	301.0214	61.82808	299.143	65.70059
301.3461	70.30673	301.0672	61.82784	299.1819	65.69932
301.3882	70.29643	301.1052	61.82789	299.2226	65.6971
301.4294	70.28972	301.1474	61.82576	299.2636	65.6954
301.473	70.28447	301.1874	61.82777	299.3074	65.69475
301.513	70.28342	301.23	61.82655	299.3485	65.69378
301.5535	70.28686	301.2715	61.82475	299.3916	65.69372
301.5944	70.28812	301.3112	61.82526	299.4326	65.69302
301.638	70.28857	301.3534	61.82464	299.4654	65.69128

301.6804	70.28757	301.3936	61.82173	299.5077	65.68892
301.7176	70.28424	301.4293	61.82036	299.551	65.68645
301.7594	70.27908	301.4676	61.819	299.5918	65.68418
301.7941	70.27262	301.511	61.81462	299.6338	65.68223
301.8352	70.26866	301.5536	61.81266	299.6747	65.68082
301.8752	70.26548	301.593	61.81155	299.7159	65.67999
301.9182	70.26016	301.636	61.80913	299.759	65.67871
301.9594	70.25781	301.6772	61.80973	299.8005	65.67649
301.9986	70.25528	301.718	61.80792	299.8409	65.67416
302.0412	70.25184	301.7617	61.80545	299.8848	65.67196
302.0824	70.24708	301.8027	61.80453	299.9254	65.67034
302.1244	70.24416	301.8436	61.79895	299.9662	65.66941
302.1685	70.24166	301.8758	61.7977	300.0075	65.66952
302.2084	70.23656	301.9185	61.79727	300.0506	65.66852
302.249	70.23179	301.9572	61.79371	300.084	65.66649
302.2887	70.22686	301.9968	61.79209	300.1226	65.66502
302.3222	70.2227	302.042	61.79342	300.1672	65.66293
302.3616	70.21789	302.0836	61.79315	300.2076	65.66123
302.4044	70.21312	302.124	61.7914	300.2508	65.65961
302.4455	70.21158	302.1666	61.79252	300.2906	65.65816
302.4879	70.20776	302.209	61.78925	300.331	65.65662
302.5284	70.20159	302.2478	61.78566	300.3725	65.65528
302.5698	70.19925	302.2891	61.78545	300.4128	65.65444
302.6128	70.19709	302.3236	61.78541	300.4543	65.65308
302.655	70.19284	302.3667	61.78574	300.4965	65.65129
302.6939	70.18995	302.4076	61.78881	300.5371	65.64892
302.7338	70.18419	302.4484	61.78938	300.5781	65.64713
302.7686	70.17902	302.4902	61.78565	300.6121	65.6454
302.8096	70.17495	302.5312	61.78315	300.6548	65.64404
302.8508	70.17098	302.5732	61.77891	300.6938	65.64329
302.8926	70.17094	302.6156	61.77488	300.7379	65.64237
302.9309	70.1699	302.6568	61.77471	300.7795	65.64133
302.9718	70.166	302.6985	61.77296	300.8218	65.64112
303.0154	70.16241	302.7404	61.77033	300.8645	65.63952
303.0533	70.15994	302.7728	61.77063	300.9042	65.63729
303.0954	70.15681	302.8158	61.76887	300.9472	65.63533
303.1382	70.15278	302.8585	61.76689	300.9857	65.63336
303.1782	70.14919	302.897	61.76836	301.0273	65.63178
303.2126	70.14554	302.9402	61.76679	301.067	65.63123
303.2516	70.13997	302.9812	61.76447	301.1092	65.63072
303.2944	70.13616	303.0238	61.76361	301.1498	65.62828
303.3344	70.1328	303.0659	61.75969	301.1928	65.62627
303.3742	70.12838	303.1064	61.75817	301.2248	65.62442
303.4156	70.12213	303.1462	61.75973	301.2684	65.62196
303.4562	70.11734	303.19	61.7573	301.3091	65.62021
303.4988	70.11361	303.2264	61.75474	301.3516	65.61856
303.5409	70.10733	303.2668	61.75374	301.3912	65.61732
303.584	70.10568	303.3072	61.75106	301.429	65.61643
303.6246	70.10446	303.3472	61.74934	301.4684	65.61462
303.6588	70.10092	303.3874	61.75066	301.511	65.61287
303.7005	70.097	303.4296	61.74977	301.5525	65.61099
303.7417	70.09337	303.4702	61.74786	301.595	65.61036
303.784	70.08932	303.5148	61.74772	301.6358	65.61067
303.826	70.08361	303.5537	61.74706	301.6783	65.61099
303.8662	70.07929	303.5969	61.7463	301.7172	65.61119
303.907	70.07242	303.6371	61.74549	301.7614	65.60922
303.9472	70.06606	303.6716	61.74355	301.802	65.60659
303.9886	70.05831	303.712	61.74166	301.8352	65.60465

304.0305	70.05392	303.7544	61.74135	301.8771	65.60159
304.0707	70.04992	303.7954	61.73878	301.9184	65.59922
304.1132	70.04129	303.8348	61.73704	301.9608	65.59798
304.1542	70.03488	303.8778	61.73525	302	65.59647
304.1869	70.03105	303.9199	61.73133	302.043	65.5954
304.2283	70.03234	303.9607	61.73019	302.0859	65.59392
304.2695	70.03429	304.0012	61.72972	302.1268	65.59213
304.31	70.03341	304.0434	61.72762	302.1697	65.5909
304.3515	70.02583	304.085	61.72862	302.209	65.58889
304.3964	70.00688	304.1184	61.73169	302.2498	65.5873
304.4343	69.99424	304.1602	61.72856	302.293	65.58637
304.4764	69.98784	304.2017	61.72635	302.3346	65.58559
304.5173	69.98938	304.245	61.72477	302.3675	65.58549
304.5581	70.00042	304.2862	61.71938	302.4074	65.58483
304.5982	70.00305	304.3289	61.72086	302.4478	65.58339
304.6324	70.00202	304.3686	61.72385	302.4916	65.58086
304.6703	69.99738	304.4109	61.7214	302.5327	65.57823
304.7164	69.98764	304.4492	61.71935	302.576	65.57638
304.7576	69.97971	304.4909	61.71634	302.615	65.57437
304.8013	69.97536	304.5326	61.71231	302.6585	65.57338
304.8416	69.97101	304.5675	61.71075	302.7003	65.57265
304.8815	69.96688	304.6084	61.71122	302.7424	65.57173
304.9252	69.96661	304.6485	61.71112	302.782	65.57189
304.9651	69.96449	304.6924	61.71032	302.8236	65.57099
305.0056	69.96108	304.7356	61.71067	302.8657	65.5697
305.0469	69.95748	304.776	61.70986	302.9043	65.56839
305.0787	69.95376	304.8154	61.71068	302.9479	65.56661
305.1201	69.95306	304.8582	61.7112	302.9805	65.56584
305.1616	69.95462	304.898	61.70859	303.022	65.5654
305.206	69.95553	304.9407	61.70966	303.063	65.5635
305.2461	69.95394	304.9841	61.70974	303.1035	65.56219
305.2881	69.94959	305.017	61.70718	303.1461	65.56064
305.3278	69.94467	305.0582	61.7056	303.186	65.55844
305.3705	69.94046	305.0972	61.70451	303.2256	65.55795
305.4122	69.93933	305.1414	61.69906	303.2687	65.55658
305.4519	69.93708	305.1817	61.69577	303.3125	65.55599
305.4944	69.93466	305.2225	61.69486	303.3541	65.55589
305.5283	69.93286	305.264	61.69093	303.3954	65.55542
305.5669	69.92776	305.3081	61.69117	303.439	65.55449
305.6074	69.92432	305.3462	61.69096	303.4792	65.55307
305.65	69.92228	305.3875	61.68872	303.5224	65.55205
305.6929	69.92389	305.4326	61.69094	303.5622	65.55025
305.736	69.92384	305.465	61.69037	303.598	65.5492
305.7761	69.92199	305.5065	61.68788	303.6387	65.54795
305.8178	69.92145	305.549	61.68929	303.6788	65.54676
305.8576	69.9189	305.5888	61.68871	303.7204	65.5453
305.8982	69.91628	305.6323	61.68497	303.7604	65.54382
305.9403	69.91551	305.6742	61.68478	303.8052	65.54148
305.9822	69.91642	305.7126	61.68488	303.8435	65.53912
306.0248	69.91451	305.7535	61.68085	303.8852	65.5368
306.0562	69.91115	305.79	61.68012	303.9278	65.53553
306.0974	69.91014	305.8316	61.68008	303.9677	65.53466
306.1384	69.9053	305.8737	61.67795	304.0118	65.53282
306.1806	69.89594	305.909	61.67772	304.0512	65.53126
306.2204	69.89178	305.9517	61.67732	304.094	65.5297
306.261	69.89065	305.9923	61.67548	304.1274	65.52854
306.3047	69.88698	306.0324	61.67665	304.1682	65.52777
306.3448	69.88719	306.0752	61.67606	304.2045	65.52723

306.3874	69.88828	306.1148	61.67425	304.2456	65.52614
306.4268	69.88487	306.1594	61.67619	304.2878	65.52429
306.4711	69.88201	306.1992	61.67648	304.3278	65.52227
306.5033	69.88041	306.2408	61.6735	304.3723	65.52067
306.545	69.8801	306.2809	61.6724	304.4148	65.519
306.5865	69.88083	306.3236	61.66838	304.4575	65.51881
306.6278	69.87735	306.3582	61.66182	304.498	65.51762
306.6686	69.87303	306.3984	61.66028	304.541	65.51691
306.7096	69.86862	306.4392	61.66173	304.5804	65.5158
306.7508	69.86073	306.4802	61.66137	304.6224	65.51352
306.7902	69.85903	306.5222	61.66115	304.6654	65.51273
306.8339	69.85844	306.5625	61.6612	304.7065	65.51196
306.8738	69.85861	306.6054	61.65859	304.7428	65.51067
306.9136	69.85814	306.6475	61.65728	304.7854	65.50931
306.95	69.85533	306.691	61.65655	304.8306	65.50727
306.9913	69.85277	306.7291	61.65497	304.8674	65.50407
307.0331	69.84971	306.7728	61.65743	304.908	65.50261
307.072	69.84825	306.8062	61.65676	304.9508	65.5021
307.1144	69.84792	306.8472	61.65399	304.9929	65.50188
307.1552	69.84902	306.8891	61.65225	305.0353	65.5021
307.1962	69.84712	306.9307	61.64881	305.0752	65.50225
307.242	69.84644	306.971	61.64615	305.118	65.50145
307.2796	69.84348	307.013	61.64709	305.1606	65.50024
307.3212	69.84002	307.0546	61.64757	305.2022	65.49958
307.3636	69.83659	307.0954	61.64605	305.2456	65.49752
307.3962	69.83406	307.1396	61.64523	305.2852	65.49592
307.4365	69.83284	307.18	61.64382	305.3265	65.49501
307.48	69.83168	307.2224	61.64109	305.362	65.49335
307.5206	69.82887	307.2572	61.6393	305.4006	65.49145
307.5628	69.82494	307.295	61.63738	305.4422	65.48952
307.6026	69.82152	307.3382	61.63478	305.4837	65.48754
307.6449	69.81727	307.3779	61.63543	305.5274	65.48606
307.6872	69.81535	307.4204	61.63666	305.5672	65.48488
307.7284	69.81143	307.4611	61.63387	305.607	65.48388
307.7675	69.80466	307.5042	61.63263	305.6492	65.48279
307.809	69.8007	307.5453	61.63152	305.6906	65.48213
307.8506	69.80043	307.5888	61.62915	305.7305	65.4813
307.8915	69.80162	307.6299	61.63045	305.7724	65.48064
307.926	69.80242	307.6734	61.6309	305.8158	65.48041
307.9672	69.80152	307.7045	61.63012	305.8584	65.47984
308.0082	69.79892	307.7463	61.62953	305.8906	65.47919
308.0508	69.79694	307.79	61.62929	305.9322	65.47773
308.0907	69.79414	307.8296	61.62797	305.9729	65.4757
308.1342	69.79149	307.8711	61.62653	306.0142	65.47286
308.1735	69.78952	307.9105	61.62463	306.0518	65.47075
308.2138	69.78555	307.9509	61.6227	306.0936	65.47005
308.255	69.7819	307.9969	61.62133	306.1352	65.46969
308.2956	69.77933	308.0402	61.61997	306.1787	65.46998
308.3392	69.7781	308.0802	61.6187	306.2206	65.46923
308.3719	69.77931	308.1191	61.62072	306.2622	65.4678
308.4136	69.77973	308.1531	61.62141	306.3022	65.46616
308.4553	69.78226	308.1955	61.62034	306.3454	65.46436
308.4949	69.78386	308.2371	61.61952	306.3902	65.46239
308.5393	69.77875	308.2807	61.61526	306.4292	65.46119
308.5786	69.77392	308.323	61.61131	306.4735	65.46012
308.6213	69.77197	308.3617	61.60971	306.5031	65.45972
308.6644	69.76676	308.4054	61.60683	306.5452	65.46
308.7036	69.75938	308.4452	61.60619	306.586	65.45895

308.7466	69.75692	308.4868	61.60904	306.6284	65.45715
308.7877	69.75384	308.5277	61.60916	306.6715	65.45525
308.8176	69.75168	308.5679	61.60821	306.7125	65.45235
308.8605	69.75218	308.6031	61.60882	306.755	65.45138
308.9041	69.75149	308.6446	61.6053	306.7927	65.45051
308.9452	69.74799	308.6851	61.60168	306.8357	65.44931
308.9858	69.74749	308.7282	61.60381	306.8775	65.44927
309.029	69.74272	308.7711	61.60391	306.9201	65.44869
309.0696	69.74092	308.8104	61.60185	306.9602	65.44736
309.1096	69.74239	308.8537	61.60173	307.004	65.44662
309.1518	69.74088	308.8926	61.59914	307.0438	65.44577
309.1944	69.73986	308.9358	61.5981	307.0848	65.44462
309.2352	69.73701	308.975	61.59747	307.1167	65.44363
309.2696	69.73439	309.0159	61.5953	307.1589	65.44187
309.3094	69.72859	309.051	61.59636	307.1988	65.44057
309.3518	69.72582	309.0924	61.59429	307.2439	65.43879
309.3943	69.72747	309.1331	61.59264	307.2844	65.4369
309.4357	69.72411	309.1772	61.59301	307.325	65.43589
309.4767	69.72105	309.2178	61.59214	307.3661	65.43441
309.5207	69.72168	309.26	61.59036	307.4088	65.43335
309.5595	69.72152	309.3012	61.58792	307.4502	65.43286
309.6006	69.72196	309.3407	61.58663	307.4919	65.43057
309.6424	69.72118	309.3833	61.58287	307.533	65.42885
309.68	69.72012	309.4229	61.5818	307.5752	65.427
309.7234	69.71838	309.464	61.58255	307.618	65.42635
309.7663	69.71551	309.4992	61.5819	307.649	65.42635
309.7996	69.70942	309.5385	61.5831	307.6923	65.42535
309.8436	69.70222	309.582	61.5834	307.731	65.42398
309.8841	69.69623	309.624	61.58095	307.773	65.42269
309.9244	69.6949	309.6657	61.57909	307.8168	65.42206
309.9669	69.69618	309.7072	61.57774	307.8596	65.42146
310.0059	69.70108	309.749	61.57645	307.8991	65.42065
310.051	69.70185	309.7906	61.57771	307.9418	65.41925
310.0906	69.6958	309.8325	61.57961	307.9824	65.41795
310.133	69.69023	309.8754	61.57656	308.0239	65.41746
310.1735	69.68315	309.9194	61.57667	308.0671	65.41719
310.2183	69.68128	309.9514	61.57708	308.1086	65.41673
310.2482	69.68307	309.9925	61.57578	308.1502	65.41494
310.2894	69.68606	310.0309	61.57587	308.192	65.41291
310.3295	69.6856	310.0756	61.57459	308.2329	65.41124
310.3737	69.67937	310.1184	61.57092	308.2678	65.41032
310.4162	69.67179	310.1566	61.56813	308.3067	65.40984
310.4556	69.67139	310.1992	61.56716	308.349	65.40925
310.4982	69.67056	310.2396	61.56493	308.389	65.4078
310.5402	69.66921	310.2802	61.56544	308.4322	65.40626
310.5804	69.66489	310.3238	61.56348	308.4736	65.40436
310.621	69.65744	310.3645	61.56094	308.514	65.40303
310.6654	69.6508	310.397	61.56263	308.5547	65.40252
310.6967	69.64929	310.4378	61.56194	308.5962	65.40122
310.7388	69.65016	310.4781	61.56106	308.6396	65.39951
310.7818	69.64748	310.5198	61.56179	308.6778	65.39804
310.822	69.64536	310.5615	61.56157	308.724	65.39645
310.8644	69.64578	310.6037	61.55866	308.764	65.39549
310.9057	69.64851	310.6454	61.55653	308.8046	65.39528
310.9476	69.64952	310.6878	61.55525	308.839	65.39449
310.9889	69.64927	310.7288	61.55448	308.8727	65.39338
311.0309	69.64561	310.773	61.55467	308.9144	65.39166
311.0709	69.63487	310.8124	61.55368	308.9546	65.39059

311.1127	69.62979	310.845	61.55205	308.9962	65.38972
311.1449	69.63309	310.8878	61.55019	309.0405	65.38987
311.1873	69.63465	310.9288	61.54781	309.08	65.38851
311.2278	69.63648	310.97	61.5476	309.12	65.38738
311.2683	69.63706	311.0091	61.54906	309.1626	65.38637
311.3108	69.63279	311.052	61.54674	309.2031	65.38506
311.3513	69.63024	311.0922	61.54363	309.2456	65.38456
311.3922	69.6315	311.1316	61.54632	309.2875	65.38354
311.434	69.62991	311.1759	61.54651	309.3284	65.38173
311.4717	69.63234	311.2167	61.54627	309.3696	65.38031
311.5178	69.63381	311.2567	61.54657	309.4028	65.37995
311.5582	69.63137	311.2906	61.54357	309.4469	65.37862
311.5991	69.63074	311.3388	61.54138	309.4864	65.3779
311.64	69.62787	311.3796	61.5414	309.5298	65.37611
311.6746	69.62233	311.419	61.5409	309.5709	65.37399
311.7164	69.61491	311.4602	61.53908	309.6102	65.37276
311.7595	69.61252	311.5032	61.53874	309.6509	65.37162
311.7976	69.61285	311.5456	61.53758	309.6919	65.37104
311.8388	69.612	311.5858	61.53559	309.7348	65.37112
311.8807	69.6115	311.6261	61.5363	309.7761	65.3708
311.921	69.61309	311.6686	61.5348	309.8174	65.37094
311.9629	69.61147	311.7084	61.53218	309.8612	65.37089
312.0056	69.60983	311.7408	61.53501	309.9037	65.36942
312.0449	69.61021	311.7846	61.53465	309.945	65.36767
312.0854	69.61152	311.8244	61.53104	309.9858	65.36567
312.1179	69.60921	311.8682	61.52918	310.0217	65.3642
312.1618	69.60486	311.9113	61.52523	310.0619	65.36317
312.2028	69.60398	311.9497	61.52338	310.1035	65.36312
312.2451	69.60059	311.9907	61.52572	310.1437	65.36187
312.2862	69.5987	312.033	61.5246	310.1884	65.36066
312.3293	69.59692	312.0763	61.52354	310.231	65.35945
312.3691	69.59138	312.117	61.52361	310.27	65.35867
312.414	69.58557	312.1592	61.5204	310.3104	65.35803
312.454	69.57904	312.1912	61.52065	310.3537	65.35724
312.4934	69.57635	312.2339	61.52193	310.3981	65.35604
312.5404	69.57935	312.277	61.52069	310.4383	65.35406
312.57	69.57968	312.319	61.51884	310.4822	65.35271
312.6129	69.57894	312.3602	61.52	310.5213	65.35185
312.6568	69.57495	312.4014	61.51763	310.5659	65.35122
312.6936	69.57479	312.4422	61.51427	310.6054	65.35034
312.7367	69.57568	312.4832	61.51284	310.6416	65.34906
312.7768	69.57308	312.5278	61.51047	310.6819	65.34771
312.8172	69.57342	312.5693	61.50938	310.7228	65.34634
312.8588	69.5714	312.6098	61.51181	310.765	65.3452
312.9018	69.56491	312.6427	61.51073	310.804	65.34448
312.9428	69.56173	312.6845	61.50934	310.8466	65.34351
312.9856	69.56036	312.726	61.5094	310.8906	65.34304
313.0184	69.557	312.769	61.50485	310.9304	65.34226
313.0592	69.55654	312.8117	61.50443	310.9704	65.34195
313.1007	69.55575	312.8518	61.50536	311.0136	65.34045
313.1422	69.55359	312.8935	61.50357	311.0574	65.33783
313.1804	69.55146	312.9333	61.50485	311.098	65.33631
313.2226	69.54941	312.9754	61.50715	311.141	65.33508
313.2637	69.54903	313.0188	61.5041	311.1736	65.33412
313.3074	69.54895	313.0576	61.50362	311.2128	65.33445
313.3478	69.54805	313.0926	61.50329	311.2545	65.33401
313.3921	69.54705	313.1333	61.49911	311.2966	65.33297
313.4316	69.5442	313.1738	61.49691	311.3376	65.33242

313.4745	69.53979	313.2166	61.49507	311.378	65.33188
313.5151	69.53866	313.2566	61.49326	311.4196	65.3313
313.5506	69.53817	313.3013	61.4953	311.4606	65.33108
313.5913	69.53695	313.3411	61.49686	311.5026	65.33066
313.6342	69.53587	313.3824	61.49558	311.546	65.32932
313.675	69.53127	313.4246	61.49456	311.5856	65.32766
313.7156	69.52781	313.4669	61.49079	311.6285	65.3257
313.7564	69.5271	313.5064	61.48782	311.669	65.32379
313.7996	69.5274	313.541	61.48779	311.7112	65.3229
313.8412	69.52825	313.5805	61.48849	311.7538	65.32188
313.8834	69.52375	313.6235	61.48753	311.787	65.32038
313.9232	69.52154	313.6656	61.48547	311.8258	65.31962
313.9648	69.51881	313.707	61.4859	311.8669	65.31852
313.9971	69.51525	313.7478	61.48529	311.9071	65.31774
314.0384	69.51565	313.7909	61.48503	311.9497	65.31766
314.0814	69.51567	313.8344	61.48624	311.9932	65.31625
314.1218	69.51479	313.873	61.48408	312.034	65.31602
314.1635	69.51885	313.9154	61.4819	312.0754	65.31548
314.2045	69.51774	313.9561	61.48072	312.1188	65.31586
314.2452	69.51348	313.99	61.47876	312.1586	65.31504
314.2876	69.51062	314.0315	61.47835	312.199	65.31306
314.3318	69.50792	314.0728	61.47988	312.2394	65.31097
314.3696	69.50379	314.1164	61.47929	312.279	65.30817
314.4129	69.50055	314.1587	61.47844	312.3248	65.30696
314.4454	69.50147	314.1984	61.47723	312.3654	65.306
314.4871	69.50236	314.2386	61.47353	312.398	65.30555
314.5281	69.50453	314.2796	61.4716	312.4402	65.30452
314.573	69.5043	314.3218	61.47235	312.4813	65.30382
314.6124	69.50426	314.3636	61.47284	312.5225	65.30312
314.6526	69.50119	314.3972	61.47317	312.5642	65.30197
314.6905	69.49481	314.4306	61.47442	312.6055	65.30157
314.7342	69.49162	314.473	61.47358	312.6478	65.3009
314.7754	69.49058	314.5136	61.47476	312.687	65.29996
314.8199	69.48759	314.5566	61.47404	312.7275	65.29964
314.8595	69.4845	314.5967	61.47111	312.7695	65.29894
314.8915	69.48419	314.6389	61.46995	312.8113	65.2978
314.933	69.48315	314.6806	61.46963	312.8542	65.29661
314.9711	69.48145	314.7232	61.46786	312.8952	65.29544
315.0142	69.47862	314.7649	61.46823	312.9298	65.29432
315.0588	69.47845	314.8043	61.46726	312.9708	65.29322
315.0996	69.47304	314.8464	61.4625	313.0148	65.2929
315.1388	69.47075	314.8789	61.46218	313.0553	65.29127
315.1817	69.46997	314.9202	61.46214	313.0935	65.2904
315.2219	69.47043	314.9626	61.45915	313.134	65.28911
315.2646	69.47469	315.0037	61.45612	313.1764	65.28787
315.3057	69.47391	315.0471	61.45542	313.22	65.28765
315.3486	69.47321	315.0856	61.45378	313.2604	65.28751
315.3896	69.47348	315.1285	61.45583	313.3013	65.28736
315.4222	69.47154	315.1711	61.45883	313.341	65.28714
315.4602	69.47012	315.2122	61.45695	313.3858	65.28575
315.5041	69.4701	315.255	61.45646	313.4252	65.28379
315.545	69.46758	315.2959	61.45583	313.4685	65.28212
315.5893	69.46346	315.3298	61.45258	313.5075	65.28014
315.6299	69.45586	315.3704	61.45273	313.5425	65.27887
315.6714	69.45094	315.4128	61.45454	313.5829	65.27791
315.7126	69.4516	315.4539	61.45375	313.6232	65.27702
315.753	69.45193	315.4972	61.45208	313.6676	65.2759
315.7983	69.45094	315.5372	61.45167	313.7085	65.27599

315.839	69.45208	315.5778	61.44814	313.75	65.27487
315.87	69.45062	315.6202	61.44489	313.7896	65.27346
315.9121	69.4468	315.6618	61.44504	313.8345	65.27269
315.9545	69.44478	315.7042	61.44291	313.8756	65.27112
315.9962	69.44493	315.7446	61.44314	313.9182	65.27098
316.0408	69.44366	315.778	61.44539	313.9575	65.27118
316.0797	69.4366	315.819	61.44448	313.9998	65.27004
316.1221	69.4302	315.8611	61.44329	314.0413	65.26915
316.1626	69.42831	315.9028	61.44288	314.0847	65.26731
316.204	69.42674	315.9426	61.43976	314.1232	65.26507
316.2422	69.42763	315.9848	61.43976	314.155	65.2645
316.286	69.4303	316.0272	61.44281	314.2	65.2638
316.3176	69.43193	316.0697	61.44138	314.2406	65.26312
316.36	69.42758	316.1102	61.44022	314.2826	65.26315
316.4027	69.42524	316.152	61.43998	314.3258	65.26257
316.4416	69.42475	316.1939	61.4356	314.3642	65.26107
316.4832	69.42277	316.2252	61.43451	314.4072	65.26031
316.5242	69.4234	316.2692	61.43669	314.4487	65.25923
316.5686	69.42462	316.3104	61.43558	314.49	65.25862
316.6063	69.42799	316.3501	61.43397	314.5318	65.25826
316.6506	69.42789	316.3928	61.43286	314.5754	65.25681
316.692	69.42204	316.4352	61.42959	314.6158	65.25544
316.733	69.41457	316.4738	61.42809	314.654	65.25385
316.7684	69.40626	316.5158	61.42818	314.6885	65.25314
316.8094	69.39889	316.5563	61.42803	314.731	65.25274
316.8508	69.39622	316.5963	61.43163	314.7726	65.25244
316.8917	69.39376	316.6403	61.43114	314.8128	65.25177
316.9332	69.39318	316.6738	61.42965	314.8555	65.25124
316.9722	69.3933	316.7176	61.42846	314.8974	65.25012
317.0159	69.39173	316.76	61.42464	314.9381	65.24902
317.0567	69.39389	316.7996	61.42174	314.9783	65.2481
317.0961	69.39452	316.8416	61.42006	315.0206	65.24601
317.1392	69.39155	316.8832	61.41871	315.0628	65.24533
317.1813	69.38672	316.9262	61.41789	315.105	65.24466
317.2249	69.38286	316.9668	61.41673	315.1456	65.24421
317.266	69.38192	317.0104	61.41806	315.1881	65.24384
317.2994	69.38049	317.0494	61.4188	315.2271	65.24282
317.3402	69.37967	317.09	61.41766	315.2688	65.24185
317.3806	69.38153	317.1246	61.41737	315.3032	65.24094
317.4218	69.3808	317.1654	61.41589	315.3438	65.23984
317.463	69.38162	317.2056	61.41403	315.3872	65.23928
317.5056	69.38357	317.2476	61.41214	315.426	65.23866
317.5459	69.37963	317.2918	61.40984	315.4711	65.23817
317.588	69.37752	317.331	61.41054	315.5099	65.23751
317.6328	69.37807	317.3723	61.41226	315.5523	65.23645
317.6708	69.3788	317.4133	61.41296	315.5944	65.235
317.7151	69.38124	317.4568	61.41468	315.6352	65.23354
317.7457	69.3827	317.4998	61.41445	315.6758	65.23201
317.788	69.38062	317.5404	61.41183	315.7168	65.23051
317.8282	69.37999	317.5704	61.40996	315.7589	65.23054
317.8709	69.3773	317.6144	61.40931	315.7984	65.22916
317.9119	69.37183	317.6552	61.406	315.8402	65.22806
317.9532	69.3665	317.6972	61.40306	315.8809	65.22681
317.994	69.36051	317.7388	61.40269	315.9176	65.22497
318.0342	69.35851	317.7804	61.4034	315.9569	65.22394
318.0806	69.35868	317.8238	61.40397	315.993	65.2232
318.121	69.35346	317.864	61.40761	316.0342	65.22243
318.1656	69.35034	317.907	61.40675	316.0738	65.22164

318.1978	69.34804	317.9479	61.40611	316.1178	65.22057
318.2394	69.34631	317.9892	61.40822	316.1561	65.22058
318.2792	69.34639	318.0237	61.40587	316.1991	65.22035
318.319	69.3437	318.0676	61.40413	316.2397	65.21962
318.3614	69.33679	318.108	61.40393	316.2826	65.21827
318.4028	69.33161	318.1485	61.4019	316.3225	65.21604
318.4446	69.32835	318.1909	61.39863	316.3657	65.21419
318.4856	69.32453	318.2333	61.39725	316.4044	65.21232
318.5263	69.32466	318.272	61.39483	316.4418	65.21168
318.5686	69.32178	318.3172	61.39045	316.4817	65.21174
318.6118	69.32176	318.3599	61.39101	316.5226	65.21161
318.6428	69.32479	318.3967	61.39009	316.5656	65.21101
318.6833	69.3252	318.441	61.38937	316.6067	65.21019
318.726	69.33073	318.4754	61.39103	316.647	65.20776
318.7666	69.33874	318.5152	61.38934	316.6883	65.20602
318.8099	69.34203	318.559	61.38726	316.7308	65.20553
318.8492	69.34255	318.6003	61.38784	316.7758	65.20543
318.8916	69.34323	318.6413	61.38672	316.8144	65.2063
318.9334	69.3364	318.6816	61.38546	316.8542	65.20636
318.9756	69.32704	318.7244	61.3867	316.8973	65.20595
319.0179	69.33159	318.7639	61.38687	316.936	65.20501
319.0591	69.33217	318.806	61.38348	316.9811	65.20364
319.0996	69.32955	318.8484	61.38509	317.0209	65.20252
319.1413	69.33398	318.8891	61.38573	317.0548	65.20137
319.1729	69.33272	318.9224	61.38154	317.0957	65.19997
319.2148	69.32777	318.962	61.3837	317.1368	65.19946
319.256	69.32487	319.0035	61.3859	317.1791	65.19989
319.2972	69.32501	319.0465	61.38372	317.2191	65.19997
319.3399	69.32443	319.0882	61.38401	317.2636	65.19976
319.3808	69.32266	319.1314	61.38266	317.3044	65.19789
319.4211	69.32149	319.1719	61.37917	317.3455	65.1956
319.4623	69.32116	319.2147	61.37658	317.3865	65.19308
319.5033	69.3171	319.2541	61.37487	317.425	65.19243
319.5432	69.3136	319.2963	61.37218	317.4698	65.19218
319.5848	69.31043	319.3378	61.37164	317.513	65.1925
319.6205	69.30948	319.3694	61.37323	317.5568	65.19288
319.6618	69.31017	319.4109	61.37278	317.598	65.19189
319.7059	69.30851	319.4537	61.37389	317.6394	65.19099
319.7465	69.30551	319.4957	61.37382	317.6722	65.18959
319.7872	69.30047	319.5372	61.37005	317.7146	65.18822
319.83	69.29413	319.5782	61.36801	317.7567	65.18759
319.869	69.28971	319.6195	61.36536	317.7951	65.18733
319.913	69.289	319.6614	61.36142	317.8374	65.18708
319.9542	69.29216	319.7024	61.36237	317.8798	65.18721
319.997	69.29112	319.7453	61.36305	317.9207	65.18641
320.0386	69.29118	319.788	61.36278	317.9634	65.18594
320.0727	69.29598	319.8181	61.36494	318.0049	65.18571
320.1119	69.30007	319.8606	61.36617	318.0476	65.18461
320.1537	69.30402	319.8978	61.36628	318.0887	65.18313
320.193	69.30225	319.9425	61.3677	318.1312	65.18191
320.2334	69.29961	319.9861	61.36628	318.1704	65.18071
320.2759	69.29606	320.0272	61.3642	318.202	65.17961
320.3186	69.28827	320.0672	61.36629	318.2437	65.17962
320.3598	69.28818	320.1077	61.36532	318.2876	65.179
320.4024	69.28501	320.1482	61.3635	318.3315	65.178
320.444	69.27773	320.1895	61.36396	318.3698	65.17679
320.4873	69.2746	320.2323	61.36004	318.4106	65.17525
320.5197	69.27338	320.2644	61.35748	318.4527	65.1739

320.5602	69.27602	320.3078	61.35864	318.4961	65.17227
320.6016	69.28061	320.3482	61.35559	318.5381	65.17142
320.6414	69.28188	320.3909	61.35346	318.5791	65.1707
320.6837	69.28383	320.4298	61.35356	318.6212	65.16955
320.7253	69.28254	320.4724	61.35425	318.6612	65.16856
320.7693	69.28204	320.5145	61.35688	318.704	65.16788
320.8084	69.28236	320.558	61.35598	318.748	65.16772
320.85	69.28014	320.5995	61.35453	318.789	65.16742
320.8913	69.27598	320.6438	61.35233	318.8188	65.16741
320.9297	69.27162	320.6833	61.34915	318.8603	65.16696
320.9735	69.26401	320.7168	61.34908	318.9019	65.16568
321.0161	69.25766	320.7588	61.34901	318.9438	65.16456
321.0485	69.25358	320.8012	61.35078	318.9854	65.16316
321.0899	69.24745	320.8405	61.35262	319.0286	65.16222
321.1322	69.2454	320.8836	61.35172	319.0728	65.1615
321.1723	69.24579	320.9242	61.35073	319.1134	65.16049
321.2157	69.2464	320.9666	61.3486	319.1544	65.16
321.2572	69.25084	321.0086	61.34608	319.196	65.1589
321.2971	69.25793	321.0495	61.34736	319.2375	65.15801
321.338	69.2636	321.0909	61.34746	319.2788	65.15761
321.3767	69.26754	321.1346	61.34436	319.321	65.15735
321.4225	69.26819	321.1667	61.34291	319.3626	65.15697
321.4661	69.26424	321.209	61.33967	319.4032	65.15661
321.4982	69.25827	321.2484	61.3356	319.4375	65.15578
321.5353	69.25347	321.2887	61.33466	319.4805	65.155
321.5779	69.25298	321.331	61.33441	319.5226	65.15431
321.6187	69.25265	321.373	61.3325	319.5652	65.15241
321.658	69.25108	321.4128	61.33386	319.6067	65.15181
321.7017	69.25195	321.4568	61.33449	319.6482	65.15068
321.7448	69.25057	321.4988	61.33216	319.6904	65.14918
321.7836	69.24778	321.539	61.33214	319.7358	65.1491
321.825	69.25134	321.5822	61.33015	319.7734	65.14756
321.8675	69.2541	321.6174	61.32841	319.8169	65.147
321.9078	69.25246	321.6564	61.3281	319.8602	65.14733
321.9424	69.25076	321.6963	61.32931	319.9004	65.14696
321.982	69.2445	321.7372	61.32863	319.9404	65.14693
322.024	69.24074	321.7781	61.32892	319.9742	65.14665
322.0683	69.23816	321.8225	61.32952	320.0172	65.14524
322.108	69.23418	321.8643	61.32851	320.0569	65.14432
322.1515	69.23787	321.9068	61.32898	320.0982	65.14342
322.1934	69.23916	321.9476	61.32876	320.1384	65.14179
322.2332	69.23515	321.9905	61.32765	320.1809	65.14026
322.2758	69.23624	322.0322	61.32679	320.2223	65.13866
322.3171	69.23836	322.0644	61.3268	320.2635	65.13738
322.3578	69.23739	322.1052	61.32379	320.3055	65.13677
322.3897	69.23615	322.1472	61.32354	320.3482	65.13673
322.4329	69.2348	322.1896	61.32377	320.3898	65.13608
322.4724	69.228	322.232	61.32074	320.433	65.13637
322.5141	69.22393	322.2729	61.32181	320.4714	65.13556
322.5572	69.2233	322.315	61.32046	320.5146	65.13453
322.597	69.22454	322.3532	61.31878	320.5548	65.13465
322.6375	69.22992	322.3965	61.31834	320.5877	65.1344
322.68	69.23107	322.4383	61.31713	320.6276	65.13393
322.725	69.2291	322.4799	61.31495	320.6684	65.13335
322.7659	69.22862	322.514	61.31366	320.7125	65.13173
322.8044	69.22671	322.5536	61.31304	320.755	65.12924
322.8467	69.22021	322.5972	61.31234	320.7941	65.1273
322.8884	69.21563	322.6358	61.31284	320.8348	65.12593

322.9234	69.21061	322.677	61.3139	320.8763	65.1252
322.9646	69.20244	322.7194	61.31303	320.9181	65.12484
323.007	69.20185	322.7628	61.31137	320.962	65.12418
323.0486	69.20184	322.8015	61.31133	321.0064	65.12322
323.091	69.20684	322.845	61.30906	321.045	65.12203
323.1299	69.21231	322.8872	61.31094	321.0871	65.12079
323.1702	69.21255	322.9265	61.31502	321.128	65.12044
323.2134	69.21615	322.9606	61.3145	321.1678	65.12064
323.257	69.21371	323.0015	61.31466	321.2019	65.1207
323.2978	69.20694	323.0448	61.31606	321.2425	65.12004
323.3414	69.20384	323.0835	61.31295	321.2835	65.11903
323.3746	69.20173	323.1257	61.31289	321.3236	65.11779
323.4128	69.19718	323.1663	61.31132	321.3655	65.11677
323.4574	69.19669	323.209	61.30779	321.4078	65.11556
323.4964	69.19473	323.2502	61.30642	321.4476	65.11491
323.536	69.19107	323.2926	61.30371	321.492	65.11336
323.5797	69.1872	323.3314	61.30186	321.5341	65.11206
323.62	69.18567	323.3691	61.30169	321.576	65.11143
323.6628	69.18326	323.4029	61.30135	321.6176	65.11006
323.7053	69.1833	323.4445	61.29827	321.6565	65.10962
323.7451	69.18315	323.4858	61.2989	321.6966	65.10908
323.7881	69.18093	323.527	61.29914	321.7302	65.10824
323.8204	69.18213	323.5684	61.29567	321.774	65.10747
323.86	69.17808	323.6098	61.29648	321.8164	65.10627
323.9005	69.17487	323.6515	61.29563	321.8562	65.10477
323.9434	69.17349	323.693	61.29364	321.8997	65.10402
323.9852	69.16903	323.7359	61.29411	321.9391	65.10468
324.0264	69.1636	323.7773	61.29263	321.9828	65.10496
324.0696	69.16183	323.8188	61.29137	322.0244	65.10464
324.1115	69.16317	323.8518	61.29113	322.0659	65.10402
324.1518	69.16728	323.8946	61.2916	322.1067	65.10208
324.194	69.17239	323.9383	61.29262	322.1476	65.10076
324.239	69.17771	323.9767	61.29355	322.187	65.10007
324.269	69.17754	324.0182	61.29339	322.2303	65.09934
324.3105	69.17526	324.0615	61.29031	322.2712	65.09908
324.354	69.17002	324.1014	61.289	322.3125	65.09814
324.3937	69.1637	324.1447	61.28723	322.3484	65.09713
324.4365	69.15822	324.184	61.28611	322.3889	65.09564
324.4764	69.15338	324.2287	61.28813	322.4265	65.09379
324.5152	69.15293	324.2696	61.28813	322.4636	65.09279
324.5576	69.15777	324.3028	61.28949	322.5064	65.09227
324.5989	69.15741	324.346	61.28992	322.5463	65.09195
324.641	69.15537	324.3872	61.28985	322.5896	65.09207
324.6822	69.15874	324.427	61.29009	322.6309	65.09138
324.7228	69.15848	324.4688	61.2868	322.6689	65.09008
324.7663	69.15686	324.5132	61.28409	322.7135	65.08917
324.8	69.15452	324.5538	61.28018	322.7554	65.0873
324.8407	69.15009	324.5944	61.27721	322.7984	65.08678
324.88	69.14817	324.6362	61.27802	322.8384	65.08618
324.9238	69.14772	324.6779	61.27888	322.8807	65.08577
324.9658	69.15167	324.7188	61.28205	322.9216	65.08533
325.0071	69.1531	324.7519	61.28255	322.9532	65.08439
325.0496	69.15067	324.7958	61.28064	322.9944	65.08341
325.0892	69.15299	324.8346	61.27801	323.0375	65.08205
325.1299	69.15516	324.8774	61.27617	323.0798	65.08079
325.1708	69.15826	324.9202	61.27344	323.1216	65.08009
325.2122	69.15704	324.9604	61.2733	323.1608	65.07948
325.2458	69.15086	325.0028	61.27404	323.2031	65.07929

325.2868	69.14733	325.041	61.27057	323.242	65.07926
325.3308	69.14543	325.0844	61.27012	323.2838	65.07878
325.3706	69.14205	325.126	61.2701	323.3264	65.07861
325.4081	69.13959	325.169	61.26872	323.3694	65.07755
325.4532	69.13341	325.2004	61.26976	323.411	65.07704
325.4949	69.12498	325.243	61.27083	323.4529	65.07639
325.5353	69.11965	325.2822	61.27092	323.4863	65.07565
325.5755	69.11844	325.326	61.27167	323.5288	65.07519
325.6181	69.12479	325.3685	61.27252	323.5687	65.07459
325.6586	69.1312	325.4067	61.26972	323.6102	65.07293
325.6932	69.13407	325.449	61.26738	323.6506	65.07109
325.7352	69.13729	325.4919	61.26833	323.694	65.06943
325.7776	69.13321	325.5336	61.26616	323.735	65.0681
325.8194	69.12763	325.5752	61.26617	323.776	65.06761
325.8592	69.12083	325.6178	61.26705	323.8211	65.0673
325.9	69.11353	325.649	61.26661	323.8608	65.06707
325.9402	69.10903	325.689	61.2687	323.8996	65.06685
325.983	69.10421	325.7305	61.27155	323.941	65.06662
326.0236	69.10283	325.7735	61.27288	323.9864	65.06627
326.0634	69.10317	325.8144	61.27288	324.0259	65.06557
326.1066	69.10437	325.8571	61.26961	324.0664	65.06468
326.1382	69.106	325.9001	61.26499	324.0996	65.06371
326.1831	69.10731	325.94	61.26137	324.1424	65.06242
326.2218	69.11169	325.9818	61.25744	324.1831	65.06057
326.2634	69.10051	326.0227	61.25526	324.2247	65.05933
326.3062	69.04456	326.0629	61.25659	324.2659	65.0584
326.3481	68.98042	326.0985	61.25625	324.3053	65.05777
326.3896	68.91343	326.1396	61.25499	324.348	65.05748
326.4292	68.8627	326.18	61.2548	324.3902	65.05714
326.4716	68.85522	326.2189	61.25485	324.431	65.05648
326.5132	68.85709	326.2598	61.25429	324.474	65.05571
326.5542	68.85544	326.3053	61.25331	324.5134	65.05567
326.5973	68.85467	326.3442	61.2523	324.5544	65.05523
326.6398	68.8527	326.3864	61.25153	324.5945	65.05427
326.6722	68.84949	326.4294	61.25056	324.6405	65.0537
326.7148	68.84433	326.4717	61.24984	324.6844	65.05234
326.7562	68.83917	326.5143	61.251	324.7145	65.05194
326.797	68.83468	326.547	61.25245	324.756	65.052
326.8364	68.8312	326.588	61.25153	324.7959	65.05105
326.8795	68.82878	326.6295	61.2508	324.8392	65.05116
326.9226	68.82756	326.669	61.25267	324.8852	65.05058
326.9624	68.82448	326.7116	61.25128	324.923	65.04974
327.0038	68.81843	326.7535	61.25096	324.966	65.04969
327.0464	68.81965	326.7951	61.25336	325.0091	65.04926
327.0886	68.82126	326.8372	61.24986	325.0484	65.04833
327.1219	68.82173	326.8762	61.24527	325.0904	65.0479
327.1636	68.82177	326.9178	61.24105	325.133	65.04634
327.2062	68.82003	326.9615	61.23863	325.175	65.04515
327.246	68.81416	326.9956	61.23774	325.2159	65.04331
327.2882	68.81042	327.039	61.24003	325.2507	65.04235
327.3296	68.80865	327.0779	61.24257	325.2928	65.04231
327.3675	68.80938	327.1178	61.24145	325.3307	65.04173
327.4106	68.81401	327.1625	61.24059	325.3735	65.04105
327.4521	68.81468	327.2033	61.23846	325.417	65.03961
327.4968	68.81464	327.243	61.23871	325.4574	65.03797
327.5384	68.8127	327.2838	61.2392	325.4993	65.03622
327.572	68.80721	327.3231	61.23935	325.5384	65.03596
327.6145	68.80292	327.366	61.23797	325.5791	65.03509

327.6531	68.80709	327.4104	61.23898	325.6204	65.03414
327.6966	68.81546	327.4428	61.2402	325.6654	65.03385
327.7352	68.81953	327.4835	61.23701	325.7058	65.03364
327.7806	68.82085	327.5263	61.23865	325.7502	65.03338
327.8203	68.82201	327.57	61.23785	325.7914	65.03358
327.8622	68.8212	327.6104	61.23713	325.8326	65.0327
327.9036	68.82466	327.6535	61.23865	325.8646	65.03215
327.9438	68.82992	327.6936	61.23615	325.9078	65.03147
327.9864	68.82958	327.735	61.23376	325.9503	65.02982
328.02	68.82459	327.7759	61.2307	325.991	65.02811
328.0621	68.81888	327.8171	61.22801	326.0289	65.02577
328.104	68.81009	327.8566	61.22732	326.0725	65.02495
328.1439	68.80311	327.8928	61.22832	326.1139	65.02486
328.1848	68.80068	327.9346	61.22719	326.1548	65.02484
328.2248	68.79796	327.9753	61.22769	326.197	65.02478
328.2649	68.80286	328.0186	61.23122	326.2389	65.0239
328.3079	68.80007	328.0594	61.23052	326.281	65.02276
328.3524	68.79352	328.1007	61.22974	326.3219	65.02231
328.3918	68.79107	328.1428	61.23153	326.3622	65.02226
328.4318	68.78573	328.1828	61.22853	326.4054	65.02208
328.4763	68.78838	328.2244	61.22596	326.4496	65.02139
328.5183	68.79278	328.2655	61.22812	326.4805	65.02059
328.5488	68.79405	328.308	61.22609	326.5224	65.01958
328.5876	68.79642	328.3385	61.22483	326.5662	65.01823
328.6317	68.79214	328.3803	61.22541	326.6062	65.01729
328.6724	68.78666	328.422	61.22381	326.6468	65.01656
328.7168	68.78314	328.4646	61.21973	326.688	65.01572
328.7571	68.775	328.5046	61.21645	326.7282	65.01482
328.7992	68.77346	328.5449	61.21471	326.7698	65.01465
328.8417	68.77349	328.5891	61.21367	326.8135	65.01414
328.8802	68.77012	328.63	61.21609	326.8522	65.01371
328.924	68.76771	328.671	61.21825	326.8926	65.01301
328.9649	68.77014	328.713	61.21929	326.9366	65.01181
328.9995	68.77378	328.7552	61.22139	326.9788	65.01139
329.039	68.77631	328.7892	61.22052	327.0106	65.01161
329.0834	68.77917	328.8313	61.21817	327.0556	65.01081
329.1212	68.78295	328.8722	61.21785	327.0955	65.00935
329.1621	68.7859	328.9143	61.21536	327.1373	65.008
329.2035	68.78259	328.9558	61.21321	327.1769	65.00565
329.2468	68.77872	328.9982	61.21386	327.2208	65.0047
329.2884	68.77077	329.0386	61.21231	327.2625	65.00437
329.332	68.7588	329.0806	61.21187	327.3056	65.00374
329.3717	68.75317	329.121	61.21425	327.3456	65.00331
329.4174	68.75116	329.1626	61.21403	327.3875	65.002
329.4479	68.74971	329.2078	61.21256	327.43	65.00151
329.4894	68.74743	329.2393	61.21304	327.4696	65.00094
329.5283	68.74795	329.2806	61.21282	327.51	65.00083
329.571	68.75709	329.321	61.2105	327.5529	65.00108
329.614	68.76805	329.3613	61.21268	327.5961	65.00042
329.654	68.77388	329.4046	61.21449	327.6287	64.99924
329.6953	68.7804	329.4459	61.21014	327.6694	64.99777
329.7374	68.78067	329.4889	61.20924	327.7102	64.99662
329.7797	68.77384	329.53	61.20733	327.7545	64.99524
329.8188	68.77177	329.5704	61.20365	327.7971	64.99484
329.8618	68.7678	329.611	61.20513	327.8383	64.99461
329.8954	68.76188	329.6543	61.20636	327.8802	64.99467
329.9358	68.75612	329.6887	61.20671	327.9213	64.99458
329.9766	68.74982	329.7289	61.20723	327.9619	64.99427

330.0181	68.74627	329.7706	61.20762	328.0023	64.9938
330.058	68.74462	329.8126	61.20728	328.0424	64.99338
330.1015	68.7427	329.8544	61.20711	328.0832	64.99322
330.1424	68.74168	329.8971	61.2051	328.1286	64.99329
330.185	68.74206	329.9366	61.20331	328.1674	64.99216
330.2268	68.74183	329.9778	61.20329	328.2094	64.99093
330.2688	68.74195	330.0212	61.2031	328.2412	64.99087
330.3108	68.74317	330.0645	61.20268	328.2853	64.98987
330.3519	68.73863	330.1078	61.20307	328.3242	64.98857
330.3916	68.73197	330.1402	61.20272	328.3658	64.98774
330.4248	68.72584	330.185	61.20038	328.4079	64.98608
330.4664	68.71901	330.2232	61.20044	328.4519	64.98509
330.5064	68.71484	330.2656	61.19996	328.4944	64.98503
330.5479	68.71143	330.3053	61.19636	328.533	64.98502
330.591	68.71147	330.3492	61.1974	328.574	64.98498
330.6344	68.71234	330.3912	61.19745	328.6156	64.98379
330.675	68.71223	330.4306	61.19568	328.6571	64.98328
330.7162	68.71728	330.4725	61.19811	328.6985	64.98237
330.7563	68.71882	330.5132	61.19691	328.7364	64.98179
330.8	68.71542	330.5565	61.19391	328.7689	64.98158
330.8412	68.71256	330.5872	61.19319	328.813	64.98088
330.8734	68.71046	330.6296	61.19406	328.8535	64.97968
330.9124	68.71171	330.6688	61.19141	328.8958	64.97817
330.9555	68.71206	330.7094	61.19007	328.9347	64.9765
330.9968	68.71183	330.7532	61.19093	328.9766	64.97565
331.0384	68.70982	330.7944	61.18755	329.0198	64.97452
331.0796	68.70523	330.836	61.18697	329.0608	64.97434
331.1194	68.70258	330.8775	61.18775	329.1045	64.97467
331.1634	68.70176	330.9178	61.18686	329.1446	64.97442
331.2042	68.70107	330.9606	61.1875	329.1864	64.97446
331.2461	68.7001	331.0026	61.18835	329.2282	64.97352
331.2876	68.70044	331.0339	61.18837	329.2696	64.97242
331.322	68.70447	331.0776	61.19002	329.3111	64.97177
331.3668	68.70689	331.1191	61.18891	329.35	64.97133
331.4074	68.7046	331.1586	61.18775	329.3828	64.97037
331.4487	68.70371	331.2022	61.18649	329.4268	64.96977
331.4898	68.70253	331.2456	61.18288	329.4698	64.96898
331.5326	68.70117	331.2845	61.18022	329.5122	64.9684
331.5723	68.70047	331.3268	61.17976	329.5521	64.96846
331.614	68.70047	331.3666	61.18168	329.593	64.96888
331.6556	68.69723	331.4094	61.18344	329.633	64.96856
331.6956	68.69195	331.4426	61.1853	329.6758	64.96842
331.7384	68.69069	331.476	61.18491	329.7172	64.96769
331.768	68.69162	331.5202	61.18267	329.7576	64.96675
331.8117	68.69406	331.5638	61.1802	329.8016	64.96612
331.855	68.6968	331.6028	61.17803	329.8422	64.96457
331.8941	68.69554	331.6455	61.17751	329.886	64.96343
331.9365	68.69566	331.6878	61.17801	329.9248	64.96224
331.9774	68.69317	331.7294	61.1768	329.9667	64.96071
332.018	68.68907	331.77	61.17793	329.9992	64.9601
332.0601	68.6884	331.8128	61.18066	330.0407	64.95923
332.101	68.68602	331.854	61.18077	330.0856	64.95854
332.143	68.68339	331.8972	61.18007	330.1258	64.95885
332.1836	68.68192	331.9294	61.17884	330.1659	64.95854
332.2255	68.68134	331.9692	61.17544	330.2102	64.95854
332.2688	68.68145	332.0122	61.17223	330.2508	64.95853
332.3024	68.68266	332.053	61.17363	330.2916	64.95764
332.3429	68.68302	332.0965	61.17448	330.3332	64.95709

332.384	68.68525	332.1368	61.17396	330.3757	64.95656
332.4272	68.68282	332.179	61.17478	330.418	64.95584
332.469	68.67774	332.2189	61.17414	330.4565	64.95491
332.5088	68.67567	332.2624	61.1722	330.498	64.95435
332.5516	68.67391	332.305	61.17196	330.5332	64.95321
332.5934	68.67366	332.3452	61.16929	330.5768	64.95169
332.6321	68.67266	332.3813	61.1671	330.6184	64.95043
332.6769	68.67216	332.4206	61.16886	330.6576	64.94886
332.7195	68.66876	332.4608	61.16814	330.7	64.94824
332.7515	68.66669	332.5045	61.1662	330.7428	64.94794
332.7934	68.667	332.5438	61.16632	330.7815	64.94757
332.8352	68.66806	332.5842	61.16502	330.8226	64.94806
332.877	68.66667	332.6266	61.16334	330.8655	64.9476
332.9156	68.66663	332.6688	61.16472	330.9086	64.94718
332.9584	68.66687	332.7108	61.16537	330.9502	64.94685
332.9998	68.66201	332.7512	61.16511	330.9896	64.9465
333.0419	68.65887	332.7928	61.16545	331.0324	64.94571
333.0836	68.66091	332.826	61.16453	331.0735	64.94414
333.124	68.66132	332.8672	61.16387	331.1149	64.94312
333.1664	68.66023	332.9097	61.16216	331.1481	64.94212
333.1986	68.65962	332.9503	61.16038	331.1918	64.94038
333.2406	68.65521	332.9908	61.1602	331.2333	64.93966
333.2826	68.65237	333.0312	61.16296	331.2755	64.93898
333.3257	68.65585	333.0735	61.16384	331.3152	64.93711
333.3674	68.65975	333.1158	61.16226	331.3554	64.93642
333.4065	68.66722	333.1578	61.16203	331.3982	64.93564
333.4486	68.6703	333.2009	61.15859	331.4397	64.93462
333.4916	68.66636	333.242	61.15532	331.4794	64.93453
333.535	68.66664	333.2744	61.1577	331.5216	64.93474
333.5734	68.65798	333.3135	61.15692	331.5632	64.93505
333.6149	68.64829	333.3553	61.15415	331.6014	64.93546
333.6498	68.64643	333.3973	61.1574	331.6447	64.93481
333.691	68.64328	333.4387	61.15754	331.6858	64.93382
333.7331	68.64188	333.4784	61.15676	331.7264	64.93285
333.771	68.64285	333.5197	61.15514	331.7565	64.93229
333.8162	68.64278	333.5622	61.15214	331.8022	64.93235
333.856	68.6415	333.603	61.14928	331.8426	64.93223
333.896	68.63788	333.646	61.15139	331.8833	64.9312
333.9406	68.63472	333.6885	61.15273	331.9269	64.92978
333.9812	68.63375	333.7219	61.15173	331.9684	64.92836
334.0247	68.63149	333.7622	61.15165	332.0096	64.928
334.0609	68.633	333.8011	61.15156	332.055	64.92848
334.1062	68.63404	333.8439	61.14841	332.0938	64.92896
334.1472	68.63658	333.8879	61.14909	332.1389	64.92947
334.1816	68.63989	333.9287	61.14795	332.1798	64.92926
334.2212	68.63928	333.9705	61.14466	332.2212	64.92853
334.2641	68.63996	334.0113	61.14611	332.2632	64.92789
334.3041	68.63903	334.052	61.14704	332.2972	64.92742
334.3479	68.63324	334.0954	61.14673	332.3376	64.9257
334.3868	68.63154	334.138	61.14647	332.38	64.92516
334.4287	68.62987	334.1697	61.14647	332.4202	64.92464
334.468	68.625	334.2138	61.14471	332.4627	64.92398
334.5102	68.62622	334.2544	61.14321	332.5046	64.92355
334.5525	68.62809	334.2988	61.14291	332.5473	64.92305
334.5956	68.63217	334.3403	61.14107	332.5865	64.92204
334.6272	68.63462	334.3808	61.1394	332.6306	64.92101
334.6676	68.63715	334.4236	61.13769	332.6702	64.92068
334.709	68.64043	334.4635	61.13581	332.7119	64.91992

334.7498	68.63898	334.5024	61.13638	332.7533	64.91942
334.7946	68.63736	334.545	61.1364	332.7954	64.91842
334.8326	68.63322	334.5874	61.13648	332.8358	64.91737
334.876	68.62537	334.6236	61.13795	332.877	64.9167
334.9168	68.61744	334.6627	61.13841	332.9114	64.916
334.9587	68.61592	334.706	61.13755	332.951	64.91547
334.9975	68.6195	334.7484	61.138	332.9934	64.91605
335.0386	68.62157	334.7894	61.13622	333.0355	64.91474
335.0727	68.6207	334.831	61.13329	333.0798	64.91422
335.113	68.61974	334.8715	61.13041	333.1199	64.91362
335.1545	68.61558	334.912	61.1265	333.161	64.91258
335.1958	68.61024	334.9546	61.12469	333.2028	64.91249
335.2389	68.60816	334.9974	61.12181	333.2441	64.9119
335.28	68.60726	335.0381	61.11982	333.2885	64.91217
335.3194	68.60748	335.0738	61.11902	333.328	64.91158
335.3626	68.60766	335.1131	61.11787	333.3691	64.91064
335.4071	68.60898	335.1531	61.117	333.4126	64.90968
335.446	68.60955	335.1943	61.11682	333.4528	64.90848
335.487	68.6067	335.239	61.1162	333.4944	64.90731
335.5188	68.60403	335.2802	61.11437	333.5289	64.90685
335.5632	68.60366	335.3186	61.11297	333.5678	64.90694
335.6054	68.60079	335.3625	61.11164	333.6111	64.90663
335.6453	68.59775	335.4019	61.11077	333.6528	64.90645
335.6863	68.59767	335.444	61.11025	333.6936	64.90612
335.7266	68.5992	335.488	61.109	333.7352	64.90448
335.7674	68.59947	335.5206	61.10819	333.7756	64.90368
335.8082	68.60005	335.5648	61.10656	333.82	64.90285
335.8505	68.5982	335.6058	61.10496	333.8611	64.90251
335.8938	68.59446	335.644	61.1038	333.9023	64.90273
335.9362	68.59198	335.6874	61.10198	333.9445	64.90199
335.9758	68.58922	335.729	61.10063	333.9857	64.90181
336.0168	68.58972	335.7691	61.10026	334.0264	64.90113
336.0504	68.59155	335.8112	61.09992	334.0576	64.90012
336.093	68.59328	335.8522	61.09988	334.1006	64.89904
336.1341	68.59461	335.8951	61.10064	334.141	64.89876
336.1786	68.59487	335.9372	61.1007	334.1822	64.89759
336.2226	68.59124	335.971	61.10014	334.2225	64.89704
336.2619	68.5886	336.0128	61.09972	334.266	64.89642
336.303	68.58709	336.0555	61.09896	334.3098	64.89547
336.3442	68.58437	336.0952	61.09773	334.3512	64.89507
336.3844	68.58307	336.1372	61.09743	334.3906	64.89499
336.428	68.5821	336.1774	61.09765	334.4336	64.89489
336.4678	68.58066	336.2203	61.09743	334.4738	64.89434
336.5034	68.58062	336.2615	61.09741	334.5166	64.89368
336.5426	68.58109	336.303	61.09651	334.5606	64.89328
336.584	68.58277	336.344	61.09491	334.5974	64.89214
336.6289	68.58619	336.3849	61.09445	334.6393	64.89129
336.666	68.58662	336.4178	61.0943	334.6714	64.89106
336.7116	68.58716	336.4605	61.09445	334.7124	64.89038
336.7531	68.5872	336.5022	61.09491	334.7528	64.89013
336.7948	68.58271	336.5427	61.09551	334.7915	64.88979
336.8355	68.58055	336.583	61.09595	334.8372	64.88949
336.8784	68.58108	336.6278	61.09624	334.879	64.88869
336.9187	68.5787	336.6665	61.09615	334.9208	64.88803
336.9516	68.57716	336.7097	61.09564	334.9614	64.88685
336.9913	68.57531	336.7529	61.09497	335.001	64.88568
337.0325	68.57166	336.7933	61.0947	335.0422	64.88494
337.0733	68.56892	336.833	61.09409	335.0792	64.88433

337.1158	68.56737	336.8657	61.09387	335.124	64.88403
337.1558	68.56877	336.9082	61.09315	335.1678	64.88282
337.1984	68.56884	336.9498	61.0914	335.2068	64.88248
337.2419	68.56635	336.9908	61.09051	335.2496	64.88176
337.2828	68.56422	337.0314	61.08947	335.283	64.88096
337.3226	68.565	337.073	61.08893	335.3258	64.8809
337.3638	68.56596	337.1146	61.08876	335.3664	64.88041
337.397	68.56597	337.1554	61.08882	335.4069	64.87979
337.4378	68.56621	337.1986	61.0884	335.4498	64.87972
337.4797	68.56521	337.2417	61.08766	335.4905	64.87989
337.5208	68.56262	337.2842	61.08737	335.5336	64.87972
337.5631	68.5645	337.3159	61.08666	335.5737	64.8794
337.6062	68.56683	337.356	61.08581	335.6178	64.87924
337.6468	68.5649	337.399	61.086	335.658	64.87844
337.6885	68.56377	337.439	61.08625	335.6992	64.8777
337.7294	68.56359	337.4804	61.08775	335.7415	64.87678
337.7728	68.566	337.522	61.08987	335.7832	64.87616
337.8157	68.56919	337.5632	61.09063	335.8176	64.87536
337.8555	68.56929	337.6067	61.09121	335.856	64.87416
337.8982	68.56601	337.6434	61.09022	335.897	64.87338
337.9326	68.56153	337.684	61.0884	335.9411	64.87265
337.9722	68.55539	337.7268	61.08724	335.98	64.87165
338.0156	68.55073	337.7599	61.08576	336.0236	64.8713
338.0578	68.54977	337.8014	61.08546	336.0646	64.87078
338.0985	68.54555	337.84	61.08661	336.1069	64.87034
338.1393	68.54457	337.8844	61.08772	336.1486	64.8705
338.1826	68.54805	337.925	61.08688	336.1886	64.87033
338.2231	68.54903	337.9675	61.08479	336.23	64.86989
338.265	68.54876	338.011	61.08224	336.2714	64.86881
338.3066	68.54945	338.0504	61.08063	336.3144	64.86801
338.3475	68.54838	338.0906	61.08099	336.3558	64.86806
338.3812	68.54511	338.1327	61.08235	336.3996	64.86745
338.4226	68.54478	338.176	61.08241	336.4303	64.86705
338.4634	68.54579	338.2078	61.08241	336.472	64.86674
338.5055	68.54316	338.2494	61.08332	336.5126	64.86609
338.5464	68.54079	338.2908	61.08351	336.553	64.86609
338.5879	68.54127	338.3317	61.08349	336.5947	64.86603
338.6265	68.5414	338.3733	61.08334	336.6362	64.86514
338.67	68.53884	338.4152	61.08172	336.6758	64.86397
338.7114	68.5384	338.4553	61.07984	336.7172	64.86315
338.7533	68.53761	338.4959	61.07941	336.7593	64.86294
338.795	68.53684	338.5383	61.07862	336.8014	64.8631
338.8241	68.53716	338.5787	61.07826	336.8452	64.86278
338.8681	68.53547	338.6216	61.07974	336.889	64.86217
338.9138	68.53314	338.6554	61.08017	336.9274	64.86189
338.9516	68.52885	338.6954	61.08029	336.9696	64.86105
338.991	68.52629	338.738	61.07978	337.0118	64.8606
339.0334	68.52514	338.7824	61.07859	337.0436	64.86054
339.0766	68.52442	338.822	61.07734	337.0832	64.85977
339.1182	68.52264	338.8624	61.07682	337.1288	64.8589
339.1589	68.52225	338.906	61.07741	337.1711	64.85813
339.1984	68.5226	338.9481	61.07828	337.2145	64.85746
339.2415	68.52366	338.9924	61.07852	337.2566	64.85715
339.2724	68.52609	339.034	61.0779	337.2968	64.856
339.315	68.5243	339.0755	61.07669	337.3348	64.85538
339.3554	68.52175	339.1094	61.07552	337.377	64.85464
339.3965	68.52109	339.15	61.07446	337.4196	64.85348
339.4382	68.52246	339.1885	61.07313	337.4597	64.8541

339.482	68.52386	339.2324	61.07279	337.502	64.85438
339.5224	68.52605	339.2736	61.07233	337.544	64.85464
339.564	68.52644	339.3168	61.07135	337.5794	64.85432
339.6051	68.52362	339.3564	61.07255	337.6216	64.85315
339.648	68.52266	339.3996	61.07298	337.6644	64.85235
339.6864	68.52137	339.4387	61.07451	337.7063	64.85082
339.7294	68.51991	339.4816	61.07465	337.7474	64.85047
339.7732	68.51974	339.5224	61.07383	337.7904	64.85017
339.8054	68.52101	339.5576	61.07334	337.833	64.8498
339.8471	68.52047	339.596	61.07138	337.8752	64.84945
339.8859	68.51932	339.637	61.07055	337.9134	64.84851
339.929	68.5188	339.6808	61.06992	337.9553	64.84746
339.969	68.51537	339.7223	61.06975	337.9982	64.84605
340.0092	68.51135	339.7629	61.06951	338.0391	64.84523
340.0542	68.51297	339.8037	61.06924	338.0781	64.84509
340.0934	68.51514	339.849	61.06999	338.121	64.8445
340.1348	68.51639	339.889	61.07013	338.1636	64.84412
340.1765	68.51714	339.932	61.06862	338.1972	64.84322
340.2206	68.51538	339.9738	61.06846	338.2399	64.84178
340.252	68.51359	340.0089	61.06792	338.28	64.84129
340.2956	68.5104	340.0483	61.0678	338.3233	64.84127
340.3362	68.51045	340.0894	61.06942	338.365	64.84179
340.3795	68.51251	340.1314	61.07015	338.4059	64.84197
340.4201	68.51014	340.1738	61.0697	338.4484	64.84244
340.4626	68.50641	340.2155	61.06937	338.4902	64.84204
340.5028	68.50606	340.259	61.06809	338.5338	64.8416
340.5454	68.50495	340.2994	61.06646	338.5758	64.84162
340.5872	68.50343	340.3388	61.06562	338.6128	64.84144
340.627	68.50363	340.3786	61.06396	338.6553	64.84115
340.6703	68.50182	340.4196	61.06388	338.6977	64.84068
340.7054	68.499	340.4546	61.06393	338.7391	64.84009
340.7456	68.4969	340.4972	61.06463	338.781	64.8391
340.784	68.49683	340.5388	61.06473	338.8143	64.8385
340.8265	68.50056	340.5808	61.06519	338.8551	64.83784
340.8674	68.50164	340.6208	61.06692	338.895	64.83747
340.91	68.49934	340.6608	61.06588	338.9348	64.83658
340.9512	68.49845	340.7046	61.06445	338.9758	64.8359
340.993	68.49356	340.7416	61.06383	339.021	64.8353
341.0346	68.49071	340.7868	61.06117	339.0616	64.83432
341.0756	68.49049	340.8296	61.06124	339.1022	64.83367
341.1175	68.48956	340.871	61.06349	339.146	64.83342
341.1506	68.48897	340.9034	61.06285	339.1866	64.8335
341.194	68.48654	340.9439	61.06182	339.226	64.83288
341.2338	68.48459	340.9856	61.06135	339.269	64.83196
341.2762	68.48143	341.0301	61.05991	339.3122	64.83131
341.3171	68.47838	341.0691	61.05885	339.345	64.8305
341.3589	68.47734	341.1113	61.05978	339.3852	64.83042
341.3987	68.47633	341.1505	61.05961	339.4289	64.83088
341.4403	68.47653	341.1946	61.06003	339.4686	64.8302
341.4822	68.47995	341.2366	61.06124	339.5105	64.82947
341.5256	68.48114	341.2798	61.05978	339.552	64.82939
341.5668	68.48277	341.3194	61.06175	339.5925	64.8287
341.6074	68.4825	341.353	61.06129	339.6348	64.8286
341.651	68.48088	341.3942	61.05799	339.6747	64.82814
341.6814	68.48182	341.4352	61.05636	339.7175	64.82748
341.7234	68.48173	341.476	61.05153	339.7586	64.82704
341.7639	68.48026	341.5194	61.04686	339.8014	64.82694
341.8044	68.47926	341.5582	61.04568	339.8426	64.82705

341.8466	68.47365	341.5994	61.04573	339.8828	64.82593
341.888	68.46838	341.6433	61.04465	339.9228	64.82497
341.9288	68.46791	341.683	61.04543	339.9592	64.82382
341.9712	68.46963	341.7262	61.04691	339.9994	64.82275
342.0128	68.4719	341.7659	61.04742	340.0405	64.82185
342.0532	68.47155	341.8015	61.04875	340.083	64.82163
342.0967	68.4721	341.8431	61.05065	340.1266	64.82142
342.1283	68.47165	341.8828	61.0489	340.1633	64.82066
342.1687	68.47204	341.924	61.04708	340.2096	64.82066
342.2138	68.47226	341.9654	61.0453	340.2494	64.82087
342.2539	68.47365	342.0069	61.04338	340.2884	64.82061
342.2957	68.47165	342.05	61.04556	340.3316	64.82089
342.3369	68.4689	342.0914	61.04735	340.3735	64.82011
342.3794	68.4703	342.1336	61.04611	340.4156	64.81919
342.4227	68.46821	342.173	61.04847	340.4563	64.81902
342.4631	68.46623	342.2135	61.0511	340.4958	64.81906
342.5041	68.46231	342.2485	61.04984	340.5406	64.81836
342.544	68.45949	342.2906	61.05059	340.571	64.81719
342.576	68.45765	342.3308	61.04984	340.6164	64.81641
342.6199	68.45418	342.3726	61.0431	340.6538	64.81448
342.663	68.45343	342.4115	61.04117	340.6958	64.81434
342.7022	68.45399	342.4547	61.04367	340.7361	64.81469
342.7432	68.4537	342.497	61.04358	340.7769	64.81496
342.7864	68.45442	342.5381	61.04495	340.8192	64.81526
342.8272	68.45668	342.5809	61.04461	340.8625	64.81469
342.8697	68.45791	342.6233	61.04219	340.9028	64.81403
342.9094	68.45764	342.6658	61.04531	340.9446	64.81291
342.9496	68.45508	342.6989	61.04698	340.9856	64.81167
342.9924	68.45178	342.7395	61.04662	341.024	64.8115
343.0275	68.449	342.7838	61.04776	341.0676	64.8112
343.0662	68.44708	342.824	61.04735	341.1012	64.81077
343.1105	68.44582	342.8657	61.04359	341.1419	64.81064
343.1497	68.44613	342.9048	61.04359	341.1837	64.81042
343.1914	68.44441	342.9497	61.04288	341.225	64.80942
343.2332	68.44537	342.9878	61.04057	341.2684	64.80883
343.2738	68.44733	343.0297	61.0413	341.31	64.80831
343.318	68.44506	343.0725	61.04076	341.35	64.80747
343.3578	68.44378	343.1122	61.03976	341.393	64.80672
343.3989	68.44387	343.1469	61.03885	341.4347	64.8062
343.4422	68.44387	343.19	61.03883	341.4758	64.80665
343.4817	68.44331	343.2339	61.03987	341.5181	64.807
343.5217	68.44685	343.2734	61.04183	341.559	64.80708
343.5562	68.4464	343.3153	61.04093	341.5975	64.80665
343.596	68.44621	343.3562	61.03846	341.639	64.80544
343.6382	68.4471	343.3953	61.03833	341.6832	64.80468
343.6805	68.44453	343.4364	61.03642	341.7202	64.8045
343.723	68.44071	343.4769	61.03466	341.7574	64.80441
343.7662	68.43831	343.5194	61.03595	341.8004	64.80446
343.8069	68.43569	343.5596	61.0349	341.8408	64.8039
343.8472	68.43375	343.5938	61.03384	341.8837	64.80332
343.8884	68.43156	343.6343	61.03463	341.9258	64.80305
343.9292	68.42754	343.6769	61.03299	341.9649	64.80186
343.971	68.4255	343.7172	61.02982	342.0072	64.80165
344.0056	68.42349	343.7615	61.02948	342.0465	64.80148
344.047	68.42246	343.8	61.03078	342.0906	64.80034
344.09	68.42148	343.843	61.03179	342.1322	64.79984
344.1298	68.42058	343.8857	61.03223	342.1728	64.79893
344.1722	68.42218	343.9273	61.03108	342.2142	64.79814

344.214	68.4233	343.9691	61.029	342.2548	64.79787
344.254	68.42304	344.0076	61.03222	342.2972	64.79815
344.2967	68.42319	344.0425	61.03613	342.3297	64.79803
344.3374	68.42176	344.0828	61.0365	342.3733	64.79729
344.3794	68.41912	344.1254	61.0372	342.4152	64.79726
344.421	68.41943	344.1687	61.03553	342.4562	64.79656
344.454	68.42054	344.2105	61.03137	342.4961	64.7959
344.4965	68.41895	344.2518	61.03035	342.5372	64.79619
344.5372	68.41585	344.2937	61.02962	342.5806	64.79549
344.5798	68.4156	344.3352	61.02868	342.6224	64.79603
344.6196	68.41439	344.378	61.0289	342.6611	64.79621
344.661	68.41411	344.4184	61.02926	342.7052	64.79521
344.7028	68.41472	344.4608	61.02772	342.7467	64.79512
344.7439	68.41388	344.493	61.02559	342.7883	64.79479
344.7838	68.41132	344.5346	61.02245	342.8296	64.79353
344.8266	68.41111	344.5776	61.01911	342.867	64.79281
344.8698	68.41173	344.6183	61.02134	342.9062	64.79183
344.9024	68.41102	344.6567	61.02181	342.9469	64.78967
344.9443	68.40948	344.7017	61.02026	342.9903	64.78918
344.9844	68.41004	344.745	61.02191	343.034	64.78937
345.0269	68.41115	344.786	61.02004	343.0744	64.78987
345.0676	68.40921	344.8266	61.01946	343.1164	64.78981
345.1072	68.40751	344.8689	61.02215	343.1578	64.78935
345.1492	68.40388	344.908	61.02116	343.2006	64.78921
345.1906	68.39961	344.9416	61.01957	343.2396	64.78855
345.2334	68.3978	344.9845	61.01985	343.2832	64.78801
345.2738	68.39941	345.0244	61.01857	343.3234	64.78874
345.3157	68.40165	345.0665	61.01813	343.364	64.79053
345.3564	68.40175	345.1095	61.01981	343.4068	64.79065
345.3979	68.40046	345.1504	61.01829	343.4496	64.79075
345.4316	68.39904	345.1934	61.01636	343.4808	64.7905
345.4722	68.39668	345.2316	61.01612	343.5228	64.78803
345.5171	68.39349	345.2732	61.01354	343.5666	64.78594
345.5575	68.39552	345.3172	61.01169	343.6063	64.7853
345.6018	68.3979	345.36	61.0137	343.6488	64.78394
345.6406	68.39691	345.391	61.01434	343.6883	64.78287
345.6825	68.39592	345.432	61.01377	343.7294	64.7826
345.7252	68.39541	345.473	61.01549	343.7716	64.78241
345.7661	68.39435	345.5144	61.01458	343.8144	64.782
345.8064	68.39409	345.5562	61.01254	343.855	64.78214
345.8459	68.39271	345.5984	61.01228	343.8943	64.78192
345.8794	68.39157	345.6418	61.01018	343.9386	64.78193
345.9212	68.39128	345.6822	61.0091	343.9808	64.78205
345.9617	68.38826	345.7216	61.0107	344.0208	64.78115
346.0046	68.39102	345.764	61.00869	344.0637	64.7805
346.0467	68.39167	345.8065	61.00538	344.0939	64.78001
346.0864	68.38961	345.8381	61.00472	344.1366	64.77915
346.13	68.38894	345.882	61.00157	344.177	64.7794
346.1695	68.38621	345.9211	61.00068	344.219	64.77923
346.2112	68.38481	345.9643	61.00357	344.2608	64.77862
346.2538	68.38378	346.0044	61.00305	344.3028	64.77864
346.296	68.38467	346.0463	61.00155	344.3424	64.77822
346.3302	68.38476	346.0876	61.00089	344.3841	64.77736
346.3702	68.38337	346.1282	60.99951	344.4267	64.7766
346.4122	68.38423	346.1715	60.99874	344.469	64.77572
346.454	68.38549	346.212	60.99844	344.5094	64.77552
346.4974	68.38652	346.2526	60.99692	344.55	64.77466
346.5355	68.38685	346.2847	60.99734	344.5926	64.7744

346.5781	68.38516	346.3252	60.998	344.6261	64.77461
346.6176	68.38272	346.3681	60.9962	344.6672	64.77409
346.6593	68.37969	346.4102	60.99168	344.7088	64.77401
346.7023	68.37823	346.4528	60.9874	344.7509	64.77385
346.7406	68.3775	346.4938	60.98337	344.7914	64.77322
346.7761	68.37605	346.5375	60.9835	344.8342	64.77204
346.8186	68.37437	346.5765	60.98819	344.8728	64.77136
346.8588	68.37424	346.6176	60.99201	344.9166	64.77116
346.9012	68.37384	346.6594	60.99383	344.9578	64.77005
346.9434	68.37411	346.7011	60.99386	344.9998	64.77003
346.9861	68.37373	346.7348	60.9939	345.0438	64.77023
347.025	68.37387	346.7759	60.99218	345.0845	64.76916
347.0668	68.37318	346.8185	60.99246	345.1256	64.76875
347.1086	68.37133	346.8603	60.9939	345.1644	64.76794
347.1496	68.37038	346.9015	60.99462	345.2076	64.7679
347.1938	68.36847	346.942	60.99879	345.2446	64.7678
347.2346	68.36729	346.9824	60.99832	345.2834	64.76867
347.2765	68.36437	347.0245	60.99867	345.3232	64.76841
347.3082	68.36559	347.0668	60.99886	345.3657	64.76758
347.3498	68.36542	347.1085	60.99481	345.4087	64.76719
347.3929	68.3643	347.153	60.99432	345.4487	64.76581
347.4328	68.36524	347.1839	60.99321	345.4899	64.76519
347.477	68.3645	347.2263	60.99066	345.5313	64.76436
347.5166	68.36321	347.2692	60.99066	345.5714	64.76367
347.5572	68.36148	347.3106	60.99191	345.6112	64.76389
347.6004	68.35987	347.3517	60.99279	345.6569	64.76405
347.6411	68.35819	347.3914	60.99303	345.6994	64.76431
347.6842	68.3567	347.4368	60.99418	345.7396	64.76394
347.7246	68.35437	347.4759	60.99409	345.781	64.76373
347.7562	68.35444	347.52	60.9924	345.8204	64.76291
347.7981	68.35273	347.5619	60.99349	345.853	64.76272
347.8395	68.35181	347.6024	60.99354	345.8971	64.76225
347.8818	68.34984	347.6338	60.99433	345.9378	64.76104
347.9222	68.34779	347.6762	60.99863	345.9775	64.76026
347.9645	68.34713	347.7182	60.99741	346.0195	64.75978
348.0066	68.34661	347.76	60.99288	346.0602	64.75953
348.0466	68.34868	347.7998	60.99178	346.1026	64.76016
348.0884	68.34908	347.8408	60.98966	346.1434	64.76062
348.1295	68.3498	347.8822	60.99005	346.1836	64.76029
348.17	68.34755	347.925	60.99105	346.2272	64.76062
348.2057	68.34392	347.9654	60.98918	346.2708	64.75941
348.2446	68.34149	348.0091	60.98575	346.3089	64.75824
348.2859	68.34248	348.0469	60.98782	346.3533	64.75798
348.3265	68.34531	348.0794	60.98845	346.3853	64.75795
348.3727	68.34896	348.1207	60.98986	346.4263	64.75815
348.4145	68.34707	348.1604	60.9901	346.4695	64.75714
348.4558	68.34142	348.2031	60.98627	346.508	64.75609
348.4967	68.33962	348.247	60.98593	346.5517	64.755
348.5383	68.33677	348.2861	60.98662	346.592	64.75447
348.5794	68.33778	348.3272	60.98475	346.635	64.75503
348.6218	68.34243	348.3691	60.98304	346.6748	64.7552
348.6553	68.34174	348.4126	60.98281	346.719	64.75552
348.698	68.33905	348.4544	60.97942	346.7602	64.75454
348.7402	68.34009	348.4956	60.98046	346.7972	64.75326
348.7809	68.33922	348.5288	60.98347	346.8389	64.75222
348.8186	68.3352	348.5694	60.98264	346.8821	64.75081
348.859	68.33352	348.6116	60.98286	346.9213	64.75015
348.9022	68.33146	348.6539	60.98448	346.963	64.74995

348.944	68.33014	348.6944	60.98453	346.9957	64.74986
348.9863	68.33153	348.737	60.98572	347.0386	64.74956
349.0268	68.33383	348.7794	60.98704	347.0798	64.74921
349.0674	68.3336	348.8209	60.98586	347.1216	64.74923
349.11	68.32981	348.8632	60.98485	347.1638	64.74927
349.1492	68.32651	348.9036	60.98518	347.2064	64.74865
349.1842	68.32533	348.9434	60.98089	347.2475	64.74868
349.2234	68.32376	348.979	60.98049	347.2875	64.74808
349.269	68.3238	349.022	60.98297	347.3306	64.74679
349.3086	68.32356	349.0634	60.98032	347.3712	64.74647
349.3528	68.32339	349.1047	60.97912	347.4127	64.74589
349.3916	68.32234	349.1467	60.98006	347.4556	64.746
349.4364	68.32157	349.1887	60.97786	347.499	64.74621
349.4757	68.32141	349.2262	60.97613	347.5409	64.74808
349.5174	68.31888	349.2707	60.97555	347.5818	64.74872
349.5598	68.32095	349.3138	60.973	347.6166	64.749
349.601	68.32205	349.3519	60.97192	347.657	64.74928
349.6372	68.32334	349.3928	60.97007	347.6967	64.74698
349.6745	68.32407	349.4262	60.96926	347.7392	64.74564
349.7197	68.32062	349.4667	60.96981	347.7825	64.74487
349.7601	68.3183	349.5067	60.96939	347.8224	64.74413
349.8023	68.31507	349.5502	60.96761	347.8662	64.74458
349.8436	68.31184	349.5924	60.96841	347.9052	64.74408
349.8867	68.31313	349.6356	60.96788	347.9489	64.74413
349.9258	68.31392	349.6759	60.96536	347.991	64.74467
349.9681	68.31266	349.7185	60.9673	348.033	64.74404
350.0074	68.31359	349.7577	60.97068	348.0744	64.74342
350.051	68.31404	349.8015	60.97081	348.1162	64.74258
350.081	68.31344	349.8434	60.97185	348.1506	64.74202
350.1252	68.31212	349.8767	60.97381	348.1903	64.74069
350.166	68.31266	349.9169	60.97243	348.232	64.74044
350.2048	68.30972	349.9573	60.97283	348.2717	64.7404
350.2461	68.30809	349.9994	60.97418	348.3137	64.73925
350.2878	68.30707	350.04	60.97193	348.3547	64.7389
350.331	68.30484	350.0816	60.96943	348.3964	64.73864
350.3722	68.30421	350.1238	60.96642	348.4395	64.73757
350.4126	68.30253	350.1637	60.96296	348.4798	64.73726
350.4554	68.30154	350.2068	60.96541	348.5222	64.73681
350.4966	68.30151	350.2481	60.96842	348.562	64.73665
350.5289	68.30025	350.2889	60.9679	348.6011	64.73651
350.5709	68.29744	350.323	60.96834	348.6413	64.73661
350.6124	68.29568	350.3669	60.96429	348.6856	64.7363
350.6528	68.29636	350.4044	60.95916	348.727	64.73537
350.6969	68.29785	350.449	60.95836	348.7605	64.73529
350.7374	68.29869	350.4911	60.95899	348.803	64.73494
350.7804	68.29944	350.531	60.95899	348.844	64.73431
350.8214	68.29816	350.5716	60.96061	348.886	64.73461
350.8637	68.29573	350.615	60.95907	348.9229	64.73525
350.907	68.29498	350.6552	60.95743	348.9669	64.73498
350.9476	68.29199	350.6958	60.95743	349.0098	64.73502
350.9895	68.28868	350.738	60.95495	349.0508	64.73387
351.0294	68.28818	350.7713	60.95375	349.0912	64.73227
351.0625	68.28821	350.8118	60.95284	349.133	64.73131
351.1042	68.28844	350.8522	60.95019	349.1742	64.7303
351.1438	68.28913	350.8955	60.94763	349.2181	64.73003
351.1866	68.29018	350.9378	60.94838	349.2616	64.72941
351.2286	68.28825	350.9797	60.94751	349.301	64.72889
351.2697	68.28628	351.0232	60.94759	349.342	64.72878

351.3117	68.28597	351.0663	60.94895	349.373	64.72853
351.352	68.2854	351.1066	60.94858	349.4162	64.72819
351.3962	68.28442	351.1461	60.94979	349.4582	64.72779
351.4366	68.28549	351.1892	60.95057	349.5017	64.72777
351.4754	68.28475	351.2222	60.94848	349.5437	64.72834
351.5095	68.28265	351.2636	60.94984	349.5834	64.72826
351.5502	68.28067	351.3042	60.95327	349.6255	64.72841
351.5925	68.27863	351.346	60.95339	349.6656	64.72791
351.634	68.278	351.3883	60.95524	349.7066	64.72711
351.675	68.27974	351.4319	60.95483	349.7492	64.72736
351.7181	68.27799	351.4729	60.95144	349.7921	64.7267
351.7594	68.27827	351.5141	60.95149	349.8306	64.7258
351.8	68.27884	351.5541	60.95238	349.8744	64.72529
351.8448	68.27427	351.5947	60.95138	349.9085	64.72461
351.8835	68.27318	351.6364	60.95309	349.949	64.72544
351.9258	68.27333	351.6687	60.95287	349.9883	64.72567
351.9561	68.27305	351.712	60.94984	350.0288	64.72513
351.9982	68.27205	351.7507	60.9504	350.071	64.72373
352.0398	68.27355	351.7944	60.94674	350.1144	64.72176
352.0804	68.27455	351.8347	60.94375	350.1567	64.7218
352.123	68.27182	351.8777	60.94701	350.196	64.72204
352.1656	68.27147	351.9175	60.94585	350.2376	64.72193
352.2078	68.27136	351.9614	60.94486	350.2806	64.7215
352.2483	68.26791	352.0015	60.94712	350.3231	64.72042
352.289	68.26771	352.0445	60.94604	350.3634	64.71912
352.3306	68.26653	352.0846	60.94549	350.404	64.71895
352.374	68.26527	352.1199	60.9465	350.4464	64.71887
352.4072	68.26633	352.1609	60.94499	350.488	64.71803
352.4482	68.26519	352.2037	60.94426	350.5209	64.71798
352.4883	68.26298	352.2454	60.945	350.5666	64.71814
352.5313	68.26173	352.2861	60.94474	350.6045	64.71733
352.5734	68.25903	352.3253	60.94455	350.6463	64.71695
352.6132	68.26181	352.3665	60.94394	350.6866	64.71665
352.6554	68.26492	352.411	60.9434	350.7302	64.716
352.6962	68.26362	352.4496	60.9429	350.769	64.71582
352.7385	68.26354	352.4945	60.94481	350.8113	64.71633
352.7765	68.26103	352.5355	60.94457	350.8533	64.71695
352.8194	68.25875	352.566	60.94234	350.8962	64.71599
352.8614	68.25958	352.6108	60.94576	350.9365	64.71595
352.9023	68.25862	352.6514	60.94374	350.9789	64.71572
352.9365	68.25703	352.6935	60.94044	351.0196	64.71405
352.9766	68.25383	352.735	60.94123	351.0615	64.71335
353.0205	68.2505	352.7766	60.9367	351.1023	64.71273
353.0606	68.25147	352.817	60.93746	351.135	64.71165
353.1036	68.25084	352.8596	60.94079	351.178	64.71134
353.1463	68.25065	352.8985	60.94031	351.217	64.71181
353.1867	68.25127	352.9402	60.9426	351.2591	64.71191
353.2318	68.24987	352.9802	60.94241	351.3006	64.71134
353.2702	68.2483	353.0154	60.9389	351.342	64.71086
353.3123	68.24783	353.0566	60.93909	351.3871	64.70988
353.3542	68.24944	353.0975	60.94159	351.4274	64.70895
353.3864	68.24888	353.1399	60.94052	351.4664	64.70904
353.4285	68.25001	353.1799	60.9397	351.508	64.70951
353.47	68.24836	353.2213	60.93914	351.5484	64.70958
353.5111	68.2451	353.2648	60.93474	351.5918	64.70944
353.5532	68.24499	353.3064	60.93413	351.6312	64.70921
353.5945	68.24323	353.3479	60.9355	351.6654	64.70848
353.6353	68.24382	353.3918	60.93488	351.709	64.7085

353.6774	68.24346	353.4336	60.93433	351.7472	64.70834
353.7213	68.24058	353.4632	60.93434	351.7928	64.70811
353.7613	68.23977	353.5058	60.932	351.8344	64.70853
353.8048	68.23957	353.5483	60.9299	351.878	64.70807
353.8362	68.23923	353.5893	60.93004	351.9185	64.70866
353.8798	68.23879	353.63	60.92959	351.9598	64.70961
353.9207	68.24089	353.671	60.93203	352.0012	64.70805
353.9605	68.24112	353.7128	60.93657	352.0439	64.70732
354.0012	68.23905	353.7558	60.93632	352.0832	64.707
354.0422	68.23875	353.7963	60.93604	352.1278	64.70515
354.0844	68.23754	353.8363	60.93695	352.1695	64.70557
354.1242	68.23351	353.8796	60.936	352.21	64.70517
354.164	68.23306	353.9117	60.93469	352.2526	64.70413
354.2066	68.23312	353.9529	60.93527	352.288	64.70379
354.2488	68.23194	353.993	60.93297	352.3274	64.70264
354.2805	68.23221	354.0356	60.92767	352.3702	64.70195
354.3252	68.23284	354.0805	60.92659	352.4081	64.70246
354.3672	68.23188	354.1209	60.92421	352.4525	64.70224
354.4062	68.22825	354.1641	60.92377	352.4941	64.70232
354.4478	68.2266	354.204	60.92709	352.5369	64.70248
354.4893	68.22745	354.2444	60.92794	352.5776	64.70068
354.5316	68.22759	354.2879	60.92658	352.6192	64.69942
354.5734	68.22622	354.3292	60.92762	352.6605	64.69876
354.6145	68.22515	354.3626	60.92616	352.701	64.69841
354.6554	68.22304	354.4028	60.92566	352.7419	64.69902
354.6976	68.22012	354.447	60.92882	352.7838	64.69975
354.7402	68.21954	354.4889	60.92797	352.826	64.6994
354.7787	68.2211	354.5295	60.9243	352.8688	64.69855
354.8137	68.22131	354.5702	60.9229	352.9021	64.6975
354.8548	68.21925	354.6125	60.91978	352.9414	64.6966
354.896	68.21722	354.6542	60.91647	352.9836	64.69622
354.9395	68.21459	354.6946	60.91702	353.0235	64.69595
354.9788	68.21178	354.7367	60.91629	353.0646	64.69654
355.0195	68.21152	354.7803	60.91352	353.1072	64.69682
355.0616	68.21206	354.812	60.91278	353.1498	64.69595
355.1019	68.21191	354.8542	60.91167	353.1906	64.6952
355.1431	68.21161	354.8955	60.91157	353.2325	64.6937
355.1858	68.20991	354.9392	60.9133	353.2738	64.69223
355.2271	68.20906	354.98	60.91386	353.3152	64.69292
355.2608	68.20847	355.0203	60.91358	353.3556	64.69329
355.3012	68.20838	355.0613	60.913	353.3962	64.69316
355.3422	68.20838	355.1014	60.91377	353.4315	64.69279
355.3847	68.20969	355.1456	60.91459	353.473	64.69227
355.4254	68.2088	355.1858	60.91658	353.515	64.69152
355.4675	68.20657	355.23	60.91612	353.5552	64.69091
355.509	68.20741	355.2625	60.91419	353.595	64.69036
355.5508	68.2078	355.304	60.91431	353.6387	64.69129
355.5934	68.20673	355.3454	60.91307	353.6796	64.69189
355.6334	68.20978	355.3866	60.91065	353.72	64.69237
355.6744	68.20882	355.4278	60.90941	353.7624	64.69275
355.708	68.20461	355.469	60.90978	353.8021	64.69103
355.7494	68.2019	355.5104	60.9114	353.8444	64.68948
355.7912	68.19959	355.5528	60.91379	353.8865	64.68882
355.8326	68.19808	355.5959	60.91386	353.9268	64.68798
355.8706	68.19617	355.6361	60.9114	353.9692	64.68763
355.9157	68.19645	355.6772	60.90811	354.0122	64.68725
355.9583	68.19526	355.712	60.90701	354.0455	64.68733
355.9983	68.19217	355.7534	60.90513	354.0882	64.68696

356.0393	68.1913	355.7962	60.90613	354.1282	64.68682
356.08	68.19168	355.8345	60.90666	354.1694	64.68652
356.123	68.19105	355.8757	60.90552	354.2112	64.68585
356.1559	68.18952	355.9196	60.90953	354.254	64.68532
356.1985	68.18831	355.9597	60.9131	354.2944	64.68432
356.2388	68.18858	356.0011	60.91472	354.335	64.68379
356.2799	68.18719	356.0441	60.91496	354.376	64.68344
356.3215	68.1885	356.0864	60.9134	354.4166	64.68323
356.3639	68.18751	356.1263	60.90853	354.4602	64.68325
356.4059	68.18643	356.159	60.90554	354.5028	64.68351
356.4473	68.18456	356.2016	60.90482	354.5435	64.68302
356.491	68.18161	356.241	60.90339	354.5859	64.68355
356.531	68.18271	356.2835	60.90567	354.6274	64.68381
356.5716	68.18004	356.3268	60.90913	354.6576	64.68325
356.613	68.17691	356.3668	60.9085	354.7004	64.68307
356.655	68.17617	356.4059	60.90876	354.7442	64.68219
356.6888	68.17565	356.4478	60.90607	354.7828	64.68166
356.7308	68.17541	356.4922	60.90119	354.826	64.68155
356.7728	68.1773	356.5299	60.90151	354.8672	64.68172
356.8166	68.17808	356.5738	60.90125	354.908	64.68206
356.857	68.17515	356.6074	60.90107	354.9493	64.68122
356.8984	68.17298	356.6496	60.90425	354.9922	64.68065
356.9388	68.1705	356.6898	60.90709	355.0345	64.67944
356.9805	68.17087	356.7301	60.90649	355.0713	64.67897
357.0225	68.17168	356.7716	60.90585	355.1148	64.67976
357.0645	68.17439	356.8122	60.90291	355.1556	64.67998
357.1058	68.17548	356.8541	60.8998	355.1882	64.68003
357.139	68.1751	356.8979	60.89978	355.2321	64.67868
357.1806	68.1724	356.9416	60.89959	355.2734	64.6774
357.222	68.17048	356.9792	60.89999	355.314	64.67691
357.263	68.1694	357.0232	60.90234	355.3528	64.67637
357.3047	68.16749	357.0569	60.90124	355.3942	64.67604
357.3452	68.16847	357.0974	60.89949	355.4411	64.67614
357.389	68.16735	357.139	60.90195	355.4788	64.67538
357.4313	68.16617	357.1824	60.90343	355.5196	64.67508
357.472	68.16451	357.2265	60.90214	355.5642	64.67457
357.5135	68.16241	357.266	60.9016	355.604	64.67377
357.555	68.16128	357.3082	60.89676	355.646	64.6733
357.5856	68.15921	357.3492	60.8909	355.69	64.67261
357.628	68.15685	357.3894	60.89097	355.7296	64.67265
357.6709	68.1545	357.4312	60.89209	355.7709	64.67265
357.712	68.15412	357.4734	60.89676	355.8056	64.67269
357.7539	68.1542	357.5064	60.8998	355.8494	64.67247
357.7963	68.15309	357.5473	60.90051	355.8889	64.67253
357.8366	68.15404	357.589	60.89923	355.9322	64.67248
357.8783	68.15368	357.6294	60.8976	355.9721	64.67183
357.9202	68.14937	357.6709	60.89646	356.0133	64.67108
357.962	68.1485	357.7124	60.89407	356.0541	64.67045
358.0034	68.14604	357.7544	60.89495	356.0954	64.66914
358.0362	68.14453	357.7959	60.89468	356.1391	64.66813
358.076	68.14411	357.8384	60.89153	356.179	64.66813
358.1188	68.14341	357.8798	60.89402	356.2198	64.66846
358.1612	68.14346	357.9198	60.89763	356.264	64.66919
358.202	68.14299	357.9527	60.89739	356.3037	64.66986
358.2425	68.14233	357.9946	60.89637	356.3468	64.66949
358.2838	68.14145	358.0362	60.89123	356.3883	64.6683
358.3248	68.13982	358.0765	60.8841	356.4229	64.66733
358.3664	68.13648	358.1172	60.88106	356.4632	64.66635

358.4125	68.13419	358.1593	60.88049	356.5054	64.66652
358.4547	68.13176	358.2002	60.88461	356.5456	64.66647
358.4926	68.1286	358.2412	60.88729	356.587	64.66592
358.5364	68.12558	358.2837	60.88751	356.6308	64.66509
358.5672	68.12363	358.3262	60.88693	356.6688	64.66371
358.6101	68.12029	358.3706	60.88704	356.7116	64.66347
358.652	68.12051	358.4025	60.88643	356.7539	64.66379
358.6926	68.12128	358.443	60.88388	356.796	64.66407
358.7341	68.11997	358.482	60.88281	356.8366	64.66402
358.7753	68.11927	358.5252	60.88243	356.8785	64.66344
358.816	68.11505	358.5663	60.88001	356.9198	64.66289
358.8574	68.11185	358.611	60.88027	356.9534	64.6626
358.8996	68.11057	358.6522	60.88344	356.9953	64.66229
358.9412	68.11069	358.6935	60.88222	357.0361	64.66213
358.9822	68.11172	358.7317	60.88176	357.079	64.66192
359.0181	68.1111	358.7762	60.8825	357.1226	64.66142
359.058	68.11231	358.813	60.88035	357.1625	64.66121
359.0984	68.11259	358.8482	60.88228	357.1992	64.66135
359.1389	68.11239	358.8895	60.88542	357.2439	64.66092
359.1819	68.11502	358.9316	60.88499	357.2867	64.66061
359.2225	68.11383	358.9734	60.88701	357.3262	64.65969
359.2661	68.113	359.0163	60.8894	357.369	64.65891
359.3044	68.11334	359.0567	60.88634	357.4092	64.65868
359.3452	68.11407	359.0976	60.88729	357.4496	64.65832
359.3862	68.11663	359.1403	60.88838	357.4924	64.65831
359.4311	68.11852	359.1838	60.88303	357.535	64.65785
359.462	68.1186	359.2239	60.88077	357.5692	64.65719
359.5044	68.11501	359.267	60.87853	357.611	64.65611
359.5466	68.10994	359.298	60.87473	357.6537	64.6558
359.5861	68.10726	359.34	60.87425	357.6948	64.65596
359.629	68.10656	359.3818	60.87581	357.7342	64.65569
359.6696	68.10712	359.425	60.87521	357.7734	64.65576
359.7133	68.10854	359.463	60.87567	357.8143	64.65493
359.7521	68.10937	359.5072	60.87746	357.8579	64.65443
359.7917	68.10856	359.5488	60.87696	357.8987	64.65417
359.834	68.10628	359.5883	60.87841	357.941	64.65419
359.8761	68.10655	359.63	60.88107	357.9803	64.65433
359.9128	68.10585	359.6722	60.87771	358.0225	64.6544
359.9534	68.1048	359.7144	60.87726	358.065	64.65405
359.9945	68.10568	359.746	60.87783	358.106	64.65402
360.034	68.10447	359.786	60.87231	358.148	64.65373
360.0778	68.10168	359.8284	60.86894	358.1804	64.6534
360.1185	68.10052	359.8696	60.87015	358.2225	64.65272
360.1605	68.09943	359.9112	60.87029	358.2632	64.6516
360.2008	68.09981	359.9536	60.87109	358.3052	64.65103
360.2412	68.10098	359.9958	60.87187	358.3496	64.65013
360.2831	68.10185	360.0368	60.87015	358.3882	64.64977
360.3246	68.10232	360.0794	60.86887	358.4282	64.64974
360.3674	68.10074	360.1215	60.86956	358.4717	64.65018
360.407	68.10068	360.1622	60.86844	358.5135	64.65094
360.44	68.10035	360.193	60.86877	358.5566	64.65145
360.4817	68.09846	360.2382	60.87175	358.5973	64.65131
360.5237	68.09682	360.2794	60.87155	358.6381	64.65019
360.5648	68.09575	360.3214	60.87141	358.6813	64.64937
360.6074	68.09411	360.3618	60.87214	358.7148	64.64953
360.6473	68.09411	360.4038	60.86704	358.7562	64.6492
360.6906	68.0954	360.4454	60.86413	358.7947	64.64917
360.7329	68.09519	360.4891	60.86577	358.8367	64.64787

360.7748	68.09424	360.529	60.86305	358.8774	64.6458
360.8162	68.09167	360.5714	60.86188	358.9206	64.64508
360.857	68.09118	360.6103	60.86352	358.9637	64.64469
360.8899	68.09136	360.6446	60.86139	359.0052	64.64464
360.9304	68.09037	360.6874	60.86205	359.0448	64.6451
360.9718	68.08948	360.7276	60.86491	359.0856	64.64603
361.0134	68.08837	360.7696	60.8645	359.1291	64.64707
361.0537	68.08818	360.8108	60.86457	359.1688	64.64776
361.0954	68.08817	360.854	60.86718	359.2111	64.64803
361.137	68.08844	360.894	60.86499	359.2536	64.64732
361.1786	68.0896	360.936	60.86526	359.2936	64.64587
361.2208	68.08885	360.9778	60.86875	359.3306	64.64486
361.264	68.08968	361.0202	60.86773	359.3676	64.64484
361.3039	68.08945	361.062	60.86606	359.4114	64.64396
361.3375	68.08806	361.0933	60.86664	359.4541	64.64337
361.379	68.08681	361.137	60.86502	359.4952	64.64414
361.42	68.08381	361.1792	60.86091	359.5363	64.64359
361.4612	68.08342	361.221	60.86146	359.5789	64.64367
361.5042	68.08429	361.2608	60.86156	359.6204	64.64402
361.5448	68.08383	361.302	60.85978	359.6618	64.64405
361.5869	68.08416	361.345	60.85904	359.704	64.64374
361.6302	68.08326	361.3867	60.8581	359.7444	64.64371
361.6684	68.08156	361.4275	60.85729	359.7872	64.64339
361.7112	68.07979	361.4668	60.85855	359.829	64.64252
361.7502	68.079	361.5068	60.85858	359.8712	64.64231
361.7859	68.0801	361.5441	60.86052	359.9115	64.64128
361.8279	68.07905	361.5848	60.86266	359.943	64.64015
361.8697	68.07795	361.6262	60.85909	359.987	64.63944
361.9111	68.07756	361.6688	60.85701	360.0261	64.63806
361.9494	68.07376	361.7084	60.85607	360.07	64.63761
361.9925	68.07259	361.7504	60.85333	360.1088	64.63786
362.0344	68.07345	361.7922	60.85229	360.1512	64.63821
362.0771	68.07203	361.8342	60.85305	360.1946	64.63885
362.1184	68.07196	361.8762	60.85317	360.2334	64.63838
362.1592	68.0737	361.916	60.85425	360.2743	64.63864
362.199	68.07379	361.9588	60.85621	360.316	64.63791
362.2439	68.07703	361.9933	60.85645	360.3586	64.63707
362.2857	68.07746	362.0338	60.85529	360.4006	64.63753
362.3181	68.07798	362.075	60.85309	360.442	64.63658
362.3598	68.07708	362.1154	60.84873	360.4744	64.63601
362.4012	68.07326	362.159	60.8473	360.5175	64.63626
362.4431	68.07353	362.2015	60.84733	360.5572	64.63585
362.4839	68.07156	362.2444	60.84634	360.6009	64.63578
362.5268	68.06965	362.2846	60.84643	360.6421	64.63573
362.5698	68.06801	362.3269	60.84774	360.6832	64.63473
362.61	68.06564	362.3684	60.8459	360.7248	64.63471
362.653	68.06454	362.4079	60.8422	360.7649	64.63465
362.695	68.06327	362.441	60.84099	360.806	64.63411
362.7354	68.06267	362.4825	60.83794	360.8459	64.63346
362.7688	68.06173	362.523	60.83506	360.8907	64.63264
362.811	68.06126	362.5648	60.83705	360.9331	64.63166
362.8535	68.06116	362.6067	60.83859	360.9731	64.63061
362.8925	68.06023	362.649	60.84135	361.016	64.63002
362.9344	68.06118	362.6908	60.84648	361.0574	64.62882
362.9769	68.06253	362.7332	60.8474	361.0903	64.62872
363.017	68.06257	362.7748	60.84781	361.1314	64.62866
363.0583	68.06399	362.8137	60.84646	361.173	64.62864
363.1012	68.06433	362.8548	60.84068	361.2158	64.62897

363.1406	68.0624	362.889	60.8389	361.2582	64.62794
363.1826	68.05882	362.9309	60.83935	361.2997	64.62735
363.2172	68.05675	362.9739	60.83659	361.3391	64.62704
363.2582	68.05538	363.014	60.83472	361.3798	64.62691
363.3021	68.05346	363.0567	60.83636	361.4237	64.62715
363.3408	68.05357	363.0989	60.83508	361.4628	64.62756
363.384	68.05517	363.1398	60.83455	361.5057	64.62794
363.4241	68.05474	363.1828	60.83755	361.547	64.62728
363.4663	68.05449	363.222	60.8366	361.5902	64.62649
363.5095	68.05465	363.2636	60.83555	361.6311	64.62596
363.5492	68.05511	363.3062	60.83652	361.673	64.62522
363.5905	68.05507	363.3375	60.83458	361.7056	64.62494
363.6322	68.05505	363.3795	60.83319	361.7474	64.62416
363.666	68.05544	363.4239	60.83403	361.7892	64.62347
363.7072	68.05488	363.4635	60.83364	361.83	64.62243
363.7481	68.05488	363.5068	60.83341	361.8711	64.62204
363.7896	68.05264	363.5468	60.8337	361.9142	64.62183
363.8342	68.0501	363.589	60.83223	361.957	64.62133
363.872	68.0479	363.6315	60.83109	361.9967	64.62164
363.9146	68.04513	363.6709	60.83256	362.04	64.62206
363.9568	68.04462	363.7154	60.83566	362.0804	64.62265
363.9952	68.04438	363.7554	60.83631	362.1216	64.6232
364.0382	68.04118	363.791	60.8378	362.1634	64.62286
364.0798	68.03826	363.833	60.8372	362.2035	64.62162
364.1206	68.03677	363.8732	60.83294	362.237	64.621
364.1635	68.03562	363.9134	60.834	362.2759	64.61983
364.1952	68.03636	363.9558	60.83398	362.3198	64.61915
364.2351	68.03711	363.9964	60.83508	362.3612	64.61894
364.2771	68.03694	364.0359	60.84065	362.4026	64.61919
364.3179	68.03639	364.0804	60.84007	362.4447	64.61904
364.3598	68.03643	364.1218	60.8384	362.4862	64.61831
364.4004	68.03771	364.165	60.8399	362.5264	64.61706
364.4444	68.03621	364.204	60.83677	362.5685	64.61576
364.484	68.0344	364.2395	60.83259	362.6106	64.61519
364.5266	68.03279	364.2809	60.83286	362.652	64.61499
364.5666	68.03198	364.32	60.83215	362.6922	64.61477
364.6097	68.03336	364.3636	60.82949	362.7334	64.6147
364.643	68.03344	364.4056	60.8326	362.7758	64.6146
364.6838	68.03368	364.4475	60.83375	362.8178	64.6143
364.7248	68.03449	364.4872	60.83178	362.8506	64.61416
364.7692	68.03229	364.5287	60.83128	362.8906	64.61344
364.8089	68.03228	364.5711	60.828	362.9316	64.61204
364.85	68.03166	364.6108	60.82572	362.9769	64.61173
364.893	68.02901	364.6524	60.82549	363.0156	64.61239
364.9333	68.02705	364.6886	60.82252	363.0579	64.61332
364.9739	68.02653	364.7272	60.82206	363.0991	64.61397
365.0176	68.02663	364.7676	60.82417	363.1412	64.61396
365.0588	68.02598	364.81	60.82545	363.18	64.6129
365.0915	68.02577	364.8513	60.82584	363.2241	64.61202
365.1332	68.02518	364.8948	60.82639	363.2666	64.61158
365.1738	68.02166	364.934	60.82639	363.3091	64.61047
365.2168	68.01851	364.9792	60.82414	363.348	64.60918
365.2578	68.01732	365.0196	60.82453	363.3912	64.60848
365.2978	68.01785	365.0587	60.82569	363.4316	64.6072
365.343	68.01877	365.1006	60.8237	363.4672	64.60688
365.3828	68.01915	365.1338	60.82293	363.507	64.60732
365.4244	68.01921	365.1737	60.82306	363.5498	64.60779
365.4662	68.01571	365.2162	60.81972	363.5904	64.60917

365.5064	68.01427	365.2571	60.82025	363.631	64.6093
365.5418	68.01314	365.299	60.81905	363.6742	64.60882
365.5843	68.01232	365.3412	60.81673	363.715	64.60779
365.6244	68.01207	365.3828	60.81843	363.7578	64.60682
365.6642	68.01184	365.4219	60.81501	363.7966	64.60628
365.7054	68.0101	365.4652	60.8097	363.8397	64.60634
365.7483	68.00833	365.5064	60.81174	363.8803	64.60677
365.7895	68.00869	365.549	60.81254	363.92	64.60582
365.8314	68.00787	365.5817	60.81185	363.9656	64.60549
365.872	68.0082	365.6232	60.81728	363.9956	64.60522
365.9132	68.00947	365.6652	60.81726	364.0362	64.6048
365.9566	68.00814	365.7065	60.8143	364.08	64.60548
365.998	68.00689	365.747	60.814	364.1222	64.60595
366.0387	68.00608	365.79	60.81344	364.1628	64.60629
366.0726	68.00373	365.8307	60.81433	364.2068	64.60555
366.1126	68.0026	365.8728	60.81654	364.2493	64.60421
366.156	68.00201	365.914	60.81695	364.2899	64.60266
366.1976	68.00143	365.9554	60.81596	364.3312	64.60084
366.2383	68.00062	365.998	60.81801	364.3709	64.59886
366.2804	67.99927	366.03	60.81821	364.4145	64.59779
366.3225	67.99872	366.0719	60.81458	364.4557	64.59787
366.3611	67.99639	366.1156	60.81216	364.4945	64.59829
366.4031	67.99667	366.1566	60.80806	364.5376	64.60007
366.444	67.9965	366.1959	60.80429	364.5806	64.5996
366.487	67.99675	366.2387	60.80557	364.6138	64.59846
366.5194	67.99654	366.2779	60.80811	364.6563	64.59617
366.5625	67.99554	366.3201	60.80946	364.6965	64.59364
366.6028	67.99497	366.3614	60.81131	364.7385	64.59314
366.6452	67.99206	366.4014	60.81102	364.7804	64.59346
366.6854	67.98996	366.445	60.8095	364.8233	64.59385
366.7294	67.99064	366.479	60.8078	364.8635	64.59451
366.7698	67.98973	366.5212	60.80298	364.904	64.59375
366.8118	67.98997	366.5618	60.80227	364.9468	64.59402
366.8533	67.98976	366.6018	60.80455	364.9865	64.59636
366.8943	67.98896	366.6415	60.80442	365.029	64.59669
366.9359	67.98854	366.6853	60.80497	365.0716	64.59868
366.97	67.98735	366.7282	60.8061	365.1114	64.59941
367.0109	67.98773	366.768	60.80545	365.153	64.59714
367.0552	67.98836	366.8093	60.80497	365.1933	64.59761
367.0941	67.98804	366.8512	60.80585	365.2275	64.59693
367.135	67.98804	366.8944	60.80452	365.2693	64.59614
367.1766	67.98648	366.9273	60.80138	365.3097	64.59629
367.2166	67.98521	366.97	60.79904	365.3525	64.5949
367.258	67.98455	367.0126	60.79663	365.394	64.59361
367.303	67.98117	367.052	60.7945	365.435	64.59266
367.3436	67.98017	367.0924	60.79509	365.477	64.59219
367.3848	67.97822	367.1357	60.79588	365.5182	64.5927
367.4165	67.97543	367.1781	60.79526	365.5603	64.59264
367.4597	67.9758	367.218	60.79545	365.6017	64.59111
367.4999	67.97591	367.2587	60.79296	365.6438	64.58968
367.5408	67.9756	367.3015	60.79145	365.6834	64.58756
367.5845	67.97597	367.3442	60.79137	365.7253	64.58702
367.624	67.97604	367.3787	60.7911	365.7584	64.58693
367.6683	67.9765	367.4183	60.78996	365.8008	64.58675
367.7096	67.97499	367.4602	60.79087	365.8418	64.58693
367.7515	67.97312	367.5006	60.79095	365.8855	64.58623
367.7914	67.97363	367.5441	60.79051	365.927	64.58595
367.835	67.9718	367.5856	60.79042	365.9673	64.58665

367.8746	67.96845	367.6261	60.78916	366.009	64.58777
367.9162	67.96817	367.6688	60.78951	366.0518	64.5878
367.9489	67.96736	367.711	60.78979	366.0954	64.58807
367.9907	67.9667	367.7511	60.79306	366.1346	64.58831
368.0324	67.96832	367.7935	60.79468	366.1738	64.58704
368.0743	67.96821	367.8265	60.79582	366.2198	64.58625
368.1152	67.96595	367.8677	60.79551	366.256	64.58568
368.159	67.96416	367.9101	60.79284	366.2983	64.58665
368.1992	67.96361	367.9515	60.79468	366.3416	64.58818
368.2412	67.96461	367.9932	60.79402	366.3738	64.58815
368.2804	67.96359	368.0355	60.79294	366.4157	64.58784
368.3231	67.96384	368.077	60.79428	366.459	64.58605
368.365	67.96372	368.1192	60.79117	366.4989	64.58374
368.3965	67.96228	368.1594	60.78897	366.5417	64.58356
368.44	67.9578	368.1997	60.78882	366.5831	64.58398
368.4812	67.95471	368.24	60.78584	366.623	64.58361
368.5232	67.95284	368.2772	60.78412	366.6658	64.58257
368.5634	67.95141	368.3169	60.78398	366.7047	64.58041
368.6053	67.95269	368.3592	60.78335	366.748	64.5789
368.6461	67.95369	368.3998	60.78296	366.7906	64.57809
368.6884	67.95386	368.4384	60.78521	366.8303	64.57819
368.7311	67.95266	368.4807	60.78395	366.8708	64.57822
368.7733	67.95139	368.5217	60.78279	366.9112	64.5775
368.8128	67.95152	368.5628	60.78475	366.954	64.57697
368.8475	67.95093	368.607	60.78487	366.988	64.57699
368.8896	67.94832	368.6498	60.78563	367.0275	64.57756
368.9289	67.94782	368.6921	60.78785	367.0678	64.5778
368.9708	67.94817	368.7225	60.78633	367.108	64.57768
369.0125	67.94617	368.7667	60.78381	367.1497	64.57756
369.053	67.94421	368.8062	60.78222	367.191	64.57763
369.0941	67.94534	368.8483	60.77943	367.2325	64.57775
369.136	67.94447	368.8902	60.77681	367.2738	64.5769
369.1776	67.94286	368.932	60.77664	367.3128	64.57549
369.2191	67.94357	368.975	60.77865	367.3576	64.57434
369.2594	67.94306	369.0157	60.77992	367.4016	64.57444
369.2948	67.94117	369.0575	60.78051	367.4415	64.5745
369.3364	67.94037	369.0982	60.77698	367.4854	64.57474
369.3789	67.94187	369.1396	60.77531	367.5156	64.57528
369.4196	67.93989	369.1731	60.77588	367.558	64.57549
369.4582	67.93832	369.2135	60.77558	367.5992	64.57563
369.502	67.93678	369.2551	60.77675	367.639	64.57487
369.5432	67.93395	369.2962	60.77938	367.682	64.57483
369.5874	67.93245	369.3389	60.77882	367.7234	64.57478
369.6262	67.93125	369.3806	60.77781	367.764	64.57452
369.6665	67.93083	369.4193	60.77748	367.8069	64.57383
369.7089	67.92921	369.4622	60.77422	367.8455	64.57226
369.7502	67.92747	369.504	60.77177	367.887	64.56992
369.7927	67.92623	369.5447	60.77132	367.93	64.5687
369.825	67.92639	369.5862	60.77178	367.9712	64.56789
369.8682	67.92685	369.6187	60.77211	368.0144	64.56727
369.9089	67.92718	369.661	60.77278	368.0538	64.56647
369.9468	67.92846	369.7022	60.77213	368.0975	64.56597
369.99	67.92825	369.7475	60.77122	368.1281	64.56606
370.0316	67.92694	369.788	60.76925	368.1684	64.56636
370.072	67.92602	369.8286	60.76693	368.2114	64.56758
370.1156	67.92485	369.8702	60.76673	368.2534	64.56854
370.1572	67.92248	369.9114	60.76919	368.2963	64.56859
370.1988	67.91989	369.9539	60.76883	368.3389	64.56775

370.2398	67.92028	369.9954	60.76873	368.3795	64.56625
370.273	67.91956	370.0345	60.76887	368.4218	64.56637
370.3152	67.91777	370.0688	60.7663	368.4639	64.56732
370.355	67.91869	370.1082	60.76461	368.505	64.56833
370.3985	67.9195	370.1512	60.76383	368.544	64.56876
370.4399	67.91803	370.1928	60.76156	368.5854	64.56711
370.4828	67.91577	370.237	60.7613	368.627	64.56575
370.5234	67.91444	370.2766	60.76319	368.6693	64.56496
370.5666	67.91359	370.3212	60.76323	368.711	64.56451
370.6078	67.9121	370.3612	60.76372	368.7434	64.5647
370.6494	67.91304	370.4006	60.76222	368.7829	64.5667
370.6907	67.91416	370.4426	60.76038	368.8279	64.56887
370.7231	67.91273	370.4837	60.75906	368.8674	64.56959
370.7652	67.9105	370.5168	60.76107	368.9066	64.56917
370.808	67.90897	370.5585	60.76219	368.9476	64.56683
370.852	67.90728	370.6004	60.76055	368.9907	64.56383
370.8928	67.90537	370.6438	60.76072	369.0332	64.56288
370.9328	67.90362	370.6848	60.75884	369.0739	64.56199
370.9734	67.90295	370.7264	60.7581	369.116	64.56152
371.0152	67.9002	370.7676	60.75871	369.1602	64.56112
371.057	67.89835	370.8105	60.75974	369.201	64.56145
371.0959	67.89957	370.8544	60.76049	369.2422	64.56267
371.1394	67.90163	370.8956	60.75899	369.2748	64.56241
371.1719	67.90307	370.9378	60.75687	369.3172	64.56267
371.2125	67.90121	370.969	60.75437	369.358	64.56198
371.2554	67.89929	371.01	60.7494	369.3987	64.56039
371.2959	67.89674	371.0512	60.7468	369.4434	64.55927
371.3364	67.8931	371.092	60.74706	369.4846	64.5574
371.378	67.89378	371.1332	60.74849	369.5248	64.55655
371.417	67.89331	371.1731	60.75314	369.5688	64.55575
371.4602	67.89054	371.2159	60.75648	369.6128	64.55646
371.5046	67.88821	371.2563	60.75796	369.6533	64.55671
371.545	67.88713	371.2959	60.76015	369.6958	64.55692
371.5869	67.88684	371.3394	60.76101	369.7384	64.55566
371.6267	67.88728	371.3805	60.75884	369.7776	64.55367
371.6702	67.8894	371.412	60.76026	369.8194	64.5529
371.7038	67.88913	371.4557	60.76084	369.8614	64.55314
371.7441	67.88921	371.4952	60.75735	369.8938	64.55329
371.787	67.88826	371.5399	60.76031	369.9346	64.55485
371.8255	67.88866	371.5798	60.76178	369.9777	64.5551
371.8663	67.88887	371.6208	60.76187	370.0198	64.55478
371.9093	67.88746	371.6636	60.76062	370.061	64.55514
371.9521	67.88787	371.7052	60.75733	370.1027	64.55401
371.9914	67.88897	371.7469	60.75474	370.1455	64.55374
372.0344	67.8889	371.7881	60.75299	370.1841	64.55159
372.0746	67.88713	371.8274	60.75617	370.229	64.54889
372.1157	67.88691	371.861	60.7559	370.2698	64.54821
372.1514	67.88591	371.9028	60.7537	370.3125	64.54857
372.1894	67.88388	371.9481	60.75201	370.3545	64.54856
372.2316	67.88411	371.9891	60.74779	370.3977	64.5501
372.2732	67.88398	372.028	60.74446	370.435	64.55062
372.3156	67.88368	372.0684	60.74446	370.477	64.54965
372.3569	67.88133	372.113	60.74171	370.511	64.55112
372.3983	67.88081	372.1521	60.74101	370.5528	64.55006
372.4406	67.88123	372.196	60.74464	370.5942	64.54726
372.4816	67.88044	372.2363	60.74389	370.6356	64.54612
372.5241	67.88194	372.2766	60.74336	370.6774	64.54376
372.563	67.88271	372.311	60.74573	370.7191	64.54348

372.5972	67.88202	372.3509	60.74418	370.759	64.54341
372.6385	67.87966	372.3919	60.7423	370.805	64.54305
372.6796	67.87828	372.4353	60.74366	370.8442	64.54351
372.7208	67.87765	372.4802	60.74144	370.887	64.54416
372.7631	67.87567	372.52	60.73724	370.9288	64.54537
372.8046	67.87501	372.5606	60.73684	370.9708	64.5449
372.848	67.87386	372.6022	60.7341	371.0119	64.54328
372.886	67.87198	372.6421	60.73103	371.0434	64.54218
372.9246	67.87125	372.6831	60.73095	371.0835	64.54185
372.968	67.87043	372.725	60.72775	371.127	64.54419
373.0098	67.86956	372.7578	60.72766	371.168	64.54523
373.0434	67.86835	372.7988	60.73181	371.2094	64.54716
373.0856	67.86618	372.839	60.73475	371.2497	64.54718
373.126	67.86324	372.8832	60.73588	371.2905	64.54501
373.169	67.86247	372.9265	60.73886	371.3334	64.54512
373.2111	67.86195	372.9661	60.73574	371.3728	64.54433
373.2537	67.86131	373.0072	60.73167	371.4164	64.54478
373.2928	67.86174	373.0488	60.73252	371.458	64.54369
373.3366	67.86035	373.0904	60.73049	371.498	64.54305
373.3778	67.85848	373.13	60.7308	371.5392	64.54213
373.4197	67.85891	373.172	60.73483	371.5826	64.53929
373.4594	67.85874	373.2063	60.73472	371.6237	64.53891
373.503	67.85728	373.2483	60.73427	371.6559	64.53918
373.5444	67.85745	373.2894	60.73578	371.6986	64.53857
373.5756	67.85674	373.331	60.73368	371.7398	64.53823
373.6178	67.8554	373.3756	60.7303	371.7804	64.53734
373.6602	67.85337	373.4163	60.73017	371.8218	64.53631
373.7	67.85393	373.4578	60.72869	371.8641	64.53612
373.7426	67.85361	373.4996	60.72552	371.9048	64.53714
373.7825	67.85077	373.5433	60.72811	371.9472	64.53733
373.8261	67.84928	373.582	60.7292	371.987	64.53676
373.8652	67.8487	373.6238	60.72632	372.0292	64.53499
373.9072	67.84709	373.6583	60.72551	372.0708	64.53323
373.9468	67.84657	373.6989	60.72254	372.1128	64.53272
373.9932	67.84641	373.74	60.71828	372.1546	64.53171
374.0253	67.84393	373.7822	60.71678	372.1984	64.53121
374.0666	67.84211	373.8244	60.71739	372.2378	64.53142
374.1064	67.84189	373.8684	60.71862	372.2713	64.53142
374.1494	67.84198	373.9057	60.72006	372.3129	64.53331
374.1894	67.84141	373.9504	60.72073	372.3552	64.53412
374.2304	67.84055	373.9919	60.72127	372.395	64.5335
374.2734	67.83793	374.0327	60.72024	372.4369	64.53489
374.3148	67.83747	374.0758	60.71658	372.4795	64.53376
374.3561	67.83759	374.1064	60.71452	372.5203	64.53273
374.4005	67.83646	374.1474	60.71385	372.5609	64.53352
374.4403	67.8368	374.1906	60.71521	372.604	64.53219
374.4747	67.8362	374.2315	60.7157	372.6455	64.53091
374.5154	67.83535	374.2736	60.71805	372.6868	64.53081
374.5582	67.83465	374.3157	60.7216	372.7296	64.52933
374.5983	67.83506	374.3578	60.72173	372.7704	64.52941
374.64	67.83403	374.3978	60.72245	372.8028	64.52971
374.6828	67.83379	374.4396	60.72189	372.845	64.52884
374.7234	67.83396	374.4822	60.71833	372.8862	64.52824
374.7634	67.83263	374.5253	60.7163	372.9277	64.52672
374.804	67.8319	374.5547	60.71537	372.9695	64.52514
374.8488	67.83158	374.5956	60.71468	373.0112	64.52444
374.8916	67.83098	374.6365	60.71753	373.0537	64.52475
374.9231	67.82942	374.6814	60.71549	373.0941	64.52571

374.967	67.8285	374.7217	60.71354	373.1352	64.52573
375.007	67.82764	374.762	60.71355	373.1765	64.52521
375.0498	67.82658	374.8028	60.70885	373.219	64.52427
375.091	67.82658	374.845	60.70628	373.2597	64.52194
375.1326	67.82485	374.8858	60.70668	373.3028	64.52077
375.1721	67.82434	374.9288	60.70478	373.3425	64.52053
375.2136	67.82253	374.9716	60.70182	373.3852	64.52072
375.2579	67.81943	375.0043	60.70076	373.418	64.52041
375.2986	67.81918	375.0437	60.70133	373.4575	64.52002
375.3406	67.81896	375.0859	60.70118	373.4975	64.51998
375.381	67.81575	375.1267	60.70214	373.54	64.52055
375.4254	67.81494	375.1676	60.7041	373.5812	64.52131
375.4574	67.81562	375.2114	60.70534	373.6218	64.52209
375.4982	67.8149	375.2541	60.7036	373.6653	64.52097
375.5391	67.81344	375.295	60.7037	373.705	64.51816
375.579	67.8137	375.3369	60.70197	373.7488	64.51544
375.6224	67.81358	375.3792	60.6997	373.7882	64.51414
375.6617	67.81014	375.4187	60.70018	373.8285	64.51559
375.7028	67.80803	375.4521	60.69938	373.8708	64.51624
375.7472	67.80805	375.4976	60.69963	373.9124	64.51742
375.7864	67.80695	375.5369	60.70081	373.9543	64.51699
375.8289	67.80452	375.5779	60.69783	373.9967	64.51613
375.8692	67.80398	375.6204	60.69563	374.031	64.51587
375.8996	67.80369	375.6622	60.69432	374.072	64.51533
375.9413	67.80133	375.7016	60.69306	374.1126	64.5161
375.9844	67.80314	375.743	60.69379	374.1548	64.51664
376.025	67.80482	375.7844	60.69676	374.195	64.51597
376.065	67.80416	375.8266	60.69826	374.2346	64.51565
376.1094	67.80401	375.8684	60.69575	374.2781	64.51449
376.1512	67.80123	375.9008	60.69587	374.3204	64.51262
376.1906	67.7991	375.9412	60.69364	374.3619	64.5133
376.2304	67.79745	375.982	60.69083	374.4053	64.51309
376.274	67.79707	376.0234	60.691	374.4447	64.51216
376.3189	67.79623	376.0671	60.69015	374.4865	64.51246
376.3515	67.79552	376.1084	60.68957	374.5265	64.51142
376.3916	67.79423	376.1518	60.69182	374.56	64.51126
376.4344	67.79075	376.1934	60.69156	374.6028	64.51195
376.4756	67.78605	376.2332	60.6927	374.6438	64.51027
376.5156	67.78339	376.2762	60.693	374.6857	64.50857
376.5572	67.78208	376.3168	60.69107	374.73	64.50733
376.5996	67.78202	376.349	60.68939	374.7694	64.50667
376.64	67.78483	376.3914	60.68948	374.8142	64.50767
376.6818	67.78534	376.4312	60.68942	374.8562	64.5085
376.724	67.78425	376.4738	60.6873	374.8971	64.50849
376.7645	67.78299	376.5131	60.68767	374.9362	64.50784
376.7978	67.77888	376.5562	60.68732	374.9771	64.50744
376.8383	67.77421	376.5974	60.68617	375.0184	64.50688
376.8816	67.7728	376.6384	60.68568	375.0615	64.50706
376.923	67.7746	376.6815	60.6858	375.1022	64.50664
376.9664	67.77707	376.7219	60.68456	375.1443	64.50541
377.007	67.77778	376.7607	60.68557	375.1777	64.5051
377.0487	67.77642	376.7956	60.68779	375.2187	64.5037
377.0898	67.77134	376.8367	60.68892	375.2588	64.50281
377.13	67.769	376.8795	60.69094	375.3004	64.50273
377.1722	67.76833	376.9216	60.69054	375.3441	64.50241
377.212	67.76954	376.9633	60.68838	375.3871	64.50308
377.2535	67.76904	377.0052	60.68747	375.4262	64.50279
377.2944	67.76645	377.0461	60.68577	375.4664	64.50224

377.3295	67.7661	377.088	60.6851	375.5096	64.50175
377.3702	67.76477	377.1292	60.68471	375.552	64.50105
377.4142	67.76338	377.1736	60.68255	375.5925	64.50066
377.4535	67.76341	377.2112	60.68039	375.6348	64.50273
377.496	67.76515	377.2459	60.67833	375.6778	64.50269
377.5387	67.766	377.2868	60.67739	375.7174	64.50111
377.5792	67.76766	377.3291	60.67653	375.7598	64.50064
377.6211	67.76887	377.3712	60.67665	375.7954	64.49987
377.6636	67.76891	377.4106	60.67771	375.8332	64.49906
377.7039	67.7686	377.4512	60.67976	375.876	64.49948
377.7439	67.76773	377.4956	60.68304	375.916	64.49877
377.7786	67.76847	377.5372	60.68439	375.9559	64.4976
377.8188	67.76664	377.5791	60.68301	376.0008	64.49574
377.8614	67.76462	377.6179	60.68171	376.0394	64.49477
377.903	67.76227	377.6616	60.67672	376.0798	64.49451
377.9456	67.75991	377.6937	60.67326	376.1209	64.49404
377.9872	67.75839	377.735	60.67247	376.1655	64.49419
378.0275	67.75694	377.774	60.67275	376.2056	64.49451
378.0686	67.75615	377.8187	60.67309	376.2498	64.49378
378.1076	67.75297	377.8625	60.6753	376.289	64.49285
378.1502	67.75064	377.9041	60.67453	376.3215	64.49252
378.1928	67.74917	377.9468	60.67104	376.3634	64.49224
378.2246	67.74718	377.9874	60.67009	376.4059	64.49353
378.264	67.74643	378.0288	60.66694	376.445	64.49446
378.3104	67.74738	378.0697	60.66345	376.4902	64.49344
378.35	67.74784	378.1115	60.6603	376.5306	64.49269
378.3922	67.74675	378.1452	60.65697	376.5746	64.49165
378.4332	67.74491	378.1866	60.65416	376.614	64.49026
378.4736	67.74532	378.2285	60.65263	376.6565	64.49225
378.5152	67.74532	378.2708	60.65332	376.6976	64.49384
378.5578	67.74534	378.311	60.65464	376.7387	64.49357
378.5996	67.74556	378.3533	60.65469	376.783	64.49326
378.6422	67.74396	378.3968	60.65338	376.8218	64.49128
378.6752	67.74175	378.4378	60.65607	376.8665	64.49026
378.7174	67.74034	378.4818	60.65825	376.9058	64.49067
378.7599	67.74004	378.5206	60.66077	376.943	64.49029
378.8016	67.73954	378.5619	60.66487	376.9846	64.48998
378.8413	67.73797	378.5961	60.66523	377.0233	64.48947
378.884	67.73569	378.6356	60.66343	377.0658	64.48844
378.9258	67.73621	378.679	60.66161	377.1067	64.48871
378.9688	67.73523	378.7198	60.65974	377.1474	64.488
379.0089	67.73451	378.7612	60.65582	377.19	64.4867
379.05	67.73666	378.8017	60.65276	377.233	64.48597
379.0934	67.73437	378.8436	60.65124	377.276	64.4853
379.1336	67.73247	378.8872	60.65184	377.3195	64.48557
379.1761	67.73257	378.9312	60.65392	377.3576	64.48739
379.2113	67.73086	378.9695	60.65441	377.399	64.48684
379.2516	67.73013	379.0117	60.65566	377.4406	64.48756
379.2928	67.73101	379.0434	60.65612	377.4826	64.48784
379.3336	67.73104	379.0861	60.65397	377.5256	64.48533
379.3758	67.72869	379.1286	60.65473	377.5586	64.48413
379.4178	67.7257	379.1684	60.65455	377.5989	64.4821
379.4597	67.72436	379.2094	60.65398	377.6394	64.48188
379.4982	67.72292	379.2523	60.65479	377.6813	64.48165
379.541	67.72221	379.2945	60.65481	377.7228	64.48121
379.582	67.72428	379.3351	60.65572	377.765	64.48135
379.6216	67.7256	379.3792	60.65537	377.805	64.47971
379.6553	67.72502	379.4198	60.65464	377.8452	64.47944

379.696	67.72332	379.4612	60.65727	377.886	64.47861
379.739	67.72104	379.4974	60.65716	377.9285	64.47766
379.7811	67.71746	379.5359	60.6566	377.9688	64.47815
379.8228	67.71409	379.5758	60.65646	378.0104	64.47714
379.8637	67.71395	379.6188	60.6533	378.0516	64.47779
379.9072	67.7162	379.6617	60.65107	378.0854	64.47804
379.9469	67.71629	379.7016	60.64946	378.1283	64.47593
379.986	67.71567	379.7438	60.6472	378.1692	64.47568
380.03	67.71555	379.7872	60.6462	378.2095	64.47423
380.0715	67.71334	379.8273	60.64571	378.2502	64.47235
380.1028	67.71011	379.8665	60.64559	378.2916	64.47203
380.1456	67.70722	379.9078	60.64753	378.3322	64.47146
380.184	67.70593	379.943	60.64806	378.3754	64.47197
380.2263	67.70528	379.9816	60.64651	378.4158	64.47287
380.268	67.70723	380.0231	60.64685	378.4596	64.47268
380.3104	67.70906	380.0631	60.64842	378.5012	64.47186
380.3518	67.70836	380.1037	60.64822	378.5424	64.47104
380.3925	67.70495	380.1462	60.64797	378.5856	64.46959
380.436	67.70272	380.188	60.64709	378.6287	64.46954
380.4759	67.70265	380.2282	60.64273	378.6676	64.46985
380.5153	67.70132	380.2705	60.63949	378.7021	64.4706
380.5486	67.7026	380.3124	60.63789	378.7436	64.47199
380.5916	67.70333	380.3508	60.63662	378.7846	64.47181
380.631	67.70108	380.3852	60.63746	378.826	64.47102
380.6729	67.69706	380.428	60.64006	378.8662	64.47012
380.7146	67.69709	380.4662	60.64086	378.908	64.46785
380.7572	67.6955	380.5102	60.64024	378.9504	64.46749
380.7997	67.69375	380.5479	60.63927	378.9929	64.46634
380.8406	67.69513	380.5906	60.63523	379.0345	64.46544
380.8809	67.69447	380.6348	60.63257	379.0731	64.46554
380.9233	67.69233	380.6763	60.63229	379.1145	64.46564
380.9646	67.69133	380.719	60.63128	379.1555	64.46667
381.007	67.69058	380.7605	60.63316	379.1987	64.46601
381.0494	67.68982	380.8035	60.63594	379.2391	64.46542
381.0816	67.69028	380.8372	60.63507	379.2817	64.46495
381.1244	67.68989	380.8796	60.63196	379.3158	64.46336
381.1658	67.68869	380.9228	60.62906	379.3548	64.46229
381.2055	67.68704	380.9632	60.62742	379.3974	64.46234
381.2485	67.68507	381.0036	60.62992	379.4384	64.46182
381.2898	67.68352	381.0421	60.63299	379.482	64.46163
381.3324	67.6831	381.0828	60.63506	379.5248	64.46212
381.3721	67.68213	381.1273	60.63487	379.5667	64.46214
381.4106	67.68077	381.168	60.63373	379.6076	64.46112
381.4544	67.68187	381.21	60.63249	379.648	64.46095
381.4976	67.68191	381.2509	60.63322	379.6898	64.4609
381.53	67.68058	381.2845	60.63443	379.7298	64.45937
381.5719	67.68028	381.326	60.63171	379.7728	64.4586
381.6122	67.67895	381.3683	60.62953	379.814	64.45648
381.6561	67.67691	381.4088	60.63161	379.8466	64.45551
381.6946	67.67602	381.4505	60.63067	379.8886	64.45678
381.7366	67.67392	381.4937	60.6308	379.9316	64.45752
381.7795	67.67035	381.5344	60.63042	379.9723	64.45905
381.821	67.67001	381.5761	60.62614	380.0141	64.46041
381.8611	67.66793	381.6192	60.62308	380.0552	64.45983
381.9036	67.66605	381.6592	60.62247	380.0954	64.46018
381.9441	67.6668	381.6998	60.6239	380.1384	64.45905
381.9777	67.66454	381.7333	60.62395	380.1778	64.45781
382.0194	67.66398	381.7735	60.6252	380.2194	64.45744

382.0585	67.66521	381.815	60.62655	380.2604	64.45517
382.1014	67.66296	381.8564	60.62637	380.303	64.45425
382.142	67.66126	381.8975	60.62677	380.344	64.45425
382.1856	67.6604	381.9409	60.62506	380.3859	64.45276
382.2246	67.65858	381.9816	60.62204	380.428	64.45195
382.2656	67.65763	382.0236	60.62061	380.4592	64.45263
382.3055	67.65985	382.0676	60.62027	380.5	64.45156
382.3467	67.66011	382.1089	60.61787	380.544	64.45133
382.3906	67.65831	382.15	60.61998	380.5848	64.45267
382.4235	67.65678	382.1826	60.62228	380.6306	64.45168
382.4657	67.6522	382.2252	60.61957	380.67	64.45102
382.507	67.64844	382.2694	60.61855	380.7122	64.44884
382.5514	67.64584	382.3075	60.61729	380.7516	64.44767
382.5893	67.64708	382.3474	60.61395	380.7934	64.44796
382.6305	67.64926	382.3886	60.6141	380.835	64.447
382.6734	67.64823	382.4299	60.6168	380.8786	64.44781
382.714	67.64828	382.4722	60.61739	380.9194	64.44797
382.7555	67.64644	382.5152	60.61659	380.9628	64.44776
382.7978	67.64366	382.5591	60.61553	381.0038	64.44699
382.8388	67.6437	382.6002	60.61275	381.0446	64.44559
382.8812	67.64328	382.6316	60.61171	381.0761	64.44443
382.9204	67.64203	382.6748	60.60907	381.1172	64.44229
382.952	67.6405	382.7159	60.6068	381.16	64.4432
382.996	67.64057	382.7556	60.60583	381.2025	64.44381
383.0375	67.63978	382.7989	60.60371	381.2414	64.44333
383.0794	67.63866	382.8406	60.60345	381.2836	64.44442
383.1215	67.63896	382.8828	60.60295	381.323	64.44373
383.1626	67.63735	382.9244	60.60323	381.3668	64.44366
383.2032	67.6347	382.9653	60.60243	381.4078	64.44325
383.2456	67.63275	383.0091	60.60381	381.4502	64.44277
383.2872	67.63036	383.0478	60.605	381.4906	64.44227
383.3276	67.62763	383.0806	60.60642	381.5316	64.44208
383.3719	67.6245	383.1226	60.60848	381.5727	64.44194
383.407	67.62122	383.1645	60.60788	381.606	64.4404
383.4466	67.62172	383.2058	60.60785	381.6477	64.43903
383.486	67.62075	383.2478	60.60472	381.6887	64.43842
383.5302	67.61709	383.2888	60.60283	381.7302	64.43992
383.5697	67.61922	383.3278	60.60255	381.7708	64.44104
383.6114	67.61951	383.3696	60.60288	381.8165	64.44126
383.6521	67.61861	383.4136	60.60412	381.8544	64.44039
383.6941	67.62422	383.4552	60.60449	381.8944	64.43755
383.7352	67.62768	383.4963	60.60432	381.9372	64.43592
383.7762	67.62737	383.529	60.60316	381.9786	64.43561
383.8163	67.6287	383.5705	60.6014	382.0219	64.43552
383.8523	67.62902	383.613	60.59856	382.061	64.43473
383.8914	67.62817	383.6521	60.59859	382.1047	64.43532
383.9314	67.62771	383.6965	60.59874	382.1465	64.43567
383.9752	67.62514	383.7339	60.59785	382.1877	64.43503
384.0184	67.62242	383.7763	60.6	382.2205	64.43571
384.0581	67.61845	383.8182	60.59936	382.2651	64.43446
384.1018	67.61442	383.8616	60.59729	382.3083	64.4325
384.143	67.6145	383.9017	60.59626	382.3494	64.43138
384.1846	67.61399	383.9456	60.59313	382.3885	64.43057
384.2263	67.61194	383.9765	60.59207	382.4303	64.43084
384.2687	67.61296	384.0178	60.59407	382.472	64.43124
384.3028	67.61149	384.059	60.5962	382.5161	64.43138
384.343	67.6079	384.1005	60.59689	382.5574	64.43278
384.3836	67.60748	384.1428	60.59738	382.5991	64.43177

384.4239	67.60769	384.185	60.59536	382.6418	64.43091
384.4674	67.60657	384.2244	60.59166	382.6824	64.43047
384.508	67.60712	384.265	60.58967	382.7234	64.42735
384.5478	67.60882	384.3094	60.58829	382.7639	64.42654
384.591	67.60946	384.3516	60.58481	382.8065	64.4262
384.6327	67.60959	384.3911	60.58555	382.8371	64.4262
384.6743	67.60892	384.4234	60.58798	382.8815	64.42674
384.7169	67.60731	384.4634	60.5873	382.9246	64.42787
384.7585	67.60445	384.5038	60.58793	382.9648	64.42653
384.7988	67.60059	384.5481	60.5887	383.0052	64.42374
384.8341	67.60018	384.5872	60.58778	383.0492	64.42192
384.8722	67.59916	384.6284	60.58616	383.0871	64.41964
384.9162	67.59607	384.6713	60.58634	383.1319	64.42091
384.9568	67.59566	384.7124	60.58472	383.1723	64.42326
384.9992	67.59535	384.7541	60.58447	383.2149	64.42478
385.0396	67.59464	384.7962	60.5849	383.2562	64.42557
385.0829	67.59459	384.839	60.58394	383.297	64.42395
385.1248	67.59415	384.8698	60.58561	383.3406	64.42315
385.1665	67.59297	384.91	60.58557	383.3724	64.42302
385.207	67.59058	384.9527	60.58383	383.4123	64.42221
385.2456	67.58869	384.9926	60.58389	383.456	64.42158
385.2812	67.58852	385.0358	60.58461	383.4965	64.41982
385.322	67.58725	385.0806	60.58171	383.5393	64.41589
385.3652	67.58506	385.1201	60.58089	383.5804	64.41476
385.4022	67.58409	385.1622	60.58061	383.6208	64.41531
385.4469	67.5822	385.2047	60.57678	383.6634	64.41648
385.4876	67.57992	385.2468	60.57563	383.7045	64.41835
385.5269	67.57982	385.2859	60.57428	383.7476	64.4191
385.5708	67.57964	385.3199	60.57149	383.7865	64.41842
385.6109	67.58027	385.3616	60.57079	383.8283	64.41765
385.6536	67.57912	385.4033	60.57067	383.8694	64.41775
385.693	67.57729	385.446	60.56905	383.9102	64.4174
385.7244	67.57399	385.4878	60.56956	383.9534	64.4166
385.77	67.56955	385.5308	60.56987	383.9884	64.41601
385.8122	67.56753	385.5719	60.5691	384.029	64.41501
385.8517	67.56501	385.6106	60.5691	384.0669	64.41388
385.893	67.56607	385.6531	60.5683	384.1091	64.41366
385.9353	67.56418	385.6941	60.56779	384.1501	64.41282
385.977	67.56369	385.7368	60.5666	384.192	64.41238
386.0178	67.56596	385.7729	60.56929	384.2334	64.4123
386.0586	67.56462	385.8136	60.57157	384.278	64.41142
386.0984	67.56495	385.8543	60.56877	384.3186	64.41045
386.143	67.56393	385.8964	60.56824	384.3592	64.4102
386.1737	67.56252	385.9364	60.56641	384.4019	64.41015
386.2176	67.56013	385.9792	60.56451	384.4407	64.41083
386.2563	67.5594	386.0225	60.56504	384.4865	64.41041
386.2985	67.55865	386.0612	60.56646	384.5269	64.40913
386.3398	67.55773	386.102	60.56599	384.5683	64.40751
386.3786	67.55688	386.1453	60.56418	384.5998	64.4069
386.4212	67.55707	386.1872	60.56253	384.6411	64.40771
386.4626	67.55753	386.2198	60.55945	384.682	64.40702
386.5034	67.55473	386.2628	60.55731	384.7241	64.40797
386.5468	67.55147	386.3044	60.55627	384.7666	64.40684
386.5847	67.54838	386.348	60.55511	384.8094	64.40448
386.6262	67.54601	386.3886	60.55567	384.8507	64.40349
386.6694	67.5449	386.4309	60.55606	384.889	64.40043
386.7033	67.54678	386.4685	60.55496	384.9317	64.39869
386.7427	67.5484	386.5109	60.55682	384.9736	64.39973

386.7847	67.54884	386.551	60.55796	385.0147	64.40126
386.8276	67.54757	386.5938	60.5564	385.0566	64.4036
386.8679	67.54623	386.6351	60.55651	385.0983	64.40384
386.9082	67.54337	386.6676	60.55449	385.1309	64.40303
386.9514	67.54178	386.7098	60.5511	385.1714	64.402
386.992	67.54022	386.7515	60.55082	385.2128	64.40036
387.0354	67.5389	386.795	60.55111	385.2561	64.40003
387.0756	67.53795	386.8365	60.55284	385.2956	64.39936
387.1178	67.53793	386.878	60.55679	385.3383	64.39894
387.1494	67.53876	386.9181	60.55728	385.3787	64.39878
387.1914	67.538	386.9585	60.55521	385.4205	64.39882
387.2336	67.53825	387.0015	60.55321	385.4622	64.39964
387.2746	67.53671	387.0432	60.54997	385.5046	64.3991
387.3156	67.53447	387.0844	60.55005	385.5438	64.39882
387.3595	67.53199	387.119	60.55196	385.5861	64.39781
387.4	67.53023	387.1594	60.55365	385.6284	64.39596
387.441	67.53095	387.2027	60.55382	385.6696	64.39518
387.4847	67.53014	387.2442	60.55206	385.7102	64.39377
387.525	67.52873	387.2878	60.54799	385.7434	64.39295
387.5632	67.52761	387.3262	60.54494	385.7866	64.39359
387.5986	67.52597	387.3691	60.54501	385.8281	64.39363
387.6391	67.52135	387.4106	60.54455	385.8696	64.39401
387.6781	67.5202	387.454	60.54471	385.9106	64.39469
387.7246	67.52014	387.4933	60.54567	385.9512	64.39423
387.7646	67.51873	387.5355	60.54252	385.9936	64.39338
387.8075	67.52102	387.5676	60.5424	386.033	64.39114
387.8485	67.52262	387.6105	60.54438	386.0768	64.38959
387.89	67.52226	387.6534	60.54424	386.1162	64.38835
387.9297	67.52136	387.6943	60.54392	386.1596	64.38815
387.9722	67.52051	387.7358	60.54344	386.2014	64.38929
388.0146	67.51959	387.779	60.54499	386.243	64.3897
388.0472	67.51648	387.8194	60.54421	386.2829	64.38983
388.0894	67.5135	387.8595	60.54427	386.3256	64.38904
388.1305	67.51178	387.9011	60.54235	386.3597	64.38853
388.173	67.51006	387.9452	60.54136	386.4008	64.38798
388.2149	67.50988	387.9852	60.54188	386.442	64.387
388.2578	67.50884	388.02	60.53978	386.4836	64.38544
388.2976	67.50634	388.0612	60.53924	386.5246	64.384
388.3402	67.50434	388.1019	60.53635	386.5678	64.38307
388.382	67.50186	388.1435	60.53182	386.6085	64.38385
388.4233	67.49993	388.184	60.53131	386.6512	64.38424
388.4628	67.50112	388.2264	60.5316	386.6962	64.38463
388.5084	67.5009	388.2686	60.53104	386.735	64.38517
388.5472	67.50159	388.311	60.53227	386.7754	64.38405
388.5798	67.50314	388.3494	60.53246	386.817	64.38266
388.6228	67.50123	388.3909	60.53225	386.8581	64.3811
388.6628	67.49871	388.4339	60.53271	386.8919	64.37936
388.7068	67.4998	388.466	60.53311	386.9332	64.37751
388.749	67.49912	388.5072	60.53234	386.9746	64.37844
388.7904	67.49751	388.5508	60.5354	387.015	64.37916
388.8328	67.49671	388.592	60.53705	387.0564	64.37936
388.8737	67.49271	388.6328	60.53541	387.0968	64.38051
388.9138	67.4886	388.6763	60.53667	387.1385	64.37995
388.9544	67.4883	388.7161	60.53356	387.1824	64.37947
388.995	67.48947	388.756	60.52884	387.2235	64.37826
389.0302	67.48928	388.7978	60.52979	387.2668	64.37584
389.0725	67.48695	388.8422	60.52905	387.3072	64.37548
389.1142	67.48565	388.8824	60.52717	387.3506	64.37466

389.1538	67.48281	388.915	60.52799	387.3922	64.37465
389.193	67.48206	388.956	60.52705	387.4314	64.3757
389.2352	67.48191	388.9981	60.52752	387.4748	64.37541
389.2786	67.48175	389.0372	60.52906	387.5078	64.37488
389.3204	67.48034	389.0794	60.53106	387.5466	64.37418
389.3604	67.47804	389.1219	60.53049	387.5895	64.37338
389.404	67.47591	389.1617	60.5282	387.6309	64.37259
389.445	67.47434	389.2012	60.52476	387.6732	64.37271
389.4776	67.47317	389.2431	60.52076	387.7164	64.37266
389.5205	67.4716	389.2851	60.51881	387.7582	64.37157
389.5602	67.47448	389.3267	60.51759	387.8004	64.36956
389.6024	67.47379	389.3617	60.51594	387.8387	64.36879
389.6446	67.47145	389.4014	60.51616	387.8828	64.36924
389.688	67.47021	389.4425	60.51819	387.9216	64.36933
389.7292	67.4657	389.4845	60.5175	387.9632	64.36954
389.771	67.46252	389.5256	60.5173	388.0093	64.36864
389.8134	67.46176	389.5688	60.51818	388.0491	64.36666
389.8526	67.46117	389.6126	60.51657	388.0908	64.36667
389.8952	67.46183	389.652	60.51674	388.1262	64.36661
389.9278	67.4624	389.6944	60.51817	388.164	64.36626
389.9685	67.46428	389.7336	60.51667	388.2066	64.36477
390.012	67.46482	389.7764	60.51731	388.2474	64.36394
390.0533	67.4614	389.8094	60.51752	388.2898	64.36378
390.0942	67.45747	389.8508	60.51548	388.3325	64.36332
390.136	67.45584	389.8924	60.5165	388.3745	64.36369
390.1752	67.45322	389.9328	60.51548	388.4144	64.36261
390.2166	67.45214	389.973	60.51255	388.4554	64.36155
390.26	67.45338	390.0166	60.51118	388.4953	64.36148
390.3003	67.44965	390.0574	60.50877	388.5386	64.36149
390.3397	67.44672	390.1015	60.50604	388.5828	64.3619
390.3838	67.44608	390.1422	60.50496	388.6243	64.36018
390.4213	67.44702	390.1811	60.50851	388.655	64.35973
390.4549	67.44878	390.2248	60.50949	388.6956	64.3607
390.4967	67.44799	390.2572	60.50881	388.7376	64.36046
390.54	67.4465	390.3021	60.51036	388.7774	64.36241
390.5828	67.44417	390.3414	60.50952	388.8175	64.36274
390.6263	67.44054	390.3836	60.5087	388.8602	64.36001
390.6658	67.43849	390.4248	60.50771	388.9045	64.35884
390.709	67.43794	390.4655	60.505	388.944	64.35697
390.7484	67.43683	390.5059	60.50309	388.9844	64.35588
390.7907	67.43482	390.5495	60.50276	389.0263	64.35639
390.8332	67.43387	390.5915	60.50229	389.0708	64.35656
390.8714	67.43265	390.6319	60.50065	389.1096	64.35635
390.9072	67.43174	390.6732	60.49904	389.1511	64.3557
390.9469	67.43373	390.7098	60.4965	389.1941	64.35596
390.9892	67.43606	390.7477	60.49437	389.2347	64.3549
391.032	67.43615	390.7883	60.49568	389.2676	64.35505
391.0725	67.43689	390.8307	60.49316	389.3089	64.35504
391.114	67.4326	390.8716	60.49166	389.352	64.35361
391.1538	67.43022	390.914	60.4911	389.3945	64.35237
391.1972	67.4278	390.9575	60.48976	389.4329	64.35087
391.24	67.42435	391.0002	60.49028	389.4754	64.3495
391.2792	67.42605	391.0406	60.49069	389.5161	64.34764
391.3214	67.42531	391.0821	60.49217	389.5579	64.34709
391.354	67.42635	391.1228	60.4943	389.5983	64.34825
391.3933	67.42616	391.1544	60.49517	389.6434	64.34898
391.4367	67.42552	391.1959	60.49484	389.683	64.34938
391.4762	67.42476	391.2352	60.49411	389.7258	64.34991

391.5163	67.42246	391.279	60.49334	389.768	64.34823
391.5594	67.42242	391.3213	60.49064	389.8074	64.34645
391.601	67.42173	391.3645	60.48827	389.8492	64.34663
391.6413	67.4168	391.4052	60.48785	389.8817	64.34555
391.6858	67.41452	391.4466	60.48486	389.9255	64.34561
391.7248	67.41435	391.4894	60.48422	389.9662	64.34494
391.7683	67.41235	391.5292	60.48447	390.0076	64.34434
391.8	67.41217	391.5691	60.4836	390.0493	64.34547
391.8429	67.41336	391.6044	60.48341	390.0918	64.34347
391.884	67.41186	391.6473	60.48273	390.1322	64.34158
391.923	67.40798	391.6865	60.48	390.1744	64.33966
391.9631	67.4059	391.728	60.47697	390.2154	64.33683
392.0045	67.40564	391.7705	60.47534	390.2561	64.33711
392.0438	67.40432	391.8122	60.47548	390.2974	64.33819
392.091	67.40454	391.8552	60.47705	390.3389	64.33868
392.1312	67.40384	391.8965	60.48118	390.3821	64.33989
392.1731	67.40039	391.9416	60.48174	390.4139	64.34004
392.2148	67.39864	391.9778	60.48077	390.4556	64.34026
392.2564	67.39926	392.0202	60.48079	390.4981	64.3407
392.297	67.40102	392.0517	60.47869	390.54	64.34029
392.3324	67.40191	392.0947	60.47712	390.5778	64.33937
392.3709	67.40141	392.1363	60.4768	390.623	64.33775
392.4139	67.39953	392.1776	60.47566	390.6638	64.33563
392.4546	67.39465	392.2197	60.47324	390.7061	64.33312
392.4985	67.39029	392.2612	60.47252	390.748	64.33142
392.538	67.38728	392.304	60.47116	390.7889	64.33154
392.5783	67.38624	392.3473	60.46944	390.8288	64.33224
392.6224	67.38715	392.3862	60.469	390.8702	64.33247
392.6612	67.38887	392.428	60.46881	390.9093	64.33188
392.7029	67.39115	392.468	60.46933	390.9524	64.3309
392.7449	67.38965	392.5049	60.4707	390.9958	64.32919
392.7771	67.38752	392.5458	60.47324	391.0294	64.32884
392.8194	67.386	392.5867	60.47242	391.0711	64.32784
392.8612	67.38406	392.6277	60.47248	391.1103	64.3272
392.9017	67.38167	392.6691	60.47284	391.1531	64.32729
392.9435	67.37876	392.7103	60.47026	391.1942	64.32718
392.9853	67.37807	392.7534	60.47004	391.235	64.32675
393.027	67.37767	392.7919	60.47025	391.2762	64.32606
393.0698	67.37769	392.8347	60.46796	391.3176	64.32525
393.1104	67.37856	392.8766	60.46859	391.3614	64.32361
393.1518	67.37731	392.9211	60.47312	391.4025	64.324
393.1947	67.37536	392.9542	60.474	391.4454	64.32489
393.2265	67.37202	392.9955	60.47213	391.4867	64.32513
393.269	67.37042	393.037	60.47075	391.5266	64.32544
393.3125	67.36983	393.0794	60.4654	391.5697	64.32436
393.3524	67.365	393.1194	60.46099	391.6096	64.32229
393.3925	67.36066	393.1622	60.46299	391.6422	64.32153
393.433	67.3543	393.2024	60.46276	391.6848	64.31981
393.4764	67.34761	393.2442	60.46324	391.7297	64.31903
393.5169	67.34069	393.285	60.46474	391.7706	64.31985
393.5604	67.33348	393.3267	60.46253	391.8095	64.31889
393.6002	67.33439	393.3695	60.46232	391.8527	64.32129
393.6425	67.33898	393.4016	60.46235	391.8948	64.32279
393.6742	67.34424	393.443	60.45972	391.9364	64.3217
393.716	67.35221	393.4822	60.45792	391.9762	64.32163
393.7552	67.35779	393.525	60.4584	392.019	64.31916
393.7998	67.35444	393.5672	60.45537	392.0604	64.31636
393.8403	67.35005	393.6087	60.4531	392.0997	64.31437

393.8814	67.35097	393.6498	60.4515	392.1438	64.31296
393.9234	67.35074	393.6935	60.44811	392.1772	64.31223
393.9662	67.35268	393.733	60.4477	392.2167	64.31242
394.0064	67.35472	393.7744	60.44896	392.2626	64.31338
394.0483	67.35133	393.813	60.44802	392.303	64.31389
394.0896	67.34465	393.8476	60.44923	392.3433	64.31285
394.134	67.33955	393.8889	60.45114	392.3835	64.31117
394.1741	67.33403	393.9313	60.44949	392.4252	64.31042
394.209	67.32946	393.9716	60.45011	392.4682	64.30923
394.248	67.32885	394.015	60.45271	392.5094	64.30929
394.294	67.32965	394.0544	60.4505	392.5518	64.3104
394.3332	67.3264	394.0972	60.44942	392.5934	64.3107
394.375	67.31927	394.1392	60.44959	392.6326	64.31153
394.416	67.31417	394.1822	60.44527	392.675	64.31244
394.4574	67.31107	394.223	60.44182	392.7172	64.31155
394.4991	67.31385	394.266	60.43933	392.7574	64.31005
394.5407	67.32116	394.3004	60.43684	392.791	64.30865
394.584	67.32746	394.344	60.43373	392.8317	64.30752
394.625	67.32885	394.386	60.43366	392.873	64.30602
394.6563	67.32803	394.4259	60.43341	392.9141	64.30539
394.6986	67.32789	394.4666	60.43296	392.9537	64.30499
394.7402	67.32883	394.5083	60.43519	392.9961	64.30312
394.7818	67.3299	394.5508	60.43395	393.0375	64.30291
394.8236	67.33129	394.5928	60.43669	393.0798	64.30372
394.8642	67.33177	394.6341	60.4377	393.1243	64.30415
394.907	67.32989	394.6758	60.43442	393.1645	64.30476
394.9468	67.32694	394.7144	60.43339	393.2054	64.30598
394.9897	67.32526	394.749	60.43257	393.2476	64.30657
395.0311	67.32437	394.7931	60.43232	393.2889	64.30593
395.0715	67.32451	394.8334	60.43237	393.3276	64.30427
395.1056	67.32344	394.875	60.43316	393.37	64.30233
395.1472	67.32153	394.919	60.4309	393.408	64.30077
395.1878	67.31914	394.9586	60.4288	393.4474	64.29877
395.2295	67.31419	394.999	60.429	393.4883	64.29845
395.272	67.31281	395.04	60.42915	393.5304	64.29713
395.3135	67.31273	395.0805	60.42915	393.5703	64.2957
395.3543	67.31241	395.123	60.42877	393.6118	64.29445
395.3937	67.31161	395.1619	60.42898	393.655	64.29427
395.4391	67.30819	395.195	60.42785	393.6958	64.29368
395.4814	67.30599	395.237	60.4288	393.7372	64.2923
395.5222	67.30529	395.2798	60.42785	393.7797	64.29142
395.5549	67.30618	395.3228	60.42355	393.8192	64.29071
395.5964	67.30773	395.3652	60.42214	393.8614	64.29075
395.6372	67.30775	395.4037	60.41839	393.9036	64.29047
395.6775	67.30487	395.4438	60.41662	393.9376	64.29006
395.7182	67.30345	395.4876	60.41834	393.978	64.28914
395.7609	67.30147	395.5288	60.41822	394.0192	64.28804
395.8011	67.29865	395.568	60.41778	394.0602	64.28794
395.843	67.29712	395.61	60.418	394.1025	64.28881
395.8844	67.29629	395.6428	60.4171	394.1428	64.28931
395.9262	67.29351	395.6831	60.41558	394.1839	64.28839
395.9692	67.29159	395.7265	60.41635	394.2258	64.28547
396.0098	67.29159	395.7674	60.41575	394.2668	64.28401
396.0525	67.28936	395.8081	60.4157	394.3086	64.28334
396.0851	67.28703	395.8512	60.41621	394.3502	64.28461
396.1269	67.28647	395.893	60.4147	394.3912	64.28802
396.1671	67.28568	395.9368	60.41339	394.4326	64.29022
396.2086	67.2846	395.9767	60.41391	394.4748	64.28999

396.251	67.28388	396.0179	60.41301	394.5167	64.28801
396.2928	67.28372	396.0593	60.41127	394.5513	64.2857
396.332	67.28314	396.092	60.41173	394.5948	64.28198
396.3761	67.28375	396.1354	60.41131	394.6355	64.28013
396.4172	67.28462	396.1756	60.40879	394.6743	64.28046
396.458	67.28526	396.2173	60.40849	394.7154	64.28061
396.4989	67.2824	396.2595	60.408	394.7574	64.28055
396.5344	67.28072	396.304	60.4067	394.7989	64.27866
396.5752	67.27873	396.3413	60.40504	394.8408	64.27811
396.6164	67.27601	396.3844	60.40472	394.8832	64.27759
396.6576	67.27563	396.428	60.40282	394.923	64.27654
396.6994	67.27386	396.468	60.40079	394.964	64.27677
396.737	67.27211	396.512	60.40127	395.0078	64.27593
396.782	67.27081	396.5415	60.4009	395.0489	64.27492
396.8244	67.26916	396.5844	60.40122	395.0896	64.27495
396.8662	67.26722	396.6258	60.40156	395.1301	64.27495
396.9047	67.26723	396.6688	60.40044	395.1642	64.27391
396.9442	67.26564	396.71	60.39955	395.207	64.27418
396.9814	67.26575	396.7526	60.3996	395.2492	64.27413
397.0246	67.26601	396.7908	60.39696	395.2902	64.27361
397.0645	67.26199	396.8342	60.3971	395.3298	64.27413
397.1063	67.26019	396.8761	60.40004	395.3745	64.27361
397.1469	67.25822	396.9178	60.39958	395.4145	64.27181
397.1885	67.25504	396.9597	60.40151	395.4575	64.27103
397.2282	67.25371	396.9924	60.40302	395.4981	64.26942
397.269	67.25269	397.031	60.39914	395.5389	64.26825
397.3112	67.25258	397.0747	60.3966	395.5814	64.26849
397.3552	67.25282	397.1156	60.39591	395.6228	64.26891
397.3992	67.25084	397.1588	60.39303	395.6631	64.26869
397.4307	67.24835	397.1998	60.39305	395.6992	64.26741
397.4741	67.24496	397.2416	60.39304	395.7383	64.26791
397.5174	67.24439	397.2838	60.39043	395.7792	64.26719
397.5571	67.24201	397.3255	60.39106	395.8219	64.26576
397.5968	67.24295	397.3663	60.39203	395.8644	64.26588
397.6393	67.24311	397.4095	60.39027	395.907	64.2639
397.679	67.23986	397.4388	60.38962	395.9456	64.26295
397.7184	67.24206	397.4811	60.38778	395.9917	64.26253
397.7612	67.24331	397.5242	60.38411	396.0322	64.26149
397.8035	67.24299	397.5668	60.38202	396.074	64.26192
397.8462	67.24179	397.6078	60.3836	396.1136	64.26129
397.8877	67.23848	397.65	60.38214	396.156	64.25974
397.9272	67.23485	397.6911	60.38062	396.1997	64.2583
397.9622	67.23156	397.7317	60.38202	396.2398	64.25535
398.003	67.22975	397.7744	60.38101	396.2822	64.255
398.0459	67.23034	397.8158	60.38193	396.3165	64.25474
398.0881	67.22954	397.8561	60.38177	396.3571	64.25473
398.1279	67.23067	397.8874	60.38031	396.396	64.25717
398.169	67.2313	397.9276	60.37823	396.4378	64.25945
398.21	67.22974	397.97	60.37599	396.4795	64.2609
398.2535	67.2293	398.0102	60.37507	396.5244	64.26103
398.2943	67.22608	398.0537	60.3742	396.5649	64.26036
398.334	67.22459	398.0961	60.37663	396.6053	64.25609
398.3767	67.22281	398.1363	60.37659	396.6478	64.25337
398.4114	67.22223	398.1787	60.37721	396.689	64.2524
398.452	67.22251	398.2226	60.37757	396.7279	64.25134
398.492	67.21934	398.2626	60.37466	396.772	64.25148
398.5352	67.2135	398.3052	60.37321	396.8128	64.25077
398.5735	67.20952	398.3359	60.37289	396.8534	64.25033

398.6151	67.20758	398.3788	60.36974	396.8954	64.24879
398.6581	67.20544	398.4232	60.36726	396.931	64.24728
398.6995	67.2061	398.4638	60.36794	396.9724	64.24679
398.7398	67.20834	398.5058	60.36704	397.0125	64.24608
398.7814	67.20621	398.5452	60.36698	397.0564	64.24545
398.8216	67.20513	398.587	60.36852	397.0978	64.24576
398.857	67.20658	398.629	60.36623	397.1375	64.24548
398.8968	67.20516	398.6693	60.3652	397.178	64.24466
398.9389	67.20156	398.7106	60.36782	397.2186	64.24419
398.9798	67.20041	398.752	60.36593	397.2613	64.24319
399.0211	67.20133	398.7843	60.36602	397.3028	64.24154
399.0603	67.20165	398.8287	60.36433	397.3466	64.24117
399.1022	67.20268	398.8702	60.35933	397.385	64.24146
399.1448	67.2039	398.9107	60.35719	397.4294	64.24291
399.1828	67.20182	398.9525	60.35593	397.4625	64.24448
399.2272	67.19965	398.9946	60.35545	397.5048	64.24357
399.2659	67.19874	399.0354	60.35618	397.5442	64.24101
399.3012	67.19838	399.0774	60.35555	397.5867	64.23848
399.341	67.19771	399.1183	60.35432	397.6295	64.23663
399.383	67.19698	399.1613	60.35518	397.6726	64.23605
399.4237	67.19457	399.1997	60.35491	397.7116	64.23683
399.4658	67.19119	399.2351	60.35424	397.754	64.23627
399.5074	67.18778	399.2756	60.35443	397.7944	64.23457
399.5498	67.18437	399.3174	60.35451	397.8352	64.23501
399.5899	67.18461	399.3594	60.35233	397.876	64.23391
399.6308	67.18435	399.4002	60.35196	397.9191	64.23377
399.6724	67.18341	399.4414	60.35208	397.962	64.23443
399.7124	67.18186	399.4812	60.35015	398.001	64.23296
399.755	67.17709	399.5255	60.35008	398.0436	64.23288
399.7947	67.17365	399.5669	60.34987	398.0759	64.23283
399.8294	67.17415	399.6082	60.34816	398.1181	64.23144
399.8711	67.17391	399.6504	60.34752	398.1584	64.2311
399.9147	67.17331	399.6819	60.3479	398.2011	64.23042
399.9545	67.17129	399.7236	60.34713	398.2416	64.22955
399.9977	67.16921	399.7672	60.35012	398.2862	64.22944
400.0388	67.16835	399.807	60.35284	398.3267	64.22942
400.0812	67.1677	399.8494	60.35182	398.3692	64.22815
400.1215	67.16976	399.889	60.35276	398.4102	64.22772
400.1639	67.16802	399.9318	60.34935	398.4514	64.22761
400.2042	67.16052	399.9736	60.34365	398.4958	64.22698
400.2455	67.15583	400.0153	60.34336	398.5338	64.22762
400.2792	67.15436	400.0572	60.34145	398.5754	64.22646
400.3205	67.15482	400.0995	60.33964	398.6167	64.22497
400.3605	67.15615	400.1317	60.34067	398.6578	64.22375
400.402	67.15925	400.1764	60.33901	398.6915	64.22355
400.444	67.15929	400.2141	60.33755	398.7316	64.22322
400.4865	67.15665	400.258	60.33817	398.7747	64.22235
400.5269	67.15701	400.2993	60.33683	398.818	64.22147
400.5678	67.15372	400.3395	60.33581	398.8569	64.21889
400.6112	67.14942	400.3817	60.3356	398.8988	64.21651
400.6531	67.14607	400.4228	60.33433	398.9394	64.21542
400.6949	67.14306	400.4667	60.33502	398.9814	64.21501
400.725	67.14149	400.5054	60.33579	399.0214	64.21475
400.7646	67.13811	400.5456	60.33284	399.0631	64.21478
400.8088	67.13772	400.581	60.33029	399.1052	64.21437
400.8512	67.13692	400.6233	60.32672	399.1442	64.21363
400.8906	67.1367	400.666	60.32384	399.1864	64.21303
400.9322	67.13841	400.706	60.32362	399.2202	64.21295

400.9769	67.13928	400.7473	60.32479	399.2626	64.21281
401.0128	67.1388	400.7895	60.3266	399.3057	64.21169
401.0578	67.13874	400.8304	60.32669	399.346	64.21108
401.0984	67.13693	400.8732	60.32667	399.388	64.20961
401.1418	67.13355	400.9164	60.32597	399.427	64.20905
401.1728	67.13144	400.956	60.32466	399.4678	64.20971
401.2159	67.12721	400.9966	60.32398	399.5094	64.20923
401.256	67.12579	401.0305	60.32364	399.5527	64.20969
401.2982	67.12635	401.0727	60.3227	399.5934	64.20984
401.3386	67.12605	401.1118	60.32319	399.6345	64.20828
401.383	67.12474	401.1554	60.32289	399.6756	64.20734
401.4223	67.12505	401.1962	60.32143	399.7196	64.20683
401.4633	67.12496	401.2375	60.32213	399.76	64.20717
401.5064	67.12431	401.279	60.32174	399.8008	64.20778
401.5468	67.12549	401.3198	60.32042	399.834	64.20775
401.5928	67.12427	401.364	60.31979	399.8766	64.20714
401.6315	67.12201	401.4041	60.31619	399.9176	64.20572
401.6741	67.11926	401.4451	60.31352	399.9579	64.20391
401.7055	67.11771	401.4786	60.3121	399.9995	64.20303
401.7494	67.11498	401.5208	60.31133	400.0408	64.20274
401.79	67.10955	401.562	60.31159	400.0834	64.20186
401.8336	67.10775	401.6026	60.31326	400.1255	64.20058
401.8739	67.10725	401.6433	60.31523	400.167	64.20021
401.9158	67.10834	401.6862	60.31519	400.2094	64.19924
401.9598	67.11094	401.7252	60.31694	400.2492	64.19749
401.9989	67.11031	401.7682	60.31792	400.29	64.19792
402.042	67.10777	401.8066	60.3157	400.3352	64.19776
402.0833	67.10477	401.85	60.31812	400.3754	64.19722
402.1228	67.09931	401.8941	60.31553	400.4172	64.19843
402.1576	67.09886	401.926	60.3102	400.4494	64.1978
402.201	67.09927	401.9674	60.30776	400.4912	64.19711
402.2451	67.09956	402.007	60.30395	400.5337	64.19619
402.2838	67.10242	402.0496	60.30125	400.5742	64.19418
402.3244	67.10139	402.0941	60.30203	400.6155	64.19258
402.3678	67.0984	402.1343	60.30222	400.6586	64.19221
402.4084	67.09601	402.176	60.30074	400.6994	64.19132
402.4491	67.09168	402.2175	60.30025	400.7418	64.19084
402.4925	67.08938	402.2618	60.29928	400.7865	64.19072
402.5318	67.08729	402.3011	60.29779	400.8268	64.1897
402.5732	67.08738	402.3422	60.29819	400.8672	64.1904
402.6057	67.08961	402.3762	60.29956	400.9088	64.18985
402.6487	67.08887	402.4178	60.29815	400.9528	64.18874
402.6938	67.08816	402.459	60.29663	400.984	64.18836
402.734	67.08687	402.5004	60.29492	401.0255	64.18659
402.7762	67.08279	402.5408	60.2911	401.0659	64.18609
402.8187	67.08165	402.583	60.28964	401.1072	64.18616
402.8616	67.08181	402.6247	60.28989	401.1517	64.18619
402.9018	67.0805	402.6652	60.28946	401.1897	64.18546
402.9407	67.07931	402.7058	60.28975	401.2314	64.18374
402.9841	67.07851	402.7478	60.29009	401.2722	64.18221
403.0246	67.07888	402.789	60.28952	401.3149	64.18096
403.0568	67.07763	402.8227	60.28967	401.3562	64.17935
403.0986	67.07454	402.8635	60.2897	401.3987	64.17954
403.1392	67.07489	402.906	60.28921	401.4375	64.17954
403.1798	67.07376	402.9457	60.28956	401.4816	64.17796
403.2202	67.07318	402.9872	60.29039	401.5207	64.17838
403.2615	67.07486	403.0321	60.28835	401.564	64.17811
403.306	67.07386	403.0742	60.286	401.5964	64.17811

403.3457	67.07168	403.1142	60.28542	401.639	64.17823
403.387	67.06744	403.1575	60.28329	401.6791	64.1781
403.4269	67.06501	403.1998	60.28306	401.7217	64.17627
403.471	67.06307	403.2395	60.28582	401.7644	64.17466
403.5124	67.06066	403.2727	60.28465	401.8076	64.17435
403.5548	67.0618	403.3134	60.28308	401.8482	64.17389
403.5866	67.06149	403.3544	60.28295	401.8878	64.17444
403.6302	67.05997	403.398	60.28138	401.9281	64.17314
403.671	67.05979	403.437	60.28057	401.9702	64.17103
403.7113	67.05713	403.481	60.28129	402.0132	64.16944
403.7537	67.05255	403.5229	60.27976	402.0546	64.16844
403.7929	67.05081	403.565	60.2779	402.0972	64.16826
403.8346	67.04799	403.6048	60.27637	402.1387	64.1677
403.8776	67.04786	403.6479	60.27472	402.1802	64.16703
403.9175	67.05031	403.6875	60.27261	402.215	64.16626
403.9604	67.05184	403.7222	60.27099	402.257	64.16574
404.0008	67.05297	403.7632	60.26943	402.2976	64.16654
404.0338	67.05287	403.806	60.26661	402.3383	64.16756
404.0762	67.05	403.8454	60.26804	402.3795	64.16732
404.1188	67.04429	403.886	60.26828	402.4205	64.16672
404.1599	67.04309	403.9299	60.26605	402.4617	64.16662
404.2035	67.04135	403.974	60.26593	402.505	64.16567
404.2445	67.03838	404.0127	60.26596	402.5448	64.16558
404.2841	67.04131	404.052	60.265	402.587	64.16557
404.3256	67.04053	404.0957	60.26593	402.628	64.16462
404.366	67.0388	404.1355	60.26492	402.669	64.16379
404.4082	67.03839	404.1709	60.26357	402.7126	64.16296
404.45	67.03734	404.2104	60.26274	402.7455	64.16252
404.4819	67.03437	404.2516	60.26238	402.7864	64.16117
404.5256	67.03219	404.2932	60.26152	402.829	64.16013
404.5656	67.03155	404.3363	60.2602	402.868	64.15957
404.609	67.02854	404.3786	60.25894	402.9116	64.15865
404.6509	67.02496	404.423	60.25811	402.952	64.15818
404.691	67.02208	404.4607	60.25836	402.9929	64.15735
404.7334	67.02102	404.5016	60.25732	403.0324	64.15648
404.7753	67.01986	404.542	60.25528	403.0746	64.15562
404.8164	67.02156	404.5834	60.25245	403.1152	64.15468
404.8558	67.02358	404.6196	60.24972	403.1584	64.1544
404.8995	67.02238	404.6583	60.24699	403.199	64.15372
404.932	67.0221	404.6975	60.24585	403.2418	64.15269
404.974	67.01956	404.741	60.24535	403.2826	64.15236
405.0136	67.0172	404.7822	60.24611	403.3237	64.15233
405.0558	67.01666	404.8235	60.24598	403.3555	64.1522
405.098	67.01545	404.8684	60.24503	403.3985	64.15286
405.1387	67.01496	404.9101	60.2432	403.4384	64.15211
405.1798	67.01458	404.9503	60.24217	403.4812	64.1511
405.2216	67.01139	404.995	60.24069	403.5236	64.15057
405.2622	67.00821	405.0332	60.23962	403.5627	64.14895
405.3028	67.00732	405.0664	60.23863	403.6043	64.14773
405.3452	67.00486	405.1084	60.23669	403.6484	64.14583
405.3874	67.00339	405.1506	60.23537	403.6887	64.1443
405.4285	67.00438	405.1936	60.23618	403.7305	64.14391
405.46	67.00323	405.2368	60.23771	403.771	64.14382
405.5031	67.00051	405.278	60.23758	403.8141	64.14401
405.5429	66.99978	405.3188	60.23802	403.8531	64.14405
405.5846	66.99926	405.3598	60.23722	403.8962	64.14272
405.627	66.99886	405.4018	60.23427	403.9382	64.14157
405.6672	66.99708	405.4433	60.23467	403.9718	64.14047

405.7107	66.99508	405.4844	60.23397	404.0129	64.13969
405.7518	66.99203	405.5168	60.23195	404.0556	64.13895
405.793	66.98693	405.5594	60.23328	404.0964	64.13789
405.8324	66.98349	405.6004	60.23357	404.1367	64.13766
405.874	66.98295	405.6422	60.23171	404.1778	64.13499
405.9079	66.98262	405.6865	60.23204	404.2203	64.13311
405.9498	66.98109	405.7246	60.23151	404.2641	64.13242
405.9925	66.9841	405.769	60.22909	404.303	64.13158
406.0319	66.97933	405.8092	60.22928	404.3458	64.13153
406.0747	66.97071	405.8513	60.22892	404.388	64.13122
406.117	66.97026	405.893	60.22579	404.4294	64.13085
406.1563	66.9685	405.9348	60.22476	404.4709	64.12979
406.1984	66.96972	405.9657	60.2236	404.5053	64.12951
406.2407	66.97531	406.0083	60.22181	404.5468	64.13012
406.2834	66.97419	406.0531	60.22349	404.5869	64.12969
406.3269	66.97054	406.0908	60.22417	404.6287	64.12904
406.3591	66.96958	406.1356	60.22479	404.6697	64.12903
406.3996	66.96945	406.1761	60.22587	404.7113	64.12817
406.4424	66.96834	406.2176	60.22493	404.7522	64.12794
406.483	66.96742	406.2593	60.22391	404.7959	64.12767
406.5244	66.96432	406.3012	60.22398	404.8348	64.12542
406.5672	66.96221	406.3422	60.22239	404.8763	64.12403
406.6065	66.95981	406.3815	60.21949	404.9222	64.12336
406.6506	66.95534	406.4162	60.21944	404.9608	64.12277
406.6904	66.95519	406.4544	60.21717	405.0032	64.12433
406.7324	66.9545	406.4968	60.21433	405.0442	64.12462
406.7738	66.95072	406.5383	60.21458	405.0856	64.12353
406.805	66.94868	406.5786	60.21365	405.1189	64.12239
406.8483	66.94481	406.6233	60.21299	405.161	64.11947
406.8886	66.93882	406.6624	60.21278	405.202	64.11737
406.9301	66.93535	406.7061	60.21253	405.2422	64.11676
406.9716	66.93765	406.7459	60.21157	405.2843	64.11702
407.0132	66.94208	406.7861	60.2118	405.3269	64.11712
407.054	66.9458	406.8293	60.21129	405.3664	64.11717
407.0948	66.94793	406.8623	60.20935	405.4089	64.11551
407.138	66.94874	406.905	60.20859	405.4492	64.11336
407.1808	66.94682	406.9465	60.20677	405.492	64.11215
407.2217	66.94476	406.9875	60.20488	405.5322	64.11114
407.2617	66.94424	407.0281	60.20423	405.5757	64.11142
407.304	66.94356	407.0714	60.20375	405.6154	64.11202
407.3356	66.94183	407.1105	60.20411	405.6587	64.11255
407.3772	66.94106	407.1533	60.20277	405.7031	64.1118
407.4181	66.93854	407.1954	60.20268	405.7323	64.11075
407.461	66.93504	407.2347	60.20401	405.7778	64.10955
407.5024	66.93553	407.2758	60.20292	405.8184	64.10845
407.543	66.93513	407.3063	60.20531	405.86	64.10831
407.5889	66.93465	407.3498	60.20474	405.8994	64.10789
407.6263	66.93247	407.3925	60.20154	405.9398	64.10673
407.6688	66.92902	407.4318	60.19986	405.981	64.10478
407.7094	66.92656	407.4737	60.19494	406.0252	64.10306
407.7509	66.92422	407.5145	60.19312	406.0684	64.10203
407.786	66.92539	407.5534	60.19435	406.1067	64.10126
407.8257	66.92564	407.5986	60.19268	406.1475	64.10117
407.8672	66.92419	407.64	60.19239	406.1907	64.1004
407.9109	66.92169	407.683	60.19329	406.2323	64.09902
407.953	66.92015	407.7265	60.1898	406.2662	64.09869
407.9934	66.91956	407.7566	60.18748	406.3065	64.09872
408.0348	66.91908	407.7968	60.18796	406.3495	64.09787

408.0756	66.91789	407.8419	60.18735	406.3906	64.09738
408.1178	66.91457	407.8808	60.18687	406.4317	64.09656
408.1602	66.90972	407.9209	60.18717	406.4742	64.09473
408.2017	66.90497	407.9641	60.18489	406.5142	64.09482
408.2328	66.90313	408.0065	60.18145	406.5562	64.0947
408.2754	66.90329	408.0494	60.1807	406.5976	64.09429
408.3176	66.90263	408.0892	60.18	406.6405	64.09426
408.3594	66.90357	408.1333	60.18126	406.6806	64.09272
408.403	66.90429	408.175	60.18343	406.7224	64.0908
408.4426	66.903	408.208	60.18144	406.7634	64.09033
408.4846	66.90179	408.249	60.18092	406.8069	64.09002
408.5259	66.8991	408.2907	60.18068	406.8466	64.08951
408.5648	66.89735	408.3322	60.17696	406.8812	64.08991
408.6101	66.89477	408.3757	60.17576	406.9222	64.08949
408.6501	66.89268	408.4153	60.17413	406.962	64.0877
408.6834	66.8919	408.459	60.17235	407.0044	64.08702
408.7254	66.89115	408.5008	60.17216	407.0458	64.08705
408.7664	66.89315	408.5405	60.17078	407.088	64.08588
408.8092	66.89387	408.5828	60.17104	407.1301	64.08543
408.8478	66.89374	408.626	60.17204	407.1706	64.08523
408.8911	66.89042	408.6602	60.17229	407.2133	64.08302
408.9322	66.88715	408.6992	60.1717	407.255	64.08161
408.9741	66.8866	408.7404	60.17102	407.2966	64.08129
409.0144	66.8866	408.7848	60.16962	407.3368	64.08136
409.0563	66.88554	408.8238	60.16601	407.379	64.08195
409.0976	66.88511	408.8653	60.16591	407.4208	64.08217
409.1393	66.88409	408.908	60.16458	407.463	64.08028
409.1796	66.88246	408.9498	60.16117	407.4944	64.07832
409.2146	66.88161	408.9903	60.1602	407.536	64.07723
409.255	66.88113	409.034	60.1585	407.578	64.07646
409.2958	66.87909	409.075	60.15732	407.6208	64.07744
409.3367	66.87659	409.1106	60.15829	407.6647	64.0781
409.3809	66.87574	409.1512	60.15746	407.7038	64.07768
409.4214	66.87408	409.1918	60.15732	407.7454	64.07751
409.46	66.87235	409.2332	60.15783	407.7862	64.07662
409.5025	66.86837	409.2722	60.1574	407.829	64.0757
409.5454	66.86438	409.317	60.15744	407.8689	64.07508
409.5861	66.86337	409.3572	60.15756	407.9113	64.0731
409.6292	66.86179	409.398	60.15633	407.953	64.07216
409.6617	66.86237	409.4394	60.1537	407.9942	64.07201
409.7035	66.86479	409.4822	60.15374	408.0277	64.07113
409.7455	66.86366	409.5205	60.1518	408.0714	64.07036
409.7868	66.862	409.556	60.14903	408.112	64.07025
409.8294	66.85882	409.5969	60.14903	408.1512	64.06914
409.8698	66.85511	409.6406	60.14837	408.1936	64.06823
409.9112	66.85255	409.6803	60.1472	408.2346	64.06693
409.9544	66.85054	409.722	60.14843	408.2782	64.06585
409.9936	66.85193	409.7665	60.14731	408.32	64.06552
410.0344	66.85131	409.8046	60.14621	408.3611	64.0661
410.0761	66.84861	409.8474	60.1456	408.4012	64.06632
410.1108	66.84685	409.8894	60.14456	408.4433	64.06655
410.1504	66.84413	409.9287	60.14449	408.4841	64.06491
410.1918	66.84083	409.9717	60.1448	408.526	64.06269
410.2344	66.84119	410.0031	60.14446	408.5672	64.06171
410.2772	66.83981	410.0436	60.14277	408.6111	64.05932
410.317	66.83822	410.0875	60.14034	408.6429	64.05854
410.3588	66.8384	410.1281	60.13732	408.6858	64.05825
410.4	66.83802	410.1676	60.13466	408.7256	64.05696

410.443	66.83879	410.2114	60.13253	408.7672	64.05727
410.4826	66.83691	410.2524	60.13028	408.8086	64.05665
410.5258	66.82753	410.2953	60.1275	408.8518	64.056
410.5572	66.8216	410.3375	60.12609	408.8936	64.05695
410.5992	66.81786	410.3785	60.1252	408.9348	64.05536
410.641	66.81605	410.4194	60.12592	408.9775	64.05412
410.6811	66.82259	410.452	60.12508	409.0175	64.05274
410.7254	66.8282	410.4924	60.12411	409.061	64.05093
410.7653	66.82772	410.5351	60.12362	409.1007	64.05098
410.8058	66.82496	410.5788	60.12333	409.1428	64.05103
410.8497	66.82257	410.6184	60.12311	409.1828	64.05087
410.8925	66.82049	410.6612	60.12218	409.2236	64.05026
410.9334	66.81805	410.701	60.1209	409.2587	64.04915
410.9765	66.8176	410.7432	60.11917	409.2994	64.04847
411.017	66.81429	410.7829	60.11777	409.3429	64.04761
411.0592	66.81294	410.8259	60.11785	409.3848	64.04614
411.0936	66.8127	410.8679	60.11795	409.4252	64.0451
411.1354	66.81299	410.9	60.11735	409.4657	64.0438
411.1736	66.8112	410.9442	60.1189	409.5066	64.04252
411.2159	66.80593	410.9838	60.11992	409.5493	64.04203
411.2579	66.80086	411.0259	60.12031	409.5919	64.04159
411.3	66.79611	411.0679	60.11914	409.6319	64.04037
411.3418	66.7894	411.109	60.11879	409.6734	64.0406
411.382	66.78245	411.1516	60.11728	409.7144	64.03978
411.422	66.77616	411.1951	60.1158	409.7564	64.03887
411.4638	66.77687	411.233	60.11527	409.791	64.03916
411.5076	66.78499	411.2759	60.11317	409.833	64.03771
411.5414	66.79003	411.317	60.1126	409.8762	64.03642
411.5824	66.79555	411.3506	60.11249	409.9172	64.03559
411.625	66.7944	411.3934	60.11133	409.9575	64.03422
411.6655	66.79067	411.4344	60.11082	409.9981	64.03353
411.707	66.78659	411.476	60.10912	410.04	64.03286
411.7497	66.78505	411.5196	60.10832	410.0847	64.03204
411.7892	66.78577	411.5579	60.1082	410.1226	64.03196
411.8316	66.78539	411.6008	60.10832	410.1642	64.0317
411.8741	66.78421	411.6413	60.10801	410.2078	64.03096
411.916	66.78569	411.6817	60.10704	410.2467	64.03147
411.9554	66.78497	411.724	60.10733	410.2886	64.03016
411.9882	66.78382	411.765	60.10624	410.3275	64.02854
412.0295	66.78505	411.7995	60.10619	410.3687	64.02761
412.0718	66.78542	411.8396	60.10508	410.4034	64.02621
412.1142	66.78432	411.881	60.10184	410.4447	64.02541
412.1547	66.78317	411.9223	60.09997	410.4867	64.02515
412.1957	66.78295	411.9653	60.10169	410.5285	64.0251
412.236	66.78062	412.0059	60.10285	410.5733	64.02445
412.2799	66.77771	412.045	60.10334	410.6128	64.02356
412.3227	66.77619	412.089	60.10346	410.6528	64.02259
412.3625	66.77329	412.1316	60.10193	410.6943	64.02238
412.4033	66.77266	412.1717	60.09931	410.7368	64.02218
412.4353	66.77232	412.2161	60.09876	410.7761	64.02089
412.4766	66.76786	412.2476	60.09857	410.8194	64.02021
412.5193	66.76289	412.2877	60.09603	410.86	64.01869
412.5626	66.75995	412.3315	60.09343	410.9006	64.01704
412.6023	66.75886	412.3709	60.09146	410.9415	64.0167
412.6456	66.7581	412.4126	60.08889	410.9832	64.01609
412.6875	66.75799	412.453	60.0869	411.016	64.0152
412.7281	66.75735	412.4937	60.0862	411.0597	64.01467
412.771	66.75494	412.5373	60.08499	411.0998	64.01391

412.8106	66.75151	412.5787	60.08467	411.1439	64.01286
412.8548	66.74745	412.6204	60.08384	411.1847	64.01327
412.8945	66.74579	412.6626	60.08252	411.2246	64.01316
412.9352	66.74456	412.695	60.08361	411.2662	64.01243
412.9702	66.74197	412.7376	60.08259	411.3096	64.01215
413.0109	66.74455	412.7764	60.08083	411.3492	64.01025
413.0511	66.74438	412.8209	60.08022	411.3928	64.00851
413.0926	66.73999	412.8624	60.08102	411.4351	64.00726
413.1348	66.73648	412.905	60.07982	411.4761	64.00689
413.1772	66.73066	412.9478	60.07942	411.5152	64.00683
413.2188	66.72745	412.9861	60.07894	411.5488	64.00745
413.2584	66.72891	413.0281	60.07511	411.5914	64.00754
413.2992	66.72943	413.0692	60.074	411.6318	64.00606
413.3419	66.72905	413.11	60.07279	411.6739	64.00335
413.382	66.72998	413.1418	60.07039	411.7156	64.00026
413.4148	66.72869	413.1866	60.07108	411.754	63.99915
413.457	66.72464	413.2268	60.07022	411.7976	63.99902
413.4994	66.72399	413.2675	60.06834	411.839	63.99948
413.5414	66.72143	413.3108	60.068	411.8789	64.00038
413.5834	66.71858	413.3519	60.06475	411.923	63.9998
413.6225	66.71925	413.3936	60.06173	411.9662	63.99828
413.6642	66.71984	413.4354	60.06163	412.0064	63.99753
413.7055	66.716	413.478	60.06093	412.0462	63.99697
413.7481	66.71254	413.5172	60.05928	412.0881	63.99669
413.789	66.70937	413.5592	60.05906	412.1297	63.99592
413.8297	66.70587	413.5936	60.05796	412.1646	63.99576
413.8638	66.70556	413.6326	60.05703	412.2047	63.99537
413.9053	66.70493	413.6762	60.05805	412.2486	63.99479
413.9488	66.70584	413.7176	60.05957	412.2894	63.99491
413.9894	66.70762	413.7594	60.05949	412.331	63.99438
414.0305	66.71006	413.7998	60.05836	412.3713	63.99379
414.0713	66.70867	413.8434	60.05719	412.412	63.99239
414.1132	66.70519	413.8832	60.05512	412.4552	63.99226
414.1528	66.70317	413.925	60.05531	412.4954	63.9918
414.1944	66.70078	413.9666	60.05456	412.5362	63.99129
414.2344	66.70174	414.0111	60.05169	412.58	63.99102
414.2744	66.70271	414.0447	60.05049	412.6216	63.98932
414.3089	66.70231	414.0855	60.04793	412.6625	63.98813
414.3511	66.69799	414.125	60.04556	412.7057	63.98589
414.3953	66.69039	414.1666	60.04507	412.7475	63.98451
414.4368	66.68718	414.21	60.04531	412.7782	63.98423
414.4766	66.68485	414.251	60.04369	412.8215	63.98383
414.5234	66.68104	414.293	60.04326	412.8652	63.98278
414.5614	66.67817	414.336	60.0436	412.9056	63.9814
414.6036	66.67401	414.3746	60.04323	412.9466	63.97988
414.6412	66.67091	414.4164	60.04421	412.9912	63.97934
414.6843	66.67165	414.4571	60.04568	413.0295	63.97996
414.7269	66.67348	414.491	60.04495	413.0721	63.98
414.7671	66.67185	414.5325	60.04459	413.1136	63.97948
414.8075	66.66911	414.5741	60.04408	413.1535	63.97698
414.8426	66.66652	414.6168	60.03955	413.1968	63.97479
414.882	66.66034	414.6592	60.03774	413.2382	63.97346
414.927	66.657	414.699	60.03673	413.2794	63.97314
414.9666	66.65333	414.7406	60.03568	413.3132	63.97316
415.0103	66.65129	414.7813	60.03688	413.3558	63.97356
415.0496	66.65062	414.8243	60.03702	413.3967	63.97357
415.0924	66.65011	414.8659	60.03471	413.4385	63.97202
415.135	66.65102	414.9089	60.03302	413.4795	63.97077

415.1762	66.65083	414.9394	60.03176	413.5214	63.96933
415.2188	66.65202	414.9816	60.02919	413.5617	63.96859
415.258	66.65244	415.0218	60.02876	413.6058	63.96734
415.293	66.65158	415.0672	60.02813	413.6484	63.96761
415.3337	66.64962	415.1064	60.02697	413.69	63.96787
415.375	66.64625	415.1468	60.02646	413.7296	63.96788
415.4186	66.63993	415.1883	60.02426	413.7702	63.96839
415.4596	66.63274	415.2291	60.02244	413.814	63.96678
415.4989	66.62383	415.2732	60.02179	413.8548	63.96502
415.5388	66.61825	415.3116	60.02135	413.8964	63.96325
415.5815	66.61544	415.3552	60.02064	413.9316	63.96262
415.626	66.61549	415.3892	60.02007	413.9714	63.96171
415.6674	66.6154	415.4295	60.01912	414.0123	63.9596
415.707	66.61369	415.4717	60.01711	414.055	63.9587
415.741	66.61366	415.5128	60.01716	414.0969	63.95693
415.7846	66.61214	415.5541	60.0175	414.1398	63.95596
415.8242	66.61033	415.5968	60.01694	414.179	63.95681
415.866	66.61077	415.6366	60.01692	414.222	63.95581
415.9062	66.60942	415.6789	60.01485	414.2626	63.95466
415.9466	66.61032	415.7211	60.01279	414.3032	63.95387
415.9888	66.61442	415.7623	60.01288	414.3443	63.95261
416.031	66.61317	415.8045	60.01276	414.3871	63.95314
416.0725	66.61342	415.8368	60.01202	414.4277	63.95328
416.1141	66.60957	415.8775	60.01191	414.4694	63.95334
416.1567	66.60483	415.9189	60.01166	414.51	63.95297
416.186	66.60234	415.962	60.01068	414.5453	63.95152
416.2296	66.5962	416.0014	60.01121	414.5867	63.94981
416.2728	66.59664	416.0444	60.01135	414.6272	63.94857
416.3161	66.59821	416.0863	60.00953	414.6678	63.94661
416.356	66.59947	416.1258	60.00898	414.7109	63.9455
416.3983	66.60303	416.1697	60.00991	414.7526	63.94483
416.4397	66.60049	416.2094	60.01034	414.7926	63.94435
416.479	66.596	416.2509	60.01026	414.834	63.94411
416.5197	66.59564	416.2848	60.00868	414.8772	63.94351
416.5622	66.59542	416.3252	60.00358	414.9172	63.94206
416.603	66.59416	416.3652	59.99938	414.9566	63.94056
416.6452	66.59283	416.4068	59.99837	414.999	63.94013
416.6887	66.58955	416.4495	59.9973	415.0406	63.93912
416.7207	66.58564	416.4916	59.99786	415.0734	63.93913
416.7634	66.58253	416.5318	60.00012	415.1143	63.93865
416.8055	66.58256	416.5728	59.99908	415.1589	63.93738
416.8477	66.58375	416.6161	59.99879	415.1988	63.93703
416.8878	66.58577	416.6594	59.99877	415.2388	63.93613
416.9295	66.58727	416.7006	59.99417	415.28	63.93622
416.969	66.58965	416.7344	59.9915	415.3228	63.93617
417.0115	66.59109	416.7744	59.98952	415.3653	63.93457
417.0538	66.58809	416.8166	59.98635	415.4063	63.93349
417.096	66.58685	416.8571	59.98641	415.4476	63.93248
417.134	66.58349	416.8994	59.98623	415.4906	63.93037
417.1695	66.57881	416.9411	59.98523	415.5316	63.92985
417.2109	66.57811	416.9838	59.9874	415.575	63.92963
417.2538	66.5768	417.0244	59.98811	415.6152	63.92882
417.295	66.57431	417.0678	59.98753	415.6547	63.92903
417.3347	66.57351	417.1088	59.98734	415.6889	63.92852
417.3769	66.57242	417.1504	59.98561	415.7296	63.9275
417.4172	66.57358	417.1814	59.98325	415.7727	63.9265
417.4603	66.5733	417.2228	59.9807	415.8116	63.92584
417.5028	66.57186	417.2674	59.97918	415.8546	63.92563

417.546	66.57256	417.3078	59.97715	415.8966	63.92511
417.5848	66.56848	417.349	59.97579	415.936	63.92414
417.6176	66.56783	417.3914	59.97698	415.9785	63.92285
417.6602	66.56905	417.4316	59.97832	416.0215	63.92245
417.7016	66.56799	417.473	59.97768	416.0601	63.92165
417.7443	66.56869	417.5167	59.97788	416.103	63.92058
417.7842	66.56981	417.5572	59.97537	416.1447	63.91943
417.8238	66.57065	417.597	59.97342	416.1879	63.91706
417.8648	66.56957	417.6328	59.97301	416.229	63.91618
417.9094	66.5642	417.6748	59.97117	416.2696	63.91568
417.9484	66.56042	417.7164	59.97061	416.303	63.91555
417.991	66.55894	417.758	59.96986	416.3412	63.9146
418.0345	66.55902	417.8009	59.96858	416.3845	63.9133
418.0684	66.55891	417.841	59.96709	416.4259	63.91246
418.1074	66.55823	417.8828	59.96693	416.4672	63.91194
418.1493	66.5576	417.9234	59.96522	416.5086	63.91212
418.1936	66.55455	417.9653	59.96315	416.5506	63.91152
418.2342	66.55664	418.0055	59.96141	416.5903	63.91076
418.2739	66.55961	418.0488	59.95914	416.6344	63.9097
418.3159	66.55749	418.0809	59.95752	416.6758	63.90944
418.3574	66.55425	418.1244	59.95611	416.7182	63.90981
418.398	66.55152	418.167	59.95568	416.759	63.90845
418.441	66.54836	418.2072	59.95471	416.7995	63.90707
418.4791	66.54702	418.2496	59.95544	416.8328	63.9055
418.5231	66.54583	418.2914	59.95587	416.875	63.90365
418.5613	66.54659	418.3329	59.95488	416.918	63.90308
418.5954	66.54624	418.3743	59.95534	416.958	63.90296
418.6369	66.54407	418.4162	59.95501	416.9994	63.90198
418.682	66.54595	418.4582	59.95459	417.04	63.90021
418.7217	66.54651	418.4988	59.95563	417.0816	63.89908
418.765	66.54515	418.5319	59.95502	417.125	63.89789
418.8058	66.54401	418.5744	59.95272	417.1687	63.89784
418.8469	66.54218	418.6148	59.95252	417.21	63.89844
418.8875	66.53939	418.6576	59.94934	417.25	63.89853
418.9298	66.53712	418.6994	59.94714	417.2916	63.89882
418.9692	66.5346	418.7398	59.94667	417.3305	63.89822
419.0123	66.53196	418.7825	59.9436	417.3708	63.89739
419.0456	66.52999	418.8239	59.94207	417.4141	63.89584
419.088	66.52823	418.8656	59.94119	417.4498	63.89461
419.1276	66.52773	418.907	59.93876	417.489	63.89379
419.1694	66.52689	418.9488	59.93833	417.5302	63.89243
419.2113	66.52624	418.9813	59.93924	417.5708	63.89105
419.2524	66.5254	419.0228	59.93922	417.6128	63.88991
419.2941	66.52566	419.0663	59.93934	417.655	63.88859
419.3359	66.52463	419.1062	59.94029	417.6922	63.88824
419.3774	66.52186	419.1488	59.9398	417.7382	63.88831
419.4175	66.52008	419.1909	59.93774	417.7759	63.88751
419.459	66.51535	419.232	59.93692	417.8224	63.88685
419.4919	66.51101	419.2746	59.93534	417.862	63.88639
419.5344	66.5063	419.3129	59.93435	417.9045	63.8857
419.5748	66.50416	419.3546	59.93396	417.9459	63.88577
419.616	66.50511	419.396	59.9317	417.9848	63.88551
419.6604	66.50566	419.4266	59.93009	418.0268	63.88442
419.7013	66.50701	419.4713	59.93113	418.0624	63.88372
419.7433	66.50766	419.5146	59.93274	418.1014	63.88196
419.7838	66.50499	419.5536	59.93145	418.1456	63.88086
419.8242	66.50167	419.5948	59.9322	418.1862	63.8798
419.8664	66.49845	419.6359	59.92895	418.2262	63.87872

419.9074	66.49152	419.6812	59.9257	418.268	63.87877
419.9394	66.48632	419.7211	59.92542	418.3094	63.87771
419.9806	66.48395	419.7619	59.92343	418.3524	63.87765
420.022	66.48523	419.805	59.92138	418.3948	63.87704
420.0644	66.48697	419.8454	59.92053	418.4343	63.87627
420.1028	66.48719	419.8809	59.91938	418.4784	63.87501
420.1454	66.48664	419.9196	59.91875	418.5178	63.87359
420.1896	66.48436	419.959	59.91908	418.5566	63.87228
420.2298	66.48287	420.001	59.91753	418.5899	63.87168
420.2719	66.48382	420.0444	59.91576	418.634	63.87116
420.3126	66.48323	420.0863	59.91485	418.6746	63.87017
420.3554	66.48626	420.1272	59.91257	418.717	63.87002
420.3968	66.4859	420.1708	59.91198	418.7586	63.86924
420.4366	66.48373	420.2117	59.91334	418.7992	63.86874
420.4712	66.48565	420.25	59.91133	418.8394	63.86784
420.5116	66.48545	420.2907	59.91065	418.8812	63.86669
420.5529	66.48236	420.327	59.91073	418.9216	63.86493
420.5926	66.48028	420.3683	59.90883	418.966	63.86287
420.635	66.4773	420.4087	59.90758	419.0074	63.86149
420.6778	66.47484	420.4538	59.90615	419.0502	63.8609
420.7205	66.47295	420.4946	59.90478	419.0902	63.86077
420.7634	66.47371	420.535	59.90476	419.1316	63.86108
420.806	66.47377	420.5756	59.903	419.1756	63.86023
420.8492	66.46993	420.6168	59.9023	419.2064	63.85872
420.8882	66.46954	420.6592	59.90136	419.249	63.85665
420.9206	66.469	420.6986	59.89896	419.2911	63.85496
420.9624	66.46837	420.7404	59.89847	419.332	63.85465
421.004	66.46855	420.7742	59.89838	419.3728	63.85447
421.0466	66.46629	420.8144	59.89659	419.4162	63.85417
421.0884	66.46243	420.8562	59.89632	419.4558	63.85366
421.1282	66.46167	420.8978	59.89657	419.4978	63.85195
421.1711	66.45987	420.9412	59.89637	419.5388	63.8503
421.2115	66.45783	420.9834	59.8952	419.5801	63.84901
421.2526	66.45802	421.0235	59.89385	419.6237	63.84723
421.2966	66.45614	421.0648	59.89157	419.6672	63.84758
421.3374	66.45411	421.106	59.89124	419.7068	63.84893
421.3719	66.45176	421.1473	59.89148	419.7475	63.84916
421.4114	66.45131	421.1914	59.88964	419.7885	63.84933
421.4541	66.44858	421.2226	59.8893	419.822	63.84897
421.4952	66.44573	421.2649	59.88715	419.8628	63.84724
421.5352	66.44375	421.3059	59.88345	419.9036	63.84566
421.576	66.44235	421.3466	59.88253	419.946	63.84512
421.6195	66.44315	421.3879	59.88182	419.9863	63.84393
421.6619	66.44126	421.4302	59.88093	420.0307	63.84267
421.7037	66.44035	421.473	59.88018	420.072	63.84168
421.7441	66.44039	421.5138	59.87853	420.1137	63.83977
421.7844	66.43515	421.5556	59.87839	420.1554	63.83826
421.818	66.43124	421.5983	59.87939	420.195	63.83751
421.8575	66.43193	421.6389	59.88004	420.2362	63.83687
421.9006	66.4298	421.672	59.8799	420.2791	63.8377
421.9425	66.42852	421.7128	59.879	420.3205	63.83783
421.9882	66.4305	421.7548	59.87692	420.3536	63.83715
422.0292	66.43098	421.794	59.87502	420.3952	63.83605
422.0694	66.43055	421.837	59.87497	420.437	63.83537
422.1118	66.43133	421.8775	59.87393	420.479	63.83509
422.15	66.42991	421.9247	59.87175	420.518	63.83543
422.194	66.42628	421.9611	59.87109	420.5616	63.83594
422.2386	66.42448	422.0025	59.87016	420.6016	63.83517

422.277	66.42417	422.0439	59.86908	420.6448	63.83457
422.3203	66.42341	422.0863	59.86849	420.6848	63.83372
422.3533	66.42125	422.1209	59.86788	420.7259	63.83338
422.3976	66.41909	422.1619	59.86674	420.7657	63.83202
422.4374	66.41715	422.2041	59.8653	420.8097	63.83044
422.4798	66.41603	422.245	59.86452	420.8506	63.82899
422.5186	66.41778	422.2878	59.86368	420.8918	63.82638
422.562	66.41754	422.33	59.86183	420.935	63.82485
422.6018	66.41702	422.3703	59.86095	420.968	63.82432
422.644	66.41627	422.4117	59.85866	421.0107	63.82358
422.6882	66.41439	422.4544	59.85752	421.0512	63.82222
422.7272	66.41144	422.4956	59.85885	421.0933	63.8217
422.7692	66.40749	422.5358	59.85866	421.1336	63.81956
422.8046	66.40586	422.5689	59.85813	421.1761	63.81812
422.845	66.40491	422.612	59.85729	421.2167	63.81845
422.8862	66.40273	422.6515	59.85464	421.2594	63.81856
422.928	66.40168	422.6928	59.85286	421.2992	63.81838
422.9687	66.40055	422.7344	59.85232	421.3422	63.81731
423.0131	66.39647	422.7769	59.85229	421.3838	63.81565
423.0528	66.3963	422.8172	59.85337	421.4277	63.81373
423.0932	66.39367	422.8596	59.85303	421.4666	63.81277
423.1332	66.38709	422.9006	59.85244	421.5086	63.81269
423.1756	66.38581	422.9426	59.85365	421.5486	63.81243
423.2204	66.38322	422.9828	59.85159	421.5824	63.81207
423.2497	66.38218	423.017	59.84924	421.6256	63.81216
423.2914	66.3821	423.0582	59.84864	421.667	63.81225
423.334	66.38194	423.0985	59.84549	421.7094	63.81091
423.375	66.38084	423.1411	59.84287	421.7504	63.81013
423.4184	66.38144	423.184	59.84237	421.7929	63.8085
423.4596	66.38139	423.2242	59.84084	421.833	63.8062
423.5008	66.38096	423.2646	59.8399	421.874	63.80449
423.5404	66.3828	423.3057	59.83978	421.915	63.80223
423.5818	66.38307	423.349	59.83825	421.9572	63.80168
423.623	66.38057	423.3904	59.83592	421.998	63.8016
423.6659	66.38088	423.4335	59.83474	422.0403	63.80163
423.6972	66.37974	423.4653	59.83397	422.0812	63.8023
423.7384	66.37513	423.5062	59.8344	422.1174	63.8018
423.78	66.37509	423.55	59.83507	422.1588	63.79953
423.8196	66.37519	423.5926	59.83595	422.1998	63.79842
423.861	66.37342	423.6352	59.83351	422.2393	63.79781
423.9033	66.37403	423.6783	59.8314	422.2822	63.79764
423.9454	66.37535	423.7206	59.82911	422.3233	63.79718
423.9879	66.37331	423.7591	59.82605	422.3641	63.79663
424.0271	66.372	423.804	59.82597	422.4064	63.79575
424.0697	66.37116	423.8447	59.82629	422.4473	63.79376
424.1125	66.36642	423.8842	59.82581	422.4878	63.79341
424.1554	66.36436	423.917	59.826	422.5292	63.7923
424.1941	66.36206	423.9586	59.82499	422.5713	63.79071
424.227	66.36027	423.9984	59.82191	422.6111	63.78992
424.2702	66.36101	424.0419	59.82127	422.6549	63.78975
424.3121	66.35763	424.0852	59.82073	422.6956	63.78975
424.353	66.35459	424.1256	59.81925	422.7282	63.79018
424.3938	66.35277	424.1664	59.81984	422.7691	63.79002
424.4379	66.35085	424.2078	59.82029	422.8093	63.7898
424.4804	66.35183	424.2494	59.81799	422.8533	63.78834
424.521	66.3521	424.2902	59.81702	422.8933	63.78669
424.562	66.35178	424.332	59.81545	422.9354	63.78611
424.6031	66.35033	424.3668	59.81441	422.9768	63.78465

424.6451	66.34851	424.407	59.81356	423.0182	63.78409
424.6778	66.3464	424.4474	59.81258	423.0594	63.78345
424.7192	66.34279	424.4892	59.81212	423.1024	63.78187
424.7608	66.34068	424.5294	59.81181	423.1438	63.78055
424.803	66.33875	424.572	59.81087	423.1862	63.77921
424.8433	66.33784	424.614	59.81032	423.2278	63.77807
424.8853	66.33657	424.6566	59.80952	423.2681	63.77717
424.9294	66.33164	424.6969	59.80715	423.3116	63.77543
424.9691	66.33001	424.7388	59.80552	423.343	63.77402
425.0108	66.32814	424.7808	59.80613	423.387	63.77249
425.0537	66.32229	424.8143	59.80598	423.427	63.77198
425.0936	66.32427	424.8556	59.80439	423.47	63.7721
425.1264	66.3259	424.8963	59.80425	423.5104	63.7727
425.1688	66.32568	424.9398	59.80314	423.5522	63.77334
425.208	66.32866	424.9813	59.80184	423.5932	63.77273
425.2493	66.33034	425.0229	59.80051	423.635	63.7716
425.291	66.32835	425.0656	59.8001	423.6768	63.77019
425.334	66.32599	425.1046	59.79746	423.7188	63.76842
425.375	66.32375	425.1454	59.79597	423.7594	63.76744
425.4162	66.32127	425.1897	59.79686	423.8018	63.76661
425.4579	66.31915	425.2294	59.79555	423.8436	63.76605
425.4978	66.31427	425.2641	59.79475	423.8759	63.76619
425.54	66.31314	425.3046	59.79256	423.9176	63.76511
425.5754	66.31268	425.3472	59.78911	423.9614	63.76427
425.617	66.31129	425.3867	59.78783	424.003	63.76406
425.6604	66.31079	425.4297	59.7873	424.0422	63.76238
425.699	66.31061	425.4728	59.78808	424.0846	63.76101
425.7424	66.30641	425.5132	59.78856	424.1266	63.76003
425.7836	66.30264	425.5546	59.78987	424.1676	63.75858
425.8234	66.3001	425.5954	59.78906	424.2101	63.75737
425.866	66.29748	425.6378	59.78633	424.251	63.75711
425.9082	66.297	425.6776	59.78651	424.2944	63.75651
425.9511	66.29433	425.7115	59.78543	424.336	63.75609
425.9916	66.29273	425.7534	59.78364	424.375	63.75657
426.034	66.29316	425.7952	59.78379	424.4164	63.75652
426.0766	66.29327	425.8348	59.78261	424.457	63.75587
426.1098	66.2934	425.8779	59.78162	424.4874	63.75516
426.1496	66.2946	425.9189	59.78083	424.5309	63.75324
426.19	66.29452	425.9606	59.77865	424.5732	63.75152
426.2331	66.29311	426.0019	59.77689	424.6158	63.74999
426.277	66.29222	426.0436	59.77465	424.6598	63.74854
426.318	66.29142	426.0852	59.77278	424.702	63.74715
426.3585	66.29034	426.1243	59.77323	424.7431	63.7456
426.3972	66.28734	426.1594	59.77299	424.7841	63.7451
426.4419	66.28177	426.2006	59.77004	424.8266	63.74524
426.4807	66.27874	426.2432	59.76821	424.8681	63.74617
426.5211	66.27502	426.283	59.7675	424.908	63.74598
426.5557	66.273	426.3232	59.76658	424.9516	63.74512
426.5949	66.27303	426.366	59.76667	424.9948	63.74399
426.6364	66.27378	426.4098	59.76624	425.0346	63.74317
426.6781	66.27432	426.4506	59.76535	425.0754	63.74233
426.7201	66.27406	426.4933	59.76363	425.1072	63.74099
426.7632	66.27601	426.535	59.76189	425.1512	63.73922
426.8045	66.27718	426.577	59.75954	425.1896	63.73738
426.8455	66.27626	426.6104	59.7573	425.2315	63.73601
426.8832	66.27325	426.6506	59.75638	425.273	63.73553
426.9282	66.26896	426.6922	59.75447	425.3131	63.73516
426.9699	66.26523	426.7346	59.75424	425.356	63.73477

427.0031	66.26373	426.776	59.75488	425.3985	63.73464
427.0457	66.2606	426.8178	59.75246	425.4391	63.73378
427.0855	66.25986	426.8589	59.75227	425.4817	63.7335
427.1279	66.25712	426.9002	59.75112	425.5234	63.73228
427.1679	66.2533	426.9424	59.74791	425.5648	63.73085
427.21	66.24863	426.9844	59.74628	425.6065	63.72961
427.25	66.24948	427.0272	59.74414	425.6404	63.72794
427.2928	66.24829	427.058	59.74464	425.6803	63.72704
427.3346	66.24493	427.1002	59.74748	425.7232	63.72619
427.3769	66.24682	427.1419	59.74745	425.7644	63.72602
427.418	66.24531	427.1828	59.74616	425.8053	63.72559
427.4508	66.24399	427.2221	59.74531	425.8474	63.72455
427.4924	66.24355	427.267	59.74238	425.8903	63.72401
427.5332	66.2438	427.3062	59.74141	425.9305	63.72361
427.5762	66.23982	427.3482	59.74051	425.9746	63.72367
427.6174	66.23687	427.3883	59.73815	426.013	63.72286
427.6566	66.23371	427.4322	59.73836	426.0562	63.72137
427.6962	66.22864	427.4722	59.7392	426.0965	63.71996
427.7383	66.22823	427.5078	59.73971	426.1384	63.71795
427.7785	66.2239	427.5472	59.7403	426.1806	63.71752
427.8219	66.2224	427.59	59.73943	426.2224	63.71694
427.8628	66.22442	427.63	59.73733	426.2544	63.71626
427.9039	66.22334	427.6743	59.73601	426.2954	63.71603
427.9467	66.22393	427.715	59.73467	426.3344	63.71514
427.9816	66.22653	427.7578	59.73183	426.378	63.71375
428.0244	66.22745	427.7976	59.73058	426.4195	63.71276
428.0656	66.22486	427.84	59.72914	426.4608	63.71112
428.1093	66.22189	427.882	59.72642	426.5017	63.71032
428.1494	66.21971	427.9238	59.72601	426.5426	63.71037
428.191	66.21791	427.953	59.72615	426.5834	63.70999
428.2325	66.21569	427.994	59.72533	426.6266	63.71024
428.275	66.21651	428.0376	59.72564	426.669	63.70986
428.3154	66.21765	428.0788	59.72496	426.7118	63.7089
428.3576	66.21209	428.1216	59.72298	426.7507	63.70691
428.3994	66.20862	428.1633	59.72228	426.7946	63.70469
428.4334	66.20869	428.2034	59.72131	426.8352	63.70283
428.4738	66.20554	428.2441	59.72015	426.8689	63.7011
428.5158	66.20126	428.2869	59.71913	426.9106	63.70071
428.5568	66.205	428.3281	59.71908	426.953	63.70069
428.5967	66.20575	428.3668	59.7177	426.9922	63.69994
428.6388	66.20335	428.4022	59.71654	427.0342	63.69994
428.6817	66.20171	428.4438	59.7166	427.0768	63.69946
428.7248	66.19862	428.4852	59.71561	427.1171	63.6989
428.765	66.19398	428.5278	59.7148	427.158	63.69863
428.8048	66.19089	428.5668	59.71465	427.1981	63.69702
428.8456	66.18865	428.6094	59.71155	427.2406	63.69566
428.8779	66.18786	428.6516	59.70935	427.2804	63.69408
428.9216	66.19013	428.6933	59.70785	427.3244	63.69253
428.9648	66.18725	428.7347	59.70607	427.3653	63.69139
429.0068	66.18567	428.7776	59.70607	427.3981	63.69053
429.0465	66.18393	428.8209	59.70562	427.4409	63.69057
429.0899	66.17952	428.8519	59.70478	427.4812	63.68998
429.1316	66.17554	428.893	59.70449	427.5234	63.69003
429.1732	66.1772	428.9346	59.70449	427.5665	63.69038
429.2133	66.17685	428.9766	59.70386	427.6076	63.69005
429.2572	66.17258	429.0191	59.70335	427.65	63.69024
429.298	66.17115	429.0608	59.70226	427.6879	63.68989
429.3313	66.17072	429.104	59.70016	427.7307	63.68912

429.3732	66.17029	429.142	59.6988	427.7738	63.68776
429.4145	66.17322	429.1842	59.69886	427.8145	63.68531
429.4558	66.17828	429.228	59.69701	427.8575	63.68424
429.4992	66.17935	429.2694	59.69569	427.8969	63.68338
429.5394	66.17889	429.3006	59.69541	427.9417	63.68289
429.5811	66.17738	429.3438	59.69255	427.984	63.683
429.6256	66.17656	429.3854	59.69253	428.0156	63.68291
429.6661	66.17503	429.4289	59.69282	428.0576	63.68227
429.707	66.17409	429.4687	59.69064	428.0982	63.6813
429.7466	66.17298	429.5098	59.68888	428.139	63.68017
429.788	66.17015	429.5524	59.68752	428.182	63.6784
429.831	66.16802	429.5918	59.68507	428.2238	63.67682
429.8646	66.16702	429.635	59.68403	428.265	63.67558
429.905	66.16302	429.6766	59.68517	428.3075	63.67483
429.944	66.15733	429.7174	59.68415	428.3482	63.67382
429.9889	66.15687	429.7522	59.68386	428.3899	63.67288
430.0294	66.15512	429.7933	59.68226	428.4318	63.67194
430.0706	66.15387	429.8383	59.67871	428.4728	63.67139
430.111	66.1556	429.8782	59.67601	428.516	63.67059
430.1528	66.15647	429.9178	59.67477	428.5544	63.66984
430.1949	66.15518	429.9612	59.67558	428.5969	63.66918
430.2378	66.15125	430.005	59.6776	428.6295	63.66811
430.2774	66.14983	430.0442	59.67758	428.6714	63.66713
430.3102	66.14677	430.0869	59.67563	428.712	63.66662
430.352	66.1409	430.1294	59.67297	428.7544	63.66552
430.3946	66.13494	430.1684	59.67144	428.7958	63.66432
430.437	66.13314	430.1994	59.66999	428.838	63.66326
430.4756	66.12971	430.2426	59.66868	428.8794	63.66214
430.518	66.12737	430.2836	59.66841	428.9214	63.66146
430.559	66.13042	430.3246	59.66704	428.9628	63.66066
430.6006	66.13137	430.3662	59.6668	429.0059	63.66075
430.6454	66.12965	430.4059	59.66718	429.0459	63.66043
430.6855	66.12929	430.4524	59.66672	429.0874	63.66028
430.7261	66.13022	430.4916	59.66295	429.1272	63.66009
430.7613	66.12898	430.5342	59.65949	429.1634	63.65938
430.8028	66.1276	430.5753	59.65728	429.2038	63.65804
430.8434	66.12601	430.6198	59.65606	429.2444	63.65637
430.8829	66.1238	430.6515	59.65662	429.2872	63.65515
430.9256	66.12177	430.6937	59.65687	429.3289	63.65378
430.9672	66.12218	430.7359	59.65527	429.37	63.65302
431.0085	66.12235	430.7752	59.65288	429.4104	63.65196
431.0506	66.12087	430.8201	59.6507	429.4544	63.65097
431.092	66.11766	430.86	59.64852	429.495	63.64944
431.1336	66.11247	430.9	59.6475	429.5352	63.64898
431.1756	66.11181	430.9416	59.64702	429.578	63.64881
431.2076	66.11162	430.9875	59.64687	429.6196	63.64872
431.2472	66.10953	431.0273	59.64785	429.6604	63.6494
431.2907	66.10914	431.0696	59.64752	429.7015	63.64918
431.3324	66.10689	431.1016	59.64702	429.7425	63.64815
431.3753	66.10407	431.1425	59.64668	429.7753	63.64738
431.4187	66.10568	431.1842	59.64651	429.8189	63.64631
431.4586	66.10809	431.2258	59.64696	429.861	63.64398
431.499	66.10782	431.2662	59.64656	429.9024	63.64324
431.5422	66.1084	431.3086	59.64777	429.9428	63.64264
431.5822	66.10715	431.3498	59.64712	429.9851	63.6423
431.6224	66.1039	431.3912	59.64587	430.0269	63.64239
431.6656	66.10225	431.4322	59.64517	430.0672	63.64045
431.705	66.10054	431.4743	59.64184	430.1086	63.63794

431.7385	66.09863	431.5158	59.64005	430.1528	63.63581
431.7782	66.09797	431.5488	59.63821	430.1916	63.63446
431.8191	66.09718	431.5904	59.63487	430.235	63.63505
431.8621	66.09618	431.6318	59.63204	430.2756	63.63645
431.9046	66.09057	431.6734	59.62866	430.3168	63.63592
431.9474	66.08416	431.7114	59.6261	430.3576	63.63546
431.9889	66.08207	431.7552	59.62545	430.3895	63.63494
432.0304	66.08118	431.7979	59.62562	430.4338	63.63341
432.0726	66.08375	431.839	59.62497	430.476	63.63236
432.1132	66.08429	431.8818	59.62555	430.5167	63.63021
432.1554	66.08294	431.9204	59.62564	430.558	63.62876
432.1887	66.07706	431.9604	59.62505	430.599	63.62801
432.23	66.06944	431.9934	59.62399	430.6398	63.62712
432.2739	66.06599	432.0377	59.62209	430.6832	63.62802
432.3117	66.06464	432.0778	59.62021	430.722	63.62715
432.3526	66.06431	432.121	59.61717	430.7657	63.62508
432.3964	66.06199	432.1622	59.61476	430.808	63.62399
432.4371	66.05728	432.2039	59.61313	430.8514	63.62235
432.4782	66.04471	432.245	59.61175	430.8942	63.62152
432.5211	66.0302	432.2852	59.61151	430.924	63.62114
432.5628	66.01827	432.3278	59.6123	430.9681	63.62012
432.6052	66.01285	432.3696	59.61243	431.0084	63.6186
432.6389	66.01441	432.4088	59.61257	431.0506	63.61753
432.68	66.02405	432.4416	59.61207	431.0918	63.61684
432.7207	66.04032	432.484	59.61088	431.1312	63.61668
432.7628	66.05063	432.5282	59.61044	431.1711	63.61648
432.8054	66.05677	432.569	59.60886	431.2146	63.61575
432.8464	66.06155	432.6084	59.60733	431.2556	63.61481
432.8869	66.06072	432.6528	59.60619	431.2965	63.61335
432.9286	66.05738	432.695	59.60491	431.3396	63.61273
432.9698	66.05516	432.7366	59.60483	431.3795	63.61104
433.0131	66.05099	432.777	59.60538	431.4201	63.60953
433.0559	66.04785	432.8196	59.60567	431.4631	63.60847
433.0874	66.04786	432.8622	59.60521	431.5059	63.60761
433.1264	66.04916	432.8928	59.60446	431.539	63.60825
433.1662	66.04942	432.936	59.60492	431.5797	63.60807
433.2119	66.04549	432.9764	59.60408	431.621	63.60795
433.2534	66.04508	433.0205	59.60495	431.6623	63.60728
433.2943	66.04425	433.0616	59.60548	431.703	63.60527
433.3363	66.04322	433.1039	59.60383	431.7459	63.60474
433.3754	66.04473	433.1453	59.60362	431.7875	63.60342
433.4175	66.04216	433.1839	59.60133	431.8282	63.60283
433.4636	66.03496	433.2252	59.59941	431.8706	63.60269
433.5011	66.02805	433.2672	59.59688	431.9134	63.601
433.5448	66.02315	433.3102	59.59369	431.9558	63.6002
433.5859	66.01827	433.3443	59.59113	431.9966	63.59931
433.6184	66.01827	433.3828	59.58817	432.0389	63.59856
433.6609	66.01973	433.4257	59.58699	432.0782	63.5984
433.7025	66.01881	433.4678	59.58723	432.1196	63.59818
433.7442	66.01575	433.5092	59.58612	432.1534	63.59782
433.7842	66.01397	433.552	59.58553	432.1953	63.59685
433.827	66.01368	433.5928	59.58539	432.2369	63.5963
433.865	66.01291	433.6336	59.58449	432.2781	63.59514
433.9082	66.01419	433.6742	59.58546	432.3197	63.59349
433.9483	66.01412	433.7168	59.58529	432.3634	63.59186
433.9929	66.01093	433.7586	59.58276	432.4034	63.59031
434.0324	66.00782	433.7922	59.58072	432.4448	63.58949
434.0652	66.00941	433.8328	59.57733	432.4866	63.58822

434.1063	66.01066	433.8711	59.57555	432.5275	63.5875
434.148	66.00975	433.9172	59.57446	432.5704	63.58639
434.1917	66.00582	433.9567	59.57467	432.6117	63.58499
434.232	66.00554	433.9989	59.57583	432.6513	63.58463
434.2754	66.00491	434.0403	59.57533	432.6825	63.5845
434.3194	66.00445	434.0832	59.57583	432.7235	63.58396
434.3576	66.01124	434.1228	59.57641	432.7668	63.58365
434.3984	66.00843	434.1625	59.57413	432.8069	63.58285
434.4409	66.00521	434.2063	59.57298	432.849	63.58156
434.48	66.00411	434.2402	59.57194	432.892	63.58069
434.5132	66.00094	434.2814	59.56907	432.9344	63.57987
434.556	66.00081	434.3219	59.56917	432.9748	63.57872
434.598	66.00149	434.3654	59.56796	433.0169	63.57715
434.639	65.99979	434.4065	59.56631	433.0606	63.57557
434.6812	65.99512	434.4491	59.56412	433.1012	63.57513
434.7239	65.99333	434.493	59.56124	433.1425	63.57619
434.765	65.99105	434.5321	59.55957	433.1817	63.57693
434.8074	65.98932	434.5744	59.5587	433.2246	63.57633
434.8484	65.98886	434.6152	59.55833	433.2678	63.57515
434.889	65.98528	434.6592	59.55776	433.2971	63.57357
434.9308	65.98197	434.69	59.55749	433.342	63.57183
434.9648	65.97953	434.7324	59.55557	433.384	63.5701
435.0039	65.9776	434.7685	59.55327	433.426	63.56822
435.048	65.97864	434.8133	59.5515	433.4658	63.56644
435.0894	65.97975	434.8544	59.55172	433.5076	63.56532
435.128	65.97664	434.897	59.55227	433.5494	63.56543
435.1718	65.97671	434.9394	59.55281	433.5904	63.56527
435.2134	65.97569	434.9797	59.5524	433.6318	63.56486
435.2556	65.97046	435.024	59.54925	433.6711	63.56391
435.2981	65.9699	435.0661	59.54631	433.7154	63.56273
435.3404	65.97051	435.1066	59.54377	433.7559	63.56127
435.3828	65.96849	435.1402	59.54191	433.7963	63.55965
435.4228	65.96809	435.1821	59.5411	433.8391	63.55909
435.4654	65.96672	435.2204	59.54037	433.8806	63.5588
435.4959	65.96601	435.2636	59.53994	433.9144	63.55851
435.5383	65.96495	435.3063	59.53981	433.9559	63.55795
435.5784	65.96503	435.3474	59.5393	433.9959	63.5571
435.6202	65.96537	435.3895	59.53807	434.0385	63.55627
435.6626	65.96465	435.4295	59.537	434.0812	63.55504
435.7056	65.96377	435.4721	59.53644	434.1202	63.55391
435.7472	65.96071	435.5118	59.53586	434.1609	63.5532
435.7904	65.95902	435.5572	59.53556	434.2066	63.5515
435.8266	65.95773	435.5896	59.53521	434.2456	63.55049
435.8709	65.95432	435.6302	59.53492	434.2872	63.55109
435.9146	65.95408	435.6714	59.53318	434.3281	63.55017
435.9467	65.95382	435.7136	59.53286	434.37	63.54986
435.9887	65.95292	435.7559	59.53218	434.412	63.54899
436.0274	65.94865	435.796	59.53003	434.4438	63.54738
436.0674	65.9453	435.8374	59.52951	434.4854	63.5468
436.1107	65.94435	435.8776	59.52771	434.5261	63.54597
436.152	65.94136	435.9192	59.52577	434.5685	63.54503
436.1953	65.9413	435.9636	59.52503	434.6085	63.54449
436.2359	65.94253	436.0044	59.5241	434.6512	63.54313
436.2782	65.94035	436.0398	59.52361	434.6929	63.54208
436.32	65.93811	436.079	59.52502	434.7366	63.54175
436.3613	65.9395	436.1234	59.52507	434.7766	63.54093
436.3937	65.93665	436.164	59.52492	434.8174	63.53993
436.4342	65.93298	436.2044	59.52559	434.8587	63.53891

436.474	65.92708	436.2453	59.52531	434.9012	63.5374
436.5172	65.92145	436.2866	59.52443	434.9446	63.53635
436.56	65.92076	436.3296	59.52445	434.985	63.53585
436.6013	65.9214	436.37	59.52347	435.027	63.53492
436.6413	65.92526	436.4094	59.52113	435.0606	63.53477
436.6839	65.92804	436.4516	59.51836	435.1012	63.53387
436.726	65.928	436.4843	59.5171	435.1417	63.53318
436.7656	65.92698	436.5262	59.51479	435.1824	63.53249
436.807	65.9265	436.5676	59.51301	435.225	63.5316
436.8418	65.92475	436.6085	59.51169	435.2684	63.5301
436.883	65.92259	436.651	59.50957	435.309	63.52854
436.9242	65.91789	436.693	59.50849	435.3496	63.52713
436.9678	65.91669	436.7361	59.50804	435.394	63.52572
437.0111	65.91702	436.777	59.50897	435.4328	63.52538
437.0533	65.91528	436.8167	59.50961	435.4752	63.52477
437.094	65.91652	436.859	59.50832	435.5158	63.52395
437.1358	65.91535	436.9006	59.50731	435.5567	63.5232
437.1778	65.91073	436.933	59.50622	435.6006	63.52241
437.2178	65.9073	436.9746	59.50415	435.641	63.52176
437.2606	65.90541	437.0172	59.50163	435.6758	63.52136
437.3019	65.90538	437.0562	59.49868	435.7156	63.52048
437.3434	65.9065	437.0992	59.49628	435.7553	63.51873
437.3778	65.90586	437.1426	59.49493	435.7998	63.51728
437.4172	65.90521	437.183	59.4951	435.844	63.5162
437.4612	65.90387	437.2246	59.49597	435.8852	63.51579
437.5004	65.90211	437.2649	59.49534	435.927	63.51584
437.5433	65.90107	437.3072	59.49432	435.966	63.5151
437.5832	65.89922	437.3472	59.49212	436.0092	63.51435
437.627	65.89726	437.3818	59.49112	436.0504	63.51395
437.6683	65.89545	437.4232	59.49103	436.0916	63.51304
437.7108	65.89412	437.4646	59.4898	436.1318	63.51202
437.7518	65.89369	437.5074	59.48868	436.1758	63.51132
437.7926	65.8922	437.5493	59.48812	436.2092	63.51004
437.825	65.89068	437.5894	59.48648	436.2486	63.5092
437.8679	65.88866	437.6313	59.48588	436.2913	63.50916
437.9094	65.88724	437.6719	59.48624	436.3326	63.50822
437.9492	65.88479	437.7156	59.4845	436.3726	63.50805
437.994	65.87983	437.7574	59.48246	436.414	63.50735
438.0332	65.87995	437.7958	59.48123	436.459	63.50644
438.0746	65.87957	437.83	59.47938	436.5006	63.50546
438.118	65.87748	437.8728	59.47888	436.5389	63.50398
438.1606	65.8783	437.9135	59.47912	436.5816	63.50319
438.2004	65.87463	437.9522	59.47847	436.6236	63.50247
438.243	65.87177	437.9989	59.47874	436.6613	63.50185
438.2773	65.87057	438.0395	59.47774	436.7042	63.50098
438.316	65.87069	438.0808	59.4766	436.7456	63.50018
438.3585	65.87154	438.1219	59.47489	436.7872	63.4985
438.398	65.87046	438.1652	59.47312	436.8197	63.49742
438.4416	65.86837	438.2062	59.4709	436.8633	63.49646
438.484	65.86742	438.2464	59.46881	436.9054	63.49571
438.523	65.86533	438.2798	59.46837	436.9476	63.4959
438.5633	65.86342	438.3212	59.46794	436.9886	63.49491
438.6071	65.86265	438.3614	59.46779	437.0309	63.49393
438.6484	65.86143	438.4046	59.46629	437.0716	63.49231
438.6919	65.85994	438.4477	59.4644	437.1125	63.49087
438.724	65.85936	438.488	59.46269	437.1562	63.49051
438.7654	65.85831	438.5314	59.46223	437.1968	63.48975
438.8064	65.85525	438.5703	59.46177	437.2386	63.48906

438.8486	65.84996	438.61	59.46152	437.278	63.48806
438.8887	65.84742	438.6527	59.46085	437.3216	63.48682
438.9287	65.84585	438.6947	59.45816	437.363	63.48569
438.9726	65.84579	438.7271	59.45679	437.407	63.48508
439.0128	65.84857	438.7706	59.45732	437.4354	63.4848
439.0542	65.85025	438.8114	59.45671	437.48	63.48399
439.0961	65.84932	438.8525	59.45585	437.5196	63.48371
439.1371	65.84521	438.8948	59.45523	437.5636	63.48267
439.1786	65.84311	438.9365	59.45435	437.605	63.48094
439.2224	65.83812	438.978	59.45385	437.645	63.47973
439.2534	65.83573	439.0201	59.45387	437.6866	63.47839
439.2966	65.83633	439.0624	59.45344	437.7286	63.47777
439.3391	65.83429	439.1014	59.44946	437.77	63.47682
439.3796	65.83531	439.144	59.44562	437.8134	63.47604
439.4216	65.83654	439.1767	59.44291	437.8566	63.47524
439.461	65.83257	439.22	59.44084	437.8978	63.47453
439.5033	65.83202	439.2597	59.44123	437.9377	63.47407
439.5428	65.8307	439.3022	59.44243	437.9738	63.47307
439.5854	65.82903	439.346	59.44298	438.0146	63.47171
439.6269	65.82838	439.384	59.44278	438.0575	63.47046
439.6662	65.82736	439.4272	59.44062	438.0979	63.4689
439.7004	65.82518	439.4688	59.43821	438.1406	63.46801
439.7444	65.82141	439.5098	59.4371	438.1796	63.46802
439.7862	65.81813	439.5504	59.43675	438.2196	63.46653
439.8261	65.81672	439.5904	59.43555	438.263	63.46604
439.8686	65.81428	439.6261	59.43403	438.307	63.46571
439.9089	65.81349	439.6678	59.43306	438.3481	63.4643
439.9502	65.81431	439.7092	59.43107	438.3906	63.46375
439.9904	65.81208	439.752	59.43093	438.4316	63.46304
440.032	65.81194	439.7916	59.43154	438.4729	63.46207
440.0728	65.80878	439.8337	59.4306	438.5124	63.46149
440.1146	65.80759	439.8766	59.42969	438.5554	63.46111
440.1503	65.80793	439.9162	59.42872	438.5864	63.4602
440.1907	65.80656	439.9576	59.42724	438.6309	63.45955
440.2333	65.8069	439.9986	59.42616	438.6728	63.45878
440.2744	65.80618	440.0406	59.42541	438.7116	63.45717
440.316	65.80247	440.0734	59.42606	438.7537	63.4559
440.3588	65.79844	440.1174	59.42546	438.7942	63.45444
440.3996	65.79669	440.16	59.42565	438.8346	63.45339
440.4416	65.79516	440.2012	59.42531	438.8738	63.45245
440.4844	65.7939	440.2424	59.42229	438.918	63.45161
440.5278	65.79171	440.2825	59.42047	438.96	63.45164
440.565	65.78975	440.3252	59.41793	439.0019	63.45124
440.598	65.78932	440.3684	59.41577	439.043	63.45081
440.6406	65.78831	440.4094	59.4138	439.0846	63.45084
440.684	65.78769	440.45	59.41307	439.1248	63.45029
440.7254	65.78781	440.4903	59.41151	439.1644	63.44965
440.7672	65.78675	440.5268	59.41055	439.1996	63.44962
440.8091	65.78645	440.568	59.41139	439.243	63.44911
440.85	65.78723	440.6075	59.41184	439.2836	63.44761
440.8916	65.7854	440.6508	59.41199	439.3277	63.44558
440.9353	65.78183	440.6898	59.41176	439.3661	63.44406
440.9759	65.78036	440.7326	59.40955	439.4086	63.44243
441.0178	65.77901	440.776	59.40586	439.4511	63.44129
441.0572	65.77649	440.815	59.40328	439.491	63.44059
441.098	65.77465	440.8582	59.40021	439.5334	63.43903
441.1321	65.77356	440.898	59.39781	439.5741	63.43733
441.1756	65.77184	440.9406	59.39692	439.6152	63.43564

441.2175	65.76895	440.9735	59.39546	439.6602	63.43431
441.2585	65.76812	441.0158	59.39404	439.7012	63.43399
441.2993	65.76699	441.0596	59.39434	439.7347	63.43337
441.3409	65.76488	441.102	59.3945	439.7776	63.43372
441.3838	65.76324	441.1417	59.39452	439.8171	63.43452
441.4254	65.76323	441.1825	59.39582	439.8592	63.43385
441.468	65.76271	441.2246	59.39536	439.8993	63.43346
441.5084	65.76182	441.2658	59.39423	439.9402	63.43215
441.5482	65.76217	441.3072	59.39399	439.9825	63.42965
441.5817	65.76087	441.3492	59.39254	440.0226	63.42879
441.6234	65.75628	441.3899	59.39087	440.0676	63.42817
441.6646	65.74887	441.4239	59.38985	440.1068	63.42704
441.7054	65.74579	441.4636	59.38832	440.149	63.42684
441.7456	65.74473	441.5072	59.38745	440.191	63.42541
441.7885	65.74607	441.5472	59.38665	440.2342	63.42414
441.8334	65.75067	441.5902	59.38541	440.2739	63.42307
441.8724	65.75154	441.6317	59.38392	440.3176	63.4222
441.9146	65.75083	441.6722	59.38227	440.35	63.42214
441.9576	65.74899	441.713	59.38064	440.3912	63.42188
442.0016	65.74532	441.7554	59.379	440.4328	63.42138
442.0324	65.74453	441.7964	59.37844	440.4752	63.42084
442.0753	65.74346	441.8398	59.37731	440.5165	63.41992
442.1167	65.74043	441.8734	59.37581	440.5578	63.41875
442.1598	65.73804	441.9157	59.37511	440.6012	63.41803
442.2018	65.73623	441.9549	59.37459	440.6424	63.41627
442.2422	65.73534	441.9966	59.37324	440.6838	63.41472
442.2846	65.73415	442.0412	59.37391	440.726	63.41295
442.3254	65.73476	442.0806	59.37378	440.7658	63.4109
442.367	65.73421	442.1197	59.37231	440.8082	63.40981
442.4087	65.73037	442.1631	59.37082	440.8504	63.40862
442.4512	65.72822	442.2026	59.36782	440.8918	63.40736
442.4822	65.72547	442.2445	59.36593	440.932	63.40672
442.5232	65.7229	442.2864	59.3646	440.9648	63.40707
442.564	65.72128	442.3202	59.36341	441.007	63.40617
442.607	65.71758	442.3628	59.36354	441.0497	63.40595
442.6462	65.7173	442.4038	59.36271	441.0902	63.40572
442.6891	65.71637	442.4446	59.36089	441.1315	63.40452
442.7294	65.71498	442.4855	59.35995	441.1719	63.40391
442.7722	65.71533	442.5288	59.35779	441.2144	63.40315
442.8152	65.71304	442.5707	59.35538	441.2541	63.40229
442.8553	65.70873	442.6116	59.35491	441.2949	63.40241
442.8971	65.70476	442.6558	59.35337	441.3398	63.40171
442.9404	65.70448	442.6967	59.3525	441.3794	63.40088
442.9787	65.7053	442.7368	59.35245	441.419	63.40055
443.0133	65.70568	442.7707	59.35124	441.4624	63.39903
443.0533	65.70663	442.8113	59.35071	441.4935	63.39808
443.095	65.70307	442.8528	59.35083	441.5356	63.39764
443.1376	65.70042	442.895	59.35101	441.5788	63.39628
443.1806	65.69581	442.9372	59.35032	441.6202	63.39538
443.223	65.69609	442.9787	59.34921	441.6597	63.39447
443.2626	65.69617	443.0196	59.34718	441.7011	63.39359
443.306	65.69357	443.0601	59.34522	441.7428	63.393
443.346	65.69429	443.1023	59.34461	441.7852	63.39194
443.387	65.69274	443.145	59.34318	441.8231	63.39102
443.4285	65.6913	443.184	59.34164	441.8702	63.38929
443.4638	65.69043	443.2208	59.34021	441.91	63.3874
443.5047	65.69041	443.2613	59.33715	441.9528	63.38657
443.5474	65.68889	443.3024	59.33535	441.9942	63.38556

443.5889	65.68809	443.3446	59.33354	442.0344	63.3849
443.6306	65.68809	443.3859	59.33212	442.0776	63.38406
443.671	65.68733	443.4264	59.33195	442.1088	63.38294
443.713	65.68656	443.4669	59.33227	442.15	63.38198
443.752	65.68433	443.5105	59.33265	442.1924	63.38116
443.7959	65.68117	443.5506	59.33241	442.233	63.38108
443.8374	65.67896	443.593	59.33209	442.2758	63.38042
443.8783	65.67736	443.6342	59.33052	442.3175	63.38
443.9102	65.67508	443.6666	59.32879	442.3592	63.37887
443.9516	65.67569	443.7092	59.32756	442.3993	63.37774
443.9956	65.67539	443.7498	59.32623	442.4424	63.37722
444.0375	65.67296	443.7919	59.32447	442.484	63.37596
444.0774	65.67097	443.8344	59.32343	442.524	63.3753
444.12	65.66989	443.8747	59.32183	442.5662	63.37437
444.1614	65.66789	443.9168	59.31999	442.6103	63.37252
444.2034	65.66589	443.9551	59.32082	442.6504	63.37098
444.2446	65.66404	443.9969	59.32167	442.6933	63.36945
444.287	65.66178	444.0392	59.32077	442.7254	63.36868
444.33	65.65952	444.0808	59.32035	442.7668	63.36838
444.36	65.6593	444.1153	59.31887	442.8075	63.36845
444.4027	65.65951	444.1558	59.31693	442.8494	63.36887
444.4422	65.65831	444.1992	59.31658	442.8922	63.36821
444.4855	65.65715	444.2402	59.31558	442.9332	63.36684
444.527	65.65537	444.2798	59.31326	442.9746	63.36502
444.5684	65.65173	444.3219	59.3112	443.0162	63.36249
444.608	65.64934	444.3638	59.30953	443.0583	63.36086
444.6507	65.64802	444.4042	59.30951	443.0983	63.35955
444.6921	65.64512	444.4477	59.30957	443.139	63.35839
444.7336	65.64601	444.4898	59.30912	443.1816	63.35757
444.7756	65.64496	444.5302	59.30776	443.223	63.35671
444.8163	65.64311	444.5648	59.30621	443.255	63.35615
444.8578	65.64294	444.6068	59.30575	443.2972	63.35494
444.8904	65.64083	444.6497	59.30378	443.3408	63.35459
444.933	65.63803	444.6898	59.30323	443.3819	63.35391
444.9754	65.63719	444.7334	59.30235	443.4242	63.35371
445.0164	65.63472	444.7718	59.30021	443.4652	63.35381
445.0596	65.63225	444.8135	59.29926	443.5062	63.35357
445.1	65.63324	444.856	59.29842	443.5458	63.35323
445.1433	65.63205	444.8974	59.29624	443.5883	63.35213
445.1817	65.62957	444.9389	59.29588	443.6306	63.35049
445.2236	65.62885	444.9794	59.29529	443.6723	63.34871
445.267	65.62347	445.0133	59.29394	443.7133	63.34707
445.3109	65.61971	445.0549	59.29342	443.7567	63.34497
445.3404	65.61807	445.098	59.29283	443.7972	63.34411
445.384	65.61433	445.1384	59.2921	443.8378	63.34281
445.4252	65.61259	445.1809	59.29171	443.8686	63.34236
445.4674	65.6129	445.2224	59.29054	443.9125	63.34275
445.5058	65.61284	445.2656	59.28749	443.9544	63.34261
445.5506	65.61258	445.3067	59.2854	443.997	63.34302
445.5899	65.61109	445.3472	59.28393	444.0355	63.34252
445.6313	65.60916	445.3876	59.28264	444.0788	63.34089
445.6728	65.60746	445.4278	59.28256	444.1214	63.33932
445.7143	65.60744	445.4628	59.28227	444.1627	63.338
445.7559	65.60833	445.5041	59.28121	444.2034	63.3366
445.789	65.60681	445.546	59.28063	444.244	63.3361
445.8296	65.60372	445.587	59.27974	444.2856	63.33591
445.8728	65.60231	445.6292	59.27857	444.3258	63.3346
445.9135	65.60225	445.6711	59.27748	444.3674	63.33368

445.9559	65.60203	445.711	59.27702	444.4102	63.33219
445.998	65.60163	445.7532	59.27649	444.4528	63.33063
446.0383	65.60129	445.7937	59.27482	444.4834	63.33024
446.08	65.59868	445.8372	59.27308	444.5264	63.329
446.121	65.59566	445.88	59.27058	444.5676	63.32843
446.1632	65.59141	445.9113	59.26901	444.6094	63.32783
446.2039	65.58869	445.954	59.26676	444.649	63.32679
446.237	65.58798	445.9942	59.26396	444.6919	63.32535
446.2803	65.58531	446.036	59.26142	444.7324	63.32428
446.3209	65.58625	446.0764	59.26063	444.7744	63.32246
446.3632	65.58617	446.1184	59.26115	444.8153	63.32132
446.4033	65.58381	446.1612	59.26264	444.859	63.32023
446.4466	65.58473	446.2019	59.26366	444.899	63.31854
446.4894	65.58364	446.2433	59.26238	444.939	63.31802
446.5304	65.58053	446.285	59.26046	444.984	63.31648
446.5726	65.57703	446.3268	59.25865	445.0168	63.31594
446.6122	65.57452	446.3591	59.25688	445.059	63.3149
446.6544	65.57365	446.4002	59.25605	445.1012	63.31363
446.6946	65.57167	446.4426	59.25429	445.1422	63.3122
446.7378	65.57284	446.4861	59.25163	445.183	63.31099
446.7725	65.57298	446.526	59.25003	445.2248	63.31018
446.812	65.57131	446.5678	59.24766	445.2687	63.30938
446.8528	65.57105	446.6096	59.24651	445.3067	63.30915
446.8948	65.56966	446.6494	59.24679	445.3489	63.30849
446.9391	65.56742	446.691	59.24722	445.3922	63.30814
446.9804	65.56589	446.7348	59.24744	445.4312	63.30769
447.0213	65.56292	446.7746	59.24675	445.472	63.30692
447.061	65.56021	446.8092	59.24565	445.5141	63.30641
447.1031	65.55714	446.8496	59.24328	445.5564	63.30515
447.1444	65.55353	446.8933	59.24022	445.6004	63.30385
447.1848	65.55368	446.9355	59.23733	445.6334	63.3025
447.2156	65.55327	446.9788	59.23573	445.6754	63.30165
447.2612	65.55378	447.0184	59.23431	445.7146	63.30064
447.303	65.55345	447.0578	59.23311	445.7558	63.29985
447.3418	65.55119	447.1016	59.2329	445.7969	63.29887
447.3847	65.54686	447.1404	59.23185	445.8406	63.29765
447.4278	65.54519	447.1847	59.23105	445.882	63.29612
447.4703	65.54382	447.2246	59.23063	445.9239	63.29517
447.5118	65.54348	447.2588	59.22963	445.9644	63.29416
447.5516	65.54449	447.3002	59.22827	446.0066	63.29297
447.5948	65.54136	447.342	59.22638	446.0507	63.29236
447.6354	65.54062	447.3854	59.22457	446.0895	63.29118
447.6682	65.53962	447.4264	59.2231	446.1316	63.28987
447.7111	65.53821	447.4666	59.22317	446.1742	63.28913
447.7522	65.53769	447.5066	59.22307	446.2152	63.28833
447.795	65.5361	447.55	59.2221	446.2496	63.28751
447.8364	65.5331	447.5899	59.22207	446.29	63.2875
447.879	65.53025	447.6334	59.22021	446.33	63.28669
447.919	65.52672	447.6746	59.21808	446.3747	63.28565
447.9618	65.52561	447.7097	59.21698	446.4148	63.28458
448.0017	65.5264	447.7506	59.21486	446.4567	63.28338
448.044	65.52587	447.7916	59.2136	446.498	63.28238
448.0844	65.5267	447.8338	59.21244	446.5378	63.28134
448.119	65.52586	447.8739	59.21205	446.582	63.28037
448.1613	65.52262	447.9168	59.21034	446.6238	63.27941
448.204	65.52027	447.9563	59.20895	446.6652	63.27798
448.2459	65.51886	448.0013	59.20789	446.7074	63.27687
448.2884	65.51693	448.044	59.20631	446.7494	63.27559

448.3284	65.51621	448.0838	59.20693	446.7829	63.27431
448.3689	65.51487	448.1264	59.20629	446.8258	63.27351
448.4134	65.51337	448.1601	59.2056	446.8683	63.27299
448.4524	65.51129	448.2012	59.20486	446.9091	63.27216
448.4962	65.5101	448.2432	59.20446	446.948	63.27117
448.5366	65.50895	448.2824	59.20343	446.9912	63.27012
448.579	65.50749	448.3256	59.20364	447.0322	63.26897
448.6187	65.50638	448.3668	59.2029	447.0731	63.2681
448.6518	65.50369	448.4091	59.20049	447.1142	63.26738
448.6947	65.50098	448.449	59.19894	447.157	63.2666
448.7344	65.49871	448.4928	59.19787	447.1981	63.26527
448.7784	65.49783	448.535	59.19501	447.2388	63.26405
448.82	65.49784	448.5761	59.19347	447.2809	63.26338
448.86	65.49684	448.6102	59.19283	447.3215	63.26181
448.9	65.49496	448.6498	59.1899	447.365	63.26139
448.9443	65.49259	448.6914	59.18879	447.3988	63.26039
448.9851	65.49006	448.7316	59.18784	447.4384	63.25901
449.0261	65.48789	448.7744	59.18597	447.4822	63.25835
449.0679	65.48595	448.8156	59.18476	447.5234	63.25691
449.1016	65.48413	448.8567	59.18358	447.5652	63.25619
449.1435	65.48283	448.8972	59.18288	447.6054	63.25535
449.1846	65.48188	448.9381	59.18202	447.6484	63.25458
449.2262	65.48154	448.98	59.18102	447.689	63.254
449.2691	65.48037	449.0216	59.18017	447.7314	63.25347
449.3094	65.47869	449.0556	59.17889	447.773	63.25293
449.3508	65.477	449.098	59.17804	447.8133	63.25256
449.3934	65.47499	449.1398	59.17549	447.8543	63.25142
449.4336	65.4738	449.1812	59.17341	447.895	63.25025
449.475	65.47256	449.2193	59.17236	447.937	63.24867
449.5147	65.46806	449.2621	59.17136	447.9788	63.24659
449.5496	65.46448	449.3063	59.17204	448.011	63.24577
449.59	65.46411	449.3478	59.17267	448.0537	63.24455
449.632	65.4631	449.3882	59.17142	448.0948	63.2429
449.6744	65.46294	449.4278	59.17097	448.1368	63.24167
449.7166	65.46201	449.4707	59.1699	448.177	63.24077
449.7604	65.46013	449.5041	59.16864	448.2206	63.23949
449.8017	65.45866	449.5463	59.16838	448.2586	63.23964
449.8408	65.45806	449.5884	59.16693	448.3036	63.23894
449.8847	65.46013	449.6283	59.16593	448.345	63.23728
449.9258	65.45908	449.671	59.16499	448.3858	63.23581
449.9688	65.45543	449.7115	59.1641	448.4263	63.23405
449.9998	65.45112	449.756	59.16265	448.4689	63.23247
450.0417	65.44766	449.7962	59.16047	448.5105	63.23148
450.0832	65.44565	449.8363	59.1594	448.5437	63.23093
450.125	65.44484	449.8808	59.15851	448.586	63.22997
450.166	65.44702	449.9196	59.15749	448.627	63.22973
450.2084	65.44685	449.9528	59.15629	448.6691	63.22926
450.2484	65.444	449.9934	59.15501	448.7102	63.22865
450.29	65.44004	450.0354	59.15239	448.7512	63.22739
450.3341	65.43706	450.078	59.1508	448.7934	63.22604
450.3769	65.43437	450.1191	59.15003	448.8316	63.22473
450.4174	65.43077	450.1611	59.14853	448.8754	63.22352
450.4578	65.43059	450.204	59.14773	448.9185	63.22285
450.5002	65.42844	450.2434	59.1458	448.9602	63.22205
450.5318	65.4263	450.2881	59.14495	448.9998	63.22139
450.5756	65.42767	450.33	59.14508	449.0414	63.22081
450.6158	65.42574	450.3707	59.14495	449.0824	63.22001
450.6576	65.42388	450.4033	59.14551	449.1261	63.21896

450.6996	65.42188	450.4461	59.14524	449.157	63.21819
450.7408	65.41903	450.4883	59.14384	449.199	63.2171
450.7839	65.41862	450.5289	59.14144	449.2416	63.21598
450.8264	65.4182	450.5705	59.13857	449.2831	63.21518
450.8638	65.41603	450.6109	59.13696	449.3234	63.21387
450.9054	65.4152	450.6514	59.1349	449.3644	63.21249
450.9462	65.41389	450.6953	59.13384	449.4071	63.2117
450.9812	65.41203	450.735	59.13251	449.4494	63.21069
451.0218	65.41255	450.7791	59.13077	449.489	63.21001
451.0648	65.41246	450.8211	59.12905	449.5313	63.20896
451.1034	65.41013	450.8516	59.12805	449.5744	63.20762
451.1444	65.40652	450.8941	59.12779	449.6158	63.20647
451.1886	65.40522	450.9366	59.1274	449.6574	63.20571
451.2296	65.40304	450.9774	59.12677	449.6988	63.20515
451.2711	65.40031	451.0194	59.12694	449.7393	63.2046
451.313	65.39781	451.0588	59.12536	449.7722	63.20422
451.3549	65.39666	451.1033	59.12485	449.8138	63.20383
451.398	65.39509	451.1431	59.12389	449.8548	63.20317
451.4306	65.3931	451.1856	59.12215	449.8979	63.20189
451.4704	65.39403	451.2276	59.12164	449.9416	63.20056
451.5122	65.39278	451.2668	59.11998	449.9794	63.19858
451.5534	65.39196	451.3	59.11852	450.02	63.19717
451.594	65.39149	451.3442	59.11721	450.0626	63.19616
451.6359	65.39066	451.3844	59.11636	450.106	63.19542
451.6788	65.38843	451.426	59.11375	450.1466	63.19454
451.7212	65.38607	451.4653	59.11207	450.187	63.19381
451.762	65.38274	451.51	59.11104	450.229	63.19269
451.8026	65.38011	451.5513	59.10819	450.2723	63.19119
451.8429	65.37631	451.5922	59.10784	450.3066	63.19026
451.877	65.37027	451.6322	59.10799	450.3477	63.18862
451.9207	65.36483	451.6739	59.10701	450.3884	63.18717
451.9598	65.36431	451.7164	59.10725	450.4309	63.18594
452.0029	65.36468	451.7512	59.10682	450.47	63.1853
452.044	65.3669	451.7925	59.10415	450.5121	63.18437
452.0856	65.36815	451.8332	59.10186	450.5546	63.18376
452.128	65.36256	451.8757	59.09987	450.596	63.18263
452.169	65.35837	451.9156	59.09685	450.6392	63.18148
452.209	65.35829	451.9561	59.09637	450.681	63.18032
452.2508	65.35751	451.9994	59.09575	450.7197	63.17933
452.2916	65.36067	452.0426	59.09508	450.7637	63.17827
452.3331	65.36242	452.0824	59.09569	450.8028	63.17687
452.3772	65.36155	452.1254	59.09395	450.8444	63.17578
452.4107	65.36235	452.1648	59.09213	450.8861	63.17465
452.4528	65.36201	452.1984	59.09114	450.9187	63.17397
452.4938	65.36012	452.24	59.08954	450.9602	63.17313
452.5342	65.35709	452.2822	59.08844	451.0032	63.17211
452.5762	65.35504	452.3221	59.08791	451.0441	63.17036
452.6179	65.35262	452.3638	59.0869	451.0854	63.16916
452.6602	65.35027	452.4084	59.08504	451.1259	63.16847
452.701	65.3474	452.448	59.08378	451.1687	63.1682
452.7446	65.34467	452.4892	59.08257	451.2084	63.16813
452.7826	65.34266	452.5313	59.08094	451.25	63.16779
452.8248	65.33783	452.5718	59.08032	451.2918	63.16686
452.8594	65.33731	452.6128	59.07918	451.3362	63.16569
452.8994	65.33694	452.6469	59.07772	451.378	63.16406
452.9398	65.33657	452.6866	59.07672	451.4176	63.16238
452.9816	65.33833	452.7302	59.07557	451.4616	63.1608
453.0208	65.33835	452.77	59.07451	451.5029	63.15905

453.0648	65.33493	452.8124	59.07376	451.5337	63.15868
453.1057	65.33106	452.8532	59.07228	451.5741	63.15766
453.1472	65.32903	452.8961	59.07015	451.6187	63.15659
453.1884	65.32503	452.9378	59.0693	451.658	63.15549
453.2298	65.32144	452.9804	59.06823	451.6989	63.15411
453.2731	65.32017	453.0209	59.06653	451.7412	63.15333
453.3062	65.31936	453.0621	59.06509	451.7824	63.15254
453.3489	65.31942	453.0972	59.06346	451.8256	63.15204
453.391	65.32075	453.1394	59.06107	451.8688	63.15101
453.4312	65.32016	453.1804	59.06088	451.9096	63.15001
453.4705	65.31797	453.223	59.06061	451.9474	63.14879
453.5123	65.31508	453.2633	59.06054	451.9908	63.1474
453.5538	65.31414	453.3064	59.05928	452.0318	63.14633
453.5947	65.31312	453.3445	59.05668	452.0656	63.14548
453.6389	65.31199	453.3878	59.0539	452.1064	63.1442
453.6791	65.30925	453.43	59.05113	452.1496	63.14371
453.7207	65.30617	453.4722	59.05001	452.1885	63.14299
453.7552	65.30541	453.5125	59.04952	452.2328	63.14225
453.7952	65.30349	453.5465	59.04992	452.2735	63.14183
453.8376	65.30042	453.5884	59.05038	452.3138	63.1406
453.879	65.30066	453.6309	59.04987	452.3572	63.13953
453.9204	65.29849	453.6714	59.04874	452.3994	63.13777
453.962	65.29631	453.7125	59.04701	452.4417	63.136
454.0037	65.29703	453.7546	59.04461	452.4838	63.13403
454.0454	65.29611	453.7938	59.04244	452.5234	63.13215
454.087	65.29497	453.838	59.04132	452.5654	63.13061
454.129	65.29359	453.877	59.04043	452.606	63.12931
454.1697	65.29258	453.918	59.03897	452.6502	63.12877
454.2131	65.29077	453.9615	59.03784	452.6798	63.12749
454.2537	65.28891	453.9926	59.03683	452.7235	63.12645
454.2869	65.28823	454.034	59.03535	452.7642	63.12509
454.3288	65.28682	454.0772	59.03457	452.8049	63.12368
454.3702	65.28496	454.1191	59.03341	452.8461	63.12265
454.4107	65.28247	454.159	59.03191	452.888	63.12195
454.453	65.27729	454.204	59.03118	452.9306	63.12143
454.4933	65.273	454.246	59.02978	452.9718	63.12054
454.5359	65.27014	454.2879	59.02925	453.0165	63.12013
454.5766	65.26892	454.3282	59.02929	453.057	63.1191
454.6203	65.2711	454.3679	59.02844	453.0984	63.1179
454.6601	65.27217	454.4133	59.02765	453.1392	63.11687
454.7016	65.27045	454.4454	59.02687	453.1819	63.11589
454.7336	65.26849	454.4864	59.0246	453.2238	63.11506
454.7768	65.2648	454.5284	59.02274	453.2617	63.11483
454.8187	65.26217	454.5738	59.02118	453.2948	63.11419
454.86	65.26069	454.6128	59.01958	453.3368	63.11298
454.8996	65.25907	454.6541	59.01874	453.3794	63.11171
454.9407	65.2588	454.6944	59.01782	453.4206	63.11031
454.9848	65.25758	454.7375	59.01728	453.463	63.10917
455.0268	65.25567	454.7786	59.01626	453.5048	63.10872
455.0686	65.25345	454.8196	59.01482	453.5458	63.10814
455.1096	65.25045	454.858	59.01212	453.5869	63.10719
455.1519	65.24817	454.8924	59.01008	453.6274	63.10614
455.186	65.24826	454.9353	59.00869	453.6707	63.10486
455.226	65.24869	454.978	59.00713	453.712	63.10371
455.2642	65.24828	455.018	59.0068	453.7533	63.10253
455.3078	65.24478	455.0604	59.00628	453.794	63.10123
455.3509	65.24126	455.1019	59.00562	453.8287	63.10066
455.3932	65.23908	455.1443	59.00487	453.87	63.1

455.4356	65.2376	455.1852	59.00315	453.9116	63.09858
455.4758	65.2368	455.2266	59.00224	453.9539	63.09753
455.5217	65.23711	455.2691	59.00159	453.9946	63.09666
455.5628	65.23494	455.313	58.9996	454.0364	63.0952
455.6014	65.23076	455.3424	58.99914	454.0778	63.09455
455.637	65.22964	455.3844	58.99756	454.1191	63.09324
455.6784	65.22719	455.427	58.99462	454.1596	63.09151
455.7224	65.22392	455.4687	58.99372	454.2014	63.09065
455.764	65.22015	455.5088	58.99233	454.2429	63.08981
455.8051	65.21919	455.5514	58.99143	454.284	63.0895
455.8452	65.21828	455.5928	58.99049	454.3262	63.08888
455.8889	65.2185	455.6344	58.98835	454.3678	63.0881
455.9292	65.22019	455.6758	58.98737	454.4092	63.08701
455.9728	65.22052	455.7176	58.98632	454.4434	63.08622
456.0136	65.21865	455.7584	58.98583	454.4844	63.08553
456.0536	65.21448	455.7918	58.98657	454.5269	63.08438
456.0958	65.21145	455.8348	58.98635	454.5679	63.08293
456.1365	65.20872	455.8728	58.98507	454.6089	63.08137
456.1699	65.20714	455.913	58.98308	454.6534	63.08028
456.209	65.20617	455.9568	58.98068	454.6954	63.07927
456.2513	65.20539	455.9974	58.97809	454.736	63.07825
456.2918	65.20426	456.0376	58.97621	454.7764	63.07744
456.3306	65.20194	456.0807	58.97575	454.8183	63.07647
456.3759	65.19956	456.1225	58.97495	454.8582	63.07538
456.4178	65.19865	456.1628	58.97385	454.9018	63.07384
456.4572	65.19714	456.205	58.97379	454.9441	63.07226
456.4986	65.19644	456.2398	58.97333	454.9841	63.07067
456.54	65.19525	456.2813	58.97232	455.0267	63.06891
456.5824	65.19341	456.3203	58.97049	455.0594	63.06784
456.6178	65.19113	456.361	58.9679	455.1002	63.06651
456.6586	65.18698	456.4019	58.96448	455.1419	63.06453
456.7022	65.18607	456.445	58.96203	455.1813	63.06316
456.741	65.18548	456.4856	58.96022	455.2224	63.0622
456.7841	65.18464	456.5281	58.95952	455.2665	63.06151
456.8237	65.1847	456.569	58.95936	455.3071	63.06071
456.8658	65.18272	456.6124	58.95938	455.3487	63.06
456.9064	65.18059	456.6539	58.95979	455.3926	63.05919
456.9484	65.17804	456.6877	58.96033	455.4348	63.05808
456.9886	65.17621	456.7276	58.95978	455.4746	63.05735
457.0283	65.17522	456.77	58.95839	455.5178	63.05649
457.062	65.17246	456.8108	58.95711	455.5589	63.05586
457.105	65.17137	456.8532	58.95509	455.5916	63.0553
457.1475	65.17089	456.8942	58.95351	455.6328	63.05438
457.1889	65.16982	456.9329	58.95139	455.6734	63.05296
457.2303	65.16881	456.9758	58.94851	455.7163	63.0516
457.2716	65.16747	457.0195	58.94534	455.758	63.04989
457.3128	65.16582	457.0623	58.94327	455.799	63.04871
457.3539	65.16369	457.102	58.942	455.8404	63.04806
457.3989	65.16098	457.1368	58.94113	455.8846	63.04753
457.4388	65.15953	457.1791	58.94158	455.926	63.04686
457.4818	65.15765	457.2217	58.94152	455.966	63.04548
457.5144	65.1554	457.2622	58.93964	456.0074	63.04337
457.5562	65.15296	457.3054	58.9382	456.0486	63.04138
457.5964	65.14839	457.3443	58.93595	456.0913	63.04053
457.6372	65.14593	457.3846	58.93323	456.1328	63.03951
457.6784	65.14392	457.4276	58.93171	456.1732	63.03871
457.7205	65.14245	457.4709	58.93041	456.2086	63.03793
457.7611	65.14409	457.5109	58.92931	456.2512	63.03628

457.805	65.14339	457.5514	58.92805	456.2891	63.03506
457.847	65.14123	457.5896	58.9265	456.332	63.03386
457.8872	65.14028	457.629	58.92583	456.371	63.03222
457.9298	65.13728	457.6736	58.92526	456.4144	63.0309
457.9691	65.13427	457.713	58.92362	456.4548	63.02968
458.0116	65.13335	457.7538	58.92285	456.496	63.02883
458.0452	65.13269	457.7985	58.92139	456.5387	63.02865
458.0868	65.13233	457.8391	58.91922	456.5796	63.02776
458.1303	65.12931	457.8816	58.91879	456.6216	63.02605
458.1713	65.1267	457.9233	58.91872	456.6628	63.02406
458.2137	65.12537	457.9632	58.91809	456.7058	63.02231
458.255	65.12426	458.0029	58.91748	456.7469	63.02131
458.2956	65.12369	458.0384	58.91723	456.7896	63.02077
458.3385	65.12281	458.0784	58.91581	456.821	63.02077
458.3778	65.12067	458.1212	58.91475	456.863	63.02027
458.418	65.11898	458.1641	58.91334	456.9064	63.01885
458.4602	65.11857	458.2022	58.91177	456.9463	63.01768
458.4924	65.11867	458.2461	58.90959	456.9856	63.01623
458.536	65.11757	458.2892	58.90722	457.0291	63.01446
458.5771	65.11633	458.329	58.90388	457.0734	63.01341
458.6198	65.11285	458.3688	58.90039	457.1142	63.01209
458.6619	65.10992	458.4134	58.89974	457.1545	63.01079
458.7017	65.10793	458.4511	58.89816	457.197	63.01009
458.7442	65.10544	458.4858	58.89792	457.2396	63.00832
458.7858	65.10365	458.5281	58.89729	457.2797	63.00623
458.8261	65.10156	458.5714	58.89567	457.3206	63.00427
458.8703	65.10016	458.6126	58.89361	457.355	63.00287
458.909	65.09855	458.654	58.89223	457.3956	63.00157
458.9434	65.09814	458.6947	58.891	457.4389	63.00096
458.9847	65.09623	458.7347	58.88893	457.4804	62.99992
459.0253	65.09157	458.7802	58.88779	457.5206	62.99881
459.068	65.08861	458.8186	58.88671	457.559	62.99704
459.1074	65.08747	458.8604	58.88565	457.6026	62.99555
459.1493	65.08725	458.901	58.88408	457.6436	62.99481
459.1918	65.08807	458.934	58.88312	457.6812	62.99385
459.2347	65.08636	458.9753	58.88231	457.726	62.99327
459.2744	65.0774	459.0167	58.88118	457.7666	62.9918
459.3168	65.07096	459.0582	58.88032	457.809	62.99023
459.359	65.06974	459.101	58.88002	457.8489	62.98878
459.3944	65.06923	459.1398	58.87877	457.8914	62.98782
459.4344	65.07284	459.1826	58.87744	457.9336	62.98713
459.4774	65.07507	459.2234	58.87652	457.9688	62.98658
459.5185	65.07296	459.2669	58.87513	458.011	62.98548
459.559	65.07013	459.3076	58.8743	458.0498	62.98417
459.6017	65.06877	459.3484	58.87393	458.0922	62.98298
459.642	65.06875	459.3826	58.87275	458.1371	62.98215
459.6825	65.06777	459.4225	58.87159	458.1774	62.98124
459.7252	65.06627	459.4658	58.86938	458.219	62.98019
459.7688	65.06051	459.5072	58.86728	458.2606	62.97928
459.8108	65.05851	459.549	58.8661	458.3025	62.97829
459.8498	65.05763	459.5904	58.86595	458.3451	62.97705
459.8925	65.0554	459.6306	58.86609	458.3856	62.97588
459.9228	65.0553	459.6724	58.86529	458.4289	62.975
459.9664	65.05505	459.7146	58.8642	458.4686	62.97373
460.0086	65.05101	459.7556	58.8635	458.5096	62.97296
460.0502	65.04571	459.7984	58.86287	458.5518	62.97195
460.0912	65.04458	459.8286	58.8629	458.5828	62.9714
460.1336	65.04285	459.8709	58.86116	458.6256	62.97017

460.1742	65.04259	459.9129	58.85816	458.667	62.96909
460.2161	65.04133	459.9549	58.8552	458.7124	62.96837
460.2564	65.04114	459.9974	58.85178	458.7545	62.96675
460.2989	65.03969	460.0372	58.84965	458.7921	62.96571
460.3409	65.03695	460.0786	58.84826	458.8354	62.9644
460.3713	65.03558	460.1203	58.84624	458.8761	62.96293
460.4142	65.03373	460.1648	58.84389	458.9187	62.96194
460.4552	65.03137	460.2071	58.84249	458.9602	62.96114
460.4969	65.02839	460.2453	58.84095	459.0008	62.96027
460.5389	65.02938	460.2824	58.84006	459.0429	62.95906
460.5806	65.02933	460.322	58.83929	459.0837	62.95825
460.6228	65.02784	460.3631	58.83875	459.1161	62.95735
460.6622	65.02646	460.404	58.83741	459.1604	62.95604
460.7054	65.02345	460.4459	58.83593	459.1997	62.95483
460.7492	65.02158	460.4886	58.83503	459.2418	62.95333
460.7875	65.02018	460.5286	58.83477	459.2846	62.95167
460.8199	65.01916	460.5706	58.83397	459.3267	62.95079
460.8617	65.01593	460.6104	58.83261	459.3675	62.94981
460.9092	65.01215	460.6553	58.83101	459.4075	62.94848
460.9482	65.00841	460.6958	58.8285	459.4502	62.947
460.9903	65.0063	460.728	58.82738	459.491	62.94517
461.0312	65.00608	460.771	58.82615	459.534	62.94382
461.071	65.00733	460.8118	58.8243	459.5726	62.94298
461.1134	65.00451	460.8549	58.82361	459.6156	62.94223
461.1533	64.99984	460.8967	58.82266	459.6578	62.94113
461.197	64.99755	460.9386	58.82103	459.6991	62.93993
461.238	64.99491	460.9803	58.81935	459.7324	62.93877
461.2708	64.99519	461.0216	58.8176	459.7722	62.93749
461.3122	64.99471	461.064	58.81547	459.8166	62.9363
461.3534	64.99275	461.1055	58.81429	459.858	62.93449
461.3944	64.9896	461.148	58.81343	459.8986	62.9333
461.4376	64.98638	461.1822	58.81155	459.942	62.93194
461.4776	64.98455	461.2216	58.80874	459.9818	62.93105
461.5186	64.98274	461.2605	58.80599	460.0226	62.93046
461.5586	64.98243	461.3038	58.80412	460.0649	62.92958
461.601	64.98071	461.3455	58.80254	460.1086	62.92888
461.6435	64.98108	461.3866	58.80118	460.1484	62.92791
461.684	64.98155	461.4294	58.80039	460.1933	62.92675
461.7278	64.97895	461.47	58.79916	460.2318	62.9255
461.77	64.97769	461.5119	58.79682	460.2724	62.92412
461.7996	64.97479	461.553	58.79585	460.3146	62.92237
461.8436	64.96928	461.5926	58.7942	460.3474	62.92101
461.8826	64.9654	461.626	58.79301	460.3902	62.91955
461.9229	64.96292	461.6664	58.79291	460.4322	62.91838
461.9675	64.96135	461.7096	58.79291	460.4732	62.91709
462.0084	64.96157	461.7504	58.79299	460.5142	62.91611
462.0487	64.95804	461.7913	58.79241	460.5554	62.91463
462.0915	64.95474	461.8326	58.7919	460.5982	62.91304
462.133	64.95155	461.8748	58.78996	460.64	62.91183
462.1722	64.94948	461.918	58.78817	460.6812	62.91087
462.2134	64.95021	461.9596	58.78623	460.7216	62.90925
462.25	64.94774	462.001	58.78345	460.763	62.90796
462.2917	64.94515	462.0446	58.78221	460.8022	62.90686
462.3334	64.94281	462.081	58.78126	460.8478	62.90557
462.375	64.93768	462.1164	58.78113	460.8791	62.90508
462.4171	64.93542	462.1597	58.78129	460.9228	62.90381
462.4582	64.9345	462.2028	58.78093	460.9619	62.90245
462.4992	64.93165	462.2428	58.77996	461.005	62.90135

462.5433	64.93291	462.2838	58.77736	461.045	62.9
462.5821	64.93424	462.3232	58.77465	461.0876	62.89847
462.6252	64.93486	462.3653	58.77461	461.1301	62.89672
462.6678	64.93415	462.4045	58.77403	461.1711	62.89525
462.7002	64.93344	462.4488	58.77546	461.2148	62.894
462.7422	64.93161	462.4918	58.77602	461.2552	62.89335
462.7822	64.92821	462.5234	58.77567	461.2953	62.89257
462.8248	64.92699	462.5656	58.77602	461.3391	62.89132
462.8652	64.92654	462.6082	58.77464	461.3797	62.89025
462.9093	64.92409	462.6485	58.77512	461.421	62.8884
462.9516	64.92039	462.6887	58.77452	461.4634	62.88636
462.9904	64.91909	462.732	58.77265	461.4944	62.88513
463.0313	64.91674	462.773	58.77028	461.539	62.88395
463.0742	64.91486	462.8153	58.76882	461.5801	62.88279
463.117	64.91427	462.8565	58.76619	461.6222	62.88222
463.1474	64.91307	462.898	58.76393	461.6642	62.88107
463.1884	64.9092	462.9411	58.76115	461.707	62.87936
463.231	64.90804	462.9746	58.75873	461.7463	62.87813
463.2726	64.90933	463.0156	58.75585	461.7889	62.87735
463.3152	64.90769	463.0572	58.75444	461.8328	62.87608
463.357	64.90705	463.1002	58.75455	461.8717	62.87442
463.4	64.90155	463.1407	58.7529	461.9126	62.87248
463.4408	64.89566	463.1816	58.75262	461.955	62.87065
463.4814	64.8935	463.2247	58.75123	461.9967	62.869
463.5236	64.88944	463.2694	58.74872	462.0366	62.8681
463.5657	64.88785	463.3074	58.74618	462.0771	62.86738
463.6072	64.88618	463.3497	58.74213	462.1106	62.86636
463.6503	64.87831	463.3891	58.73753	462.1526	62.86566
463.6832	64.87366	463.4222	58.73537	462.1942	62.86463
463.724	64.86923	463.4639	58.73358	462.2367	62.86402
463.7686	64.8686	463.5074	58.73216	462.2769	62.86336
463.8067	64.87254	463.549	58.73085	462.3184	62.86231
463.8477	64.87424	463.5884	58.72989	462.36	62.86152
463.8881	64.87224	463.6322	58.72855	462.4013	62.85993
463.9324	64.87009	463.672	58.72751	462.441	62.85795
463.9738	64.86498	463.7148	58.72532	462.4848	62.85598
464.0156	64.86222	463.7535	58.7229	462.525	62.85415
464.0553	64.86285	463.796	58.72074	462.5694	62.85295
464.0976	64.86328	463.8414	58.71979	462.6094	62.85159
464.1313	64.86426	463.8738	58.71904	462.6456	62.85045
464.172	64.86343	463.9146	58.71809	462.6855	62.84875
464.2152	64.86277	463.9564	58.71751	462.7252	62.84664
464.257	64.86188	463.9968	58.71549	462.768	62.84486
464.2966	64.86079	464.0395	58.71424	462.8084	62.84294
464.3378	64.86195	464.08	58.71343	462.8513	62.84074
464.3778	64.86392	464.12	58.71257	462.8906	62.83826
464.424	64.86009	464.1626	58.71179	462.9334	62.83701
464.4648	64.85519	464.2038	58.7111	462.9761	62.83646
464.5032	64.8519	464.2485	58.71049	463.0163	62.83588
464.5464	64.84931	464.2898	58.70927	463.0588	62.83494
464.5799	64.84592	464.3222	58.70708	463.0976	62.83381
464.6218	64.84381	464.3632	58.70543	463.1422	62.8325
464.6622	64.84177	464.4047	58.70379	463.1836	62.83193
464.7044	64.83931	464.446	58.70199	463.2236	62.83262
464.7449	64.84001	464.488	58.70204	463.2574	62.83169
464.7874	64.8401	464.5288	58.7007	463.3	62.83098
464.8291	64.83955	464.5709	58.70008	463.3422	62.83027
464.8698	64.83714	464.614	58.69903	463.382	62.82861

464.9126	64.8331	464.6544	58.69696	463.425	62.82803
464.9517	64.82715	464.6959	58.69646	463.4672	62.82681
464.9961	64.82473	464.7378	58.69563	463.5088	62.82562
465.0266	64.82256	464.7724	58.69425	463.5504	62.82408
465.0716	64.81965	464.812	58.6925	463.5908	62.82243
465.1116	64.82248	464.8528	58.69125	463.6309	62.82116
465.1534	64.82228	464.8944	58.68701	463.6741	62.81908
465.1961	64.81868	464.9387	58.6846	463.7154	62.81749
465.2384	64.81814	464.9794	58.68349	463.7588	62.81615
465.2802	64.81429	465.0228	58.6826	463.801	62.81456
465.3199	64.81087	465.063	58.68268	463.8422	62.81326
465.3613	64.81116	465.1055	58.6822	463.875	62.81249
465.403	64.81052	465.1478	58.68147	463.915	62.81098
465.4463	64.81026	465.1896	58.67953	463.958	62.8095
465.4889	64.8092	465.2211	58.67762	463.9976	62.80832
465.5313	64.80617	465.264	58.67387	464.04	62.80702
465.5621	64.8042	465.3043	58.67079	464.0805	62.80533
465.6058	64.80148	465.3464	58.6687	464.1236	62.80383
465.6466	64.79571	465.387	58.66789	464.1656	62.8026
465.6875	64.79142	465.4276	58.66878	464.2065	62.80156
465.73	64.78975	465.4685	58.66879	464.2498	62.8007
465.7714	64.78701	465.5094	58.66777	464.2896	62.79976
465.8126	64.78721	465.5523	58.66687	464.3322	62.79831
465.8536	64.78967	465.5928	58.66559	464.374	62.79707
465.8954	64.78725	465.6363	58.6628	464.4084	62.79591
465.9385	64.78594	465.6713	58.66083	464.4487	62.79449
465.9786	64.78386	465.7133	58.65841	464.4903	62.79301
466.0113	64.78145	465.7502	58.65547	464.5317	62.79128
466.055	64.77929	465.7959	58.65494	464.5739	62.79015
466.0942	64.77594	465.8342	58.65414	464.6156	62.7889
466.1356	64.77459	465.8774	58.65239	464.6568	62.788
466.1772	64.77258	465.9216	58.65097	464.6996	62.78622
466.219	64.76913	465.9617	58.64899	464.7402	62.78435
466.2605	64.76562	466.0032	58.64669	464.78	62.78233
466.3038	64.76475	466.0474	58.6455	464.8238	62.78052
466.3447	64.76421	466.0909	58.64366	464.8652	62.77963
466.3866	64.76469	466.1166	58.64186	464.9072	62.77918
466.4309	64.76508	466.1594	58.64036	464.9474	62.77838
466.4644	64.7629	466.2024	58.63843	464.9875	62.77747
466.5037	64.76193	466.2455	58.63746	465.022	62.77651
466.5451	64.75741	466.289	58.63659	465.0644	62.77466
466.5862	64.75314	466.3285	58.63678	465.1052	62.77327
466.6287	64.75297	466.3714	58.63562	465.1463	62.77212
466.67	64.75063	466.4106	58.63472	465.1911	62.77077
466.7106	64.74958	466.4512	58.6333	465.2293	62.76954
466.7514	64.75058	466.492	58.63016	465.2718	62.76845
466.792	64.74842	466.5347	58.62781	465.3128	62.76654
466.834	64.74671	466.5674	58.62669	465.3542	62.76559
466.8772	64.74617	466.61	58.62418	465.3958	62.76453
466.9119	64.74539	466.6521	58.62323	465.4388	62.76353
466.9509	64.7436	466.6922	58.62379	465.4798	62.76275
466.9924	64.74189	466.7343	58.62262	465.5226	62.7606
467.0356	64.73971	466.7763	58.62202	465.563	62.75865
467.0762	64.73566	466.8172	58.62022	465.6042	62.75681
467.1168	64.73193	466.8584	58.61679	465.6406	62.75522
467.1578	64.72619	466.9012	58.61441	465.6801	62.75395
467.2004	64.724	466.942	58.6137	465.7206	62.75259
467.2434	64.72139	466.9812	58.61236	465.7634	62.7517

467.2846	64.71916	467.0158	58.61236	465.8062	62.75074
467.3242	64.72065	467.0591	58.61175	465.8476	62.74984
467.3633	64.71757	467.0985	58.60865	465.8875	62.74901
467.4071	64.71498	467.1406	58.60775	465.9282	62.74722
467.4389	64.71354	467.1844	58.60666	465.9687	62.74602
467.4812	64.71192	467.2242	58.6042	466.0116	62.74405
467.5226	64.70974	467.2653	58.6025	466.0548	62.74221
467.5643	64.70776	467.3071	58.60108	466.094	62.74023
467.6063	64.70784	467.3508	58.59861	466.137	62.73819
467.6484	64.70591	467.3908	58.59849	466.1688	62.7376
467.6888	64.7055	467.4332	58.5981	466.2124	62.7361
467.73	64.70566	467.468	58.59611	466.255	62.73504
467.7724	64.70414	467.5094	58.59345	466.2951	62.73387
467.8154	64.70143	467.5485	58.59135	466.3393	62.73173
467.8574	64.69834	467.591	58.58846	466.3776	62.73088
467.8872	64.69533	467.633	58.5875	466.4202	62.72966
467.9287	64.69225	467.675	58.58728	466.4617	62.72875
467.9674	64.68935	467.7148	58.58583	466.5029	62.72798
468.0124	64.68627	467.7571	58.58488	466.5447	62.72635
468.0528	64.68487	467.7996	58.58382	466.584	62.72509
468.0966	64.6842	467.8424	58.58181	466.6282	62.72292
468.1376	64.68337	467.8832	58.57967	466.669	62.72166
468.1795	64.68117	467.9188	58.57888	466.711	62.72047
468.2206	64.67926	467.9614	58.57622	466.7512	62.71892
468.2637	64.67518	468.0016	58.57352	466.7851	62.71859
468.3054	64.66817	468.0417	58.57136	466.8258	62.71766
468.3412	64.66607	468.0866	58.56749	466.8664	62.71697
468.3781	64.66501	468.1269	58.56511	466.9097	62.7162
468.4211	64.66289	468.17	58.56356	466.9509	62.71454
468.4638	64.66461	468.2097	58.56283	466.9928	62.71257
468.5041	64.66196	468.251	58.56294	467.0341	62.71023
468.5454	64.66026	468.2921	58.56164	467.0754	62.7081
468.587	64.65945	468.3313	58.5602	467.1181	62.70671
468.6281	64.65496	468.364	58.55828	467.1606	62.70523
468.6701	64.65279	468.4078	58.55671	467.2028	62.70432
468.7134	64.64935	468.4482	58.55625	467.2416	62.70339
468.7556	64.64262	468.4912	58.55399	467.2802	62.70189
468.7866	64.63985	468.5308	58.5536	467.3248	62.70058
468.8281	64.63906	468.572	58.55299	467.3674	62.69894
468.8687	64.63574	468.6152	58.5514	467.4	62.69702
468.9131	64.63418	468.6556	58.55104	467.4418	62.69557
468.9556	64.63392	468.6974	58.55044	467.4842	62.6942
468.996	64.63361	468.74	58.54809	467.5248	62.69256
469.0366	64.63306	468.7797	58.54584	467.5645	62.69102
469.0812	64.63234	468.8146	58.54434	467.6067	62.68942
469.1228	64.63083	468.856	58.54233	467.6494	62.68793
469.1646	64.6256	468.896	58.54056	467.6884	62.68704
469.2056	64.62366	468.9382	58.53962	467.7325	62.68687
469.2474	64.62102	468.9776	58.5375	467.7739	62.68603
469.2887	64.61944	469.0214	58.53549	467.8168	62.68488
469.3213	64.61918	469.0649	58.53385	467.8561	62.68289
469.3639	64.61481	469.104	58.53289	467.8988	62.68085
469.4039	64.61137	469.1472	58.53344	467.9304	62.67918
469.4464	64.60936	469.189	58.53264	467.9738	62.6773
469.4896	64.60684	469.2296	58.53159	468.0154	62.67571
469.5288	64.60459	469.2612	58.53044	468.059	62.67453
469.5692	64.59895	469.302	58.52778	468.099	62.67369
469.6125	64.59331	469.3433	58.52456	468.14	62.67294

469.6533	64.59031	469.3852	58.52234	468.1816	62.67231
469.6941	64.58812	469.4254	58.52042	468.2224	62.67107
469.7361	64.58979	469.4682	58.51854	468.2644	62.66978
469.77	64.5896	469.5109	58.51749	468.3055	62.66802
469.8118	64.5867	469.5532	58.51621	468.3465	62.66661
469.8519	64.58724	469.5937	58.51484	468.3917	62.66516
469.8945	64.58416	469.6376	58.51401	468.4328	62.6635
469.9356	64.57953	469.6785	58.51323	468.4726	62.66229
469.977	64.57744	469.7108	58.51248	468.5156	62.66031
470.0201	64.57347	469.7526	58.51234	468.5468	62.6587
470.0624	64.57133	469.7941	58.51076	468.5871	62.65745
470.1038	64.5693	469.8362	58.50815	468.6302	62.65652
470.1452	64.56475	469.8787	58.50643	468.6718	62.6554
470.1864	64.55797	469.9204	58.50277	468.7116	62.65451
470.2184	64.55195	469.9605	58.50006	468.755	62.65297
470.2637	64.5463	470.0022	58.49808	468.7956	62.65111
470.3044	64.54227	470.0446	58.49609	468.8378	62.64984
470.3474	64.54195	470.086	58.49491	468.8787	62.64824
470.3906	64.54343	470.1254	58.49408	468.9213	62.64684
470.4308	64.5438	470.1576	58.49379	468.9636	62.64559
470.4717	64.54165	470.1994	58.49239	469.0049	62.64373
470.5111	64.53713	470.2426	58.49128	469.0458	62.64245
470.5539	64.5313	470.2841	58.49022	469.0883	62.64125
470.5944	64.52547	470.3263	58.48801	469.1302	62.63959
470.6354	64.52186	470.3686	58.48672	469.1624	62.63851
470.6702	64.52293	470.4104	58.48481	469.205	62.63732
470.7123	64.5237	470.4522	58.48145	469.2455	62.63486
470.7525	64.52182	470.4936	58.48009	469.2872	62.63286
470.7947	64.51865	470.533	58.47894	469.3286	62.63107
470.8386	64.50882	470.5746	58.47738	469.3704	62.62946
470.8784	64.49793	470.6096	58.47713	469.4113	62.62839
470.9179	64.49108	470.6497	58.47555	469.4518	62.62724
470.9587	64.49103	470.6916	58.47325	469.4942	62.62617
471.0006	64.49591	470.735	58.47145	469.5369	62.62486
471.0445	64.49882	470.7766	58.46899	469.5773	62.62362
471.0875	64.50077	470.8188	58.46749	469.6186	62.62224
471.1256	64.49675	470.859	58.46639	469.6602	62.62066
471.168	64.49042	470.9006	58.46437	469.6944	62.61908
471.2042	64.48704	470.9424	58.46366	469.7354	62.61778
471.2441	64.48566	470.9844	58.46234	469.7786	62.61664
471.284	64.48338	471.0258	58.46041	469.8189	62.61524
471.3235	64.48105	471.061	58.4592	469.8576	62.61438
471.3647	64.48062	471.1037	58.45713	469.901	62.61312
471.406	64.47849	471.1447	58.45474	469.9439	62.61167
471.4506	64.47895	471.1861	58.45231	469.9862	62.60978
471.4916	64.47787	471.2282	58.45005	470.0272	62.60782
471.534	64.47426	471.2684	58.44841	470.0676	62.60586
471.577	64.47231	471.3094	58.44717	470.109	62.60432
471.6165	64.46641	471.353	58.44566	470.153	62.60305
471.65	64.46211	471.3906	58.44431	470.1914	62.60162
471.6915	64.46239	471.432	58.44272	470.2368	62.60011
471.7352	64.45972	471.4742	58.44102	470.2748	62.59798
471.775	64.45763	471.5053	58.43947	470.3087	62.59661
471.816	64.45562	471.5483	58.43703	470.3512	62.59499
471.8594	64.45055	471.5904	58.43524	470.3919	62.59329
471.9	64.44943	471.6324	58.43316	470.4337	62.59193
471.942	64.45013	471.6738	58.43084	470.4746	62.59092
471.9834	64.45048	471.7147	58.42951	470.518	62.58993

472.0246	64.44812	471.7552	58.42868	470.556	62.589
472.0663	64.44528	471.7972	58.42827	470.5996	62.58772
472.099	64.44292	471.8393	58.42739	470.6416	62.58637
472.1414	64.43905	471.8804	58.42686	470.683	62.58437
472.1836	64.44207	471.9239	58.42594	470.7261	62.58234
472.224	64.4407	471.9566	58.42447	470.7656	62.58137
472.2664	64.43761	471.9969	58.42364	470.8078	62.57964
472.306	64.43722	472.0396	58.42192	470.849	62.57787
472.3476	64.43225	472.0821	58.41922	470.8896	62.57623
472.3911	64.42994	472.1252	58.41614	470.9248	62.57485
472.4338	64.43148	472.1657	58.41408	470.966	62.57324
472.4756	64.432	472.2102	58.41171	471.008	62.5717
472.5176	64.42997	472.2509	58.41016	471.0492	62.57052
472.5537	64.42822	472.2921	58.40885	471.0899	62.56896
472.5934	64.4262	472.3346	58.40655	471.1328	62.56742
472.6348	64.42096	472.3748	58.40532	471.1748	62.56572
472.6768	64.42	472.4094	58.40433	471.2152	62.56389
472.7176	64.41957	472.4496	58.40263	471.257	62.56152
472.7584	64.42009	472.4922	58.40253	471.2994	62.55933
472.8014	64.41856	472.534	58.40097	471.3381	62.55784
472.8408	64.41657	472.575	58.3987	471.3812	62.5566
472.8828	64.4152	472.6164	58.3966	471.4247	62.55582
472.9262	64.40909	472.6587	58.39495	471.4571	62.55546
472.9676	64.40743	472.7004	58.39326	471.4998	62.55451
473.0086	64.407	472.7395	58.39217	471.5418	62.55305
473.0518	64.40766	472.7812	58.39031	471.583	62.55113
473.0854	64.4076	472.8252	58.3876	471.6248	62.54869
473.1246	64.40458	472.8566	58.38665	471.6668	62.54649
473.166	64.40213	472.8988	58.38529	471.7096	62.54467
473.2094	64.39809	472.9419	58.38421	471.75	62.54341
473.2496	64.395	472.9824	58.38317	471.7926	62.54231
473.2926	64.39316	473.023	58.38162	471.8338	62.54148
473.3318	64.3949	473.0646	58.37823	471.874	62.54006
473.3743	64.39502	473.1092	58.37542	471.9174	62.53872
473.4148	64.39223	473.15	58.37211	471.9605	62.53713
473.4578	64.39254	473.19	58.36811	471.9984	62.5355
473.4988	64.38631	473.2312	58.36671	472.0411	62.53447
473.5347	64.38279	473.2742	58.36589	472.0769	62.53307
473.575	64.38038	473.3078	58.36538	472.1174	62.53178
473.618	64.37874	473.3473	58.36541	472.1586	62.53026
473.6594	64.38119	473.3913	58.36434	472.2008	62.52874
473.7006	64.38239	473.4305	58.3625	472.2415	62.52701
473.7416	64.38284	473.4713	58.36101	472.2847	62.52546
473.7825	64.38088	473.5134	58.35871	472.3227	62.52381
473.8227	64.37779	473.557	58.35648	472.3653	62.52215
473.8638	64.37238	473.5992	58.35369	472.406	62.52096
473.9063	64.3673	473.6381	58.35124	472.4498	62.51944
473.95	64.36635	473.6809	58.35027	472.4902	62.51769
473.9831	64.36546	473.7248	58.35026	472.5328	62.5156
474.0235	64.36216	473.7574	58.34918	472.5768	62.51339
474.0662	64.36288	473.7974	58.3475	472.6152	62.51158
474.106	64.36219	473.8391	58.34516	472.6576	62.51027
474.1479	64.35911	473.8816	58.34234	472.6888	62.50931
474.1894	64.35944	473.9196	58.34032	472.7299	62.50815
474.232	64.35882	473.963	58.33906	472.771	62.50696
474.2744	64.35453	474.0031	58.33771	472.8119	62.50556
474.315	64.35152	474.0447	58.33643	472.8533	62.5039
474.356	64.34836	474.0869	58.33584	472.894	62.50246

474.397	64.34664	474.1282	58.33392	472.9376	62.50078
474.4306	64.34606	474.1704	58.33241	472.9766	62.49875
474.4738	64.34423	474.2044	58.33025	473.0168	62.49673
474.5143	64.3413	474.2464	58.32822	473.0602	62.49457
474.555	64.33647	474.2876	58.32621	473.1024	62.49302
474.5942	64.33412	474.3282	58.32419	473.1437	62.49186
474.6392	64.33065	474.3715	58.3224	473.1863	62.49089
474.6812	64.32977	474.4127	58.31984	473.2186	62.48997
474.7204	64.32854	474.456	58.3173	473.2598	62.4885
474.7613	64.32425	474.4958	58.31504	473.3011	62.48678
474.8056	64.32303	474.5364	58.31449	473.345	62.48456
474.8464	64.31801	474.5782	58.31341	473.3848	62.48268
474.8881	64.31238	474.62	58.31213	473.4269	62.48118
474.9296	64.30835	474.6541	58.31183	473.469	62.47968
474.962	64.30575	474.6957	58.30973	473.5094	62.47839
475.0042	64.30382	474.7364	58.30761	473.5514	62.47669
475.0426	64.30143	474.7796	58.3057	473.5932	62.47459
475.088	64.29999	474.8222	58.30392	473.6338	62.47254
475.13	64.29753	474.8627	58.30125	473.6758	62.47028
475.1709	64.29732	474.9034	58.29935	473.7164	62.46843
475.2124	64.29609	474.9457	58.29755	473.7578	62.46719
475.2544	64.29533	474.986	58.29419	473.798	62.46611
475.2937	64.29406	475.0282	58.29206	473.8336	62.46542
475.3338	64.28927	475.0668	58.28953	473.8744	62.46448
475.3774	64.28713	475.0986	58.28731	473.9174	62.463
475.4121	64.28445	475.1428	58.28687	473.958	62.46082
475.4528	64.28171	475.1846	58.28557	473.9984	62.45937
[truncated: 653,177 more chars]
